# Author Correction: High-throughput inverse design and Bayesian optimization of functionalities: spin splitting in two-dimensional compounds

**DOI:** 10.1038/s41597-022-01641-7

**Published:** 2022-08-19

**Authors:** Gabriel M. Nascimento, Elton Ogoshi, Adalberto Fazzio, Carlos Mera Acosta, Gustavo M. Dalpian

**Affiliations:** 1 Center for Natural and Human Sciences, Federal University of ABC, Santo Andre, SP Brazil; 2 Brazilian Nanotechnology National Laboratory (LNNano), CNPEM, 13083–970, Campinas, São Paulo, Brazil

**Keywords:** Computational methods, Spintronics, Electronic and spintronic devices, Electronic structure, Two-dimensional materials

Correction to: *Scientific Data* 10.1038/s41597-022-01292-8, published online 29 April 2022

Figure 2 of the paper was incorrect in the original version, with the labels for “Non-zero electric dipole” and “Zero electric dipole” switched around. This has been corrected in the HTML and pdf versions of the paper, which now indicates that “Non-zero electric dipole moment” sits within the Design principles for Rashba SS box on the right side of the figure, and “Zero electric dipole” sits within the Design Principles for Dresselhaus SS on the left, as the authors originally intended.

