# Supplementary material for: High-throughput inverse design and Bayesian optimization of functionalities: spin splitting in two-dimensional compounds
Source: Sci Data. 2022 Apr 29;9:195. doi: 10.1038/s41597-022-01292-8 (PMC9054849; doi:10.1038/s41597-022-01292-8)
Supplement: Supplementary file 1 [file 41597_2022_1292_MOESM1_ESM.pdf]

# Supplementary Information - Materials

High-throughput inverse design and optimization of functionalities: spin splitting in two-dimensional compounds

Gabriel M. Nascimento[1,a], Elton Ogoshi[1,a], Adalberto Fazzio[1,2], Carlos Mera Acosta[1,*], and Gustavo M. Dalpian[1,*]

[a] *These authors contributed equally to this work.*
[1] *Center for Natural and Human Sciences, Federal University of ABC, Santo Andre, SP, Brazil*
[2] *Brazilian Nanotechnology National Laboratory (LNNano), CNPEM, 13083-970, Campinas, São Paulo, Brazil*
[*] *Corresponding authors: cmeraacosta@gmail.com; gustavo.dalpian@ufabc.edu.br*

# Contents


**1 K-paths along high-symmetry lines** **3**

**2 Materials data** **4**

2.1 Ag2Cl2-dd5f0964d63d . . . 4
2.2 Ag2F2-44f6ed525a5a . . . 5
2.3 Ag2Se2-248fffac5959 . . . 6
2.4 Ag2Te2-648de0a225fe . . . 7
2.5 Al2Br6-fab8ea0c0979 . . . 8
2.6 Al2Cl6-450a3fc3ff50 . . . 9
2.7 Al2I6-29ae3f5ef5a7 . . . 10
2.8 Al2O2-bce0ccee4eca . . . 11
2.9 Al2P2S6-669d6f1af4d4 . . . 12
2.10 Al2P2Se6-90d84f697622 . . . 13
2.11 Al2S2-f9df9f4a5c34 . . . 14
2.12 Al2Se2-129a514b51ad . . . 15
2.13 Al2Te2-e54041554385 . . . 16
2.14 AlAs-814ae25a188e . . . 17
2.15 AlN-4fcd8dcb9c90 . . . 18
2.16 AlSb-1734deee2ac1 . . . 19
2.17 AsB-b6e76caa350b . . . 20
2.18 AsBiCr-b299416bff28 . . . 21
2.19 AsBrS-1dcd471c2288 . . . 22
2.20 AsBrS-d9f4d4011670 . . . 23
2.21 AsBrSe-206b9dcf2af6 . . . 24
2.22 AsBrSe-989f469f06bd . . . 25
2.23 AsBrTe-64921449e408 . . . 26
2.24 AsBrTe-671e6de2497a . . . 27
2.25 AsClS-0fd6ab210774 . . . 28
2.26 AsClS-afd0d75a82a2 . . . 29
2.27 AsClSe-1a3be826b3e0 . . . 30
2.28 AsClSe-df329350eef2 . . . 31
2.29 AsClTe-4fd8ad708fb0 . . . 32
2.30 AsClTe-fba4cc0df459 . . . 33
2.31 AsGa-728f322893fe . . . 34
2.32 AsIS-b13beafa16aa . . . 35
2.33 AsIS-e23390b66883 . . . 36
2.34 AsISe-5d829e480507 . . . 37
2.35 AsISe-ca926a42865b . . . 38
2.36 AsITe-114b3382699c . . . 39
2.37 AsITe-b6d803aafe3a . . . 40
2.38 AsIn-c77a730c90f8 . . . 41
2.39 Au2Te2-d57228736661 . . . 42
2.40 BN-4a5edc763604 . . . 43
2.41 BP-0a5e44762c75 . . . 44
2.42 BSb-71730c0eaab1 . . . 45
2.43 BaBr2-1a59eff92917 . . . 46
2.44 BaBr2-df54a81e64da . . . 47
2.45 BaCl2-54ec344f88a7 . . . 48
2.46 BaCl2-ef1fab58e11f . . . 49
2.47 BaF2-4b7403281822 . . . 50
2.48 BaI2-c4707a226b8f . . . 51
2.49 BaI2-e20476acac18 . . . 52
2.50 Bi2Br6-2b3b022fdf0a . . . 53
2.51 Bi2Cl6-3e134cb61e06 . . . 54
2.52 Bi2I6-01a2d5e89adc . . . 55
2.53 Bi2O2-53ac438f321b . . . 56
2.54 Bi2P2S6-287dcf4f1a19 . . . 57
2.55 Bi2P2Se6-aa9a981d89aa . . . 58
2.56 Bi2P2Te6-cf7927ab6730 . . . 59

2.57 Bi2Se2-d13cbee86f79 . . . 60
2.58 Bi2Te2-23a0796492d1 . . . 61
2.59 BiBrS-3b305c3e2c18 . . . 62
2.60 BiBrS-49b7be14f786 . . . 63
2.61 BiBrSe-11db0908d9ef . . . 64
2.62 BiBrSe-de5756e4fbfa . . . 65
2.63 BiBrTe-304bc6a92d82 . . . 66
2.64 BiBrTe-f4f45fcade85 . . . 67
2.65 BiClS-99fd027b1d0b . . . 68
2.66 BiClS-c96ef4fc869c . . . 69
2.67 BiClSe-7fe9c5cb910c . . . 70
2.68 BiClSe-a80866a2c6b4 . . . 71
2.69 BiClTe-968a6902b7f5 . . . 72
2.70 BiClTe-badda86cab42 . . . 73
2.71 BiIS-40034665f9f1 . . . 74
2.72 BiIS-acdcd16c0d76 . . . 75
2.73 BiISe-433f707c632c . . . 76
2.74 BiISe-70cbc0e44d36 . . . 77
2.75 BiITe-2d41b3dd1772 . . . 78
2.76 BiITe-a84d988e38ac . . . 79
2.77 Br2S2Tl2-b10aab79a70e . . . 80
2.78 Br2Tl2-948c61cd5626 . . . 81
2.79 BrClHf-72257f9ad66d . . . 82
2.80 BrClTi-d3f135b9cf41 . . . 83
2.81 BrClZr-8cb69386d06b . . . 84
2.82 BrHfI-836a1091409d . . . 85
2.83 BrITi-233dbbf8f473 . . . 86
2.84 BrIZr-28c61999c692 . . . 87
2.85 BrSSb-4ae37f15e1fe . . . 88
2.86 BrSSb-4da5c6be60db . . . 89
2.87 BrSbSe-89b15ddef41d . . . 90
2.88 BrSbSe-c2a344b393f0 . . . 91
2.89 BrSbTe-18e62ba75259 . . . 92
2.90 BrSbTe-f1e78a09001d . . . 93
2.91 C2O2Hf3-082ae1b027e9 . . . 94
2.92 C2O2Zr3-23672dbca7d0 . . . 95
2.93 CSiF2-ee1174d1d821 . . . 96
2.94 CSiH2-8a1587098151 . . . 97
2.95 CaBr2-49f279264c91 . . . 98
2.96 CaBr2-fbb623b6f288 . . . 99
2.97 CaCl2-3ca106221b9b . . . 100
2.98 CaCl2-55e6ef39650c . . . 101
2.99 CaF2-9632fda9fd46 . . . 102
2.100 CaI2-066f40f26c53 . . . 103
2.101 CaI2-793f311ee701 . . . 104
2.102 Cd2P2S6-ebcb2404504e . . . 105
2.103 Cd2P2Se6-0460cce85156 . . . 106
2.104 Cd2Se2-3394b92523be . . . 107
2.105 Cd2Se4-f25f251c7be8 . . . 108
2.106 Cd2Te2-ed3311682142 . . . 109
2.107 CdBr2-a7bb757c6234 . . . 110
2.108 CdBr2-bb3c9722fb14 . . . 111
2.109 CdCl2-46c028e03e8b . . . 112
2.110 CdCl2-ff50eed37ec7 . . . 113
2.111 CdF2-14736784891b . . . 114
2.112 CdI2-66c5fba8ad87 . . . 115
2.113 CdI2-d63ad801fdb5 . . . 116
2.114 Cl2Cu2-c1a86f114149 . . . 117
2.115 Cl2In2-9d0130e99f0c . . . 118
2.116 Cl2Pt2-93dfef2d1004 . . . 119
2.117 ClHfI-d3756ea15451 . . . 120

2.118 ClITi-ae06e7424bb1 . . . 121
2.119 ClIZr-73202b4b7837 . . . 122
2.120 ClSSb-0495f35048b5 . . . 123
2.121 ClSSb-9188c300265c . . . 124
2.122 ClSbSe-0c0fbdaf8f4a . . . 125
2.123 ClSbSe-f705a30af945 . . . 126
2.124 ClSbTe-04fdd7d1ec5c . . . 127
2.125 ClSbTe-da5fd2bb47af . . . 128
2.126 Cr2Mo2S8-72b286460831 . . . 129
2.127 Cr2Mo2Se8-60065d3bbcf2 . . . 130
2.128 Cr2Mo2Te8-988b11badabb . . . 131
2.129 Cr2W2S8-5974b6403c31 . . . 132
2.130 Cr2W2Se8-548aa830244c . . . 133
2.131 Cr2W2Te8-62bb754c4cb2 . . . 134
2.132 CrMo3S8-644f7c1c85c7 . . . 135
2.133 CrMo3Se8-a7233837cfe9 . . . 136
2.134 CrMo3Te8-159f028a85d0 . . . 137
2.135 CrO2-2433700165bb . . . 138
2.136 CrS2-c5ee5e35d2b4 . . . 139
2.137 CrSSe-09e1e5ef94cb . . . 140
2.138 CrSTe-8a0864d30ce1 . . . 141
2.139 CrSe2-9a6ff6a3c41a . . . 142
2.140 CrSeTe-5d9d3ded04de . . . 143
2.141 CrTe2-c31911a1b3f9 . . . 144
2.142 CrW3S8-a9f87eba4b96 . . . 145
2.143 CrW3Se8-0b7696e1f4c9 . . . 146
2.144 CrW3Te8-eef072f845ce . . . 147
2.145 Cu2I2-ac7333c6ab94 . . . 148
2.146 Cu2S2-ed5f6e977271 . . . 149
2.147 Fe2P2S6-91367f7f5dce . . . 150
2.148 FeF2-c67fbd722c30 . . . 151
2.149 Ga2Br6-e9e5bee778b6 . . . 152
2.150 Ga2Cl6-c306553fa81a . . . 153
2.151 Ga2O2-16c96094d1a0 . . . 154
2.152 Ga2P2Te6-4cb4ea247ef4 . . . 155
2.153 Ga2S2-ac002f4ce724 . . . 156
2.154 Ga2Se2-394e5709a3ac . . . 157
2.155 Ga2Te2-55c23ca88a05 . . . 158
2.156 GaN-c973e283b023 . . . 159
2.157 GaP-d467820f3f04 . . . 160
2.158 Ge2P2Se6-6152fa4b5f1b . . . 161
2.159 Ge2P2Te6-e8f4b479deef . . . 162
2.160 Ge2S2-ecbb7c185669 . . . 163
2.161 GeBr2-204ef2affa10 . . . 164
2.162 GeBr2-36a198743d35 . . . 165
2.163 GeCl2-3ea474649fa9 . . . 166
2.164 GeCl2-a7216f084785 . . . 167
2.165 GeF2-943ceb2df00b . . . 168
2.166 GeI2-694ac91aec01 . . . 169
2.167 GeO-a42f736f1682 . . . 170
2.168 GeO2-21281ac194c2 . . . 171
2.169 GeO2-77905aa4e75f . . . 172
2.170 GeS-227b12019ade . . . 173
2.171 GeS2-69b36b84eb8c . . . 174
2.172 GeSe-211bcb7f05d6 . . . 175
2.173 GeSe2-8af45b2cf14e . . . 176
2.174 GeTe-eadd37f03ca5 . . . 177
2.175 Hf2Ti2S8-c8cfffe63bfa . . . 178
2.176 Hf2Ti2Se8-cce78d90e899 . . . 179
2.177 Hf2Zr2S8-540829ada792 . . . 180
2.178 Hf2Zr2Se8-81af2831dbb2 . . . 181

2.179Hf2Zr2Te8-93099006c996 . . . 182
2.180HfBr2-84e9162c0c53 . . . 183
2.181HfCl2-864f8b497185 . . . 184
2.182HfI2-05a69240794c . . . 185
2.183HfO2-512afaae525a . . . 186
2.184HfO2-6e4ac7453419 . . . 187
2.185HfS2-2c5e65012601 . . . 188
2.186HfS2-3d4bfe131291 . . . 189
2.187HfSSe-63618e5bf062 . . . 190
2.188HfSSe-9afb20358166 . . . 191
2.189HfSe2-08401460f377 . . . 192
2.190HfSe2-d2d9fee03594 . . . 193
2.191HfSeTe-305c779b8752 . . . 194
2.192HfTe2-1e2c6946ca41 . . . 195
2.193HfTe2-59c0e014651d . . . 196
2.194HfTi3S8-fde2d81d10df . . . 197
2.195HfTi3Se8-c55716558616 . . . 198
2.196HfZr3S8-78bb1ac31c01 . . . 199
2.197HfZr3Se8-70e7ab872359 . . . 200
2.198HfZr3Te8-916e19eae465 . . . 201
2.199Hg2I2-f7e70d2b90ad . . . 202
2.200Hg2P2S6-b81eb586acfd . . . 203
2.201HgBr2-978e95bef964 . . . 204
2.202HgBr2-9965e7e32aa2 . . . 205
2.203HgCl2-6cbe2e585099 . . . 206
2.204HgCl2-ce3ed4728e8f . . . 207
2.205HgF2-f5965c8b3d89 . . . 208
2.206HgI2-0ff7ee261fec . . . 209
2.207HgI2-7c2657e15a6f . . . 210
2.208HgO-a8678fa85c38 . . . 211
2.209HgS-5256ed7d716e . . . 212
2.210HgSe-619ed885f677 . . . 213
2.211HgTe-1a3bdd1b142a . . . 214
2.212I2Tl2-c0f52097ab62 . . . 215
2.213ISSb-4c49d27e66e5 . . . 216
2.214ISSb-5b94060698bc . . . 217
2.215ISbSe-343d2125478e . . . 218
2.216ISbSe-df0019ec24b5 . . . 219
2.217ISbTe-052a3116531d . . . 220
2.218ISbTe-0f02957b17cf . . . 221
2.219In2Br6-d59933910be2 . . . 222
2.220In2Cl6-89b069b9f428 . . . 223
2.221In2I6-d90f9a6ab379 . . . 224
2.222In2O2-d14171d2ba1a . . . 225
2.223In2P2S6-793870f62166 . . . 226
2.224In2S2-172ef584c4a6 . . . 227
2.225In2Se2-eb204c739879 . . . 228
2.226In2Te2-3cf9ea4cebc4 . . . 229
2.227In2Te2-fcd97ff5abcd . . . 230
2.228InN-8cf70870bc5b . . . 231
2.229InP-c5672c6c1c78 . . . 232
2.230InSb-466fcf7fad66 . . . 233
2.231Ir2Cl6-be7870547213 . . . 234
2.232Ir2O2-06ebe3806790 . . . 235
2.233Ir2P2S6-2ddba2a8216d . . . 236
2.234Ir2P2Se6-65c3ae4ecac2 . . . 237
2.235Ir2P2Te6-1a850181de67 . . . 238
2.236Ir2S2-dd6289af8e01 . . . 239
2.237Ir2Se2-53337987551a . . . 240
2.238MgBr2-bee1987fb4e6 . . . 241
2.239MgCl2-e73a5c5ae5ac . . . 242

2.240MgI2-67bb6819958f . . . 243
2.241Mo2Cl6-61d74efeaeeb . . . 244
2.242Mo2W2S8-449640ec4d30 . . . 245
2.243Mo2W2Se8-a1d716aad84d . . . 246
2.244Mo2W2Te8-c04fc052f2ca . . . 247
2.245MoCr3S8-3fb52099b370 . . . 248
2.246MoCr3Se8-961c37d6e527 . . . 249
2.247MoCr3Te8-899032b4ad0c . . . 250
2.248MoO2-152bd69757aa . . . 251
2.249MoS2-b3b4685fb6e1 . . . 252
2.250MoSSe-de7ac5fc6945 . . . 253
2.251MoSTe-2ea941c8bc3c . . . 254
2.252MoSTe-e4bb8738150a . . . 255
2.253MoSe2-f61b14d398c7 . . . 256
2.254MoSeTe-42eb12e7b656 . . . 257
2.255MoTe2-38a53176109a . . . 258
2.256MoW3S8-2f6f133abcc8 . . . 259
2.257MoW3Se8-24d6cc0a0fed . . . 260
2.258MoW3Te8-5c3fe56a1a89 . . . 261
2.259N2O2Hf3-bb4e40ae9164 . . . 262
2.260N2O2Zr3-c317fbd68215 . . . 263
2.261Nb2P2S6-c9aa3a9d876c . . . 264
2.262O2Pb2-20f098bd3f31 . . . 265
2.263O2Rh2-740bf2751050 . . . 266
2.264O2Sc2-b757b8efeeab . . . 267
2.265O2W2-42fa50003592 . . . 268
2.266OPb-2a393480e273 . . . 269
2.267OSn-026ebfd86b48 . . . 270
2.268OsBr2-bf30e1249164 . . . 271
2.269OsCl2-d37ba63794ad . . . 272
2.270P2Pb2S6-7cf77f91eef0 . . . 273
2.271P2Pb2Se6-1811d52ac455 . . . 274
2.272P2Pb2Te6-f0b8906970f5 . . . 275
2.273P2Pt2Se6-3a65ac9ff395 . . . 276
2.274P2Rh2Se6-c10e77a99b6c . . . 277
2.275P2Rh2Te6-0f1f68e6fa79 . . . 278
2.276P2Ru2S6-9caed1a0620c . . . 279
2.277P2Ru2Te6-dc750358a911 . . . 280
2.278P2Sb2Se6-5d1a32a28ffa . . . 281
2.279P2Sb2Te6-82b85dfd7723 . . . 282
2.280P2Sc2S6-bc8b8c21ad4f . . . 283
2.281P2Sc2Se6-093920d00119 . . . 284
2.282P2Sn2S6-aa34f4a1c451 . . . 285
2.283P2Sn2Se6-a056ab5346bf . . . 286
2.284P2Sn2Te6-321533580298 . . . 287
2.285P2Ta2Se6-e2c90519357b . . . 288
2.286P2Ta2Te6-601435d29c97 . . . 289
2.287P2Zr2S6-f821141337d6 . . . 290
2.288Pb2S2-d4ed2cd9ee0c . . . 291
2.289Pb2S2-fdff29051b6d . . . 292
2.290Pb2Se2-a346b1c50933 . . . 293
2.291Pb2Se2-f615d3b872f4 . . . 294
2.292Pb2Te2-fdc4a7cc1d0d . . . 295
2.293Pb2Te6-3995fa1bee6e . . . 296
2.294PbBr2-cabd4ba0f21c . . . 297
2.295PbBr2-cbdc15b42a05 . . . 298
2.296PbCl2-b0b142073783 . . . 299
2.297PbCl2-f9d58a299674 . . . 300
2.298PbF2-ccc95033446d . . . 301
2.299PbI2-14411dde597c . . . 302
2.300PbI2-9e6494406d07 . . . 303

2.301PbO2-8d2de90b58b6 . . . 304
2.302PbS-5e4ff1f56b4a . . . 305
2.303PbS2-372c217dd52f . . . 306
2.304PbS2-9842835dff03 . . . 307
2.305PbSe-a0dbdc6630fa . . . 308
2.306PbSe2-0bc5d11454a7 . . . 309
2.307PbSe2-45aa9714a72b . . . 310
2.308PbTe-3bc08d486d65 . . . 311
2.309PbTe2-dbf3271b4bb1 . . . 312
2.310PdSe2-0ae696751911 . . . 313
2.311Re2Cl6-21e7665ac8f4 . . . 314
2.312Re2I6-04979b21e197 . . . 315
2.313Rh2Br6-c284d6de2b3e . . . 316
2.314Rh2Cl6-06f695e97385 . . . 317
2.315Rh2Se2-1a46a7cf8fab . . . 318
2.316S2Sc2-e9d256b367c7 . . . 319
2.317S2Si2-0726c763a59a . . . 320
2.318S2Sn2-7a8373382b33 . . . 321
2.319S2Sn2-7edb67bf350a . . . 322
2.320S2Tl2-751e767bff79 . . . 323
2.321S2Tl2-fb0b06c4bab6 . . . 324
2.322S2V2-605c732d5111 . . . 325
2.323SSeTi-358305cad463 . . . 326
2.324SSeW-001e03f2c095 . . . 327
2.325SSeZr-1a9901838600 . . . 328
2.326SSeZr-2be14f373da0 . . . 329
2.327SSn-f98da23471a1 . . . 330
2.328STeW-75ee10091f43 . . . 331
2.329STeW-916afba26723 . . . 332
2.330STeZr-3f3c7bc0ce7d . . . 333
2.331Sc2Br6-a43351c43cd3 . . . 334
2.332Sc2Cl6-3eef25de07b3 . . . 335
2.333Sc2I6-d59c550c4a3a . . . 336
2.334Sc2Se2-9fb15588e4d4 . . . 337
2.335Sc2Te2-c3cac8e74dc1 . . . 338
2.336Se2Si2-aa0e94e8944e . . . 339
2.337Se2Sn2-9b27b6ad7899 . . . 340
2.338Se2Tl2-16490f81af26 . . . 341
2.339Se2Tl2-625697b299d1 . . . 342
2.340Se2V2-9cf30bd127fe . . . 343
2.341Se2Zn2-90835c470691 . . . 344
2.342Se2Zr2-f89b20d72c95 . . . 345
2.343SeSn-d59c96fdfda1 . . . 346
2.344SeTeW-6e2a4c6f4f57 . . . 347
2.345SeTeZr-dd69b684c867 . . . 348
2.346Sn2Te2-03bcf7dcdaf2 . . . 349
2.347Sn2Te2-653f8247e25e . . . 350
2.348SnBr2-0155c4de2320 . . . 351
2.349SnBr2-8d365ca62c55 . . . 352
2.350SnCl2-514a8a12dca9 . . . 353
2.351SnCl2-95805103ce95 . . . 354
2.352SnF2-d6c3ab6de6b8 . . . 355
2.353SnI2-7f0ca28e3229 . . . 356
2.354SnI2-d9c422656482 . . . 357
2.355SnO2-96a036411ab6 . . . 358
2.356SnO2-d5f47e5d4cf7 . . . 359
2.357SnS2-08a9307b286e . . . 360
2.358SnS2-8f2fa65321f0 . . . 361
2.359SnSe2-bfa429d647f9 . . . 362
2.360SnTe-e688959ea45b . . . 363
2.361SnTe2-c81de95356c1 . . . 364

2.362SrBr2-2876a0cb2478 . . . . . 365
2.363SrBr2-a4c9c803de7d . . . . . 366
2.364SrCl2-77398c835c11 . . . . . 367
2.365SrCl2-e03e4067ecb2 . . . . . 368
2.366SrF2-c99805c05244 . . . . . 369
2.367SrI2-1059dc7f5fe4 . . . . . 370
2.368SrI2-6cfaae647808 . . . . . 371
2.369Te2Ti2-b43c14735d8e . . . . . 372
2.370Te2Ti2-ecf4b958e9a1 . . . . . 373
2.371Te2Tl2-44ffc9d405b8 . . . . . 374
2.372Te2Tl2-73117163f0e2 . . . . . 375
2.373Te2V2-5da53e6996e3 . . . . . 376
2.374Te2Zr2-8912432cb37b . . . . . 377
2.375Ti2Zr2S8-a99139546333 . . . . . 378
2.376Ti2Zr2Se8-846b50801a93 . . . . . 379
2.377Ti2Zr2Te8-18e377cce57f . . . . . 380
2.378TiBr2-57116f9a9a4e . . . . . 381
2.379TiCl2-95688ba68ca1 . . . . . 382
2.380TiHf3S8-eb71cb1c9077 . . . . . 383
2.381TiHf3Se8-3e1923c616ad . . . . . 384
2.382TiHf3Te8-1667d1443160 . . . . . 385
2.383TiI2-088e8488f895 . . . . . 386
2.384TiO2-1cfb690281c9 . . . . . 387
2.385TiO2-badf6957f0bb . . . . . 388
2.386TiS2-0428a52595e8 . . . . . 389
2.387TiS2-65d41aaec667 . . . . . 390
2.388TiSe2-0684166af1fd . . . . . 391
2.389TiSe2-509ef368050d . . . . . 392
2.390TiTe2-bbb8e581bf27 . . . . . 393
2.391TiZr3S8-ec37c6657ea3 . . . . . 394
2.392TiZr3Se8-a148361e5e9a . . . . . 395
2.393TiZr3Te8-4f1ab08988cc . . . . . 396
2.394Tl2Br6-ef8df28dd878 . . . . . 397
2.395Tl2Cl4-30ddf92eb9d6 . . . . . 398
2.396Tl2Cl6-a7ad562f5ed6 . . . . . 399
2.397Tl2I6-8b75f770fbf8 . . . . . 400
2.398W2Br6-14e114b103e1 . . . . . 401
2.399W2Cl6-eea8aee9e120 . . . . . 402
2.400W2I6-37deba64dc68 . . . . . 403
2.401WCr3S8-dc4259e69783 . . . . . 404
2.402WCr3Se8-c798e725e2fb . . . . . 405
2.403WCr3Te8-6523c349753c . . . . . 406
2.404WMo3S8-9c2979187585 . . . . . 407
2.405WMo3Se8-05a06afa3b20 . . . . . 408
2.406WMo3Te8-323fb700d903 . . . . . 409
2.407WO2-94cfbb3f9284 . . . . . 410
2.408WS2-64090c9845f8 . . . . . 411
2.409WSe2-1cfbe6183886 . . . . . 412
2.410WTe2-3c87365bc48c . . . . . 413
2.411Y2Br6-6d976a1e88b3 . . . . . 414
2.412Y2Cl6-e9b63ba77c55 . . . . . 415
2.413Y2I6-d2a7ba833570 . . . . . 416
2.414ZnBr2-4718298eb660 . . . . . 417
2.415ZnBr2-553cb6a56984 . . . . . 418
2.416ZnCl2-1b7175e04416 . . . . . 419
2.417ZnCl2-62c6ee7a0a25 . . . . . 420
2.418ZnF2-a57169b6ba57 . . . . . 421
2.419ZnI2-701e1fc14b22 . . . . . 422
2.420ZnI2-ce0e9cd74bb3 . . . . . 423
2.421ZrBr2-7897c7cc2491 . . . . . 424
2.422ZrCl2-dc09b7c396eb . . . . . 425

2.423ZrHf3S8-9cbc09153aeb . . . 426
2.424ZrHf3Se8-b8fb10416122 . . . 427
2.425ZrHf3Te8-3663f526cdf1 . . . 428
2.426ZrI2-9c024b5a2e89 . . . 429
2.427ZrO2-24a8929c68ce . . . 430
2.428ZrO2-da3987f48688 . . . 431
2.429ZrS2-1a039e022308 . . . 432
2.430ZrS2-2e44a755e594 . . . 433
2.431ZrSe2-001dfe9a7fa2 . . . 434
2.432ZrSe2-f17029facf63 . . . 435
2.433ZrTe2-599f0c912458 . . . 436
2.434ZrTe2-f7ad606317e6 . . . 437
2.435ZrTi3S8-e6e376c80c43 . . . 438
2.436ZrTi3Se8-52a5e2b280d4 . . . 439

# 1 K-paths along high-symmetry lines

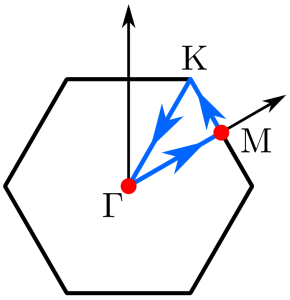


(a) Primitive hexagonal
$\Gamma \rightarrow \mathrm{M} \rightarrow \mathrm{K} \rightarrow \Gamma$

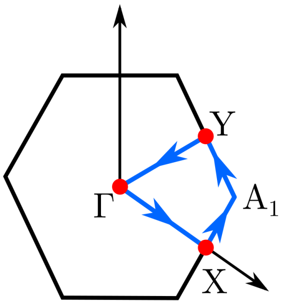


(b) Centred rectangular
$\Gamma \rightarrow \mathrm{X} \rightarrow \mathrm{A}_1 \rightarrow \mathrm{Y} \rightarrow \Gamma$

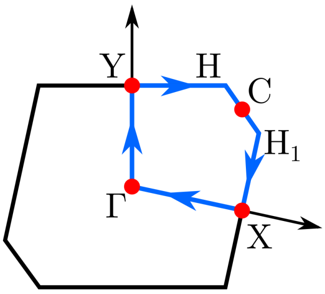


(c) Primitive oblique
$\Gamma \rightarrow \mathrm{Y} \rightarrow \mathrm{H} \rightarrow \mathrm{C} \rightarrow \mathrm{H}_1 \rightarrow \mathrm{X} \rightarrow \Gamma$

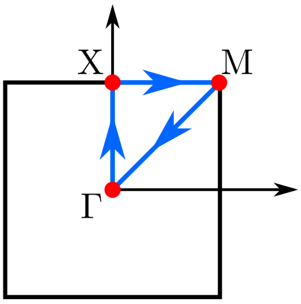


(d) Primitive square
$\mathrm{M} \rightarrow \Gamma \rightarrow \mathrm{X} \rightarrow \mathrm{M}$

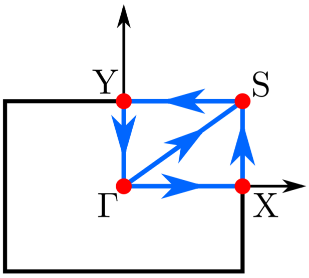


(e) Primitive rectangular
$\Gamma \rightarrow \mathrm{X} \rightarrow \mathrm{S} \rightarrow \mathrm{Y} \rightarrow \Gamma \rightarrow \mathrm{S}$

Figure 1: K-path along high-symmetry lines for the 5 possible 2D cristal lattices, first proposed by *W. Setyawan* and *S. Curtarolo* [1] and applied automatically by ASE [2], used in the band structure calculations in this work. Red circles indicate time-reversal invariant momentum (TRIM) k-points, which can be direcly identified for all Brillouin zones in 2 dimensions, and have reciprocal coordinates as $(0, 0)$, $(1/2, 0)$, $(0, 1/2)$ and $(1/2, 1/2)$ in all cases (when such coordinates in fact represent a high-symmetry k-point in the reciprocal cell). Any other high-symmetry k-point other than these ones ($K$, $A_1$, $H$ and $H_1$ in these cases) are treated as non-TRIM by the developed algorithm, in the sense that the possibility of having non-degenerate bands at these k-points is verified.

# 2 Materials data

## 2.1 Ag2Cl2-dd5f0964d63d

- **Formula**: Ag2Cl2
- **Structural Cluster**: AB-5
- **Band gap (PBE)**: 1.597 eV
- **Energy above convex hull (C2DB)**: 0.003 eV
- **Space group symbol**: $P1$
- **Space group number**: 1
- **Polar structure**: True

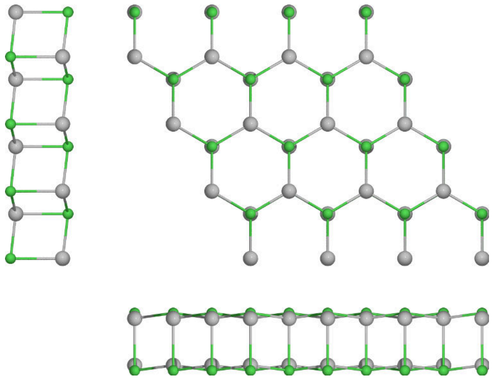

Figure 2: Structure representation

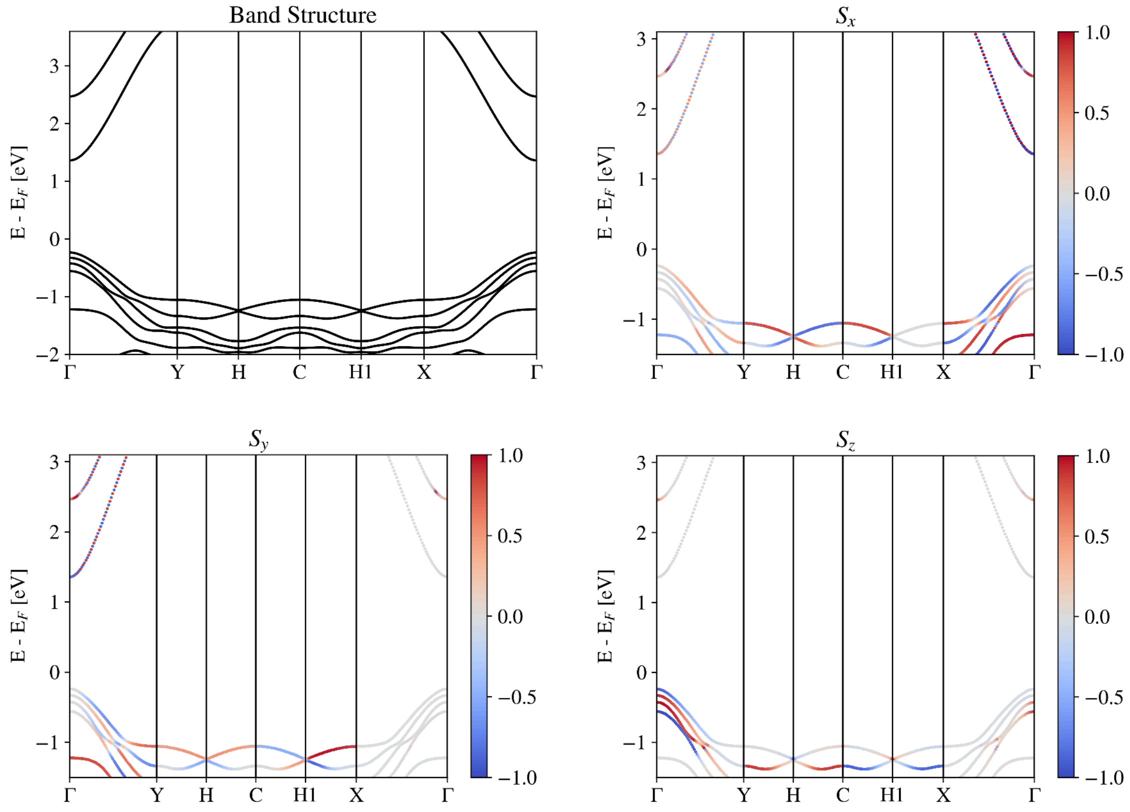


Figure 3: Band structure and spin polarization projections.

## 2.2 Ag2F2-44f6ed525a5a

- **Formula**: Ag2F2
- **Structural Cluster**: AB-0
- **Band gap (PBE)**: 0.52 eV
- **Energy above convex hull (C2DB)**: 0.048 eV
- **Space group symbol**: $P1$
- **Space group number**: 1
- **Polar structure**: True

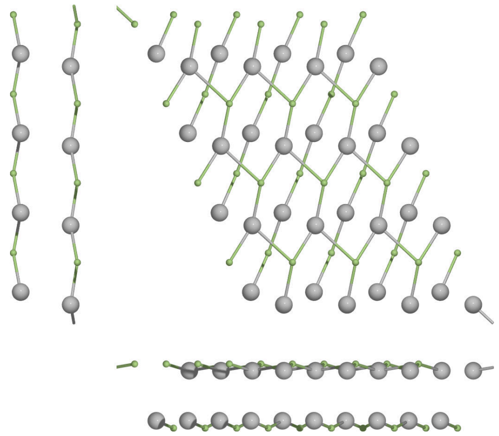

Figure 4: Structure representation

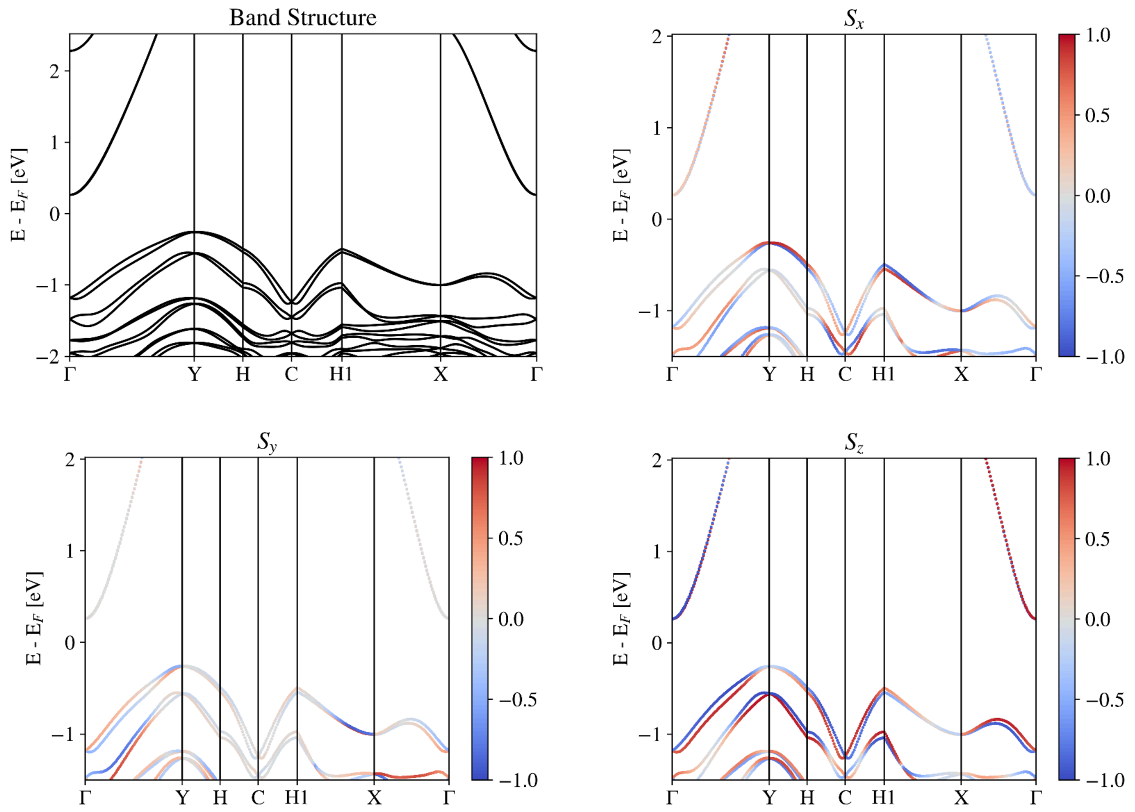


Figure 5: Band structure and spin polarization projections.

## 2.3 Ag2Se2-248fffac5959

- **Formula**: Ag2Se2
- **Structural Cluster**: AB-0
- **Band gap (PBE)**: 0.738 eV
- **Energy above convex hull (C2DB)**: 0.072 eV
- **Space group symbol**: *Pm*
- **Space group number**: 6
- **Polar structure**: True

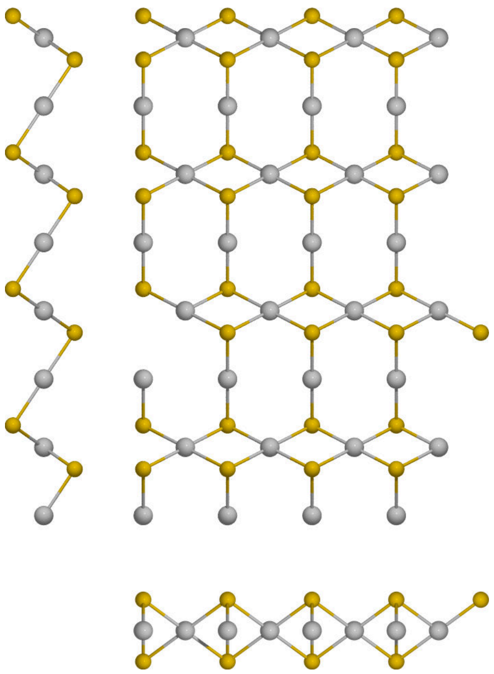

Figure 6: Structure representation

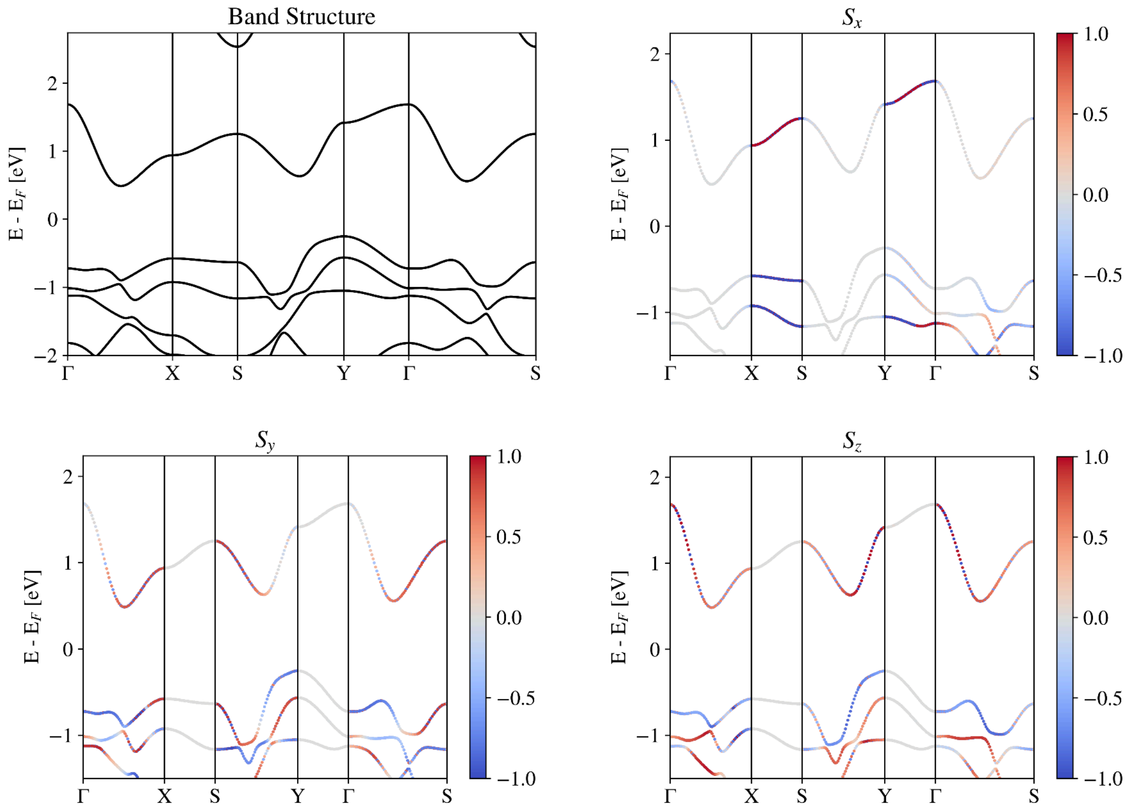


Figure 7: Band structure and spin polarization projections.

## 2.4 Ag2Te2-648de0a225fe

- **Formula**: Ag2Te2
- **Structural Cluster**: AB-0
- **Band gap (PBE)**: 0.484 eV
- **Energy above convex hull (C2DB)**: 0.102 eV
- **Space group symbol**: *Pm*
- **Space group number**: 6
- **Polar structure**: True

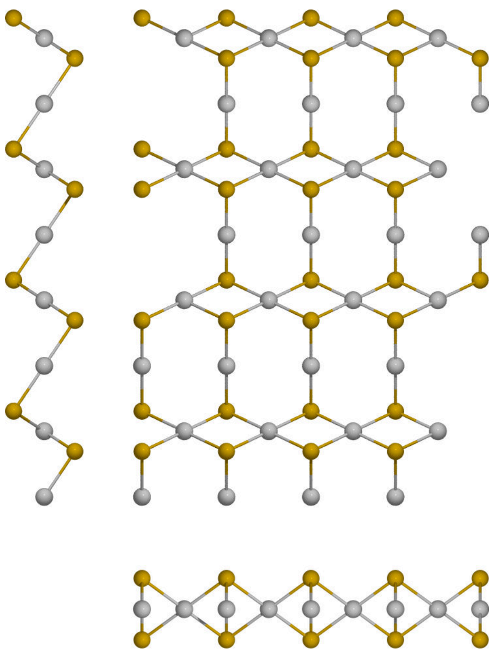

Figure 8: Structure representation

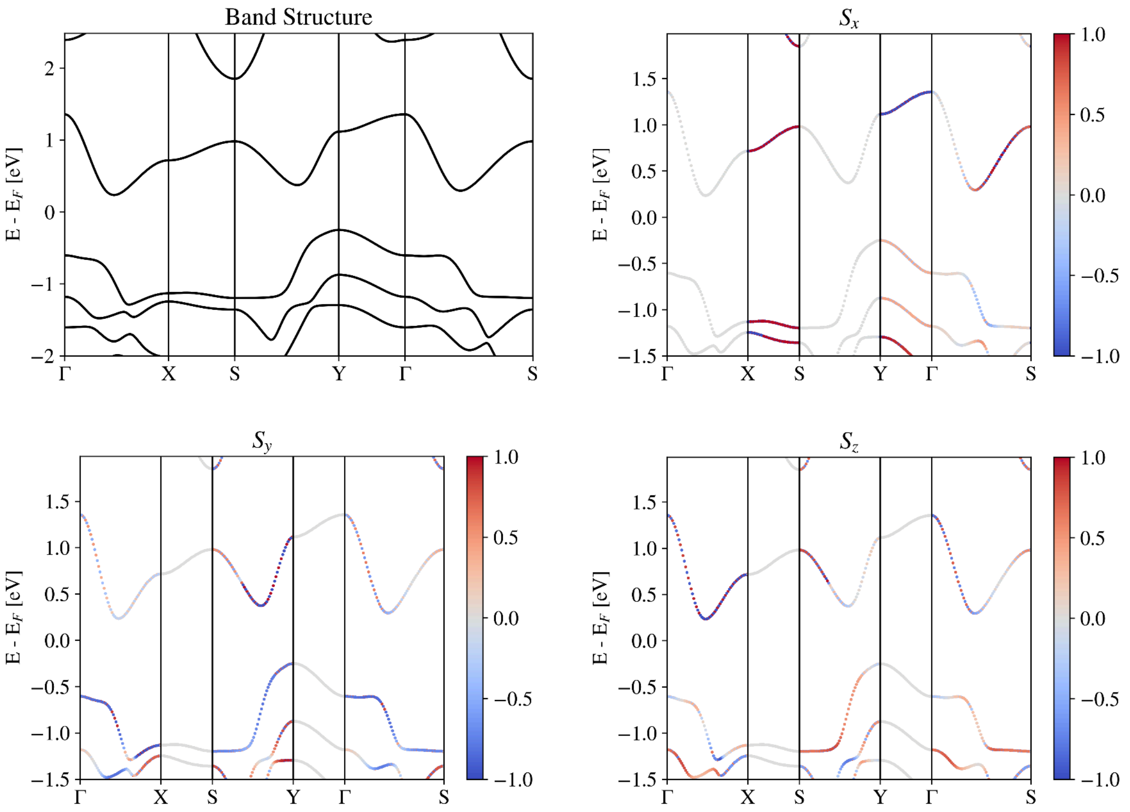


Figure 9: Band structure and spin polarization projections.

## 2.5 Al2Br6-fab8ea0c0979

- **Formula**: Al2Br6
- **Structural Cluster**: AB3-19
- **Band gap (PBE)**: 2.509 eV
- **Energy above convex hull (C2DB)**: 0.204 eV
- **Space group symbol**: $P321$
- **Space group number**: 150
- **Polar structure**: False

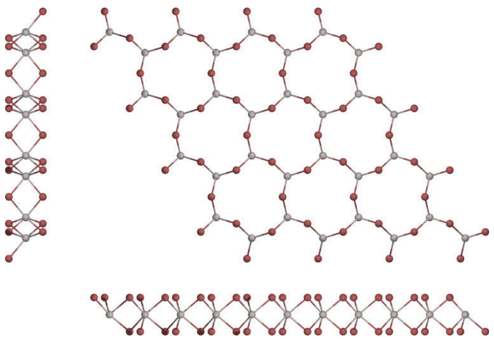

Figure 10: Structure representation

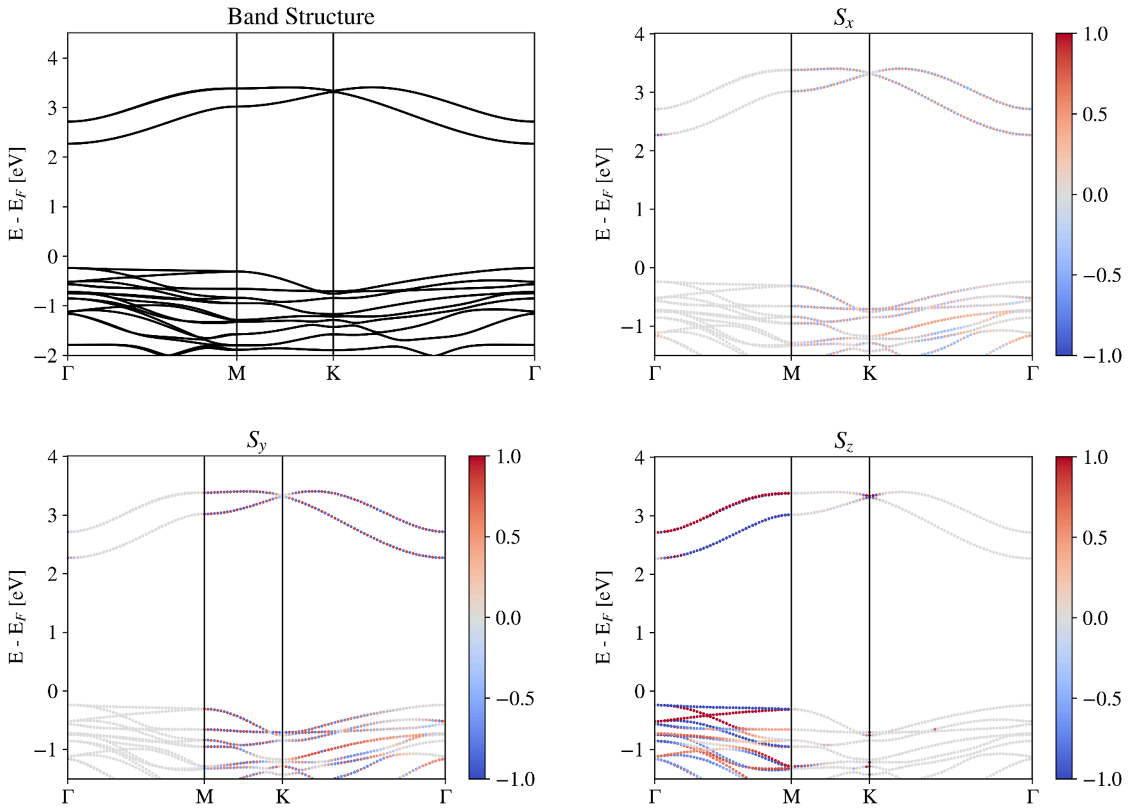


Figure 11: Band structure and spin polarization projections.

## 2.6 Al2Cl6-450a3fc3ff50

- **Formula**: Al2Cl6
- **Structural Cluster**: AB3-19
- **Band gap (PBE)**: 3.853 eV
- **Energy above convex hull (C2DB)**: 0.19 eV
- **Space group symbol**: $P\bar{6}2m$
- **Space group number**: 189
- **Polar structure**: False

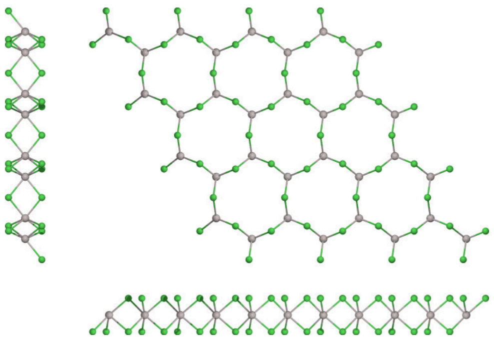

Figure 12: Structure representation

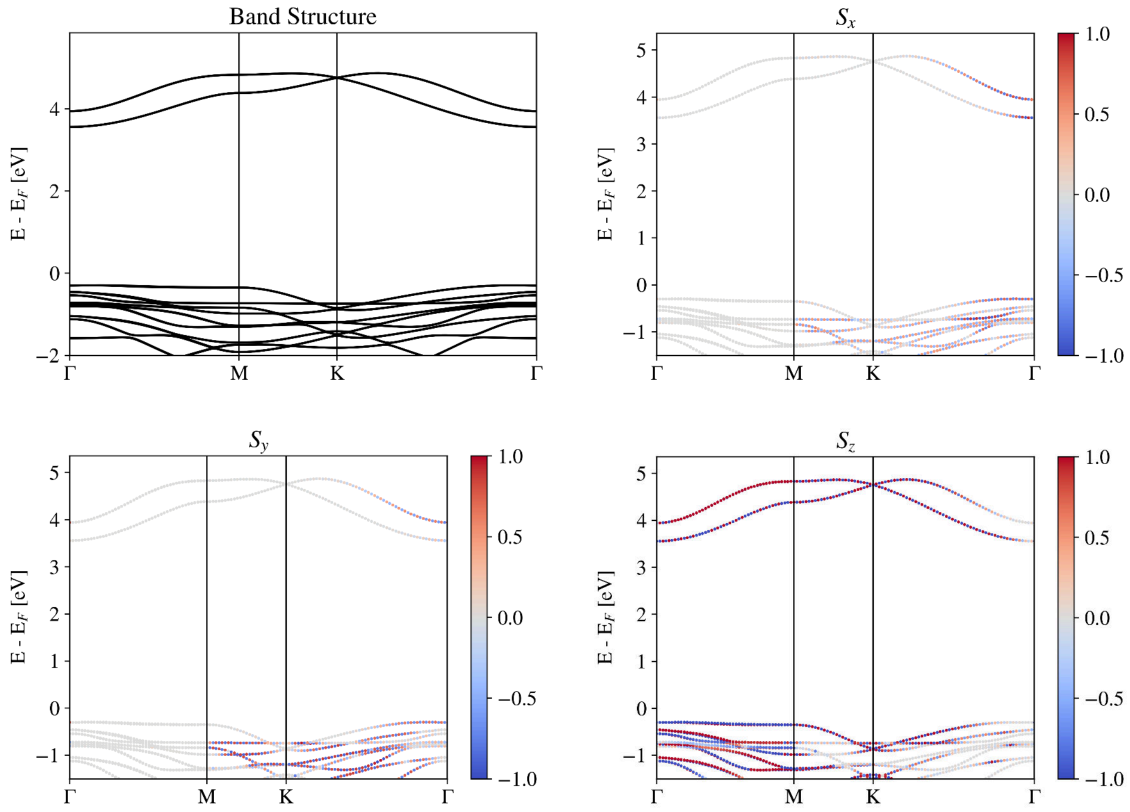


Figure 13: Band structure and spin polarization projections.

## 2.7 Al2I6-29ae3f5ef5a7

- **Formula**: Al2I6
- **Structural Cluster**: AB3-19
- **Band gap (PBE)**: 0.845 eV
- **Energy above convex hull (C2DB)**: 0.259 eV
- **Space group symbol**: $P\bar{6}2m$
- **Space group number**: 189
- **Polar structure**: False

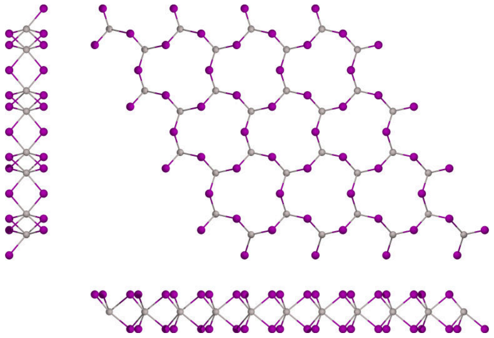

Figure 14: Structure representation

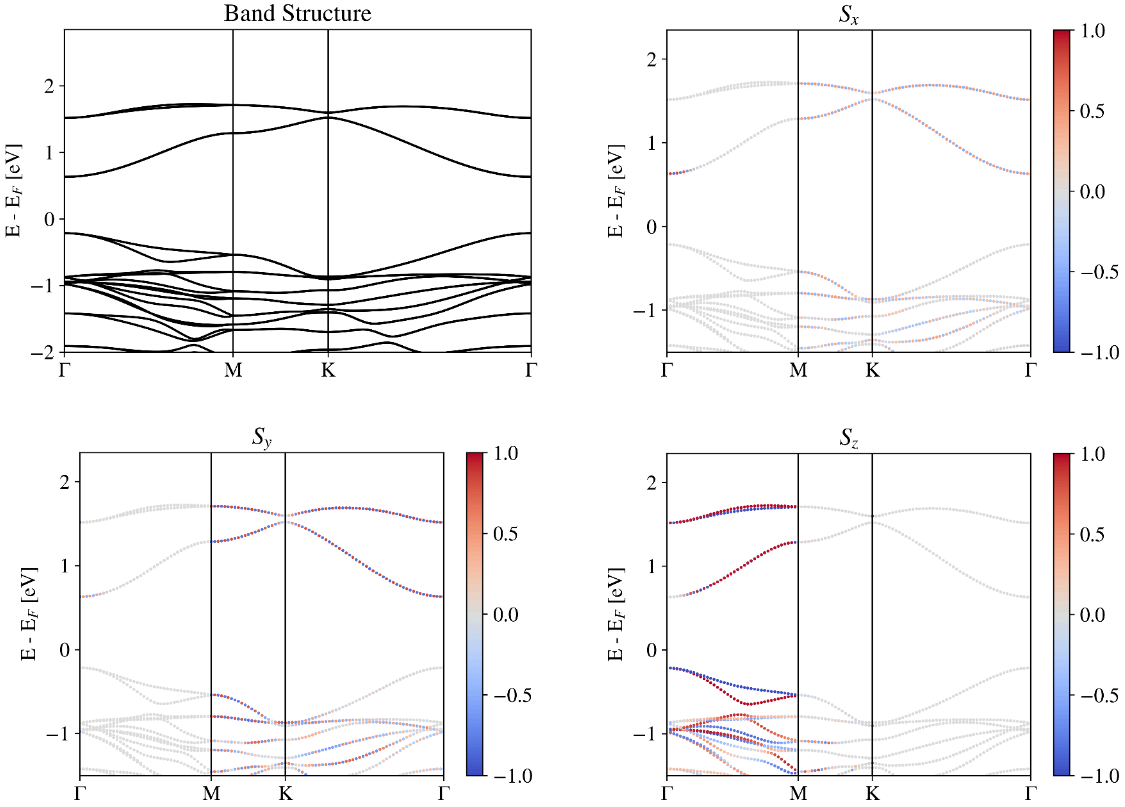


Figure 15: Band structure and spin polarization projections.

## 2.8 Al2O2-bce0ccee4eca

- **Formula**: Al2O2
- **Structural Cluster**: AB-5
- **Band gap (PBE)**: 1.324 eV
- **Energy above convex hull (C2DB)**: 0.237 eV
- **Space group symbol**: $P\bar{6}m2$
- **Space group number**: 187
- **Polar structure**: False

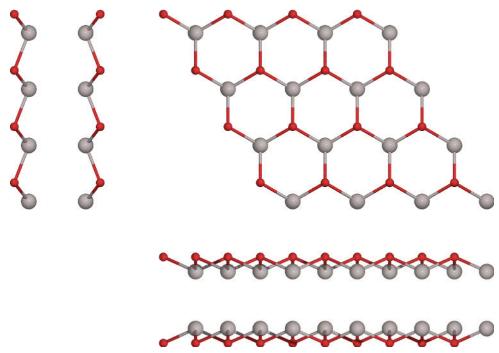

Figure 16: Structure representation

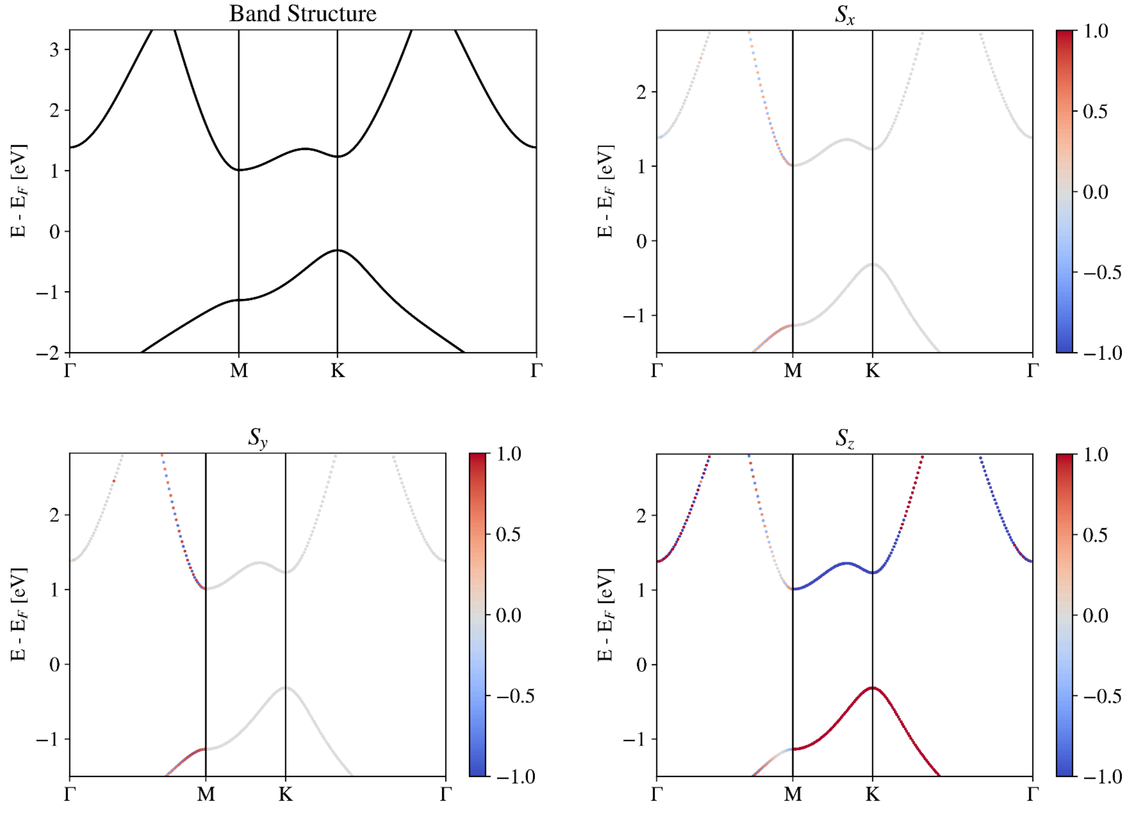


Figure 17: Band structure and spin polarization projections.

## 2.9 Al2P2S6-669d6f1af4d4

- **Formula**: Al2P2S6
- **Structural Cluster**: ABC3-16
- **Band gap (PBE)**: 1.301 eV
- **Energy above convex hull (C2DB)**: 0.083 eV
- **Space group symbol**: *P*1
- **Space group number**: 1
- **Polar structure**: True

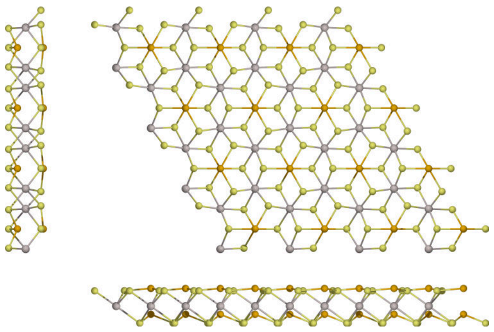

Figure 18: Structure representation

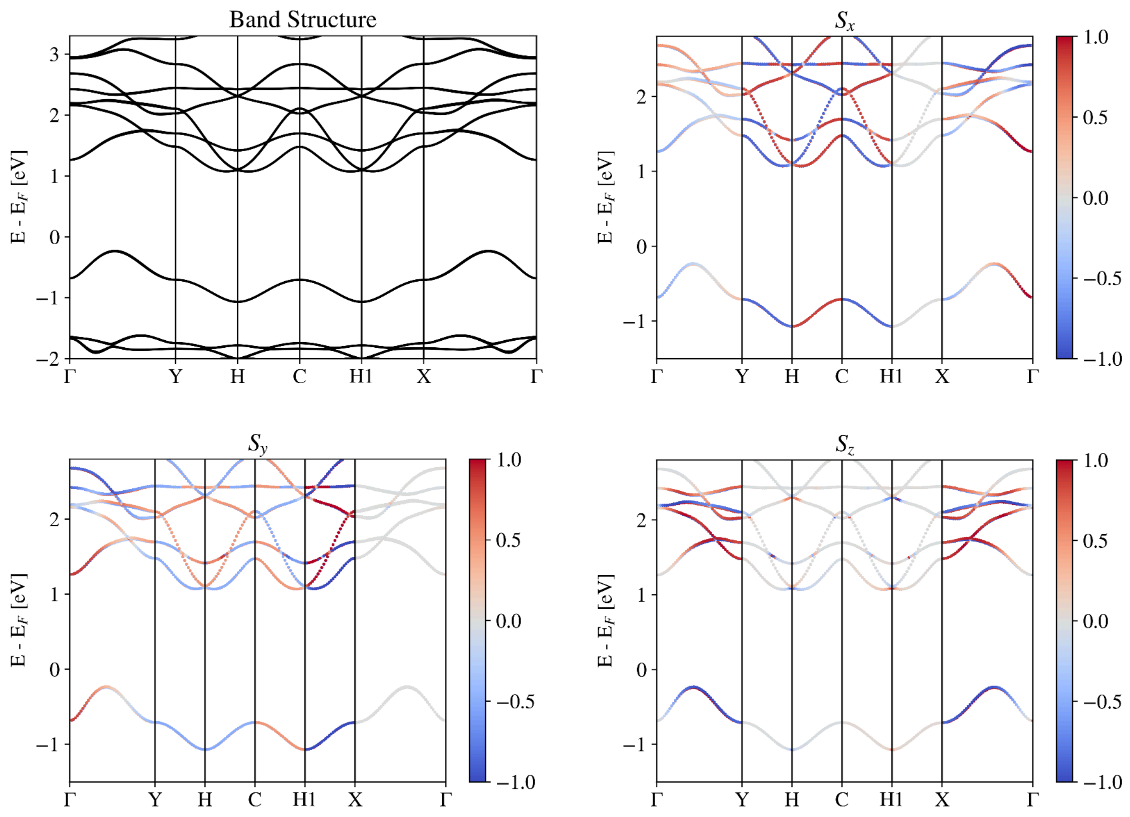


Figure 19: Band structure and spin polarization projections.

## 2.10 Al2P2Se6-90d84f697622

- **Formula**: Al2P2Se6
- **Structural Cluster**: ABC3-16
- **Band gap (PBE)**: 0.607 eV
- **Energy above convex hull (C2DB)**: 0.067 eV
- **Space group symbol**: $P1$
- **Space group number**: 1
- **Polar structure**: True

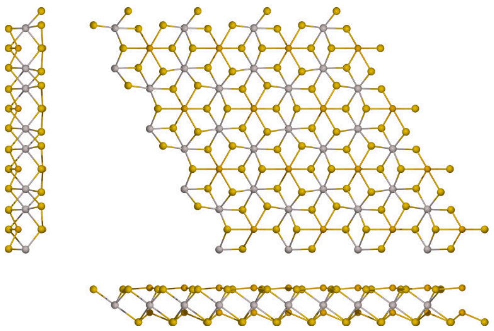

Figure 20: Structure representation

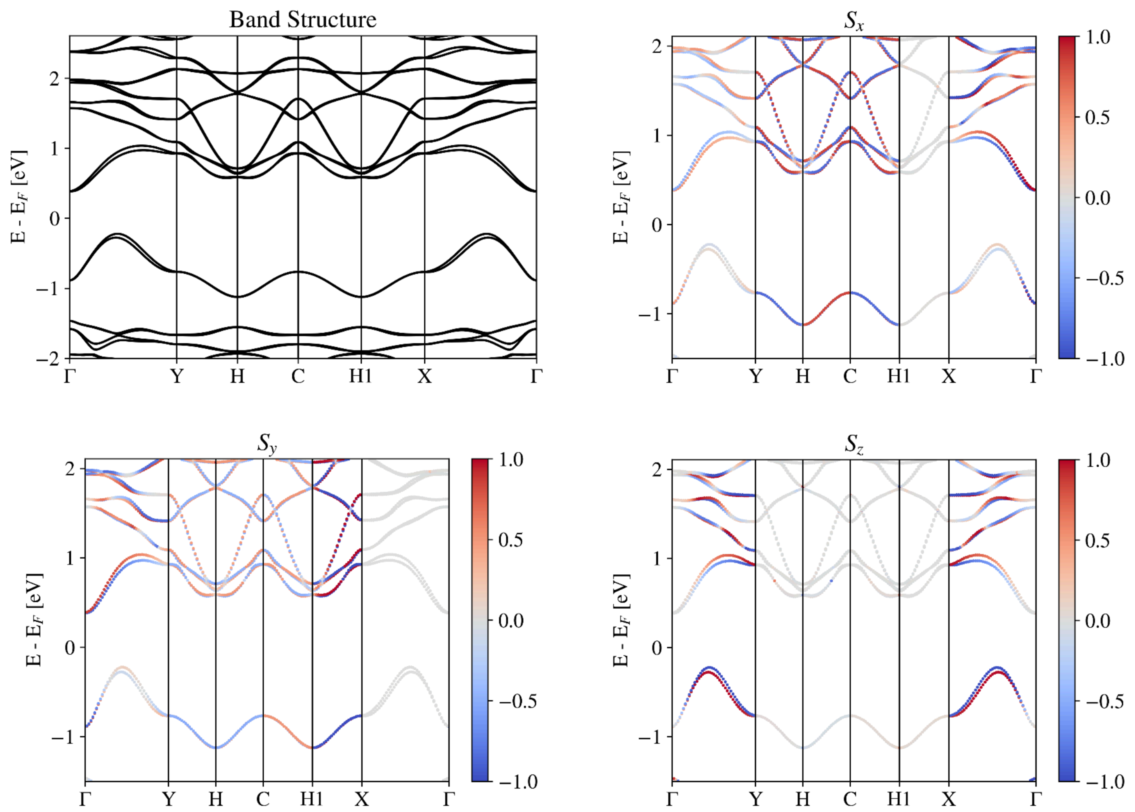


Figure 21: Band structure and spin polarization projections.

### 2.11 Al2S2-f9df9f4a5c34

- **Formula**: Al2S2
- **Structural Cluster**: AB-5
- **Band gap (PBE)**: 2.099 eV
- **Energy above convex hull (C2DB)**: 0.005 eV
- **Space group symbol**: $P\bar{6}m2$
- **Space group number**: 187
- **Polar structure**: False

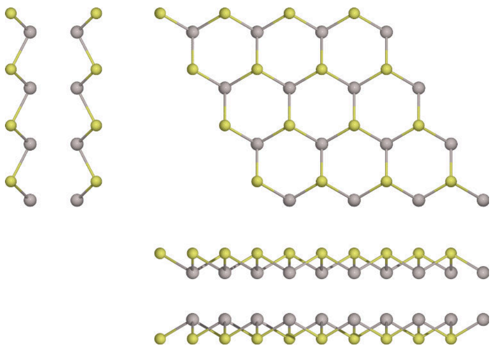

Figure 22: Structure representation

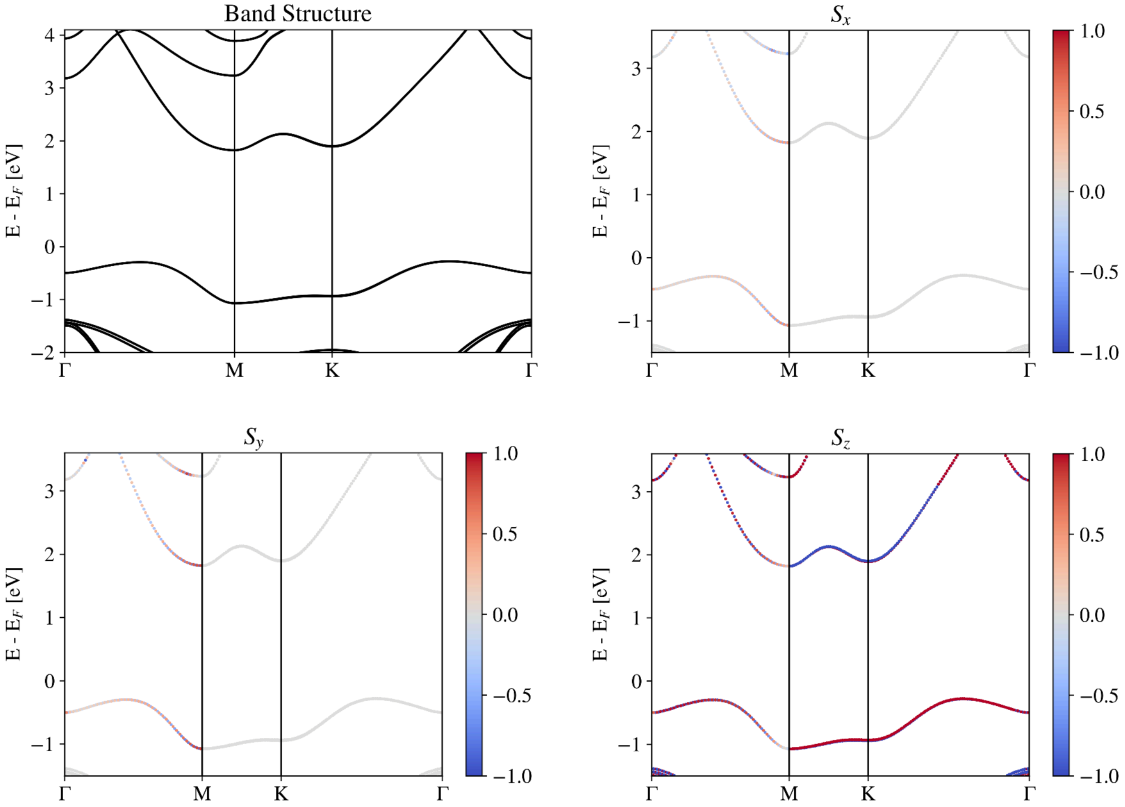


Figure 23: Band structure and spin polarization projections.

## 2.12 Al2Se2-129a514b51ad

- **Formula**: Al2Se2
- **Structural Cluster**: AB-5
- **Band gap (PBE)**: 1.997 eV
- **Energy above convex hull (C2DB)**: 0.0 eV
- **Space group symbol**: $P\bar{6}m2$
- **Space group number**: 187
- **Polar structure**: False

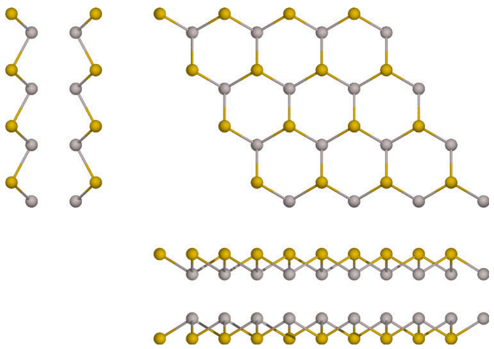

Figure 24: Structure representation

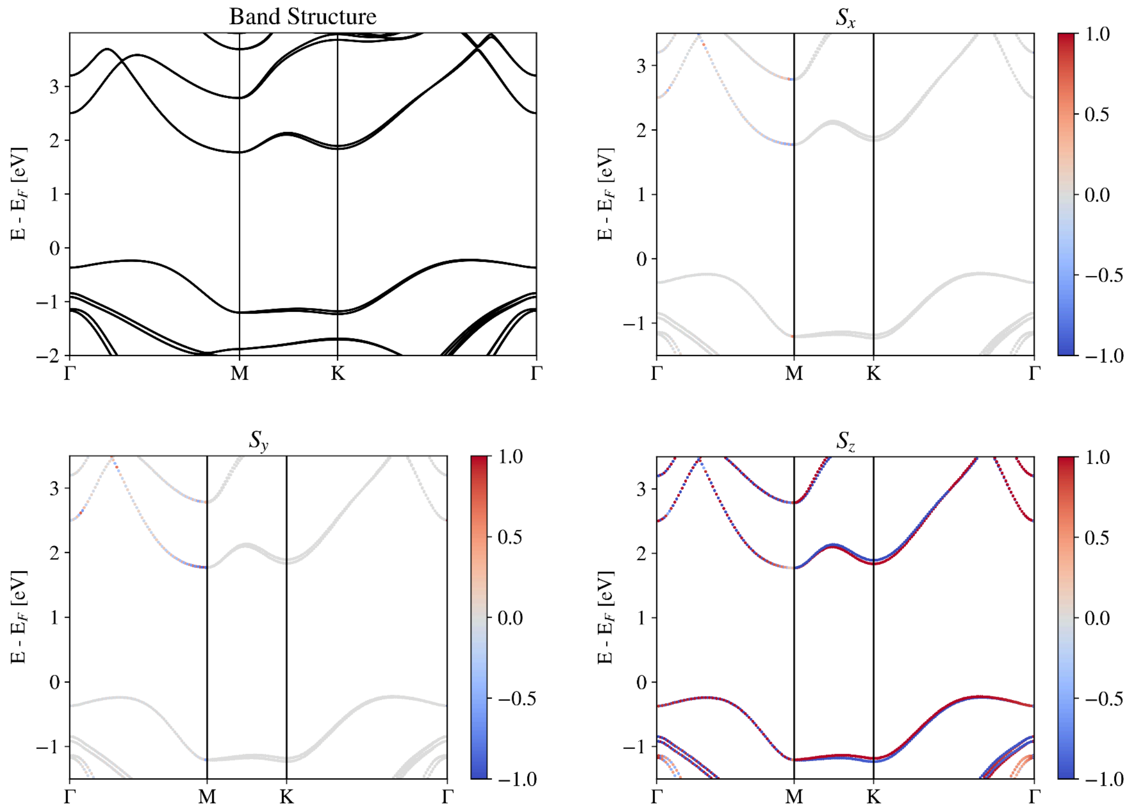


Figure 25: Band structure and spin polarization projections.

### 2.13 Al2Te2-e54041554385

- **Formula**: Al2Te2
- **Structural Cluster**: AB-5
- **Band gap (PBE)**: 1.763 eV
- **Energy above convex hull (C2DB)**: 0.0 eV
- **Space group symbol**: $P\bar{6}m2$
- **Space group number**: 187
- **Polar structure**: False

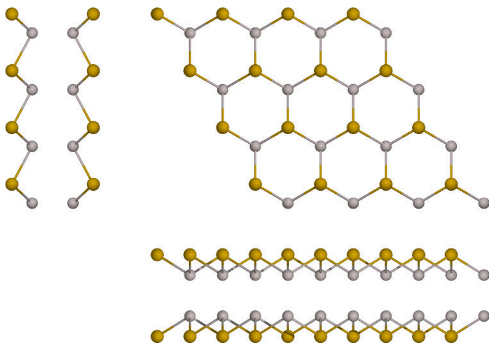

Figure 26: Structure representation

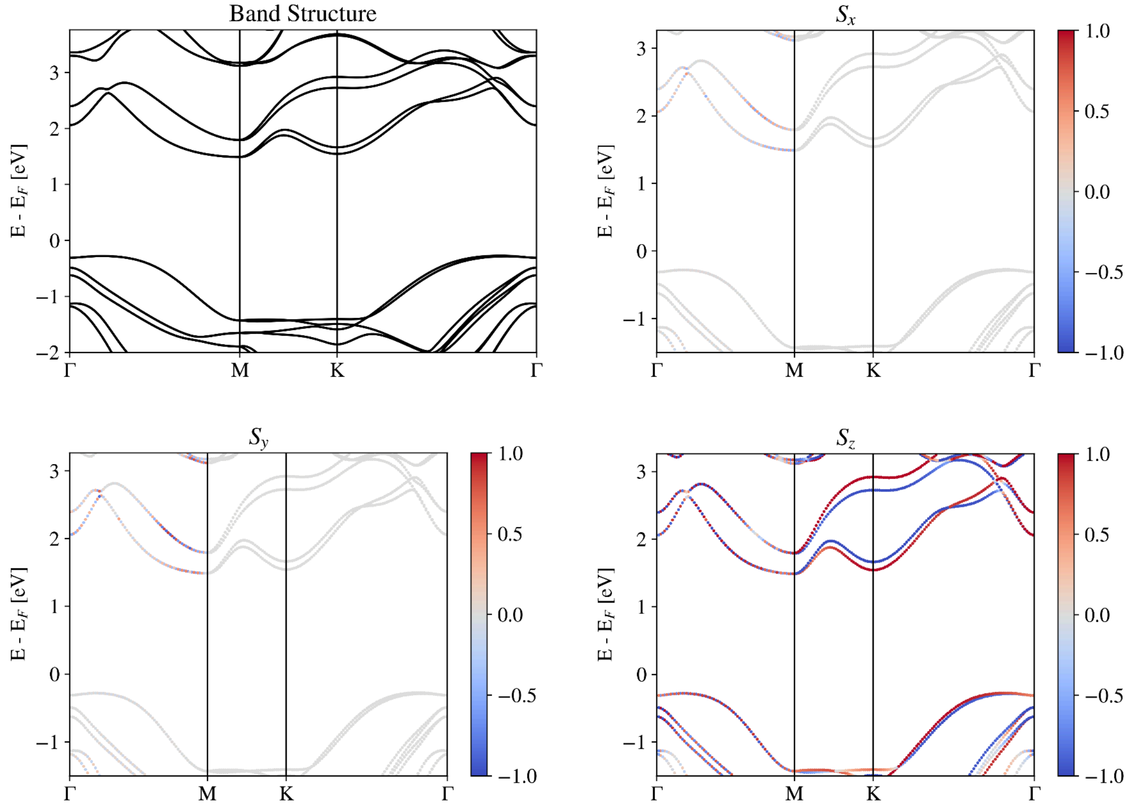


Figure 27: Band structure and spin polarization projections.

## 2.14 AlAs-814ae25a188e

- **Formula**: AlAs
- **Structural Cluster**: AB-2
- **Band gap (PBE)**: 1.241 eV
- **Energy above convex hull (C2DB)**: 0.522 eV
- **Space group symbol**: $P\bar{6}m2$
- **Space group number**: 187
- **Polar structure**: False

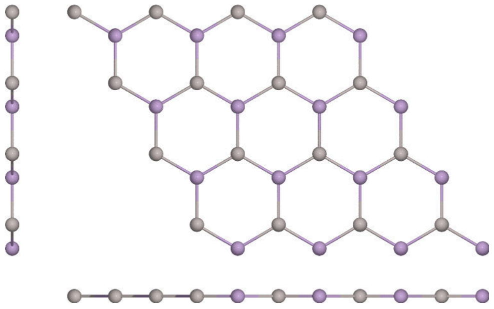

Figure 28: Structure representation

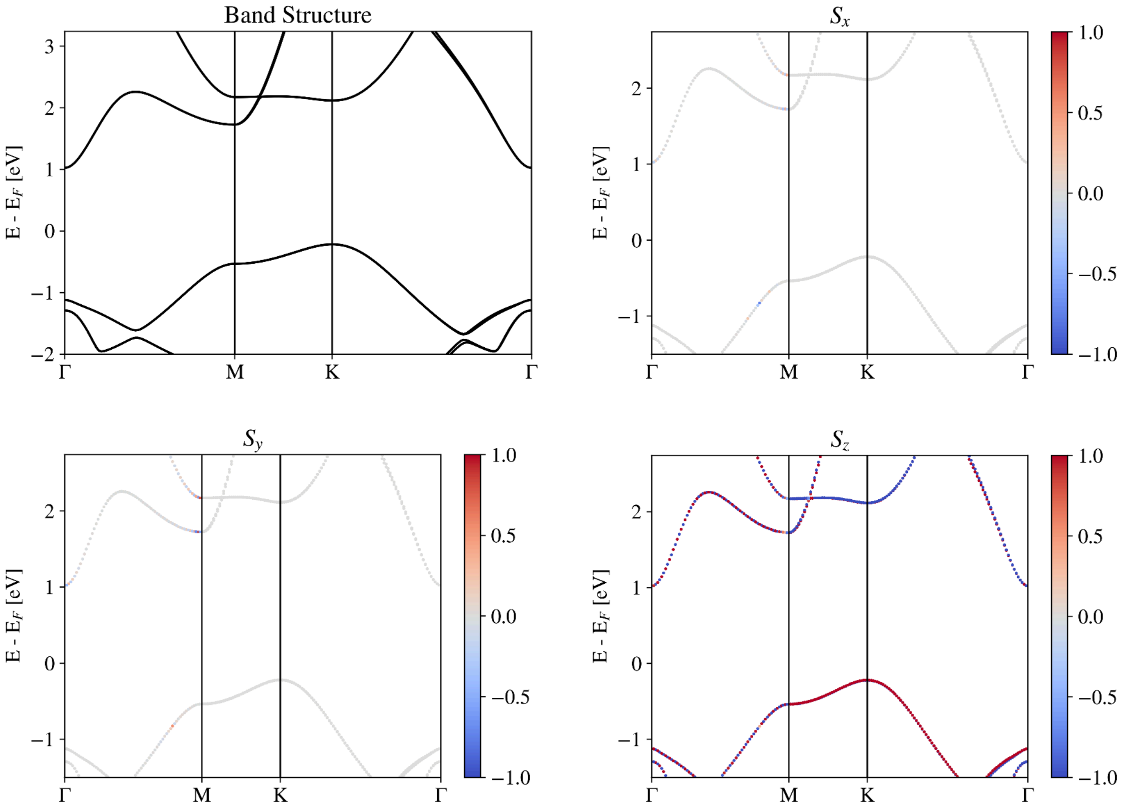


Figure 29: Band structure and spin polarization projections.

## 2.15 AlN-4fcd8dcb9c90

- **Formula**: AlN
- **Structural Cluster**: AB-2
- **Band gap (PBE)**: 2.884 eV
- **Energy above convex hull (C2DB)**: 0.512 eV
- **Space group symbol**: $P\bar{6}m2$
- **Space group number**: 187
- **Polar structure**: False

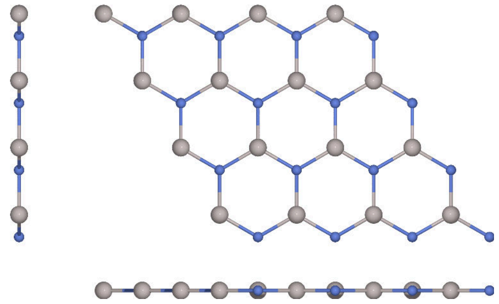

Figure 30: Structure representation

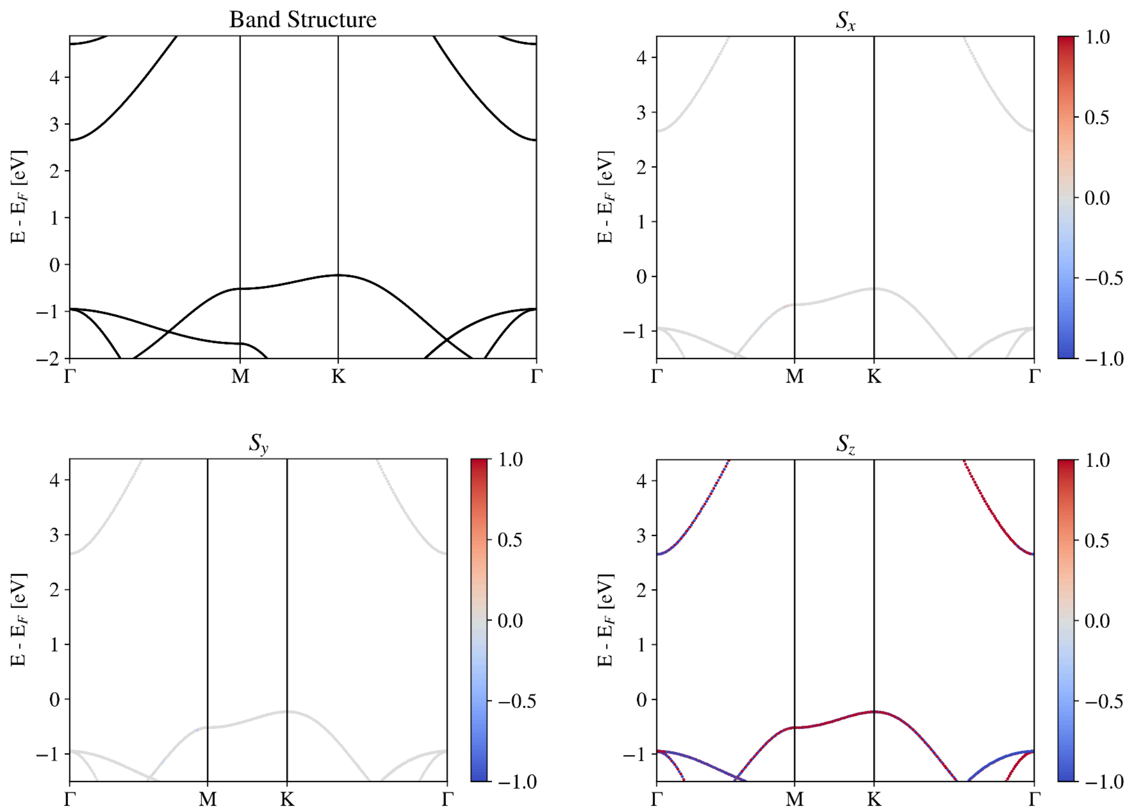


Figure 31: Band structure and spin polarization projections.

### 2.16 AlSb-1734deee2ac1

- **Formula**: AlSb
- **Structural Cluster**: AB-2
- **Band gap (PBE)**: 1.447 eV
- **Energy above convex hull (C2DB)**: 0.475 eV
- **Space group symbol**: $P3m1$
- **Space group number**: 156
- **Polar structure**: True

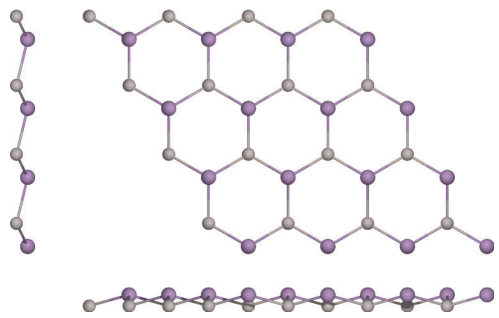

Figure 32: Structure representation

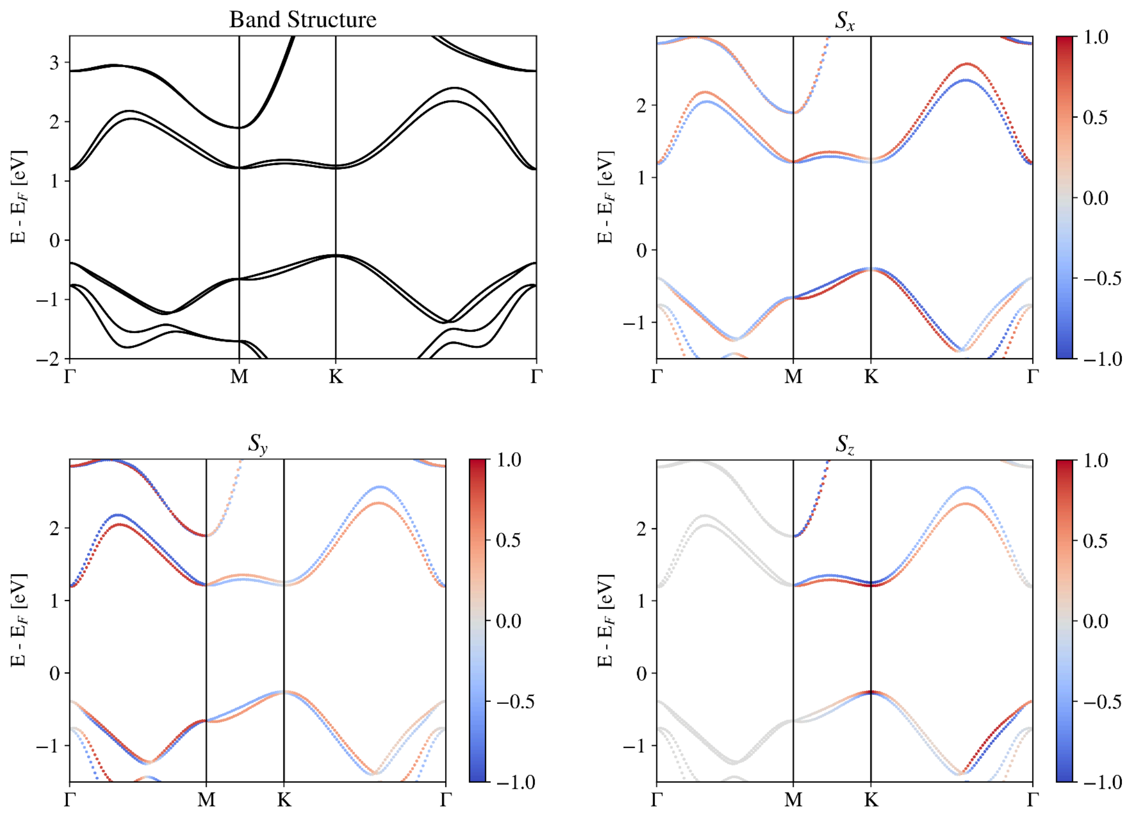


Figure 33: Band structure and spin polarization projections.

## 2.17 AsB-b6e76caa350b

- **Formula**: AsB
- **Structural Cluster**: AB-2
- **Band gap (PBE)**: 0.752 eV
- **Energy above convex hull (C2DB)**: 0.467 eV
- **Space group symbol**: $P\bar{6}m2$
- **Space group number**: 187
- **Polar structure**: False

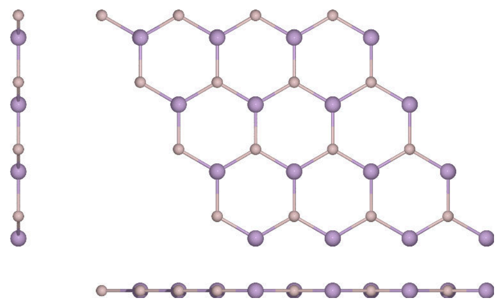

Figure 34: Structure representation

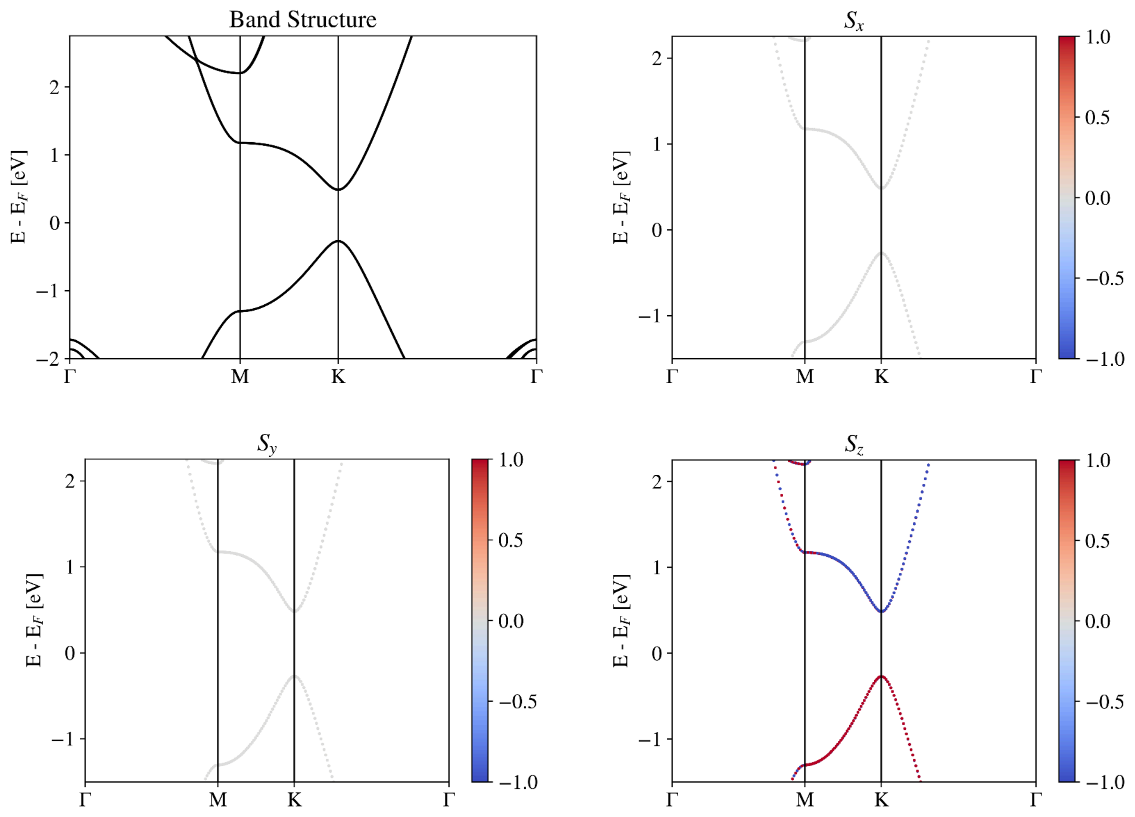


Figure 35: Band structure and spin polarization projections.

## 2.18 AsBiCr-b299416bff28

- **Formula**: AsBiCr
- **Structural Cluster**: ABC-4
- **Band gap (PBE)**: 0.037 eV
- **Energy above convex hull (C2DB)**: 0.461 eV
- **Space group symbol**: $P3m1$
- **Space group number**: 156
- **Polar structure**: True

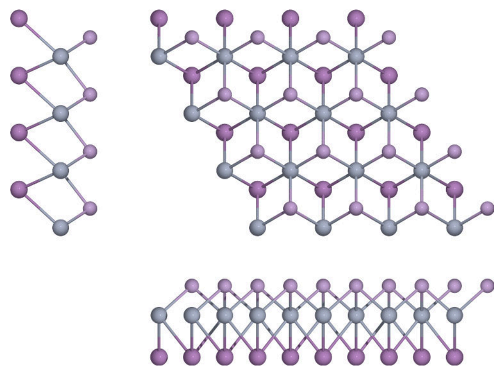

Figure 36: Structure representation

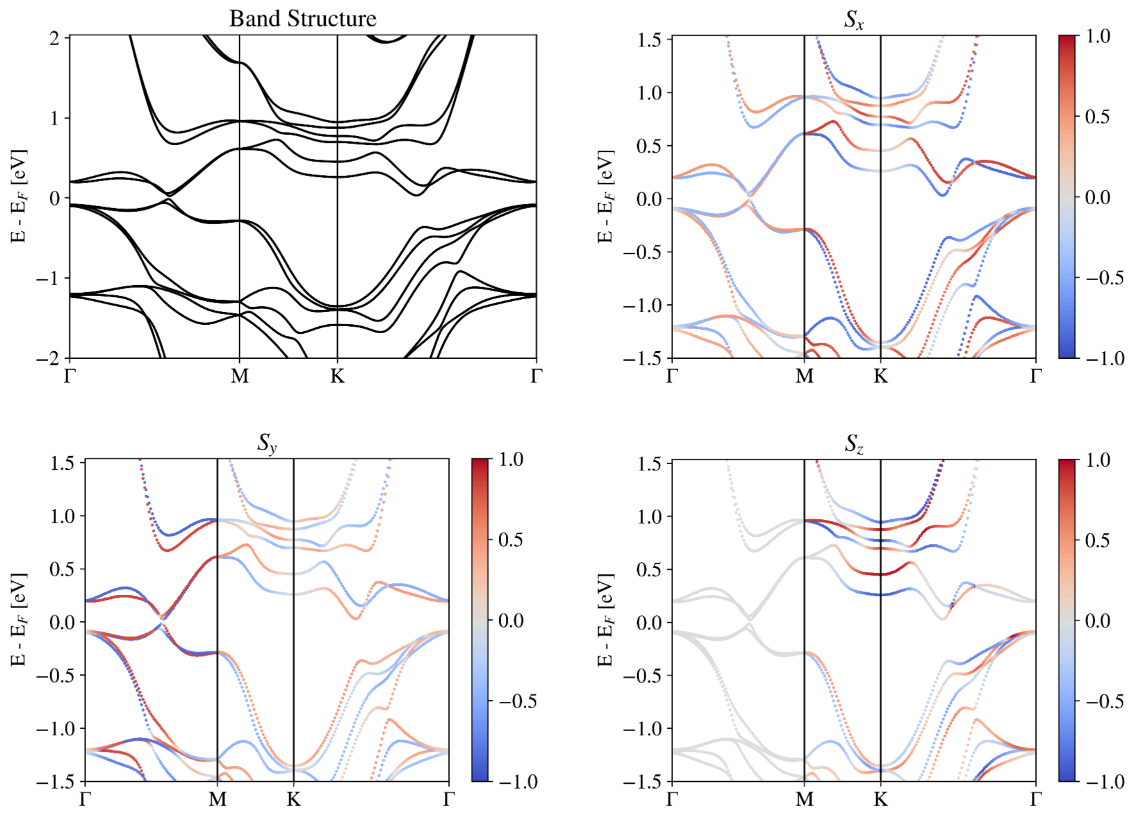


Figure 37: Band structure and spin polarization projections.

## 2.19 AsBrS-1dcd471c2288

- **Formula**: AsBrS
- **Structural Cluster**: ABC-3
- **Band gap (PBE)**: 1.38 eV
- **Energy above convex hull (C2DB)**: 0.034 eV
- **Space group symbol**: $P3m1$
- **Space group number**: 156
- **Polar structure**: True

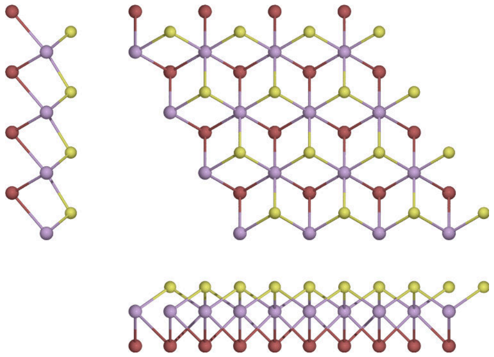

Figure 38: Structure representation

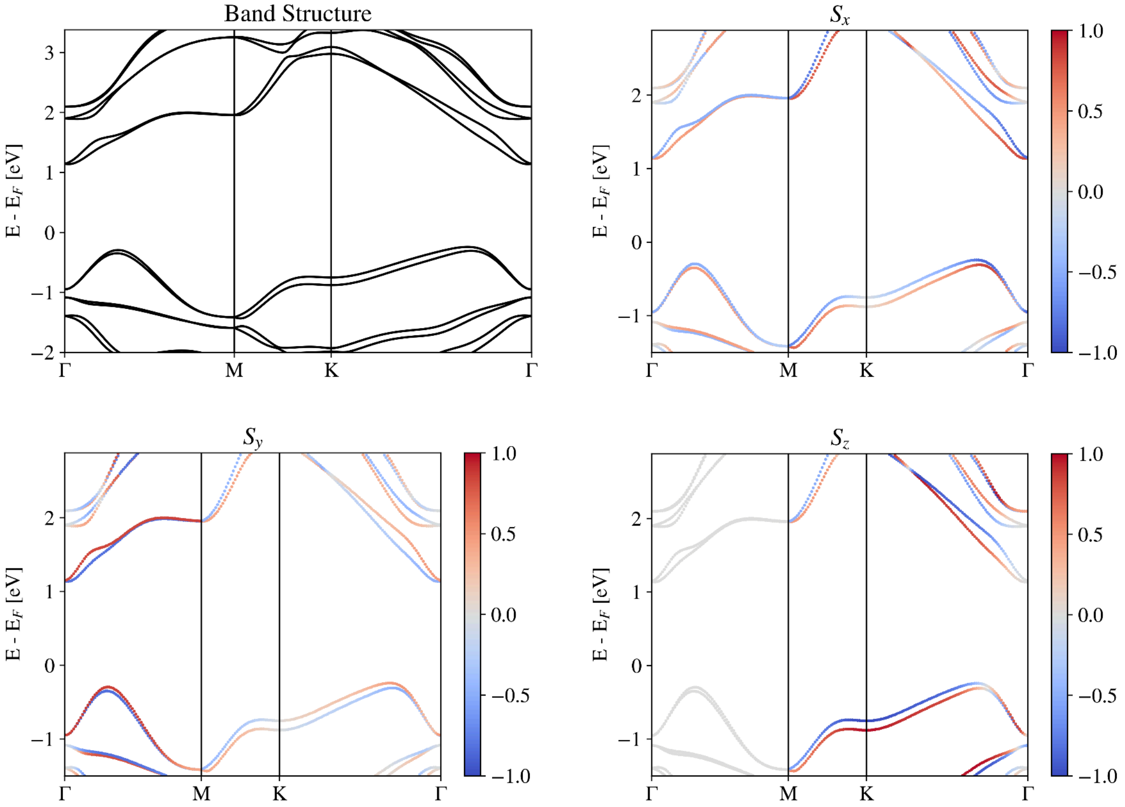


Figure 39: Band structure and spin polarization projections.

## 2.20 AsBrS-d9f4d4011670

- **Formula**: AsBrS
- **Structural Cluster**: ABC-4
- **Band gap (PBE)**: 1.425 eV
- **Energy above convex hull (C2DB)**: 0.201 eV
- **Space group symbol**: $P3m1$
- **Space group number**: 156
- **Polar structure**: True

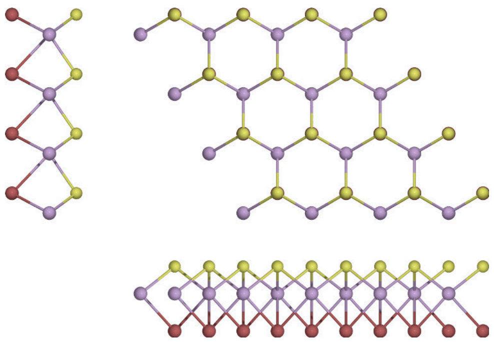

Figure 40: Structure representation

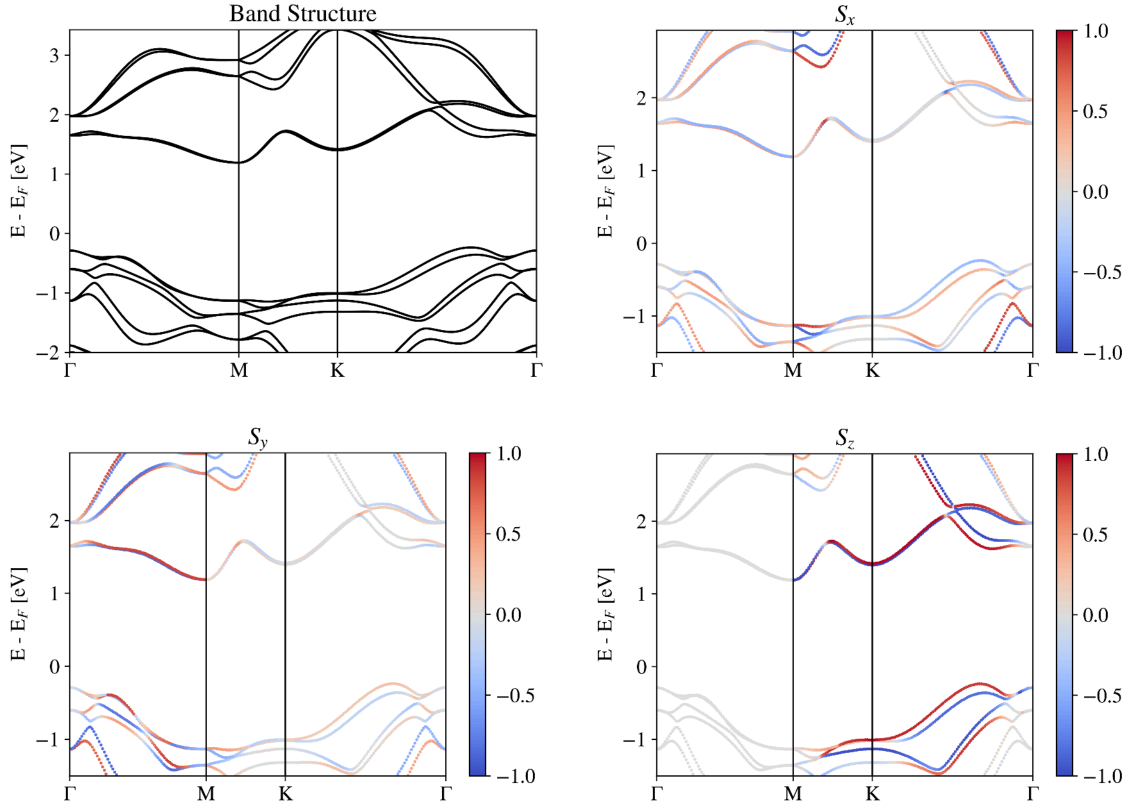


Figure 41: Band structure and spin polarization projections.

## 2.21 AsBrSe-206b9dcf2af6

- **Formula**: AsBrSe
- **Structural Cluster**: ABC-4
- **Band gap (PBE)**: 1.49 eV
- **Energy above convex hull (C2DB)**: 0.161 eV
- **Space group symbol**: $P3m1$
- **Space group number**: 156
- **Polar structure**: True

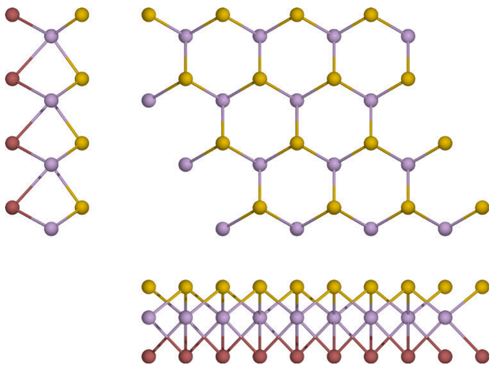

Figure 42: Structure representation

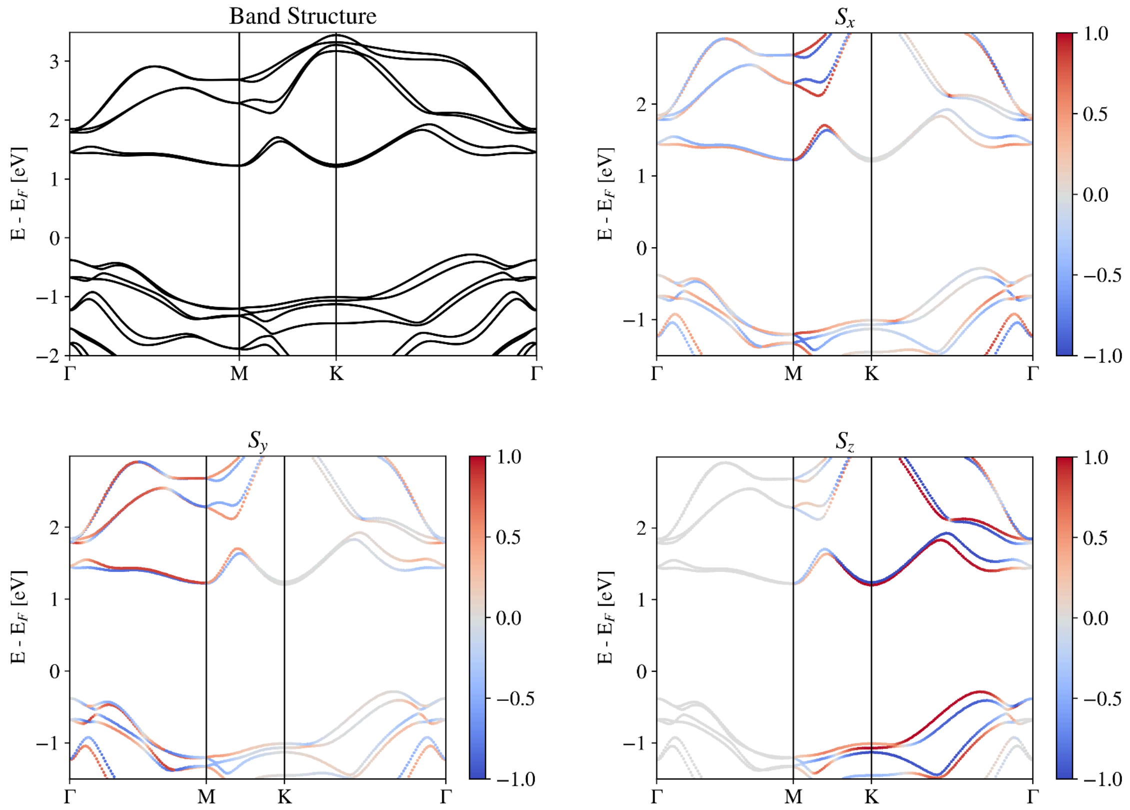


Figure 43: Band structure and spin polarization projections.

## 2.22 AsBrSe-989f469f06bd

- **Formula**: AsBrSe
- **Structural Cluster**: ABC-3
- **Band gap (PBE)**: 1.212 eV
- **Energy above convex hull (C2DB)**: 0.0 eV
- **Space group symbol**: $P3m1$
- **Space group number**: 156
- **Polar structure**: True

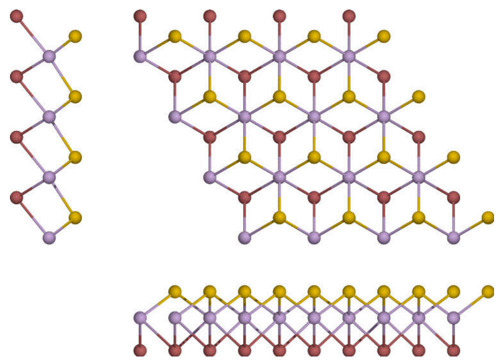

Figure 44: Structure representation

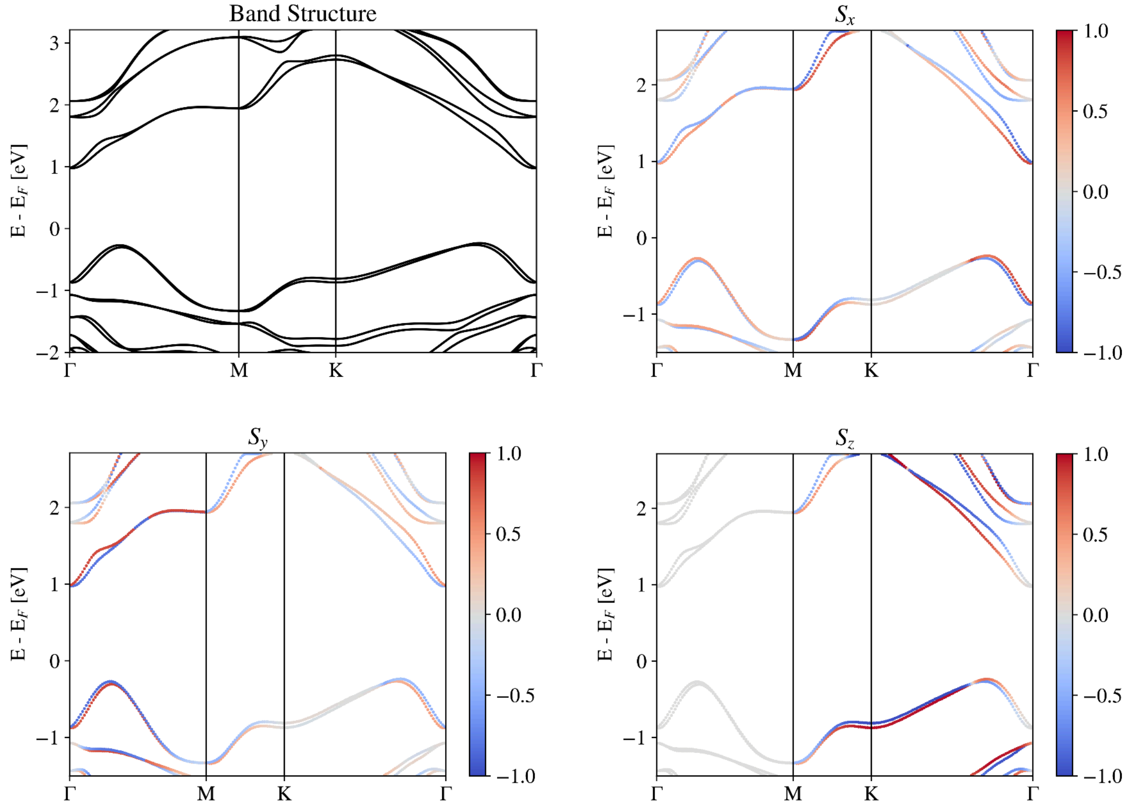


Figure 45: Band structure and spin polarization projections.

## 2.23 AsBrTe-64921449e408

- **Formula**: AsBrTe
- **Structural Cluster**: ABC-3
- **Band gap (PBE)**: 1.253 eV
- **Energy above convex hull (C2DB)**: 0.0 eV
- **Space group symbol**: $P3m1$
- **Space group number**: 156
- **Polar structure**: True

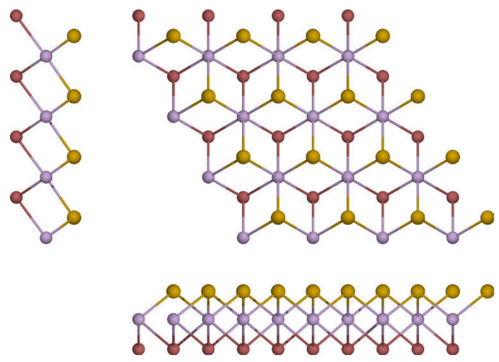

Figure 46: Structure representation

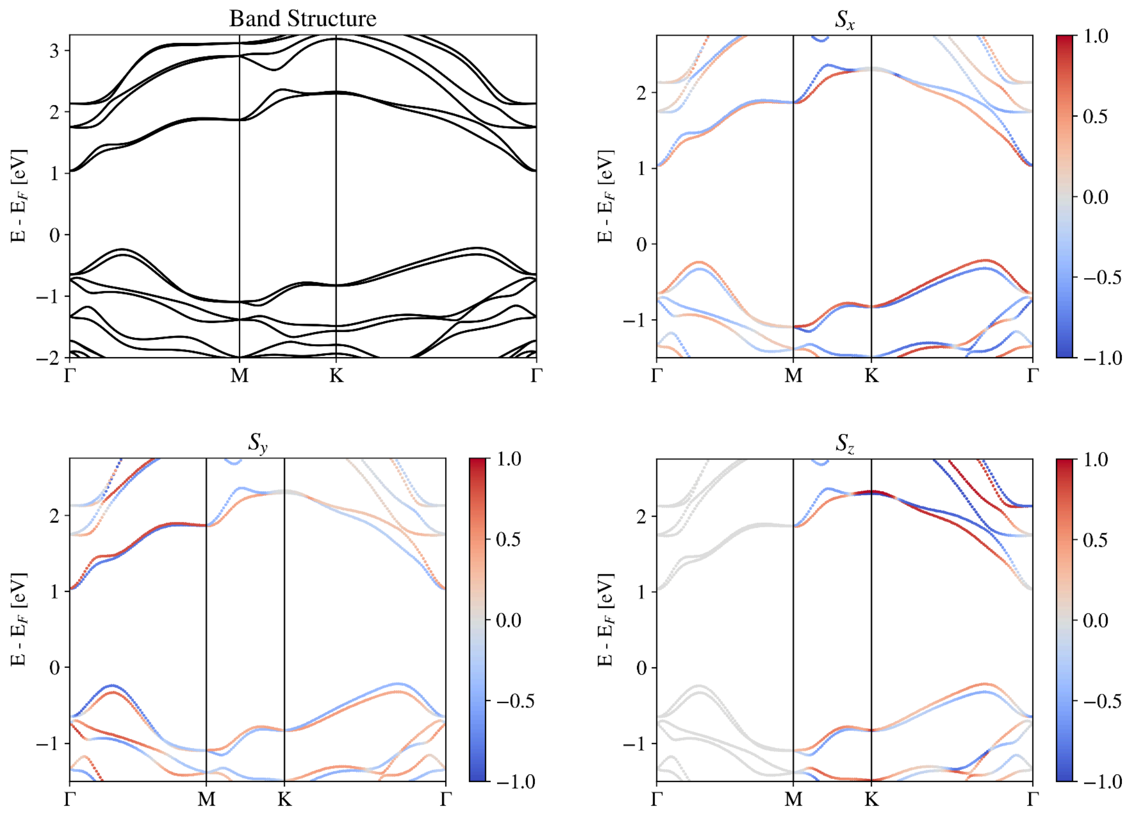


Figure 47: Band structure and spin polarization projections.

## 2.24 AsBrTe-671e6de2497a

- **Formula**: AsBrTe
- **Structural Cluster**: ABC-4
- **Band gap (PBE)**: 1.098 eV
- **Energy above convex hull (C2DB)**: 0.163 eV
- **Space group symbol**: $P3m1$
- **Space group number**: 156
- **Polar structure**: True

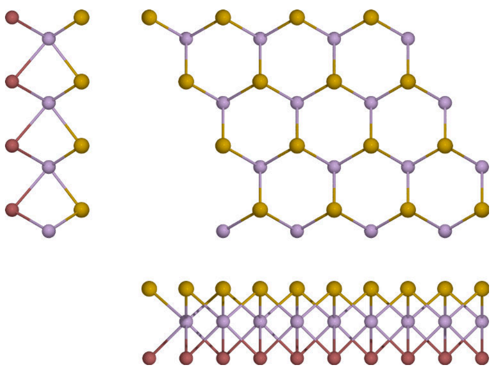

Figure 48: Structure representation

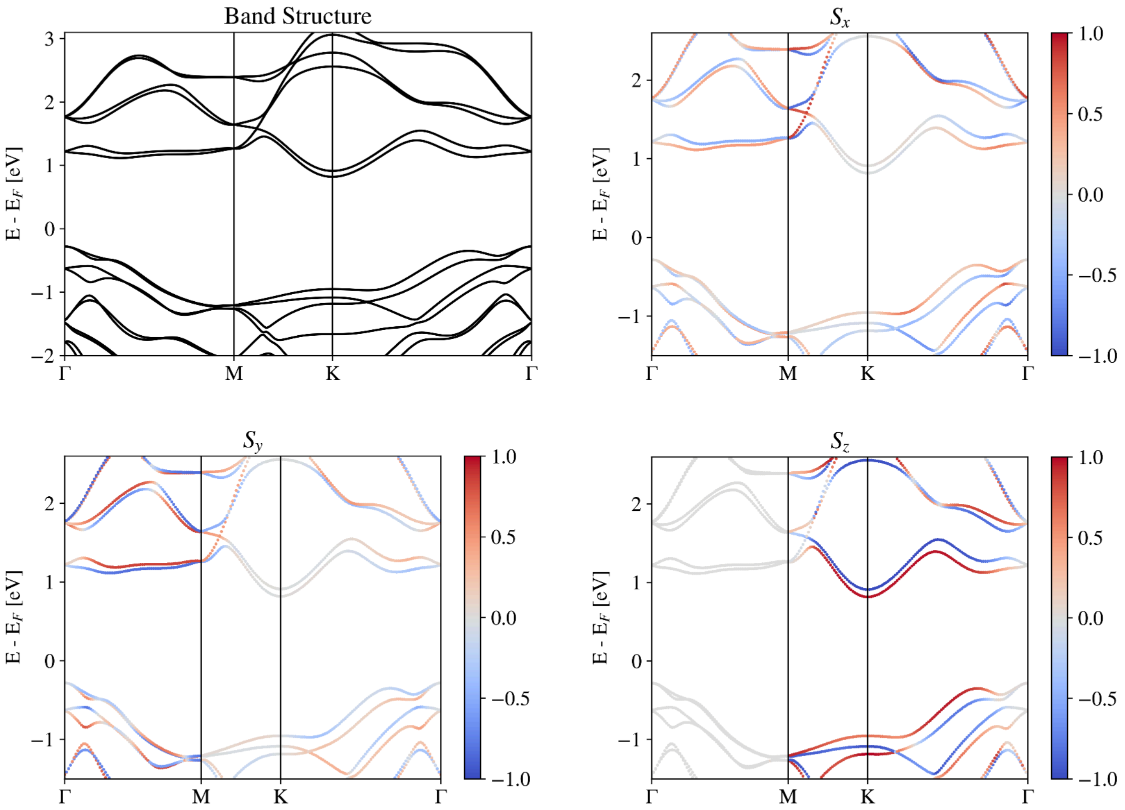


Figure 49: Band structure and spin polarization projections.

## 2.25 AsClS-0fd6ab210774

- **Formula**: AsClS
- **Structural Cluster**: ABC-4
- **Band gap (PBE)**: 1.732 eV
- **Energy above convex hull (C2DB)**: 0.224 eV
- **Space group symbol**: *P*3*m*1
- **Space group number**: 156
- **Polar structure**: True

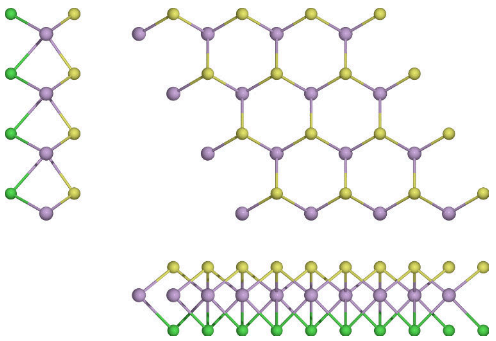

Figure 50: Structure representation

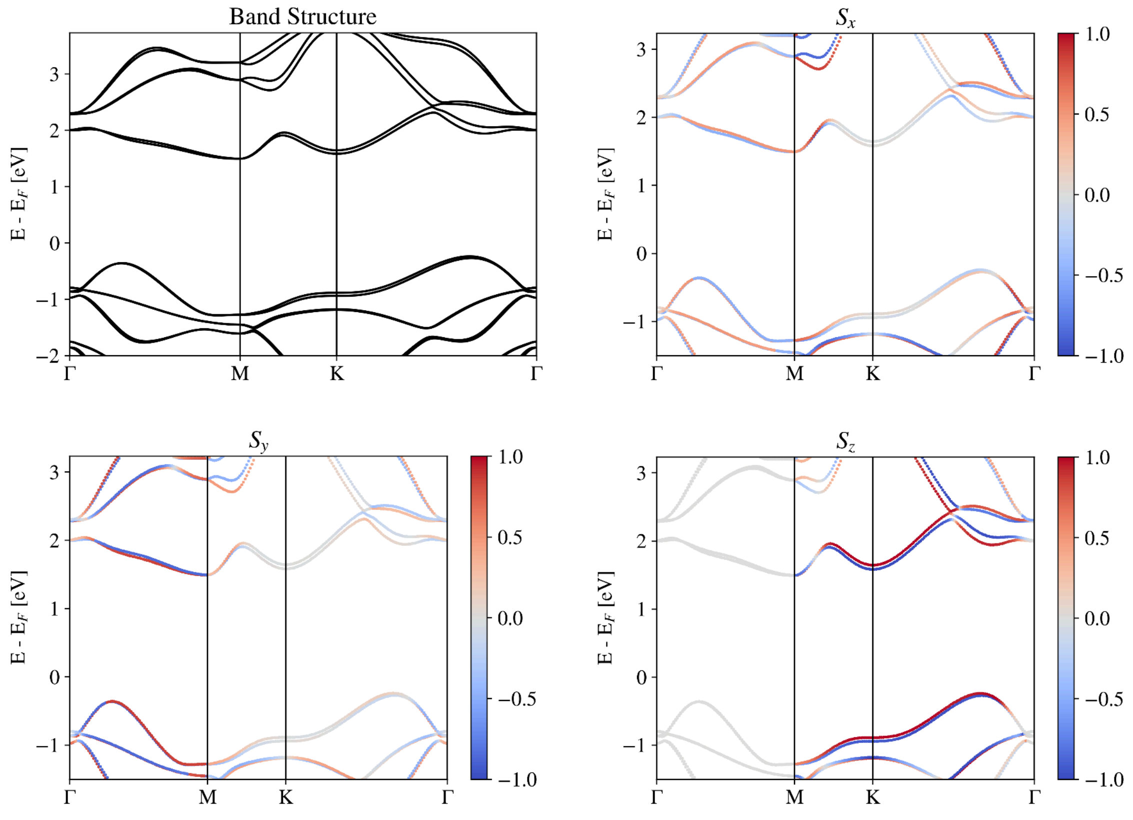


Figure 51: Band structure and spin polarization projections.

## 2.26 AsClS-afd0d75a82a2

- **Formula**: AsClS
- **Structural Cluster**: ABC-3
- **Band gap (PBE)**: 1.532 eV
- **Energy above convex hull (C2DB)**: 0.056 eV
- **Space group symbol**: $P3m1$
- **Space group number**: 156
- **Polar structure**: True

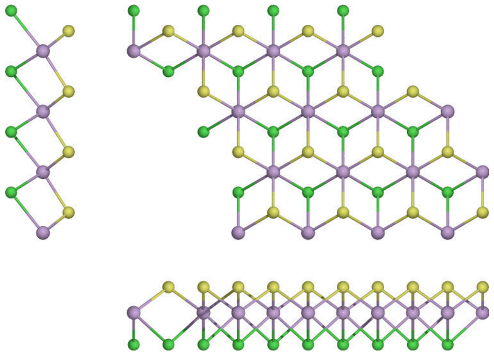

Figure 52: Structure representation

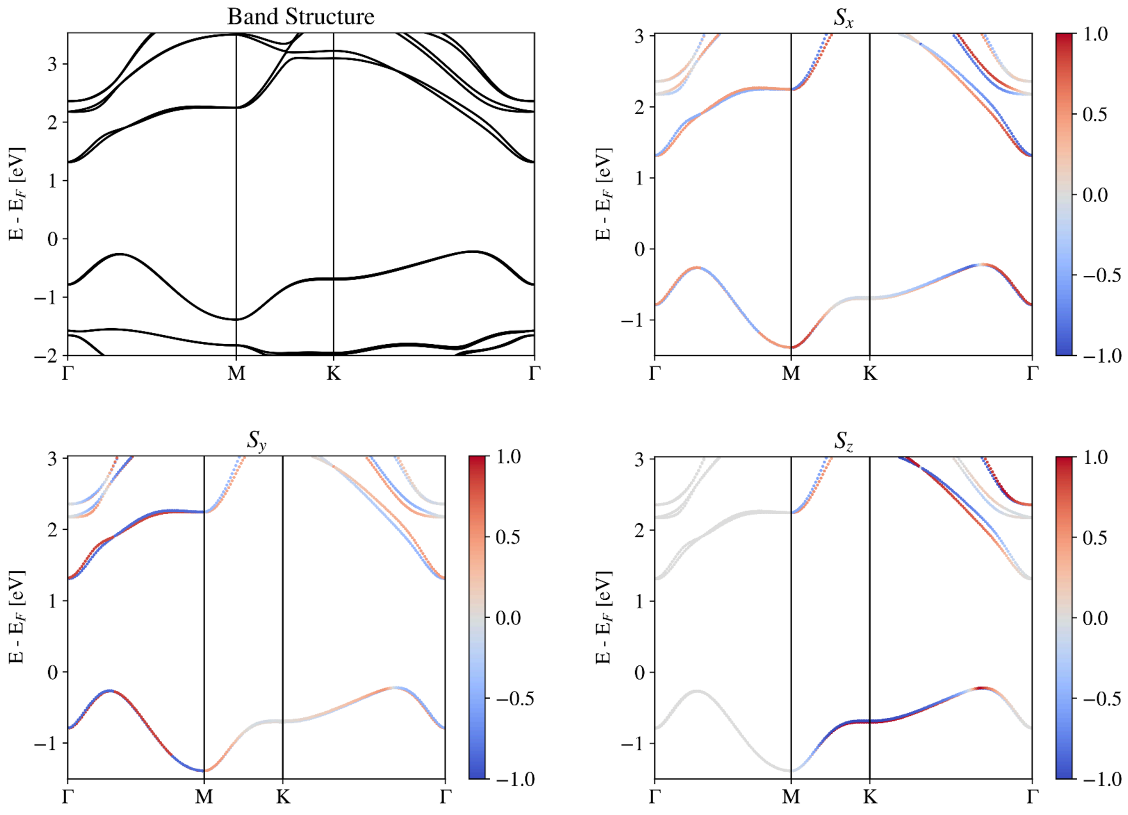


Figure 53: Band structure and spin polarization projections.

### 2.27 AsClSe-1a3be826b3e0

- **Formula**: AsClSe
- **Structural Cluster**: ABC-3
- **Band gap (PBE)**: 1.364 eV
- **Energy above convex hull (C2DB)**: 0.013 eV
- **Space group symbol**: $P3m1$
- **Space group number**: 156
- **Polar structure**: True

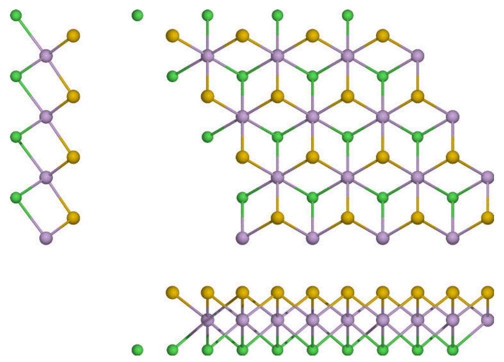

Figure 54: Structure representation

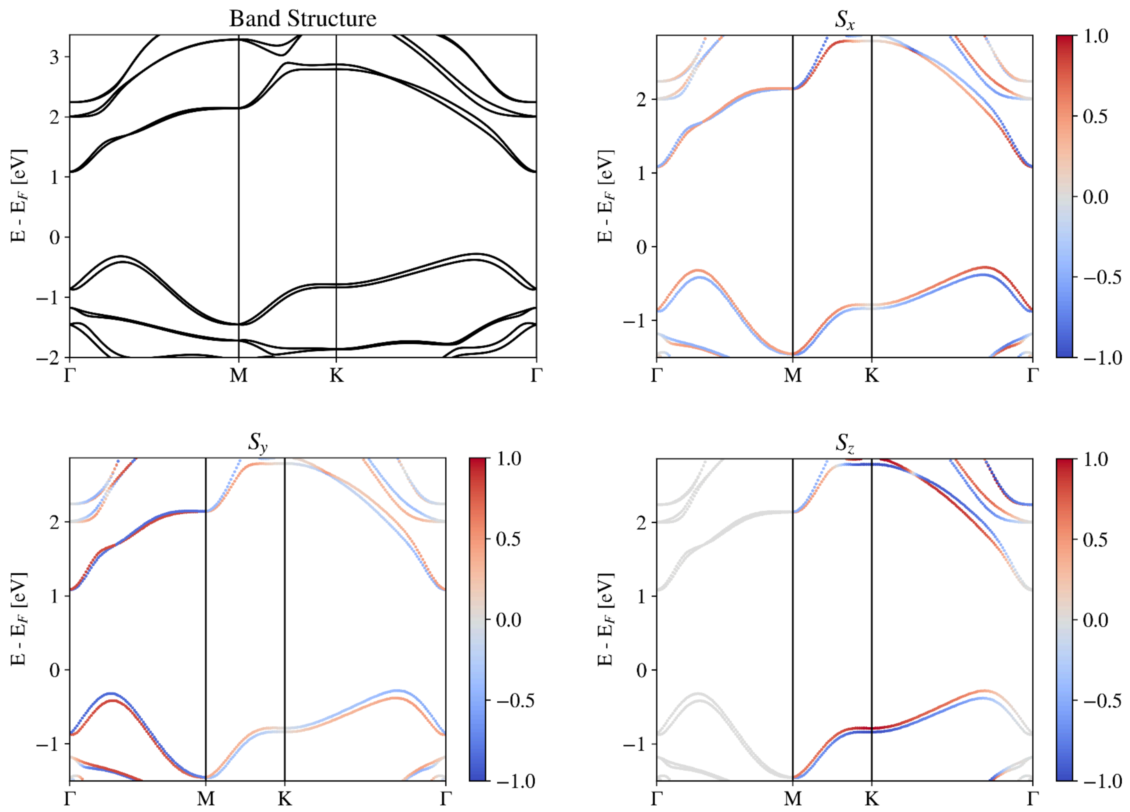


Figure 55: Band structure and spin polarization projections.

## 2.28 AsClSe-df329350eef2

- **Formula**: AsClSe
- **Structural Cluster**: ABC-4
- **Band gap (PBE)**: 1.71 eV
- **Energy above convex hull (C2DB)**: 0.179 eV
- **Space group symbol**: $P3m1$
- **Space group number**: 156
- **Polar structure**: True

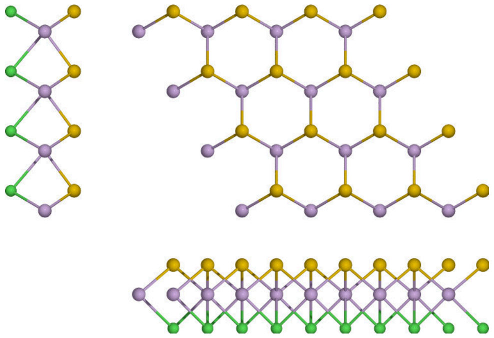

Figure 56: Structure representation

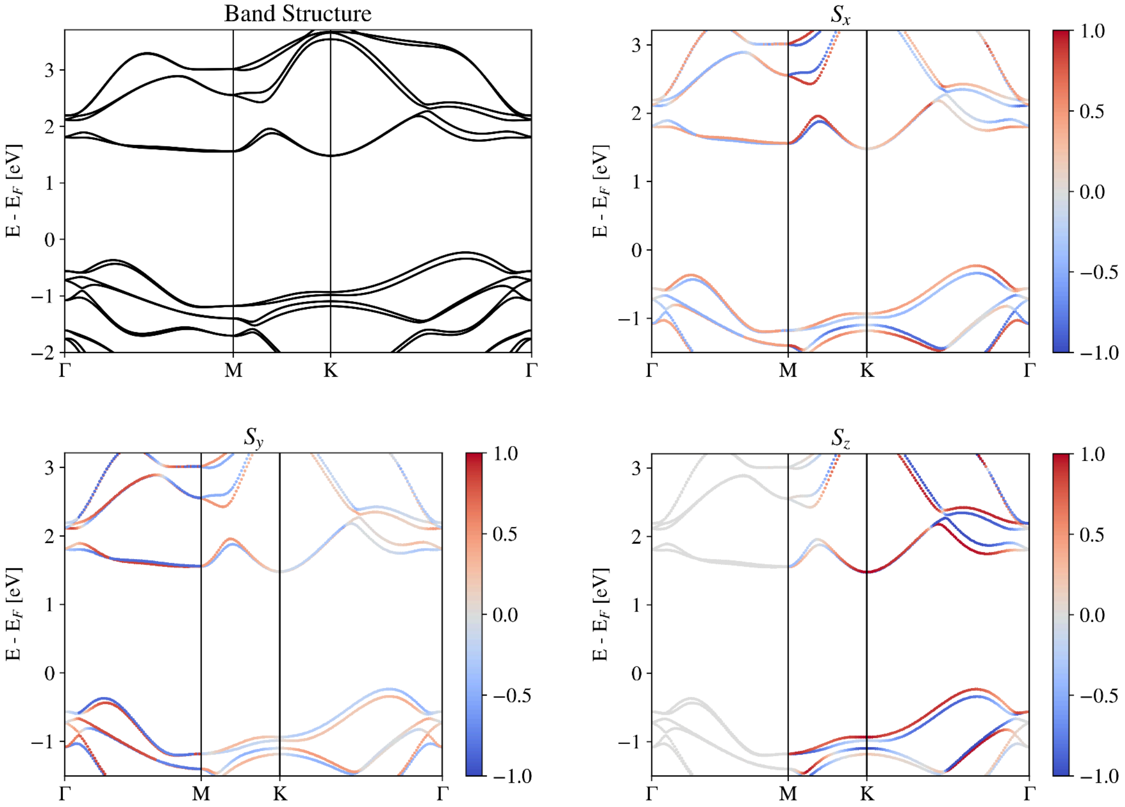


Figure 57: Band structure and spin polarization projections.

## 2.29 AsClTe-4fd8ad708fb0

- **Formula**: AsClTe
- **Structural Cluster**: ABC-3
- **Band gap (PBE)**: 1.496 eV
- **Energy above convex hull (C2DB)**: 0.018 eV
- **Space group symbol**: $P3m1$
- **Space group number**: 156
- **Polar structure**: True

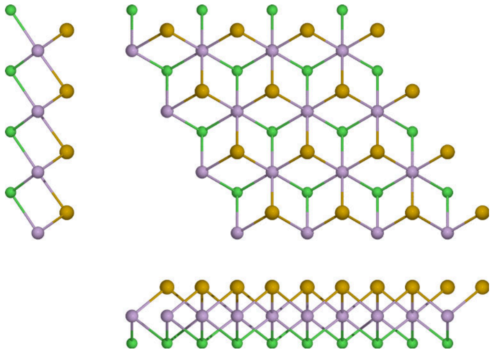

Figure 58: Structure representation

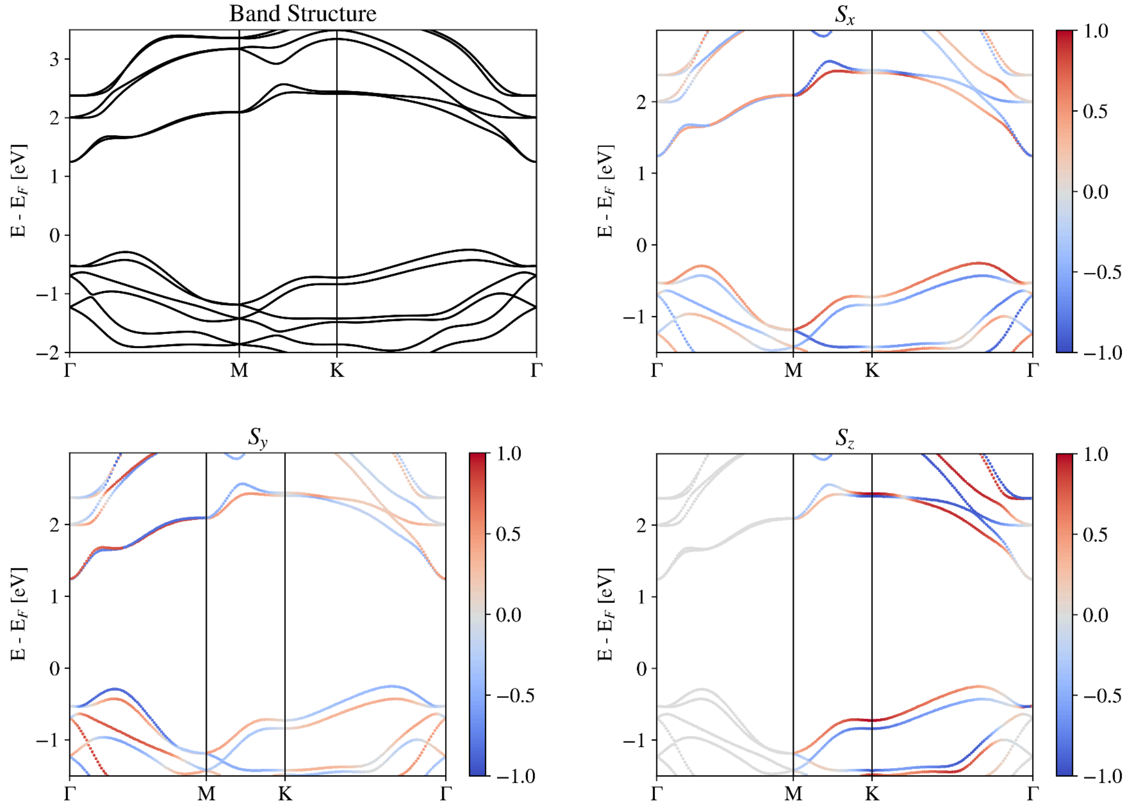


Figure 59: Band structure and spin polarization projections.

## 2.30 AsClTe-fba4cc0df459

- **Formula**: AsClTe
- **Structural Cluster**: ABC-4
- **Band gap (PBE)**: 1.316 eV
- **Energy above convex hull (C2DB)**: 0.194 eV
- **Space group symbol**: $P3m1$
- **Space group number**: 156
- **Polar structure**: True

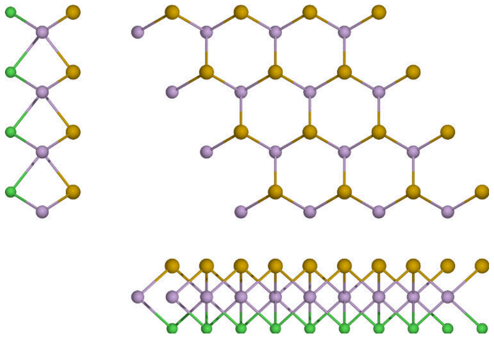

Figure 60: Structure representation

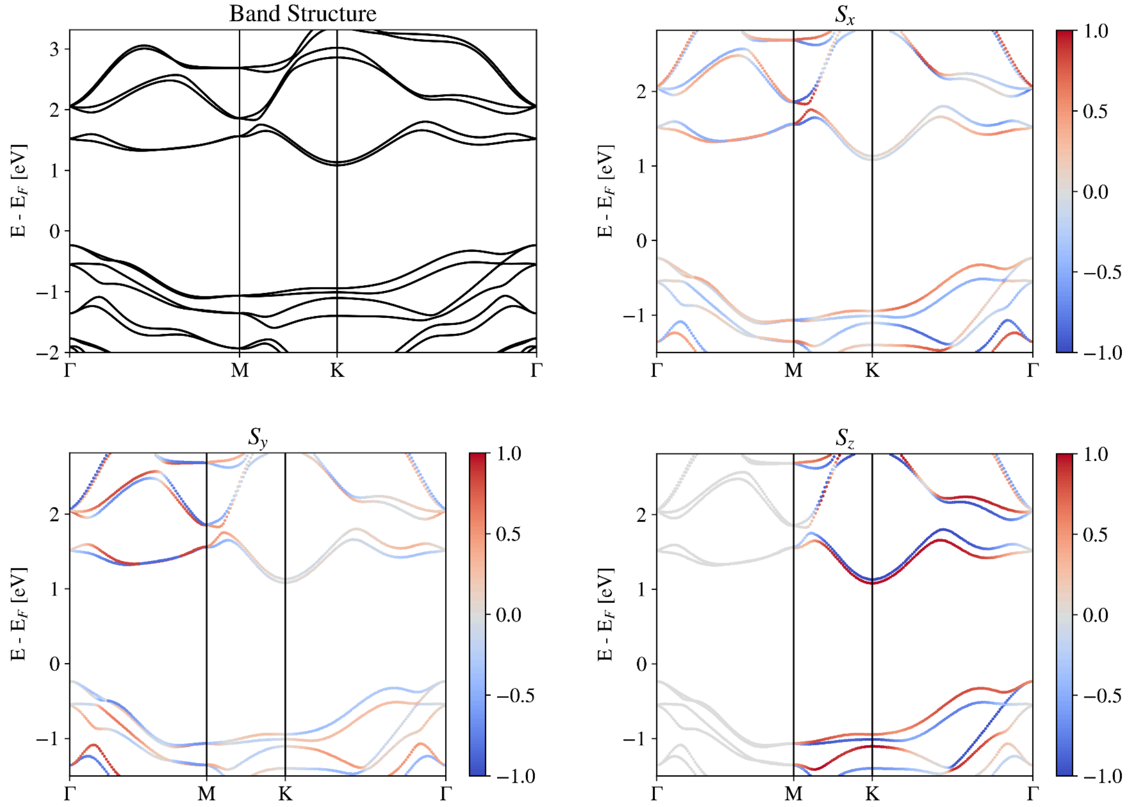


Figure 61: Band structure and spin polarization projections.

## 2.31 AsGa-728f322893fe

- **Formula**: AsGa
- **Structural Cluster**: AB-2
- **Band gap (PBE)**: 1.069 eV
- **Energy above convex hull (C2DB)**: 0.413 eV
- **Space group symbol**: $P3m1$
- **Space group number**: 156
- **Polar structure**: True

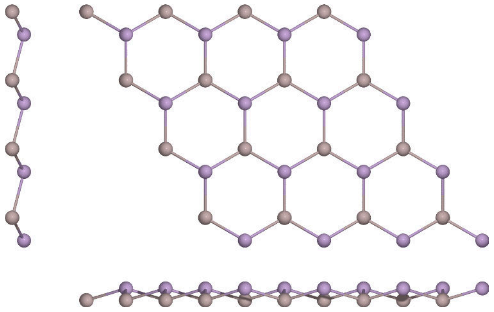

Figure 62: Structure representation

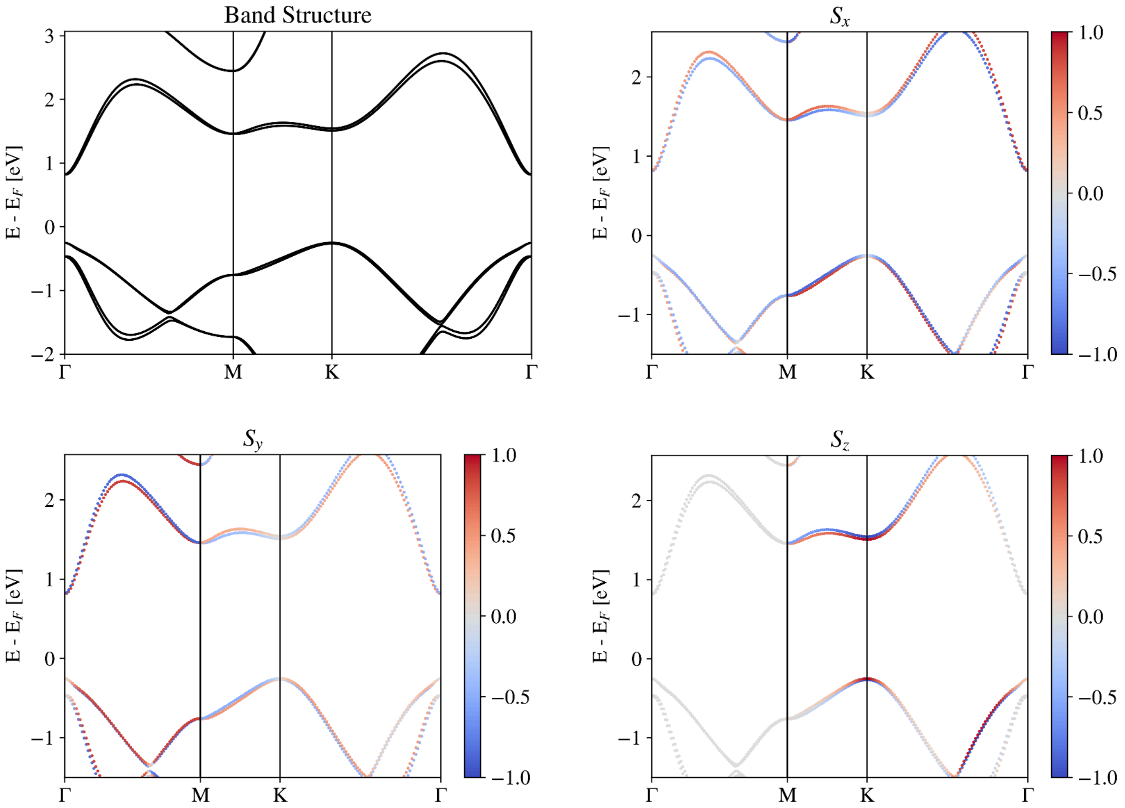


Figure 63: Band structure and spin polarization projections.

## 2.32 AsIS-b13beafa16aa

- **Formula**: AsIS
- **Structural Cluster**: ABC-3
- **Band gap (PBE)**: 1.395 eV
- **Energy above convex hull (C2DB)**: 0.064 eV
- **Space group symbol**: $P3m1$
- **Space group number**: 156
- **Polar structure**: True

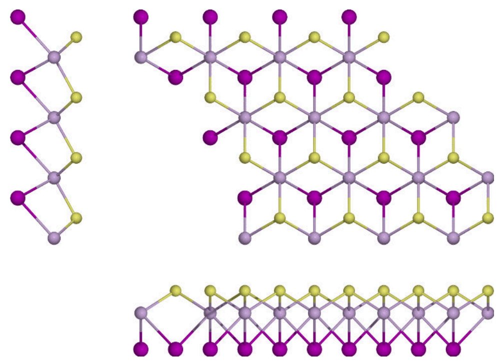

Figure 64: Structure representation

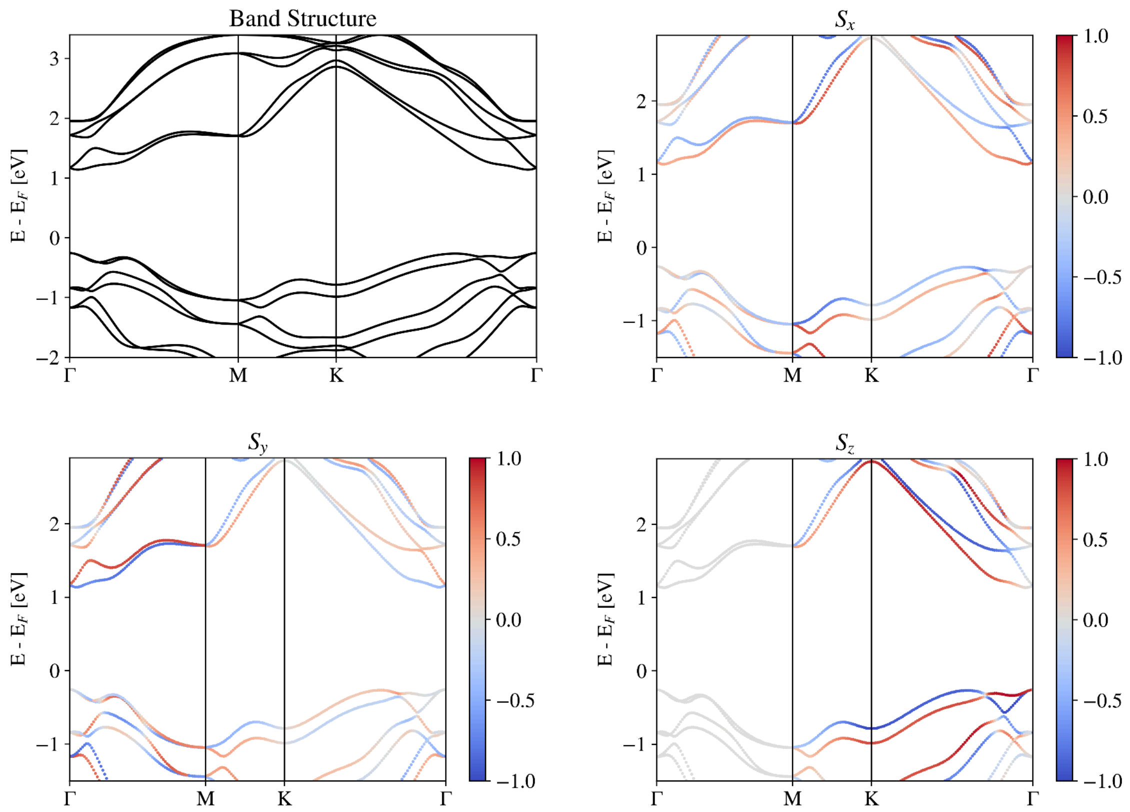


Figure 65: Band structure and spin polarization projections.

### 2.33 AsIS-e23390b66883

- **Formula**: AsIS
- **Structural Cluster**: ABC-4
- **Band gap (PBE)**: 0.295 eV
- **Energy above convex hull (C2DB)**: 0.256 eV
- **Space group symbol**: $P3m1$
- **Space group number**: 156
- **Polar structure**: True

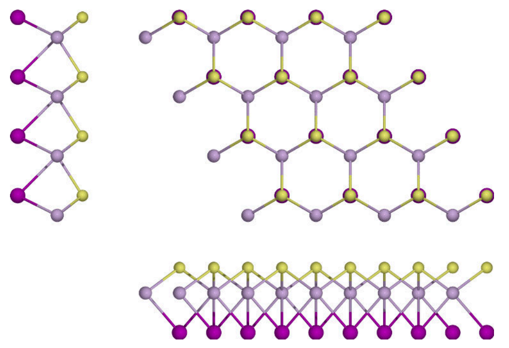

Figure 66: Structure representation

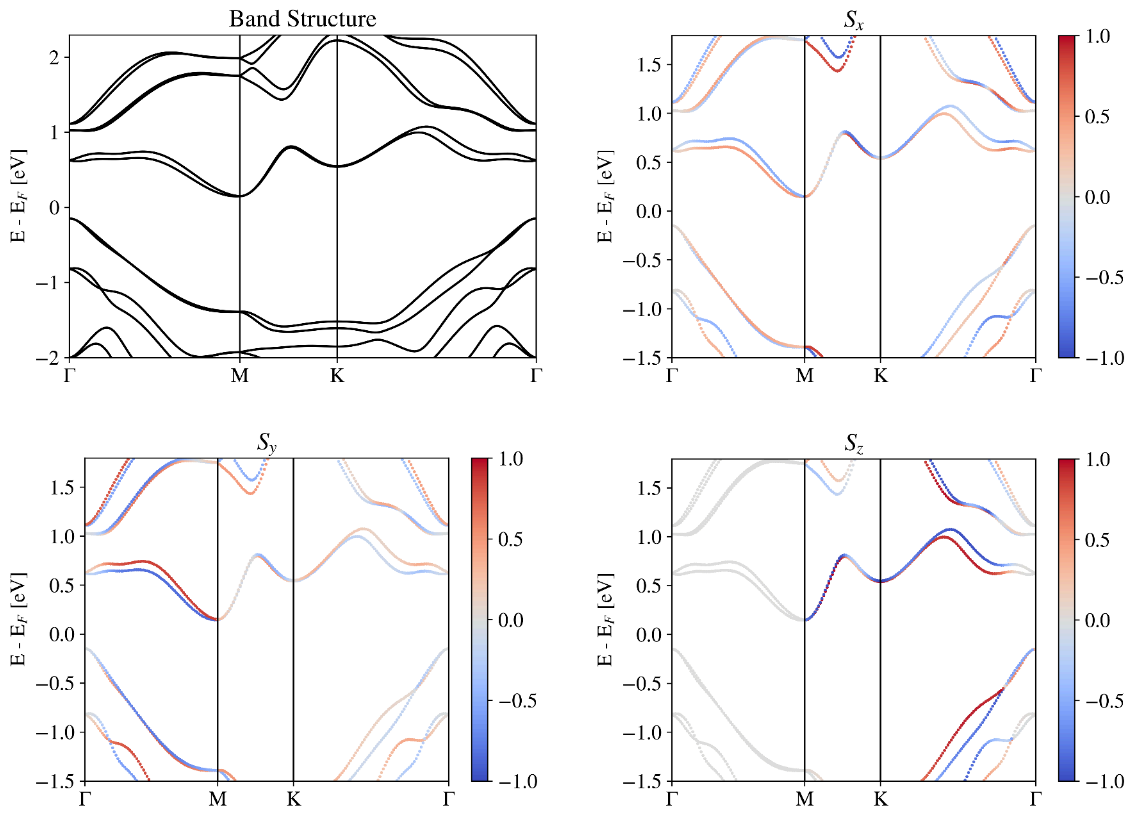


Figure 67: Band structure and spin polarization projections.

## 2.34 AsISe-5d829e480507

- **Formula**: AsISe
- **Structural Cluster**: ABC-3
- **Band gap (PBE)**: 1.164 eV
- **Energy above convex hull (C2DB)**: 0.0 eV
- **Space group symbol**: $P3m1$
- **Space group number**: 156
- **Polar structure**: True

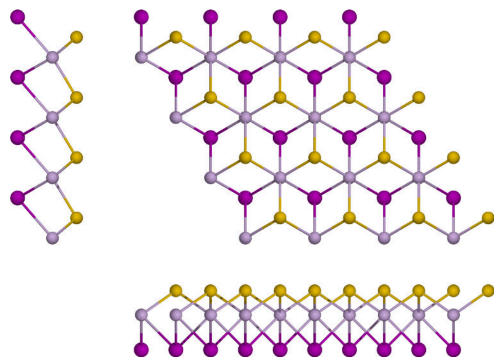

Figure 68: Structure representation

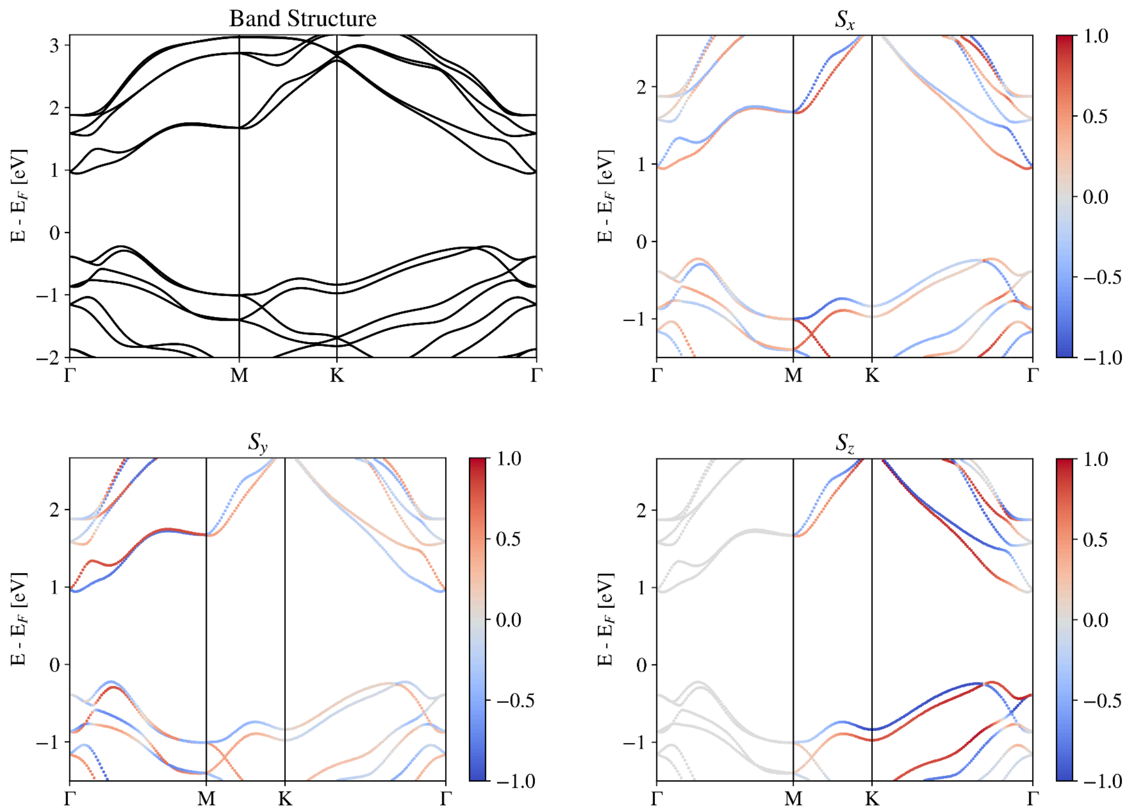


Figure 69: Band structure and spin polarization projections.

## 2.35 AsISe-ca926a42865b

- **Formula**: AsISe
- **Structural Cluster**: ABC-4
- **Band gap (PBE)**: 0.519 eV
- **Energy above convex hull (C2DB)**: 0.174 eV
- **Space group symbol**: $P3m1$
- **Space group number**: 156
- **Polar structure**: True

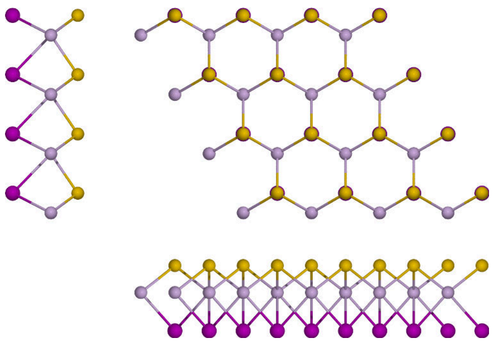

Figure 70: Structure representation

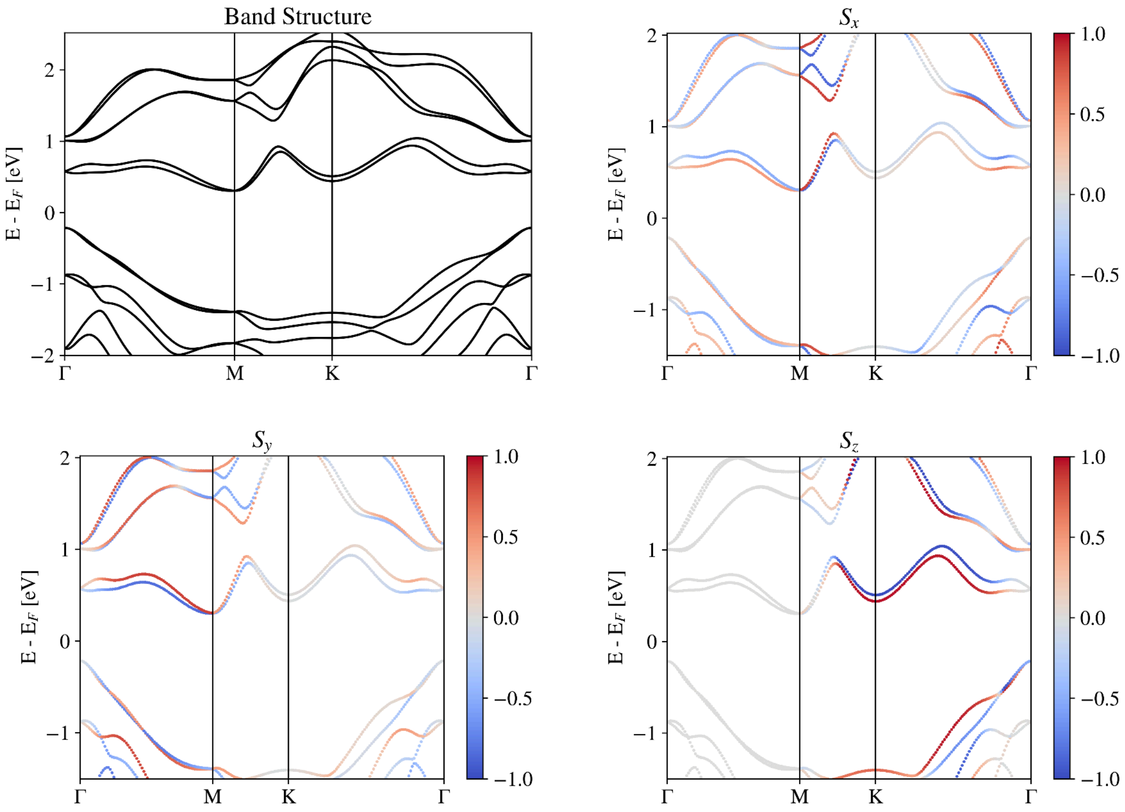


Figure 71: Band structure and spin polarization projections.

## 2.36 AsITe-114b3382699c

- **Formula**: AsITe
- **Structural Cluster**: ABC-4
- **Band gap (PBE)**: 0.416 eV
- **Energy above convex hull (C2DB)**: 0.162 eV
- **Space group symbol**: $P3m1$
- **Space group number**: 156
- **Polar structure**: True

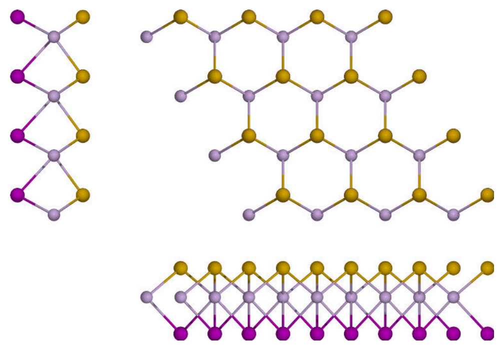

Figure 72: Structure representation

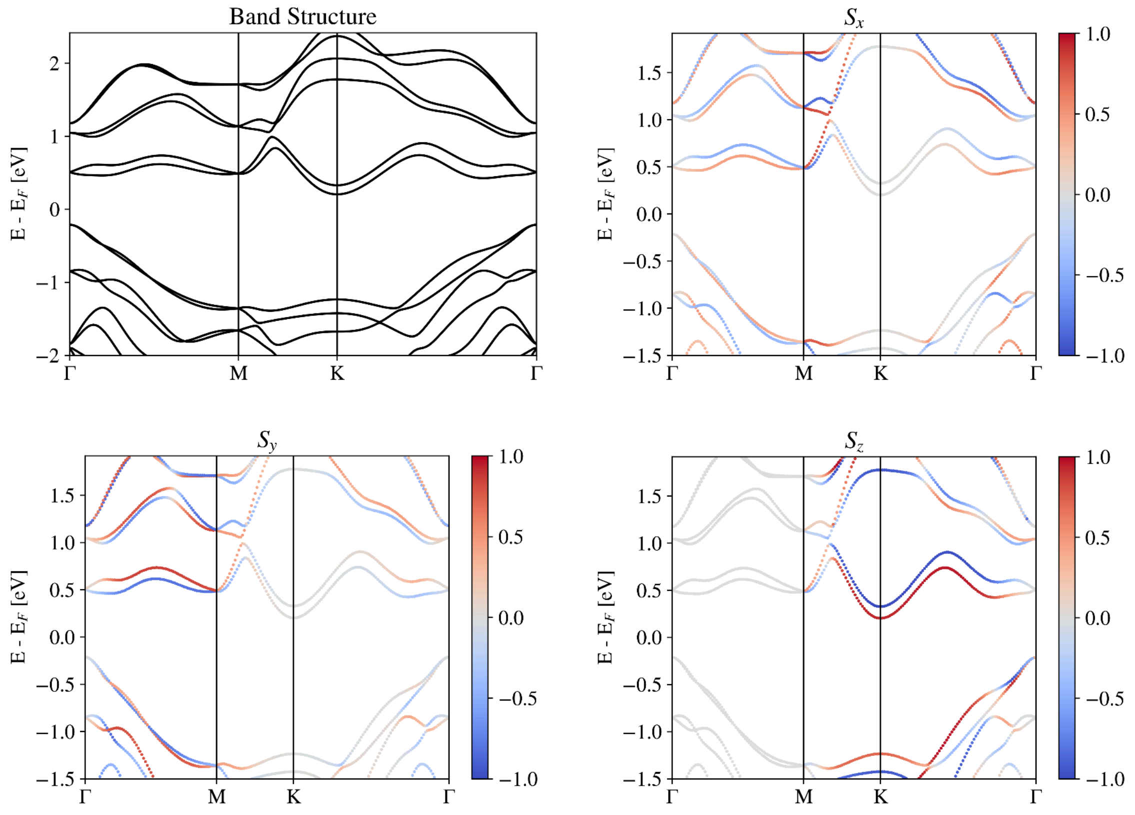


Figure 73: Band structure and spin polarization projections.

## 2.37 AsITe-b6d803aafe3a

- **Formula**: AsITe
- **Structural Cluster**: ABC-3
- **Band gap (PBE)**: 1.009 eV
- **Energy above convex hull (C2DB)**: 0.0 eV
- **Space group symbol**: $P3m1$
- **Space group number**: 156
- **Polar structure**: True

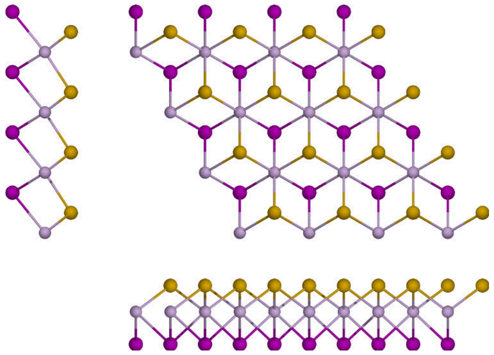

Figure 74: Structure representation

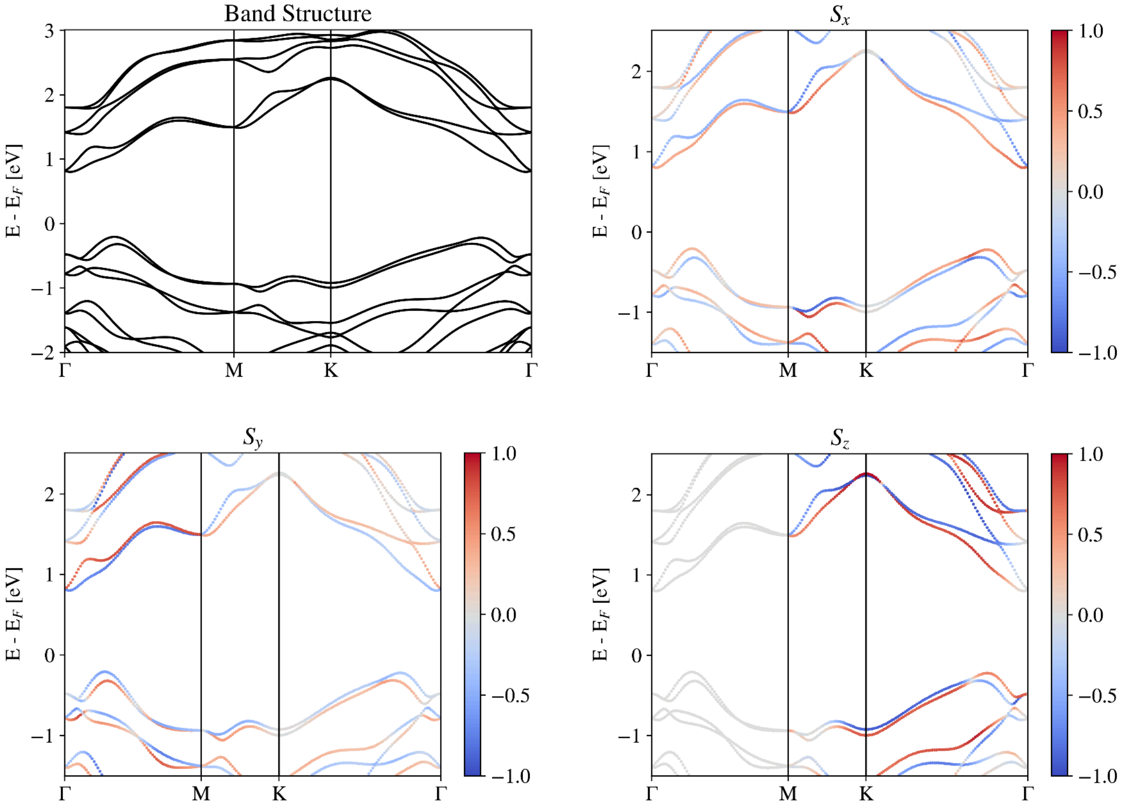


Figure 75: Band structure and spin polarization projections.

## 2.38 AsIn-c77a730c90f8

- **Formula**: AsIn
- **Structural Cluster**: AB-2
- **Band gap (PBE)**: 0.681 eV
- **Energy above convex hull (C2DB)**: 0.4 eV
- **Space group symbol**: $P3m1$
- **Space group number**: 156
- **Polar structure**: True

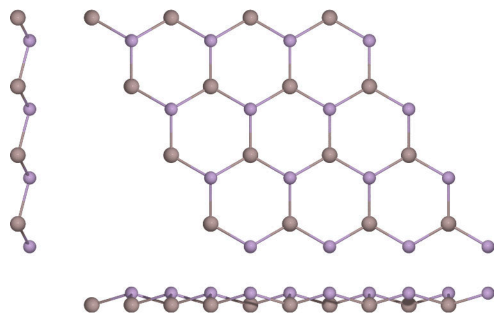

Figure 76: Structure representation

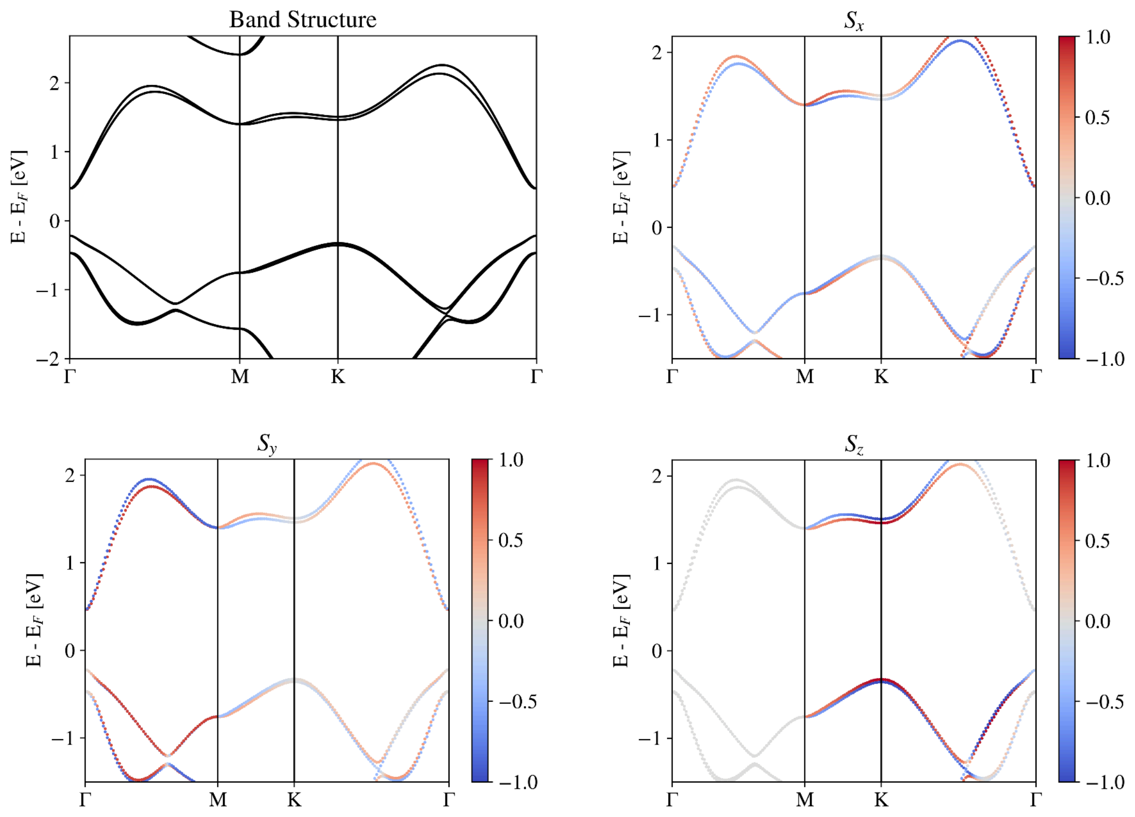


Figure 77: Band structure and spin polarization projections.

## 2.39 Au2Te2-d57228736661

- **Formula**: Au2Te2
- **Structural Cluster**: AB-0
- **Band gap (PBE)**: 0.667 eV
- **Energy above convex hull (C2DB)**: 0.0 eV
- **Space group symbol**: *Pm*
- **Space group number**: 6
- **Polar structure**: True

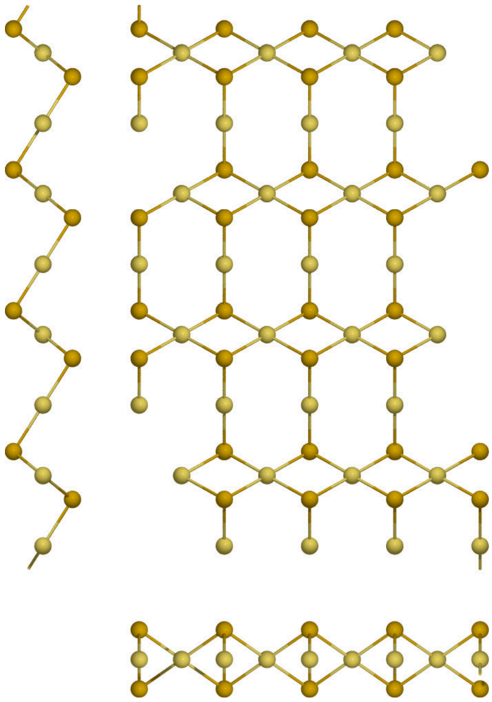

Figure 78: Structure representation

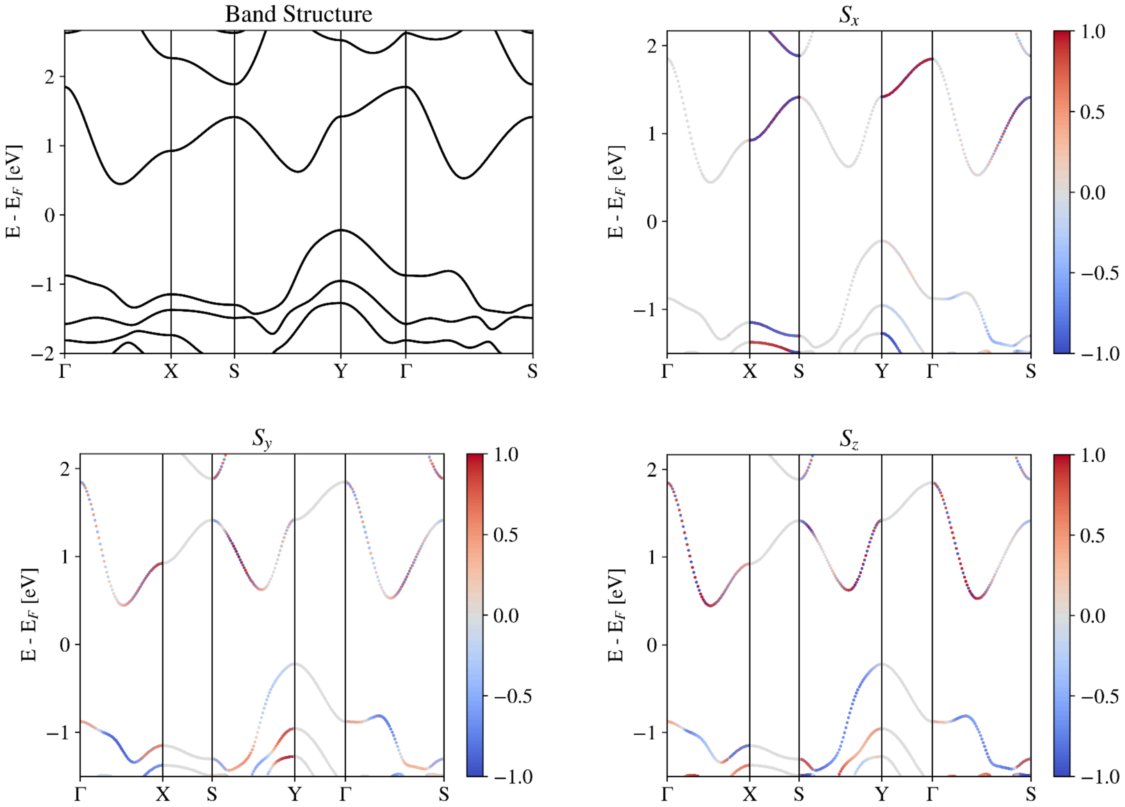


Figure 79: Band structure and spin polarization projections.

## 2.40 BN-4a5edc763604

- **Formula**: BN
- **Structural Cluster**: AB-2
- **Band gap (PBE)**: 4.66 eV
- **Energy above convex hull (C2DB)**: 0.0 eV
- **Space group symbol**: $P\bar{6}m2$
- **Space group number**: 187
- **Polar structure**: False

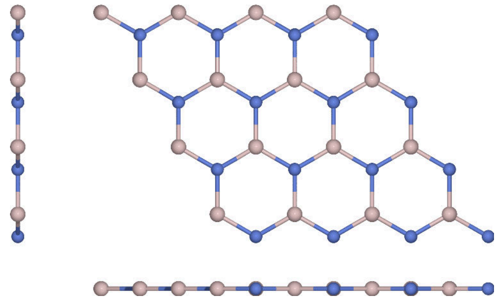

Figure 80: Structure representation

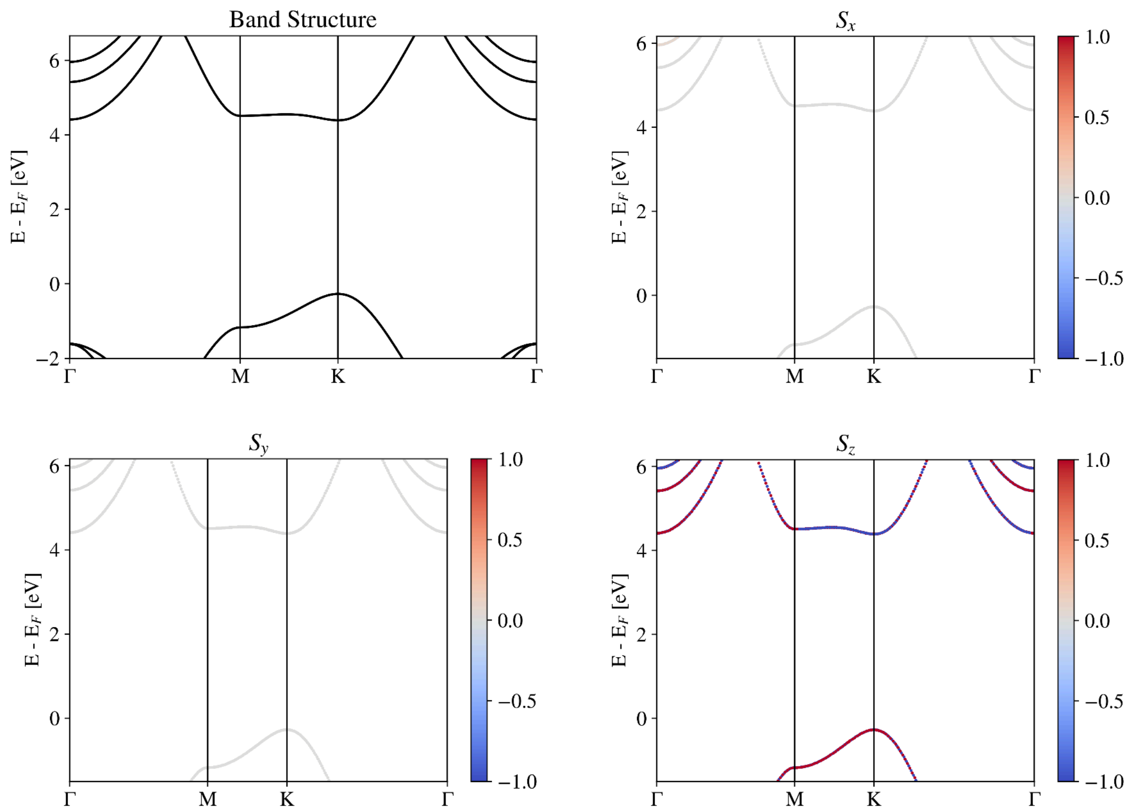


Figure 81: Band structure and spin polarization projections.

## 2.41 BP-0a5e44762c75

- **Formula**: BP
- **Structural Cluster**: AB-2
- **Band gap (PBE)**: 0.906 eV
- **Energy above convex hull (C2DB)**: 0.382 eV
- **Space group symbol**: $P\bar{6}m2$
- **Space group number**: 187
- **Polar structure**: False

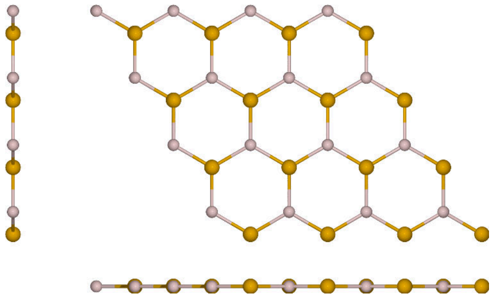

Figure 82: Structure representation

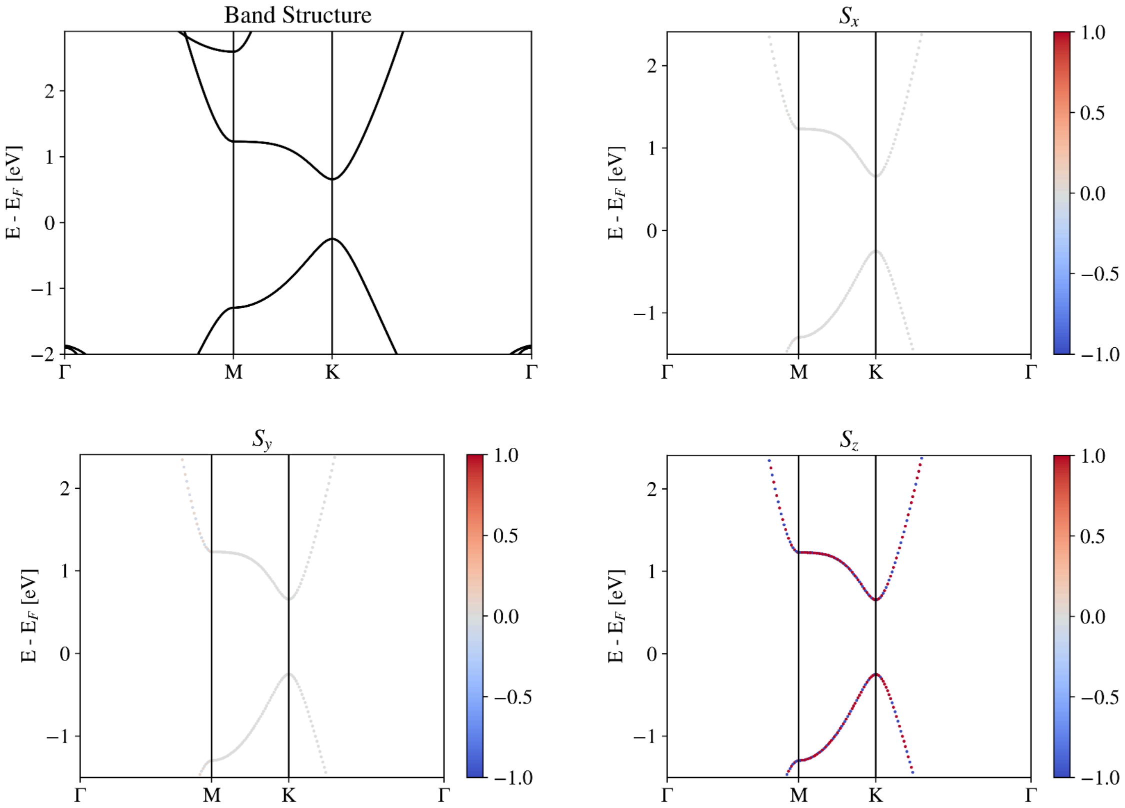


Figure 83: Band structure and spin polarization projections.

## 2.42 BSb-71730c0eaab1

- **Formula**: BSb
- **Structural Cluster**: AB-2
- **Band gap (PBE)**: 0.301 eV
- **Energy above convex hull (C2DB)**: 0.806 eV
- **Space group symbol**: $P\bar{6}m2$
- **Space group number**: 187
- **Polar structure**: False

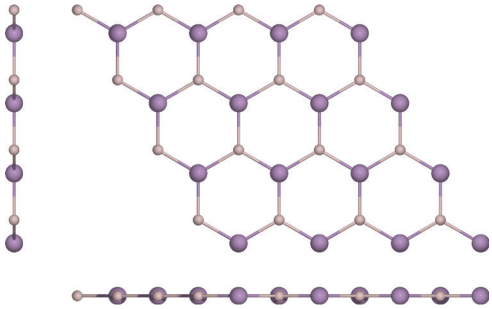

Figure 84: Structure representation

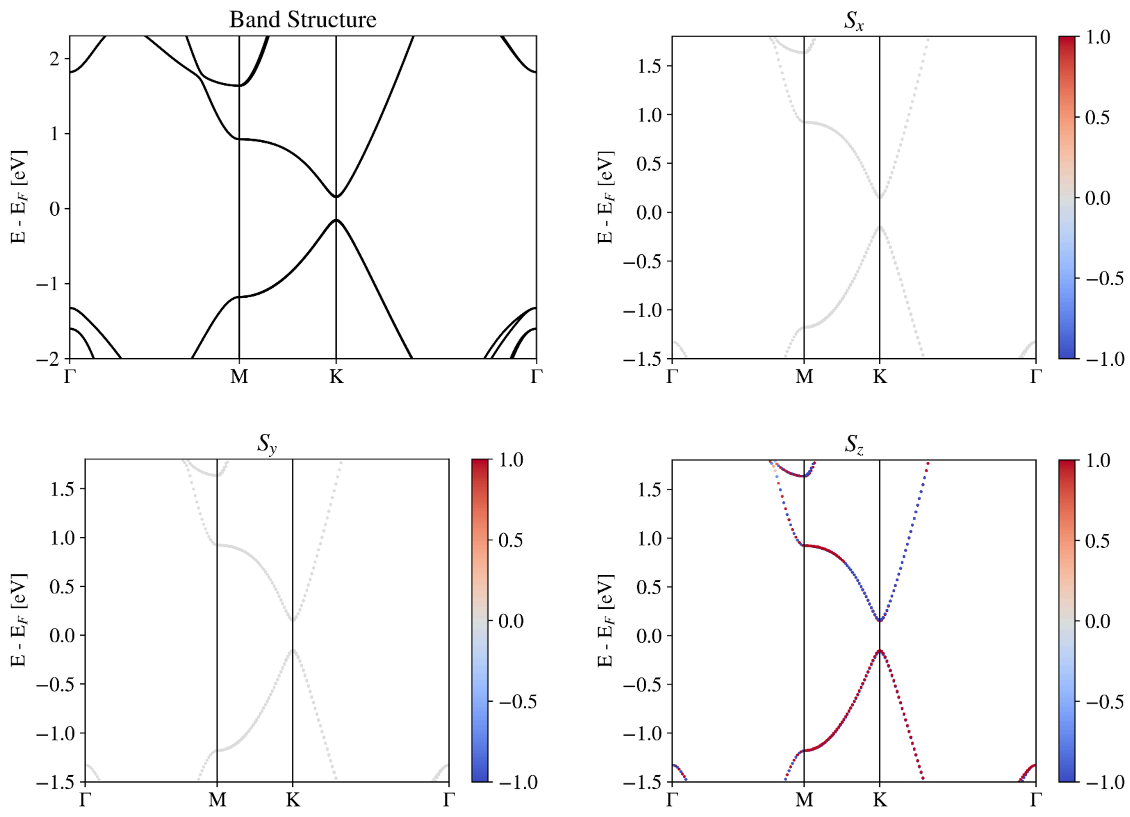


Figure 85: Band structure and spin polarization projections.

## 2.43 BaBr2-1a59eff92917

- **Formula**: BaBr2
- **Structural Cluster**: AB2-4
- **Band gap (PBE)**: 4.149 eV
- **Energy above convex hull (C2DB)**: 0.167 eV
- **Space group symbol**: $P\bar{6}m2$
- **Space group number**: 187
- **Polar structure**: False

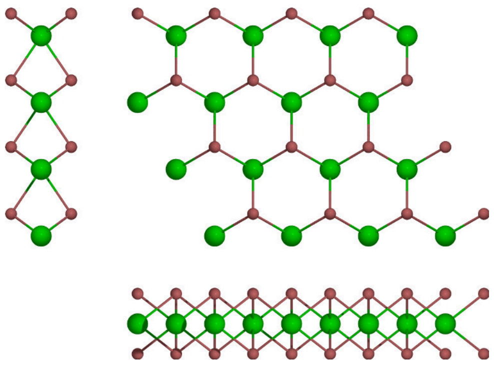

Figure 86: Structure representation

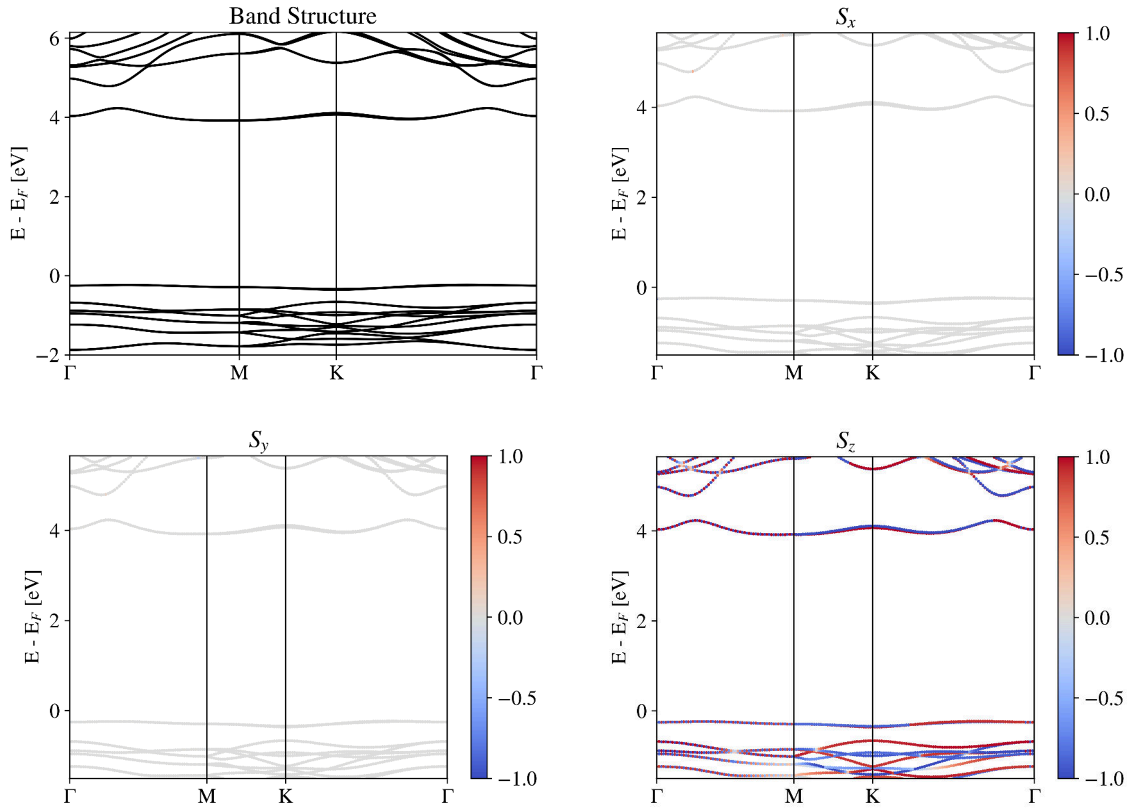


Figure 87: Band structure and spin polarization projections.

## 2.44 BaBr2-df54a81e64da

- **Formula**: BaBr2
- **Structural Cluster**: AB2-11
- **Band gap (PBE)**: 4.568 eV
- **Energy above convex hull (C2DB)**: 0.244 eV
- **Space group symbol**: $P\bar{4}m2$
- **Space group number**: 115
- **Polar structure**: False

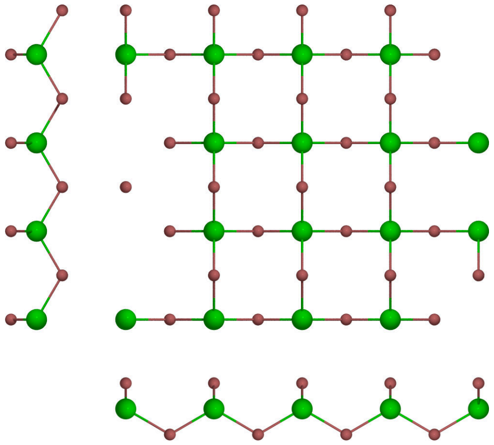

Figure 88: Structure representation

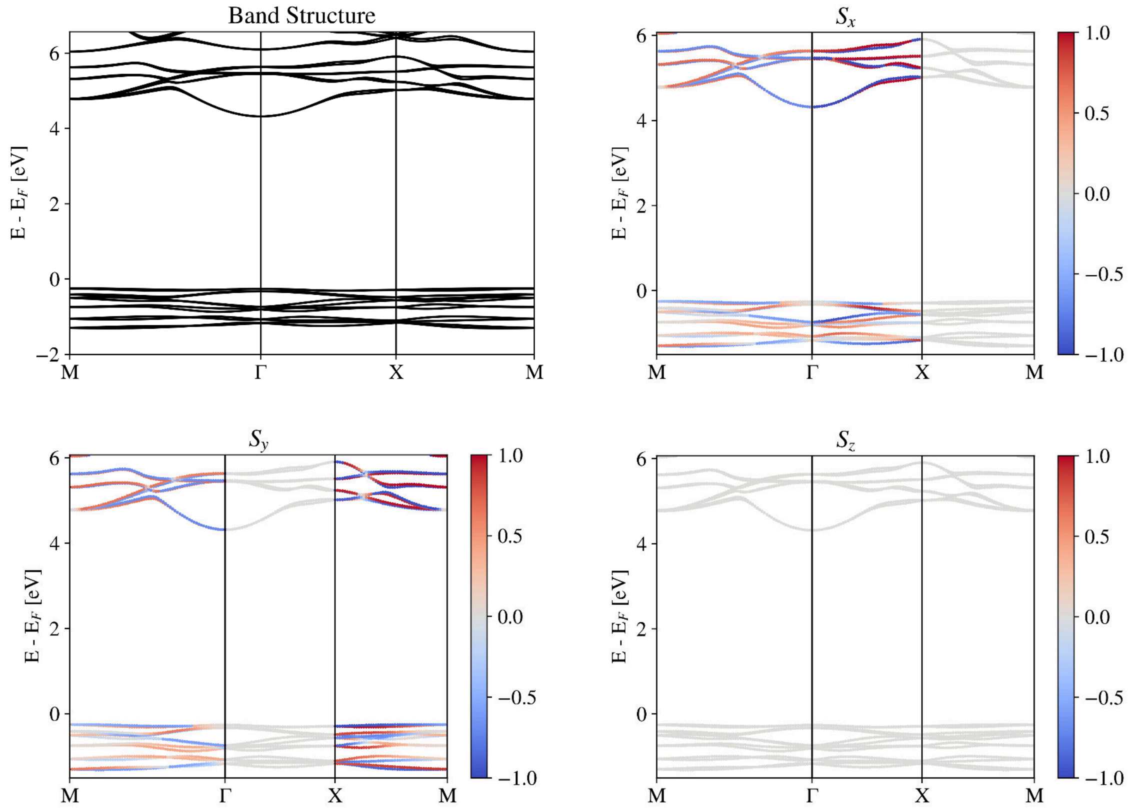


Figure 89: Band structure and spin polarization projections.

## 2.45 BaCl2-54ec344f88a7

- **Formula**: BaCl2
- **Structural Cluster**: AB2-4
- **Band gap (PBE)**: 4.736 eV
- **Energy above convex hull (C2DB)**: 0.241 eV
- **Space group symbol**: $P\bar{6}m2$
- **Space group number**: 187
- **Polar structure**: False

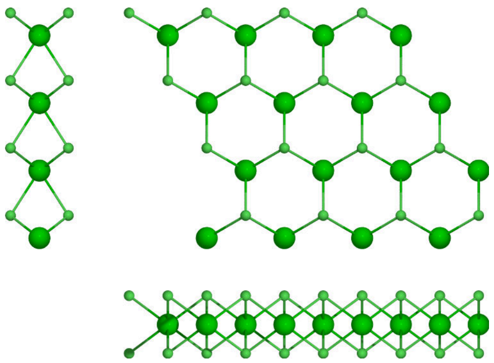

Figure 90: Structure representation

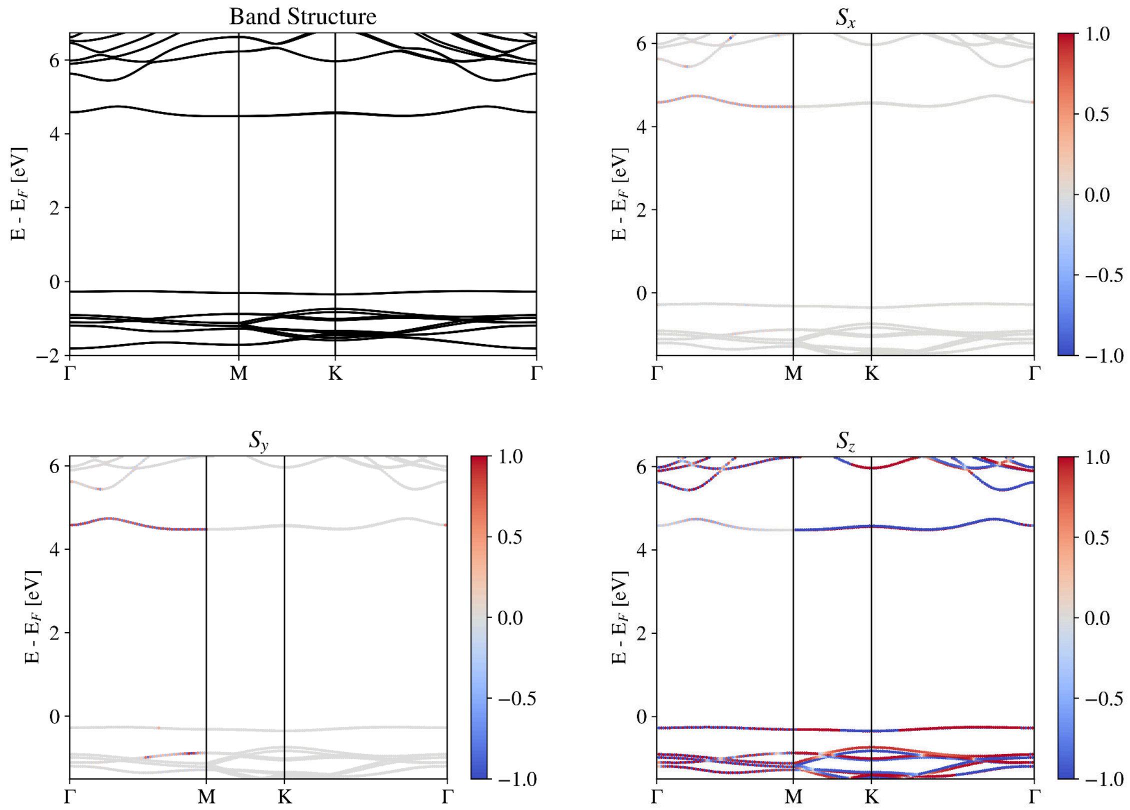


Figure 91: Band structure and spin polarization projections.

## 2.46 BaCl2-ef1fab58e11f

- **Formula**: BaCl2
- **Structural Cluster**: AB2-11
- **Band gap (PBE)**: 5.195 eV
- **Energy above convex hull (C2DB)**: 0.3 eV
- **Space group symbol**: $P\bar{4}m2$
- **Space group number**: 115
- **Polar structure**: False

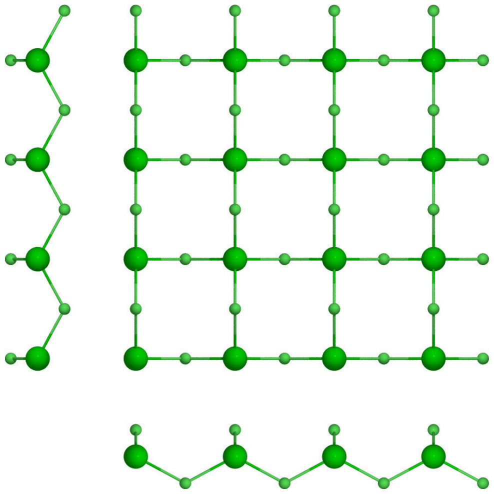

Figure 92: Structure representation

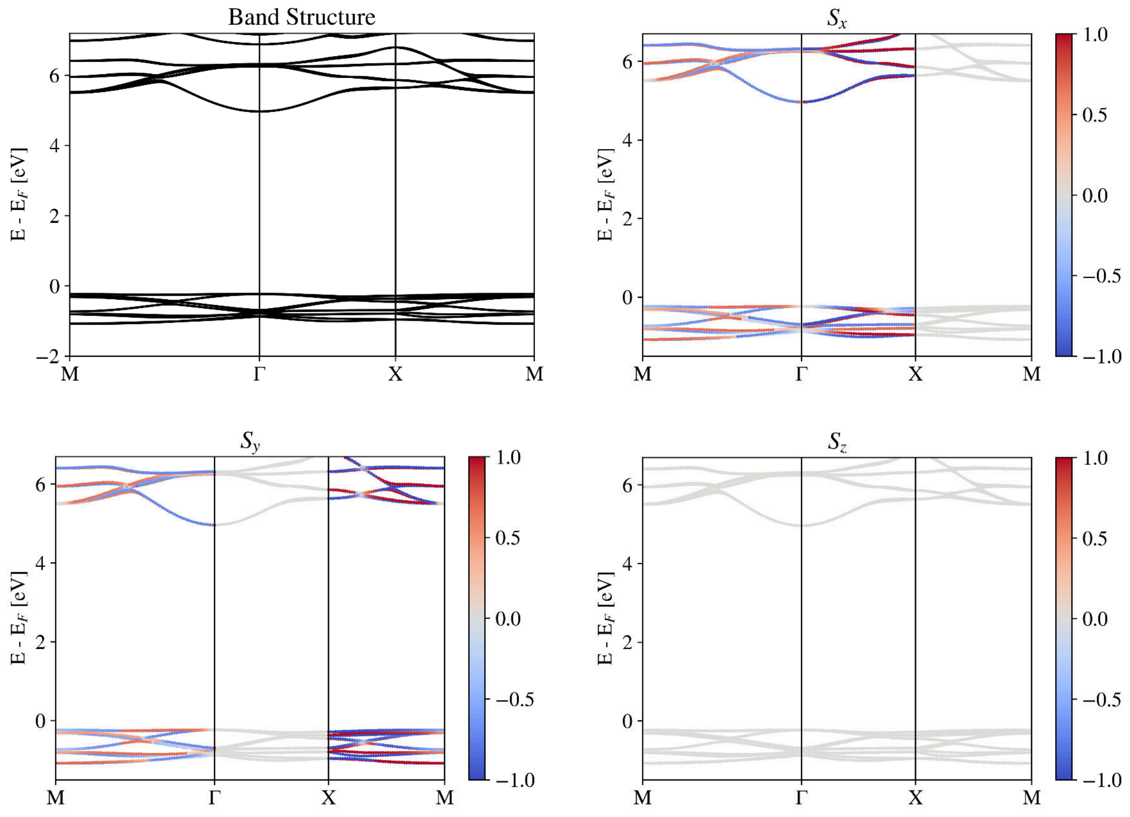


Figure 93: Band structure and spin polarization projections.

## 2.47 BaF2-4b7403281822

- **Formula**: BaF2
- **Structural Cluster**: AB2-11
- **Band gap (PBE)**: 5.659 eV
- **Energy above convex hull (C2DB)**: 0.375 eV
- **Space group symbol**: $P\bar{4}m2$
- **Space group number**: 115
- **Polar structure**: False

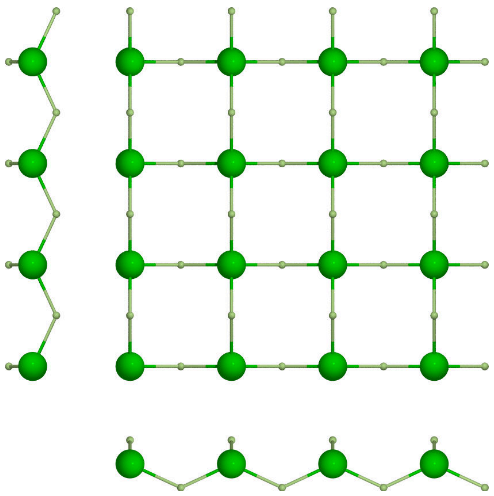

Figure 94: Structure representation

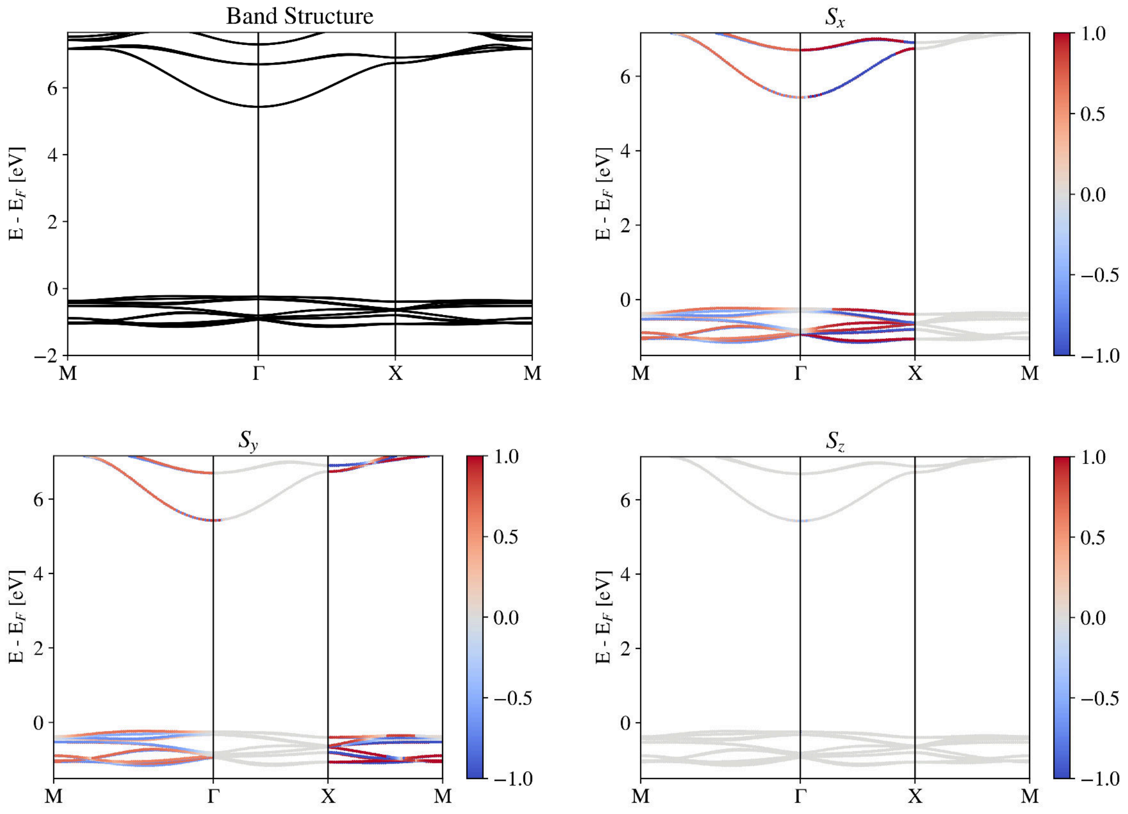


Figure 95: Band structure and spin polarization projections.

## 2.48 BaI2-c4707a226b8f

- **Formula**: BaI2
- **Structural Cluster**: AB2-4
- **Band gap (PBE)**: 3.362 eV
- **Energy above convex hull (C2DB)**: 0.105 eV
- **Space group symbol**: $P\bar{6}m2$
- **Space group number**: 187
- **Polar structure**: False

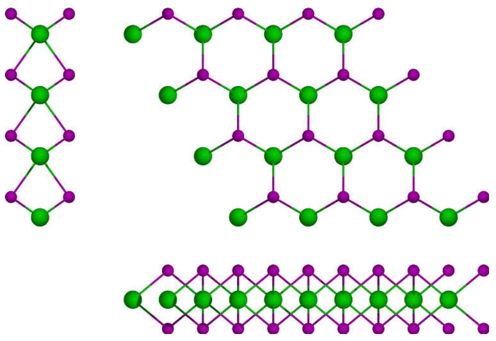

Figure 96: Structure representation

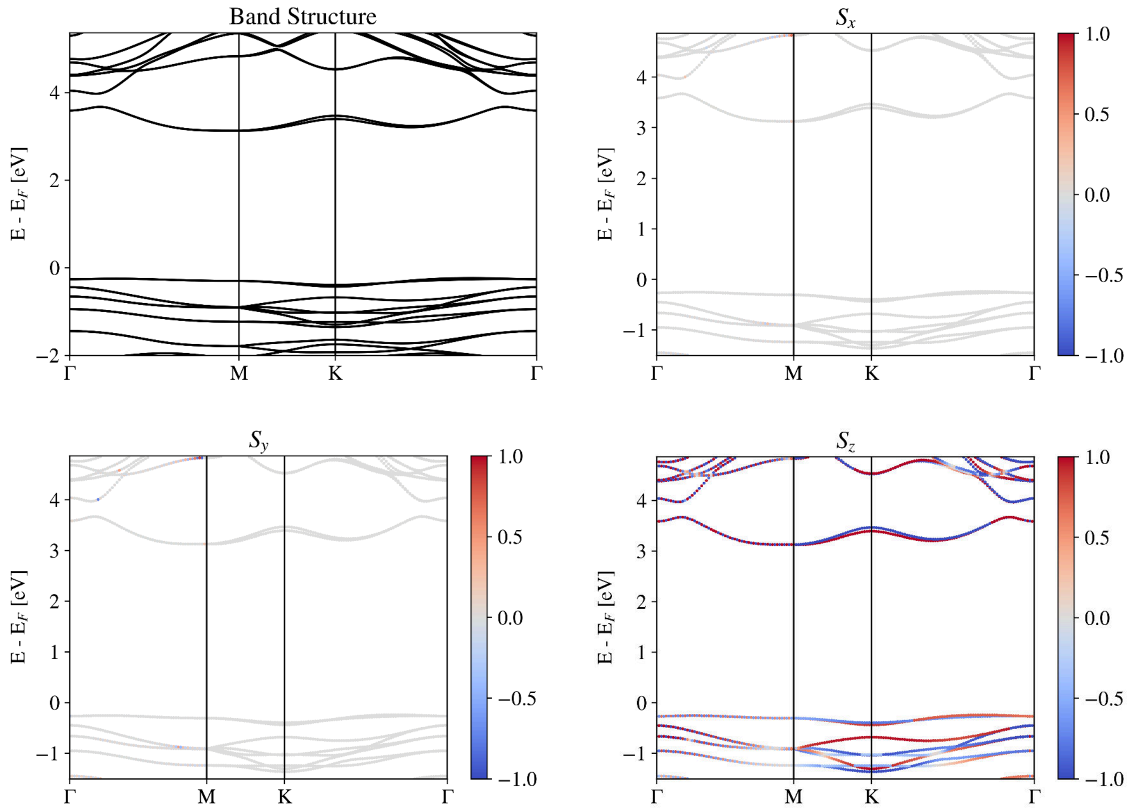


Figure 97: Band structure and spin polarization projections.

## 2.49 BaI2-e20476acac18

- **Formula**: BaI2
- **Structural Cluster**: AB2-11
- **Band gap (PBE)**: 4.002 eV
- **Energy above convex hull (C2DB)**: 0.206 eV
- **Space group symbol**: $P\bar{4}m2$
- **Space group number**: 115
- **Polar structure**: False

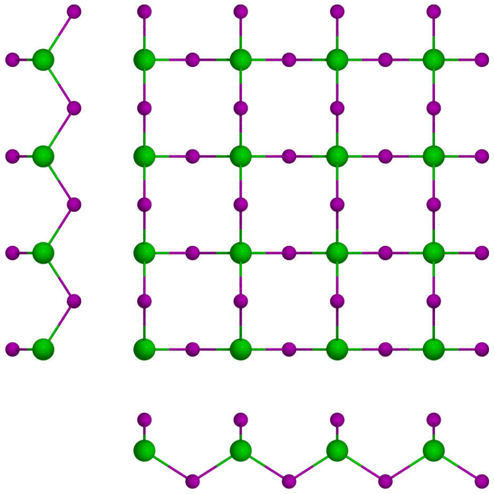

Figure 98: Structure representation

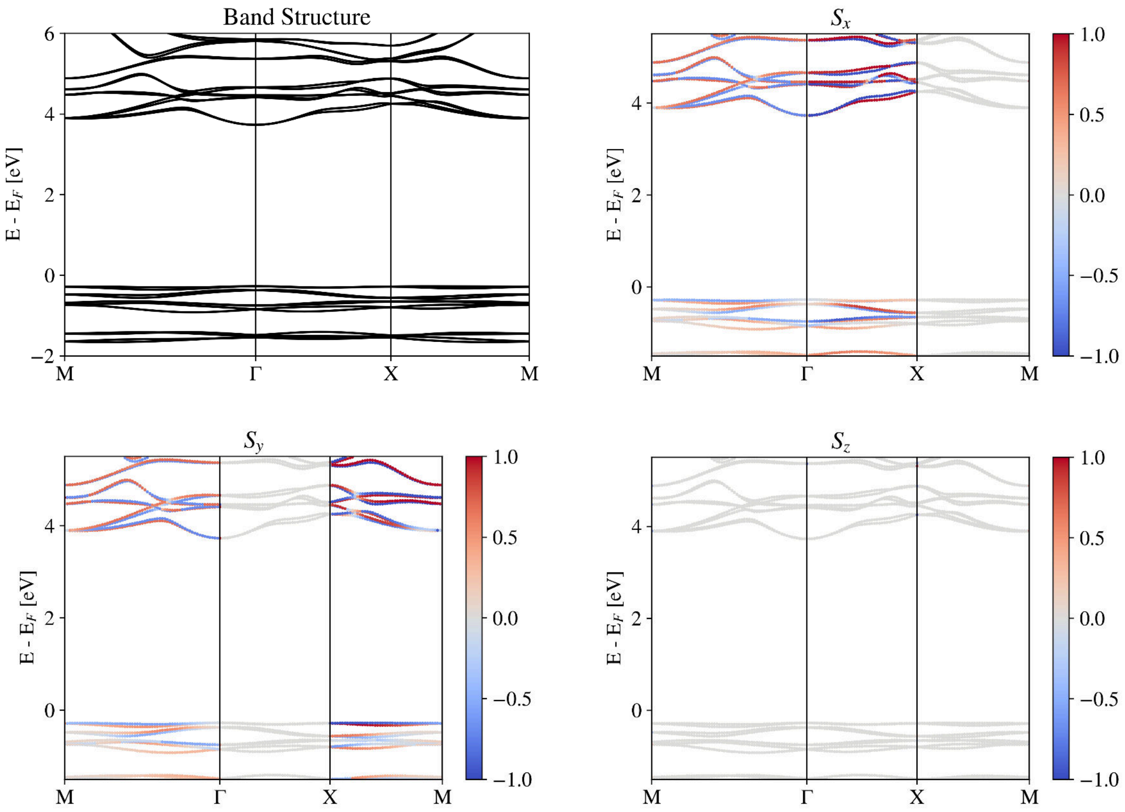


Figure 99: Band structure and spin polarization projections.

## 2.50 Bi2Br6-2b3b022fdf0a

- **Formula**: Bi2Br6
- **Structural Cluster**: AB3-19
- **Band gap (PBE)**: 2.036 eV
- **Energy above convex hull (C2DB)**: 0.061 eV
- **Space group symbol**: $P\bar{6}2m$
- **Space group number**: 189
- **Polar structure**: False

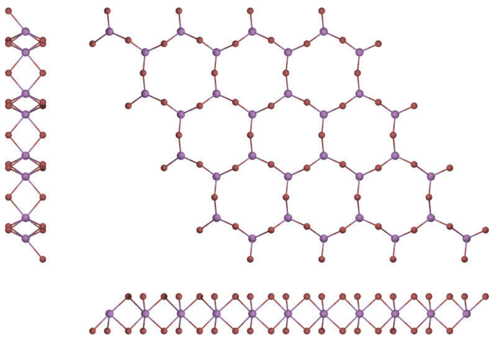

Figure 100: Structure representation

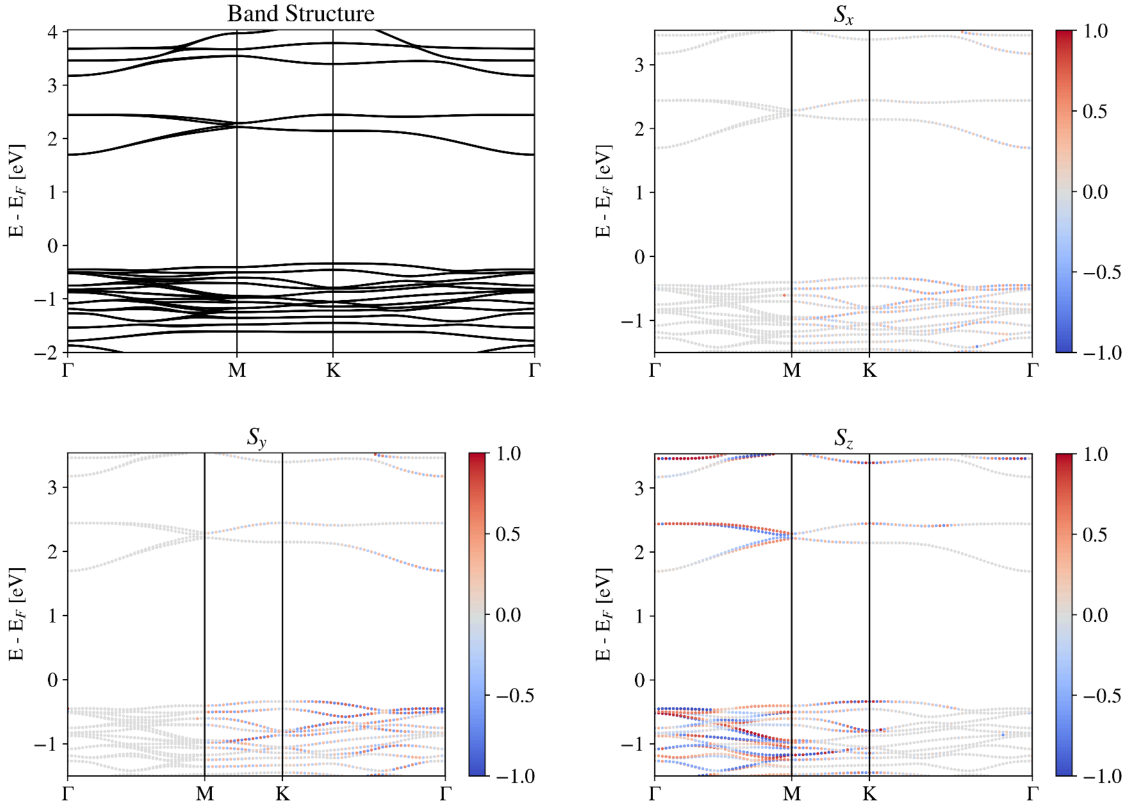


Figure 101: Band structure and spin polarization projections.

## 2.51 Bi2Cl6-3e134cb61e06

- **Formula**: Bi2Cl6
- **Structural Cluster**: AB3-19
- **Band gap (PBE)**: 2.511 eV
- **Energy above convex hull (C2DB)**: 0.048 eV
- **Space group symbol**: $P\bar{6}2m$
- **Space group number**: 189
- **Polar structure**: False

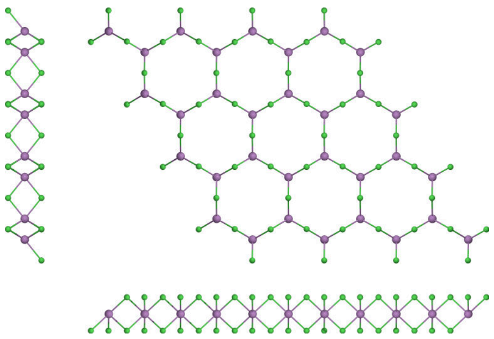

Figure 102: Structure representation

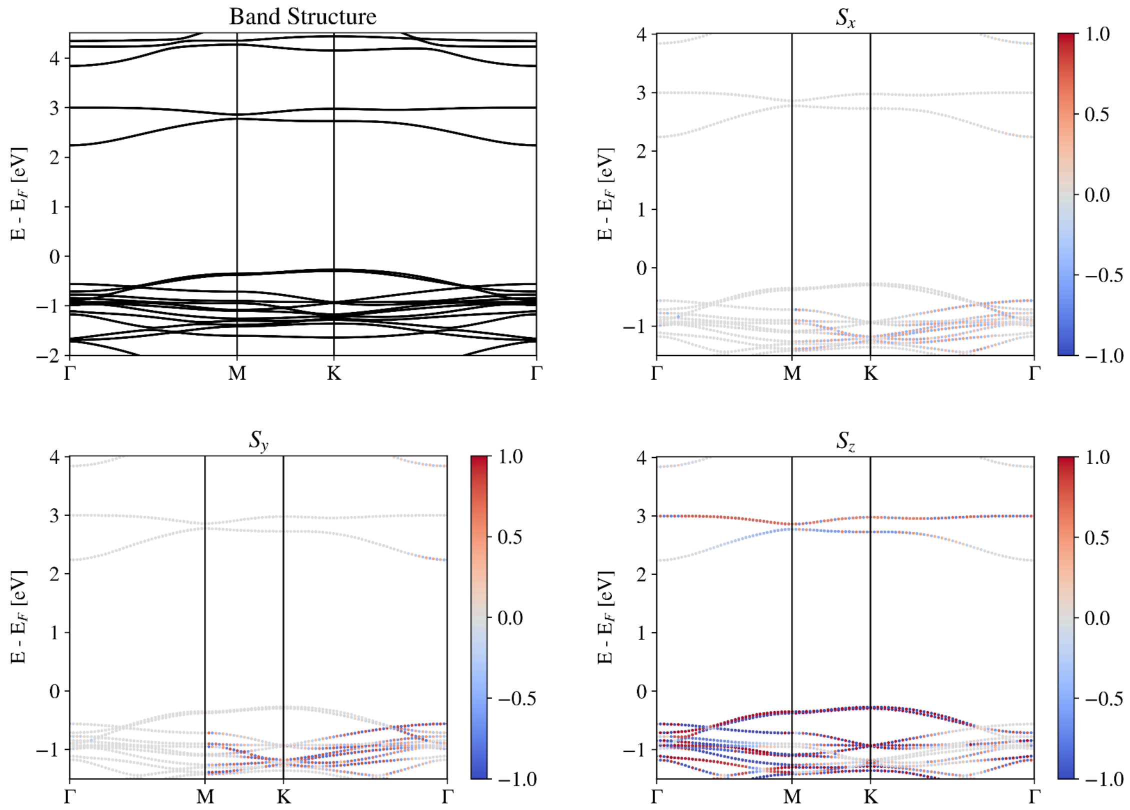


Figure 103: Band structure and spin polarization projections.

## 2.52 Bi2I6-01a2d5e89adc

- **Formula**: Bi2I6
- **Structural Cluster**: AB3-19
- **Band gap (PBE)**: 1.381 eV
- **Energy above convex hull (C2DB)**: 0.081 eV
- **Space group symbol**: $P321$
- **Space group number**: 150
- **Polar structure**: False

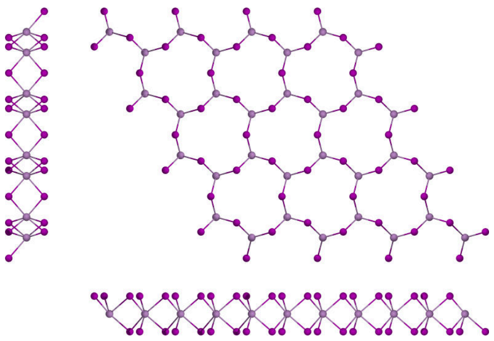

Figure 104: Structure representation

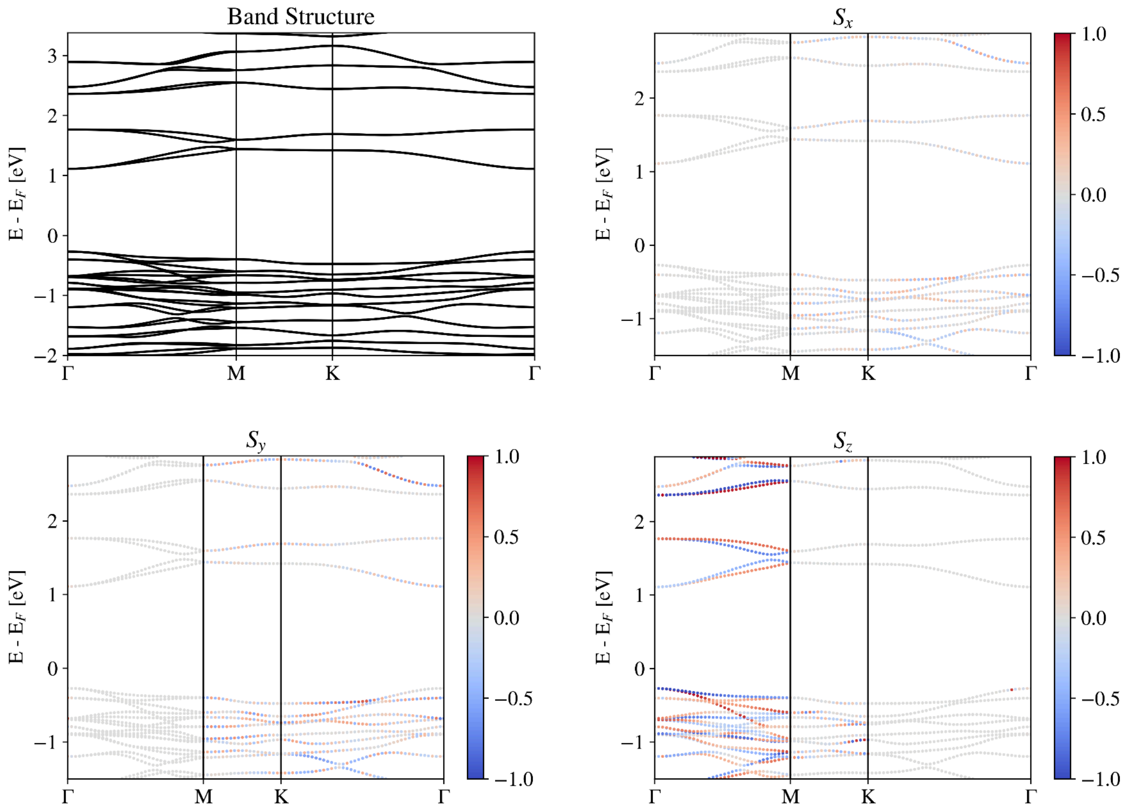


Figure 105: Band structure and spin polarization projections.

## 2.53 Bi2O2-53ac438f321b

- **Formula**: Bi2O2
- **Structural Cluster**: AB-5
- **Band gap (PBE)**: 0.449 eV
- **Energy above convex hull (C2DB)**: 0.319 eV
- **Space group symbol**: $P\bar{6}m2$
- **Space group number**: 187
- **Polar structure**: False

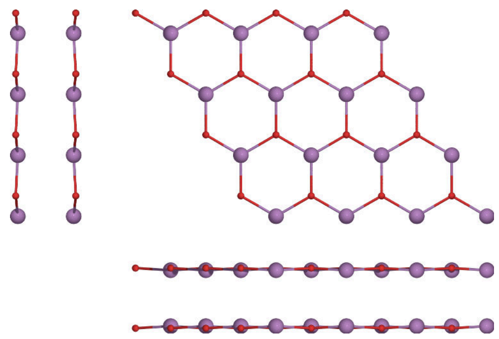

Figure 106: Structure representation

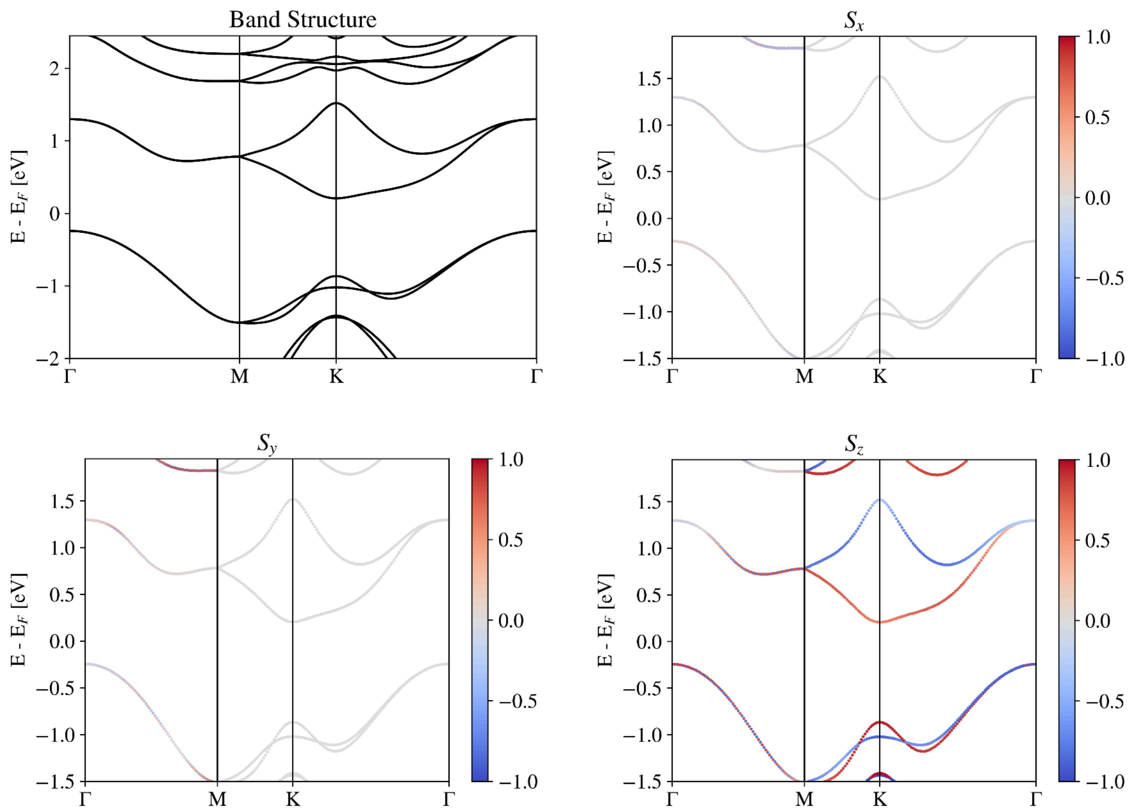


Figure 107: Band structure and spin polarization projections.

## 2.54 Bi2P2S6-287dcf4f1a19

- **Formula**: Bi2P2S6
- **Structural Cluster**: ABC3-16
- **Band gap (PBE)**: 0.953 eV
- **Energy above convex hull (C2DB)**: 0.053 eV
- **Space group symbol**: $P1$
- **Space group number**: 1
- **Polar structure**: True

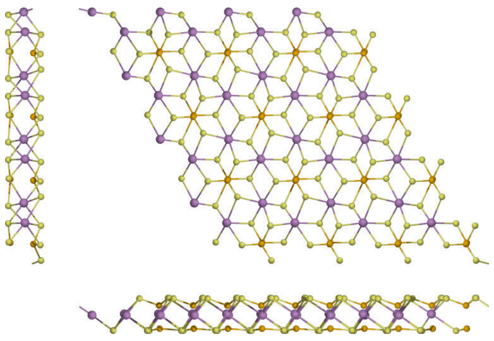

Figure 108: Structure representation

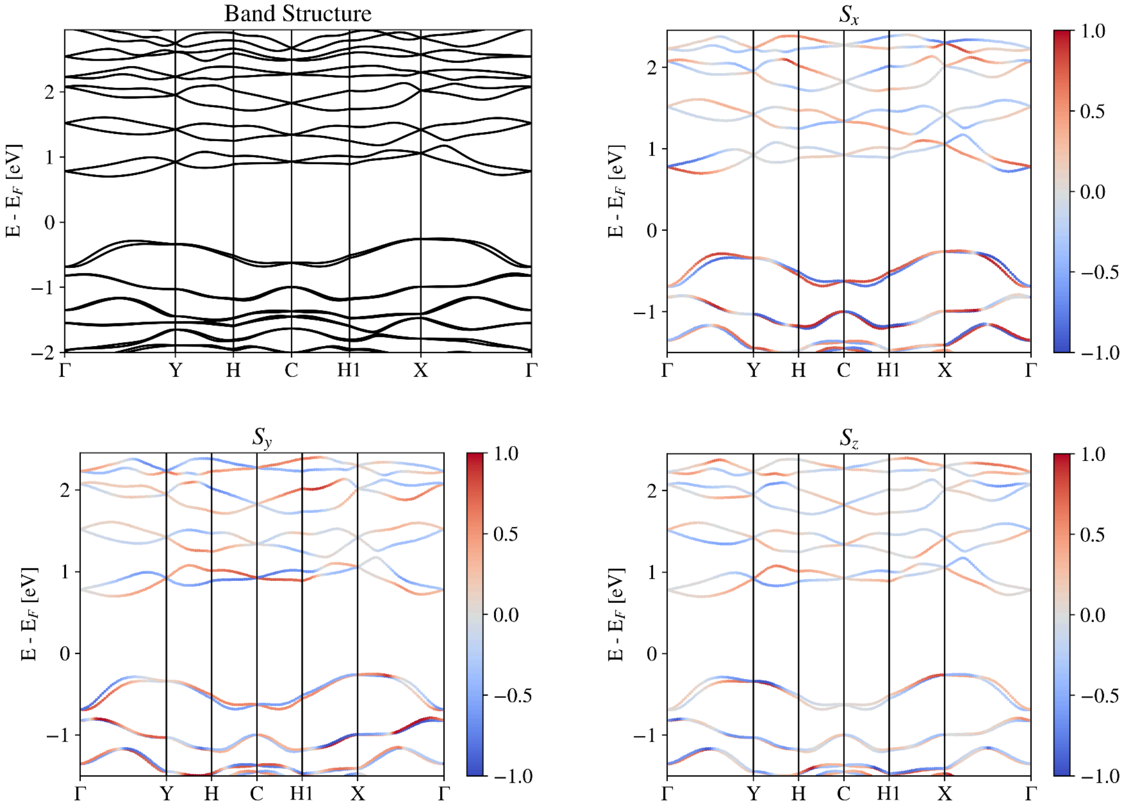


Figure 109: Band structure and spin polarization projections.

## 2.55 Bi2P2Se6-aa9a981d89aa

- **Formula**: Bi2P2Se6
- **Structural Cluster**: ABC3-16
- **Band gap (PBE)**: 0.875 eV
- **Energy above convex hull (C2DB)**: 0.054 eV
- **Space group symbol**: $P1$
- **Space group number**: 1
- **Polar structure**: True

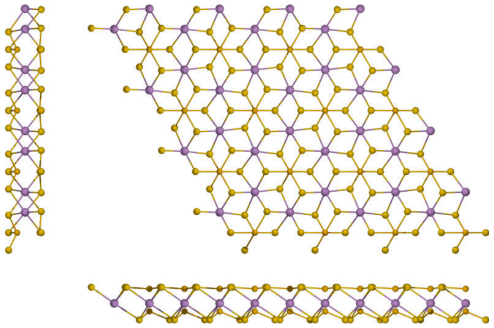

Figure 110: Structure representation

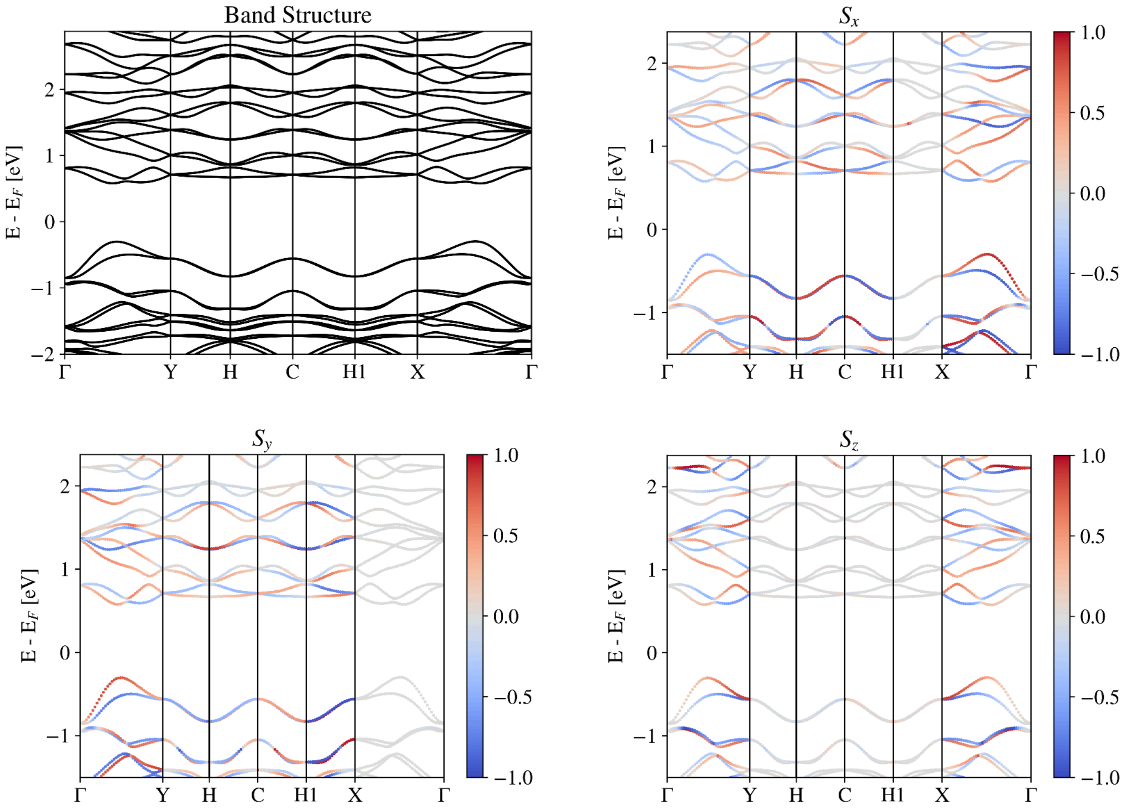


Figure 111: Band structure and spin polarization projections.

## 2.56 Bi2P2Te6-cf7927ab6730

- **Formula**: Bi2P2Te6
- **Structural Cluster**: ABC3-16
- **Band gap (PBE)**: 0.507 eV
- **Energy above convex hull (C2DB)**: 0.14 eV
- **Space group symbol**: $P1$
- **Space group number**: 1
- **Polar structure**: True

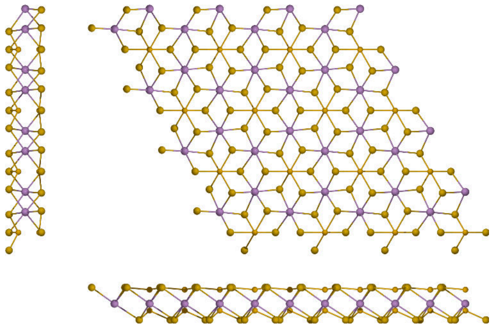

Figure 112: Structure representation

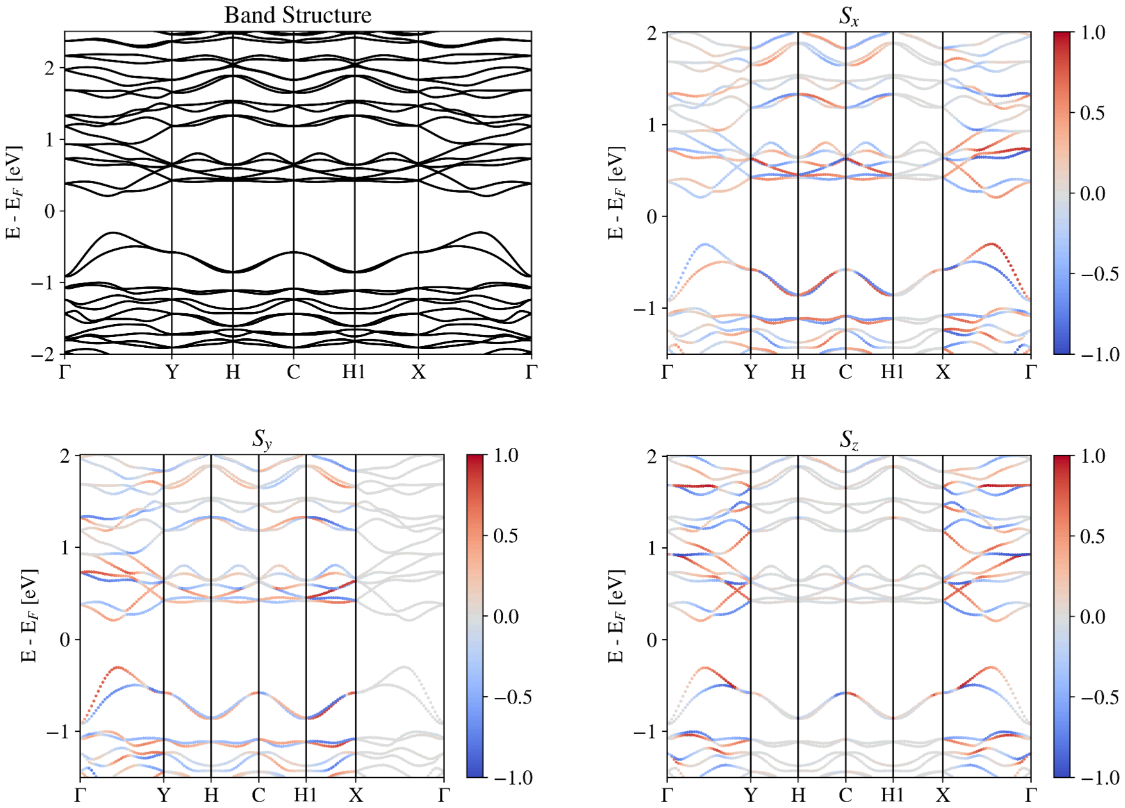


Figure 113: Band structure and spin polarization projections.

## 2.57 Bi2Se2-d13cbee86f79

- **Formula**: Bi2Se2
- **Structural Cluster**: AB-5
- **Band gap (PBE)**: 0.379 eV
- **Energy above convex hull (C2DB)**: 0.209 eV
- **Space group symbol**: $P1$
- **Space group number**: 1
- **Polar structure**: True

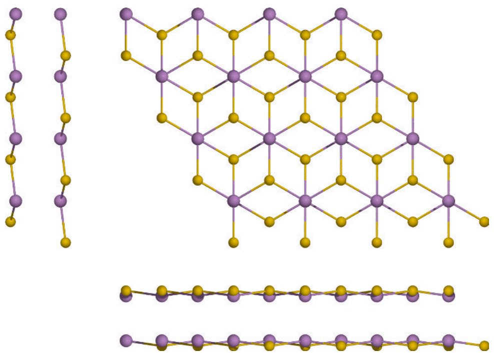

Figure 114: Structure representation

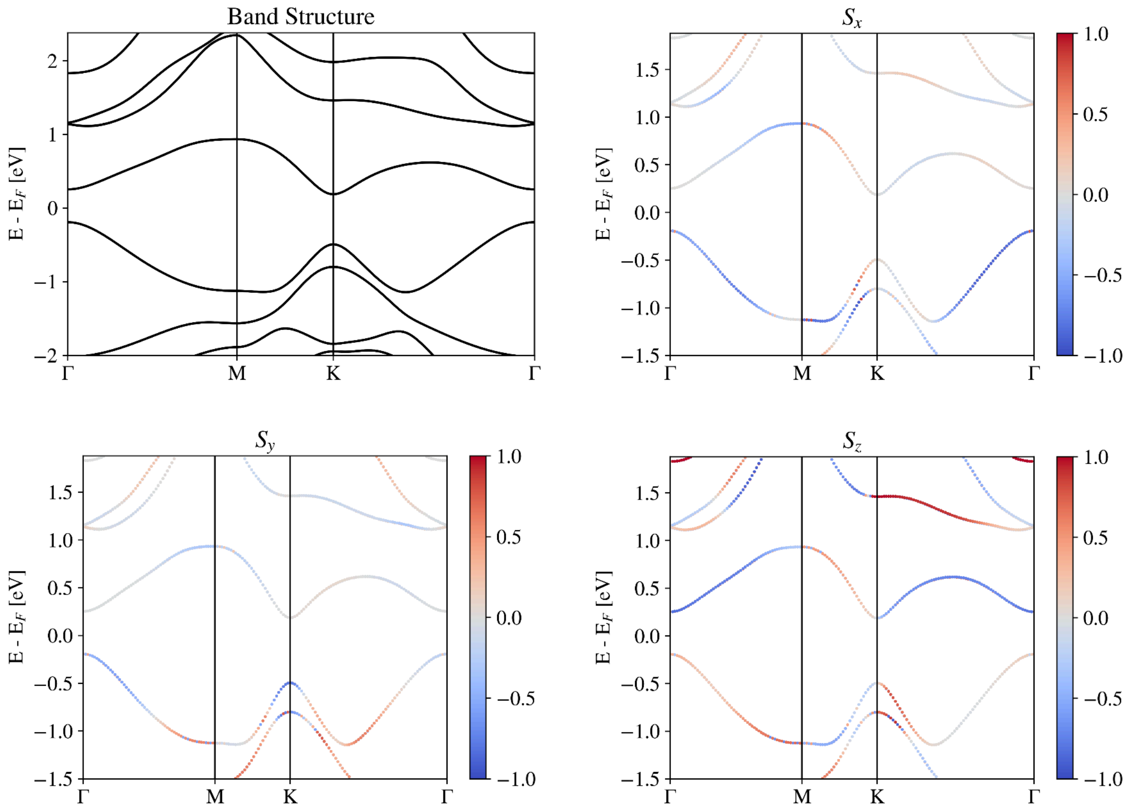


Figure 115: Band structure and spin polarization projections.

## 2.58 Bi2Te2-23a0796492d1

- **Formula**: Bi2Te2
- **Structural Cluster**: AB-5
- **Band gap (PBE)**: 0.37 eV
- **Energy above convex hull (C2DB)**: 0.219 eV
- **Space group symbol**: $P1$
- **Space group number**: 1
- **Polar structure**: True

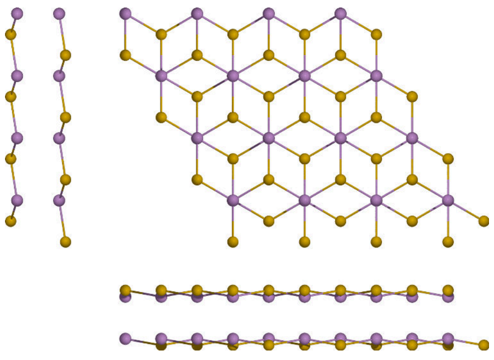

Figure 116: Structure representation

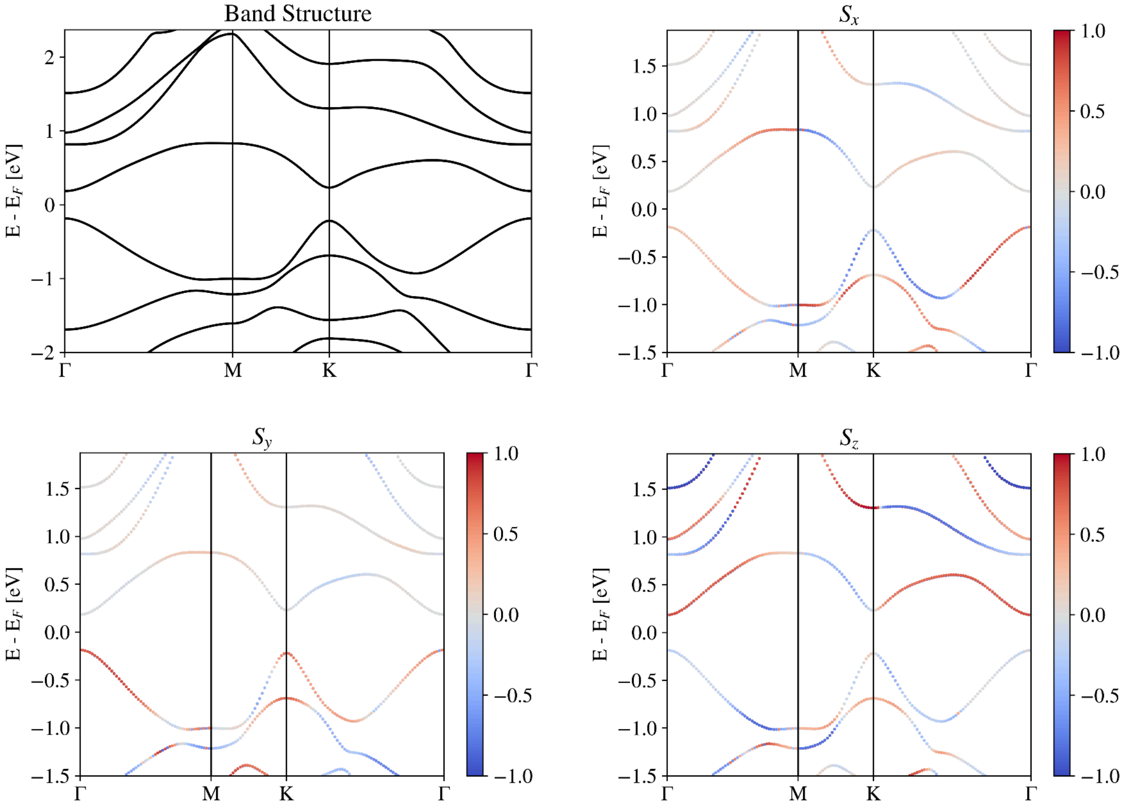


Figure 117: Band structure and spin polarization projections.

## 2.59 BiBrS-3b305c3e2c18

- **Formula**: BiBrS
- **Structural Cluster**: ABC-4
- **Band gap (PBE)**: 1.594 eV
- **Energy above convex hull (C2DB)**: 0.116 eV
- **Space group symbol**: $P3m1$
- **Space group number**: 156
- **Polar structure**: True

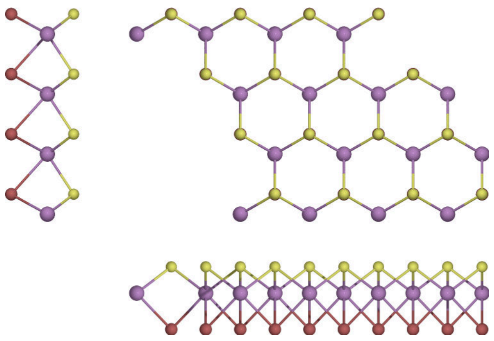

Figure 118: Structure representation

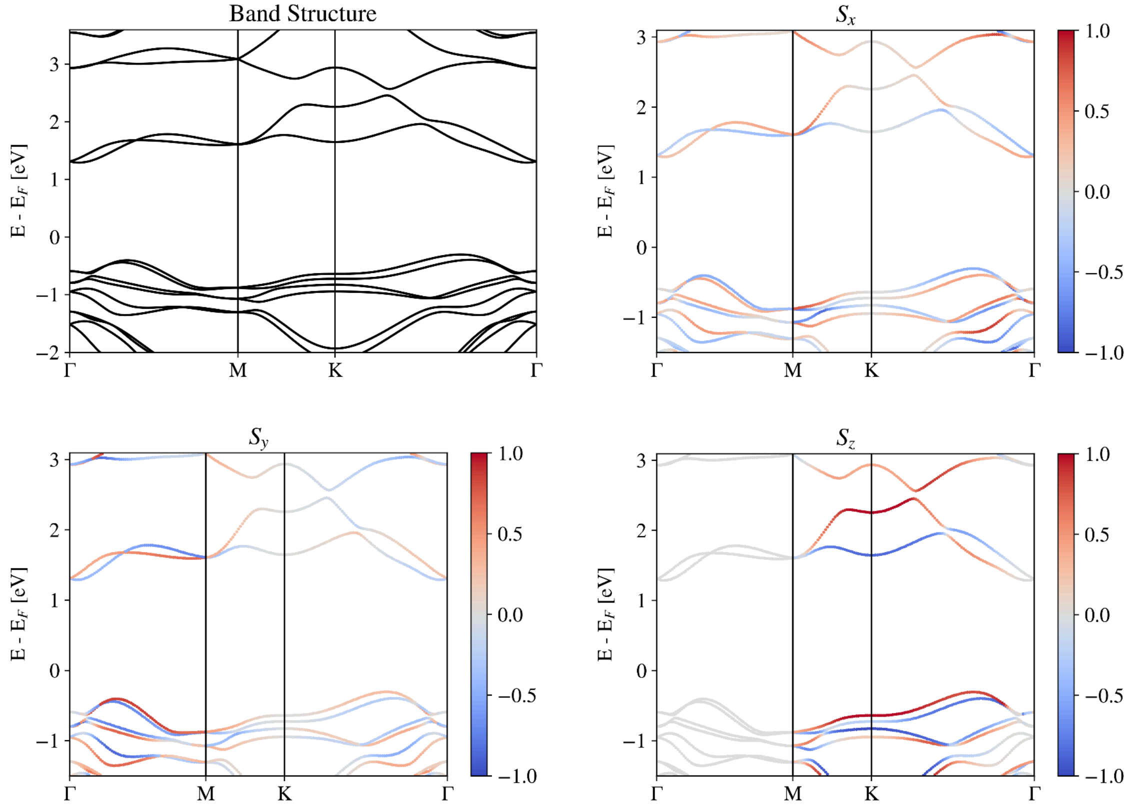


Figure 119: Band structure and spin polarization projections.

## 2.60 BiBrS-49b7be14f786

- **Formula**: BiBrS
- **Structural Cluster**: ABC-3
- **Band gap (PBE)**: 1.227 eV
- **Energy above convex hull (C2DB)**: 0.0 eV
- **Space group symbol**: $P3m1$
- **Space group number**: 156
- **Polar structure**: True

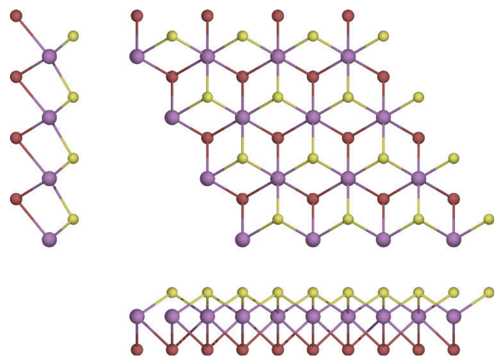

Figure 120: Structure representation

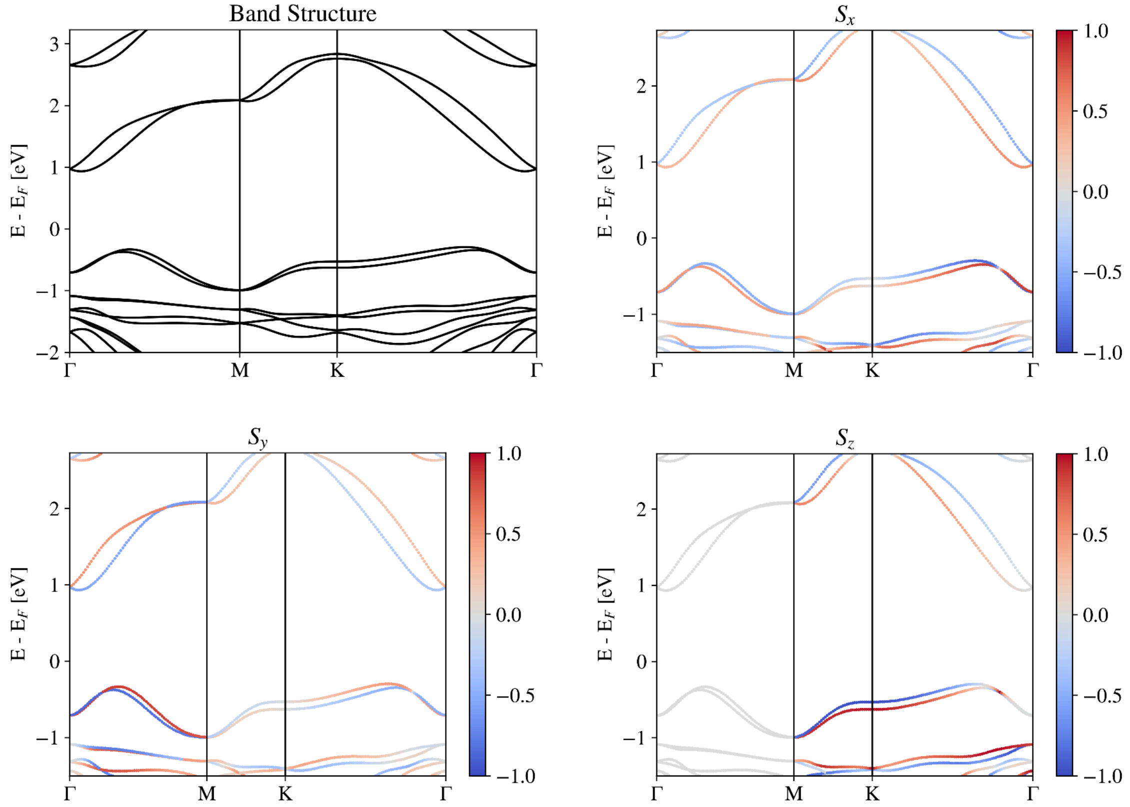


Figure 121: Band structure and spin polarization projections.

## 2.61 BiBrSe-11db0908d9ef

- **Formula**: BiBrSe
- **Structural Cluster**: ABC-4
- **Band gap (PBE)**: 1.385 eV
- **Energy above convex hull (C2DB)**: 0.111 eV
- **Space group symbol**: $P3m1$
- **Space group number**: 156
- **Polar structure**: True

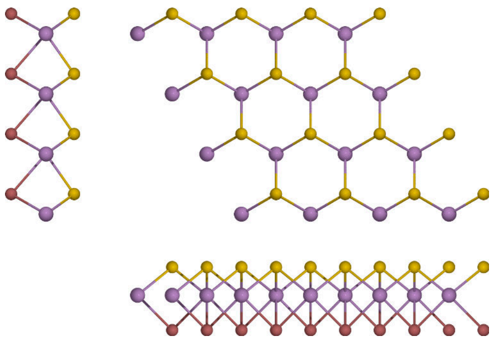

Figure 122: Structure representation

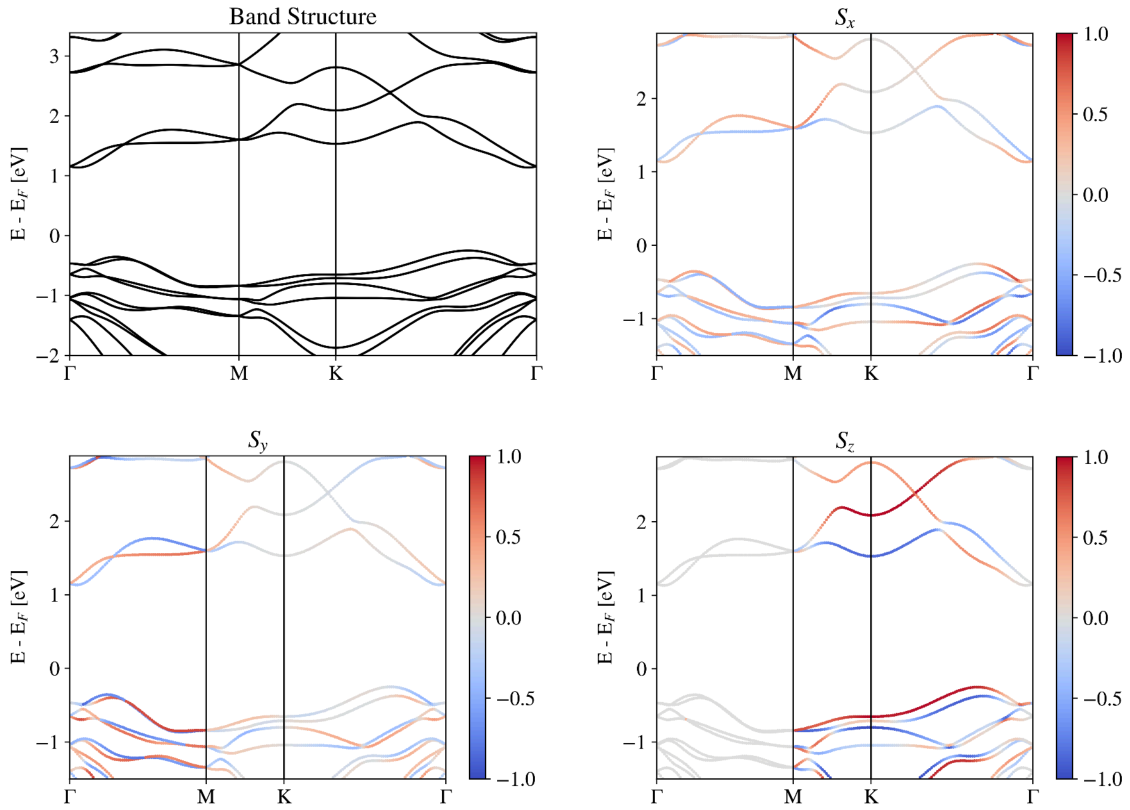


Figure 123: Band structure and spin polarization projections.

## 2.62 BiBrSe-de5756e4fbfa

- **Formula**: BiBrSe
- **Structural Cluster**: ABC-3
- **Band gap (PBE)**: 1.03 eV
- **Energy above convex hull (C2DB)**: 0.0 eV
- **Space group symbol**: $P3m1$
- **Space group number**: 156
- **Polar structure**: True

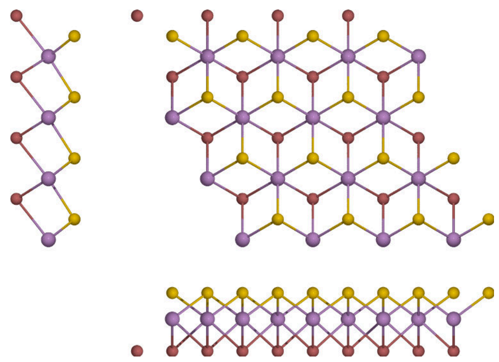

Figure 124: Structure representation

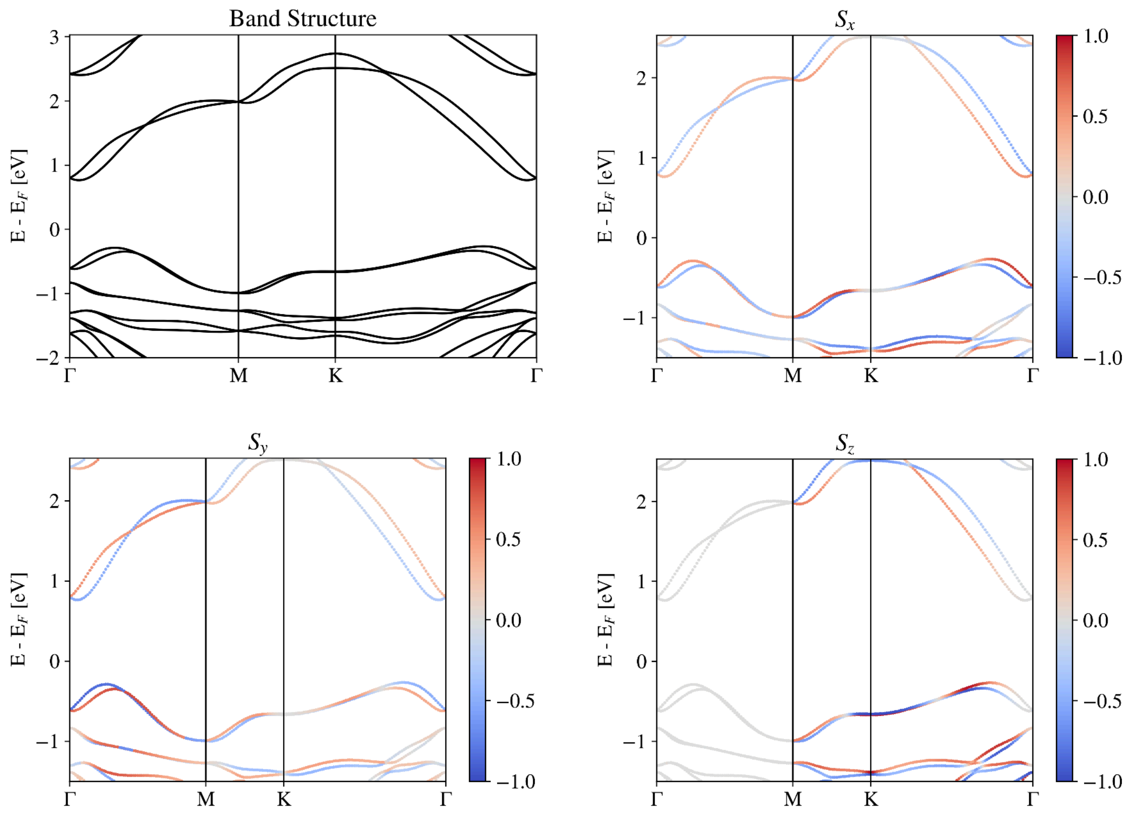


Figure 125: Band structure and spin polarization projections.

## 2.63 BiBrTe-304bc6a92d82

- **Formula**: BiBrTe
- **Structural Cluster**: ABC-3
- **Band gap (PBE)**: 0.878 eV
- **Energy above convex hull (C2DB)**: 0.0 eV
- **Space group symbol**: $P3m1$
- **Space group number**: 156
- **Polar structure**: True

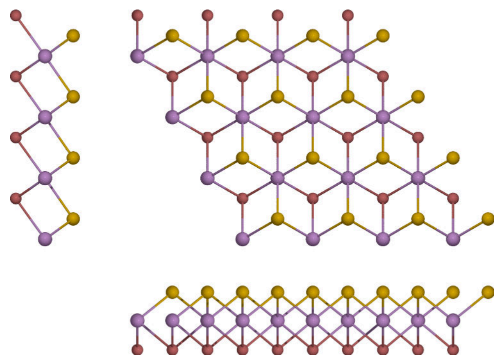

Figure 126: Structure representation

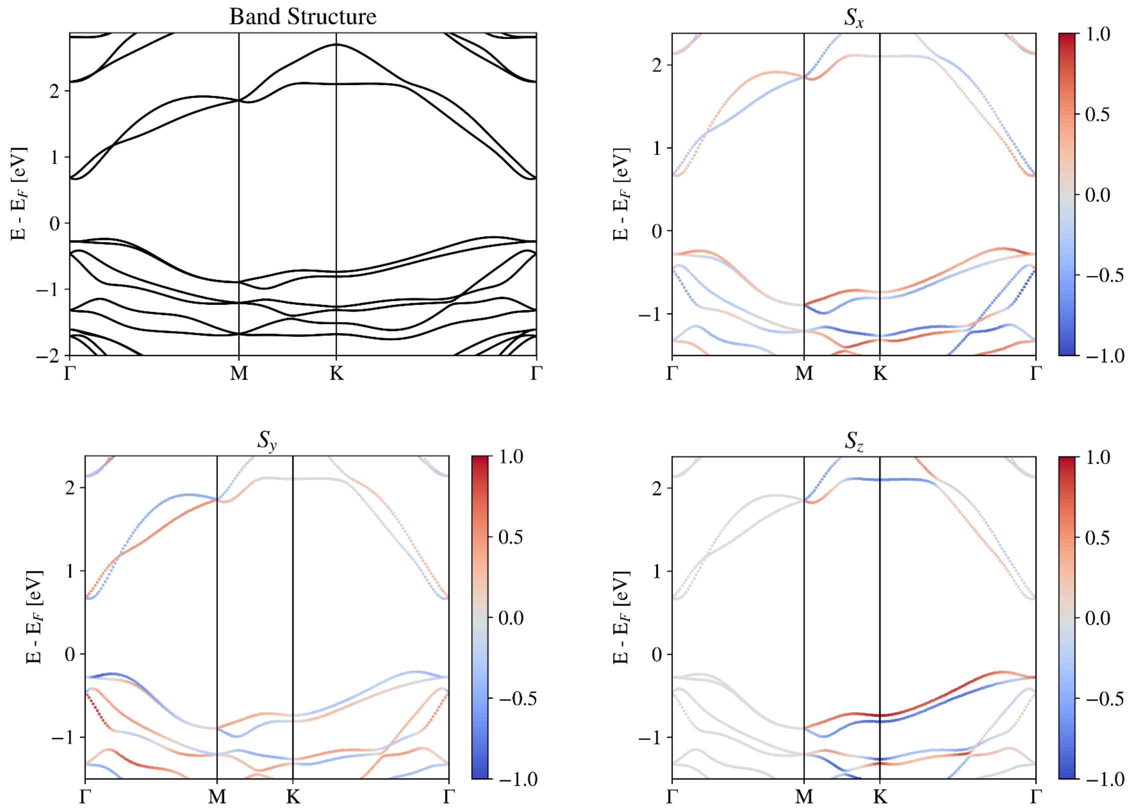


Figure 127: Band structure and spin polarization projections.

## 2.64 BiBrTe-f4f45fcade85

- **Formula**: BiBrTe
- **Structural Cluster**: ABC-4
- **Band gap (PBE)**: 0.916 eV
- **Energy above convex hull (C2DB)**: 0.117 eV
- **Space group symbol**: $P3m1$
- **Space group number**: 156
- **Polar structure**: True

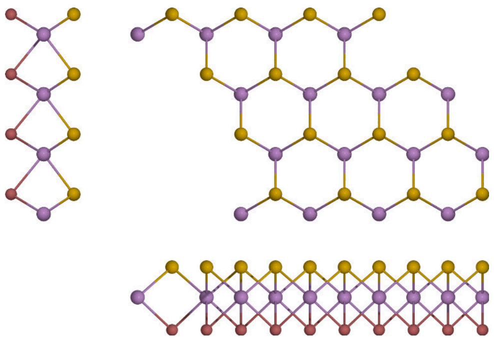

Figure 128: Structure representation

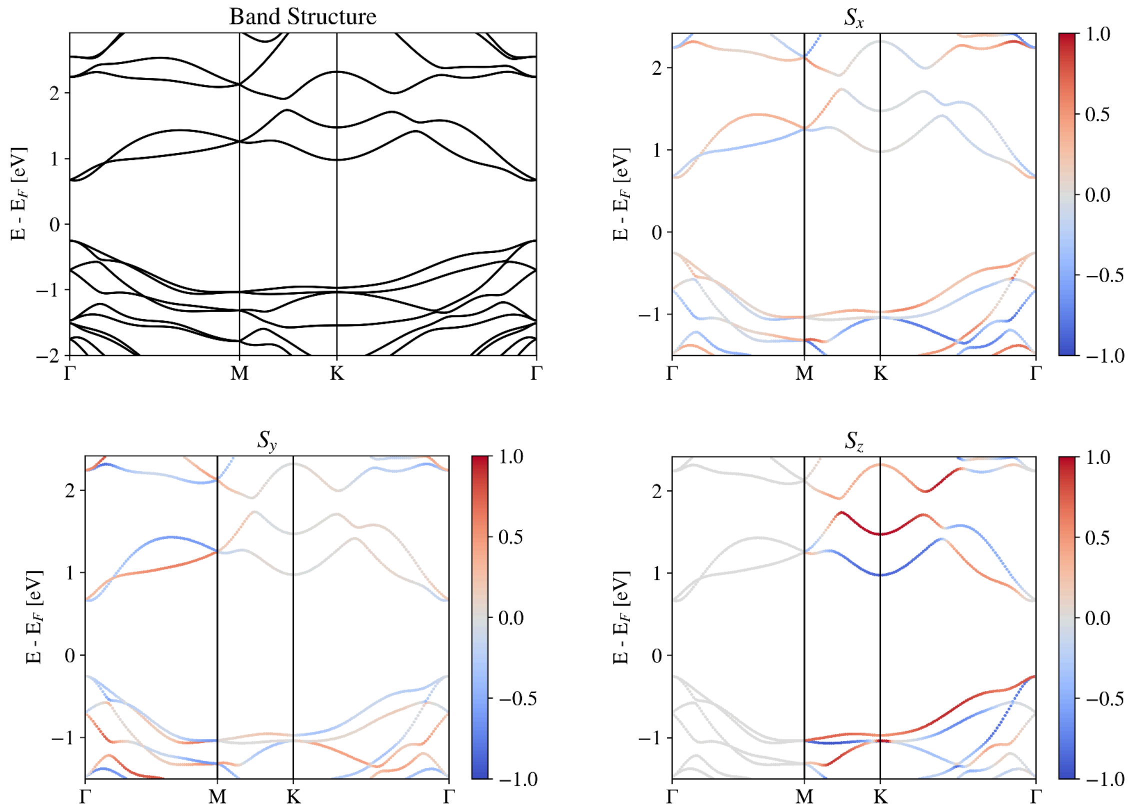


Figure 129: Band structure and spin polarization projections.

## 2.65 BiClS-99fd027b1d0b

- **Formula**: BiClS
- **Structural Cluster**: ABC-4
- **Band gap (PBE)**: 1.841 eV
- **Energy above convex hull (C2DB)**: 0.12 eV
- **Space group symbol**: $P3m1$
- **Space group number**: 156
- **Polar structure**: True

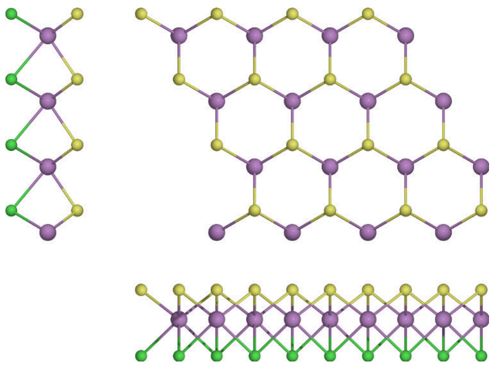

Figure 130: Structure representation

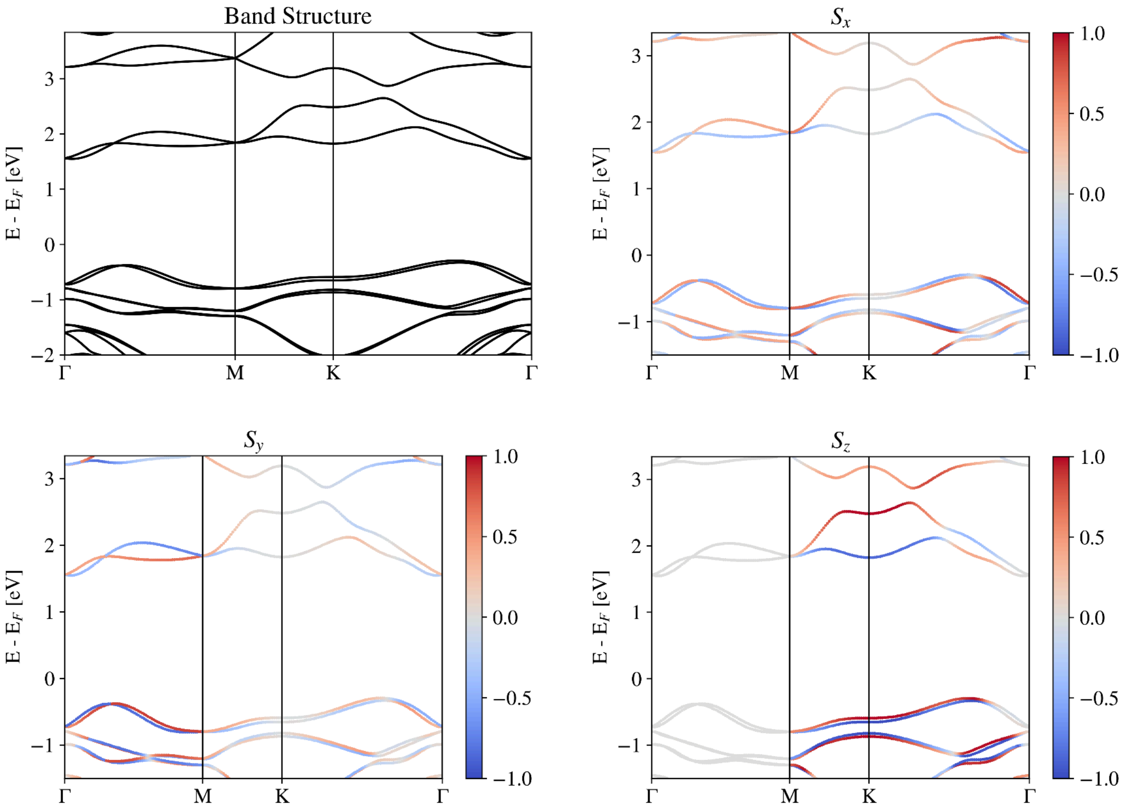


Figure 131: Band structure and spin polarization projections.

## 2.66 BiClS-c96ef4fc869c

- **Formula**: BiClS
- **Structural Cluster**: ABC-3
- **Band gap (PBE)**: 1.334 eV
- **Energy above convex hull (C2DB)**: 0.0 eV
- **Space group symbol**: $P3m1$
- **Space group number**: 156
- **Polar structure**: True

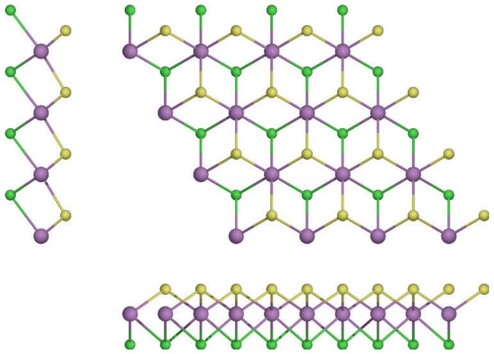

Figure 132: Structure representation

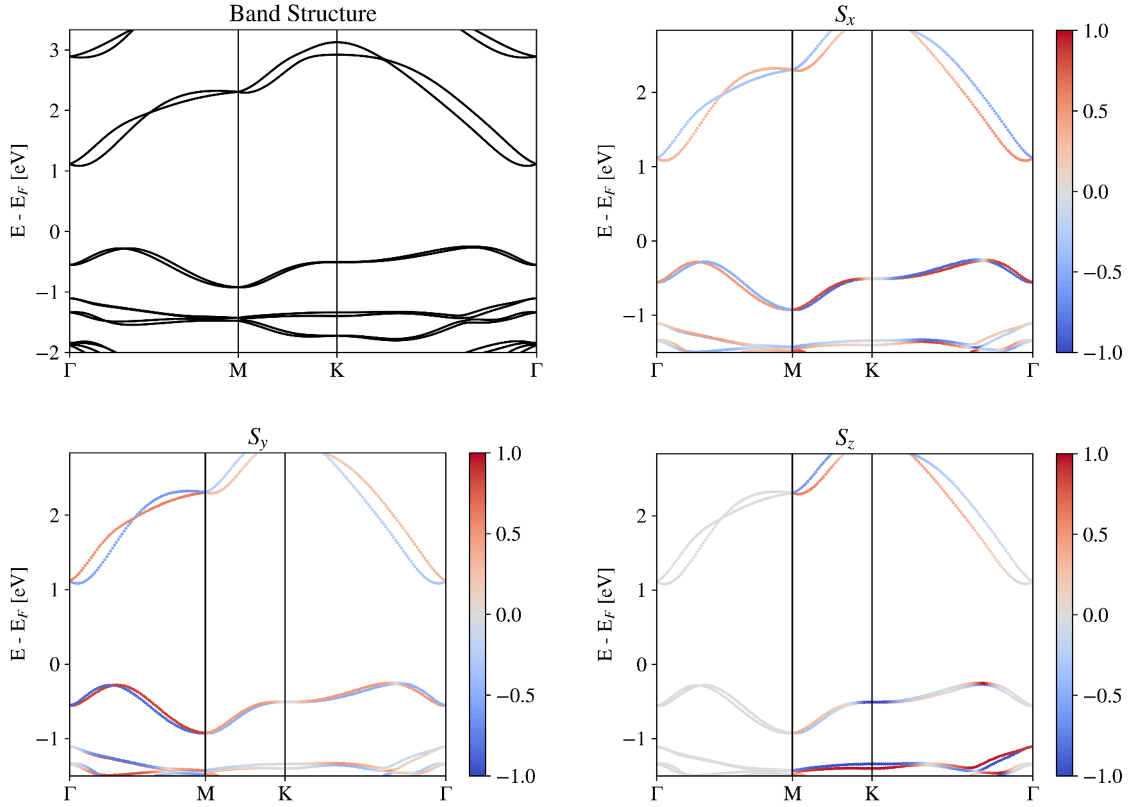


Figure 133: Band structure and spin polarization projections.

## 2.67 BiClSe-7fe9c5cb910c

- **Formula**: BiClSe
- **Structural Cluster**: ABC-4
- **Band gap (PBE)**: 1.601 eV
- **Energy above convex hull (C2DB)**: 0.119 eV
- **Space group symbol**: $P3m1$
- **Space group number**: 156
- **Polar structure**: True

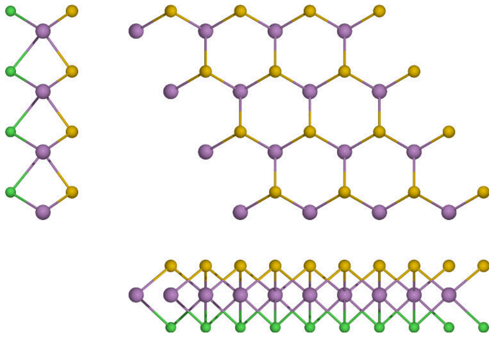

Figure 134: Structure representation

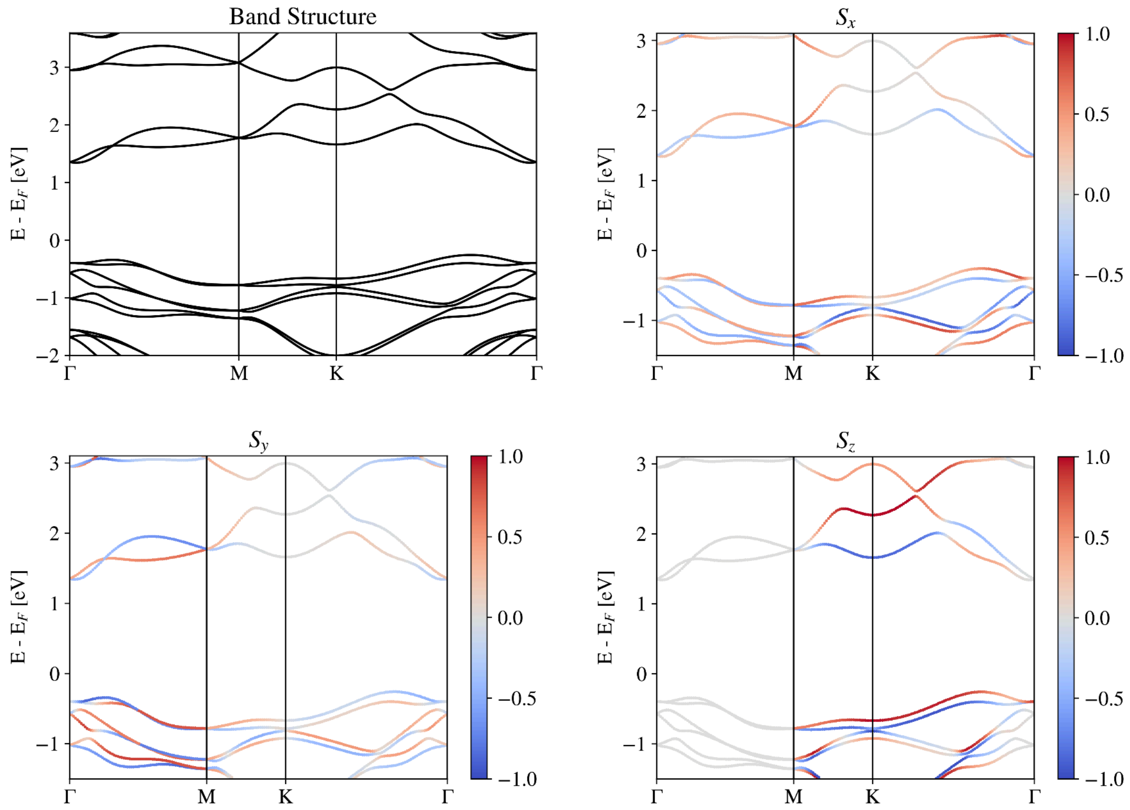


Figure 135: Band structure and spin polarization projections.

## 2.68 BiClSe-a80866a2c6b4

- **Formula**: BiClSe
- **Structural Cluster**: ABC-3
- **Band gap (PBE)**: 1.139 eV
- **Energy above convex hull (C2DB)**: 0.0 eV
- **Space group symbol**: $P3m1$
- **Space group number**: 156
- **Polar structure**: True

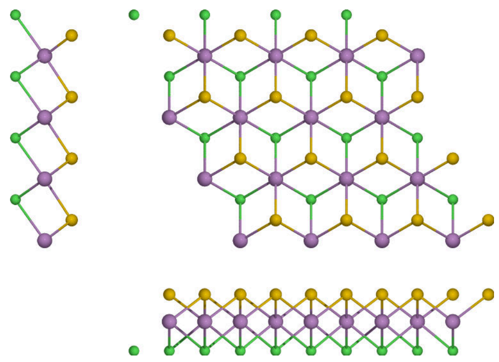

Figure 136: Structure representation

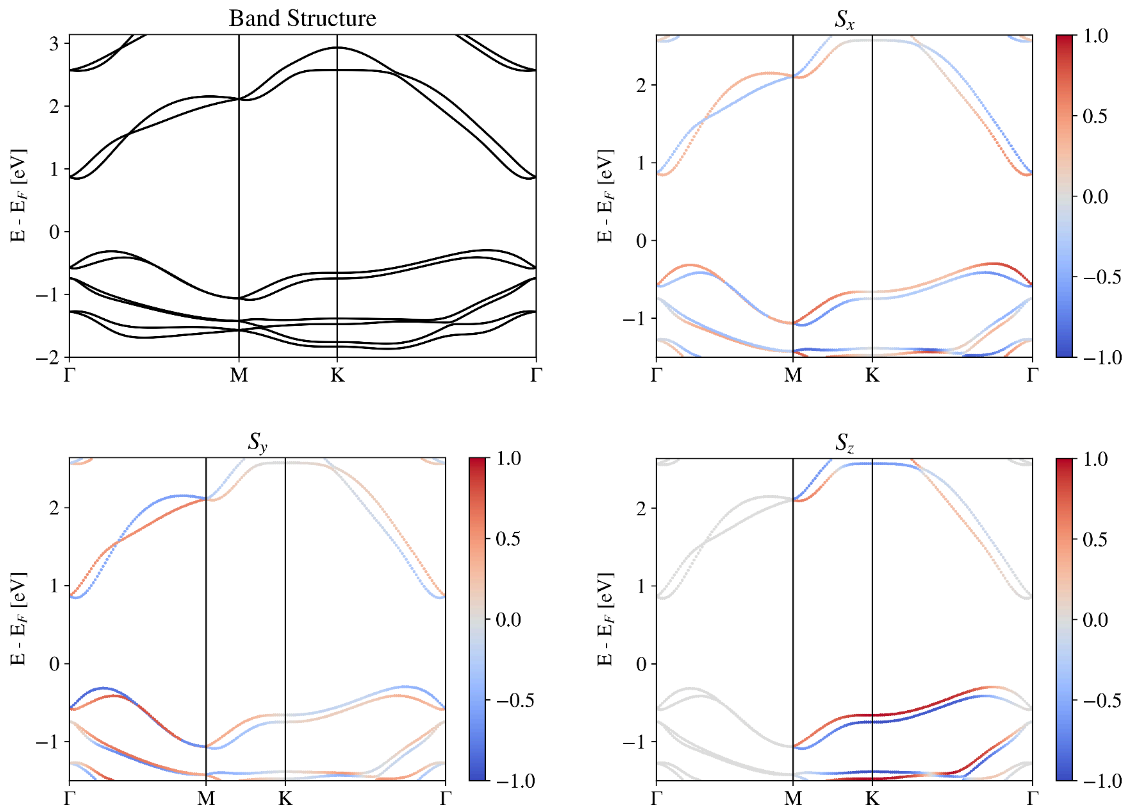


Figure 137: Band structure and spin polarization projections.

## 2.69 BiClTe-968a6902b7f5

- **Formula**: BiClTe
- **Structural Cluster**: ABC-3
- **Band gap (PBE)**: 0.938 eV
- **Energy above convex hull (C2DB)**: 0.0 eV
- **Space group symbol**: $P3m1$
- **Space group number**: 156
- **Polar structure**: True

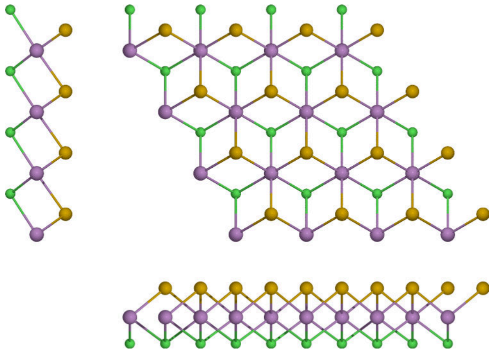

Figure 138: Structure representation

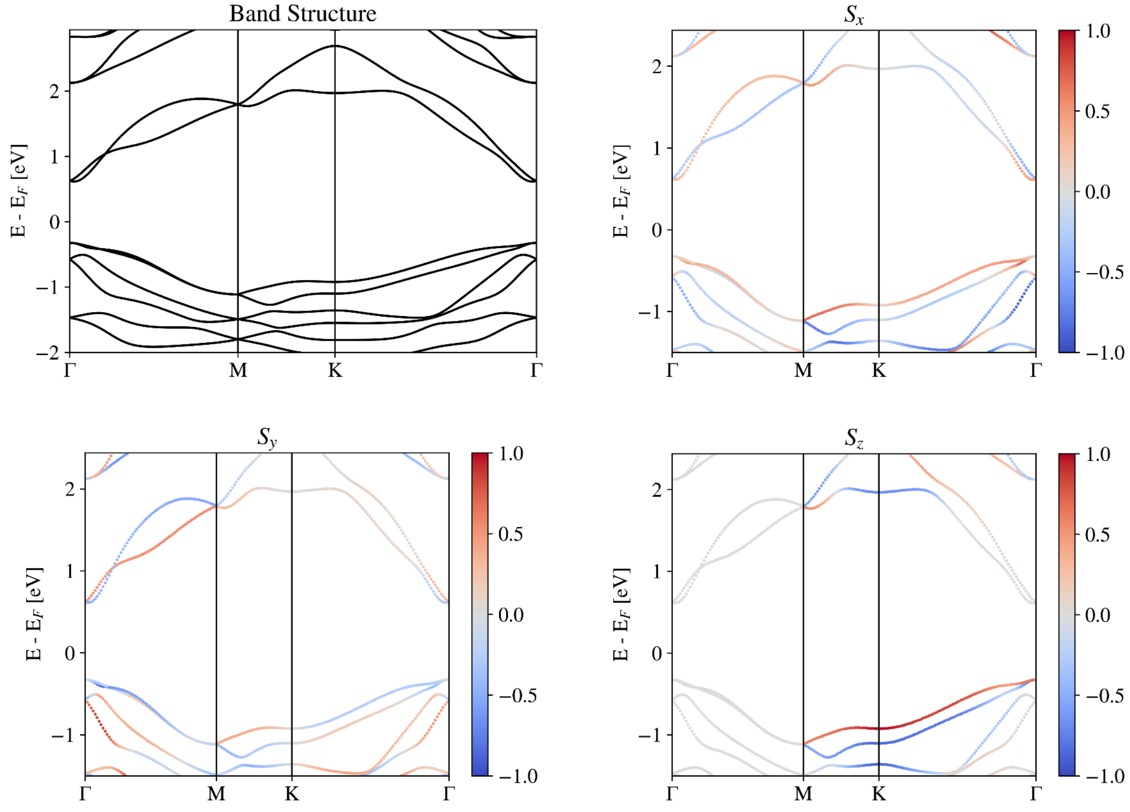


Figure 139: Band structure and spin polarization projections.

## 2.70 BiClTe-badda86cab42

- **Formula**: BiClTe
- **Structural Cluster**: ABC-4
- **Band gap (PBE)**: 0.948 eV
- **Energy above convex hull (C2DB)**: 0.129 eV
- **Space group symbol**: $P3m1$
- **Space group number**: 156
- **Polar structure**: True

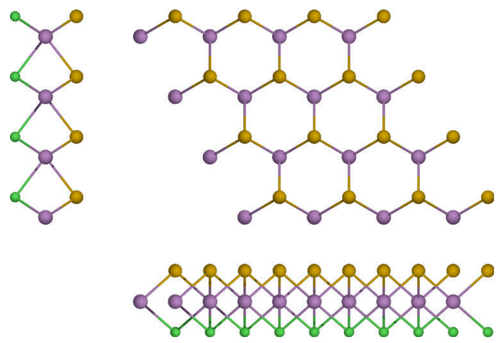

Figure 140: Structure representation

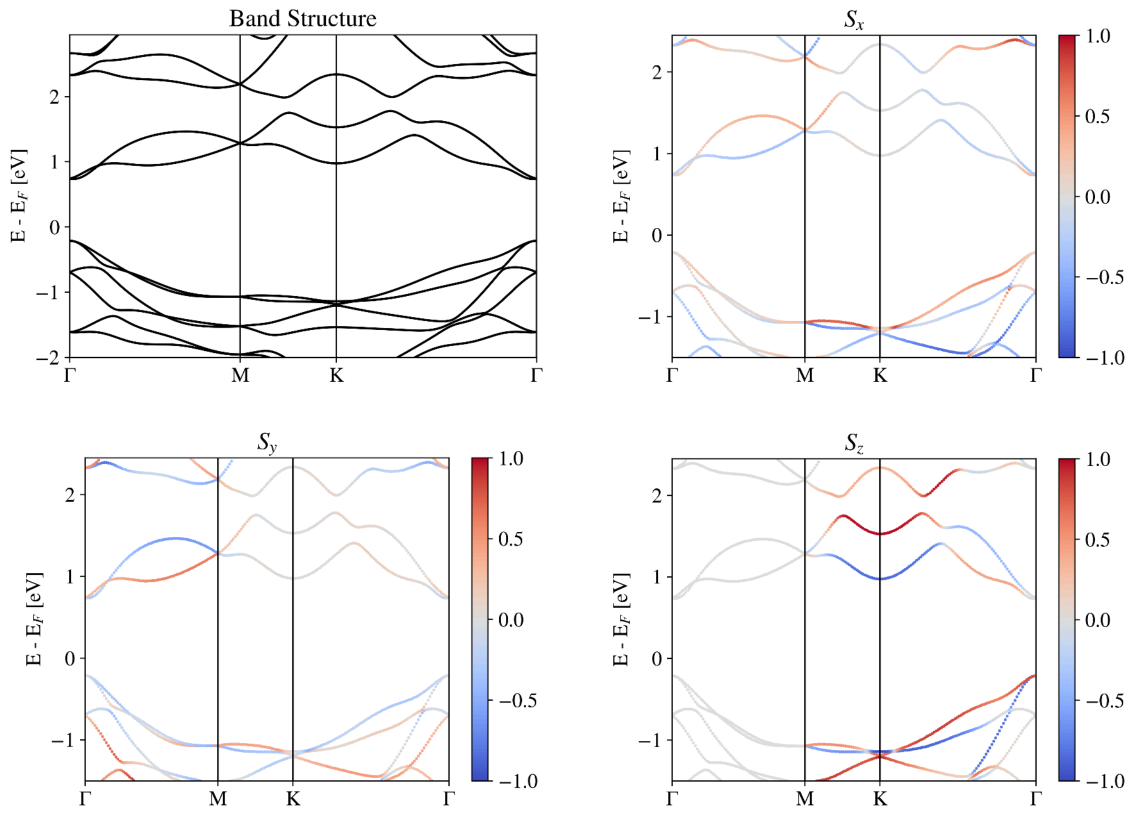


Figure 141: Band structure and spin polarization projections.

## 2.71 BiIS-40034665f9f1

- **Formula**: BiIS
- **Structural Cluster**: ABC-4
- **Band gap (PBE)**: 0.848 eV
- **Energy above convex hull (C2DB)**: 0.14 eV
- **Space group symbol**: $P3m1$
- **Space group number**: 156
- **Polar structure**: True

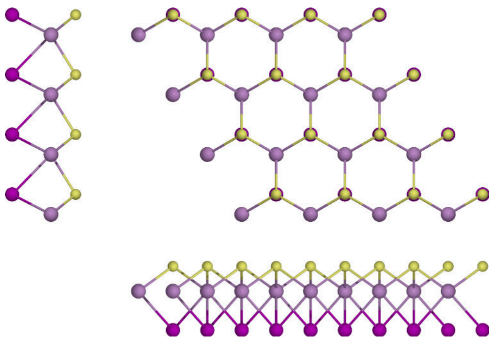

Figure 142: Structure representation

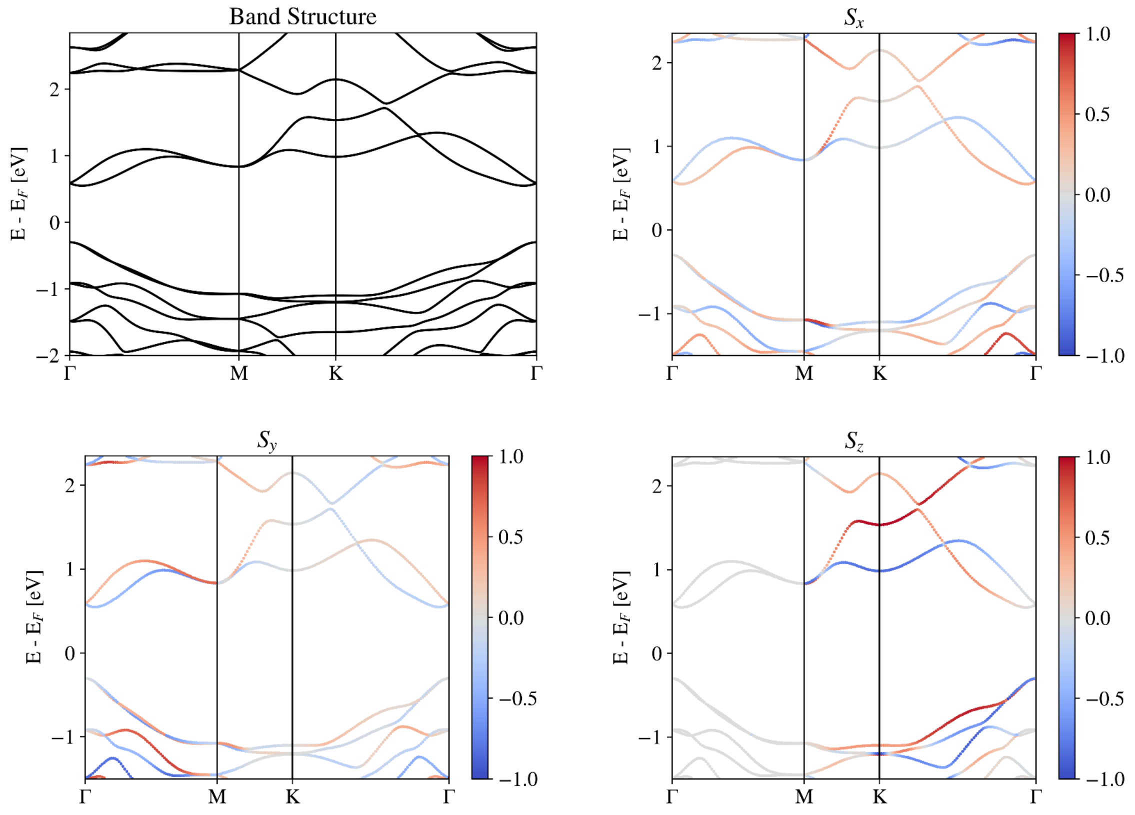


Figure 143: Band structure and spin polarization projections.

## 2.72 BiIS-acdcd16c0d76

- **Formula**: BiIS
- **Structural Cluster**: ABC-3
- **Band gap (PBE)**: 1.139 eV
- **Energy above convex hull (C2DB)**: 0.014 eV
- **Space group symbol**: $P3m1$
- **Space group number**: 156
- **Polar structure**: True

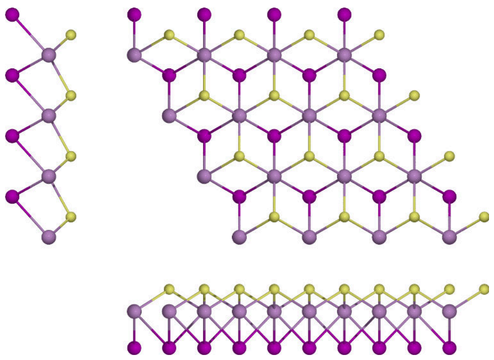

Figure 144: Structure representation

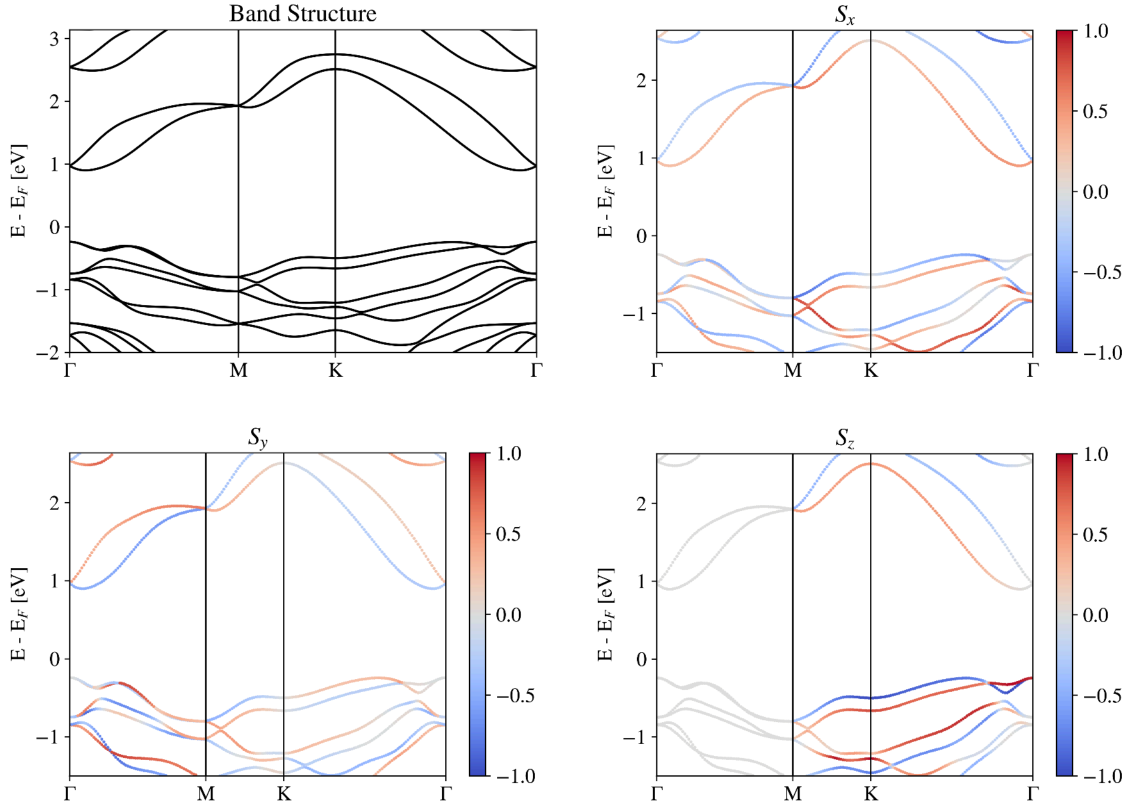


Figure 145: Band structure and spin polarization projections.

## 2.73 BiISe-433f707c632c

- **Formula**: BiISe
- **Structural Cluster**: ABC-4
- **Band gap (PBE)**: 0.84 eV
- **Energy above convex hull (C2DB)**: 0.114 eV
- **Space group symbol**: $P3m1$
- **Space group number**: 156
- **Polar structure**: True

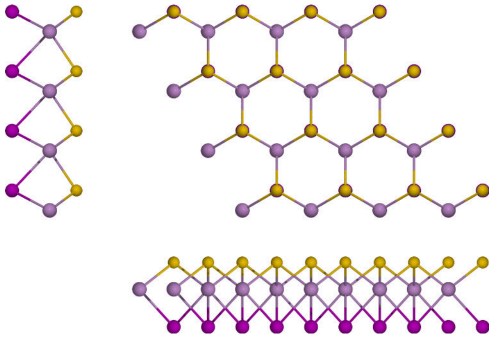

Figure 146: Structure representation

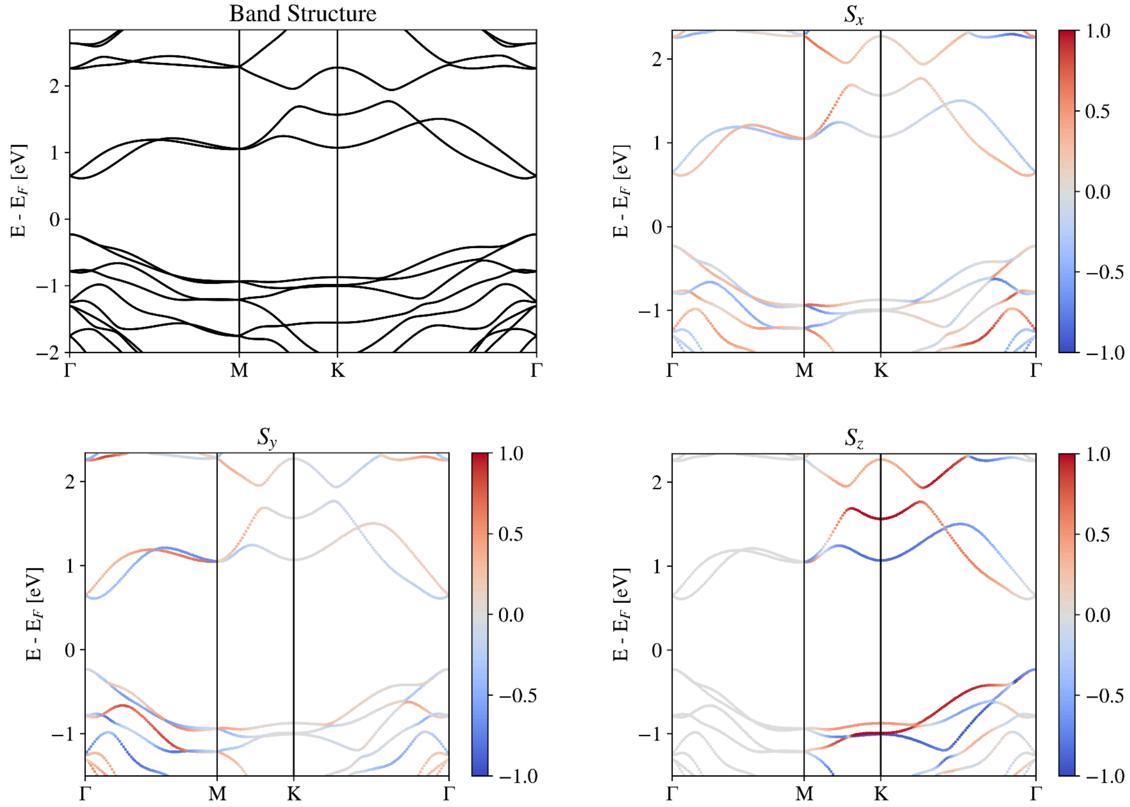


Figure 147: Band structure and spin polarization projections.

## 2.74 BiISe-70cbc0e44d36

- **Formula**: BiISe
- **Structural Cluster**: ABC-3
- **Band gap (PBE)**: 0.929 eV
- **Energy above convex hull (C2DB)**: 0.0 eV
- **Space group symbol**: $P3m1$
- **Space group number**: 156
- **Polar structure**: True

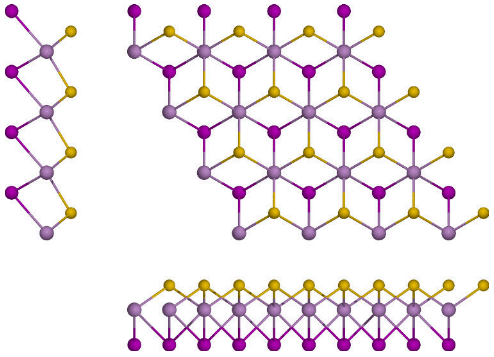

Figure 148: Structure representation

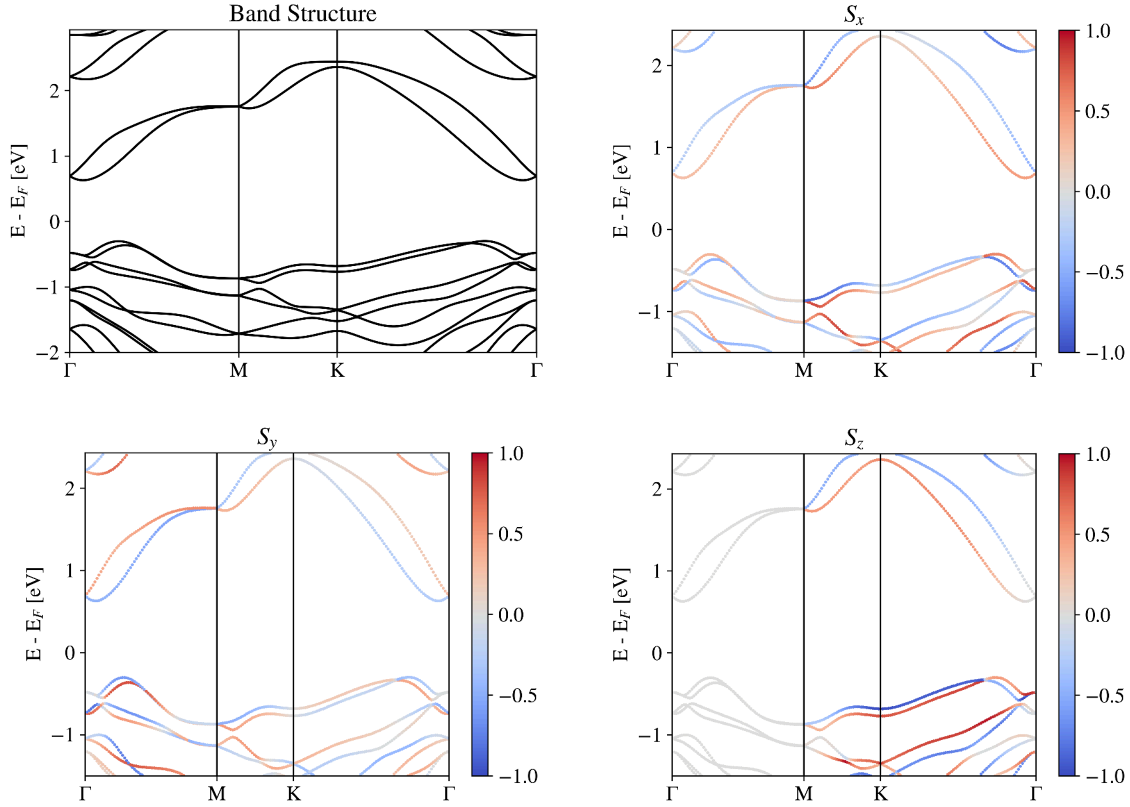


Figure 149: Band structure and spin polarization projections.

## 2.75 BiITe-2d41b3dd1772

- **Formula**: BiITe
- **Structural Cluster**: ABC-3
- **Band gap (PBE)**: 0.701 eV
- **Energy above convex hull (C2DB)**: 0.0 eV
- **Space group symbol**: $P3m1$
- **Space group number**: 156
- **Polar structure**: True

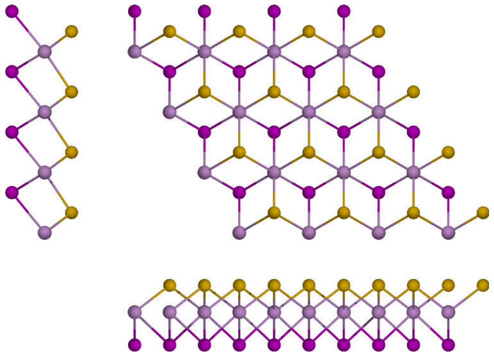

Figure 150: Structure representation

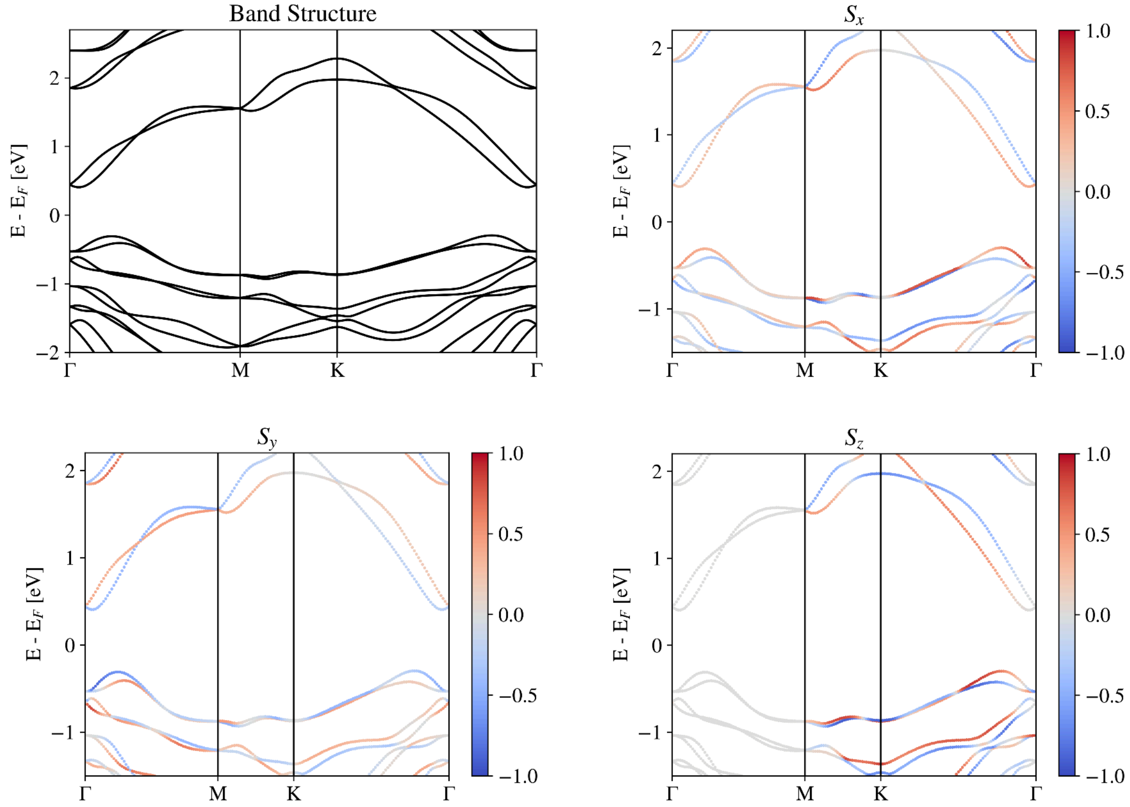


Figure 151: Band structure and spin polarization projections.

## 2.76 BiITe-a84d988e38ac

- **Formula**: BiITe
- **Structural Cluster**: ABC-4
- **Band gap (PBE)**: 0.691 eV
- **Energy above convex hull (C2DB)**: 0.11 eV
- **Space group symbol**: $P3m1$
- **Space group number**: 156
- **Polar structure**: True

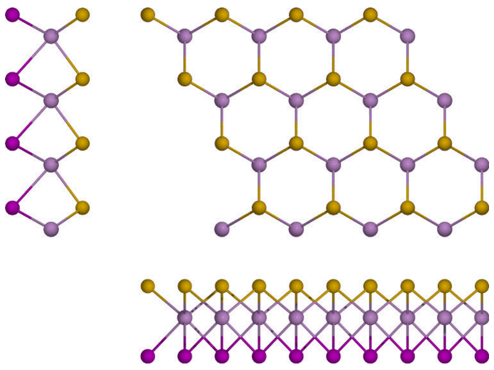

Figure 152: Structure representation

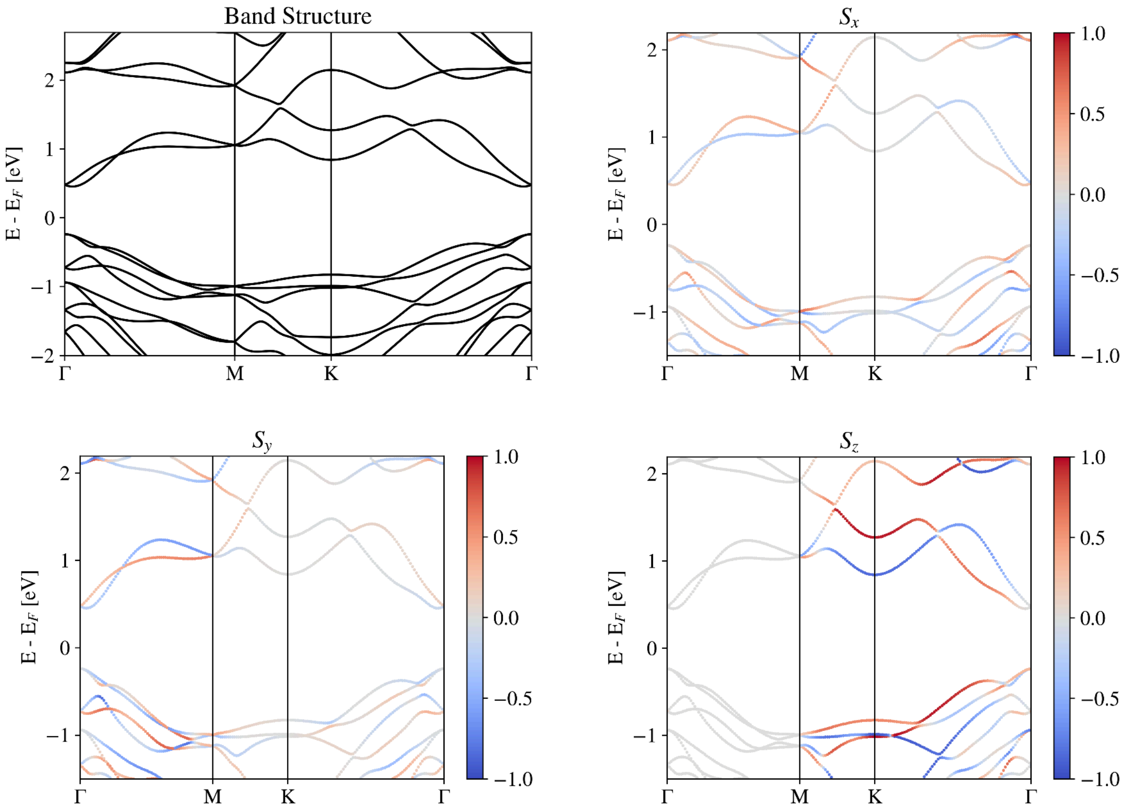


Figure 153: Band structure and spin polarization projections.

## 2.77 Br2S2Tl2-b10aab79a70e

- **Formula**: Br2S2Tl2
- **Structural Cluster**: ABC-11
- **Band gap (PBE)**: 0.282 eV
- **Energy above convex hull (C2DB)**: 0.345 eV
- **Space group symbol**: *Pmm2*
- **Space group number**: 25
- **Polar structure**: True

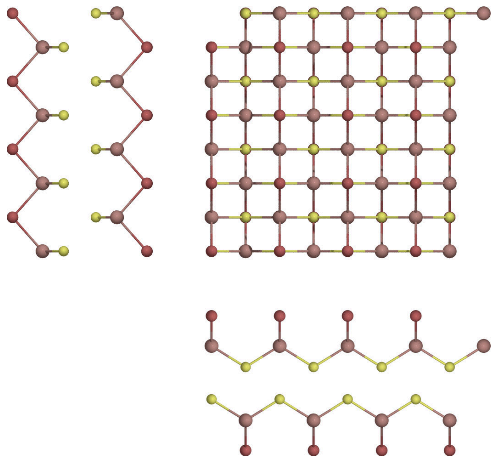

Figure 154: Structure representation

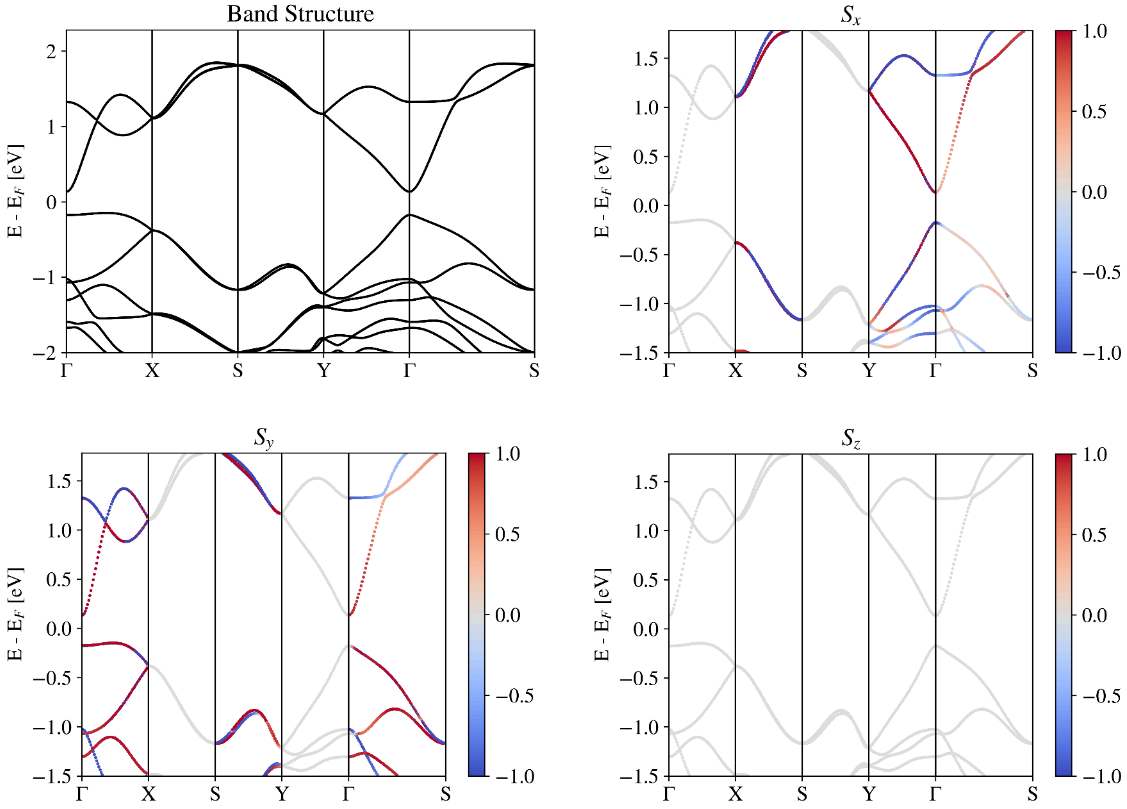


Figure 155: Band structure and spin polarization projections.

## 2.78 Br2Tl2-948c61cd5626

- **Formula**: Br2Tl2
- **Structural Cluster**: AB-8
- **Band gap (PBE)**: 3.311 eV
- **Energy above convex hull (C2DB)**: 0.063 eV
- **Space group symbol**: $P1$
- **Space group number**: 1
- **Polar structure**: True

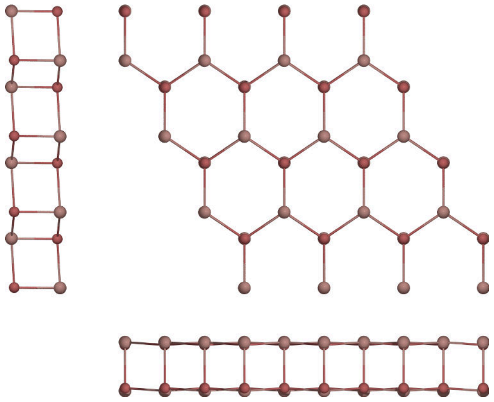

Figure 156: Structure representation

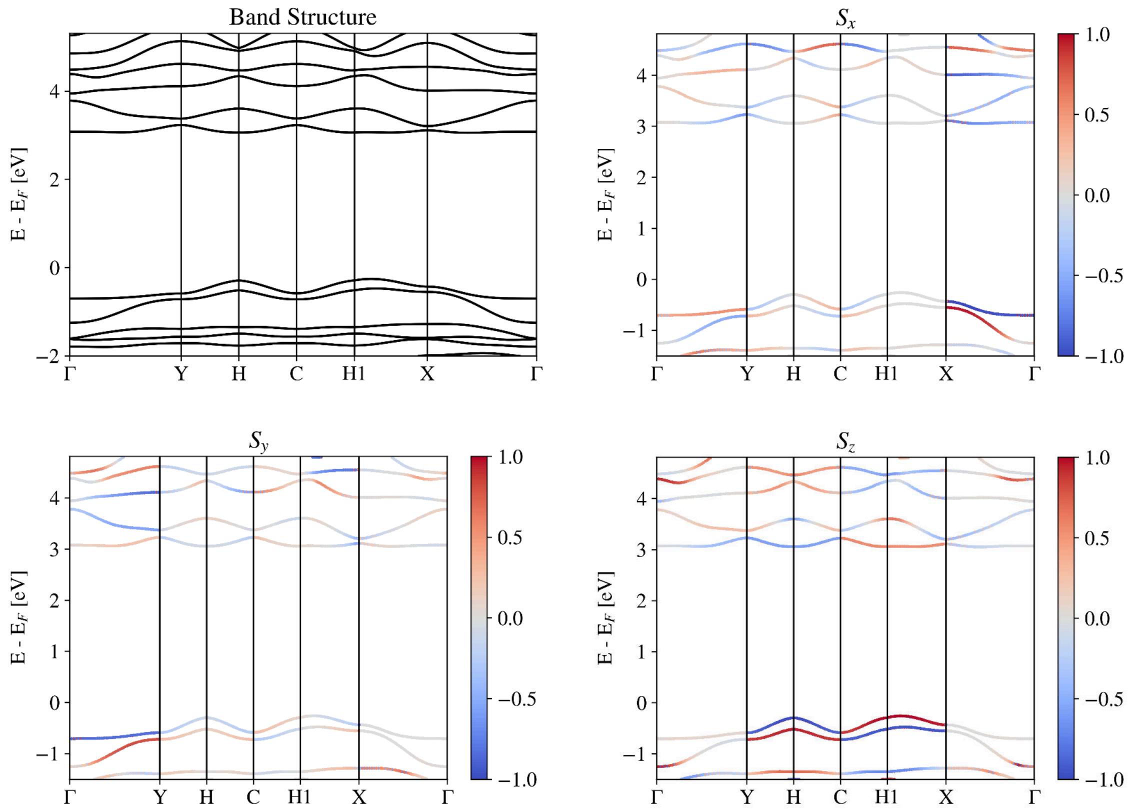


Figure 157: Band structure and spin polarization projections.

## 2.79 BrClHf-72257f9ad66d

- **Formula**: BrClHf
- **Structural Cluster**: ABC-4
- **Band gap (PBE)**: 0.819 eV
- **Energy above convex hull (C2DB)**: 0.016 eV
- **Space group symbol**: $P3m1$
- **Space group number**: 156
- **Polar structure**: True

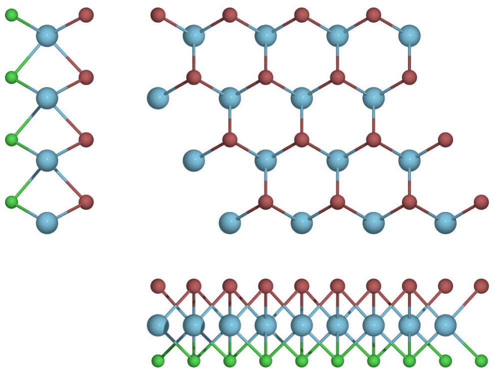

Figure 158: Structure representation

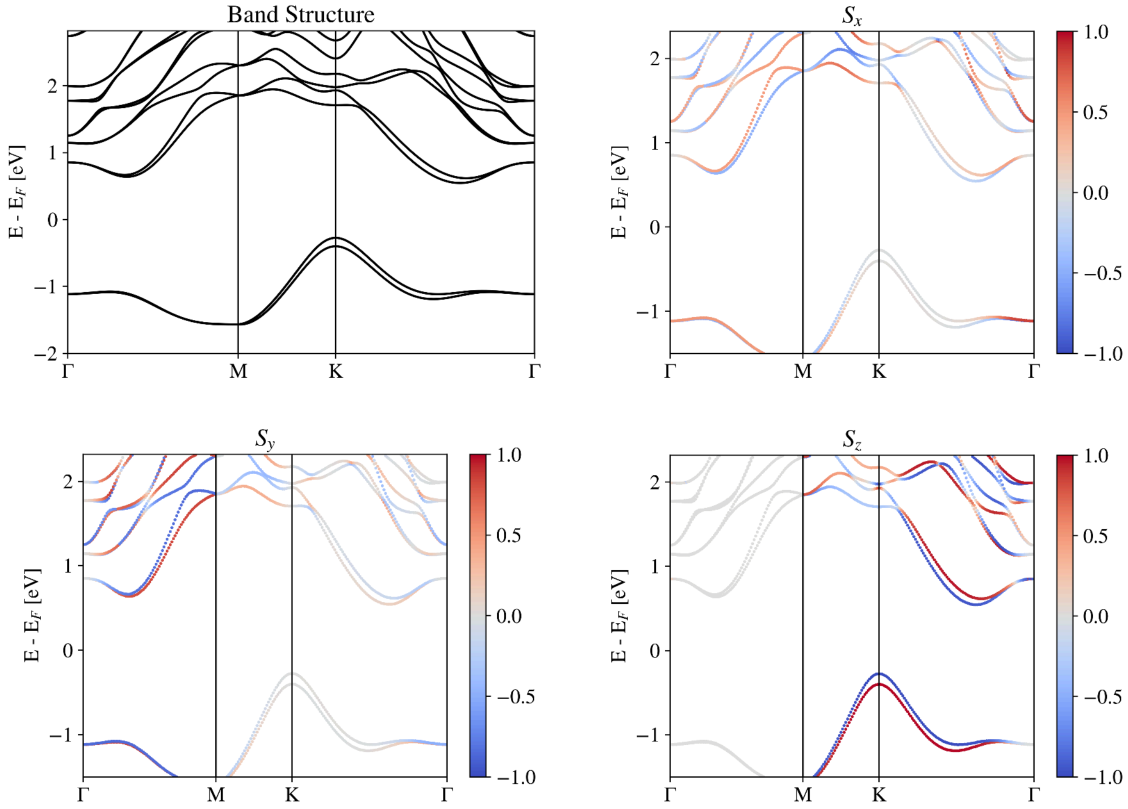


Figure 159: Band structure and spin polarization projections.

## 2.80 BrClTi-d3f135b9cf41

- **Formula**: BrClTi
- **Structural Cluster**: ABC-4
- **Band gap (PBE)**: 0.826 eV
- **Energy above convex hull (C2DB)**: 0.015 eV
- **Space group symbol**: $P3m1$
- **Space group number**: 156
- **Polar structure**: True

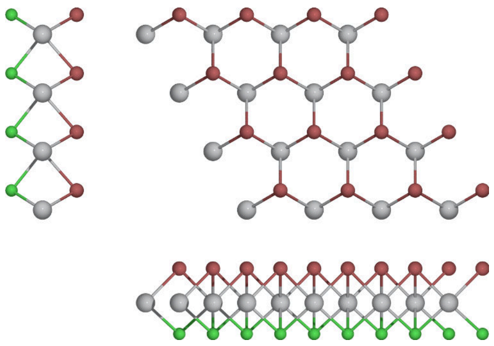

Figure 160: Structure representation

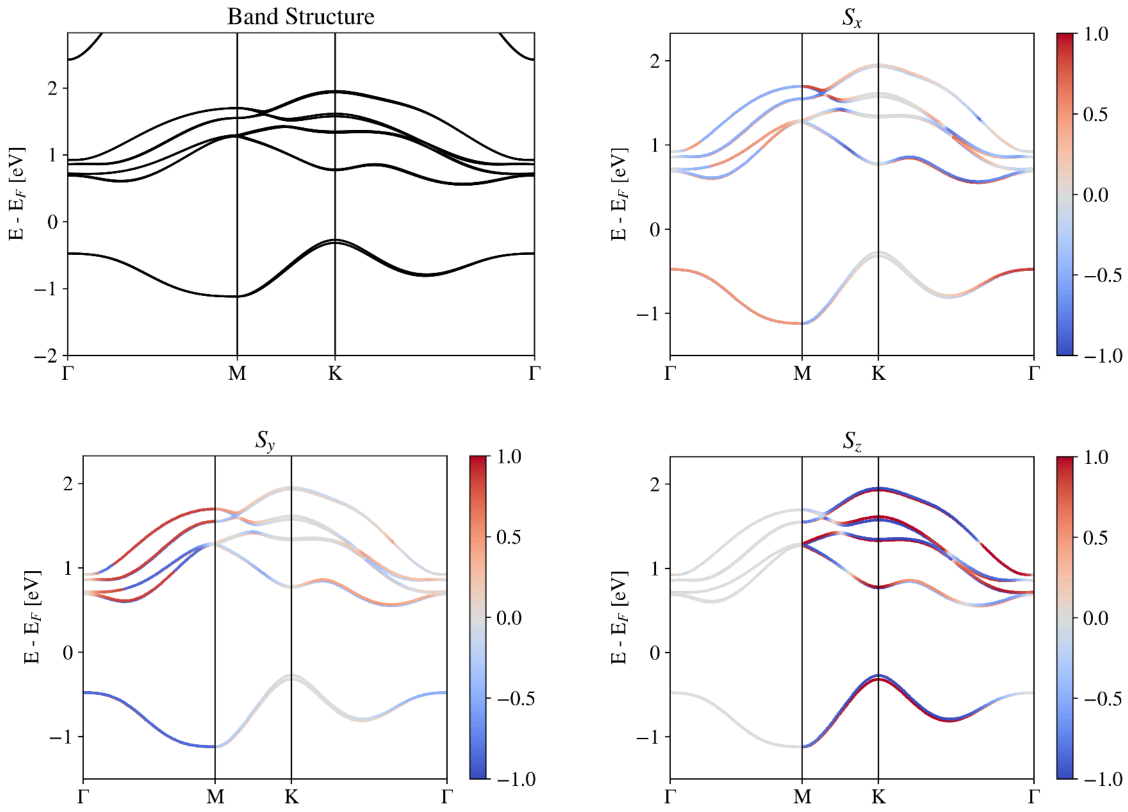


Figure 161: Band structure and spin polarization projections.

## 2.81 BrClZr-8cb69386d06b

- **Formula**: BrClZr
- **Structural Cluster**: ABC-4
- **Band gap (PBE)**: 0.912 eV
- **Energy above convex hull (C2DB)**: 0.01 eV
- **Space group symbol**: $P3m1$
- **Space group number**: 156
- **Polar structure**: True

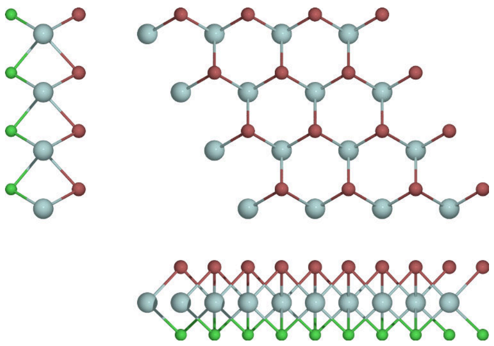

Figure 162: Structure representation

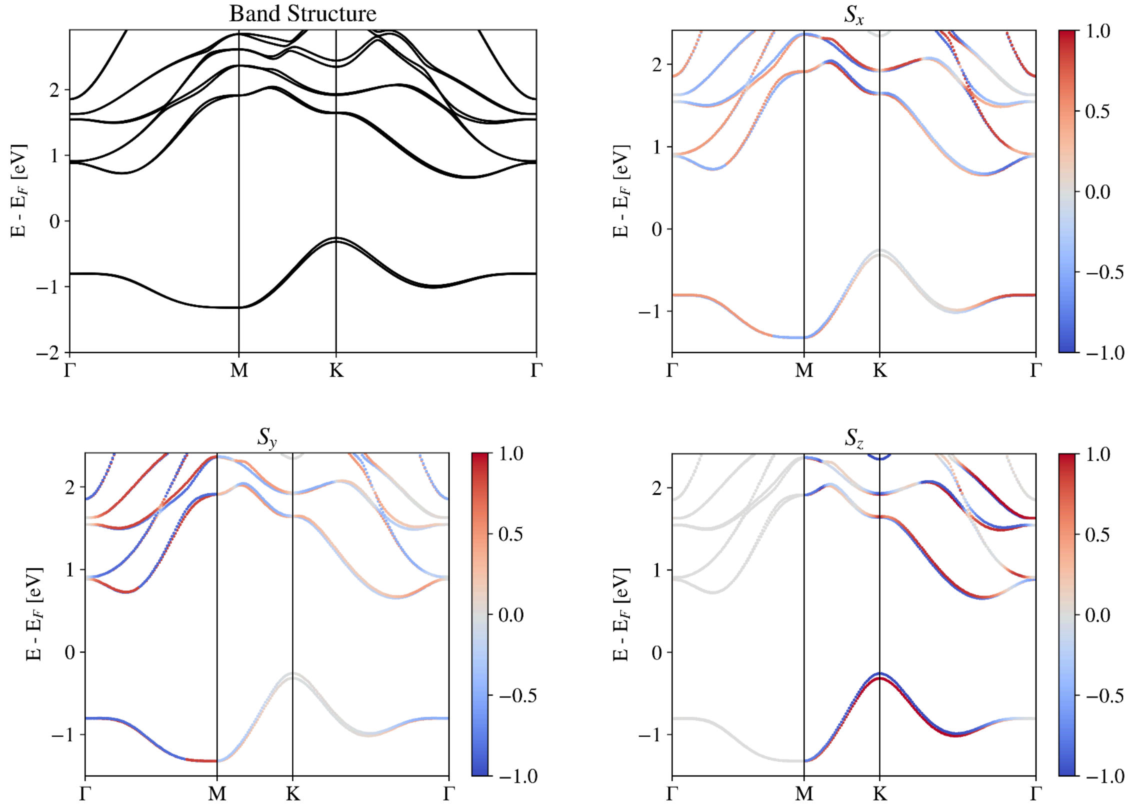


Figure 163: Band structure and spin polarization projections.

## 2.82 BrHfI-836a1091409d

- **Formula**: BrHfI
- **Structural Cluster**: ABC-4
- **Band gap (PBE)**: 0.695 eV
- **Energy above convex hull (C2DB)**: 0.087 eV
- **Space group symbol**: $P3m1$
- **Space group number**: 156
- **Polar structure**: True

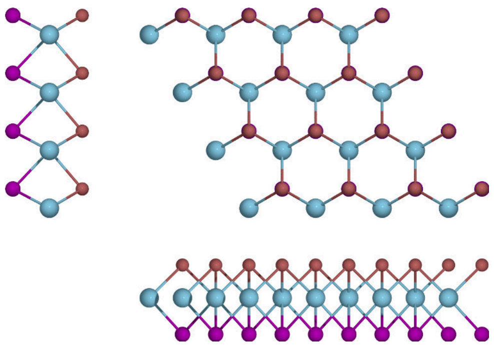

Figure 164: Structure representation

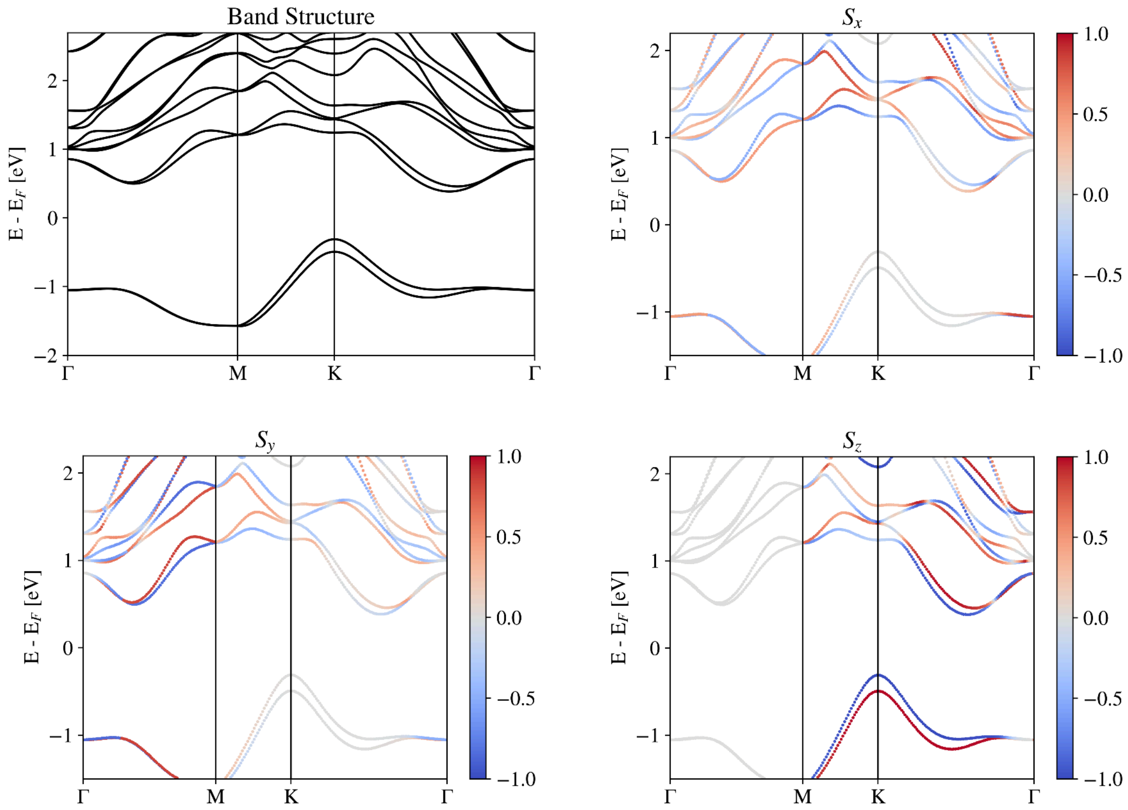


Figure 165: Band structure and spin polarization projections.

## 2.83 BrITi-233dbbf8f473

- **Formula**: BrITi
- **Structural Cluster**: ABC-4
- **Band gap (PBE)**: 0.68 eV
- **Energy above convex hull (C2DB)**: 0.057 eV
- **Space group symbol**: $P3m1$
- **Space group number**: 156
- **Polar structure**: True

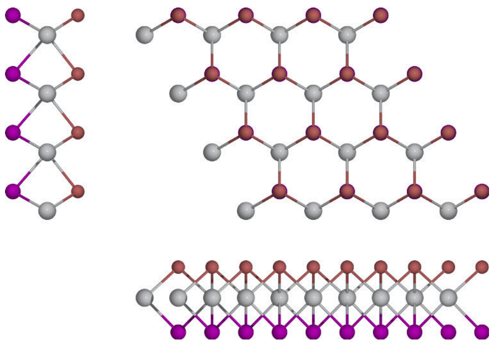

Figure 166: Structure representation

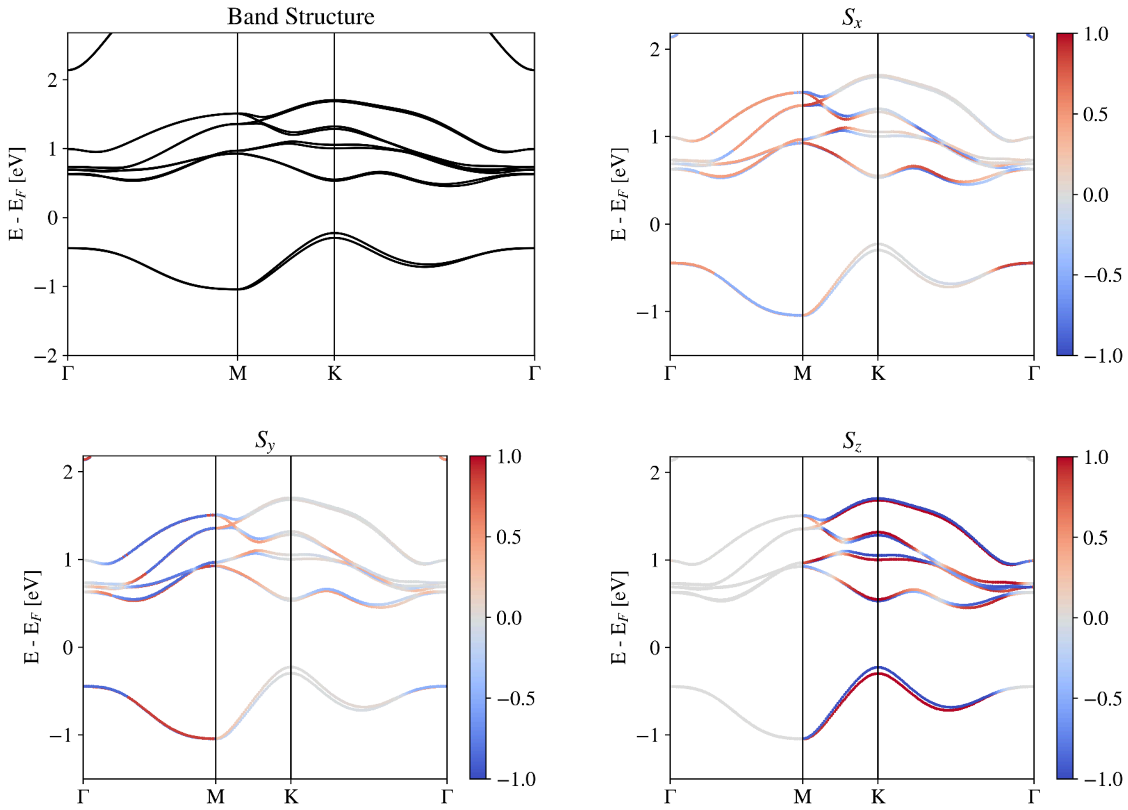


Figure 167: Band structure and spin polarization projections.

## 2.84 BrIZr-28c61999c692

- **Formula**: BrIZr
- **Structural Cluster**: ABC-4
- **Band gap (PBE)**: 0.782 eV
- **Energy above convex hull (C2DB)**: 0.038 eV
- **Space group symbol**: $P3m1$
- **Space group number**: 156
- **Polar structure**: True

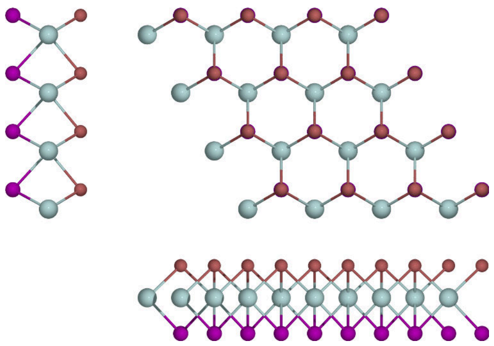

Figure 168: Structure representation

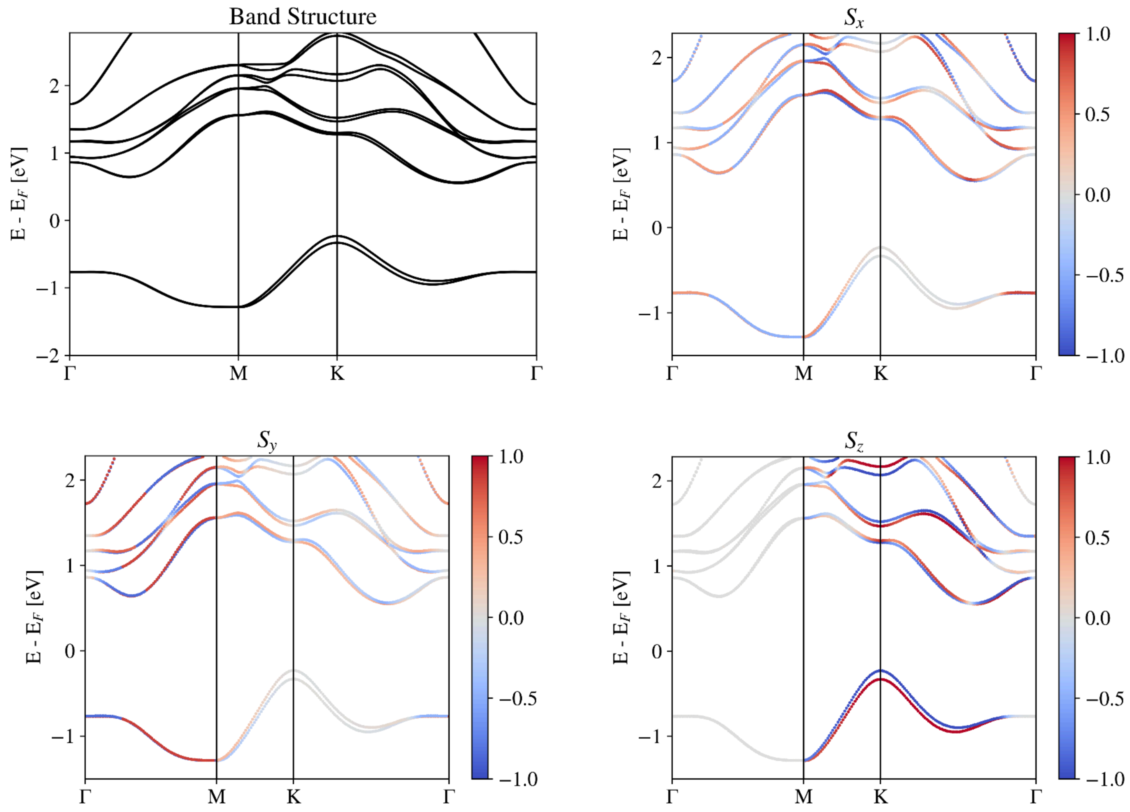


Figure 169: Band structure and spin polarization projections.

## 2.85 BrSSb-4ae37f15e1fe

- **Formula**: BrSSb
- **Structural Cluster**: ABC-4
- **Band gap (PBE)**: 1.437 eV
- **Energy above convex hull (C2DB)**: 0.157 eV
- **Space group symbol**: $P3m1$
- **Space group number**: 156
- **Polar structure**: True

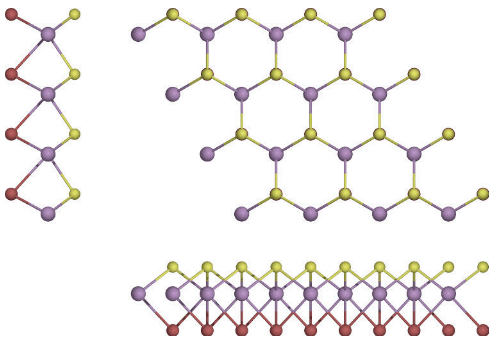

Figure 170: Structure representation

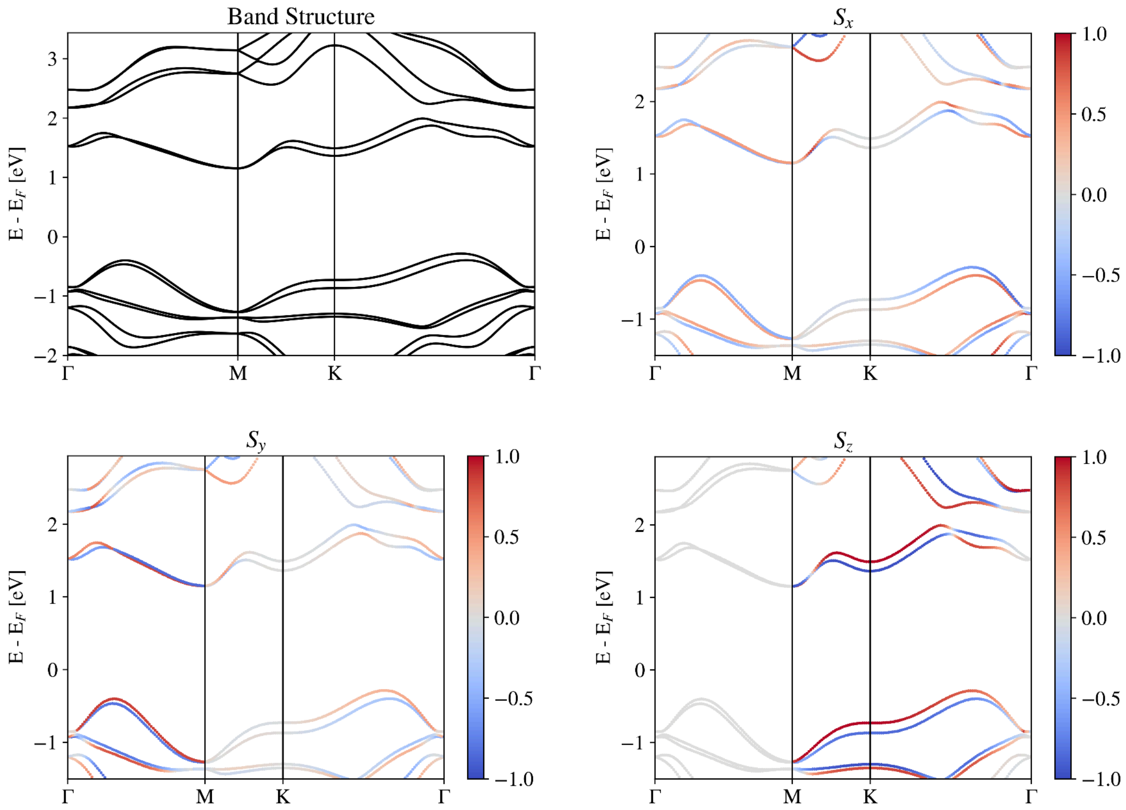


Figure 171: Band structure and spin polarization projections.

## 2.86 BrSSb-4da5c6be60db

- **Formula**: BrSSb
- **Structural Cluster**: ABC-3
- **Band gap (PBE)**: 1.233 eV
- **Energy above convex hull (C2DB)**: 0.028 eV
- **Space group symbol**: $P3m1$
- **Space group number**: 156
- **Polar structure**: True

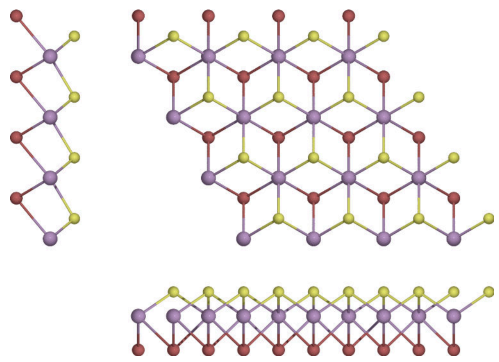

Figure 172: Structure representation

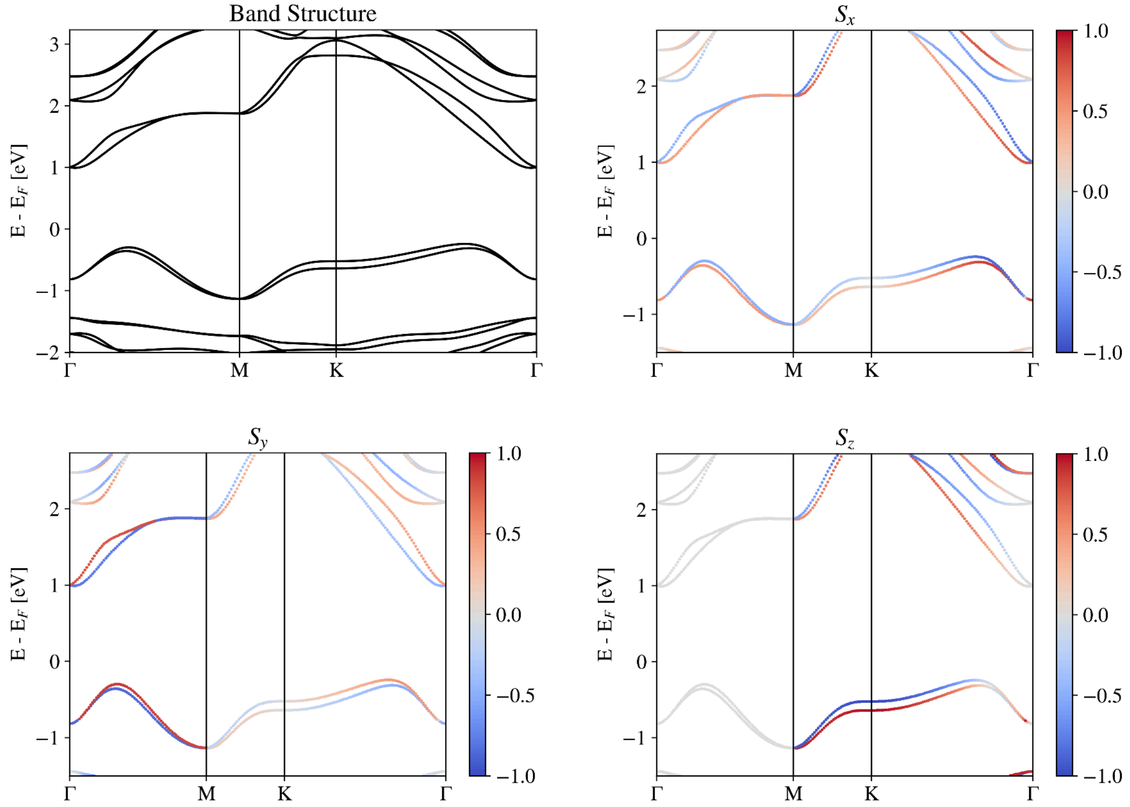


Figure 173: Band structure and spin polarization projections.

## 2.87 BrSbSe-89b15ddef41d

- **Formula**: BrSbSe
- **Structural Cluster**: ABC-3
- **Band gap (PBE)**: 1.072 eV
- **Energy above convex hull (C2DB)**: 0.0 eV
- **Space group symbol**: $P3m1$
- **Space group number**: 156
- **Polar structure**: True

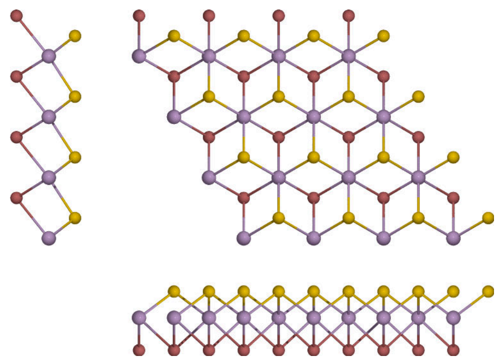

Figure 174: Structure representation

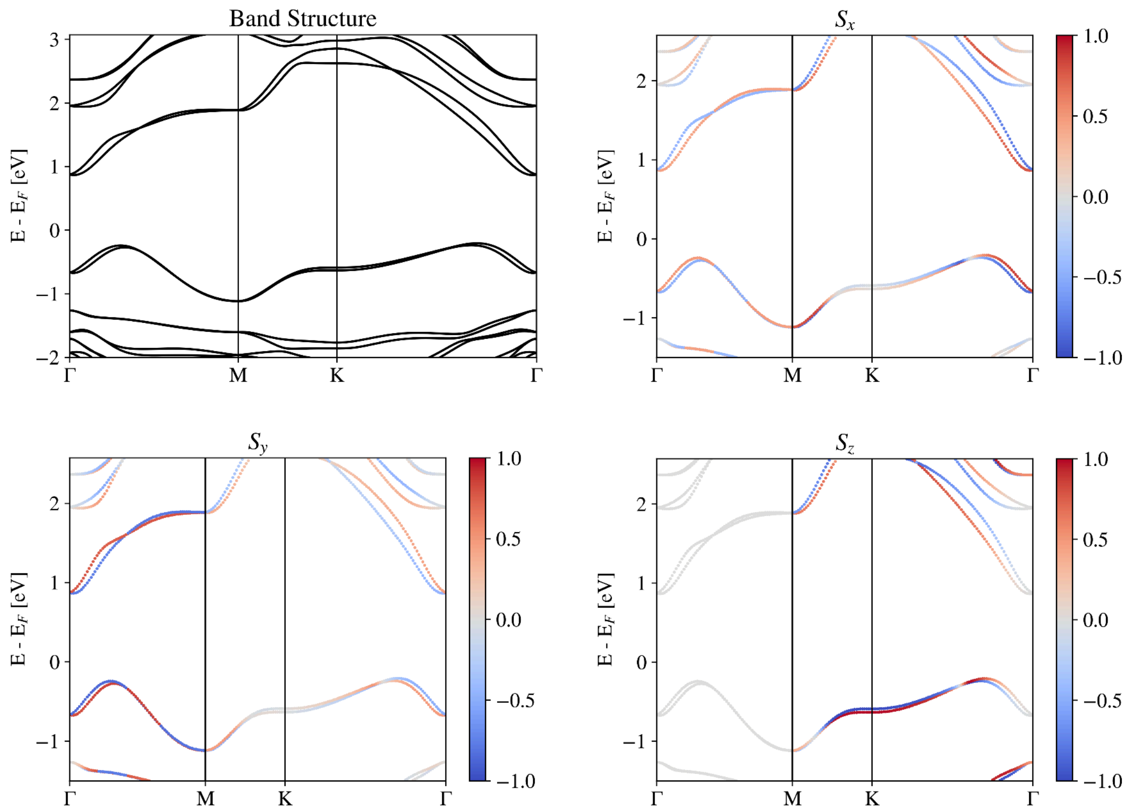


Figure 175: Band structure and spin polarization projections.

## 2.88 BrSbSe-c2a344b393f0

- **Formula**: BrSbSe
- **Structural Cluster**: ABC-4
- **Band gap (PBE)**: 1.467 eV
- **Energy above convex hull (C2DB)**: 0.124 eV
- **Space group symbol**: $P3m1$
- **Space group number**: 156
- **Polar structure**: True

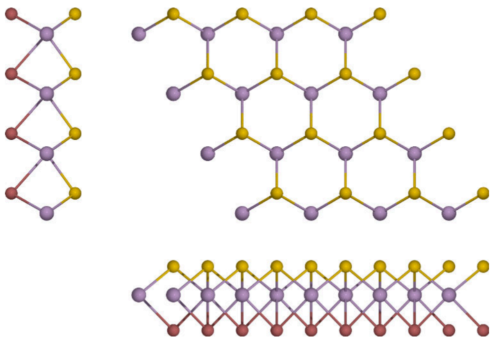

Figure 176: Structure representation

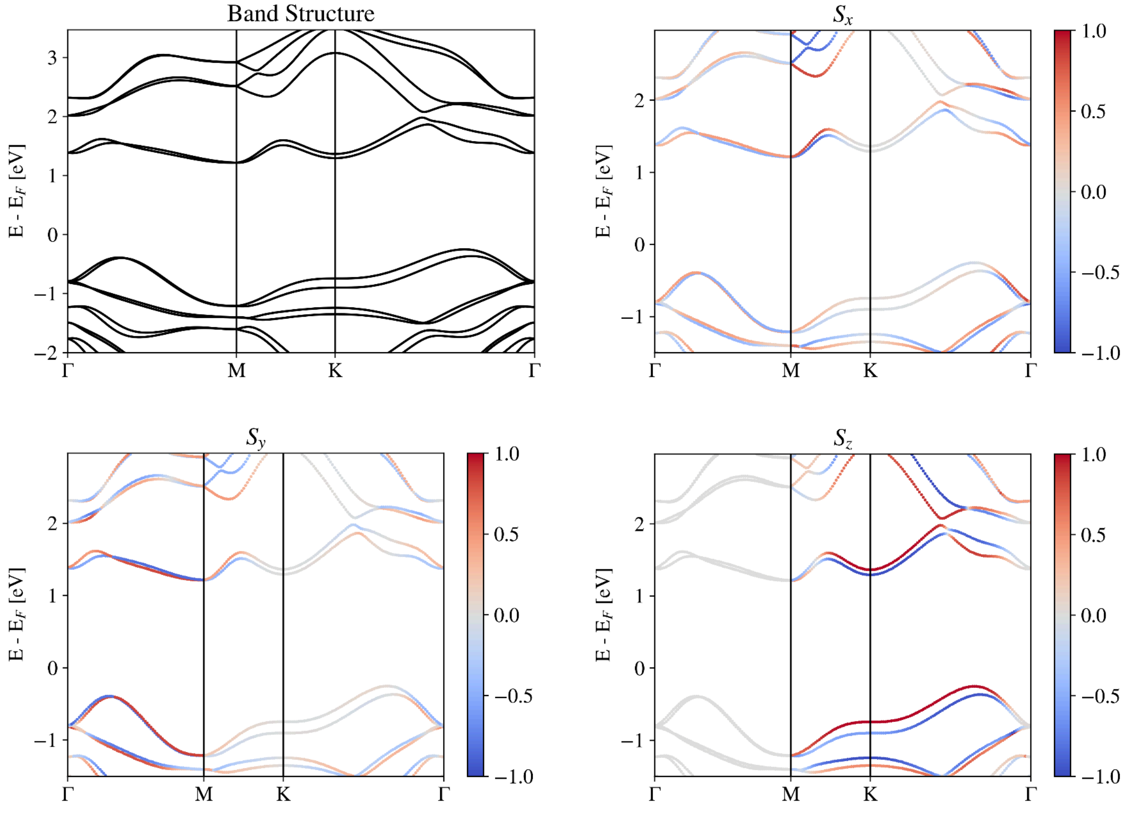


Figure 177: Band structure and spin polarization projections.

## 2.89 BrSbTe-18e62ba75259

- **Formula**: BrSbTe
- **Structural Cluster**: ABC-3
- **Band gap (PBE)**: 1.089 eV
- **Energy above convex hull (C2DB)**: 0.0 eV
- **Space group symbol**: $P3m1$
- **Space group number**: 156
- **Polar structure**: True

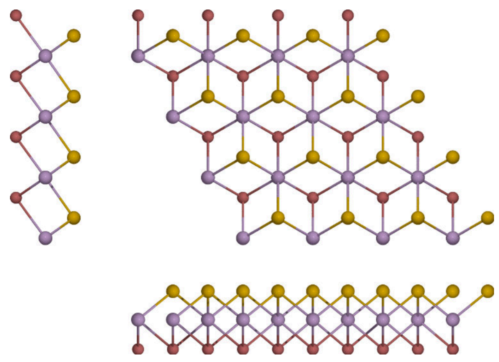

Figure 178: Structure representation

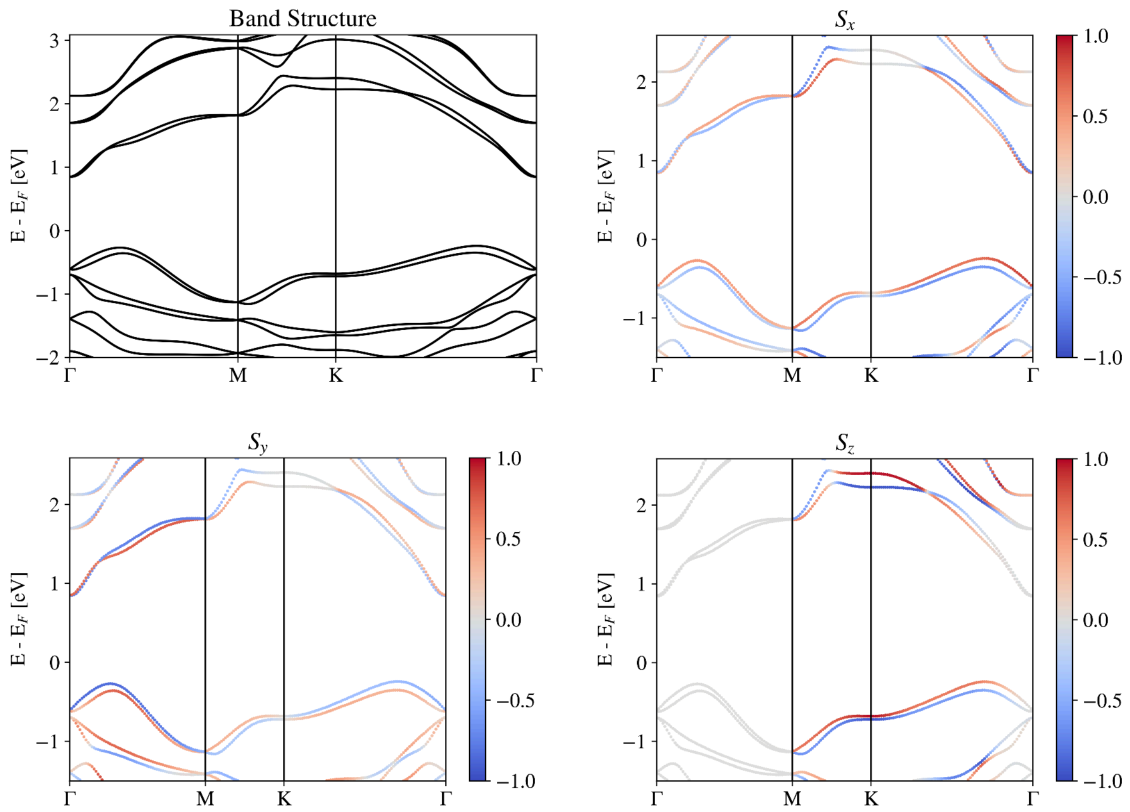


Figure 179: Band structure and spin polarization projections.

## 2.90 BrSbTe-f1e78a09001d

- **Formula**: BrSbTe
- **Structural Cluster**: ABC-4
- **Band gap (PBE)**: 1.331 eV
- **Energy above convex hull (C2DB)**: 0.13 eV
- **Space group symbol**: $P3m1$
- **Space group number**: 156
- **Polar structure**: True

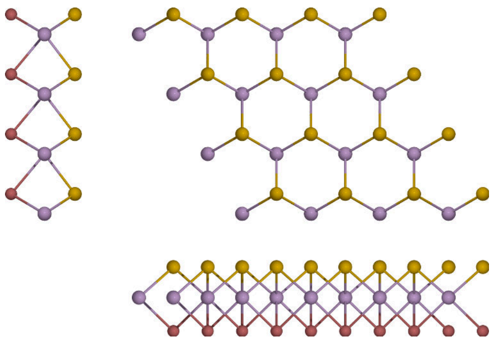

Figure 180: Structure representation

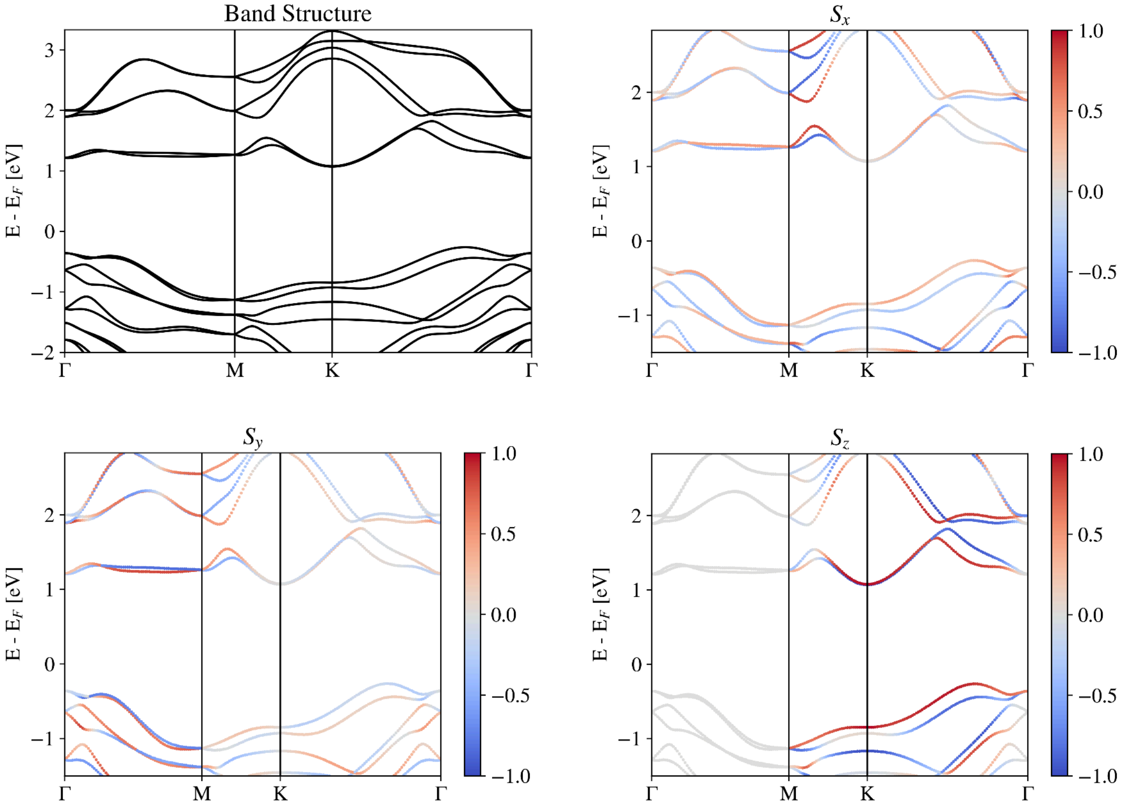


Figure 181: Band structure and spin polarization projections.

## 2.91 C2O2Hf3-082ae1b027e9

- **Formula**: C2O2Hf3
- **Structural Cluster**: A2B2C3-13
- **Band gap (PBE)**: 0.419 eV
- **Energy above convex hull (C2DB)**: 0.216 eV
- **Space group symbol**: $P\bar{6}m2$
- **Space group number**: 187
- **Polar structure**: False

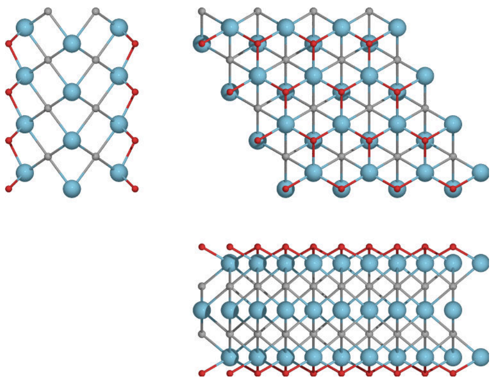

Figure 182: Structure representation

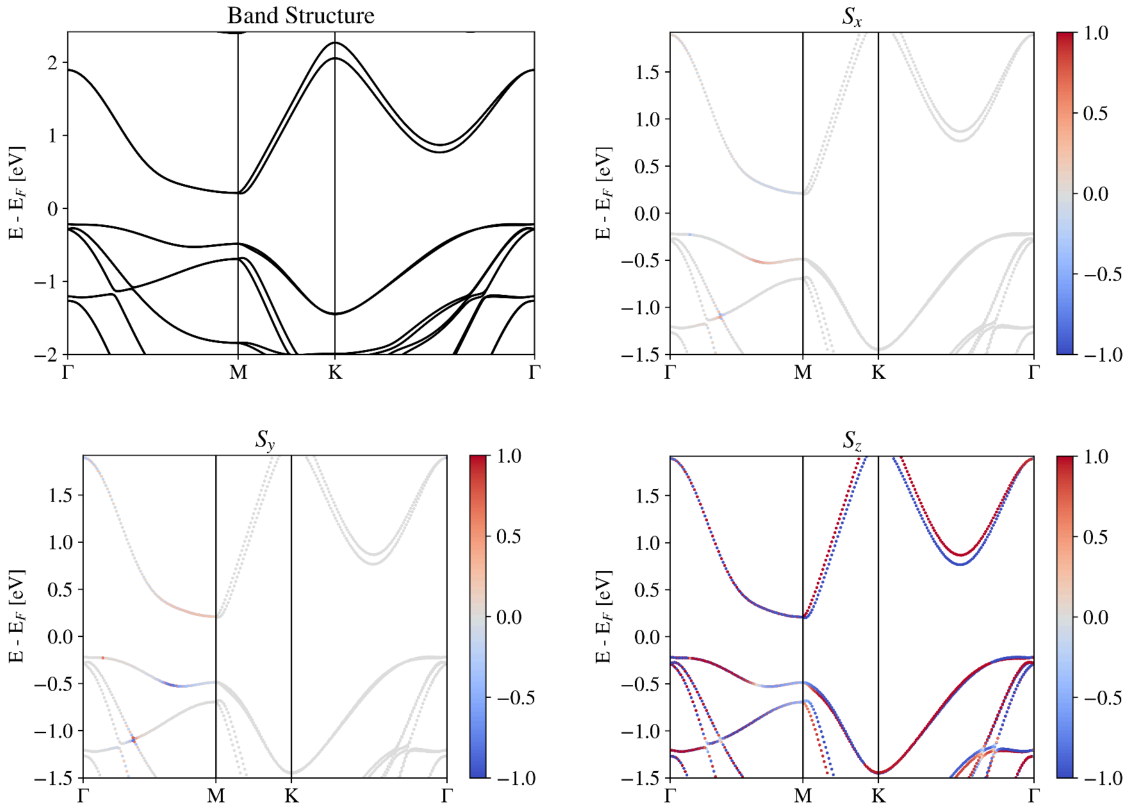


Figure 183: Band structure and spin polarization projections.

## 2.92 C2O2Zr3-23672dbca7d0

- **Formula**: C2O2Zr3
- **Structural Cluster**: A2B2C3-13
- **Band gap (PBE)**: 0.381 eV
- **Energy above convex hull (C2DB)**: 0.211 eV
- **Space group symbol**: $P\bar{6}m2$
- **Space group number**: 187
- **Polar structure**: False

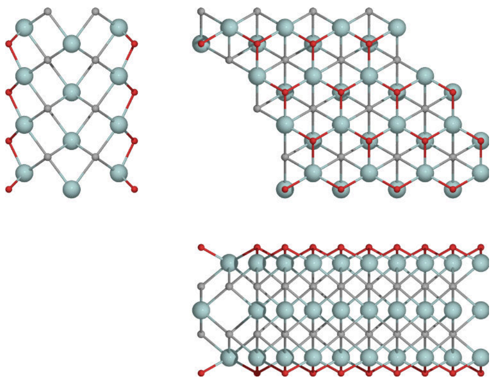

Figure 184: Structure representation

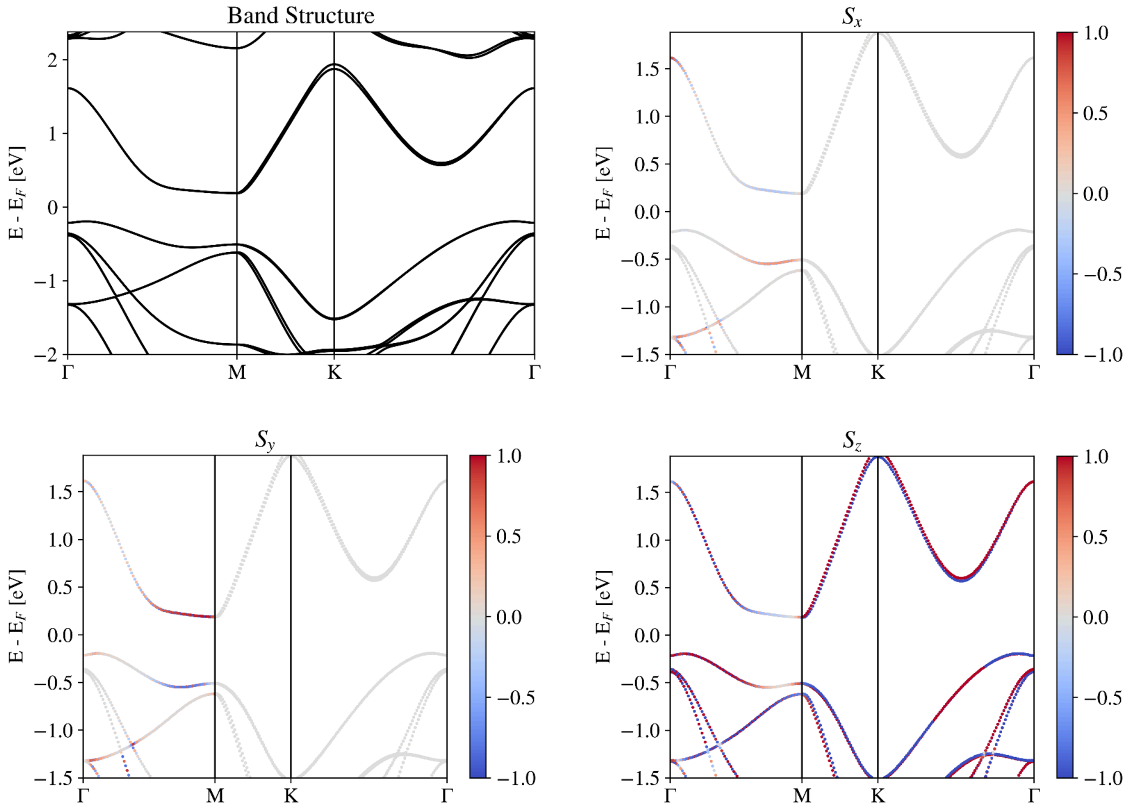


Figure 185: Band structure and spin polarization projections.

## 2.93 CSiF2-ee1174d1d821

- **Formula**: CSiF2
- **Structural Cluster**: ABC2-5
- **Band gap (PBE)**: 1.91 eV
- **Energy above convex hull (C2DB)**: 0.6 eV
- **Space group symbol**: $P3m1$
- **Space group number**: 156
- **Polar structure**: True

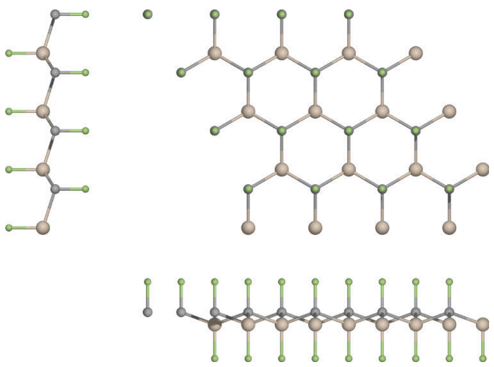

Figure 186: Structure representation

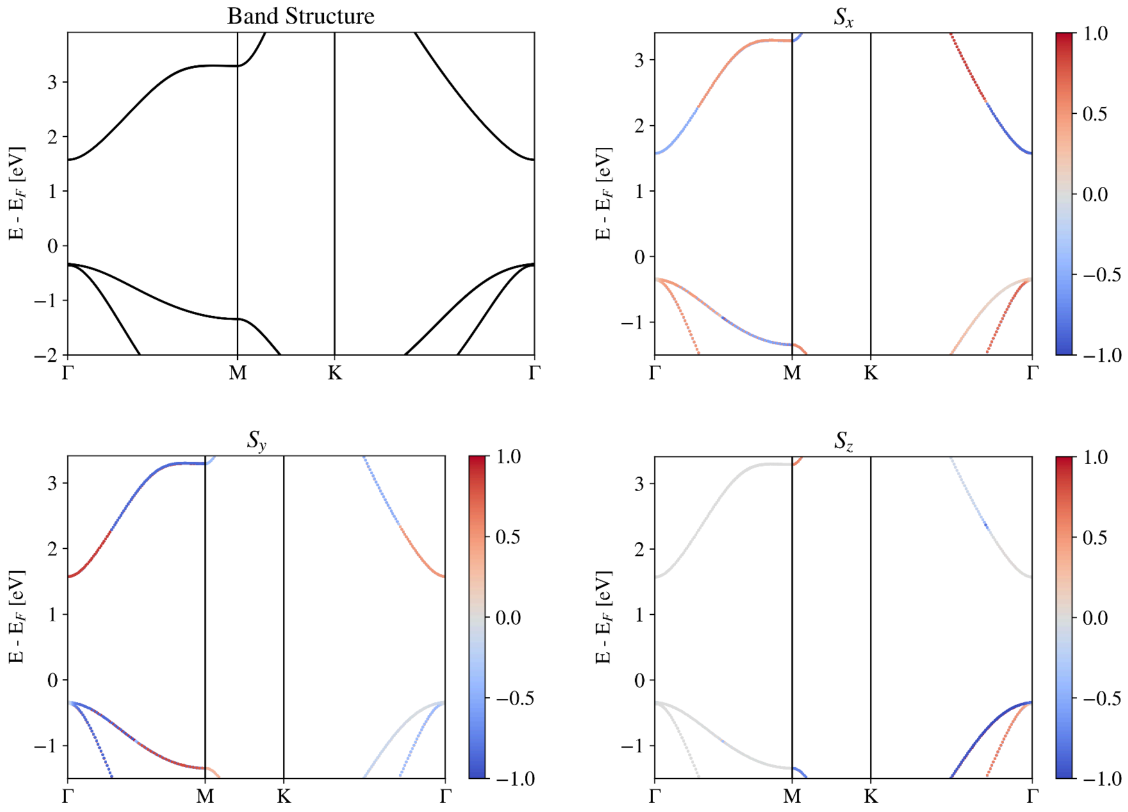


Figure 187: Band structure and spin polarization projections.

## 2.94 CSiH2-8a1587098151

- **Formula**: CSiH2
- **Structural Cluster**: ABC2-5
- **Band gap (PBE)**: 4.008 eV
- **Energy above convex hull (C2DB)**: 0.0 eV
- **Space group symbol**: $P3m1$
- **Space group number**: 156
- **Polar structure**: True

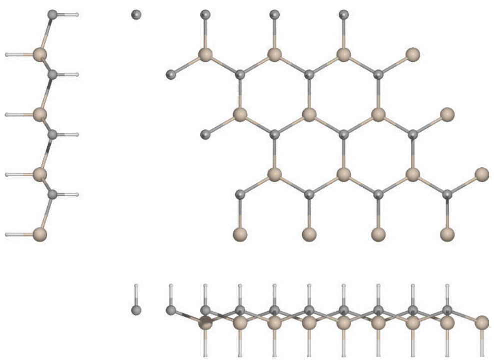

Figure 188: Structure representation

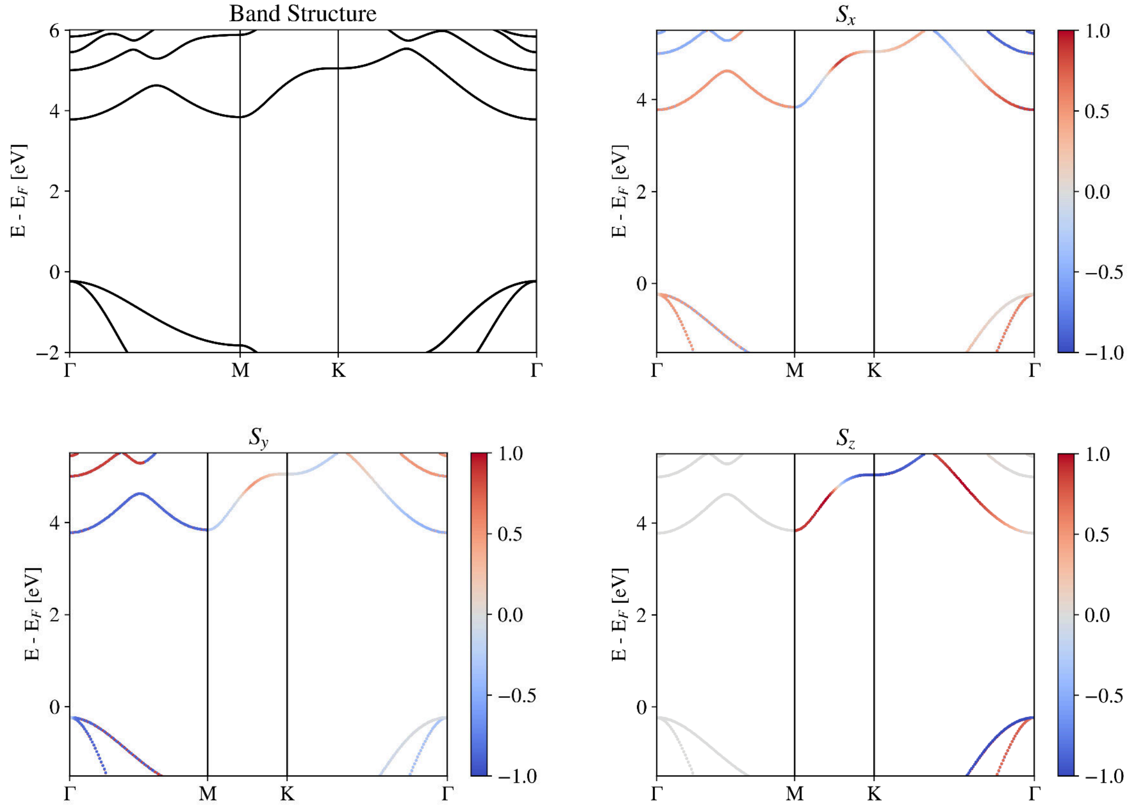


Figure 189: Band structure and spin polarization projections.

## 2.95 CaBr2-49f279264c91

- **Formula**: CaBr2
- **Structural Cluster**: AB2-11
- **Band gap (PBE)**: 4.754 eV
- **Energy above convex hull (C2DB)**: 0.179 eV
- **Space group symbol**: $P\bar{4}m2$
- **Space group number**: 115
- **Polar structure**: False

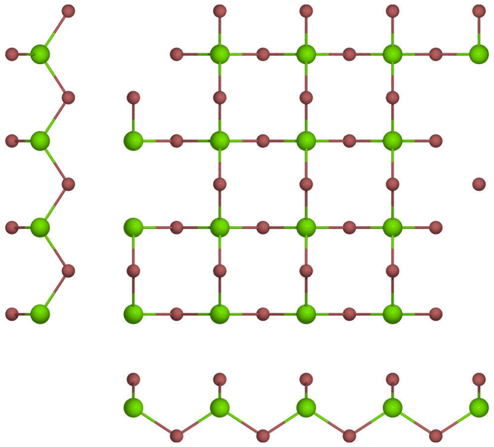

Figure 190: Structure representation

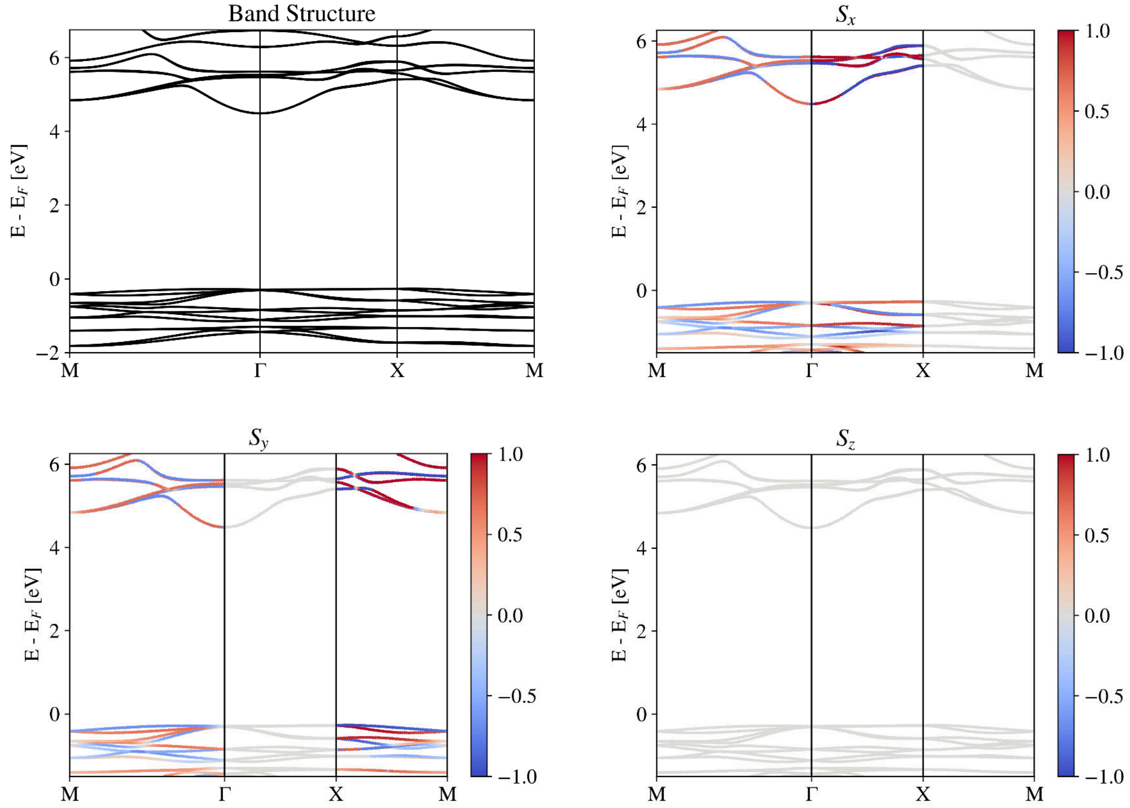


Figure 191: Band structure and spin polarization projections.

## 2.96 CaBr2-fbb623b6f288

- **Formula**: CaBr2
- **Structural Cluster**: AB2-4
- **Band gap (PBE)**: 4.141 eV
- **Energy above convex hull (C2DB)**: 0.129 eV
- **Space group symbol**: $P\bar{6}m2$
- **Space group number**: 187
- **Polar structure**: False

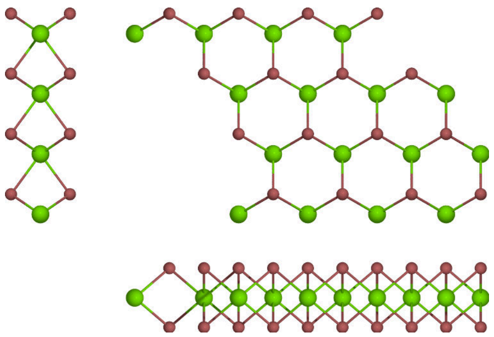

Figure 192: Structure representation

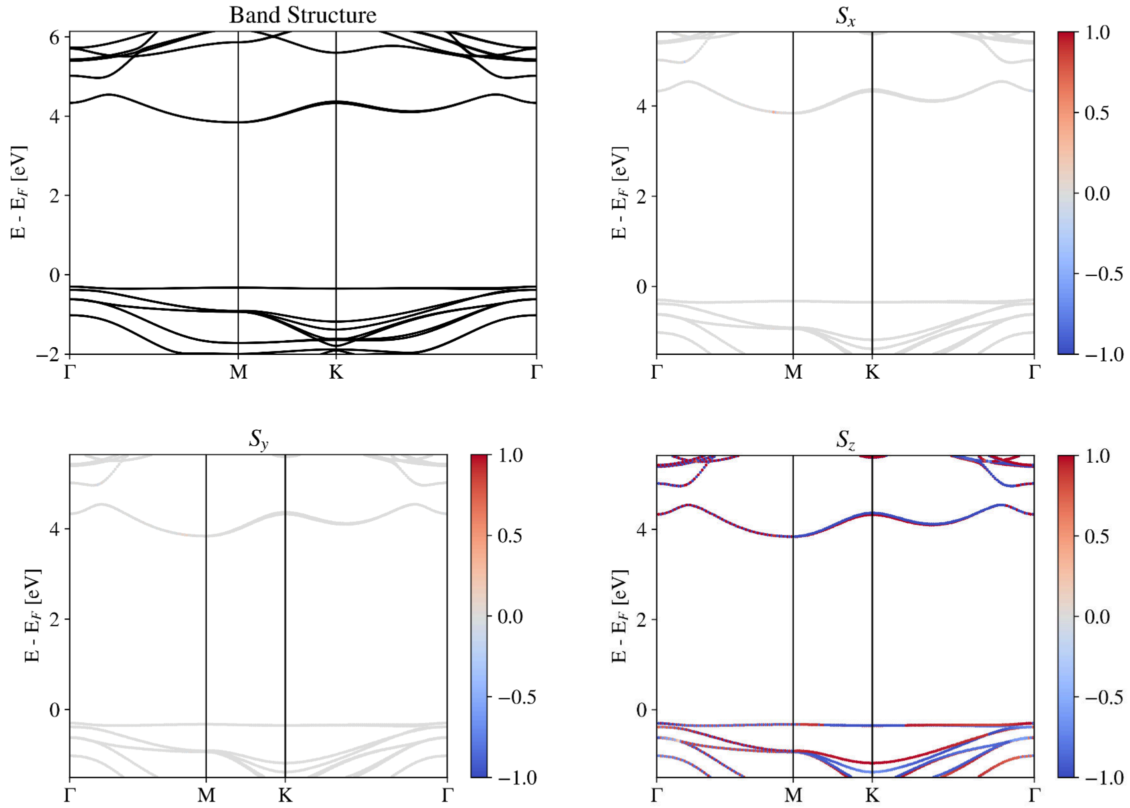


Figure 193: Band structure and spin polarization projections.

## 2.97 CaCl2-3ca106221b9b

- **Formula**: CaCl2
- **Structural Cluster**: AB2-4
- **Band gap (PBE)**: 4.784 eV
- **Energy above convex hull (C2DB)**: 0.164 eV
- **Space group symbol**: $P\bar{6}m2$
- **Space group number**: 187
- **Polar structure**: False

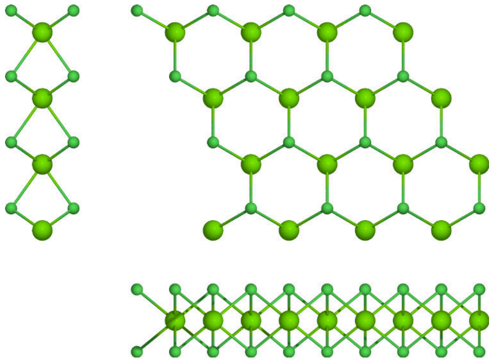

Figure 194: Structure representation

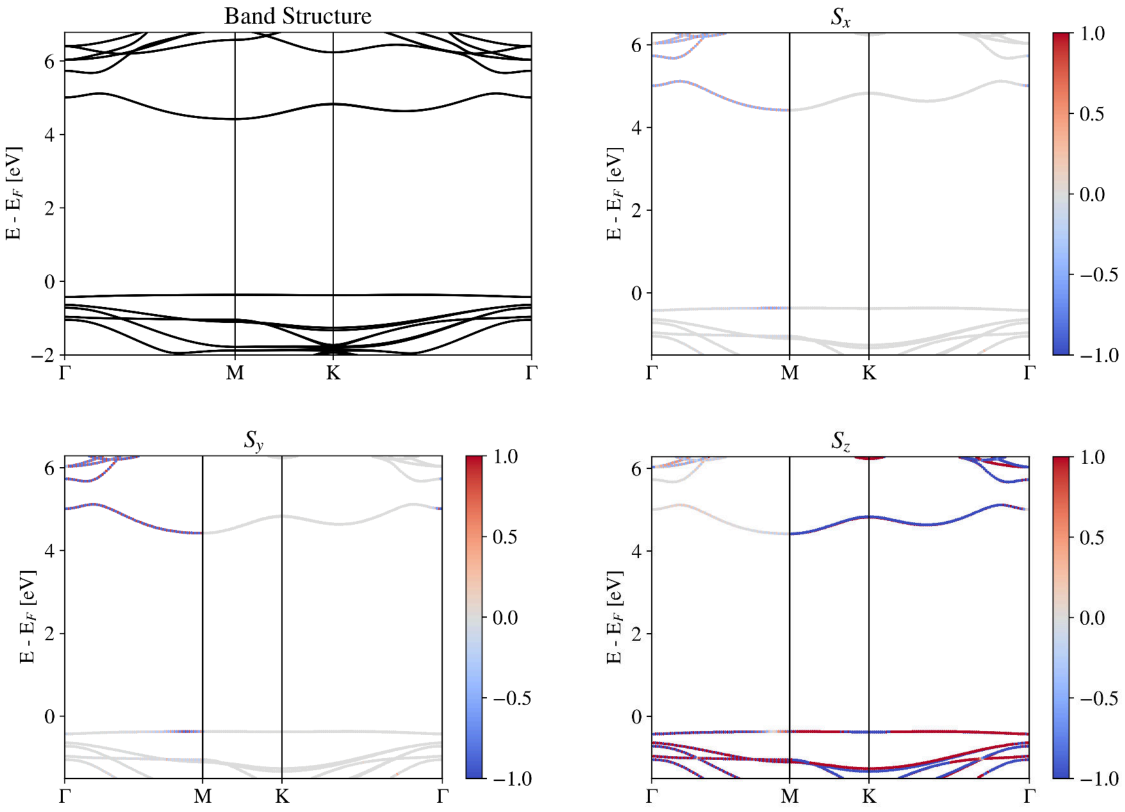


Figure 195: Band structure and spin polarization projections.

### 2.98 CaCl2-55e6ef39650c

- **Formula**: CaCl2
- **Structural Cluster**: AB2-11
- **Band gap (PBE)**: 5.539 eV
- **Energy above convex hull (C2DB)**: 0.192 eV
- **Space group symbol**: $P\bar{4}m2$
- **Space group number**: 115
- **Polar structure**: False

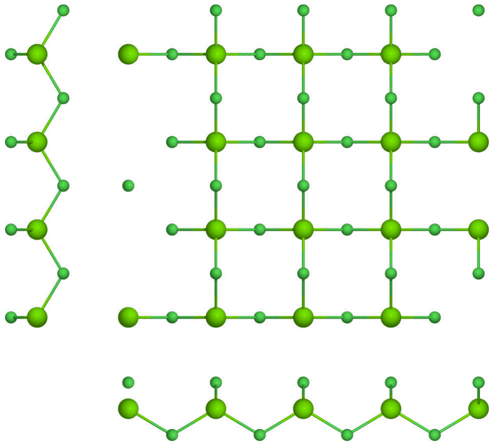

Figure 196: Structure representation

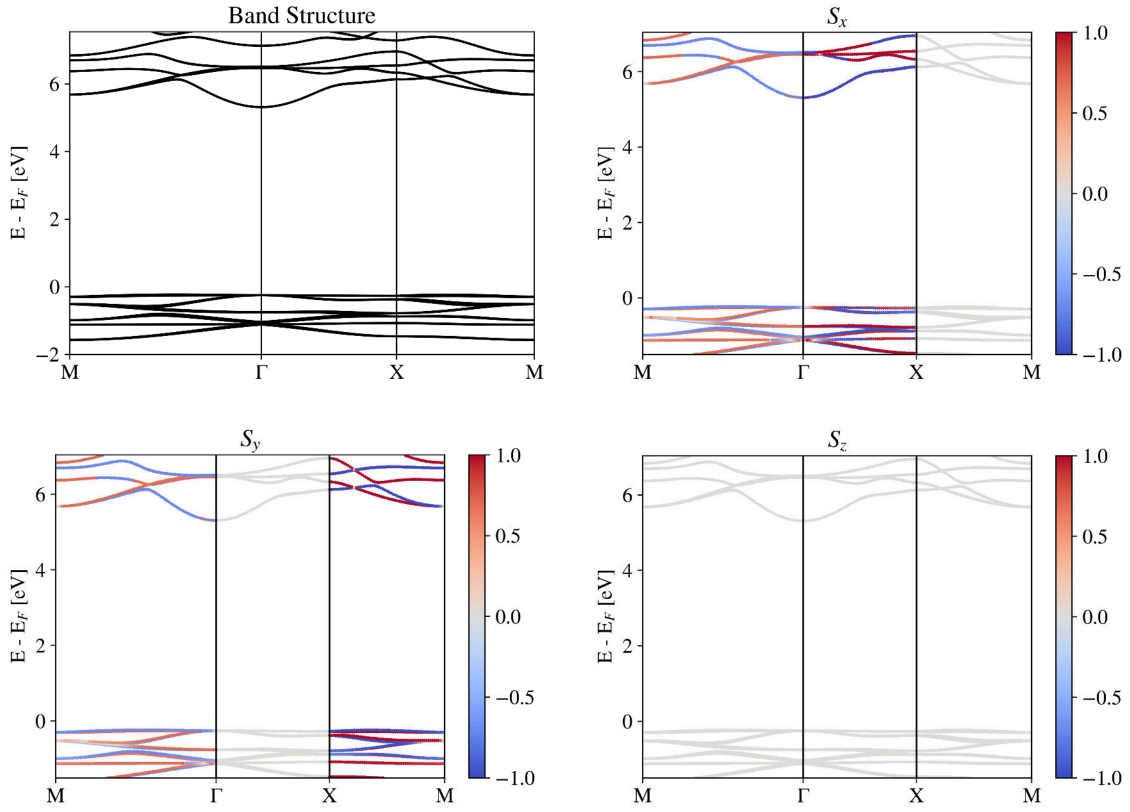


Figure 197: Band structure and spin polarization projections.

## 2.99 CaF2-9632fda9fd46

- **Formula**: CaF2
- **Structural Cluster**: AB2-11
- **Band gap (PBE)**: 6.453 eV
- **Energy above convex hull (C2DB)**: 0.326 eV
- **Space group symbol**: $P\bar{4}m2$
- **Space group number**: 115
- **Polar structure**: False

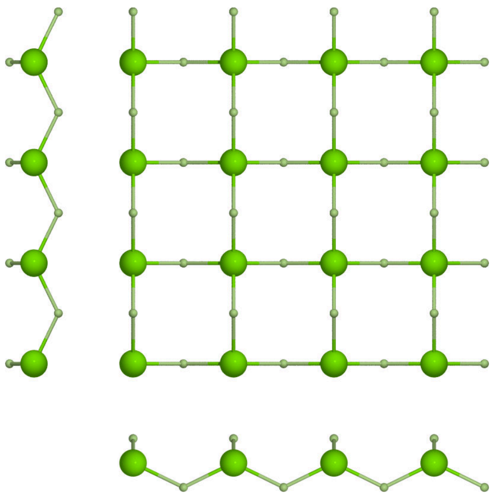

Figure 198: Structure representation

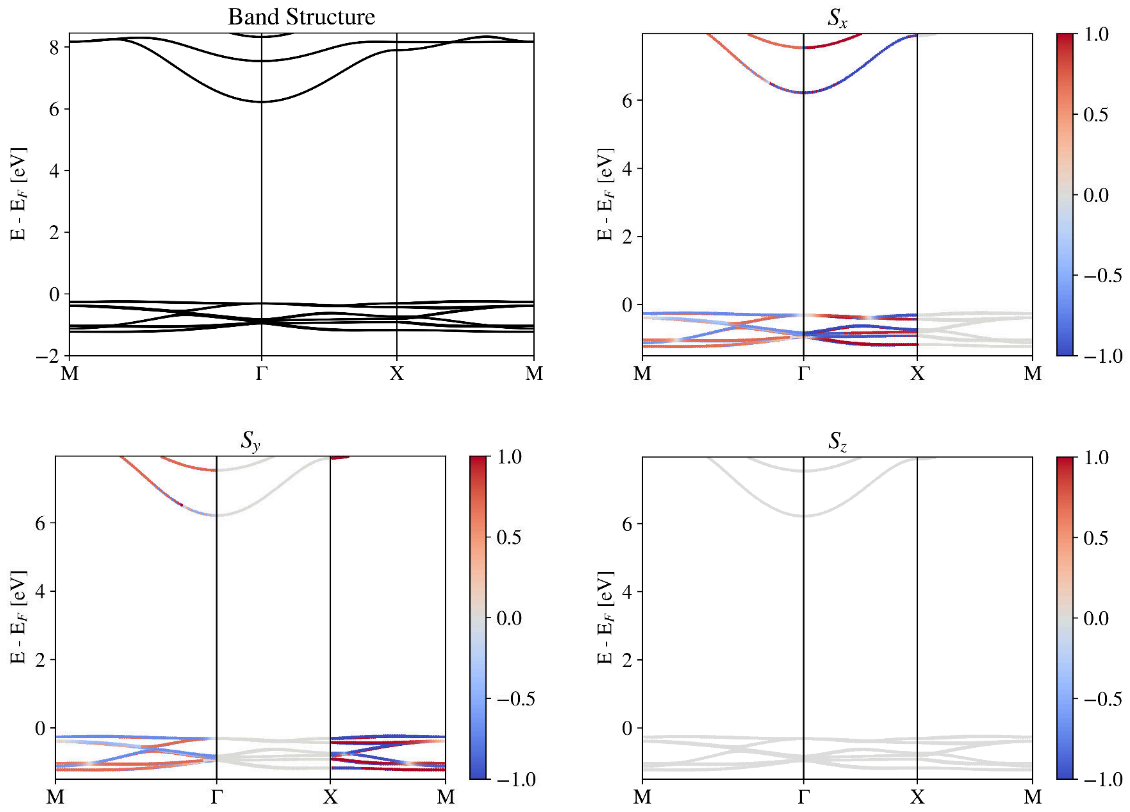


Figure 199: Band structure and spin polarization projections.

## 2.100 CaI2-066f40f26c53

- **Formula**: CaI2
- **Structural Cluster**: AB2-4
- **Band gap (PBE)**: 2.995 eV
- **Energy above convex hull (C2DB)**: 0.108 eV
- **Space group symbol**: $P\bar{6}m2$
- **Space group number**: 187
- **Polar structure**: False

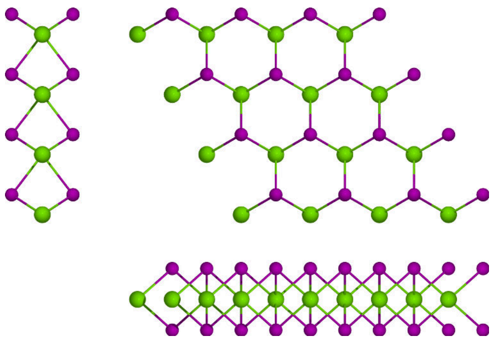

Figure 200: Structure representation

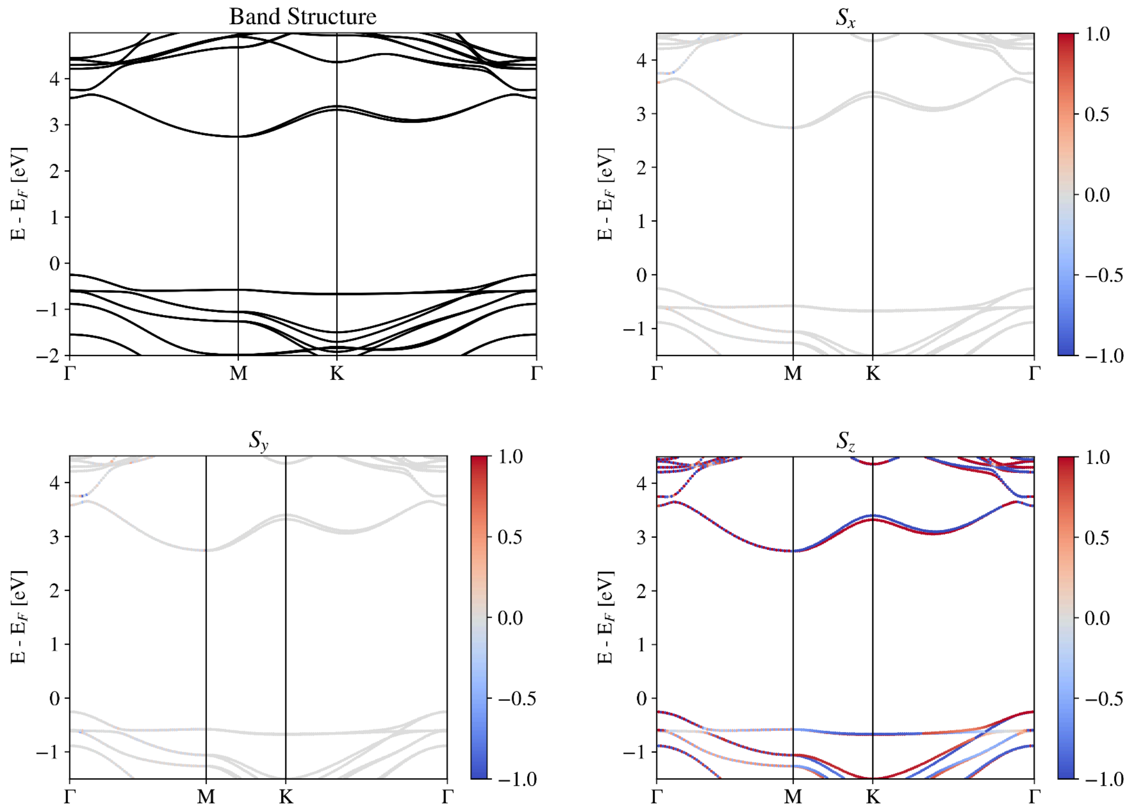


Figure 201: Band structure and spin polarization projections.

## 2.101 CaI2-793f311ee701

- **Formula**: CaI2
- **Structural Cluster**: AB2-11
- **Band gap (PBE)**: 4.022 eV
- **Energy above convex hull (C2DB)**: 0.174 eV
- **Space group symbol**: $P\bar{4}m2$
- **Space group number**: 115
- **Polar structure**: False

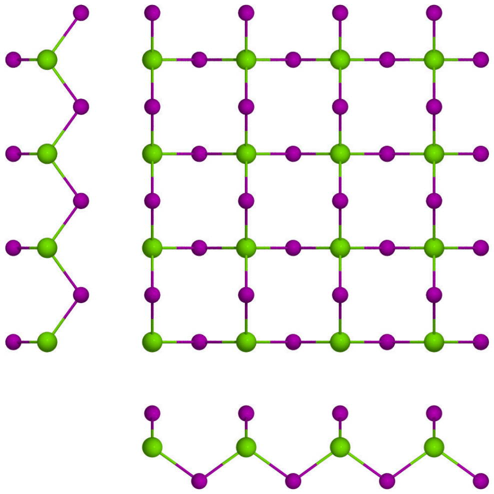

Figure 202: Structure representation

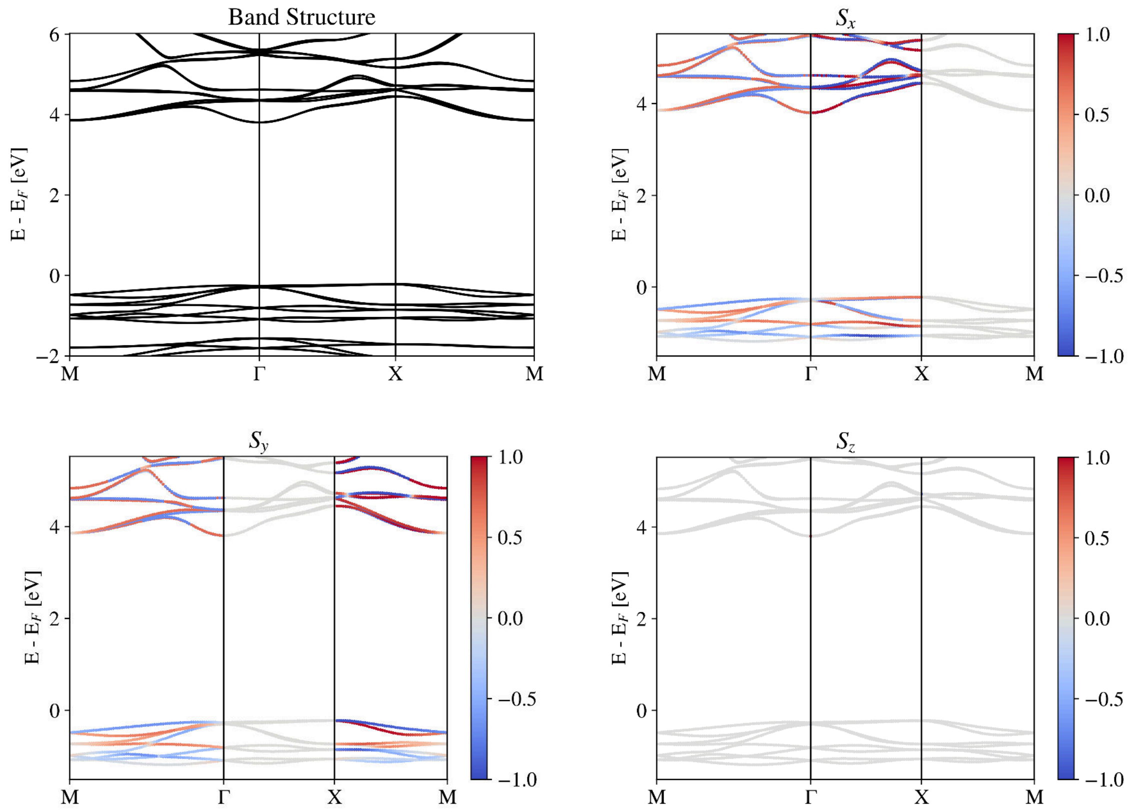


Figure 203: Band structure and spin polarization projections.

## 2.102 Cd2P2S6-ebcb2404504e

- **Formula**: Cd2P2S6
- **Structural Cluster**: ABC3-16
- **Band gap (PBE)**: 1.915 eV
- **Energy above convex hull (C2DB)**: 0.0 eV
- **Space group symbol**: $P1$
- **Space group number**: 1
- **Polar structure**: True

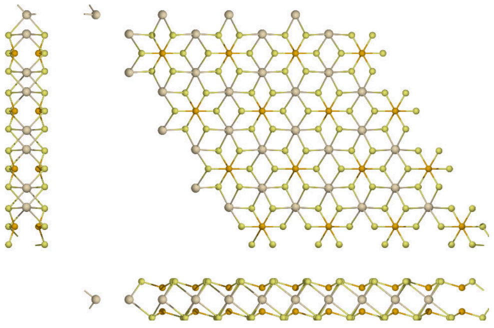

Figure 204: Structure representation

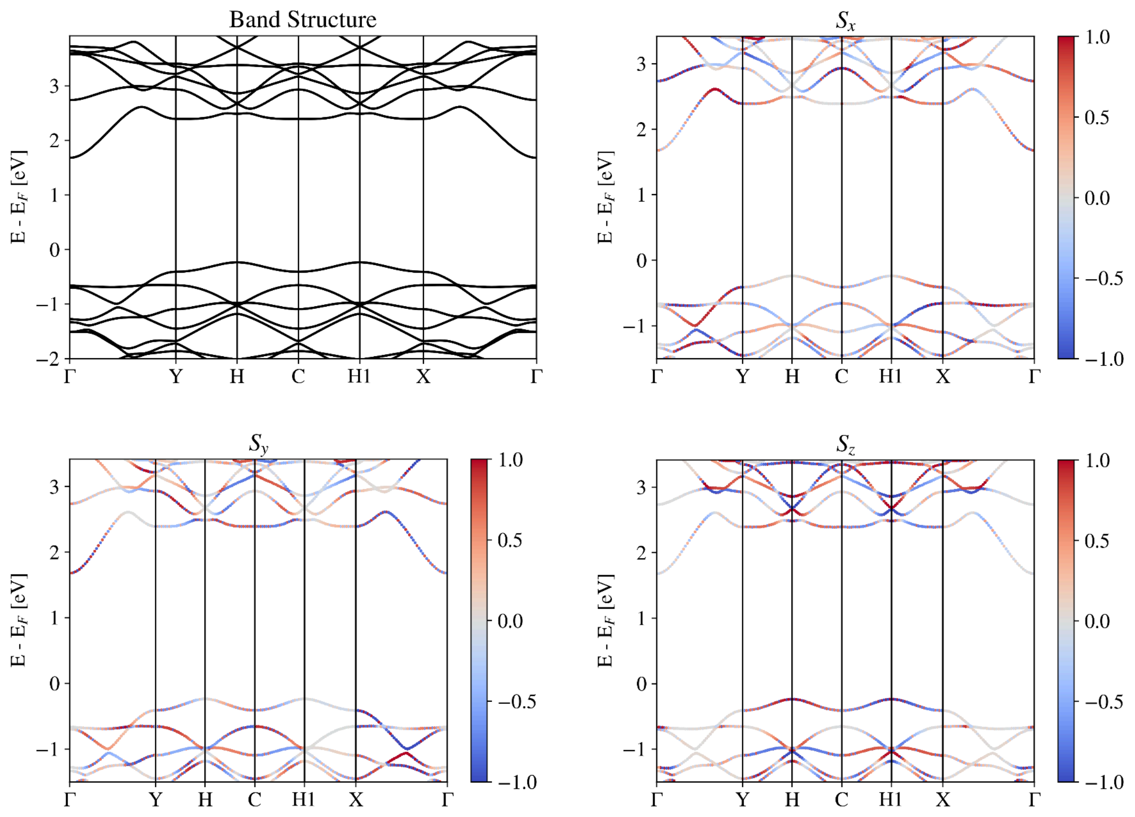


Figure 205: Band structure and spin polarization projections.

## 2.103 Cd2P2Se6-0460cce85156

- **Formula**: Cd2P2Se6
- **Structural Cluster**: ABC3-16
- **Band gap (PBE)**: 1.272 eV
- **Energy above convex hull (C2DB)**: 0.0 eV
- **Space group symbol**: $P1$
- **Space group number**: 1
- **Polar structure**: True

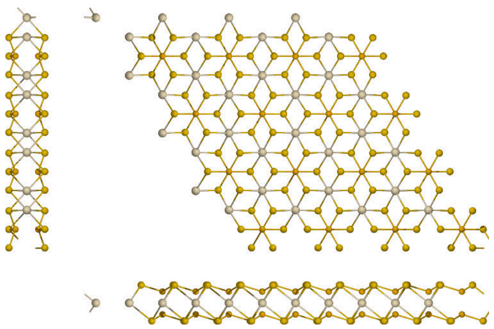

Figure 206: Structure representation

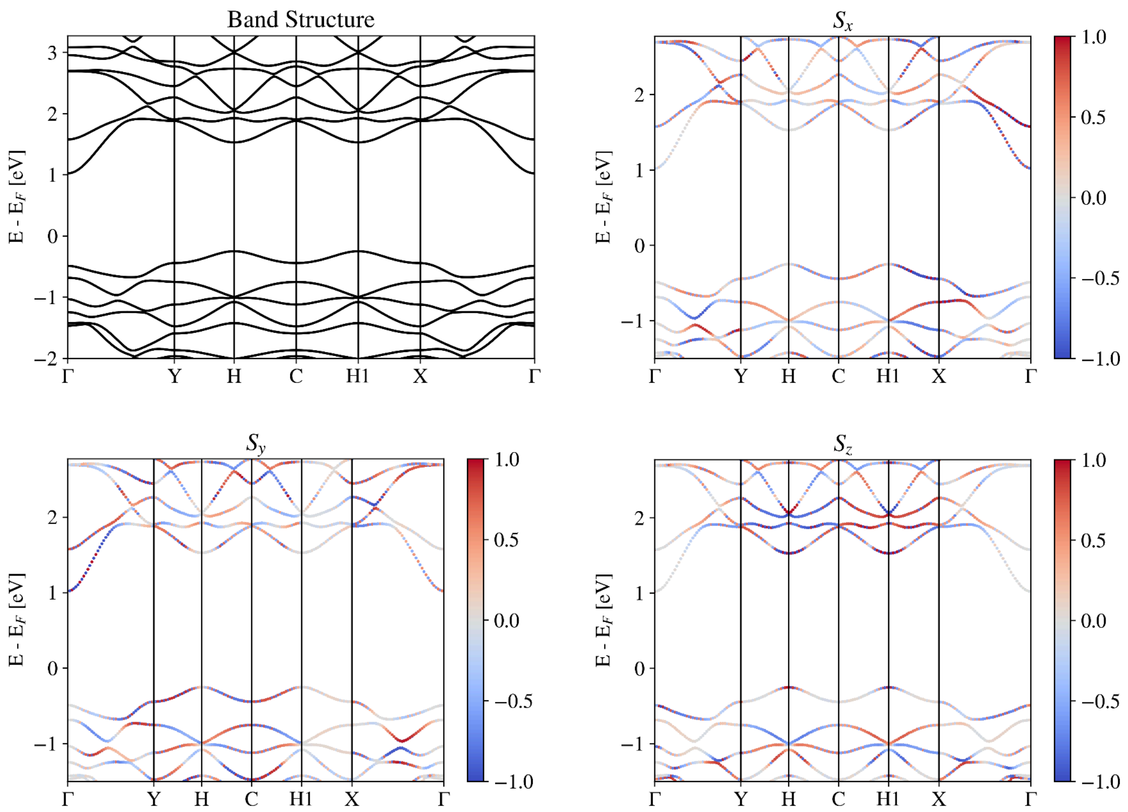


Figure 207: Band structure and spin polarization projections.

## 2.104 Cd2Se2-3394b92523be

- **Formula**: Cd2Se2
- **Structural Cluster**: AB-5
- **Band gap (PBE)**: 0.981 eV
- **Energy above convex hull (C2DB)**: 0.252 eV
- **Space group symbol**: $P1$
- **Space group number**: 1
- **Polar structure**: True

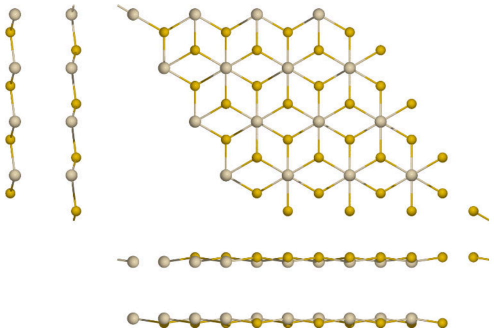

Figure 208: Structure representation

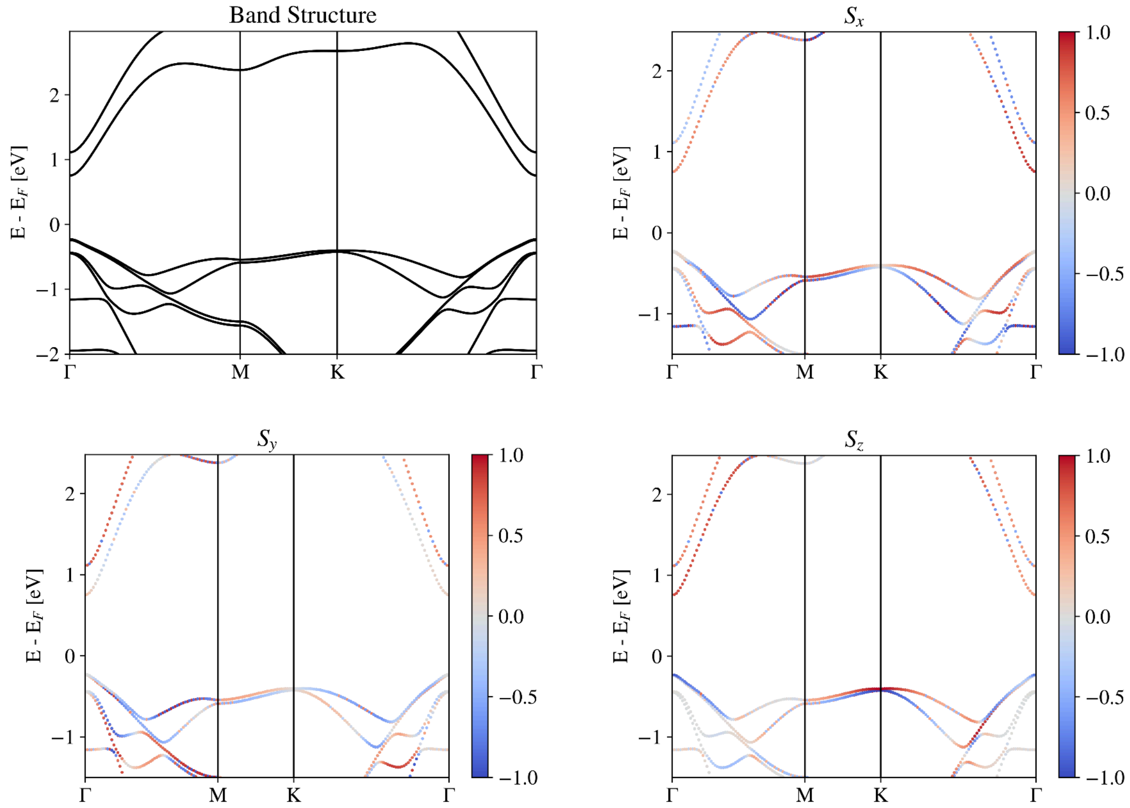


Figure 209: Band structure and spin polarization projections.

## 2.105 Cd2Se4-f25f251c7be8

- **Formula**: Cd2Se4
- **Structural Cluster**: AB2-18
- **Band gap (PBE)**: 1.362 eV
- **Energy above convex hull (C2DB)**: 0.24 eV
- **Space group symbol**: $P1$
- **Space group number**: 1
- **Polar structure**: True

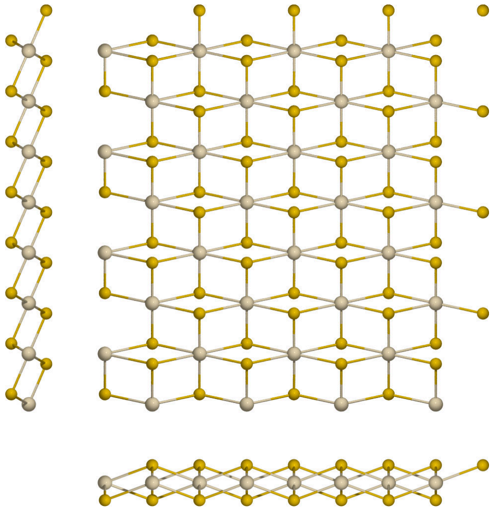

Figure 210: Structure representation

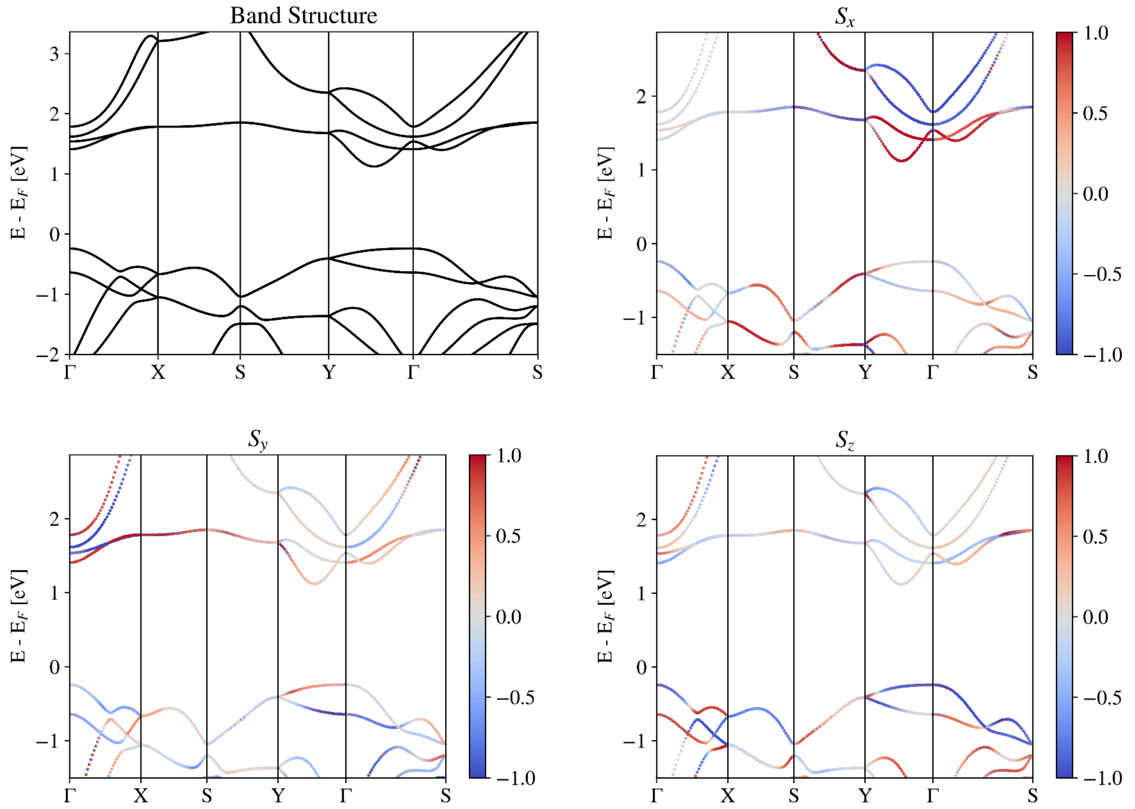


Figure 211: Band structure and spin polarization projections.

## 2.106 Cd2Te2-ed3311682142

- **Formula**: Cd2Te2
- **Structural Cluster**: AB-5
- **Band gap (PBE)**: 0.879 eV
- **Energy above convex hull (C2DB)**: 0.242 eV
- **Space group symbol**: $P1$
- **Space group number**: 1
- **Polar structure**: True

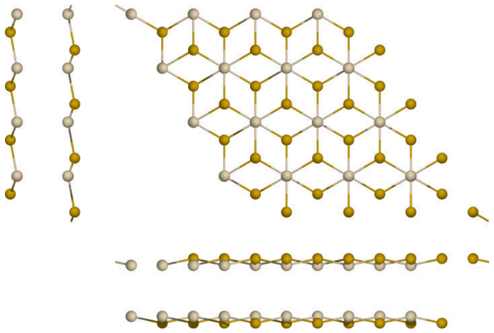

Figure 212: Structure representation

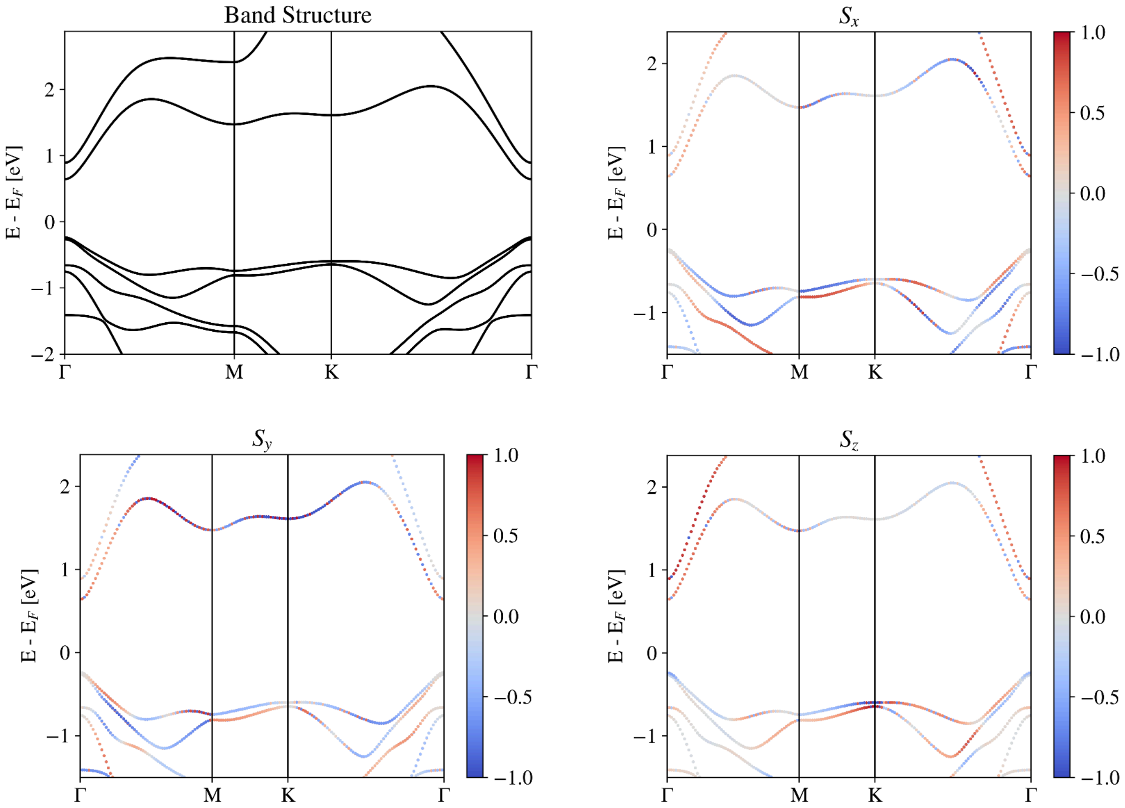


Figure 213: Band structure and spin polarization projections.

## 2.107 CdBr2-a7bb757c6234

- **Formula**: CdBr2
- **Structural Cluster**: AB2-4
- **Band gap (PBE)**: 2.377 eV
- **Energy above convex hull (C2DB)**: 0.121 eV
- **Space group symbol**: $P\bar{6}m2$
- **Space group number**: 187
- **Polar structure**: False

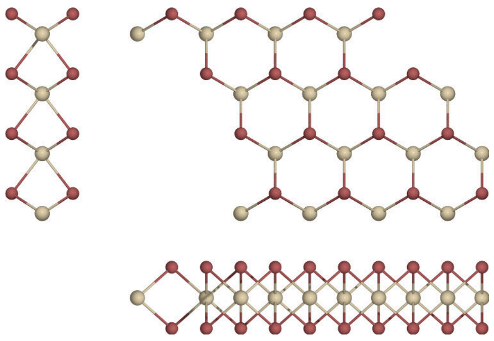

Figure 214: Structure representation

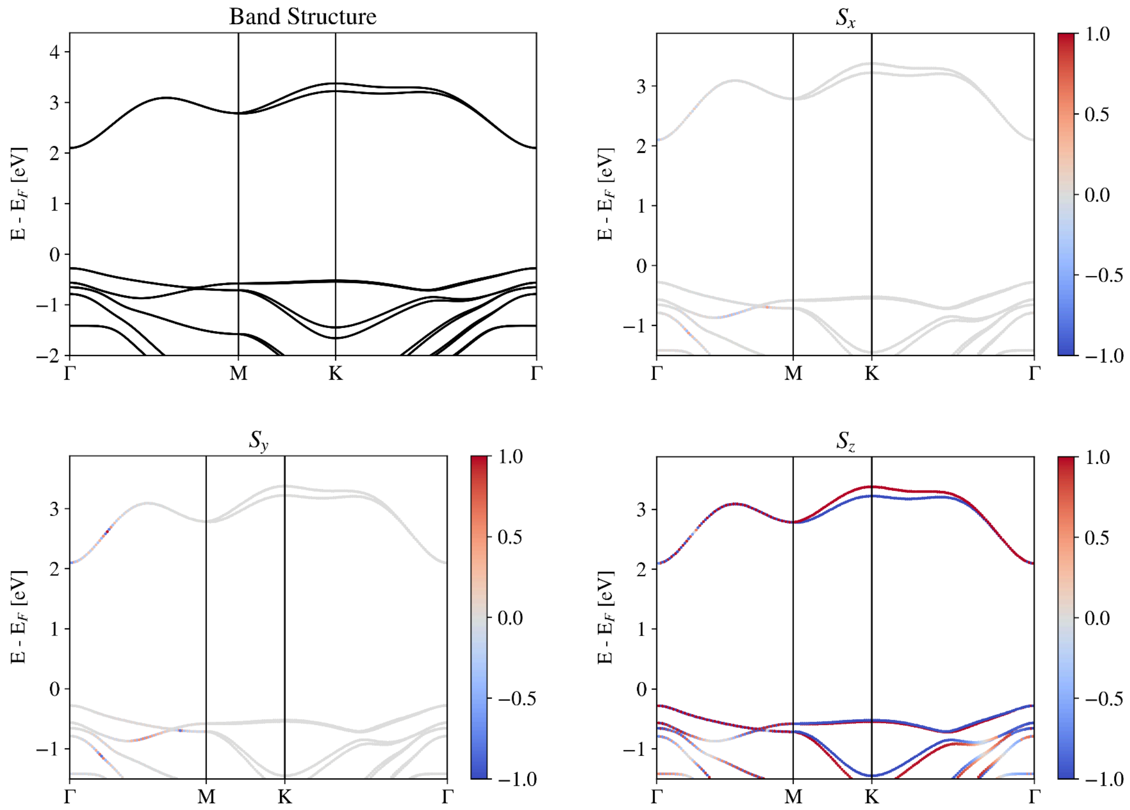


Figure 215: Band structure and spin polarization projections.

## 2.108 CdBr2-bb3c9722fb14

- **Formula**: CdBr2
- **Structural Cluster**: AB2-11
- **Band gap (PBE)**: 2.939 eV
- **Energy above convex hull (C2DB)**: 0.02 eV
- **Space group symbol**: $P\bar{4}m2$
- **Space group number**: 115
- **Polar structure**: False

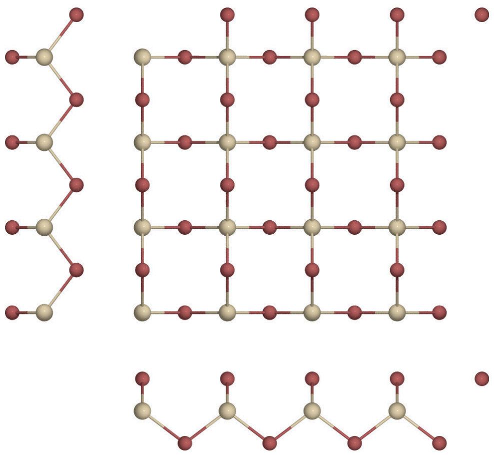

Figure 216: Structure representation

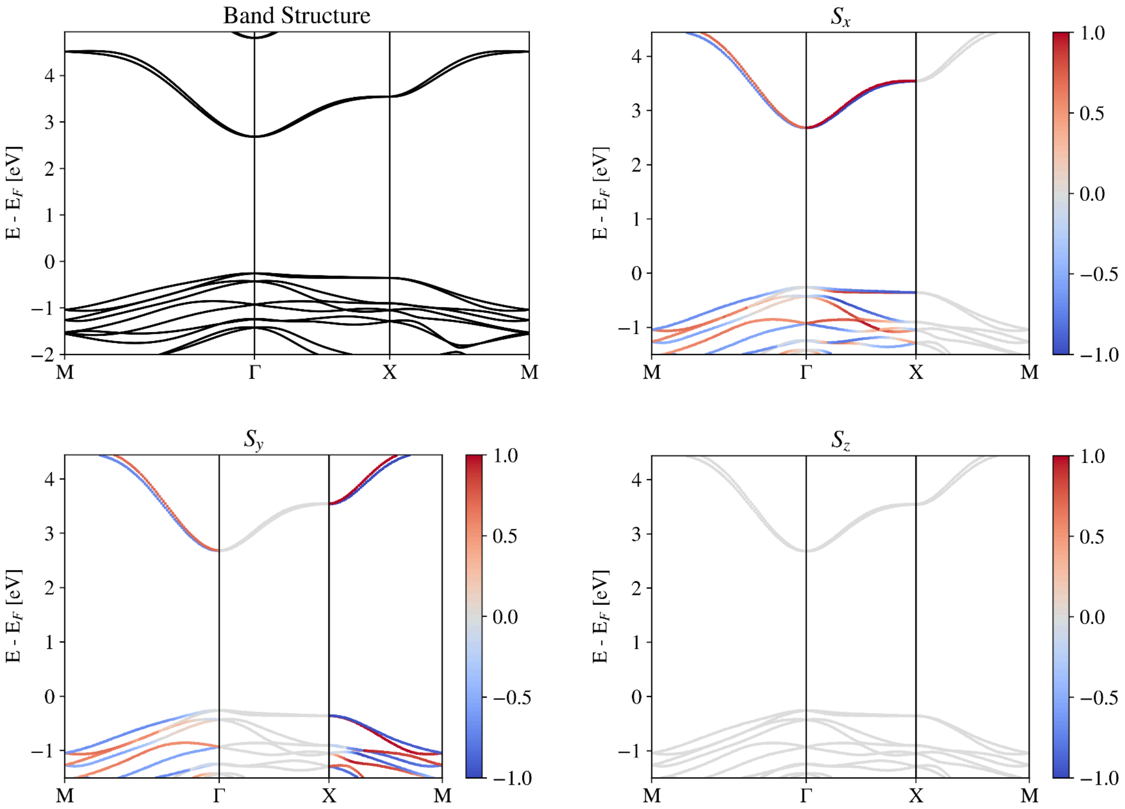


Figure 217: Band structure and spin polarization projections.

## 2.109 CdCl2-46c028e03e8b

- **Formula**: CdCl2
- **Structural Cluster**: AB2-4
- **Band gap (PBE)**: 3.111 eV
- **Energy above convex hull (C2DB)**: 0.13 eV
- **Space group symbol**: $P\bar{6}m2$
- **Space group number**: 187
- **Polar structure**: False

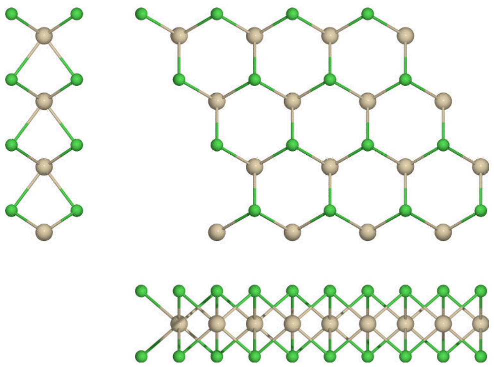

Figure 218: Structure representation

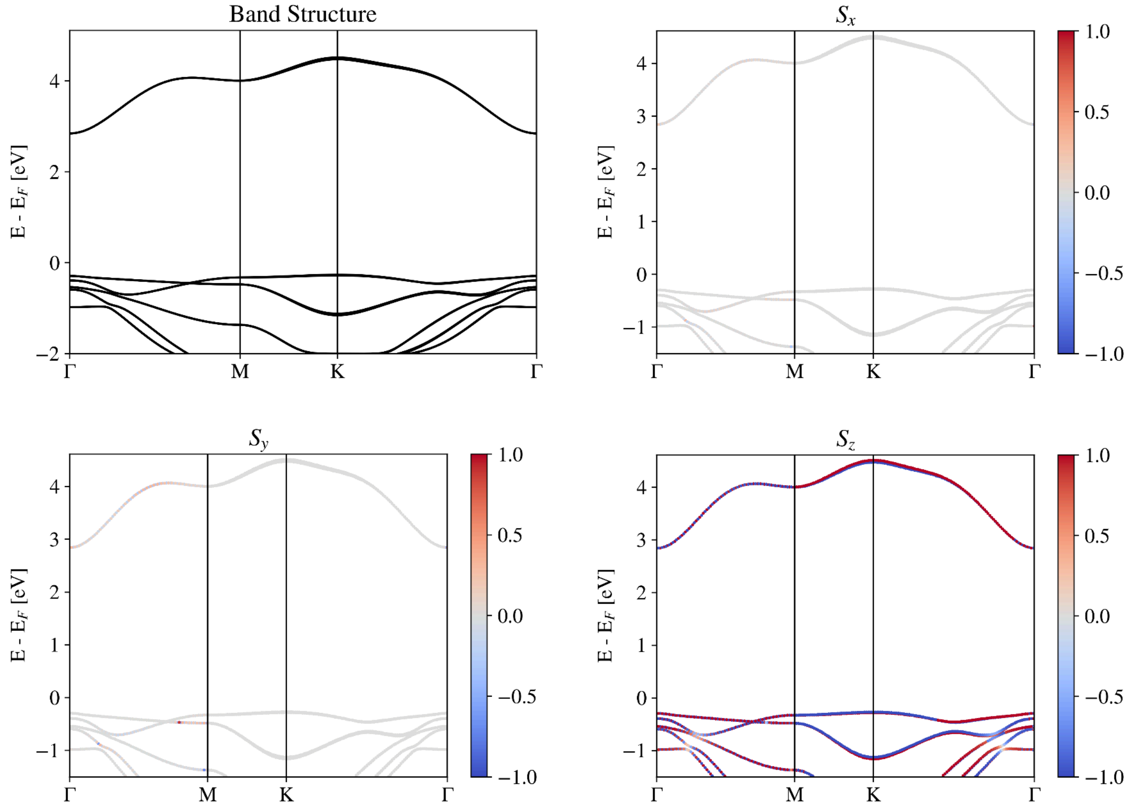


Figure 219: Band structure and spin polarization projections.

### 2.110 CdCl2-ff50eed37ec7

- **Formula**: CdCl2
- **Structural Cluster**: AB2-11
- **Band gap (PBE)**: 3.632 eV
- **Energy above convex hull (C2DB)**: 0.043 eV
- **Space group symbol**: $P\bar{4}m2$
- **Space group number**: 115
- **Polar structure**: False

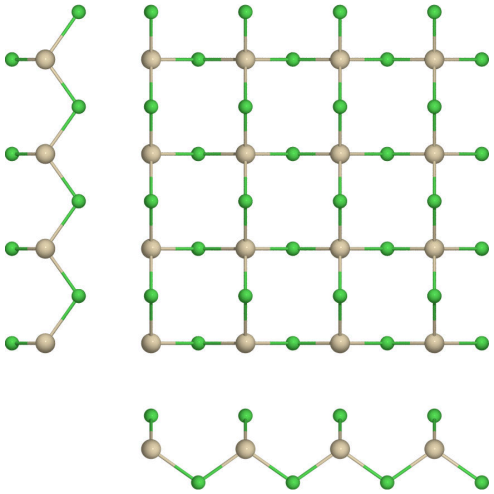

Figure 220: Structure representation

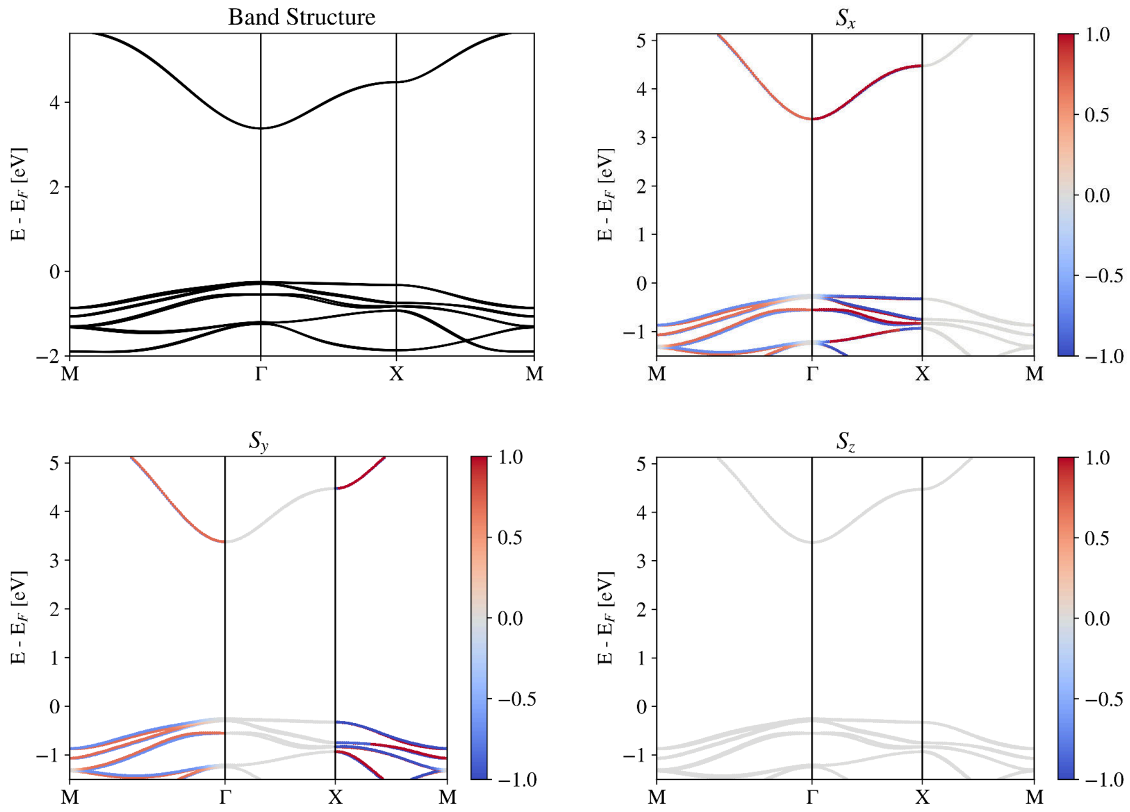


Figure 221: Band structure and spin polarization projections.

## 2.111 CdF2-14736784891b

- **Formula**: CdF2
- **Structural Cluster**: AB2-11
- **Band gap (PBE)**: 3.802 eV
- **Energy above convex hull (C2DB)**: 0.208 eV
- **Space group symbol**: $P\bar{4}m2$
- **Space group number**: 115
- **Polar structure**: False

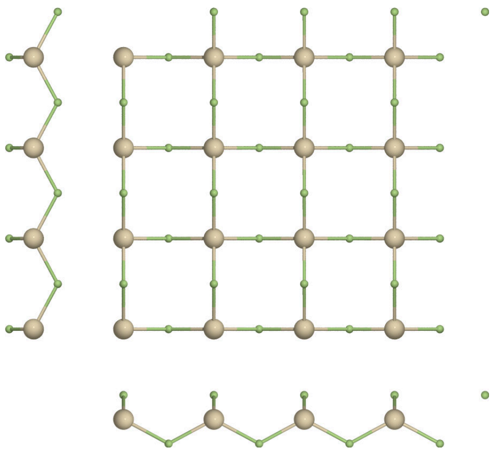

Figure 222: Structure representation

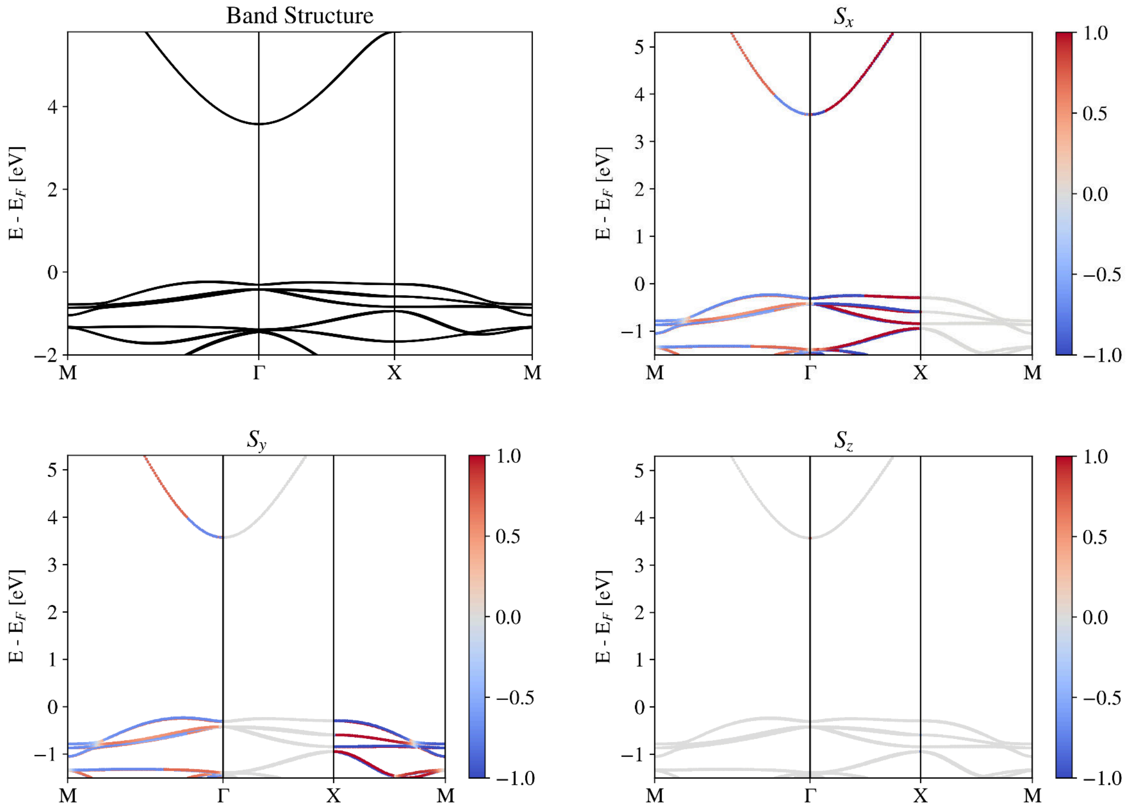


Figure 223: Band structure and spin polarization projections.

## 2.112 CdI2-66c5fba8ad87

- **Formula**: CdI2
- **Structural Cluster**: AB2-4
- **Band gap (PBE)**: 1.527 eV
- **Energy above convex hull (C2DB)**: 0.156 eV
- **Space group symbol**: $P\bar{6}m2$
- **Space group number**: 187
- **Polar structure**: False

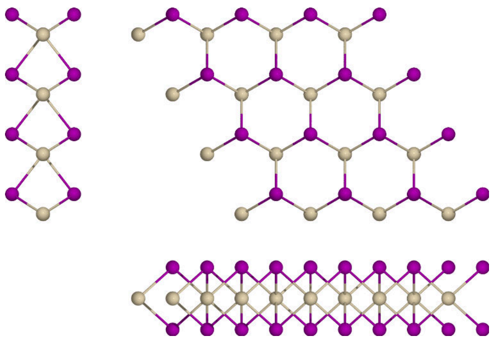

Figure 224: Structure representation

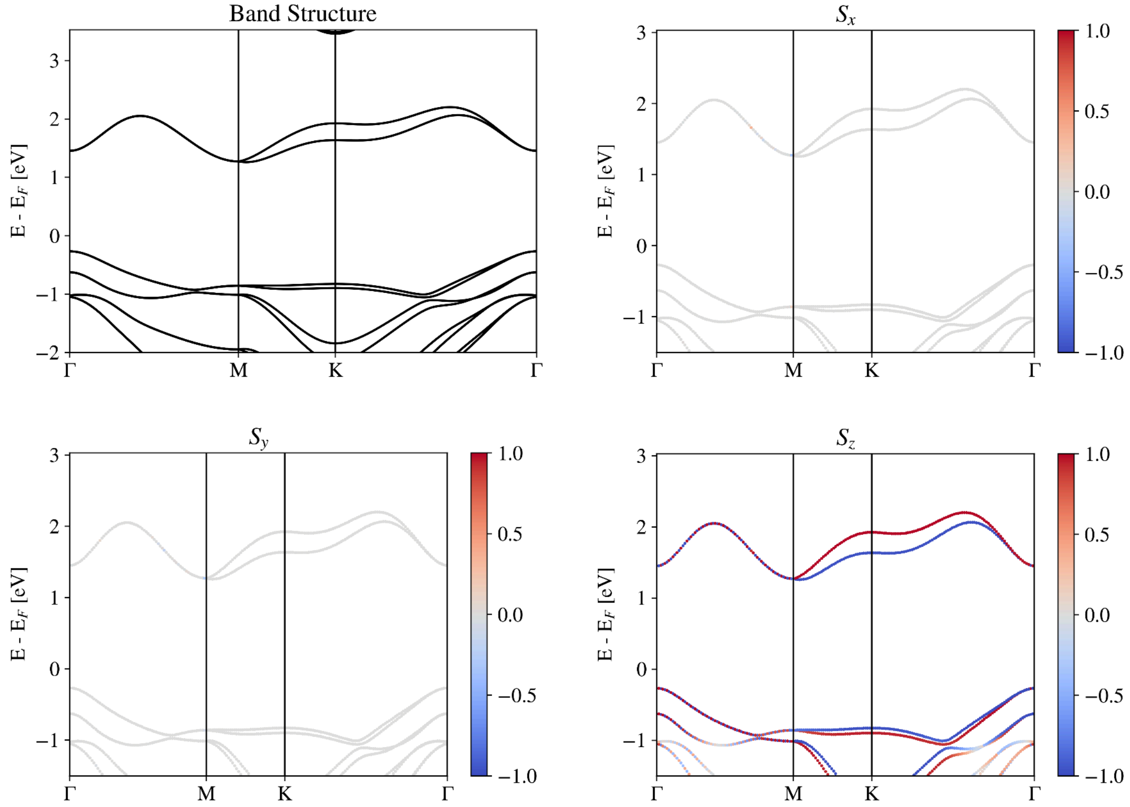


Figure 225: Band structure and spin polarization projections.

## 2.113 CdI2-d63ad801fdb5

- **Formula**: CdI2
- **Structural Cluster**: AB2-11
- **Band gap (PBE)**: 2.38 eV
- **Energy above convex hull (C2DB)**: 0.0 eV
- **Space group symbol**: $P\bar{4}m2$
- **Space group number**: 115
- **Polar structure**: False

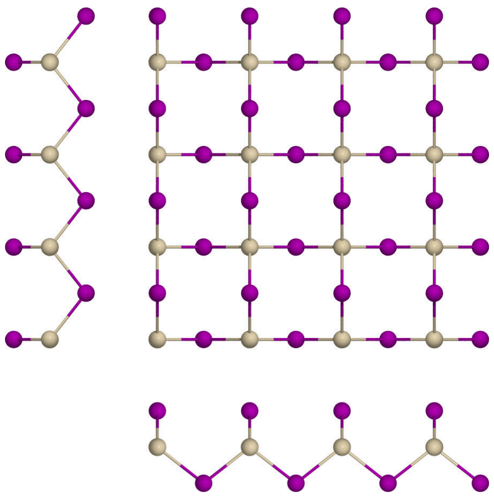

Figure 226: Structure representation

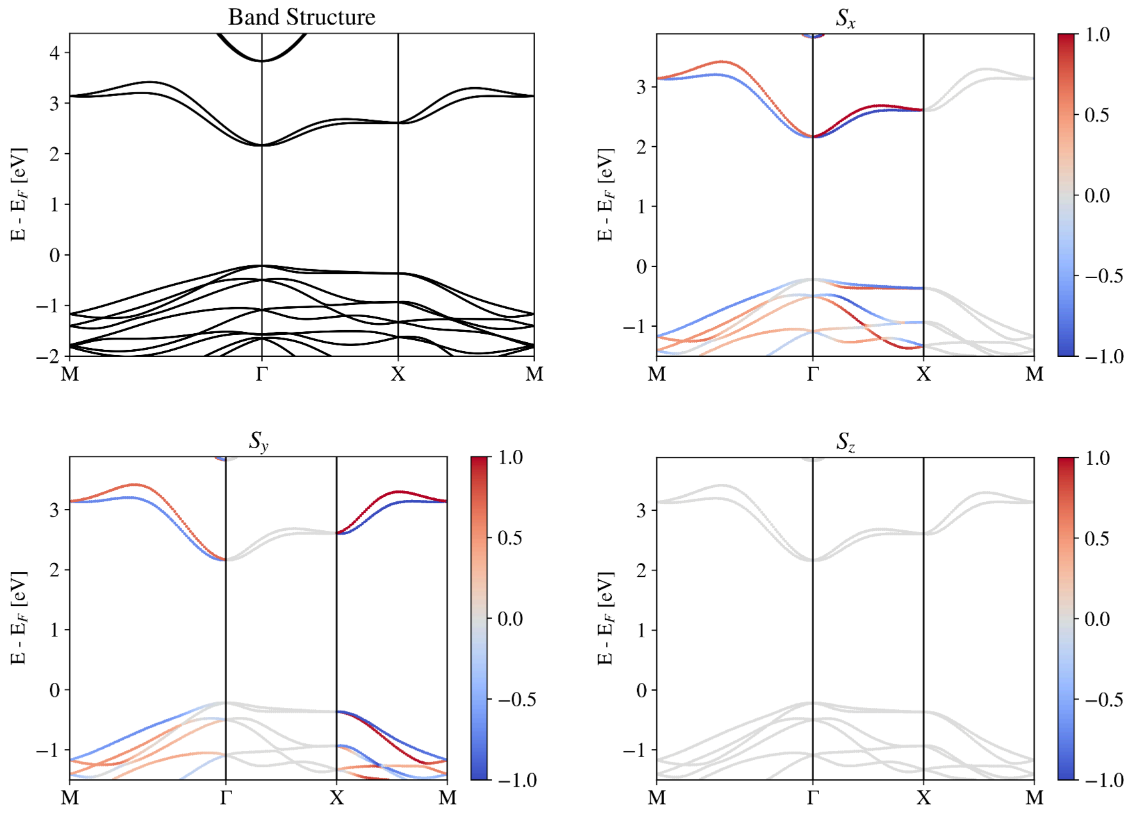


Figure 227: Band structure and spin polarization projections.

## 2.114 Cl2Cu2-c1a86f114149

- **Formula**: Cl2Cu2
- **Structural Cluster**: AB-5
- **Band gap (PBE)**: 1.218 eV
- **Energy above convex hull (C2DB)**: 0.018 eV
- **Space group symbol**: $P1$
- **Space group number**: 1
- **Polar structure**: True

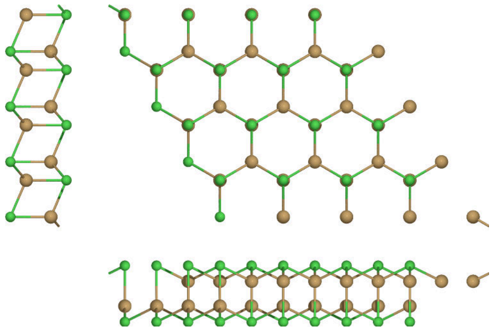

Figure 228: Structure representation

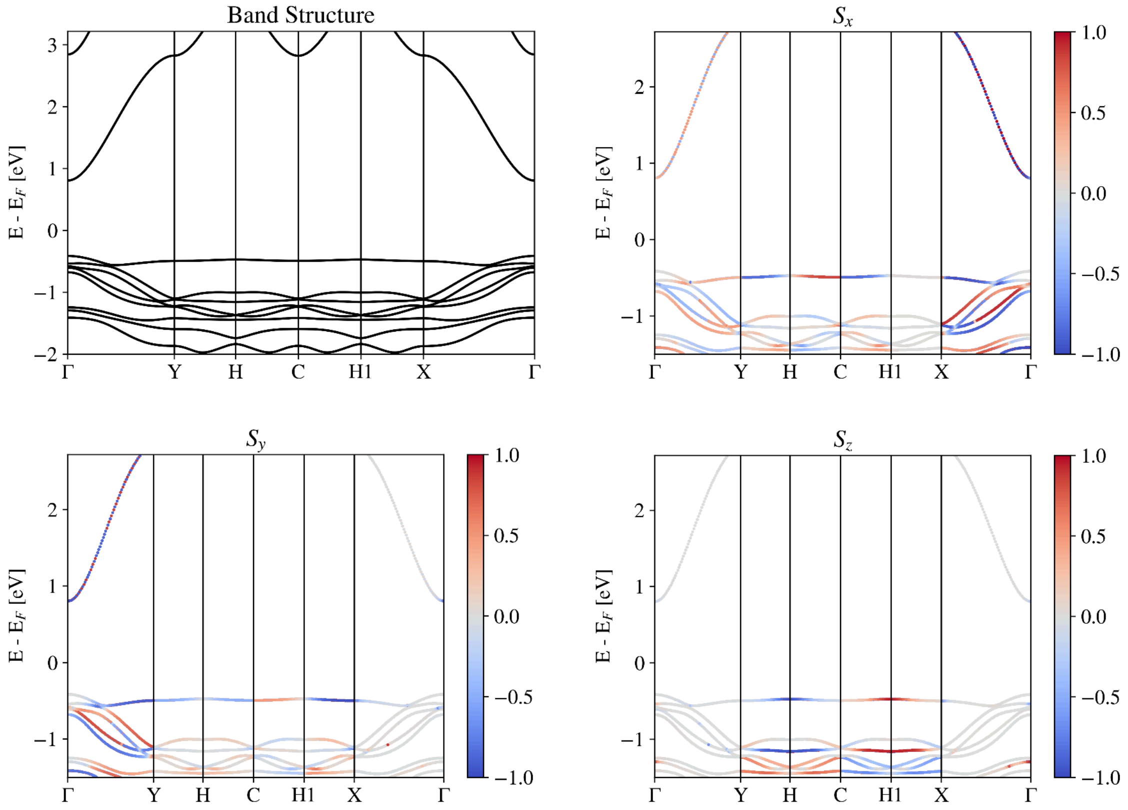


Figure 229: Band structure and spin polarization projections.

## 2.115 Cl2In2-9d0130e99f0c

- **Formula**: Cl2In2
- **Structural Cluster**: AB-6
- **Band gap (PBE)**: 1.578 eV
- **Energy above convex hull (C2DB)**: 0.096 eV
- **Space group symbol**: $P1$
- **Space group number**: 1
- **Polar structure**: True

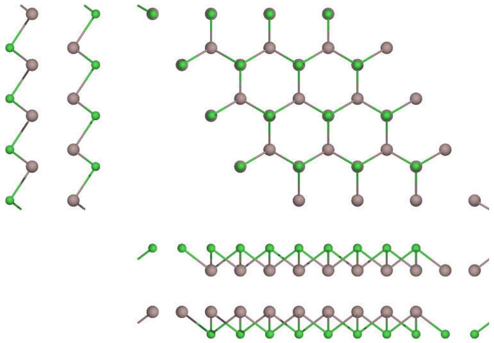

Figure 230: Structure representation

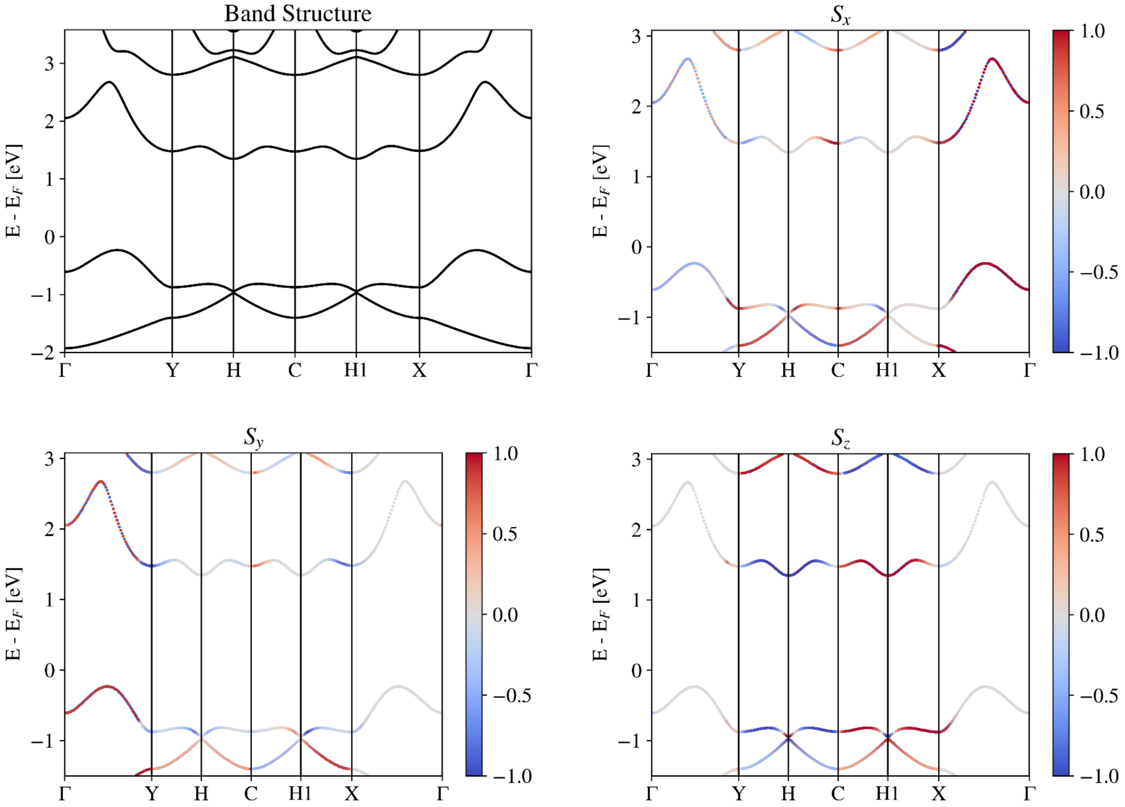


Figure 231: Band structure and spin polarization projections.

## 2.116 Cl2Pt2-93dfef2d1004

- **Formula**: Cl2Pt2
- **Structural Cluster**: AB-13
- **Band gap (PBE)**: 1.329 eV
- **Energy above convex hull (C2DB)**: 0.0 eV
- **Space group symbol**: $P1$
- **Space group number**: 1
- **Polar structure**: True

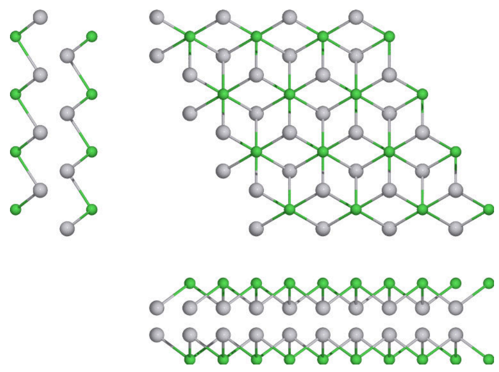

Figure 232: Structure representation

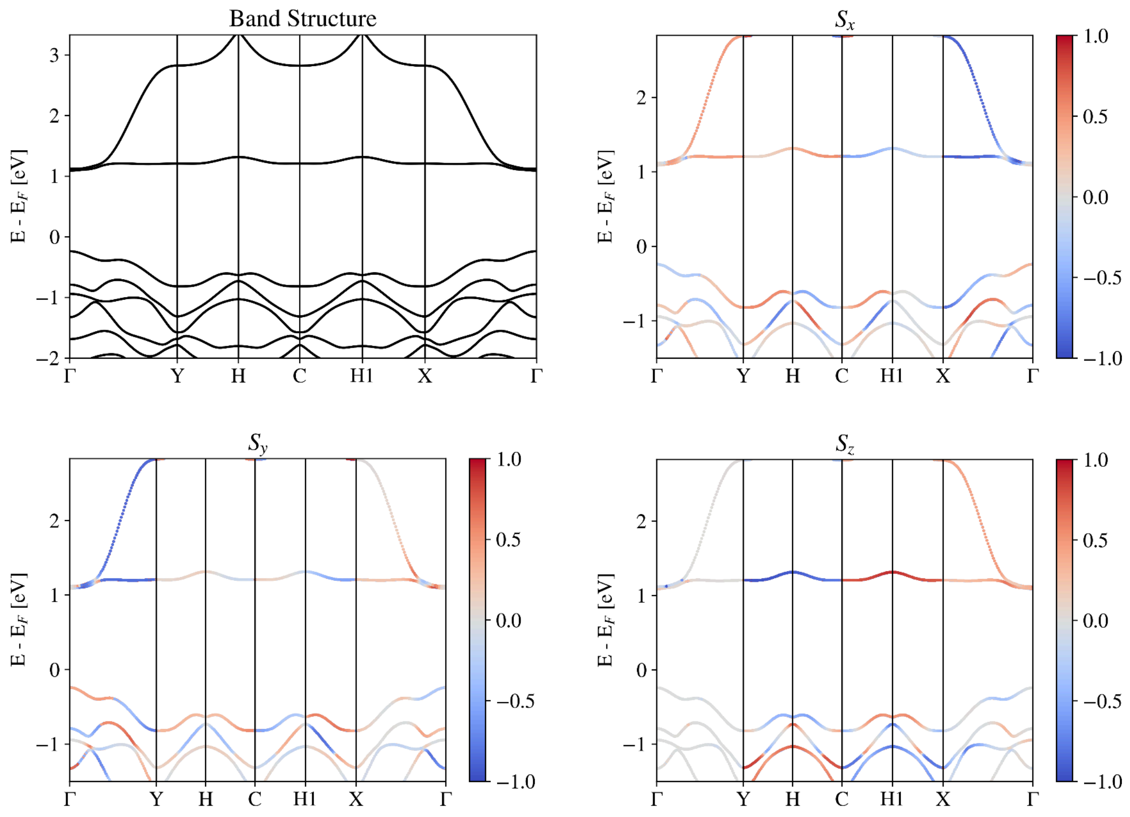


Figure 233: Band structure and spin polarization projections.

## 2.117 ClHfI-d3756ea15451

- **Formula**: ClHfI
- **Structural Cluster**: ABC-4
- **Band gap (PBE)**: 0.806 eV
- **Energy above convex hull (C2DB)**: 0.14 eV
- **Space group symbol**: $P3m1$
- **Space group number**: 156
- **Polar structure**: True

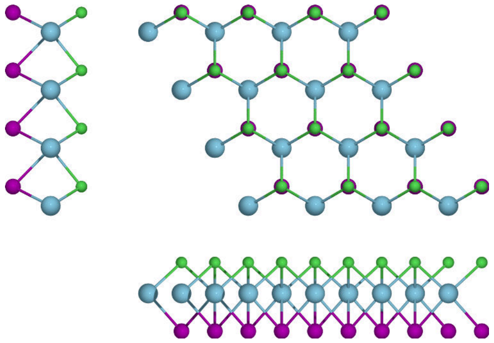

Figure 234: Structure representation

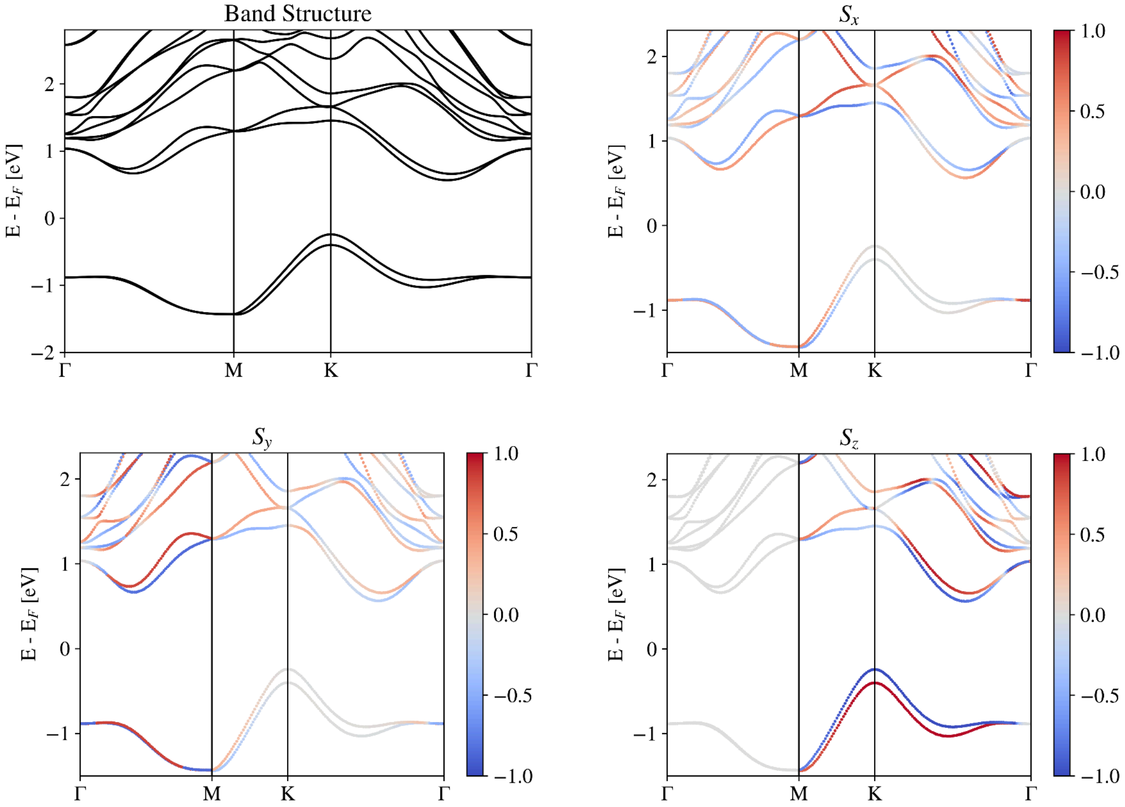


Figure 235: Band structure and spin polarization projections.

## 2.118 ClITi-ae06e7424bb1

- **Formula**: ClITi
- **Structural Cluster**: ABC-4
- **Band gap (PBE)**: 0.746 eV
- **Energy above convex hull (C2DB)**: 0.115 eV
- **Space group symbol**: $P3m1$
- **Space group number**: 156
- **Polar structure**: True

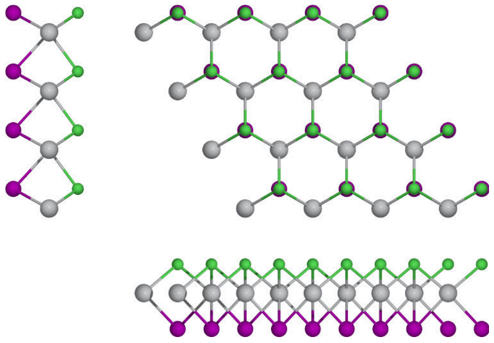

Figure 236: Structure representation

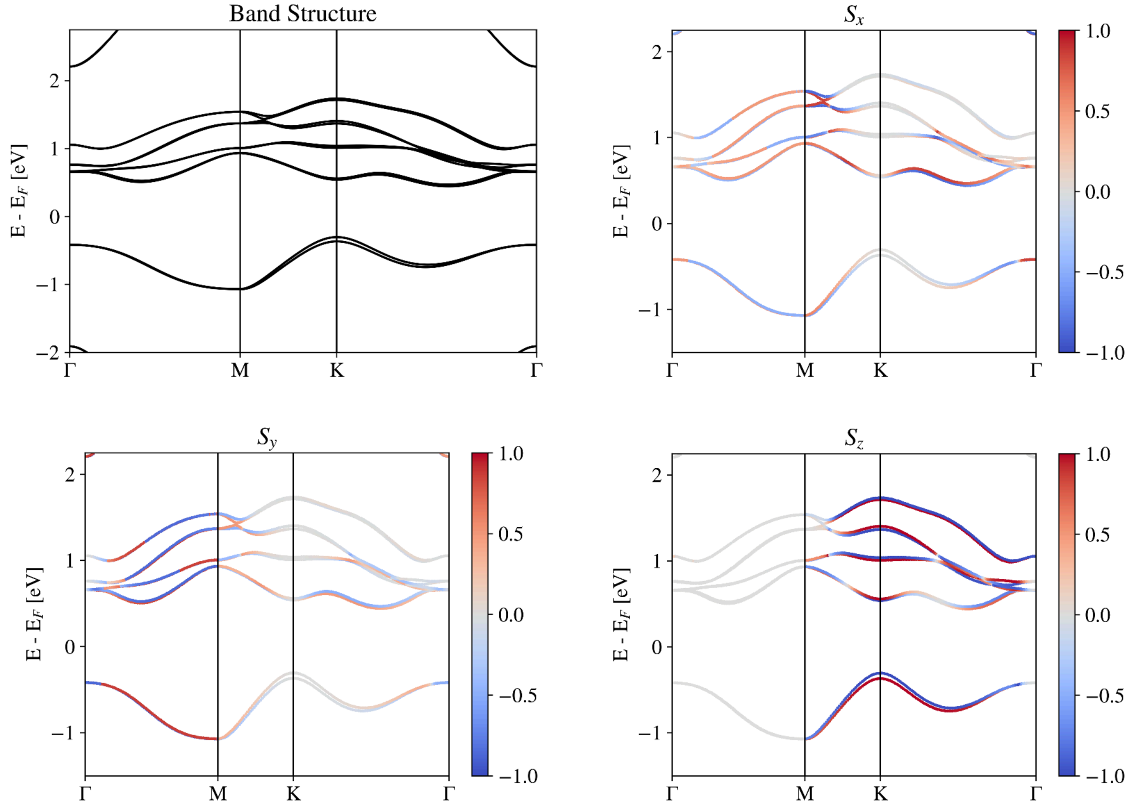


Figure 237: Band structure and spin polarization projections.

## 2.119 ClIZr-73202b4b7837

- **Formula**: ClIZr
- **Structural Cluster**: ABC-4
- **Band gap (PBE)**: 0.883 eV
- **Energy above convex hull (C2DB)**: 0.078 eV
- **Space group symbol**: $P3m1$
- **Space group number**: 156
- **Polar structure**: True

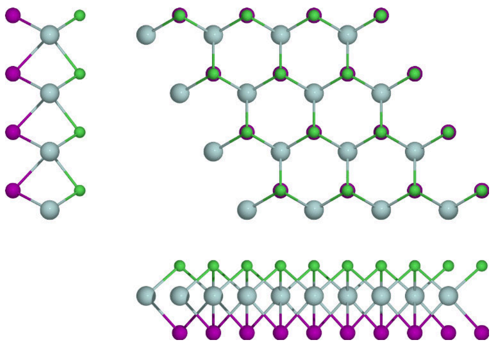

Figure 238: Structure representation

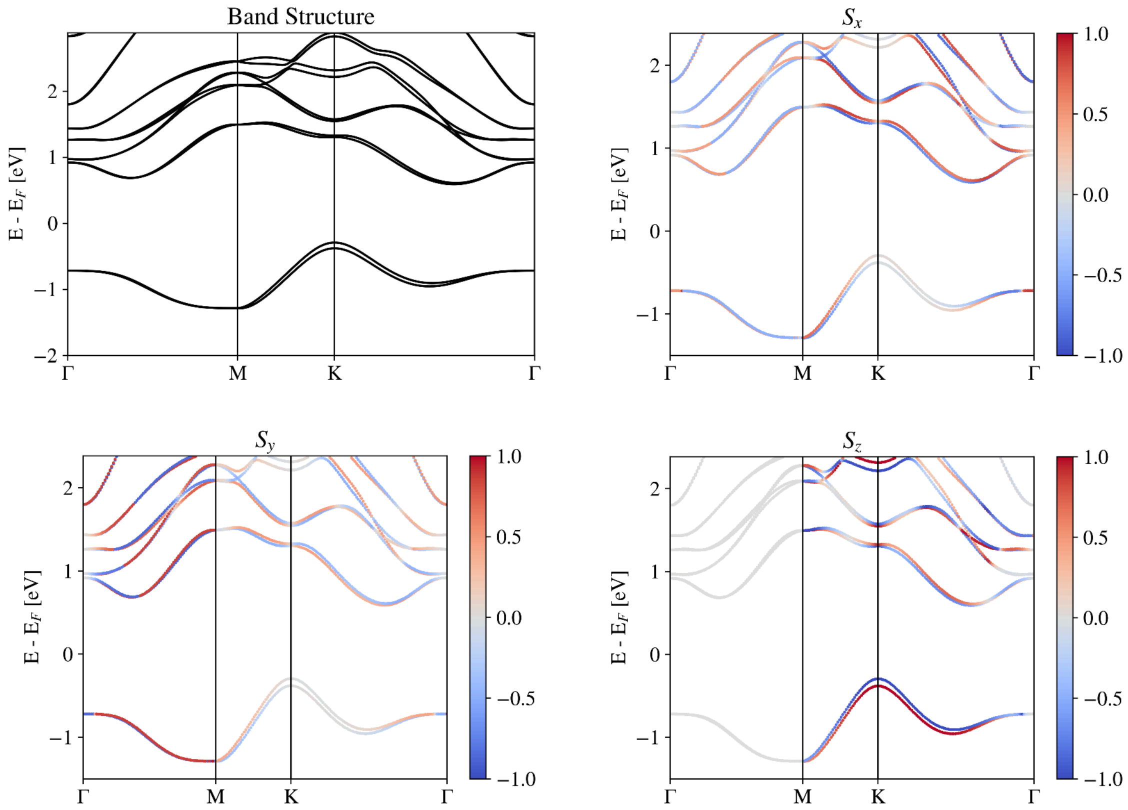


Figure 239: Band structure and spin polarization projections.

## 2.120 ClSSb-0495f35048b5

- **Formula**: ClSSb
- **Structural Cluster**: ABC-4
- **Band gap (PBE)**: 1.675 eV
- **Energy above convex hull (C2DB)**: 0.179 eV
- **Space group symbol**: $P3m1$
- **Space group number**: 156
- **Polar structure**: True

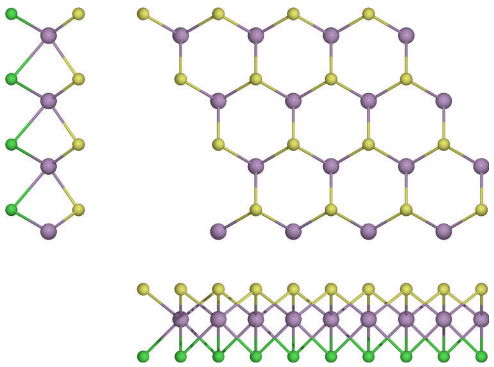

Figure 240: Structure representation

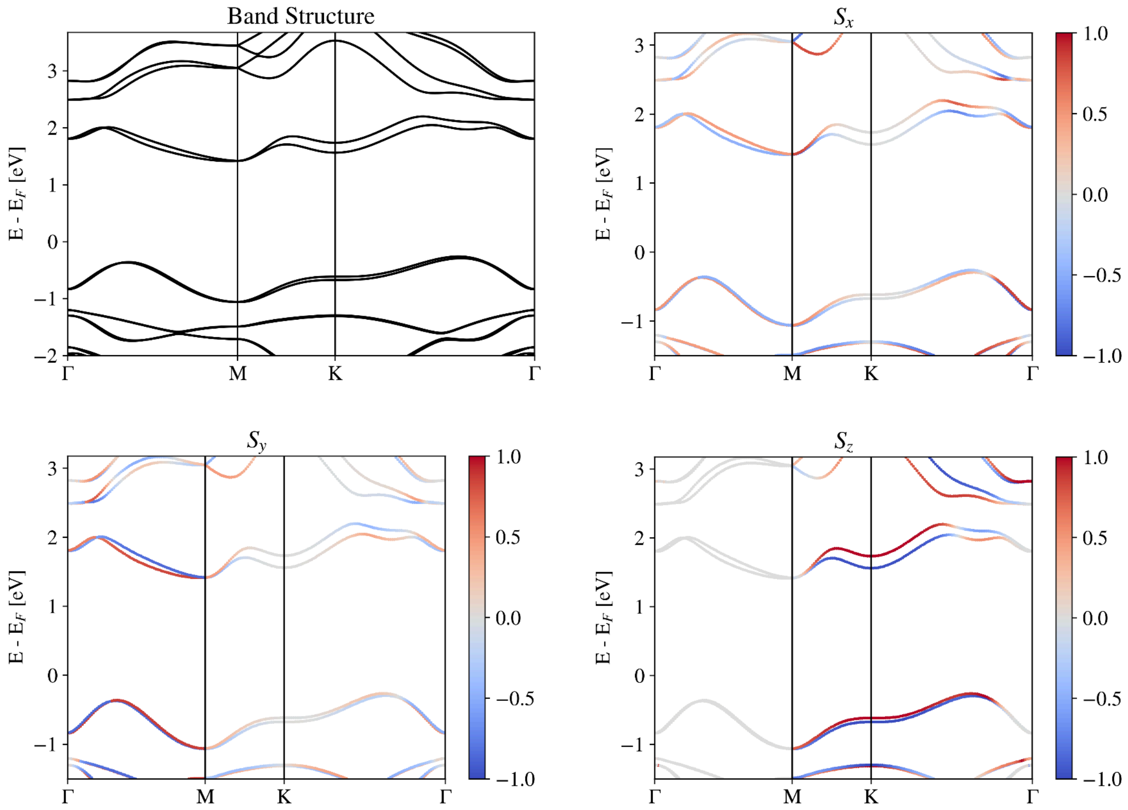


Figure 241: Band structure and spin polarization projections.

## 2.121 ClSSb-9188c300265c

- **Formula**: ClSSb
- **Structural Cluster**: ABC-3
- **Band gap (PBE)**: 1.332 eV
- **Energy above convex hull (C2DB)**: 0.048 eV
- **Space group symbol**: $P3m1$
- **Space group number**: 156
- **Polar structure**: True

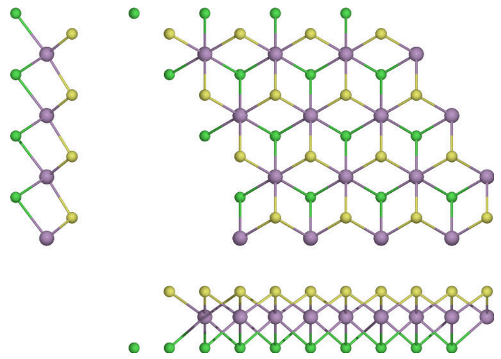

Figure 242: Structure representation

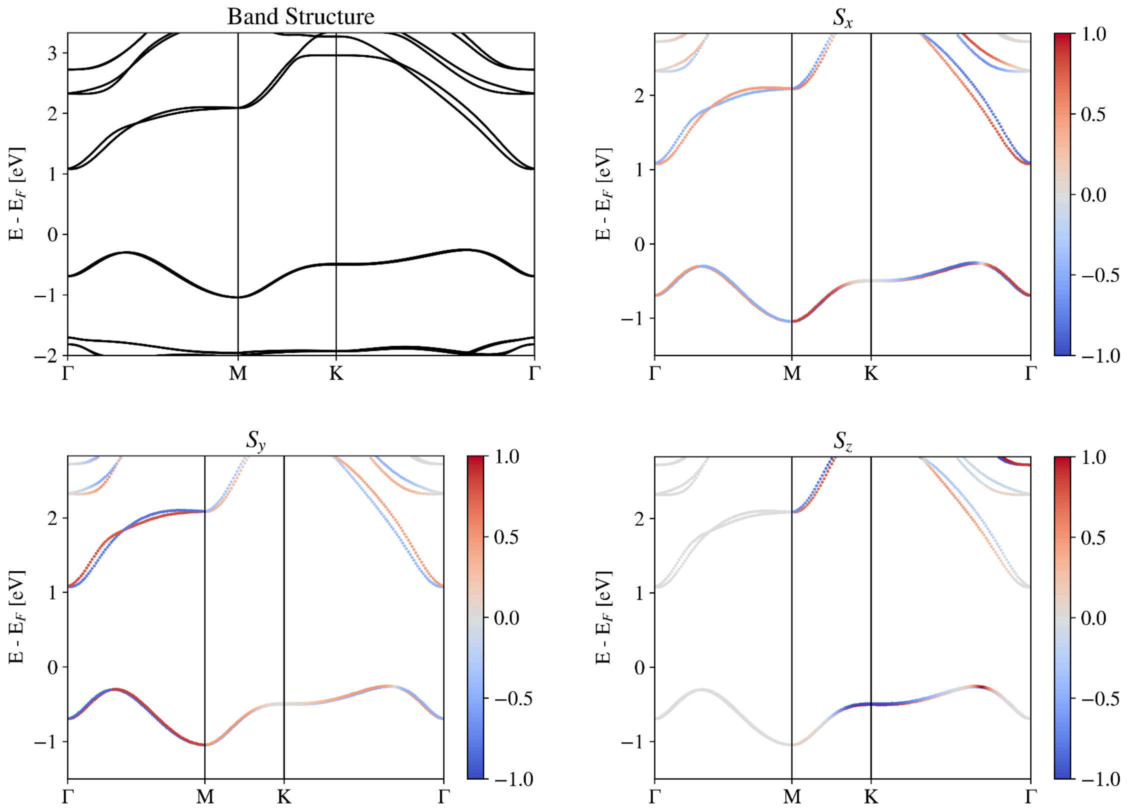


Figure 243: Band structure and spin polarization projections.

## 2.122 ClSbSe-0c0fbdaf8f4a

- **Formula**: ClSbSe
- **Structural Cluster**: ABC-3
- **Band gap (PBE)**: 1.177 eV
- **Energy above convex hull (C2DB)**: 0.014 eV
- **Space group symbol**: $P3m1$
- **Space group number**: 156
- **Polar structure**: True

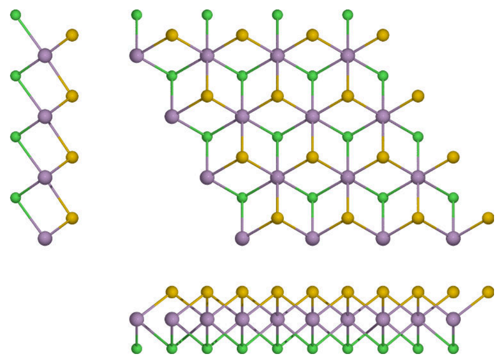

Figure 244: Structure representation

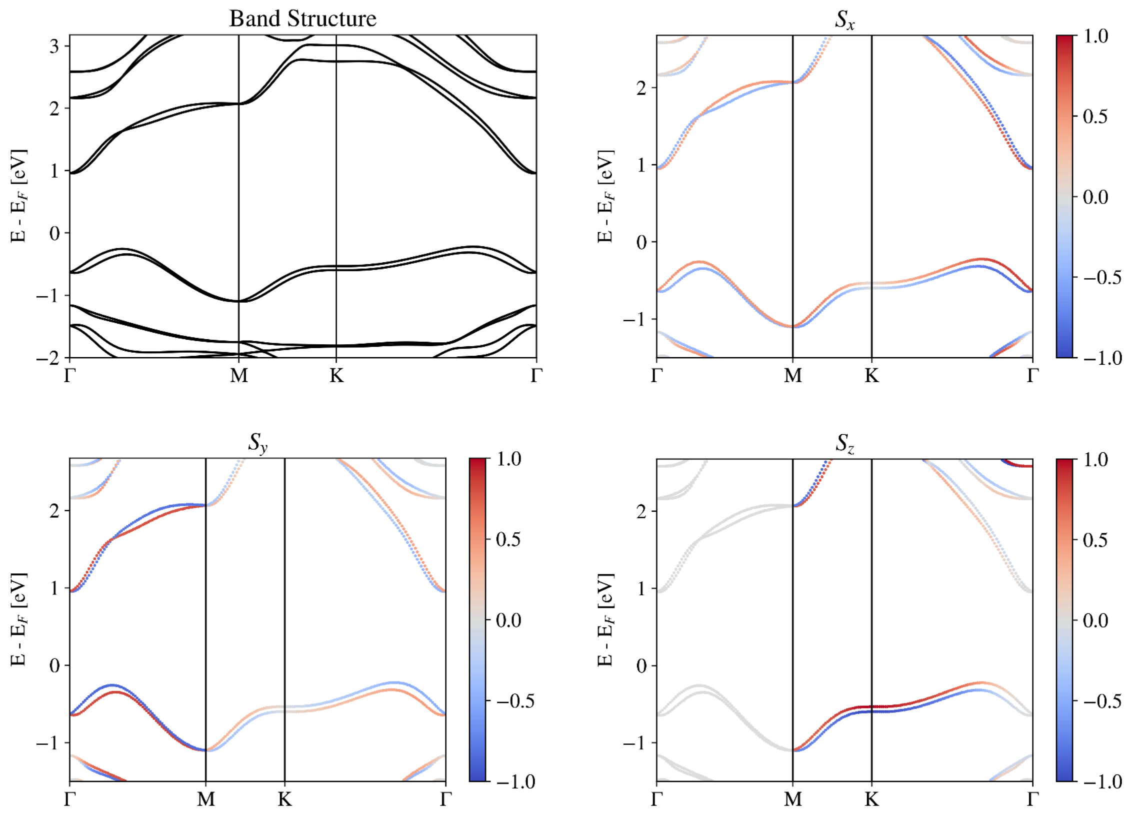


Figure 245: Band structure and spin polarization projections.

## 2.123 ClSbSe-f705a30af945

- **Formula**: ClSbSe
- **Structural Cluster**: ABC-4
- **Band gap (PBE)**: 1.68 eV
- **Energy above convex hull (C2DB)**: 0.146 eV
- **Space group symbol**: $P3m1$
- **Space group number**: 156
- **Polar structure**: True

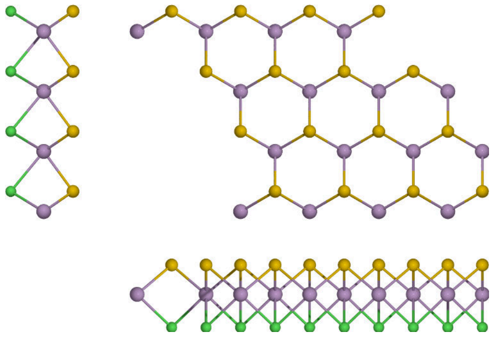

Figure 246: Structure representation

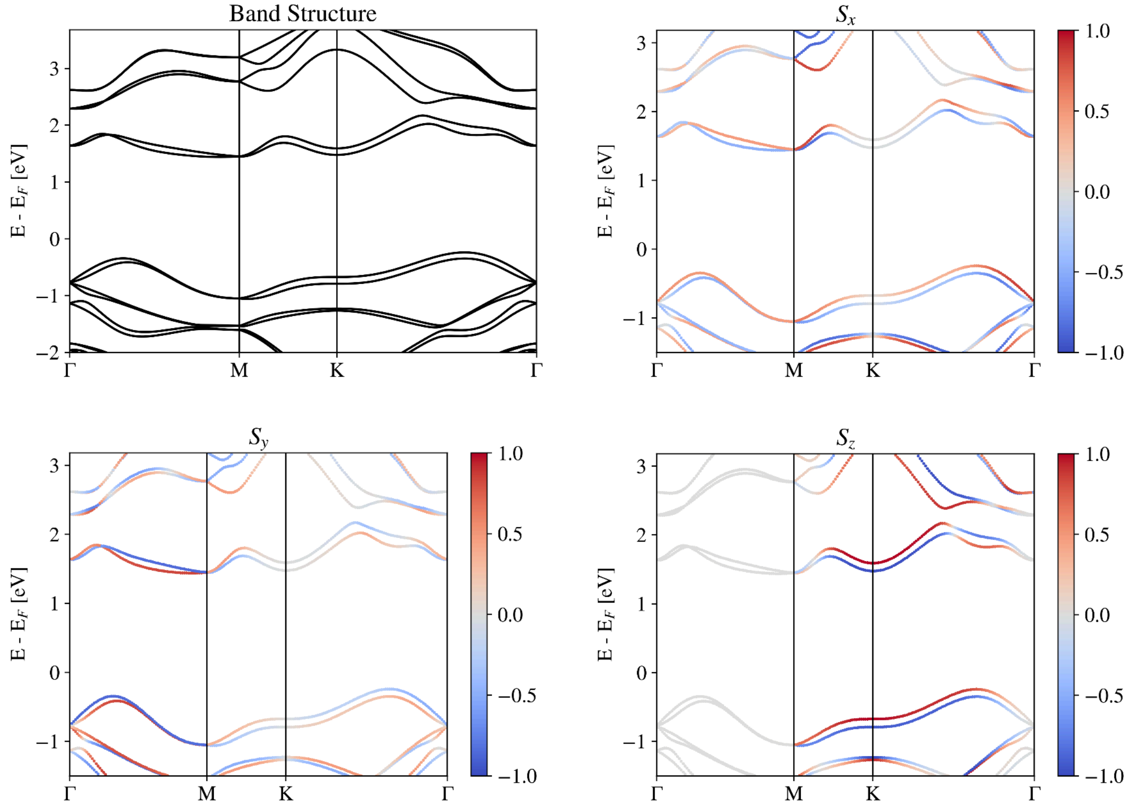


Figure 247: Band structure and spin polarization projections.

## 2.124 ClSbTe-04fdd7d1ec5c

- **Formula**: ClSbTe
- **Structural Cluster**: ABC-4
- **Band gap (PBE)**: 1.439 eV
- **Energy above convex hull (C2DB)**: 0.153 eV
- **Space group symbol**: $P3m1$
- **Space group number**: 156
- **Polar structure**: True

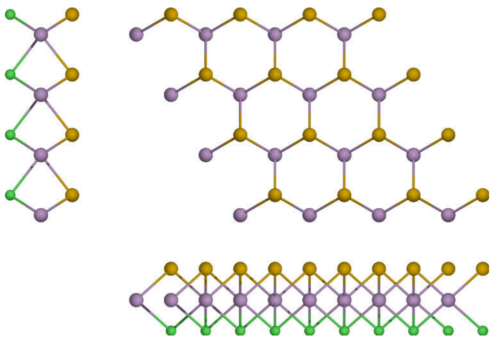

Figure 248: Structure representation

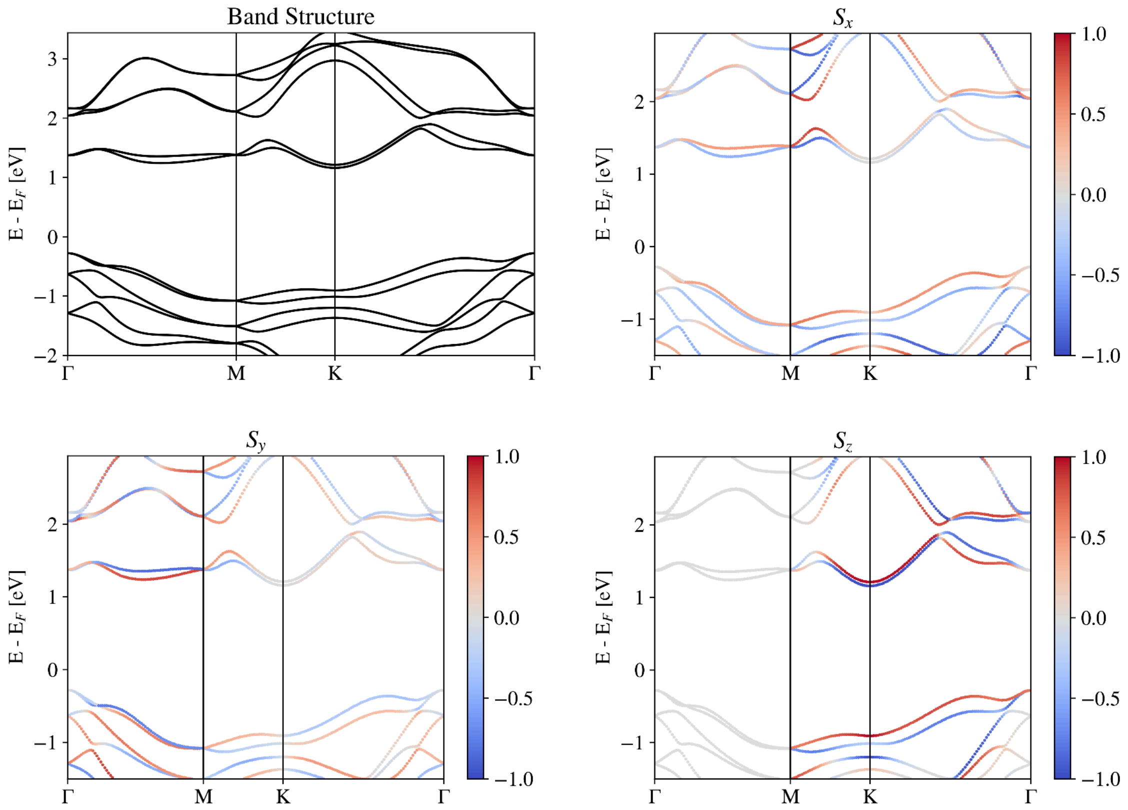


Figure 249: Band structure and spin polarization projections.

## 2.125 ClSbTe-da5fd2bb47af

- **Formula**: ClSbTe
- **Structural Cluster**: ABC-3
- **Band gap (PBE)**: 1.291 eV
- **Energy above convex hull (C2DB)**: 0.008 eV
- **Space group symbol**: $P3m1$
- **Space group number**: 156
- **Polar structure**: True

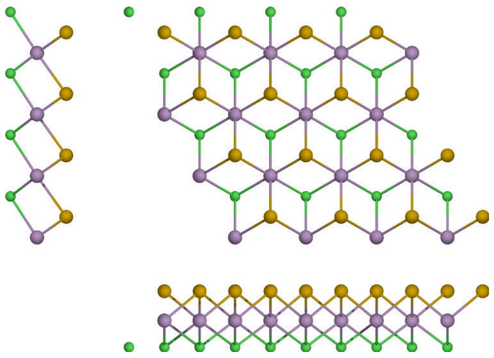

Figure 250: Structure representation

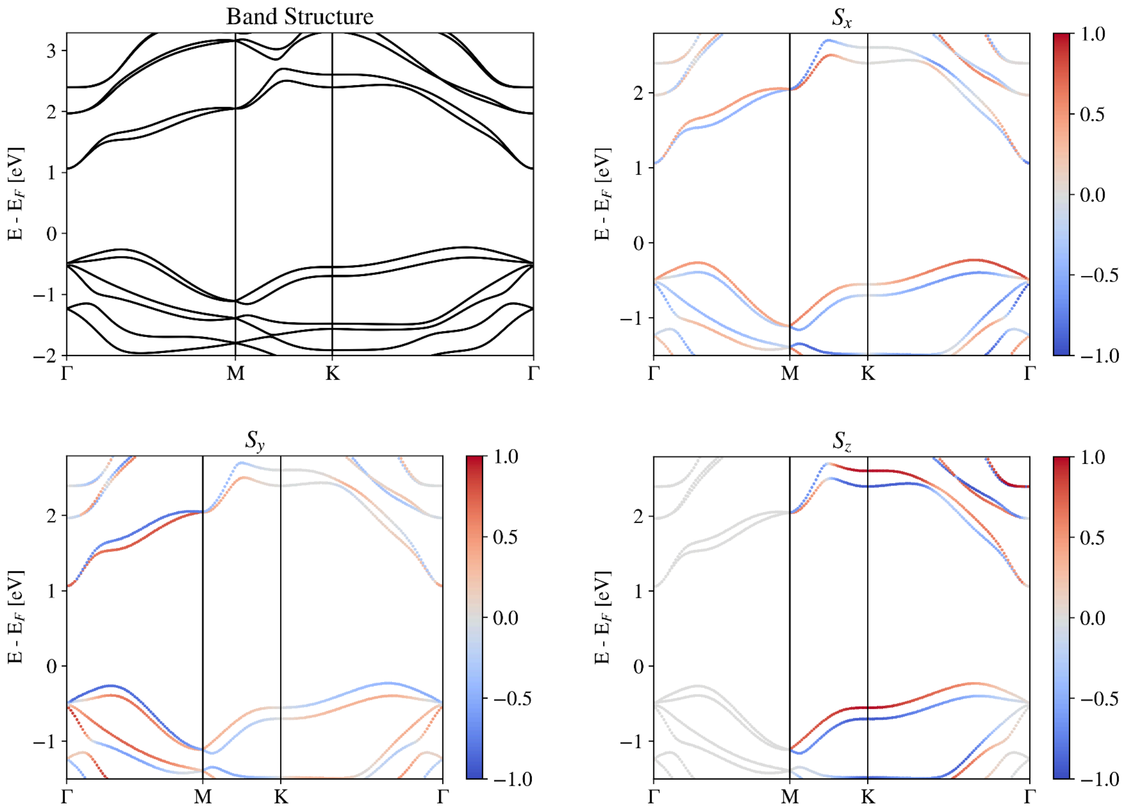


Figure 251: Band structure and spin polarization projections.

## 2.126 Cr2Mo2S8-72b286460831

- **Formula**: Cr2Mo2S8
- **Structural Cluster**: ABC4-22
- **Band gap (PBE)**: 1.039 eV
- **Energy above convex hull (C2DB)**: 0.017 eV
- **Space group symbol**: $Pma2$
- **Space group number**: 28
- **Polar structure**: True

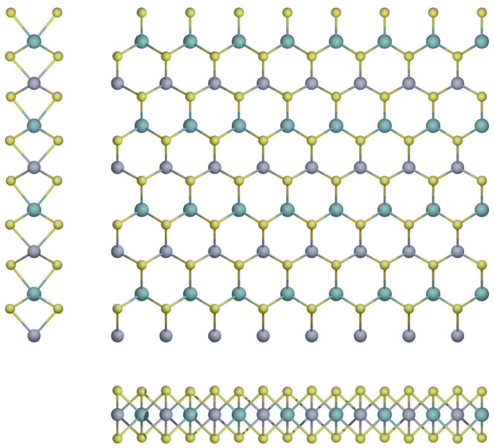

Figure 252: Structure representation

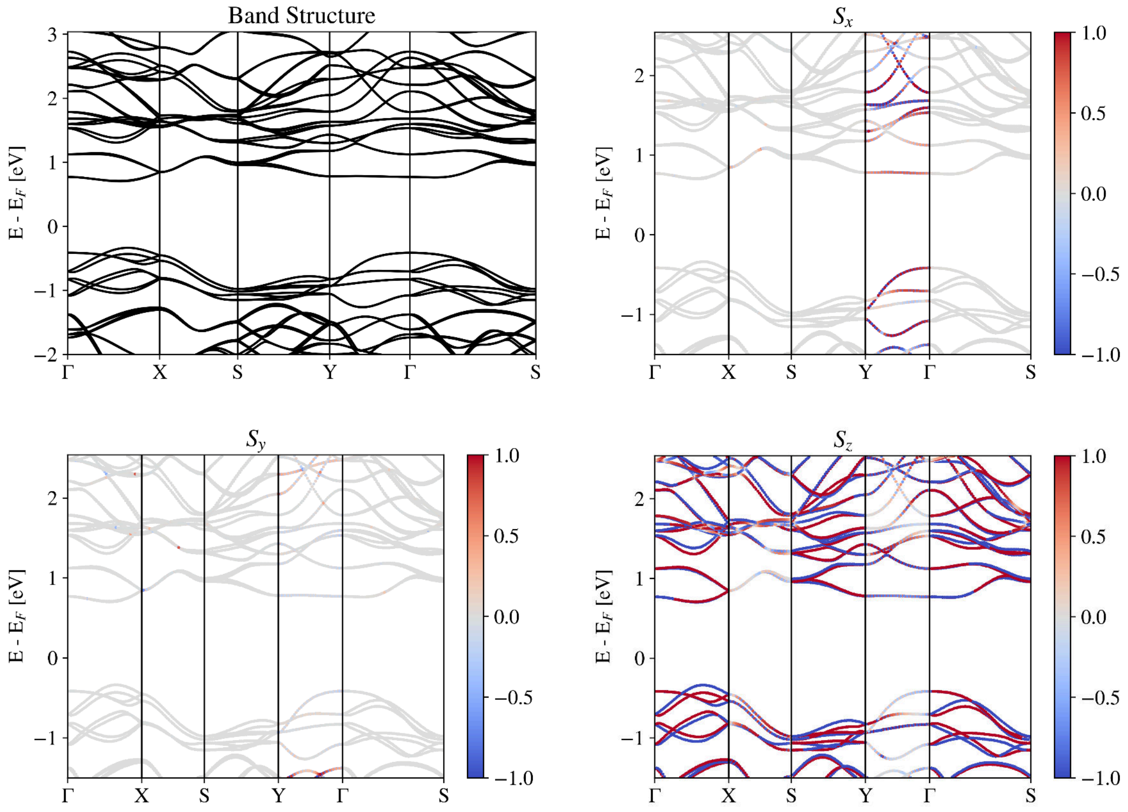


Figure 253: Band structure and spin polarization projections.

## 2.127 Cr2Mo2Se8-60065d3bbcf2

- **Formula**: Cr2Mo2Se8
- **Structural Cluster**: ABC4-22
- **Band gap (PBE)**: 0.837 eV
- **Energy above convex hull (C2DB)**: 0.016 eV
- **Space group symbol**: $P1$
- **Space group number**: 1
- **Polar structure**: True

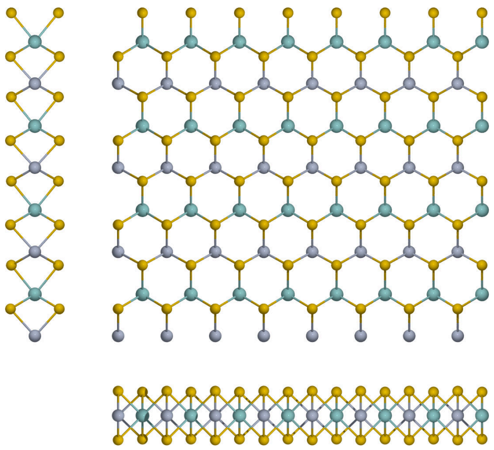

Figure 254: Structure representation

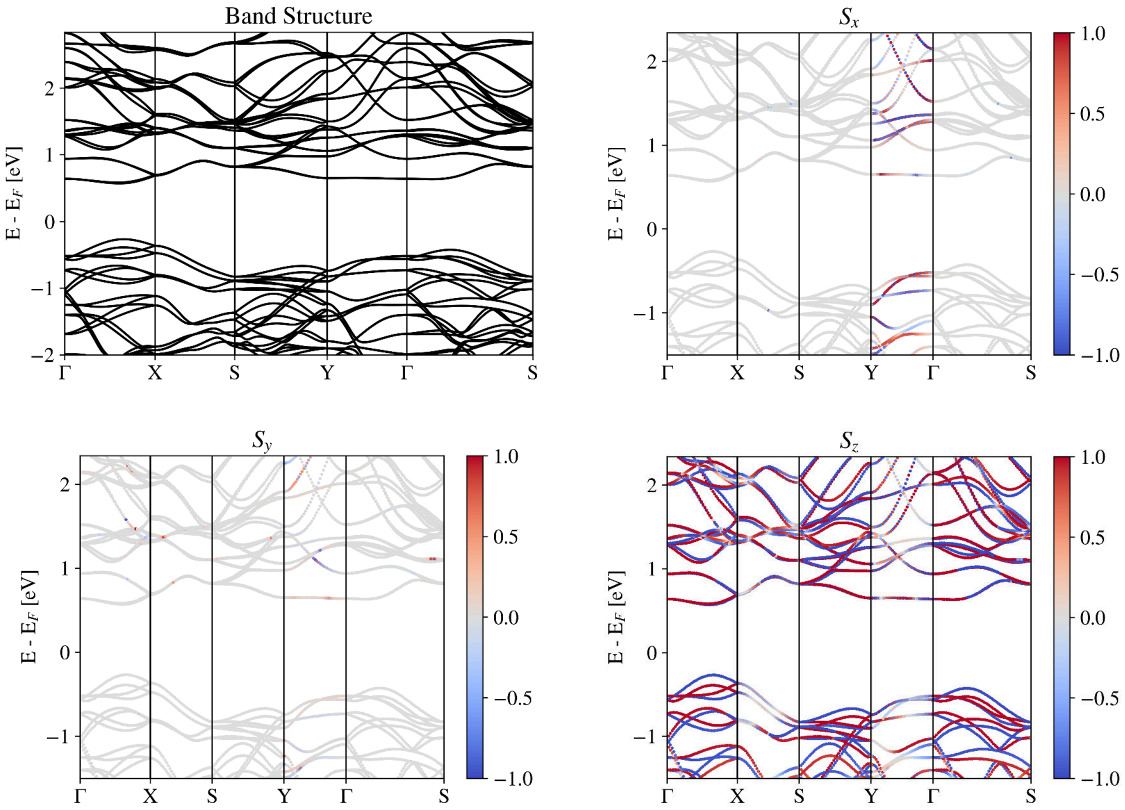


Figure 255: Band structure and spin polarization projections.

## 2.128 Cr2Mo2Te8-988b11badabb

- **Formula**: Cr2Mo2Te8
- **Structural Cluster**: ABC4-22
- **Band gap (PBE)**: 0.575 eV
- **Energy above convex hull (C2DB)**: 0.067 eV
- **Space group symbol**: $P1$
- **Space group number**: 1
- **Polar structure**: True

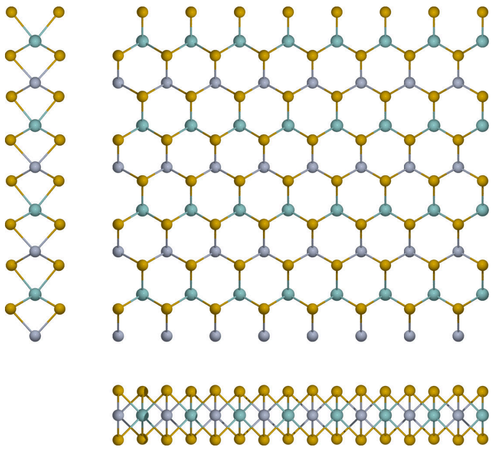

Figure 256: Structure representation

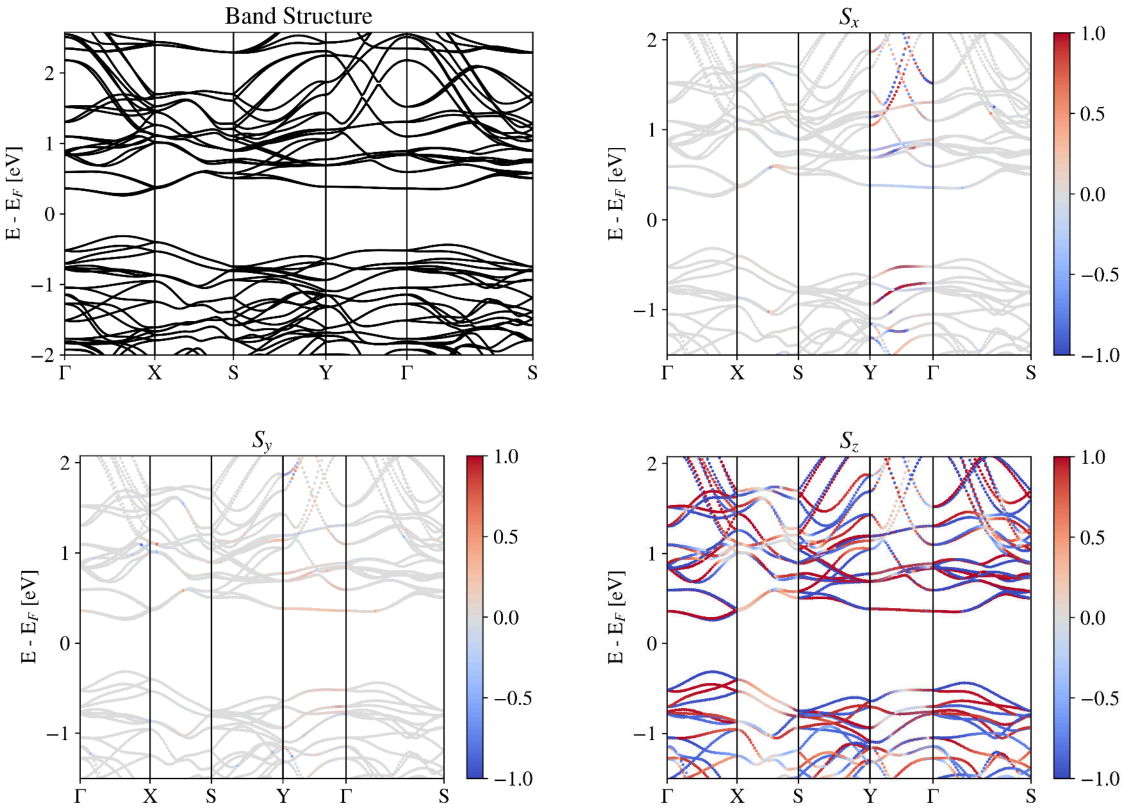


Figure 257: Band structure and spin polarization projections.

## 2.129 Cr2W2S8-5974b6403c31

- **Formula**: Cr2W2S8
- **Structural Cluster**: ABC4-22
- **Band gap (PBE)**: 0.967 eV
- **Energy above convex hull (C2DB)**: 0.014 eV
- **Space group symbol**: *Pma*2
- **Space group number**: 28
- **Polar structure**: True

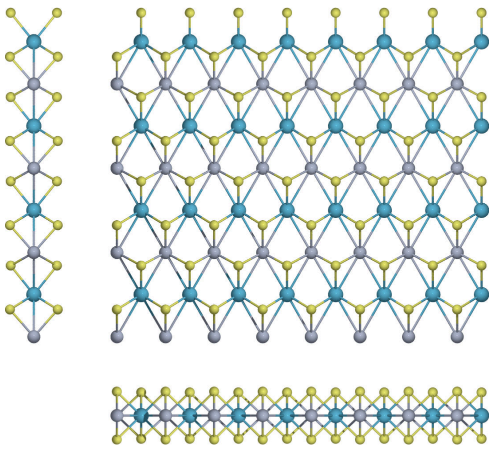

Figure 258: Structure representation

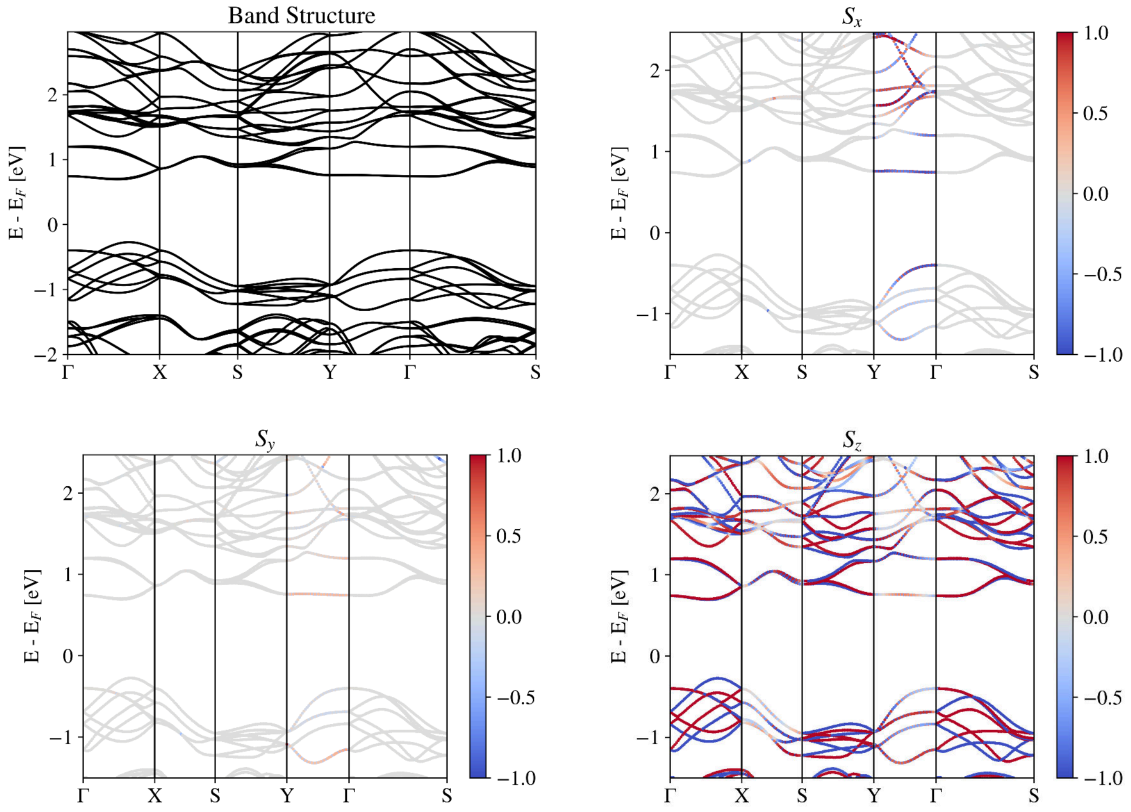


Figure 259: Band structure and spin polarization projections.

## 2.130 Cr2W2Se8-548aa830244c

- **Formula**: Cr2W2Se8
- **Structural Cluster**: ABC4-22
- **Band gap (PBE)**: 0.778 eV
- **Energy above convex hull (C2DB)**: 0.015 eV
- **Space group symbol**: $P1$
- **Space group number**: 1
- **Polar structure**: True

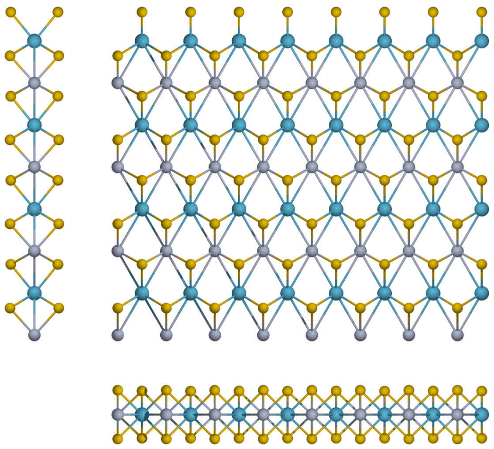

Figure 260: Structure representation

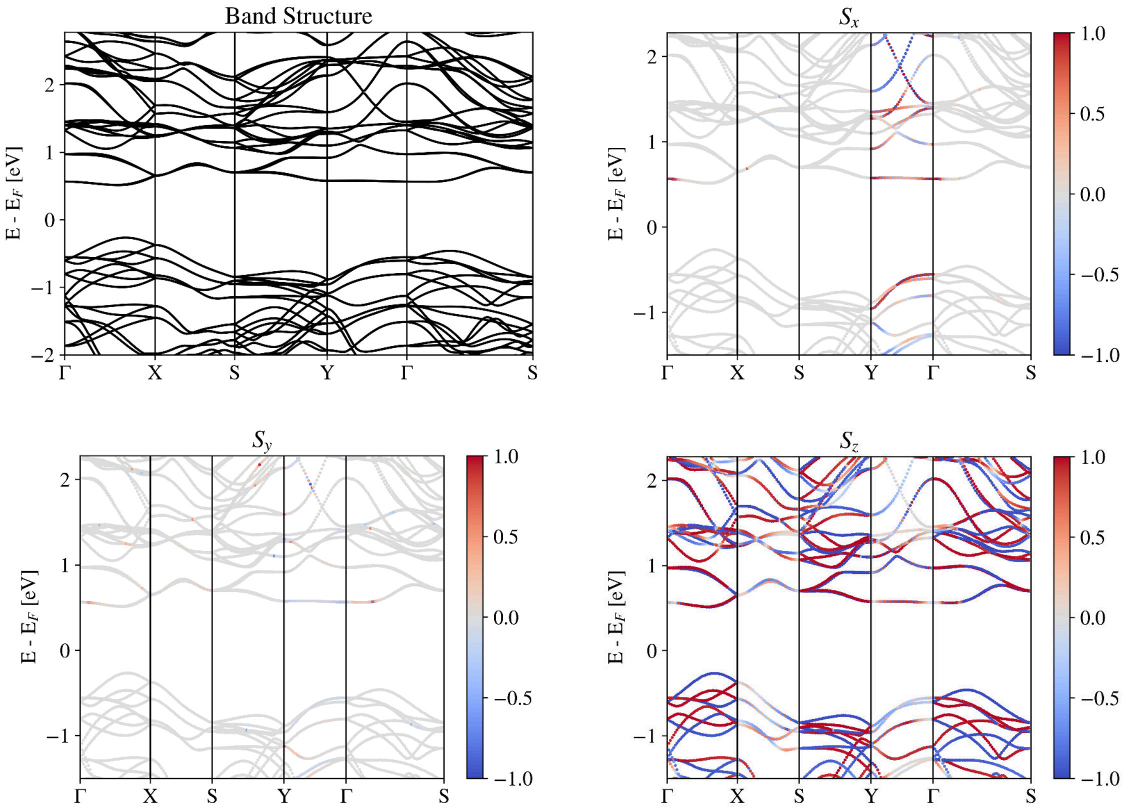


Figure 261: Band structure and spin polarization projections.

## 2.131 Cr2W2Te8-62bb754c4cb2

- **Formula**: Cr2W2Te8
- **Structural Cluster**: ABC4-22
- **Band gap (PBE)**: 0.512 eV
- **Energy above convex hull (C2DB)**: 0.082 eV
- **Space group symbol**: *Pm*
- **Space group number**: 6
- **Polar structure**: True

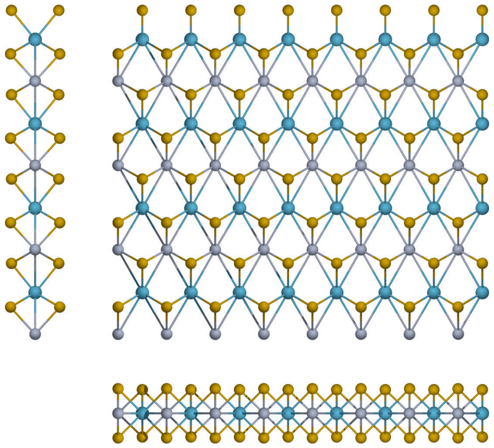

Figure 262: Structure representation

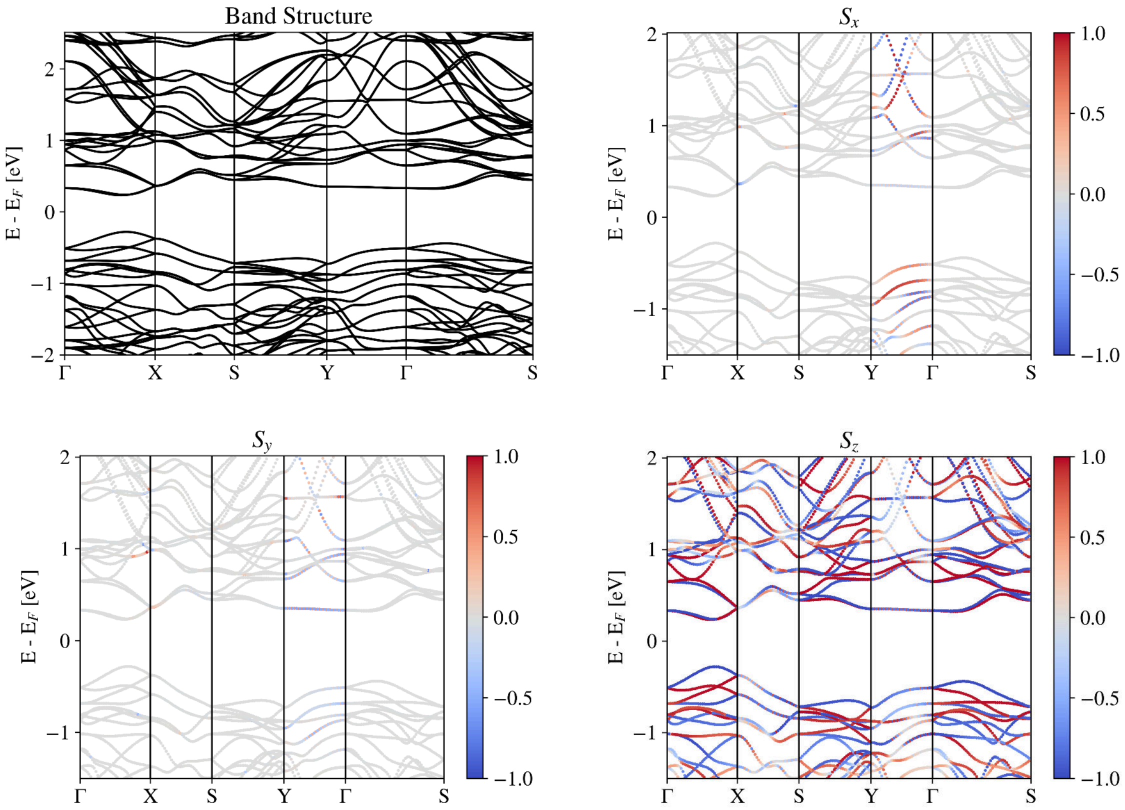


Figure 263: Band structure and spin polarization projections.

## 2.132 CrMo3S8-644f7c1c85c7

- **Formula**: CrMo3S8
- **Structural Cluster**: AB3C8-22
- **Band gap (PBE)**: 1.206 eV
- **Energy above convex hull (C2DB)**: 0.011 eV
- **Space group symbol**: *Pm*
- **Space group number**: 6
- **Polar structure**: True

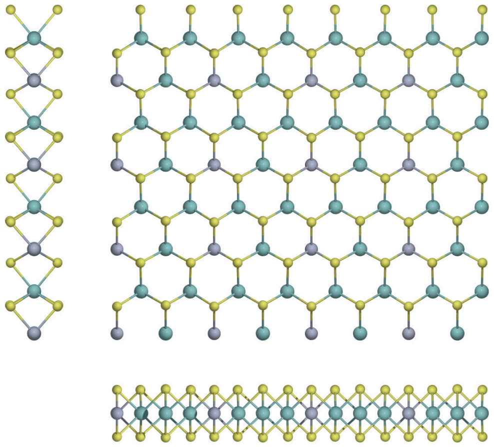

Figure 264: Structure representation

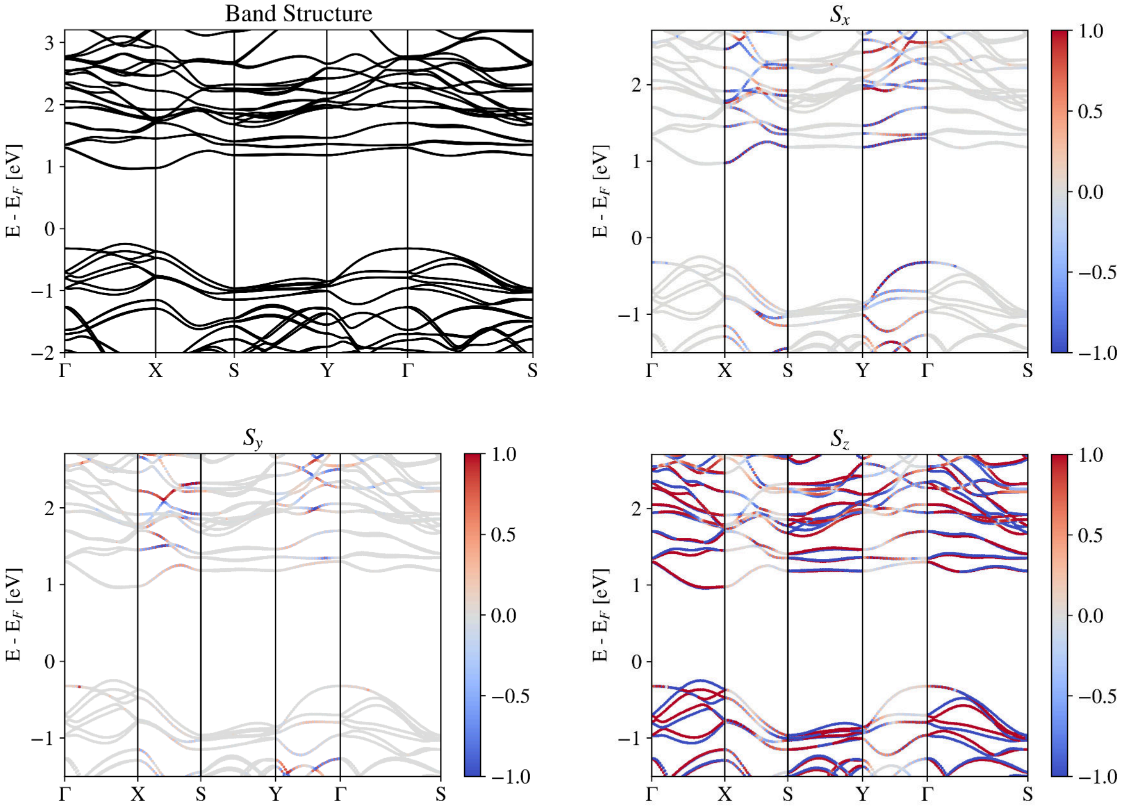


Figure 265: Band structure and spin polarization projections.

## 2.133 CrMo3Se8-a7233837cfe9

- **Formula**: CrMo3Se8
- **Structural Cluster**: AB3C8-22
- **Band gap (PBE)**: 0.971 eV
- **Energy above convex hull (C2DB)**: 0.01 eV
- **Space group symbol**: $P1$
- **Space group number**: 1
- **Polar structure**: True

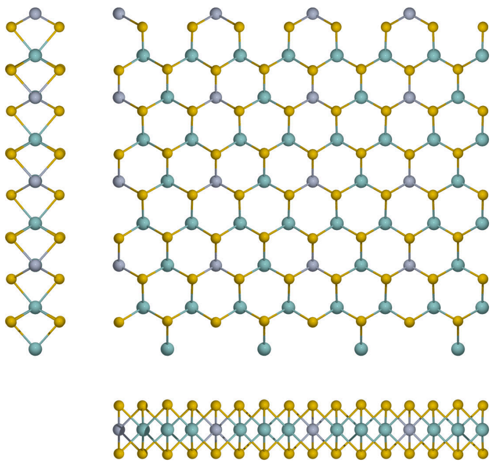

Figure 266: Structure representation

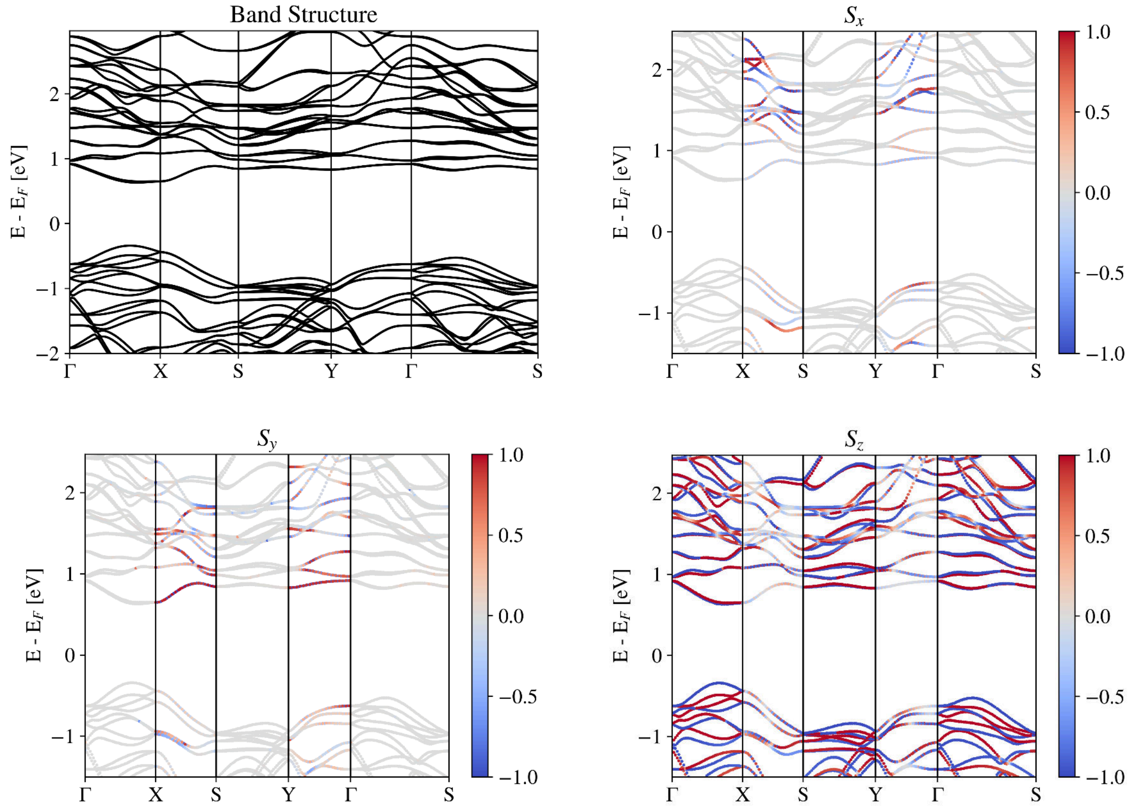


Figure 267: Band structure and spin polarization projections.

## 2.134 CrMo3Te8-159f028a85d0

- **Formula**: CrMo3Te8
- **Structural Cluster**: AB3C8-22
- **Band gap (PBE)**: 0.665 eV
- **Energy above convex hull (C2DB)**: 0.035 eV
- **Space group symbol**: $P1$
- **Space group number**: 1
- **Polar structure**: True

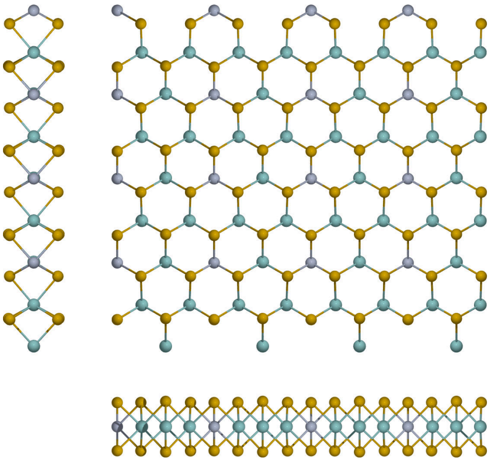

Figure 268: Structure representation

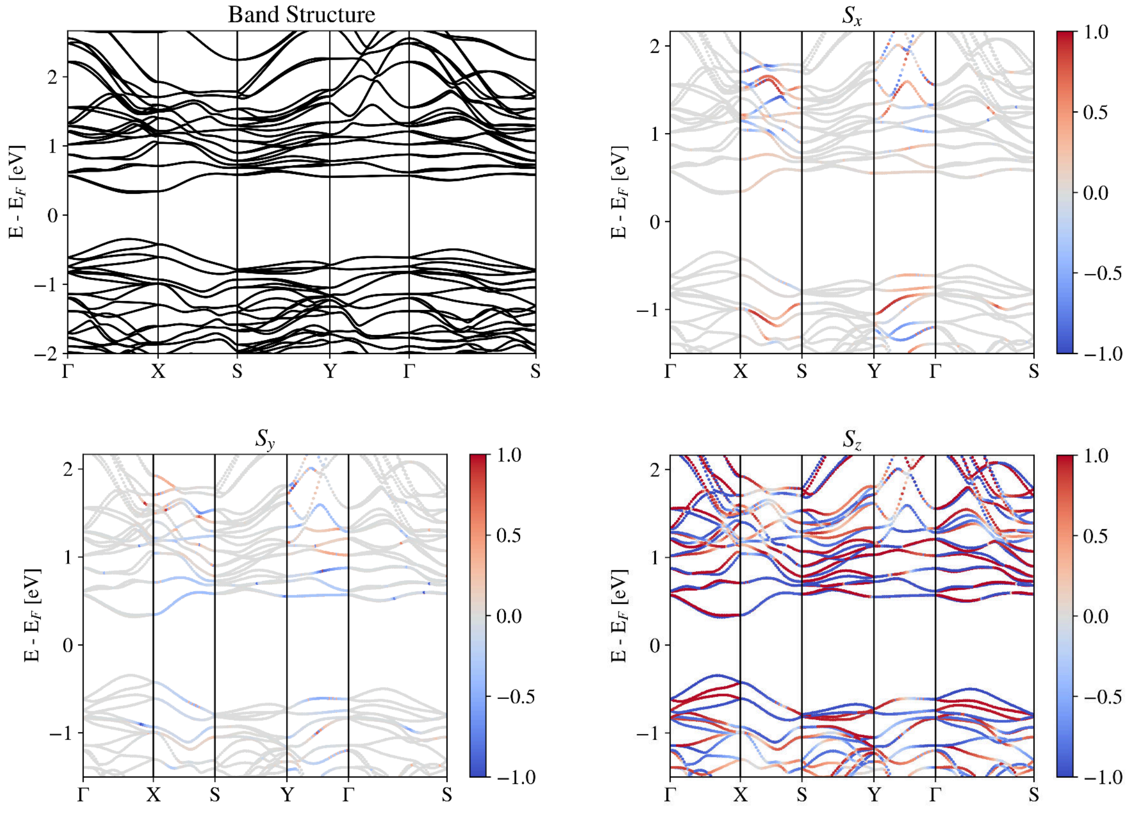


Figure 269: Band structure and spin polarization projections.

## 2.135 CrO2-2433700165bb

- **Formula**: CrO2
- **Structural Cluster**: AB2-4
- **Band gap (PBE)**: 0.422 eV
- **Energy above convex hull (C2DB)**: 0.168 eV
- **Space group symbol**: $P\bar{6}m2$
- **Space group number**: 187
- **Polar structure**: False

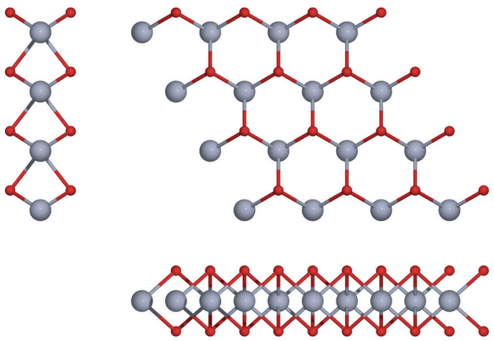

Figure 270: Structure representation

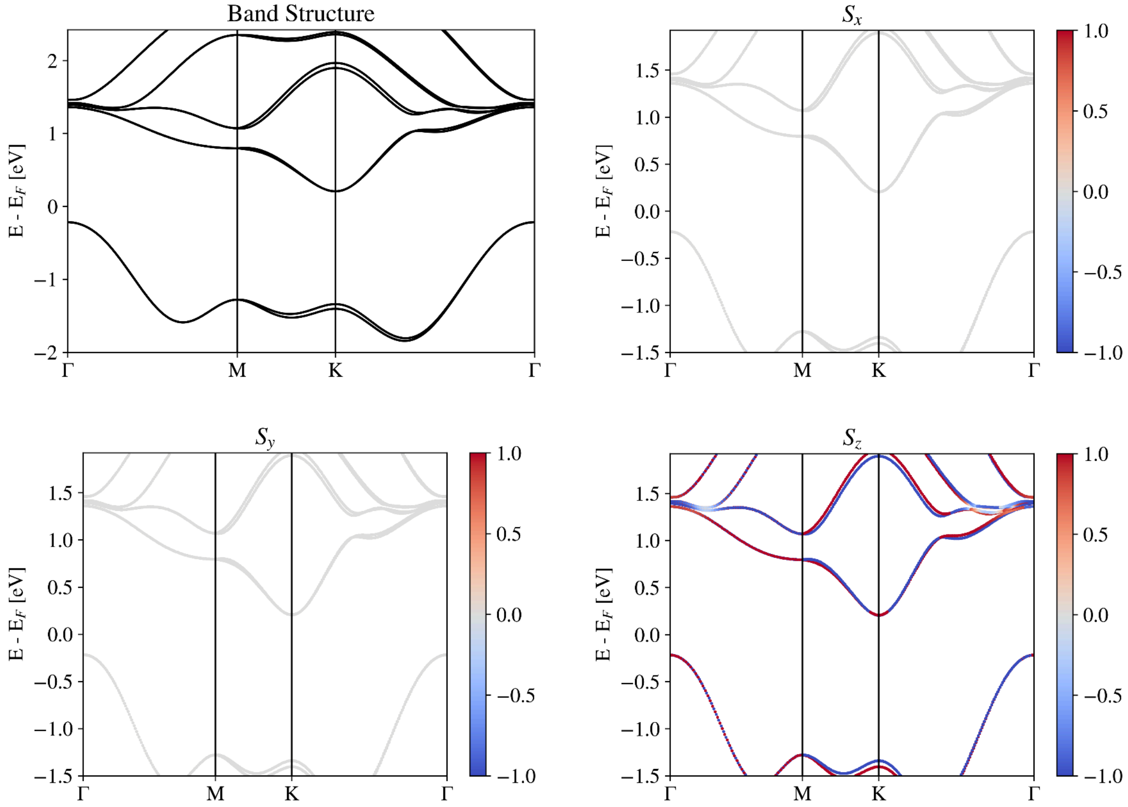


Figure 271: Band structure and spin polarization projections.

## 2.136 CrS2-c5ee5e35d2b4

- **Formula**: CrS2
- **Structural Cluster**: AB2-4
- **Band gap (PBE)**: 0.899 eV
- **Energy above convex hull (C2DB)**: 0.0 eV
- **Space group symbol**: $P\bar{6}m2$
- **Space group number**: 187
- **Polar structure**: False

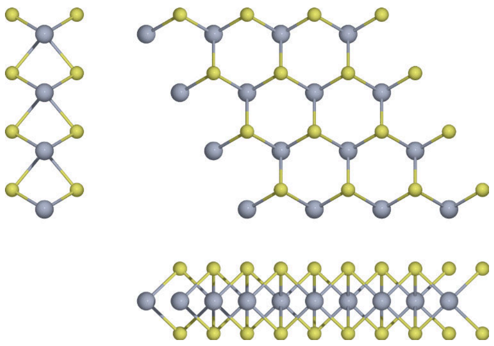

Figure 272: Structure representation

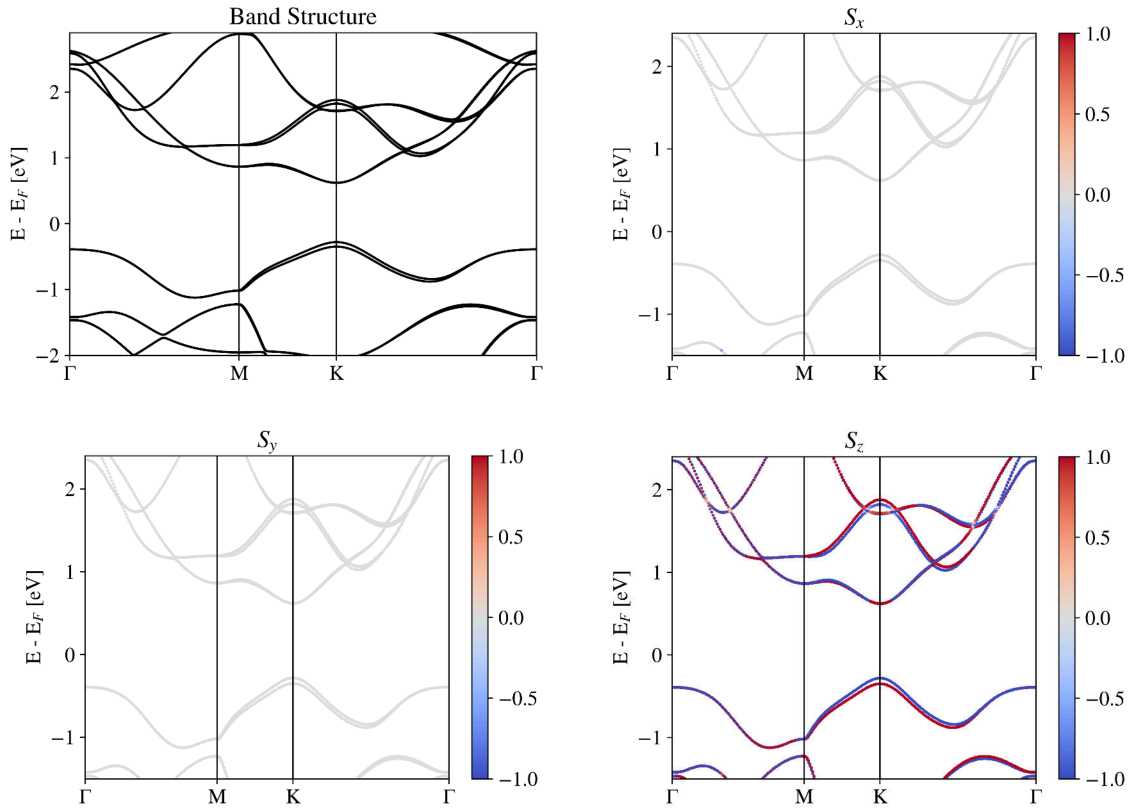


Figure 273: Band structure and spin polarization projections.

## 2.137 CrSSe-09e1e5ef94cb

- **Formula**: CrSSe
- **Structural Cluster**: ABC-4
- **Band gap (PBE)**: 0.802 eV
- **Energy above convex hull (C2DB)**: 0.01 eV
- **Space group symbol**: $P3m1$
- **Space group number**: 156
- **Polar structure**: True

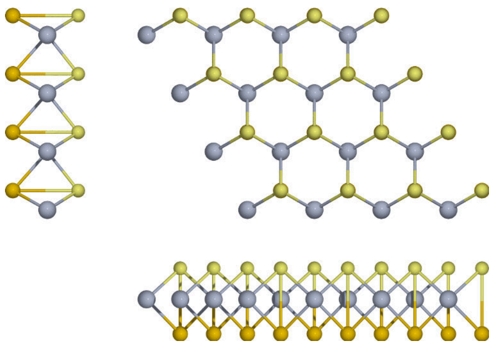

Figure 274: Structure representation

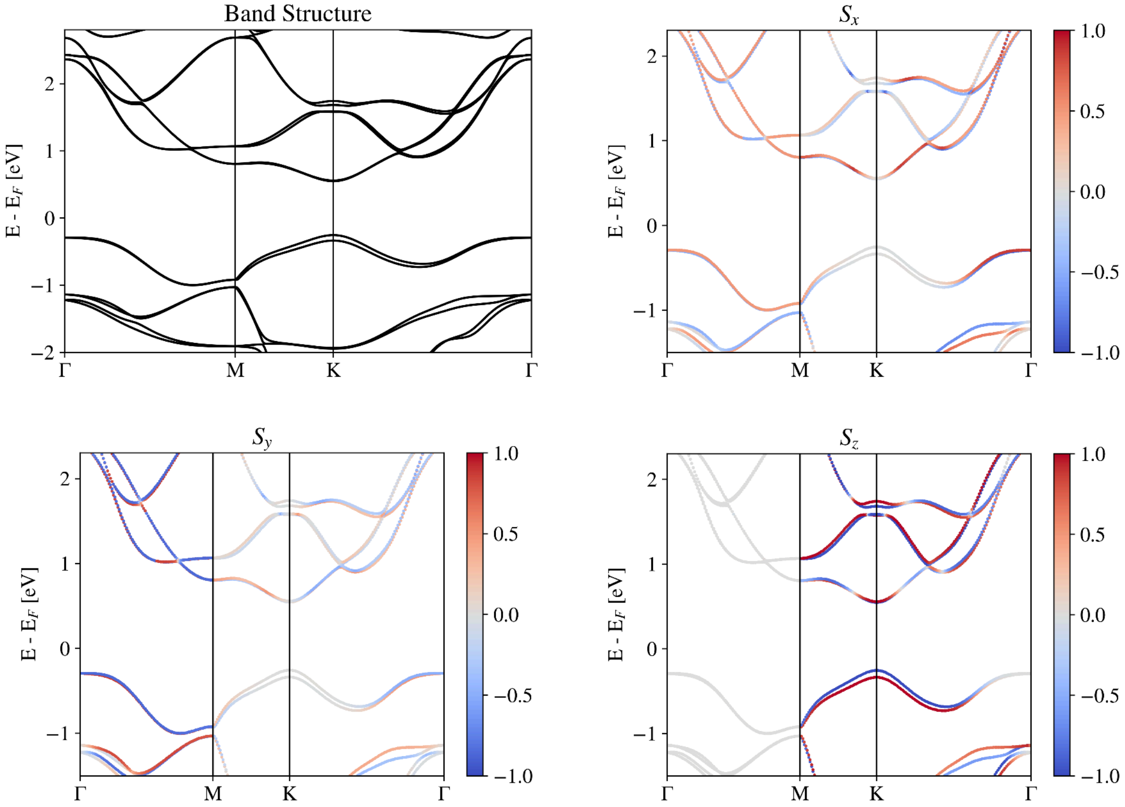


Figure 275: Band structure and spin polarization projections.

## 2.138 CrSTe-8a0864d30ce1

- **Formula**: CrSTe
- **Structural Cluster**: ABC-4
- **Band gap (PBE)**: 0.288 eV
- **Energy above convex hull (C2DB)**: 0.161 eV
- **Space group symbol**: $P3m1$
- **Space group number**: 156
- **Polar structure**: True

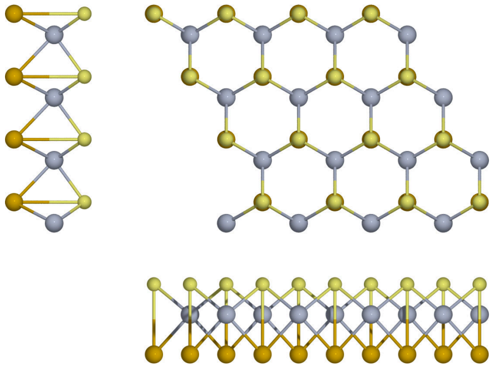

Figure 276: Structure representation

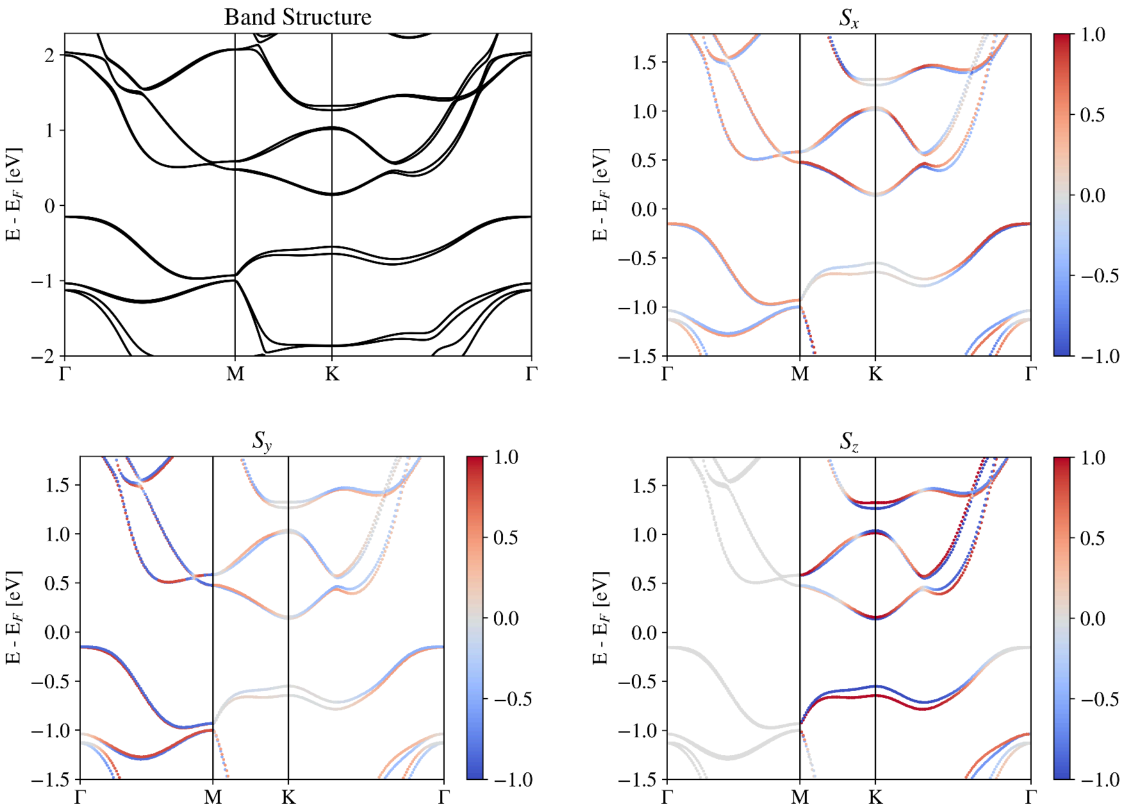


Figure 277: Band structure and spin polarization projections.

## 2.139 CrSe2-9a6ff6a3c41a

- **Formula**: CrSe2
- **Structural Cluster**: AB2-4
- **Band gap (PBE)**: 0.703 eV
- **Energy above convex hull (C2DB)**: 0.0 eV
- **Space group symbol**: $P\bar{6}m2$
- **Space group number**: 187
- **Polar structure**: False

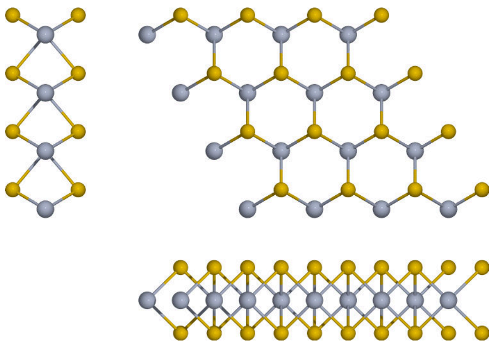

Figure 278: Structure representation

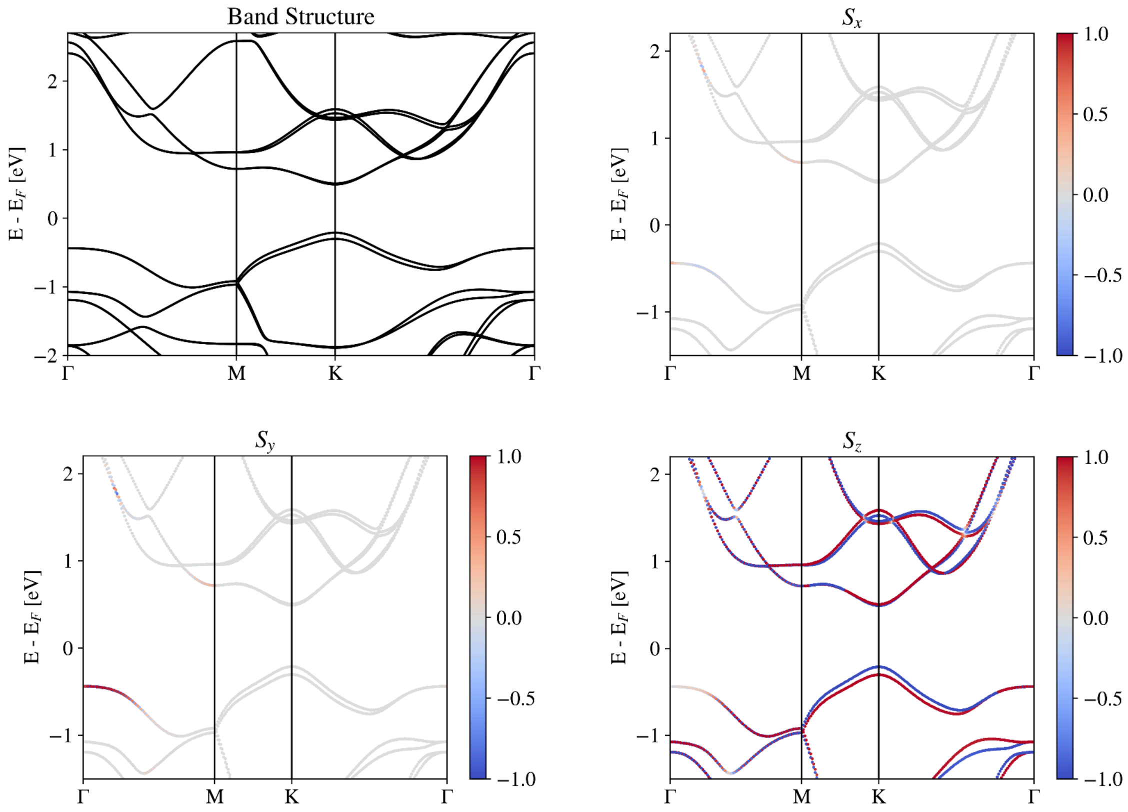


Figure 279: Band structure and spin polarization projections.

## 2.140 CrSeTe-5d9d3ded04de

- **Formula**: CrSeTe
- **Structural Cluster**: ABC-4
- **Band gap (PBE)**: 0.59 eV
- **Energy above convex hull (C2DB)**: 0.111 eV
- **Space group symbol**: $P3m1$
- **Space group number**: 156
- **Polar structure**: True

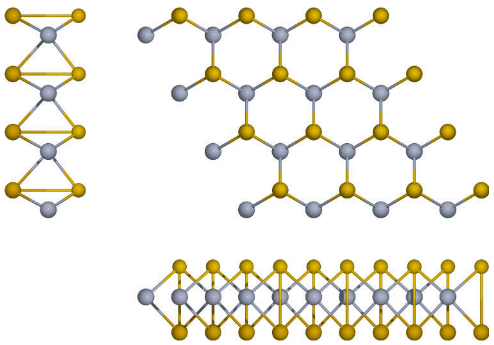

Figure 280: Structure representation

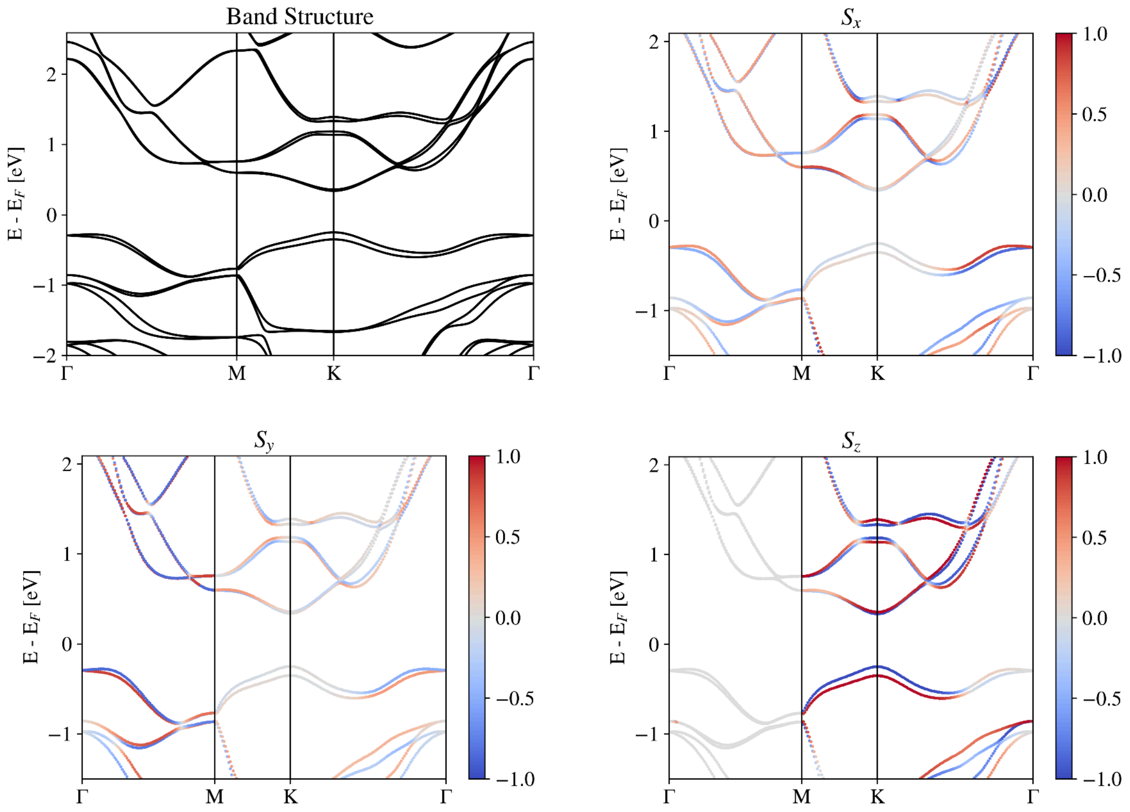


Figure 281: Band structure and spin polarization projections.

## 2.141 CrTe2-c31911a1b3f9

- **Formula**: CrTe2
- **Structural Cluster**: AB2-4
- **Band gap (PBE)**: 0.468 eV
- **Energy above convex hull (C2DB)**: 0.108 eV
- **Space group symbol**: $P\bar{6}m2$
- **Space group number**: 187
- **Polar structure**: False

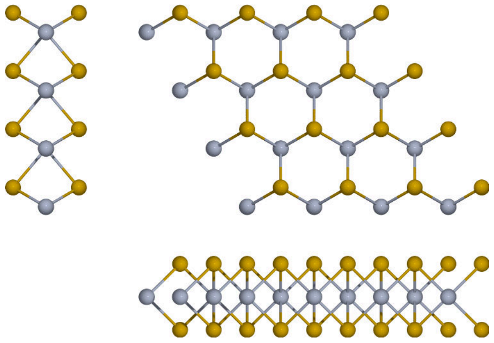

Figure 282: Structure representation

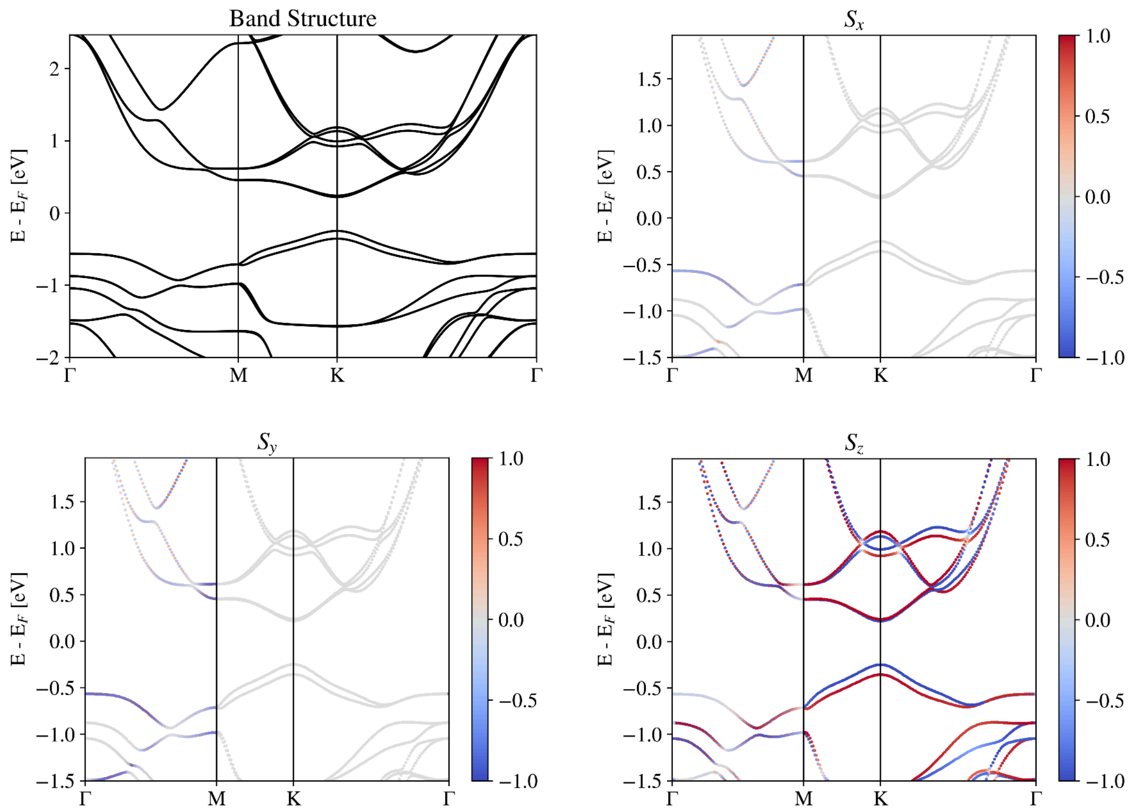


Figure 283: Band structure and spin polarization projections.

## 2.142 CrW3S8-a9f87eba4b96

- **Formula**: CrW3S8
- **Structural Cluster**: AB3C8-22
- **Band gap (PBE)**: 1.144 eV
- **Energy above convex hull (C2DB)**: 0.009 eV
- **Space group symbol**: *Pm*
- **Space group number**: 6
- **Polar structure**: True

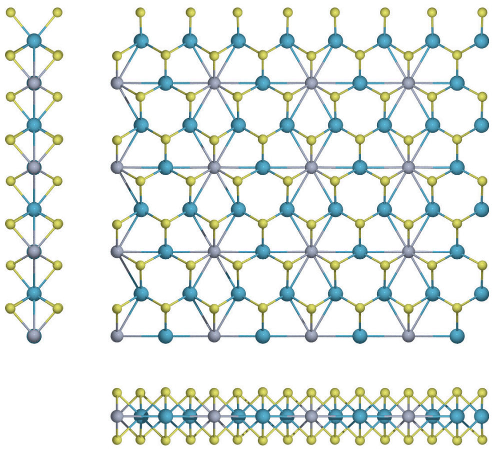

Figure 284: Structure representation

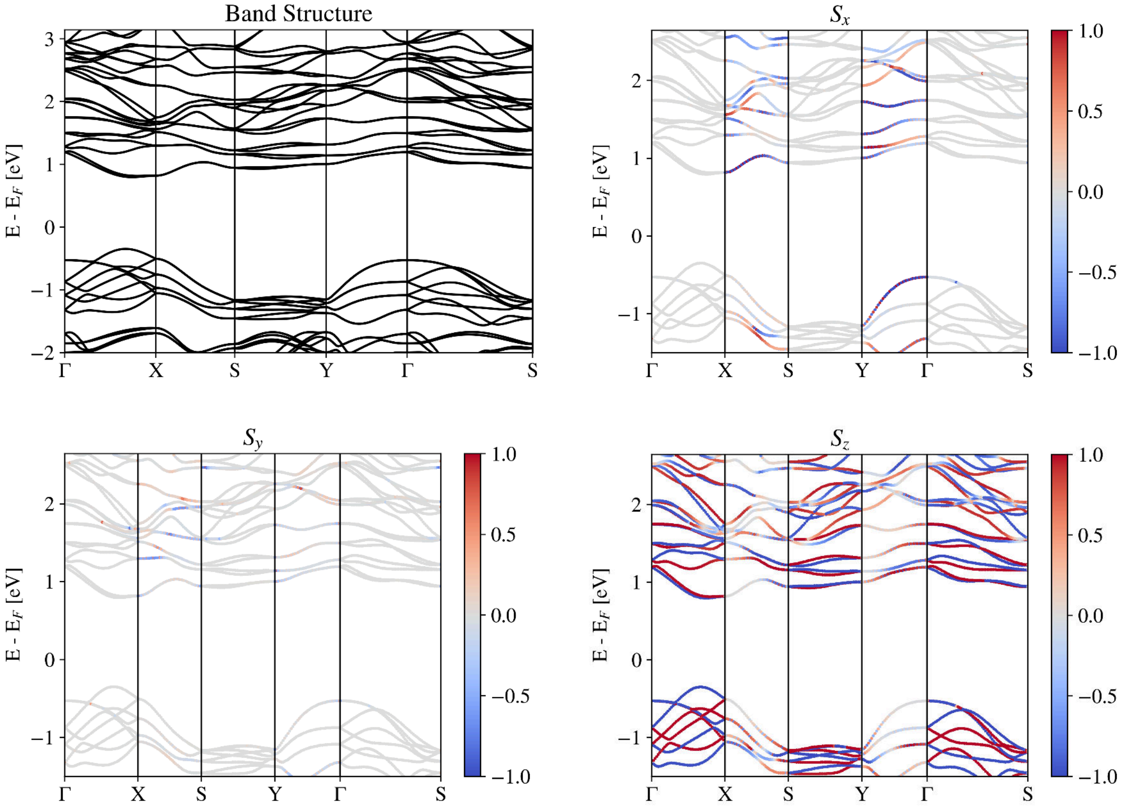


Figure 285: Band structure and spin polarization projections.

## 2.143 CrW3Se8-0b7696e1f4c9

- **Formula**: CrW3Se8
- **Structural Cluster**: AB3C8-22
- **Band gap (PBE)**: 0.902 eV
- **Energy above convex hull (C2DB)**: 0.01 eV
- **Space group symbol**: $P1$
- **Space group number**: 1
- **Polar structure**: True

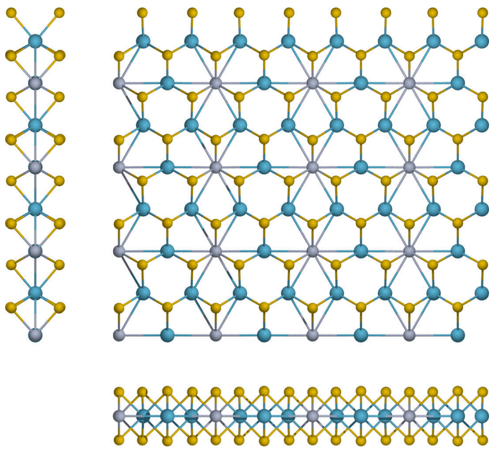

Figure 286: Structure representation

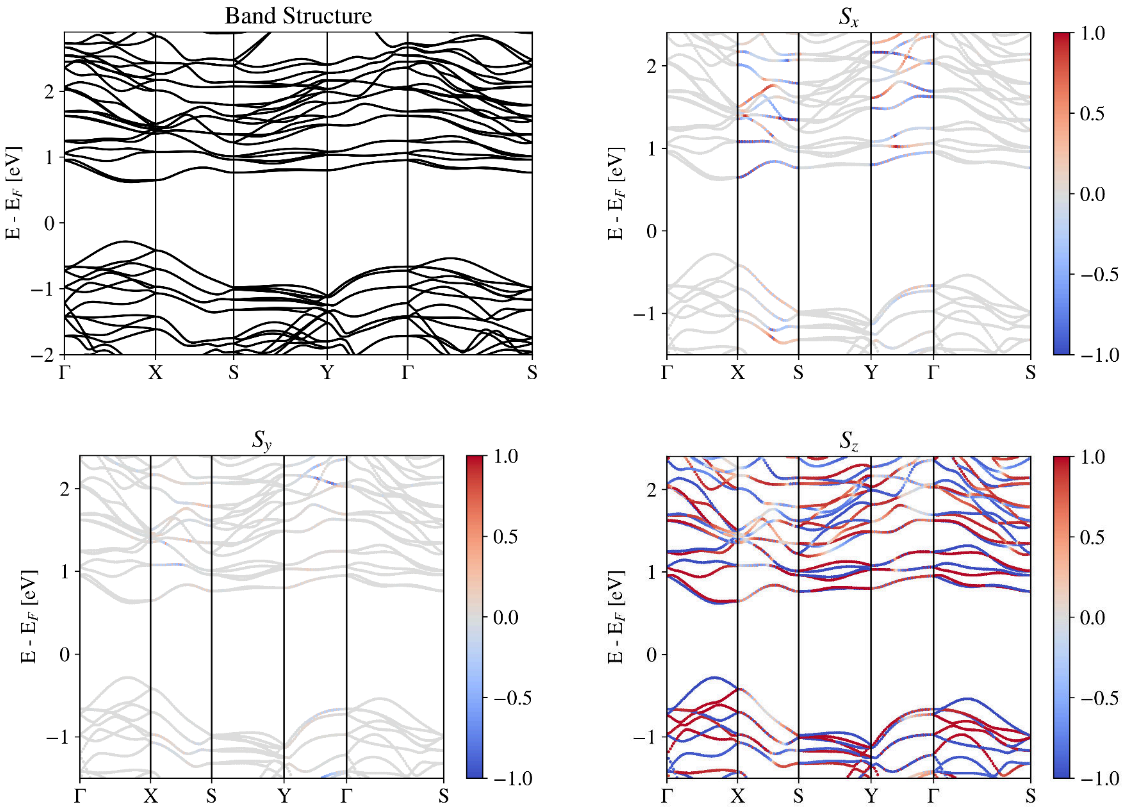


Figure 287: Band structure and spin polarization projections.

## 2.144 CrW3Te8-eef072f845ce

- **Formula**: CrW3Te8
- **Structural Cluster**: AB3C8-22
- **Band gap (PBE)**: 0.563 eV
- **Energy above convex hull (C2DB)**: 0.056 eV
- **Space group symbol**: $P1$
- **Space group number**: 1
- **Polar structure**: True

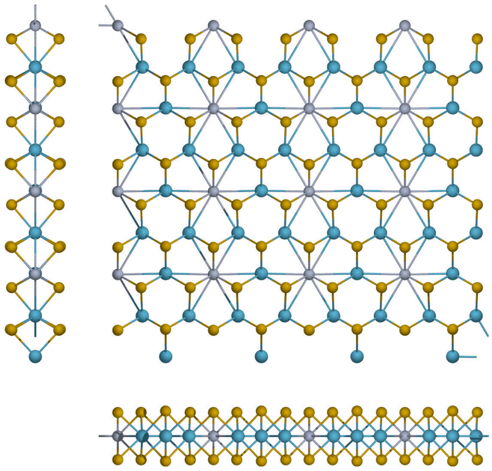

Figure 288: Structure representation

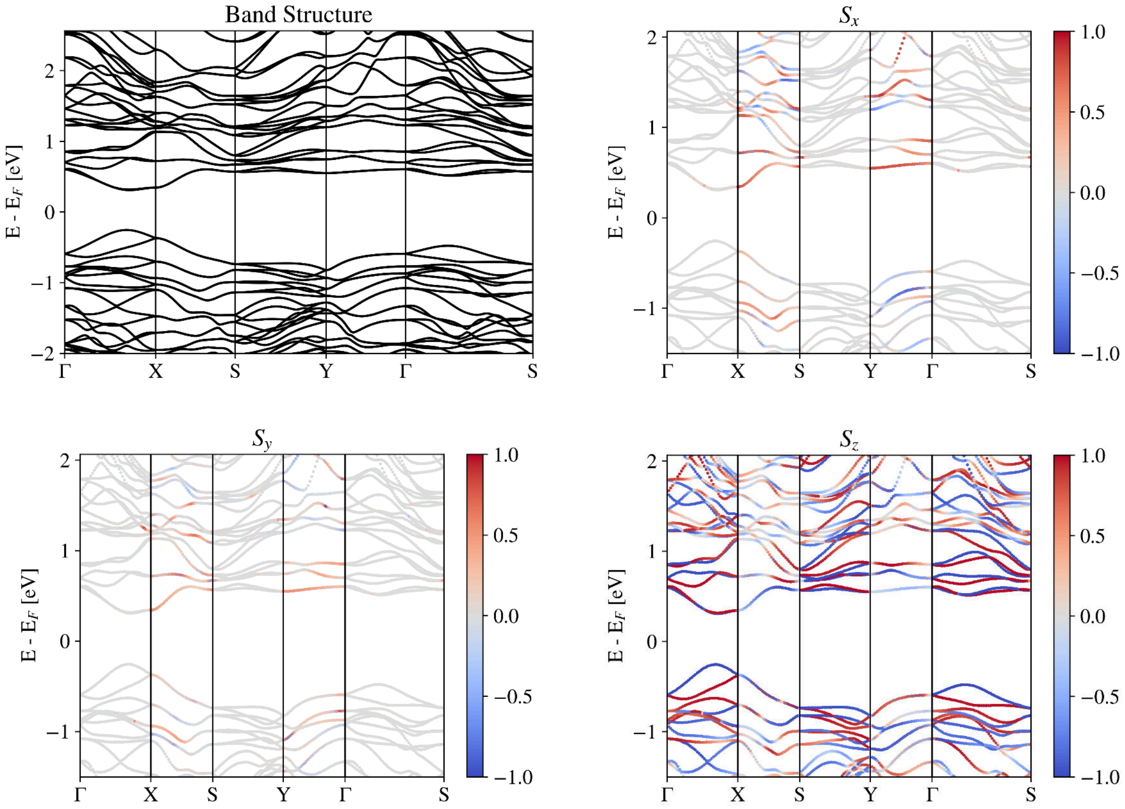


Figure 289: Band structure and spin polarization projections.

## 2.145 Cu2I2-ac7333c6ab94

- **Formula**: Cu2I2
- **Structural Cluster**: AB-5
- **Band gap (PBE)**: 1.809 eV
- **Energy above convex hull (C2DB)**: 0.0 eV
- **Space group symbol**: $P3m1$
- **Space group number**: 156
- **Polar structure**: True

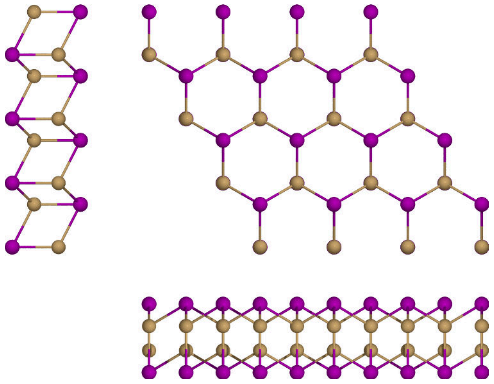

Figure 290: Structure representation

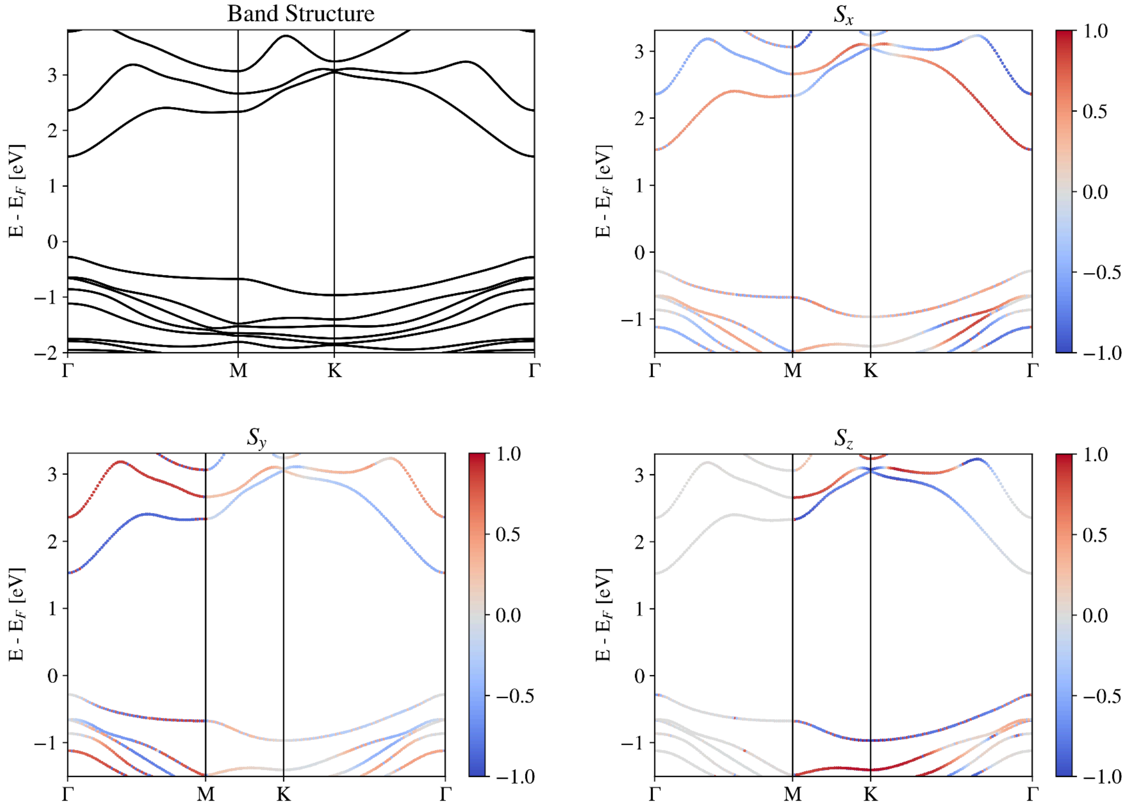


Figure 291: Band structure and spin polarization projections.

## 2.146 Cu2S2-ed5f6e977271

- **Formula**: Cu2S2
- **Structural Cluster**: AB-0
- **Band gap (PBE)**: 0.62 eV
- **Energy above convex hull (C2DB)**: 0.127 eV
- **Space group symbol**: *Pm*
- **Space group number**: 6
- **Polar structure**: True

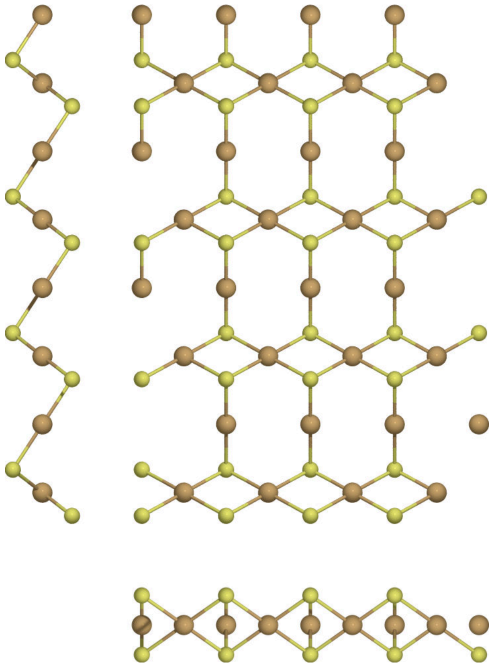

Figure 292: Structure representation

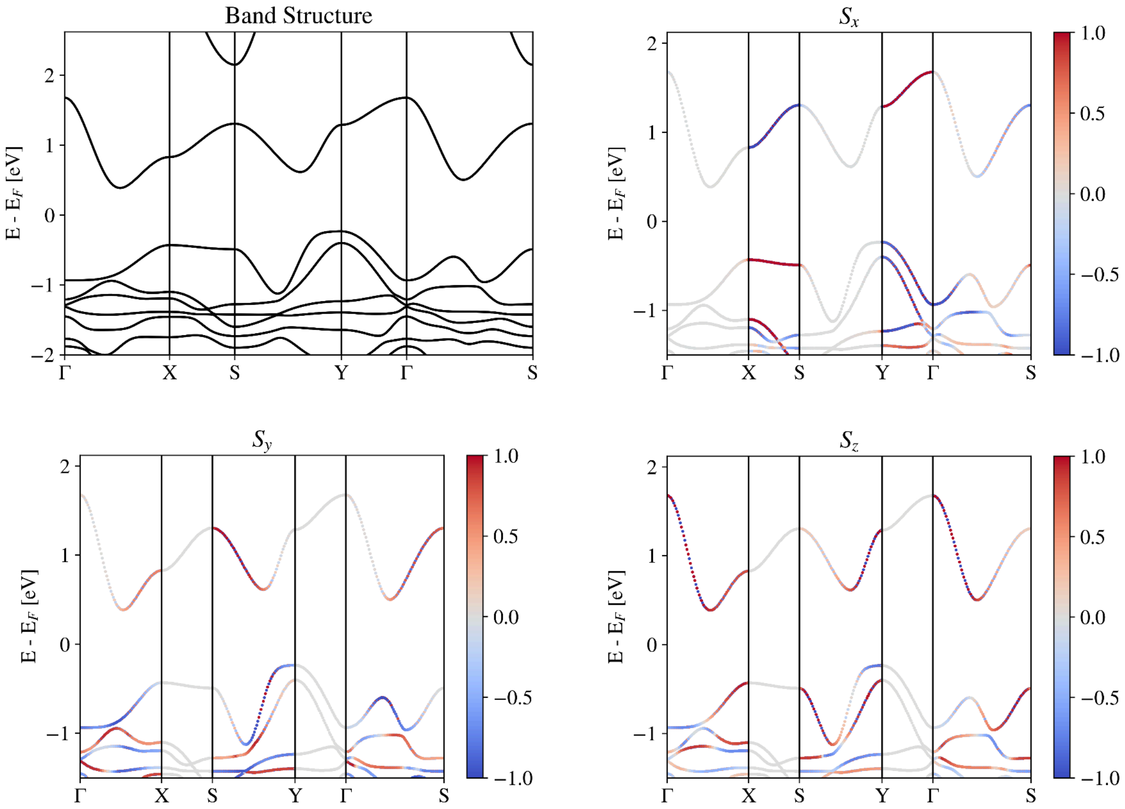


Figure 293: Band structure and spin polarization projections.

## 2.147 Fe2P2S6-91367f7f5dce

- **Formula**: Fe2P2S6
- **Structural Cluster**: ABC3-16
- **Band gap (PBE)**: 0.306 eV
- **Energy above convex hull (C2DB)**: 0.145 eV
- **Space group symbol**: $P1$
- **Space group number**: 1
- **Polar structure**: True

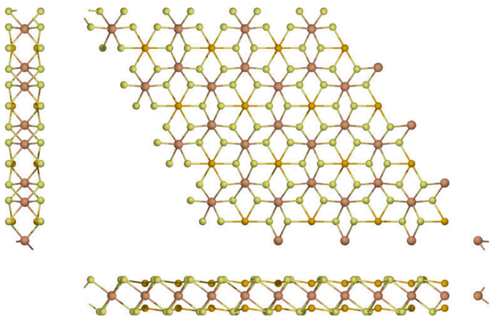

Figure 294: Structure representation

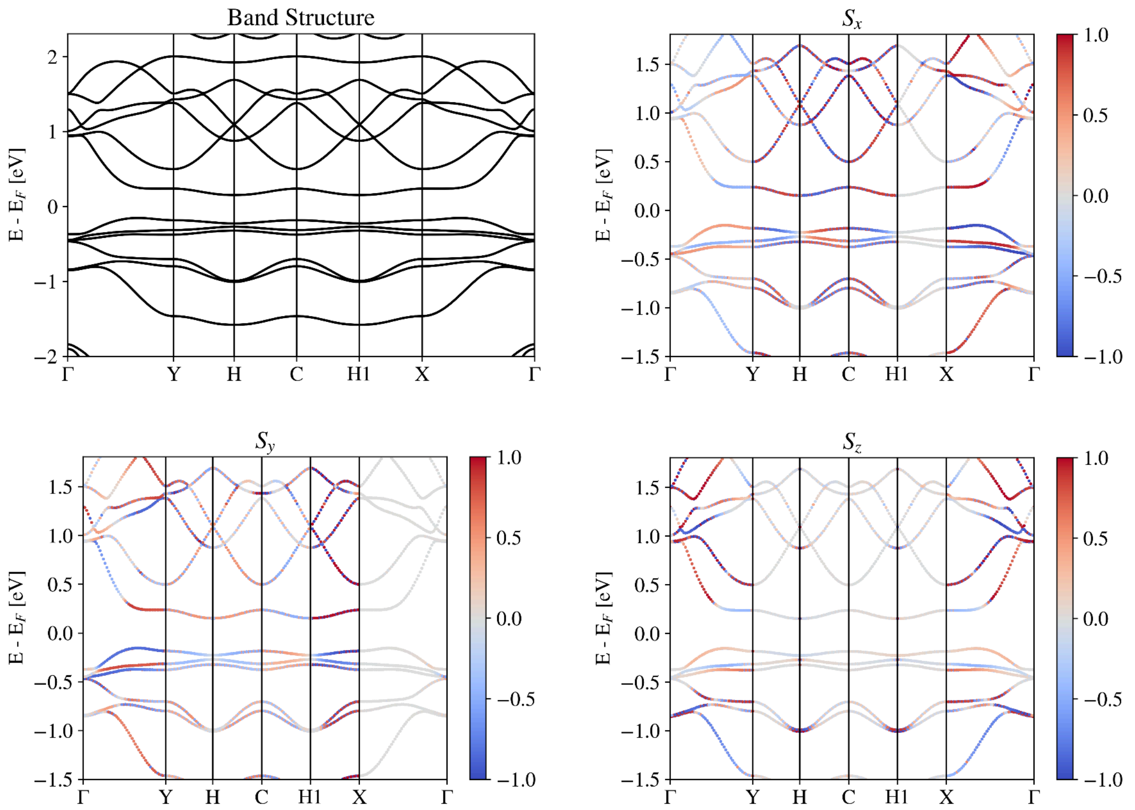


Figure 295: Band structure and spin polarization projections.

## 2.148 FeF2-c67fbd722c30

- **Formula**: FeF2
- **Structural Cluster**: AB2-12
- **Band gap (PBE)**: 0.051 eV
- **Energy above convex hull (C2DB)**: 0.719 eV
- **Space group symbol**: $P\bar{4}m2$
- **Space group number**: 115
- **Polar structure**: False

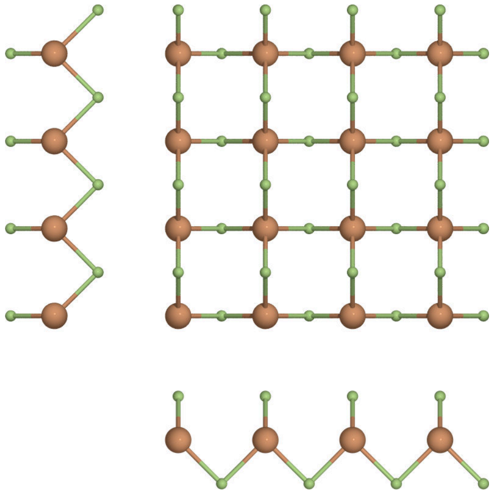

Figure 296: Structure representation

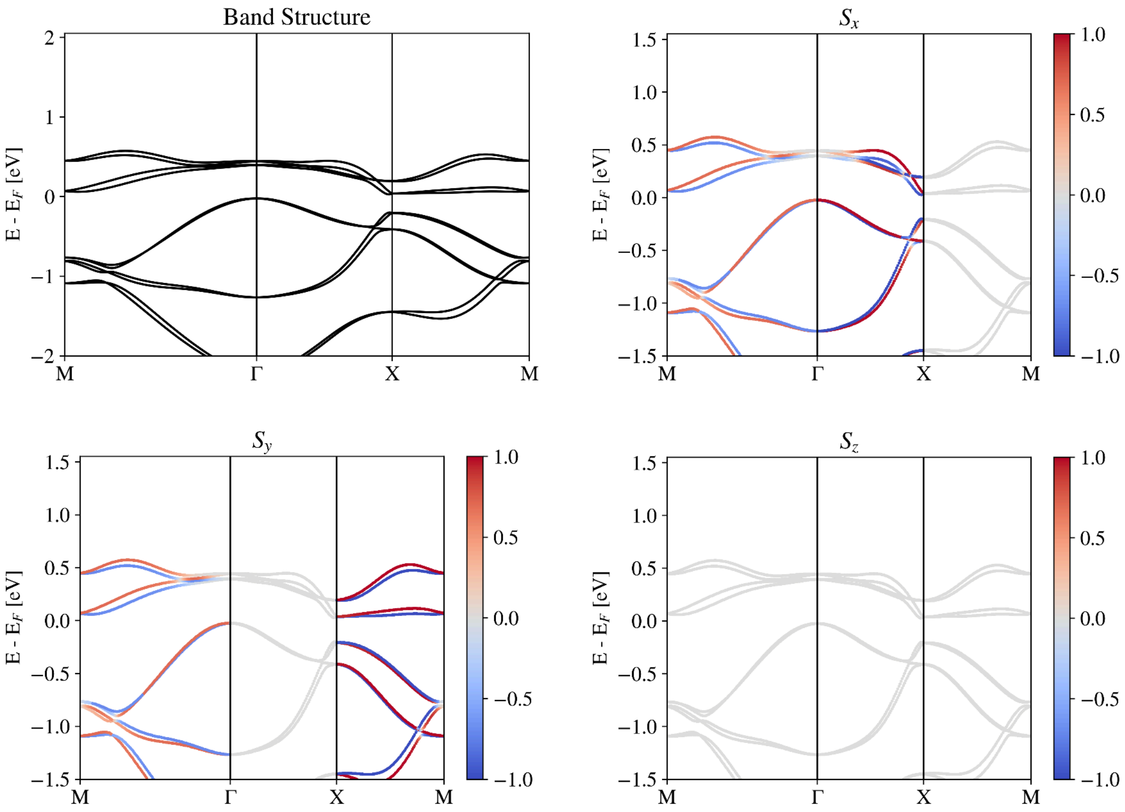


Figure 297: Band structure and spin polarization projections.

## 2.149 Ga2Br6-e9e5bee778b6

- **Formula**: Ga2Br6
- **Structural Cluster**: AB3-19
- **Band gap (PBE)**: 1.374 eV
- **Energy above convex hull (C2DB)**: 0.235 eV
- **Space group symbol**: $P\bar{6}2m$
- **Space group number**: 189
- **Polar structure**: False

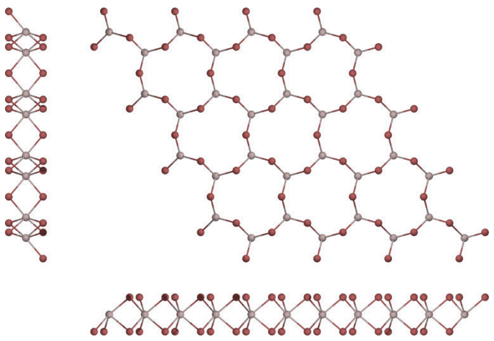

Figure 298: Structure representation

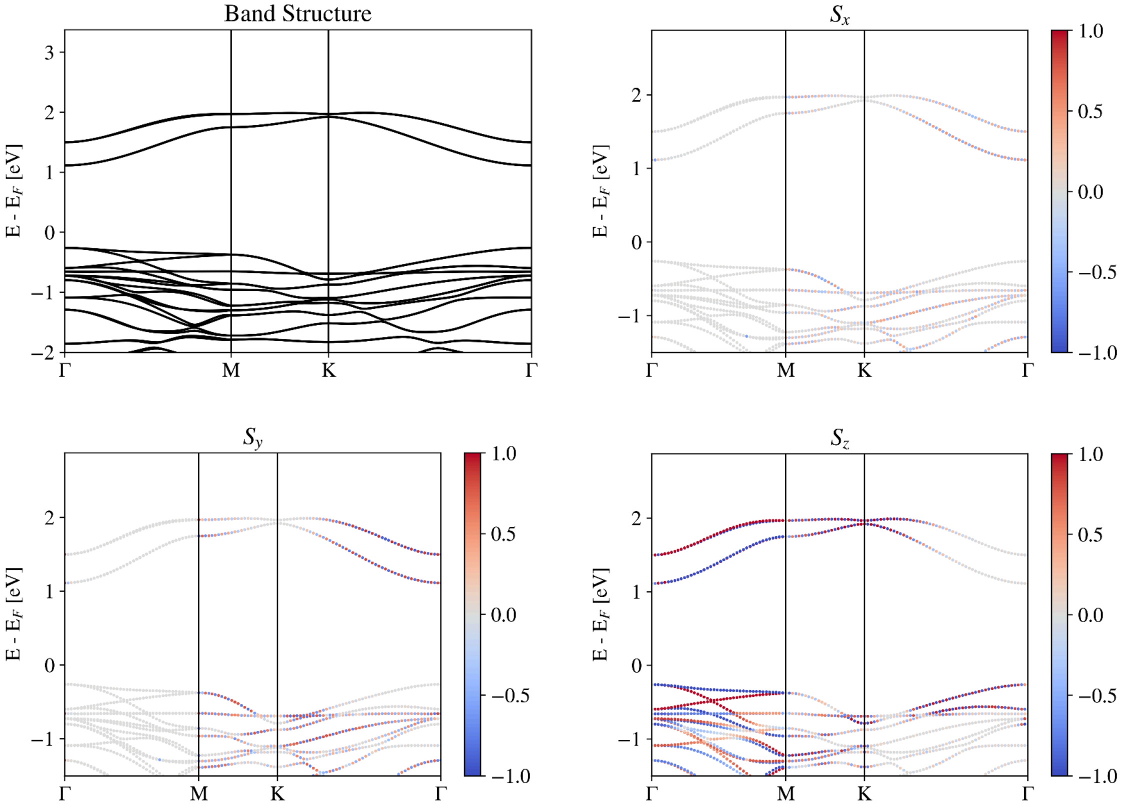


Figure 299: Band structure and spin polarization projections.

## 2.150 Ga2Cl6-c306553fa81a

- **Formula**: Ga2Cl6
- **Structural Cluster**: AB3-19
- **Band gap (PBE)**: 2.552 eV
- **Energy above convex hull (C2DB)**: 0.174 eV
- **Space group symbol**: $P\bar{6}2m$
- **Space group number**: 189
- **Polar structure**: False

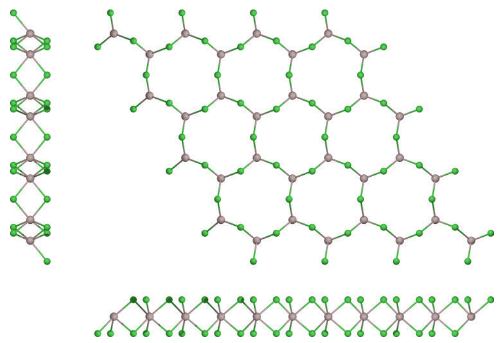

Figure 300: Structure representation

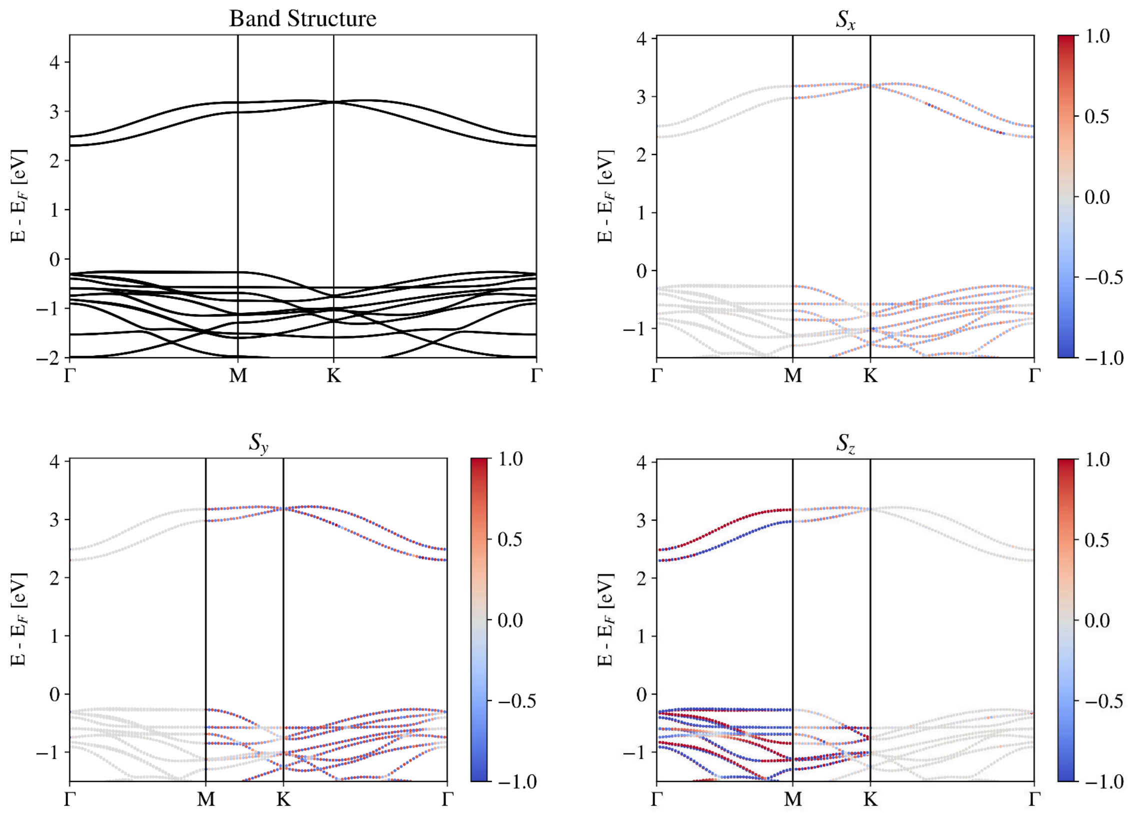


Figure 301: Band structure and spin polarization projections.

## 2.151 Ga2O2-16c96094d1a0

- **Formula**: Ga2O2
- **Structural Cluster**: AB-5
- **Band gap (PBE)**: 1.48 eV
- **Energy above convex hull (C2DB)**: 0.06 eV
- **Space group symbol**: $P\bar{6}m2$
- **Space group number**: 187
- **Polar structure**: False

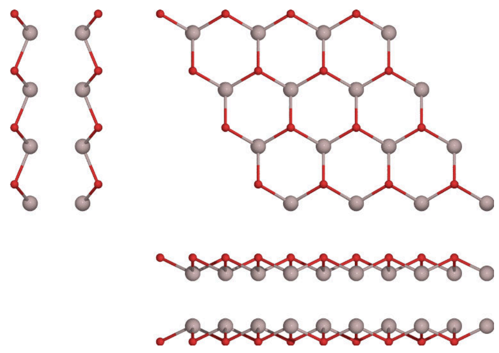

Figure 302: Structure representation

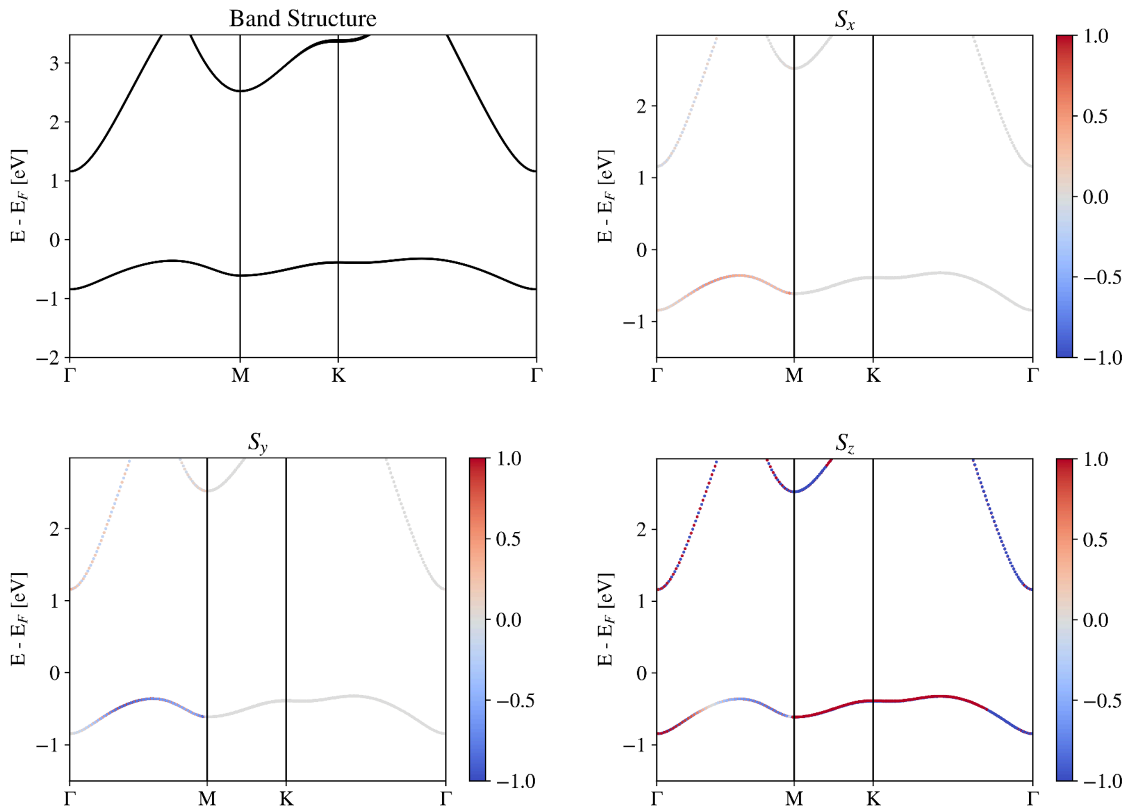


Figure 303: Band structure and spin polarization projections.

## 2.152 Ga2P2Te6-4cb4ea247ef4

- **Formula**: Ga2P2Te6
- **Structural Cluster**: ABC3-16
- **Band gap (PBE)**: 0.314 eV
- **Energy above convex hull (C2DB)**: 0.173 eV
- **Space group symbol**: $P1$
- **Space group number**: 1
- **Polar structure**: True

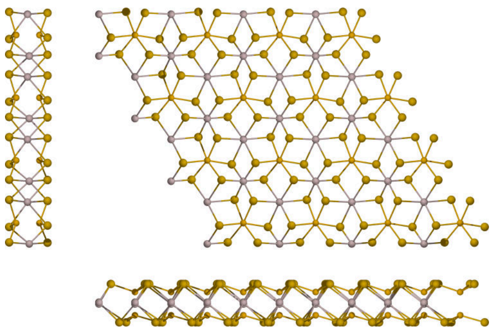

Figure 304: Structure representation

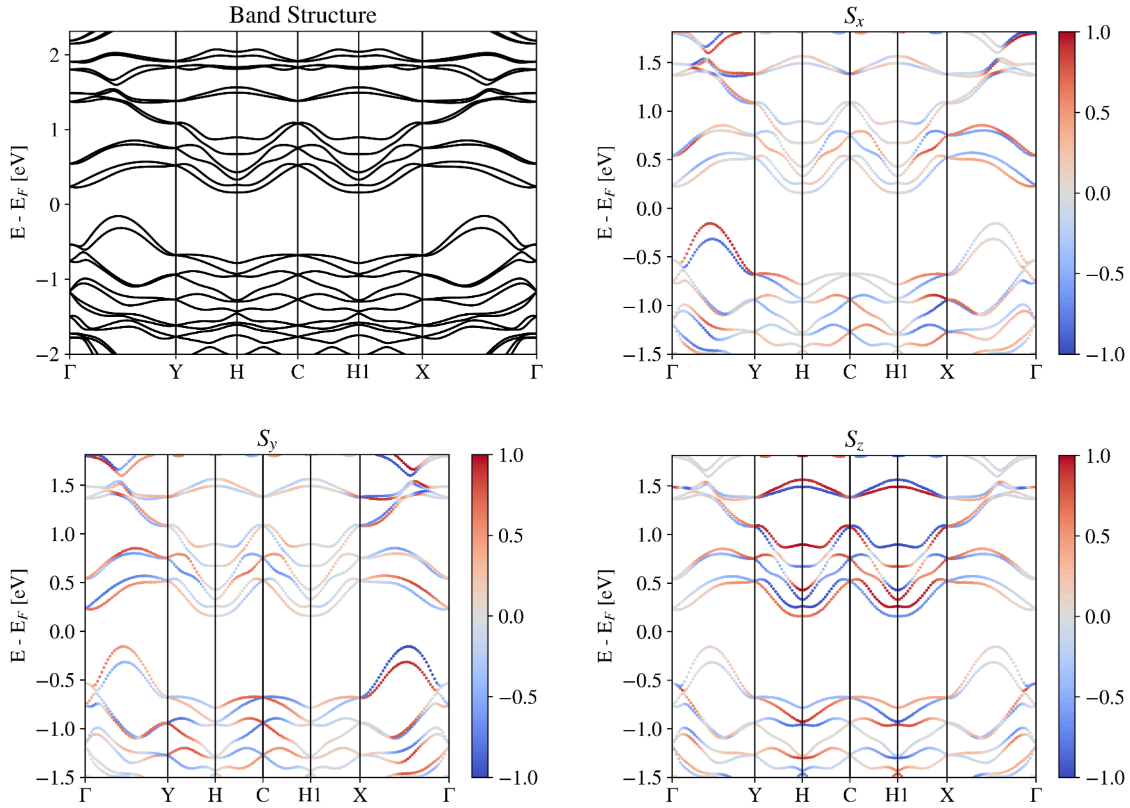


Figure 305: Band structure and spin polarization projections.

## 2.153 Ga2S2-ac002f4ce724

- **Formula**: Ga2S2
- **Structural Cluster**: AB-5
- **Band gap (PBE)**: 2.305 eV
- **Energy above convex hull (C2DB)**: 0.0 eV
- **Space group symbol**: $P\bar{6}m2$
- **Space group number**: 187
- **Polar structure**: False

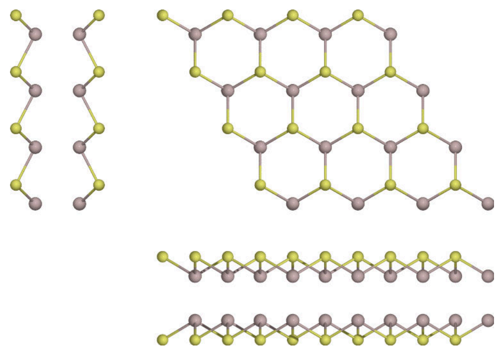

Figure 306: Structure representation

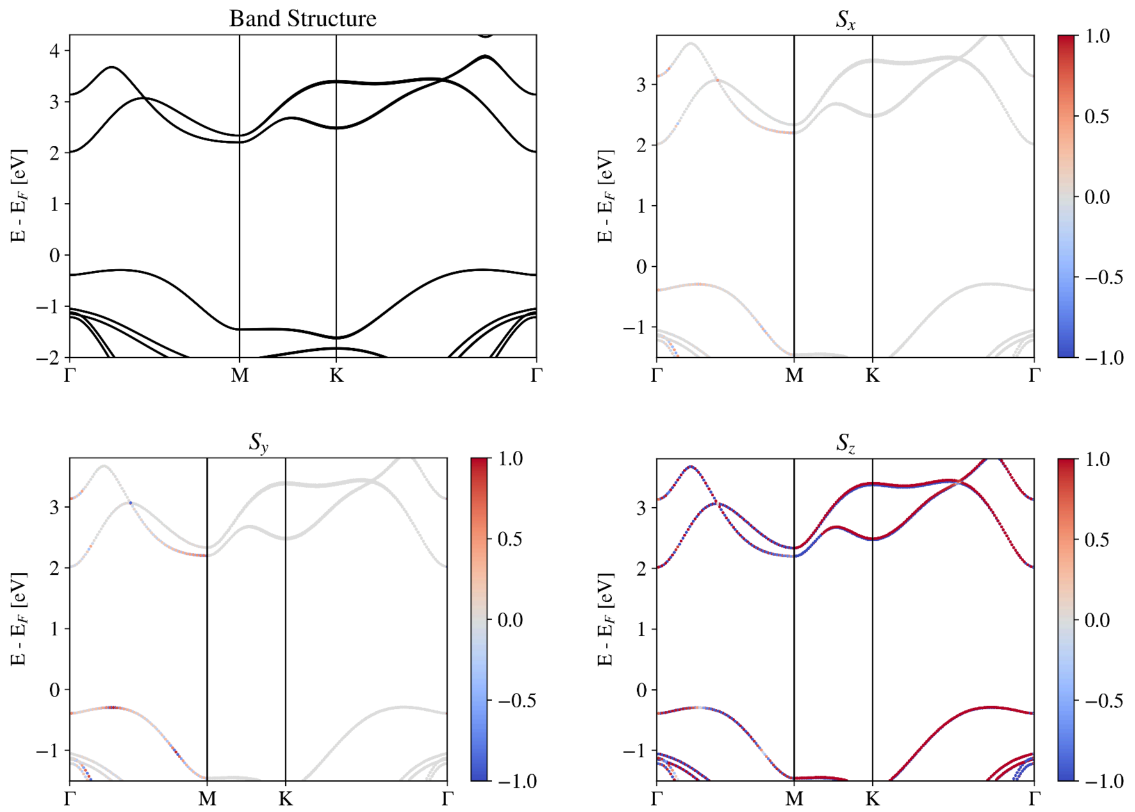


Figure 307: Band structure and spin polarization projections.

## 2.154 Ga2Se2-394e5709a3ac

- **Formula**: Ga2Se2
- **Structural Cluster**: AB-5
- **Band gap (PBE)**: 1.736 eV
- **Energy above convex hull (C2DB)**: 0.0 eV
- **Space group symbol**: $P\bar{6}m2$
- **Space group number**: 187
- **Polar structure**: False

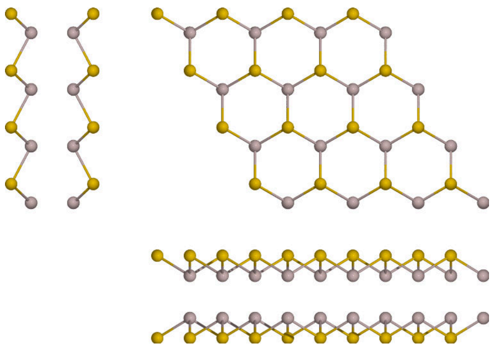

Figure 308: Structure representation

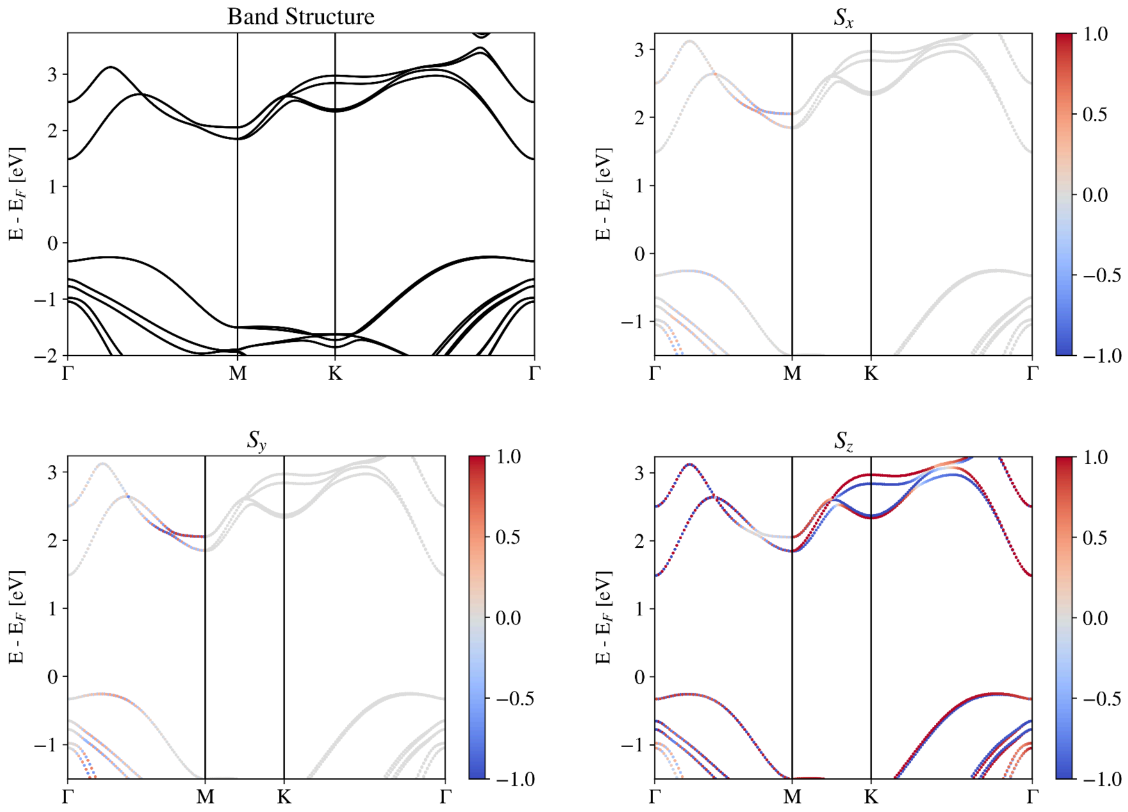


Figure 309: Band structure and spin polarization projections.

## 2.155 Ga2Te2-55c23ca88a05

- **Formula**: Ga2Te2
- **Structural Cluster**: AB-5
- **Band gap (PBE)**: 1.289 eV
- **Energy above convex hull (C2DB)**: 0.002 eV
- **Space group symbol**: $P\bar{6}m2$
- **Space group number**: 187
- **Polar structure**: False

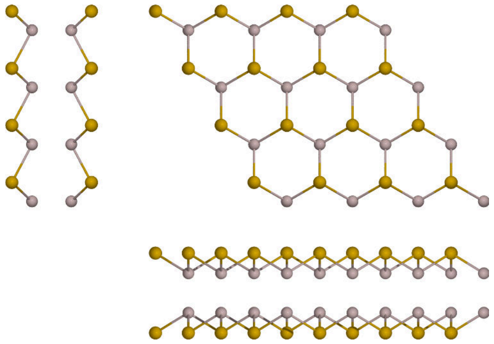

Figure 310: Structure representation

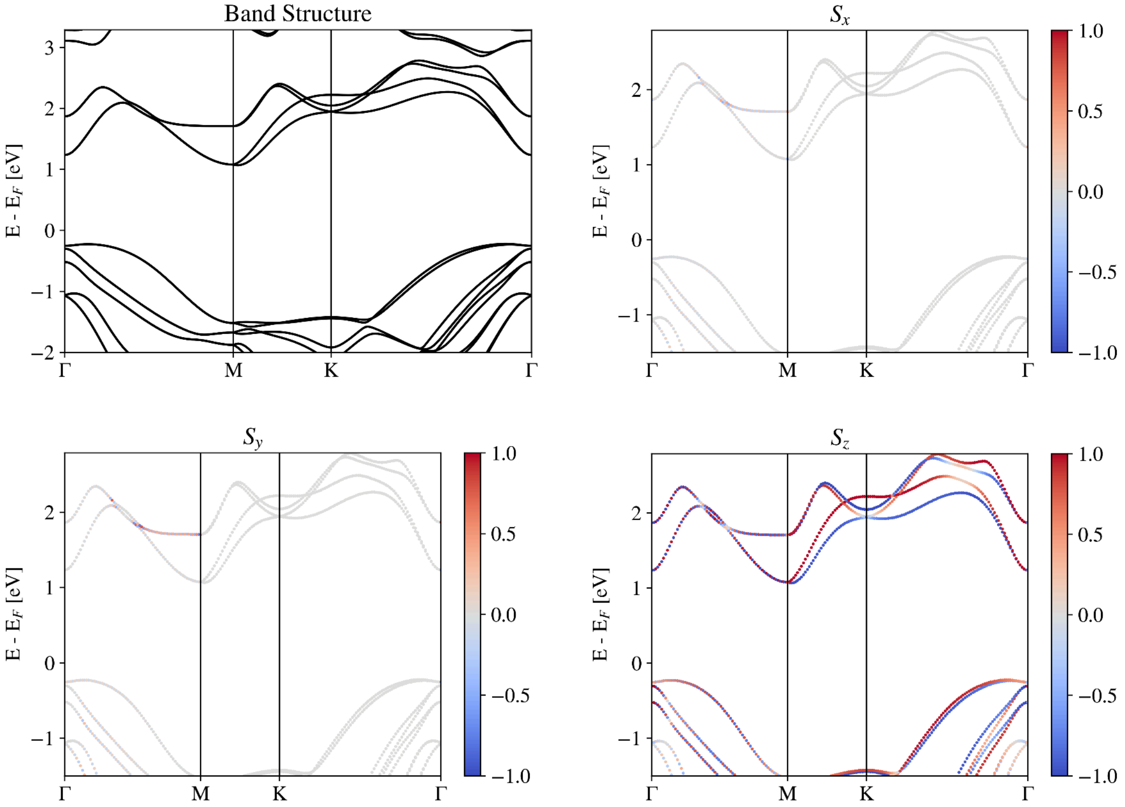


Figure 311: Band structure and spin polarization projections.

## 2.156 GaN-c973e283b023

- **Formula**: GaN
- **Structural Cluster**: AB-2
- **Band gap (PBE)**: 1.818 eV
- **Energy above convex hull (C2DB)**: 0.416 eV
- **Space group symbol**: $P\bar{6}m2$
- **Space group number**: 187
- **Polar structure**: False

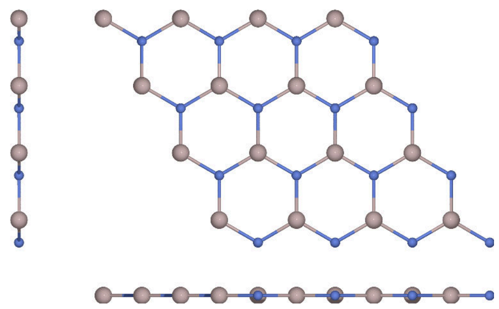

Figure 312: Structure representation

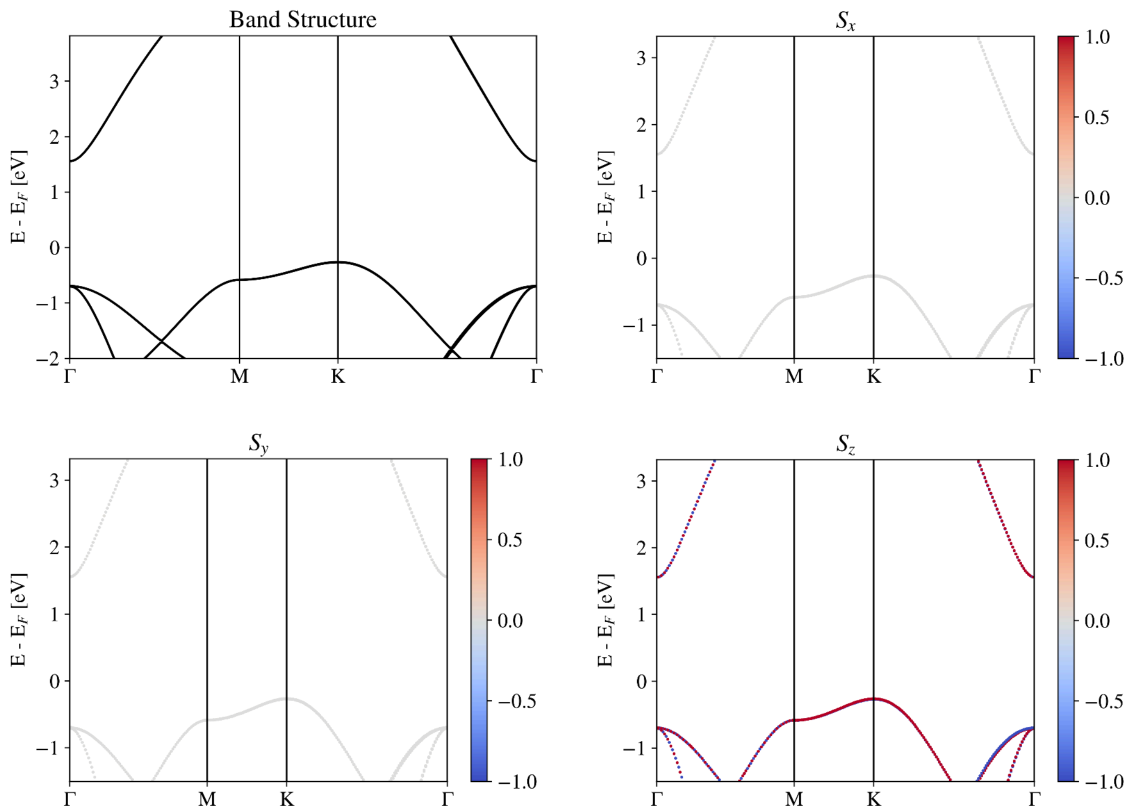


Figure 313: Band structure and spin polarization projections.

## 2.157 GaP-d467820f3f04

- **Formula**: GaP
- **Structural Cluster**: AB-2
- **Band gap (PBE)**: 1.555 eV
- **Energy above convex hull (C2DB)**: 0.447 eV
- **Space group symbol**: $P3m1$
- **Space group number**: 156
- **Polar structure**: True

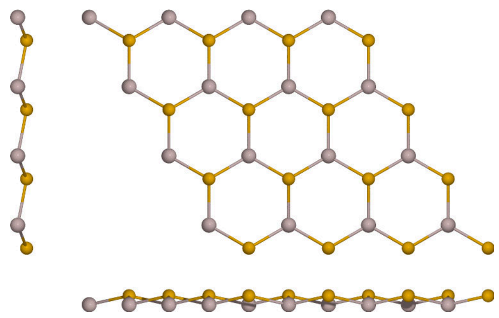

Figure 314: Structure representation

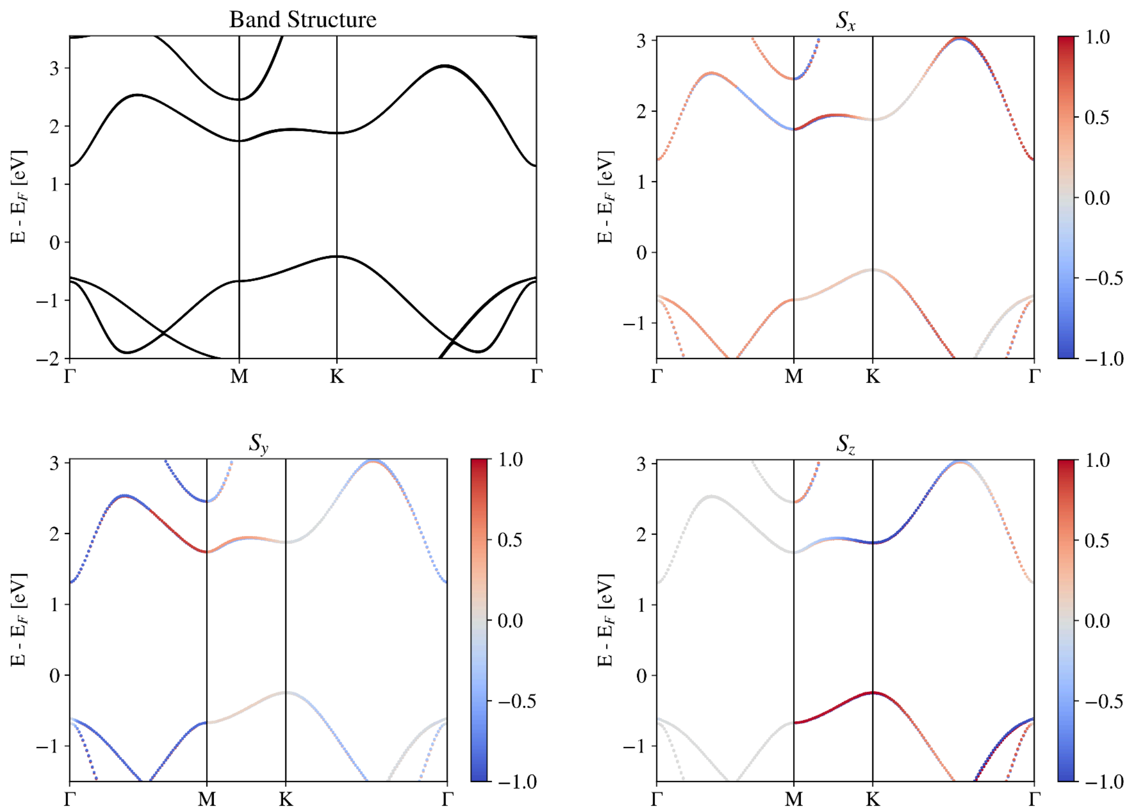


Figure 315: Band structure and spin polarization projections.

## 2.158 Ge2P2Se6-6152fa4b5f1b

- **Formula**: Ge2P2Se6
- **Structural Cluster**: ABC3-16
- **Band gap (PBE)**: 0.978 eV
- **Energy above convex hull (C2DB)**: 0.034 eV
- **Space group symbol**: *P*1
- **Space group number**: 1
- **Polar structure**: True

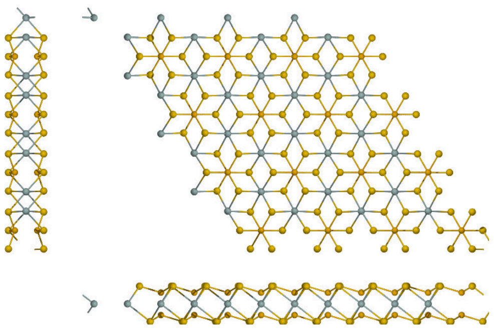

Figure 316: Structure representation

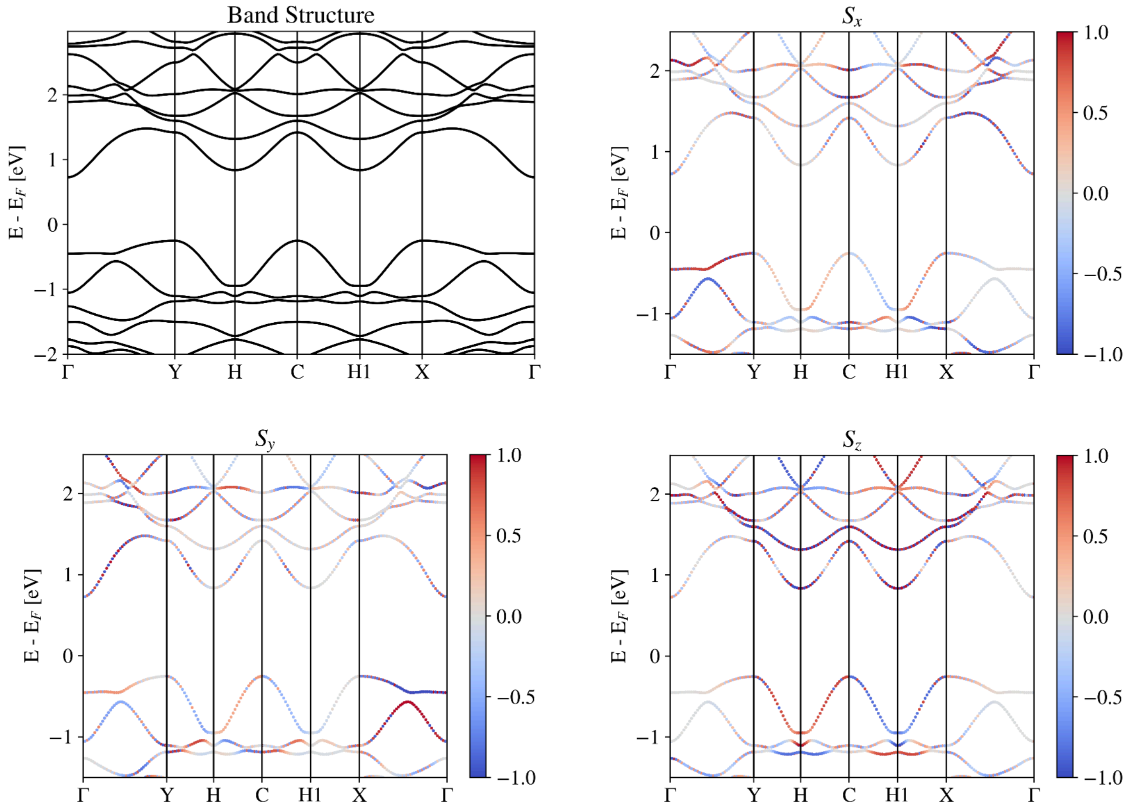


Figure 317: Band structure and spin polarization projections.

## 2.159 Ge2P2Te6-e8f4b479deef

- **Formula**: Ge2P2Te6
- **Structural Cluster**: ABC3-16
- **Band gap (PBE)**: 0.868 eV
- **Energy above convex hull (C2DB)**: 0.067 eV
- **Space group symbol**: *P*1
- **Space group number**: 1
- **Polar structure**: True

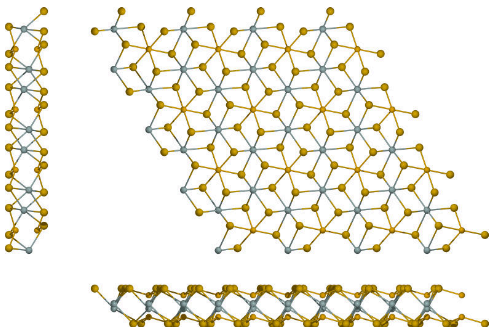

Figure 318: Structure representation

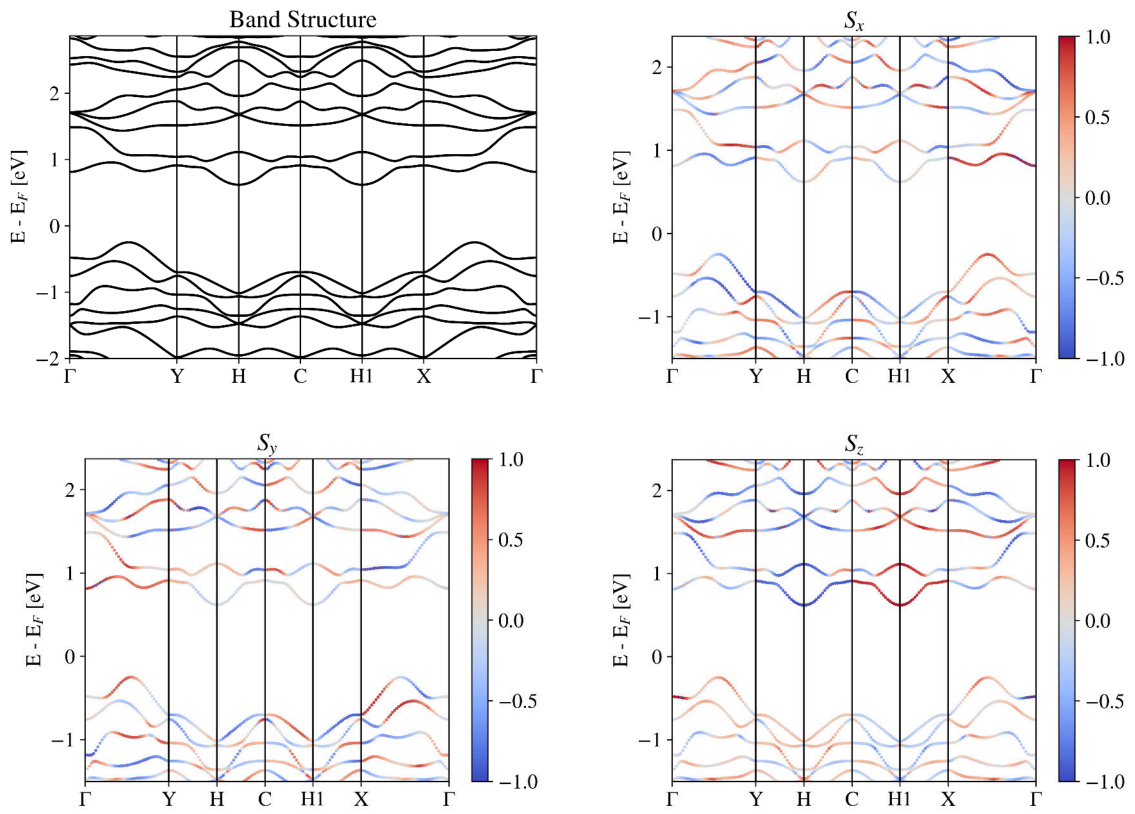


Figure 319: Band structure and spin polarization projections.

## 2.160 Ge2S2-ecbb7c185669

- **Formula**: Ge2S2
- **Structural Cluster**: AB-0
- **Band gap (PBE)**: 1.714 eV
- **Energy above convex hull (C2DB)**: 0.031 eV
- **Space group symbol**: $Pmn2_1$
- **Space group number**: 31
- **Polar structure**: True

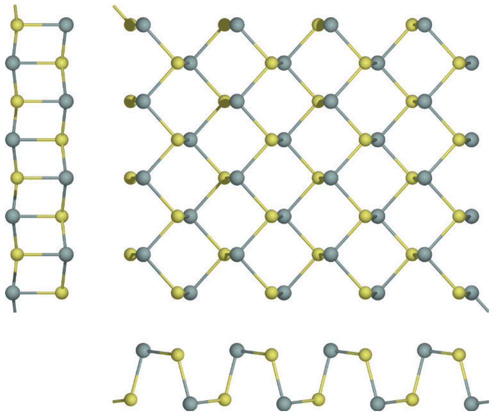

Figure 320: Structure representation

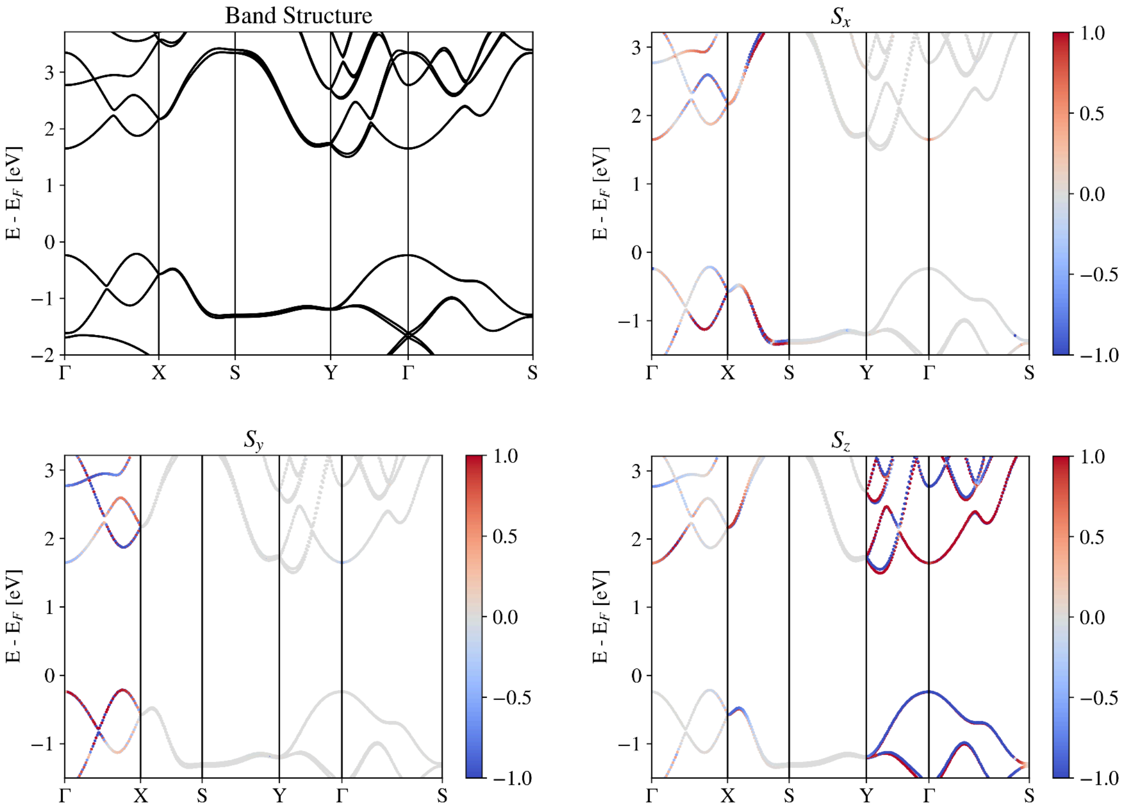


Figure 321: Band structure and spin polarization projections.

## 2.161 GeBr2-204ef2affa10

- **Formula**: GeBr2
- **Structural Cluster**: AB2-11
- **Band gap (PBE)**: 1.312 eV
- **Energy above convex hull (C2DB)**: 0.136 eV
- **Space group symbol**: $P\bar{4}m2$
- **Space group number**: 115
- **Polar structure**: False

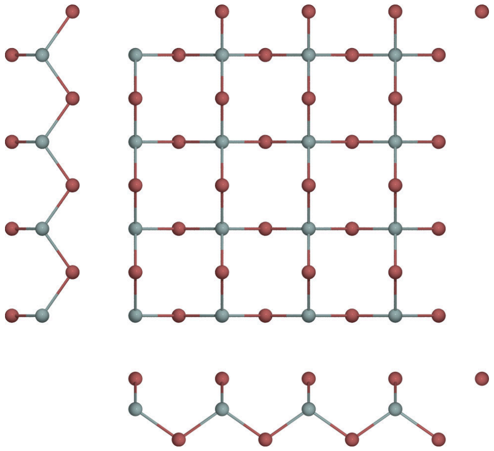

Figure 322: Structure representation

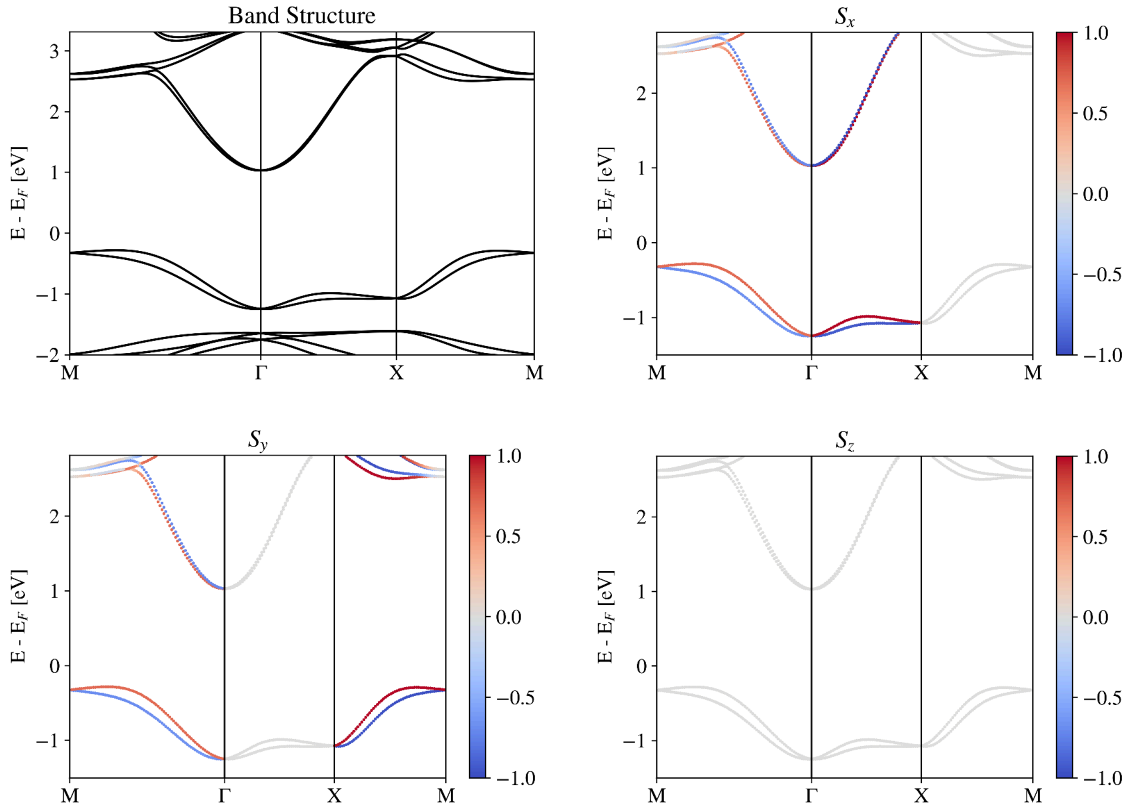


Figure 323: Band structure and spin polarization projections.

## 2.162 GeBr2-36a198743d35

- **Formula**: GeBr2
- **Structural Cluster**: AB2-4
- **Band gap (PBE)**: 2.543 eV
- **Energy above convex hull (C2DB)**: 0.112 eV
- **Space group symbol**: $P\bar{6}m2$
- **Space group number**: 187
- **Polar structure**: False

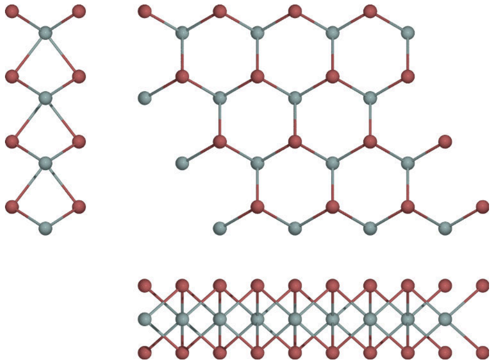

Figure 324: Structure representation

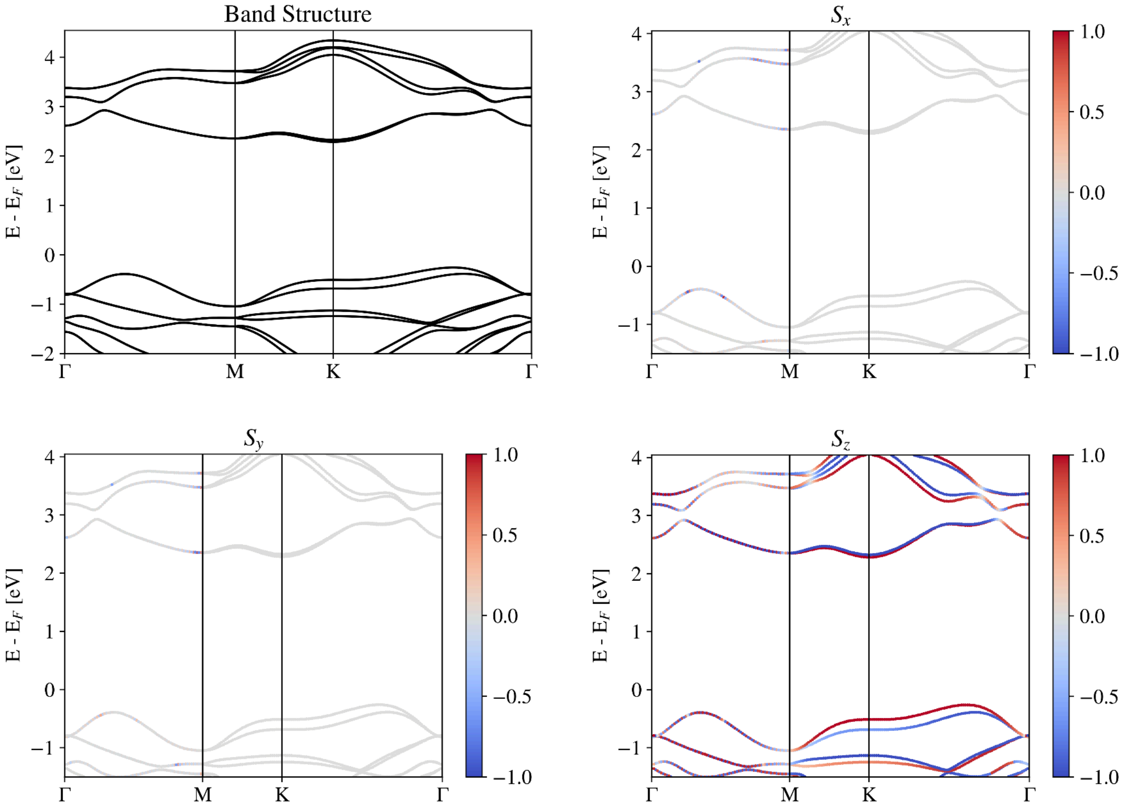


Figure 325: Band structure and spin polarization projections.

## 2.163 GeCl2-3ea474649fa9

- **Formula**: GeCl2
- **Structural Cluster**: AB2-11
- **Band gap (PBE)**: 1.381 eV
- **Energy above convex hull (C2DB)**: 0.143 eV
- **Space group symbol**: $P\bar{4}m2$
- **Space group number**: 115
- **Polar structure**: False

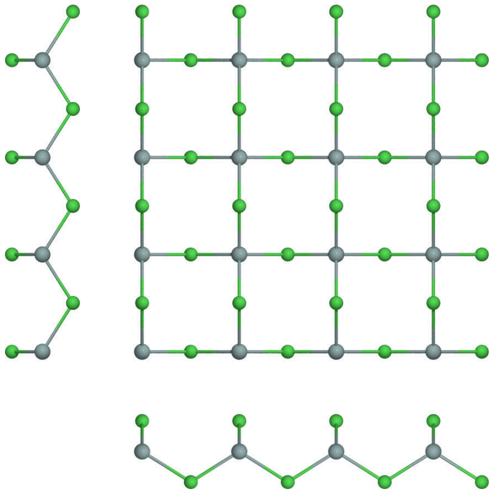

Figure 326: Structure representation

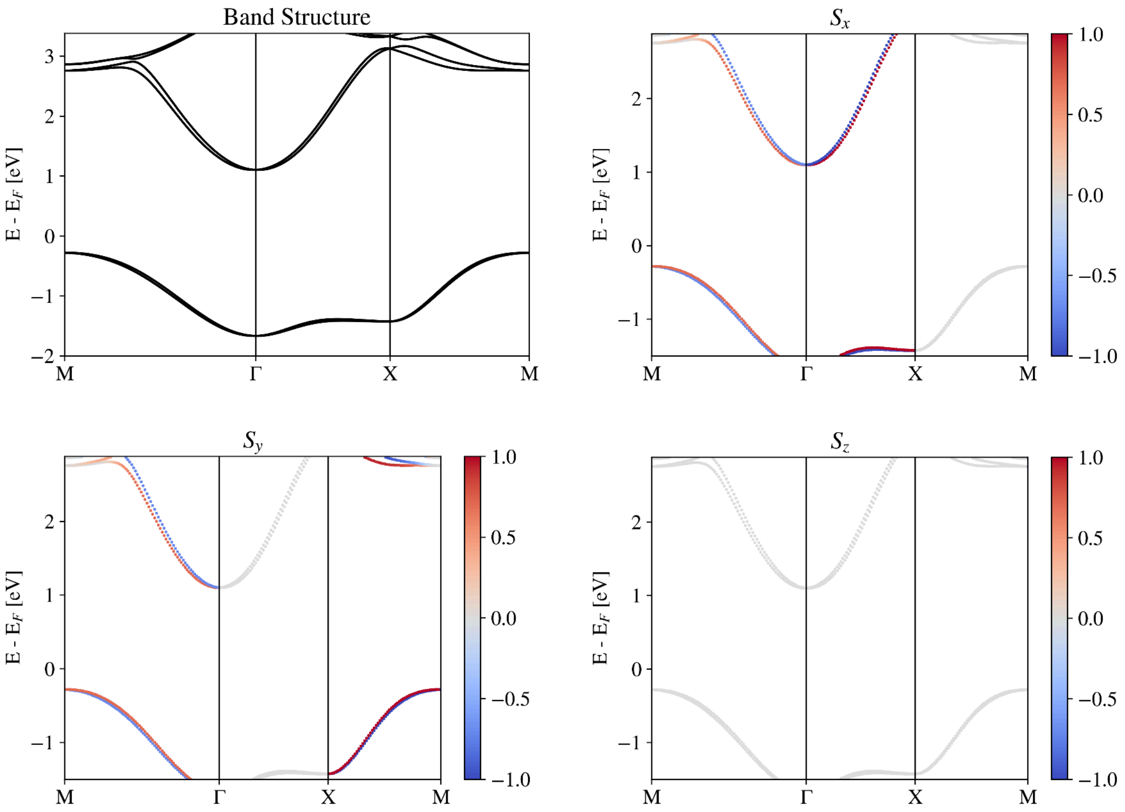


Figure 327: Band structure and spin polarization projections.

## 2.164 GeCl2-a7216f084785

- **Formula**: GeCl2
- **Structural Cluster**: AB2-4
- **Band gap (PBE)**: 2.955 eV
- **Energy above convex hull (C2DB)**: 0.136 eV
- **Space group symbol**: $P\bar{6}m2$
- **Space group number**: 187
- **Polar structure**: False

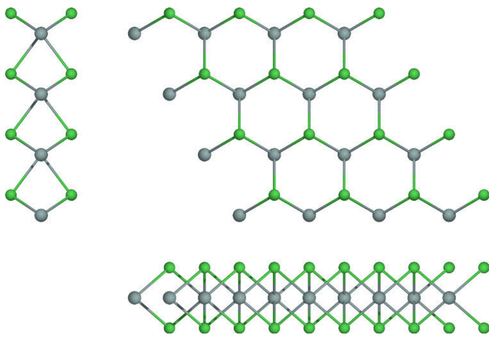

Figure 328: Structure representation

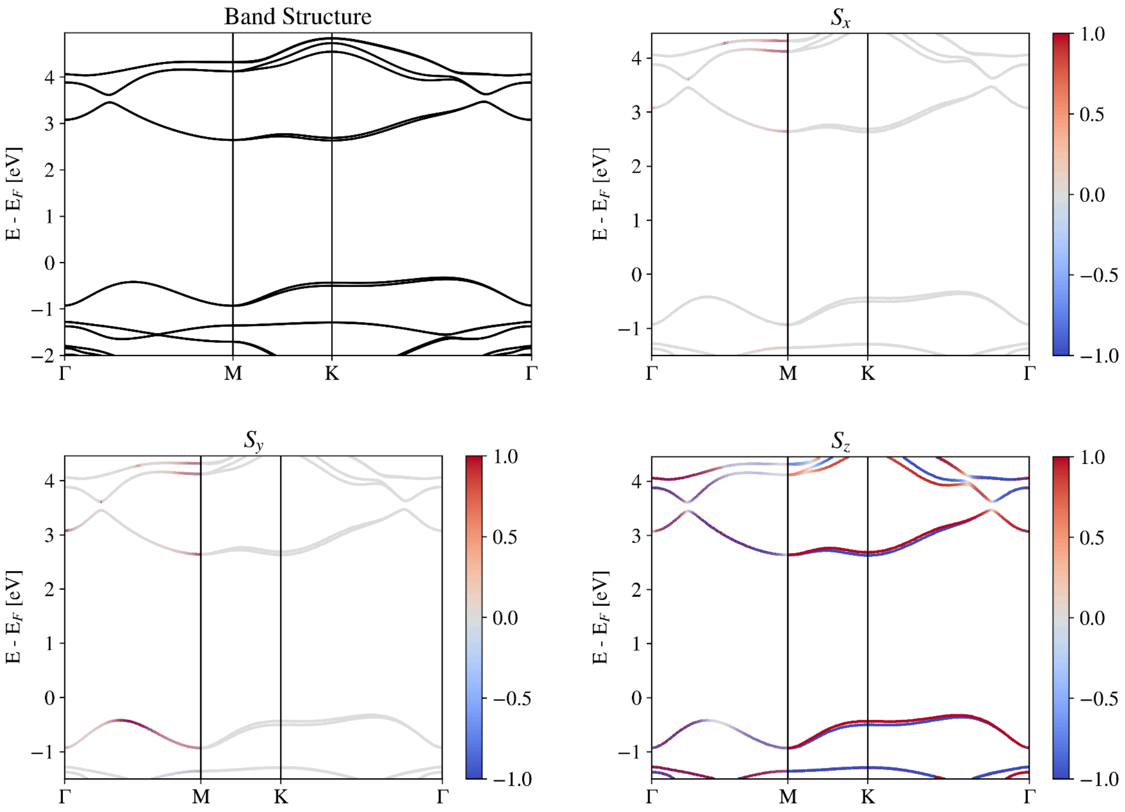


Figure 329: Band structure and spin polarization projections.

## 2.165 GeF2-943ceb2df00b

- **Formula**: GeF2
- **Structural Cluster**: AB2-11
- **Band gap (PBE)**: 1.623 eV
- **Energy above convex hull (C2DB)**: 0.215 eV
- **Space group symbol**: $P\bar{4}m2$
- **Space group number**: 115
- **Polar structure**: False

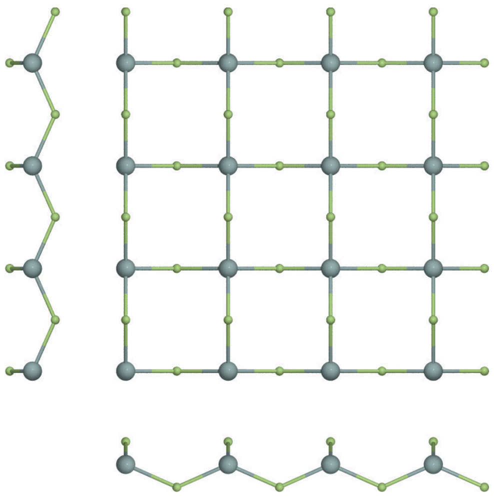

Figure 330: Structure representation

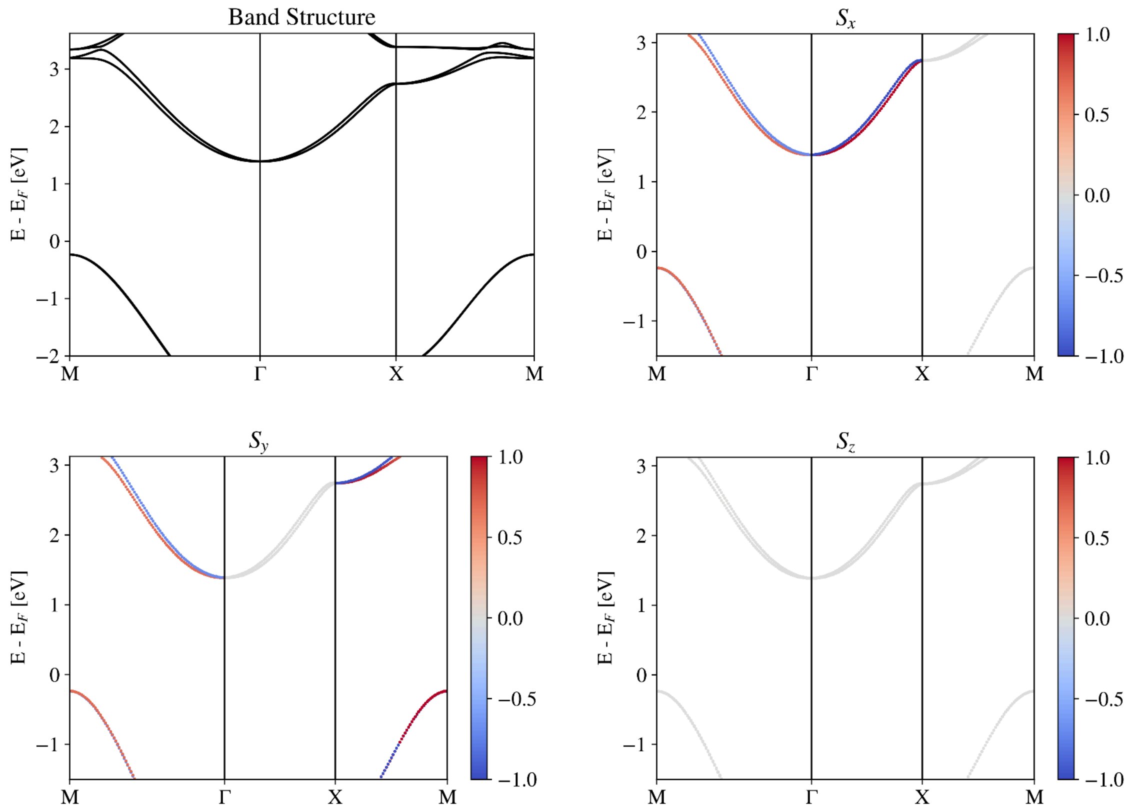


Figure 331: Band structure and spin polarization projections.

## 2.166 GeI2-694ac91aec01

- **Formula**: GeI2
- **Structural Cluster**: AB2-11
- **Band gap (PBE)**: 1.059 eV
- **Energy above convex hull (C2DB)**: 0.153 eV
- **Space group symbol**: $P\bar{4}m2$
- **Space group number**: 115
- **Polar structure**: False

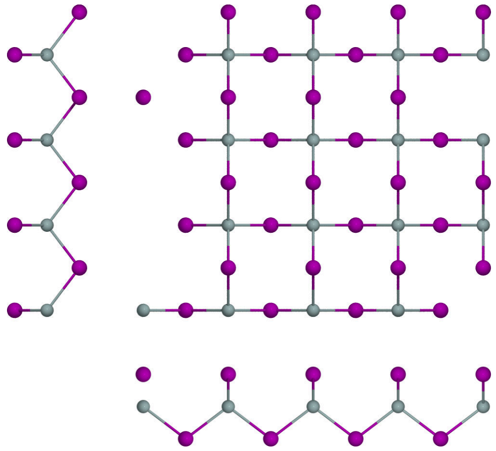

Figure 332: Structure representation

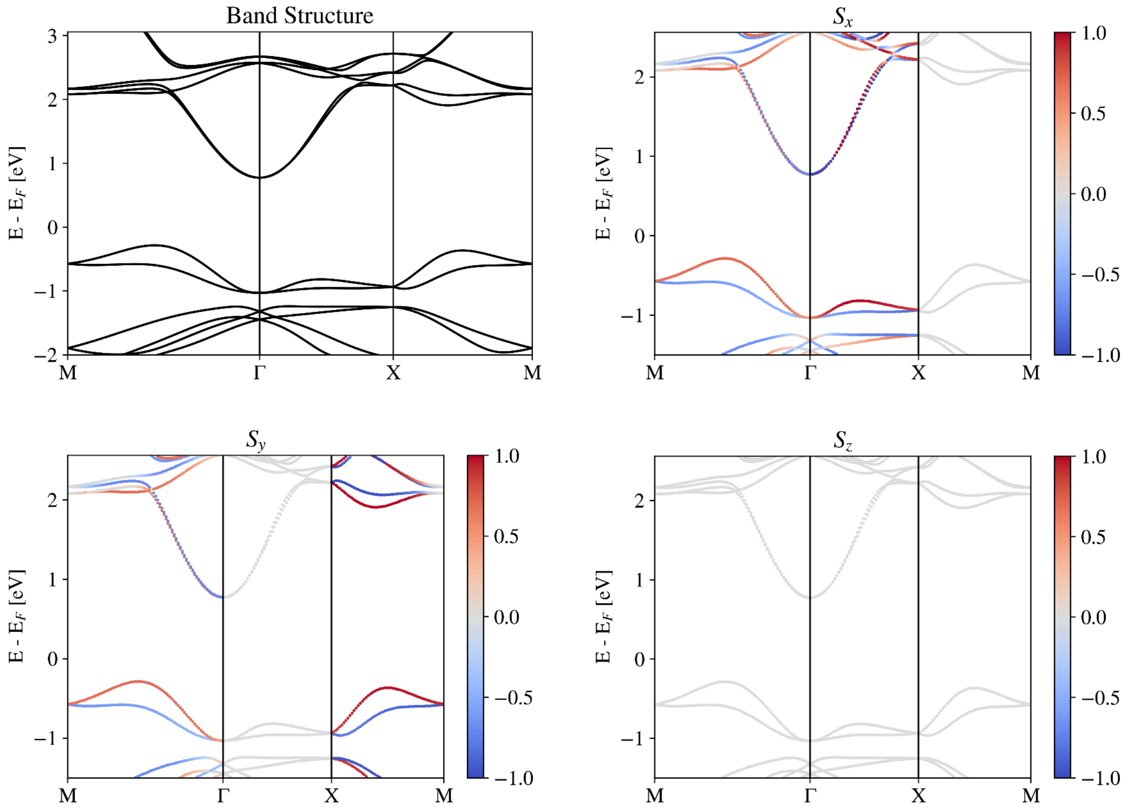


Figure 333: Band structure and spin polarization projections.

## 2.167 GeO-a42f736f1682

- **Formula**: GeO
- **Structural Cluster**: AB-25
- **Band gap (PBE)**: 2.093 eV
- **Energy above convex hull (C2DB)**: 0.311 eV
- **Space group symbol**: $P3m1$
- **Space group number**: 156
- **Polar structure**: True

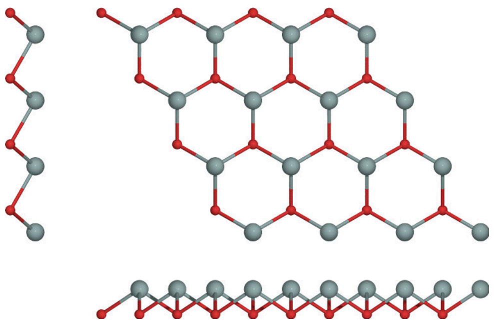

Figure 334: Structure representation

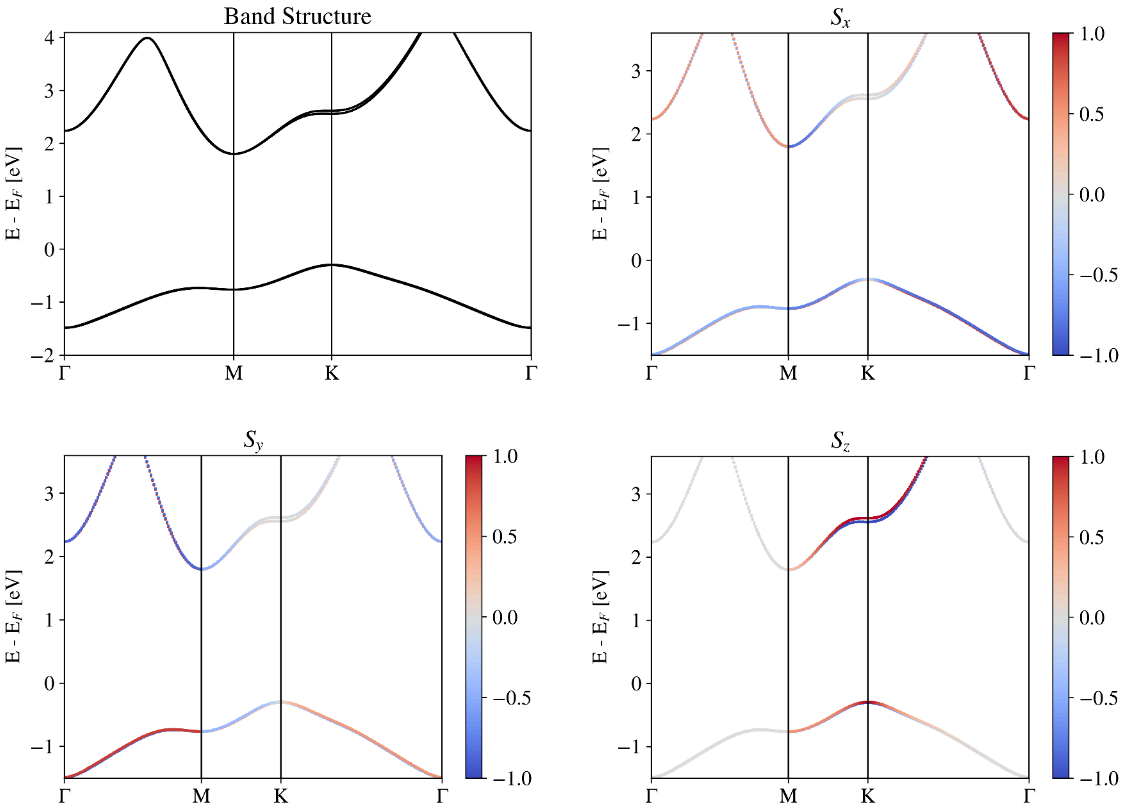


Figure 335: Band structure and spin polarization projections.

## 2.168 GeO2-21281ac194c2

- **Formula**: GeO2
- **Structural Cluster**: AB2-11
- **Band gap (PBE)**: 2.944 eV
- **Energy above convex hull (C2DB)**: 0.152 eV
- **Space group symbol**: $P\bar{4}m2$
- **Space group number**: 115
- **Polar structure**: False

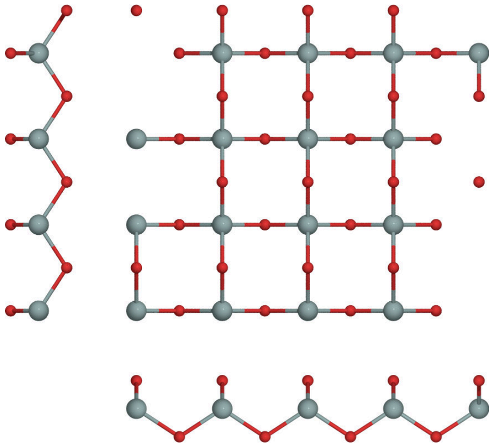

Figure 336: Structure representation

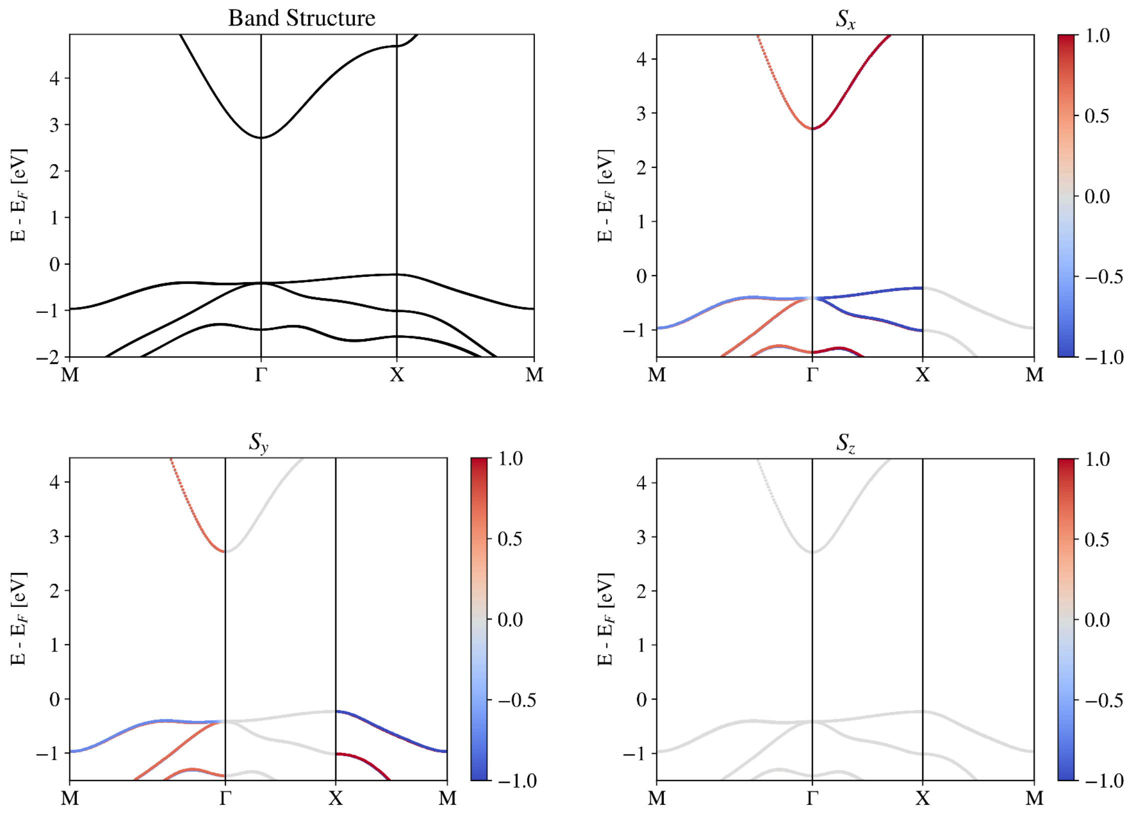


Figure 337: Band structure and spin polarization projections.

## 2.169 GeO2-77905aa4e75f

- **Formula**: GeO2
- **Structural Cluster**: AB2-4
- **Band gap (PBE)**: 1.392 eV
- **Energy above convex hull (C2DB)**: 0.639 eV
- **Space group symbol**: $P\bar{6}m2$
- **Space group number**: 187
- **Polar structure**: False

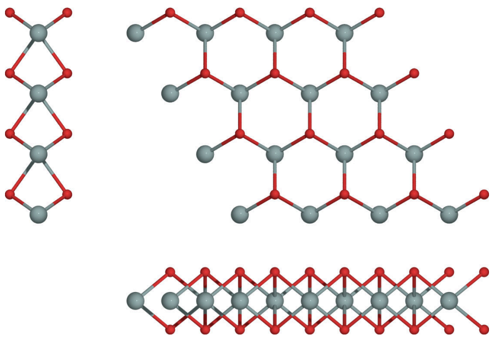

Figure 338: Structure representation

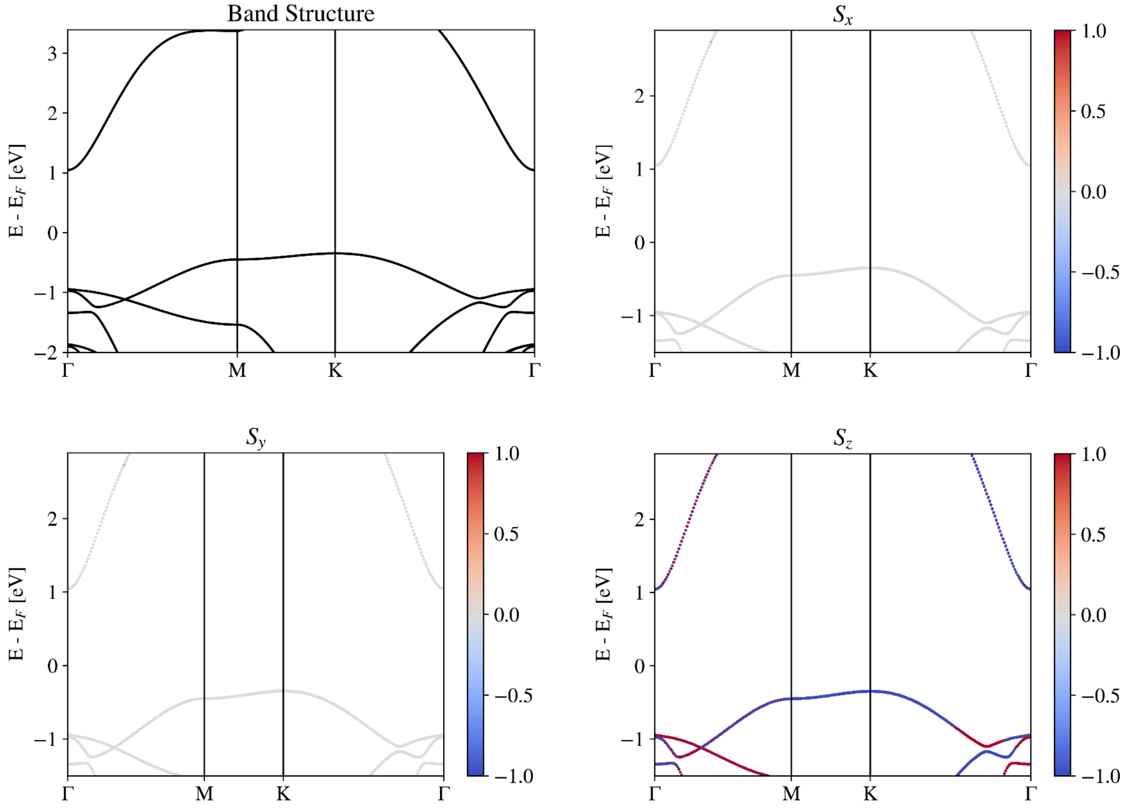


Figure 339: Band structure and spin polarization projections.

## 2.170 GeS-227b12019ade

- **Formula**: GeS
- **Structural Cluster**: AB-25
- **Band gap (PBE)**: 2.467 eV
- **Energy above convex hull (C2DB)**: 0.053 eV
- **Space group symbol**: $P3m1$
- **Space group number**: 156
- **Polar structure**: True

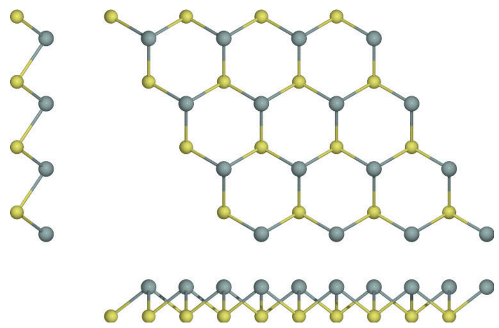

Figure 340: Structure representation

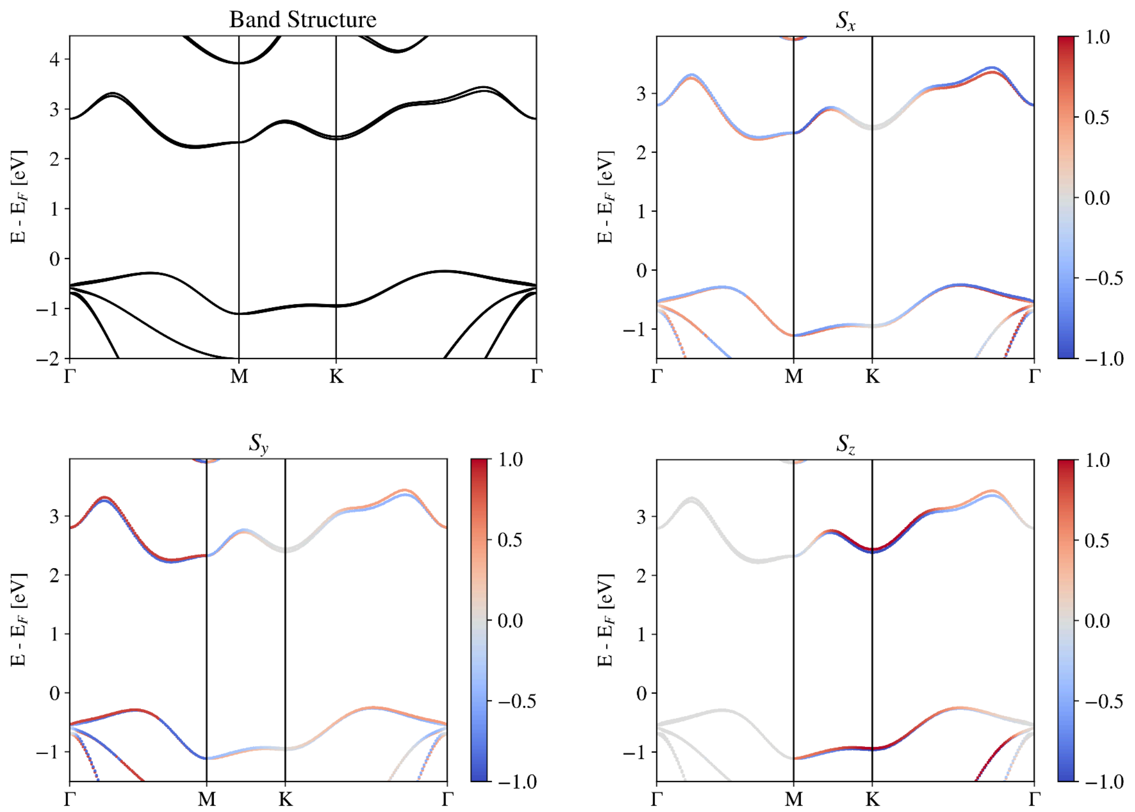


Figure 341: Band structure and spin polarization projections.

## 2.171 GeS2-69b36b84eb8c

- **Formula**: GeS2
- **Structural Cluster**: AB2-11
- **Band gap (PBE)**: 1.36 eV
- **Energy above convex hull (C2DB)**: 0.049 eV
- **Space group symbol**: $P\bar{4}m2$
- **Space group number**: 115
- **Polar structure**: False

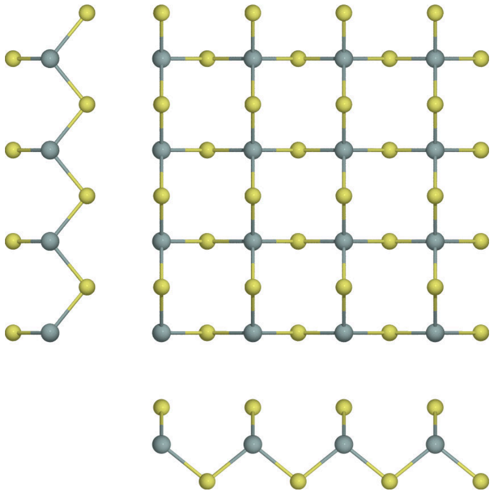

Figure 342: Structure representation

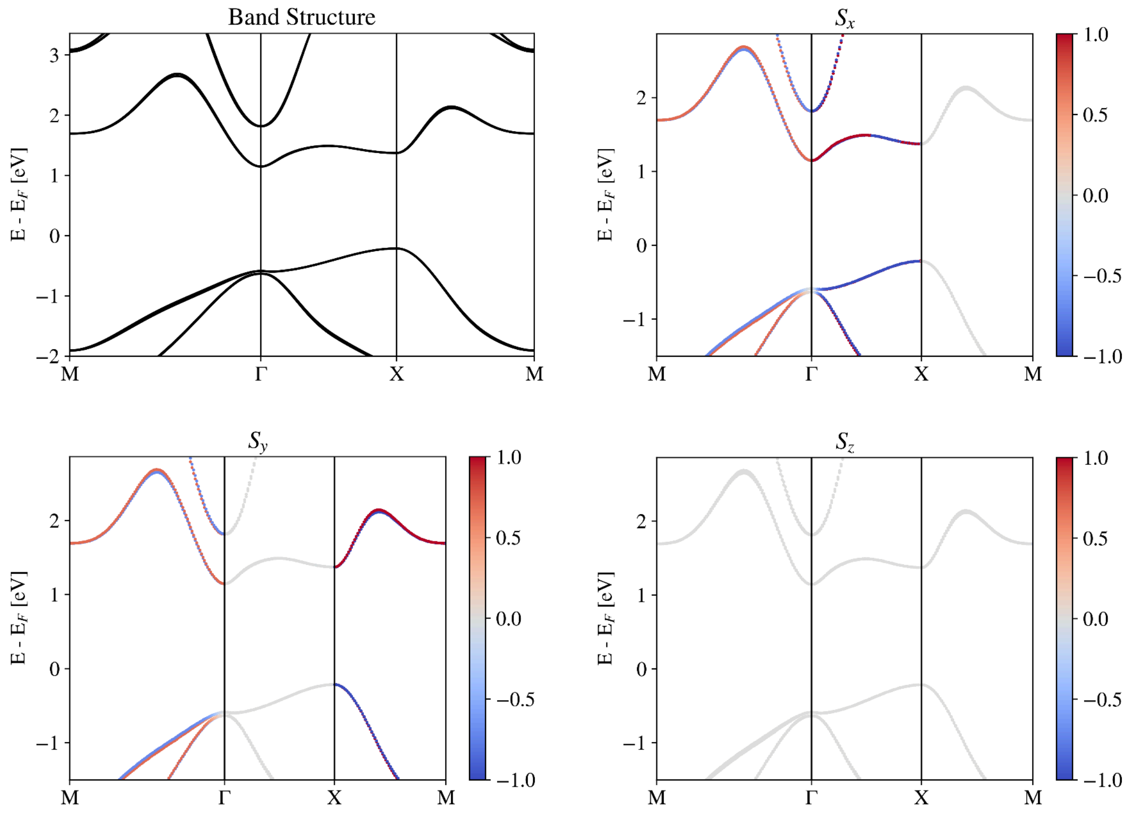


Figure 343: Band structure and spin polarization projections.

## 2.172 GeSe-211bcb7f05d6

- **Formula**: GeSe
- **Structural Cluster**: AB-25
- **Band gap (PBE)**: 2.215 eV
- **Energy above convex hull (C2DB)**: 0.04 eV
- **Space group symbol**: $P3m1$
- **Space group number**: 156
- **Polar structure**: True

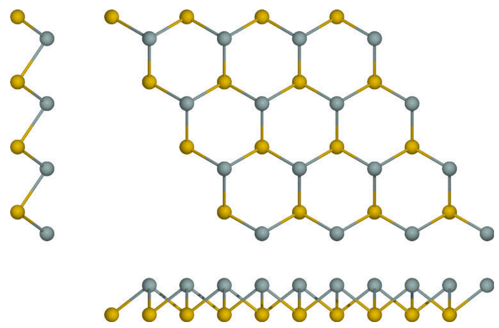

Figure 344: Structure representation

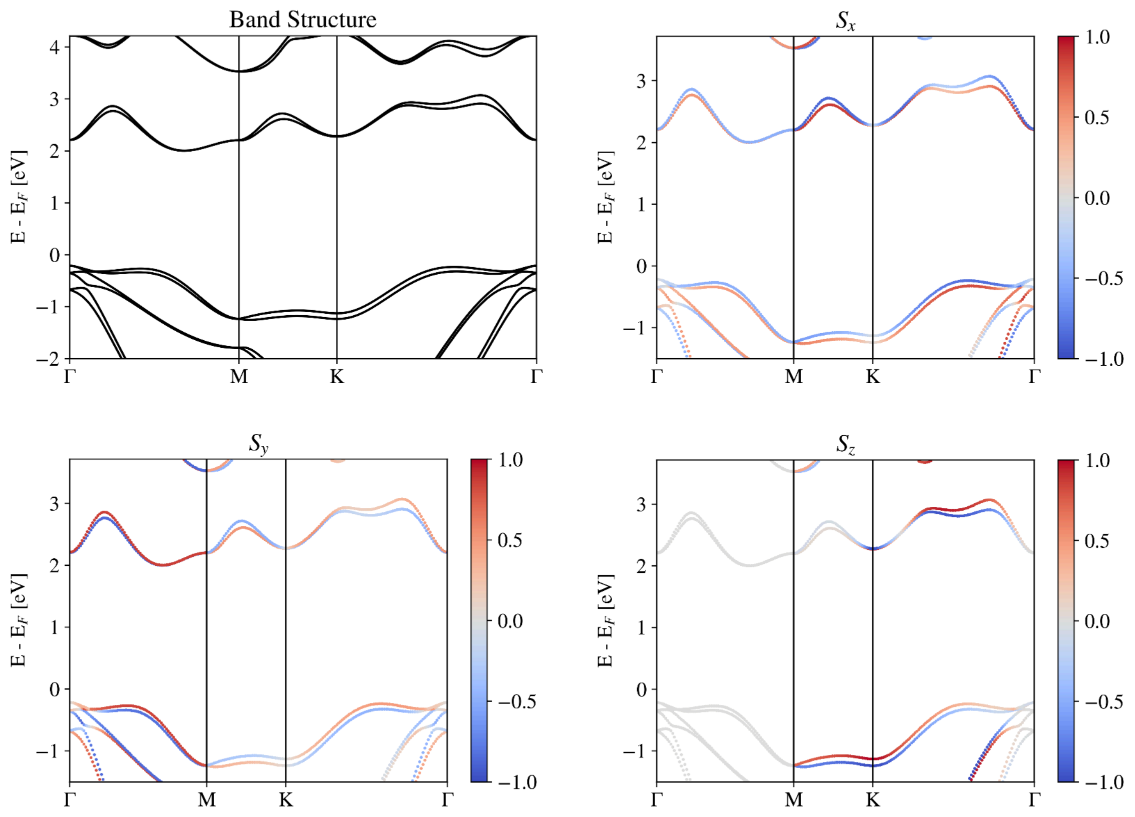


Figure 345: Band structure and spin polarization projections.

## 2.173 GeSe2-8af45b2cf14e

- **Formula**: GeSe2
- **Structural Cluster**: AB2-11
- **Band gap (PBE)**: 0.556 eV
- **Energy above convex hull (C2DB)**: 0.051 eV
- **Space group symbol**: $P\bar{4}m2$
- **Space group number**: 115
- **Polar structure**: False

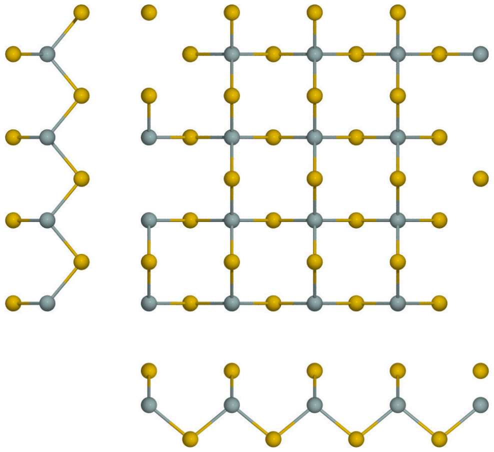

Figure 346: Structure representation

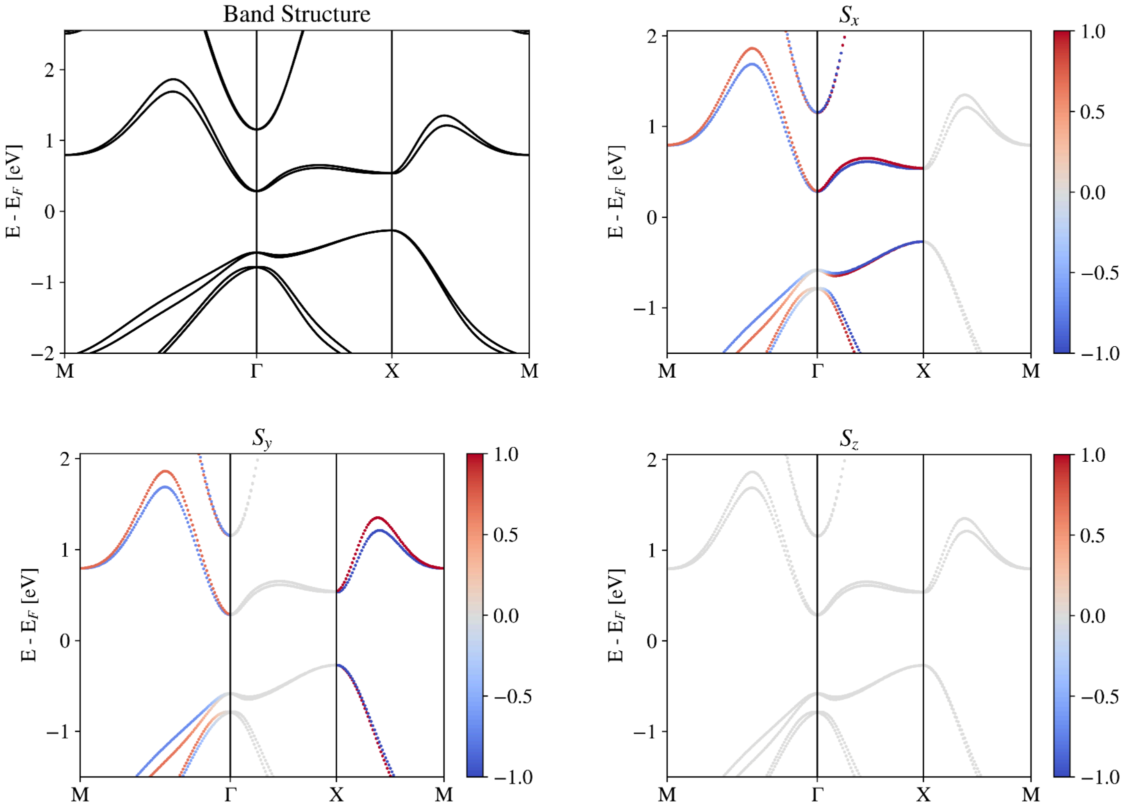


Figure 347: Band structure and spin polarization projections.

## 2.174 GeTe-eadd37f03ca5

- **Formula**: GeTe
- **Structural Cluster**: AB-25
- **Band gap (PBE)**: 1.488 eV
- **Energy above convex hull (C2DB)**: 0.087 eV
- **Space group symbol**: $P3m1$
- **Space group number**: 156
- **Polar structure**: True

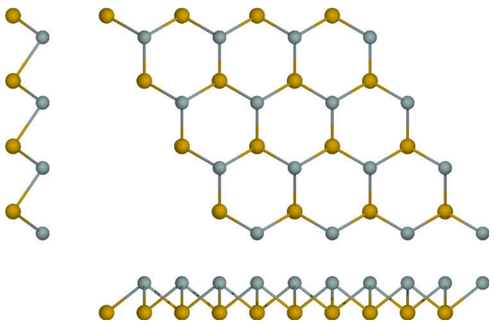

Figure 348: Structure representation

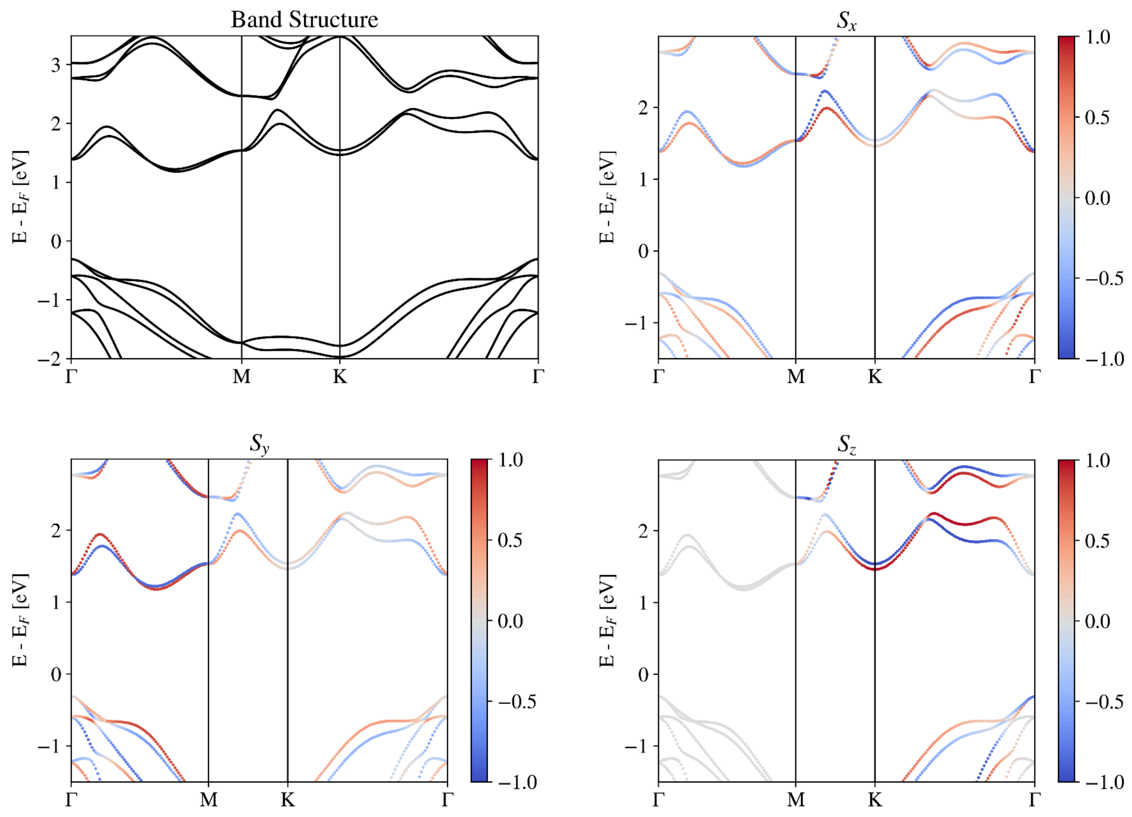


Figure 349: Band structure and spin polarization projections.

## 2.175 Hf2Ti2S8-c8cfffe63bfa

- **Formula**: Hf2Ti2S8
- **Structural Cluster**: ABC4-22
- **Band gap (PBE)**: 0.917 eV
- **Energy above convex hull (C2DB)**: 0.192 eV
- **Space group symbol**: $P1$
- **Space group number**: 1
- **Polar structure**: True

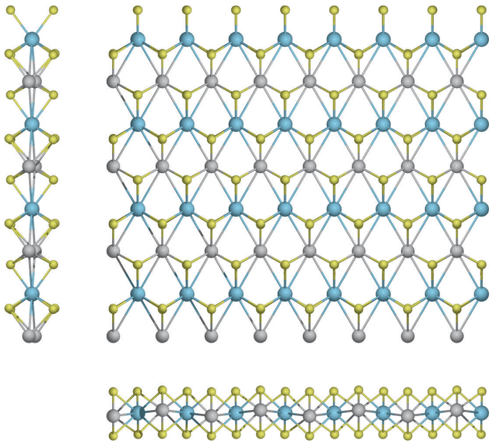

Figure 350: Structure representation

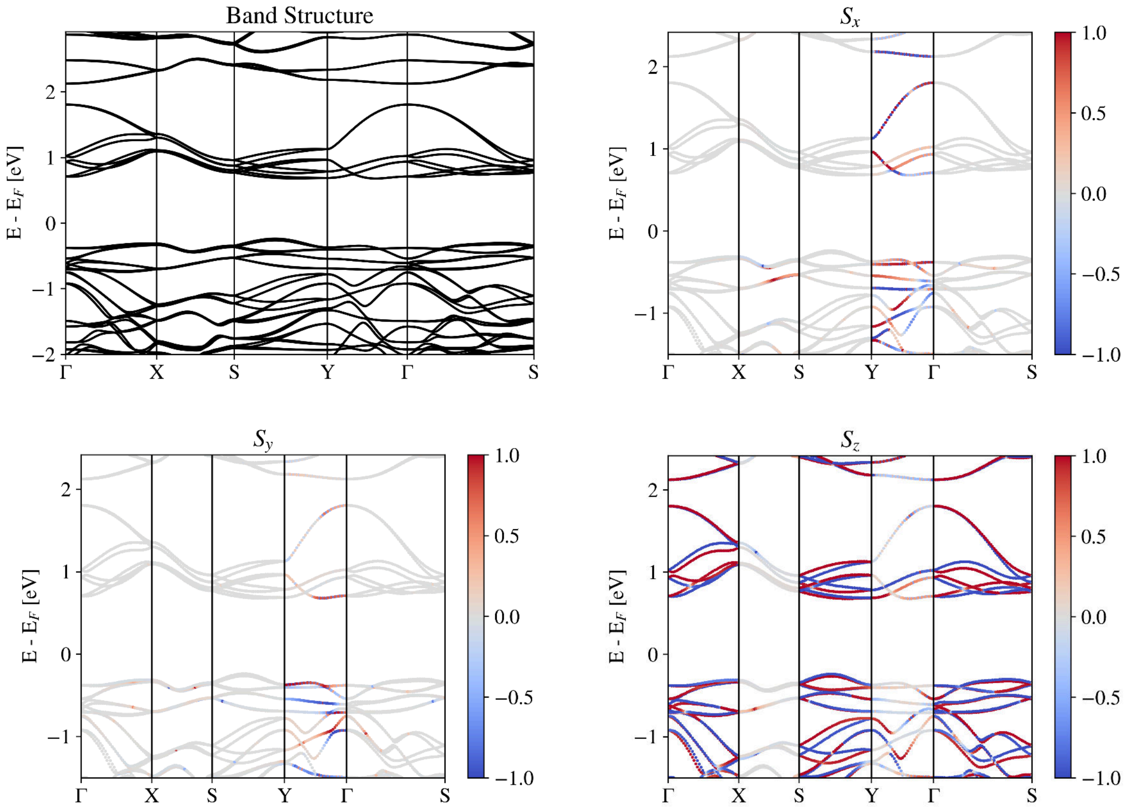


Figure 351: Band structure and spin polarization projections.

## 2.176 Hf2Ti2Se8-cce78d90e899

- **Formula**: Hf2Ti2Se8
- **Structural Cluster**: ABC4-22
- **Band gap (PBE)**: 0.656 eV
- **Energy above convex hull (C2DB)**: 0.156 eV
- **Space group symbol**: $P1$
- **Space group number**: 1
- **Polar structure**: True

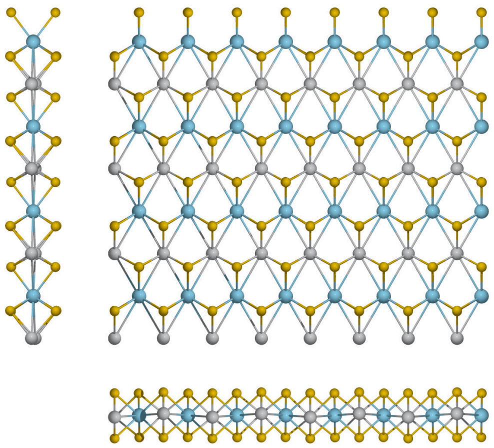

Figure 352: Structure representation

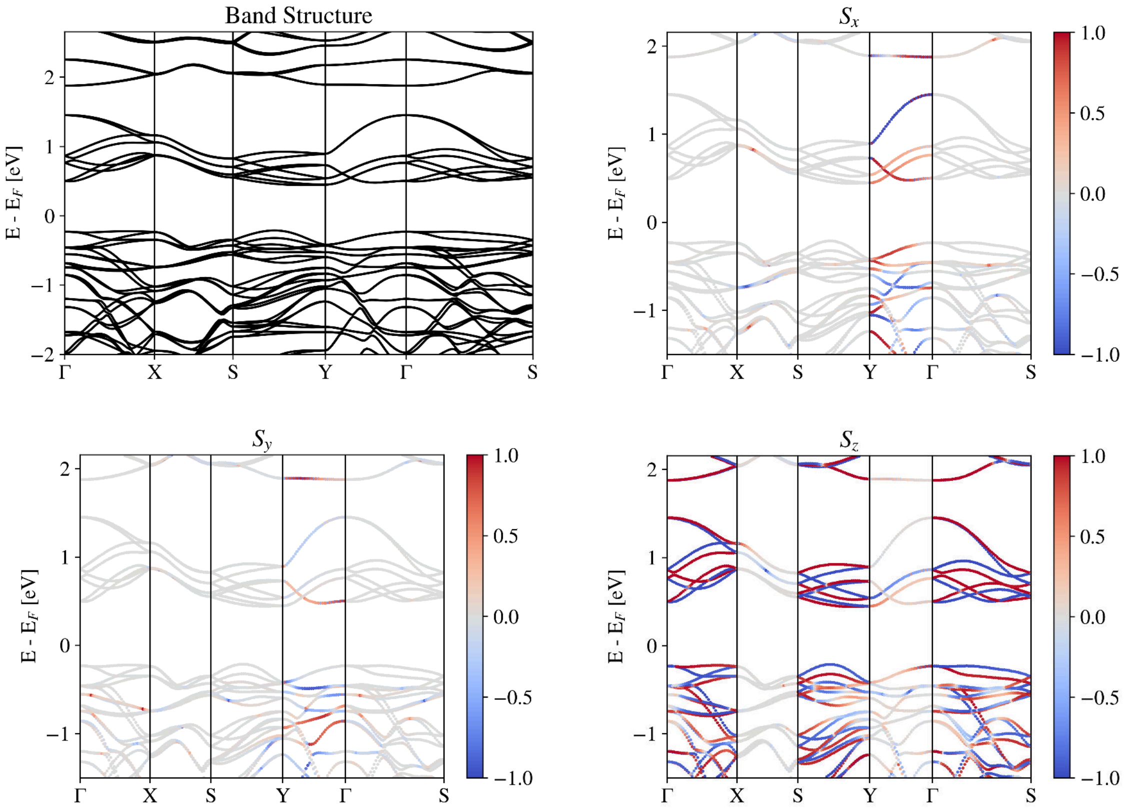


Figure 353: Band structure and spin polarization projections.

## 2.177 Hf2Zr2S8-540829ada792

- **Formula**: Hf2Zr2S8
- **Structural Cluster**: ABC4-22
- **Band gap (PBE)**: 1.145 eV
- **Energy above convex hull (C2DB)**: 0.2 eV
- **Space group symbol**: $P1$
- **Space group number**: 1
- **Polar structure**: True

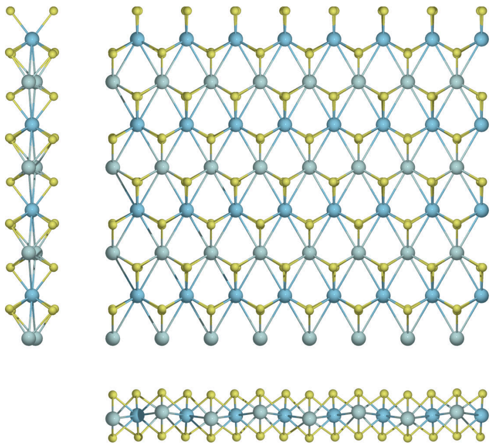

Figure 354: Structure representation

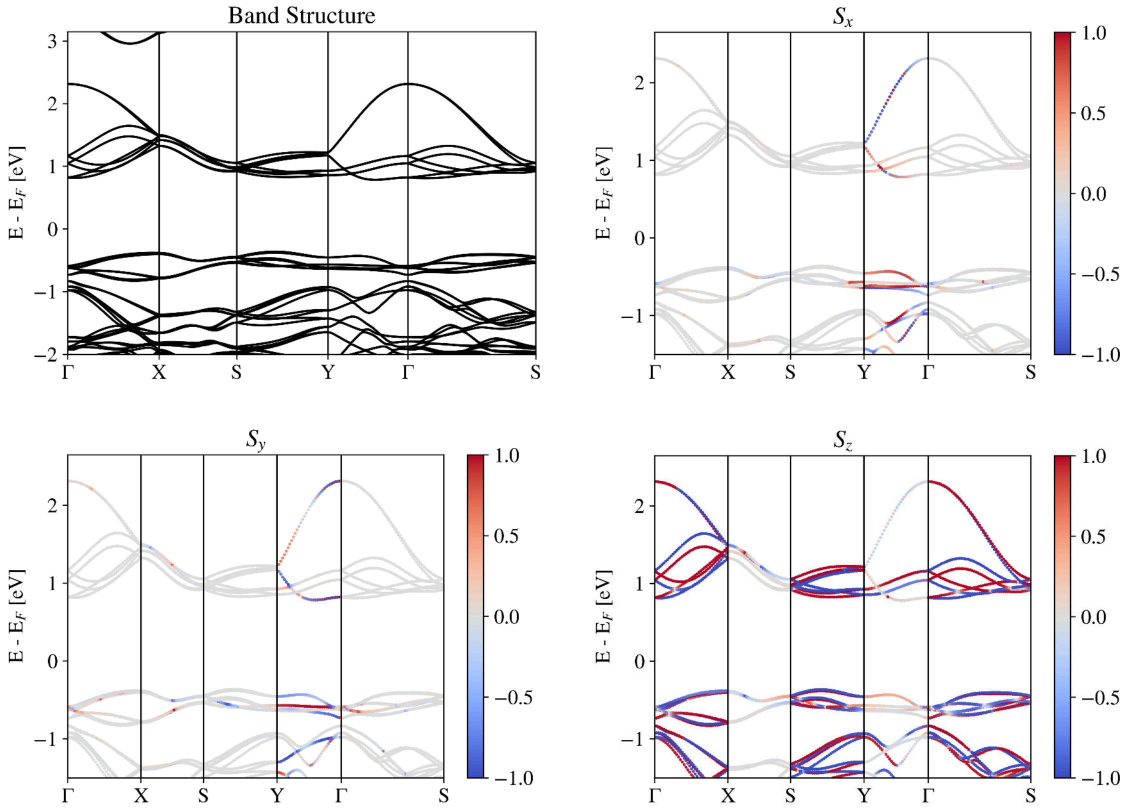


Figure 355: Band structure and spin polarization projections.

## 2.178 Hf2Zr2Se8-81af2831dbb2

- **Formula**: Hf2Zr2Se8
- **Structural Cluster**: ABC4-22
- **Band gap (PBE)**: 0.845 eV
- **Energy above convex hull (C2DB)**: 0.158 eV
- **Space group symbol**: $P1$
- **Space group number**: 1
- **Polar structure**: True

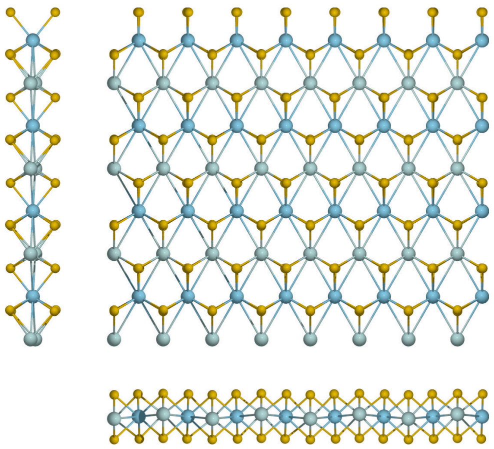

Figure 356: Structure representation

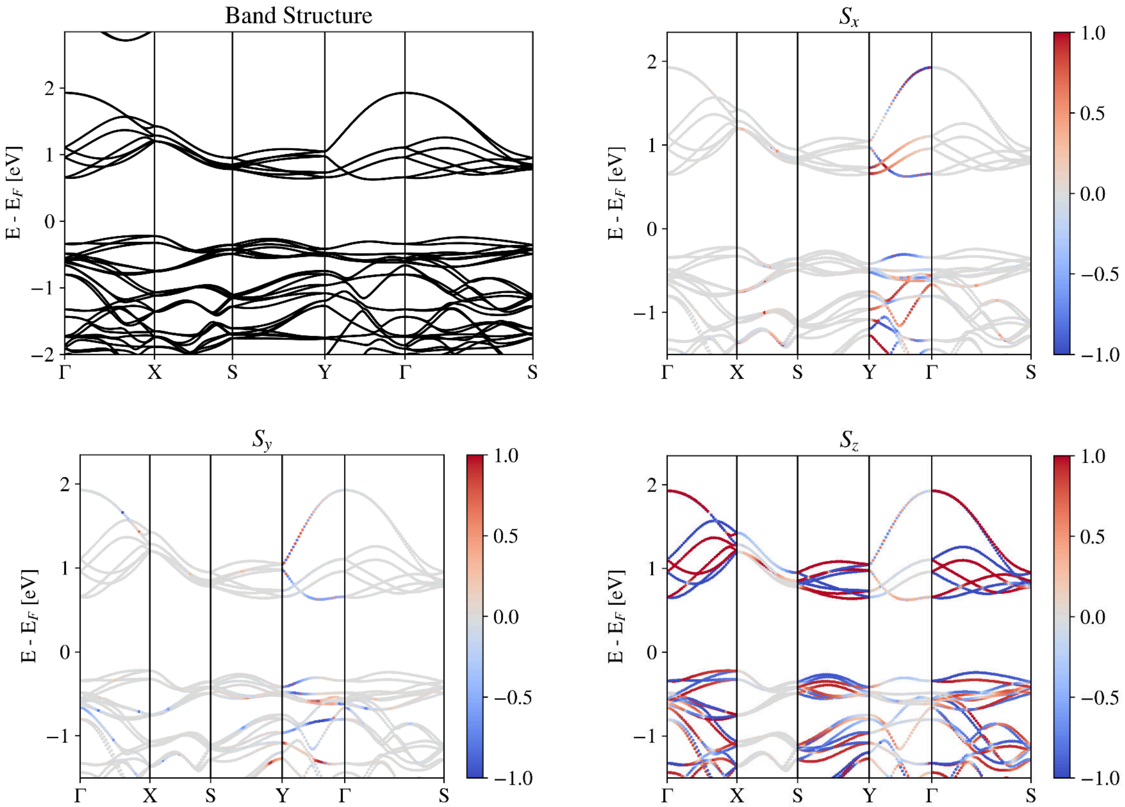


Figure 357: Band structure and spin polarization projections.

## 2.179 Hf2Zr2Te8-93099006c996

- **Formula**: Hf2Zr2Te8
- **Structural Cluster**: ABC4-22
- **Band gap (PBE)**: 0.189 eV
- **Energy above convex hull (C2DB)**: 0.121 eV
- **Space group symbol**: $P1$
- **Space group number**: 1
- **Polar structure**: True

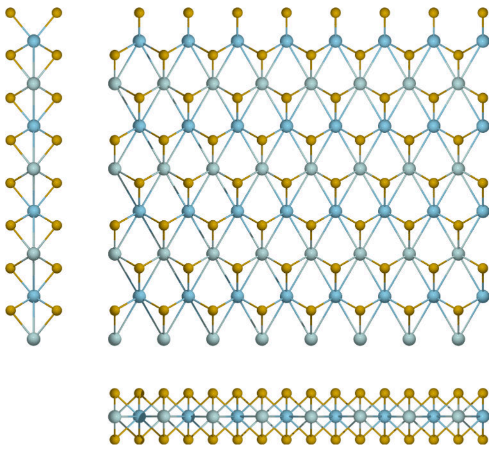

Figure 358: Structure representation

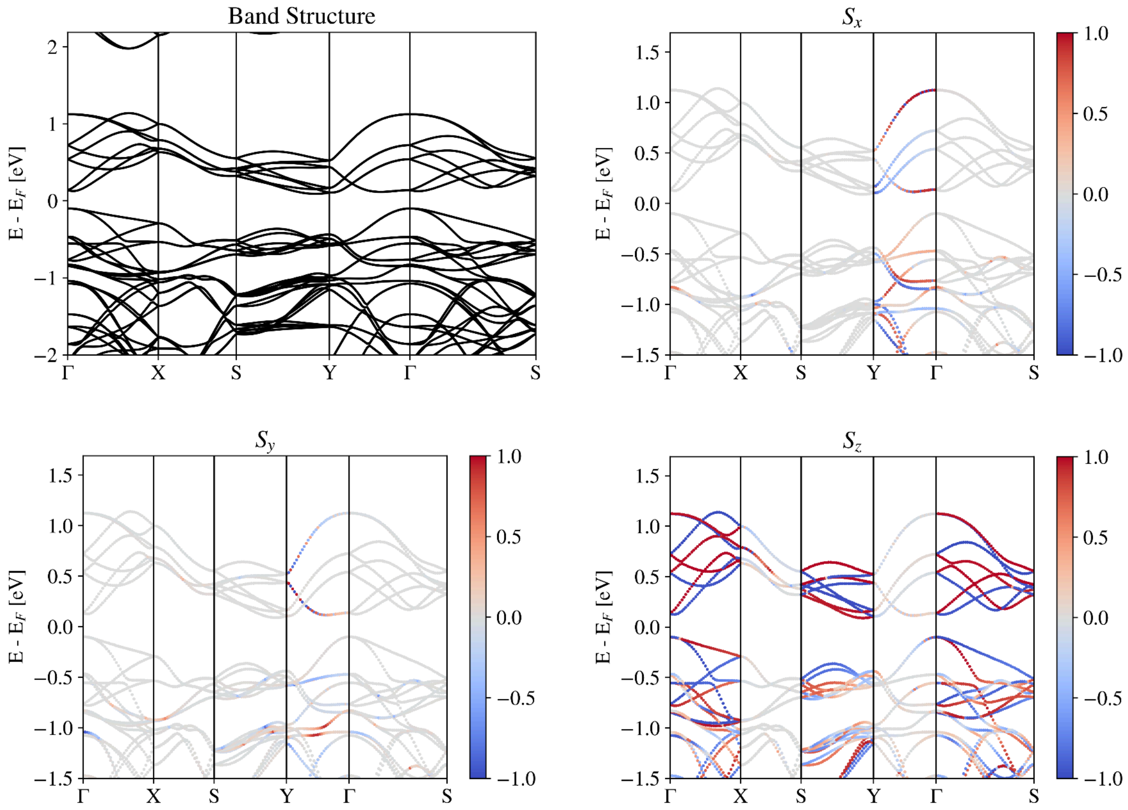


Figure 359: Band structure and spin polarization projections.

## 2.180 HfBr2-84e9162c0c53

- **Formula**: HfBr2
- **Structural Cluster**: AB2-4
- **Band gap (PBE)**: 0.722 eV
- **Energy above convex hull (C2DB)**: 0.004 eV
- **Space group symbol**: $P\bar{6}m2$
- **Space group number**: 187
- **Polar structure**: False

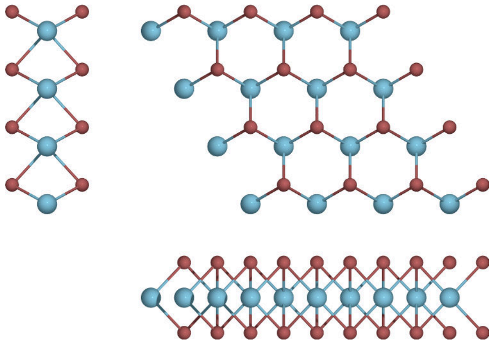

Figure 360: Structure representation

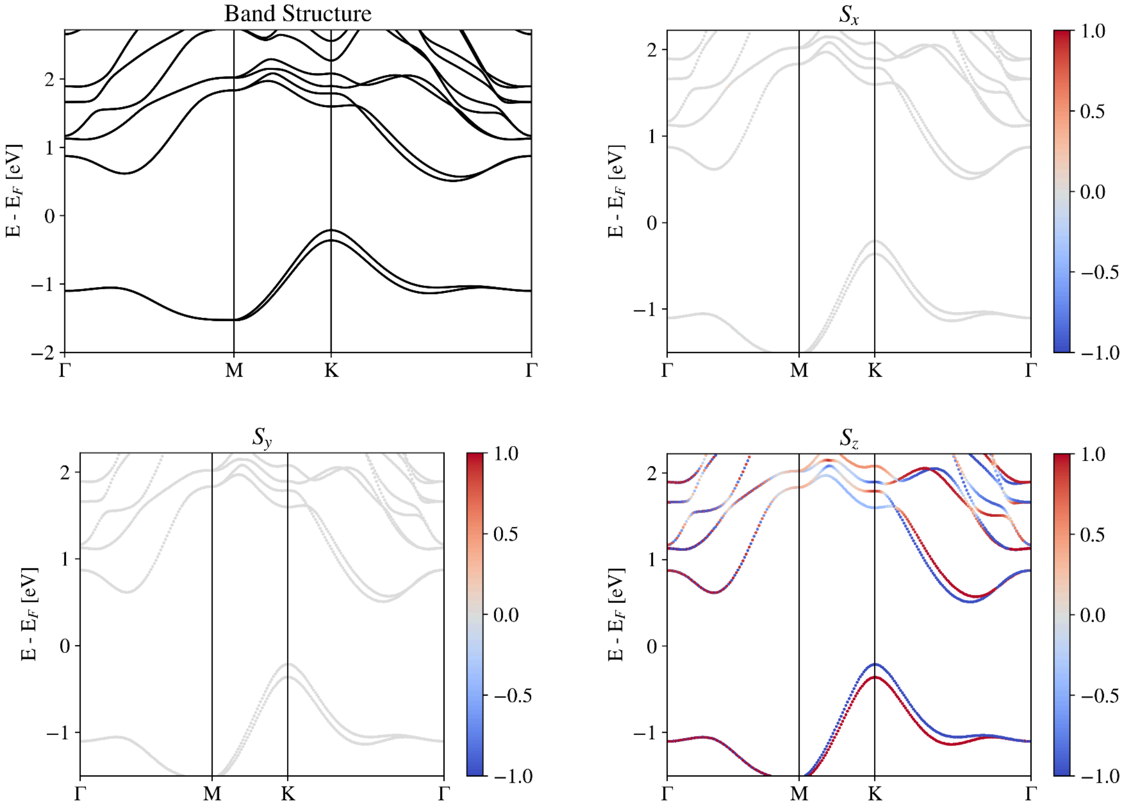


Figure 361: Band structure and spin polarization projections.

## 2.181 HfCl2-864f8b497185

- **Formula**: HfCl2
- **Structural Cluster**: AB2-4
- **Band gap (PBE)**: 0.891 eV
- **Energy above convex hull (C2DB)**: 0.007 eV
- **Space group symbol**: $P\bar{6}m2$
- **Space group number**: 187
- **Polar structure**: False

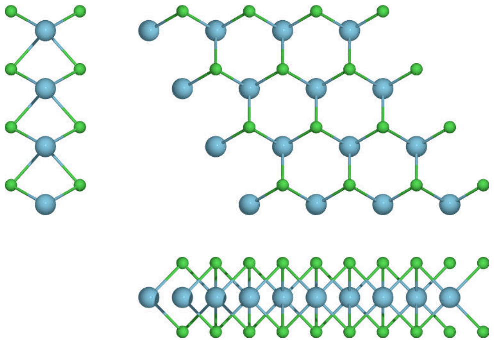

Figure 362: Structure representation

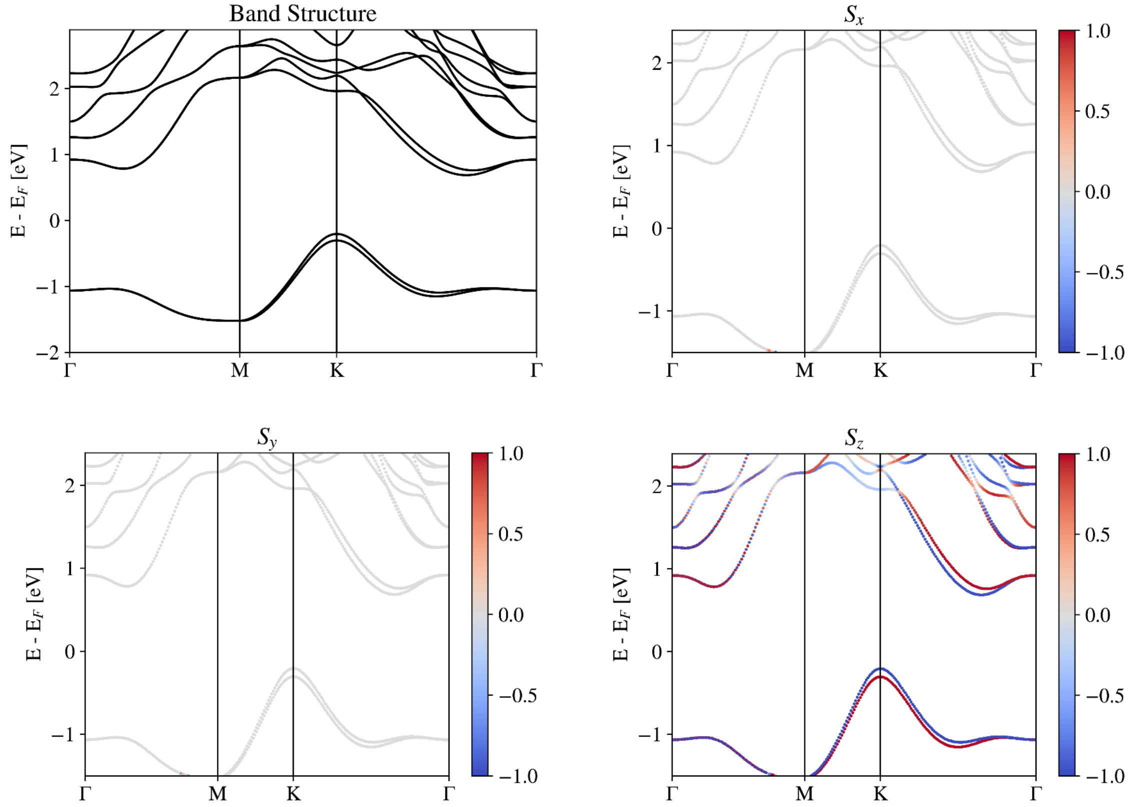


Figure 363: Band structure and spin polarization projections.

## 2.182 HfI2-05a69240794c

- **Formula**: HfI2
- **Structural Cluster**: AB2-4
- **Band gap (PBE)**: 0.616 eV
- **Energy above convex hull (C2DB)**: 0.114 eV
- **Space group symbol**: $P\bar{6}m2$
- **Space group number**: 187
- **Polar structure**: False

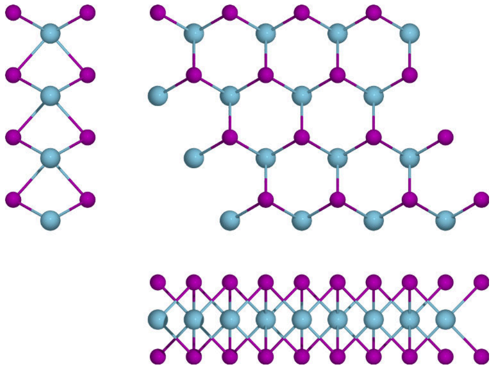

Figure 364: Structure representation

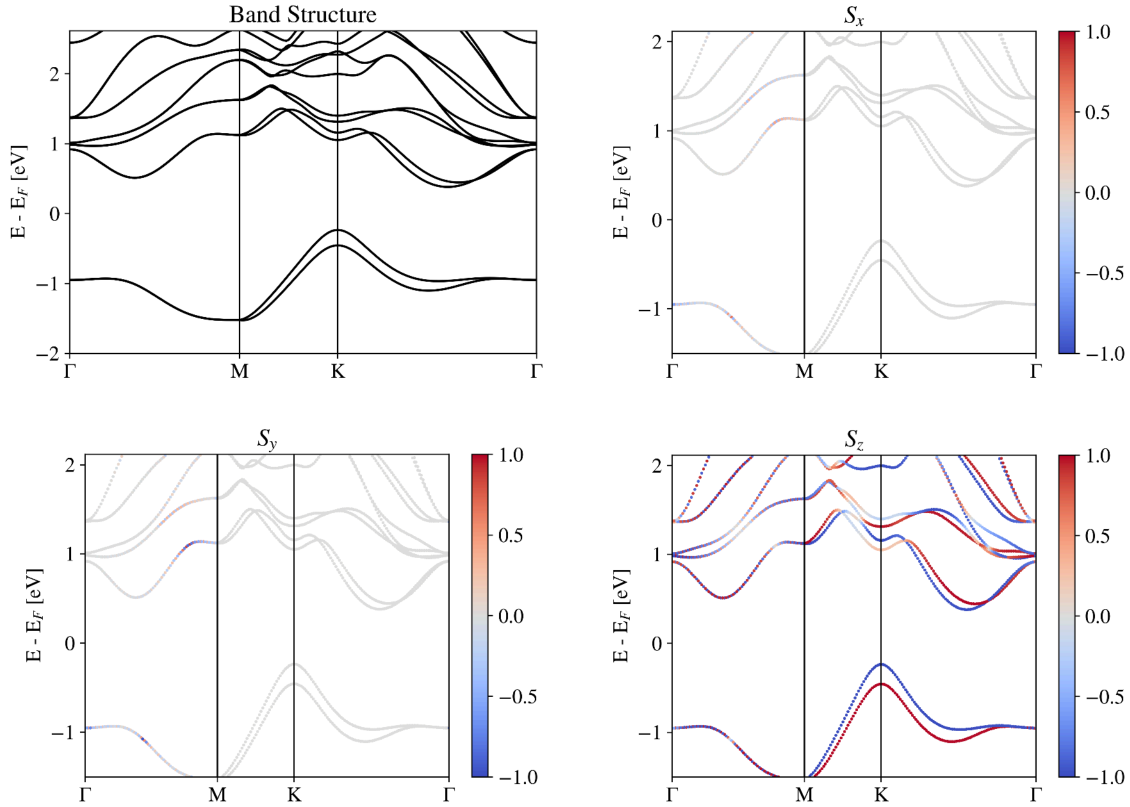


Figure 365: Band structure and spin polarization projections.

## 2.183 HfO2-512afaae525a

- **Formula**: HfO2
- **Structural Cluster**: AB2-4
- **Band gap (PBE)**: 1.903 eV
- **Energy above convex hull (C2DB)**: 0.77 eV
- **Space group symbol**: $P\bar{6}m2$
- **Space group number**: 187
- **Polar structure**: False

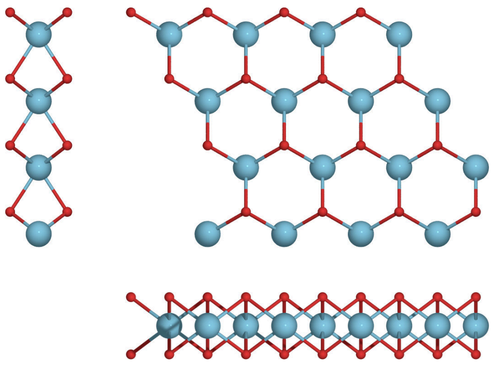

Figure 366: Structure representation

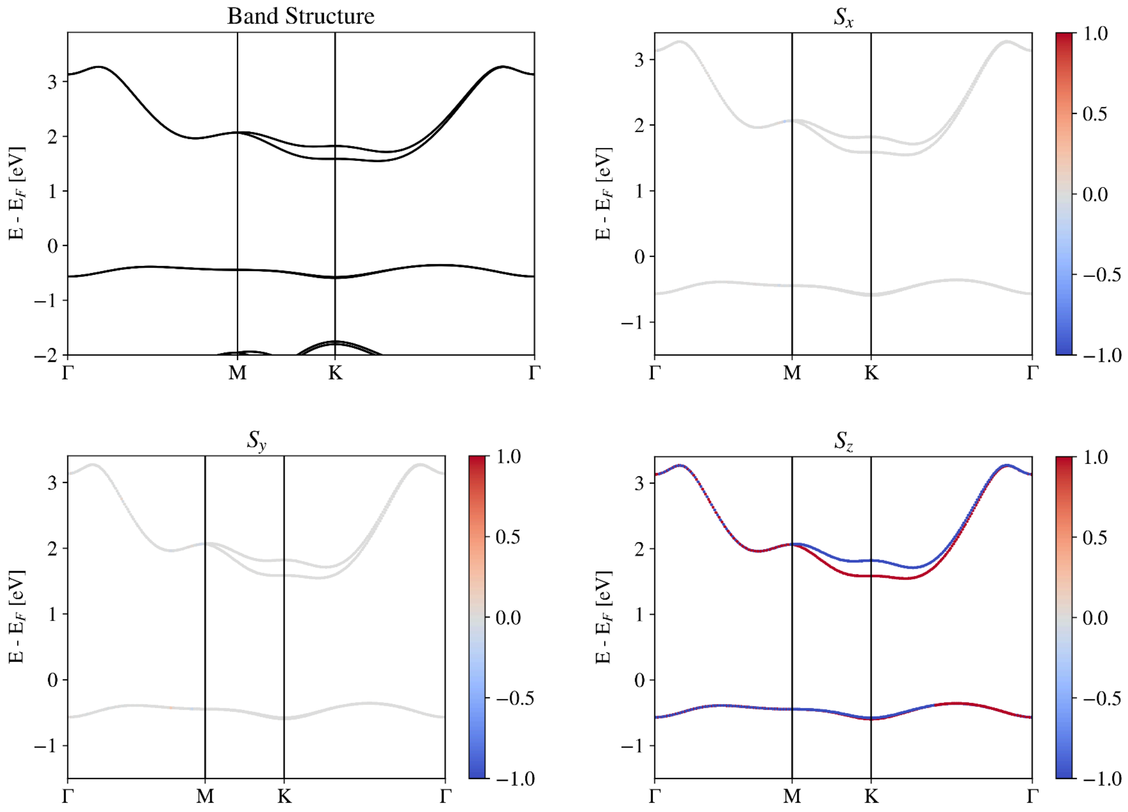


Figure 367: Band structure and spin polarization projections.

## 2.184 HfO2-6e4ac7453419

- **Formula**: HfO2
- **Structural Cluster**: AB2-11
- **Band gap (PBE)**: 4.494 eV
- **Energy above convex hull (C2DB)**: 0.51 eV
- **Space group symbol**: $P\bar{4}m2$
- **Space group number**: 115
- **Polar structure**: False

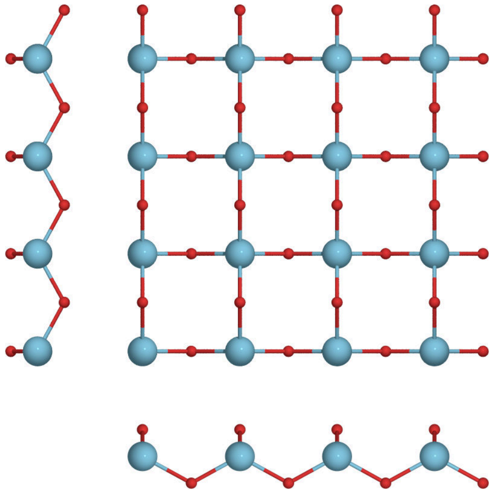

Figure 368: Structure representation

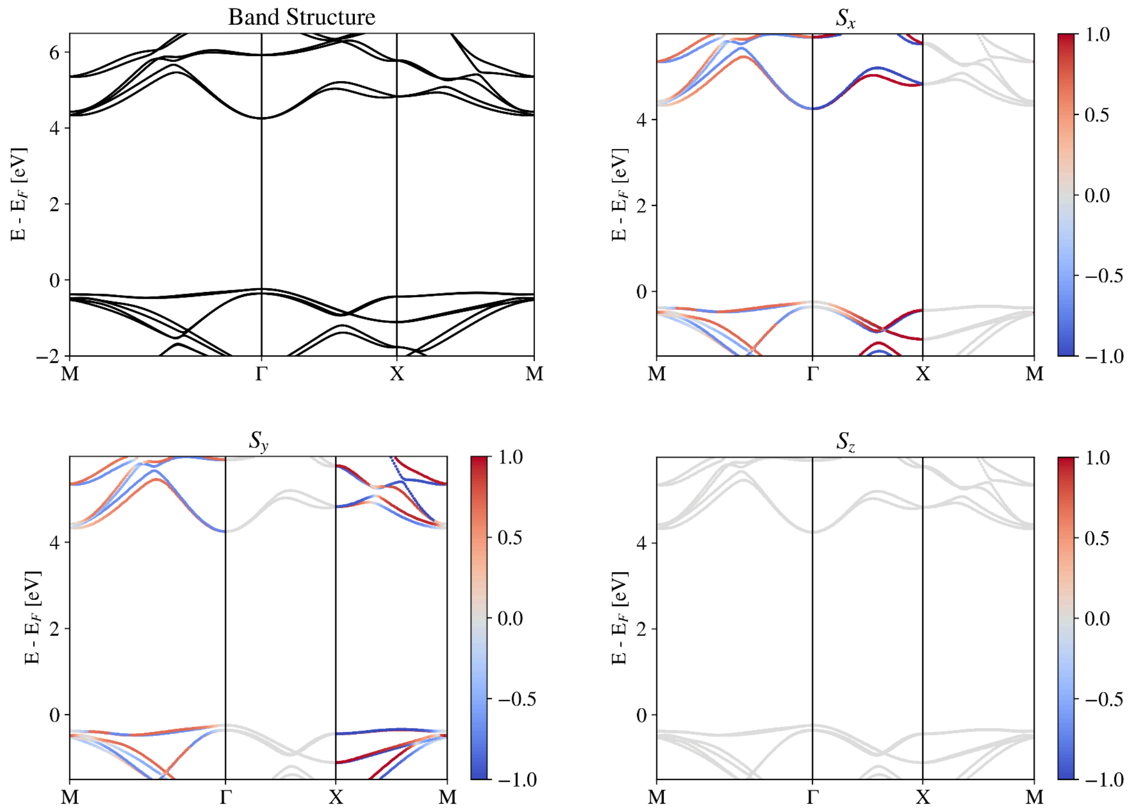


Figure 369: Band structure and spin polarization projections.

## 2.185 HfS2-2c5e65012601

- **Formula**: HfS2
- **Structural Cluster**: AB2-4
- **Band gap (PBE)**: 1.082 eV
- **Energy above convex hull (C2DB)**: 0.217 eV
- **Space group symbol**: $P\bar{6}m2$
- **Space group number**: 187
- **Polar structure**: False

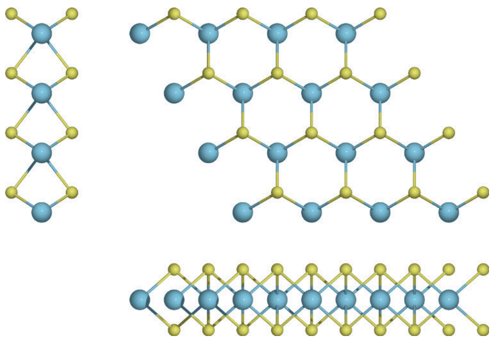

Figure 370: Structure representation

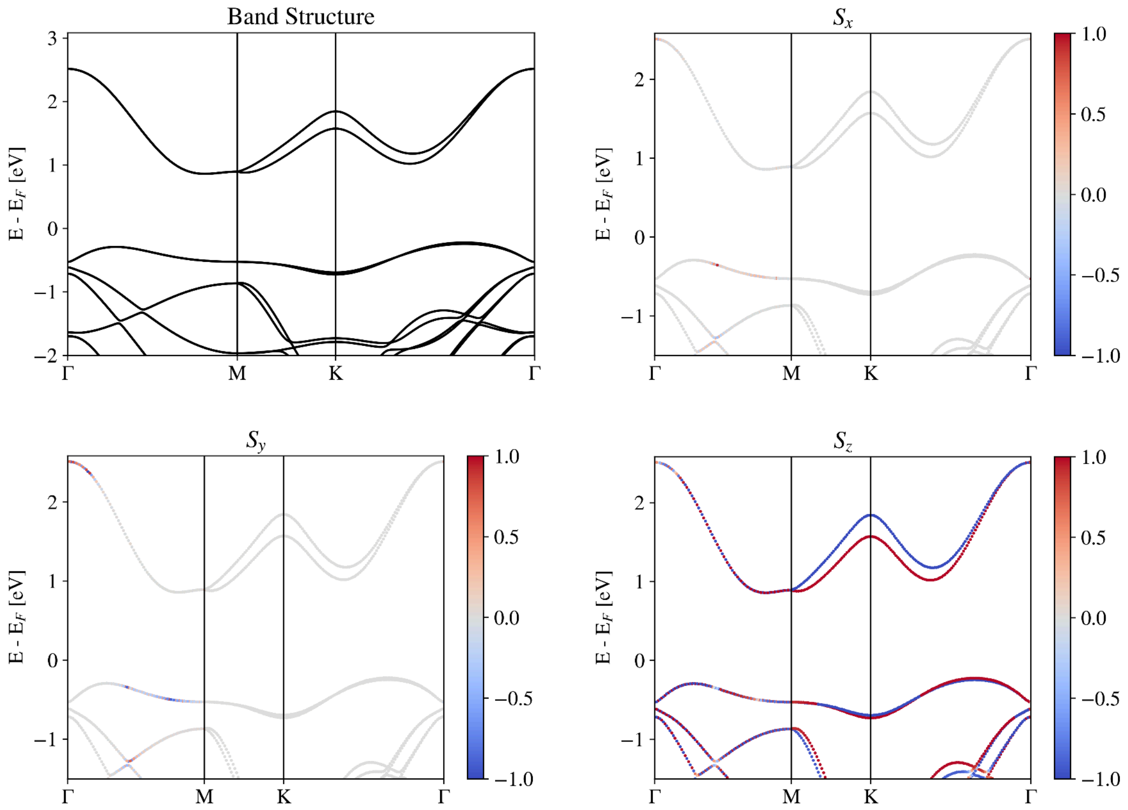


Figure 371: Band structure and spin polarization projections.

## 2.186 HfS2-3d4bfe131291

- **Formula**: HfS2
- **Structural Cluster**: AB2-11
- **Band gap (PBE)**: 2.11 eV
- **Energy above convex hull (C2DB)**: 0.333 eV
- **Space group symbol**: $P\bar{4}m2$
- **Space group number**: 115
- **Polar structure**: False

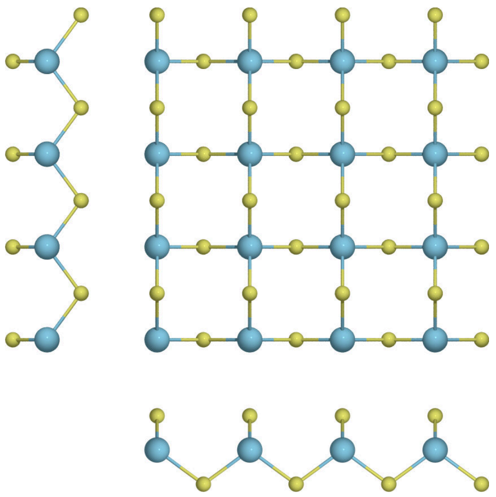

Figure 372: Structure representation

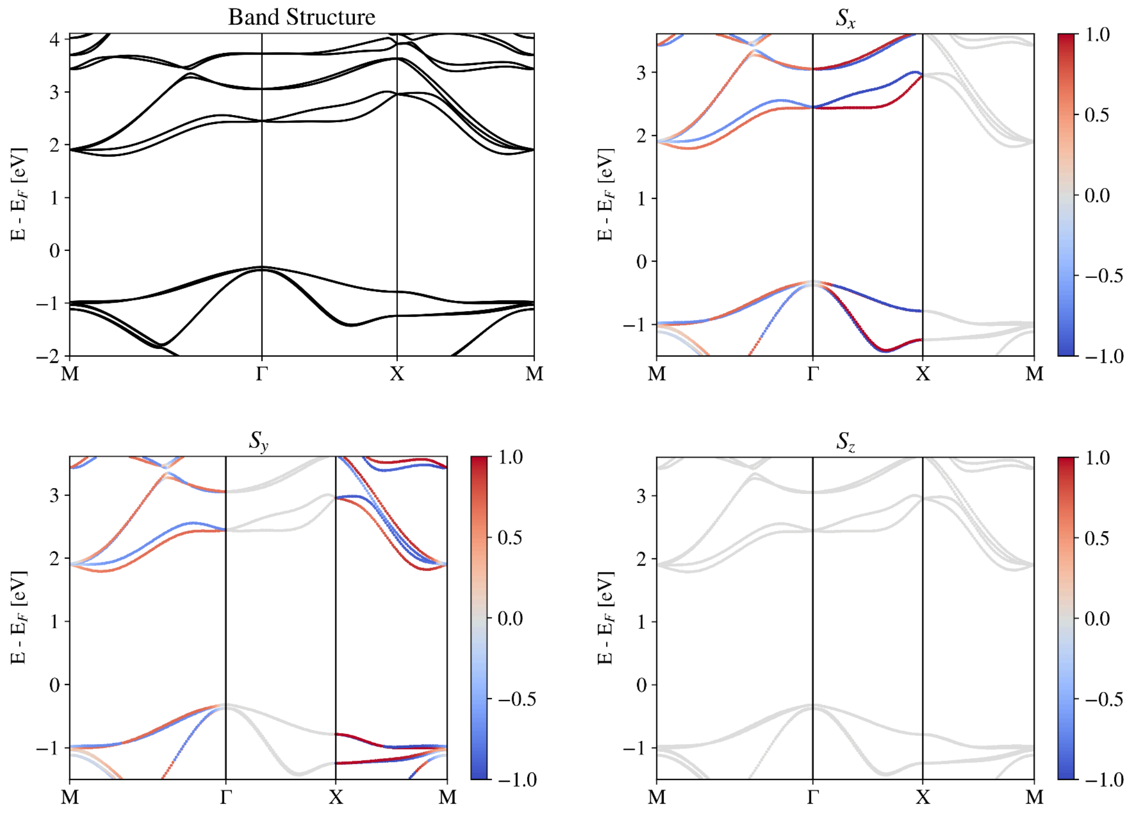


Figure 373: Band structure and spin polarization projections.

## 2.187 HfSSe-63618e5bf062

- **Formula**: HfSSe
- **Structural Cluster**: ABC-3
- **Band gap (PBE)**: 0.705 eV
- **Energy above convex hull (C2DB)**: 0.0 eV
- **Space group symbol**: $P3m1$
- **Space group number**: 156
- **Polar structure**: True

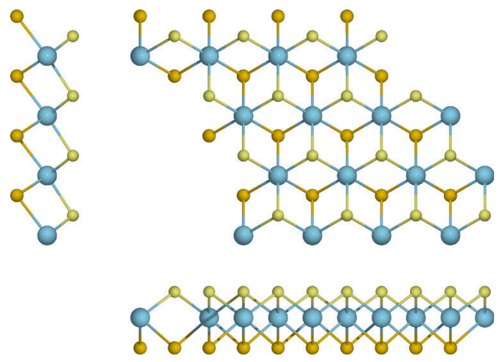

Figure 374: Structure representation

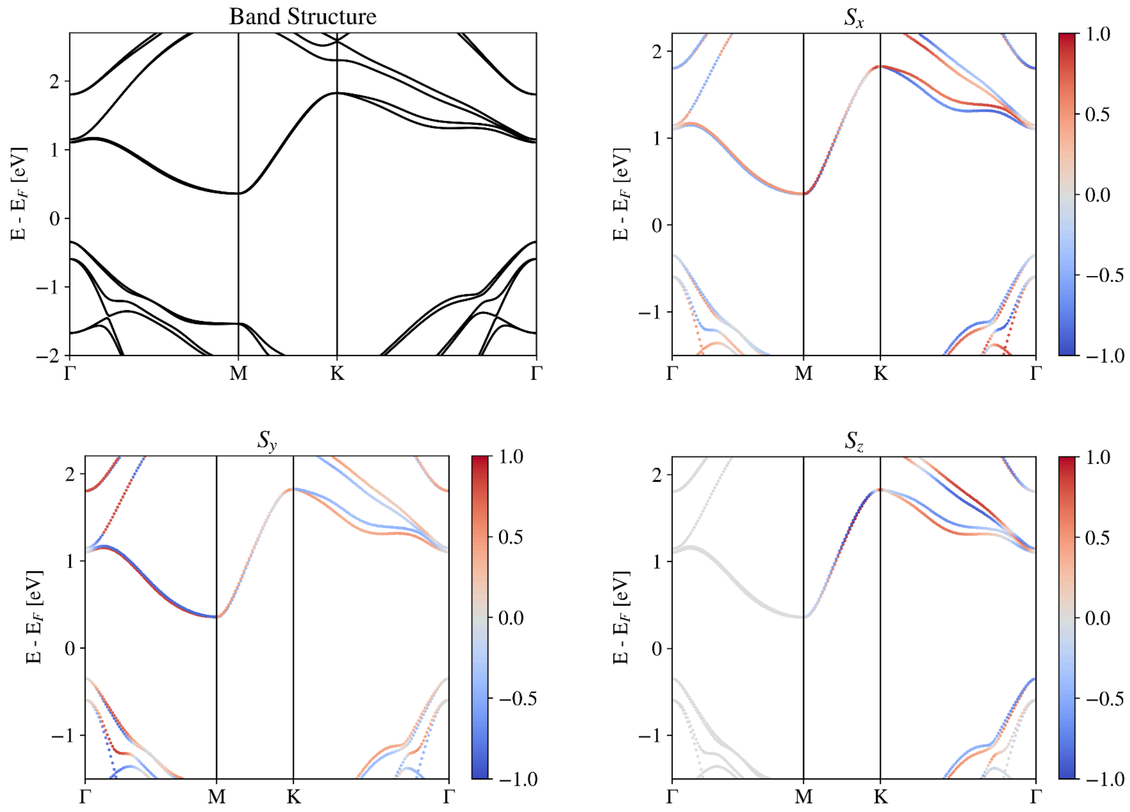


Figure 375: Band structure and spin polarization projections.

## 2.188 HfSSe-9afb20358166

- **Formula**: HfSSe
- **Structural Cluster**: ABC-4
- **Band gap (PBE)**: 0.91 eV
- **Energy above convex hull (C2DB)**: 0.193 eV
- **Space group symbol**: $P3m1$
- **Space group number**: 156
- **Polar structure**: True

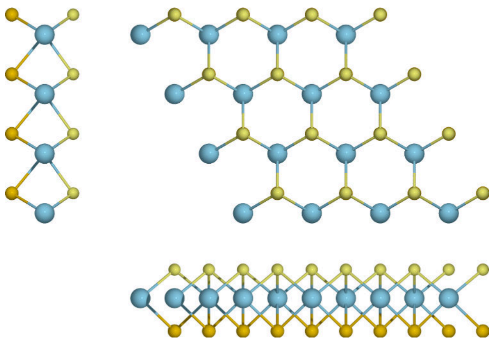

Figure 376: Structure representation

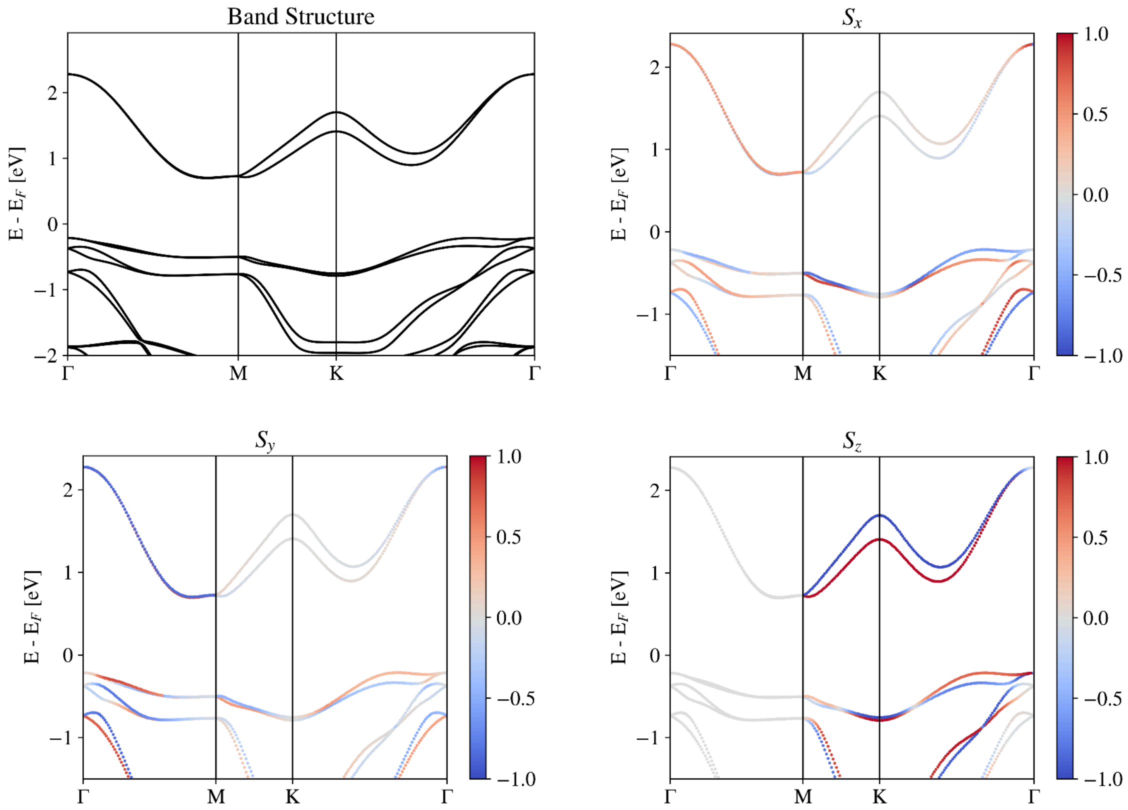


Figure 377: Band structure and spin polarization projections.

## 2.189 HfSe2-08401460f377

- **Formula**: HfSe2
- **Structural Cluster**: AB2-11
- **Band gap (PBE)**: 1.676 eV
- **Energy above convex hull (C2DB)**: 0.337 eV
- **Space group symbol**: $P\bar{4}m2$
- **Space group number**: 115
- **Polar structure**: False

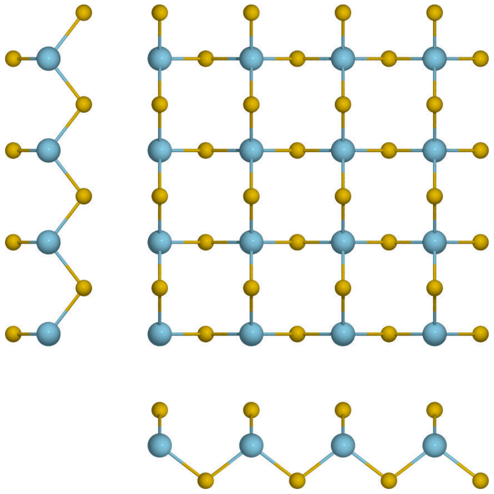

Figure 378: Structure representation

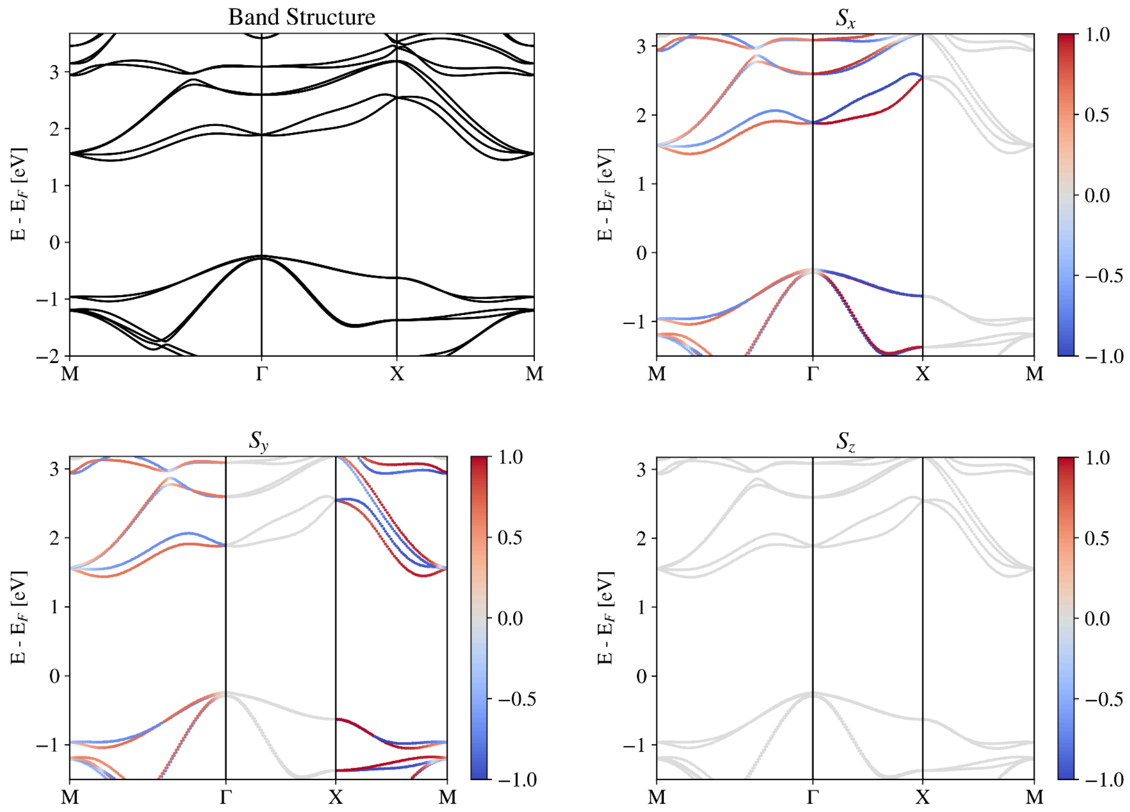


Figure 379: Band structure and spin polarization projections.

## 2.190 HfSe2-d2d9fee03594

- **Formula**: HfSe2
- **Structural Cluster**: AB2-4
- **Band gap (PBE)**: 0.827 eV
- **Energy above convex hull (C2DB)**: 0.174 eV
- **Space group symbol**: $P\bar{6}m2$
- **Space group number**: 187
- **Polar structure**: False

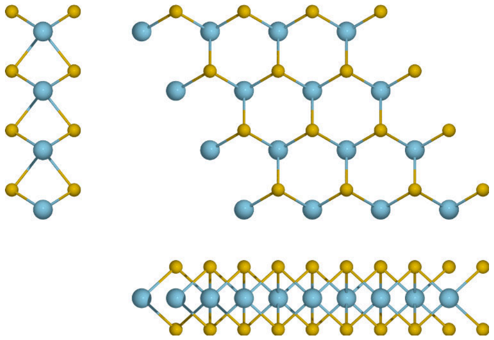

Figure 380: Structure representation

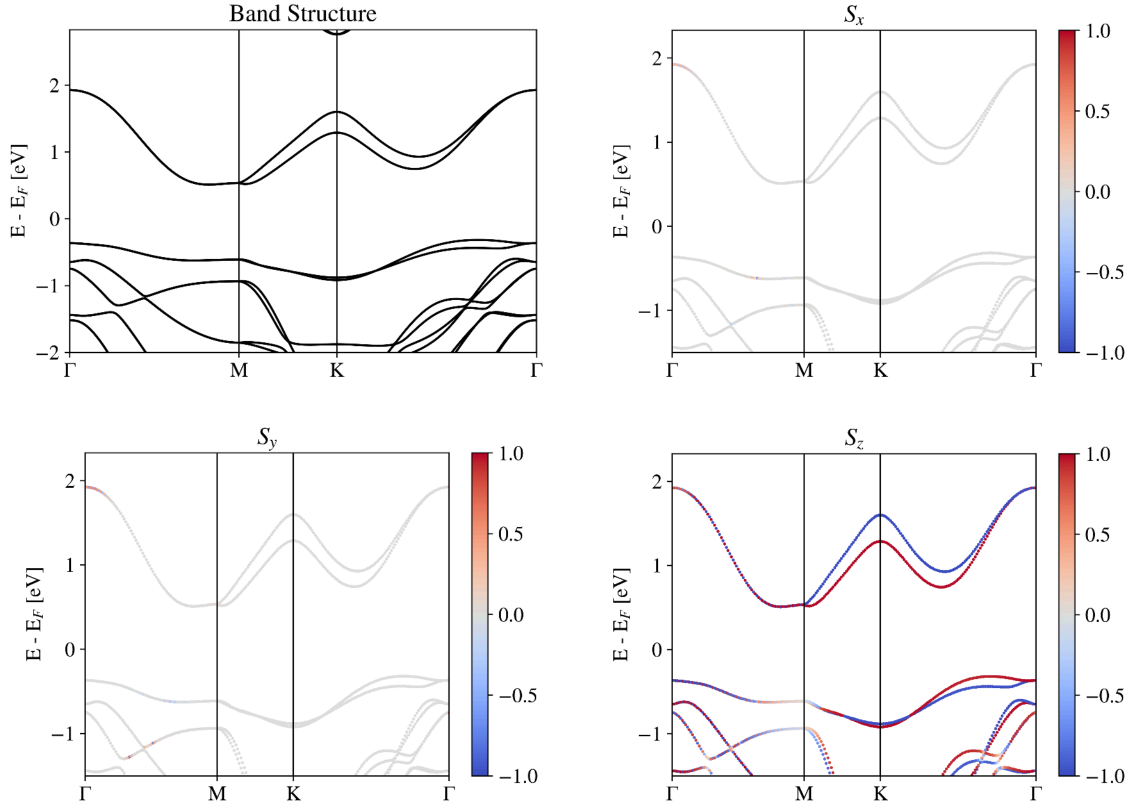


Figure 381: Band structure and spin polarization projections.

## 2.191 HfSeTe-305c779b8752

- **Formula**: HfSeTe
- **Structural Cluster**: ABC-4
- **Band gap (PBE)**: 0.16 eV
- **Energy above convex hull (C2DB)**: 0.149 eV
- **Space group symbol**: $P3m1$
- **Space group number**: 156
- **Polar structure**: True

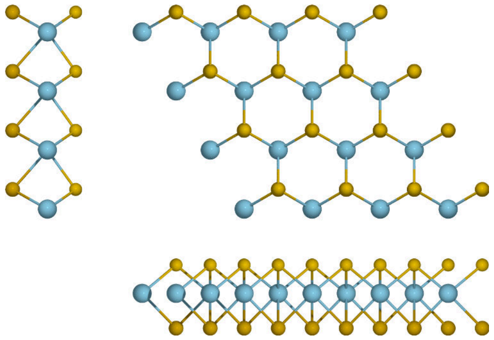

Figure 382: Structure representation

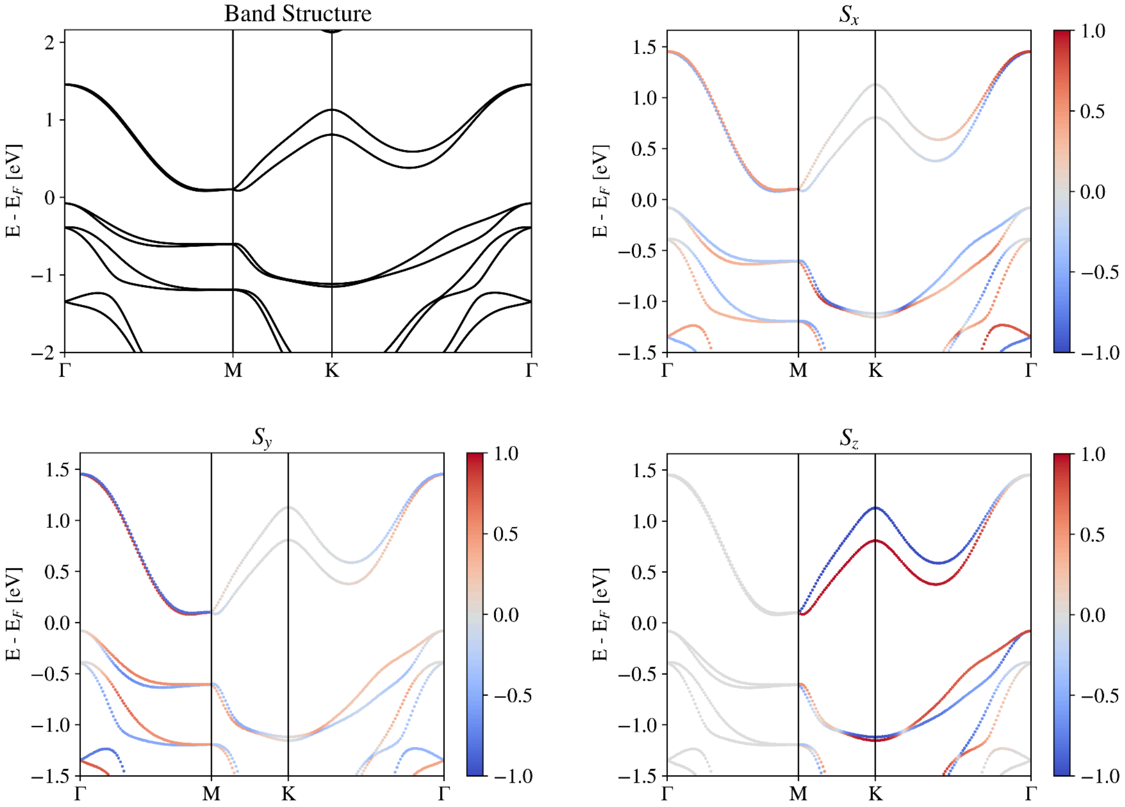


Figure 383: Band structure and spin polarization projections.

### 2.192 HfTe2-1e2c6946ca41

- **Formula**: HfTe2
- **Structural Cluster**: AB2-11
- **Band gap (PBE)**: 1.01 eV
- **Energy above convex hull (C2DB)**: 0.371 eV
- **Space group symbol**: $P\bar{4}m2$
- **Space group number**: 115
- **Polar structure**: False

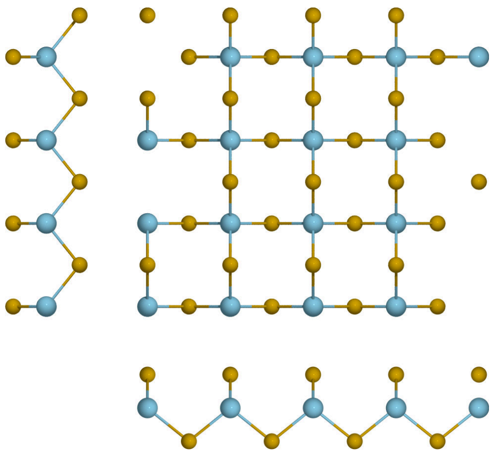

Figure 384: Structure representation

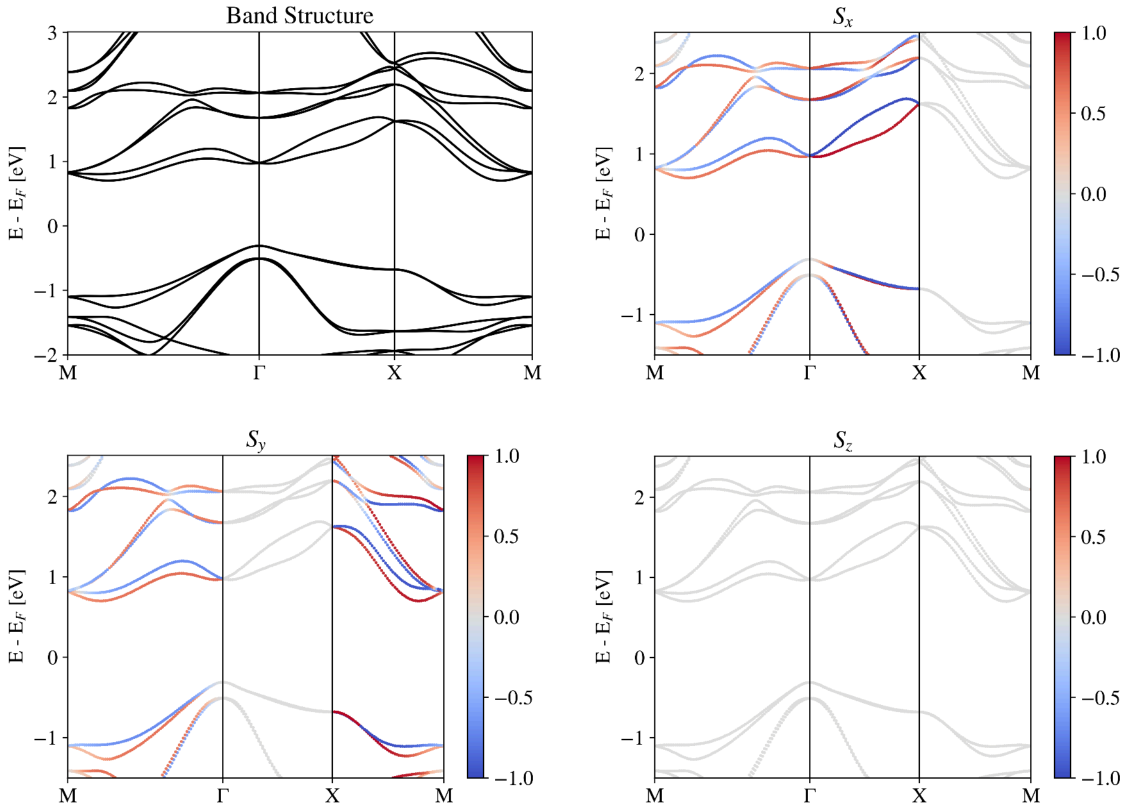


Figure 385: Band structure and spin polarization projections.

## 2.193 HfTe2-59c0e014651d

- **Formula**: HfTe2
- **Structural Cluster**: AB2-4
- **Band gap (PBE)**: 0.147 eV
- **Energy above convex hull (C2DB)**: 0.133 eV
- **Space group symbol**: $P\bar{6}m2$
- **Space group number**: 187
- **Polar structure**: False

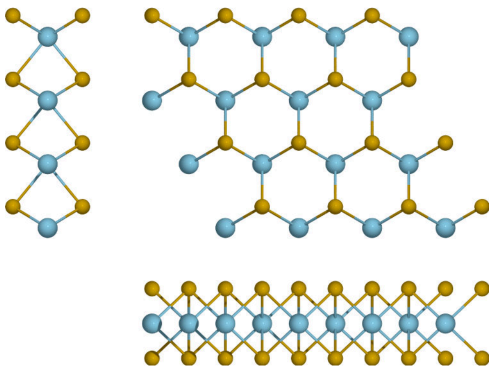

Figure 386: Structure representation

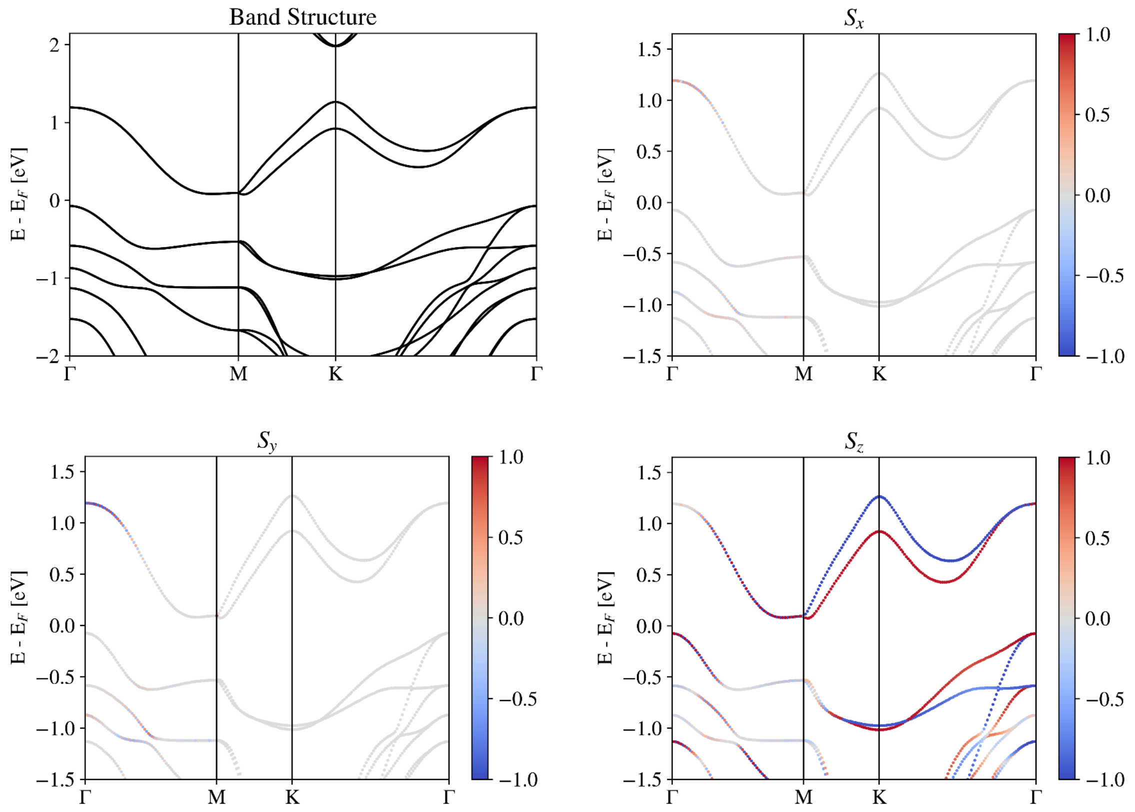


Figure 387: Band structure and spin polarization projections.

## 2.194 HfTi3S8-fde2d81d10df

- **Formula**: HfTi3S8
- **Structural Cluster**: AB3C8-22
- **Band gap (PBE)**: 0.848 eV
- **Energy above convex hull (C2DB)**: 0.169 eV
- **Space group symbol**: $P1$
- **Space group number**: 1
- **Polar structure**: True

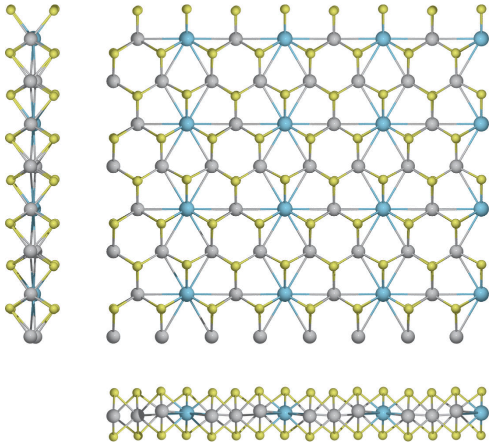

Figure 388: Structure representation

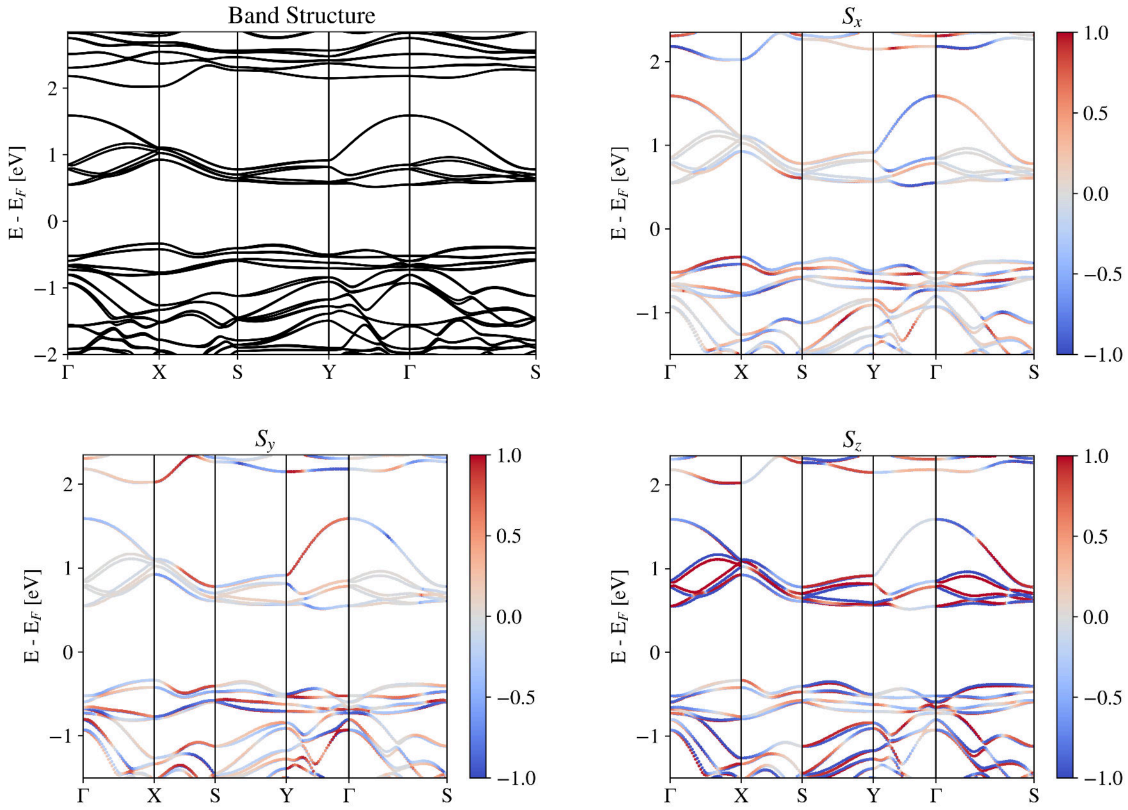


Figure 389: Band structure and spin polarization projections.

## 2.195 HfTi3Se8-c55716558616

- **Formula**: HfTi3Se8
- **Structural Cluster**: AB3C8-22
- **Band gap (PBE)**: 0.589 eV
- **Energy above convex hull (C2DB)**: 0.137 eV
- **Space group symbol**: $P1$
- **Space group number**: 1
- **Polar structure**: True

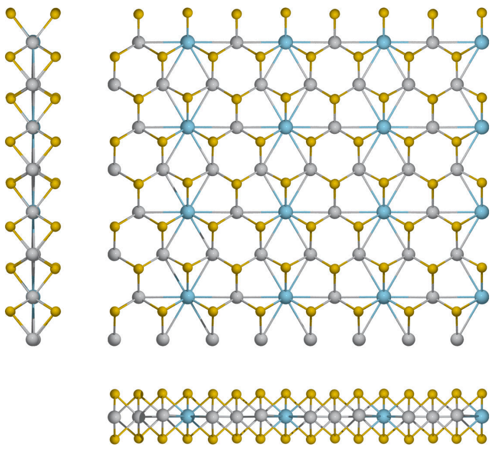

Figure 390: Structure representation

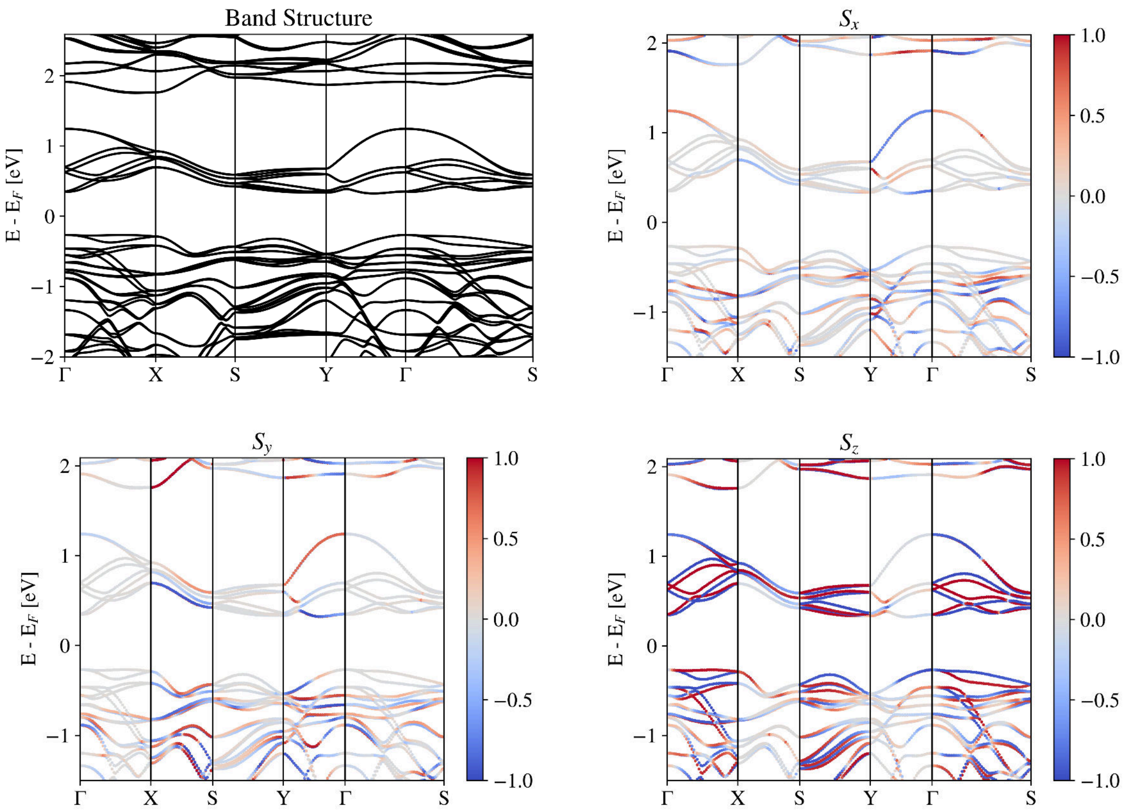


Figure 391: Band structure and spin polarization projections.

## 2.196 HfZr3S8-78bb1ac31c01

- **Formula**: HfZr3S8
- **Structural Cluster**: AB3C8-22
- **Band gap (PBE)**: 1.145 eV
- **Energy above convex hull (C2DB)**: 0.193 eV
- **Space group symbol**: $P1$
- **Space group number**: 1
- **Polar structure**: True

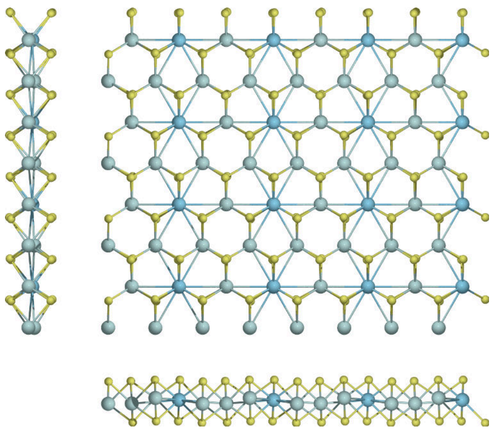

Figure 392: Structure representation

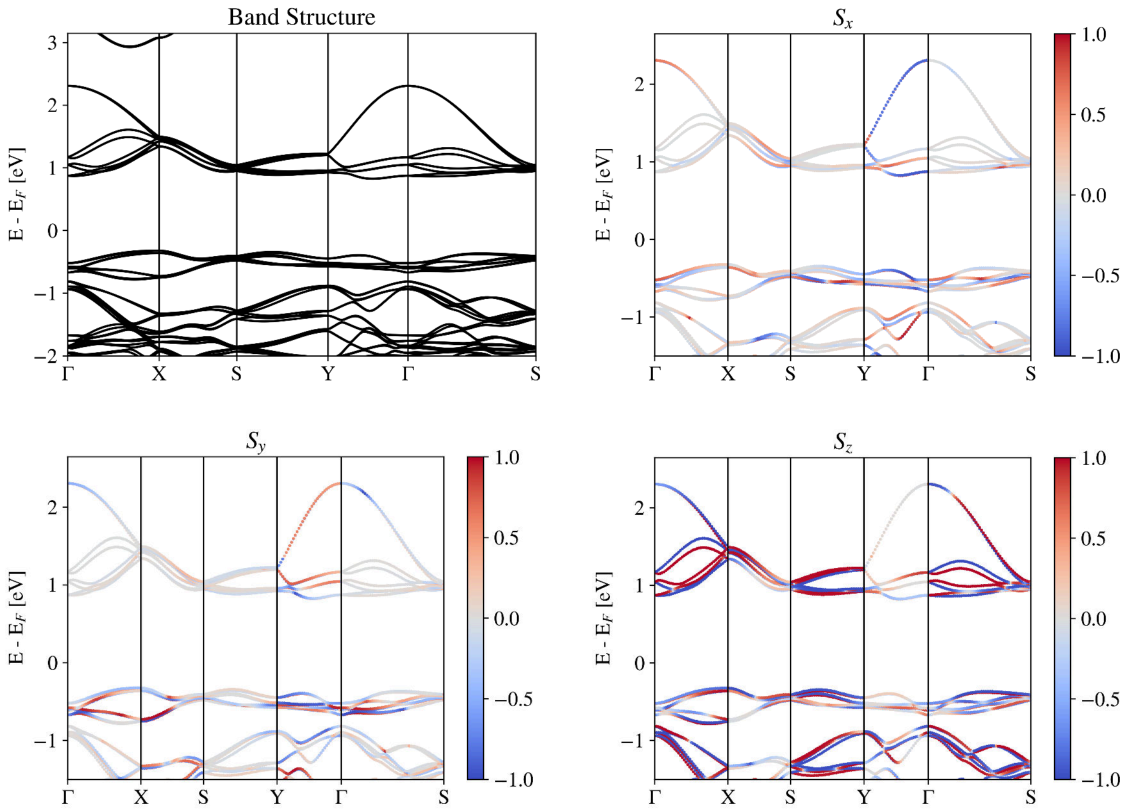


Figure 393: Band structure and spin polarization projections.

## 2.197 HfZr3Se8-70e7ab872359

- **Formula**: HfZr3Se8
- **Structural Cluster**: AB3C8-22
- **Band gap (PBE)**: 0.819 eV
- **Energy above convex hull (C2DB)**: 0.15 eV
- **Space group symbol**: $P1$
- **Space group number**: 1
- **Polar structure**: True

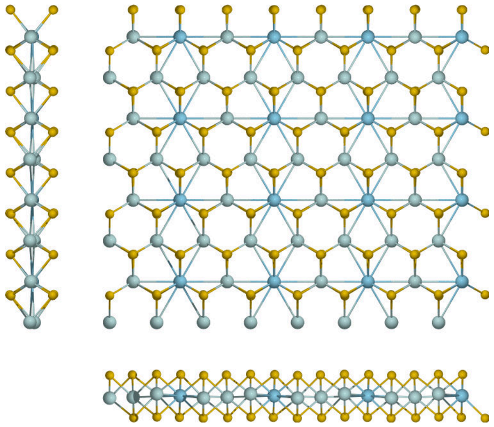

Figure 394: Structure representation

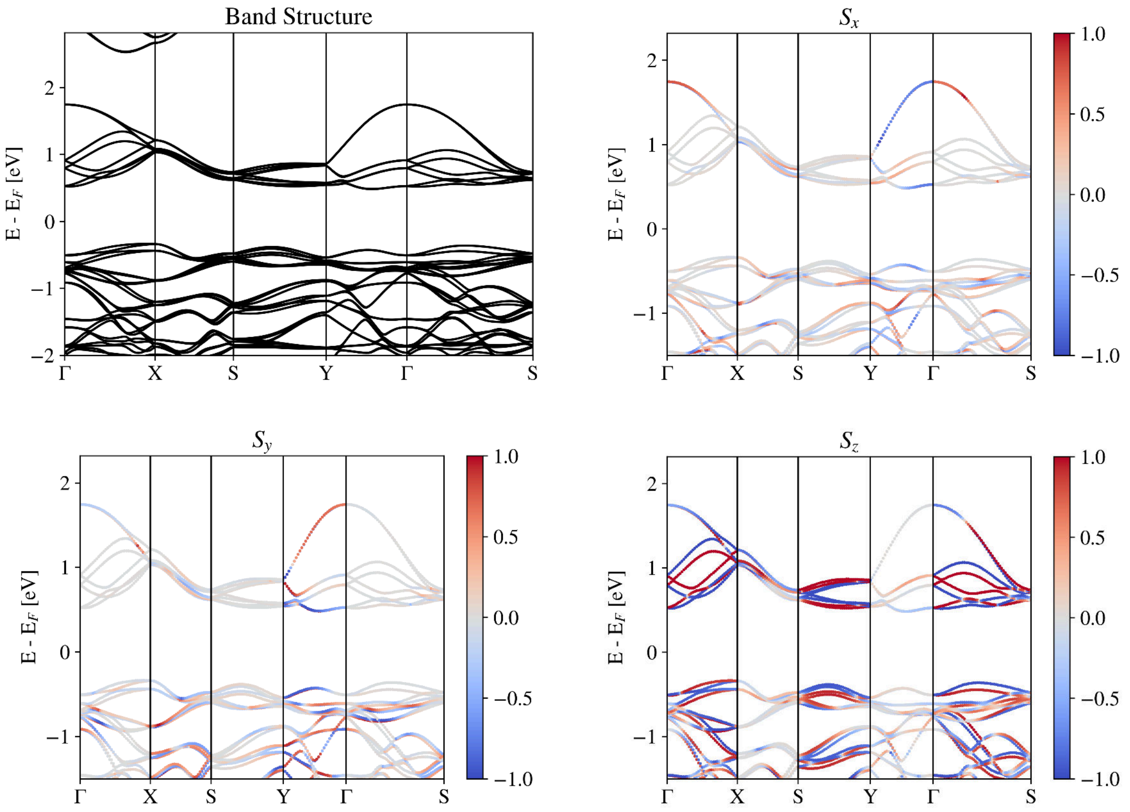


Figure 395: Band structure and spin polarization projections.

## 2.198 HfZr3Te8-916e19eae465

- **Formula**: HfZr3Te8
- **Structural Cluster**: AB3C8-22
- **Band gap (PBE)**: 0.242 eV
- **Energy above convex hull (C2DB)**: 0.115 eV
- **Space group symbol**: $P1$
- **Space group number**: 1
- **Polar structure**: True

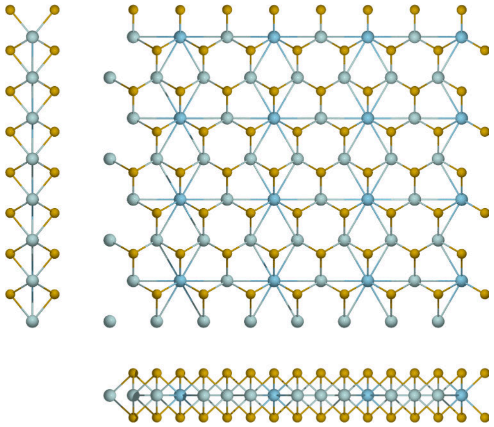

Figure 396: Structure representation

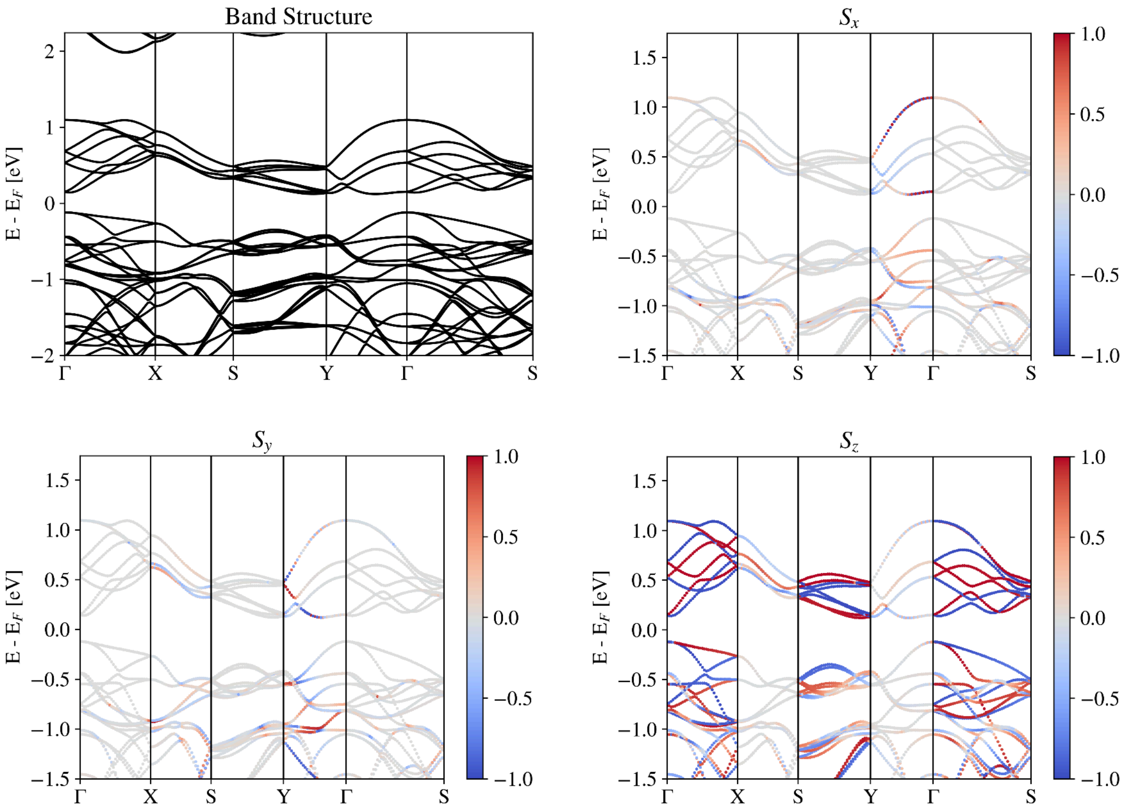


Figure 397: Band structure and spin polarization projections.

## 2.199 Hg2I2-f7e70d2b90ad

- **Formula**: Hg2I2
- **Structural Cluster**: AB-5
- **Band gap (PBE)**: 1.265 eV
- **Energy above convex hull (C2DB)**: 0.0 eV
- **Space group symbol**: $P1$
- **Space group number**: 1
- **Polar structure**: True

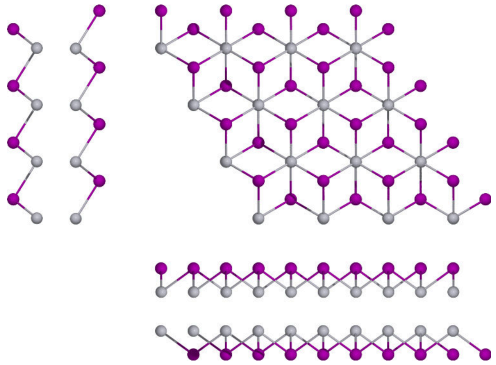

Figure 398: Structure representation

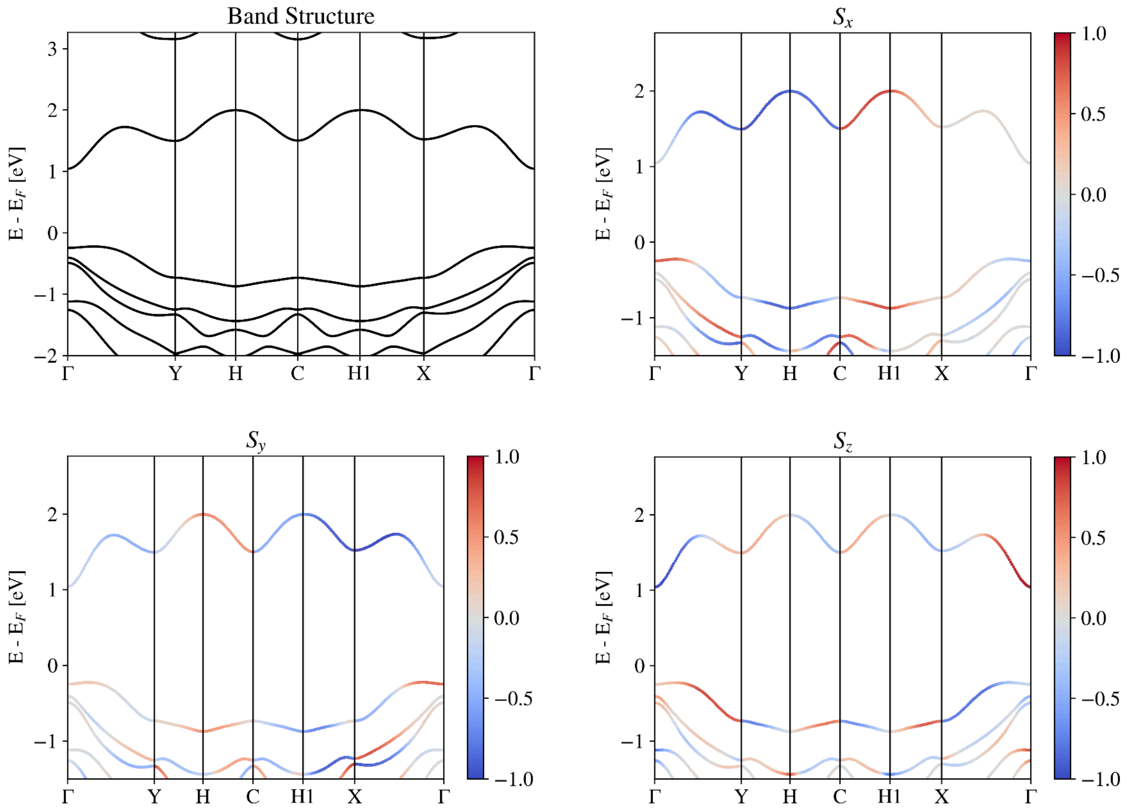


Figure 399: Band structure and spin polarization projections.

## 2.200 Hg2P2S6-b81eb586acfd

- **Formula**: Hg2P2S6
- **Structural Cluster**: ABC3-16
- **Band gap (PBE)**: 0.981 eV
- **Energy above convex hull (C2DB)**: 0.0 eV
- **Space group symbol**: $P1$
- **Space group number**: 1
- **Polar structure**: True

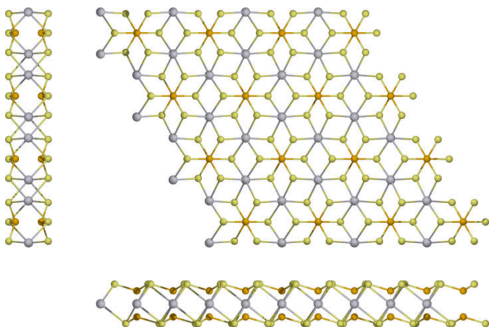

Figure 400: Structure representation

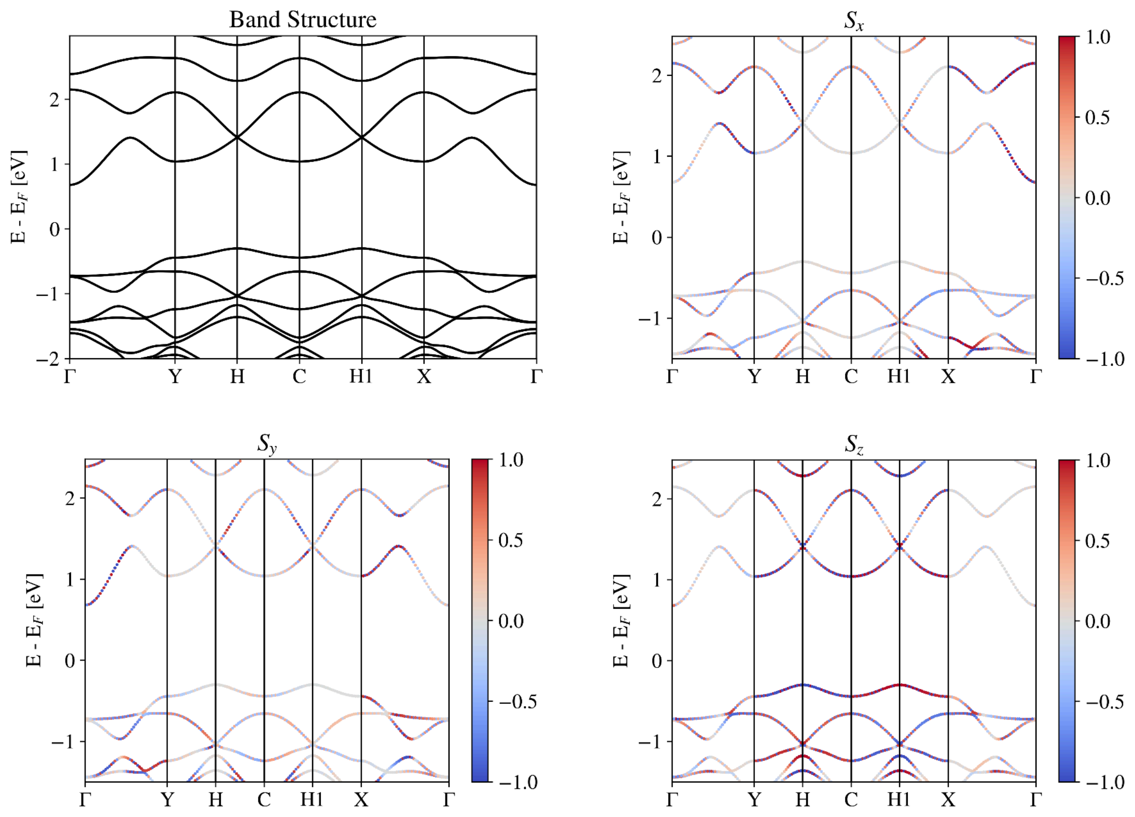


Figure 401: Band structure and spin polarization projections.

## 2.201 HgBr2-978e95bef964

- **Formula**: HgBr2
- **Structural Cluster**: AB2-11
- **Band gap (PBE)**: 1.947 eV
- **Energy above convex hull (C2DB)**: 0.001 eV
- **Space group symbol**: $P\bar{4}m2$
- **Space group number**: 115
- **Polar structure**: False

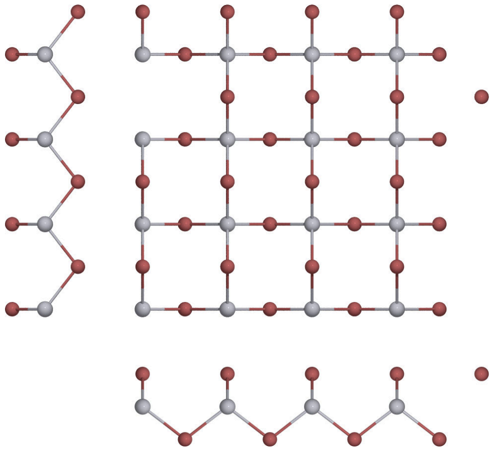

Figure 402: Structure representation

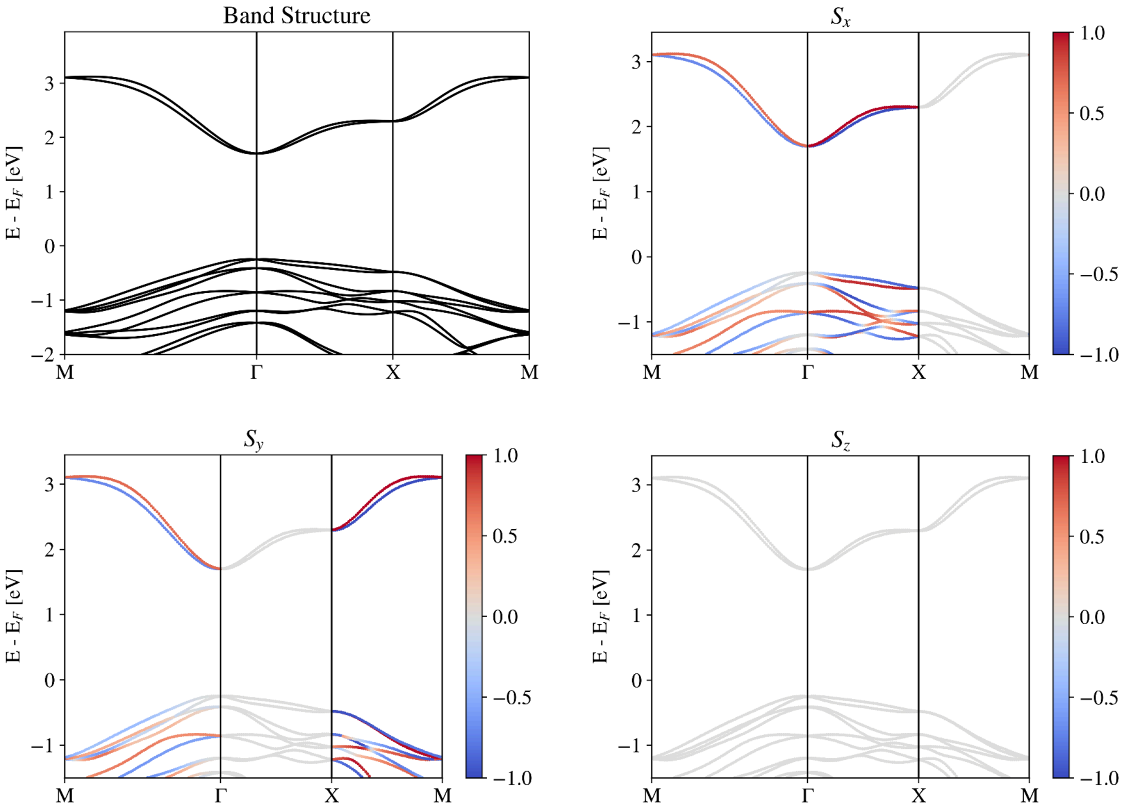


Figure 403: Band structure and spin polarization projections.

## 2.202 HgBr2-9965e7e32aa2

- **Formula**: HgBr2
- **Structural Cluster**: AB2-4
- **Band gap (PBE)**: 1.521 eV
- **Energy above convex hull (C2DB)**: 0.12 eV
- **Space group symbol**: $P\bar{6}m2$
- **Space group number**: 187
- **Polar structure**: False

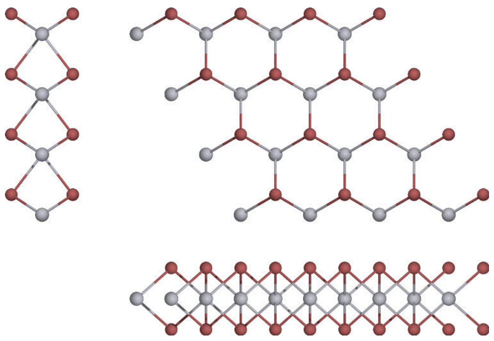

Figure 404: Structure representation

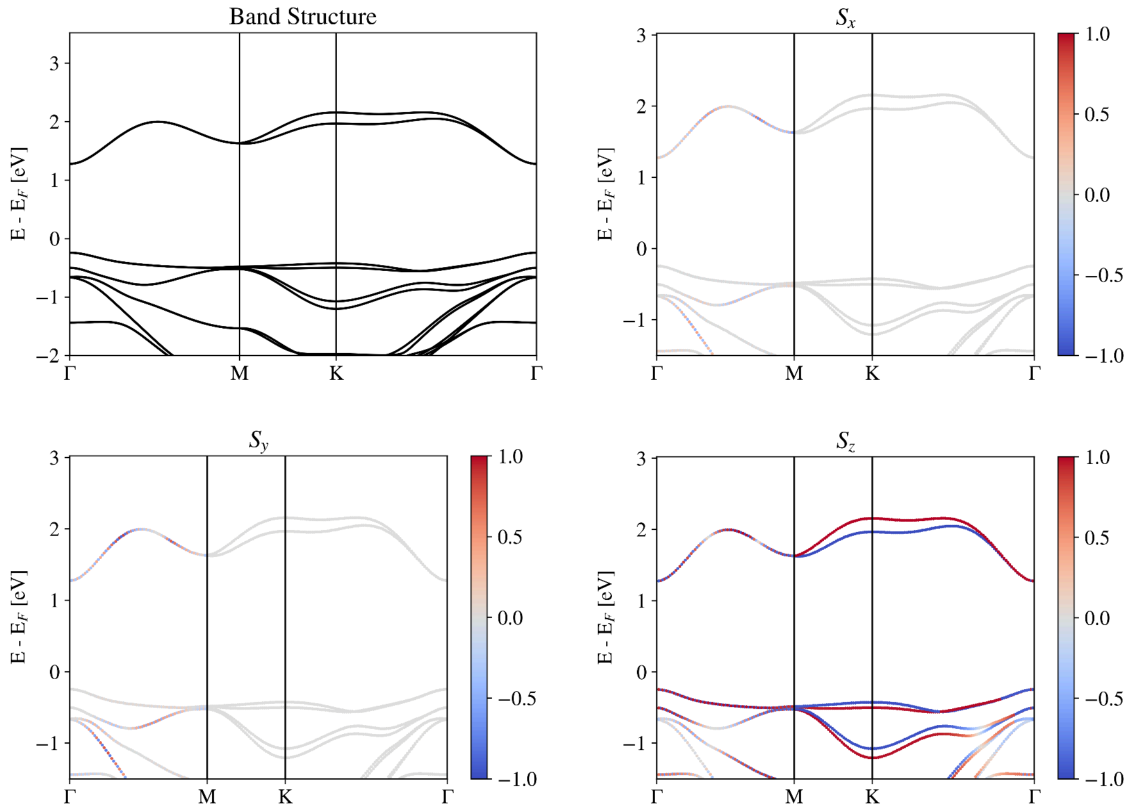


Figure 405: Band structure and spin polarization projections.

## 2.203 HgCl2-6cbe2e585099

- **Formula**: HgCl2
- **Structural Cluster**: AB2-4
- **Band gap (PBE)**: 2.023 eV
- **Energy above convex hull (C2DB)**: 0.131 eV
- **Space group symbol**: $P\bar{6}m2$
- **Space group number**: 187
- **Polar structure**: False

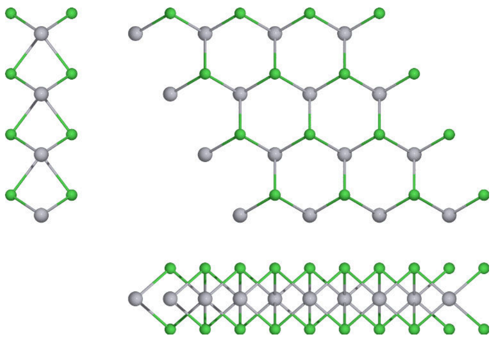

Figure 406: Structure representation

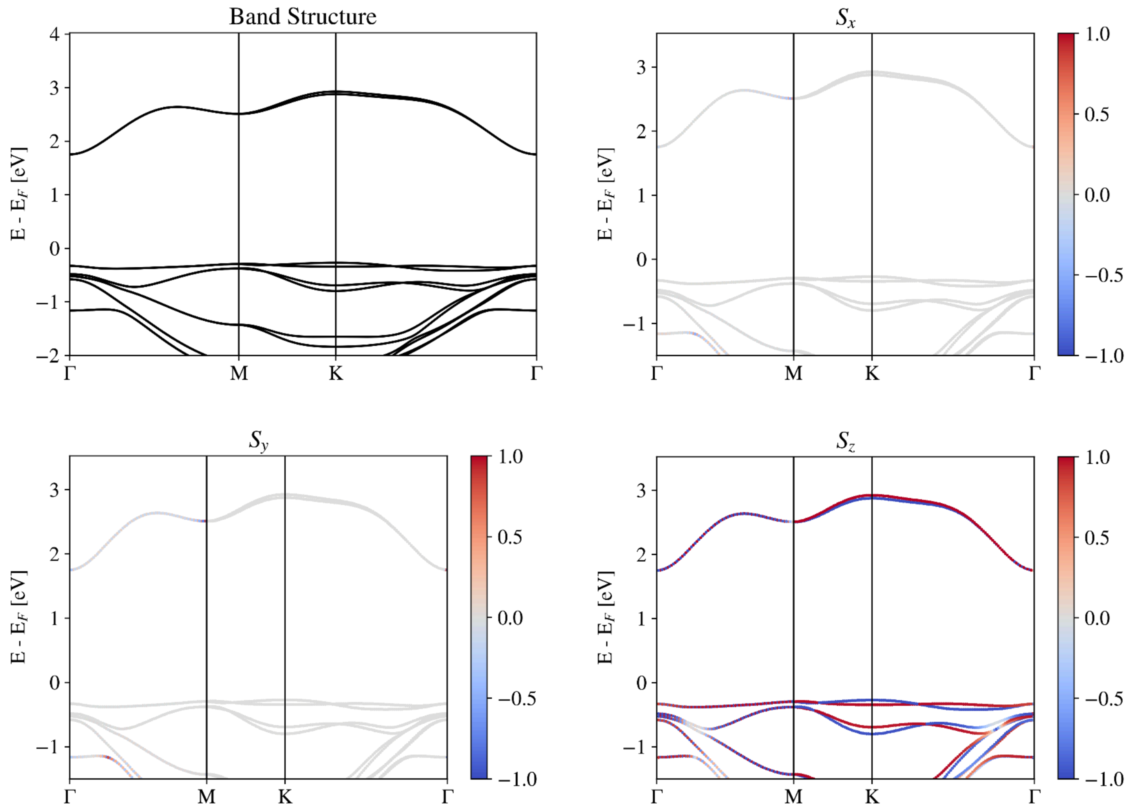


Figure 407: Band structure and spin polarization projections.

## 2.204 HgCl2-ce3ed4728e8f

- **Formula**: HgCl2
- **Structural Cluster**: AB2-11
- **Band gap (PBE)**: 2.404 eV
- **Energy above convex hull (C2DB)**: 0.033 eV
- **Space group symbol**: $P\bar{4}m2$
- **Space group number**: 115
- **Polar structure**: False

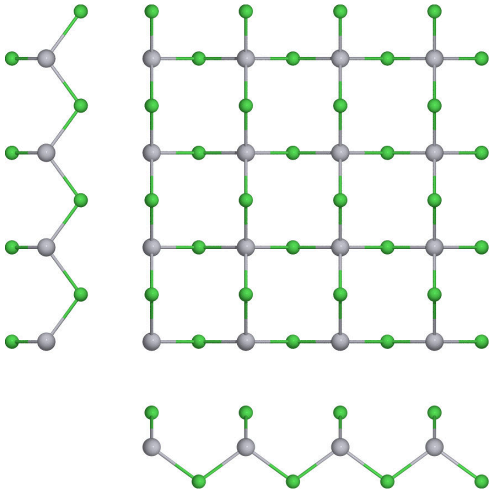

Figure 408: Structure representation

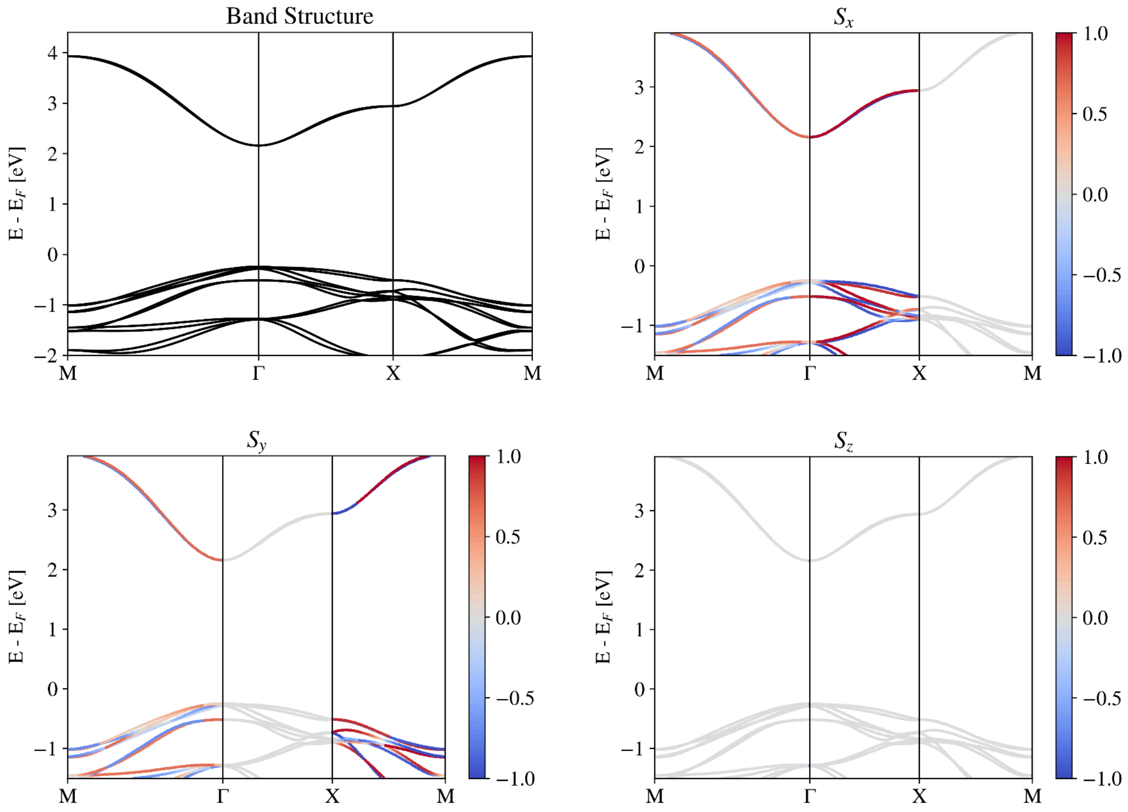


Figure 409: Band structure and spin polarization projections.

## 2.205 HgF2-f5965c8b3d89

- **Formula**: HgF2
- **Structural Cluster**: AB2-11
- **Band gap (PBE)**: 1.995 eV
- **Energy above convex hull (C2DB)**: 0.162 eV
- **Space group symbol**: $P\bar{4}m2$
- **Space group number**: 115
- **Polar structure**: False

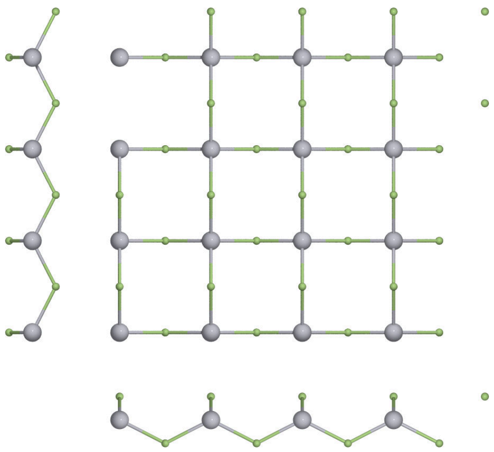

Figure 410: Structure representation

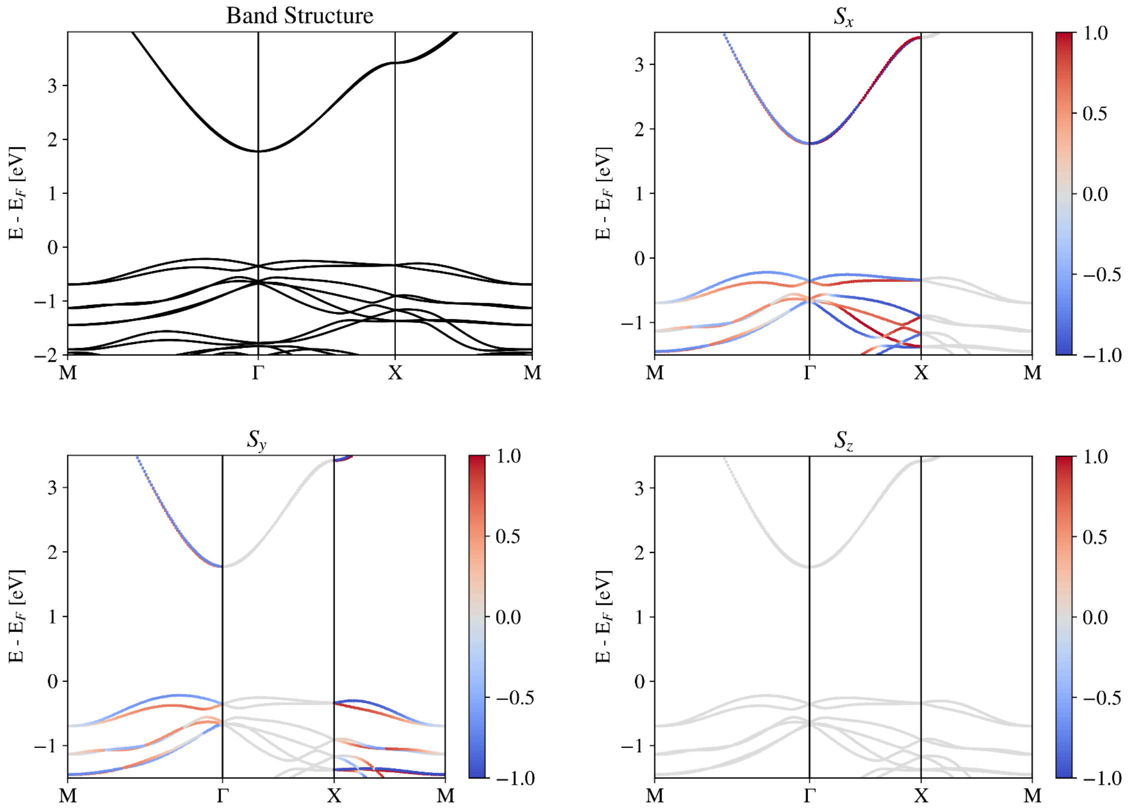


Figure 411: Band structure and spin polarization projections.

## 2.206 HgI2-0ff7ee261fec

- **Formula**: HgI2
- **Structural Cluster**: AB2-4
- **Band gap (PBE)**: 0.666 eV
- **Energy above convex hull (C2DB)**: 0.183 eV
- **Space group symbol**: $P\bar{6}m2$
- **Space group number**: 187
- **Polar structure**: False

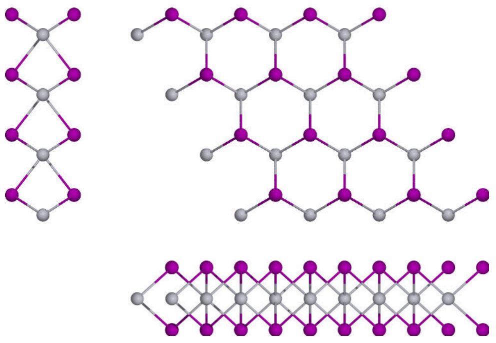

Figure 412: Structure representation

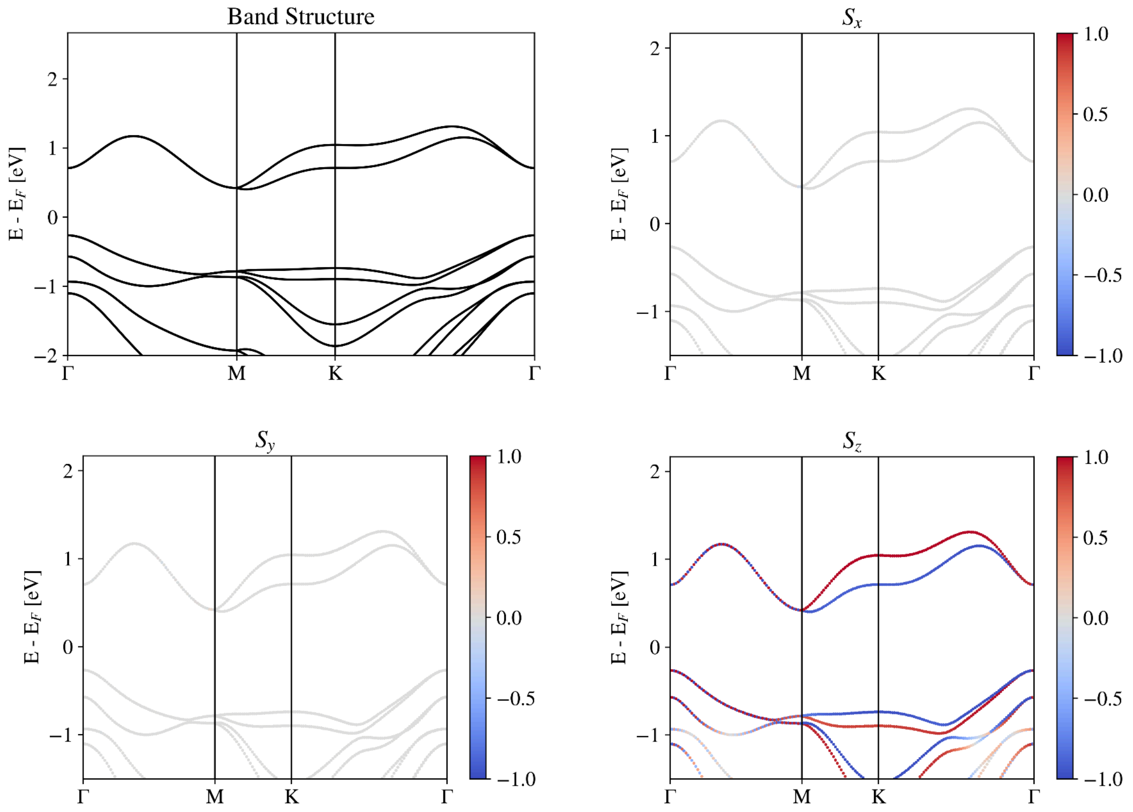


Figure 413: Band structure and spin polarization projections.

## 2.207 HgI2-7c2657e15a6f

- **Formula**: HgI2
- **Structural Cluster**: AB2-11
- **Band gap (PBE)**: 1.512 eV
- **Energy above convex hull (C2DB)**: 0.0 eV
- **Space group symbol**: $P\bar{4}m2$
- **Space group number**: 115
- **Polar structure**: False

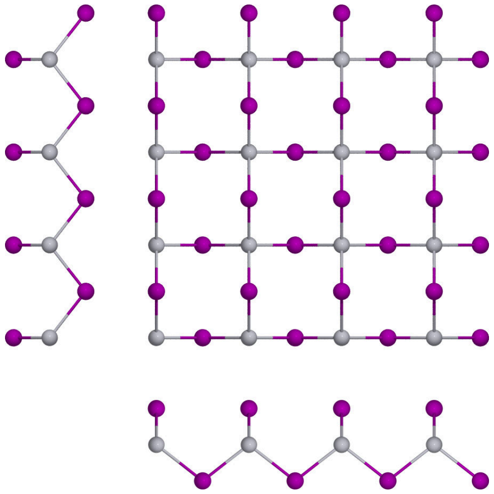

Figure 414: Structure representation

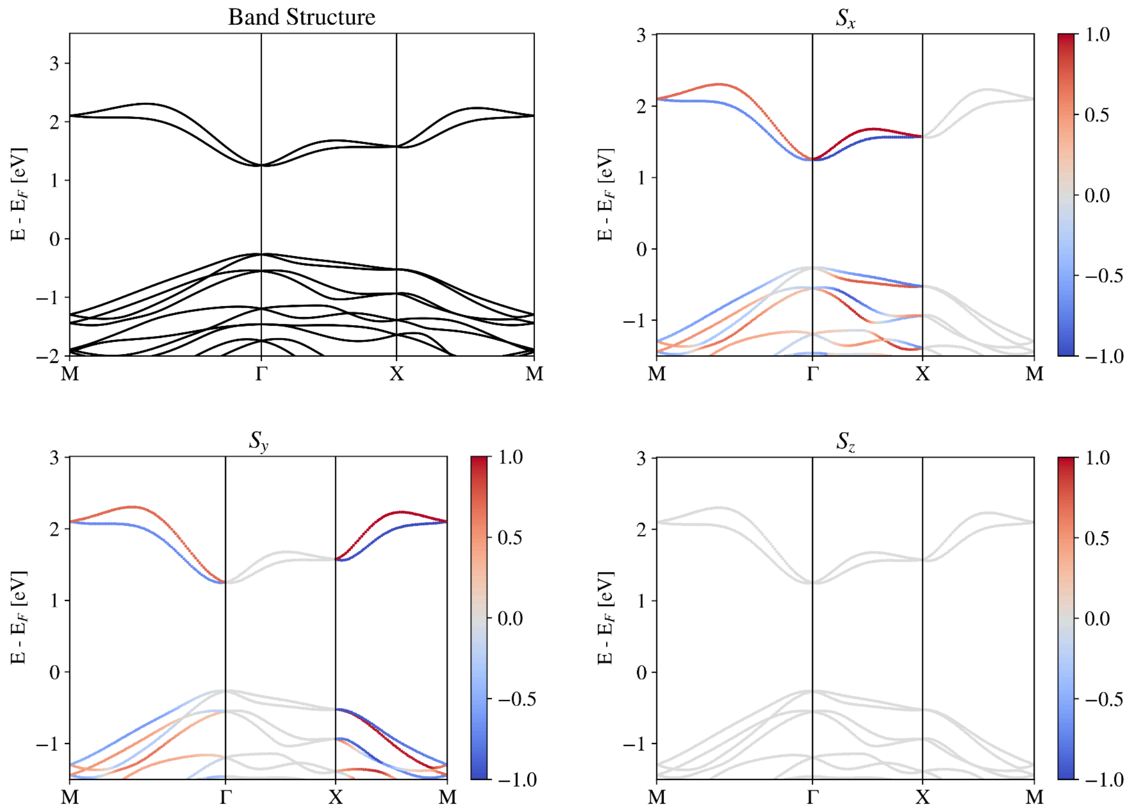


Figure 415: Band structure and spin polarization projections.

## 2.208 HgO-a8678fa85c38

- **Formula**: HgO
- **Structural Cluster**: AB-2
- **Band gap (PBE)**: 0.298 eV
- **Energy above convex hull (C2DB)**: 0.211 eV
- **Space group symbol**: $P\bar{6}m2$
- **Space group number**: 187
- **Polar structure**: False

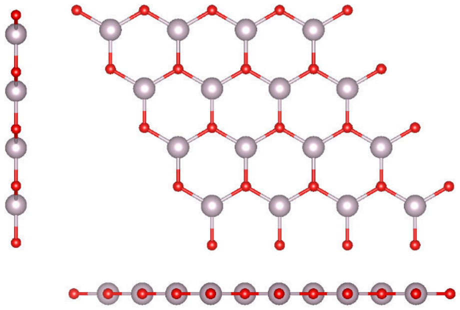

Figure 416: Structure representation

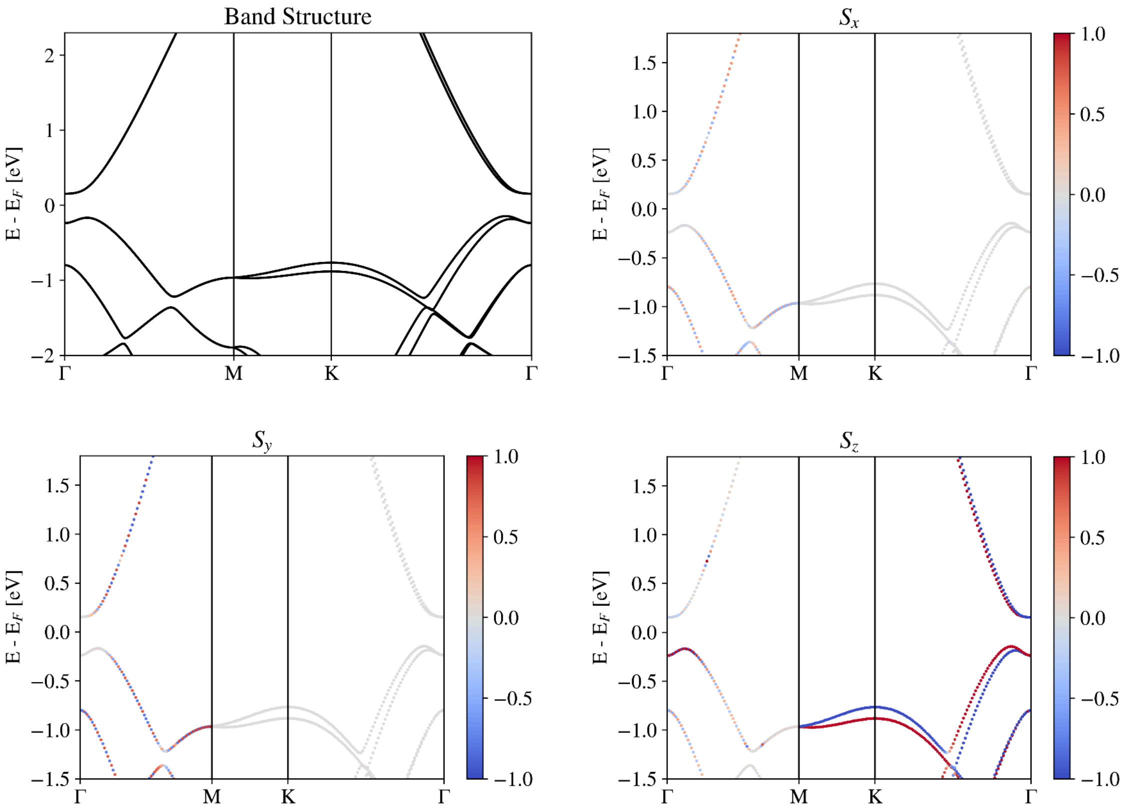


Figure 417: Band structure and spin polarization projections.

## 2.209 HgS-5256ed7d716e

- **Formula**: HgS
- **Structural Cluster**: AB-2
- **Band gap (PBE)**: 0.056 eV
- **Energy above convex hull (C2DB)**: 0.146 eV
- **Space group symbol**: $P3m1$
- **Space group number**: 156
- **Polar structure**: True

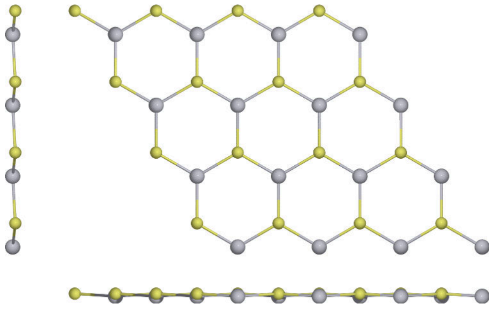

Figure 418: Structure representation

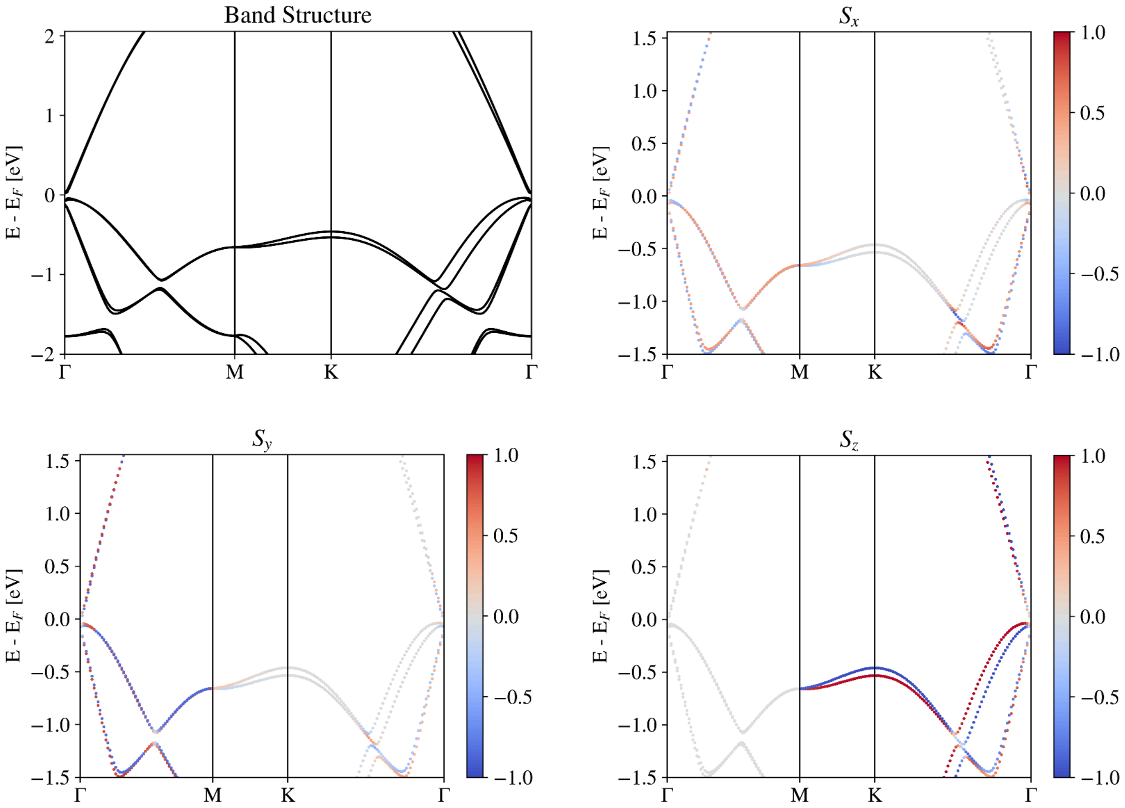


Figure 419: Band structure and spin polarization projections.

## 2.210 HgSe-619ed885f677

- **Formula**: HgSe
- **Structural Cluster**: AB-2
- **Band gap (PBE)**: 0.069 eV
- **Energy above convex hull (C2DB)**: 0.157 eV
- **Space group symbol**: $P3m1$
- **Space group number**: 156
- **Polar structure**: True

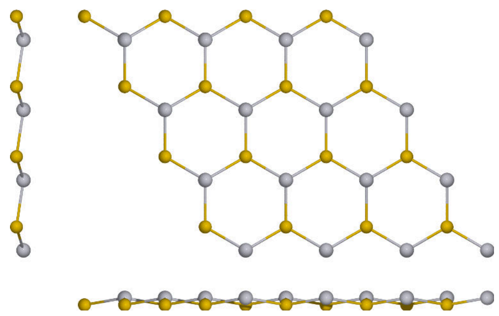

Figure 420: Structure representation

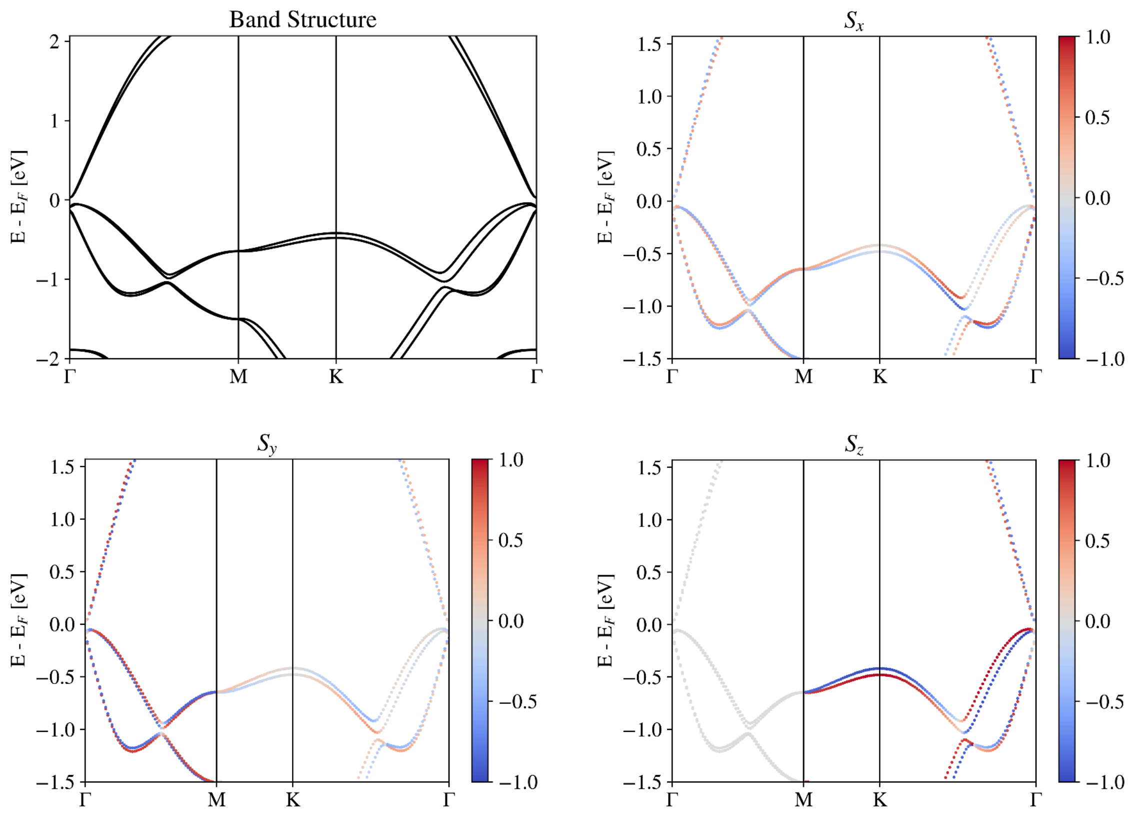


Figure 421: Band structure and spin polarization projections.

## 2.211 HgTe-1a3bdd1b142a

- **Formula**: HgTe
- **Structural Cluster**: AB-2
- **Band gap (PBE)**: 0.132 eV
- **Energy above convex hull (C2DB)**: 0.165 eV
- **Space group symbol**: $P3m1$
- **Space group number**: 156
- **Polar structure**: True

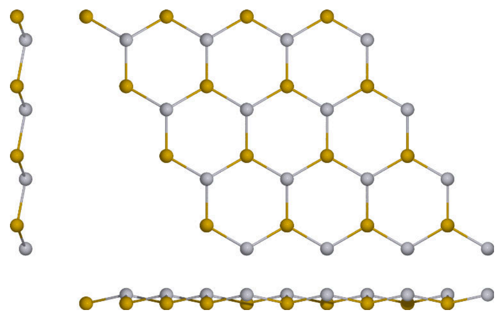

Figure 422: Structure representation

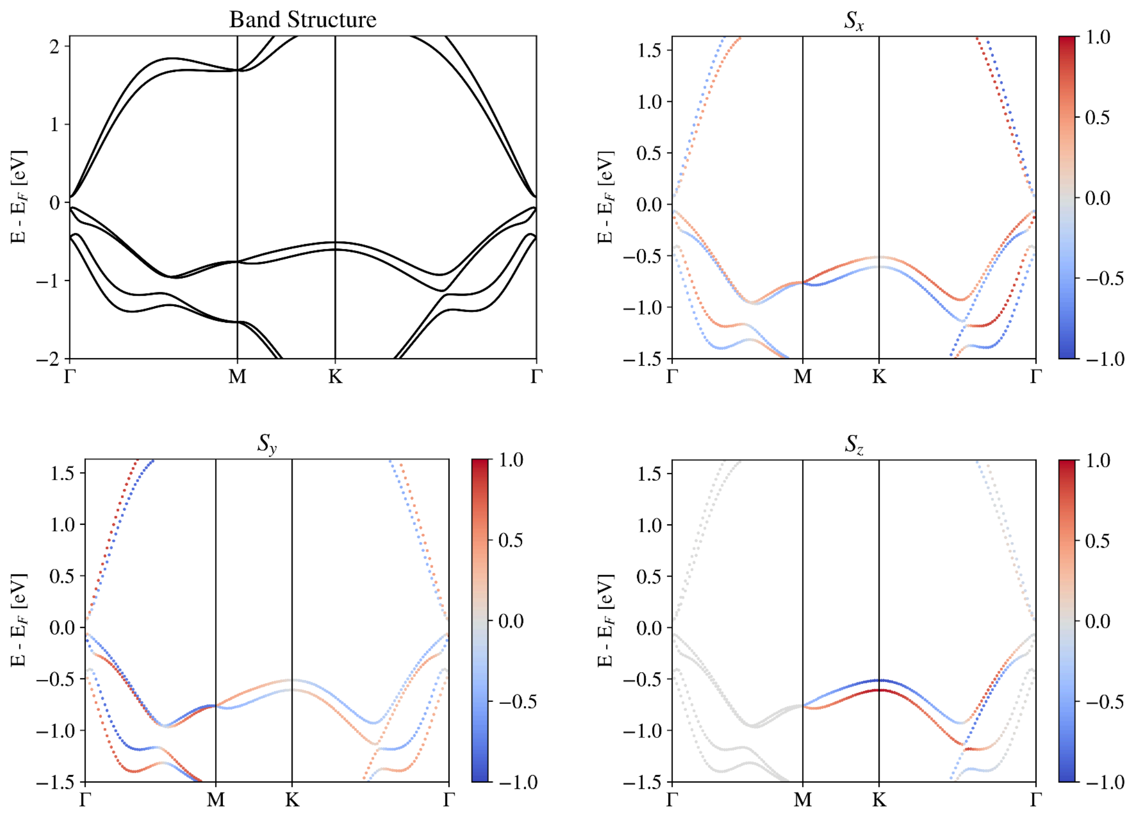


Figure 423: Band structure and spin polarization projections.

## 2.212 I2Tl2-c0f52097ab62

- **Formula**: I2Tl2
- **Structural Cluster**: AB-8
- **Band gap (PBE)**: 2.657 eV
- **Energy above convex hull (C2DB)**: 0.076 eV
- **Space group symbol**: $P1$
- **Space group number**: 1
- **Polar structure**: True

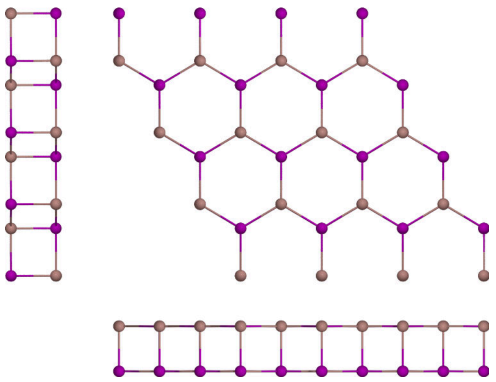

Figure 424: Structure representation

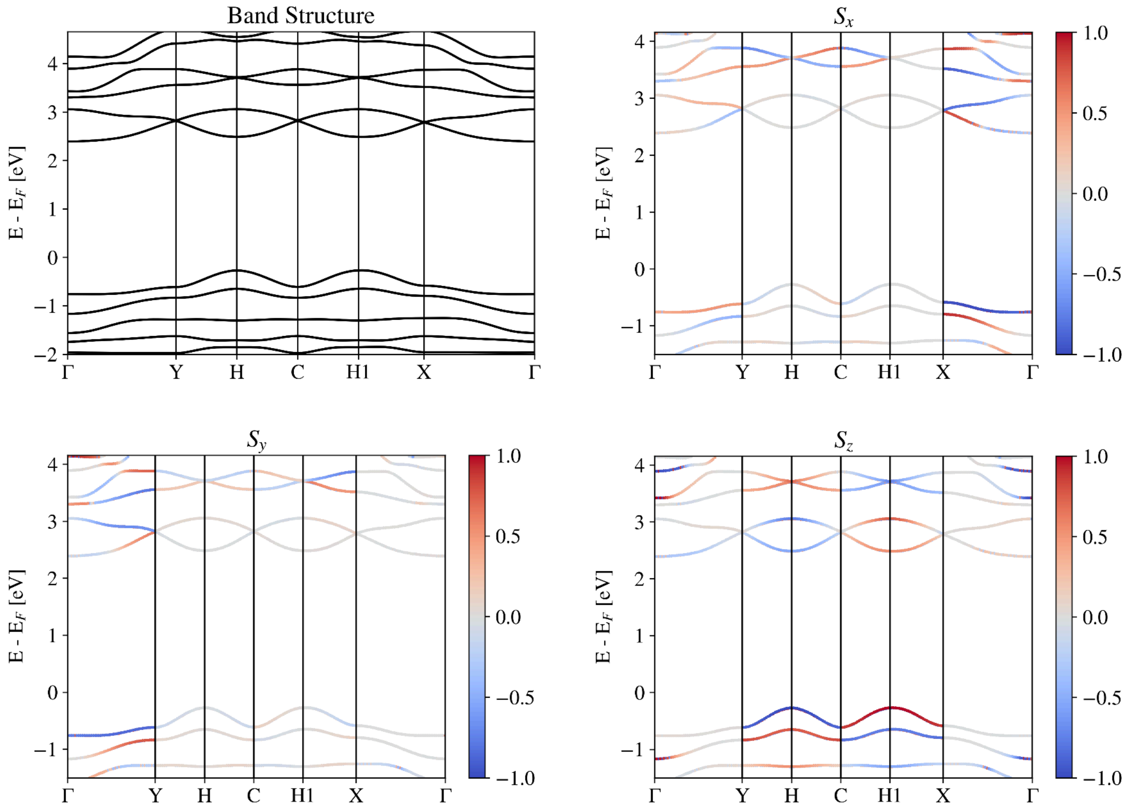


Figure 425: Band structure and spin polarization projections.

## 2.213 ISSb-4c49d27e66e5

- **Formula**: ISSb
- **Structural Cluster**: ABC-4
- **Band gap (PBE)**: 0.872 eV
- **Energy above convex hull (C2DB)**: 0.185 eV
- **Space group symbol**: $P3m1$
- **Space group number**: 156
- **Polar structure**: True

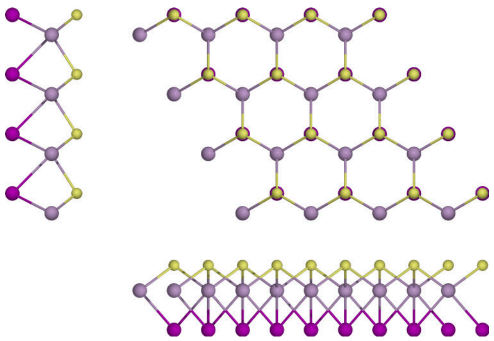

Figure 426: Structure representation

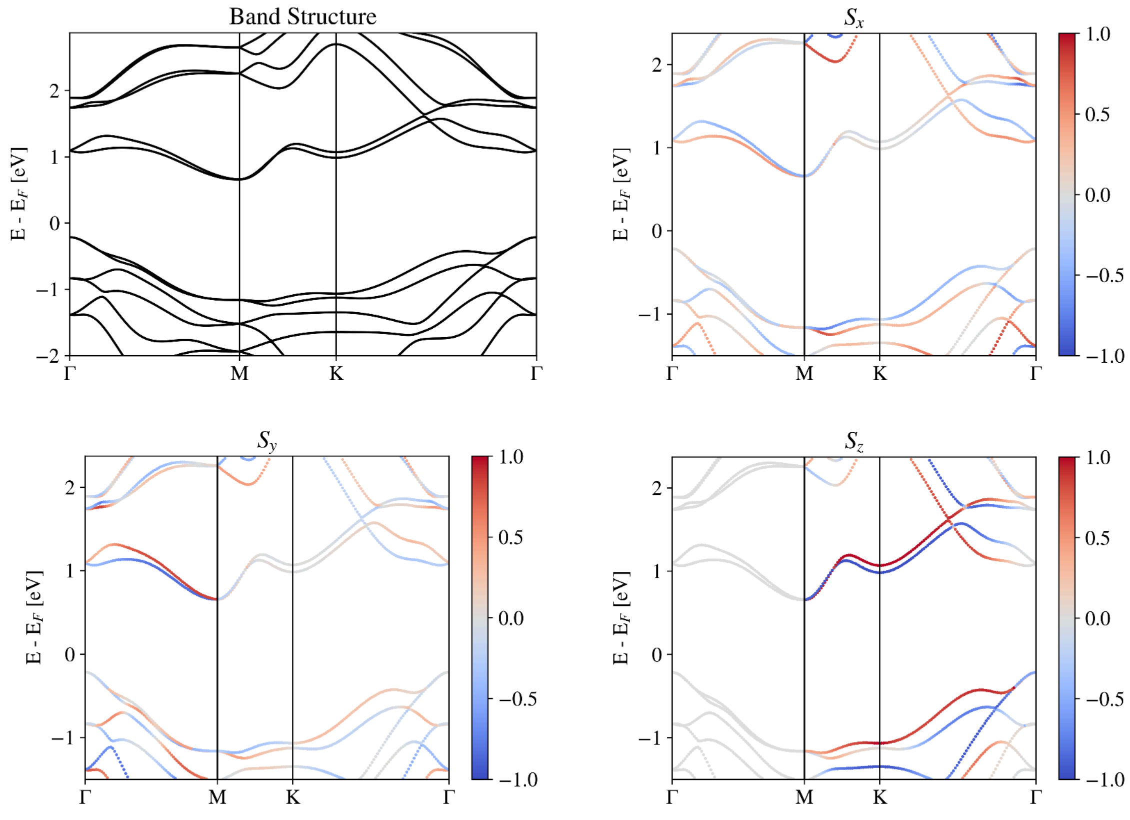


Figure 427: Band structure and spin polarization projections.

## 2.214 ISSb-5b94060698bc

- **Formula**: ISSb
- **Structural Cluster**: ABC-3
- **Band gap (PBE)**: 1.276 eV
- **Energy above convex hull (C2DB)**: 0.041 eV
- **Space group symbol**: $P3m1$
- **Space group number**: 156
- **Polar structure**: True

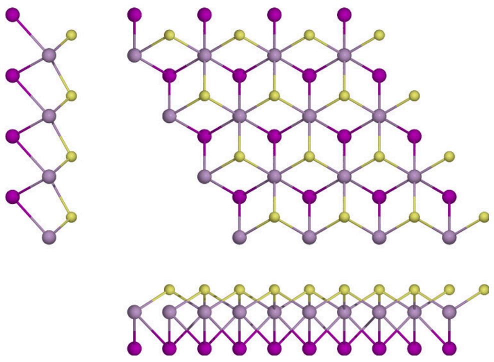

Figure 428: Structure representation

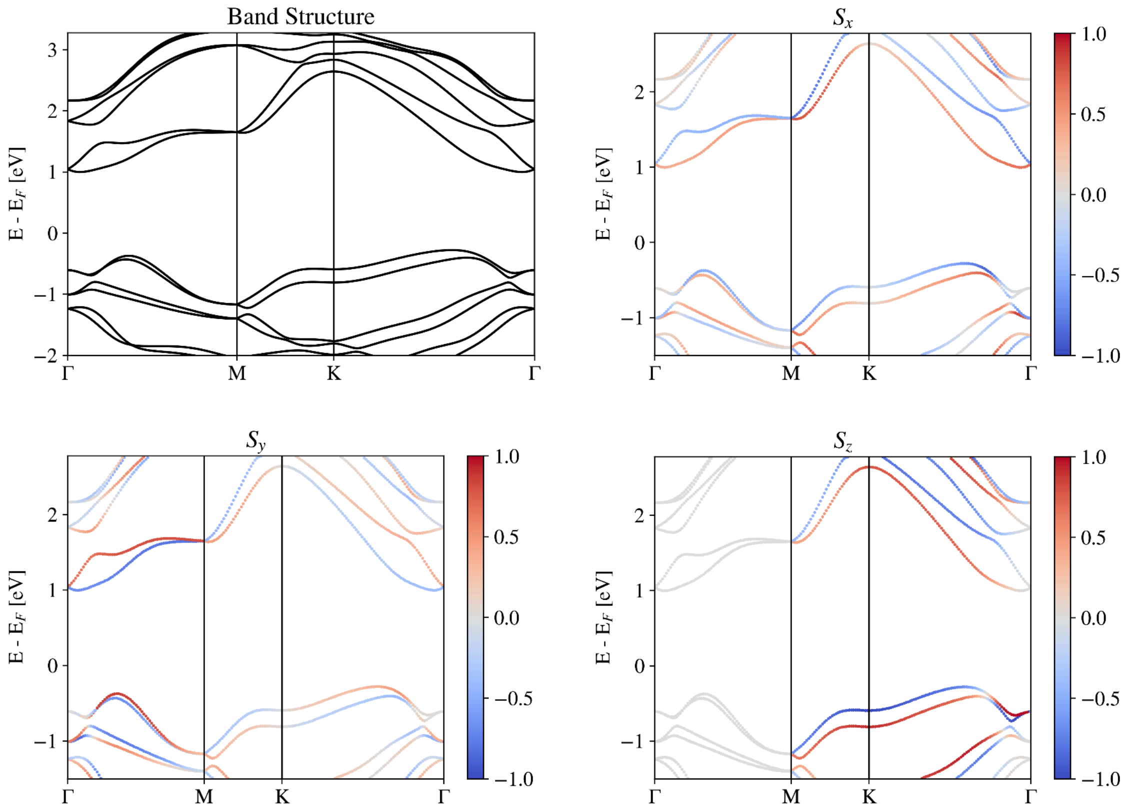


Figure 429: Band structure and spin polarization projections.

## 2.215 ISbSe-343d2125478e

- **Formula**: ISbSe
- **Structural Cluster**: ABC-4
- **Band gap (PBE)**: 1.078 eV
- **Energy above convex hull (C2DB)**: 0.13 eV
- **Space group symbol**: $P3m1$
- **Space group number**: 156
- **Polar structure**: True

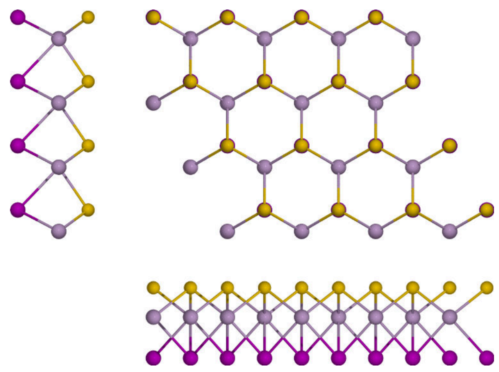

Figure 430: Structure representation

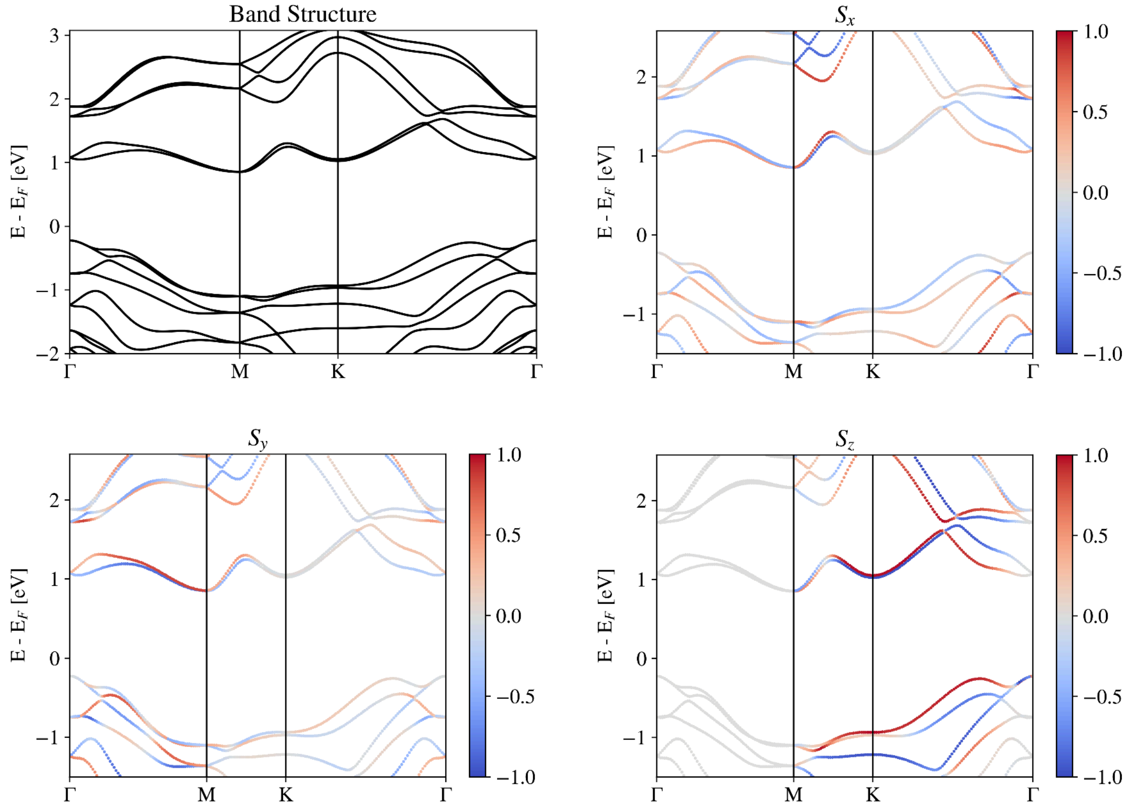


Figure 431: Band structure and spin polarization projections.

## 2.216 ISbSe-df0019ec24b5

- **Formula**: ISbSe
- **Structural Cluster**: ABC-3
- **Band gap (PBE)**: 1.061 eV
- **Energy above convex hull (C2DB)**: 0.0 eV
- **Space group symbol**: $P3m1$
- **Space group number**: 156
- **Polar structure**: True

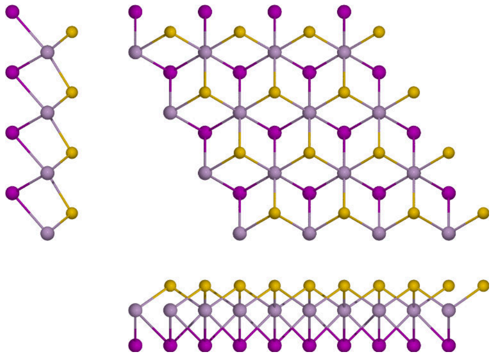

Figure 432: Structure representation

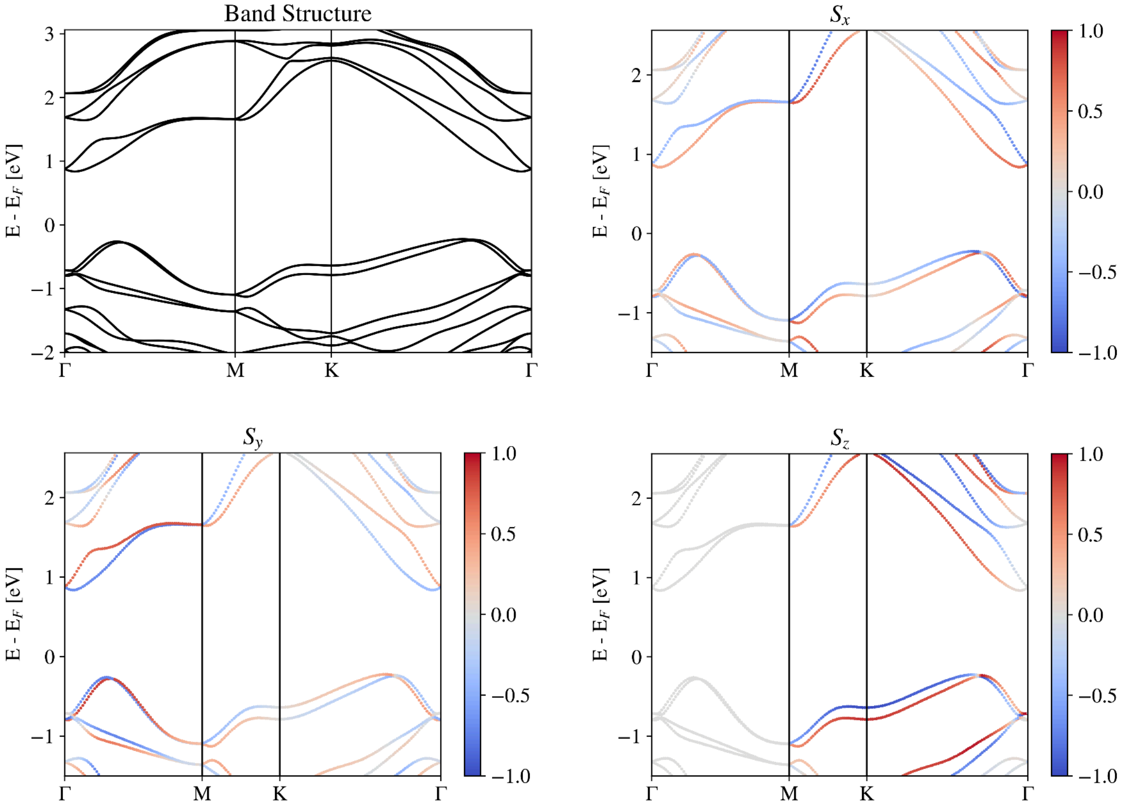


Figure 433: Band structure and spin polarization projections.

## 2.217 ISbTe-052a3116531d

- **Formula**: ISbTe
- **Structural Cluster**: ABC-4
- **Band gap (PBE)**: 1.031 eV
- **Energy above convex hull (C2DB)**: 0.123 eV
- **Space group symbol**: $P3m1$
- **Space group number**: 156
- **Polar structure**: True

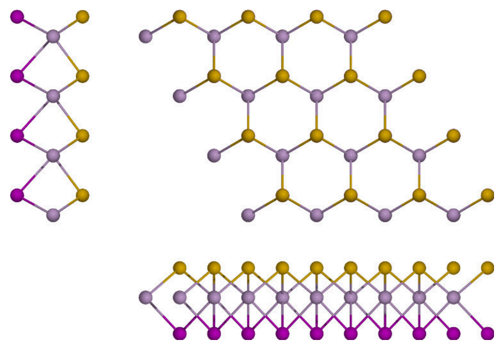

Figure 434: Structure representation

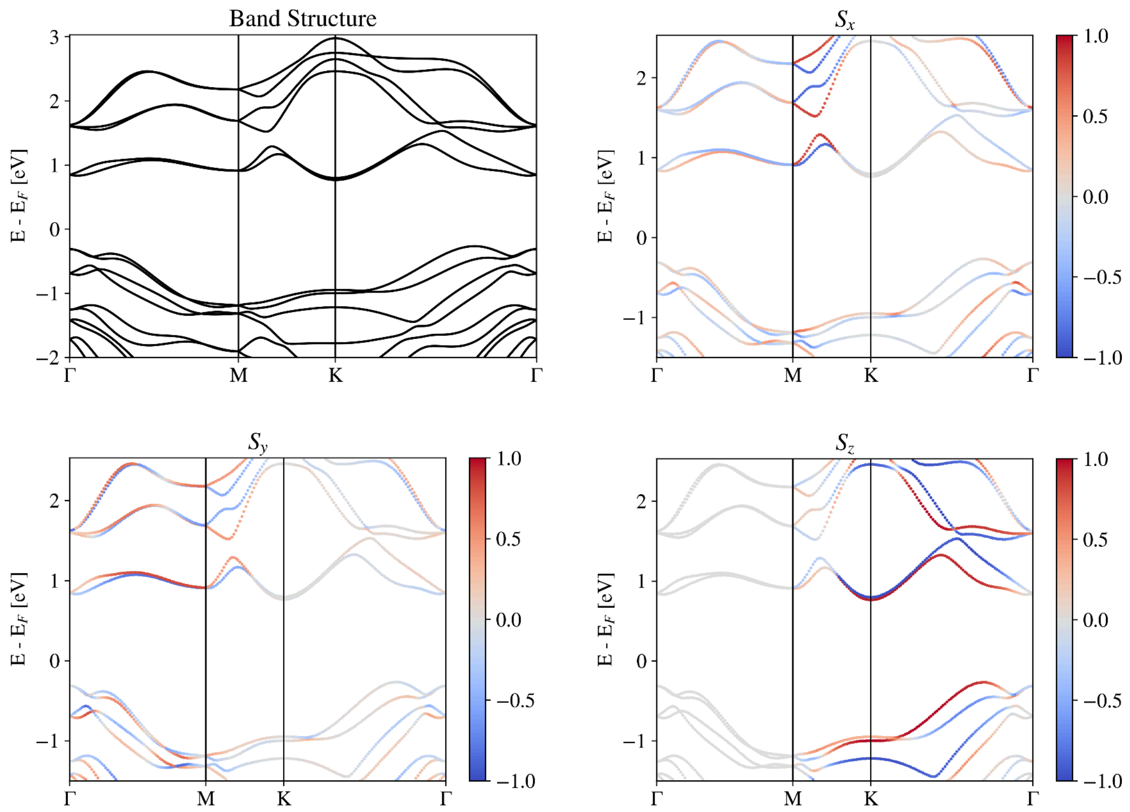


Figure 435: Band structure and spin polarization projections.

## 2.218 ISbTe-0f02957b17cf

- **Formula**: ISbTe
- **Structural Cluster**: ABC-3
- **Band gap (PBE)**: 0.886 eV
- **Energy above convex hull (C2DB)**: 0.0 eV
- **Space group symbol**: $P3m1$
- **Space group number**: 156
- **Polar structure**: True

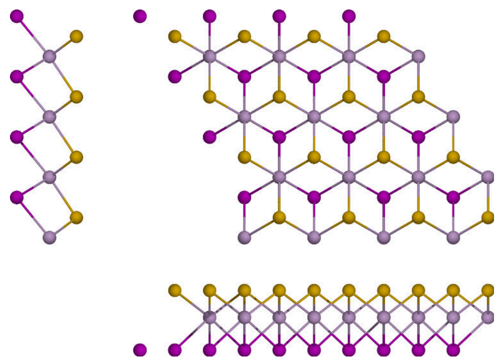

Figure 436: Structure representation

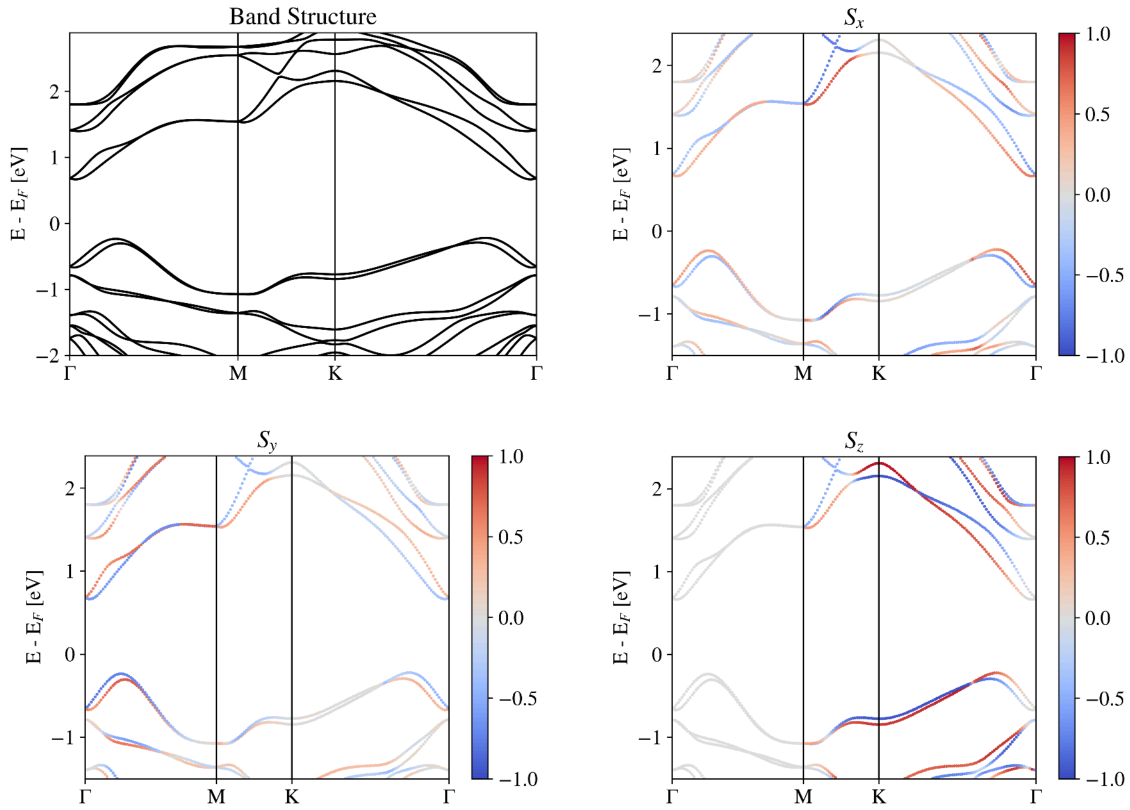


Figure 437: Band structure and spin polarization projections.

## 2.219 In2Br6-d59933910be2

- **Formula**: In2Br6
- **Structural Cluster**: AB3-19
- **Band gap (PBE)**: 2.019 eV
- **Energy above convex hull (C2DB)**: 0.114 eV
- **Space group symbol**: $P\bar{6}2m$
- **Space group number**: 189
- **Polar structure**: False

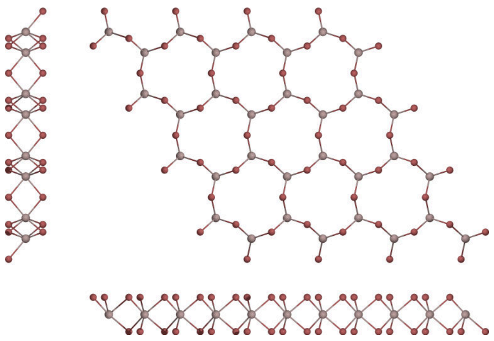

Figure 438: Structure representation

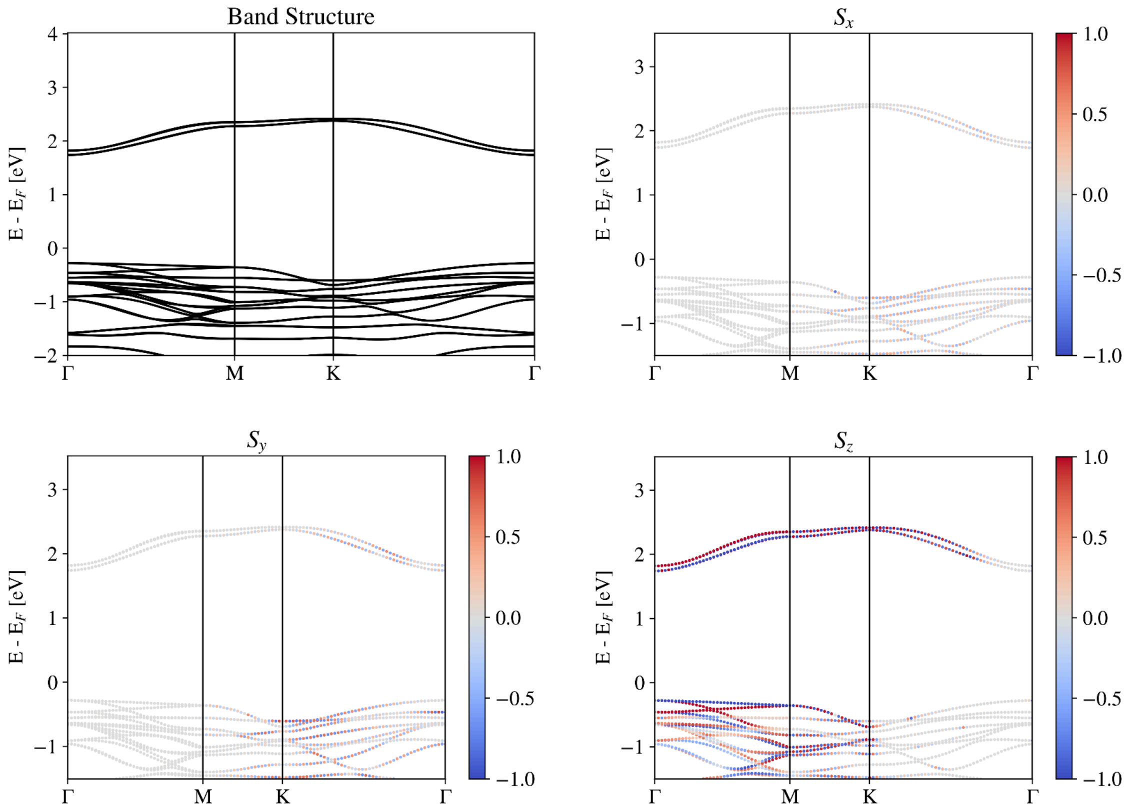


Figure 439: Band structure and spin polarization projections.

## 2.220 In2Cl6-89b069b9f428

- **Formula**: In2Cl6
- **Structural Cluster**: AB3-19
- **Band gap (PBE)**: 2.867 eV
- **Energy above convex hull (C2DB)**: 0.102 eV
- **Space group symbol**: $P\bar{6}2m$
- **Space group number**: 189
- **Polar structure**: False

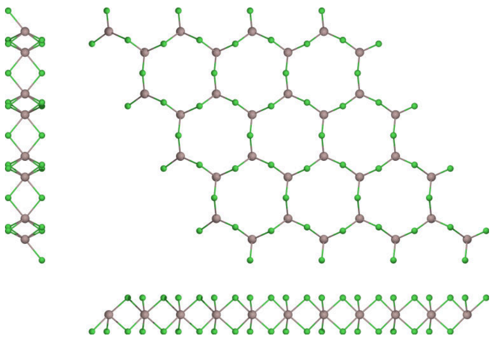

Figure 440: Structure representation

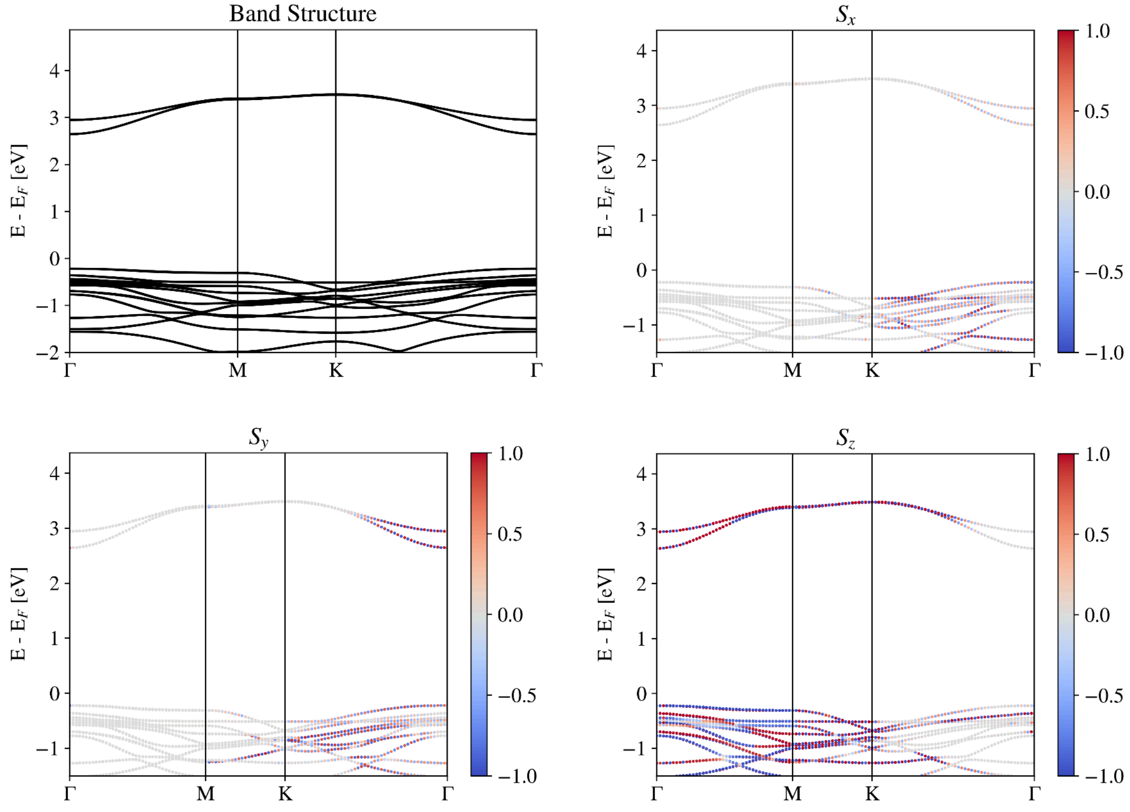


Figure 441: Band structure and spin polarization projections.

## 2.221 In2I6-d90f9a6ab379

- **Formula**: In2I6
- **Structural Cluster**: AB3-19
- **Band gap (PBE)**: 0.703 eV
- **Energy above convex hull (C2DB)**: 0.136 eV
- **Space group symbol**: $P\bar{6}2m$
- **Space group number**: 189
- **Polar structure**: False

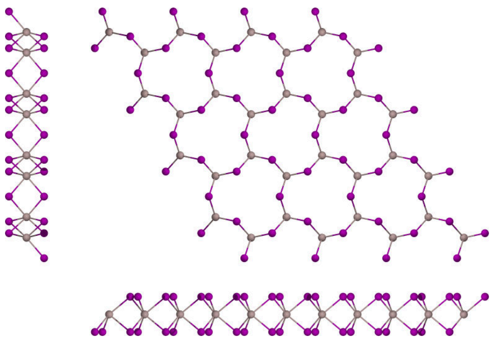

Figure 442: Structure representation

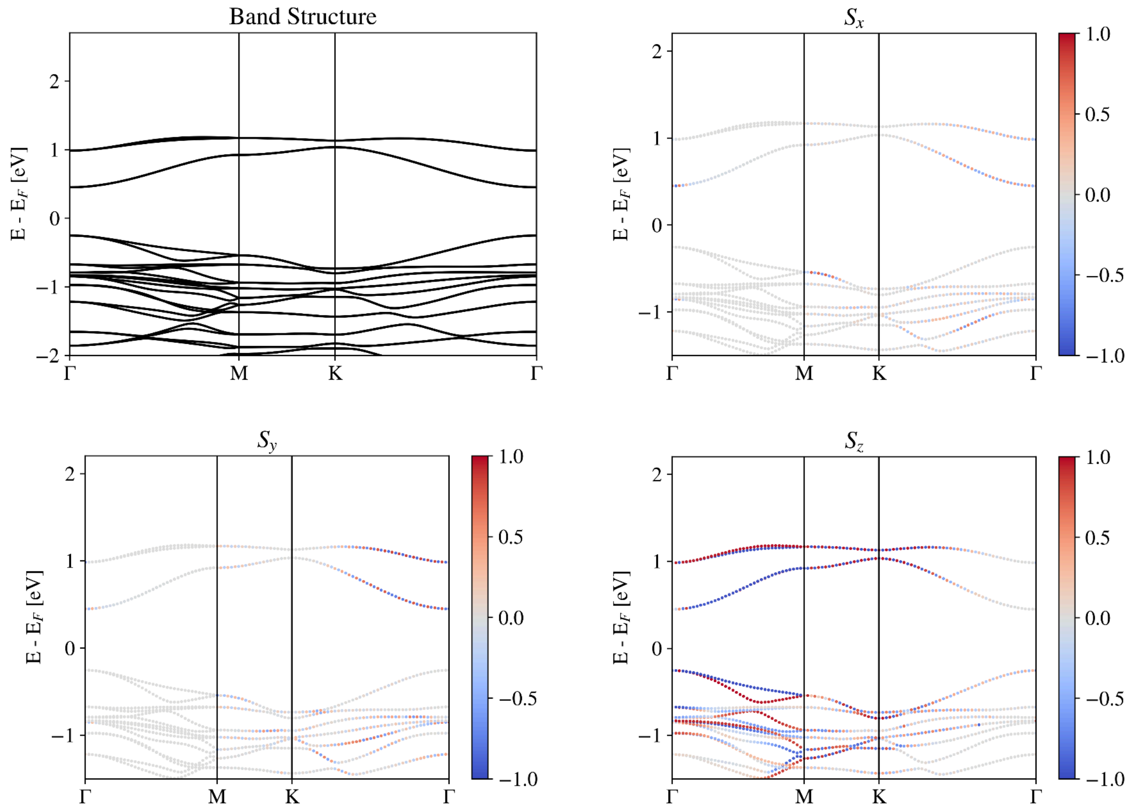


Figure 443: Band structure and spin polarization projections.

## 2.222 In2O2-d14171d2ba1a

- **Formula**: In2O2
- **Structural Cluster**: AB-5
- **Band gap (PBE)**: 0.371 eV
- **Energy above convex hull (C2DB)**: 0.208 eV
- **Space group symbol**: $P\bar{6}m2$
- **Space group number**: 187
- **Polar structure**: False

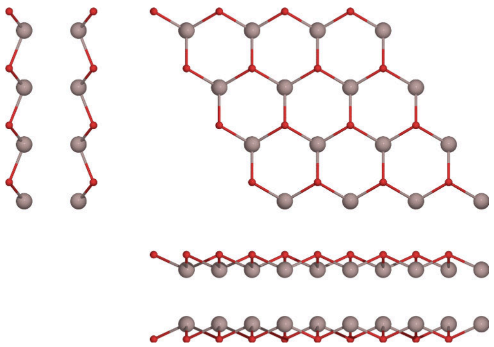

Figure 444: Structure representation

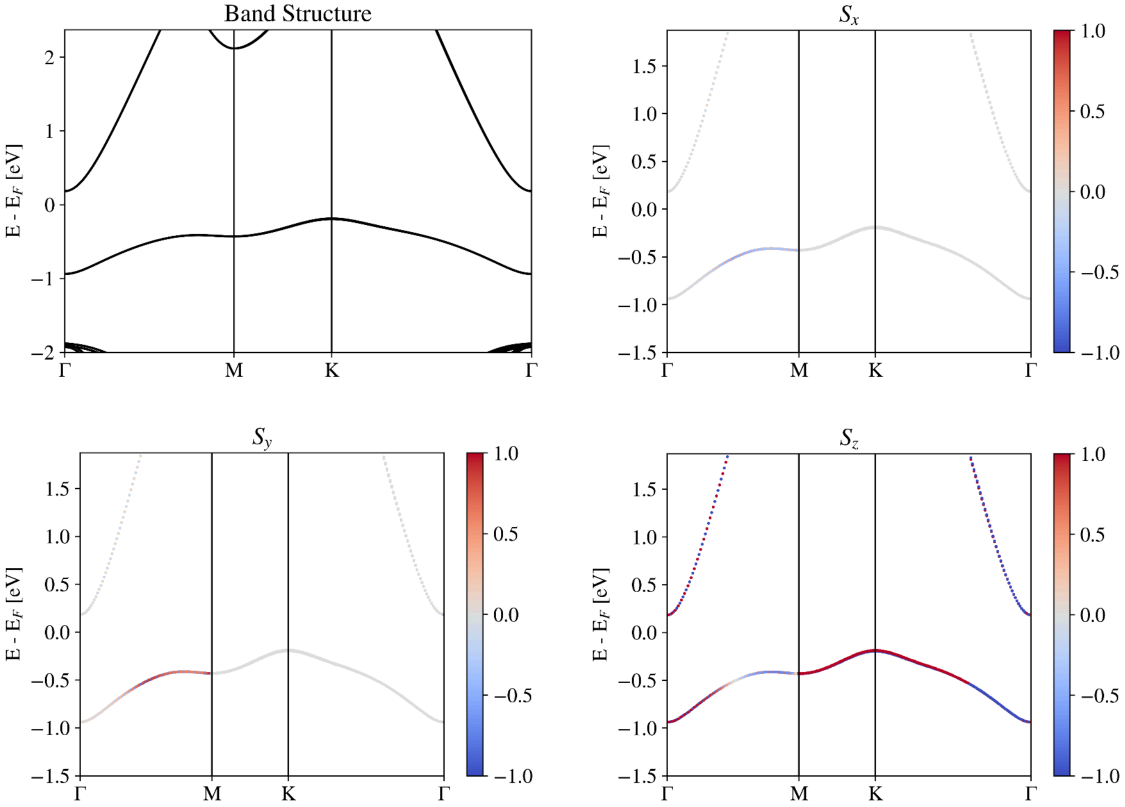


Figure 445: Band structure and spin polarization projections.

## 2.223 In2P2S6-793870f62166

- **Formula**: In2P2S6
- **Structural Cluster**: ABC3-16
- **Band gap (PBE)**: 0.852 eV
- **Energy above convex hull (C2DB)**: 0.053 eV
- **Space group symbol**: *P*1
- **Space group number**: 1
- **Polar structure**: True

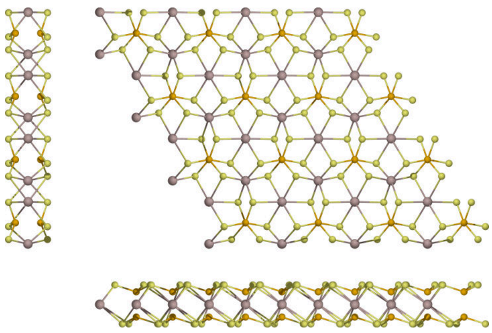

Figure 446: Structure representation

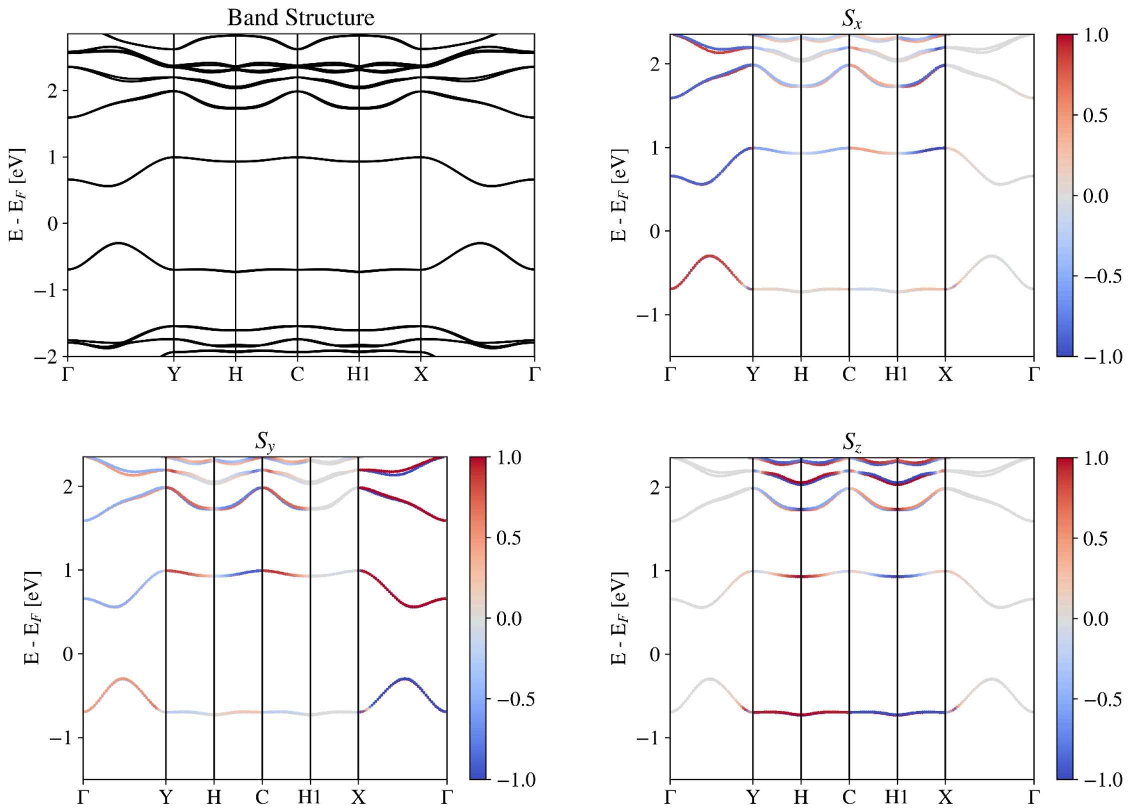


Figure 447: Band structure and spin polarization projections.

## 2.224 In2S2-172ef584c4a6

- **Formula**: In2S2
- **Structural Cluster**: AB-5
- **Band gap (PBE)**: 1.684 eV
- **Energy above convex hull (C2DB)**: 0.0 eV
- **Space group symbol**: $P\bar{6}m2$
- **Space group number**: 187
- **Polar structure**: False

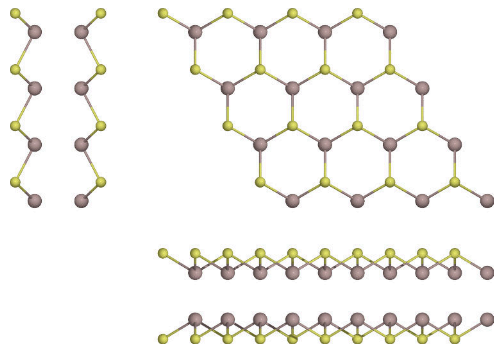

Figure 448: Structure representation

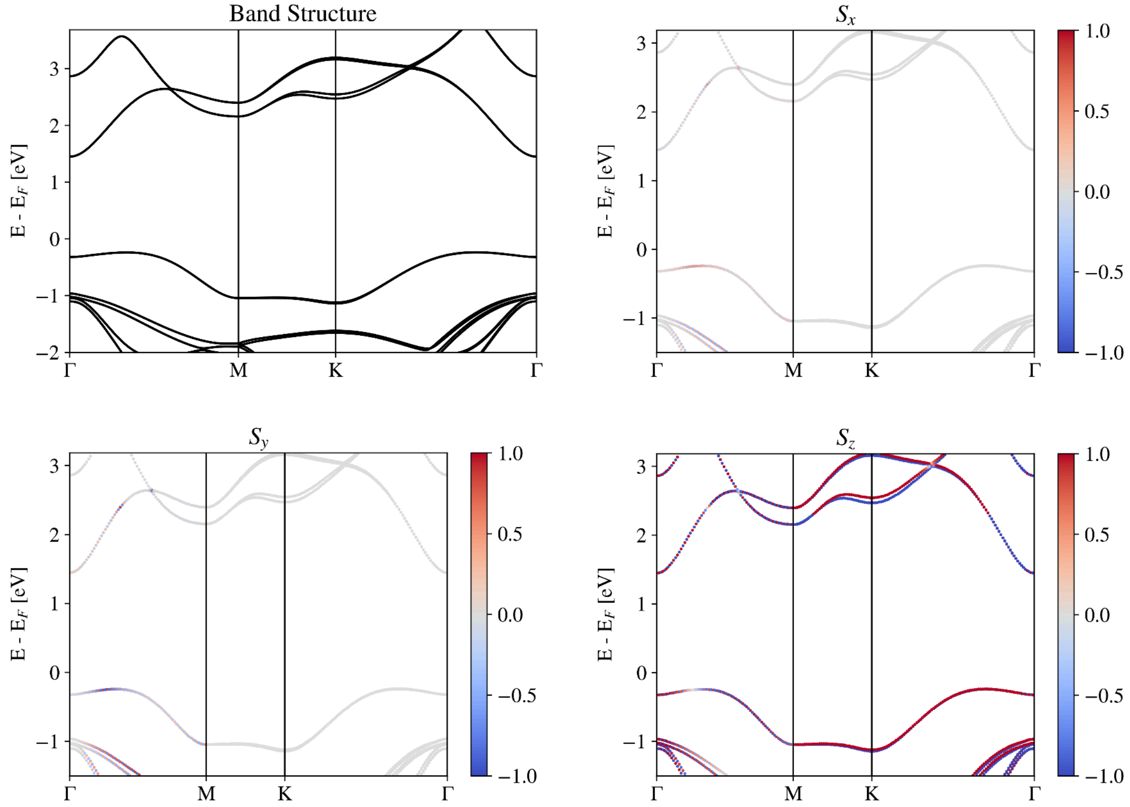


Figure 449: Band structure and spin polarization projections.

## 2.225 In2Se2-eb204c739879

- **Formula**: In2Se2
- **Structural Cluster**: AB-5
- **Band gap (PBE)**: 1.399 eV
- **Energy above convex hull (C2DB)**: 0.0 eV
- **Space group symbol**: $P\bar{6}m2$
- **Space group number**: 187
- **Polar structure**: False

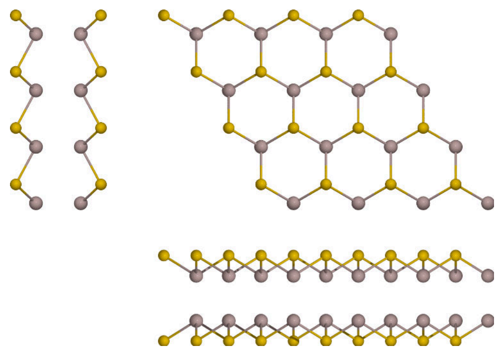

Figure 450: Structure representation

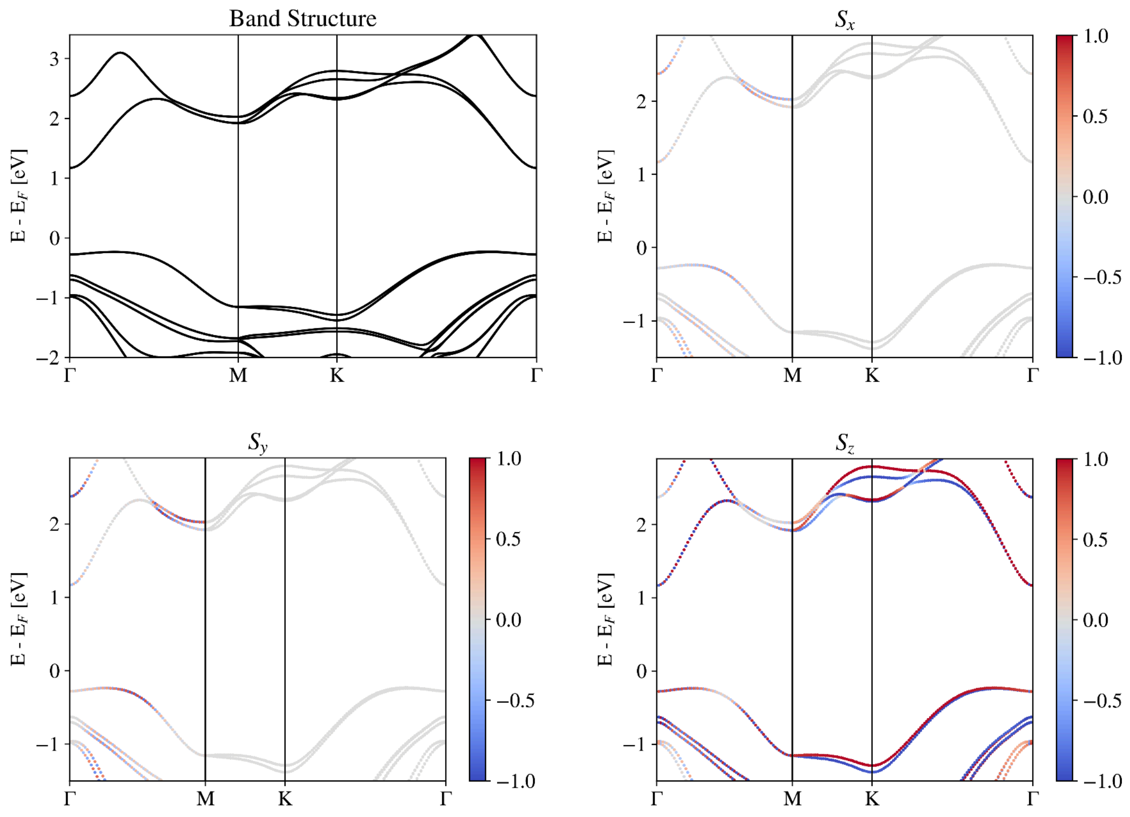


Figure 451: Band structure and spin polarization projections.

## 2.226 In2Te2-3cf9ea4cebc4

- **Formula**: In2Te2
- **Structural Cluster**: AB-5
- **Band gap (PBE)**: 1.163 eV
- **Energy above convex hull (C2DB)**: 0.003 eV
- **Space group symbol**: *Cm*
- **Space group number**: 8
- **Polar structure**: True

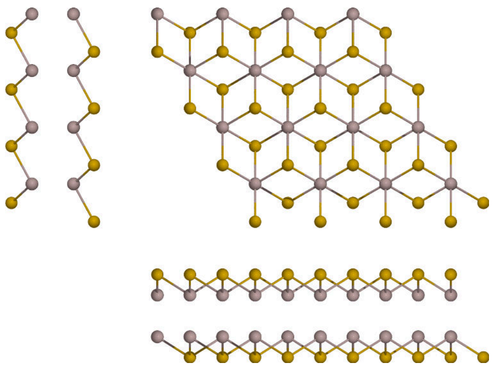

Figure 452: Structure representation

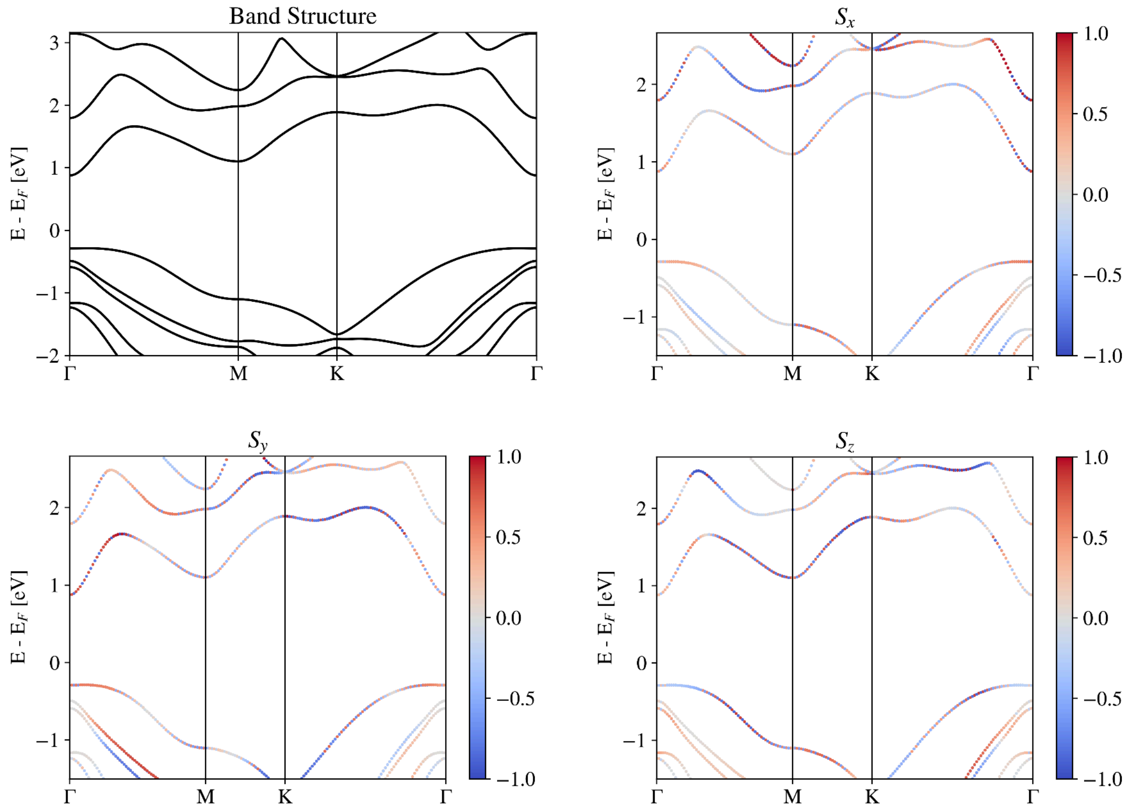


Figure 453: Band structure and spin polarization projections.

## 2.227 In2Te2-fcd97ff5abcd

- **Formula**: In2Te2
- **Structural Cluster**: AB-5
- **Band gap (PBE)**: 1.249 eV
- **Energy above convex hull (C2DB)**: 0.0 eV
- **Space group symbol**: $P\bar{6}m2$
- **Space group number**: 187
- **Polar structure**: False

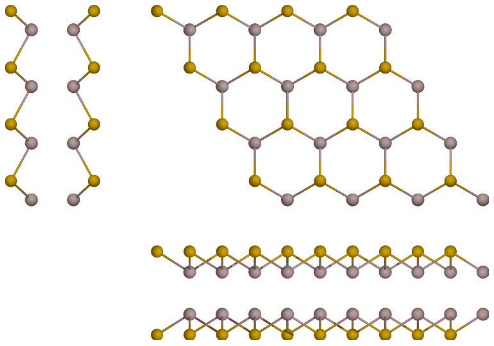

Figure 454: Structure representation

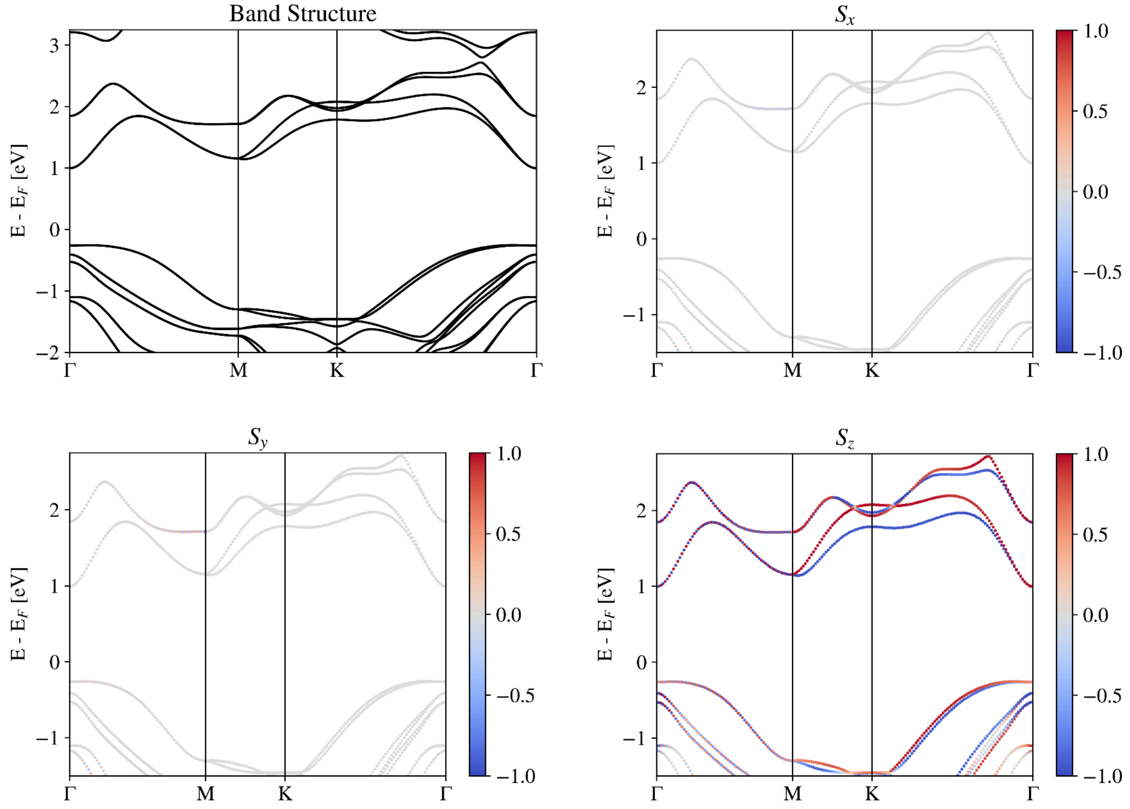


Figure 455: Band structure and spin polarization projections.

## 2.228 InN-8cf70870bc5b

- **Formula**: InN
- **Structural Cluster**: AB-2
- **Band gap (PBE)**: 0.606 eV
- **Energy above convex hull (C2DB)**: 0.482 eV
- **Space group symbol**: $P\bar{6}m2$
- **Space group number**: 187
- **Polar structure**: False

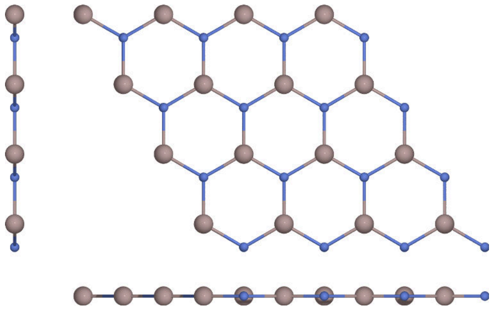

Figure 456: Structure representation

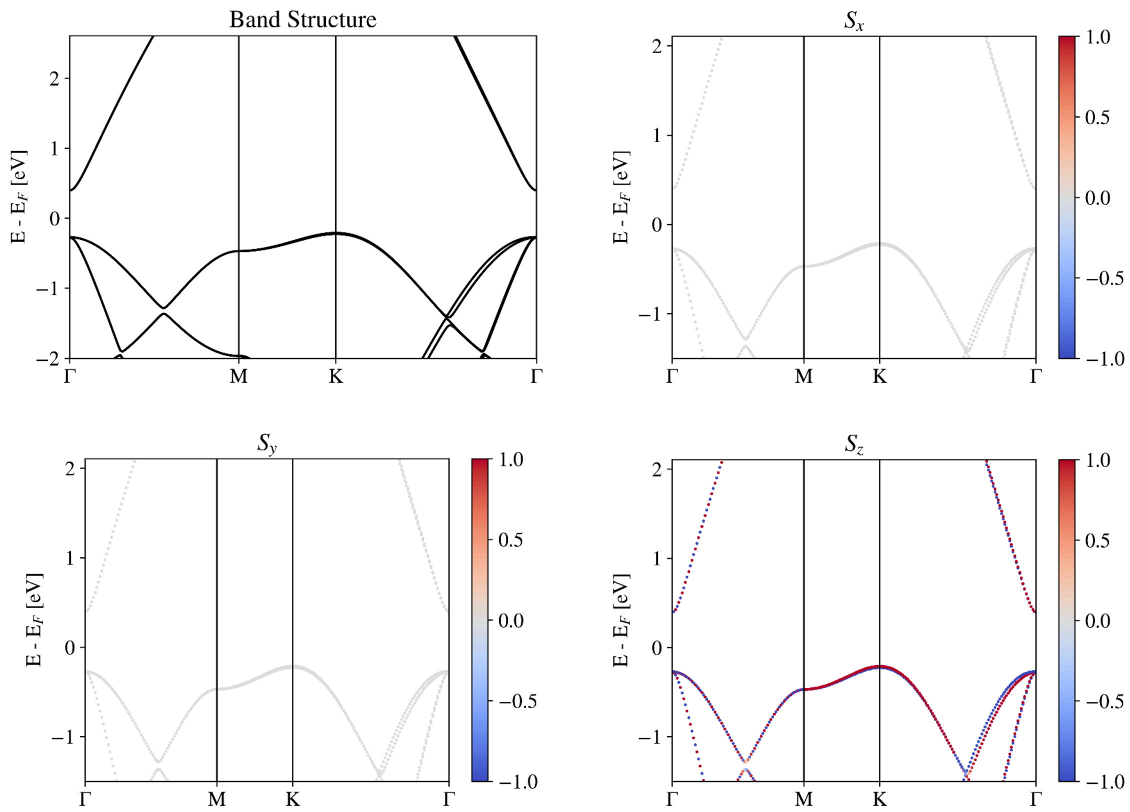


Figure 457: Band structure and spin polarization projections.

## 2.229 InP-c5672c6c1c78

- **Formula**: InP
- **Structural Cluster**: AB-2
- **Band gap (PBE)**: 1.072 eV
- **Energy above convex hull (C2DB)**: 0.432 eV
- **Space group symbol**: *P*3*m*1
- **Space group number**: 156
- **Polar structure**: True

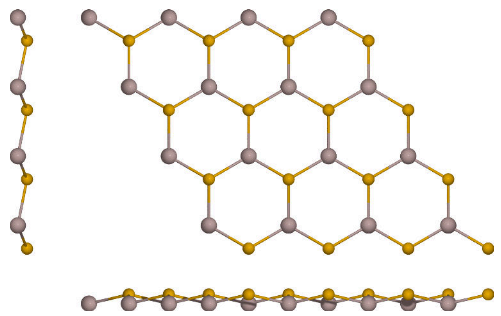

Figure 458: Structure representation

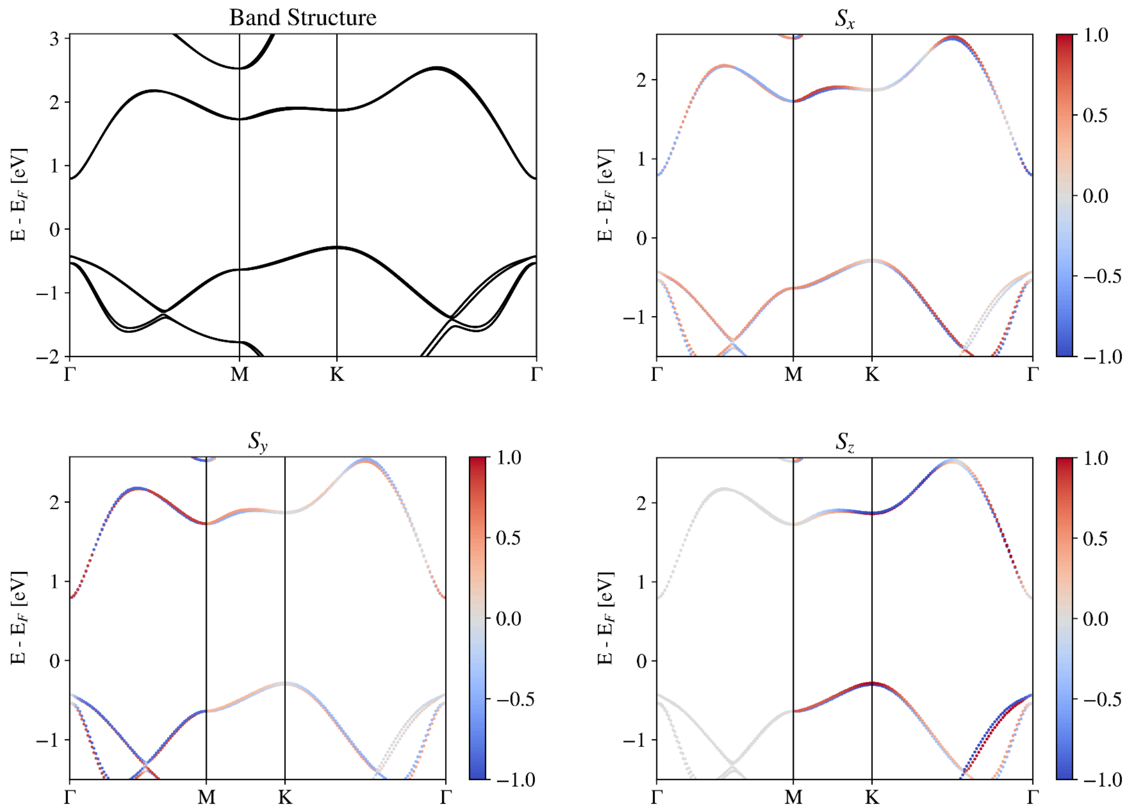


Figure 459: Band structure and spin polarization projections.

## 2.230 InSb-466fcf7fad66

- **Formula**: InSb
- **Structural Cluster**: AB-2
- **Band gap (PBE)**: 0.477 eV
- **Energy above convex hull (C2DB)**: 0.379 eV
- **Space group symbol**: $P3m1$
- **Space group number**: 156
- **Polar structure**: True

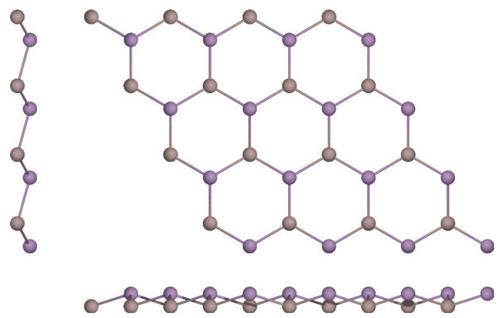

Figure 460: Structure representation

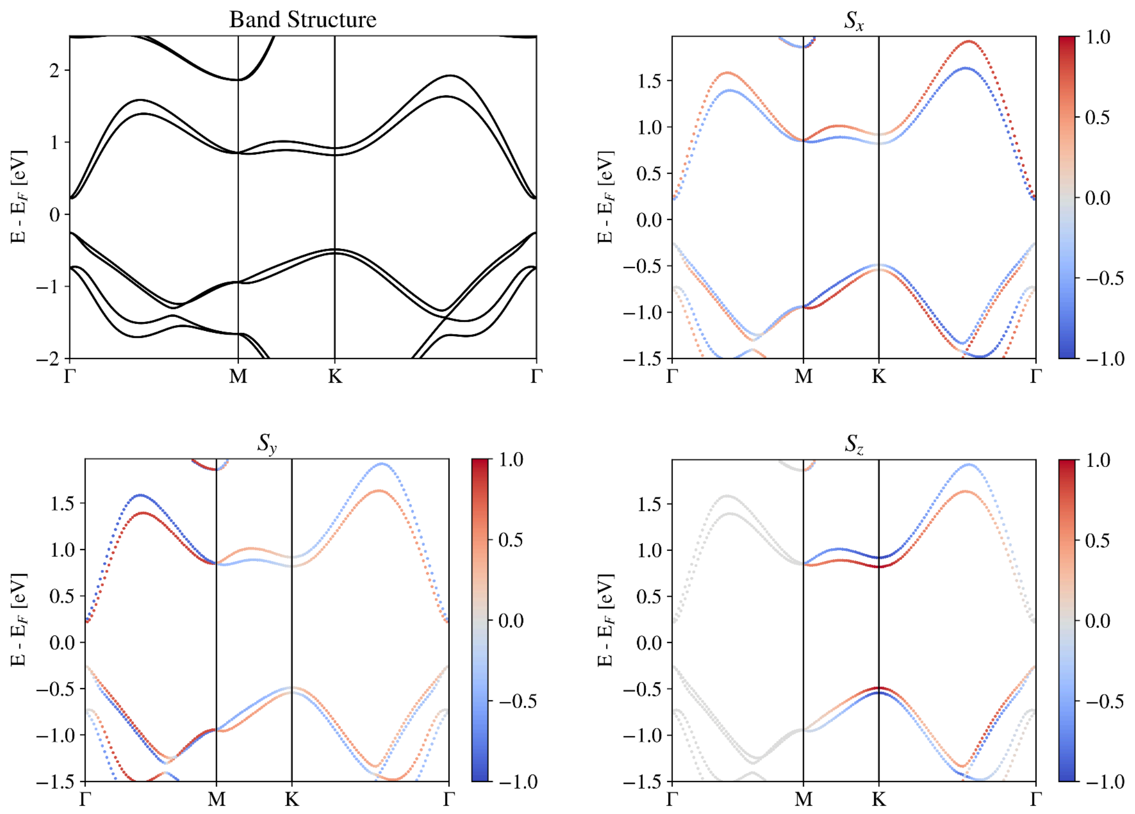


Figure 461: Band structure and spin polarization projections.

## 2.231 Ir2Cl6-be7870547213

- **Formula**: Ir2Cl6
- **Structural Cluster**: AB3-19
- **Band gap (PBE)**: 0.263 eV
- **Energy above convex hull (C2DB)**: 0.555 eV
- **Space group symbol**: *P*321
- **Space group number**: 150
- **Polar structure**: False

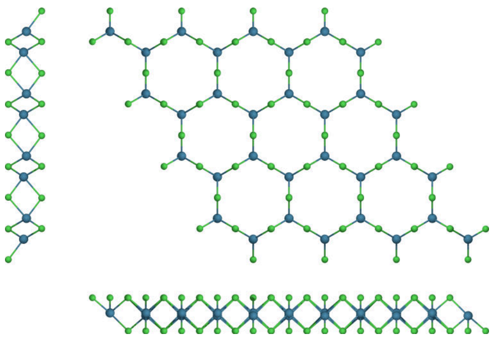

Figure 462: Structure representation

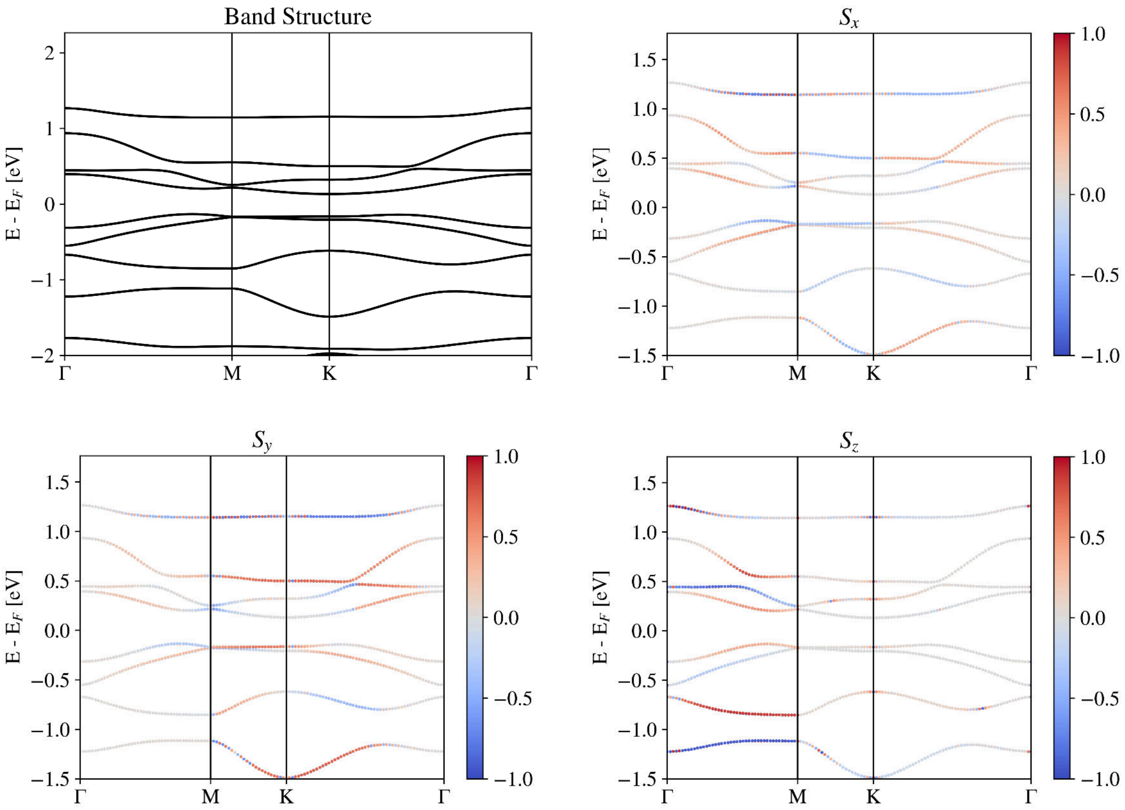


Figure 463: Band structure and spin polarization projections.

## 2.232 Ir2O2-06ebe3806790

- **Formula**: Ir2O2
- **Structural Cluster**: AB-5
- **Band gap (PBE)**: 0.098 eV
- **Energy above convex hull (C2DB)**: 0.51 eV
- **Space group symbol**: $P\bar{6}m2$
- **Space group number**: 187
- **Polar structure**: False

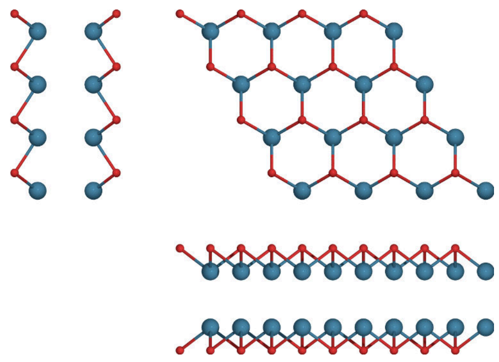

Figure 464: Structure representation

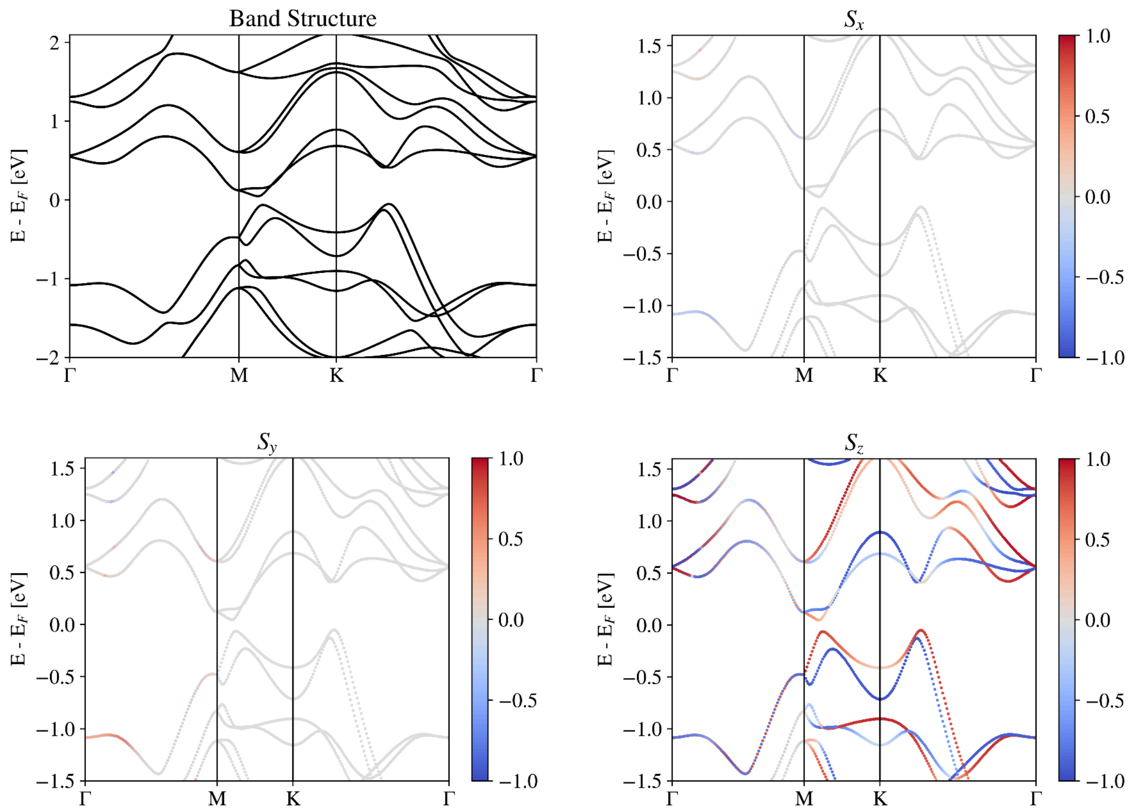


Figure 465: Band structure and spin polarization projections.

## 2.233 Ir2P2S6-2ddba2a8216d

- **Formula**: Ir2P2S6
- **Structural Cluster**: ABC3-16
- **Band gap (PBE)**: 0.006 eV
- **Energy above convex hull (C2DB)**: 0.111 eV
- **Space group symbol**: *Cm*
- **Space group number**: 8
- **Polar structure**: True

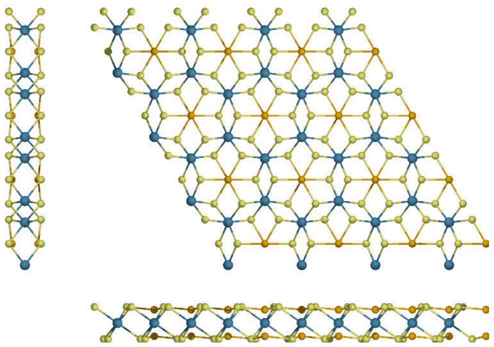

Figure 466: Structure representation

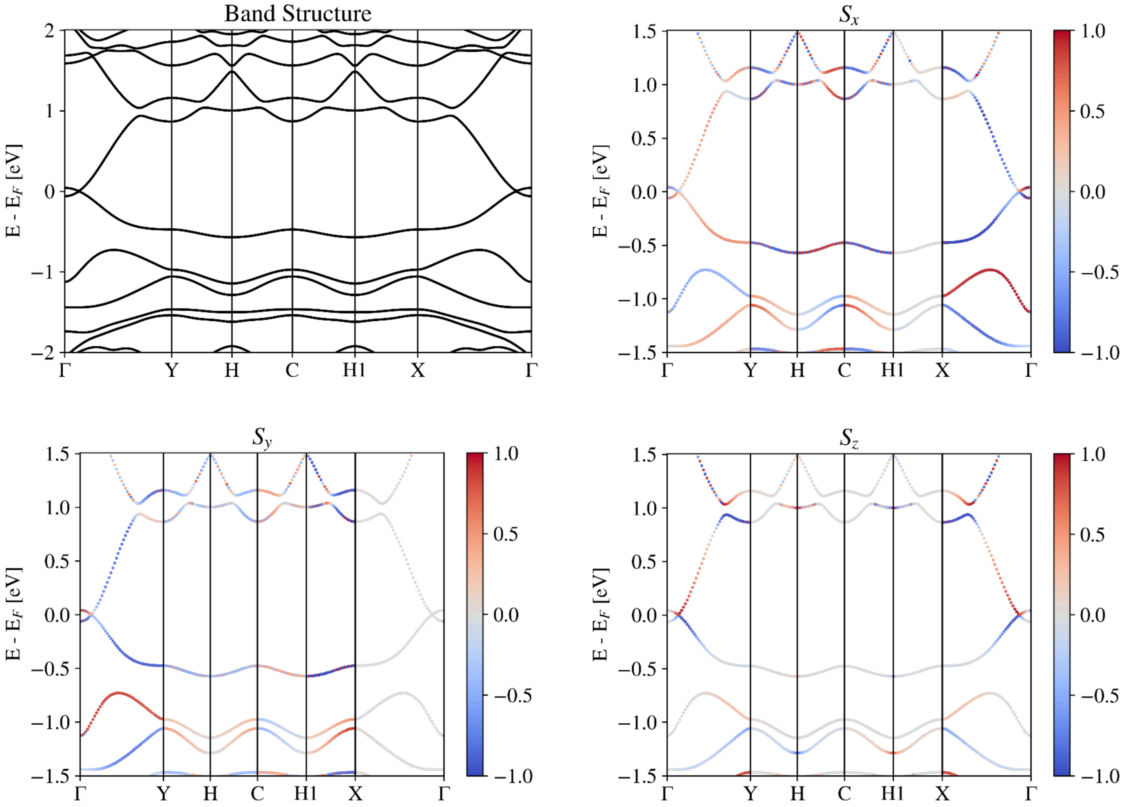


Figure 467: Band structure and spin polarization projections.

## 2.234 Ir2P2Se6-65c3ae4ecac2

- **Formula**: Ir2P2Se6
- **Structural Cluster**: ABC3-16
- **Band gap (PBE)**: 0.083 eV
- **Energy above convex hull (C2DB)**: 0.107 eV
- **Space group symbol**: *Cm*
- **Space group number**: 8
- **Polar structure**: True

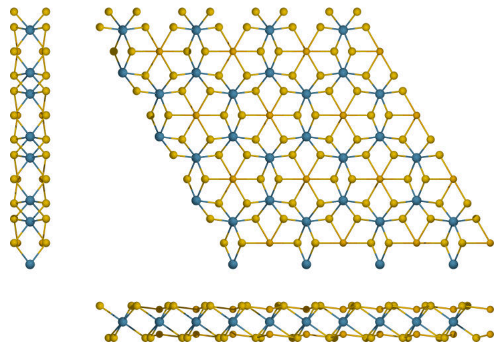

Figure 468: Structure representation

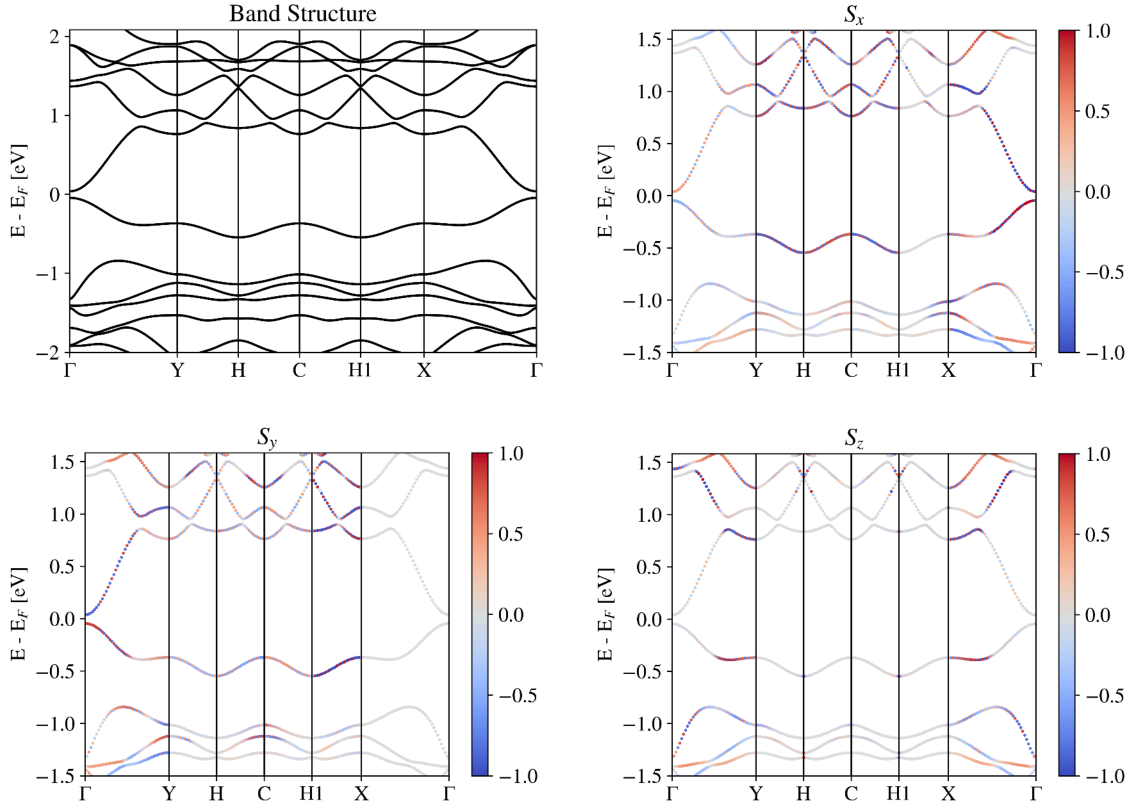


Figure 469: Band structure and spin polarization projections.

## 2.235 Ir2P2Te6-1a850181de67

- **Formula**: Ir2P2Te6
- **Structural Cluster**: ABC3-16
- **Band gap (PBE)**: 0.233 eV
- **Energy above convex hull (C2DB)**: 0.166 eV
- **Space group symbol**: *Cm*
- **Space group number**: 8
- **Polar structure**: True

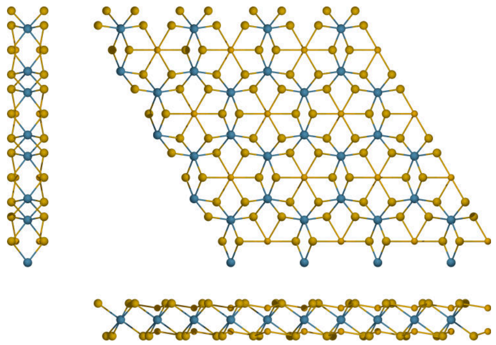

Figure 470: Structure representation

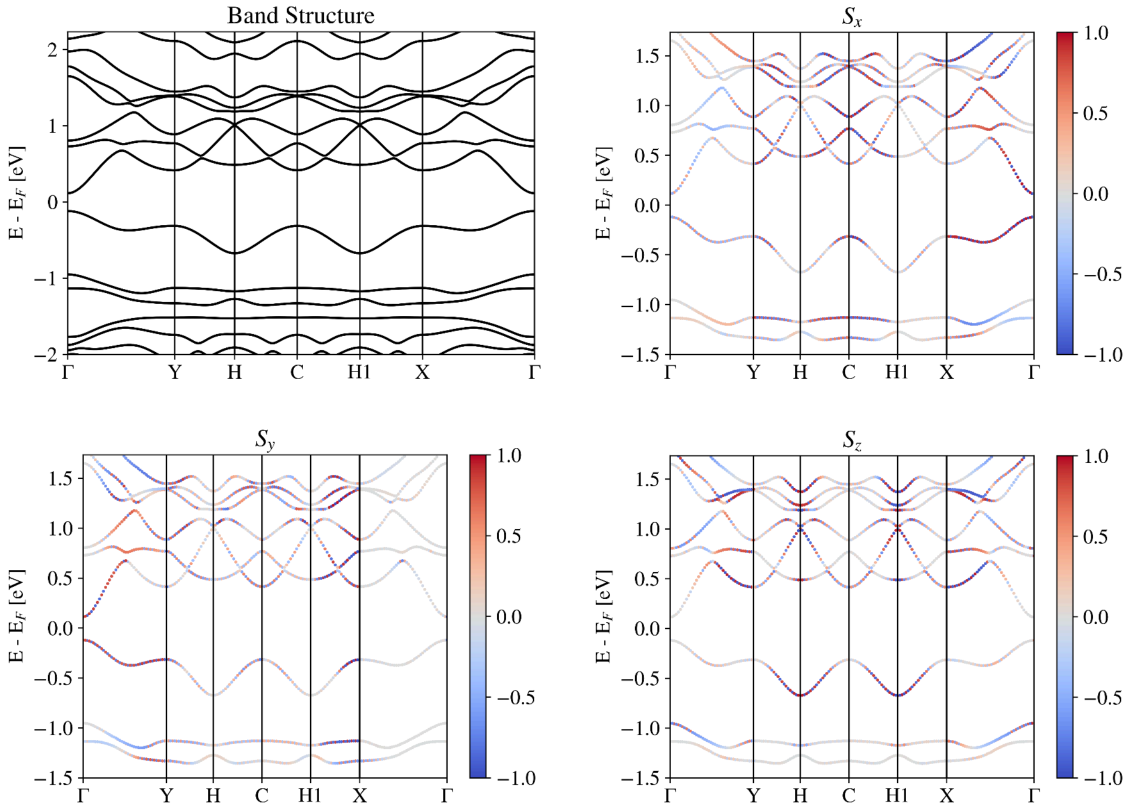


Figure 471: Band structure and spin polarization projections.

## 2.236 Ir2S2-dd6289af8e01

- **Formula**: Ir2S2
- **Structural Cluster**: AB-5
- **Band gap (PBE)**: 0.135 eV
- **Energy above convex hull (C2DB)**: 0.305 eV
- **Space group symbol**: $P\bar{6}m2$
- **Space group number**: 187
- **Polar structure**: False

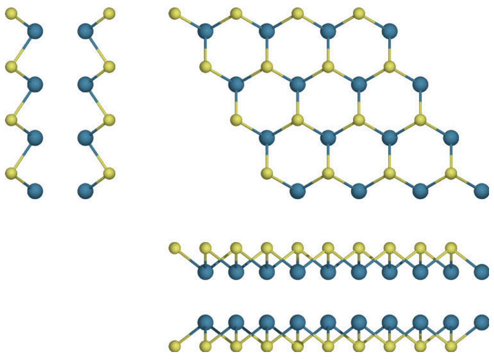

Figure 472: Structure representation

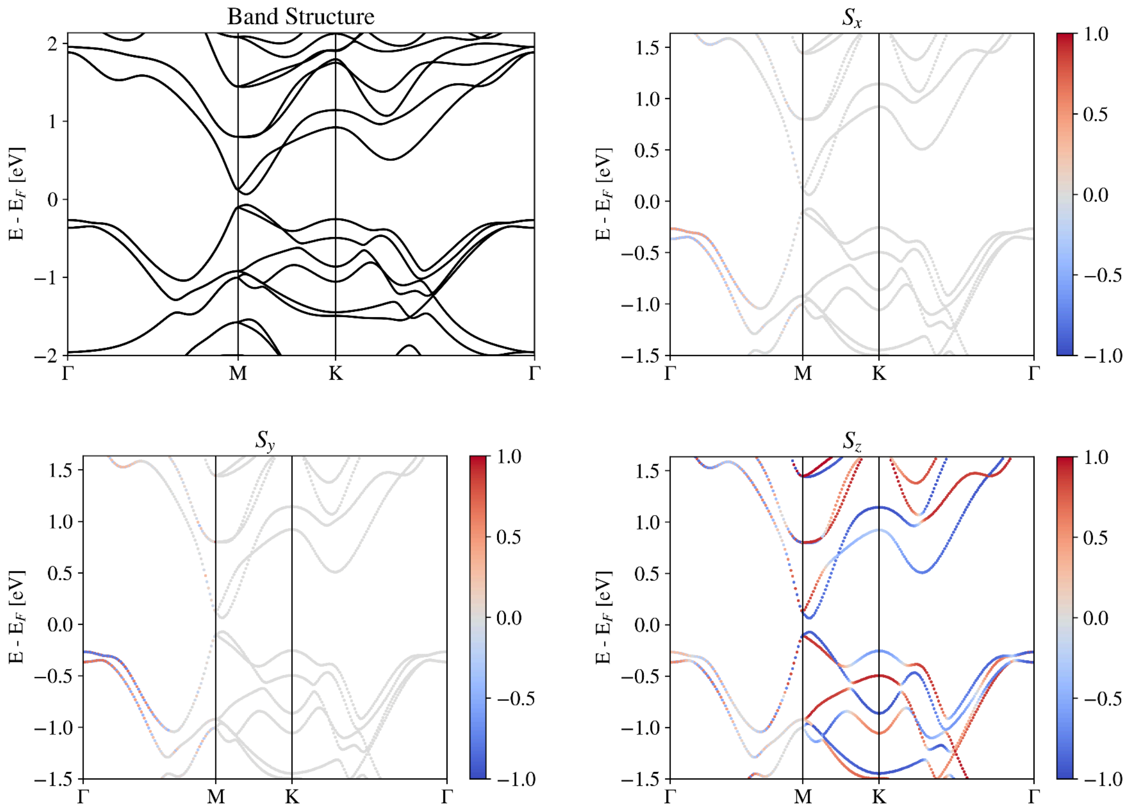


Figure 473: Band structure and spin polarization projections.

## 2.237 Ir2Se2-53337987551a

- **Formula**: Ir2Se2
- **Structural Cluster**: AB-5
- **Band gap (PBE)**: 0.288 eV
- **Energy above convex hull (C2DB)**: 0.426 eV
- **Space group symbol**: $P\bar{6}m2$
- **Space group number**: 187
- **Polar structure**: False

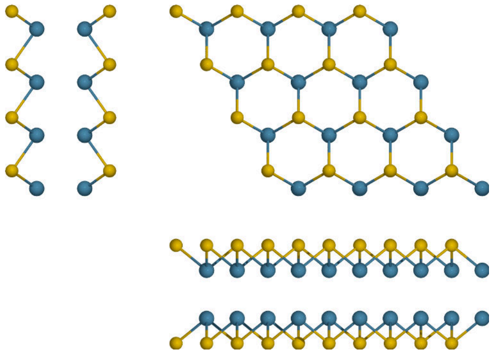

Figure 474: Structure representation

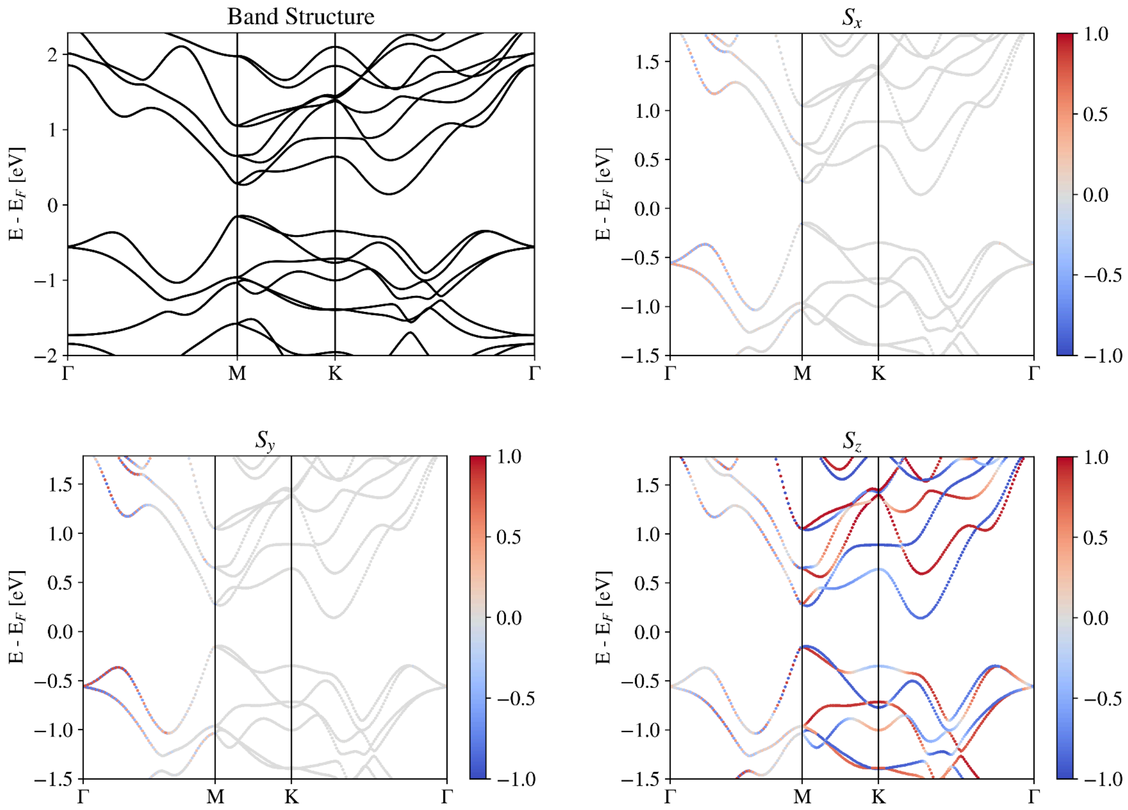


Figure 475: Band structure and spin polarization projections.

## 2.238 MgBr2-bee1987fb4e6

- **Formula**: MgBr2
- **Structural Cluster**: AB2-4
- **Band gap (PBE)**: 3.569 eV
- **Energy above convex hull (C2DB)**: 0.169 eV
- **Space group symbol**: $P\bar{6}m2$
- **Space group number**: 187
- **Polar structure**: False

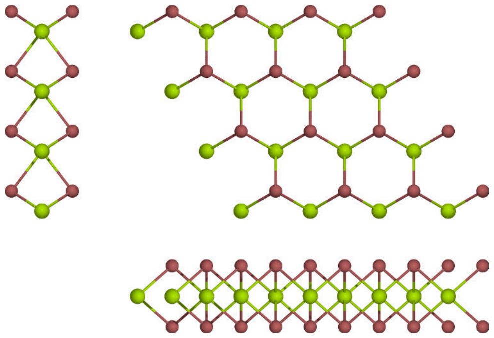

Figure 476: Structure representation

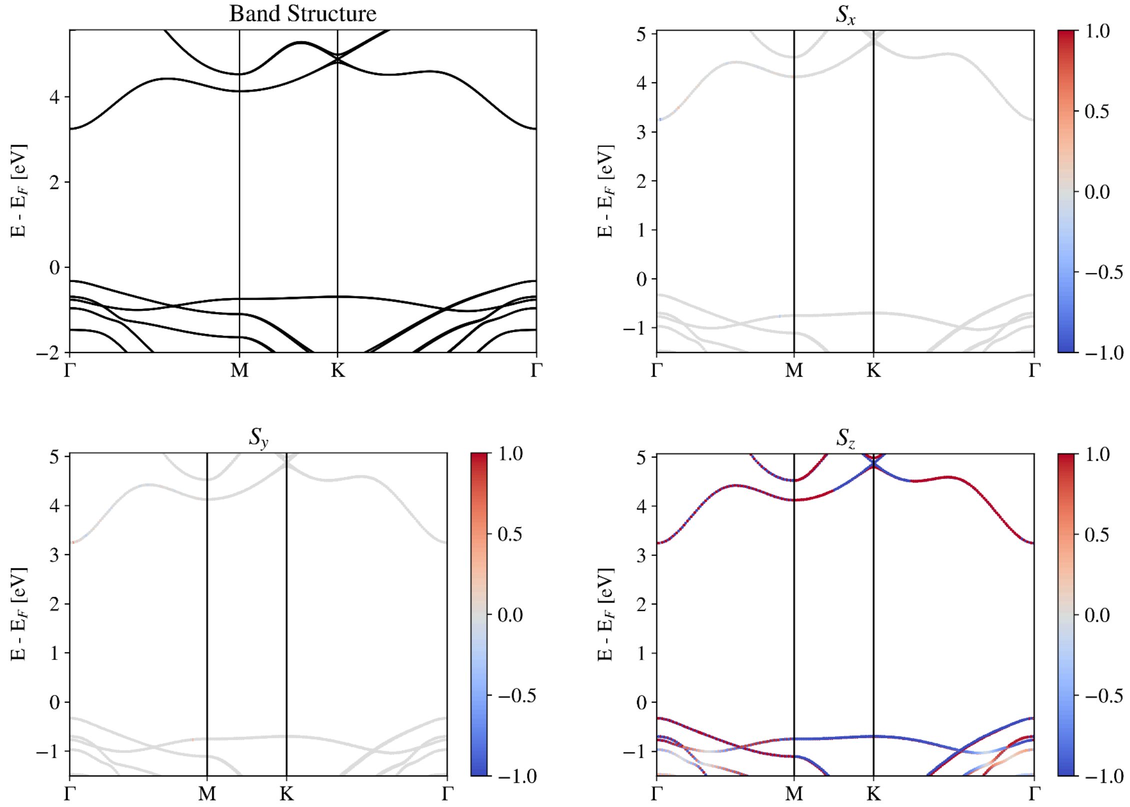


Figure 477: Band structure and spin polarization projections.

## 2.239 MgCl2-e73a5c5ae5ac

- **Formula**: MgCl2
- **Structural Cluster**: AB2-4
- **Band gap (PBE)**: 4.759 eV
- **Energy above convex hull (C2DB)**: 0.19 eV
- **Space group symbol**: $P\bar{6}m2$
- **Space group number**: 187
- **Polar structure**: False

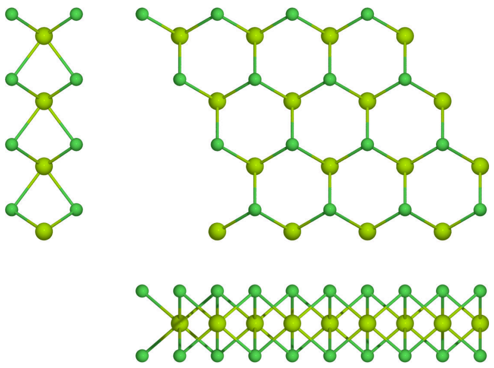

Figure 478: Structure representation

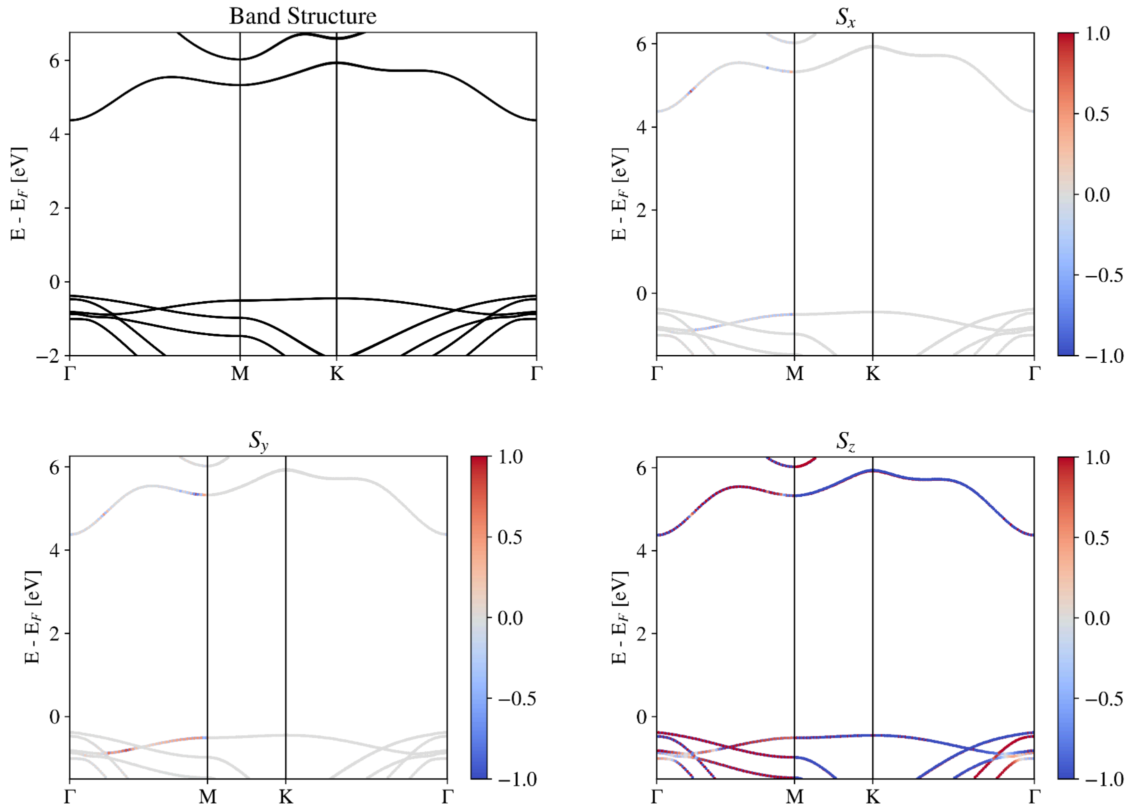


Figure 479: Band structure and spin polarization projections.

## 2.240 MgI2-67bb6819958f

- **Formula**: MgI2
- **Structural Cluster**: AB2-4
- **Band gap (PBE)**: 2.601 eV
- **Energy above convex hull (C2DB)**: 0.16 eV
- **Space group symbol**: $P\bar{6}m2$
- **Space group number**: 187
- **Polar structure**: False

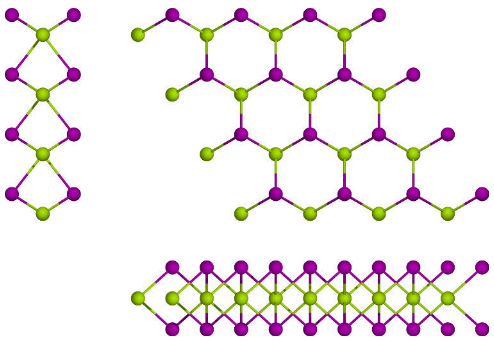

Figure 480: Structure representation

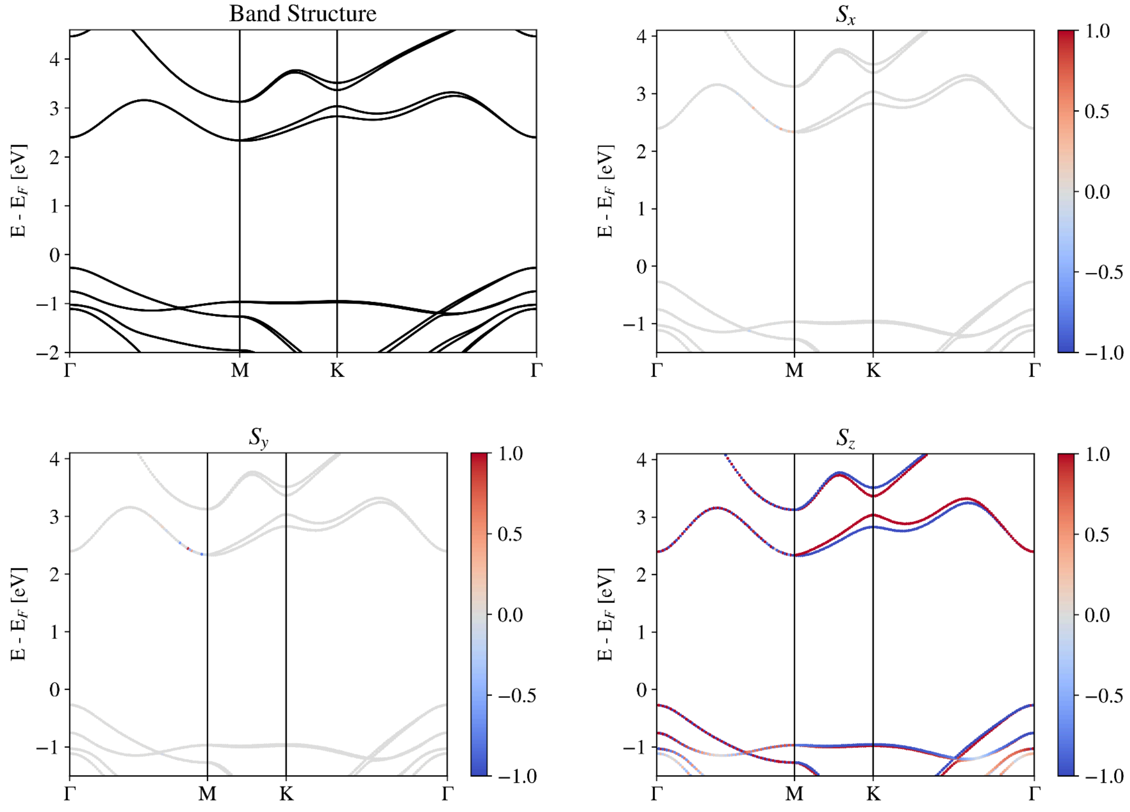


Figure 481: Band structure and spin polarization projections.

## 2.241 Mo2Cl6-61d74efeaeeb

- **Formula**: Mo2Cl6
- **Structural Cluster**: AB3-20
- **Band gap (PBE)**: 0.526 eV
- **Energy above convex hull (C2DB)**: 0.139 eV
- **Space group symbol**: $P\bar{6}2m$
- **Space group number**: 189
- **Polar structure**: False

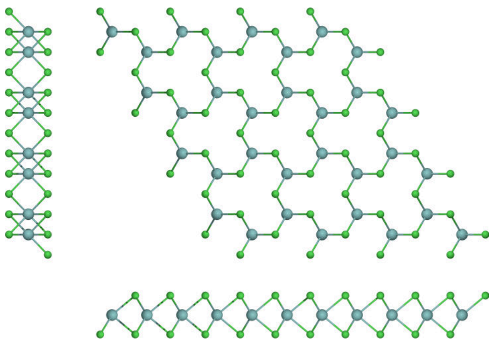

Figure 482: Structure representation

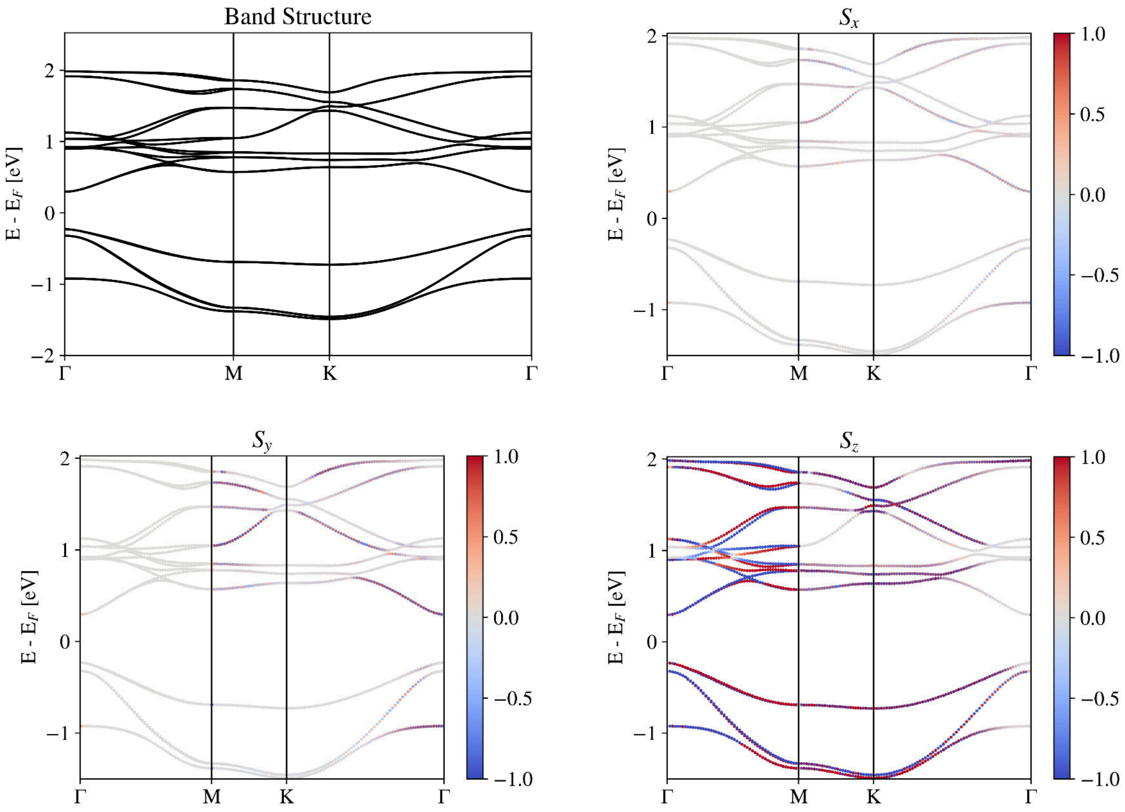


Figure 483: Band structure and spin polarization projections.

## 2.242 Mo2W2S8-449640ec4d30

- **Formula**: Mo2W2S8
- **Structural Cluster**: ABC4-22
- **Band gap (PBE)**: 1.553 eV
- **Energy above convex hull (C2DB)**: 0.0 eV
- **Space group symbol**: *Pc*
- **Space group number**: 7
- **Polar structure**: True

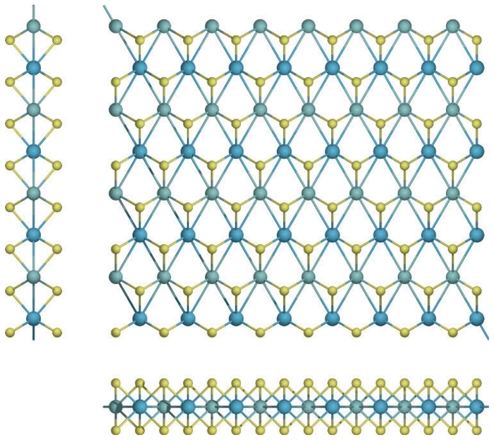

Figure 484: Structure representation

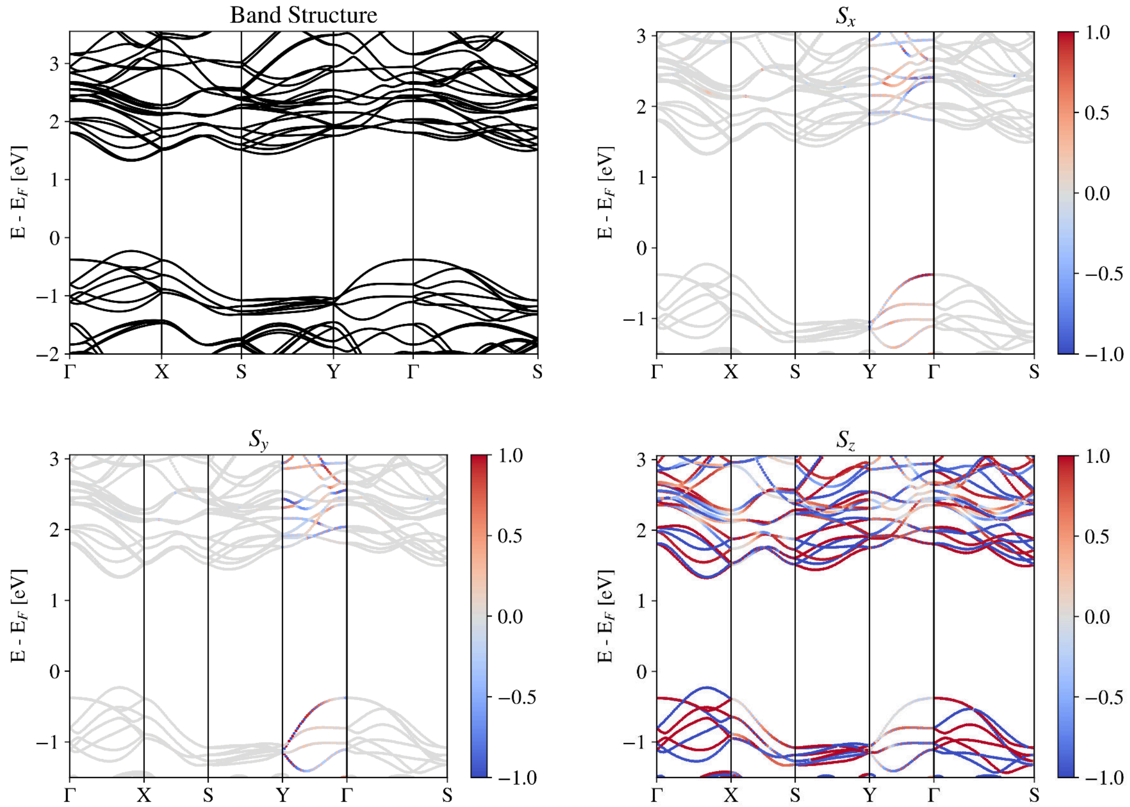


Figure 485: Band structure and spin polarization projections.

## 2.243 Mo2W2Se8-a1d716aad84d

- **Formula**: Mo2W2Se8
- **Structural Cluster**: ABC4-22
- **Band gap (PBE)**: 1.288 eV
- **Energy above convex hull (C2DB)**: 0.0 eV
- **Space group symbol**: $P1$
- **Space group number**: 1
- **Polar structure**: True

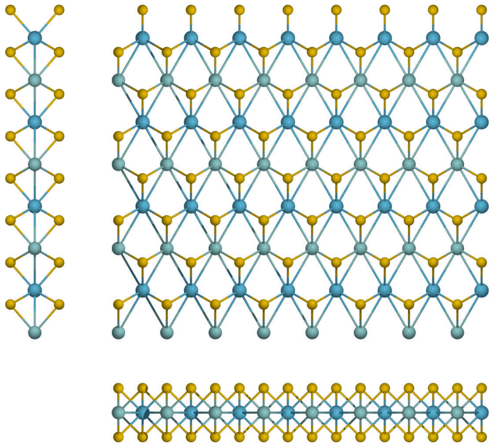

Figure 486: Structure representation

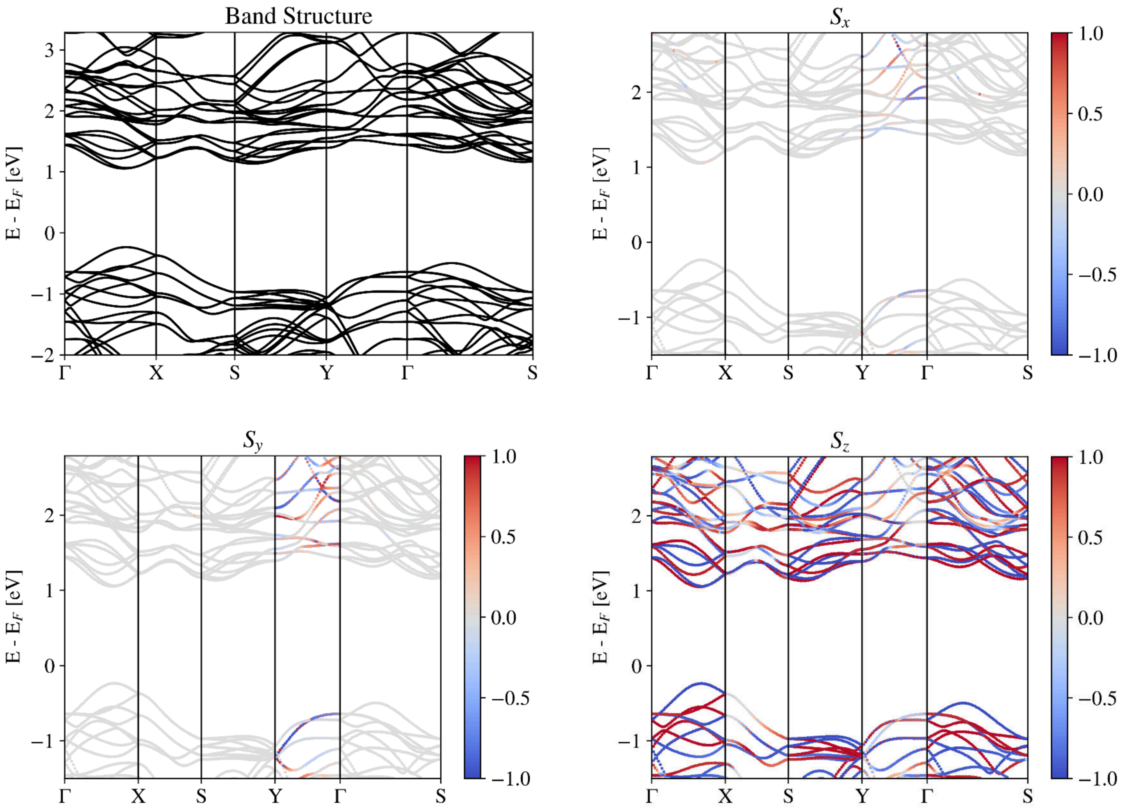


Figure 487: Band structure and spin polarization projections.

## 2.244 Mo2W2Te8-c04fc052f2ca

- **Formula**: Mo2W2Te8
- **Structural Cluster**: ABC4-22
- **Band gap (PBE)**: 0.879 eV
- **Energy above convex hull (C2DB)**: 0.011 eV
- **Space group symbol**: *Pm*
- **Space group number**: 6
- **Polar structure**: True

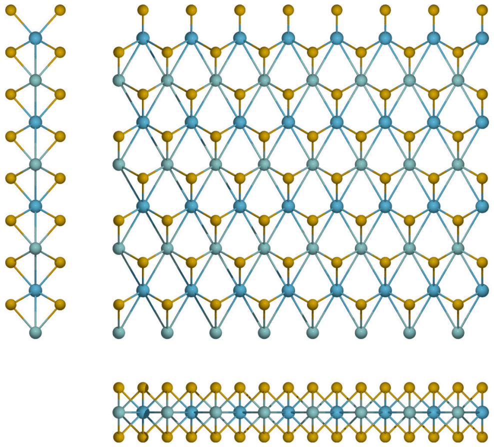

Figure 488: Structure representation

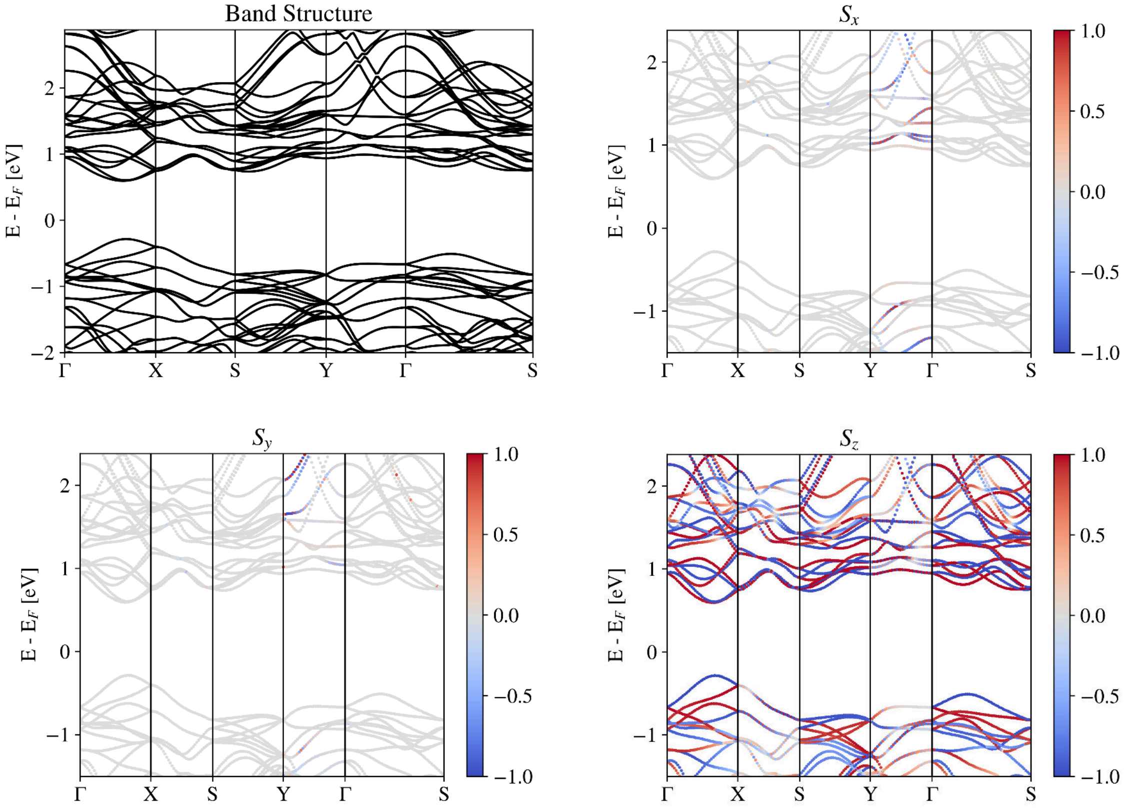


Figure 489: Band structure and spin polarization projections.

## 2.245 MoCr3S8-3fb52099b370

- **Formula**: MoCr3S8
- **Structural Cluster**: AB3C8-22
- **Band gap (PBE)**: 0.922 eV
- **Energy above convex hull (C2DB)**: 0.011 eV
- **Space group symbol**: $P1$
- **Space group number**: 1
- **Polar structure**: True

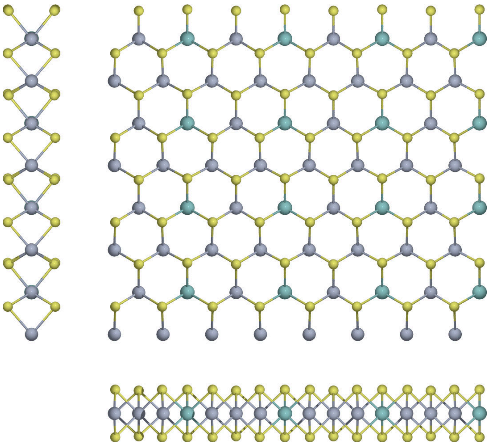

Figure 490: Structure representation

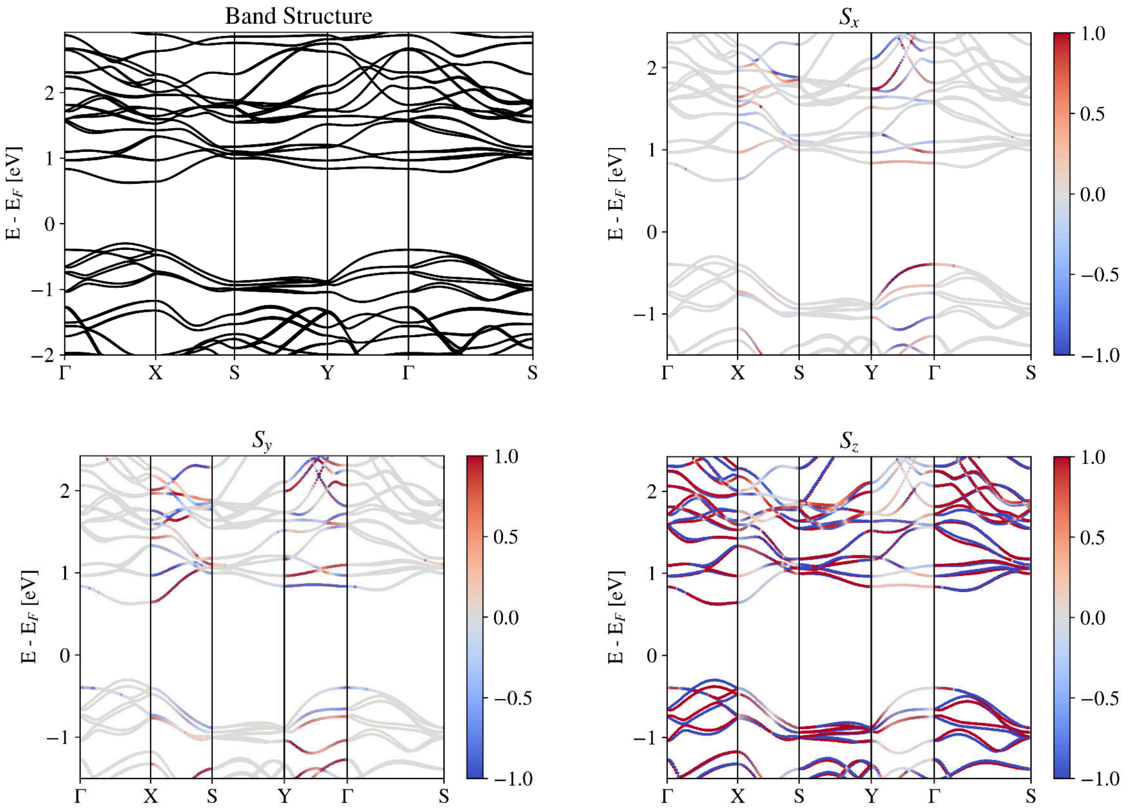


Figure 491: Band structure and spin polarization projections.

## 2.246 MoCr3Se8-961c37d6e527

- **Formula**: MoCr3Se8
- **Structural Cluster**: AB3C8-22
- **Band gap (PBE)**: 0.726 eV
- **Energy above convex hull (C2DB)**: 0.01 eV
- **Space group symbol**: *Pm*
- **Space group number**: 6
- **Polar structure**: True

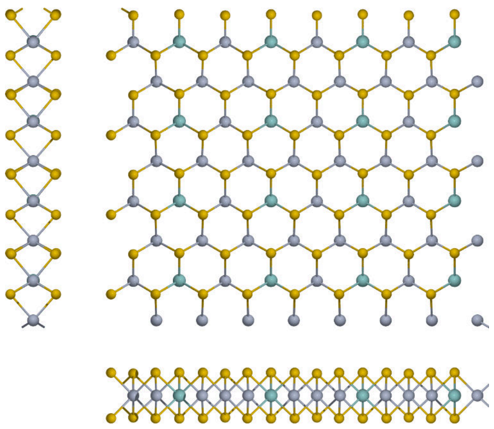

Figure 492: Structure representation

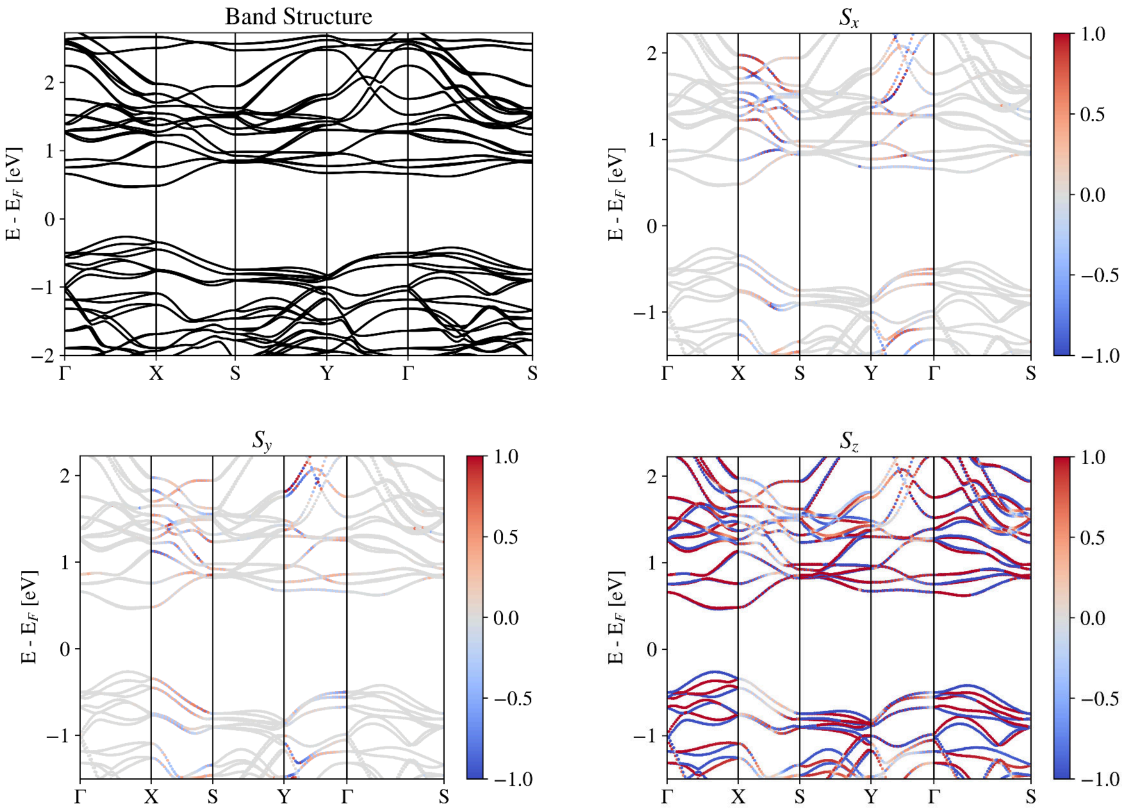


Figure 493: Band structure and spin polarization projections.

## 2.247 MoCr3Te8-899032b4ad0c

- **Formula**: MoCr3Te8
- **Structural Cluster**: AB3C8-22
- **Band gap (PBE)**: 0.481 eV
- **Energy above convex hull (C2DB)**: 0.087 eV
- **Space group symbol**: $P1$
- **Space group number**: 1
- **Polar structure**: True

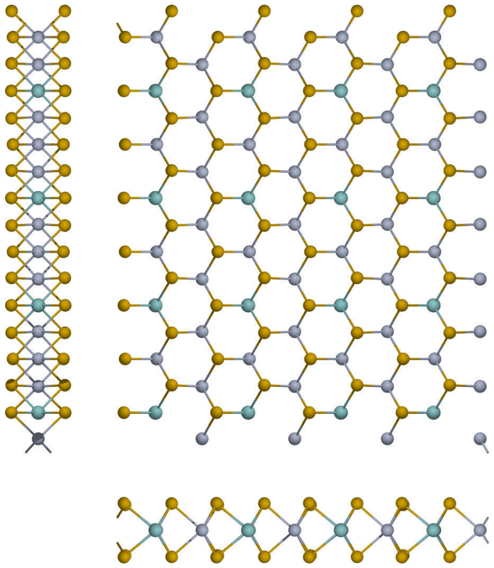

Figure 494: Structure representation

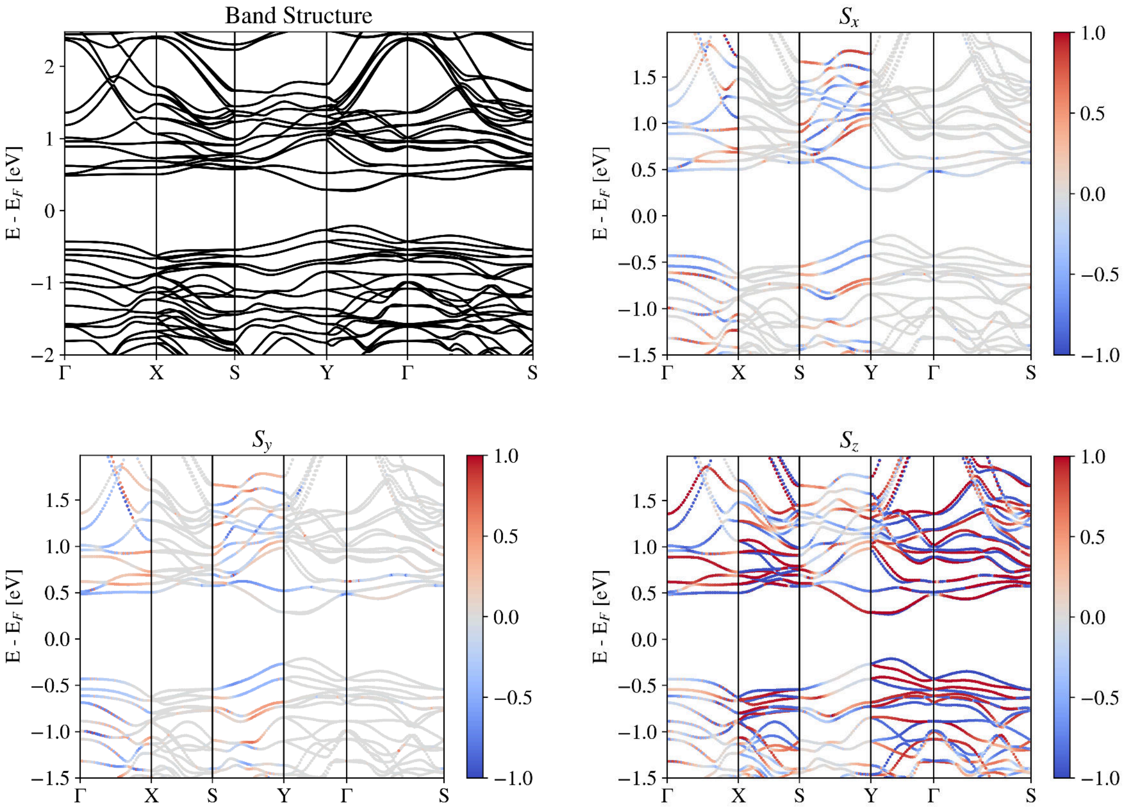


Figure 495: Band structure and spin polarization projections.

## 2.248 MoO2-152bd69757aa

- **Formula**: MoO2
- **Structural Cluster**: AB2-4
- **Band gap (PBE)**: 0.918 eV
- **Energy above convex hull (C2DB)**: 0.028 eV
- **Space group symbol**: $P\bar{6}m2$
- **Space group number**: 187
- **Polar structure**: False

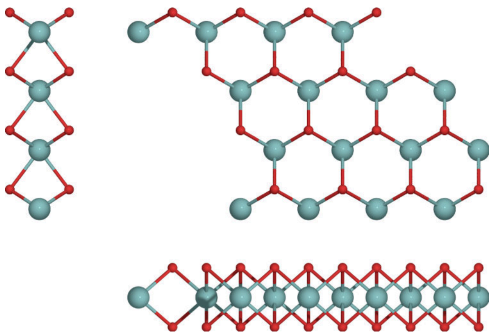

Figure 496: Structure representation

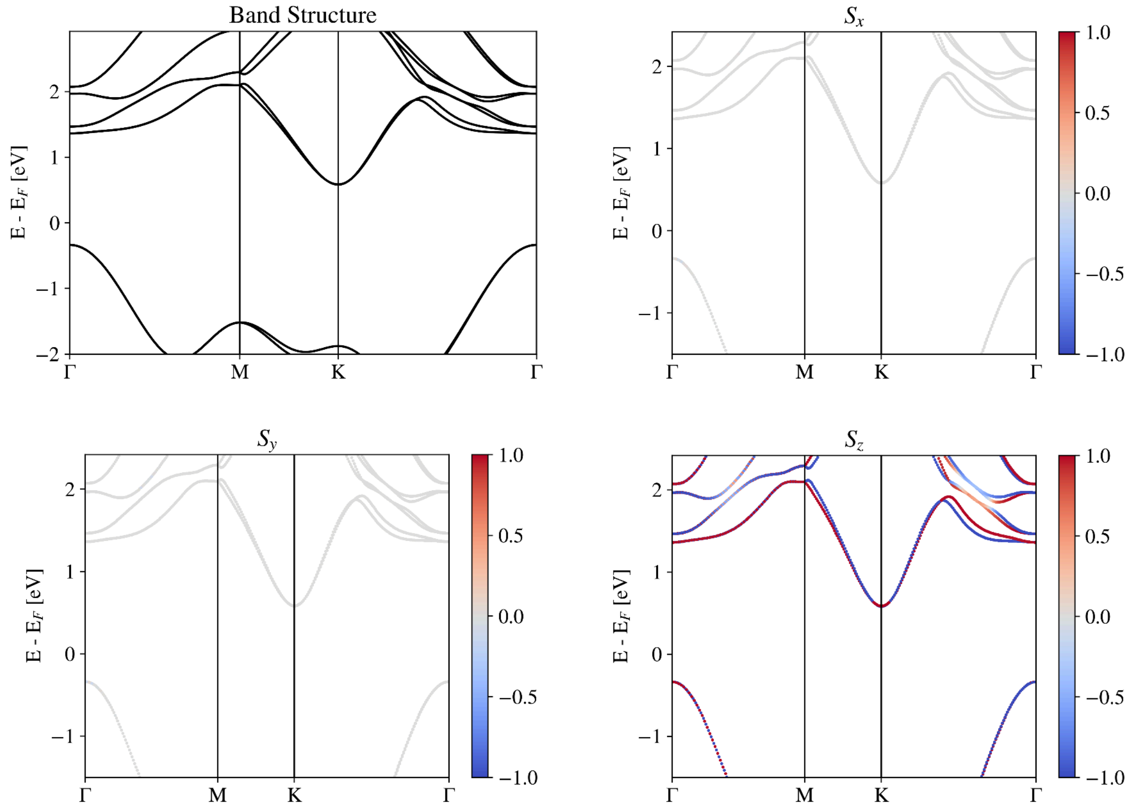


Figure 497: Band structure and spin polarization projections.

## 2.249 MoS2-b3b4685fb6e1

- **Formula**: MoS2
- **Structural Cluster**: AB2-4
- **Band gap (PBE)**: 1.604 eV
- **Energy above convex hull (C2DB)**: 0.0 eV
- **Space group symbol**: $P\bar{6}m2$
- **Space group number**: 187
- **Polar structure**: False

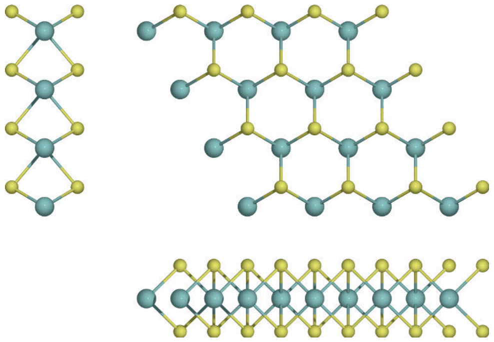

Figure 498: Structure representation

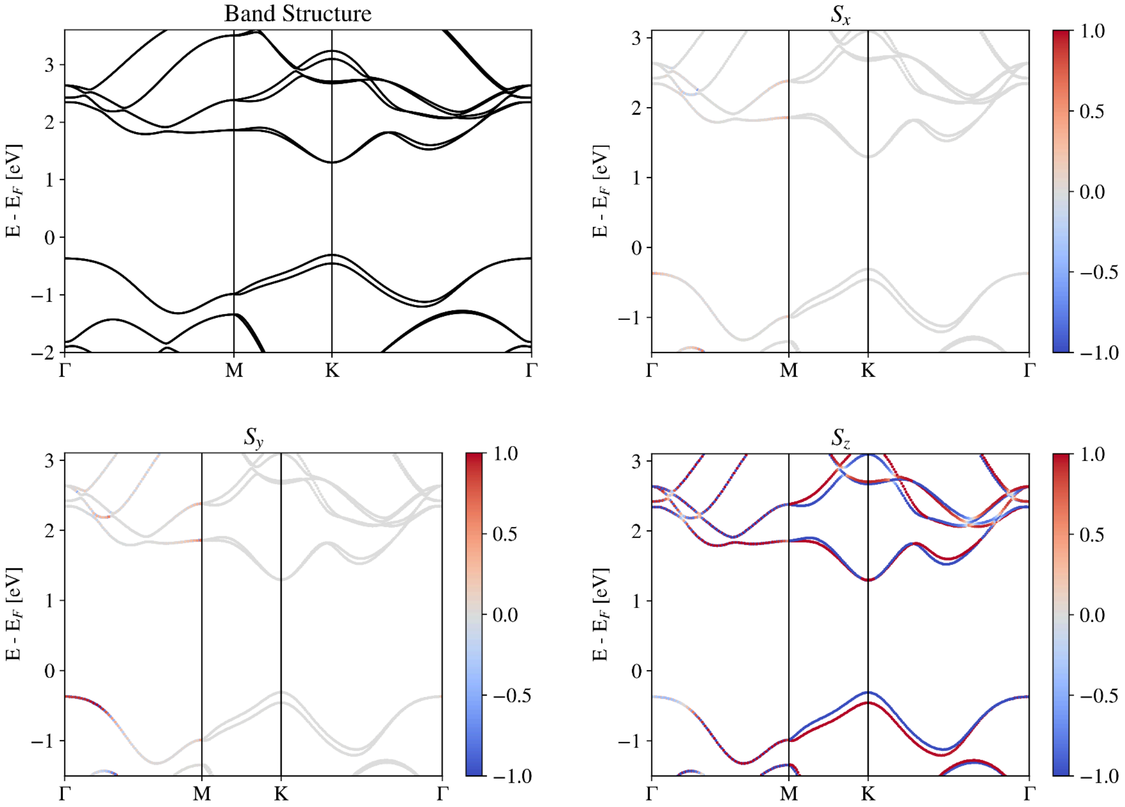


Figure 499: Band structure and spin polarization projections.

## 2.250 MoSSe-de7ac5fc6945

- **Formula**: MoSSe
- **Structural Cluster**: ABC-4
- **Band gap (PBE)**: 1.474 eV
- **Energy above convex hull (C2DB)**: 0.009 eV
- **Space group symbol**: $P3m1$
- **Space group number**: 156
- **Polar structure**: True

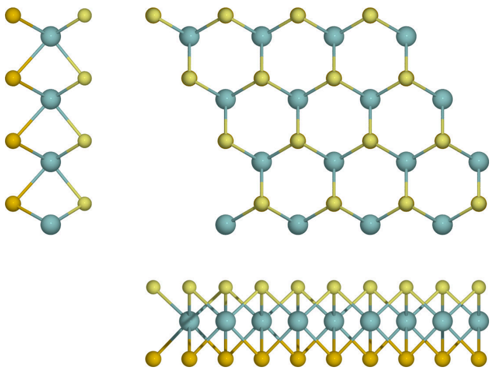

Figure 500: Structure representation

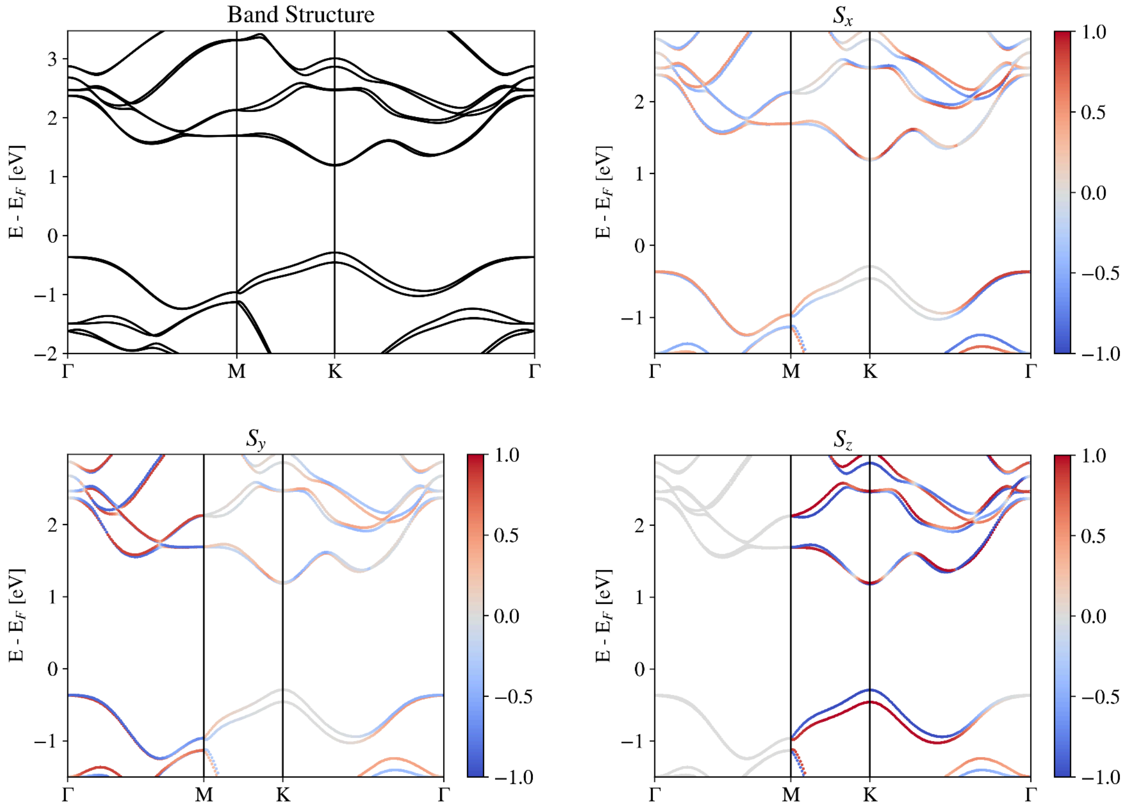


Figure 501: Band structure and spin polarization projections.

## 2.251 MoSTe-2ea941c8bc3c

- **Formula**: MoSTe
- **Structural Cluster**: ABC-4
- **Band gap (PBE)**: 0.196 eV
- **Energy above convex hull (C2DB)**: 0.223 eV
- **Space group symbol**: $P3m1$
- **Space group number**: 156
- **Polar structure**: True

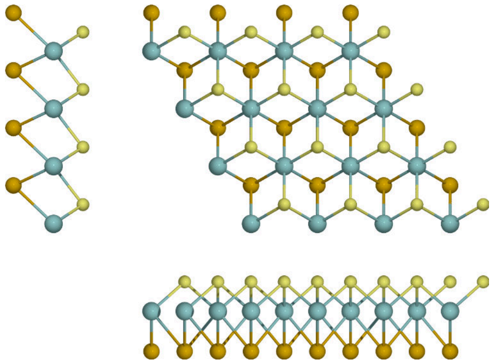

Figure 502: Structure representation

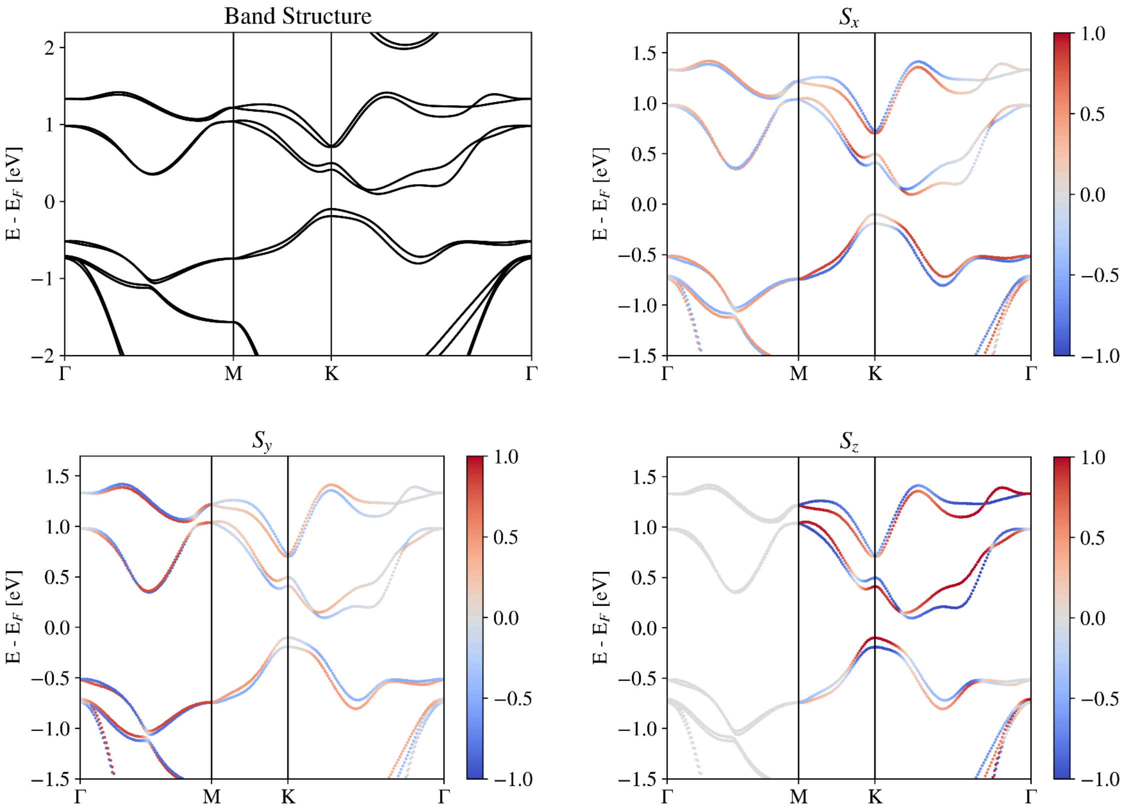


Figure 503: Band structure and spin polarization projections.

## 2.252 MoSTe-e4bb8738150a

- **Formula**: MoSTe
- **Structural Cluster**: ABC-4
- **Band gap (PBE)**: 1.027 eV
- **Energy above convex hull (C2DB)**: 0.065 eV
- **Space group symbol**: $P3m1$
- **Space group number**: 156
- **Polar structure**: True

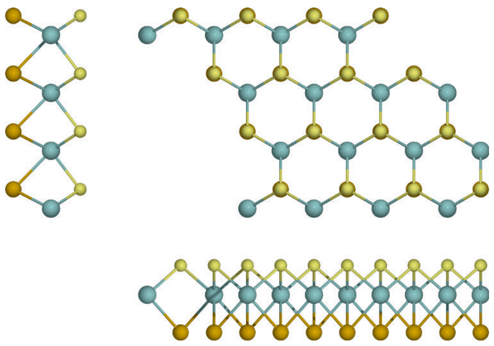

Figure 504: Structure representation

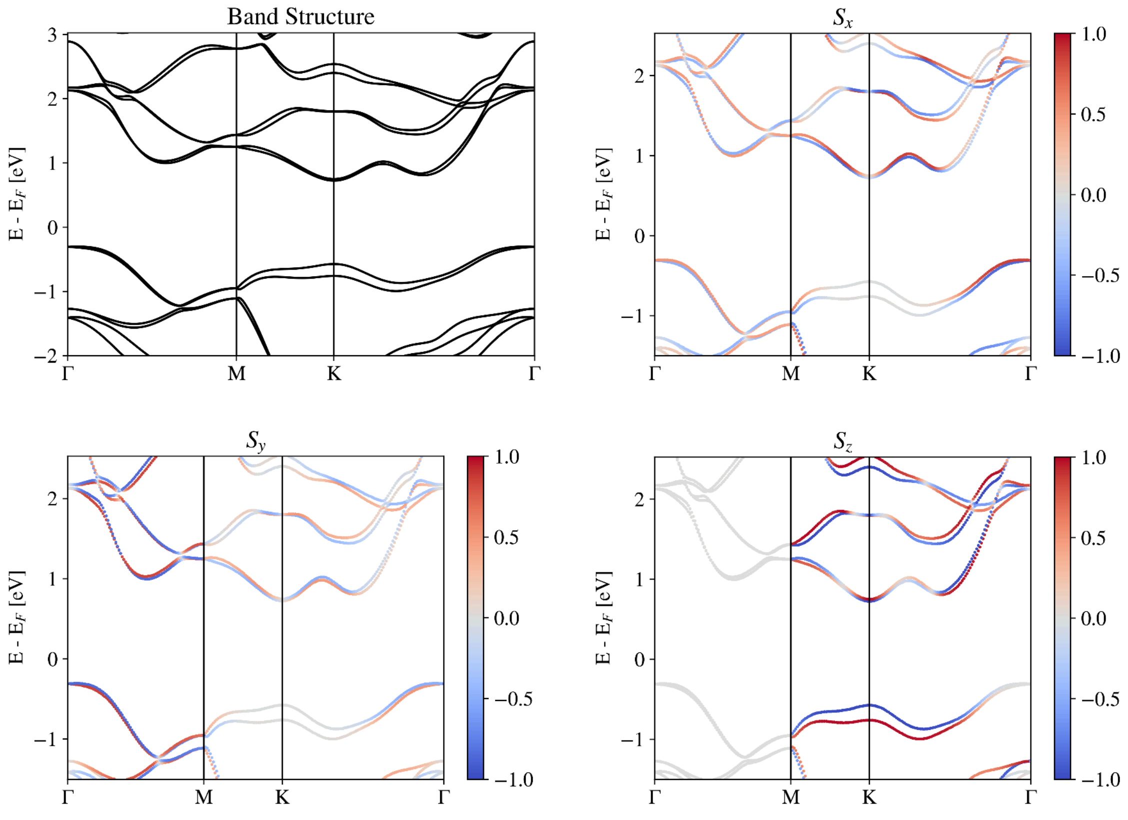


Figure 505: Band structure and spin polarization projections.

## 2.253 MoSe2-f61b14d398c7

- **Formula**: MoSe2
- **Structural Cluster**: AB2-4
- **Band gap (PBE)**: 1.342 eV
- **Energy above convex hull (C2DB)**: 0.0 eV
- **Space group symbol**: $P\bar{6}m2$
- **Space group number**: 187
- **Polar structure**: False

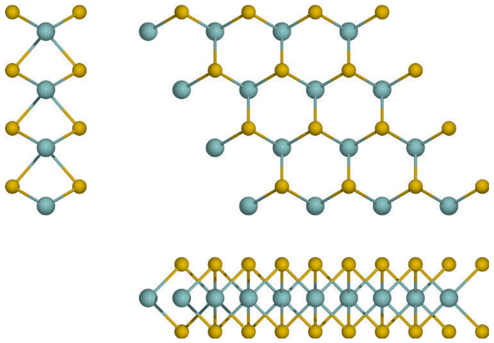

Figure 506: Structure representation

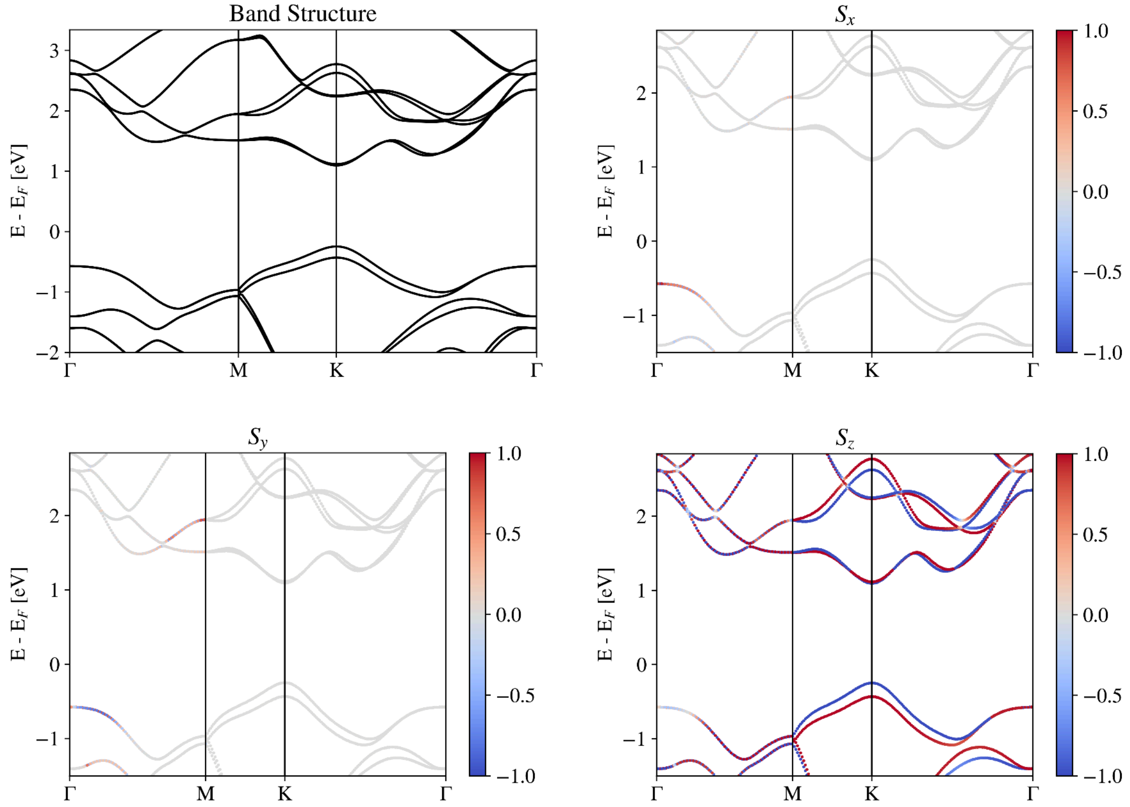


Figure 507: Band structure and spin polarization projections.

## 2.254 MoSeTe-42eb12e7b656

- **Formula**: MoSeTe
- **Structural Cluster**: ABC-4
- **Band gap (PBE)**: 1.159 eV
- **Energy above convex hull (C2DB)**: 0.025 eV
- **Space group symbol**: $P3m1$
- **Space group number**: 156
- **Polar structure**: True

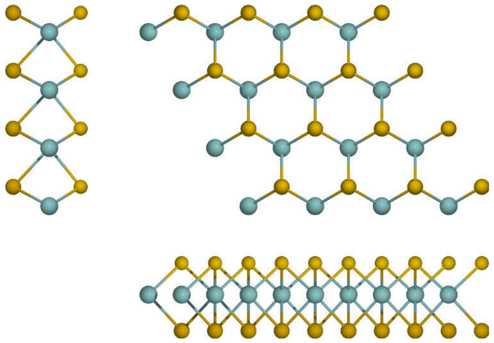

Figure 508: Structure representation

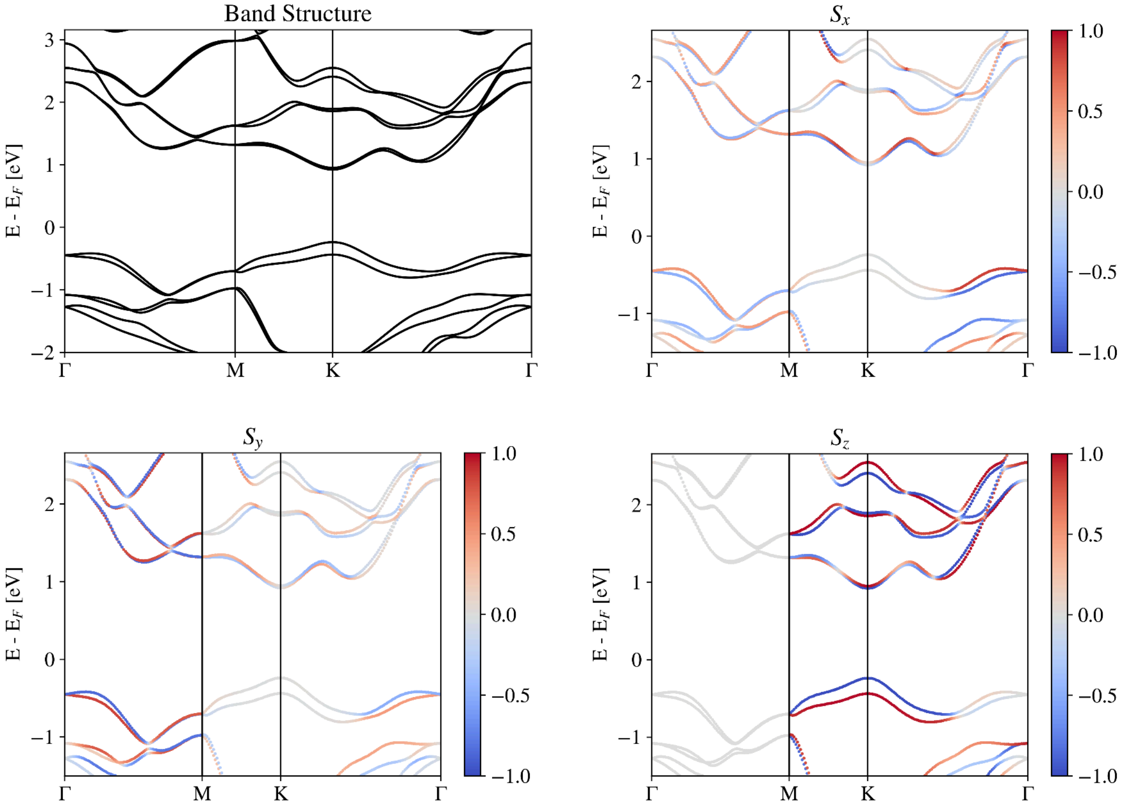


Figure 509: Band structure and spin polarization projections.

## 2.255 MoTe2-38a53176109a

- **Formula**: MoTe2
- **Structural Cluster**: AB2-4
- **Band gap (PBE)**: 0.956 eV
- **Energy above convex hull (C2DB)**: 0.0 eV
- **Space group symbol**: $P\bar{6}m2$
- **Space group number**: 187
- **Polar structure**: False

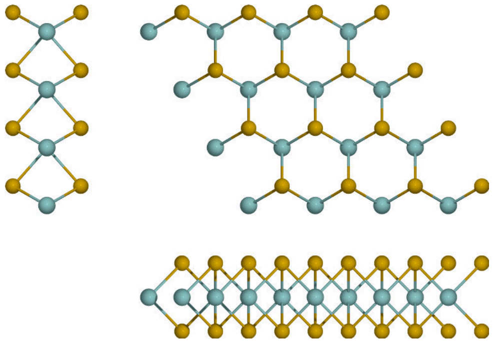

Figure 510: Structure representation

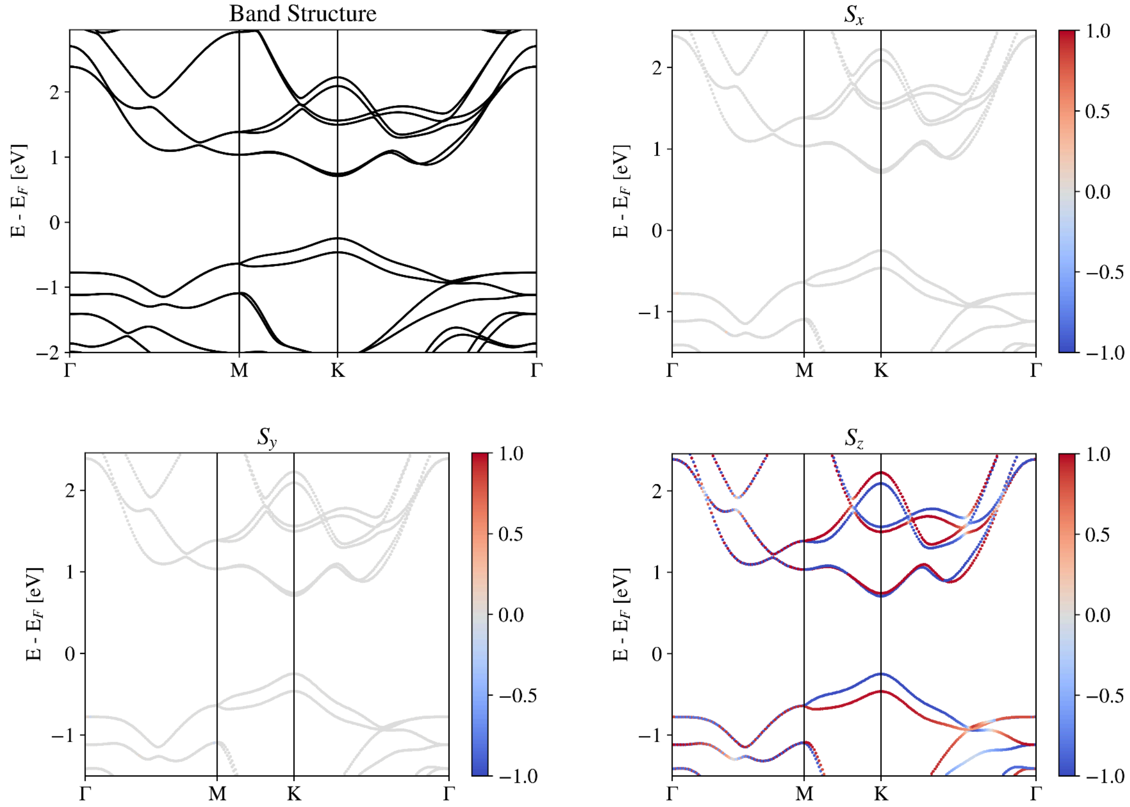


Figure 511: Band structure and spin polarization projections.

## 2.256 MoW3S8-2f6f133abcc8

- **Formula**: MoW3S8
- **Structural Cluster**: AB3C8-22
- **Band gap (PBE)**: 1.552 eV
- **Energy above convex hull (C2DB)**: 0.0 eV
- **Space group symbol**: $P1$
- **Space group number**: 1
- **Polar structure**: True

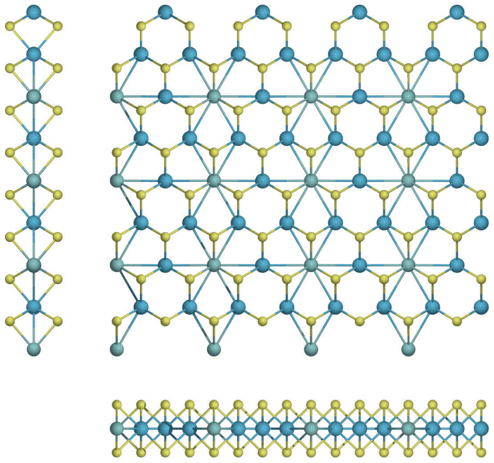

Figure 512: Structure representation

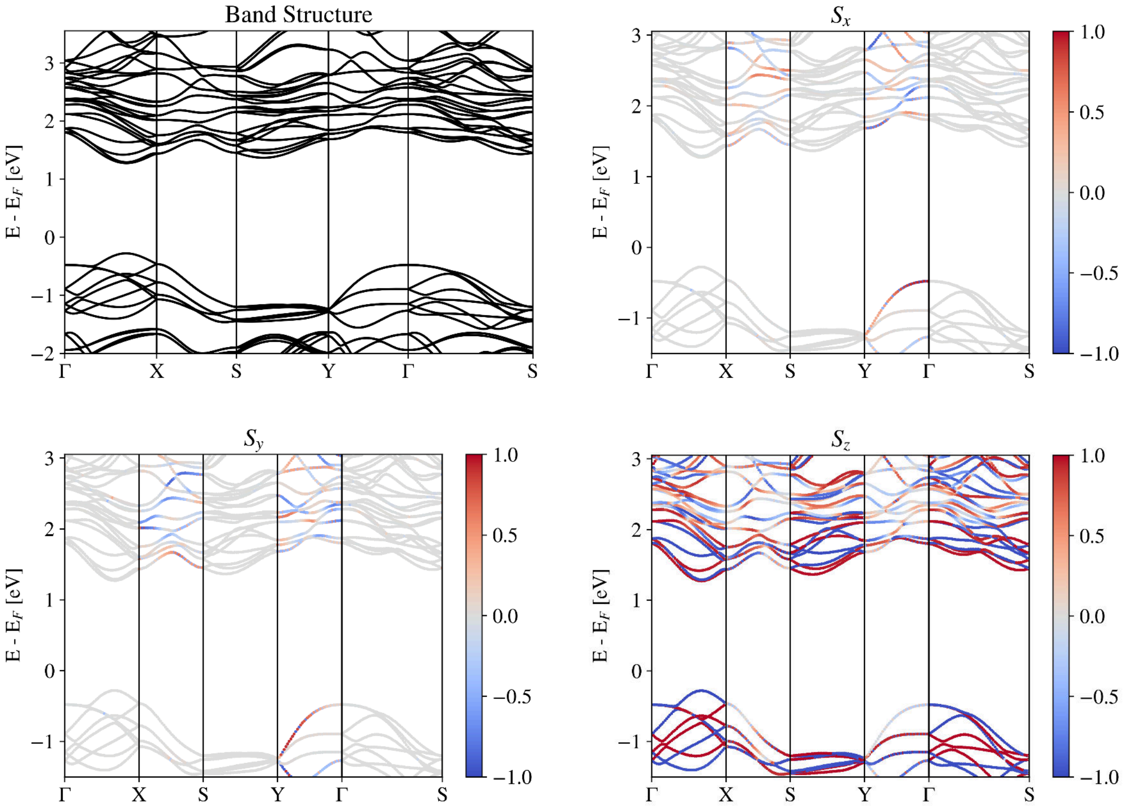


Figure 513: Band structure and spin polarization projections.

## 2.257 MoW3Se8-24d6cc0a0fed

- **Formula**: MoW3Se8
- **Structural Cluster**: AB3C8-22
- **Band gap (PBE)**: 1.276 eV
- **Energy above convex hull (C2DB)**: 0.0 eV
- **Space group symbol**: *Pm*
- **Space group number**: 6
- **Polar structure**: True

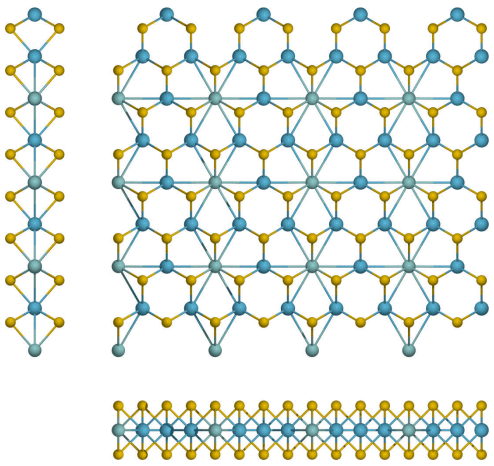

Figure 514: Structure representation

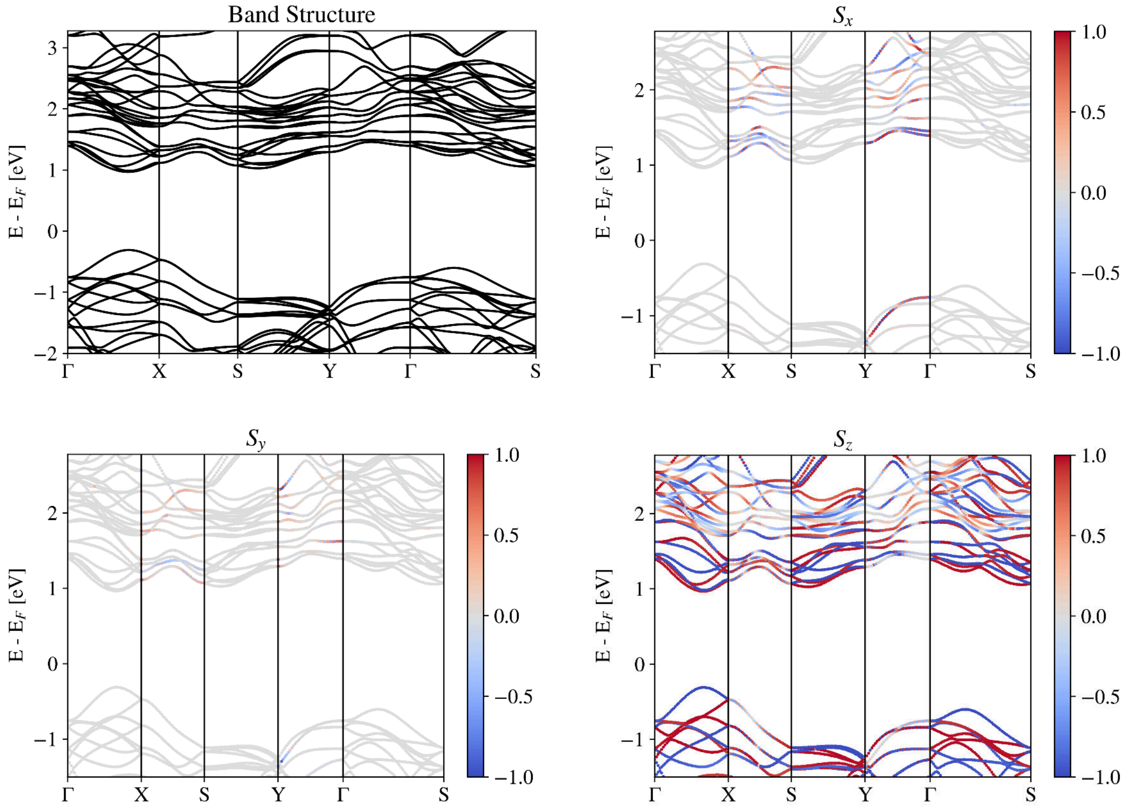


Figure 515: Band structure and spin polarization projections.

## 2.258 MoW3Te8-5c3fe56a1a89

- **Formula**: MoW3Te8
- **Structural Cluster**: AB3C8-22
- **Band gap (PBE)**: 0.825 eV
- **Energy above convex hull (C2DB)**: 0.018 eV
- **Space group symbol**: *Pm*
- **Space group number**: 6
- **Polar structure**: True

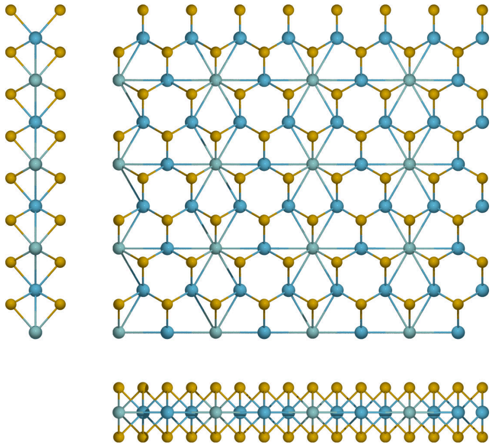

Figure 516: Structure representation

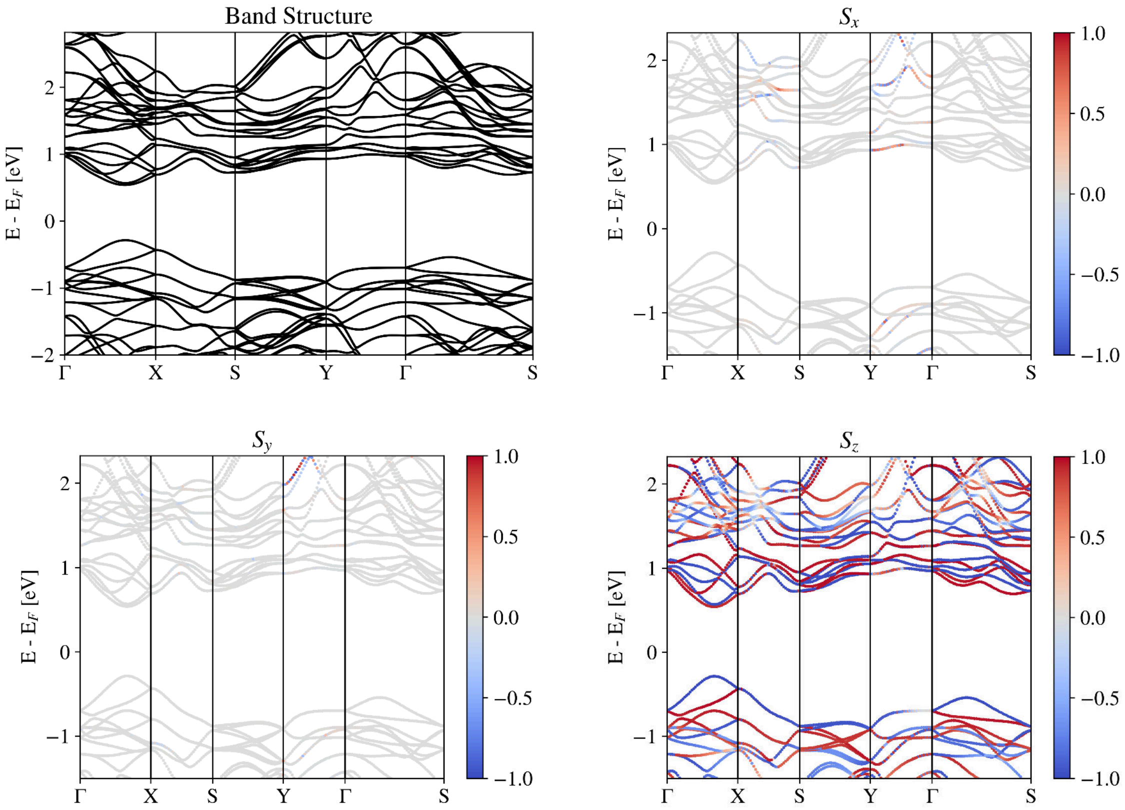


Figure 517: Band structure and spin polarization projections.

## 2.259 N2O2Hf3-bb4e40ae9164

- **Formula**: N2O2Hf3
- **Structural Cluster**: A2B2C3-13
- **Band gap (PBE)**: 0.323 eV
- **Energy above convex hull (C2DB)**: 0.088 eV
- **Space group symbol**: $P\bar{6}m2$
- **Space group number**: 187
- **Polar structure**: False

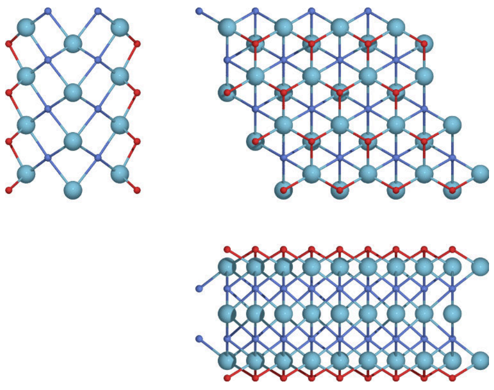

Figure 518: Structure representation

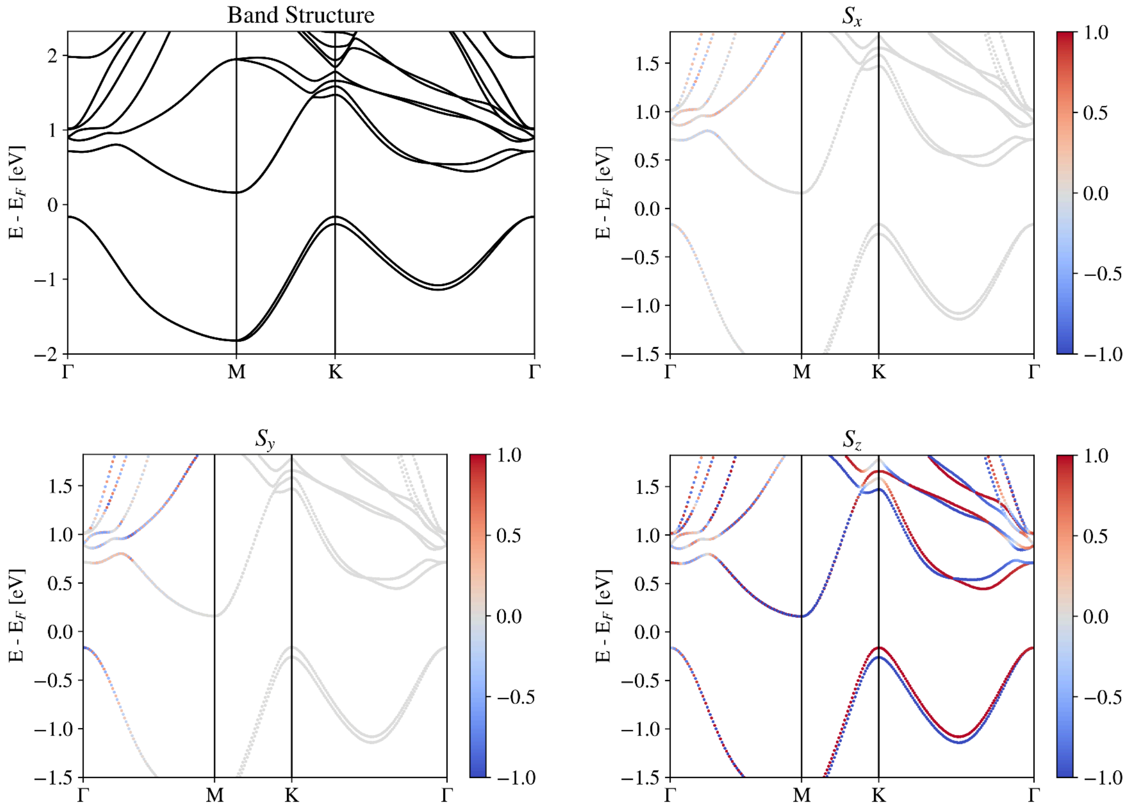


Figure 519: Band structure and spin polarization projections.

## 2.260 N2O2Zr3-c317fbd68215

- **Formula**: N2O2Zr3
- **Structural Cluster**: A2B2C3-13
- **Band gap (PBE)**: 0.404 eV
- **Energy above convex hull (C2DB)**: 0.094 eV
- **Space group symbol**: $P\bar{6}m2$
- **Space group number**: 187
- **Polar structure**: False

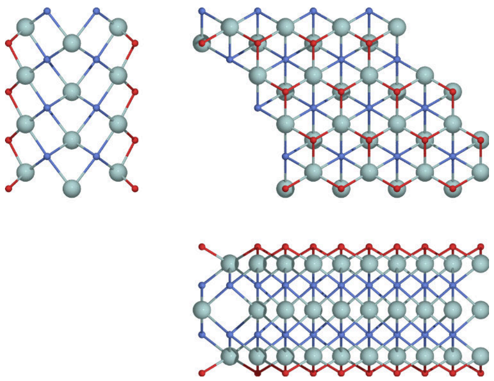

Figure 520: Structure representation

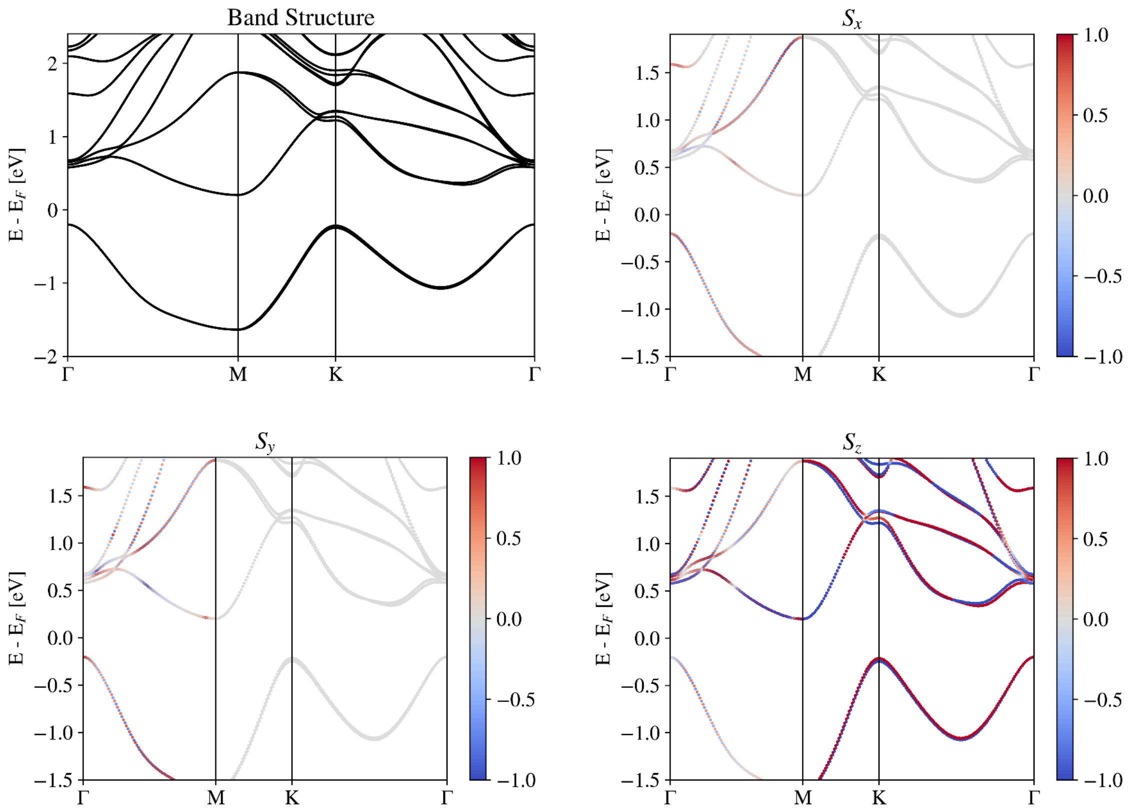


Figure 521: Band structure and spin polarization projections.

## 2.261 Nb2P2S6-c9aa3a9d876c

- **Formula**: Nb2P2S6
- **Structural Cluster**: ABC3-16
- **Band gap (PBE)**: 0.513 eV
- **Energy above convex hull (C2DB)**: 0.192 eV
- **Space group symbol**: $P1$
- **Space group number**: 1
- **Polar structure**: True

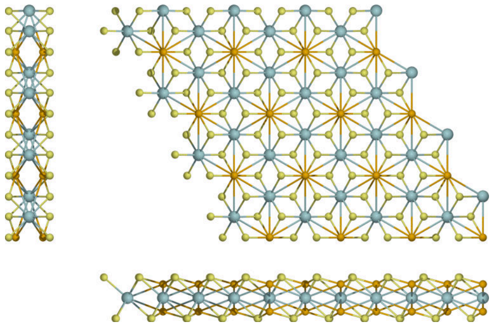

Figure 522: Structure representation

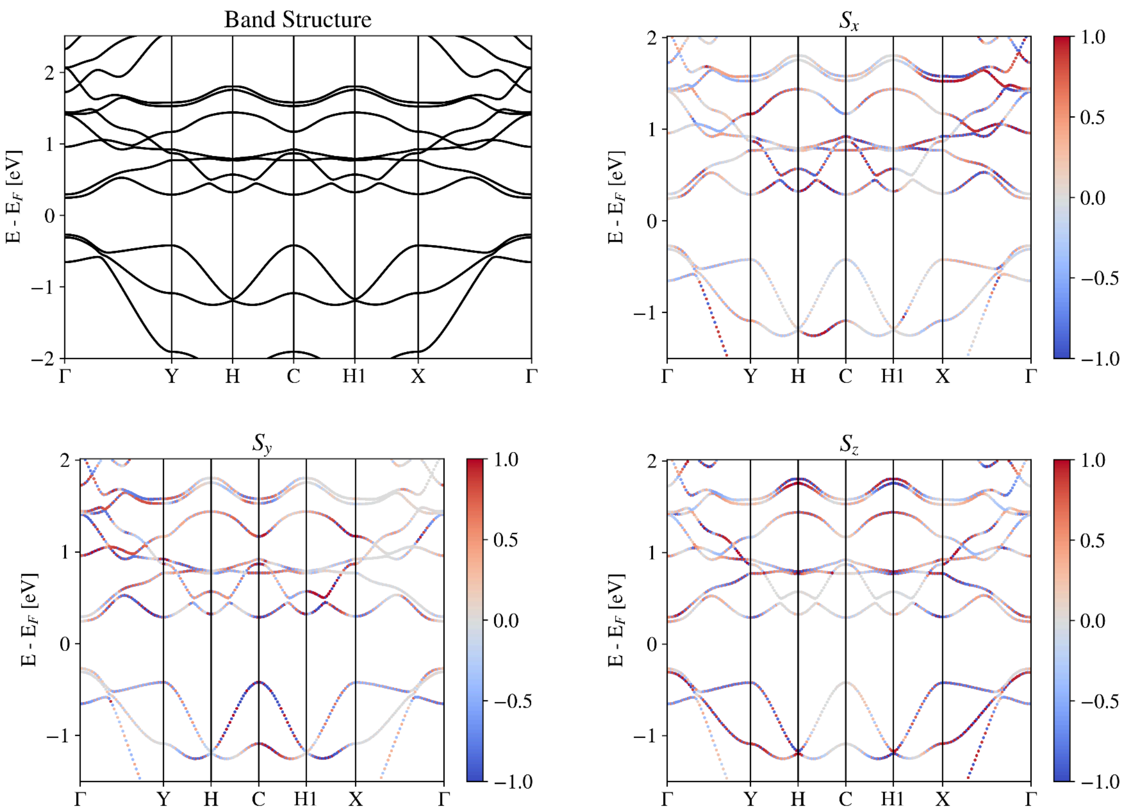


Figure 523: Band structure and spin polarization projections.

## 2.262 O2Pb2-20f098bd3f31

- **Formula**: O2Pb2
- **Structural Cluster**: AB-0
- **Band gap (PBE)**: 0.215 eV
- **Energy above convex hull (C2DB)**: 0.287 eV
- **Space group symbol**: *Pm*
- **Space group number**: 6
- **Polar structure**: True

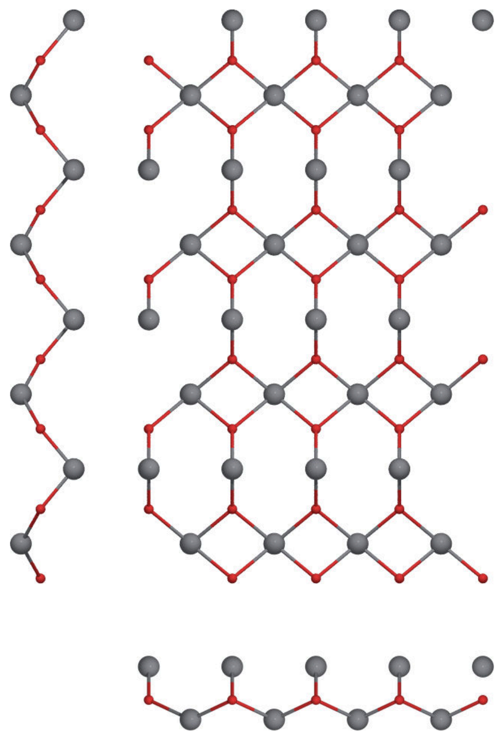

Figure 524: Structure representation

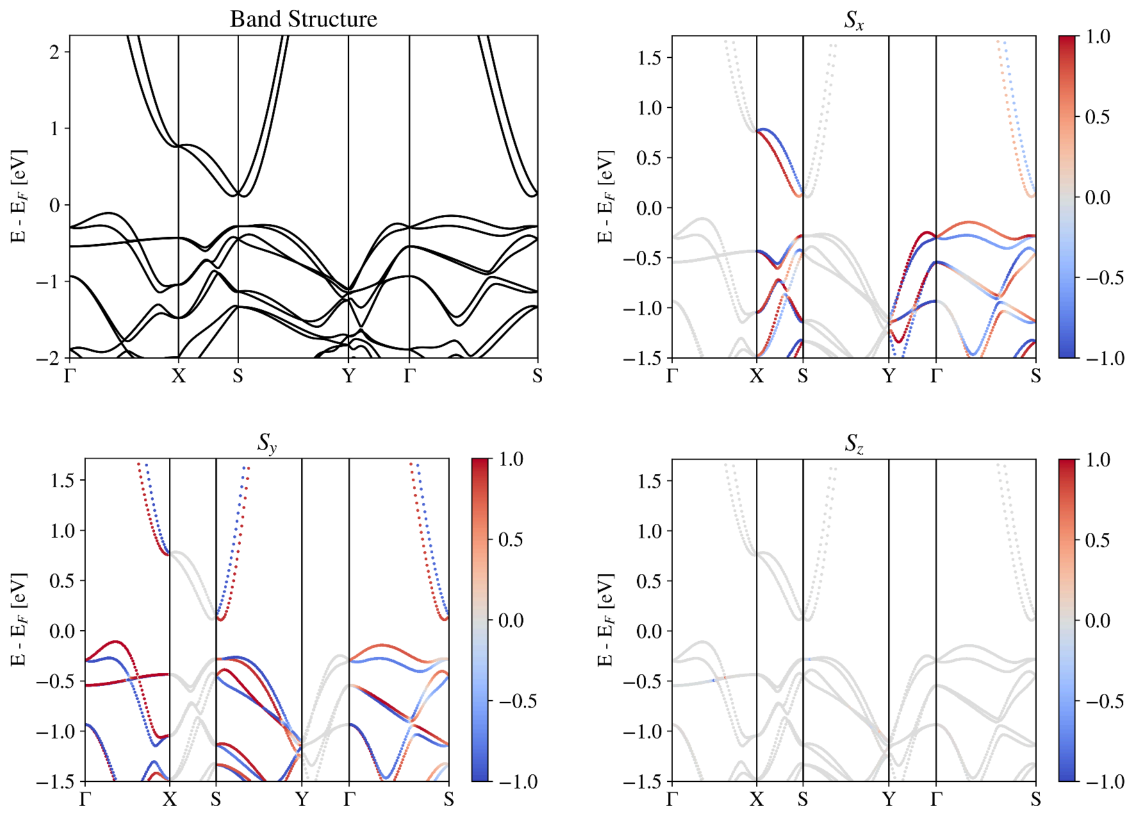


Figure 525: Band structure and spin polarization projections.

## 2.263 O2Rh2-740bf2751050

- **Formula**: O2Rh2
- **Structural Cluster**: AB-5
- **Band gap (PBE)**: 0.057 eV
- **Energy above convex hull (C2DB)**: 0.246 eV
- **Space group symbol**: $P\bar{6}m2$
- **Space group number**: 187
- **Polar structure**: False

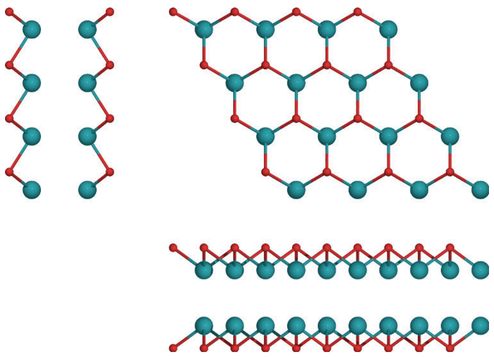

Figure 526: Structure representation

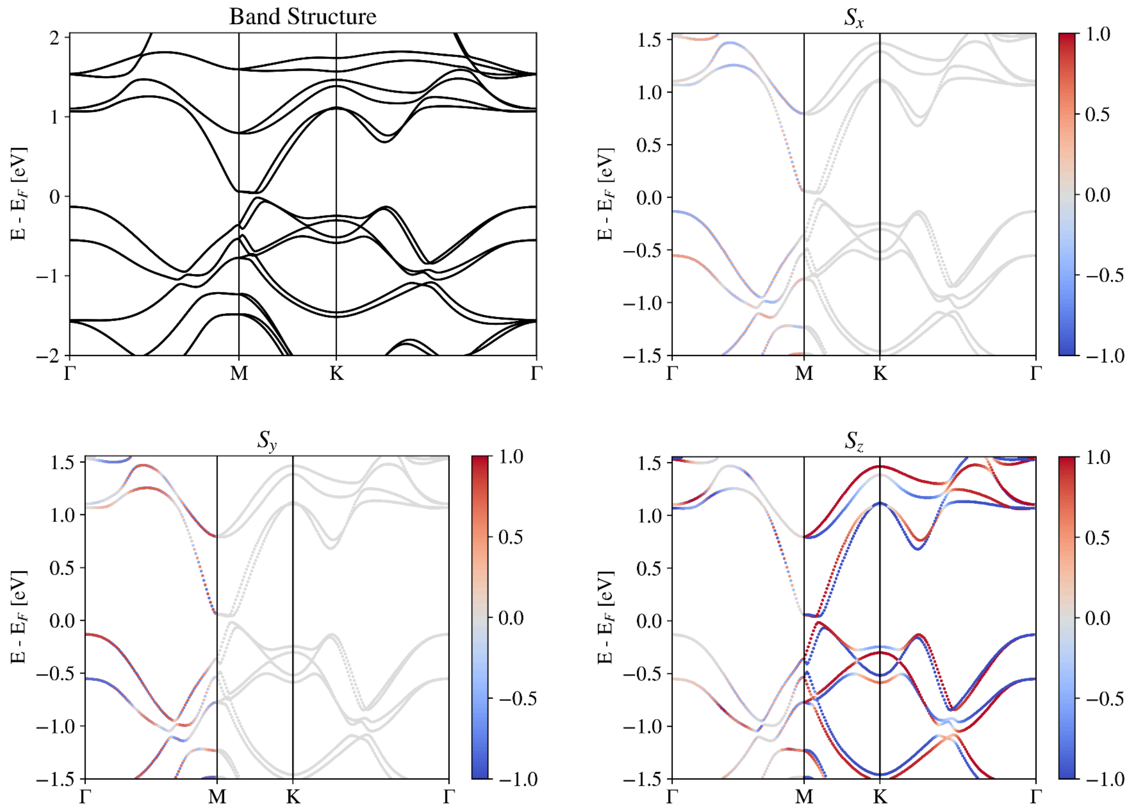


Figure 527: Band structure and spin polarization projections.

## 2.264 O2Sc2-b757b8efeeab

- **Formula**: O2Sc2
- **Structural Cluster**: AB-5
- **Band gap (PBE)**: 0.694 eV
- **Energy above convex hull (C2DB)**: 0.249 eV
- **Space group symbol**: $P\bar{6}m2$
- **Space group number**: 187
- **Polar structure**: False

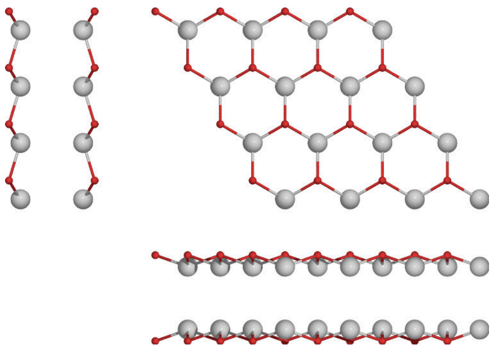

Figure 528: Structure representation

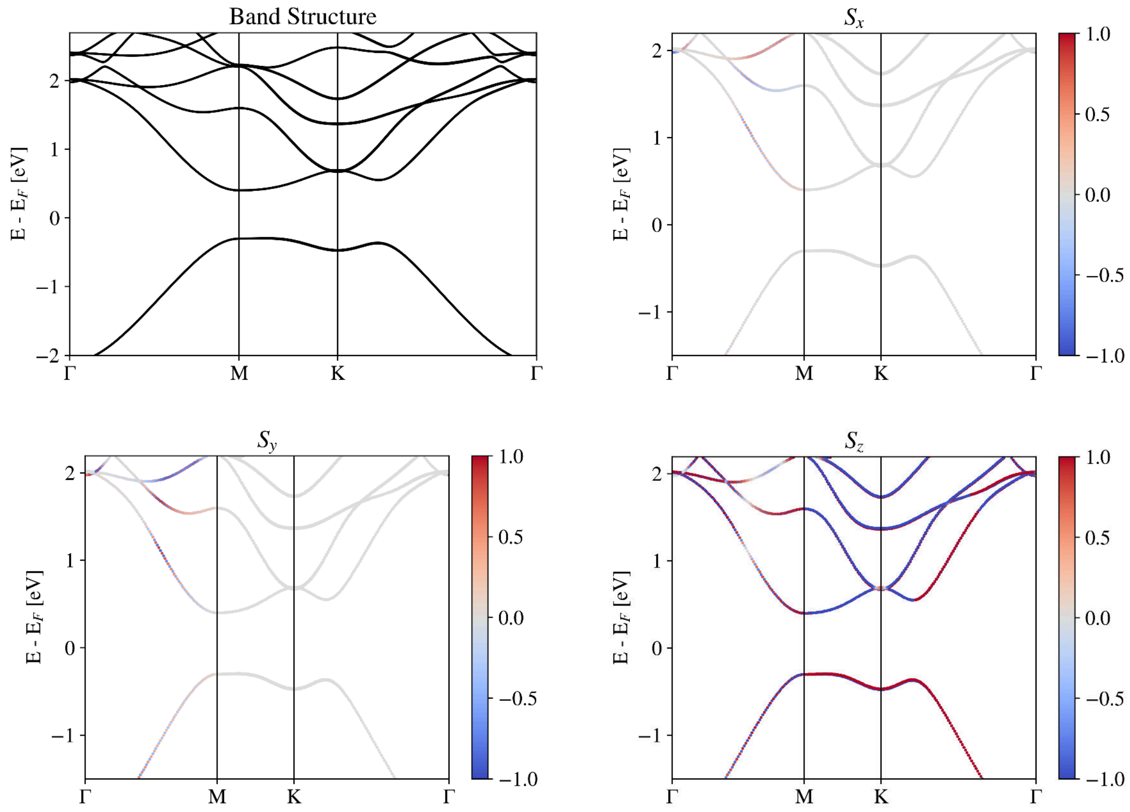


Figure 529: Band structure and spin polarization projections.

## 2.265 O2W2-42fa50003592

- **Formula**: O2W2
- **Structural Cluster**: AB-5
- **Band gap (PBE)**: 0.04 eV
- **Energy above convex hull (C2DB)**: 0.503 eV
- **Space group symbol**: $P\bar{6}m2$
- **Space group number**: 187
- **Polar structure**: False

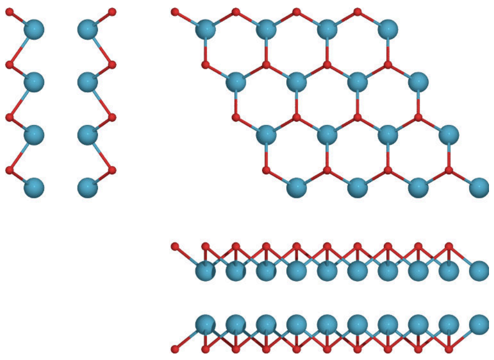

Figure 530: Structure representation

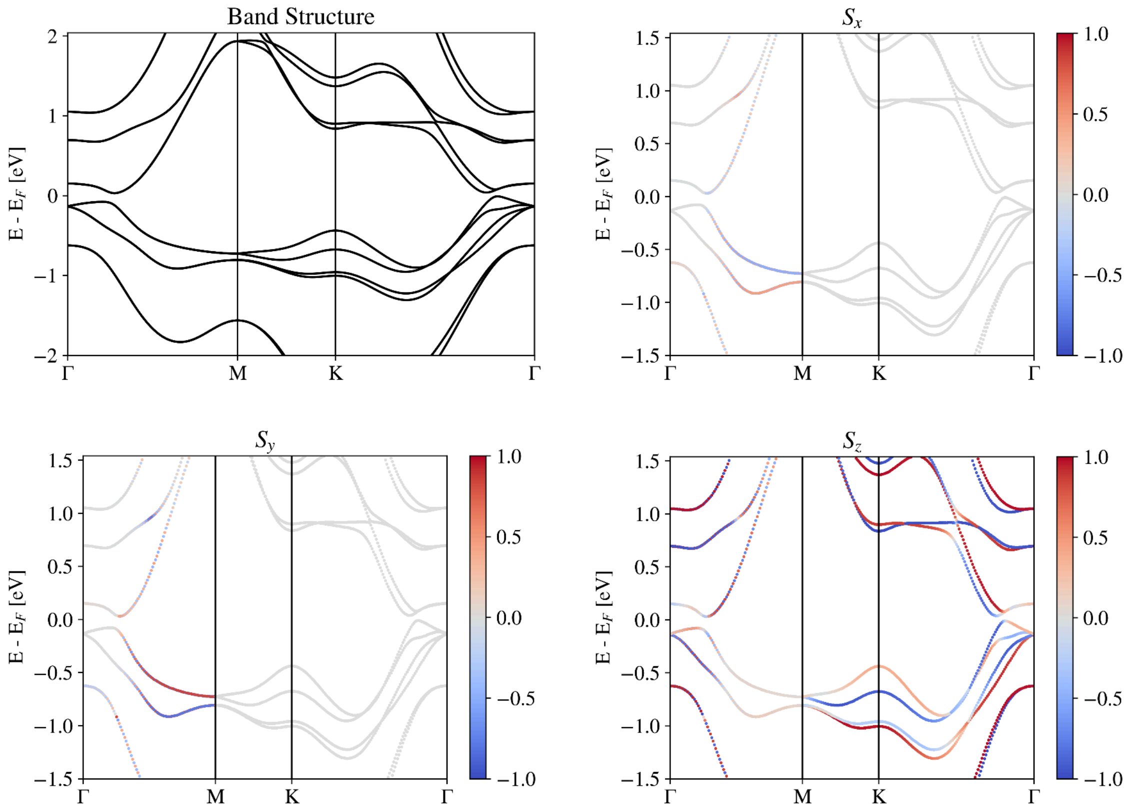


Figure 531: Band structure and spin polarization projections.

## 2.266 OPb-2a393480e273

- **Formula**: OPb
- **Structural Cluster**: AB-25
- **Band gap (PBE)**: 1.806 eV
- **Energy above convex hull (C2DB)**: 0.315 eV
- **Space group symbol**: $P3m1$
- **Space group number**: 156
- **Polar structure**: True

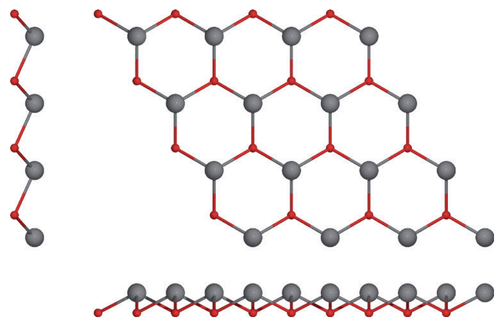

Figure 532: Structure representation

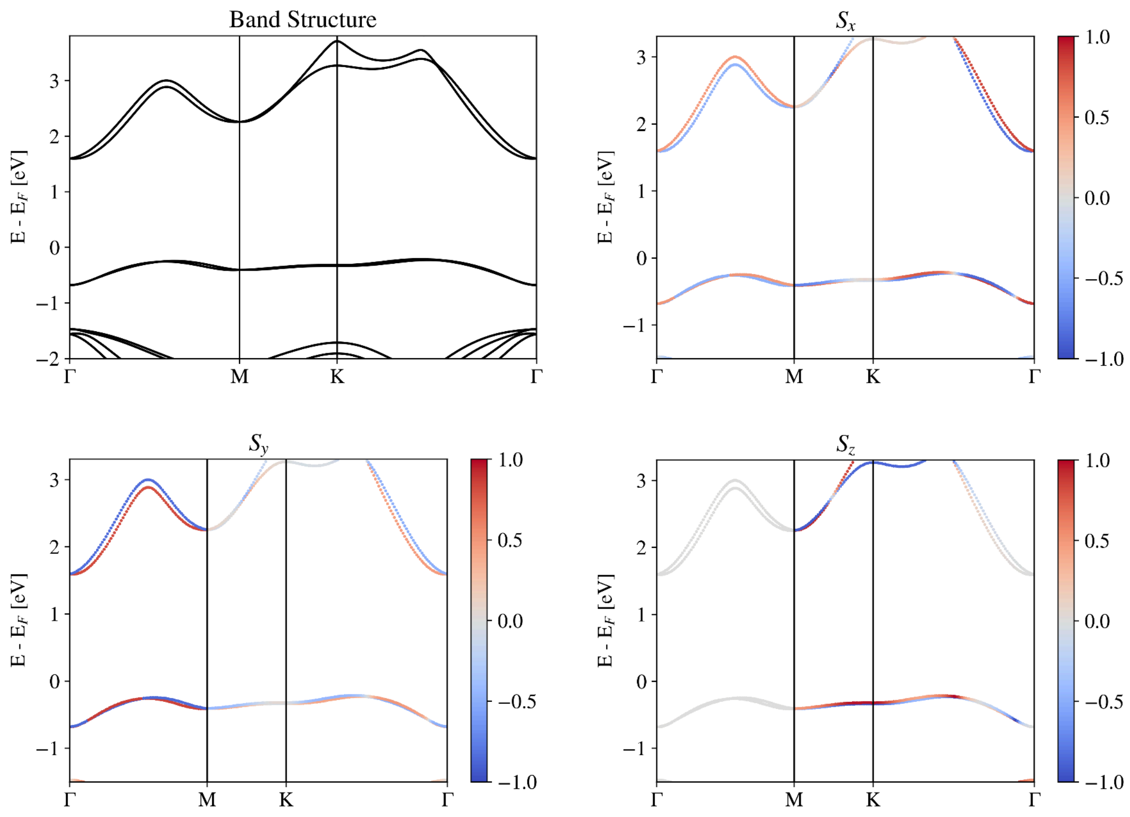


Figure 533: Band structure and spin polarization projections.

## 2.267 OSn-026ebfd86b48

- **Formula**: OSn
- **Structural Cluster**: AB-25
- **Band gap (PBE)**: 1.682 eV
- **Energy above convex hull (C2DB)**: 0.329 eV
- **Space group symbol**: $P3m1$
- **Space group number**: 156
- **Polar structure**: True

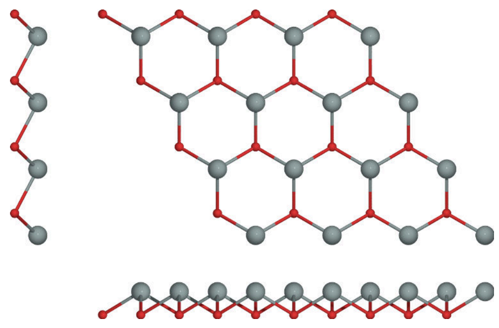

Figure 534: Structure representation

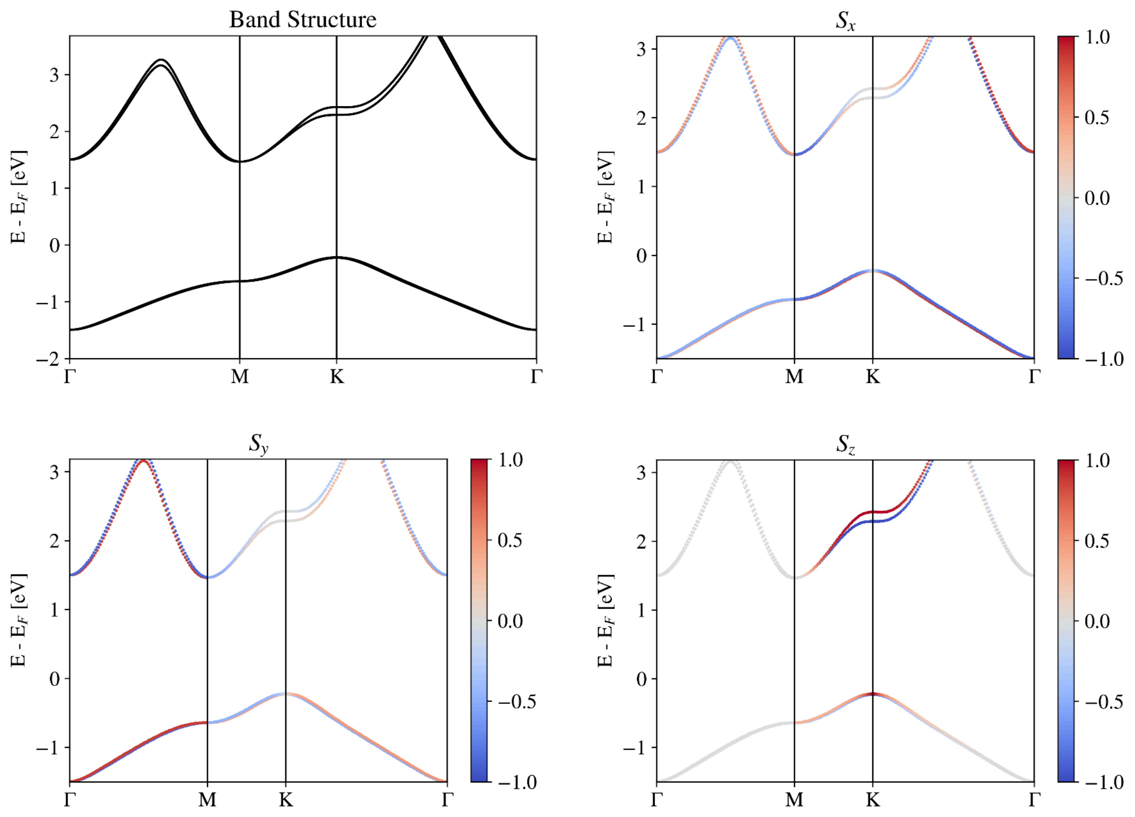


Figure 535: Band structure and spin polarization projections.

## 2.268 OsBr2-bf30e1249164

- **Formula**: OsBr2
- **Structural Cluster**: AB2-12
- **Band gap (PBE)**: 0.092 eV
- **Energy above convex hull (C2DB)**: 0.677 eV
- **Space group symbol**: $P\bar{4}m2$
- **Space group number**: 115
- **Polar structure**: False

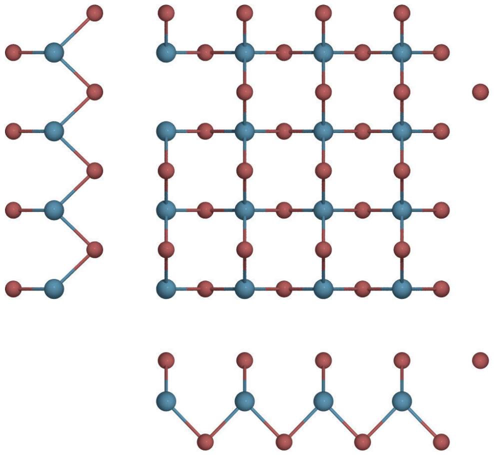

Figure 536: Structure representation

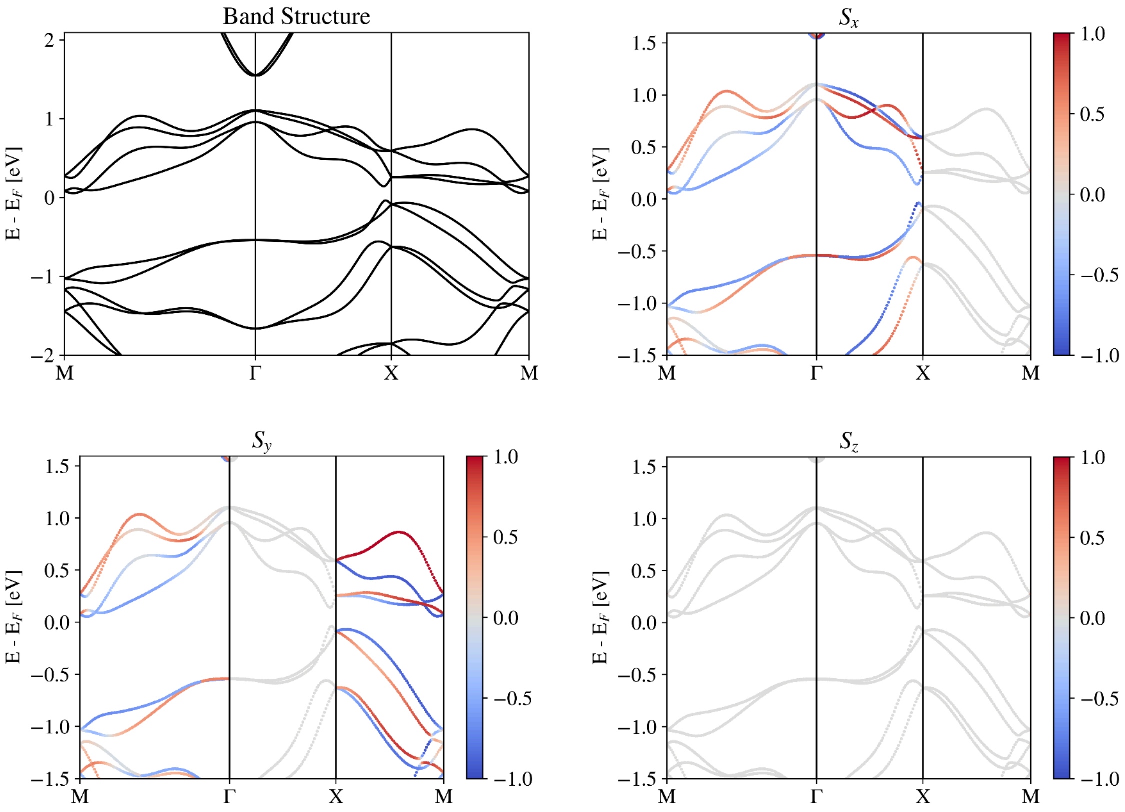


Figure 537: Band structure and spin polarization projections.

## 2.269 OsCl2-d37ba63794ad

- **Formula**: OsCl2
- **Structural Cluster**: AB2-12
- **Band gap (PBE)**: 0.308 eV
- **Energy above convex hull (C2DB)**: 0.658 eV
- **Space group symbol**: $P\bar{4}m2$
- **Space group number**: 115
- **Polar structure**: False

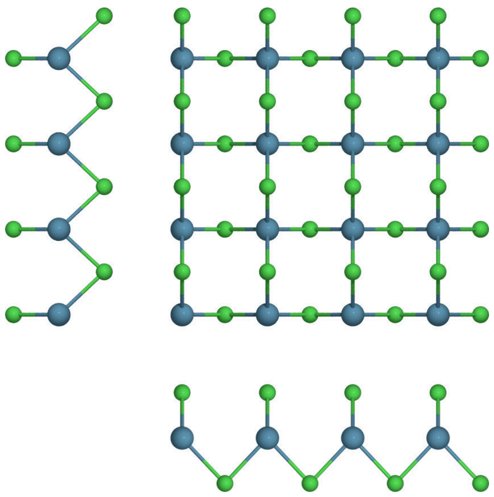

Figure 538: Structure representation

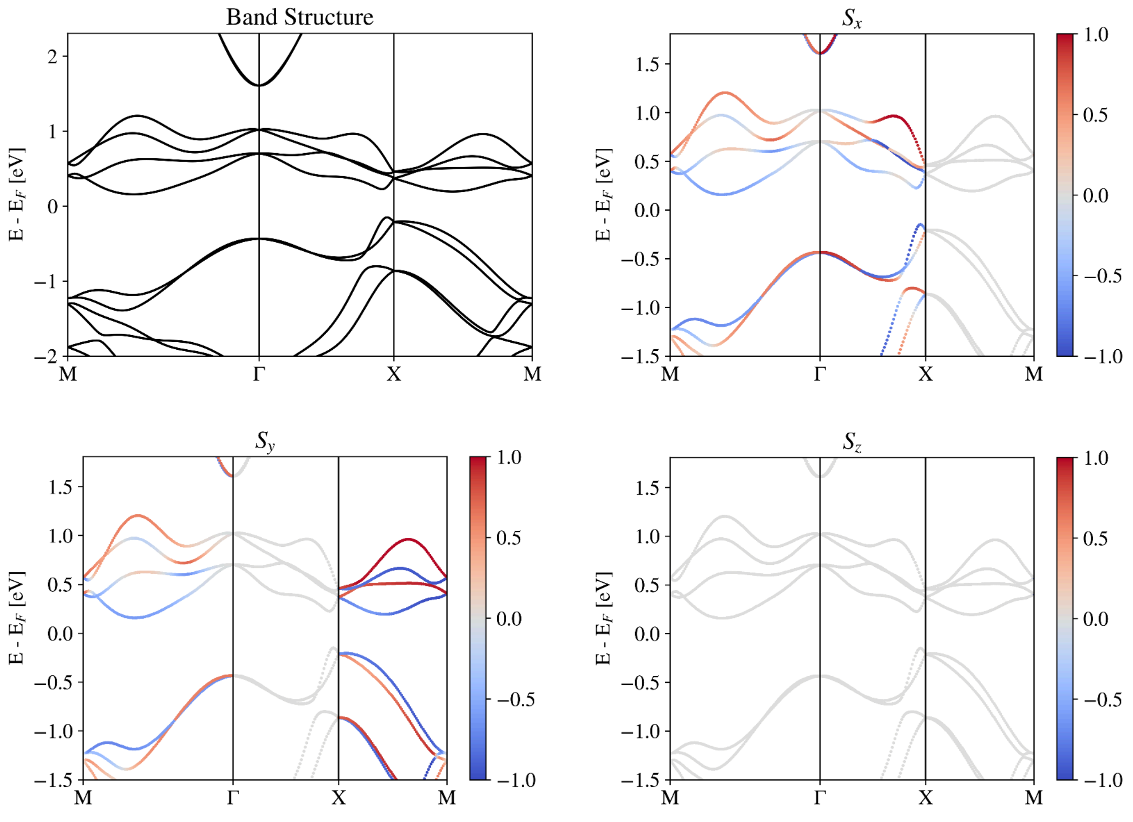


Figure 539: Band structure and spin polarization projections.

## 2.270 P2Pb2S6-7cf77f91eef0

- **Formula**: P2Pb2S6
- **Structural Cluster**: ABC3-16
- **Band gap (PBE)**: 1.477 eV
- **Energy above convex hull (C2DB)**: 0.0 eV
- **Space group symbol**: *P*1
- **Space group number**: 1
- **Polar structure**: True

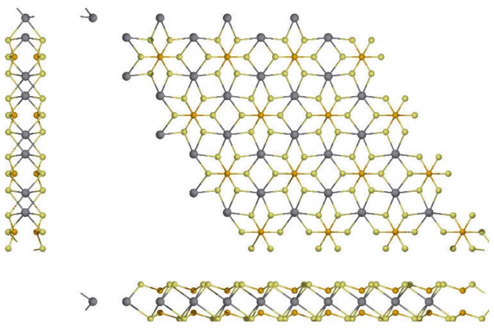

Figure 540: Structure representation

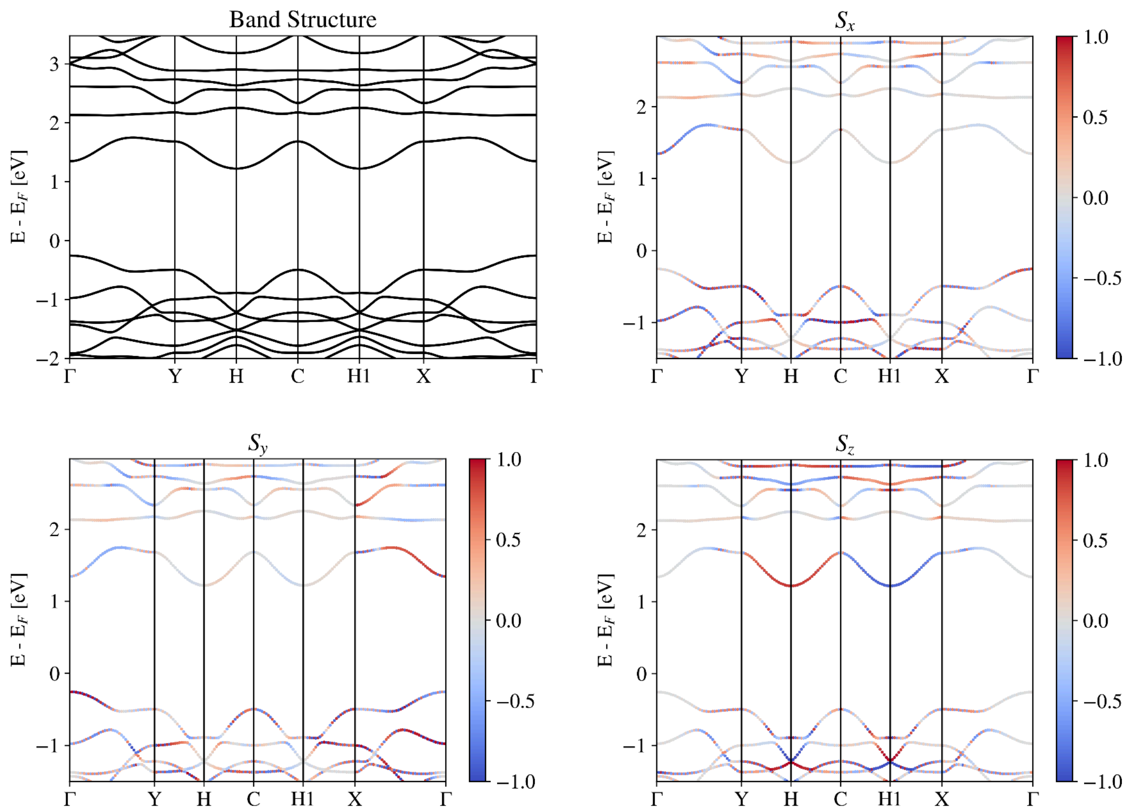


Figure 541: Band structure and spin polarization projections.

## 2.271 P2Pb2Se6-1811d52ac455

- **Formula**: P2Pb2Se6
- **Structural Cluster**: ABC3-16
- **Band gap (PBE)**: 1.052 eV
- **Energy above convex hull (C2DB)**: 0.0 eV
- **Space group symbol**: $P1$
- **Space group number**: 1
- **Polar structure**: True

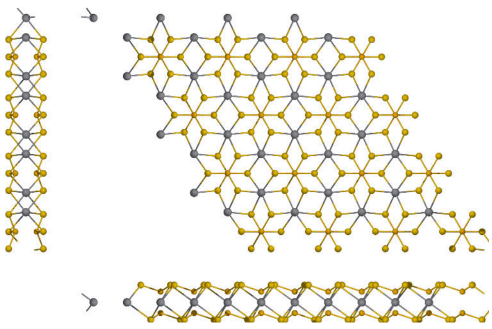

Figure 542: Structure representation

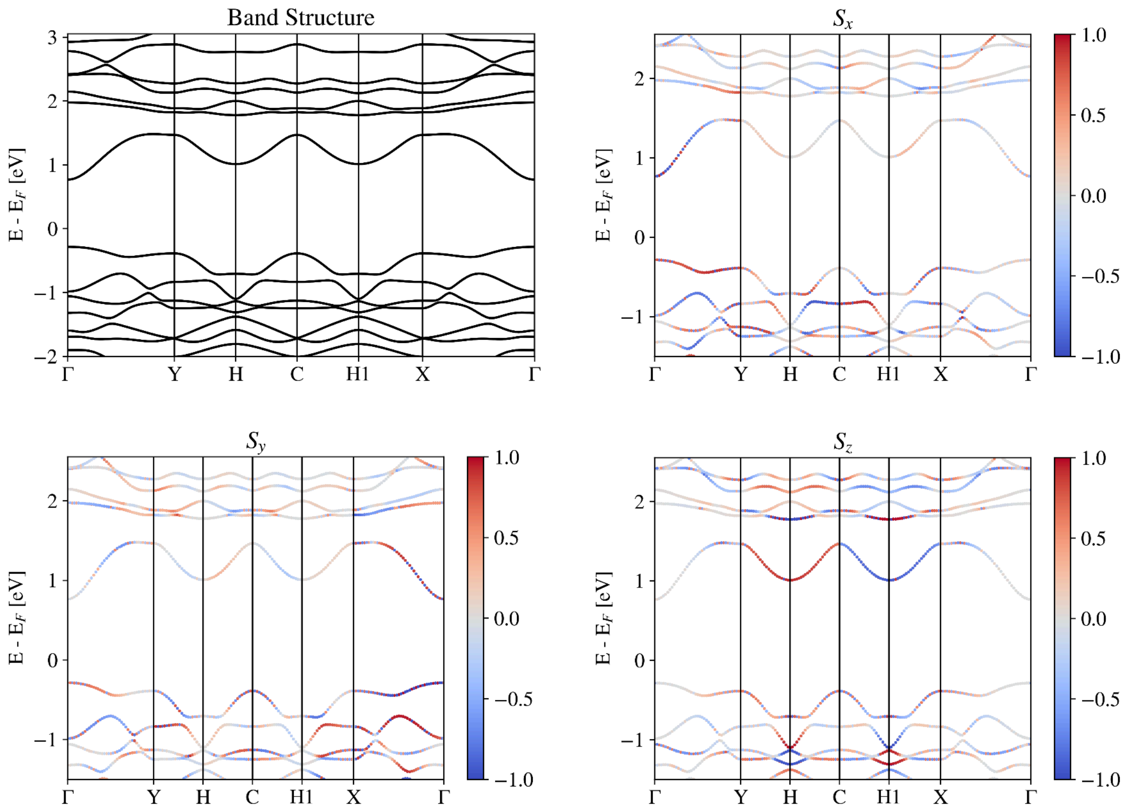


Figure 543: Band structure and spin polarization projections.

## 2.272 P2Pb2Te6-f0b8906970f5

- **Formula**: P2Pb2Te6
- **Structural Cluster**: ABC3-16
- **Band gap (PBE)**: 0.75 eV
- **Energy above convex hull (C2DB)**: 0.102 eV
- **Space group symbol**: $P1$
- **Space group number**: 1
- **Polar structure**: True

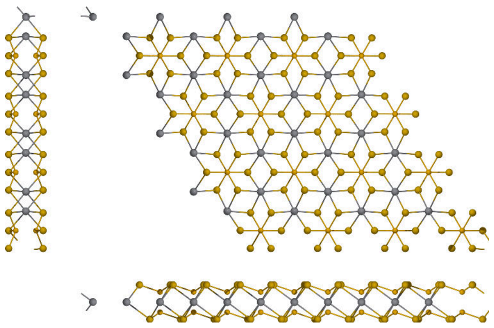

Figure 544: Structure representation

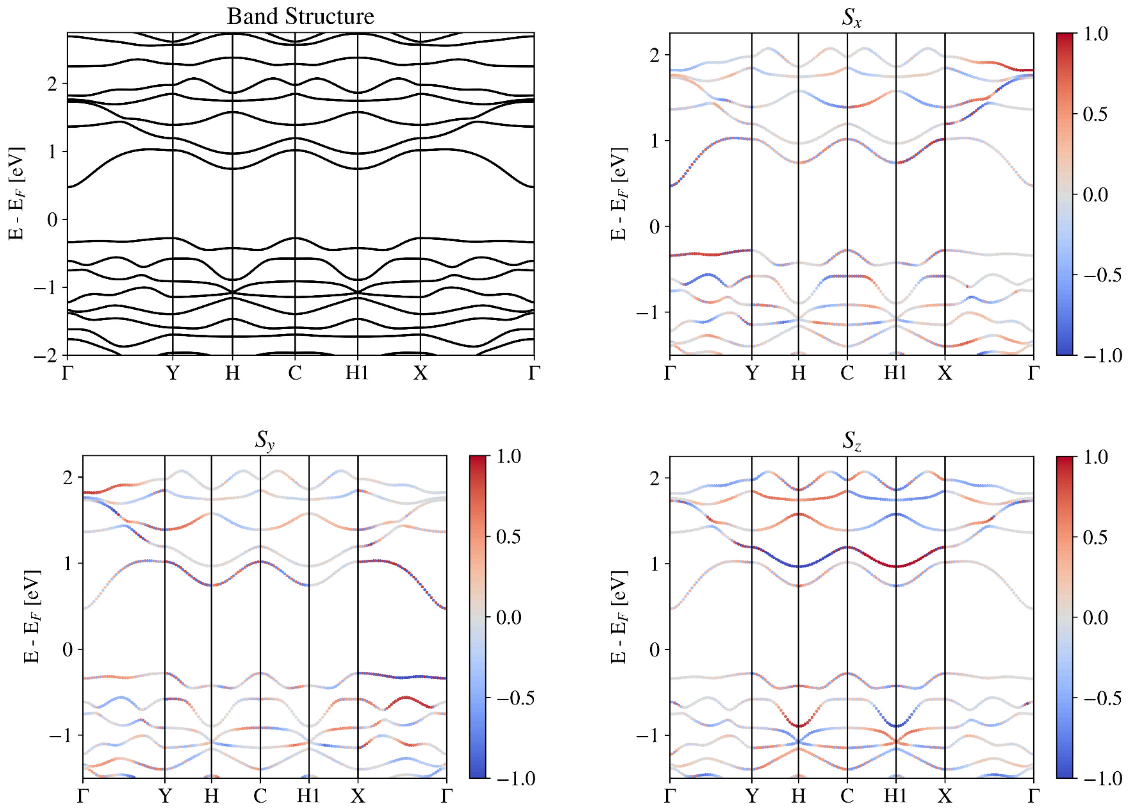


Figure 545: Band structure and spin polarization projections.

## 2.273 P2Pt2Se6-3a65ac9ff395

- **Formula**: P2Pt2Se6
- **Structural Cluster**: ABC3-16
- **Band gap (PBE)**: 0.462 eV
- **Energy above convex hull (C2DB)**: 0.191 eV
- **Space group symbol**: $P1$
- **Space group number**: 1
- **Polar structure**: True

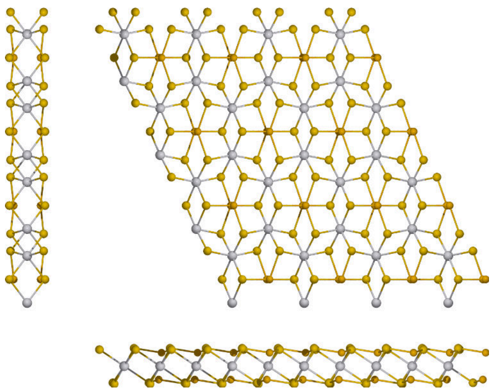

Figure 546: Structure representation

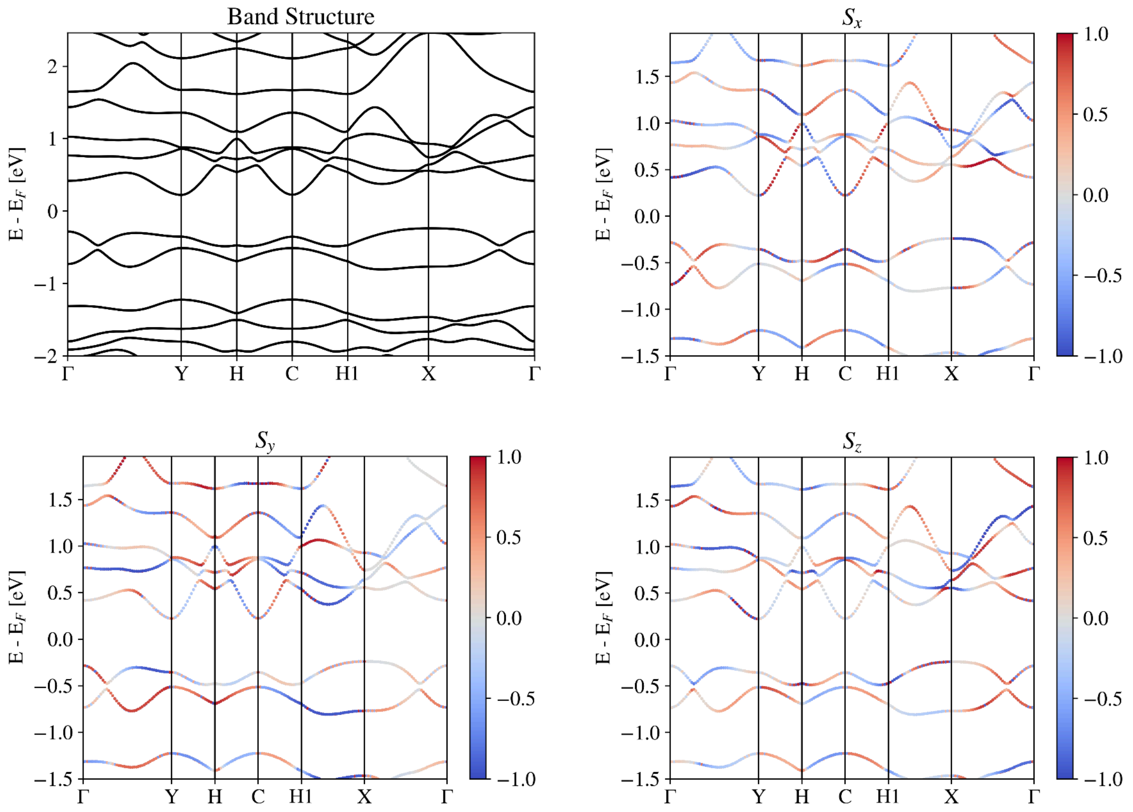


Figure 547: Band structure and spin polarization projections.

## 2.274 P2Rh2Se6-c10e77a99b6c

- **Formula**: P2Rh2Se6
- **Structural Cluster**: ABC3-16
- **Band gap (PBE)**: 0.012 eV
- **Energy above convex hull (C2DB)**: 0.082 eV
- **Space group symbol**: *Cm*
- **Space group number**: 8
- **Polar structure**: True

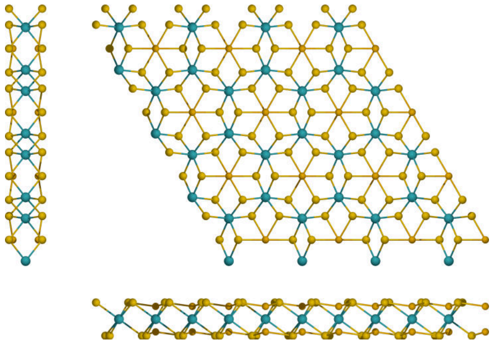

Figure 548: Structure representation

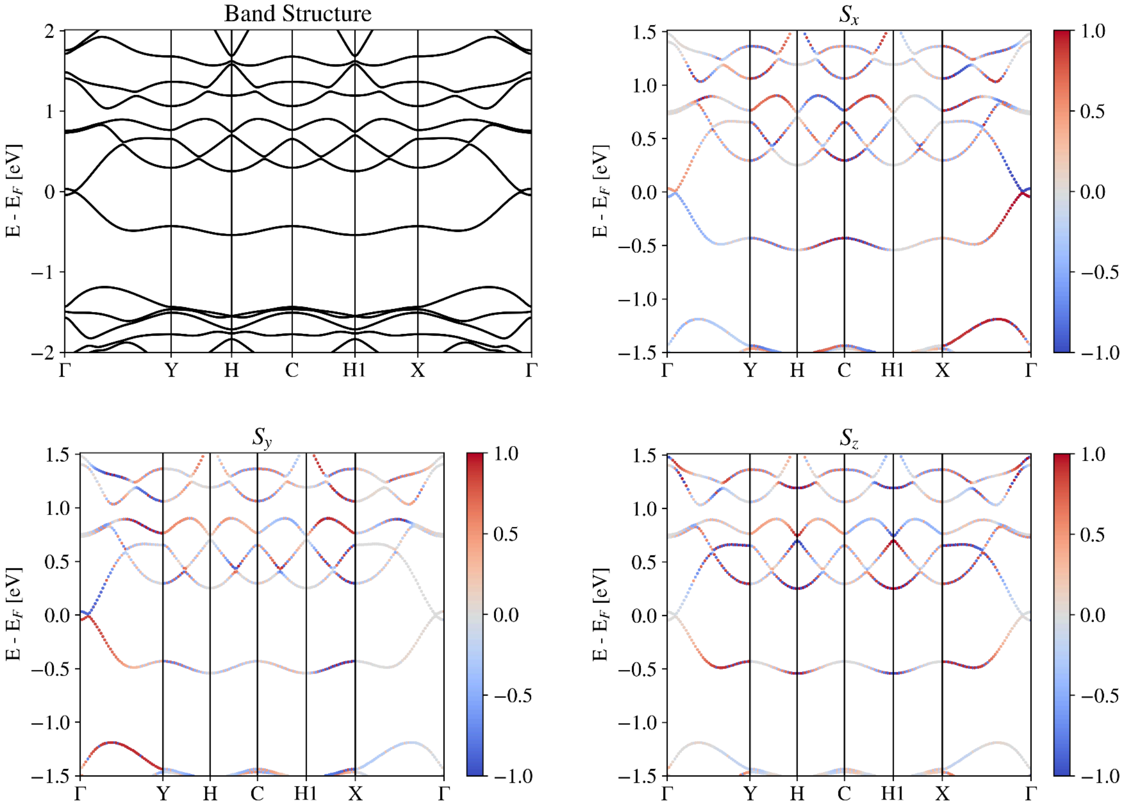


Figure 549: Band structure and spin polarization projections.

## 2.275 P2Rh2Te6-0f1f68e6fa79

- **Formula**: P2Rh2Te6
- **Structural Cluster**: ABC3-16
- **Band gap (PBE)**: 0.109 eV
- **Energy above convex hull (C2DB)**: 0.139 eV
- **Space group symbol**: *Cm*
- **Space group number**: 8
- **Polar structure**: True

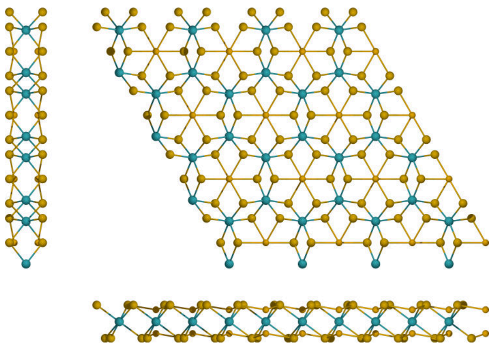

Figure 550: Structure representation

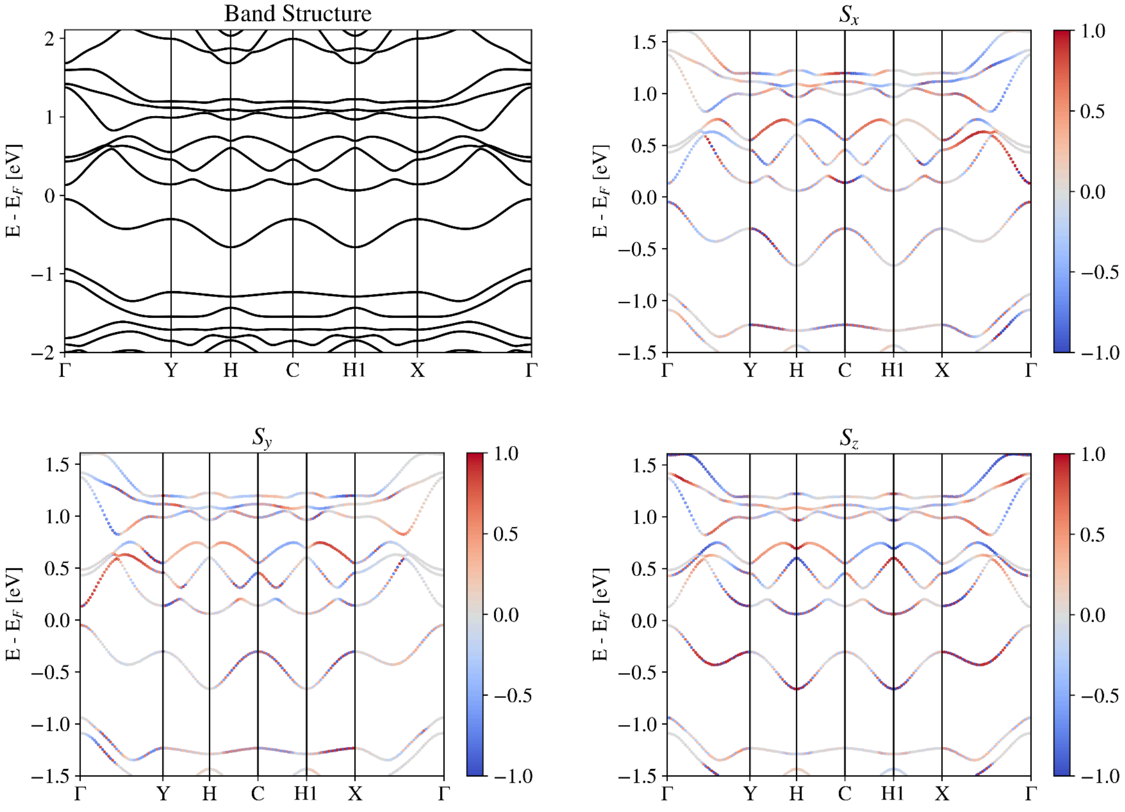


Figure 551: Band structure and spin polarization projections.

## 2.276 P2Ru2S6-9caed1a0620c

- **Formula**: P2Ru2S6
- **Structural Cluster**: ABC3-16
- **Band gap (PBE)**: 0.322 eV
- **Energy above convex hull (C2DB)**: 0.179 eV
- **Space group symbol**: $P1$
- **Space group number**: 1
- **Polar structure**: True

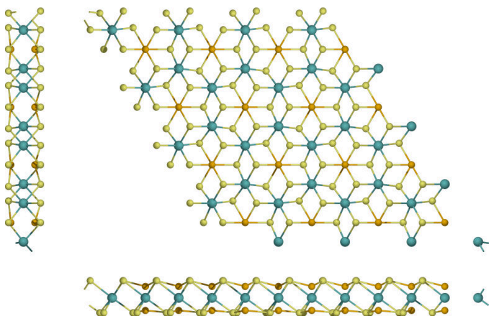

Figure 552: Structure representation

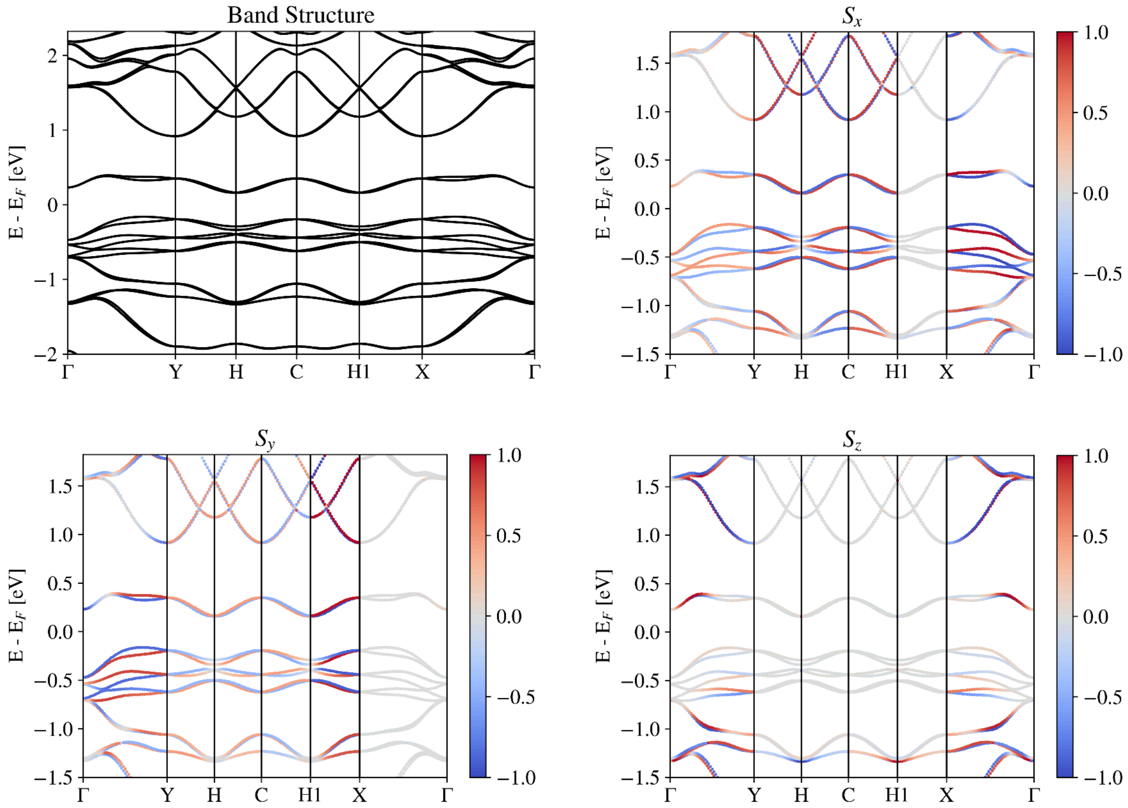


Figure 553: Band structure and spin polarization projections.

## 2.277 P2Ru2Te6-dc750358a911

- **Formula**: P2Ru2Te6
- **Structural Cluster**: ABC3-16
- **Band gap (PBE)**: 0.184 eV
- **Energy above convex hull (C2DB)**: 0.209 eV
- **Space group symbol**: *P*3
- **Space group number**: 143
- **Polar structure**: True

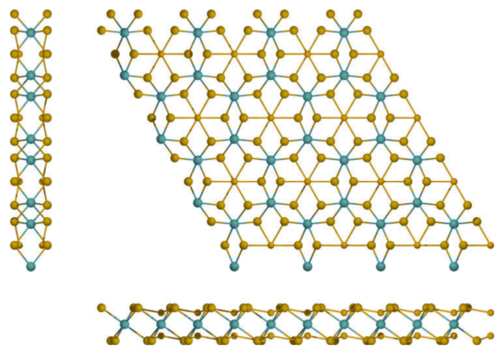

Figure 554: Structure representation

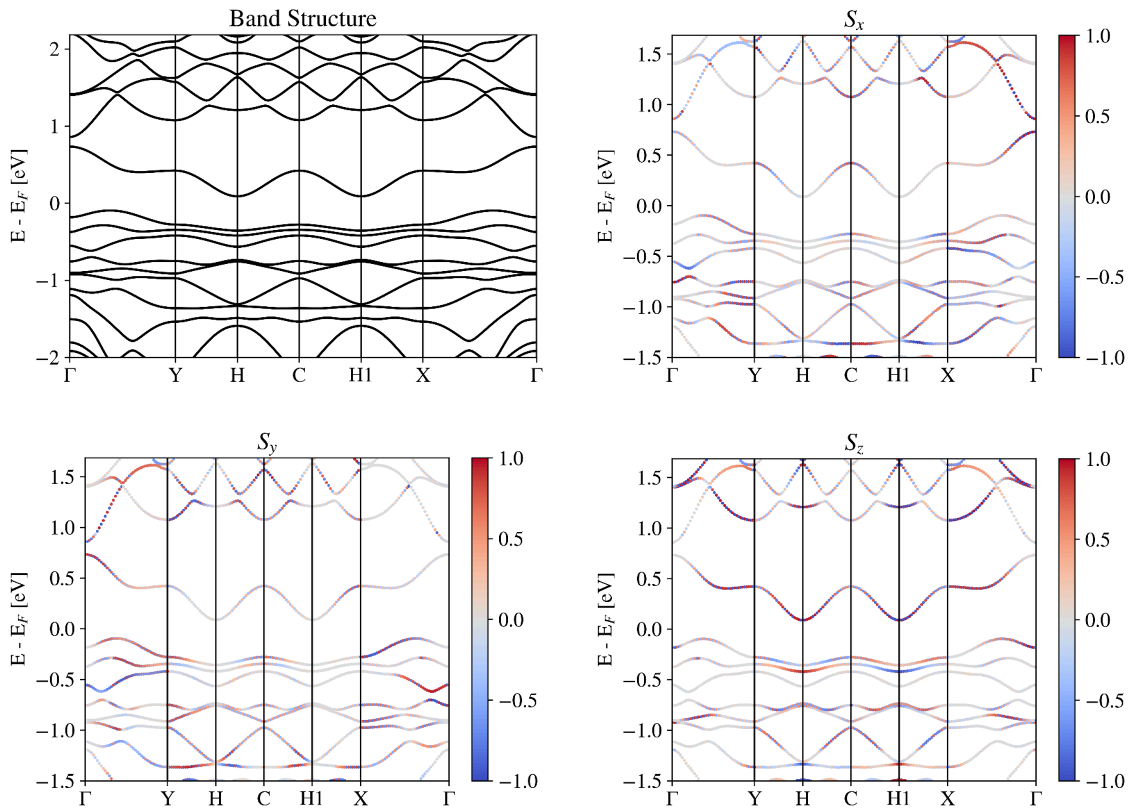


Figure 555: Band structure and spin polarization projections.

## 2.278 P2Sb2Se6-5d1a32a28ffa

- **Formula**: P2Sb2Se6
- **Structural Cluster**: ABC3-16
- **Band gap (PBE)**: 1.004 eV
- **Energy above convex hull (C2DB)**: 0.058 eV
- **Space group symbol**: $P1$
- **Space group number**: 1
- **Polar structure**: True

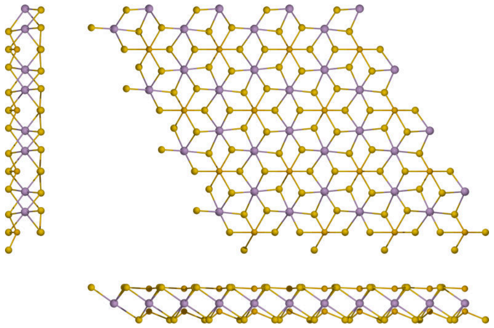

Figure 556: Structure representation

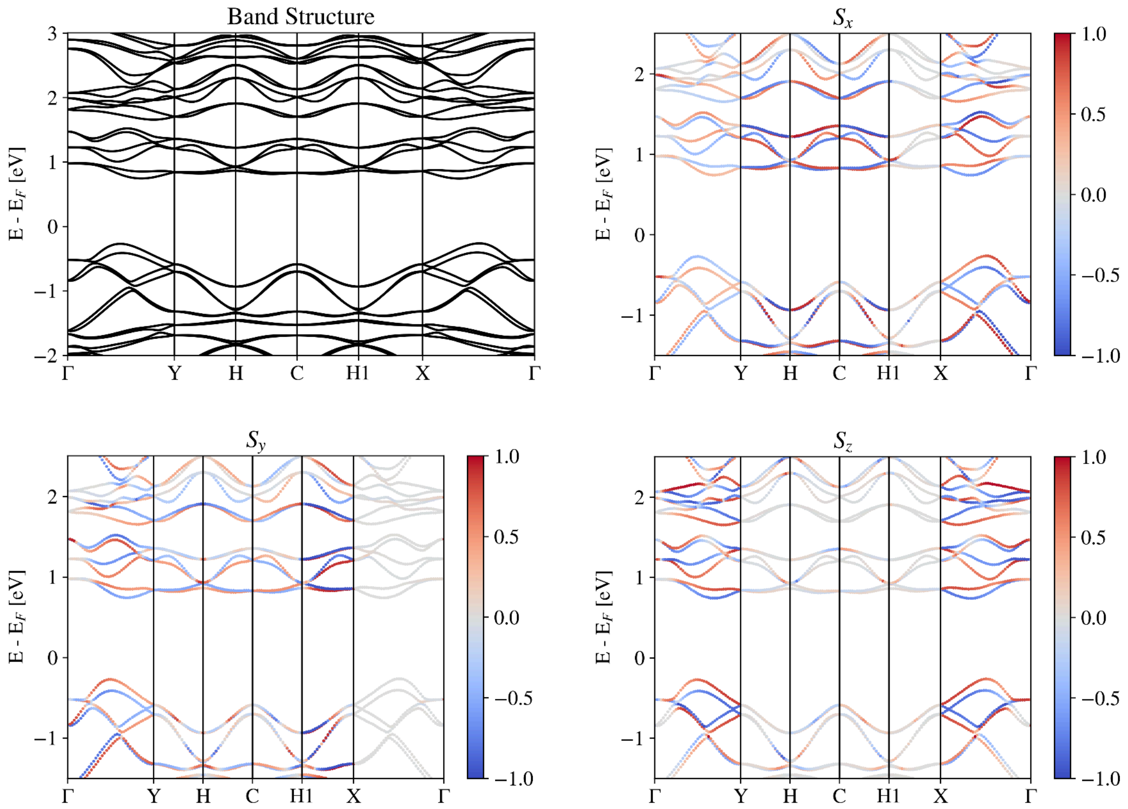


Figure 557: Band structure and spin polarization projections.

## 2.279 P2Sb2Te6-82b85dfd7723

- **Formula**: P2Sb2Te6
- **Structural Cluster**: ABC3-16
- **Band gap (PBE)**: 0.633 eV
- **Energy above convex hull (C2DB)**: 0.14 eV
- **Space group symbol**: $P1$
- **Space group number**: 1
- **Polar structure**: True

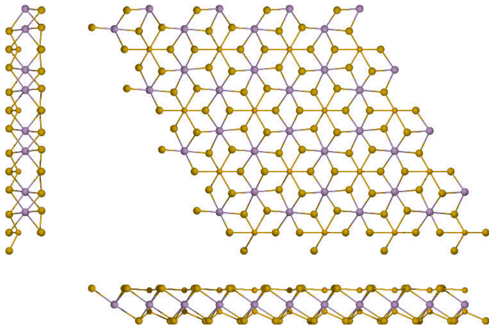

Figure 558: Structure representation

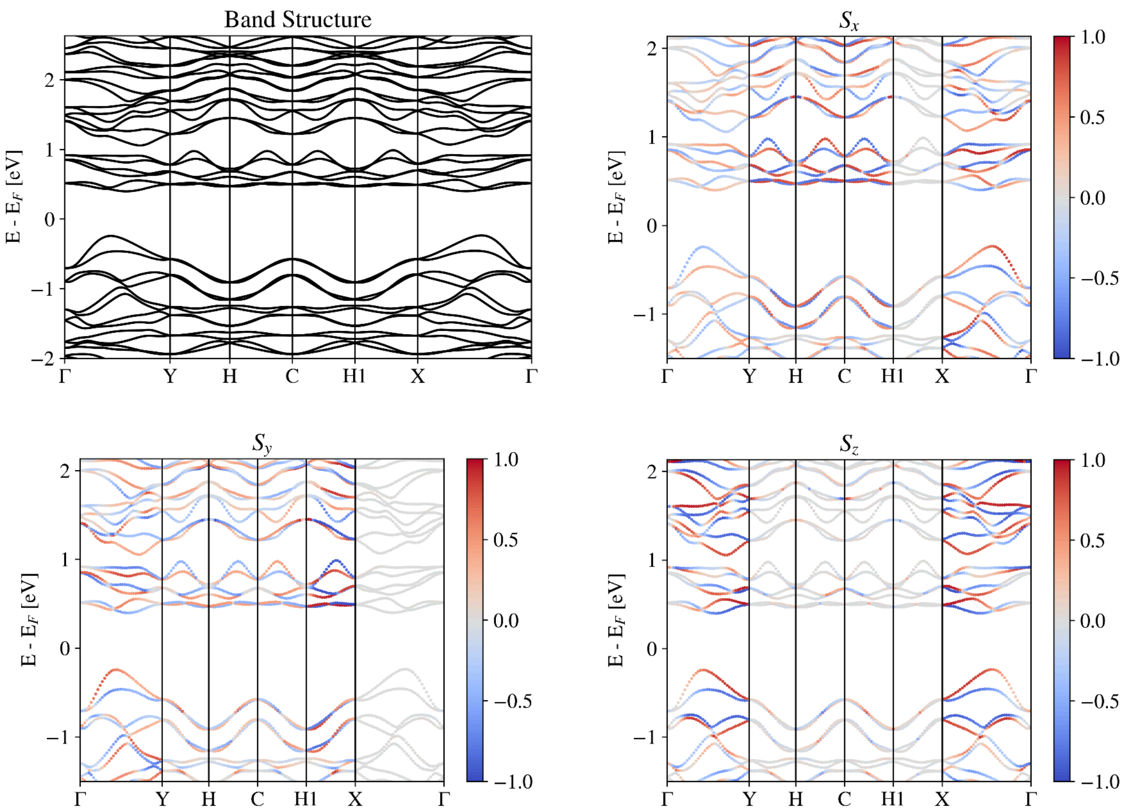


Figure 559: Band structure and spin polarization projections.

## 2.280 P2Sc2S6-bc8b8c21ad4f

- **Formula**: P2Sc2S6
- **Structural Cluster**: ABC3-16
- **Band gap (PBE)**: 0.829 eV
- **Energy above convex hull (C2DB)**: 0.08 eV
- **Space group symbol**: *P*3
- **Space group number**: 143
- **Polar structure**: True

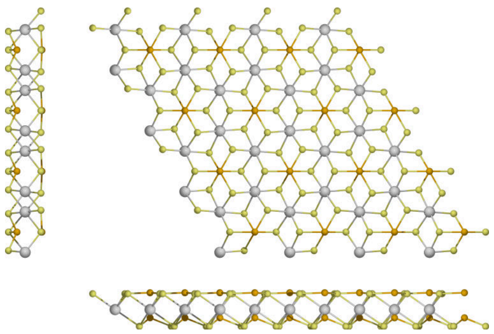

Figure 560: Structure representation

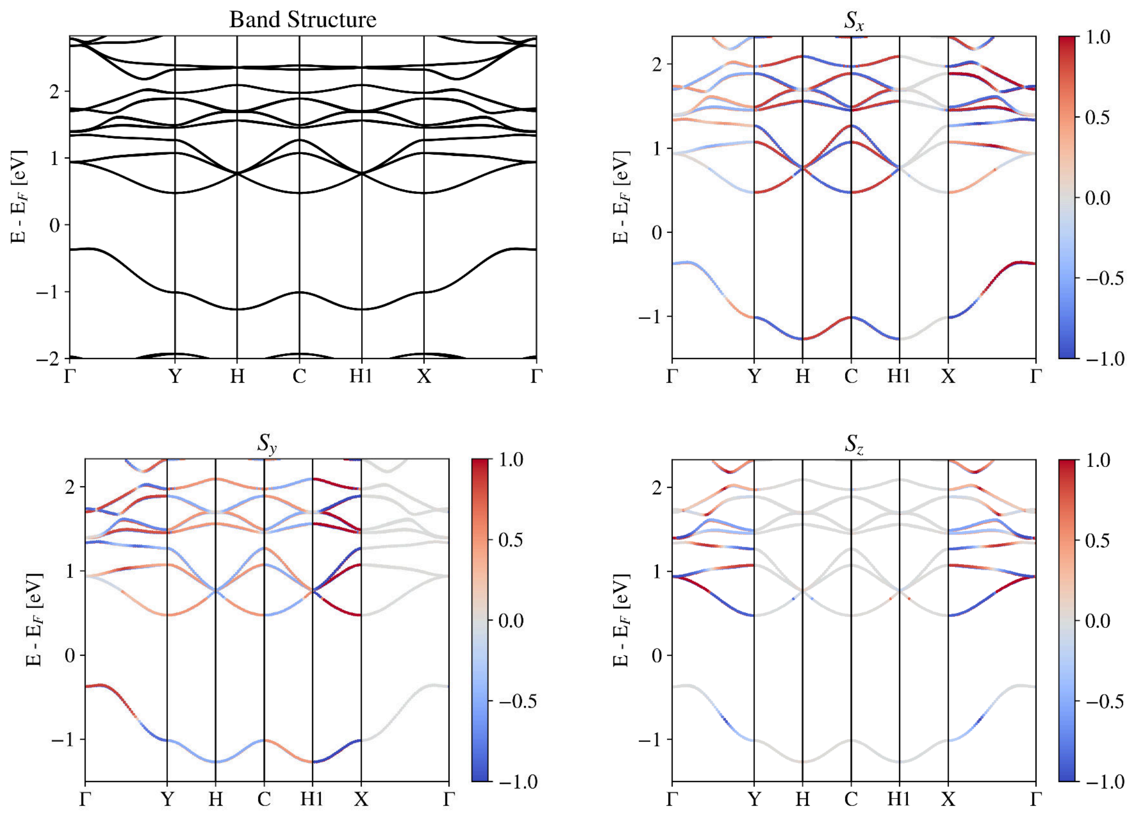


Figure 561: Band structure and spin polarization projections.

## 2.281 P2Sc2Se6-093920d00119

- **Formula**: P2Sc2Se6
- **Structural Cluster**: ABC3-16
- **Band gap (PBE)**: 0.68 eV
- **Energy above convex hull (C2DB)**: 0.0 eV
- **Space group symbol**: $P3$
- **Space group number**: 143
- **Polar structure**: True

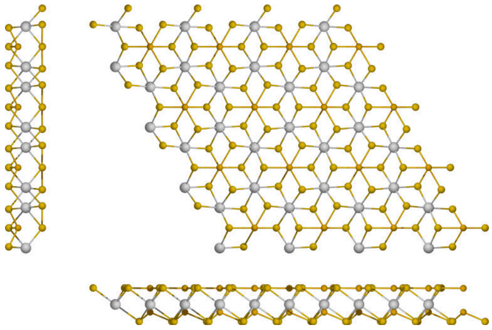

Figure 562: Structure representation

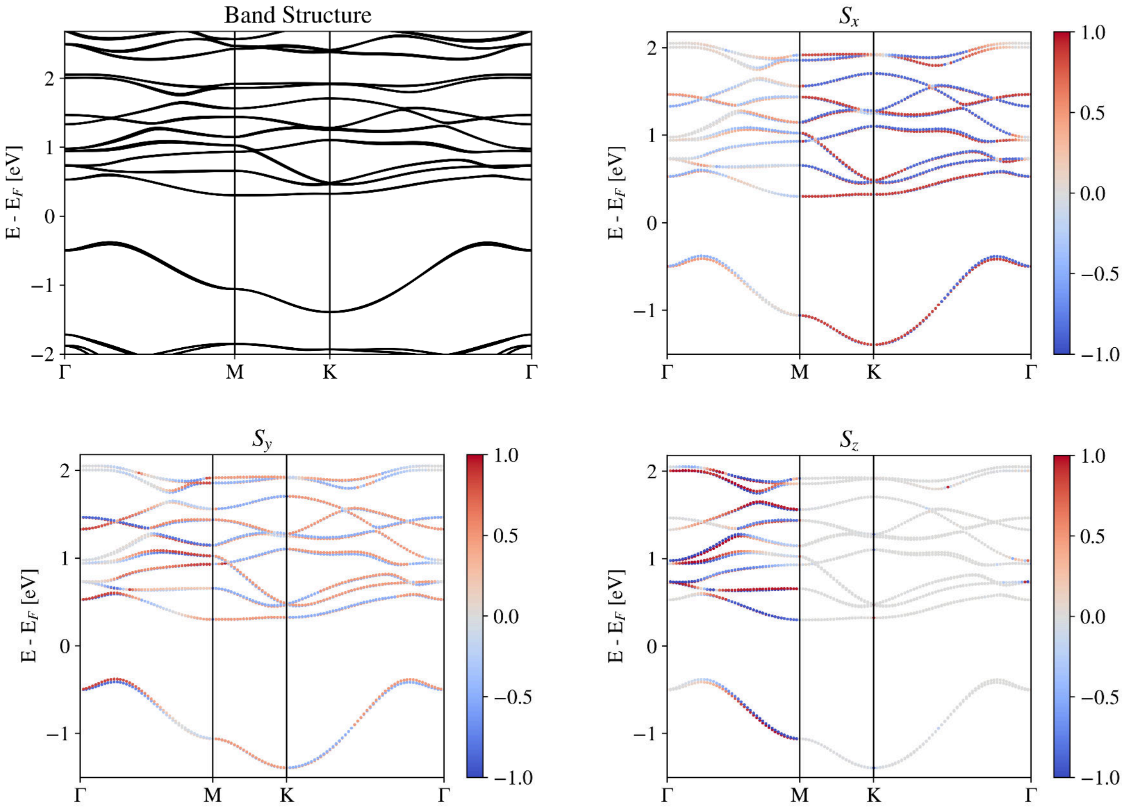


Figure 563: Band structure and spin polarization projections.

## 2.282 P2Sn2S6-aa34f4a1c451

- **Formula**: P2Sn2S6
- **Structural Cluster**: ABC3-16
- **Band gap (PBE)**: 1.06 eV
- **Energy above convex hull (C2DB)**: 0.0 eV
- **Space group symbol**: $P1$
- **Space group number**: 1
- **Polar structure**: True

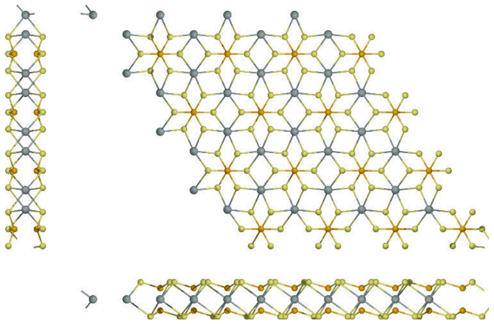

Figure 564: Structure representation

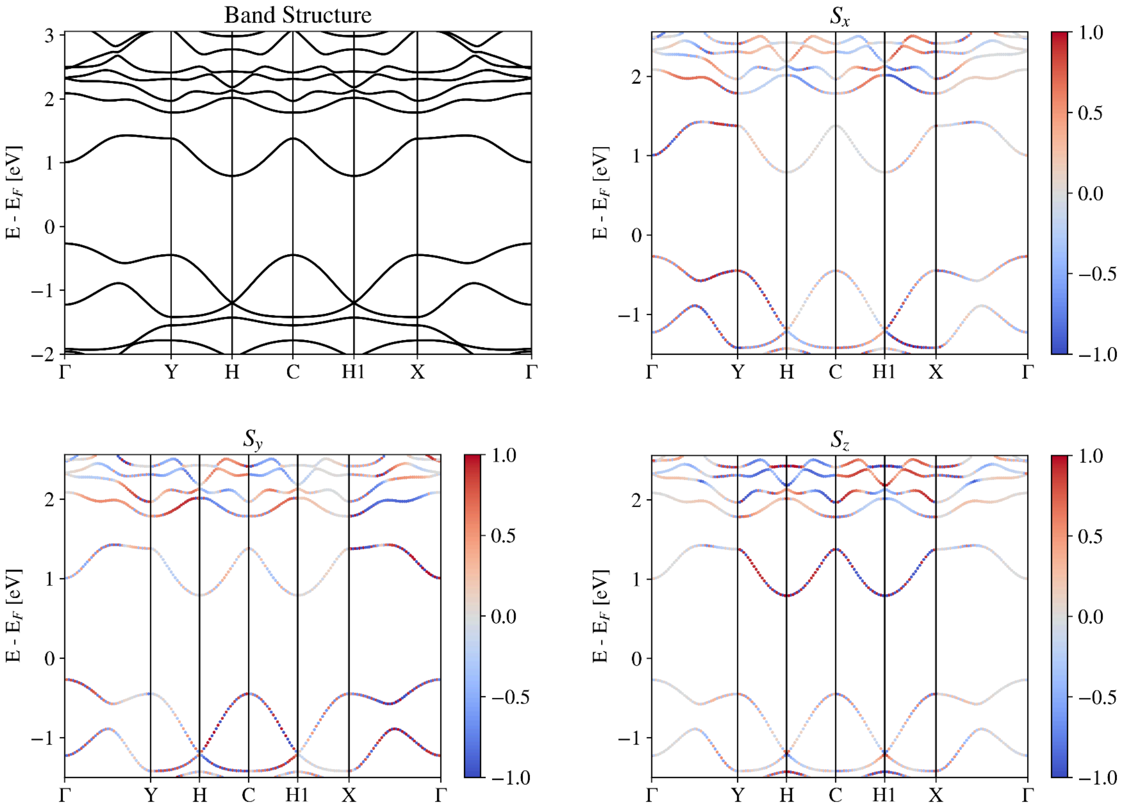


Figure 565: Band structure and spin polarization projections.

## 2.283 P2Sn2Se6-a056ab5346bf

- **Formula**: P2Sn2Se6
- **Structural Cluster**: ABC3-16
- **Band gap (PBE)**: 0.722 eV
- **Energy above convex hull (C2DB)**: 0.015 eV
- **Space group symbol**: $P1$
- **Space group number**: 1
- **Polar structure**: True

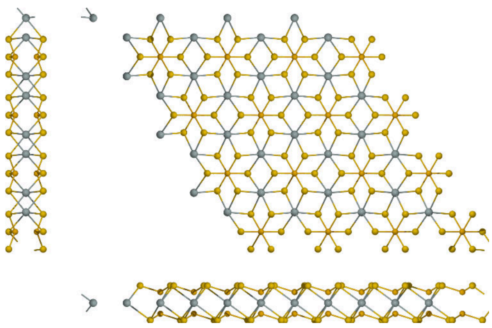

Figure 566: Structure representation

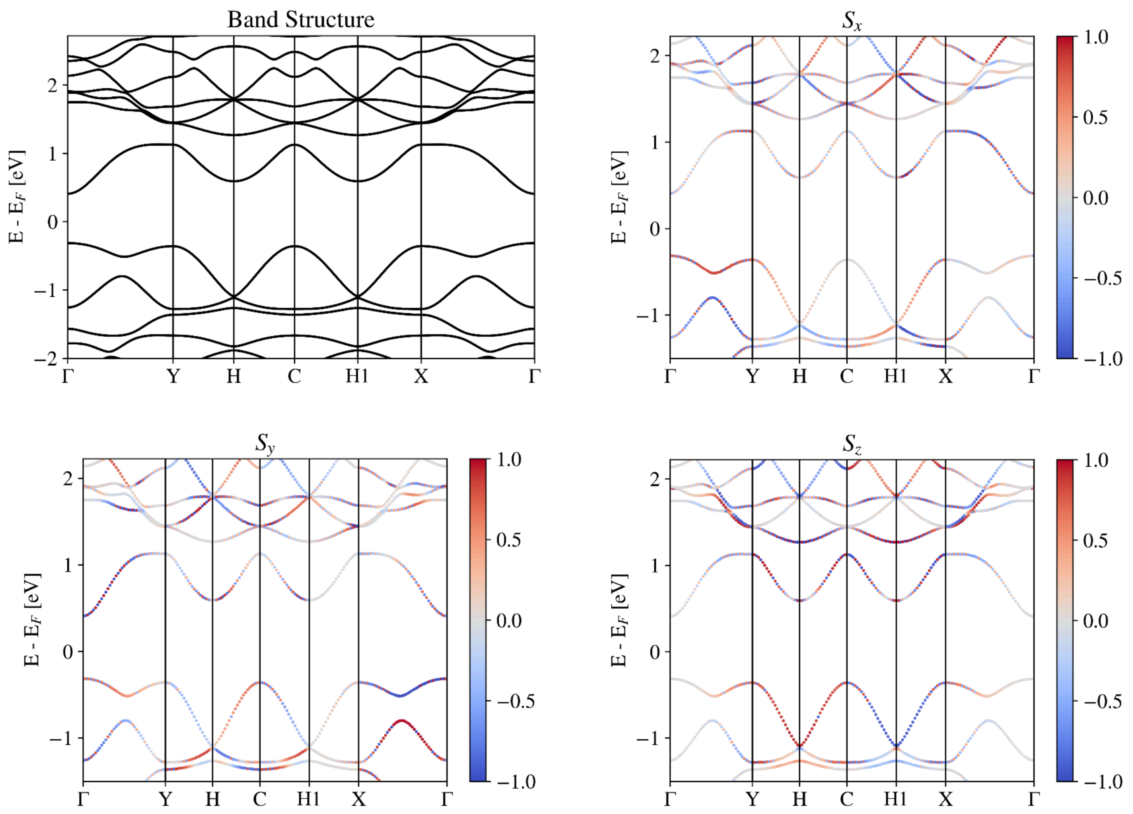


Figure 567: Band structure and spin polarization projections.

## 2.284 P2Sn2Te6-321533580298

- **Formula**: P2Sn2Te6
- **Structural Cluster**: ABC3-16
- **Band gap (PBE)**: 0.428 eV
- **Energy above convex hull (C2DB)**: 0.112 eV
- **Space group symbol**: $P1$
- **Space group number**: 1
- **Polar structure**: True

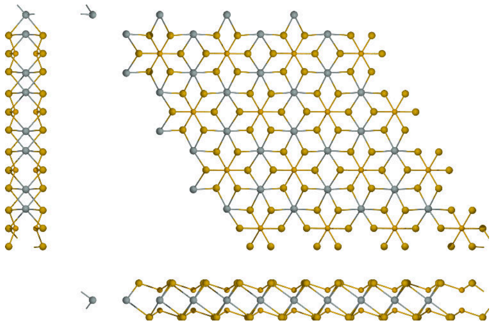

Figure 568: Structure representation

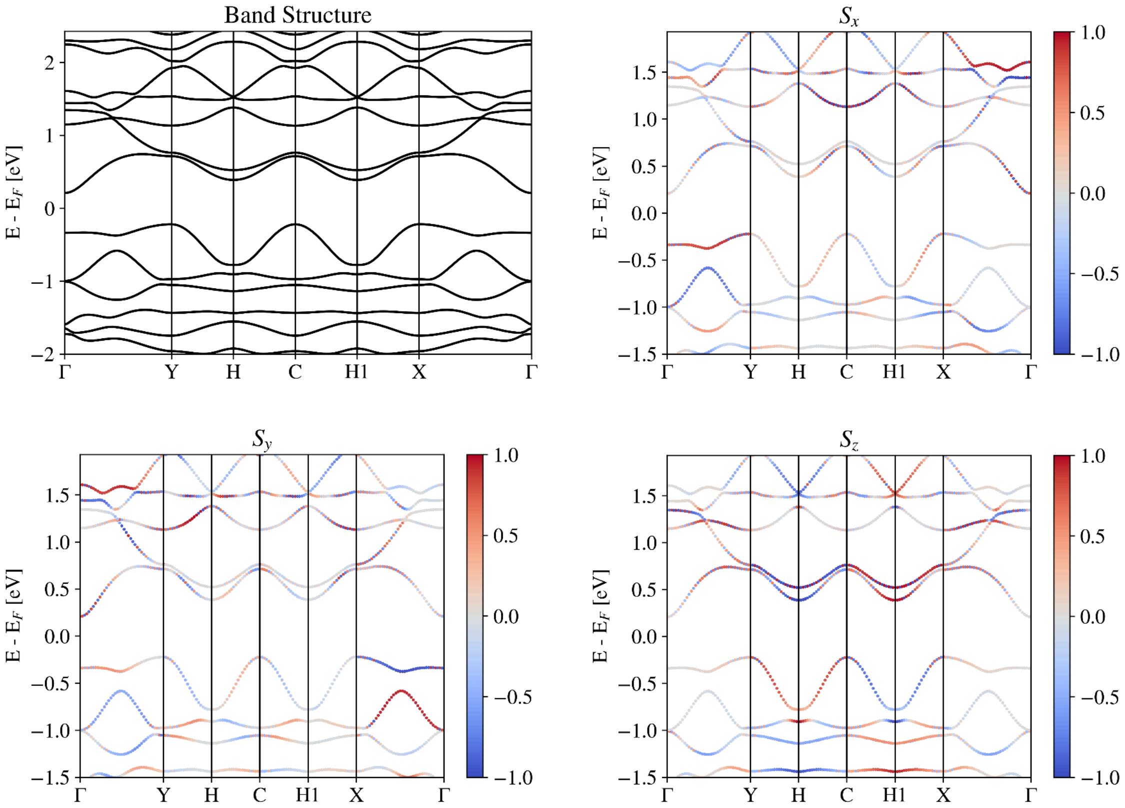


Figure 569: Band structure and spin polarization projections.

## 2.285 P2Ta2Se6-e2c90519357b

- **Formula**: P2Ta2Se6
- **Structural Cluster**: ABC3-16
- **Band gap (PBE)**: 0.29 eV
- **Energy above convex hull (C2DB)**: 0.224 eV
- **Space group symbol**: $P1$
- **Space group number**: 1
- **Polar structure**: True

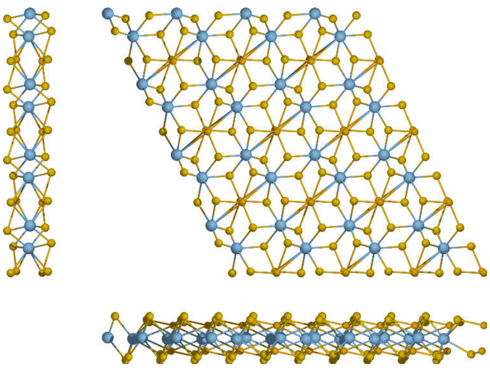

Figure 570: Structure representation

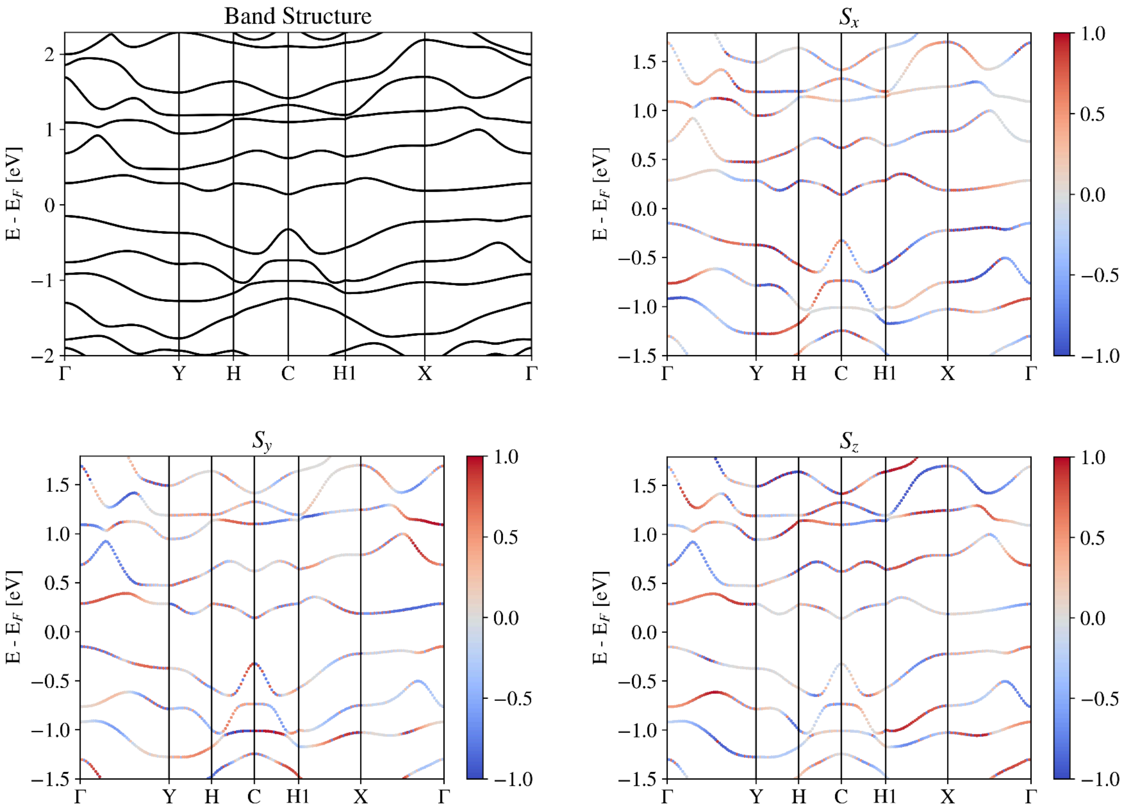


Figure 571: Band structure and spin polarization projections.

## 2.286 P2Ta2Te6-601435d29c97

- **Formula**: P2Ta2Te6
- **Structural Cluster**: ABC3-16
- **Band gap (PBE)**: 0.206 eV
- **Energy above convex hull (C2DB)**: 0.222 eV
- **Space group symbol**: $P1$
- **Space group number**: 1
- **Polar structure**: True

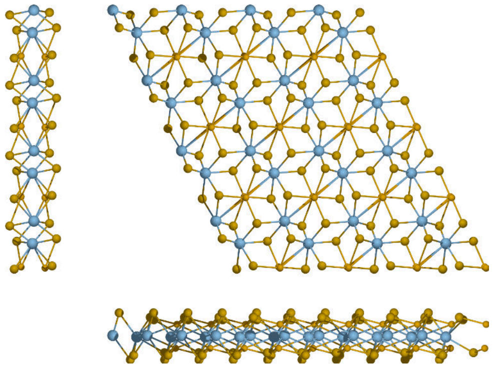

Figure 572: Structure representation

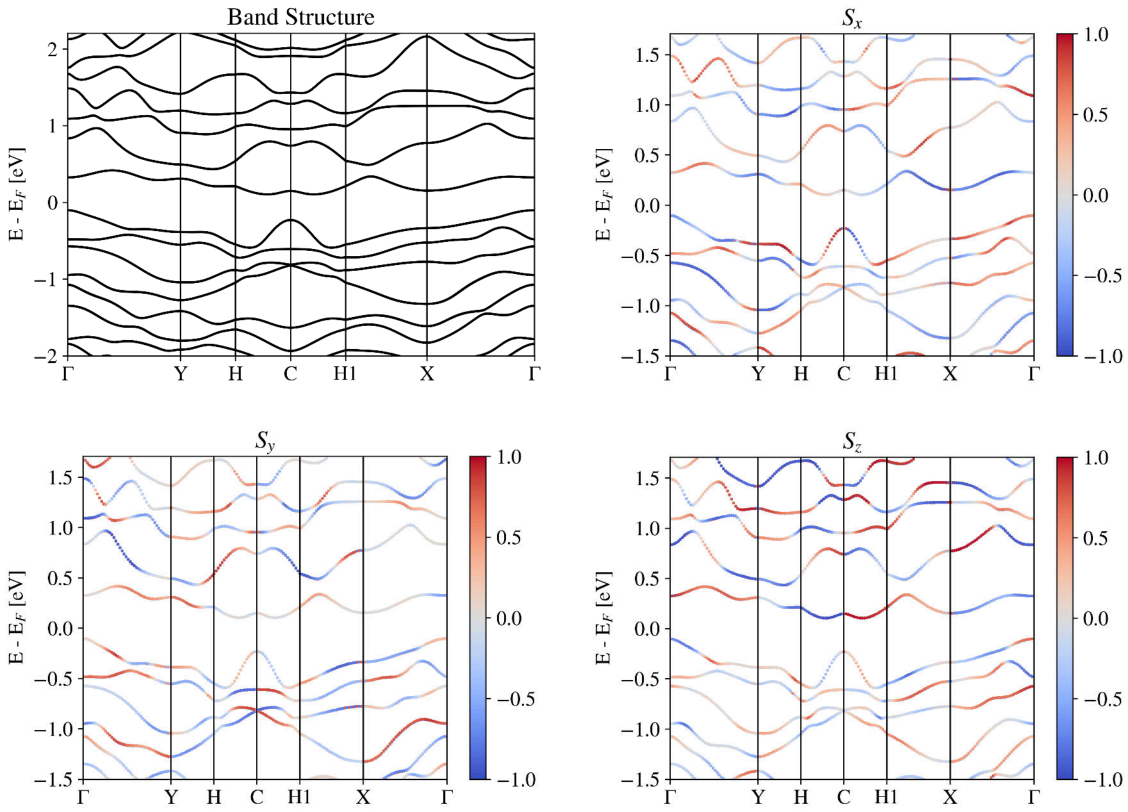


Figure 573: Band structure and spin polarization projections.

## 2.287 P2Zr2S6-f821141337d6

- **Formula**: P2Zr2S6
- **Structural Cluster**: ABC3-16
- **Band gap (PBE)**: 0.846 eV
- **Energy above convex hull (C2DB)**: 0.192 eV
- **Space group symbol**: $P1$
- **Space group number**: 1
- **Polar structure**: True

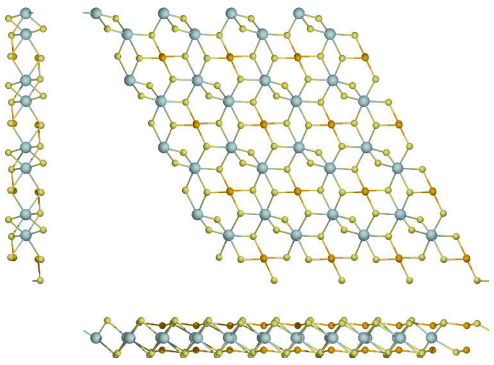

Figure 574: Structure representation

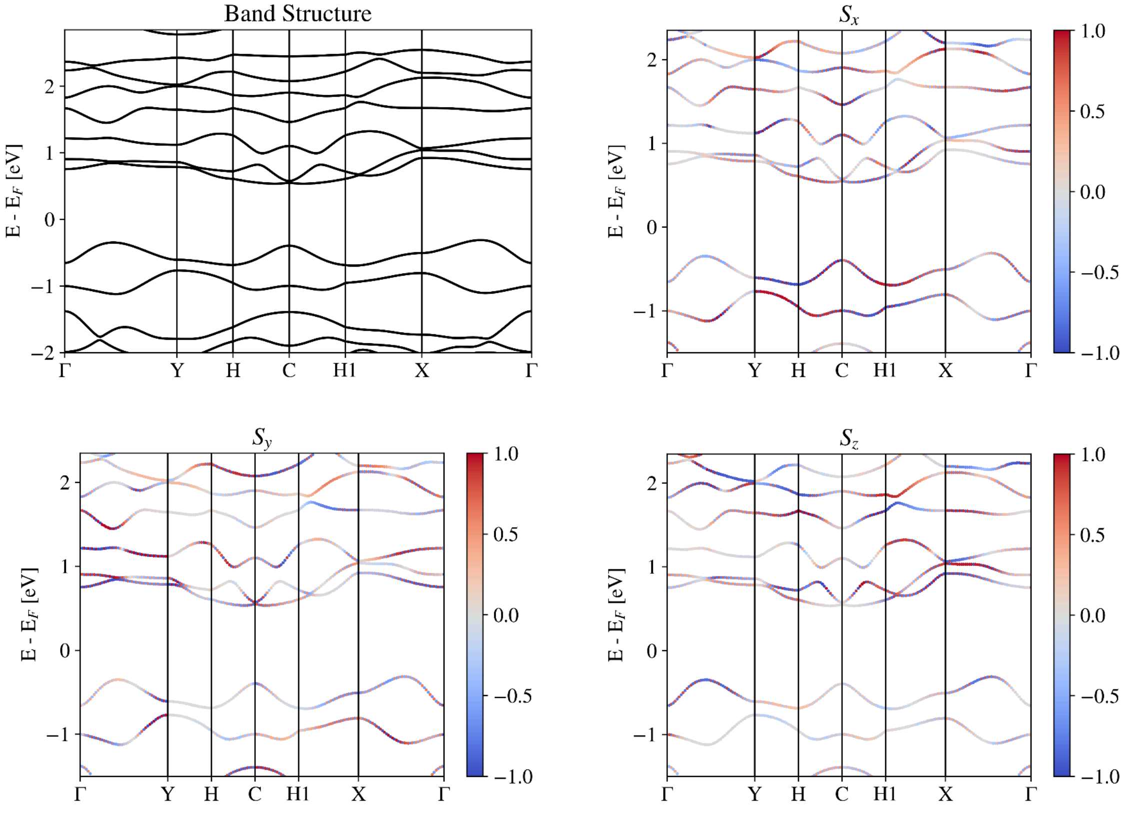


Figure 575: Band structure and spin polarization projections.

## 2.288 Pb2S2-d4ed2cd9ee0c

- **Formula**: Pb2S2
- **Structural Cluster**: AB-0
- **Band gap (PBE)**: 1.34 eV
- **Energy above convex hull (C2DB)**: 0.054 eV
- **Space group symbol**: $Pmn2_1$
- **Space group number**: 31
- **Polar structure**: True

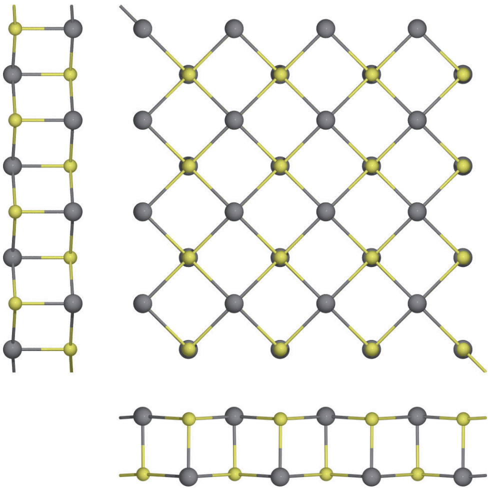

Figure 576: Structure representation

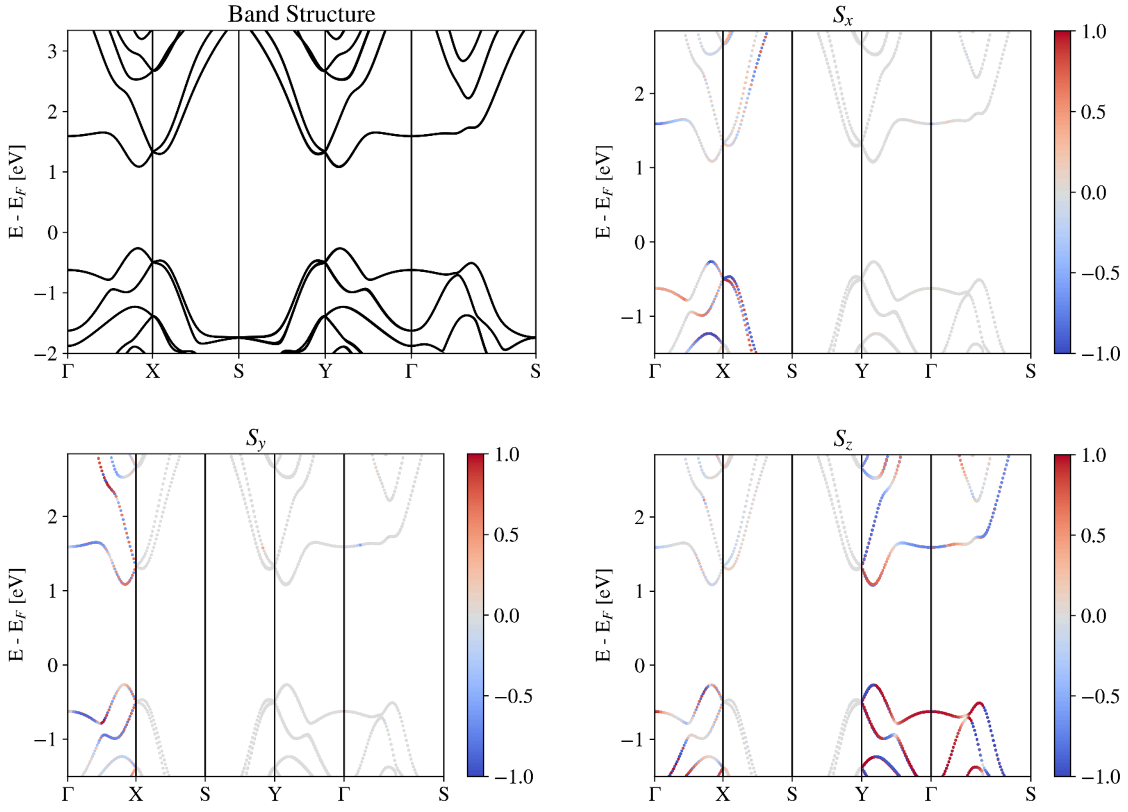


Figure 577: Band structure and spin polarization projections.

## 2.289 Pb2S2-fdff29051b6d

- **Formula**: Pb2S2
- **Structural Cluster**: AB-5
- **Band gap (PBE)**: 1.304 eV
- **Energy above convex hull (C2DB)**: 0.226 eV
- **Space group symbol**: *P*1
- **Space group number**: 1
- **Polar structure**: True

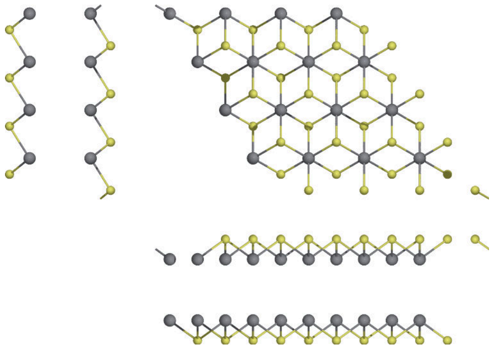

Figure 578: Structure representation

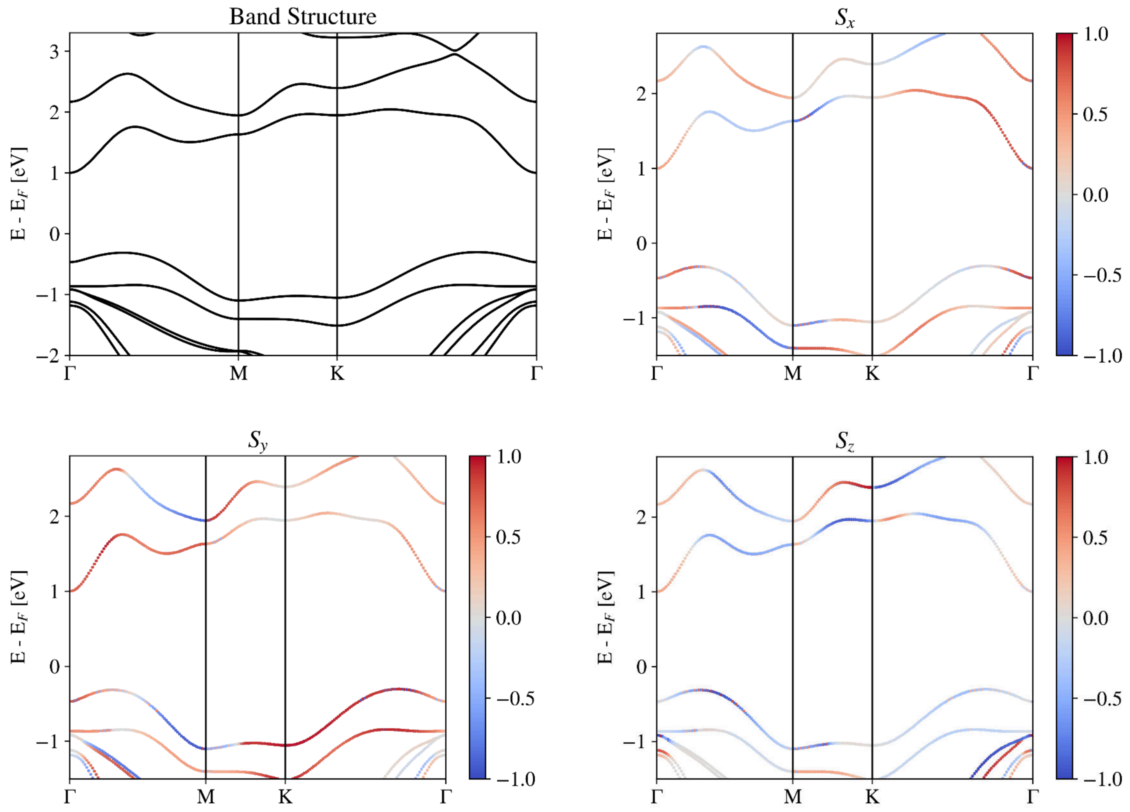


Figure 579: Band structure and spin polarization projections.

## 2.290 Pb2Se2-a346b1c50933

- **Formula**: Pb2Se2
- **Structural Cluster**: AB-5
- **Band gap (PBE)**: 1.024 eV
- **Energy above convex hull (C2DB)**: 0.211 eV
- **Space group symbol**: *Cm*
- **Space group number**: 8
- **Polar structure**: True

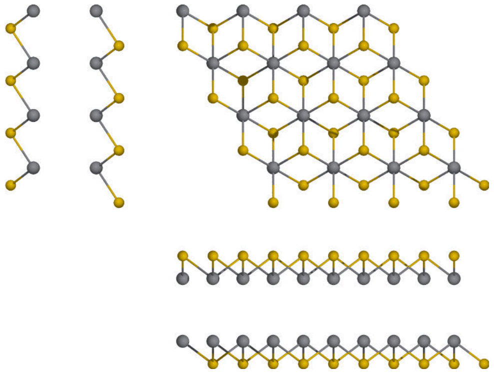

Figure 580: Structure representation

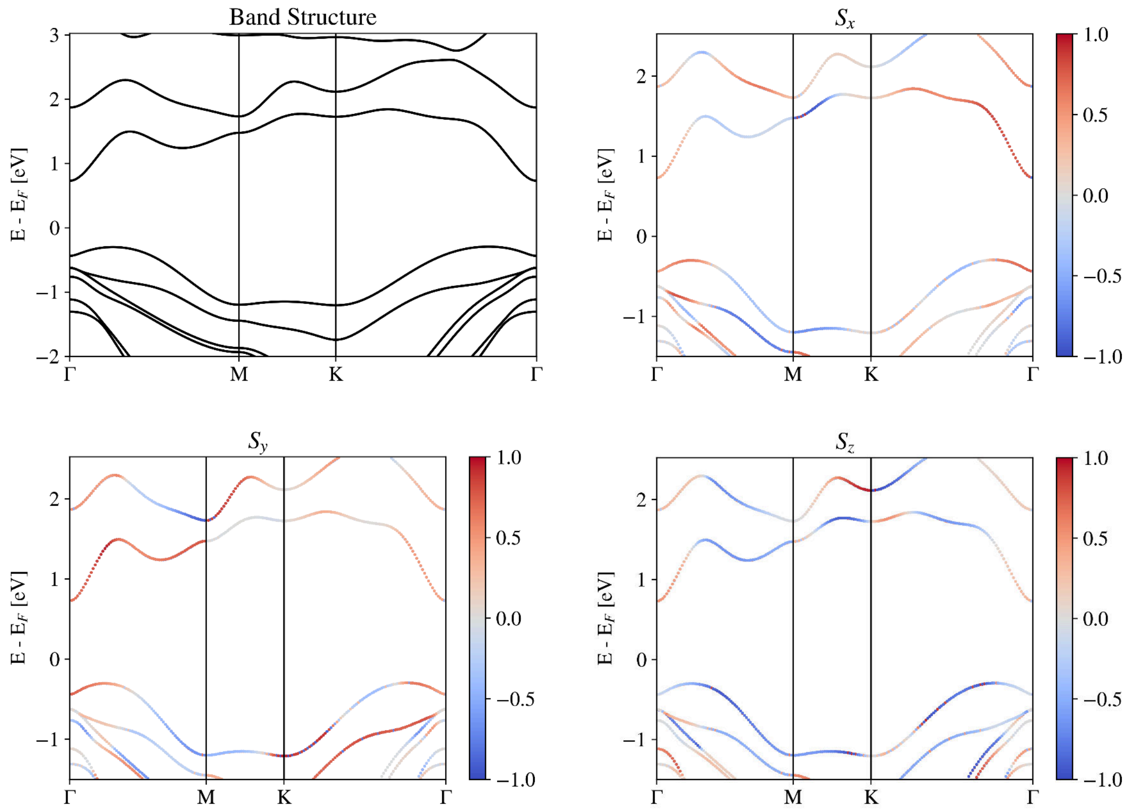


Figure 581: Band structure and spin polarization projections.

## 2.291 Pb2Se2-f615d3b872f4

- **Formula**: Pb2Se2
- **Structural Cluster**: AB-0
- **Band gap (PBE)**: 0.949 eV
- **Energy above convex hull (C2DB)**: 0.072 eV
- **Space group symbol**: $Pmn2_1$
- **Space group number**: 31
- **Polar structure**: True

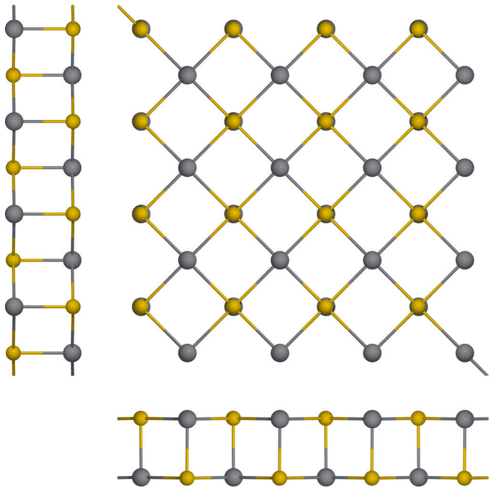

Figure 582: Structure representation

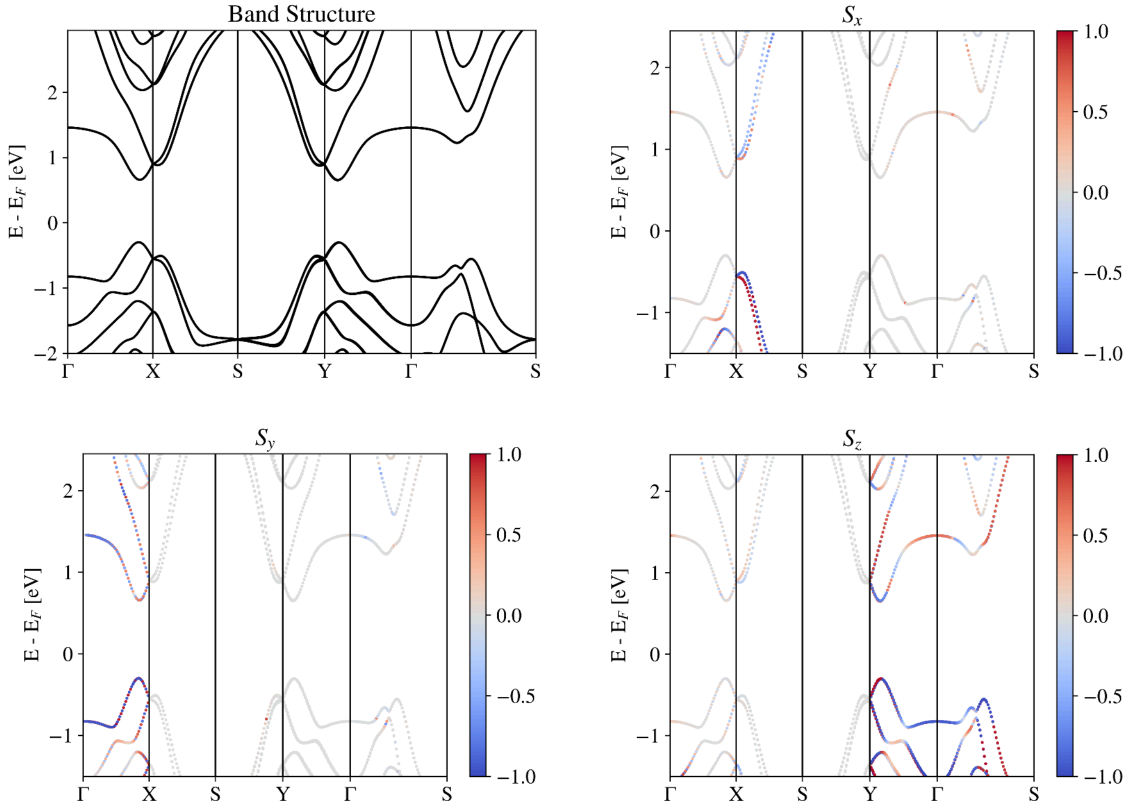


Figure 583: Band structure and spin polarization projections.

## 2.292 Pb2Te2-fdc4a7cc1d0d

- **Formula**: Pb2Te2
- **Structural Cluster**: AB-0
- **Band gap (PBE)**: 0.701 eV
- **Energy above convex hull (C2DB)**: 0.075 eV
- **Space group symbol**: $Pmn2_1$
- **Space group number**: 31
- **Polar structure**: True

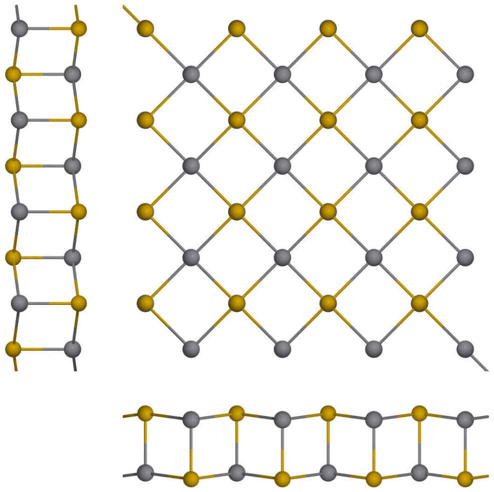

Figure 584: Structure representation

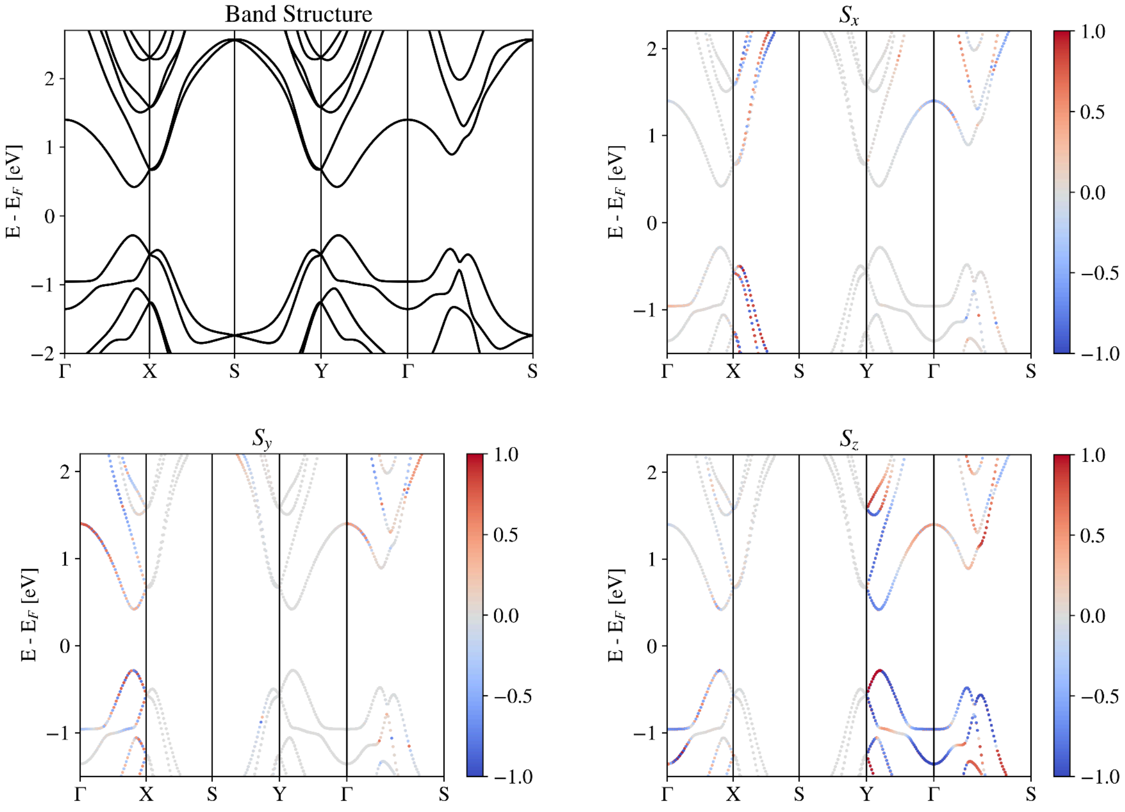


Figure 585: Band structure and spin polarization projections.

## 2.293 Pb2Te6-3995fa1bee6e

- **Formula**: Pb2Te6
- **Structural Cluster**: AB3-3
- **Band gap (PBE)**: 0.322 eV
- **Energy above convex hull (C2DB)**: 0.129 eV
- **Space group symbol**: $P2_1$
- **Space group number**: 4
- **Polar structure**: True

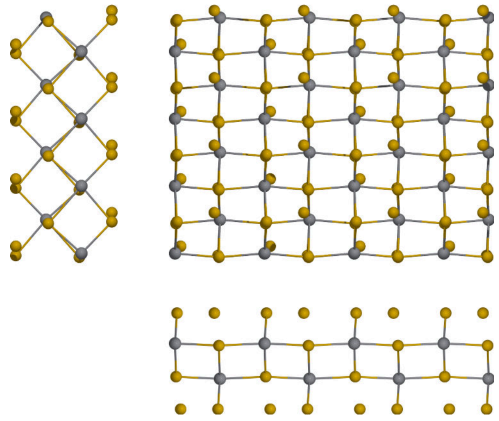

Figure 586: Structure representation

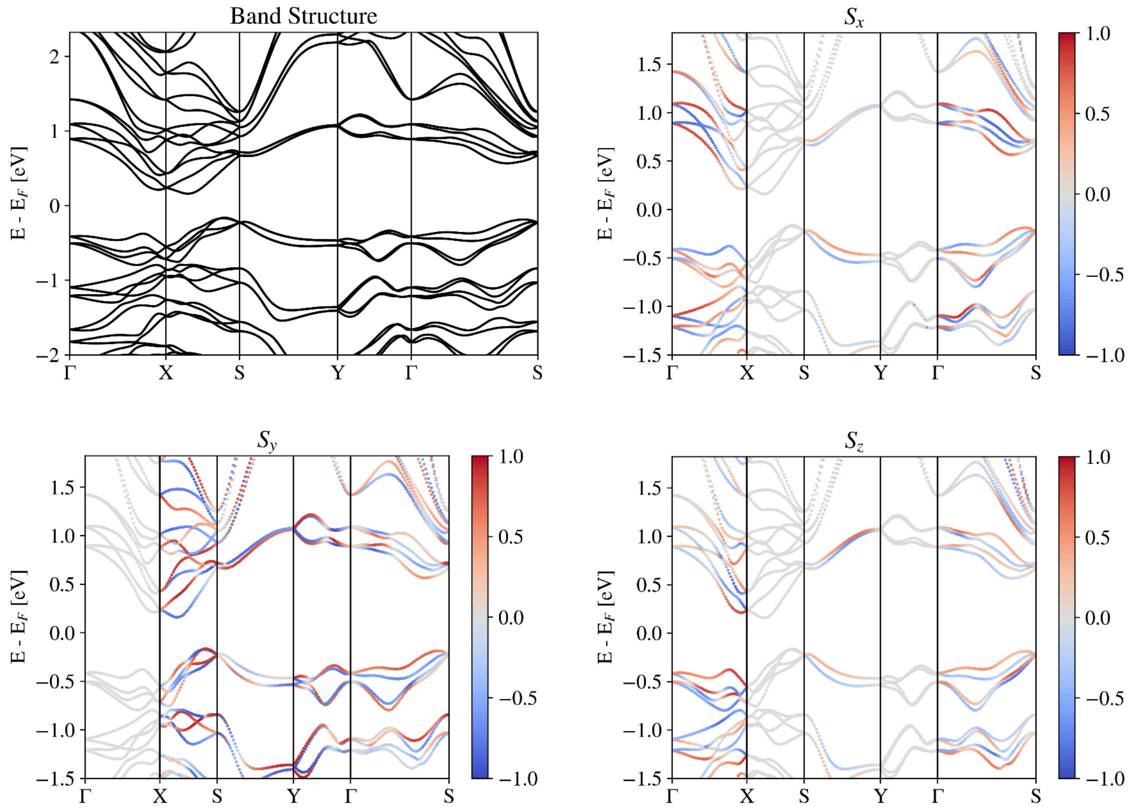


Figure 587: Band structure and spin polarization projections.

## 2.294 PbBr2-cabd4ba0f21c

- **Formula**: PbBr2
- **Structural Cluster**: AB2-11
- **Band gap (PBE)**: 1.884 eV
- **Energy above convex hull (C2DB)**: 0.136 eV
- **Space group symbol**: $P\bar{4}m2$
- **Space group number**: 115
- **Polar structure**: False

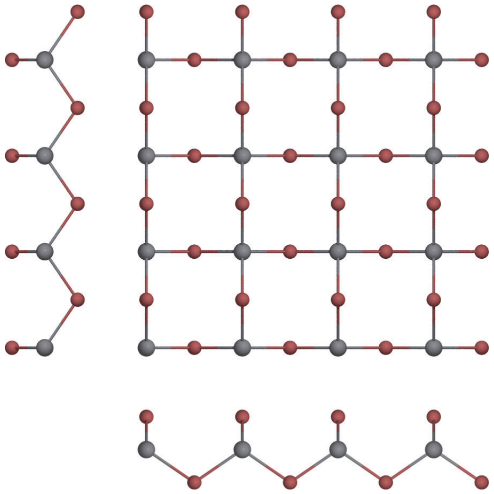

Figure 588: Structure representation

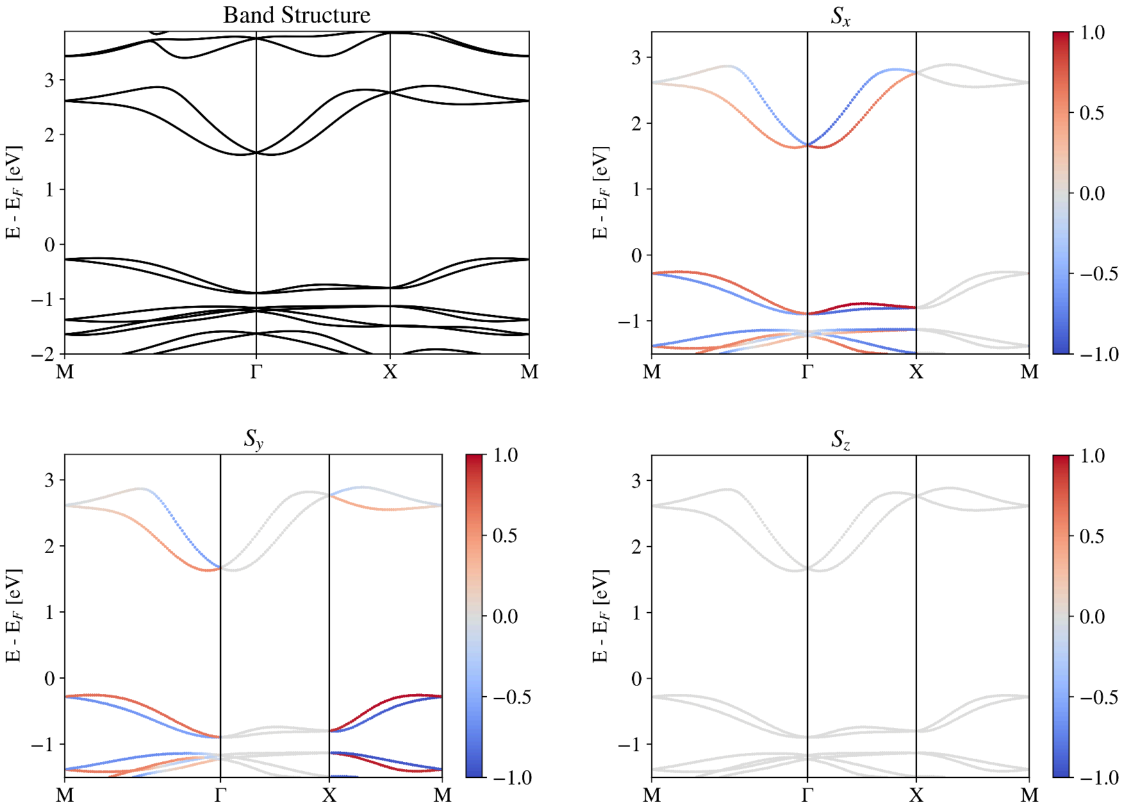


Figure 589: Band structure and spin polarization projections.

## 2.295 PbBr2-cbdc15b42a05

- **Formula**: PbBr2
- **Structural Cluster**: AB2-4
- **Band gap (PBE)**: 2.662 eV
- **Energy above convex hull (C2DB)**: 0.082 eV
- **Space group symbol**: $P\bar{6}m2$
- **Space group number**: 187
- **Polar structure**: False

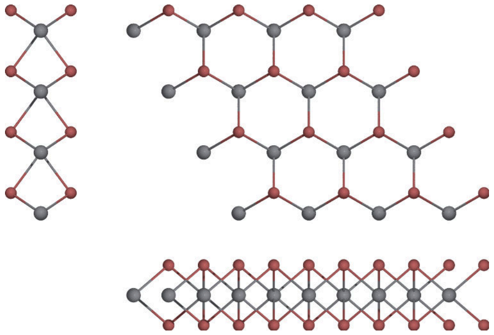

Figure 590: Structure representation

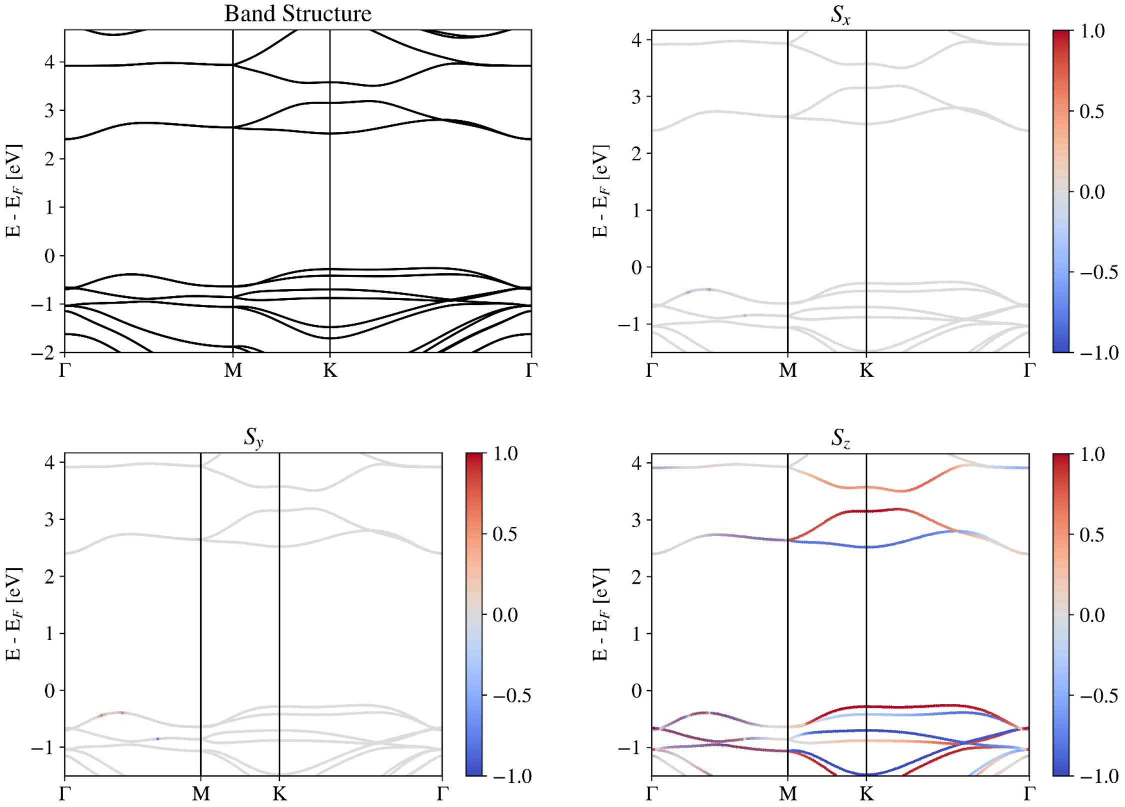


Figure 591: Band structure and spin polarization projections.

## 2.296 PbCl2-b0b142073783

- **Formula**: PbCl2
- **Structural Cluster**: AB2-4
- **Band gap (PBE)**: 3.136 eV
- **Energy above convex hull (C2DB)**: 0.105 eV
- **Space group symbol**: $P\bar{6}m2$
- **Space group number**: 187
- **Polar structure**: False

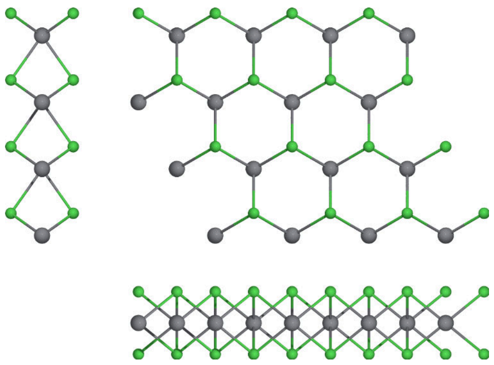

Figure 592: Structure representation

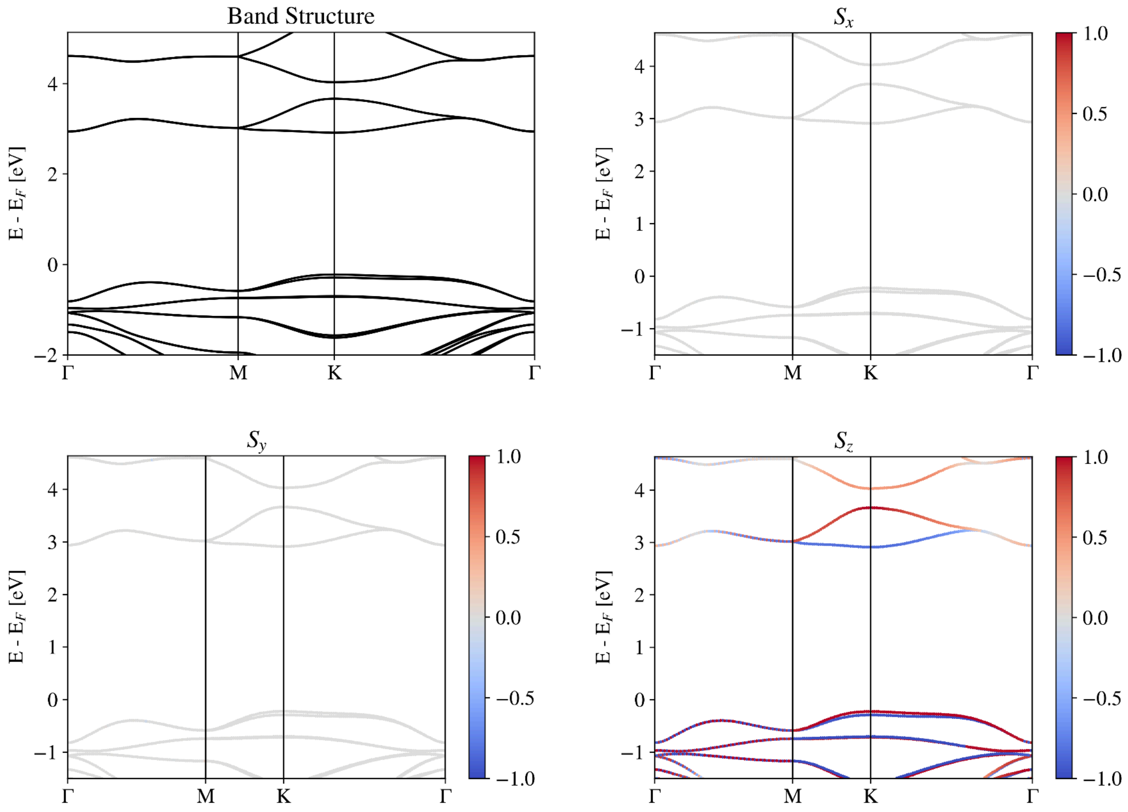


Figure 593: Band structure and spin polarization projections.

## 2.297 PbCl2-f9d58a299674

- **Formula**: PbCl2
- **Structural Cluster**: AB2-11
- **Band gap (PBE)**: 2.165 eV
- **Energy above convex hull (C2DB)**: 0.142 eV
- **Space group symbol**: $P\bar{4}m2$
- **Space group number**: 115
- **Polar structure**: False

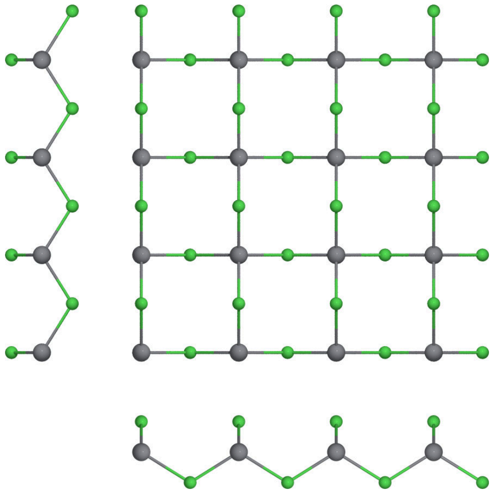

Figure 594: Structure representation

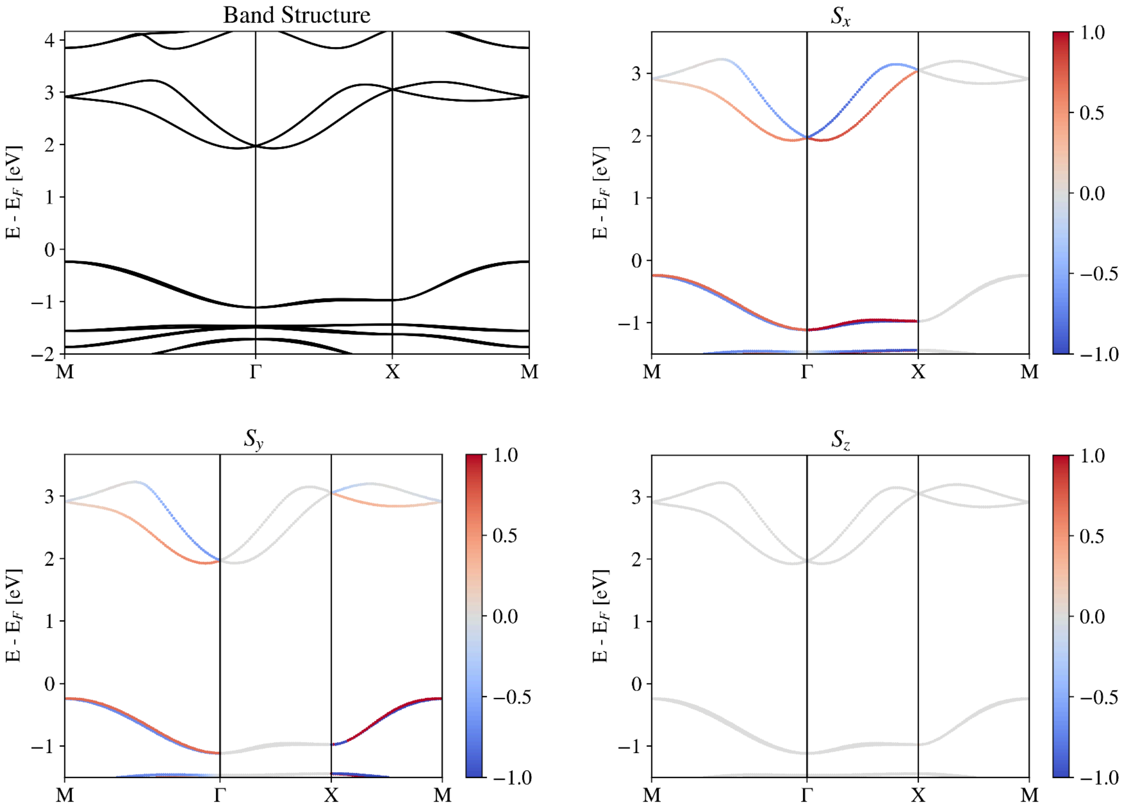


Figure 595: Band structure and spin polarization projections.

## 2.298 PbF2-ccc95033446d

- **Formula**: PbF2
- **Structural Cluster**: AB2-11
- **Band gap (PBE)**: 2.766 eV
- **Energy above convex hull (C2DB)**: 0.243 eV
- **Space group symbol**: $P\bar{4}m2$
- **Space group number**: 115
- **Polar structure**: False

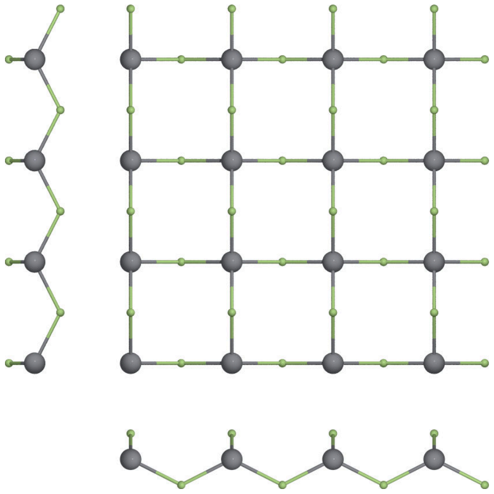

Figure 596: Structure representation

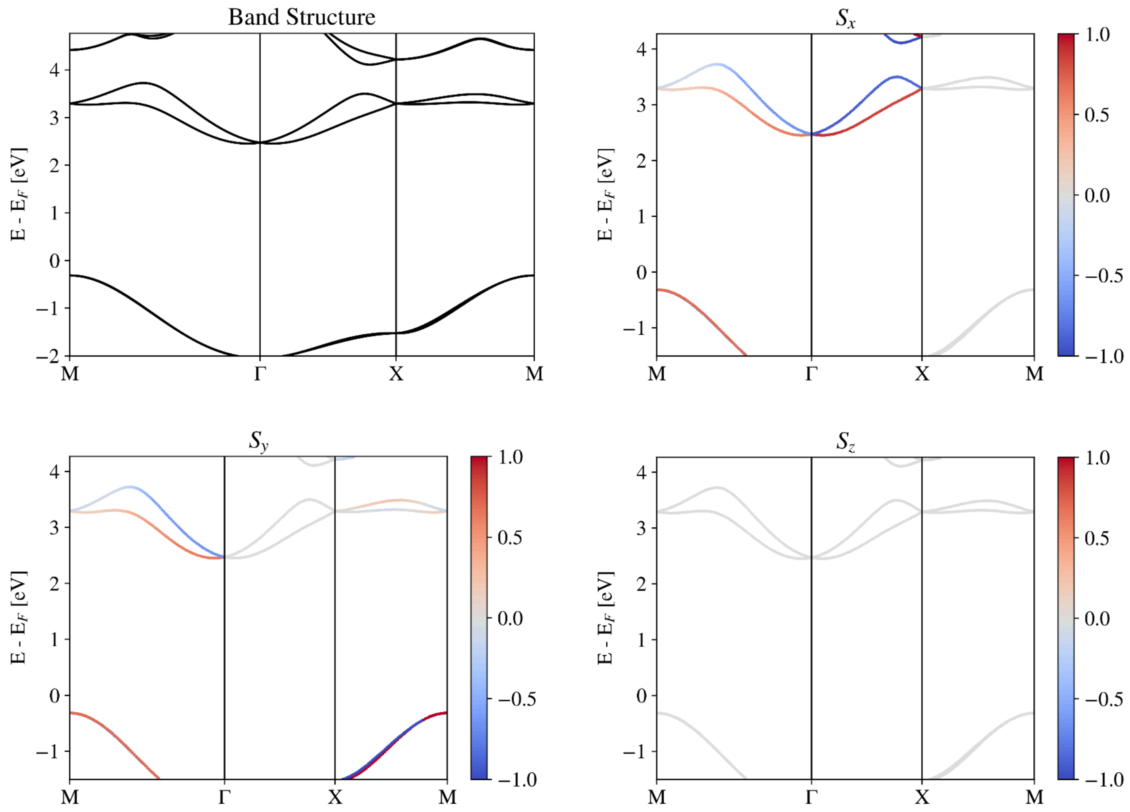


Figure 597: Band structure and spin polarization projections.

## 2.299 PbI2-14411dde597c

- **Formula**: PbI2
- **Structural Cluster**: AB2-11
- **Band gap (PBE)**: 1.531 eV
- **Energy above convex hull (C2DB)**: 0.145 eV
- **Space group symbol**: $P\bar{4}m2$
- **Space group number**: 115
- **Polar structure**: False

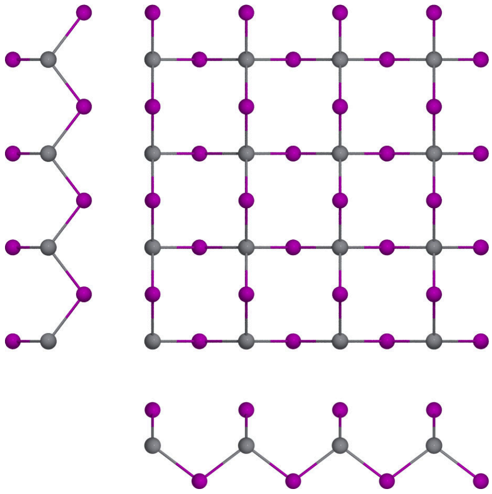

Figure 598: Structure representation

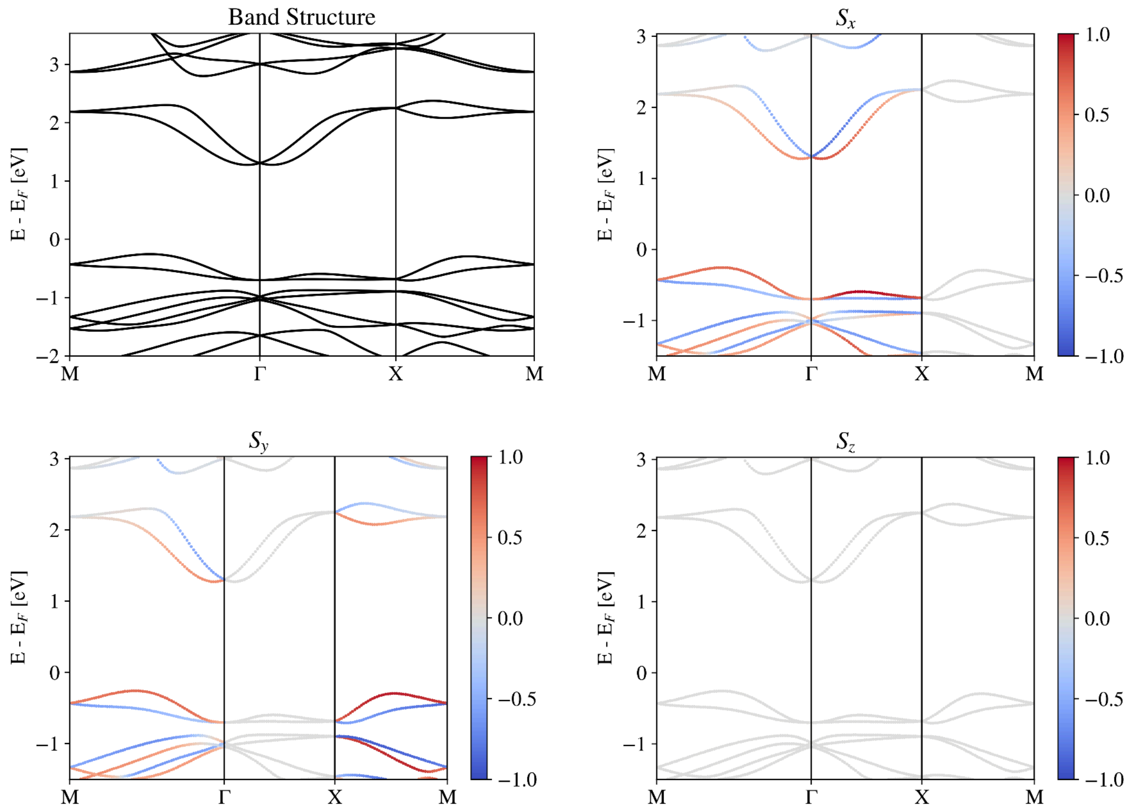


Figure 599: Band structure and spin polarization projections.

## 2.300 PbI2-9e6494406d07

- **Formula**: PbI2
- **Structural Cluster**: AB2-4
- **Band gap (PBE)**: 2.029 eV
- **Energy above convex hull (C2DB)**: 0.078 eV
- **Space group symbol**: $P\bar{6}m2$
- **Space group number**: 187
- **Polar structure**: False

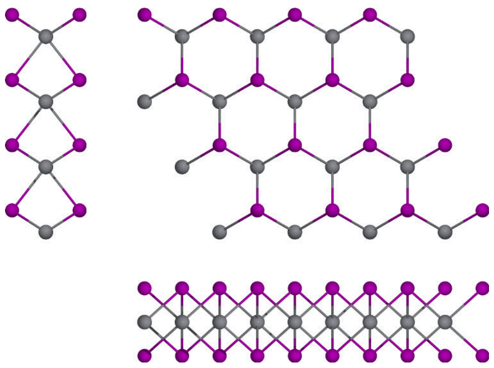

Figure 600: Structure representation

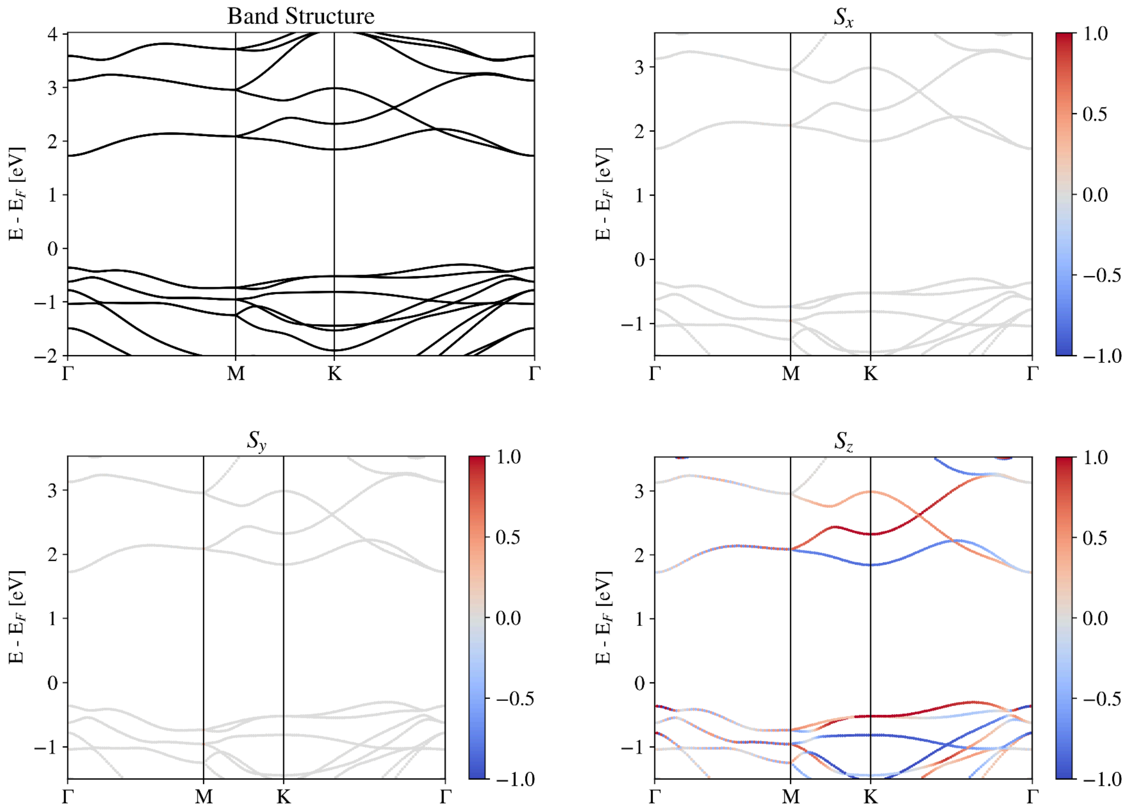


Figure 601: Band structure and spin polarization projections.

## 2.301 PbO2-8d2de90b58b6

- **Formula**: PbO2
- **Structural Cluster**: AB2-11
- **Band gap (PBE)**: 1.084 eV
- **Energy above convex hull (C2DB)**: 0.339 eV
- **Space group symbol**: $P\bar{4}m2$
- **Space group number**: 115
- **Polar structure**: False

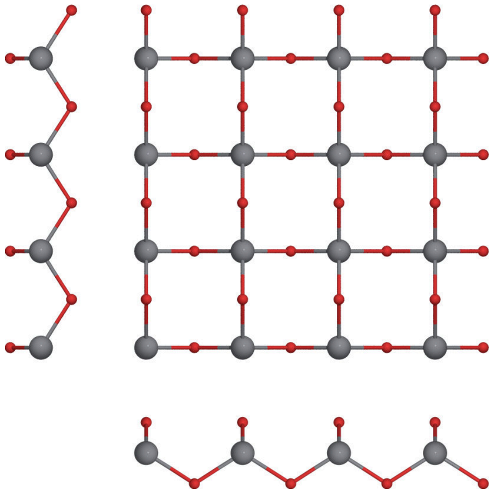

Figure 602: Structure representation

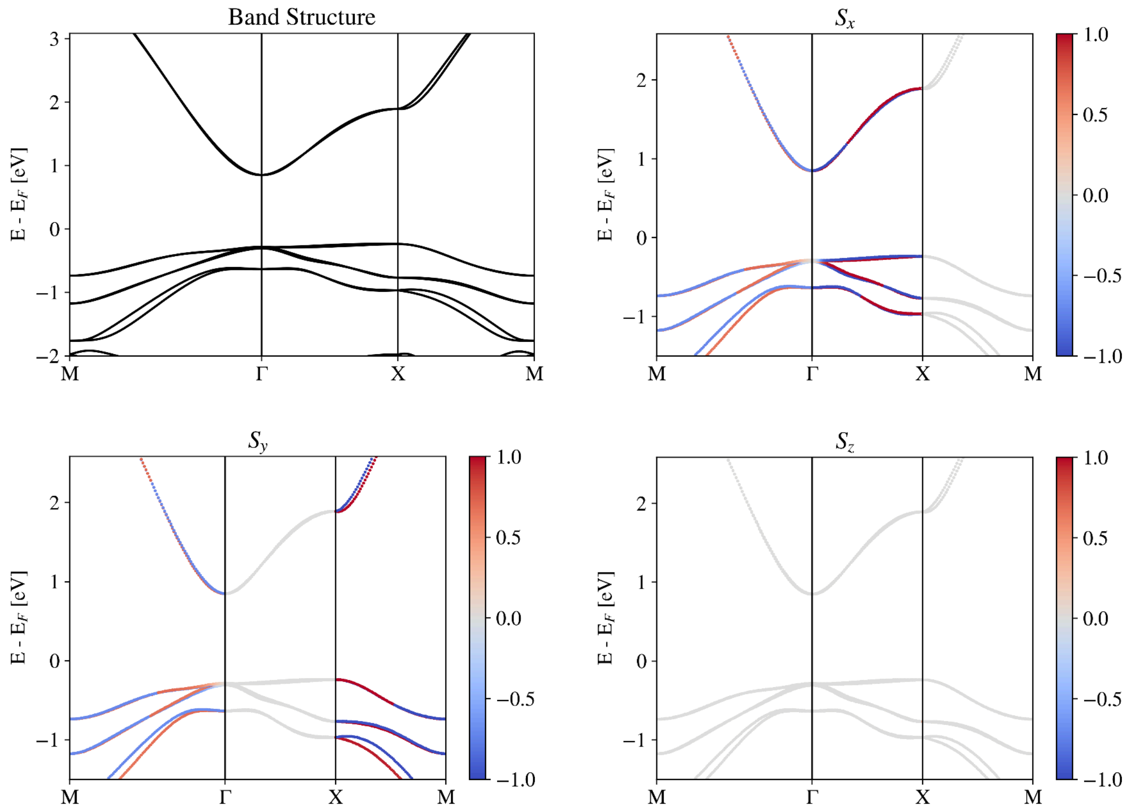


Figure 603: Band structure and spin polarization projections.

## 2.302 PbS-5e4ff1f56b4a

- **Formula**: PbS
- **Structural Cluster**: AB-25
- **Band gap (PBE)**: 1.979 eV
- **Energy above convex hull (C2DB)**: 0.231 eV
- **Space group symbol**: $P3m1$
- **Space group number**: 156
- **Polar structure**: True

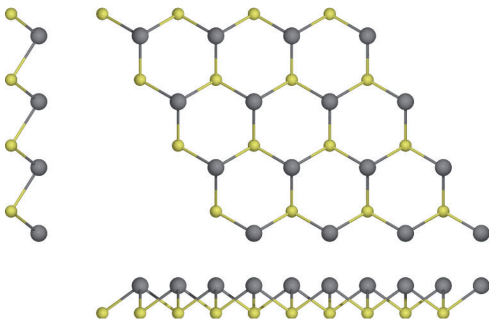

Figure 604: Structure representation

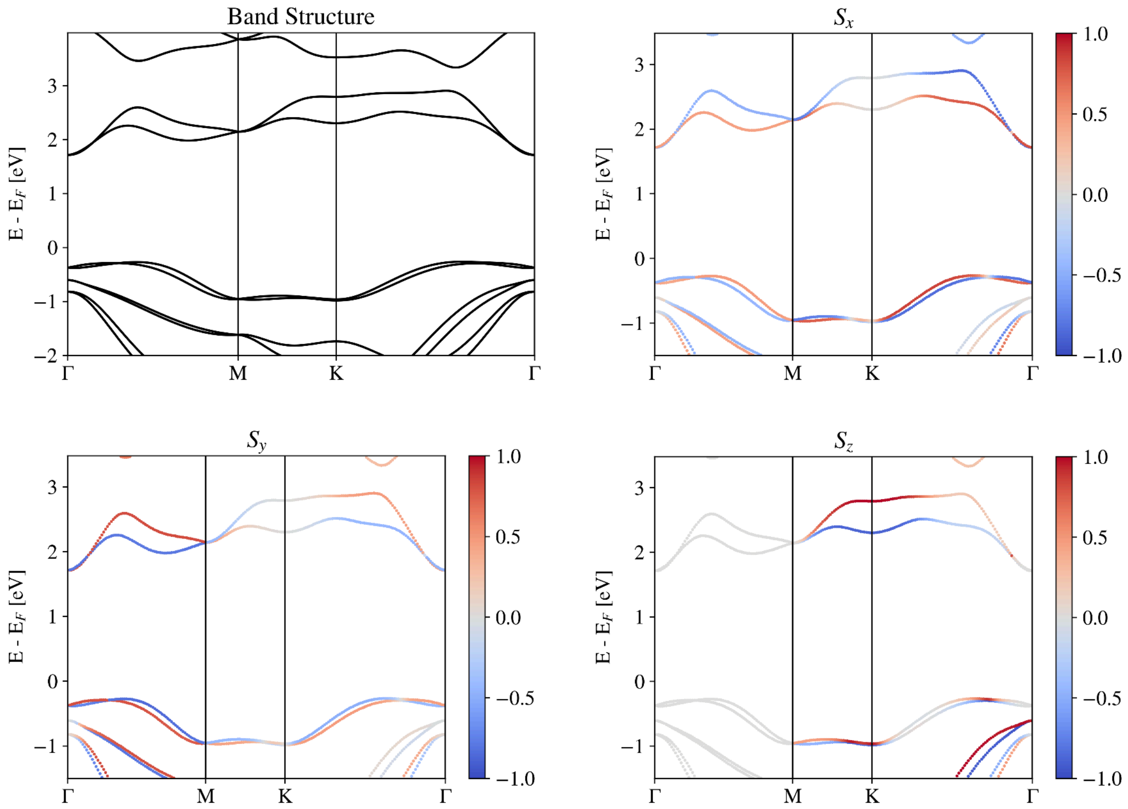


Figure 605: Band structure and spin polarization projections.

## 2.303 PbS2-372c217dd52f

- **Formula**: PbS2
- **Structural Cluster**: AB2-4
- **Band gap (PBE)**: 1.708 eV
- **Energy above convex hull (C2DB)**: 0.191 eV
- **Space group symbol**: $P\bar{6}m2$
- **Space group number**: 187
- **Polar structure**: False

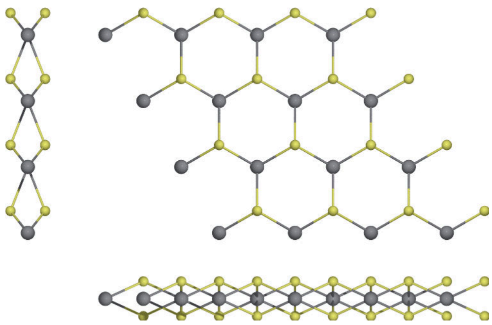

Figure 606: Structure representation

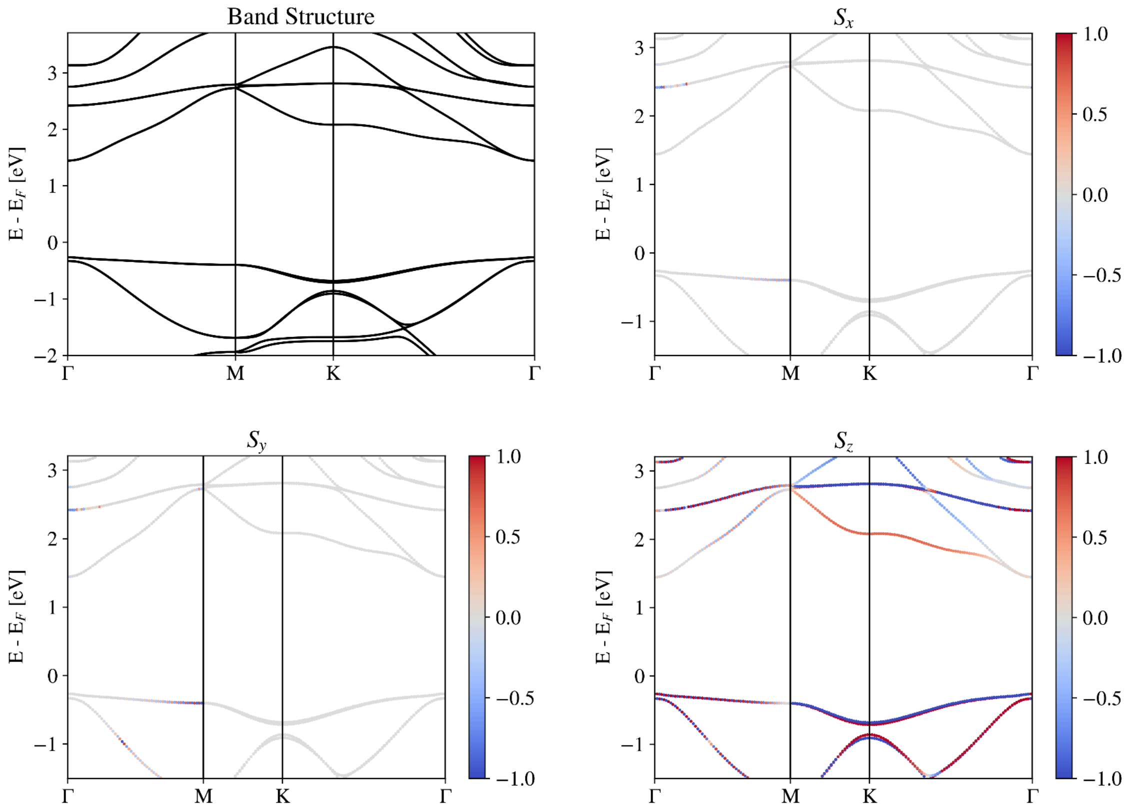


Figure 607: Band structure and spin polarization projections.

## 2.304 PbS2-9842835dff03

- **Formula**: PbS2
- **Structural Cluster**: AB2-11
- **Band gap (PBE)**: 0.671 eV
- **Energy above convex hull (C2DB)**: 0.418 eV
- **Space group symbol**: $P\bar{4}m2$
- **Space group number**: 115
- **Polar structure**: False

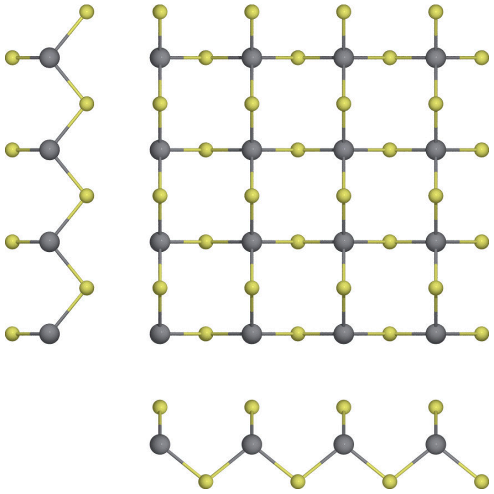

Figure 608: Structure representation

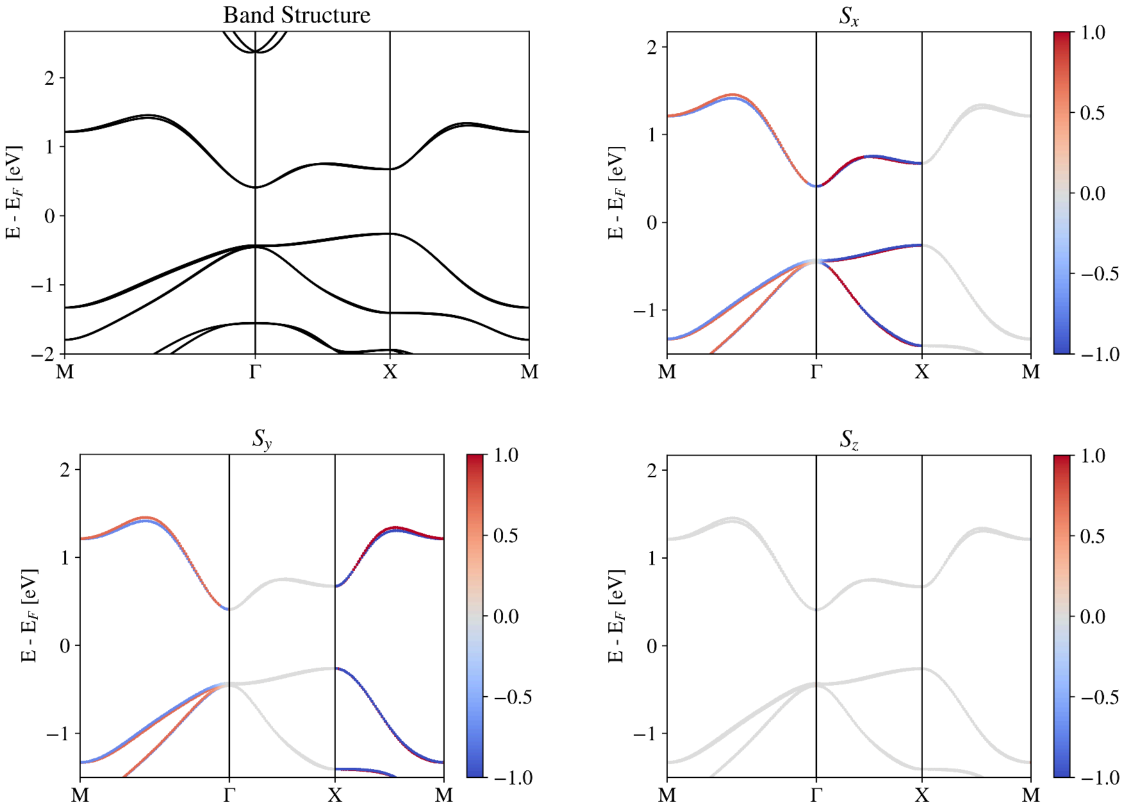


Figure 609: Band structure and spin polarization projections.

## 2.305 PbSe-a0dbdc6630fa

- **Formula**: PbSe
- **Structural Cluster**: AB-25
- **Band gap (PBE)**: 1.68 eV
- **Energy above convex hull (C2DB)**: 0.217 eV
- **Space group symbol**: $P3m1$
- **Space group number**: 156
- **Polar structure**: True

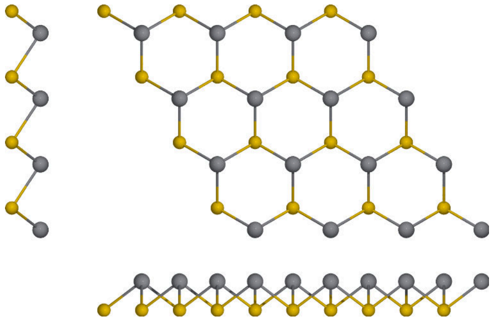

Figure 610: Structure representation

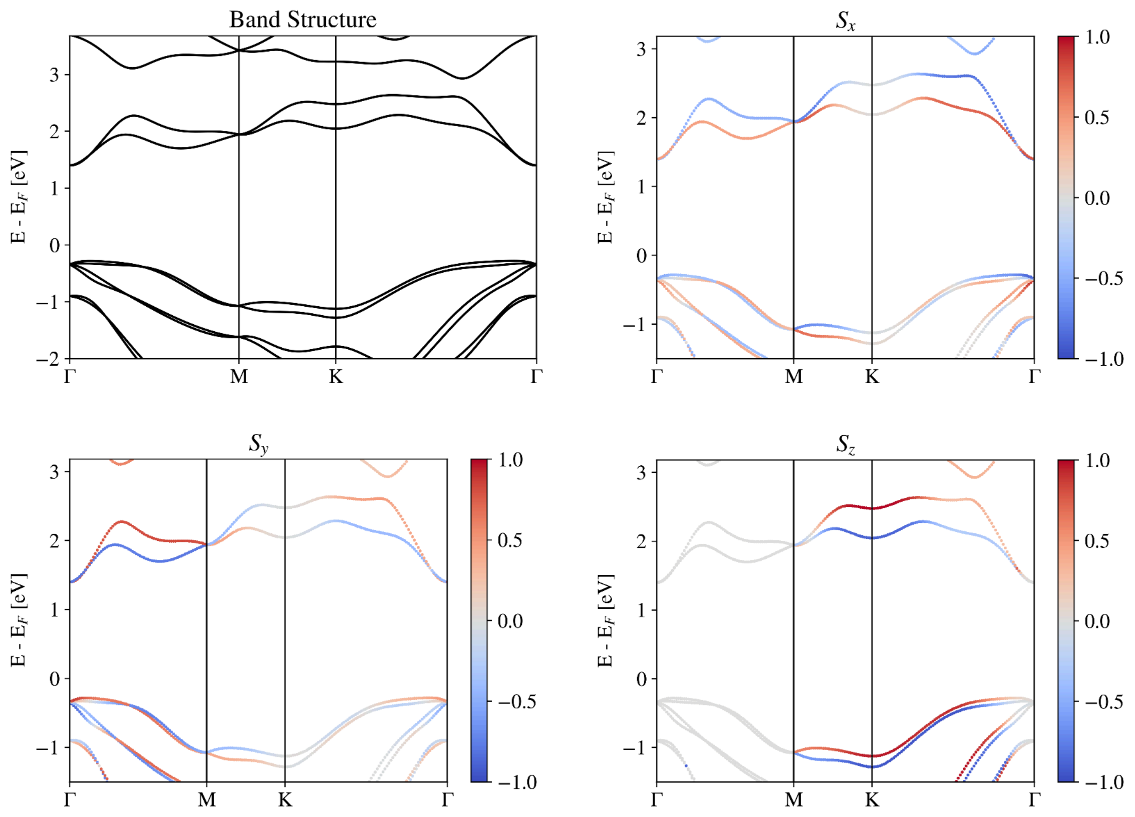


Figure 611: Band structure and spin polarization projections.

## 2.306 PbSe2-0bc5d11454a7

- **Formula**: PbSe2
- **Structural Cluster**: AB2-4
- **Band gap (PBE)**: 1.324 eV
- **Energy above convex hull (C2DB)**: 0.18 eV
- **Space group symbol**: $P\bar{6}m2$
- **Space group number**: 187
- **Polar structure**: False

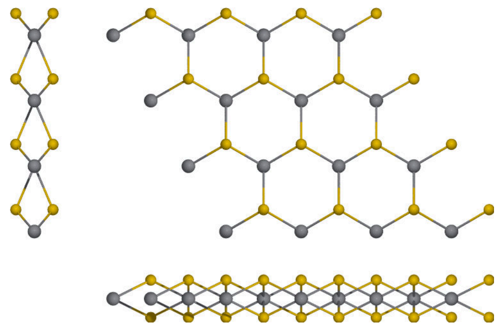

Figure 612: Structure representation

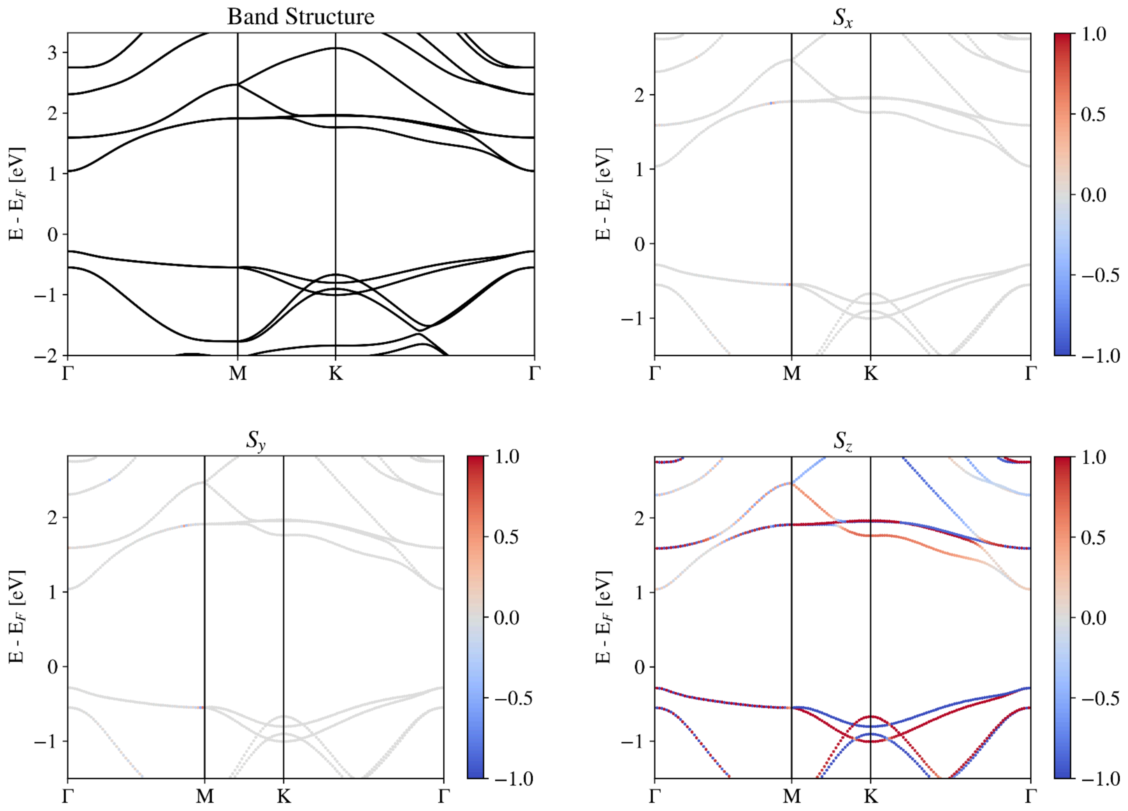


Figure 613: Band structure and spin polarization projections.

## 2.307 PbSe2-45aa9714a72b

- **Formula**: PbSe2
- **Structural Cluster**: AB2-11
- **Band gap (PBE)**: 0.286 eV
- **Energy above convex hull (C2DB)**: 0.372 eV
- **Space group symbol**: $P\bar{4}m2$
- **Space group number**: 115
- **Polar structure**: False

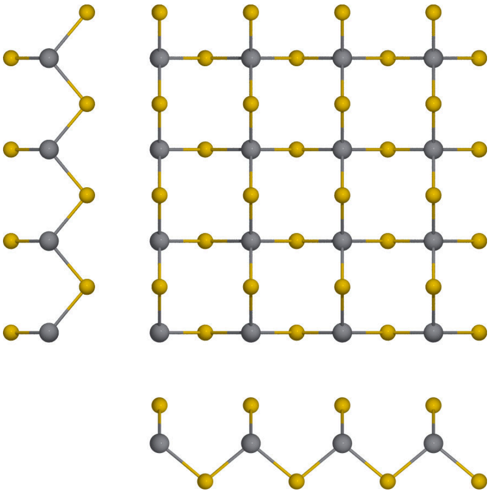

Figure 614: Structure representation

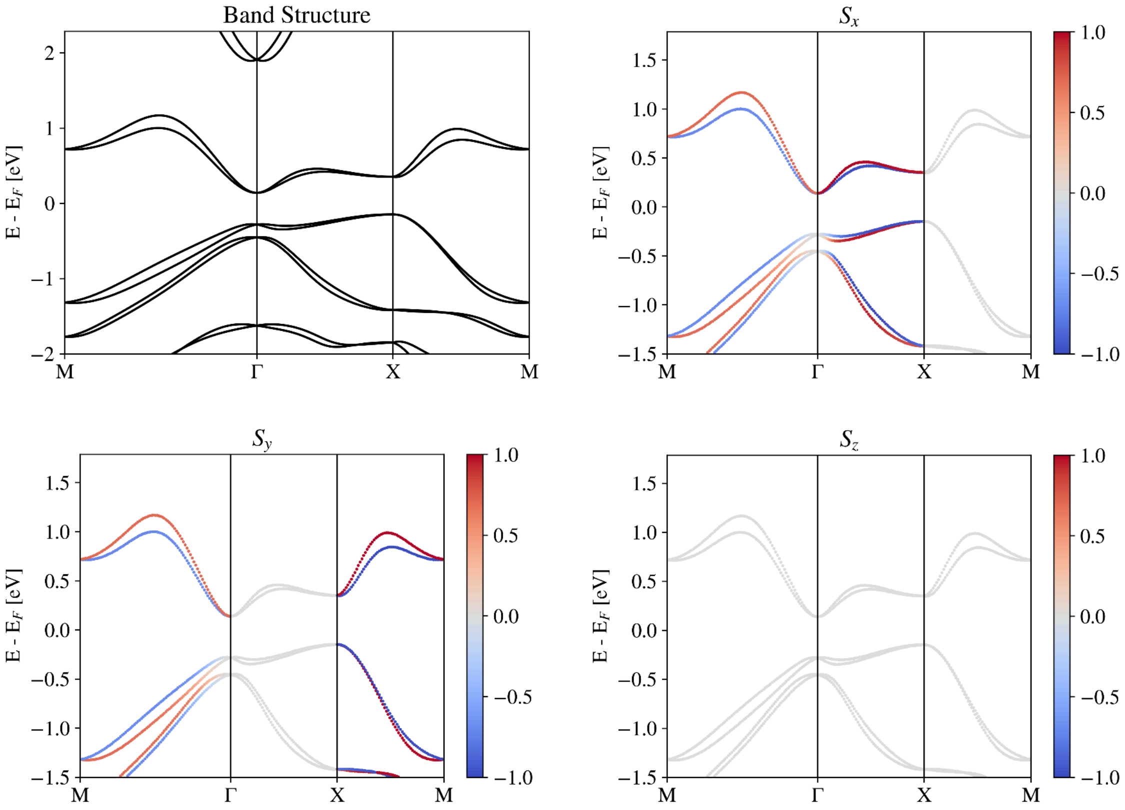


Figure 615: Band structure and spin polarization projections.

## 2.308 PbTe-3bc08d486d65

- **Formula**: PbTe
- **Structural Cluster**: AB-25
- **Band gap (PBE)**: 1.151 eV
- **Energy above convex hull (C2DB)**: 0.198 eV
- **Space group symbol**: $P3m1$
- **Space group number**: 156
- **Polar structure**: True

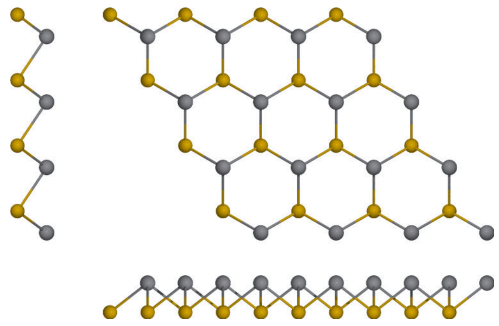

Figure 616: Structure representation

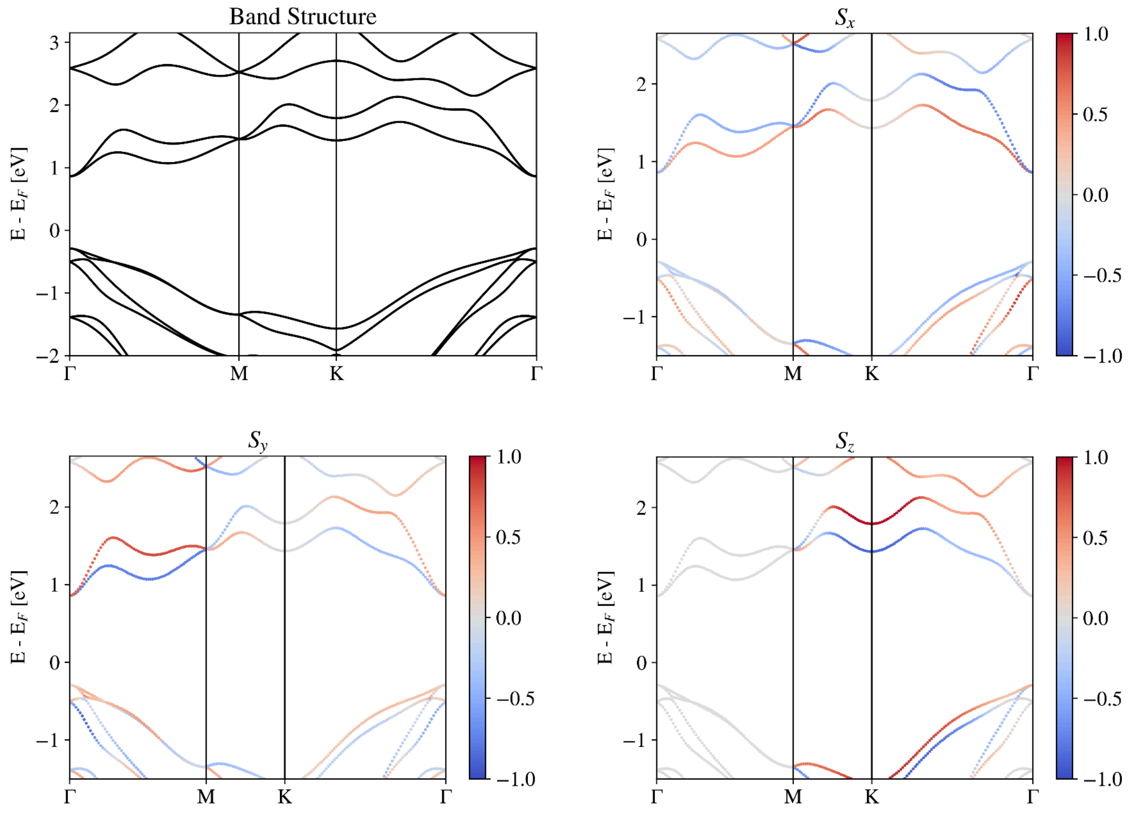


Figure 617: Band structure and spin polarization projections.

## 2.309 PbTe2-dbf3271b4bb1

- **Formula**: PbTe2
- **Structural Cluster**: AB2-11
- **Band gap (PBE)**: 0.085 eV
- **Energy above convex hull (C2DB)**: 0.423 eV
- **Space group symbol**: $P\bar{4}m2$
- **Space group number**: 115
- **Polar structure**: False

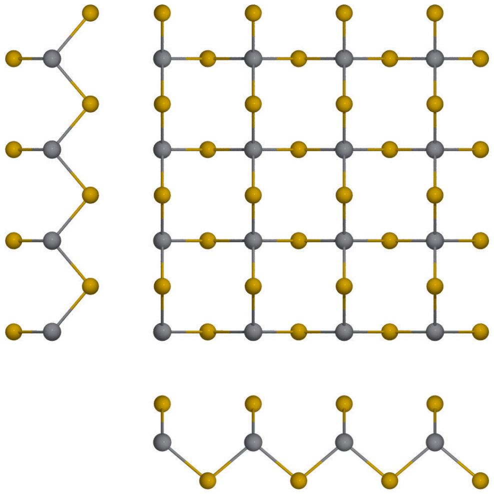

Figure 618: Structure representation

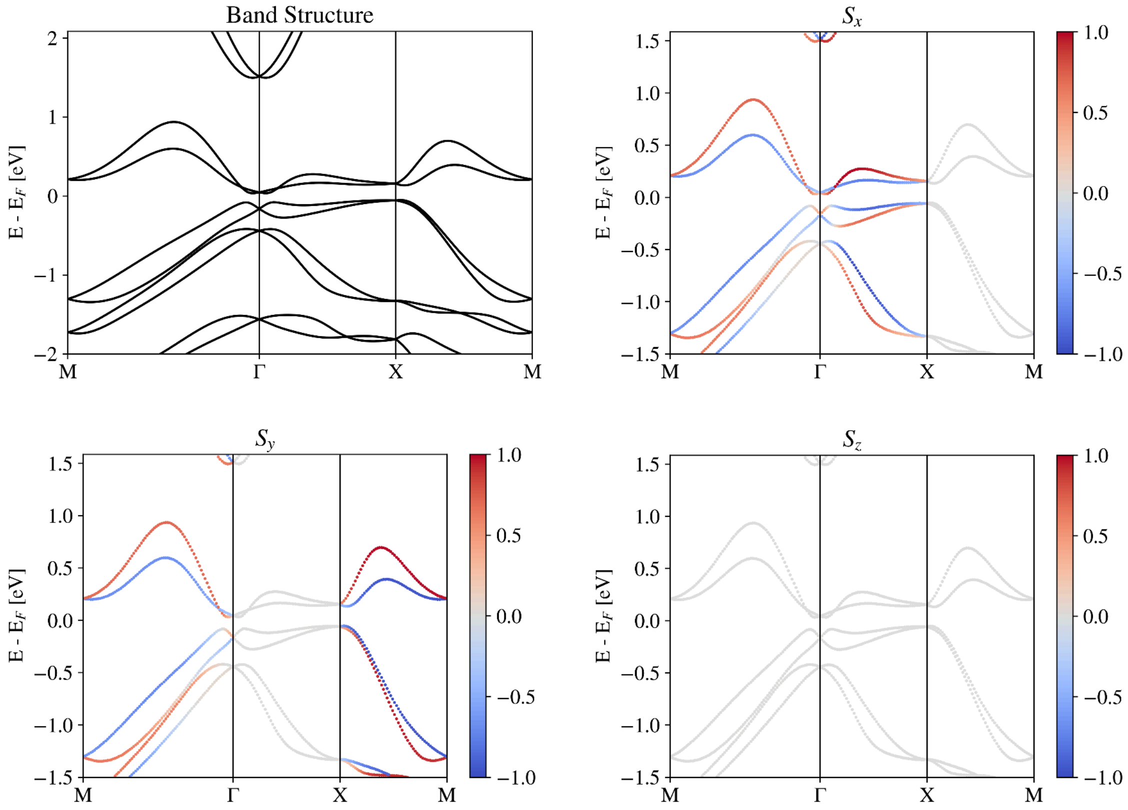


Figure 619: Band structure and spin polarization projections.

## 2.310 PdSe2-0ae696751911

- **Formula**: PdSe2
- **Structural Cluster**: AB2-4
- **Band gap (PBE)**: 0.231 eV
- **Energy above convex hull (C2DB)**: 0.268 eV
- **Space group symbol**: $P\bar{6}m2$
- **Space group number**: 187
- **Polar structure**: False

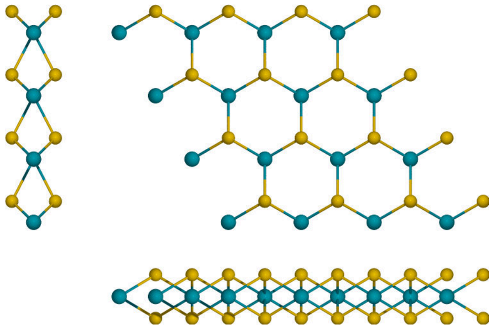

Figure 620: Structure representation

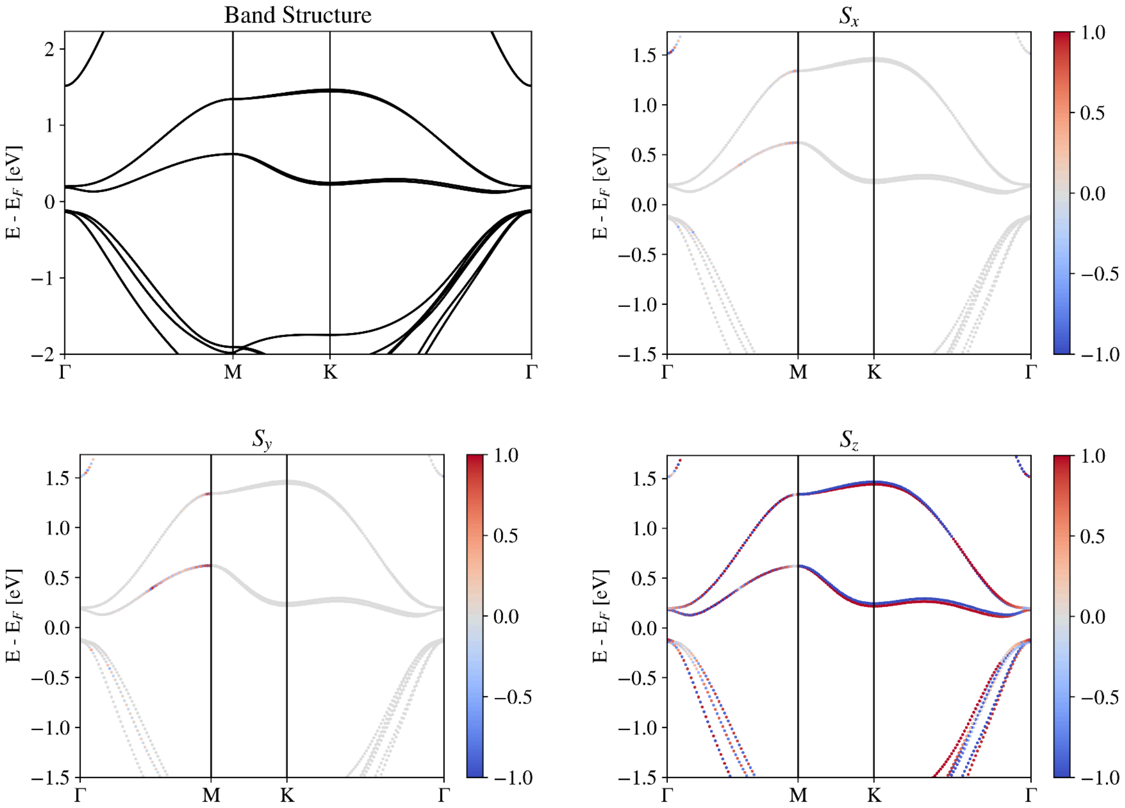


Figure 621: Band structure and spin polarization projections.

## 2.311 Re2Cl6-21e7665ac8f4

- **Formula**: Re2Cl6
- **Structural Cluster**: AB3-20
- **Band gap (PBE)**: 0.005 eV
- **Energy above convex hull (C2DB)**: 0.515 eV
- **Space group symbol**: $P\bar{6}2m$
- **Space group number**: 189
- **Polar structure**: False

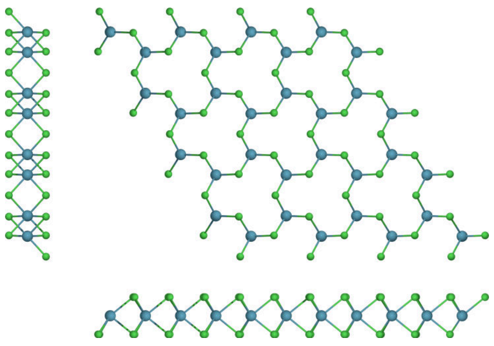

Figure 622: Structure representation

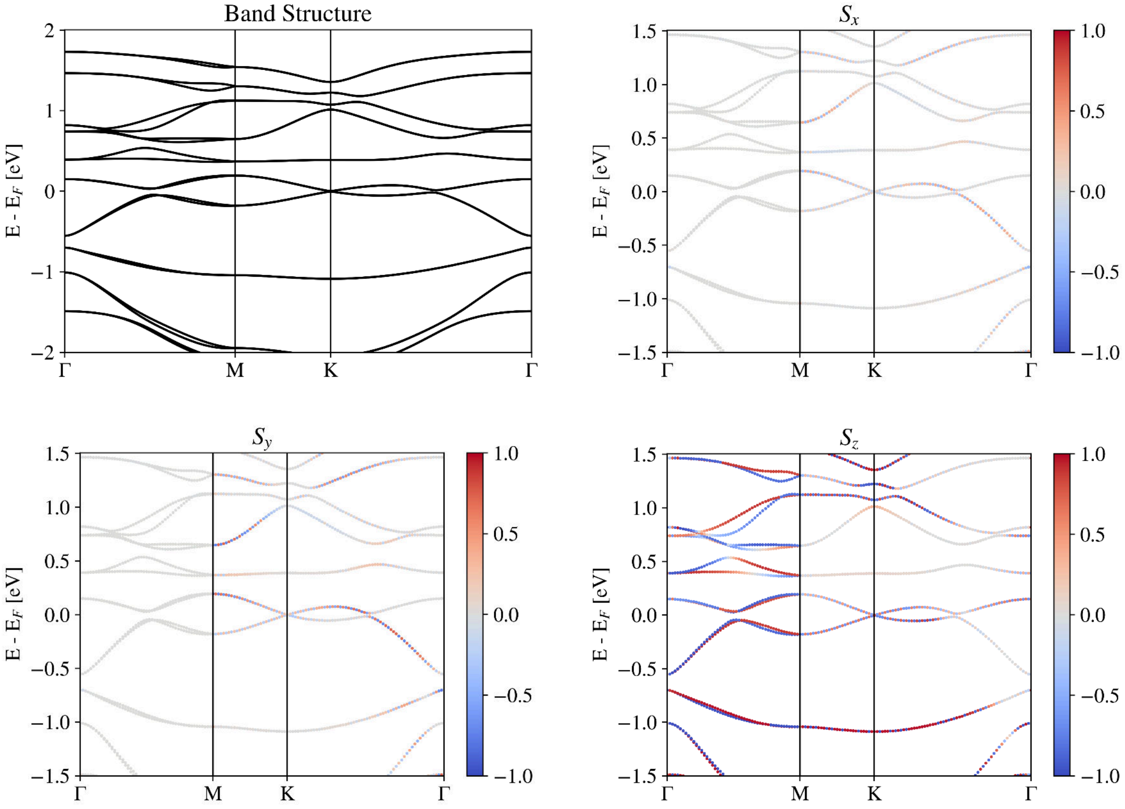


Figure 623: Band structure and spin polarization projections.

## 2.312 Re2I6-04979b21e197

- **Formula**: Re2I6
- **Structural Cluster**: AB3-19
- **Band gap (PBE)**: 0.136 eV
- **Energy above convex hull (C2DB)**: 0.425 eV
- **Space group symbol**: $P\bar{6}2m$
- **Space group number**: 189
- **Polar structure**: False

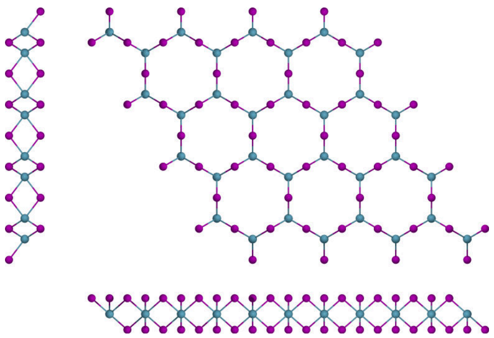

Figure 624: Structure representation

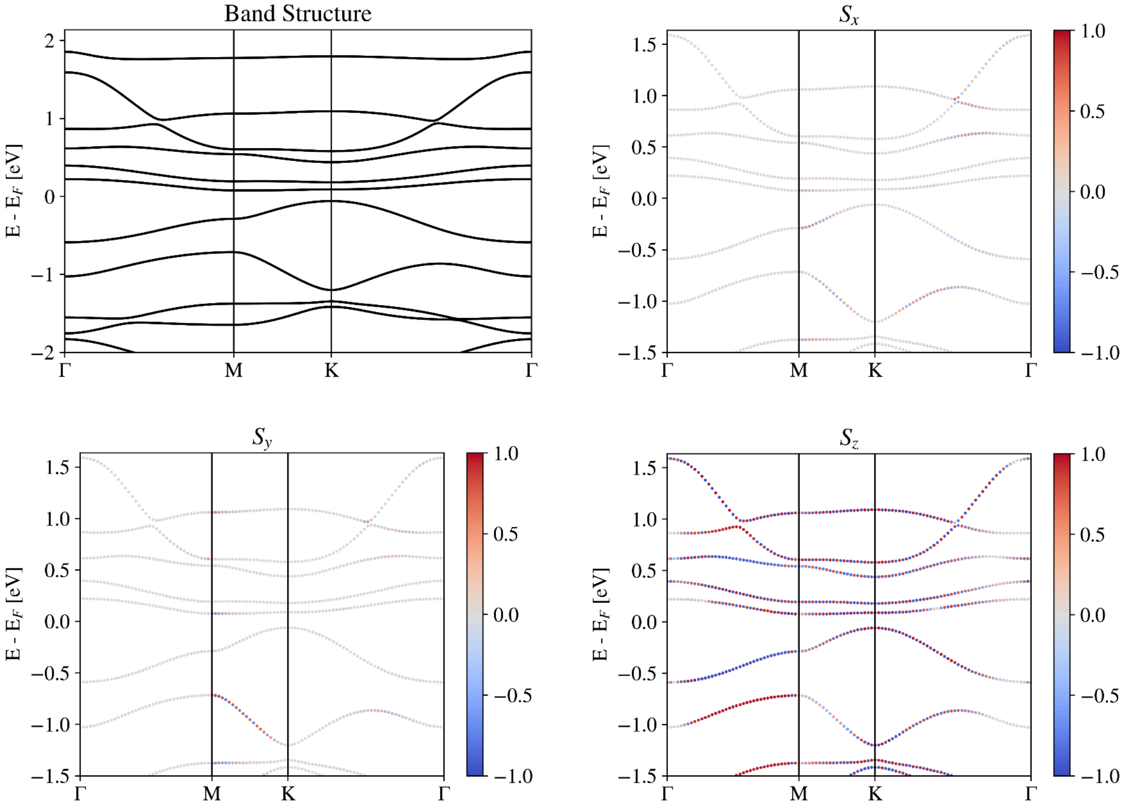


Figure 625: Band structure and spin polarization projections.

## 2.313 Rh2Br6-c284d6de2b3e

- **Formula**: Rh2Br6
- **Structural Cluster**: AB3-19
- **Band gap (PBE)**: 0.26 eV
- **Energy above convex hull (C2DB)**: 0.408 eV
- **Space group symbol**: *P*3
- **Space group number**: 143
- **Polar structure**: True

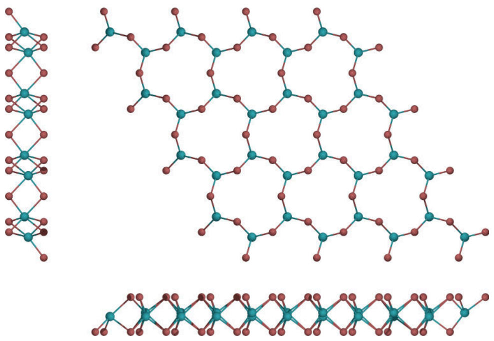

Figure 626: Structure representation

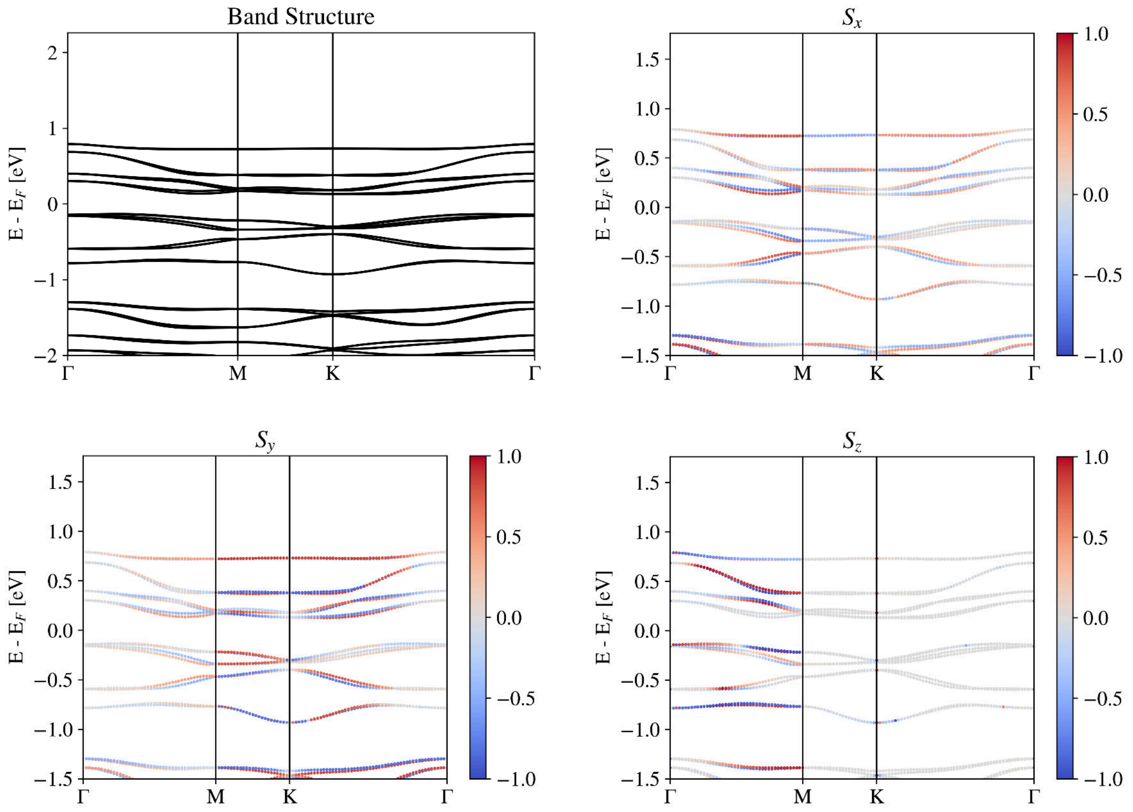


Figure 627: Band structure and spin polarization projections.

## 2.314 Rh2Cl6-06f695e97385

- **Formula**: Rh2Cl6
- **Structural Cluster**: AB3-19
- **Band gap (PBE)**: 0.208 eV
- **Energy above convex hull (C2DB)**: 0.464 eV
- **Space group symbol**: *P*321
- **Space group number**: 150
- **Polar structure**: False

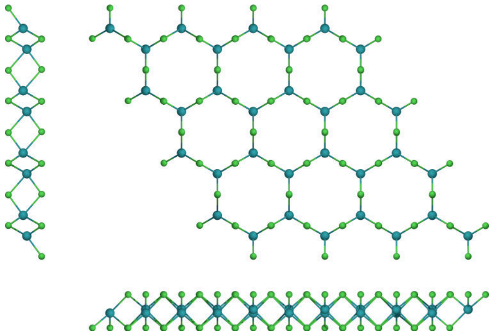

Figure 628: Structure representation

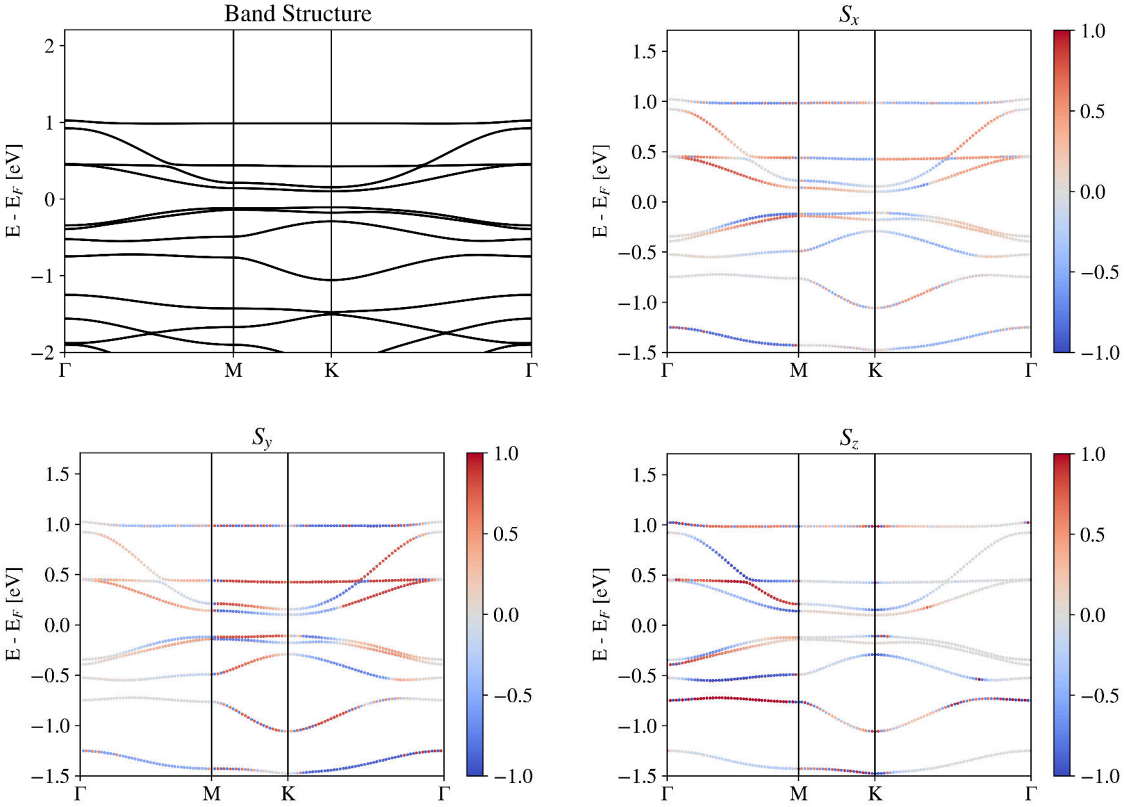


Figure 629: Band structure and spin polarization projections.

## 2.315 Rh2Se2-1a46a7cf8fab

- **Formula**: Rh2Se2
- **Structural Cluster**: AB-5
- **Band gap (PBE)**: 0.063 eV
- **Energy above convex hull (C2DB)**: 0.164 eV
- **Space group symbol**: $P\bar{6}m2$
- **Space group number**: 187
- **Polar structure**: False

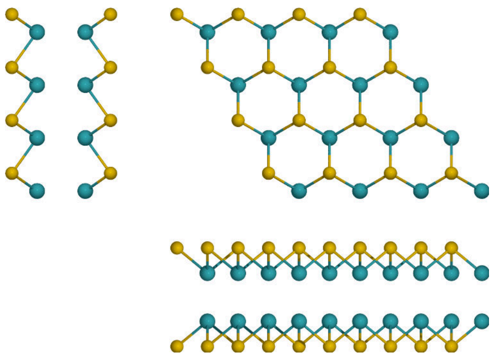

Figure 630: Structure representation

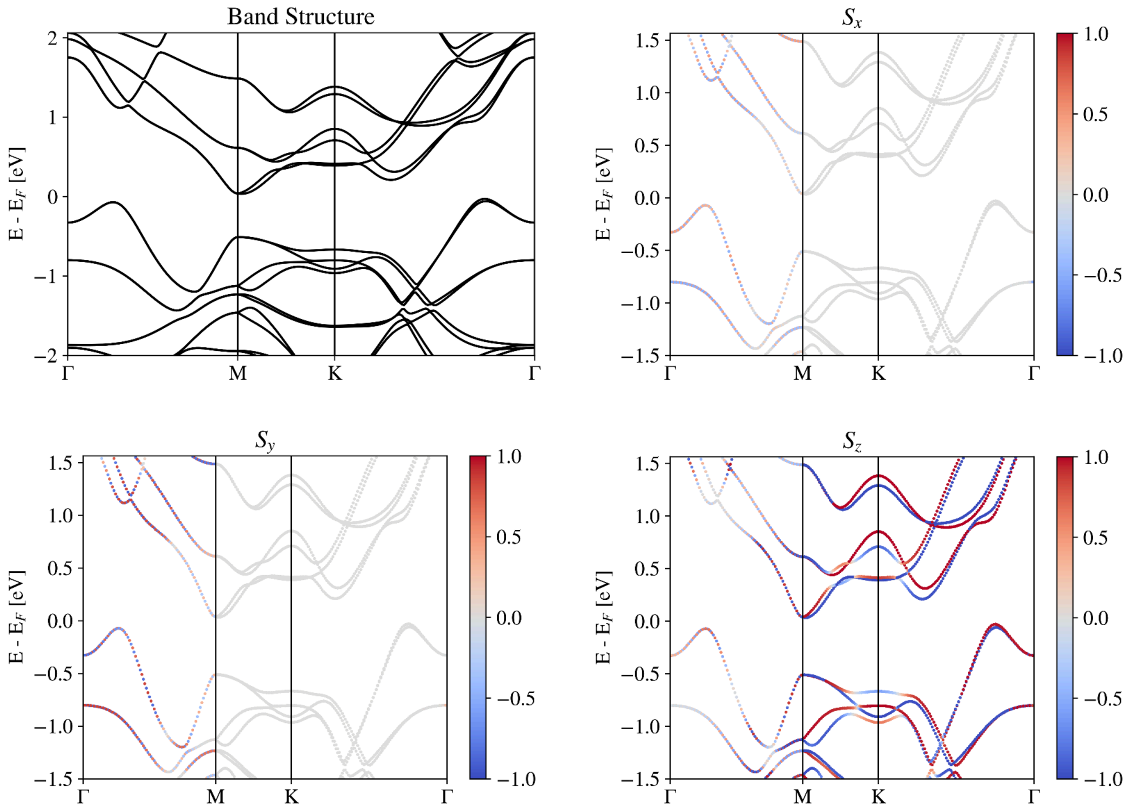


Figure 631: Band structure and spin polarization projections.

## 2.316 S2Sc2-e9d256b367c7

- **Formula**: S2Sc2
- **Structural Cluster**: AB-5
- **Band gap (PBE)**: 0.384 eV
- **Energy above convex hull (C2DB)**: 0.627 eV
- **Space group symbol**: $P\bar{6}m2$
- **Space group number**: 187
- **Polar structure**: False

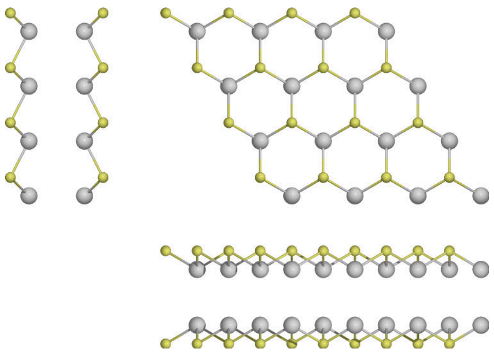

Figure 632: Structure representation

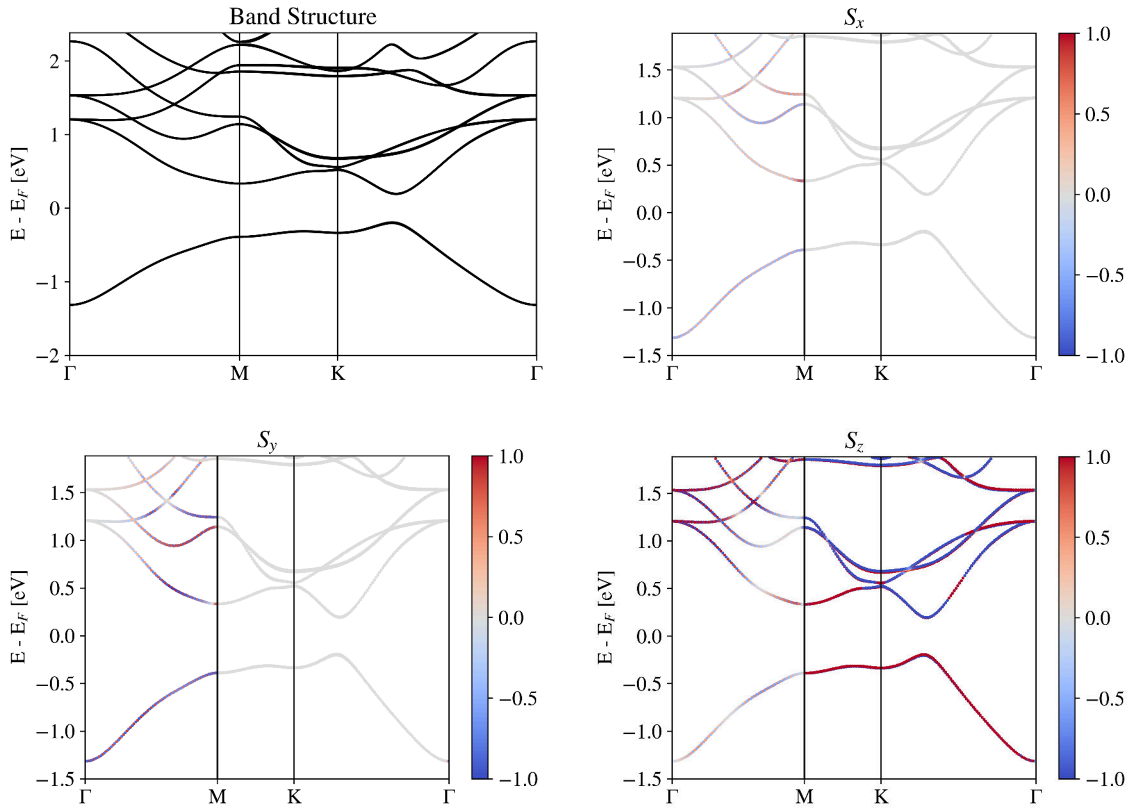


Figure 633: Band structure and spin polarization projections.

## 2.317 S2Si2-0726c763a59a

- **Formula**: S2Si2
- **Structural Cluster**: AB-5
- **Band gap (PBE)**: 1.433 eV
- **Energy above convex hull (C2DB)**: 0.446 eV
- **Space group symbol**: $Pmn2_1$
- **Space group number**: 31
- **Polar structure**: True

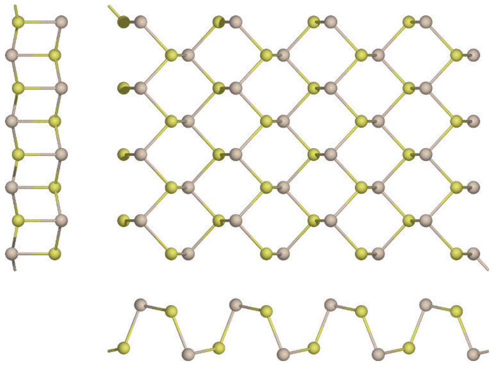

Figure 634: Structure representation

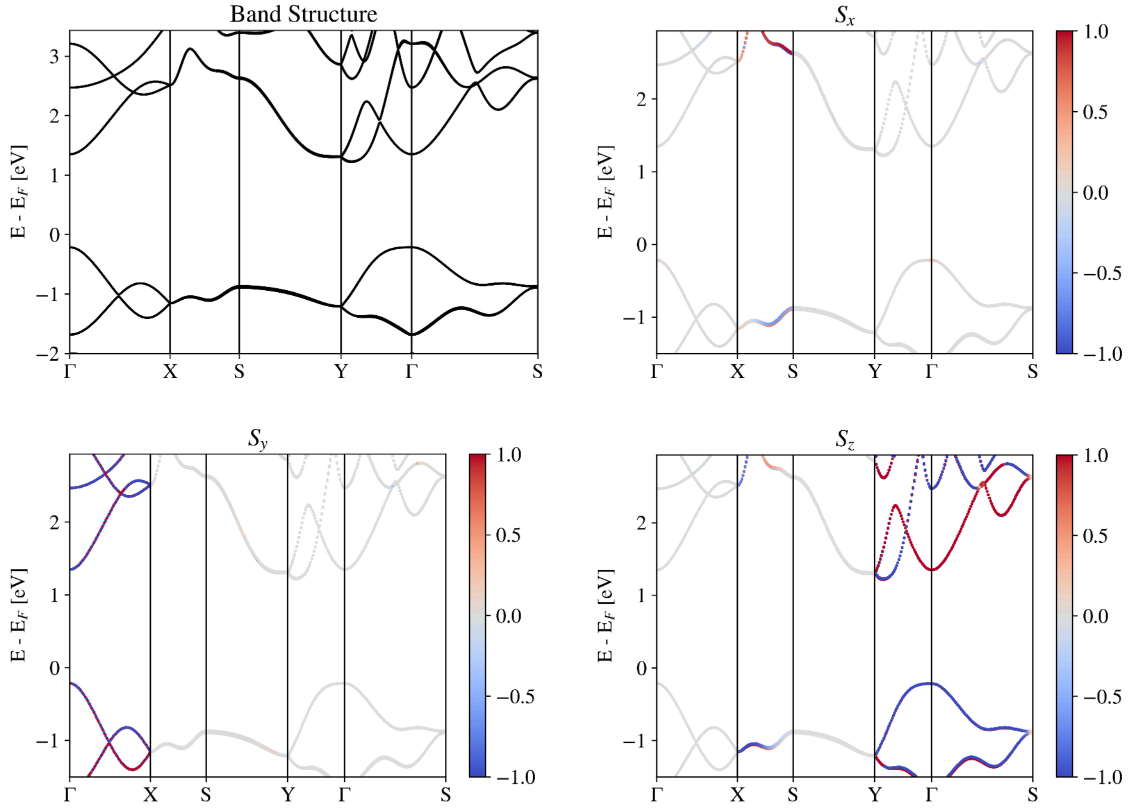


Figure 635: Band structure and spin polarization projections.

## 2.318 S2Sn2-7a8373382b33

- **Formula**: S2Sn2
- **Structural Cluster**: AB-0
- **Band gap (PBE)**: 1.434 eV
- **Energy above convex hull (C2DB)**: 0.043 eV
- **Space group symbol**: $Pmn2_1$
- **Space group number**: 31
- **Polar structure**: True

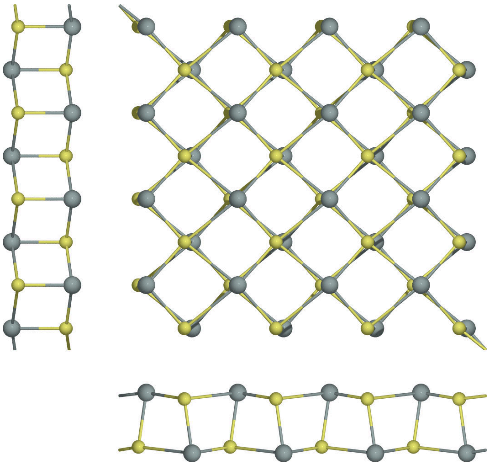

Figure 636: Structure representation

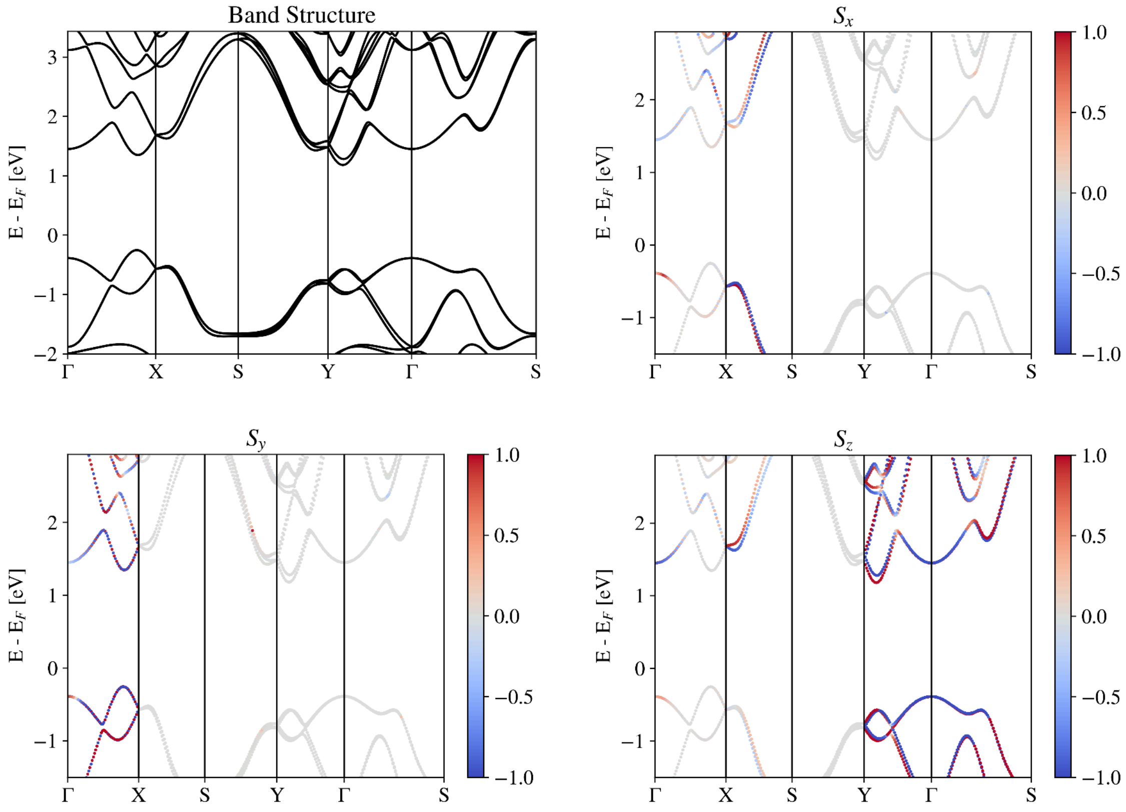


Figure 637: Band structure and spin polarization projections.

## 2.319 S2Sn2-7edb67bf350a

- **Formula**: S2Sn2
- **Structural Cluster**: AB-5
- **Band gap (PBE)**: 1.947 eV
- **Energy above convex hull (C2DB)**: 0.116 eV
- **Space group symbol**: $C2$
- **Space group number**: 5
- **Polar structure**: True

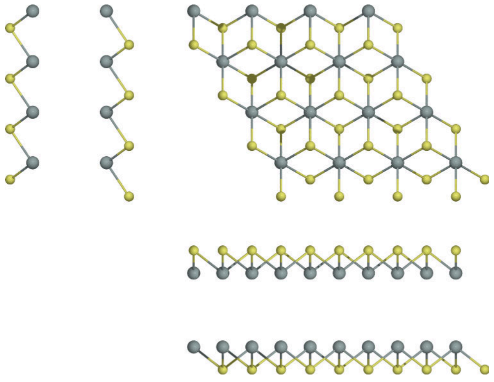

Figure 638: Structure representation

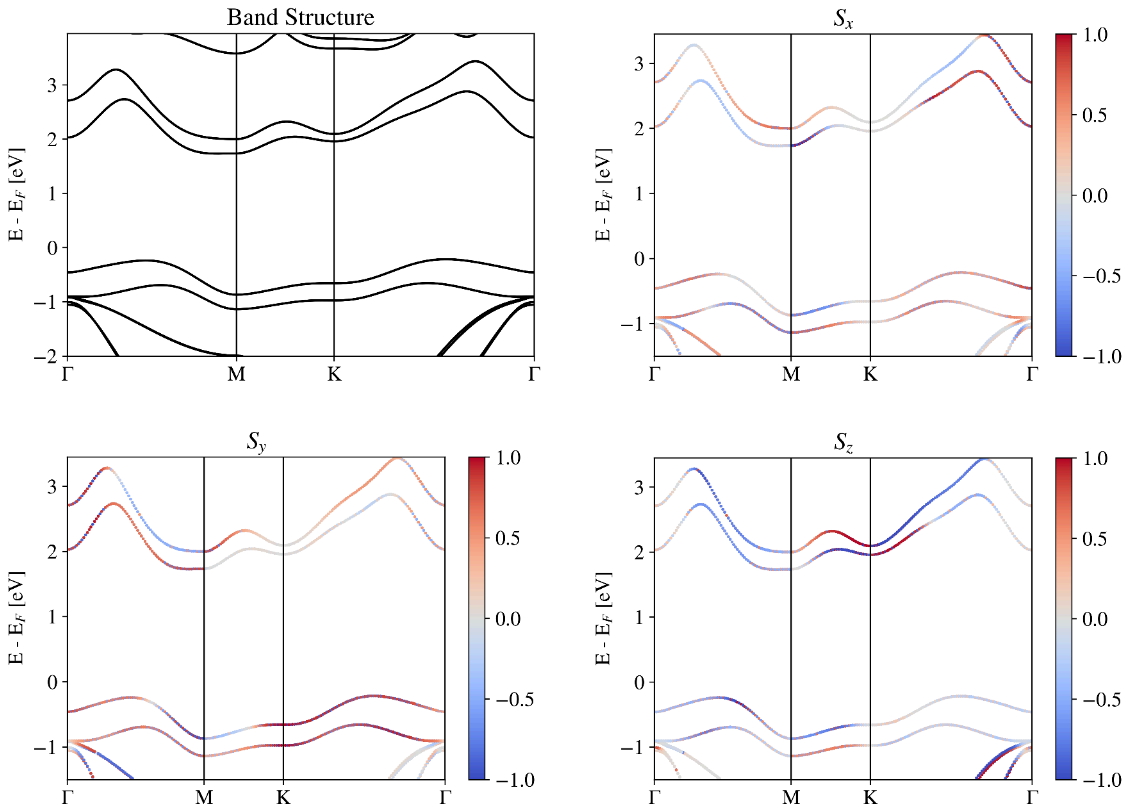


Figure 639: Band structure and spin polarization projections.

## 2.320 S2Tl2-751e767bff79

- **Formula**: S2Tl2
- **Structural Cluster**: AB-5
- **Band gap (PBE)**: 0.666 eV
- **Energy above convex hull (C2DB)**: 0.119 eV
- **Space group symbol**: $P\bar{6}m2$
- **Space group number**: 187
- **Polar structure**: False

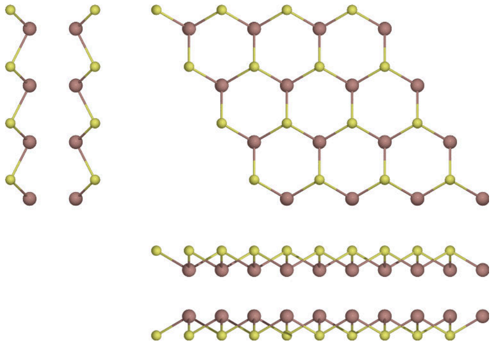

Figure 640: Structure representation

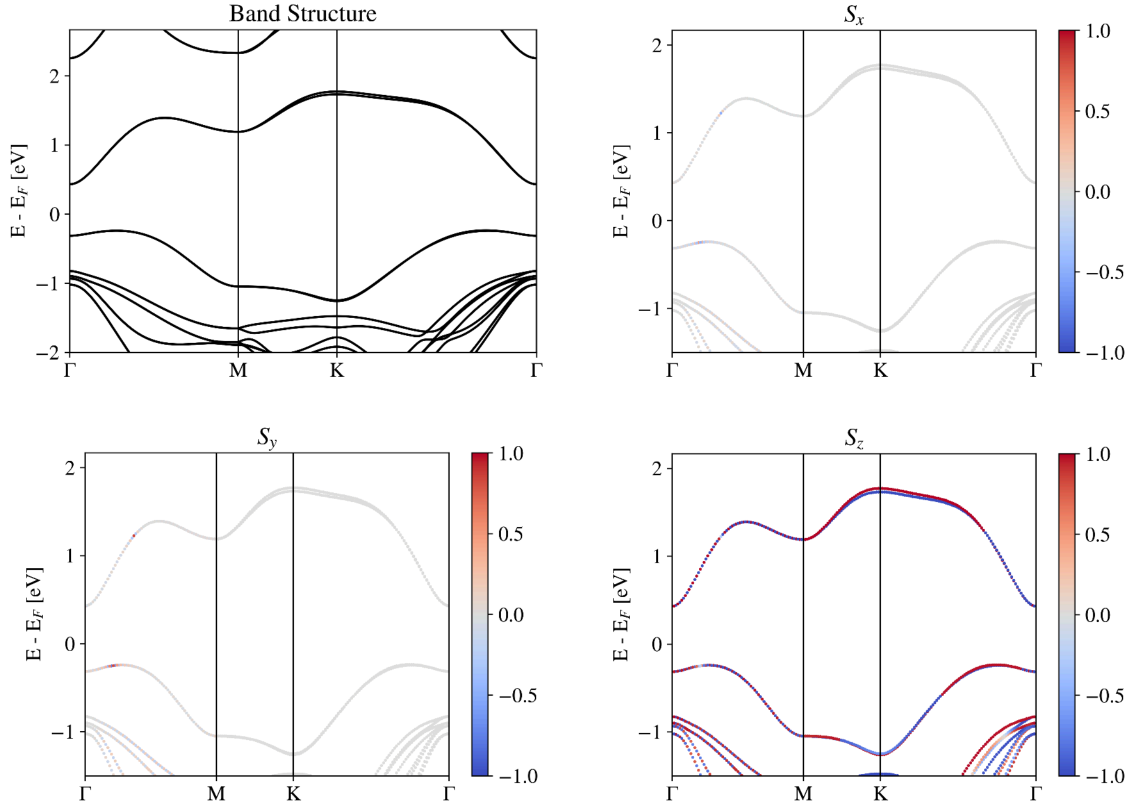


Figure 641: Band structure and spin polarization projections.

## 2.321 S2Tl2-fb0b06c4bab6

- **Formula**: S2Tl2
- **Structural Cluster**: AB-5
- **Band gap (PBE)**: 0.62 eV
- **Energy above convex hull (C2DB)**: 0.12 eV
- **Space group symbol**: $C2$
- **Space group number**: 5
- **Polar structure**: True

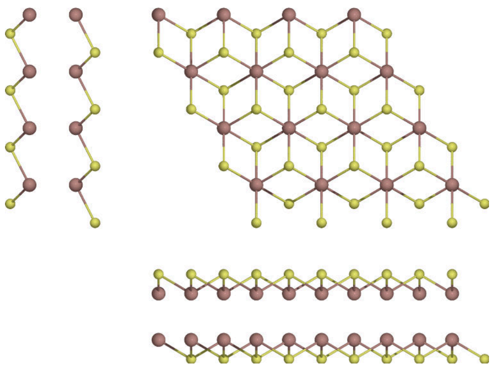

Figure 642: Structure representation

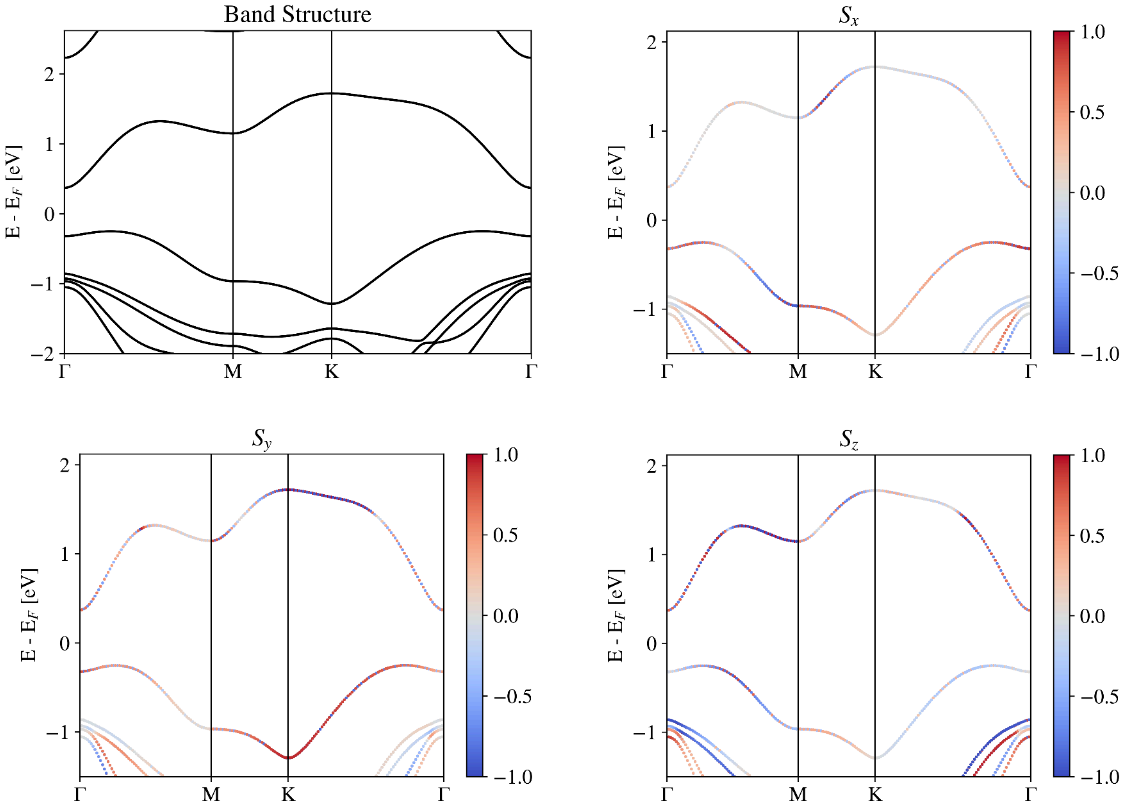


Figure 643: Band structure and spin polarization projections.

## 2.322 S2V2-605c732d5111

- **Formula**: S2V2
- **Structural Cluster**: AB-5
- **Band gap (PBE)**: 0.209 eV
- **Energy above convex hull (C2DB)**: 0.534 eV
- **Space group symbol**: $P\bar{6}m2$
- **Space group number**: 187
- **Polar structure**: False

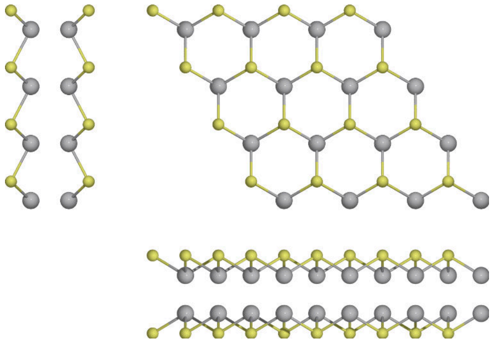

Figure 644: Structure representation

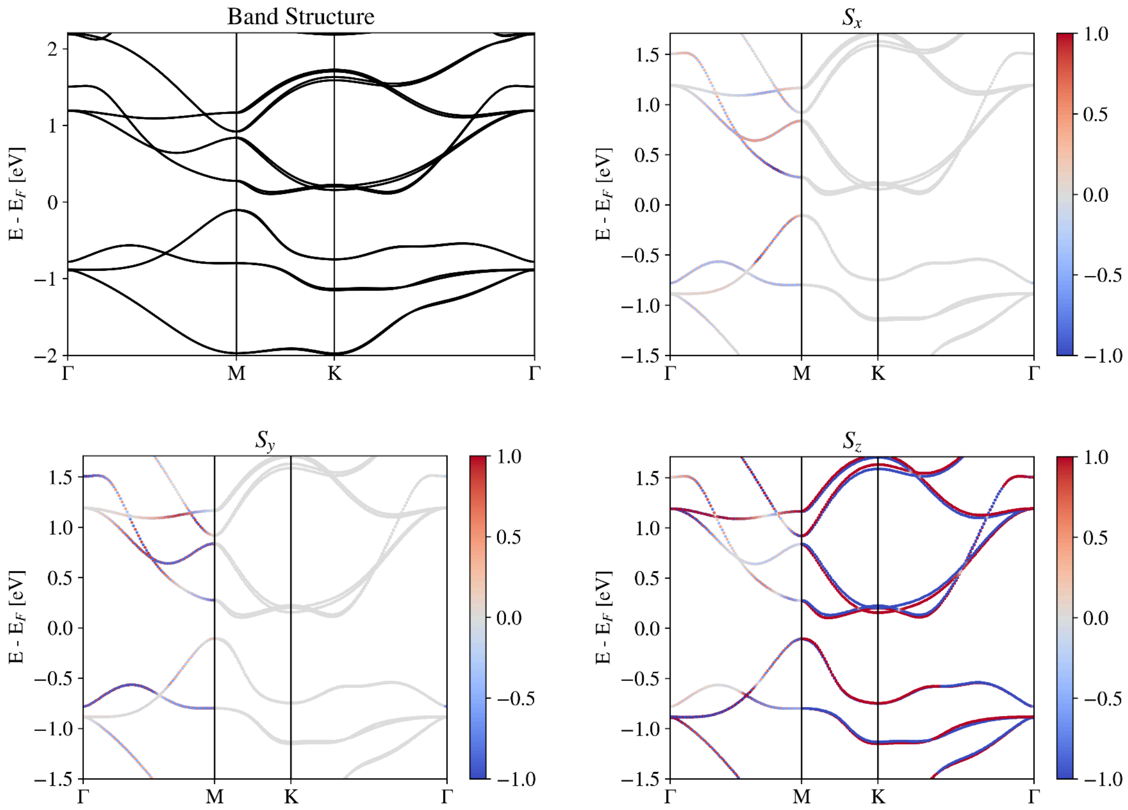


Figure 645: Band structure and spin polarization projections.

## 2.323 SSeTi-358305cad463

- **Formula**: SSeTi
- **Structural Cluster**: ABC-4
- **Band gap (PBE)**: 0.501 eV
- **Energy above convex hull (C2DB)**: 0.128 eV
- **Space group symbol**: $P3m1$
- **Space group number**: 156
- **Polar structure**: True

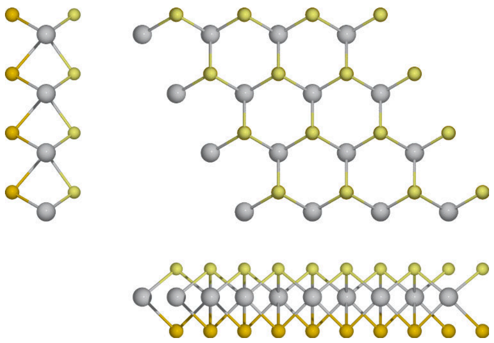

Figure 646: Structure representation

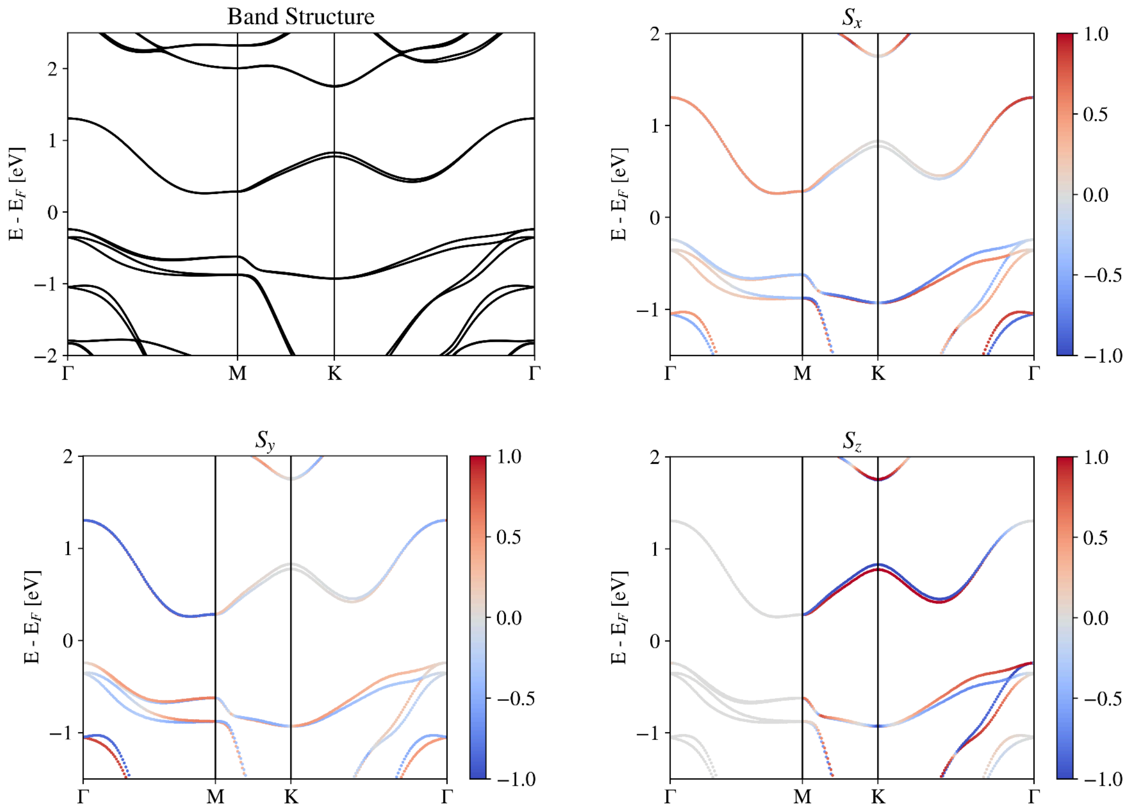


Figure 647: Band structure and spin polarization projections.

## 2.324 SSeW-001e03f2c095

- **Formula**: SSeW
- **Structural Cluster**: ABC-4
- **Band gap (PBE)**: 1.418 eV
- **Energy above convex hull (C2DB)**: 0.01 eV
- **Space group symbol**: $P3m1$
- **Space group number**: 156
- **Polar structure**: True

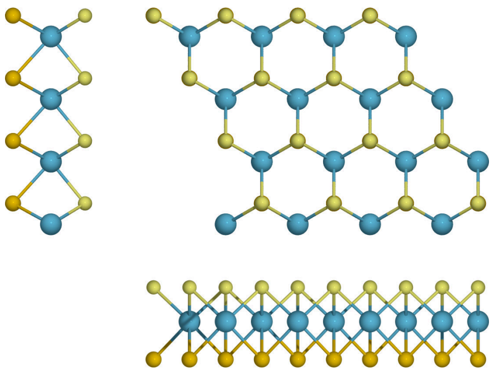

Figure 648: Structure representation

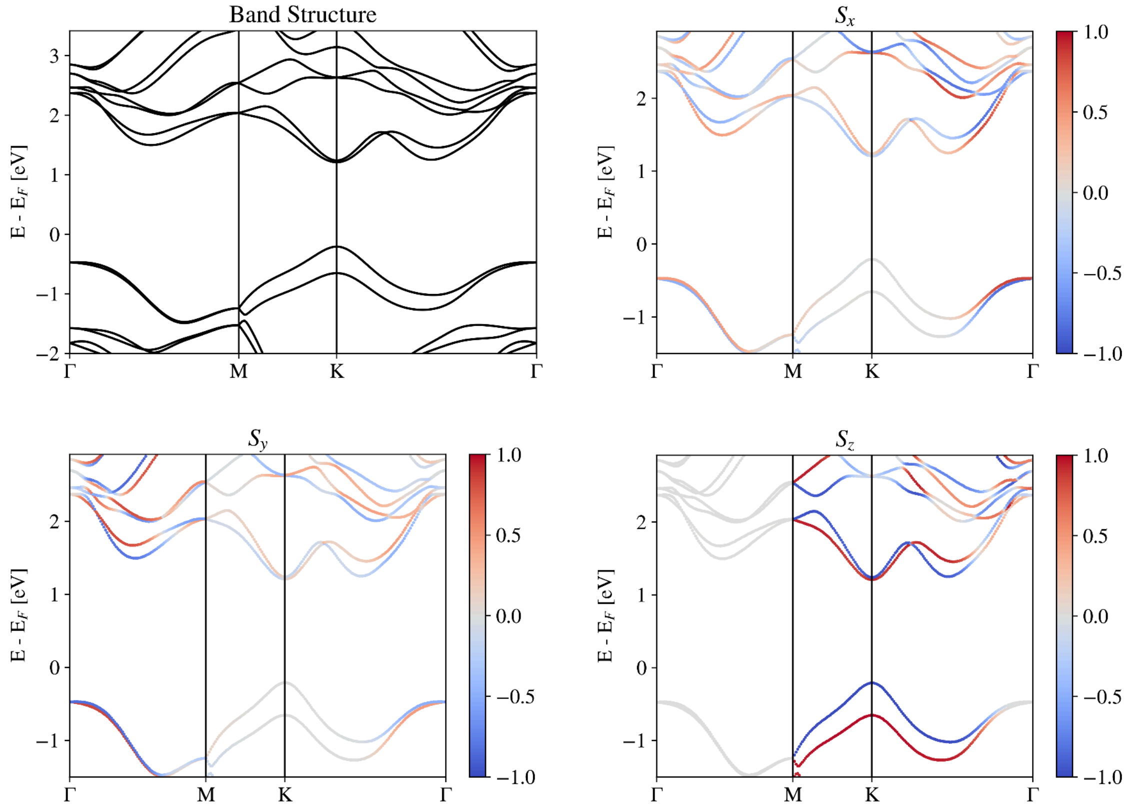


Figure 649: Band structure and spin polarization projections.

## 2.325 SSeZr-1a9901838600

- **Formula**: SSeZr
- **Structural Cluster**: ABC-3
- **Band gap (PBE)**: 0.616 eV
- **Energy above convex hull (C2DB)**: 0.0 eV
- **Space group symbol**: $P3m1$
- **Space group number**: 156
- **Polar structure**: True

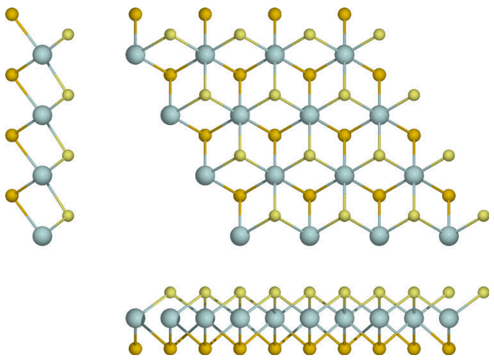

Figure 650: Structure representation

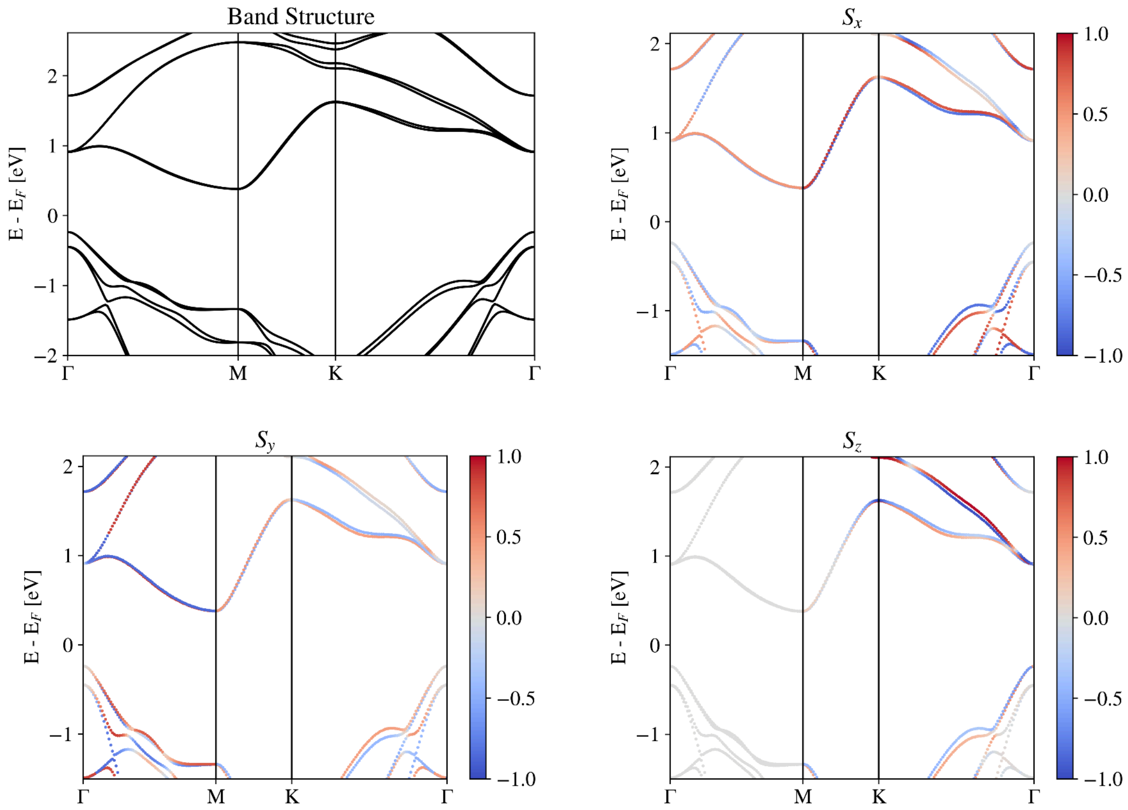


Figure 651: Band structure and spin polarization projections.

## 2.326 SSeZr-2be14f373da0

- **Formula**: SSeZr
- **Structural Cluster**: ABC-4
- **Band gap (PBE)**: 0.831 eV
- **Energy above convex hull (C2DB)**: 0.163 eV
- **Space group symbol**: $P3m1$
- **Space group number**: 156
- **Polar structure**: True

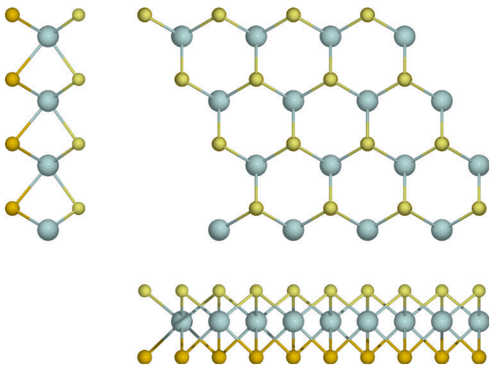

Figure 652: Structure representation

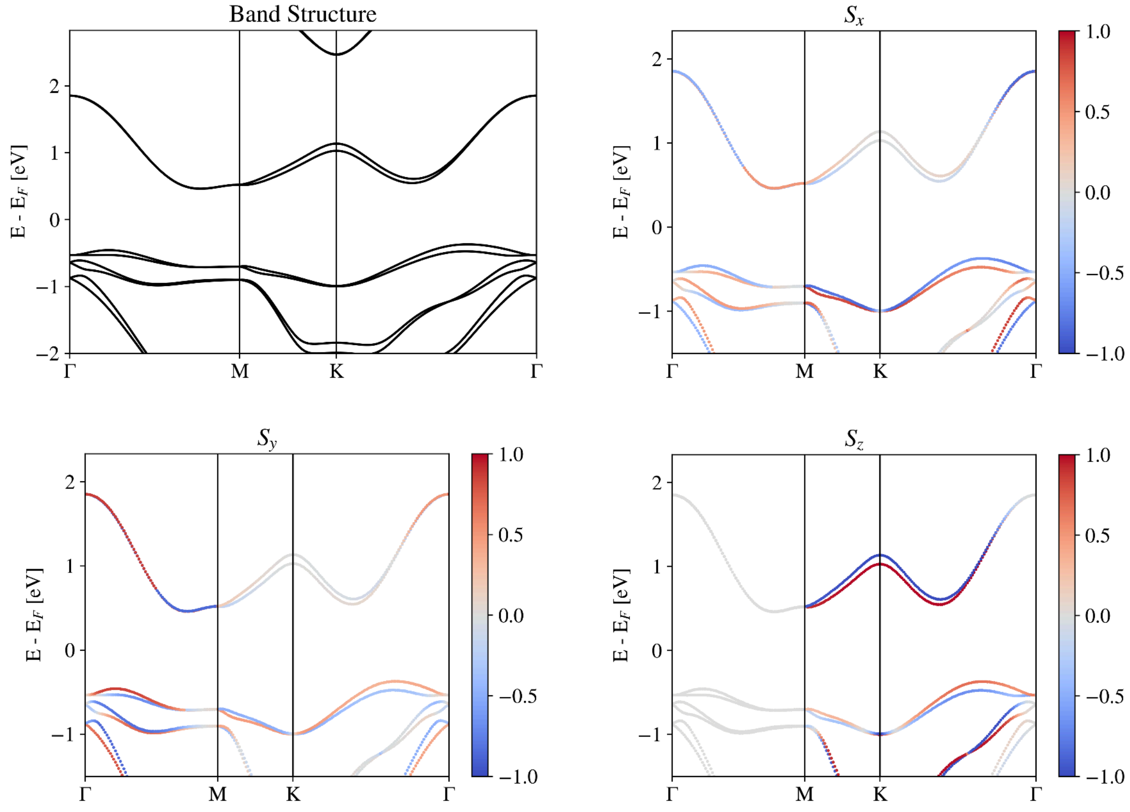


Figure 653: Band structure and spin polarization projections.

## 2.327 SSn-f98da23471a1

- **Formula**: SSn
- **Structural Cluster**: AB-25
- **Band gap (PBE)**: 2.3 eV
- **Energy above convex hull (C2DB)**: 0.118 eV
- **Space group symbol**: $P3m1$
- **Space group number**: 156
- **Polar structure**: True

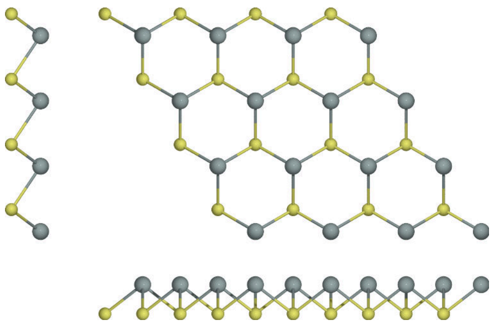

Figure 654: Structure representation

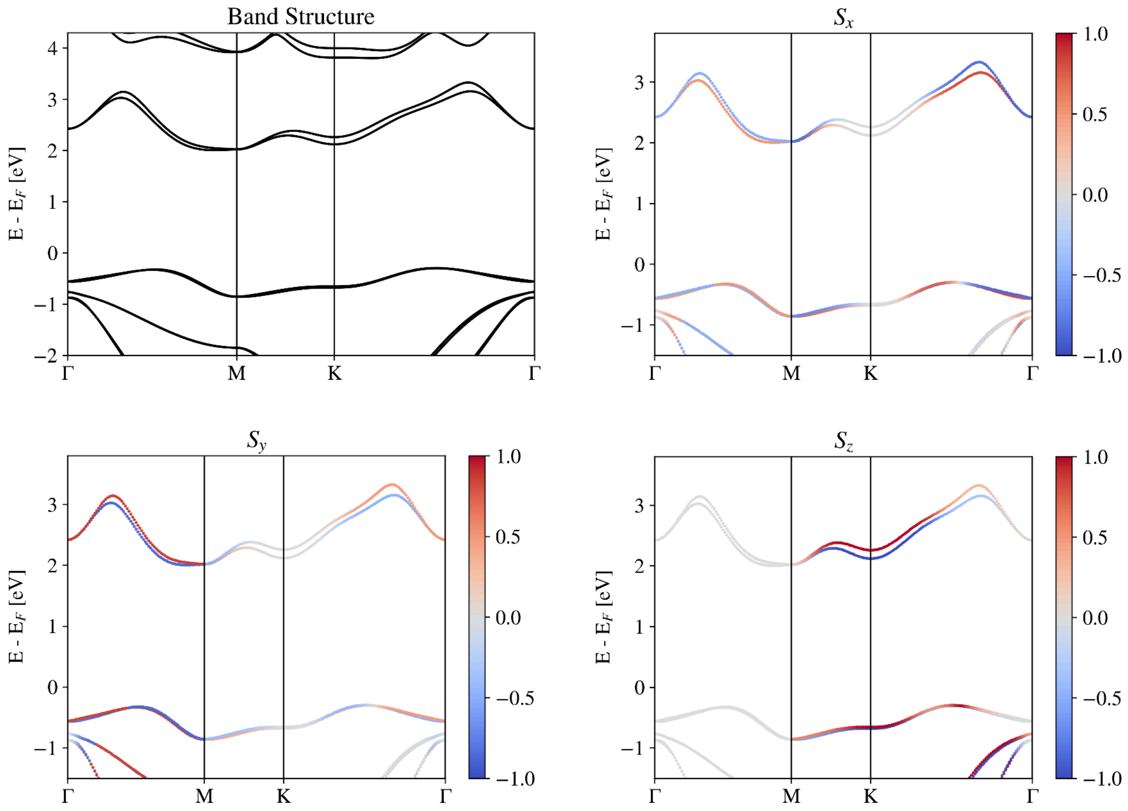


Figure 655: Band structure and spin polarization projections.

## 2.328 STeW-75ee10091f43

- **Formula**: STeW
- **Structural Cluster**: ABC-4
- **Band gap (PBE)**: 1.168 eV
- **Energy above convex hull (C2DB)**: 0.086 eV
- **Space group symbol**: $P3m1$
- **Space group number**: 156
- **Polar structure**: True

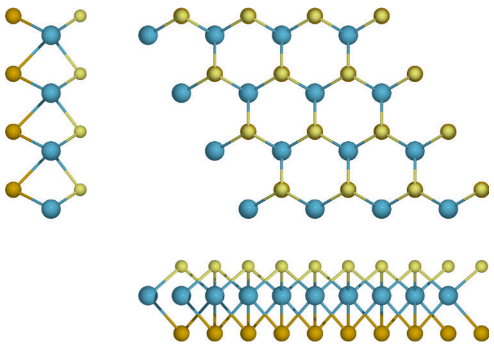

Figure 656: Structure representation

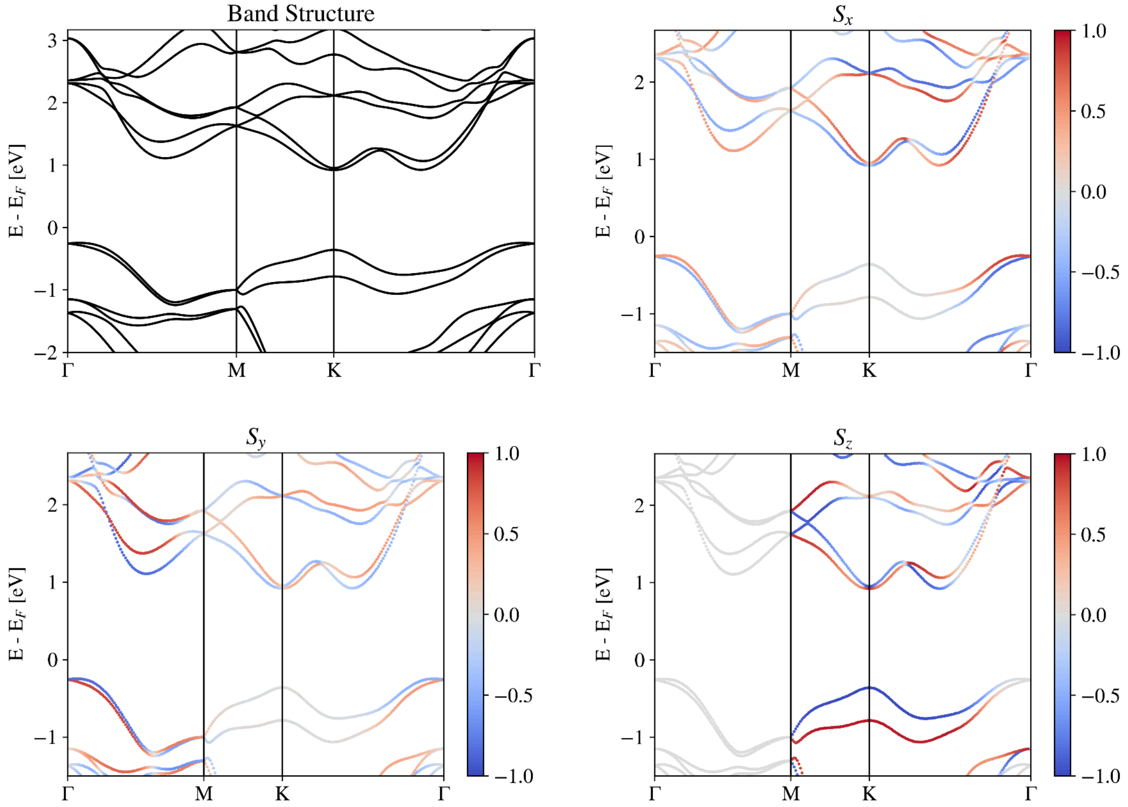


Figure 657: Band structure and spin polarization projections.

## 2.329 STeW-916afba26723

- **Formula**: STeW
- **Structural Cluster**: ABC-4
- **Band gap (PBE)**: 0.191 eV
- **Energy above convex hull (C2DB)**: 0.266 eV
- **Space group symbol**: $P3m1$
- **Space group number**: 156
- **Polar structure**: True

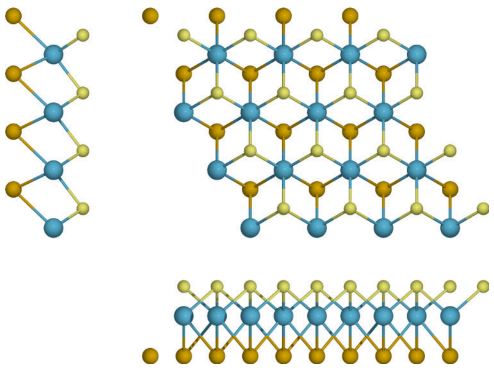

Figure 658: Structure representation

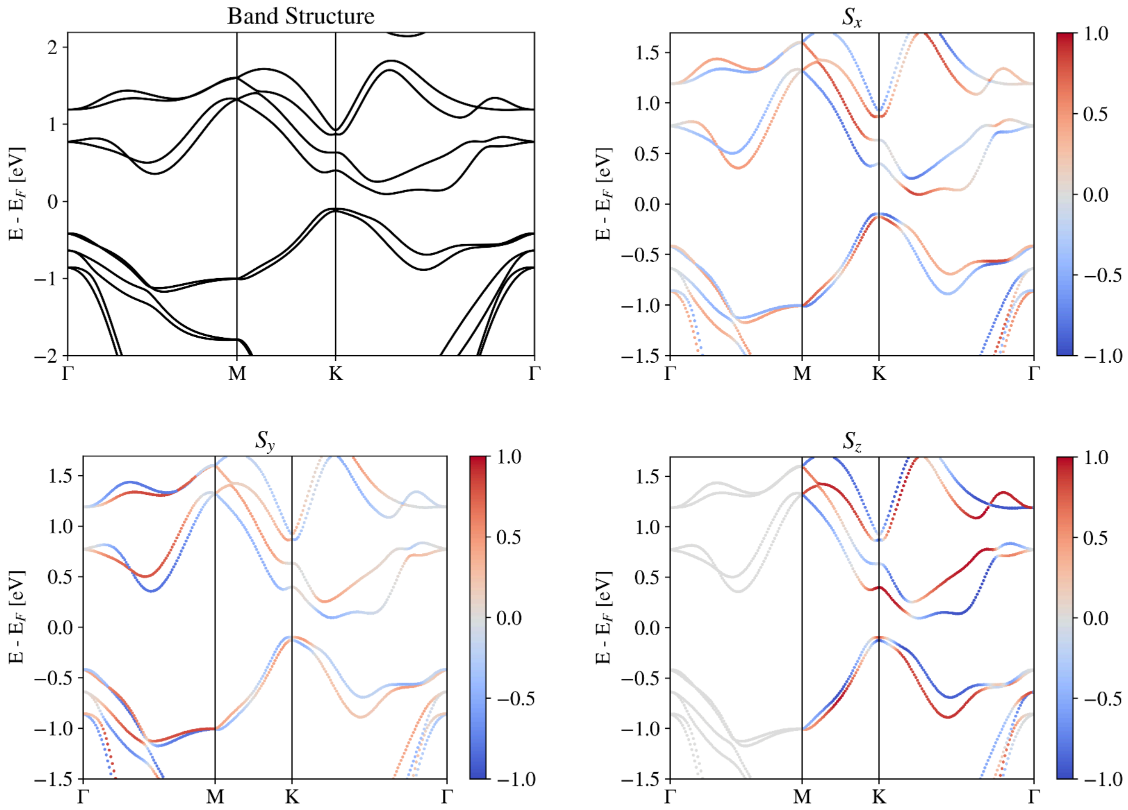


Figure 659: Band structure and spin polarization projections.

## 2.330 STeZr-3f3c7bc0ce7d

- **Formula**: STeZr
- **Structural Cluster**: ABC-4
- **Band gap (PBE)**: 0.218 eV
- **Energy above convex hull (C2DB)**: 0.122 eV
- **Space group symbol**: $P3m1$
- **Space group number**: 156
- **Polar structure**: True

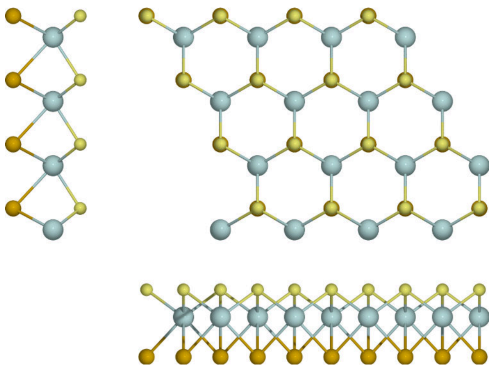

Figure 660: Structure representation

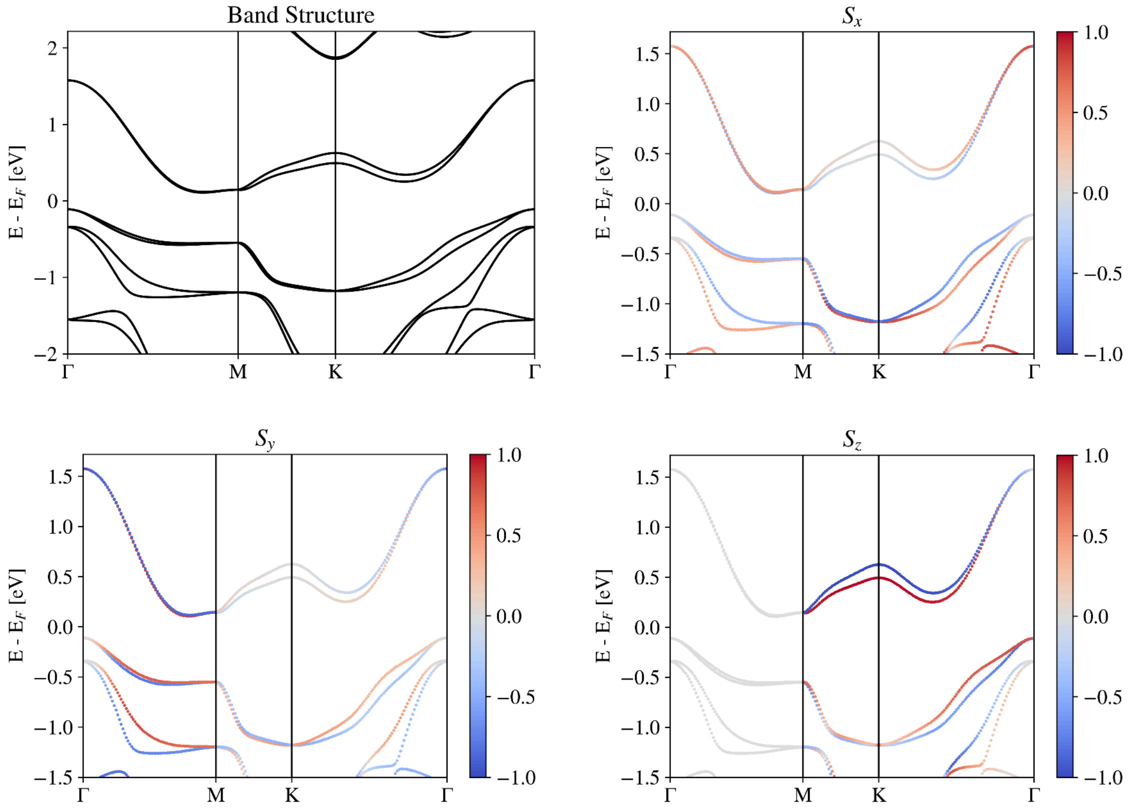


Figure 661: Band structure and spin polarization projections.

## 2.331 Sc2Br6-a43351c43cd3

- **Formula**: Sc2Br6
- **Structural Cluster**: AB3-19
- **Band gap (PBE)**: 2.646 eV
- **Energy above convex hull (C2DB)**: 0.082 eV
- **Space group symbol**: $P\bar{6}2m$
- **Space group number**: 189
- **Polar structure**: False

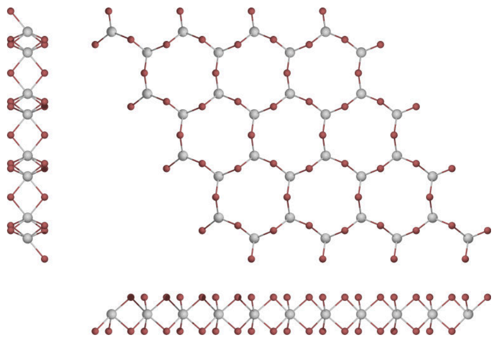

Figure 662: Structure representation

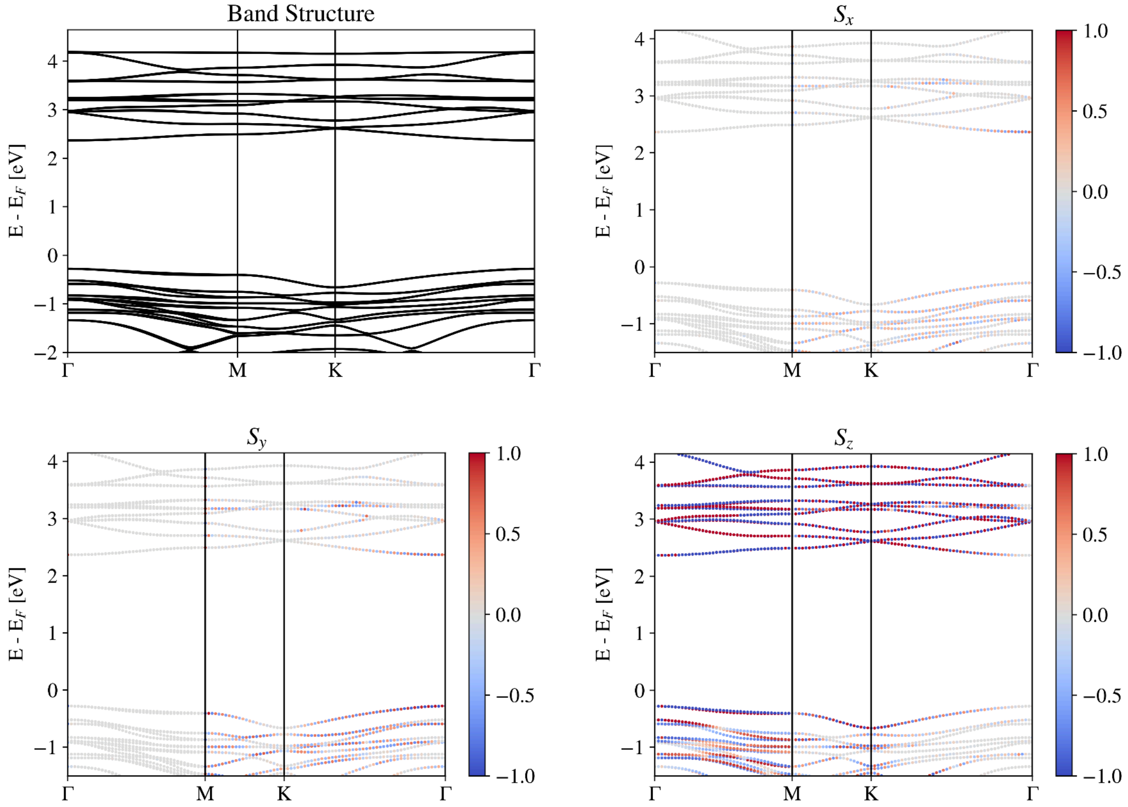


Figure 663: Band structure and spin polarization projections.

## 2.332 Sc2Cl6-3eef25de07b3

- **Formula**: Sc2Cl6
- **Structural Cluster**: AB3-19
- **Band gap (PBE)**: 3.238 eV
- **Energy above convex hull (C2DB)**: 0.072 eV
- **Space group symbol**: $P\bar{6}2m$
- **Space group number**: 189
- **Polar structure**: False

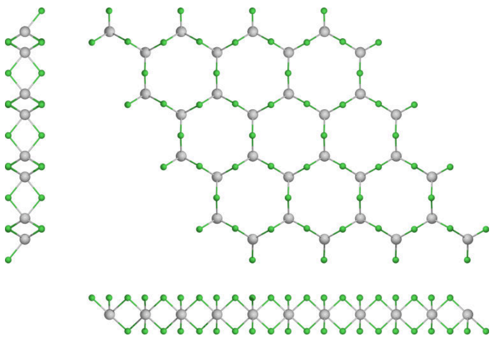

Figure 664: Structure representation

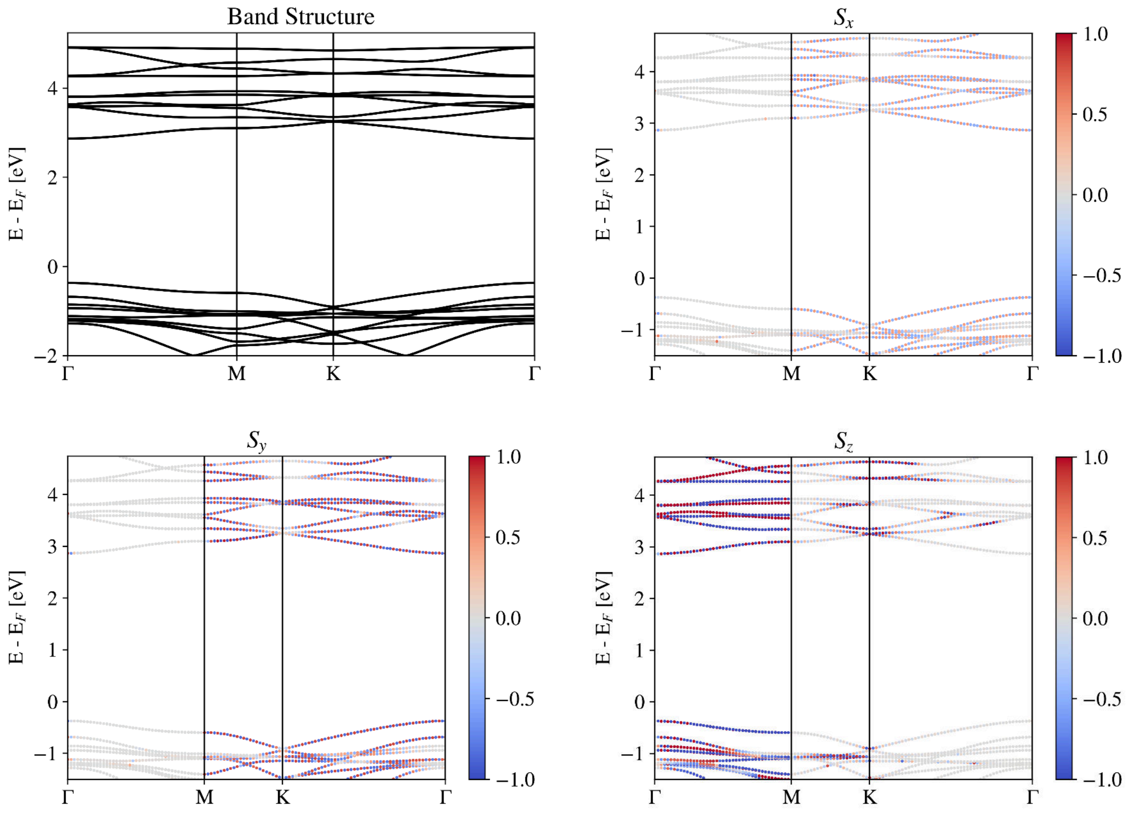


Figure 665: Band structure and spin polarization projections.

## 2.333 Sc2I6-d59c550c4a3a

- **Formula**: Sc2I6
- **Structural Cluster**: AB3-19
- **Band gap (PBE)**: 1.923 eV
- **Energy above convex hull (C2DB)**: 0.092 eV
- **Space group symbol**: $P\bar{6}2m$
- **Space group number**: 189
- **Polar structure**: False

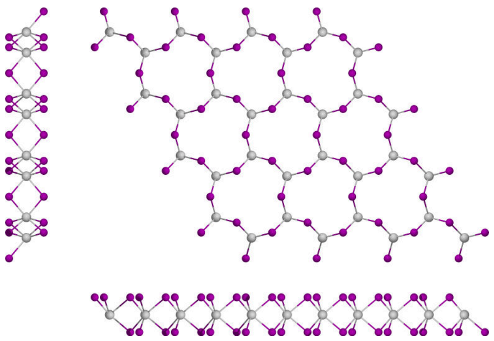

Figure 666: Structure representation

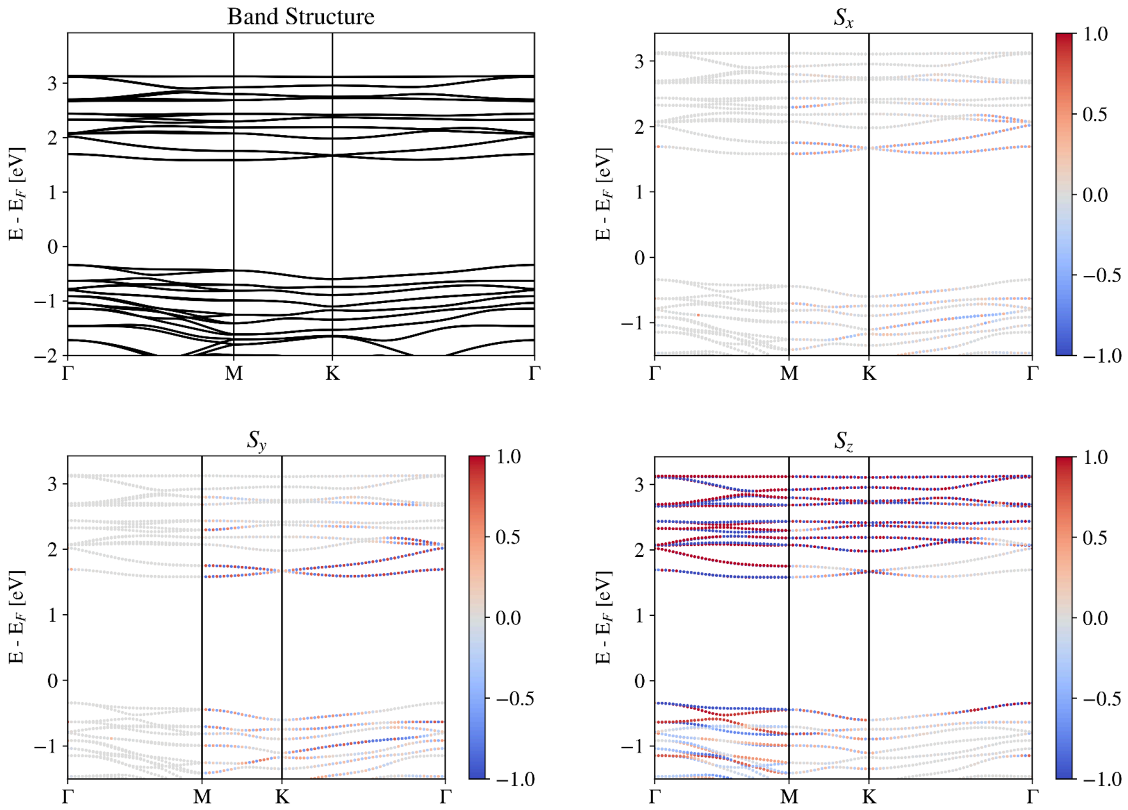


Figure 667: Band structure and spin polarization projections.

## 2.334 Sc2Se2-9fb15588e4d4

- **Formula**: Sc2Se2
- **Structural Cluster**: AB-5
- **Band gap (PBE)**: 0.373 eV
- **Energy above convex hull (C2DB)**: 0.548 eV
- **Space group symbol**: $P\bar{6}m2$
- **Space group number**: 187
- **Polar structure**: False

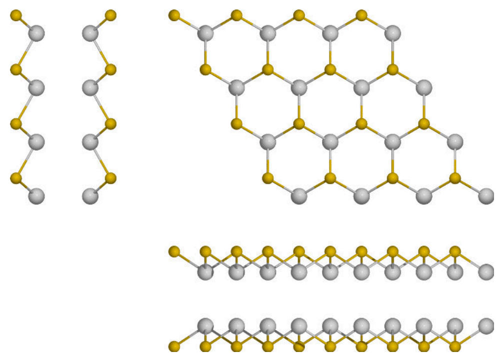

Figure 668: Structure representation

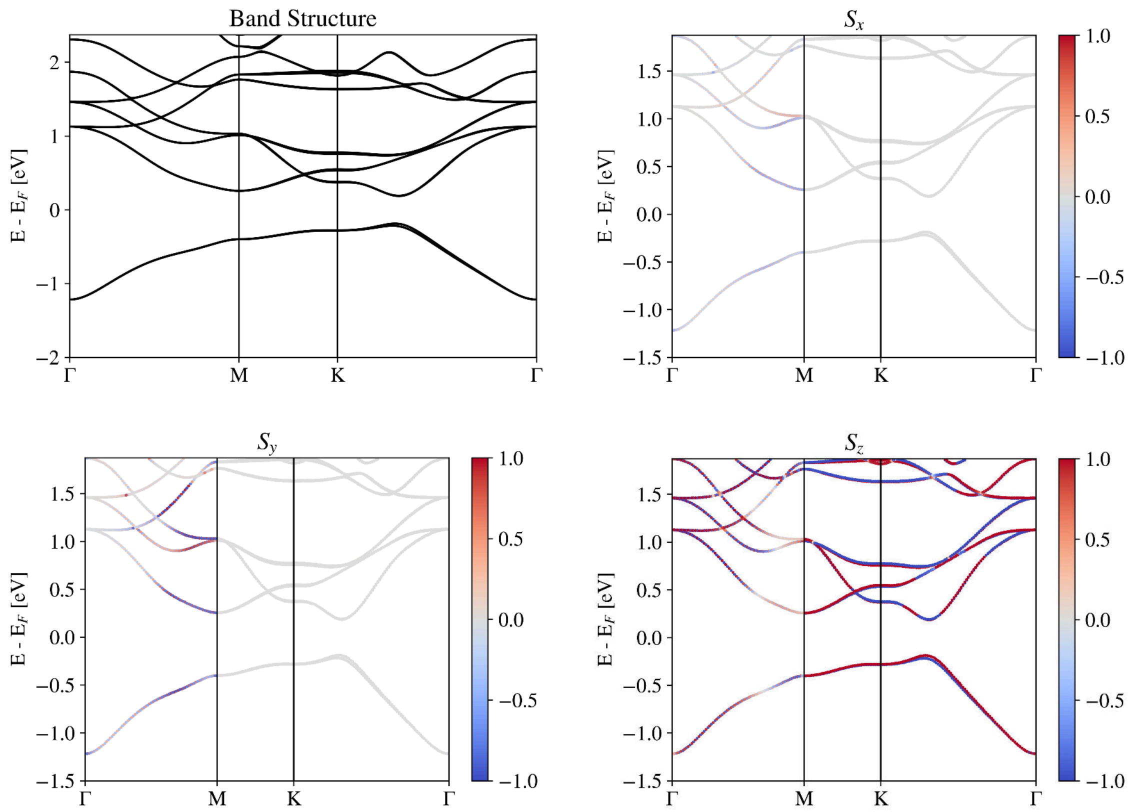


Figure 669: Band structure and spin polarization projections.

## 2.335 Sc2Te2-c3cac8e74dc1

- **Formula**: Sc2Te2
- **Structural Cluster**: AB-5
- **Band gap (PBE)**: 0.288 eV
- **Energy above convex hull (C2DB)**: 0.614 eV
- **Space group symbol**: $P\bar{6}m2$
- **Space group number**: 187
- **Polar structure**: False

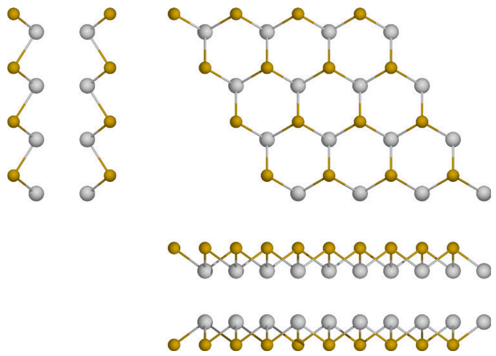

Figure 670: Structure representation

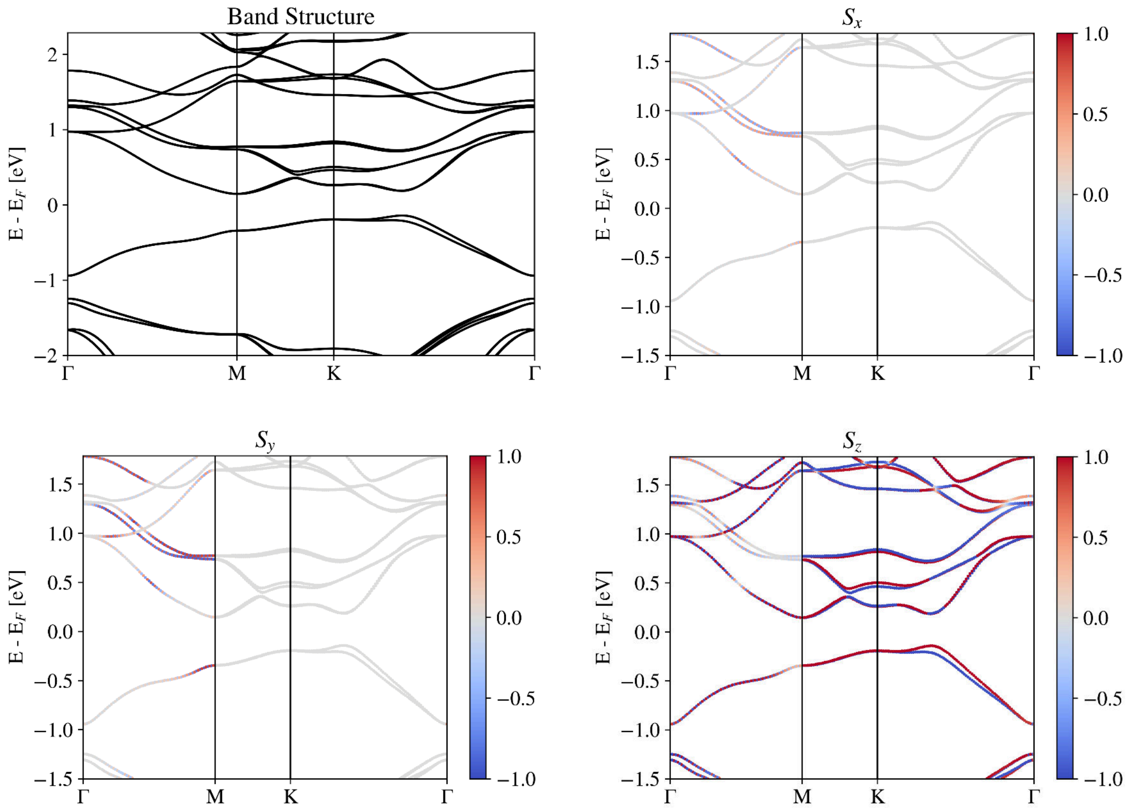


Figure 671: Band structure and spin polarization projections.

## 2.336 Se2Si2-aa0e94e8944e

- **Formula**: Se2Si2
- **Structural Cluster**: AB-5
- **Band gap (PBE)**: 1.201 eV
- **Energy above convex hull (C2DB)**: 0.402 eV
- **Space group symbol**: $Pmn2_1$
- **Space group number**: 31
- **Polar structure**: True

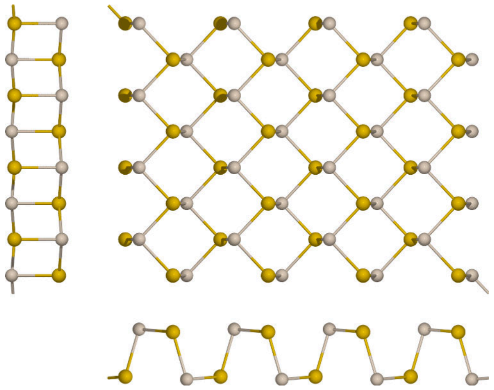

Figure 672: Structure representation

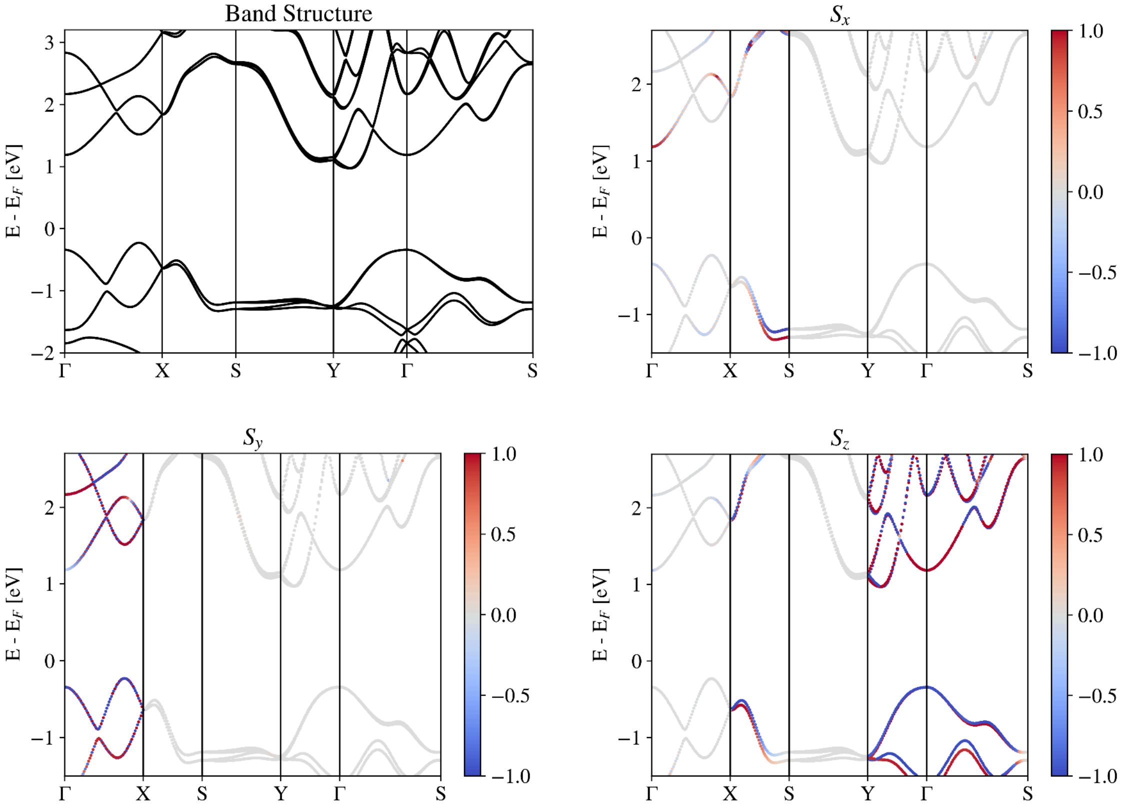


Figure 673: Band structure and spin polarization projections.

## 2.337 Se2Sn2-9b27b6ad7899

- **Formula**: Se2Sn2
- **Structural Cluster**: AB-5
- **Band gap (PBE)**: 1.834 eV
- **Energy above convex hull (C2DB)**: 0.096 eV
- **Space group symbol**: *Cm*
- **Space group number**: 8
- **Polar structure**: True

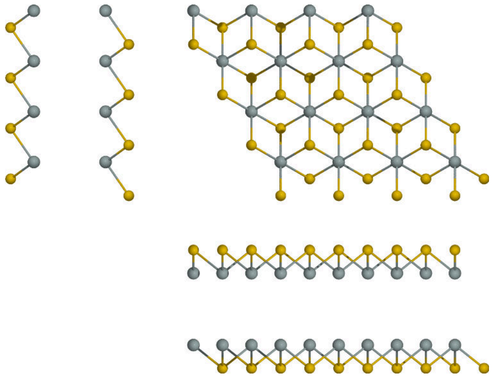

Figure 674: Structure representation

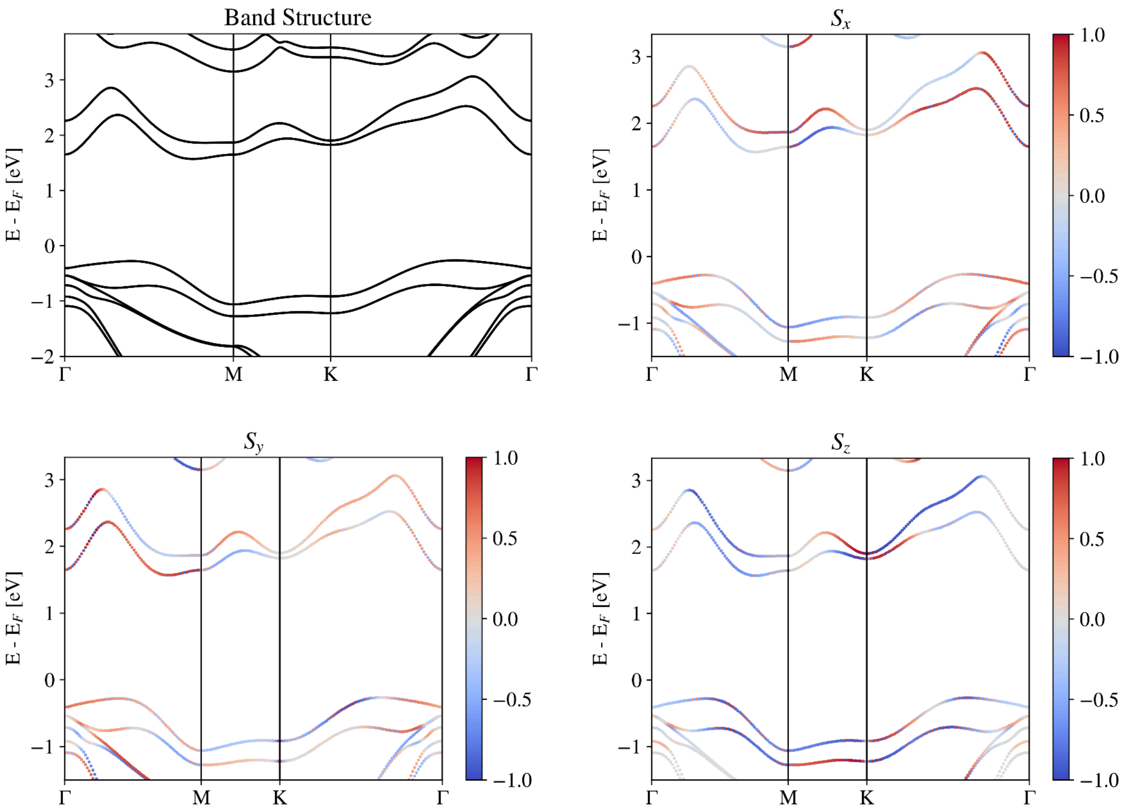


Figure 675: Band structure and spin polarization projections.

## 2.338 Se2Tl2-16490f81af26

- **Formula**: Se2Tl2
- **Structural Cluster**: AB-5
- **Band gap (PBE)**: 0.444 eV
- **Energy above convex hull (C2DB)**: 0.078 eV
- **Space group symbol**: *Cm*
- **Space group number**: 8
- **Polar structure**: True

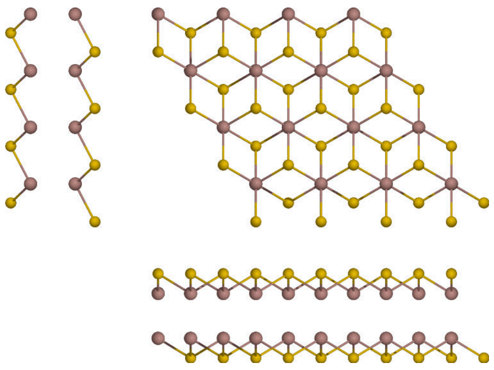

Figure 676: Structure representation

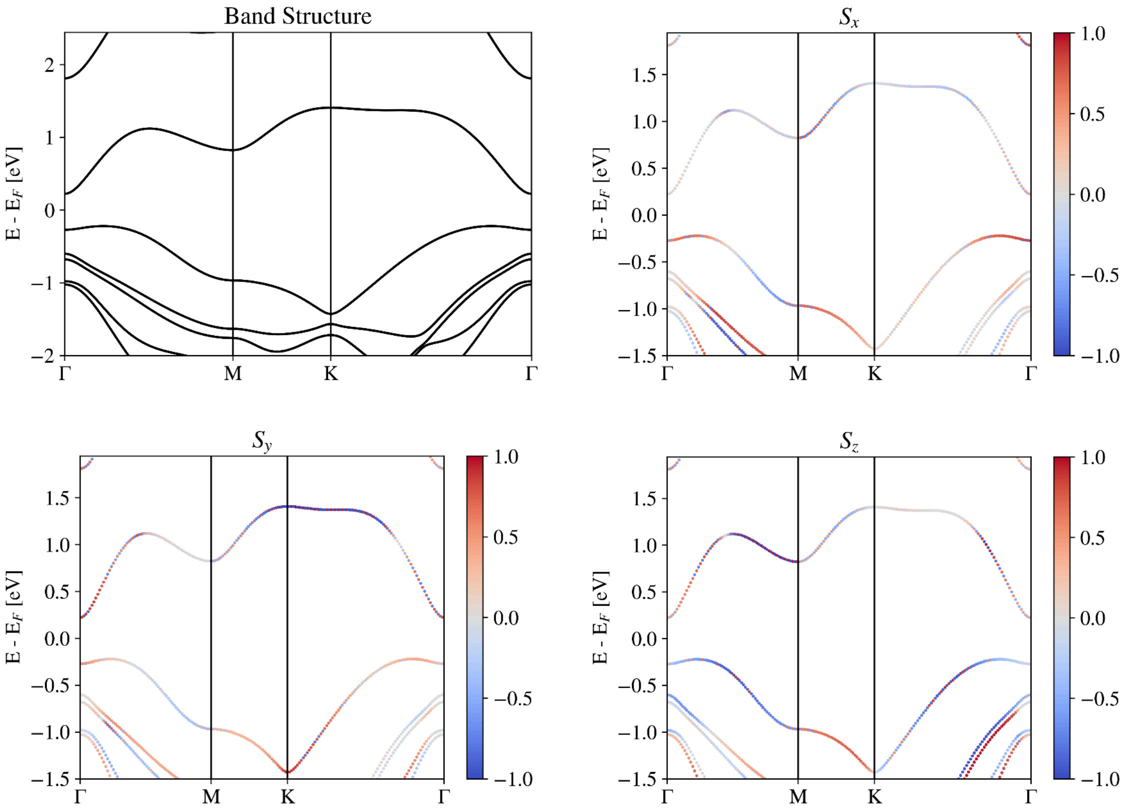


Figure 677: Band structure and spin polarization projections.

## 2.339 Se2Tl2-625697b299d1

- **Formula**: Se2Tl2
- **Structural Cluster**: AB-5
- **Band gap (PBE)**: 0.488 eV
- **Energy above convex hull (C2DB)**: 0.076 eV
- **Space group symbol**: $P\bar{6}m2$
- **Space group number**: 187
- **Polar structure**: False

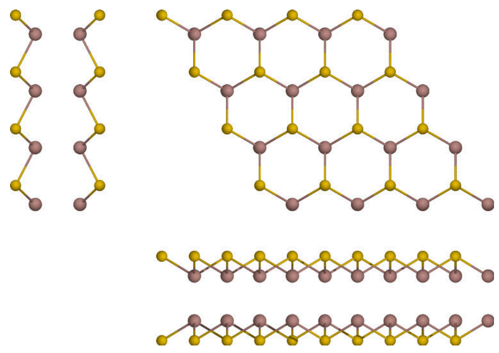

Figure 678: Structure representation

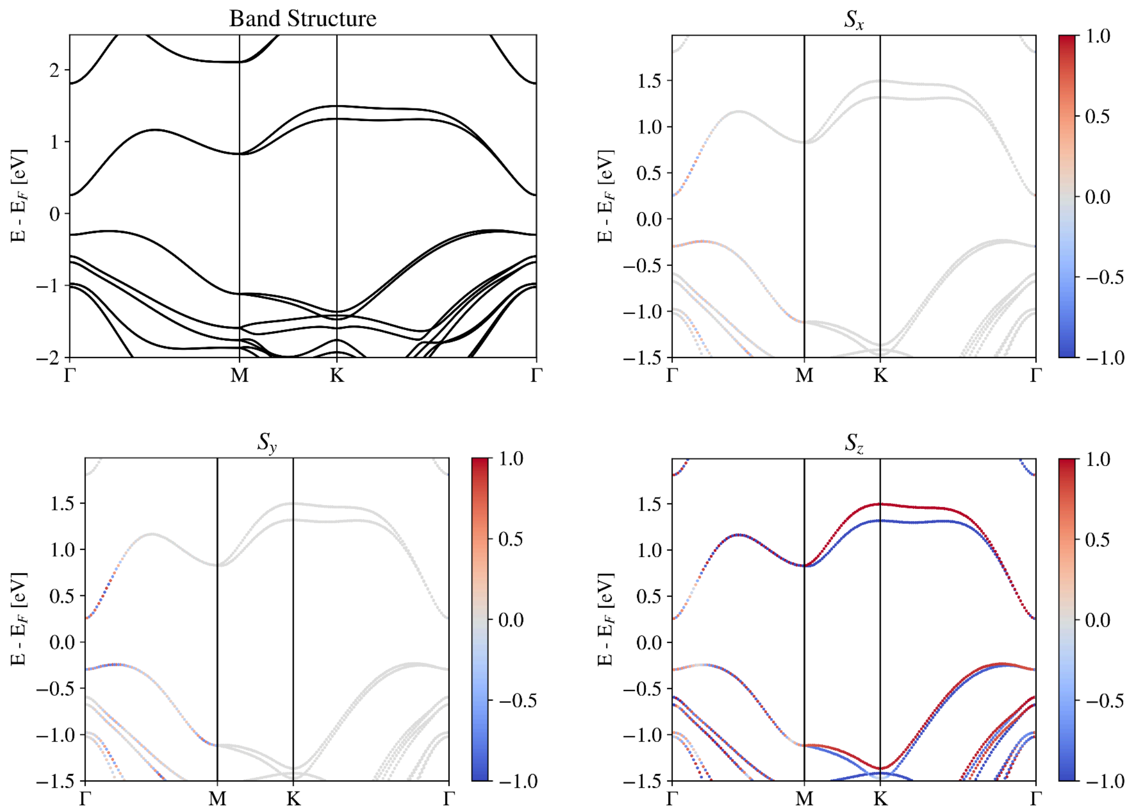


Figure 679: Band structure and spin polarization projections.

## 2.340 Se2V2-9cf30bd127fe

- **Formula**: Se2V2
- **Structural Cluster**: AB-5
- **Band gap (PBE)**: 0.248 eV
- **Energy above convex hull (C2DB)**: 0.42 eV
- **Space group symbol**: $P\bar{6}m2$
- **Space group number**: 187
- **Polar structure**: False

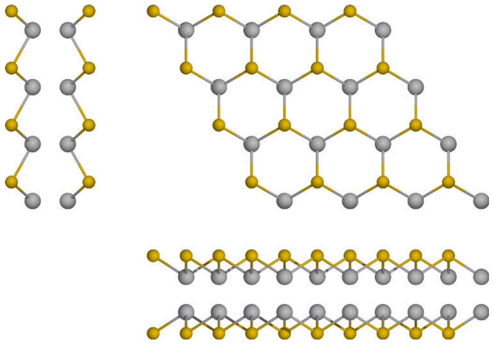

Figure 680: Structure representation

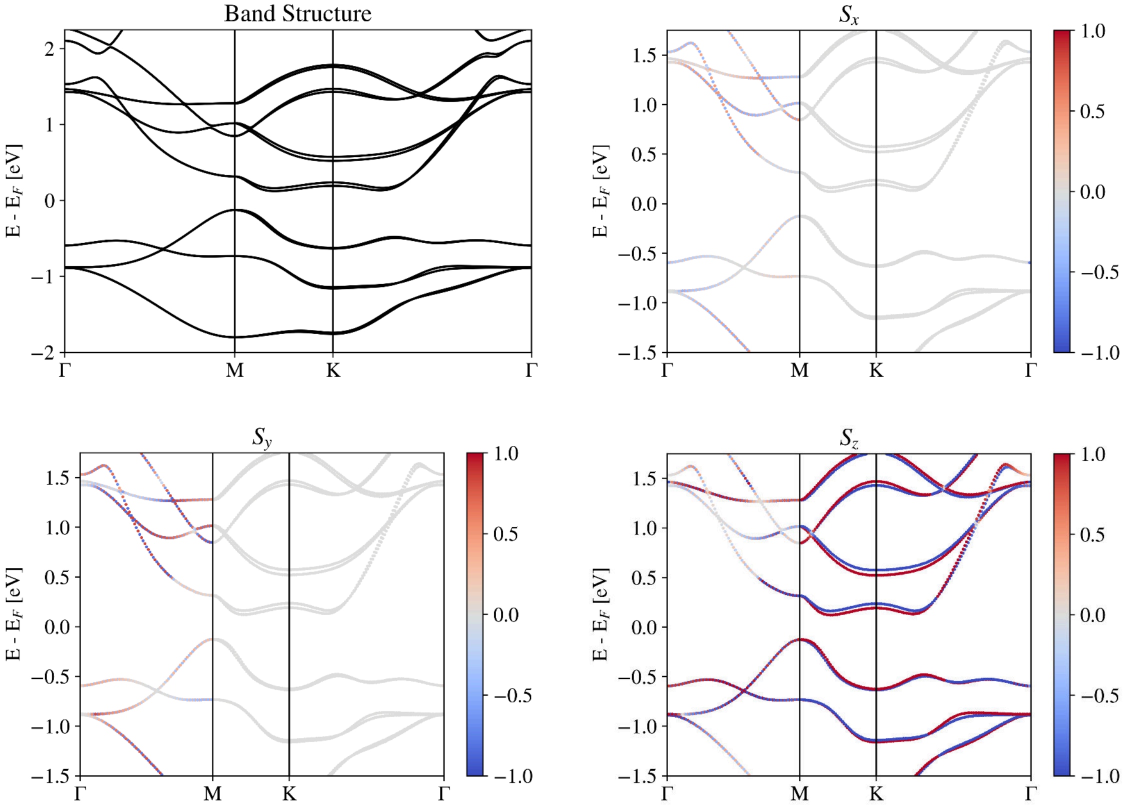


Figure 681: Band structure and spin polarization projections.

## 2.341 Se2Zn2-90835c470691

- **Formula**: Se2Zn2
- **Structural Cluster**: AB-2
- **Band gap (PBE)**: 1.61 eV
- **Energy above convex hull (C2DB)**: 0.245 eV
- **Space group symbol**: *Cm*
- **Space group number**: 8
- **Polar structure**: True

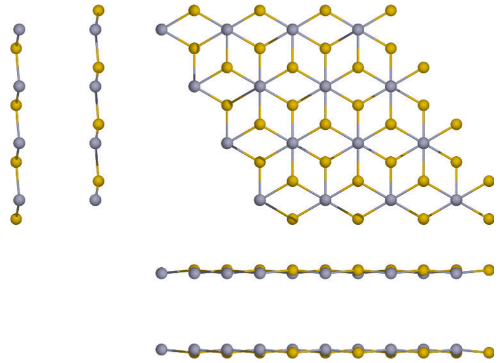

Figure 682: Structure representation

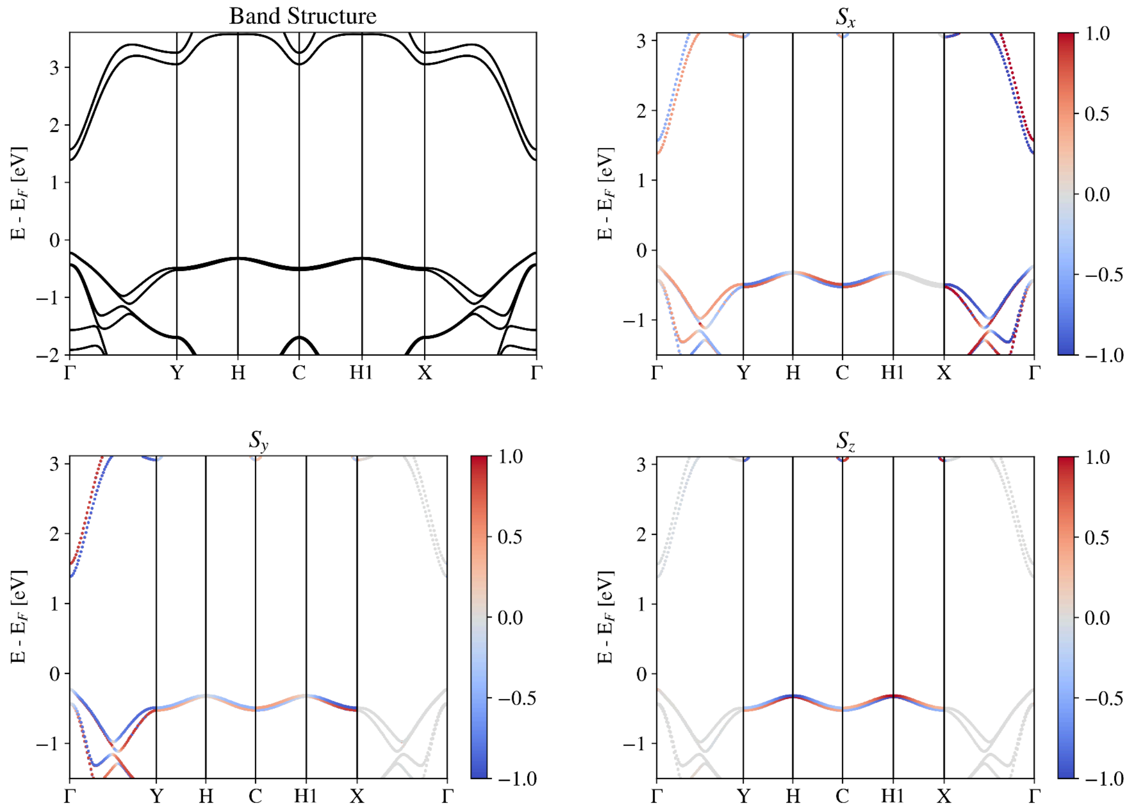


Figure 683: Band structure and spin polarization projections.

## 2.342 Se2Zr2-f89b20d72c95

- **Formula**: Se2Zr2
- **Structural Cluster**: AB-5
- **Band gap (PBE)**: 0.06 eV
- **Energy above convex hull (C2DB)**: 0.436 eV
- **Space group symbol**: $P\bar{6}m2$
- **Space group number**: 187
- **Polar structure**: False

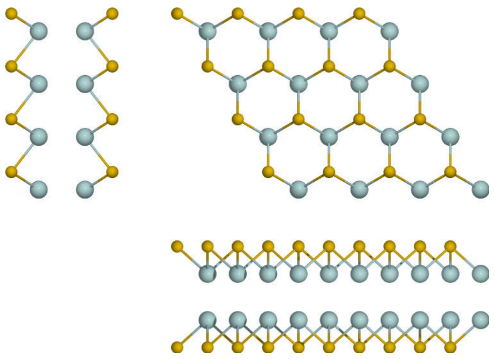

Figure 684: Structure representation

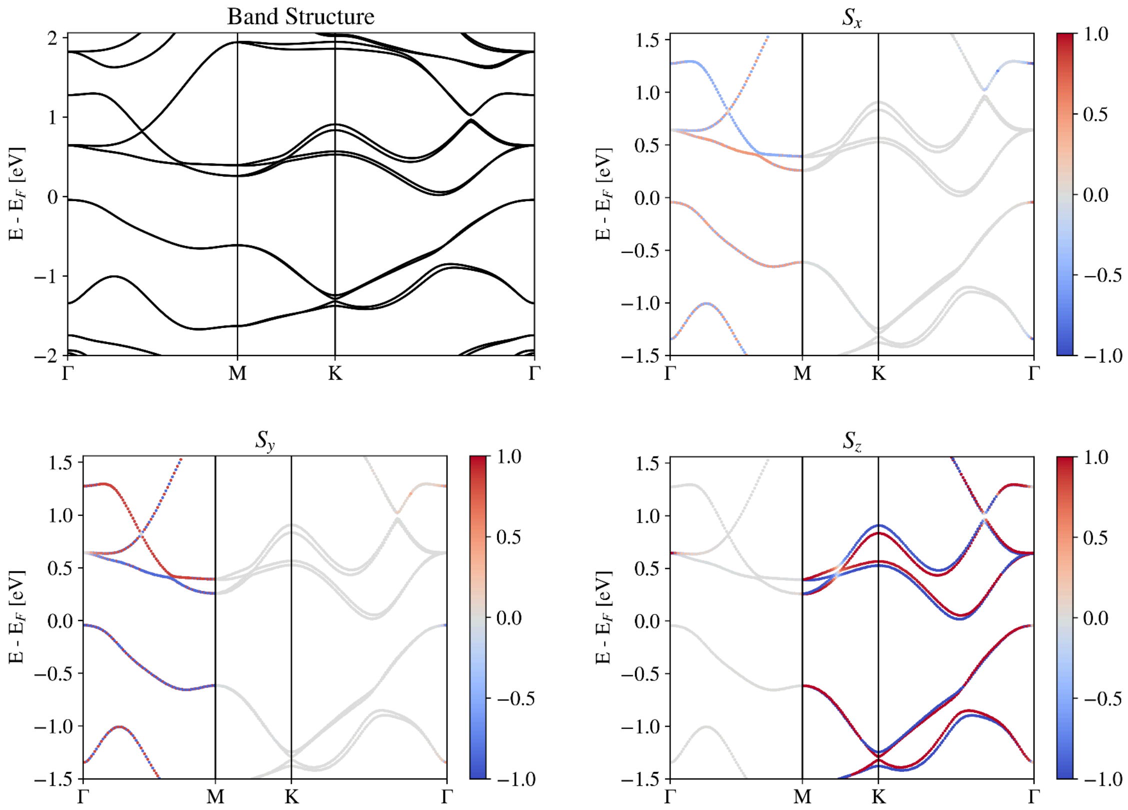


Figure 685: Band structure and spin polarization projections.

## 2.343 SeSn-d59c96fdfda1

- **Formula**: SeSn
- **Structural Cluster**: AB-25
- **Band gap (PBE)**: 2.156 eV
- **Energy above convex hull (C2DB)**: 0.098 eV
- **Space group symbol**: *P*3*m*1
- **Space group number**: 156
- **Polar structure**: True

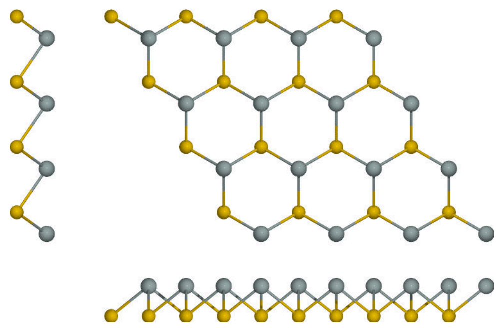

Figure 686: Structure representation

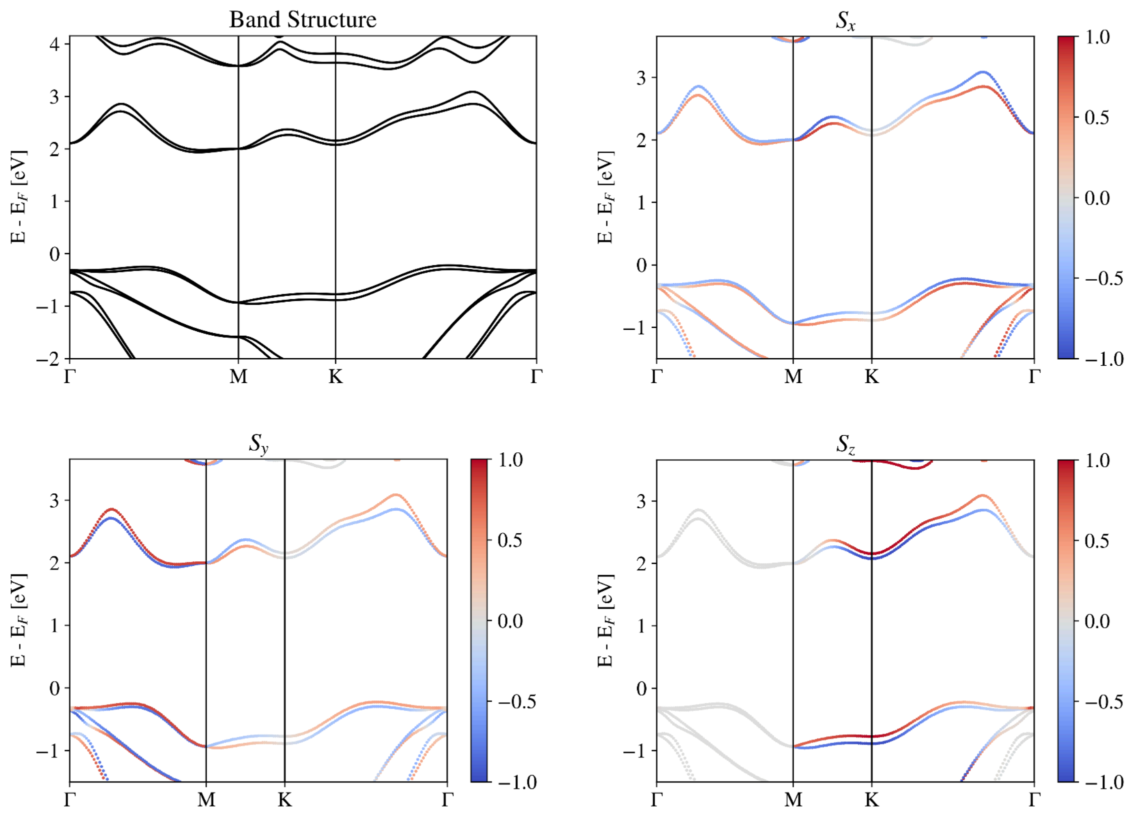


Figure 687: Band structure and spin polarization projections.

## 2.344 SeTeW-6e2a4c6f4f57

- **Formula**: SeTeW
- **Structural Cluster**: ABC-4
- **Band gap (PBE)**: 1.058 eV
- **Energy above convex hull (C2DB)**: 0.042 eV
- **Space group symbol**: $P3m1$
- **Space group number**: 156
- **Polar structure**: True

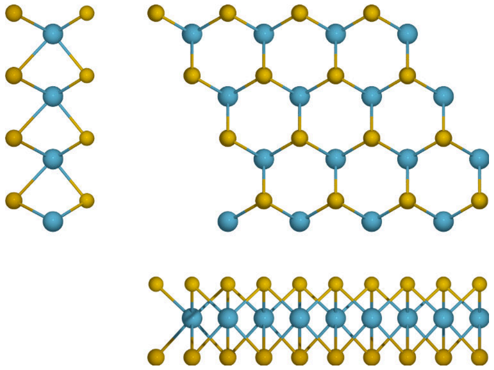

Figure 688: Structure representation

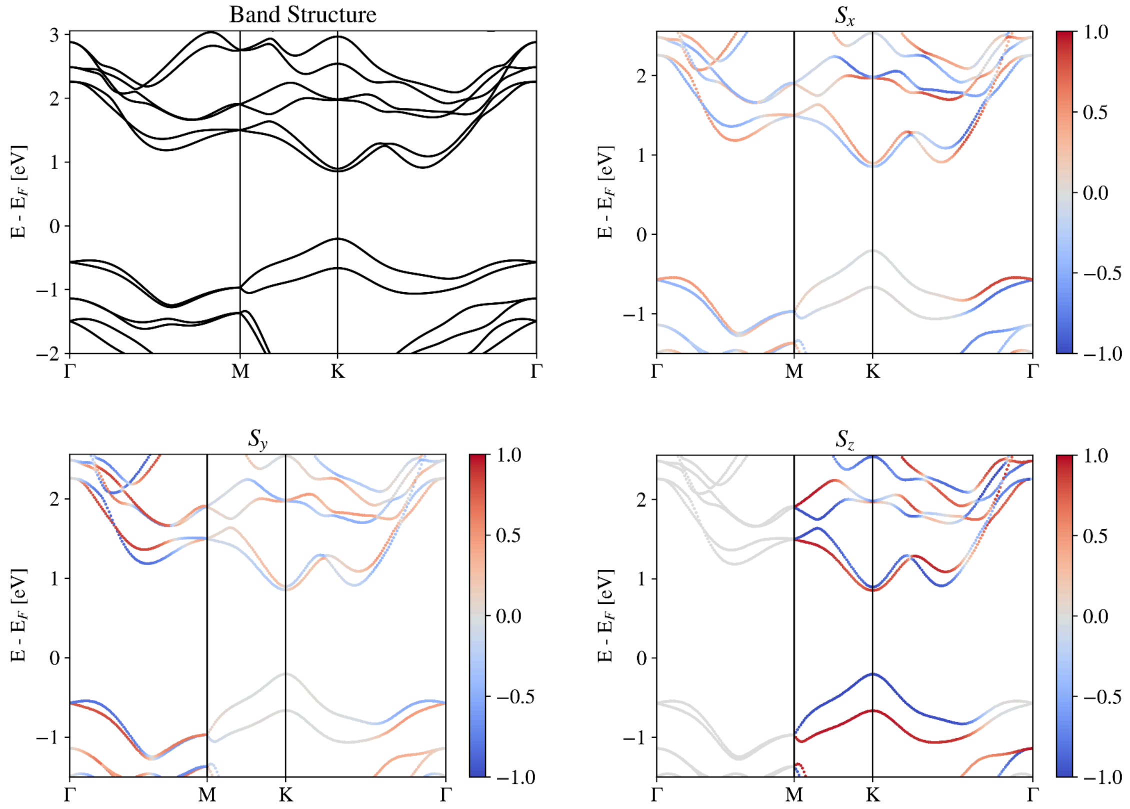


Figure 689: Band structure and spin polarization projections.

## 2.345 SeTeZr-dd69b684c867

- **Formula**: SeTeZr
- **Structural Cluster**: ABC-4
- **Band gap (PBE)**: 0.275 eV
- **Energy above convex hull (C2DB)**: 0.115 eV
- **Space group symbol**: $P3m1$
- **Space group number**: 156
- **Polar structure**: True

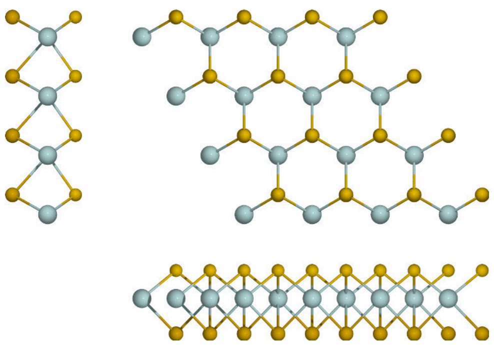

Figure 690: Structure representation

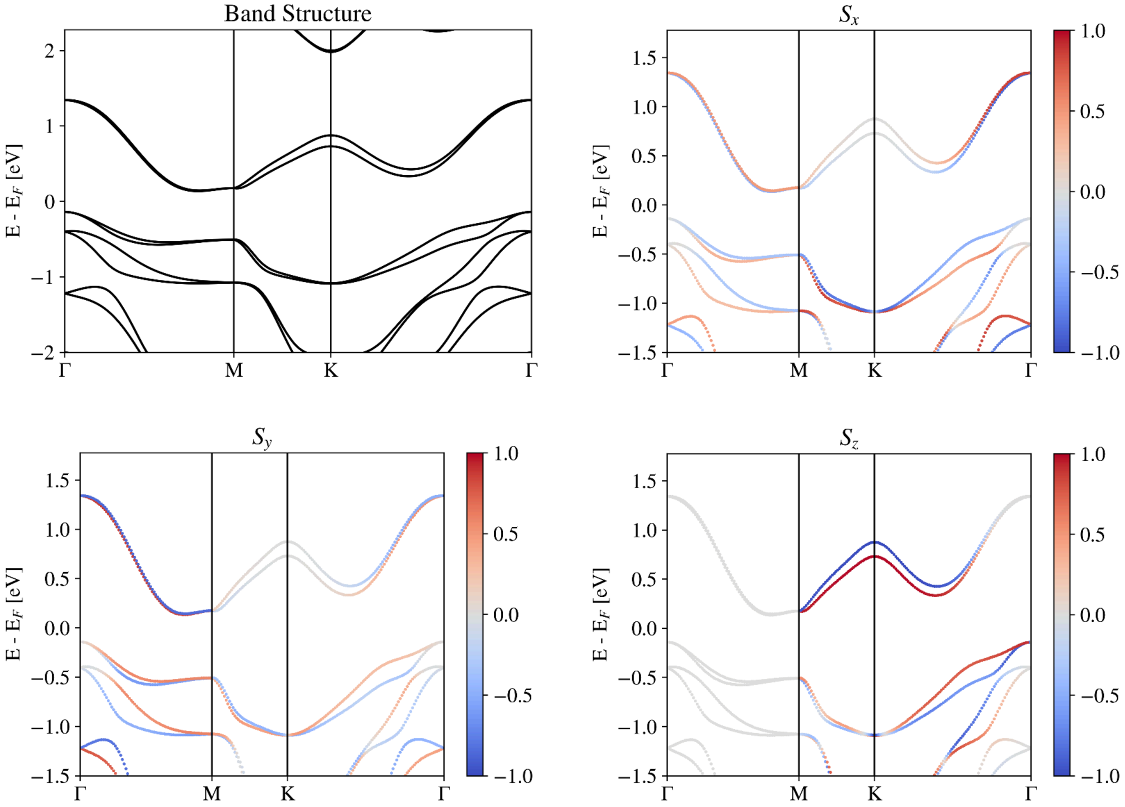


Figure 691: Band structure and spin polarization projections.

## 2.346 Sn2Te2-03bcf7dcdaf2

- **Formula**: Sn2Te2
- **Structural Cluster**: AB-0
- **Band gap (PBE)**: 0.595 eV
- **Energy above convex hull (C2DB)**: 0.063 eV
- **Space group symbol**: $Pmn2_1$
- **Space group number**: 31
- **Polar structure**: True

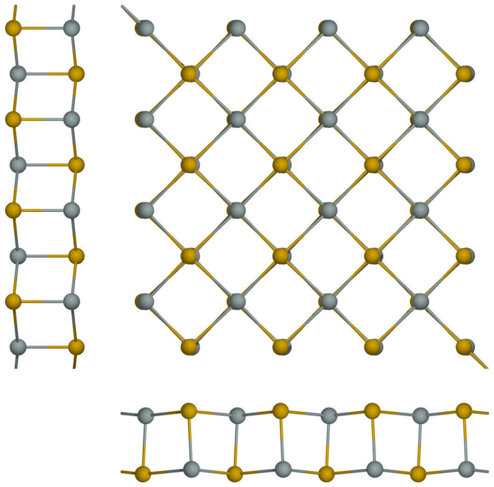

Figure 692: Structure representation

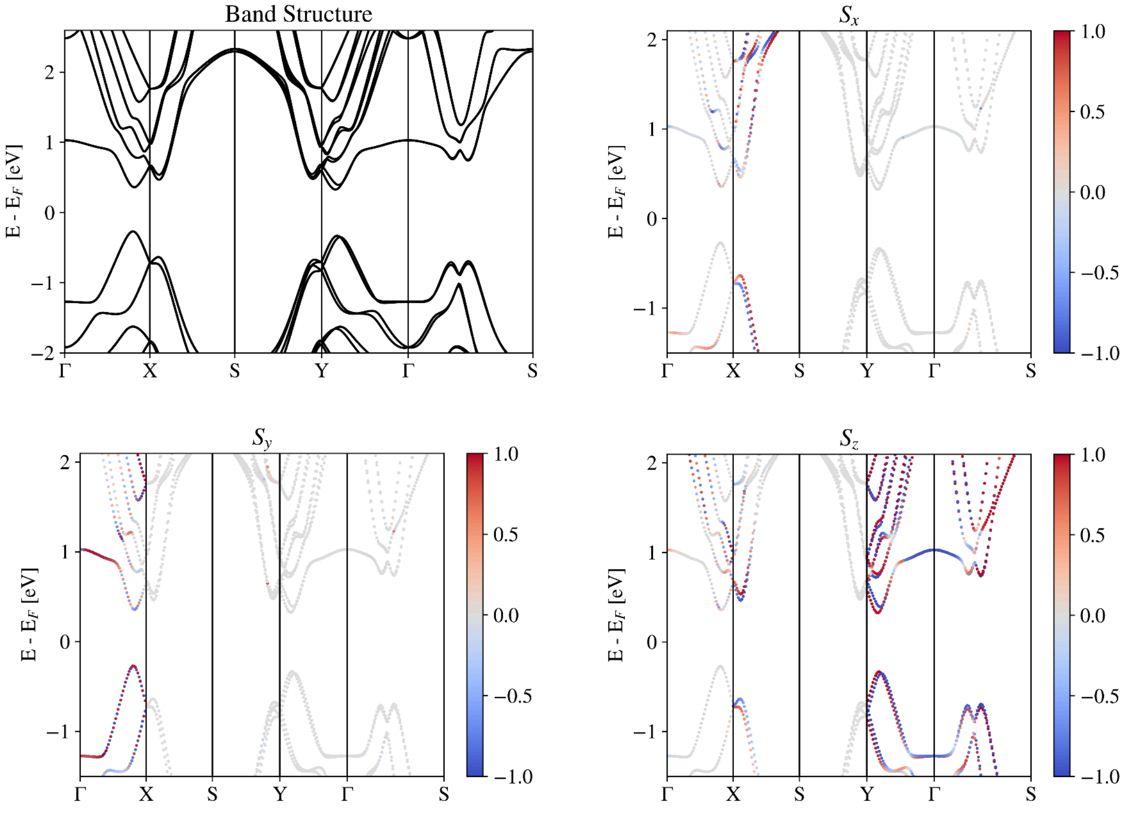


Figure 693: Band structure and spin polarization projections.

## 2.347 Sn2Te2-653f8247e25e

- **Formula**: Sn2Te2
- **Structural Cluster**: AB-6
- **Band gap (PBE)**: 0.422 eV
- **Energy above convex hull (C2DB)**: 0.087 eV
- **Space group symbol**: $P1$
- **Space group number**: 1
- **Polar structure**: True

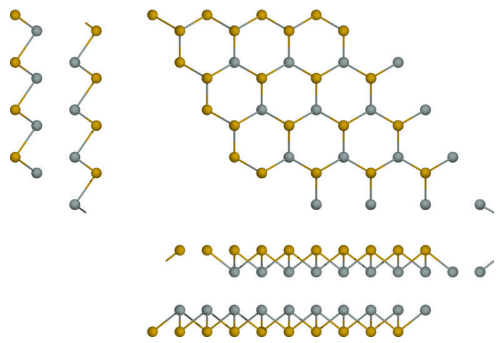

Figure 694: Structure representation

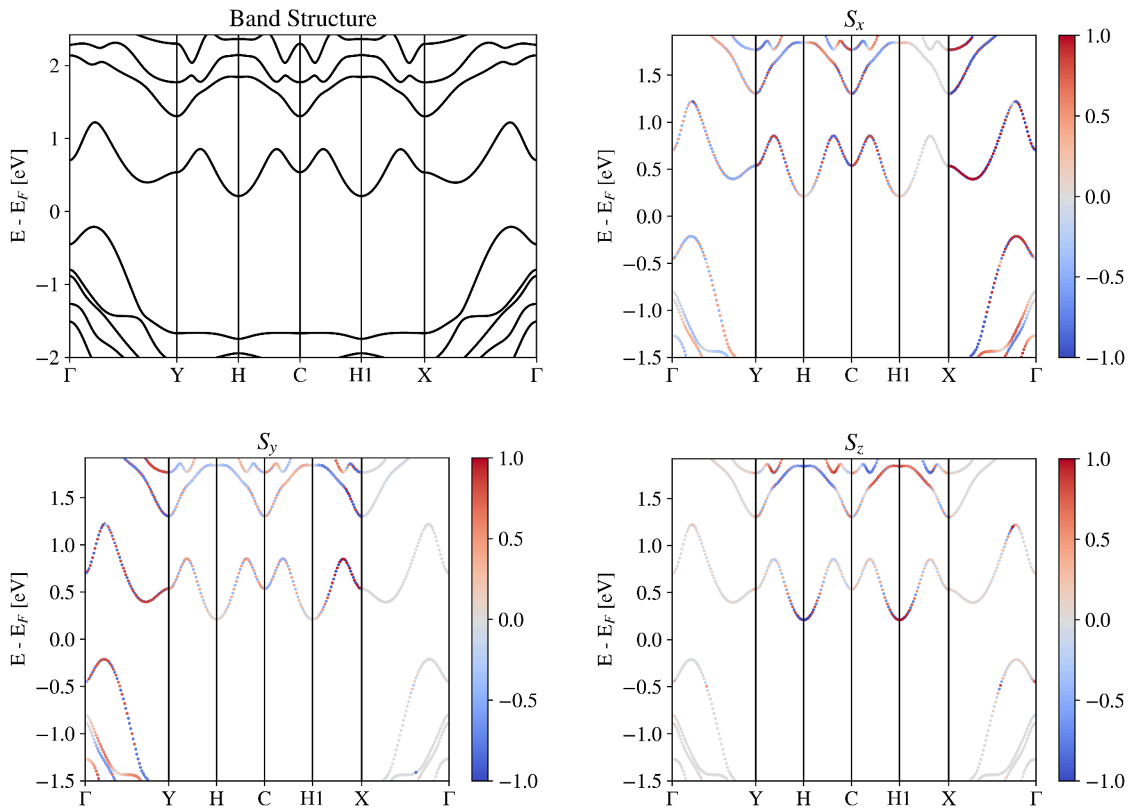


Figure 695: Band structure and spin polarization projections.

## 2.348 SnBr2-0155c4de2320

- **Formula**: SnBr2
- **Structural Cluster**: AB2-11
- **Band gap (PBE)**: 1.284 eV
- **Energy above convex hull (C2DB)**: 0.136 eV
- **Space group symbol**: $P\bar{4}m2$
- **Space group number**: 115
- **Polar structure**: False

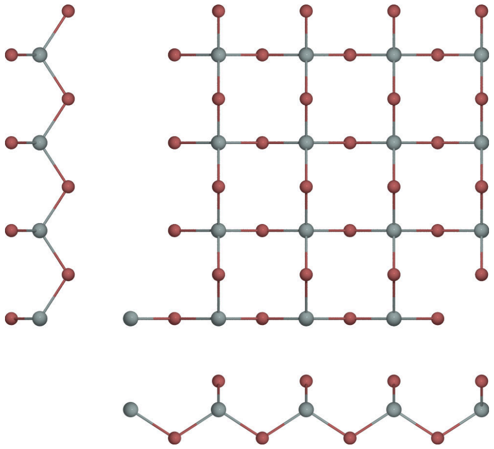

Figure 696: Structure representation

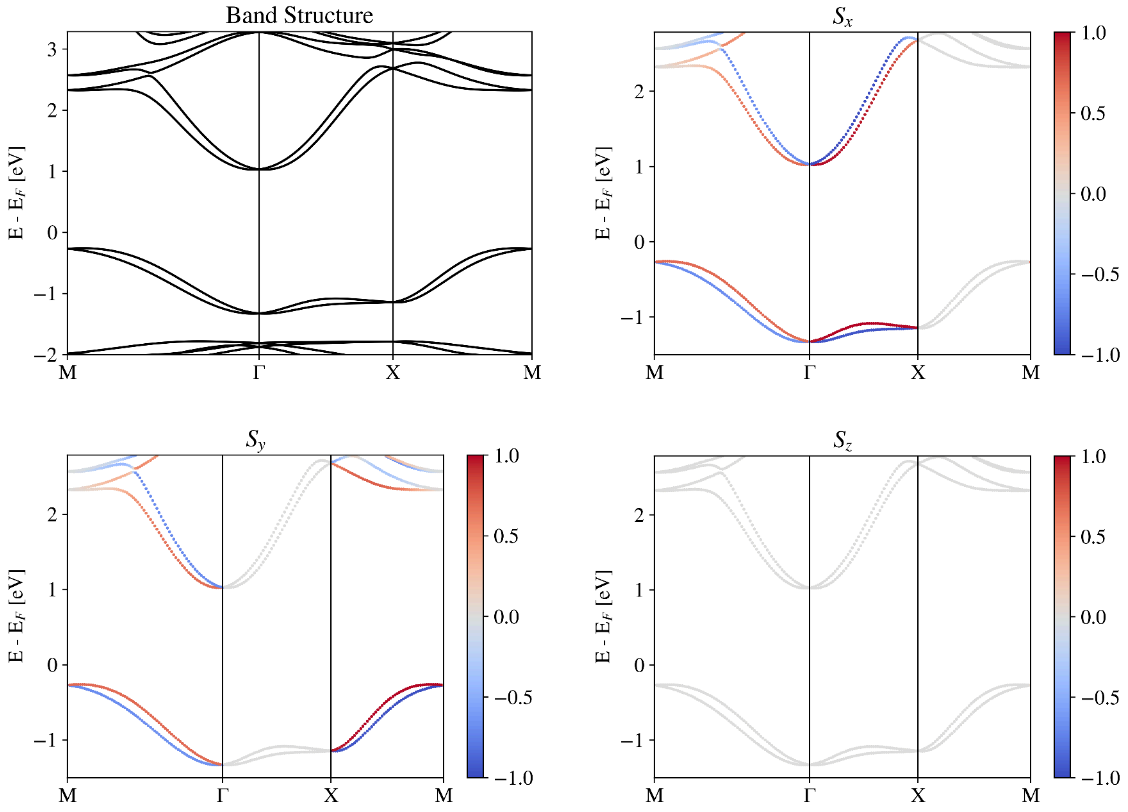


Figure 697: Band structure and spin polarization projections.

## 2.349 SnBr2-8d365ca62c55

- **Formula**: SnBr2
- **Structural Cluster**: AB2-4
- **Band gap (PBE)**: 2.514 eV
- **Energy above convex hull (C2DB)**: 0.086 eV
- **Space group symbol**: $P\bar{6}m2$
- **Space group number**: 187
- **Polar structure**: False

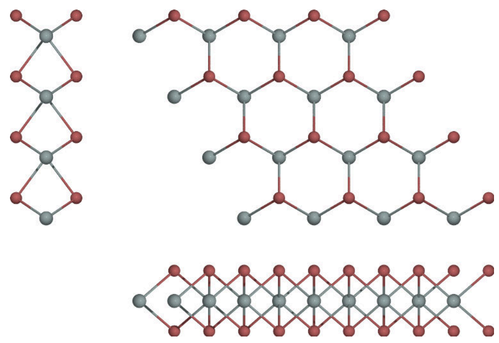

Figure 698: Structure representation

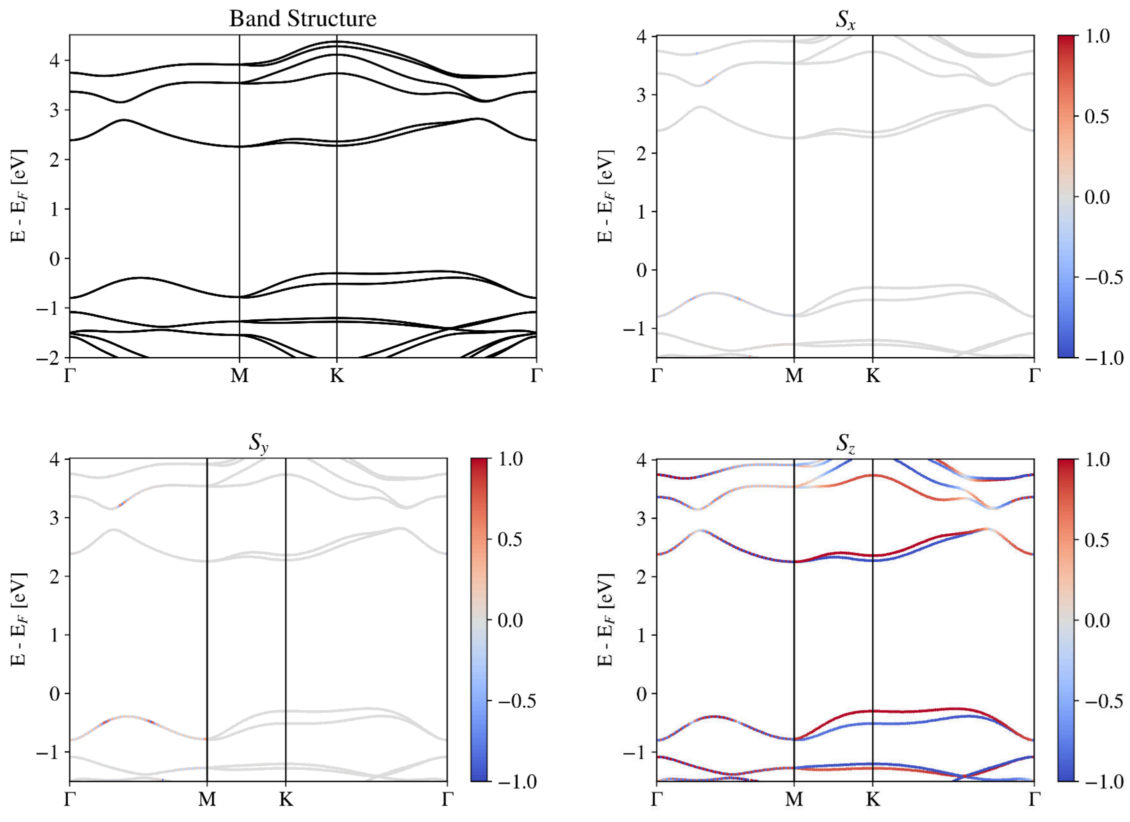


Figure 699: Band structure and spin polarization projections.

## 2.350 SnCl2-514a8a12dca9

- **Formula**: SnCl2
- **Structural Cluster**: AB2-4
- **Band gap (PBE)**: 2.76 eV
- **Energy above convex hull (C2DB)**: 0.117 eV
- **Space group symbol**: $P\bar{6}m2$
- **Space group number**: 187
- **Polar structure**: False

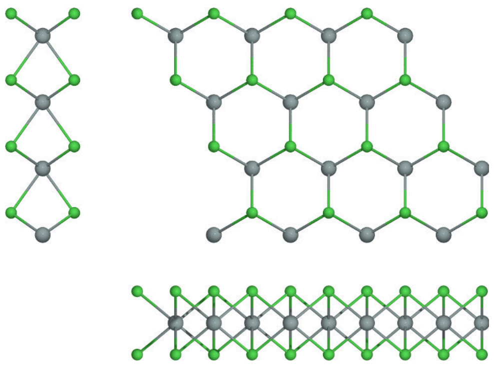

Figure 700: Structure representation

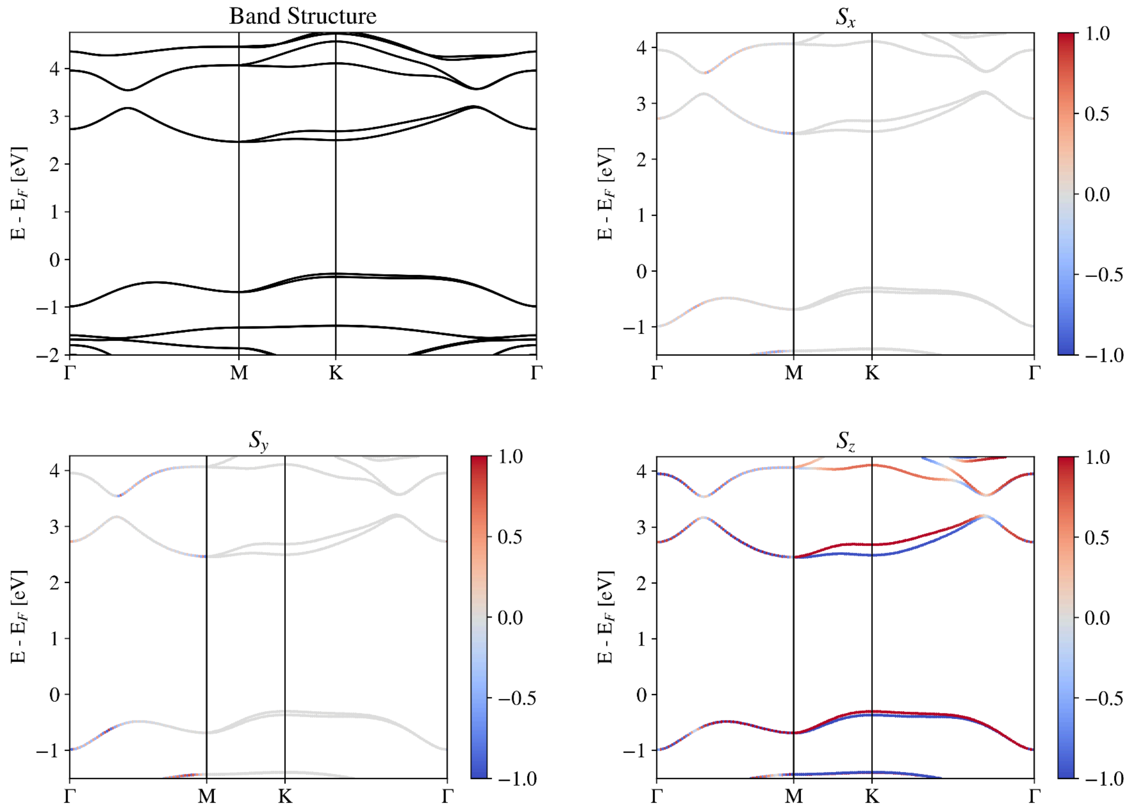


Figure 701: Band structure and spin polarization projections.

## 2.351 SnCl2-95805103ce95

- **Formula**: SnCl2
- **Structural Cluster**: AB2-11
- **Band gap (PBE)**: 1.464 eV
- **Energy above convex hull (C2DB)**: 0.146 eV
- **Space group symbol**: $P\bar{4}m2$
- **Space group number**: 115
- **Polar structure**: False

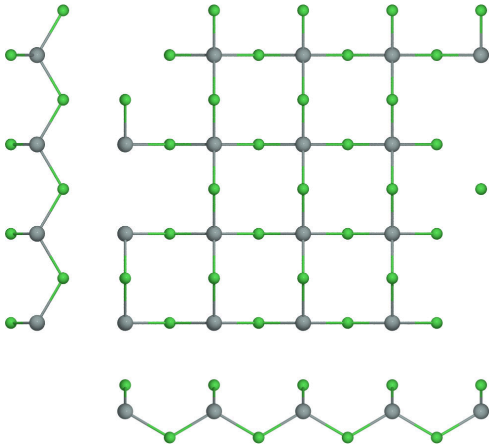

Figure 702: Structure representation

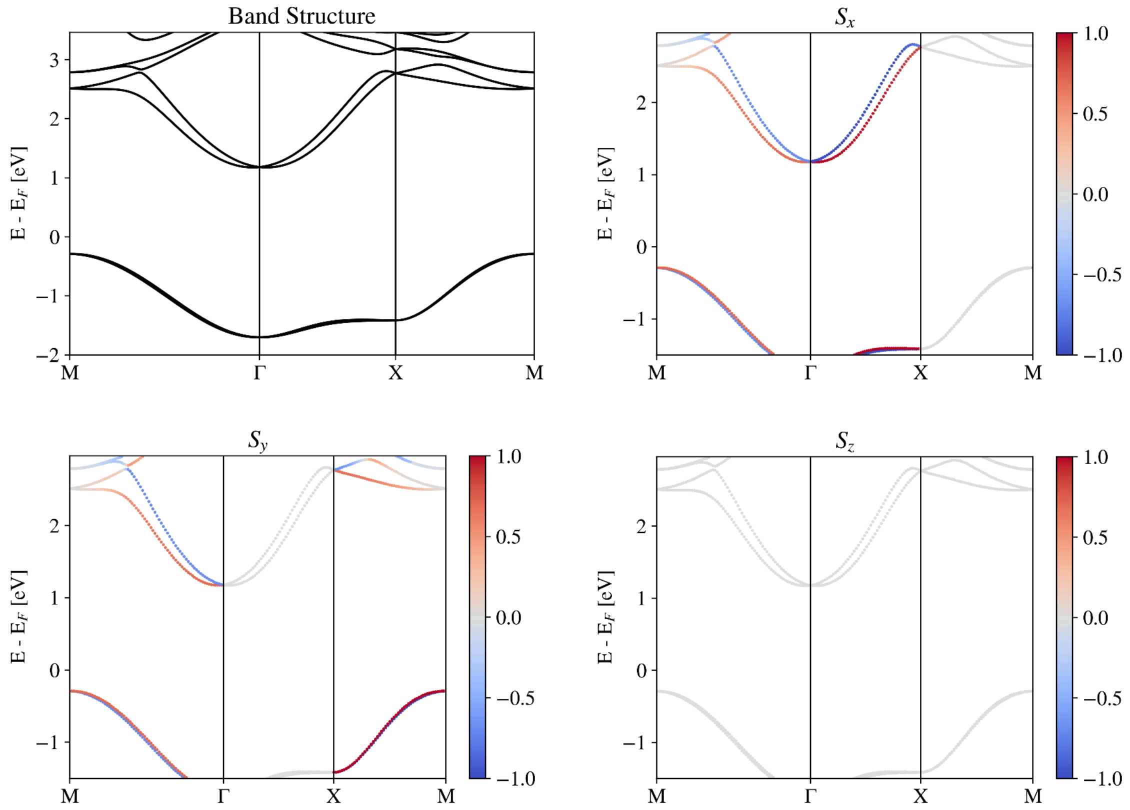


Figure 703: Band structure and spin polarization projections.

## 2.352 SnF2-d6c3ab6de6b8

- **Formula**: SnF2
- **Structural Cluster**: AB2-11
- **Band gap (PBE)**: 1.665 eV
- **Energy above convex hull (C2DB)**: 0.2 eV
- **Space group symbol**: $P\bar{4}m2$
- **Space group number**: 115
- **Polar structure**: False

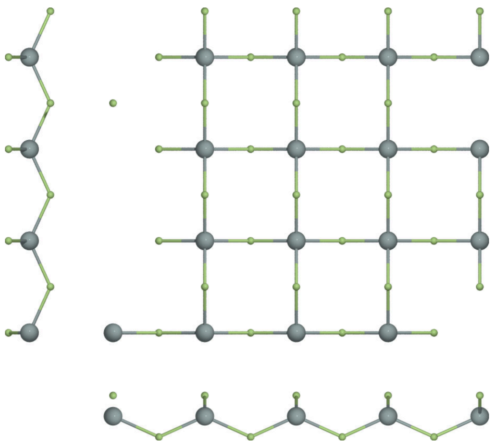

Figure 704: Structure representation

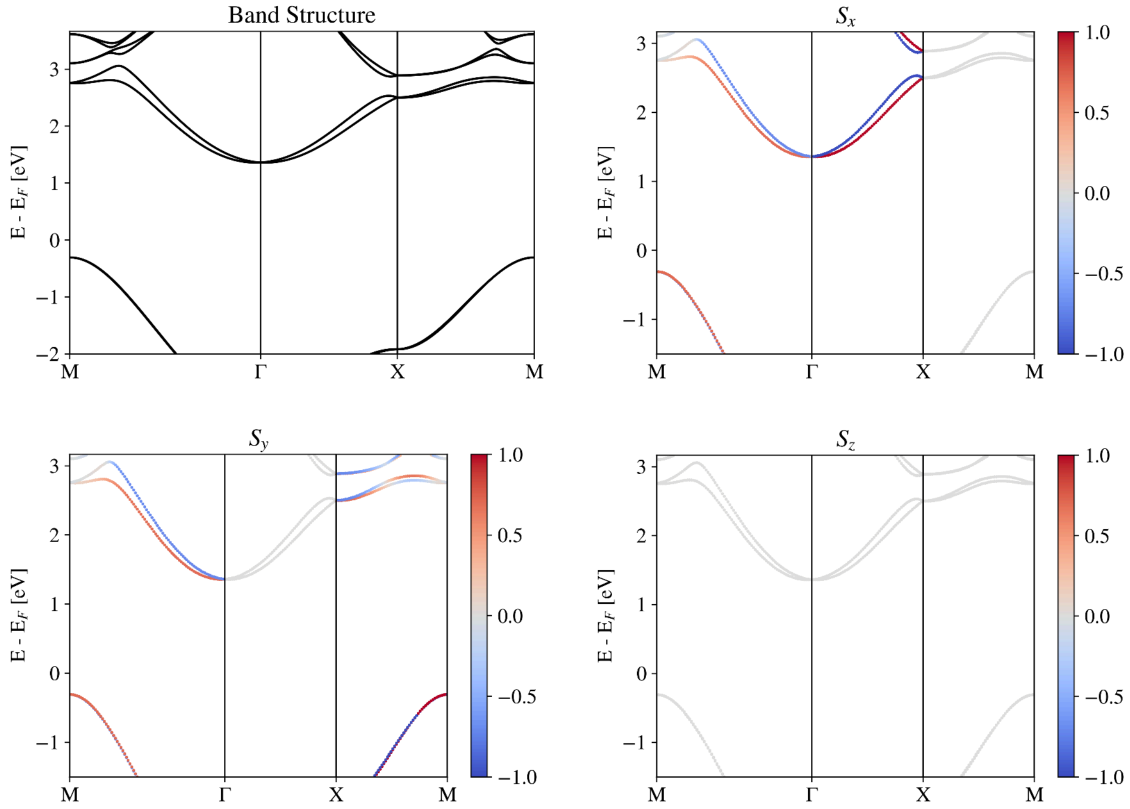


Figure 705: Band structure and spin polarization projections.

## 2.353 SnI2-7f0ca28e3229

- **Formula**: SnI2
- **Structural Cluster**: AB2-11
- **Band gap (PBE)**: 1.143 eV
- **Energy above convex hull (C2DB)**: 0.157 eV
- **Space group symbol**: $P\bar{4}m2$
- **Space group number**: 115
- **Polar structure**: False

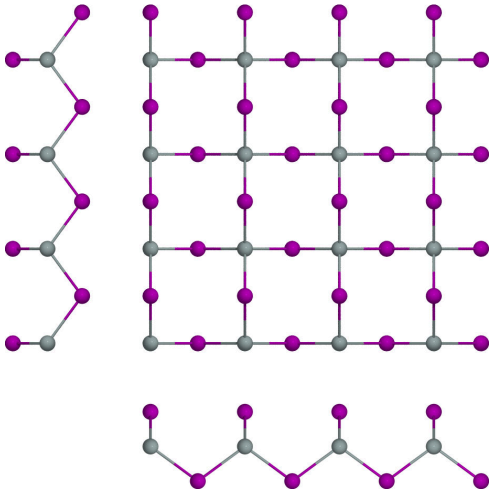

Figure 706: Structure representation

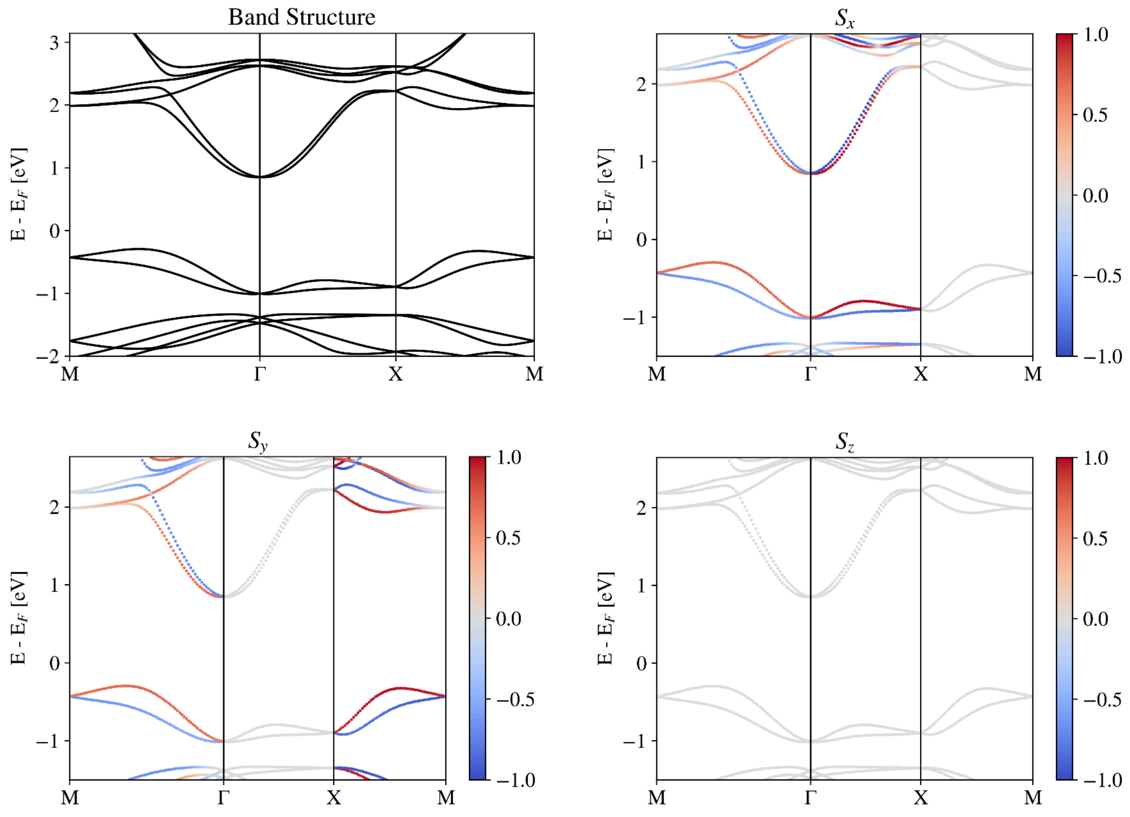


Figure 707: Band structure and spin polarization projections.

## 2.354 SnI2-d9c422656482

- **Formula**: SnI2
- **Structural Cluster**: AB2-4
- **Band gap (PBE)**: 1.965 eV
- **Energy above convex hull (C2DB)**: 0.085 eV
- **Space group symbol**: $P\bar{6}m2$
- **Space group number**: 187
- **Polar structure**: False

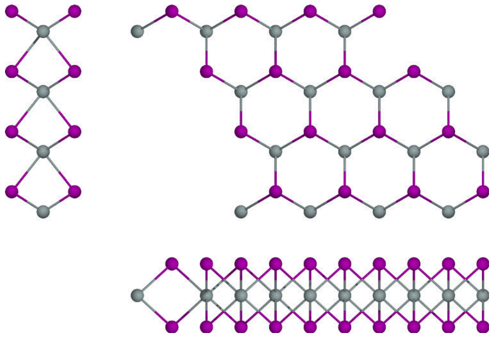

Figure 708: Structure representation

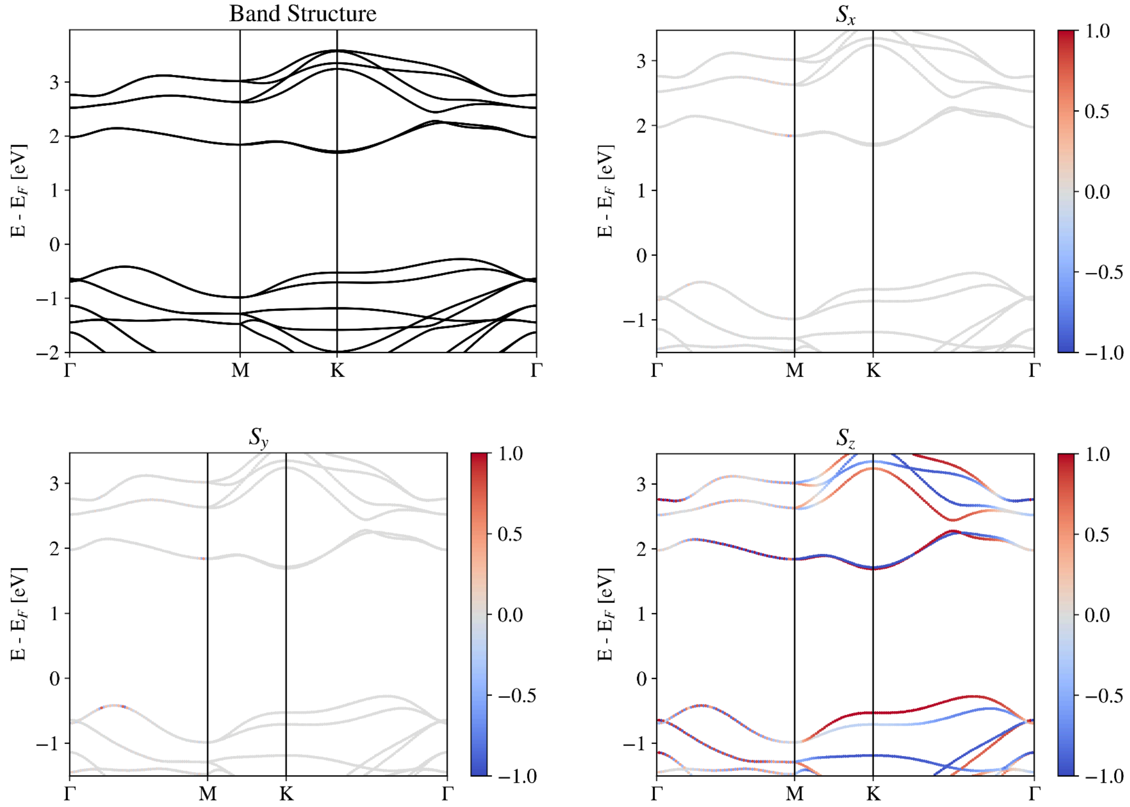


Figure 709: Band structure and spin polarization projections.

## 2.355 SnO2-96a036411ab6

- **Formula**: SnO2
- **Structural Cluster**: AB2-11
- **Band gap (PBE)**: 2.065 eV
- **Energy above convex hull (C2DB)**: 0.434 eV
- **Space group symbol**: $P\bar{4}m2$
- **Space group number**: 115
- **Polar structure**: False

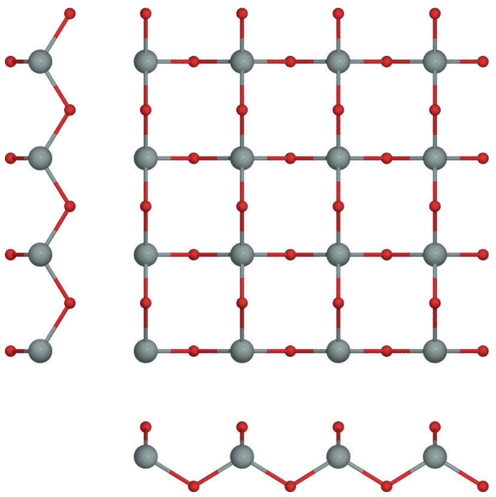

Figure 710: Structure representation

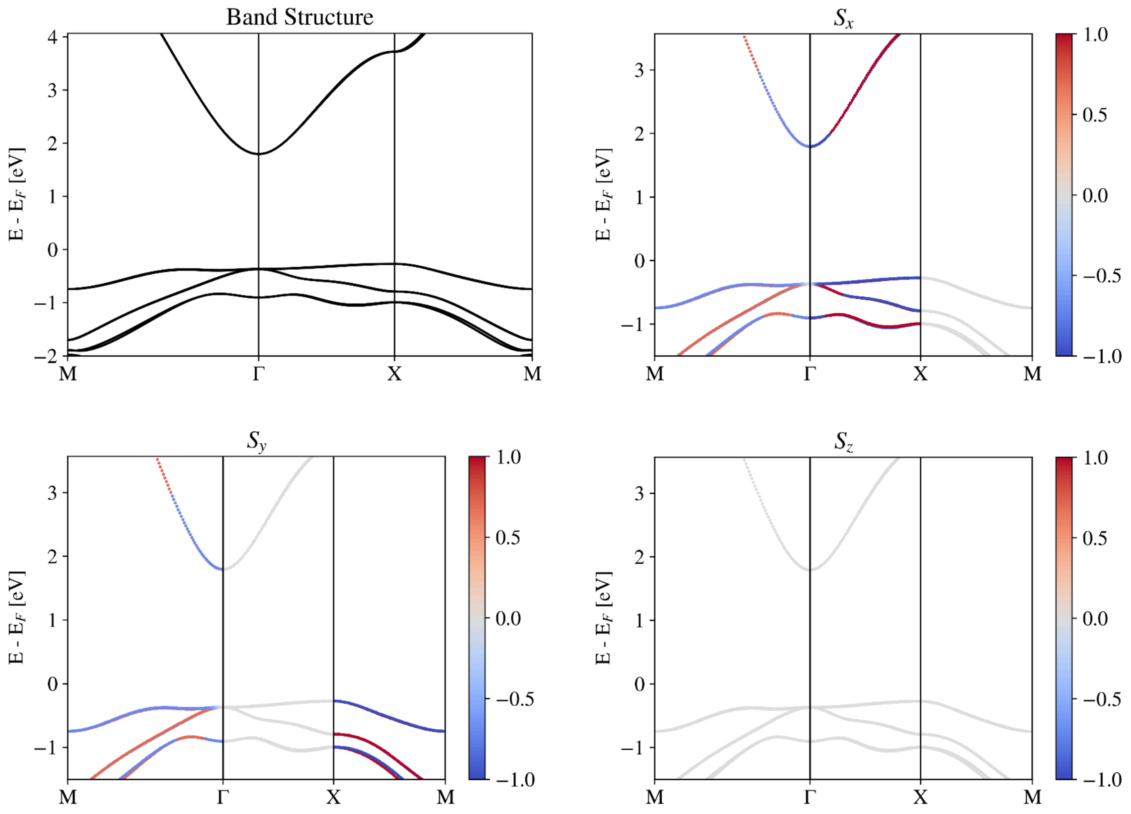


Figure 711: Band structure and spin polarization projections.

## 2.356 SnO2-d5f47e5d4cf7

- **Formula**: SnO2
- **Structural Cluster**: AB2-4
- **Band gap (PBE)**: 0.623 eV
- **Energy above convex hull (C2DB)**: 0.645 eV
- **Space group symbol**: $P\bar{6}m2$
- **Space group number**: 187
- **Polar structure**: False

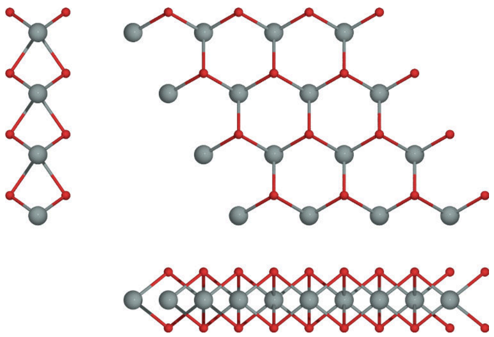

Figure 712: Structure representation

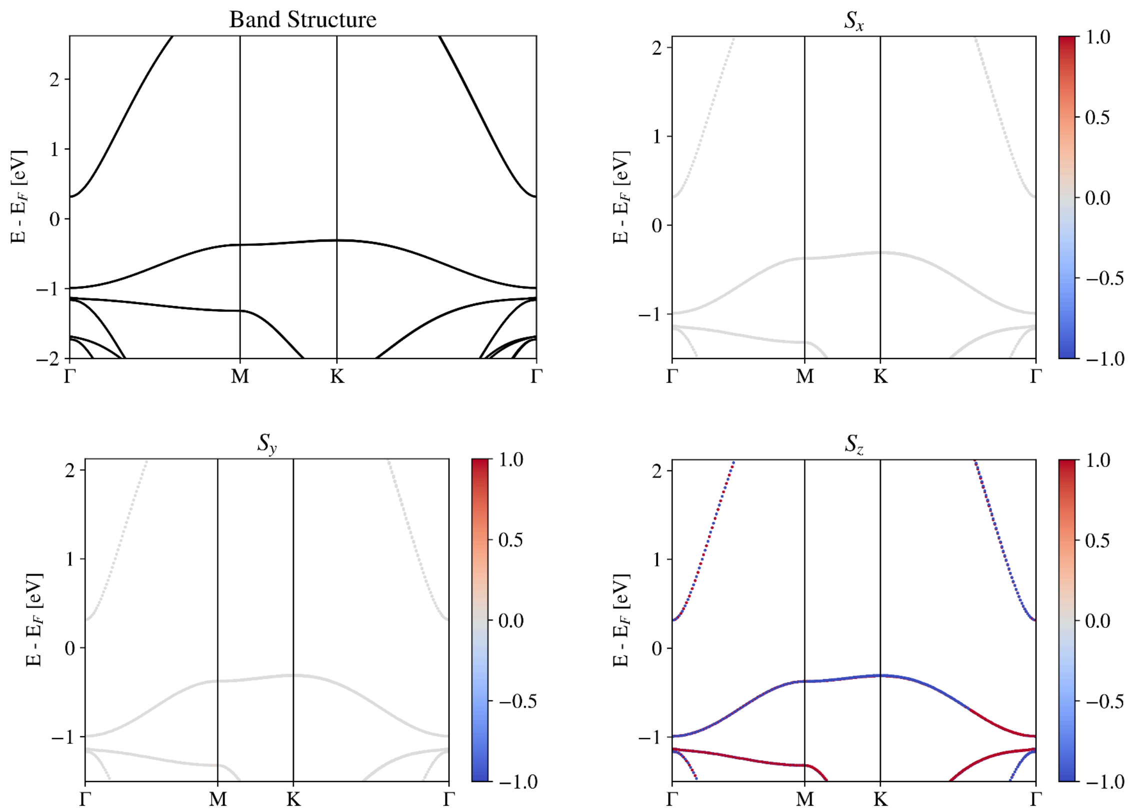


Figure 713: Band structure and spin polarization projections.

### 2.357 SnS2-08a9307b286e

- **Formula**: SnS2
- **Structural Cluster**: AB2-11
- **Band gap (PBE)**: 1.451 eV
- **Energy above convex hull (C2DB)**: 0.082 eV
- **Space group symbol**: $P\bar{4}m2$
- **Space group number**: 115
- **Polar structure**: False

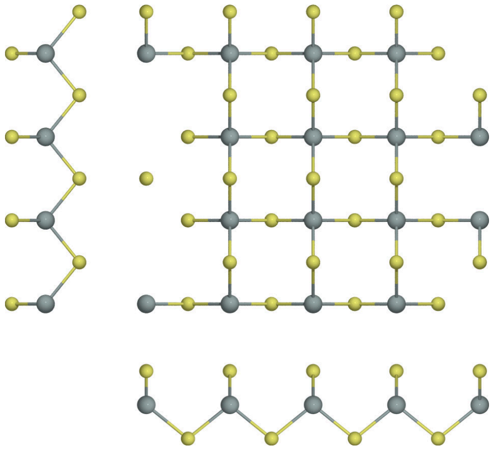

Figure 714: Structure representation

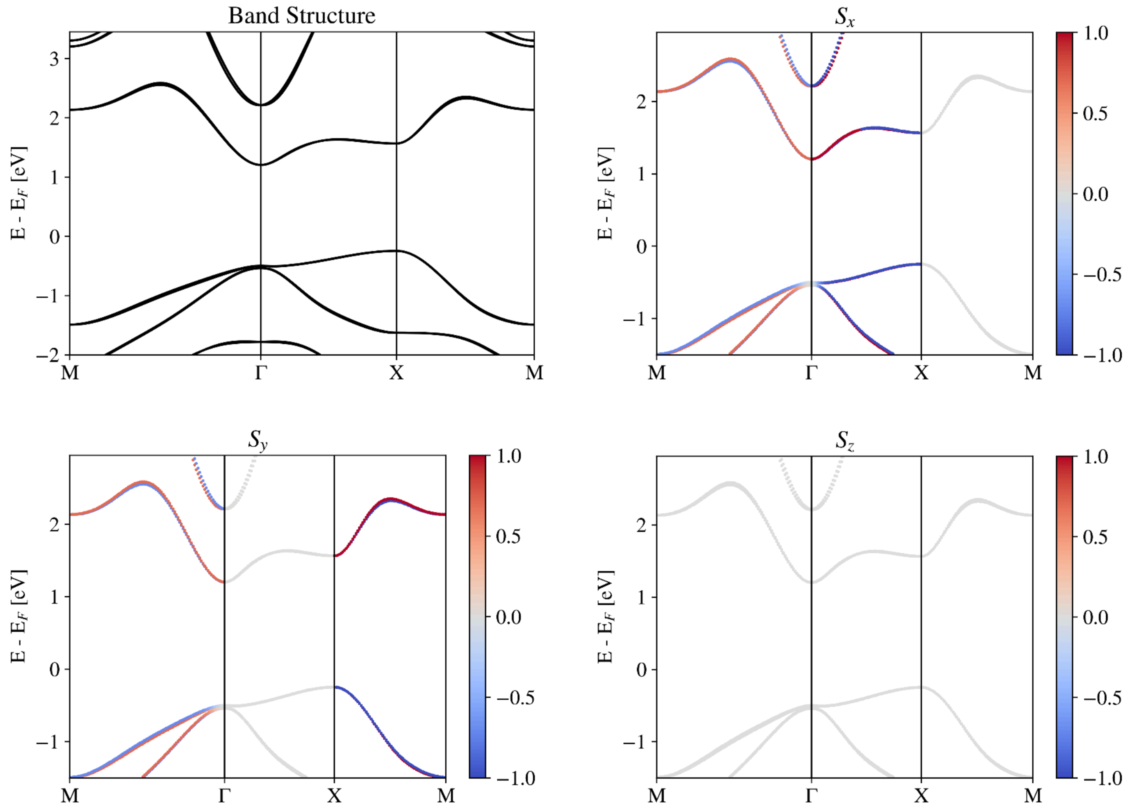


Figure 715: Band structure and spin polarization projections.

## 2.358 SnS2-8f2fa65321f0

- **Formula**: SnS2
- **Structural Cluster**: AB2-4
- **Band gap (PBE)**: 0.754 eV
- **Energy above convex hull (C2DB)**: 0.286 eV
- **Space group symbol**: $P\bar{6}m2$
- **Space group number**: 187
- **Polar structure**: False

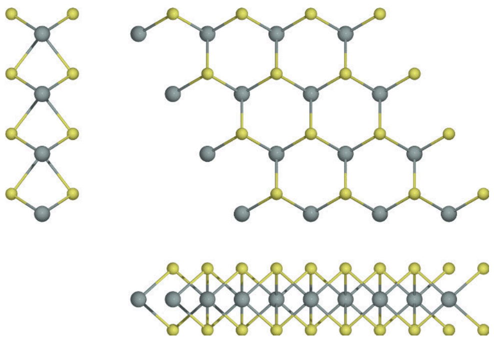

Figure 716: Structure representation

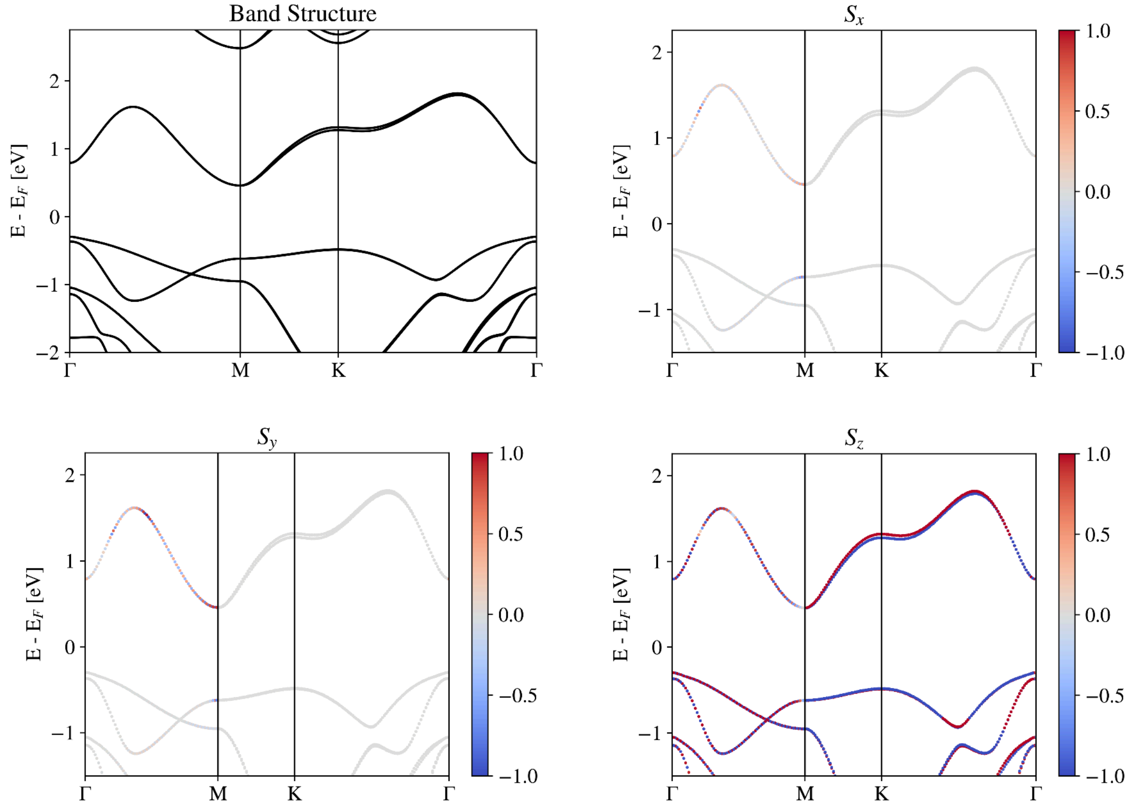


Figure 717: Band structure and spin polarization projections.

## 2.359 SnSe2-bfa429d647f9

- **Formula**: SnSe2
- **Structural Cluster**: AB2-11
- **Band gap (PBE)**: 0.854 eV
- **Energy above convex hull (C2DB)**: 0.056 eV
- **Space group symbol**: $P\bar{4}m2$
- **Space group number**: 115
- **Polar structure**: False

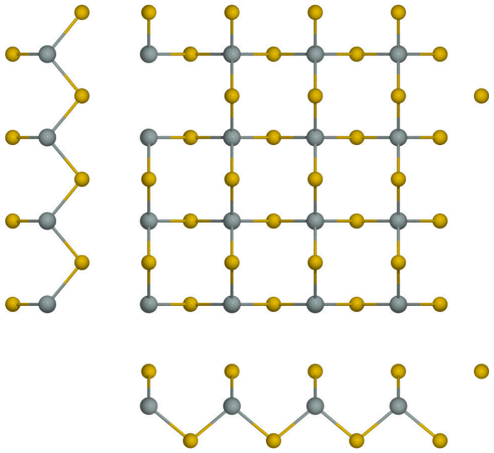

Figure 718: Structure representation

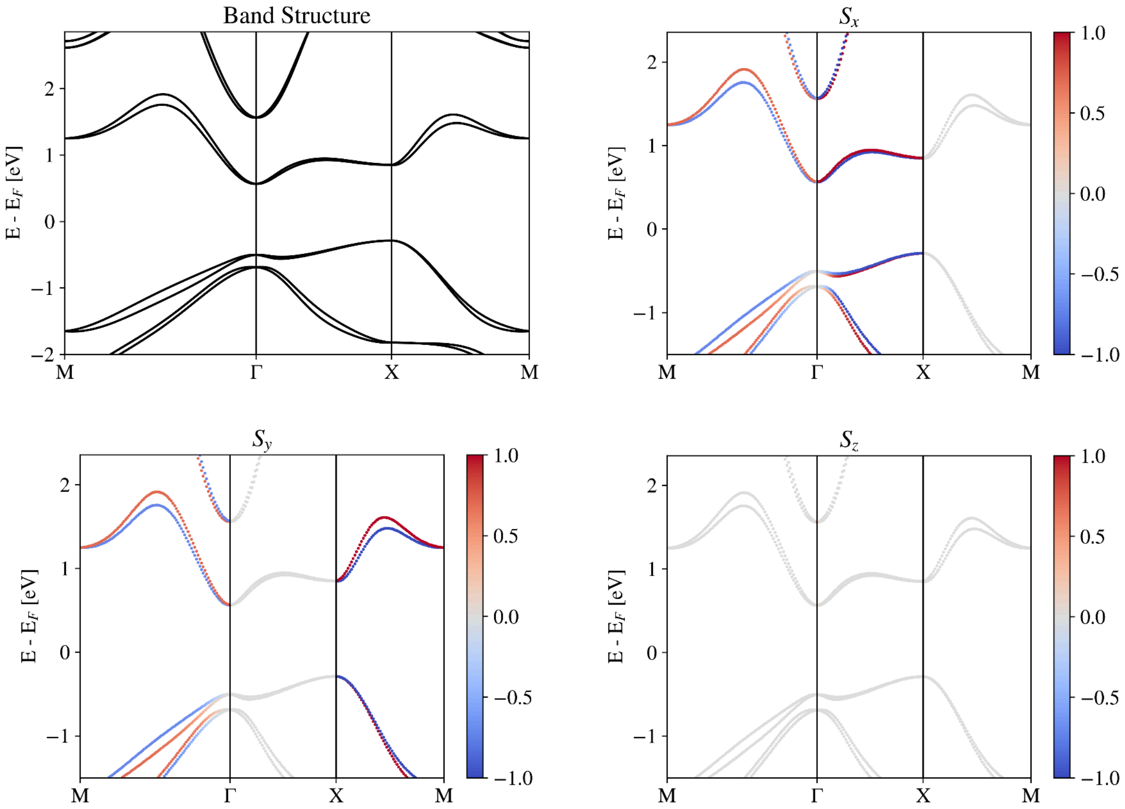


Figure 719: Band structure and spin polarization projections.

## 2.360 SnTe-e688959ea45b

- **Formula**: SnTe
- **Structural Cluster**: AB-25
- **Band gap (PBE)**: 1.592 eV
- **Energy above convex hull (C2DB)**: 0.119 eV
- **Space group symbol**: $P3m1$
- **Space group number**: 156
- **Polar structure**: True

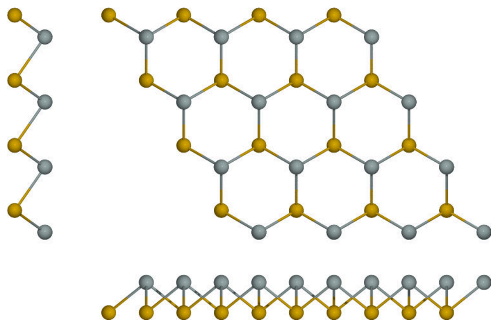

Figure 720: Structure representation

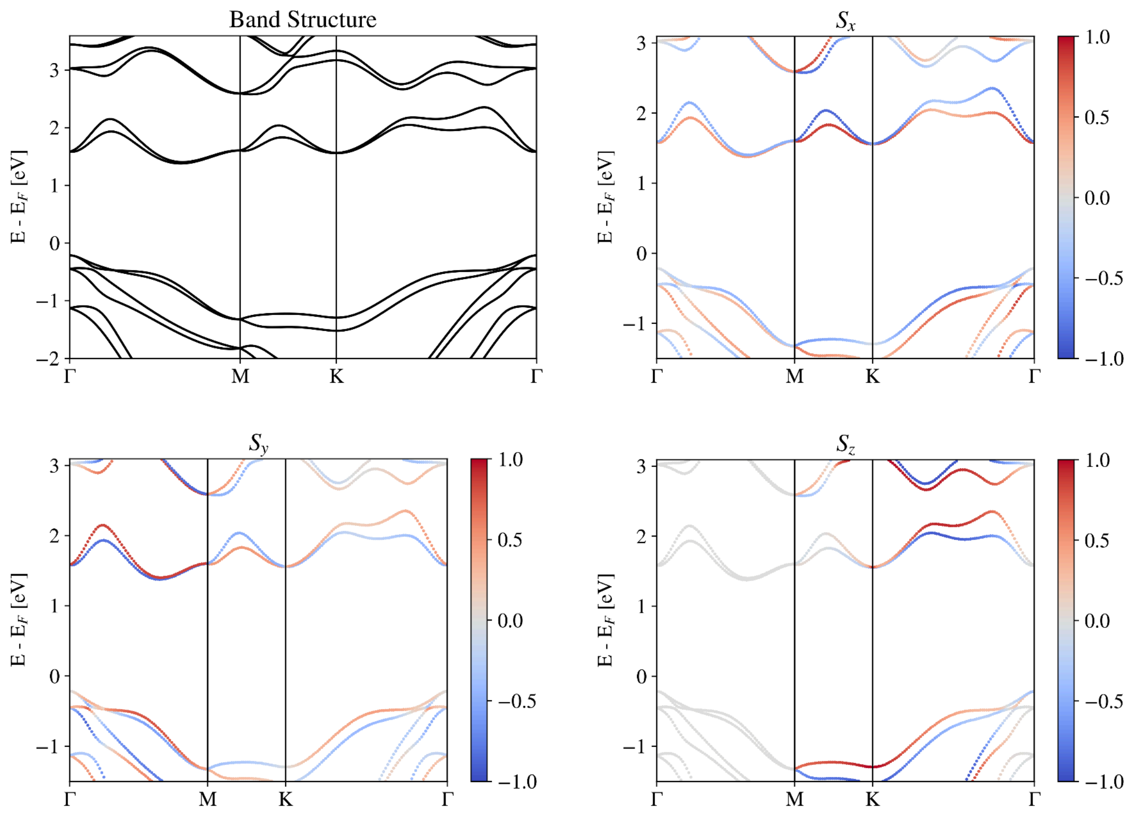


Figure 721: Band structure and spin polarization projections.

## 2.361 SnTe2-c81de95356c1

- **Formula**: SnTe2
- **Structural Cluster**: AB2-11
- **Band gap (PBE)**: 0.39 eV
- **Energy above convex hull (C2DB)**: 0.139 eV
- **Space group symbol**: $P\bar{4}m2$
- **Space group number**: 115
- **Polar structure**: False

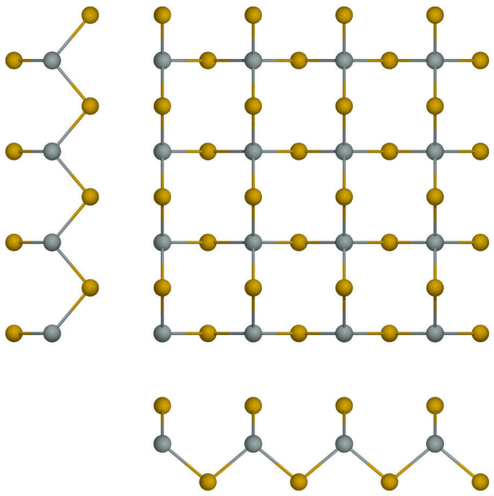

Figure 722: Structure representation

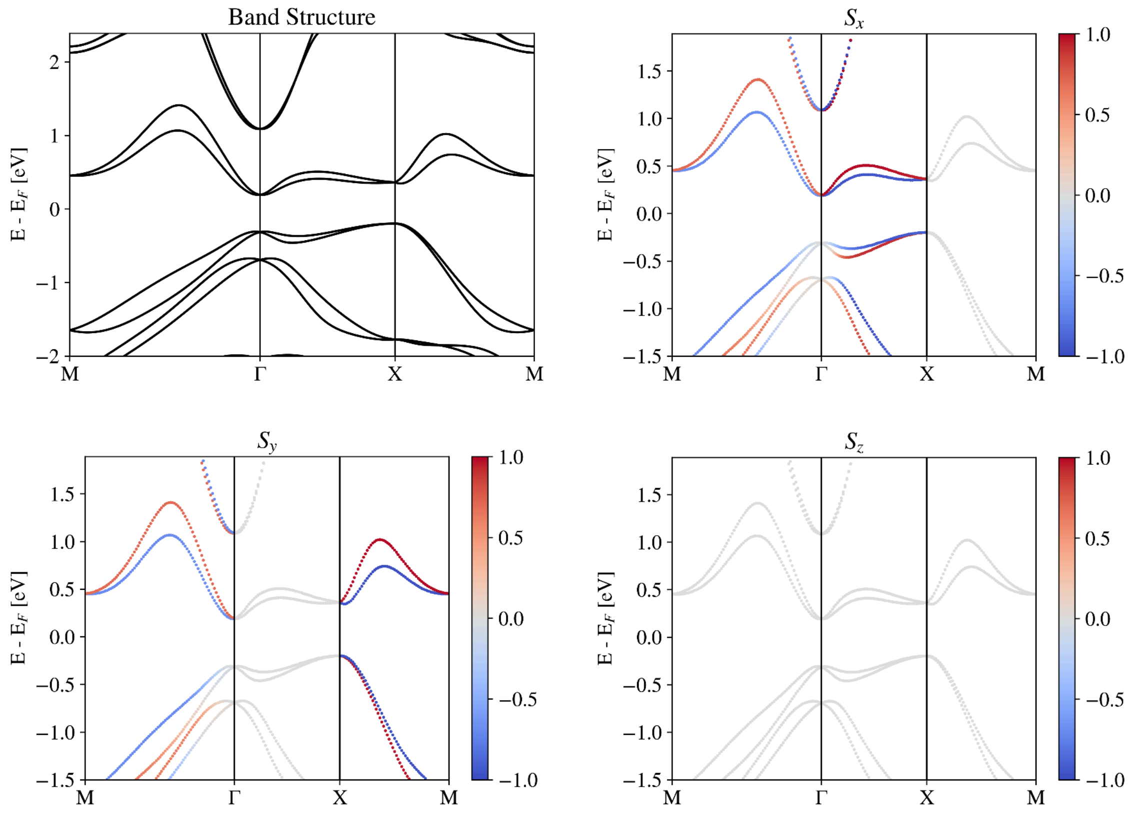


Figure 723: Band structure and spin polarization projections.

## 2.362 SrBr2-2876a0cb2478

- **Formula**: SrBr2
- **Structural Cluster**: AB2-4
- **Band gap (PBE)**: 4.324 eV
- **Energy above convex hull (C2DB)**: 0.14 eV
- **Space group symbol**: $P\bar{6}m2$
- **Space group number**: 187
- **Polar structure**: False

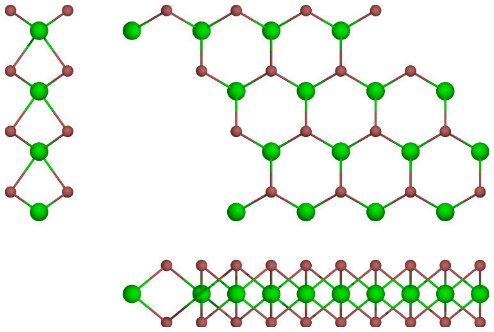

Figure 724: Structure representation

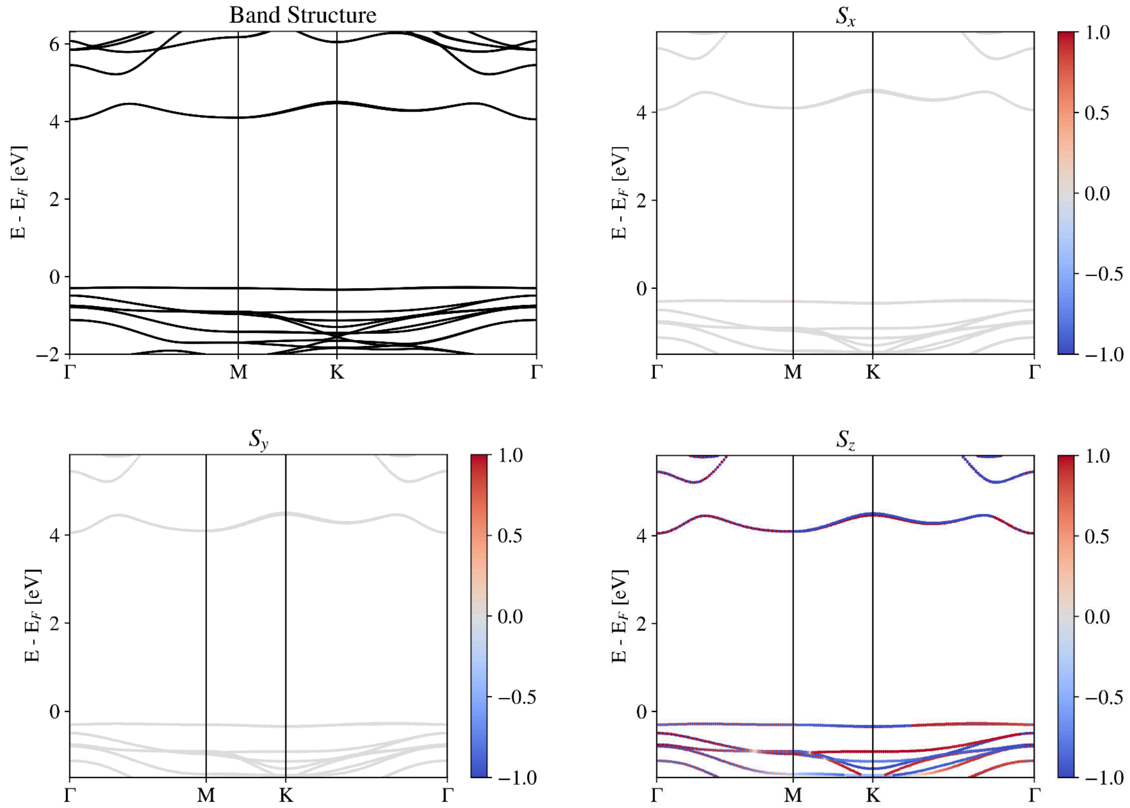


Figure 725: Band structure and spin polarization projections.

## 2.363 SrBr2-a4c9c803de7d

- **Formula**: SrBr2
- **Structural Cluster**: AB2-11
- **Band gap (PBE)**: 4.583 eV
- **Energy above convex hull (C2DB)**: 0.21 eV
- **Space group symbol**: $P\bar{4}m2$
- **Space group number**: 115
- **Polar structure**: False

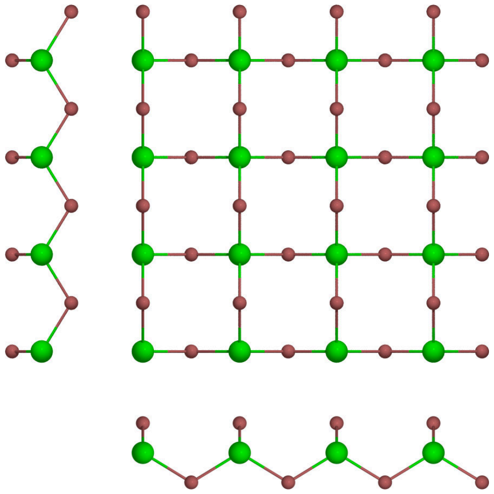

Figure 726: Structure representation

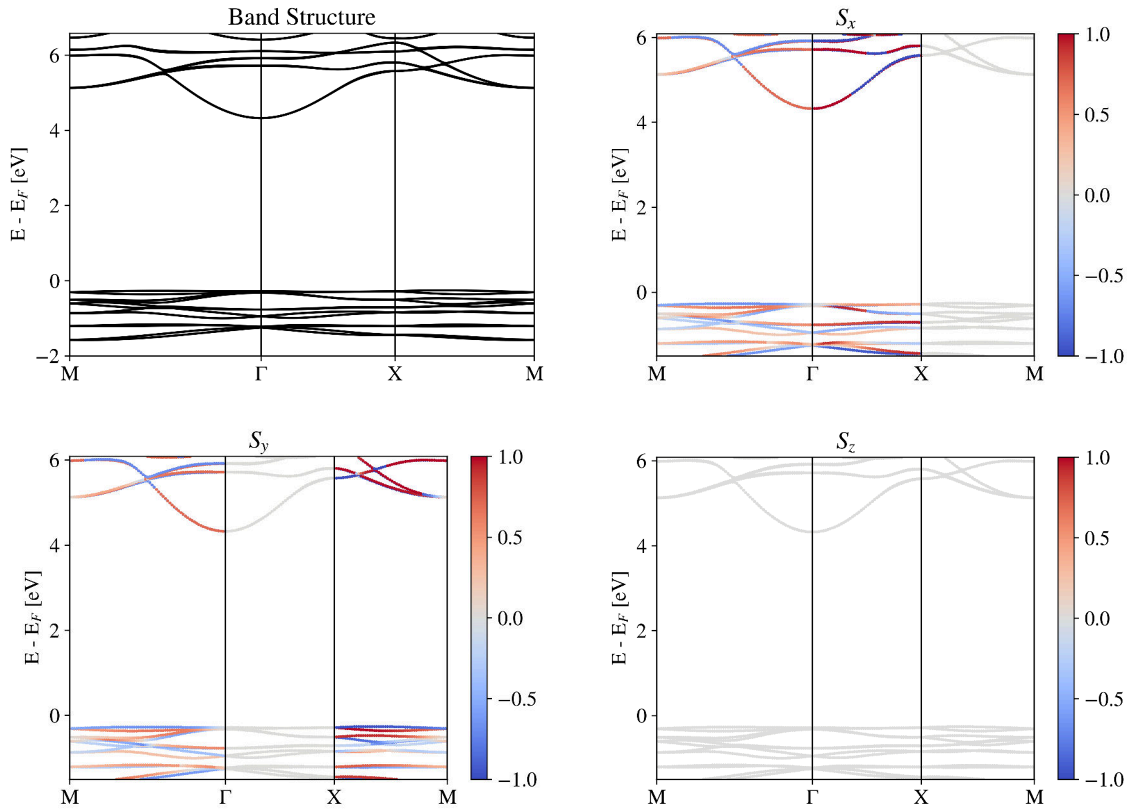


Figure 727: Band structure and spin polarization projections.

## 2.364 SrCl2-77398c835c11

- **Formula**: SrCl2
- **Structural Cluster**: AB2-4
- **Band gap (PBE)**: 4.958 eV
- **Energy above convex hull (C2DB)**: 0.194 eV
- **Space group symbol**: $P\bar{6}m2$
- **Space group number**: 187
- **Polar structure**: False

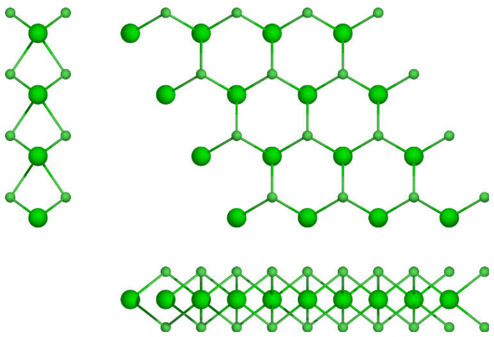

Figure 728: Structure representation

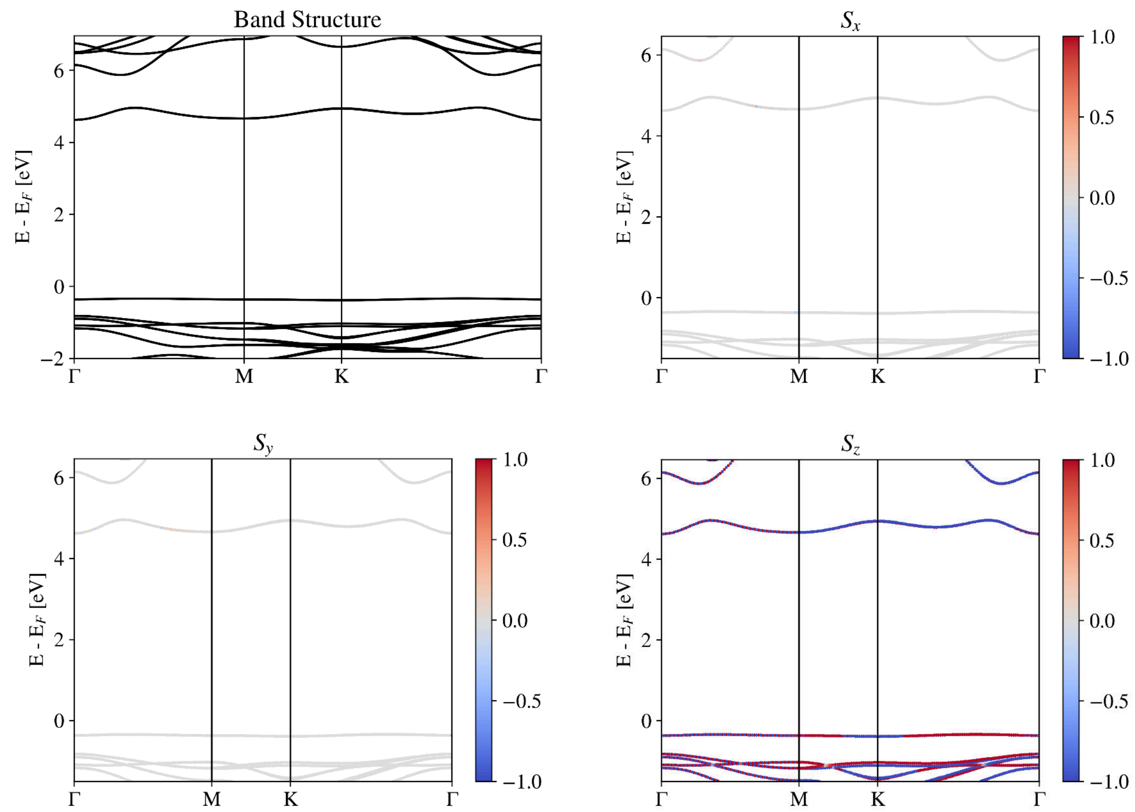


Figure 729: Band structure and spin polarization projections.

## 2.365 SrCl2-e03e4067ecb2

- **Formula**: SrCl2
- **Structural Cluster**: AB2-11
- **Band gap (PBE)**: 5.275 eV
- **Energy above convex hull (C2DB)**: 0.243 eV
- **Space group symbol**: $P\bar{4}m2$
- **Space group number**: 115
- **Polar structure**: False

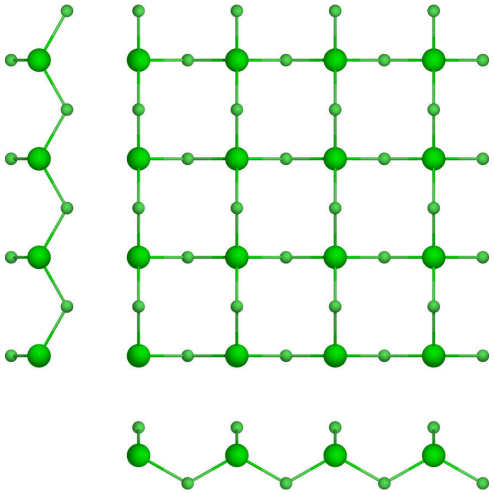

Figure 730: Structure representation

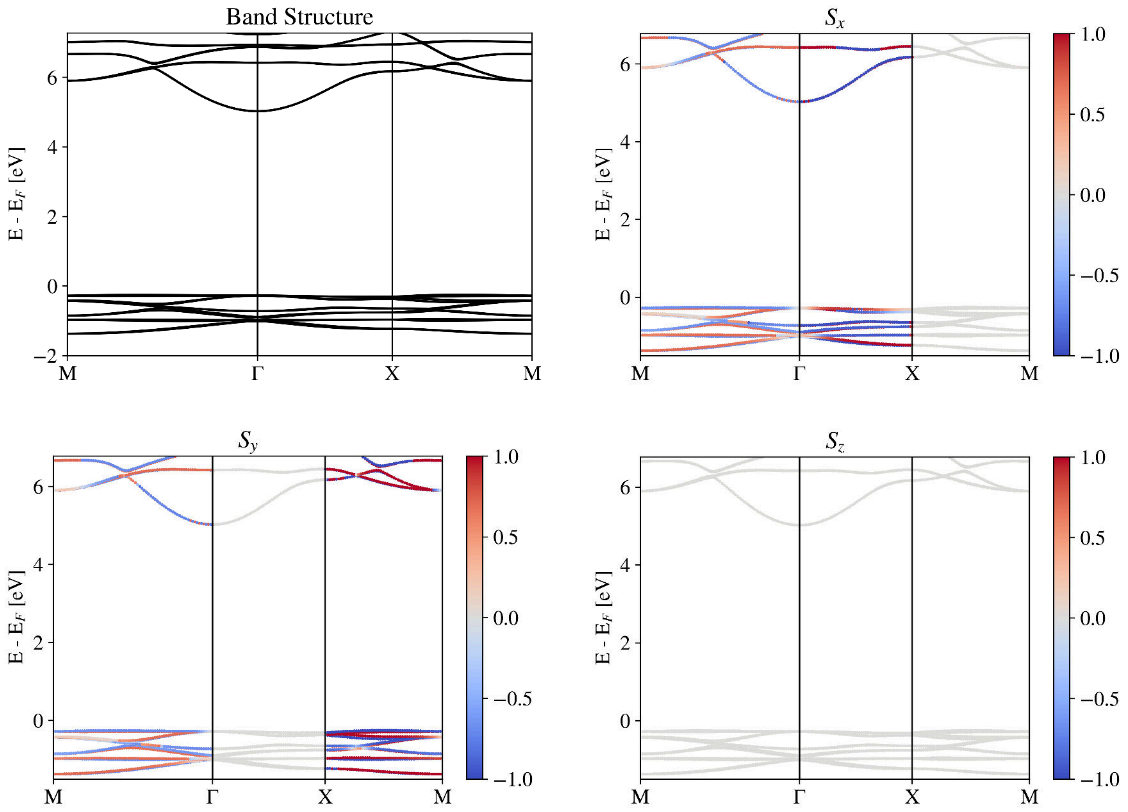


Figure 731: Band structure and spin polarization projections.

## 2.366 SrF2-c99805c05244

- **Formula**: SrF2
- **Structural Cluster**: AB2-11
- **Band gap (PBE)**: 6.025 eV
- **Energy above convex hull (C2DB)**: 0.376 eV
- **Space group symbol**: $P\bar{4}m2$
- **Space group number**: 115
- **Polar structure**: False

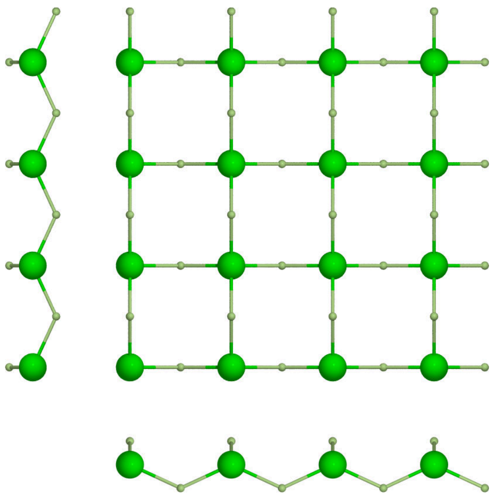

Figure 732: Structure representation

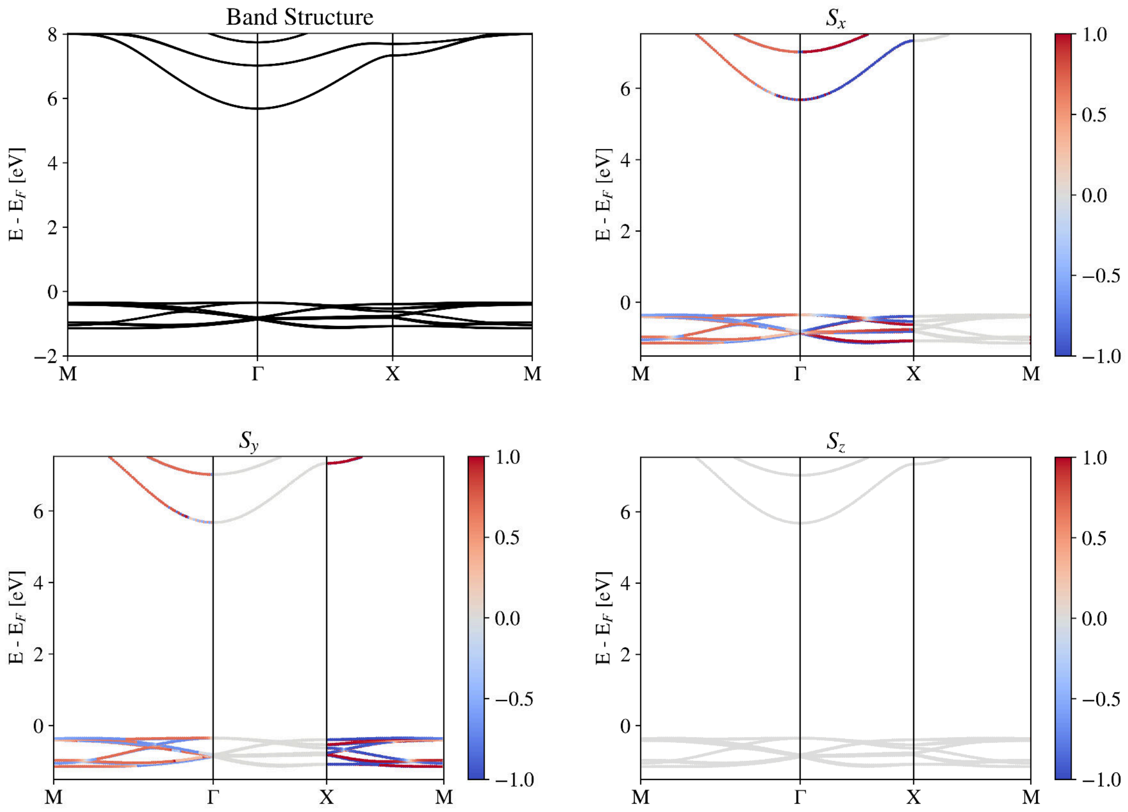


Figure 733: Band structure and spin polarization projections.

## 2.367 SrI2-1059dc7f5fe4

- **Formula**: SrI2
- **Structural Cluster**: AB2-11
- **Band gap (PBE)**: 3.964 eV
- **Energy above convex hull (C2DB)**: 0.188 eV
- **Space group symbol**: $P\bar{4}m2$
- **Space group number**: 115
- **Polar structure**: False

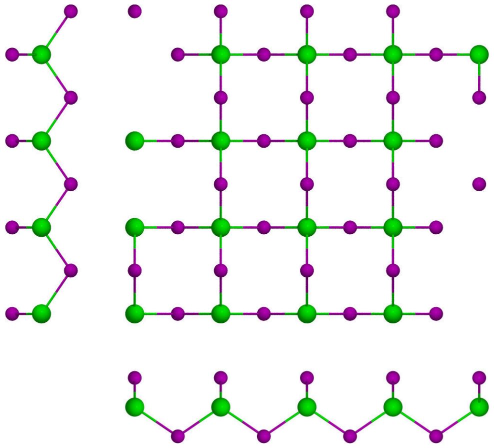

Figure 734: Structure representation

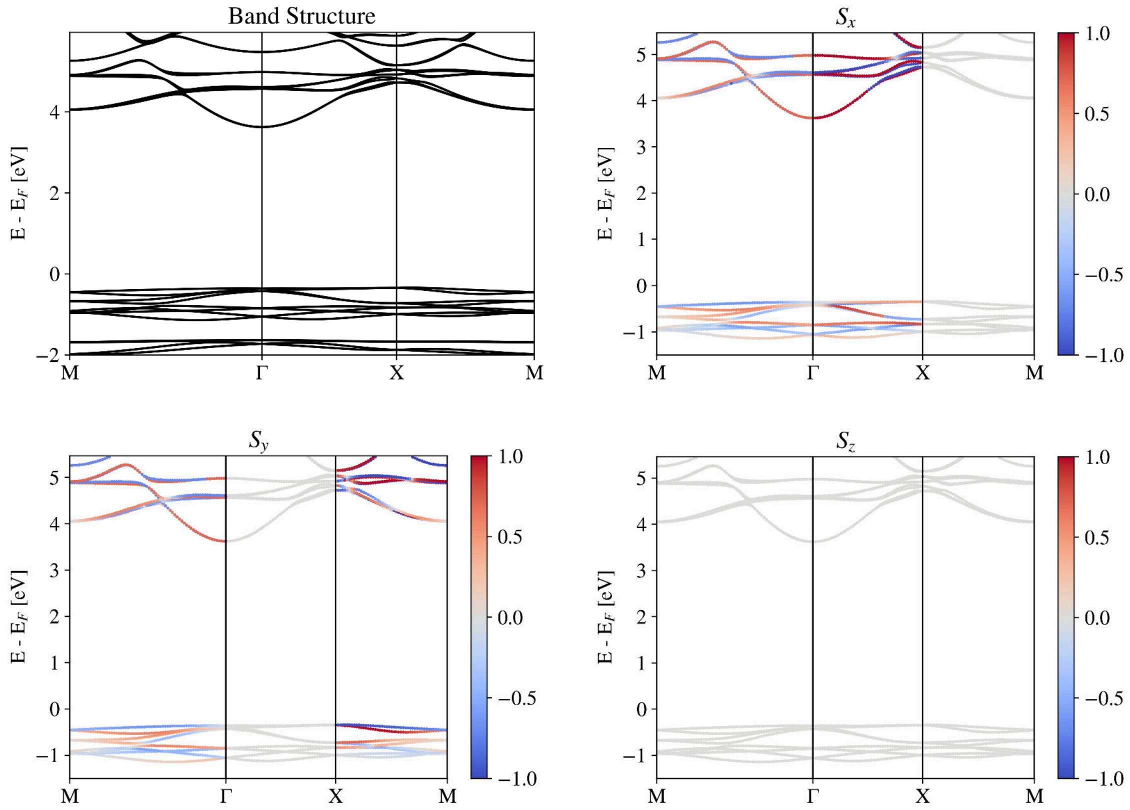


Figure 735: Band structure and spin polarization projections.

## 2.368 SrI2-6cfaae647808

- **Formula**: SrI2
- **Structural Cluster**: AB2-4
- **Band gap (PBE)**: 3.448 eV
- **Energy above convex hull (C2DB)**: 0.096 eV
- **Space group symbol**: $P\bar{6}m2$
- **Space group number**: 187
- **Polar structure**: False

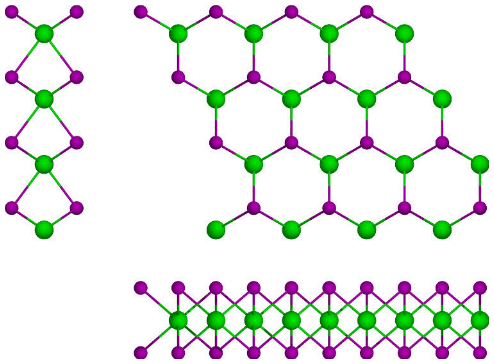

Figure 736: Structure representation

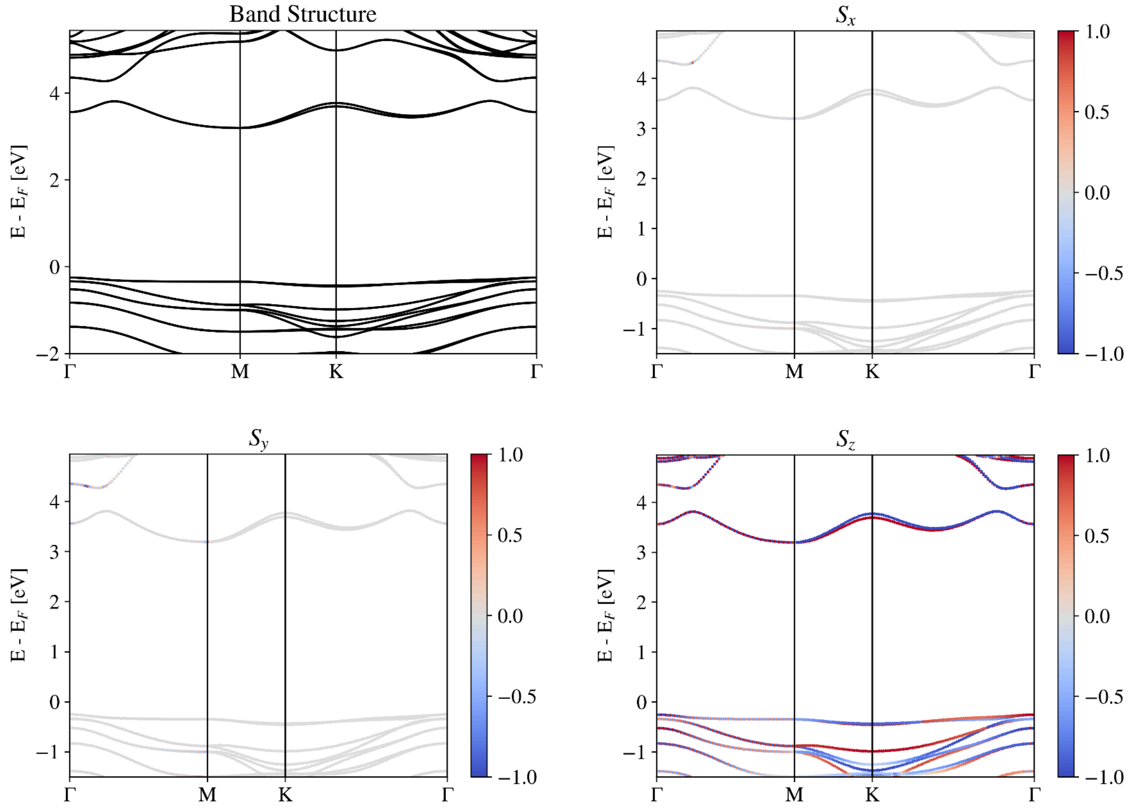


Figure 737: Band structure and spin polarization projections.

## 2.369 Te2Ti2-b43c14735d8e

- **Formula**: Te2Ti2
- **Structural Cluster**: AB-5
- **Band gap (PBE)**: 0.232 eV
- **Energy above convex hull (C2DB)**: 0.61 eV
- **Space group symbol**: $P\bar{6}m2$
- **Space group number**: 187
- **Polar structure**: False

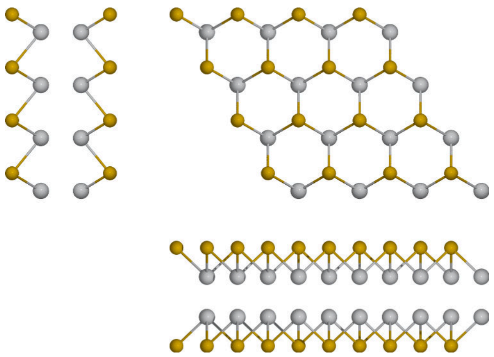

Figure 738: Structure representation

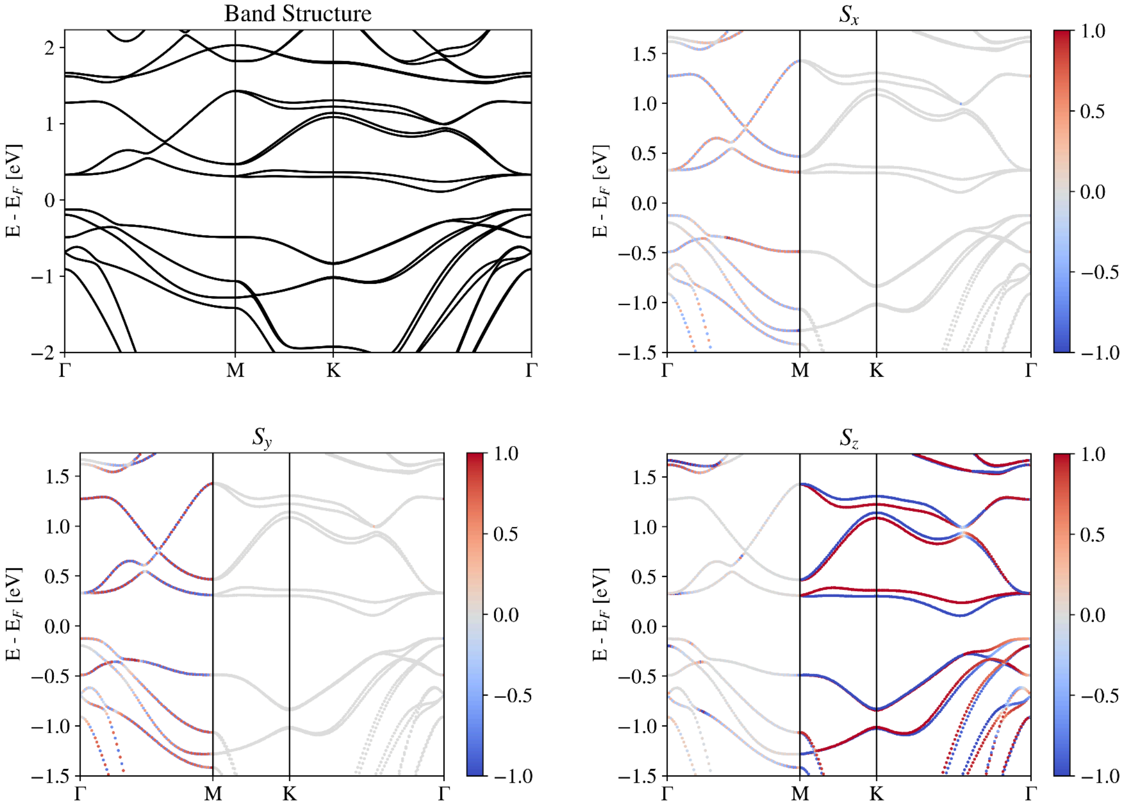


Figure 739: Band structure and spin polarization projections.

## 2.370 Te2Ti2-ecf4b958e9a1

- **Formula**: Te2Ti2
- **Structural Cluster**: AB-5
- **Band gap (PBE)**: 0.078 eV
- **Energy above convex hull (C2DB)**: 0.626 eV
- **Space group symbol**: *Cm*
- **Space group number**: 8
- **Polar structure**: True

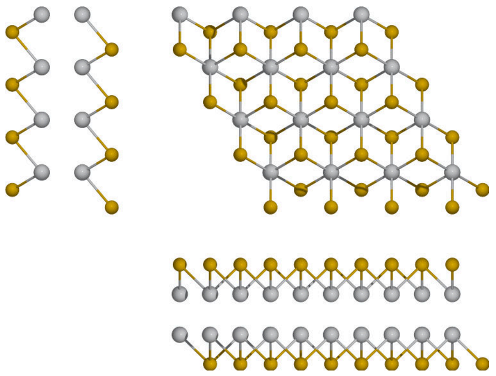

Figure 740: Structure representation

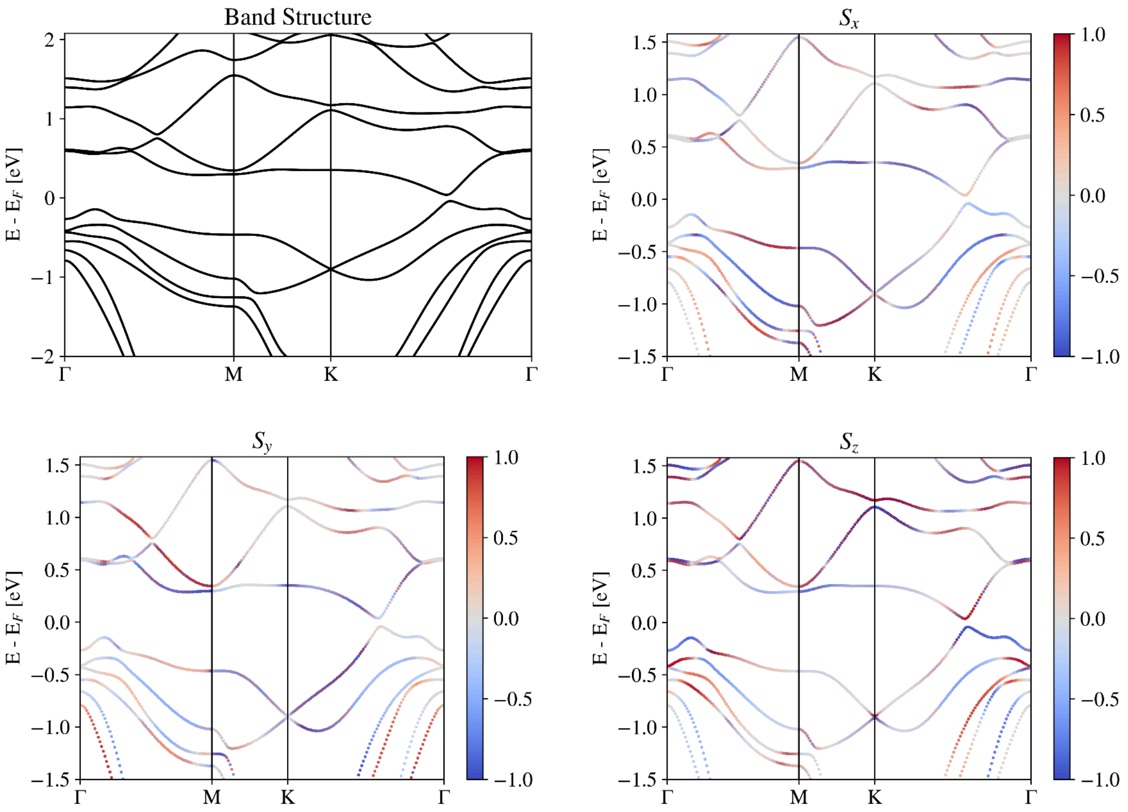


Figure 741: Band structure and spin polarization projections.

## 2.371 Te2Tl2-44ffc9d405b8

- **Formula**: Te2Tl2
- **Structural Cluster**: AB-5
- **Band gap (PBE)**: 0.322 eV
- **Energy above convex hull (C2DB)**: 0.139 eV
- **Space group symbol**: $C2$
- **Space group number**: 5
- **Polar structure**: True

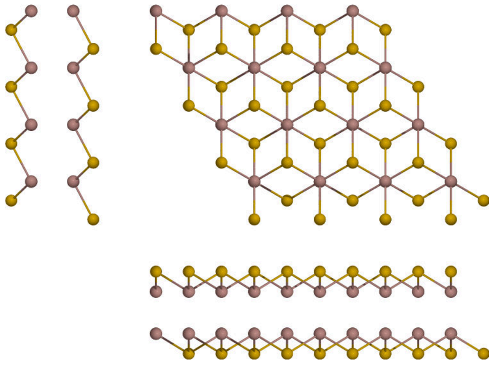

Figure 742: Structure representation

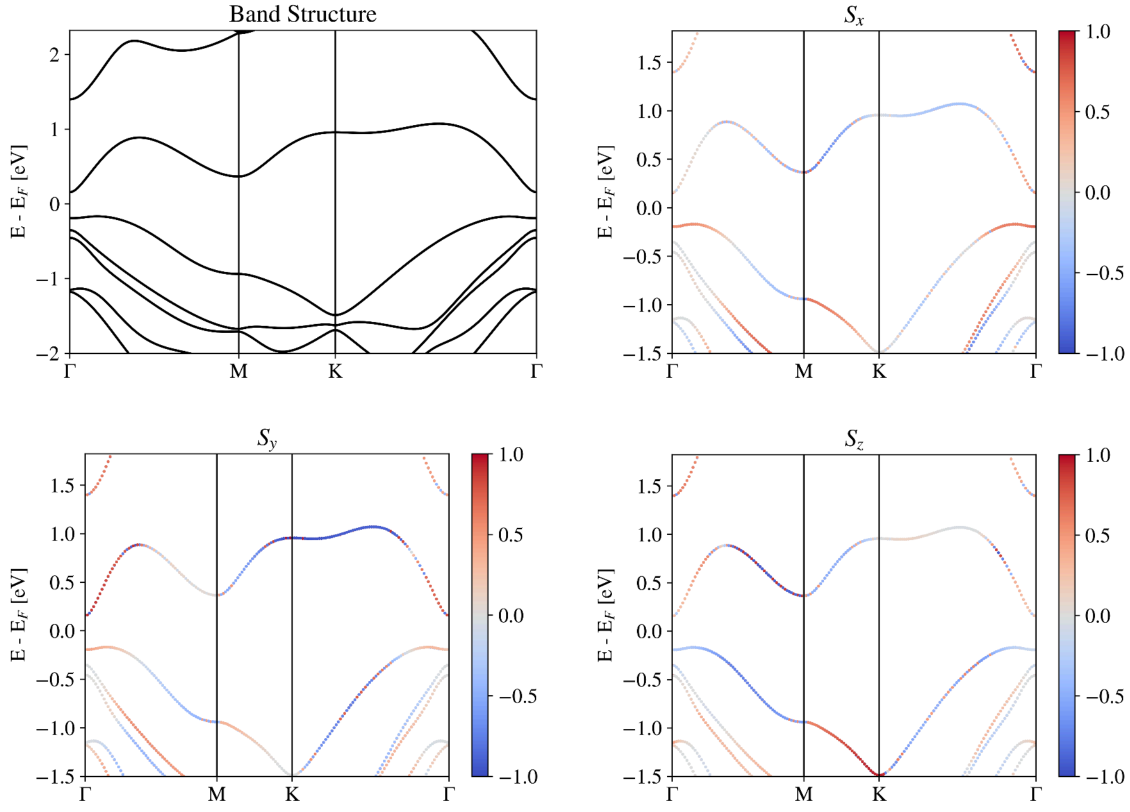


Figure 743: Band structure and spin polarization projections.

## 2.372 Te2Tl2-73117163f0e2

- **Formula**: Te2Tl2
- **Structural Cluster**: AB-5
- **Band gap (PBE)**: 0.367 eV
- **Energy above convex hull (C2DB)**: 0.137 eV
- **Space group symbol**: $P\bar{6}m2$
- **Space group number**: 187
- **Polar structure**: False

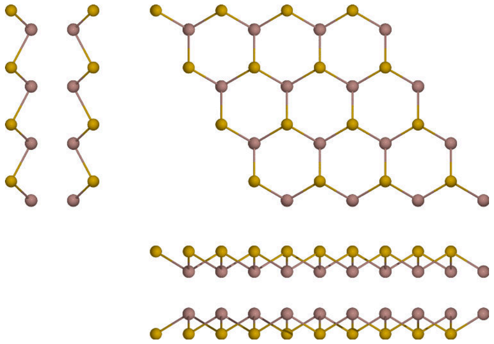

Figure 744: Structure representation

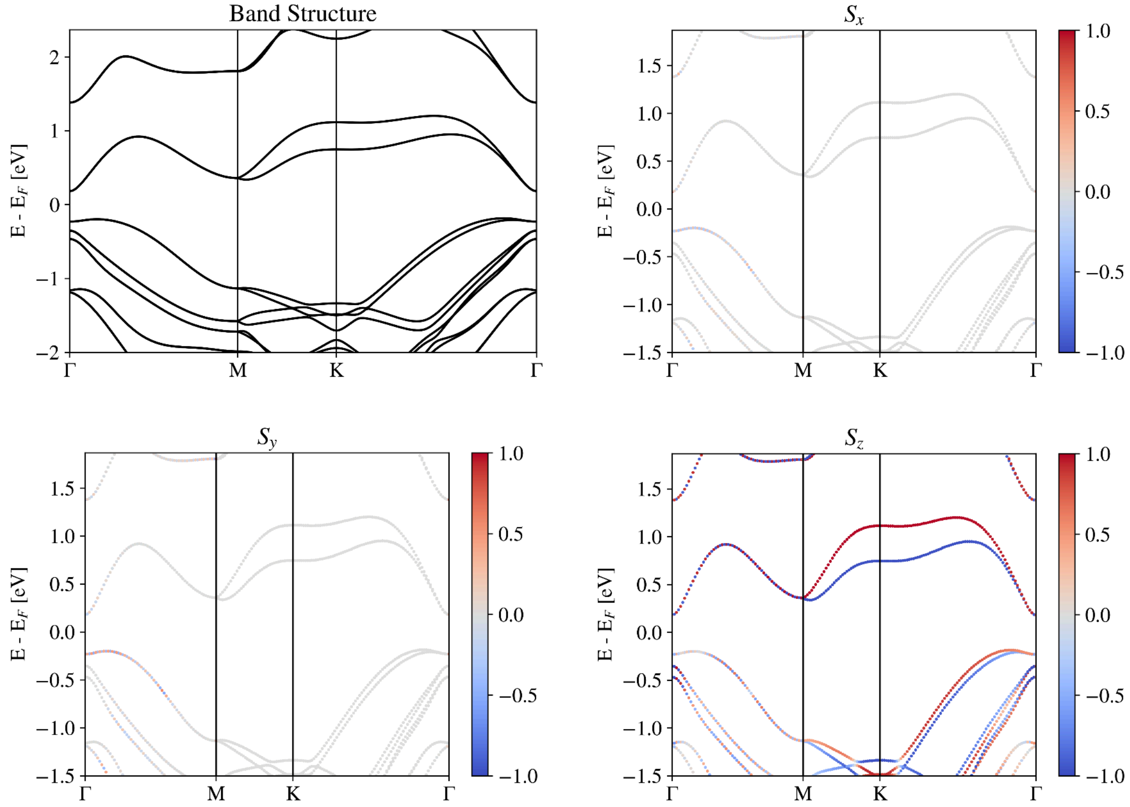


Figure 745: Band structure and spin polarization projections.

## 2.373 Te2V2-5da53e6996e3

- **Formula**: Te2V2
- **Structural Cluster**: AB-5
- **Band gap (PBE)**: 0.22 eV
- **Energy above convex hull (C2DB)**: 0.49 eV
- **Space group symbol**: $P\bar{6}m2$
- **Space group number**: 187
- **Polar structure**: False

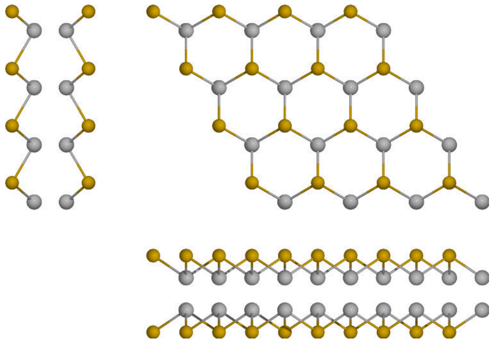

Figure 746: Structure representation

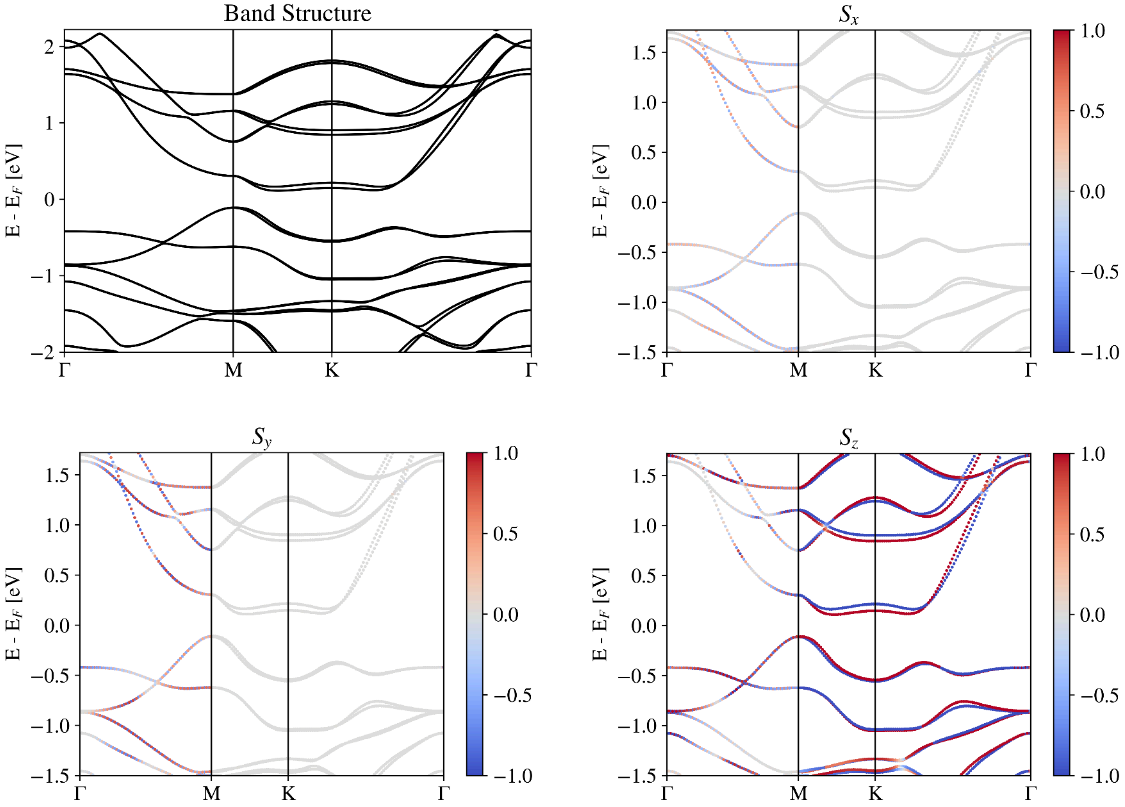


Figure 747: Band structure and spin polarization projections.

## 2.374 Te2Zr2-8912432cb37b

- **Formula**: Te2Zr2
- **Structural Cluster**: AB-5
- **Band gap (PBE)**: 0.458 eV
- **Energy above convex hull (C2DB)**: 0.624 eV
- **Space group symbol**: $P\bar{6}m2$
- **Space group number**: 187
- **Polar structure**: False

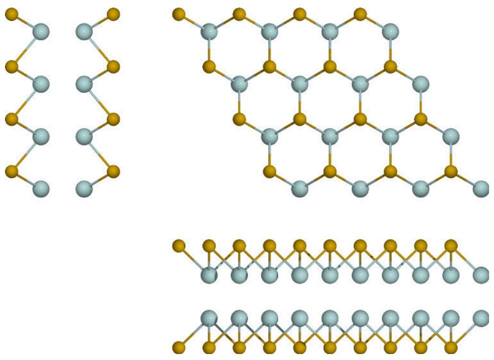

Figure 748: Structure representation

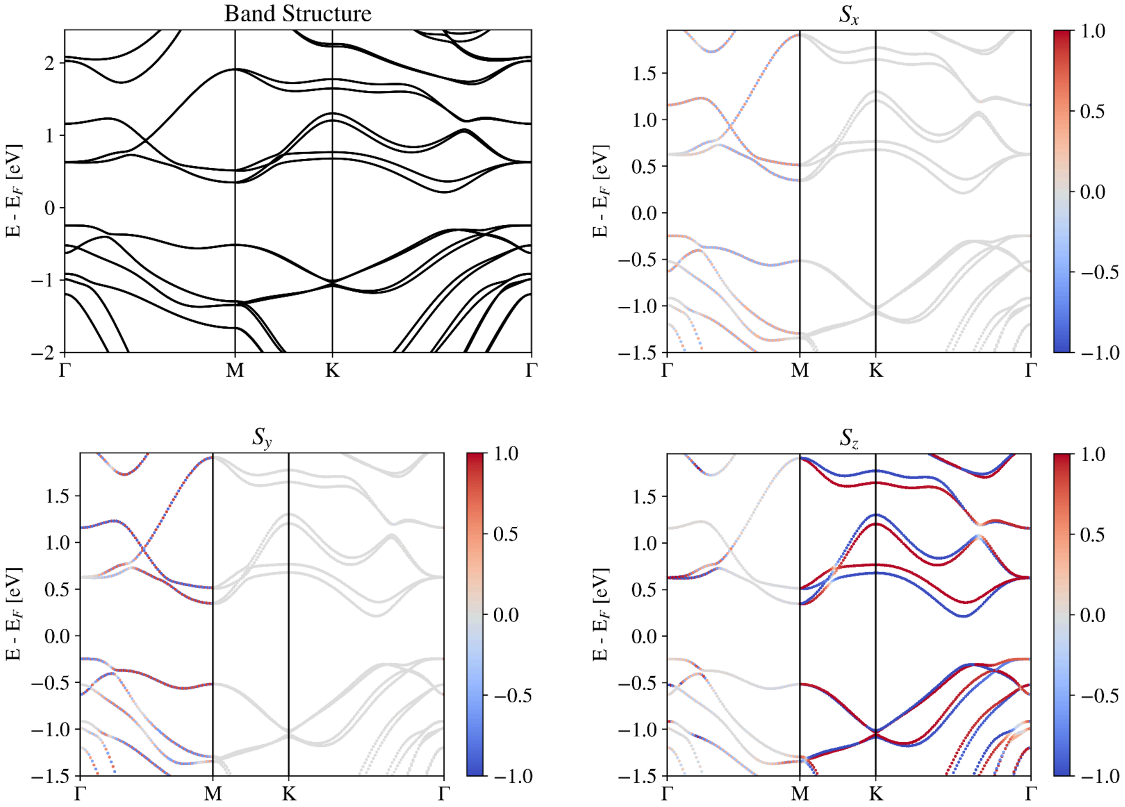


Figure 749: Band structure and spin polarization projections.

## 2.375 Ti2Zr2S8-a99139546333

- **Formula**: Ti2Zr2S8
- **Structural Cluster**: ABC4-22
- **Band gap (PBE)**: 0.842 eV
- **Energy above convex hull (C2DB)**: 0.18 eV
- **Space group symbol**: $P1$
- **Space group number**: 1
- **Polar structure**: True

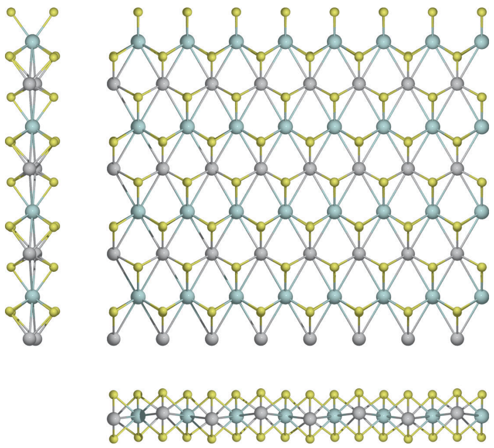

Figure 750: Structure representation

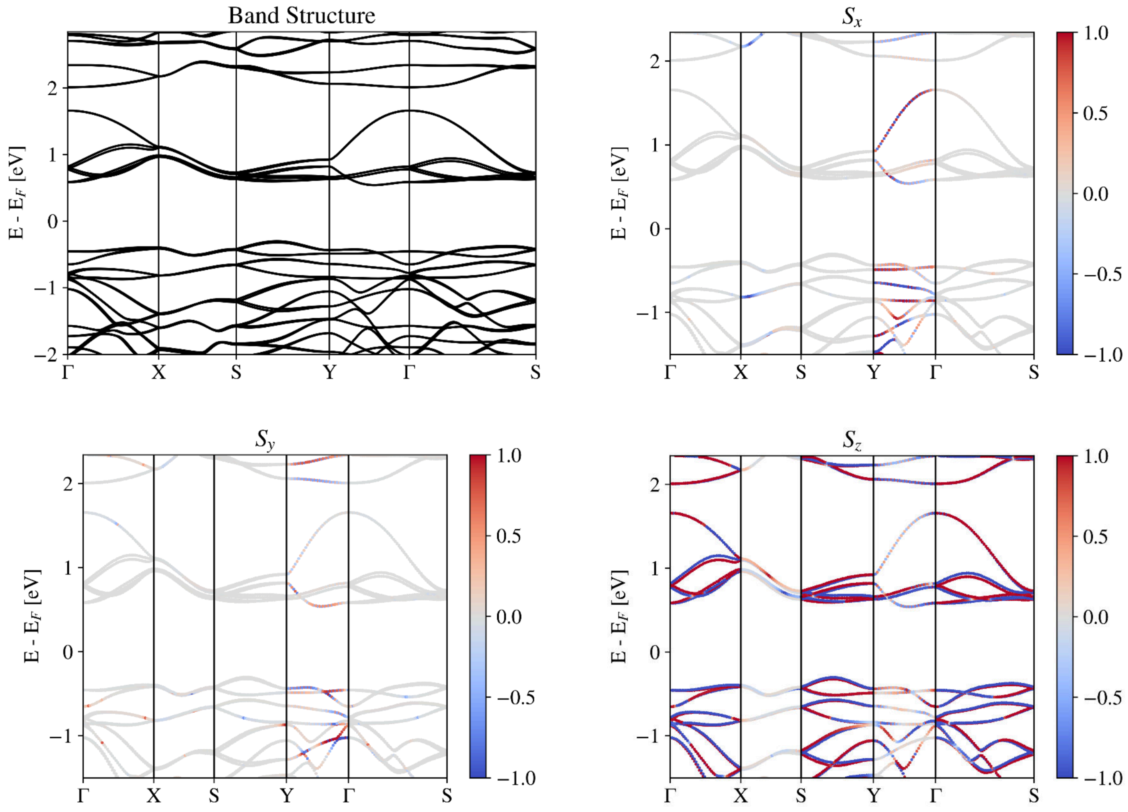


Figure 751: Band structure and spin polarization projections.

## 2.376 Ti2Zr2Se8-846b50801a93

- **Formula**: Ti2Zr2Se8
- **Structural Cluster**: ABC4-22
- **Band gap (PBE)**: 0.616 eV
- **Energy above convex hull (C2DB)**: 0.142 eV
- **Space group symbol**: $P1$
- **Space group number**: 1
- **Polar structure**: True

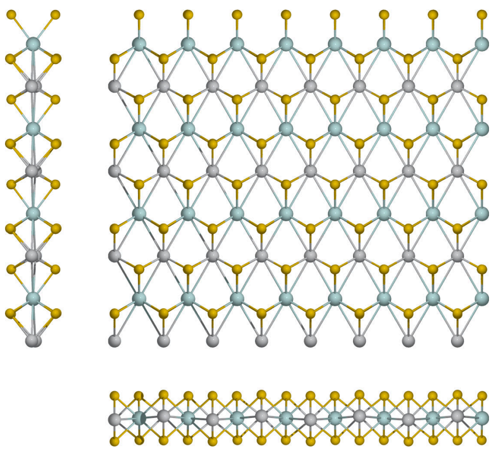

Figure 752: Structure representation

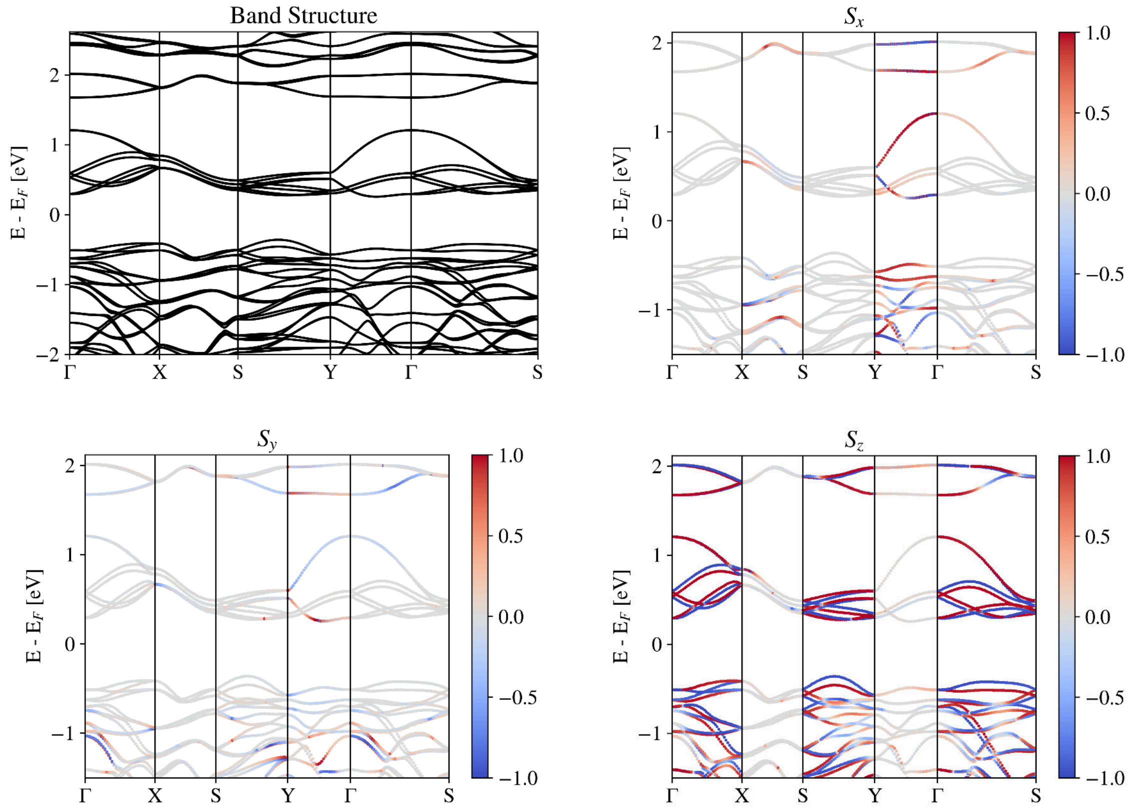


Figure 753: Band structure and spin polarization projections.

## 2.377 Ti2Zr2Te8-18e377cce57f

- **Formula**: Ti2Zr2Te8
- **Structural Cluster**: ABC4-22
- **Band gap (PBE)**: 0.143 eV
- **Energy above convex hull (C2DB)**: 0.118 eV
- **Space group symbol**: $P1$
- **Space group number**: 1
- **Polar structure**: True

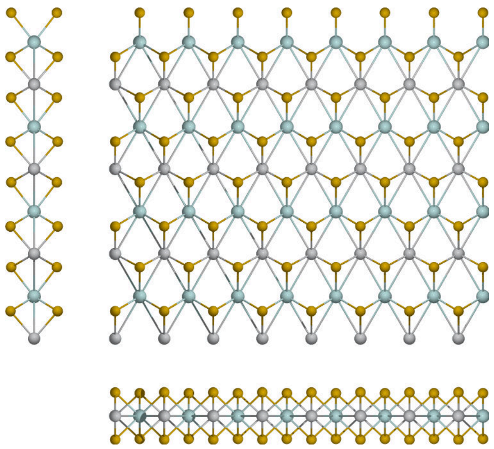

Figure 754: Structure representation

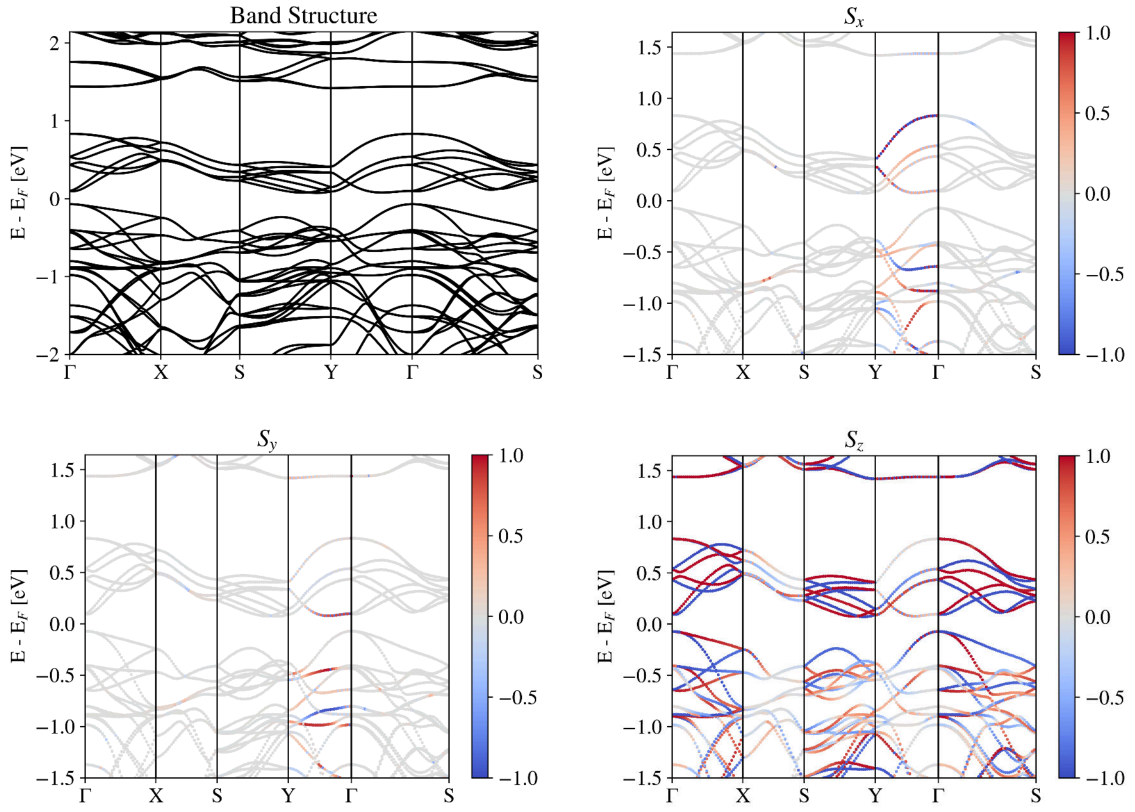


Figure 755: Band structure and spin polarization projections.

## 2.378 TiBr2-57116f9a9a4e

- **Formula**: TiBr2
- **Structural Cluster**: AB2-4
- **Band gap (PBE)**: 0.756 eV
- **Energy above convex hull (C2DB)**: 0.0 eV
- **Space group symbol**: $P\bar{6}m2$
- **Space group number**: 187
- **Polar structure**: False

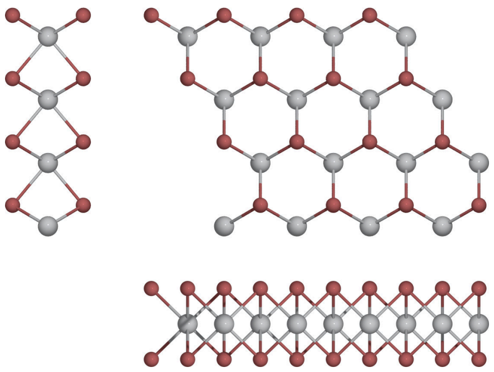

Figure 756: Structure representation

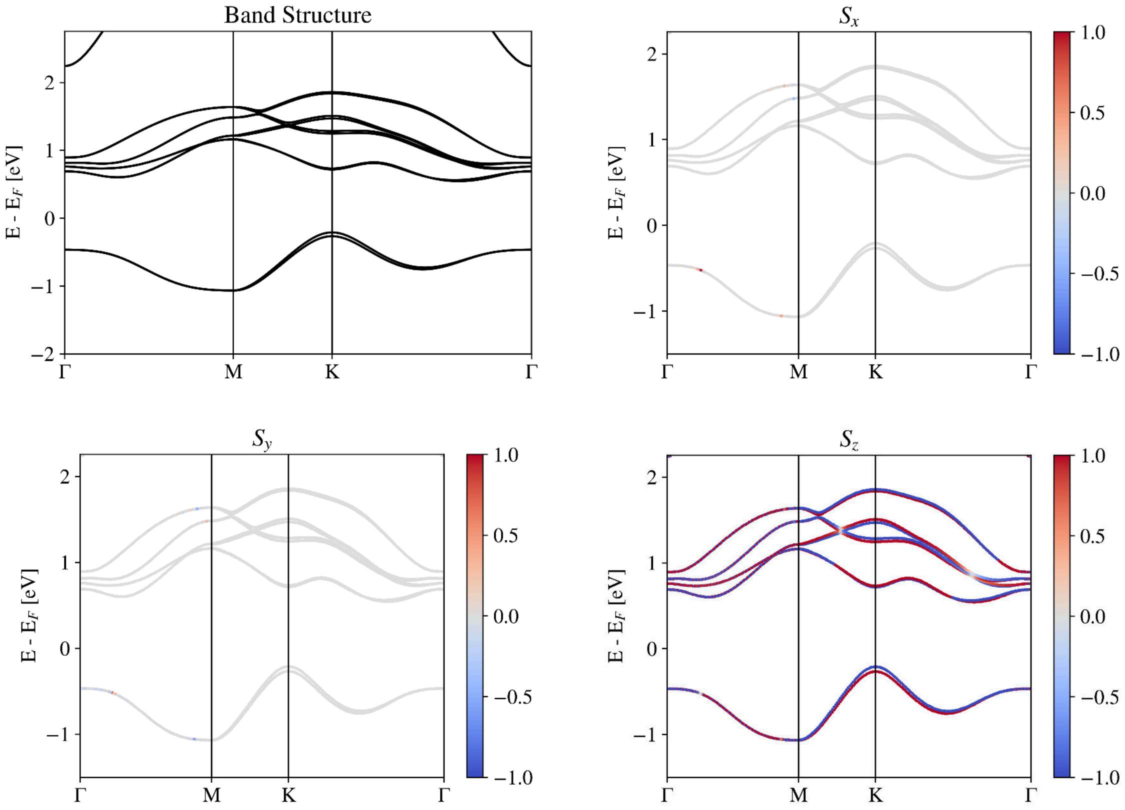


Figure 757: Band structure and spin polarization projections.

## 2.379 TiCl2-95688ba68ca1

- **Formula**: TiCl2
- **Structural Cluster**: AB2-4
- **Band gap (PBE)**: 0.901 eV
- **Energy above convex hull (C2DB)**: 0.001 eV
- **Space group symbol**: $P\bar{6}m2$
- **Space group number**: 187
- **Polar structure**: False

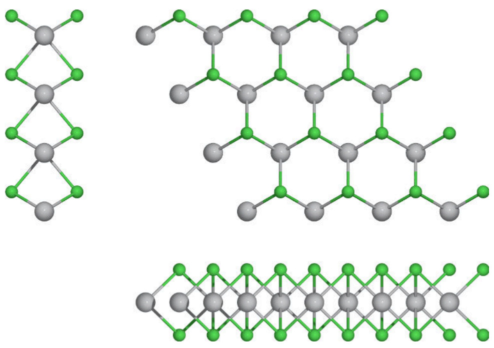

Figure 758: Structure representation

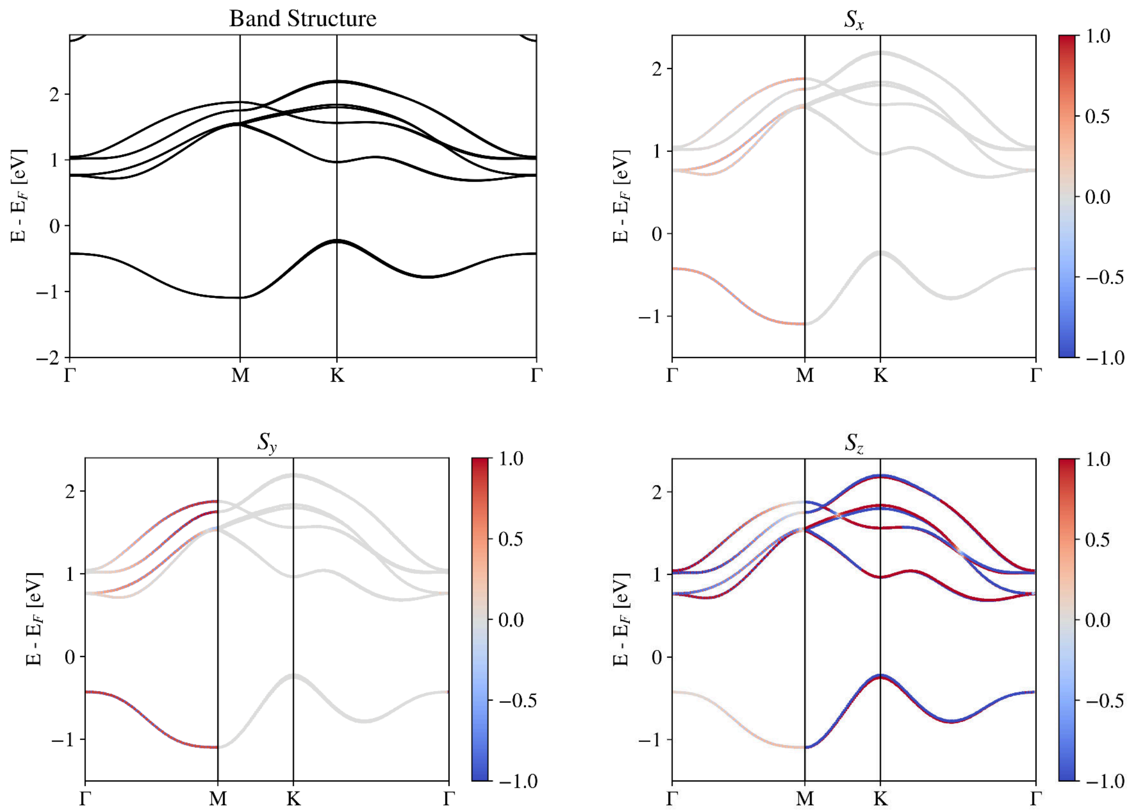


Figure 759: Band structure and spin polarization projections.

## 2.380 TiHf3S8-eb71cb1c9077

- **Formula**: TiHf3S8
- **Structural Cluster**: AB3C8-22
- **Band gap (PBE)**: 1.005 eV
- **Energy above convex hull (C2DB)**: 0.205 eV
- **Space group symbol**: $P1$
- **Space group number**: 1
- **Polar structure**: True

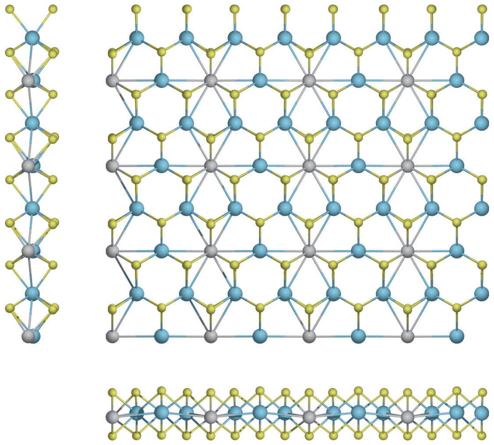

Figure 760: Structure representation

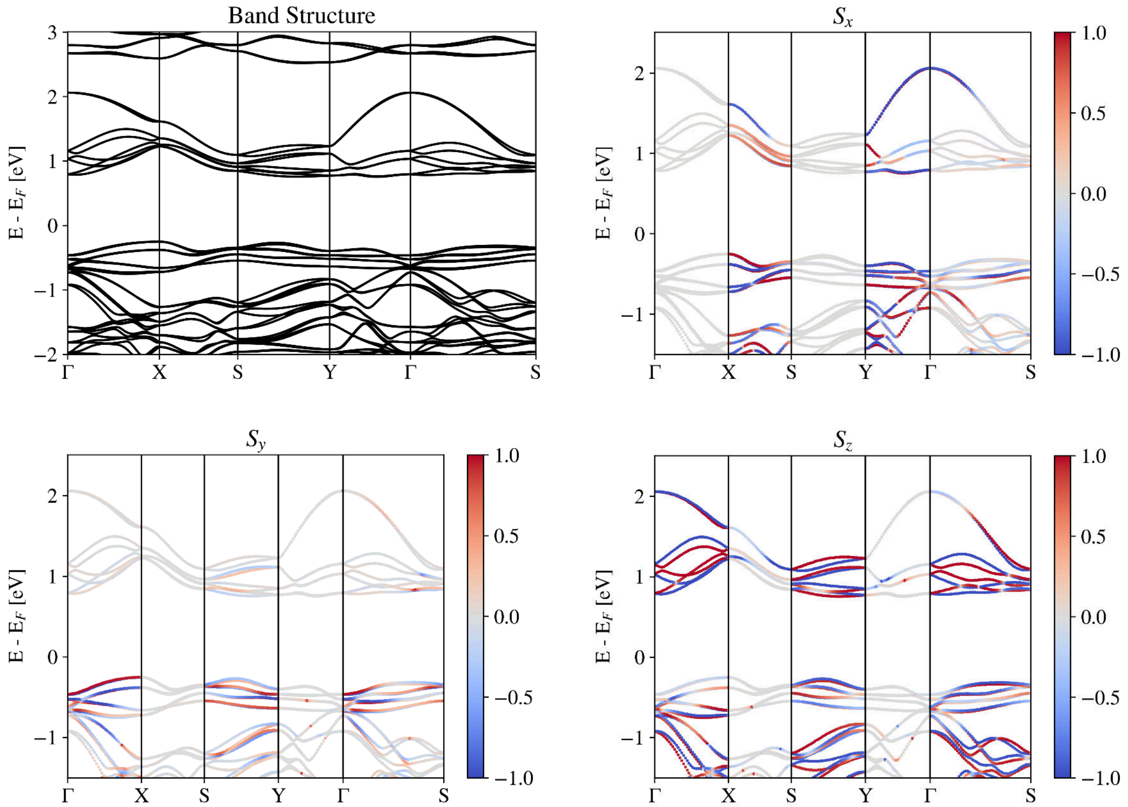


Figure 761: Band structure and spin polarization projections.

## 2.381 TiHf3Se8-3e1923c616ad

- **Formula**: TiHf3Se8
- **Structural Cluster**: AB3C8-22
- **Band gap (PBE)**: 0.722 eV
- **Energy above convex hull (C2DB)**: 0.166 eV
- **Space group symbol**: $P1$
- **Space group number**: 1
- **Polar structure**: True

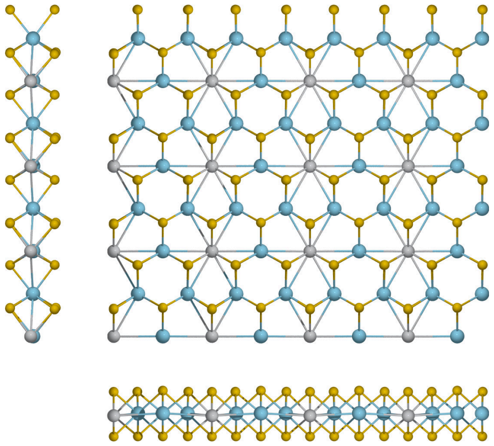

Figure 762: Structure representation

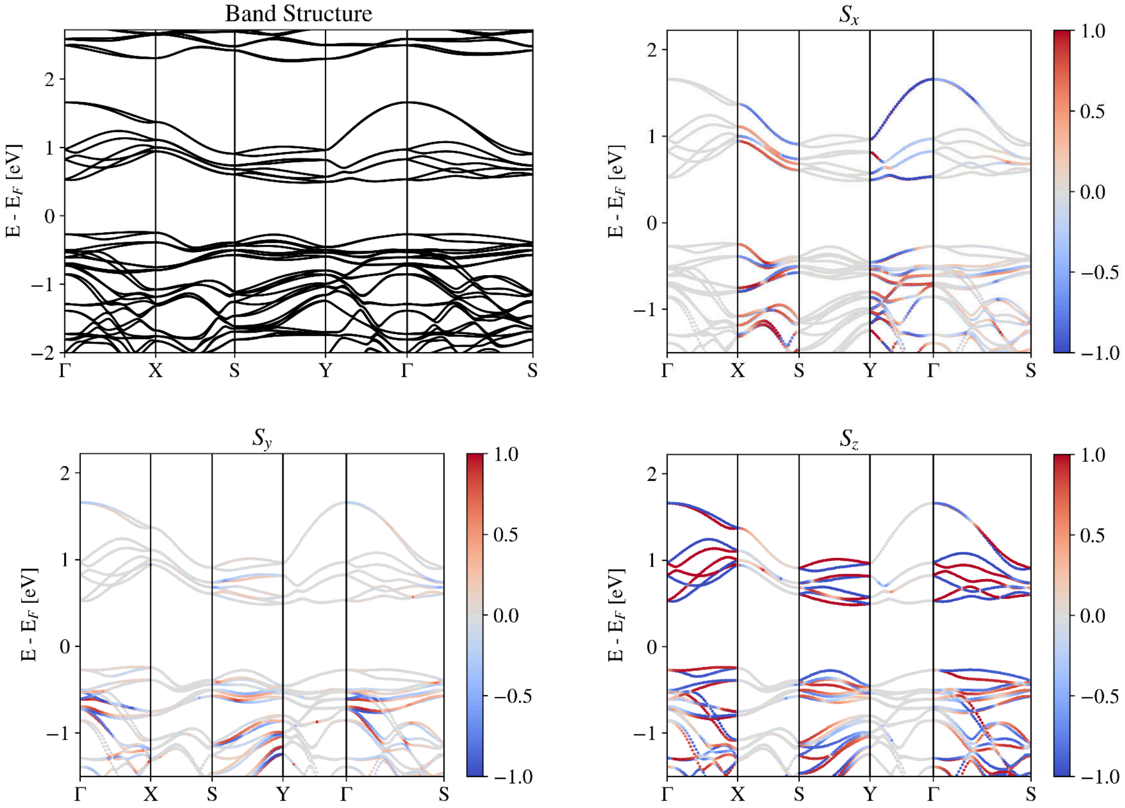


Figure 763: Band structure and spin polarization projections.

## 2.382 TiHf3Te8-1667d1443160

- **Formula**: TiHf3Te8
- **Structural Cluster**: AB3C8-22
- **Band gap (PBE)**: 0.098 eV
- **Energy above convex hull (C2DB)**: 0.132 eV
- **Space group symbol**: $P1$
- **Space group number**: 1
- **Polar structure**: True

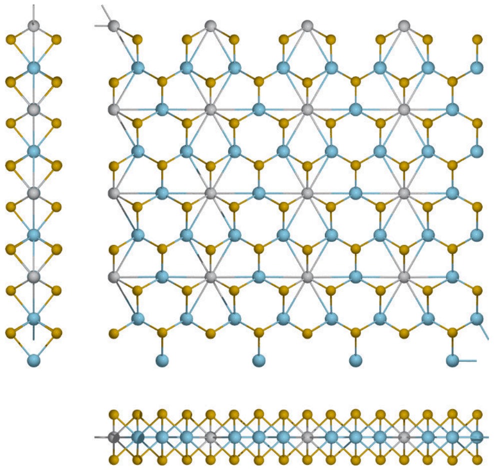

Figure 764: Structure representation

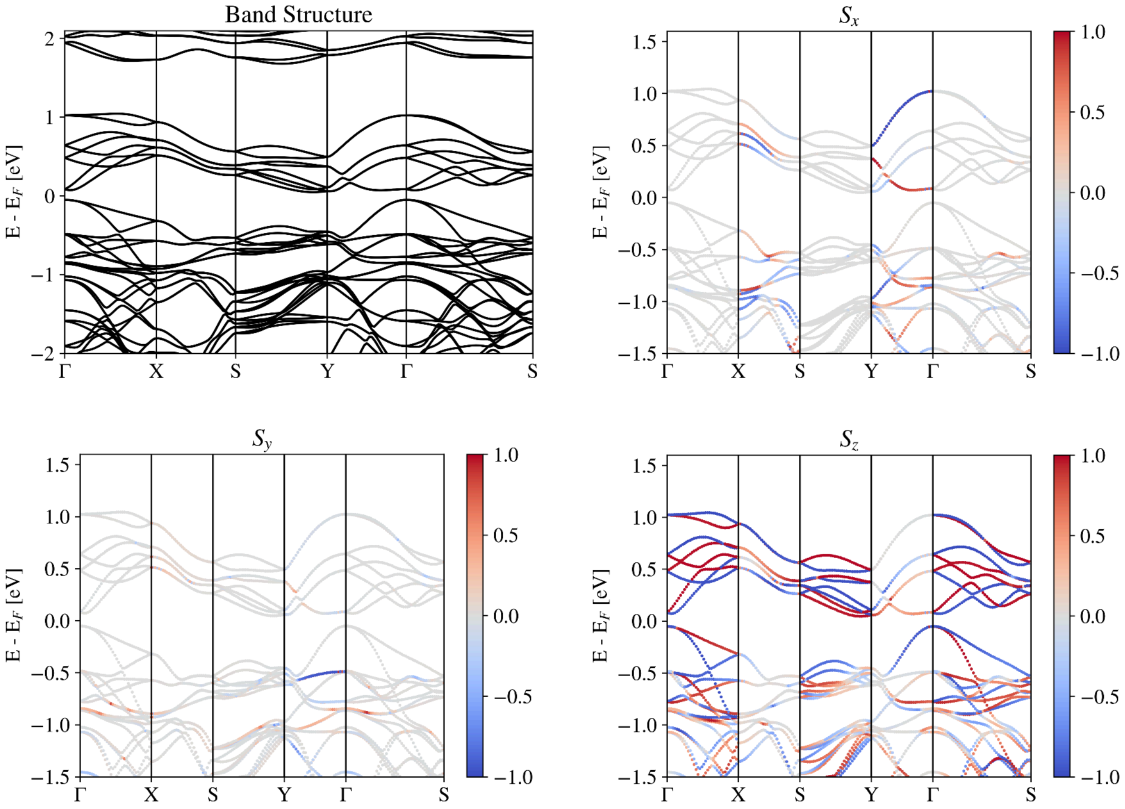


Figure 765: Band structure and spin polarization projections.

## 2.383 TiI2-088e8488f895

- **Formula**: TiI2
- **Structural Cluster**: AB2-4
- **Band gap (PBE)**: 0.602 eV
- **Energy above convex hull (C2DB)**: 0.052 eV
- **Space group symbol**: $P\bar{6}m2$
- **Space group number**: 187
- **Polar structure**: False

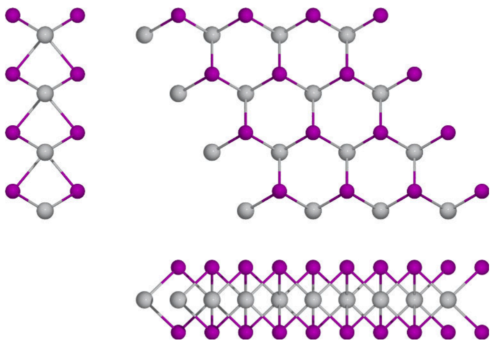

Figure 766: Structure representation

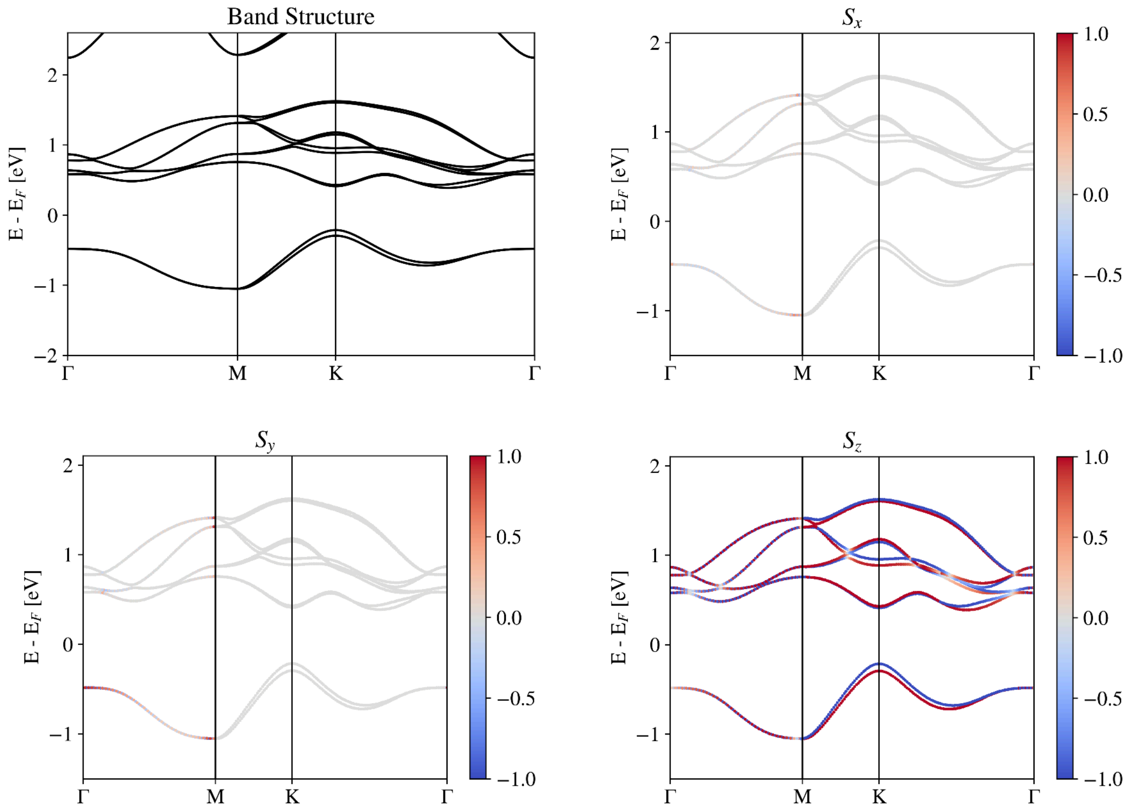


Figure 767: Band structure and spin polarization projections.

## 2.384 TiO2-1cfb690281c9

- **Formula**: TiO2
- **Structural Cluster**: AB2-4
- **Band gap (PBE)**: 1.136 eV
- **Energy above convex hull (C2DB)**: 0.587 eV
- **Space group symbol**: $P\bar{6}m2$
- **Space group number**: 187
- **Polar structure**: False

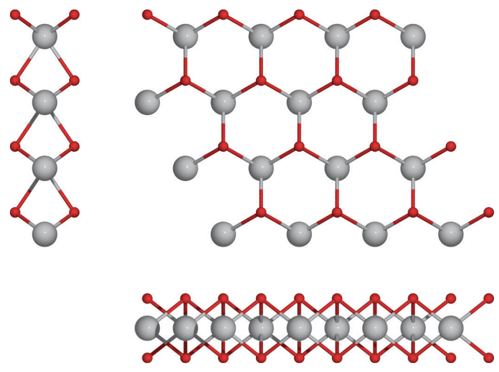

Figure 768: Structure representation

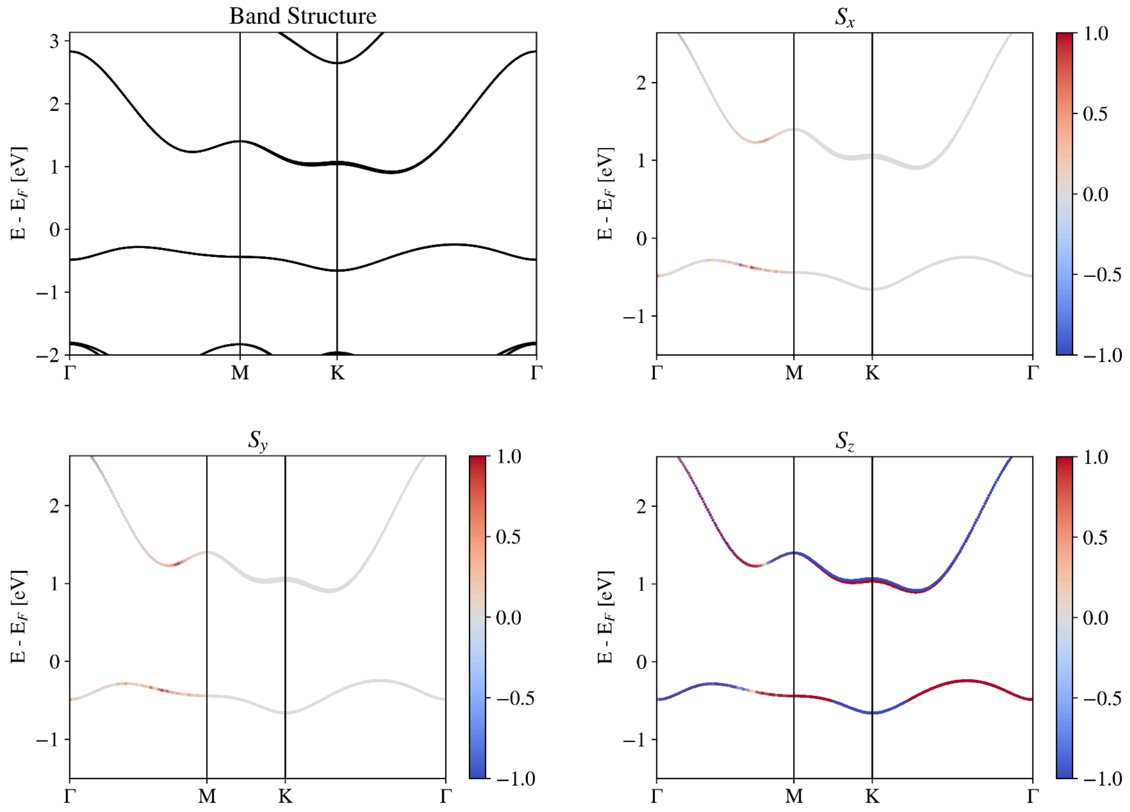


Figure 769: Band structure and spin polarization projections.

## 2.385 TiO2-badf6957f0bb

- **Formula**: TiO2
- **Structural Cluster**: AB2-11
- **Band gap (PBE)**: 3.422 eV
- **Energy above convex hull (C2DB)**: 0.213 eV
- **Space group symbol**: $P\bar{4}m2$
- **Space group number**: 115
- **Polar structure**: False

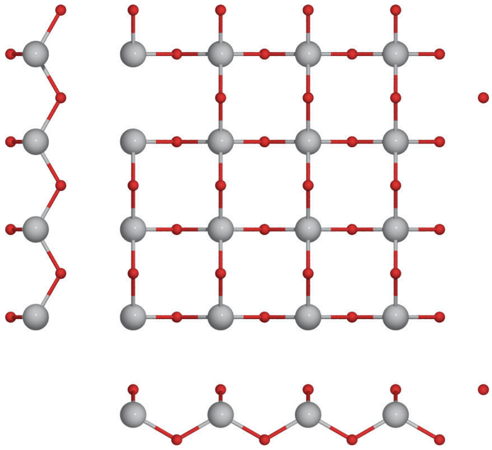

Figure 770: Structure representation

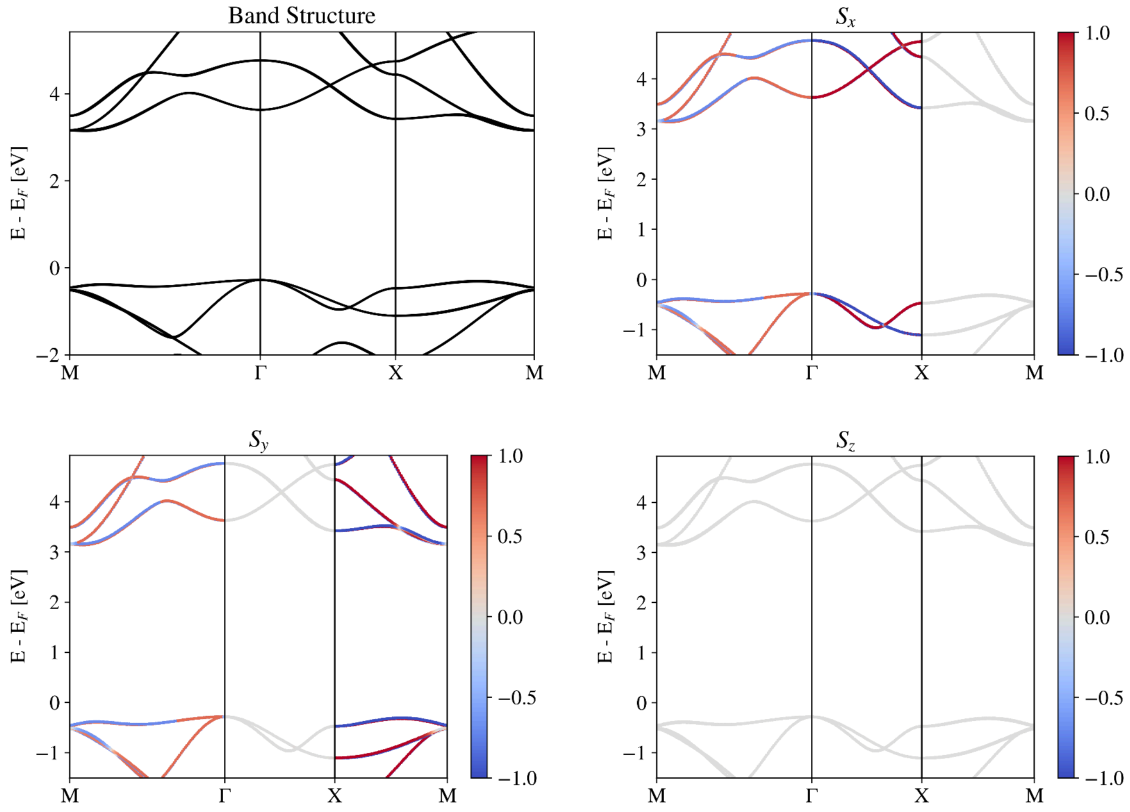


Figure 771: Band structure and spin polarization projections.

### 2.386 TiS2-0428a52595e8

- **Formula**: TiS2
- **Structural Cluster**: AB2-11
- **Band gap (PBE)**: 1.248 eV
- **Energy above convex hull (C2DB)**: 0.236 eV
- **Space group symbol**: $P\bar{4}m2$
- **Space group number**: 115
- **Polar structure**: False

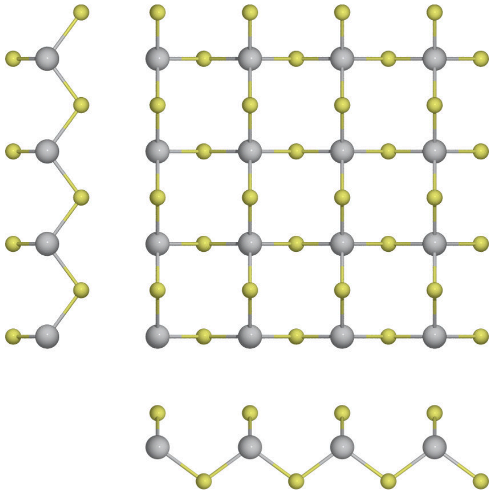

Figure 772: Structure representation

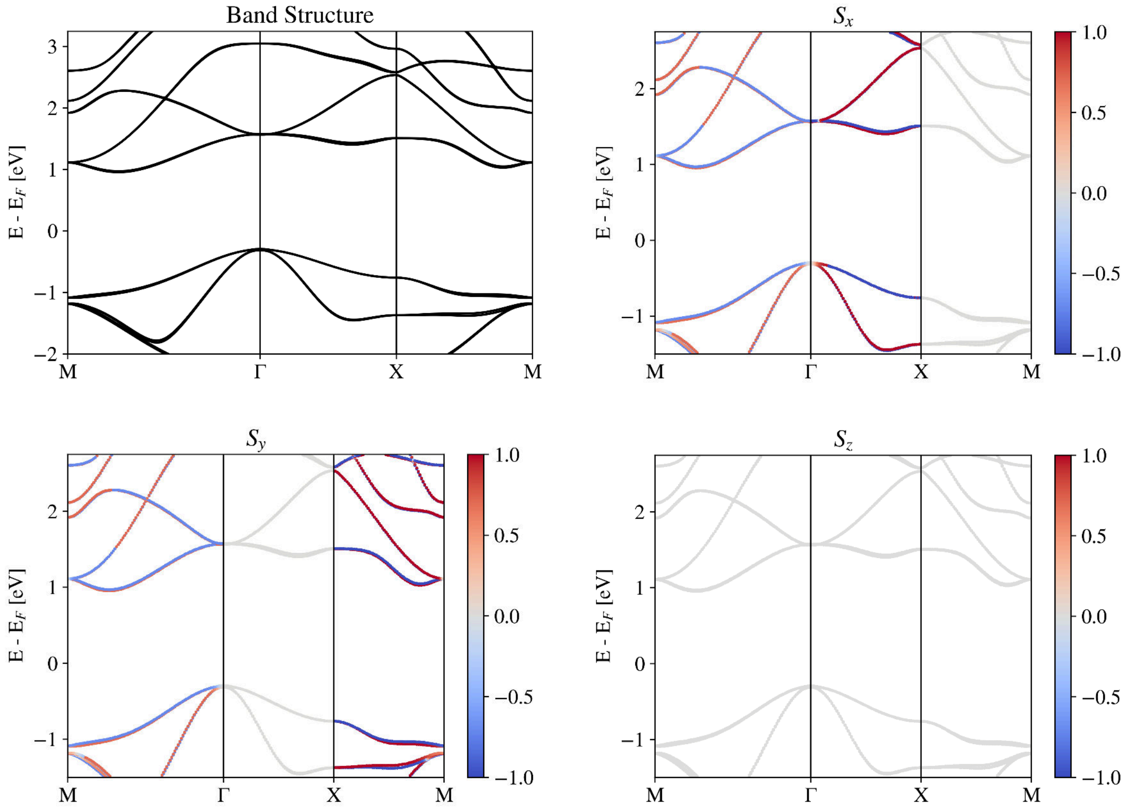


Figure 773: Band structure and spin polarization projections.

## 2.387 TiS2-65d41aaec667

- **Formula**: TiS2
- **Structural Cluster**: AB2-4
- **Band gap (PBE)**: 0.721 eV
- **Energy above convex hull (C2DB)**: 0.145 eV
- **Space group symbol**: $P\bar{6}m2$
- **Space group number**: 187
- **Polar structure**: False

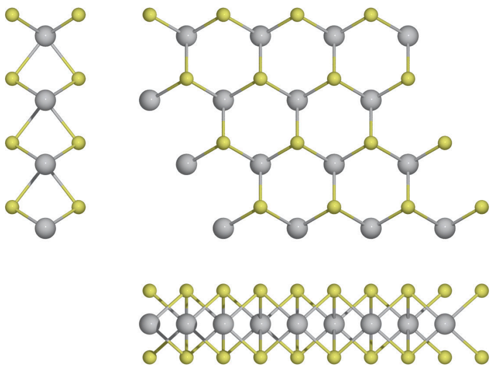

Figure 774: Structure representation

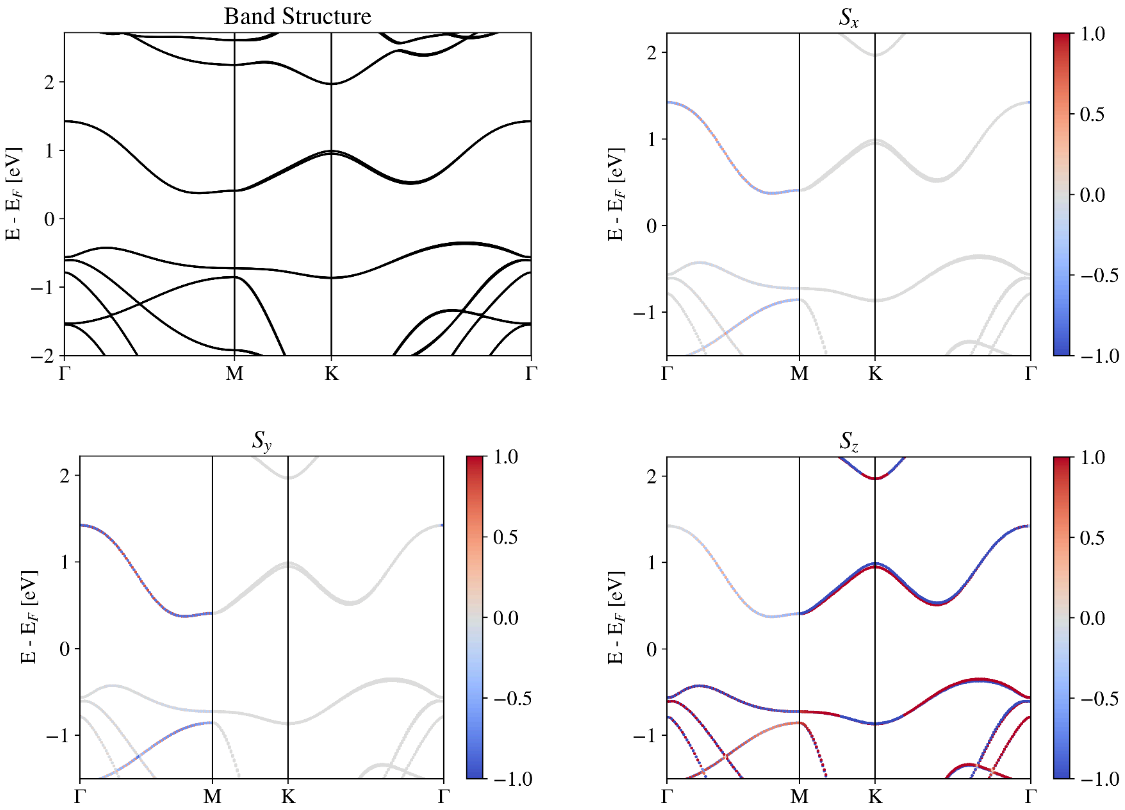


Figure 775: Band structure and spin polarization projections.

## 2.388 TiSe2-0684166af1fd

- **Formula**: TiSe2
- **Structural Cluster**: AB2-11
- **Band gap (PBE)**: 0.864 eV
- **Energy above convex hull (C2DB)**: 0.261 eV
- **Space group symbol**: $P\bar{4}m2$
- **Space group number**: 115
- **Polar structure**: False

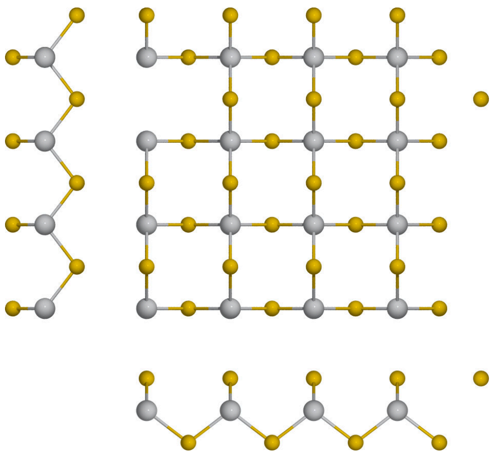

Figure 776: Structure representation

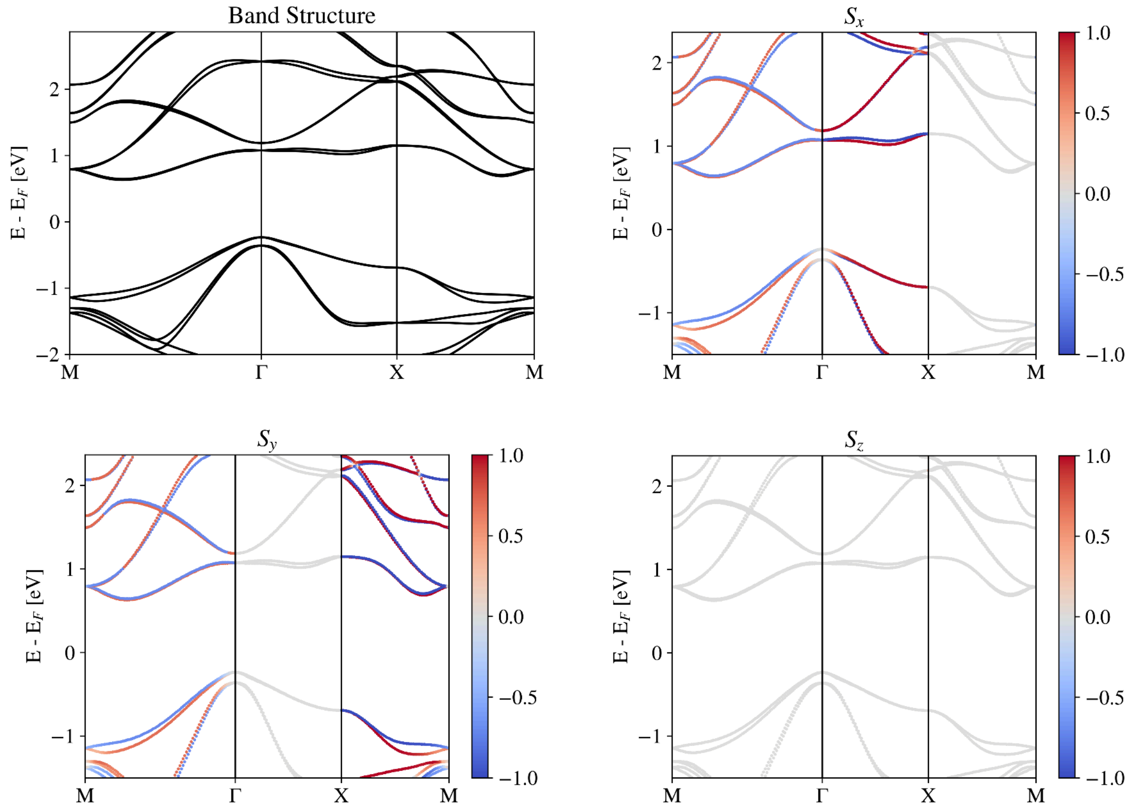


Figure 777: Band structure and spin polarization projections.

## 2.389 TiSe2-509ef368050d

- **Formula**: TiSe2
- **Structural Cluster**: AB2-4
- **Band gap (PBE)**: 0.515 eV
- **Energy above convex hull (C2DB)**: 0.117 eV
- **Space group symbol**: $P\bar{6}m2$
- **Space group number**: 187
- **Polar structure**: False

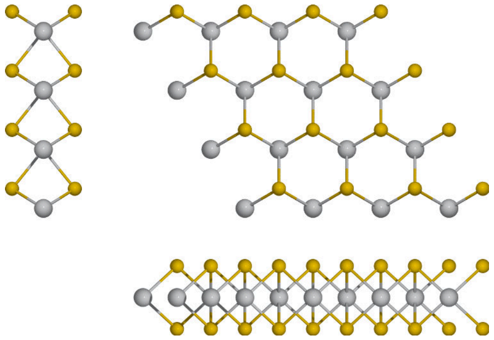

Figure 778: Structure representation

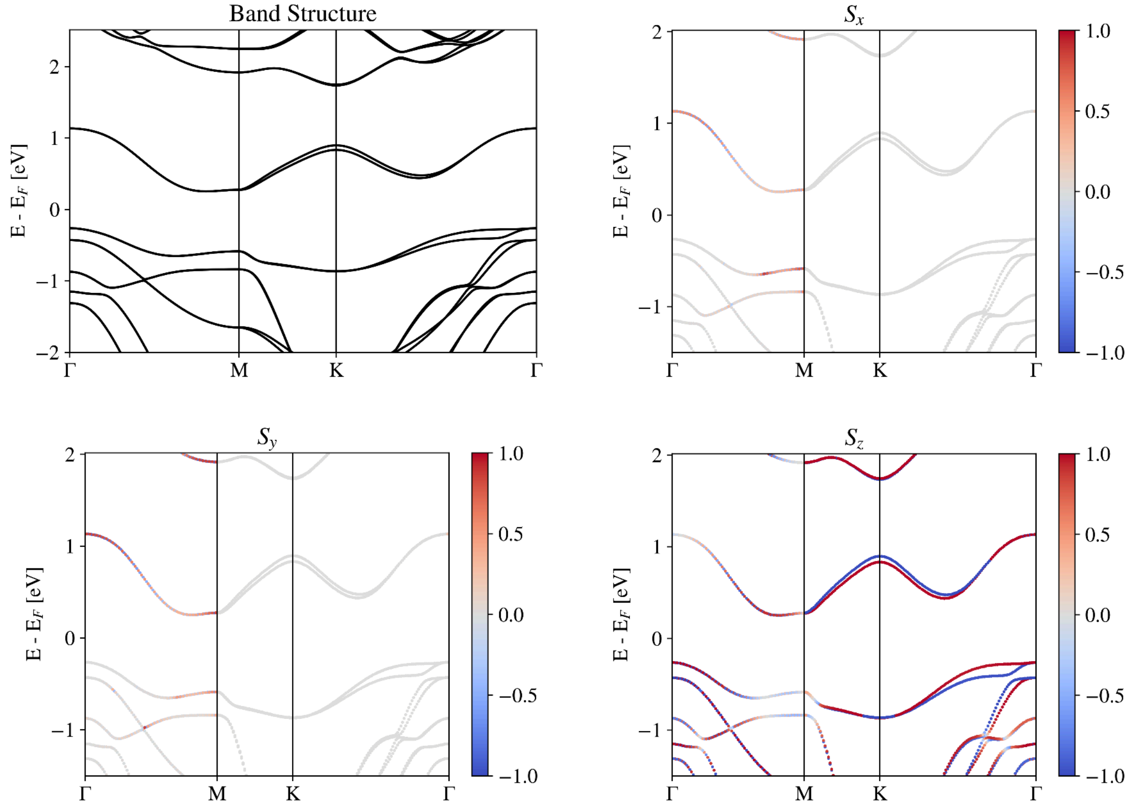


Figure 779: Band structure and spin polarization projections.

## 2.390 TiTe2-bbb8e581bf27

- **Formula**: TiTe2
- **Structural Cluster**: AB2-11
- **Band gap (PBE)**: 0.397 eV
- **Energy above convex hull (C2DB)**: 0.338 eV
- **Space group symbol**: $P\bar{4}m2$
- **Space group number**: 115
- **Polar structure**: False

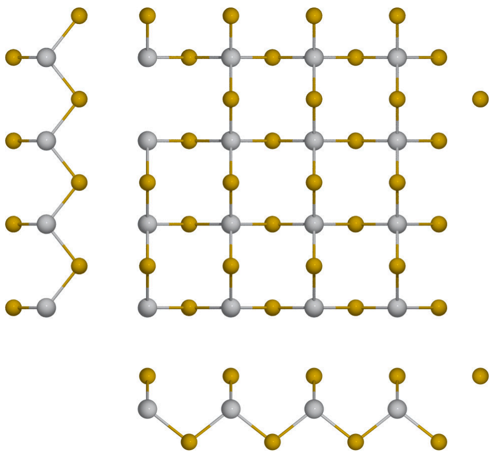

Figure 780: Structure representation

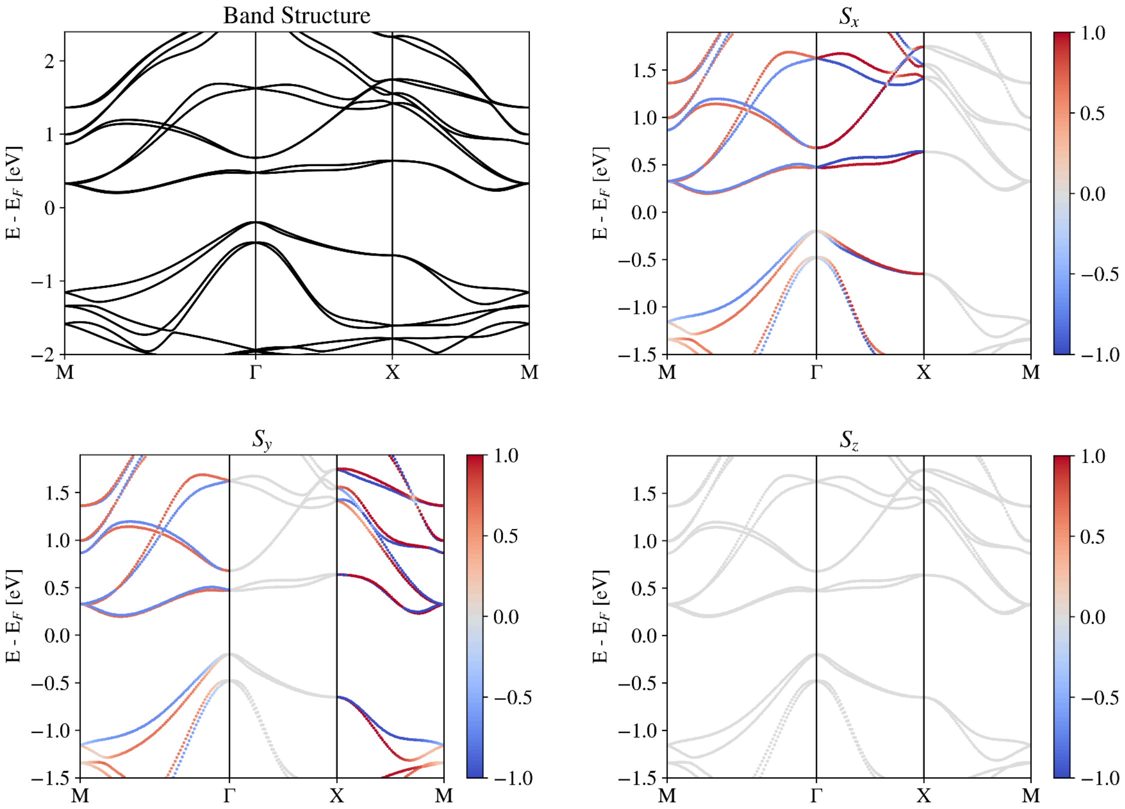


Figure 781: Band structure and spin polarization projections.

## 2.391 TiZr3S8-ec37c6657ea3

- **Formula**: TiZr3S8
- **Structural Cluster**: AB3C8-22
- **Band gap (PBE)**: 0.997 eV
- **Energy above convex hull (C2DB)**: 0.184 eV
- **Space group symbol**: $P1$
- **Space group number**: 1
- **Polar structure**: True

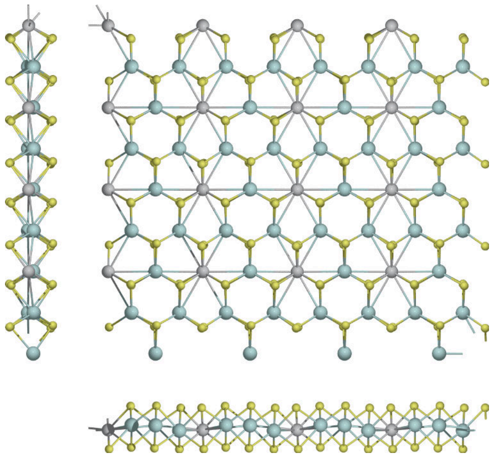

Figure 782: Structure representation

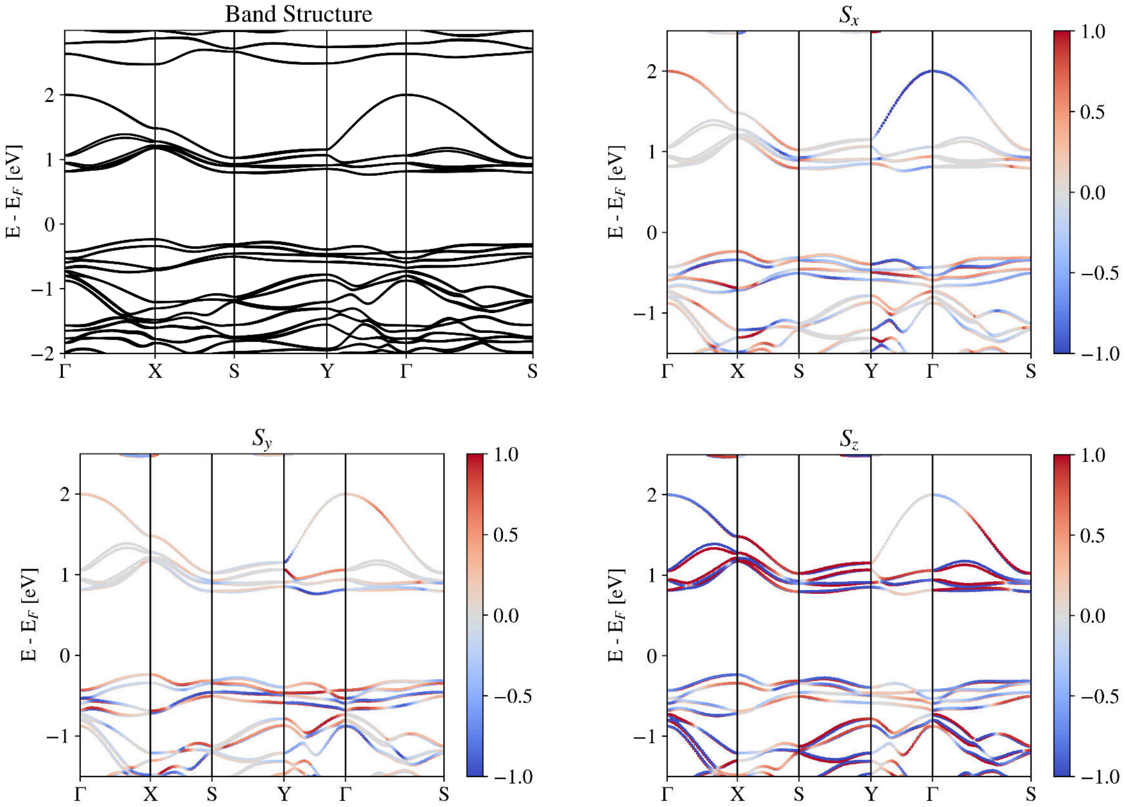


Figure 783: Band structure and spin polarization projections.

## 2.392 TiZr3Se8-a148361e5e9a

- **Formula**: TiZr3Se8
- **Structural Cluster**: AB3C8-22
- **Band gap (PBE)**: 0.701 eV
- **Energy above convex hull (C2DB)**: 0.144 eV
- **Space group symbol**: $P1$
- **Space group number**: 1
- **Polar structure**: True

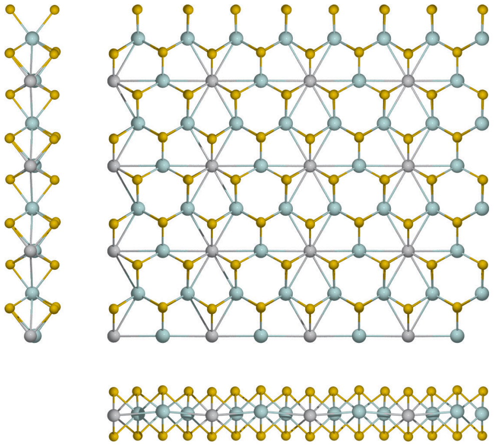

Figure 784: Structure representation

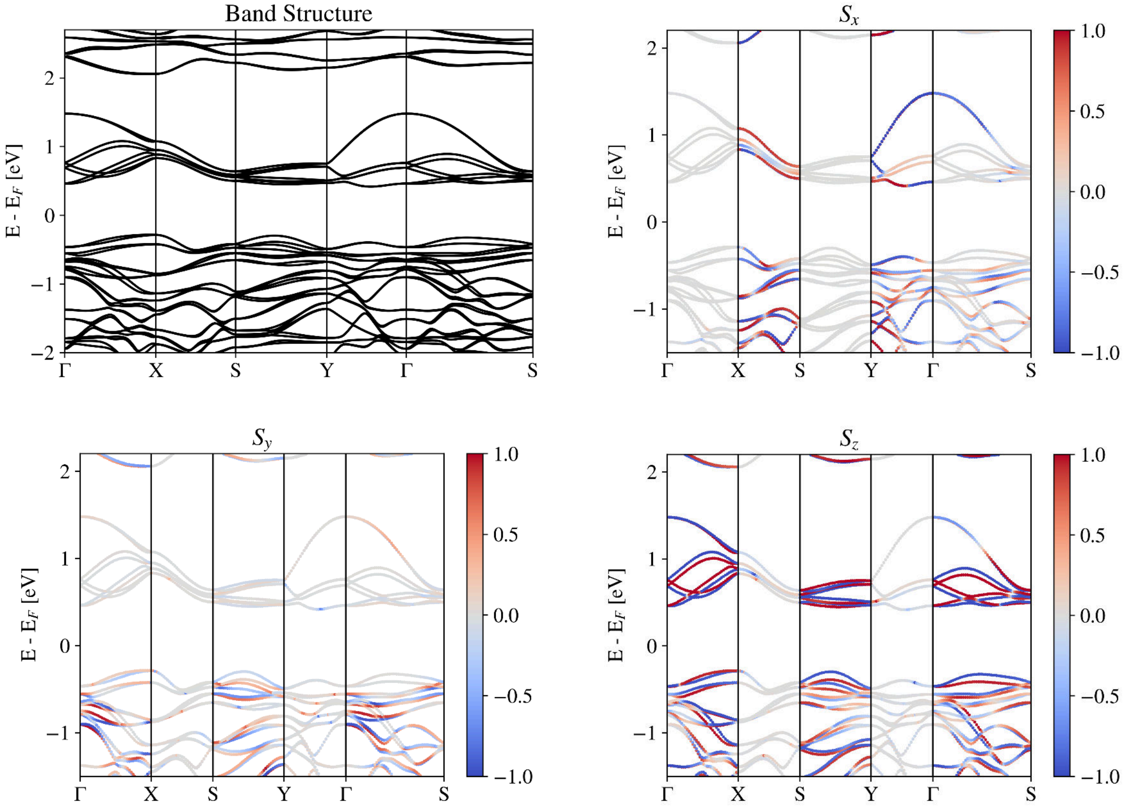


Figure 785: Band structure and spin polarization projections.

## 2.393 TiZr3Te8-4f1ab08988cc

- **Formula**: TiZr3Te8
- **Structural Cluster**: AB3C8-22
- **Band gap (PBE)**: 0.21 eV
- **Energy above convex hull (C2DB)**: 0.115 eV
- **Space group symbol**: *P*1
- **Space group number**: 1
- **Polar structure**: True

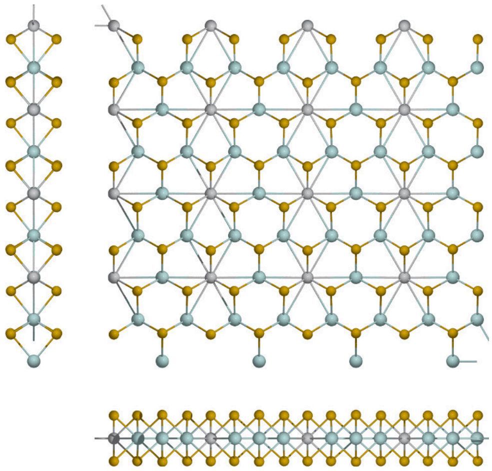

Figure 786: Structure representation

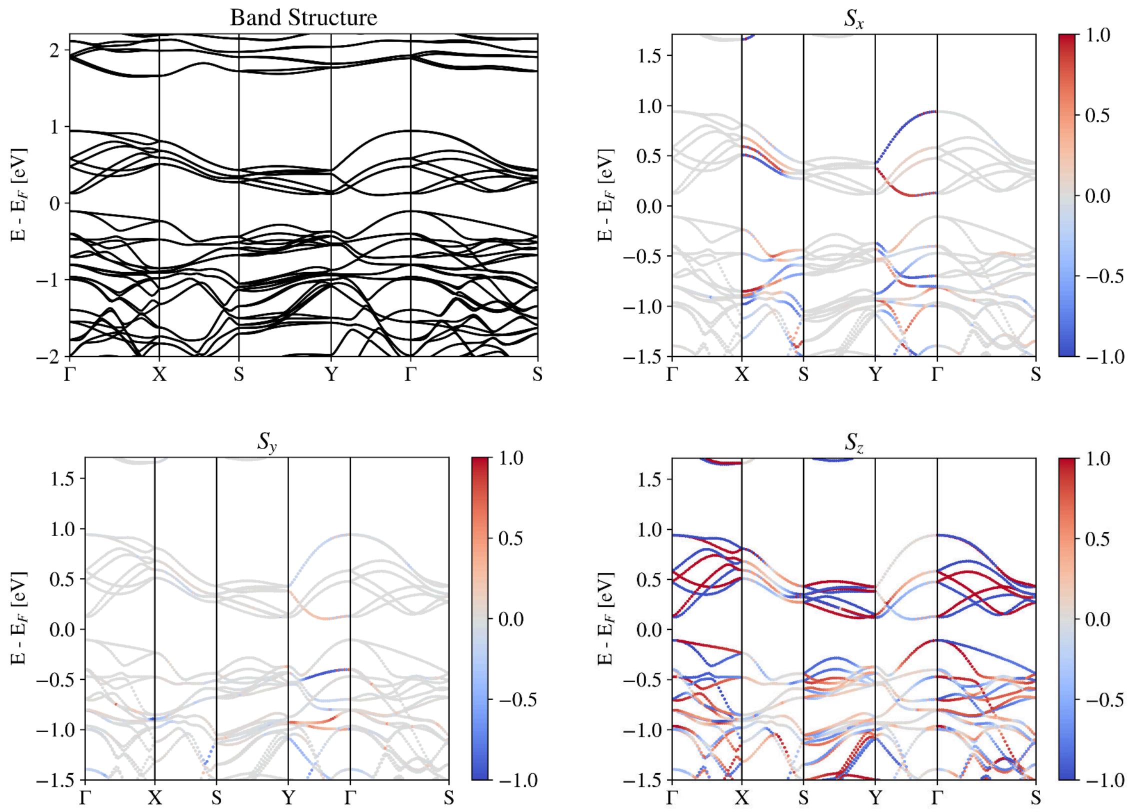


Figure 787: Band structure and spin polarization projections.

## 2.394 Tl2Br6-ef8df28dd878

- **Formula**: Tl2Br6
- **Structural Cluster**: AB3-19
- **Band gap (PBE)**: 1.085 eV
- **Energy above convex hull (C2DB)**: 0.087 eV
- **Space group symbol**: $P\bar{6}2m$
- **Space group number**: 189
- **Polar structure**: False

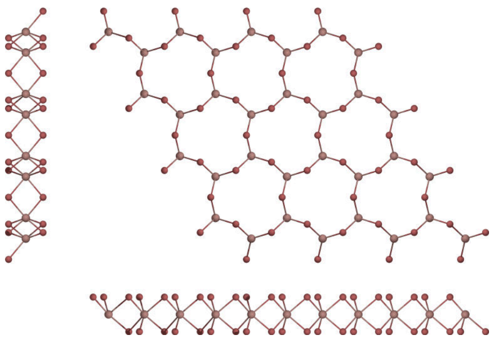

Figure 788: Structure representation

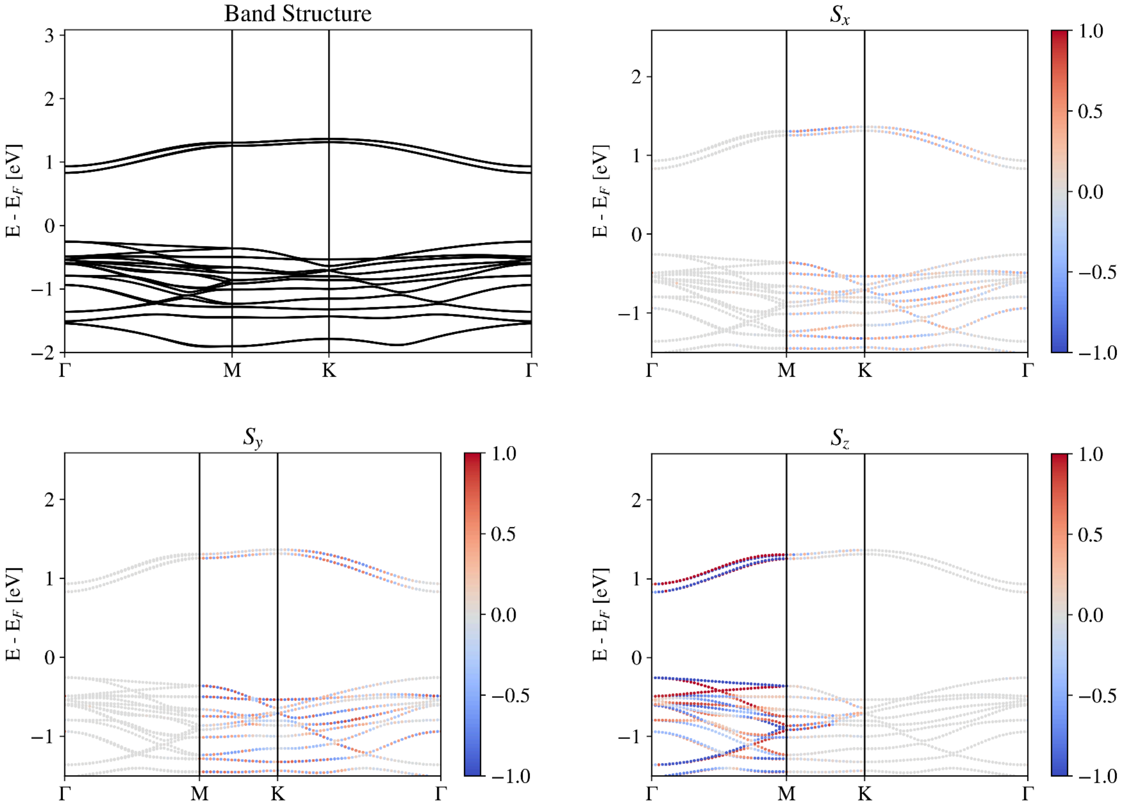


Figure 789: Band structure and spin polarization projections.

## 2.395 Tl2Cl4-30ddf92eb9d6

- **Formula**: Tl2Cl4
- **Structural Cluster**: AB2-3
- **Band gap (PBE)**: 1.866 eV
- **Energy above convex hull (C2DB)**: 0.039 eV
- **Space group symbol**: *Pm*
- **Space group number**: 6
- **Polar structure**: True

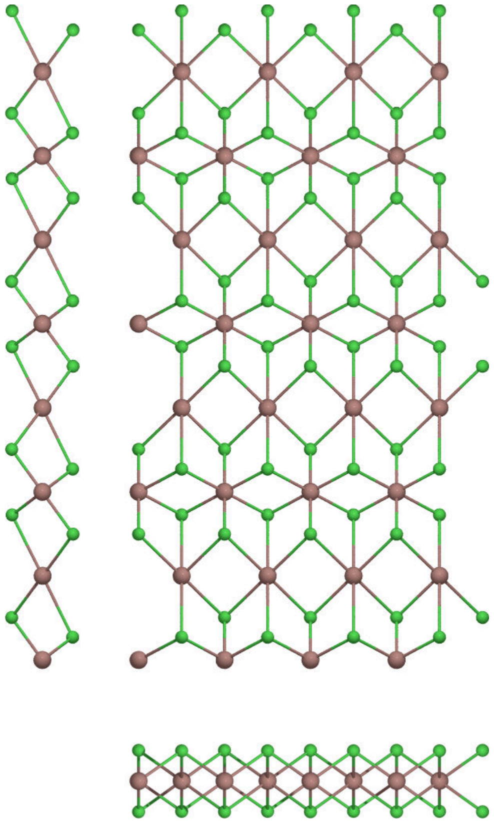

Figure 790: Structure representation

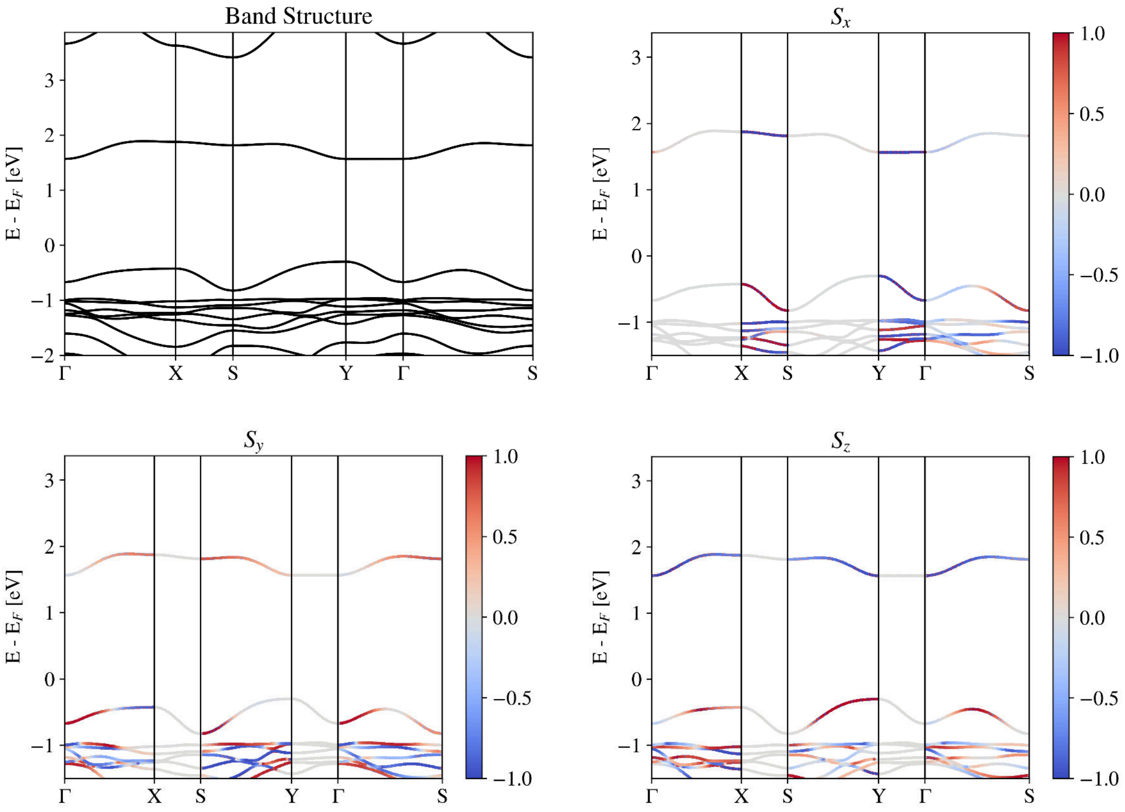


Figure 791: Band structure and spin polarization projections.

## 2.396 Tl2Cl6-a7ad562f5ed6

- **Formula**: Tl2Cl6
- **Structural Cluster**: AB3-19
- **Band gap (PBE)**: 1.663 eV
- **Energy above convex hull (C2DB)**: 0.076 eV
- **Space group symbol**: $P321$
- **Space group number**: 150
- **Polar structure**: False

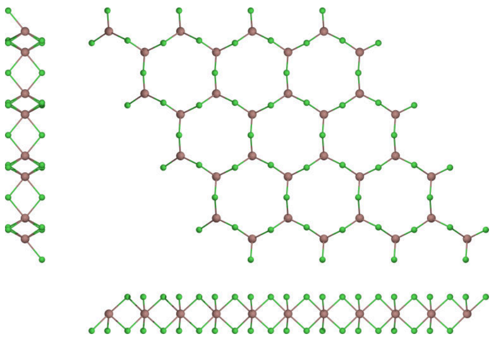

Figure 792: Structure representation

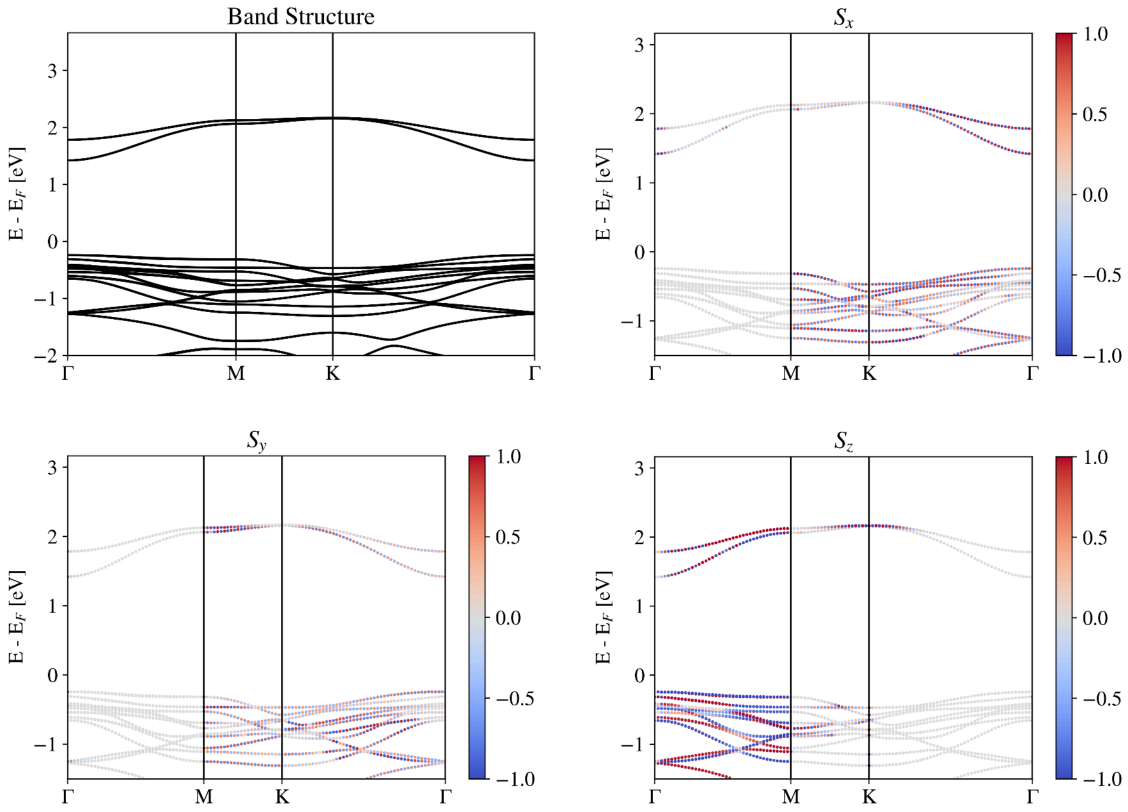


Figure 793: Band structure and spin polarization projections.

## 2.397 Tl2I6-8b75f770fbf8

- **Formula**: Tl2I6
- **Structural Cluster**: AB3-19
- **Band gap (PBE)**: 0.12 eV
- **Energy above convex hull (C2DB)**: 0.202 eV
- **Space group symbol**: $P\bar{6}2m$
- **Space group number**: 189
- **Polar structure**: False

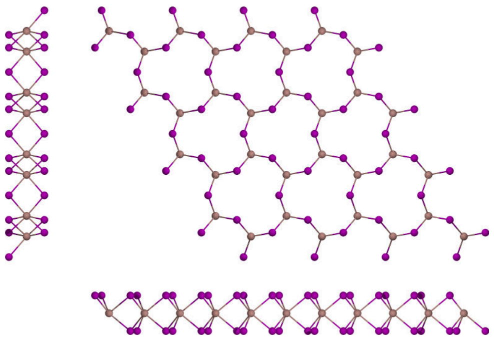

Figure 794: Structure representation

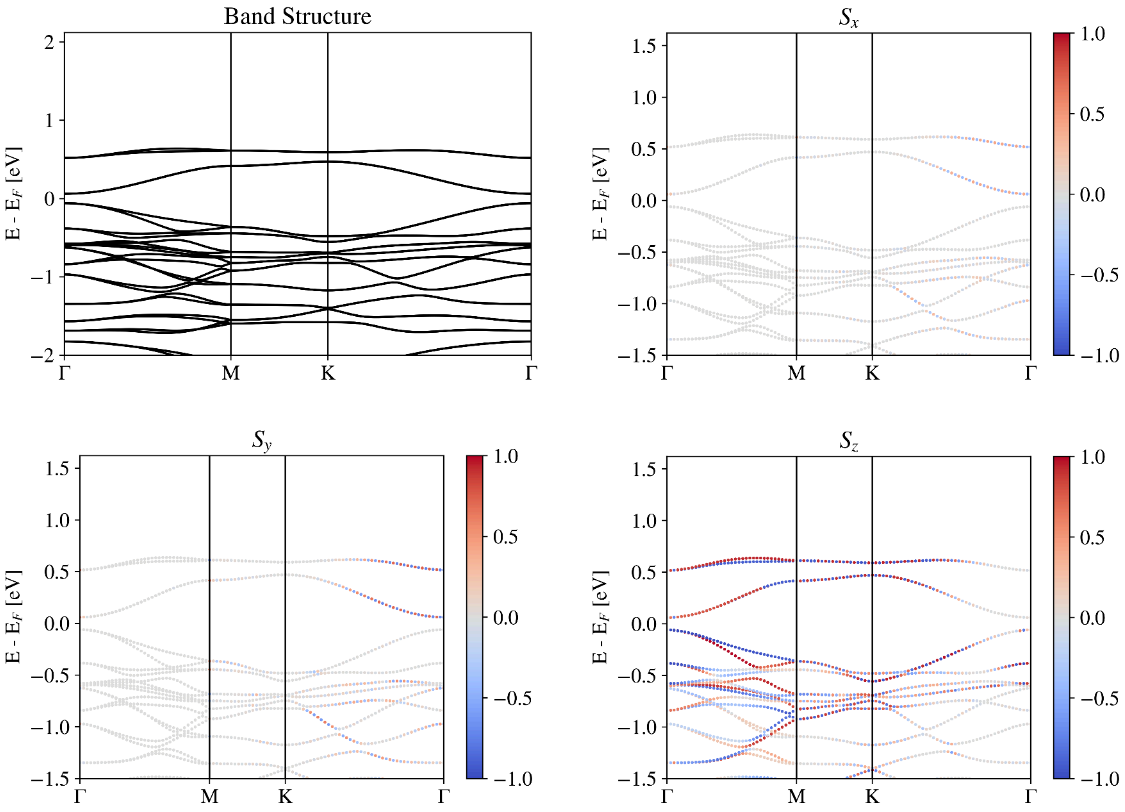


Figure 795: Band structure and spin polarization projections.

## 2.398 W2Br6-14e114b103e1

- **Formula**: W2Br6
- **Structural Cluster**: AB3-20
- **Band gap (PBE)**: 0.222 eV
- **Energy above convex hull (C2DB)**: 0.271 eV
- **Space group symbol**: $P\bar{6}2m$
- **Space group number**: 189
- **Polar structure**: False

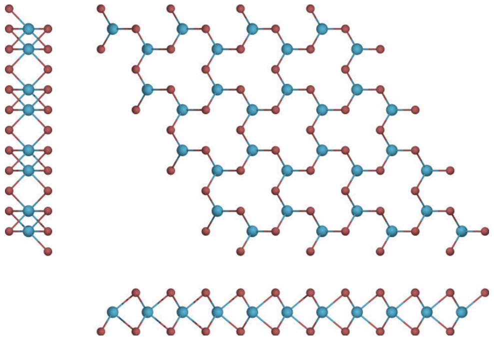

Figure 796: Structure representation

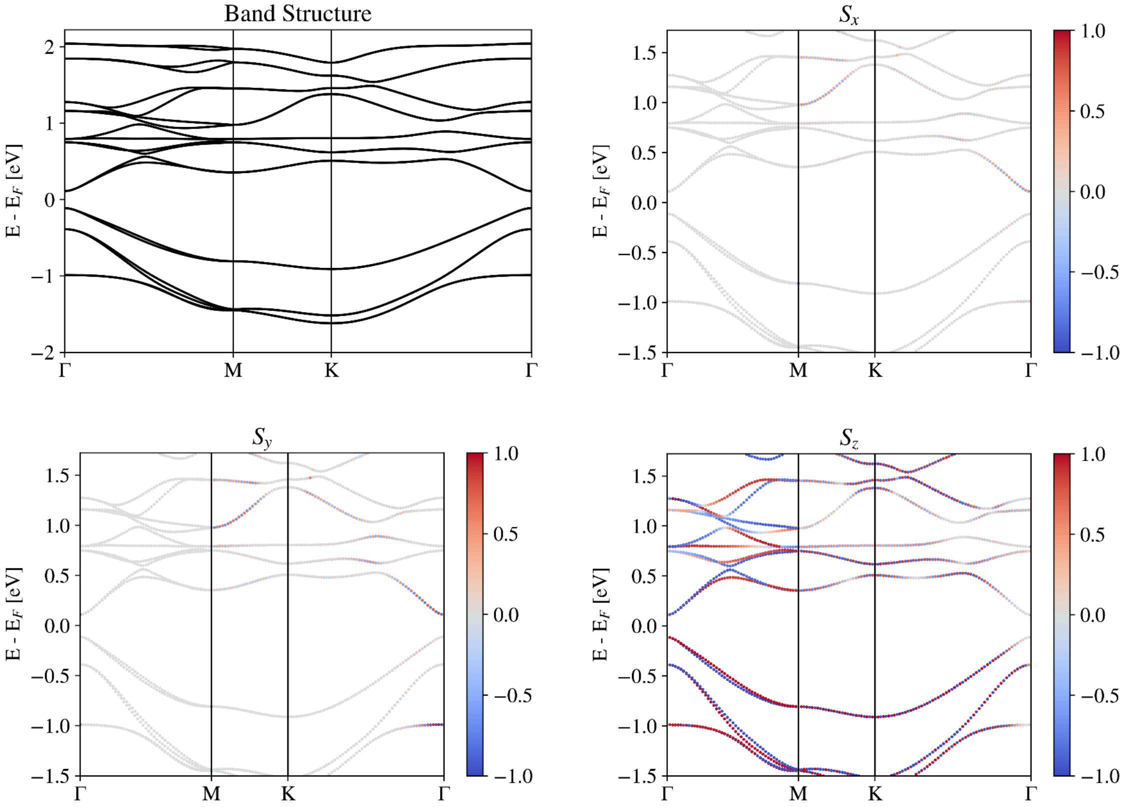


Figure 797: Band structure and spin polarization projections.

## 2.399 W2Cl6-eea8aee9e120

- **Formula**: W2Cl6
- **Structural Cluster**: AB3-20
- **Band gap (PBE)**: 0.73 eV
- **Energy above convex hull (C2DB)**: 0.164 eV
- **Space group symbol**: $P\bar{6}2m$
- **Space group number**: 189
- **Polar structure**: False

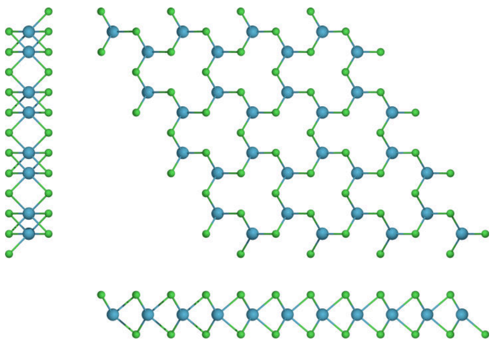

Figure 798: Structure representation

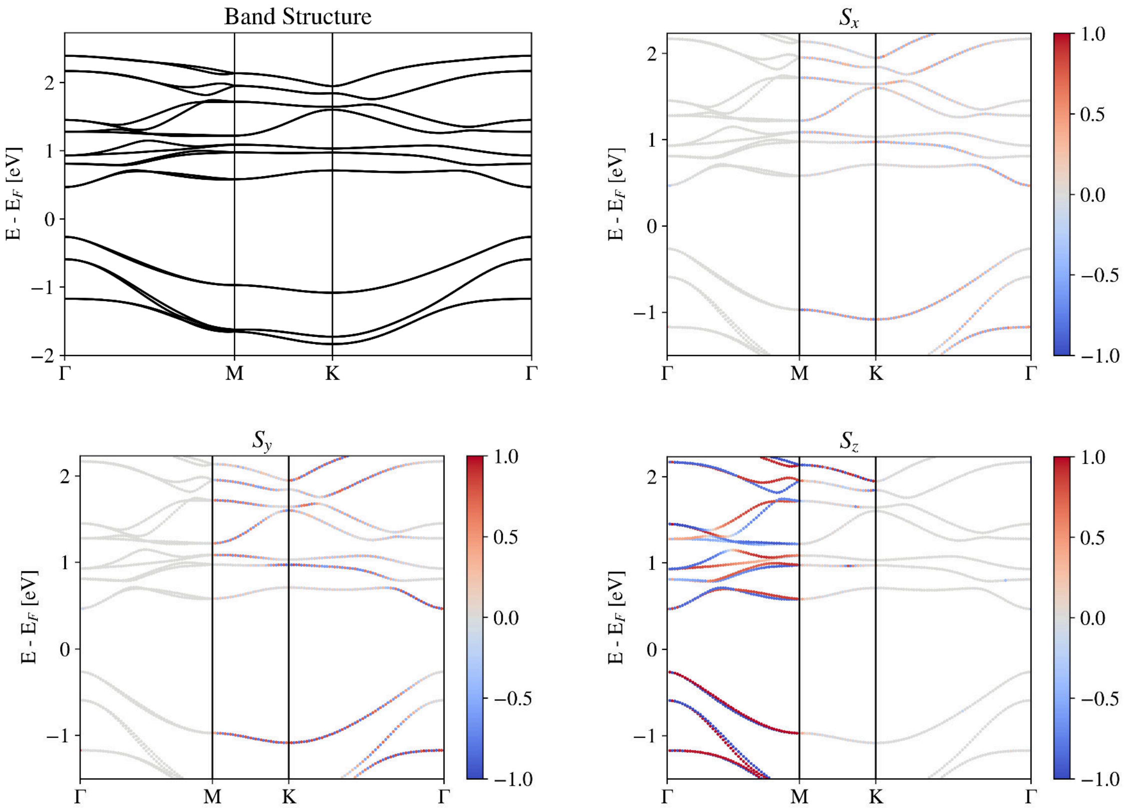


Figure 799: Band structure and spin polarization projections.

## 2.400 W2I6-37deba64dc68

- **Formula**: W2I6
- **Structural Cluster**: AB3-20
- **Band gap (PBE)**: 0.205 eV
- **Energy above convex hull (C2DB)**: 0.19 eV
- **Space group symbol**: $P\bar{6}2m$
- **Space group number**: 189
- **Polar structure**: False

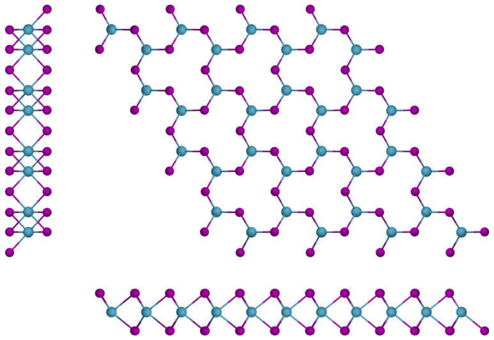

Figure 800: Structure representation

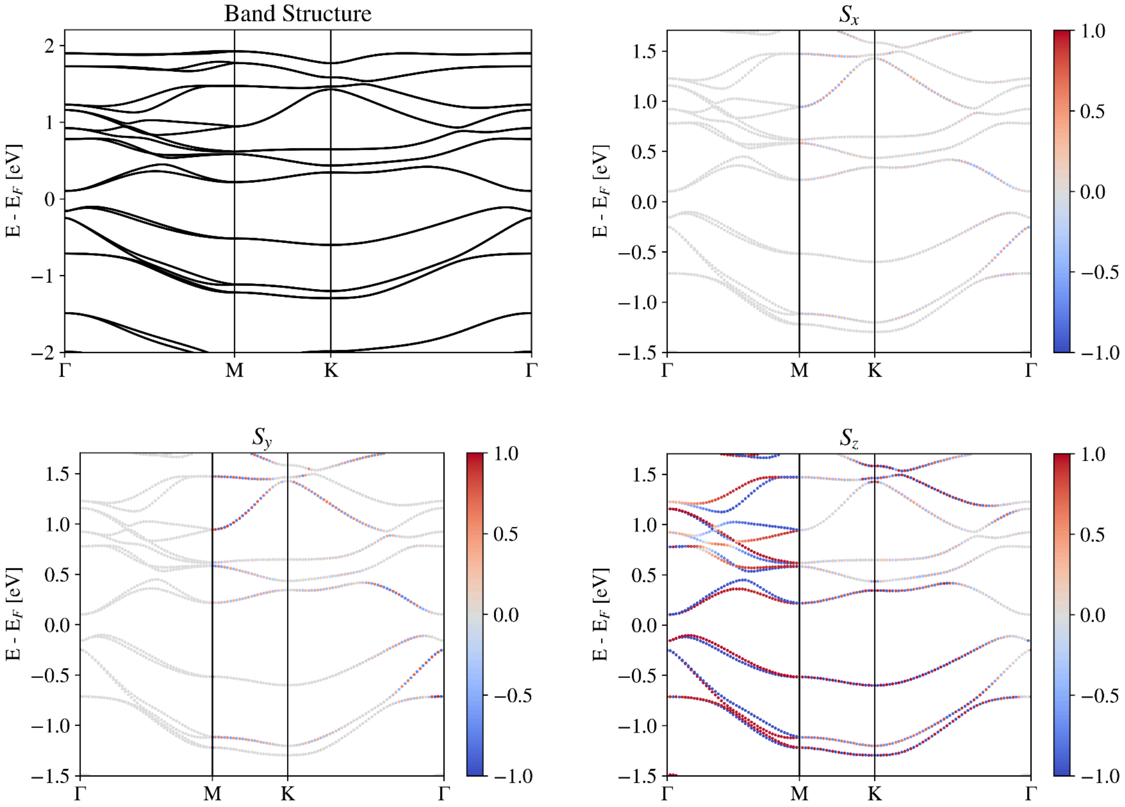


Figure 801: Band structure and spin polarization projections.

## 2.401 WCr3S8-dc4259e69783

- **Formula**: WCr3S8
- **Structural Cluster**: AB3C8-22
- **Band gap (PBE)**: 0.888 eV
- **Energy above convex hull (C2DB)**: 0.009 eV
- **Space group symbol**: *Pmm*2
- **Space group number**: 25
- **Polar structure**: True

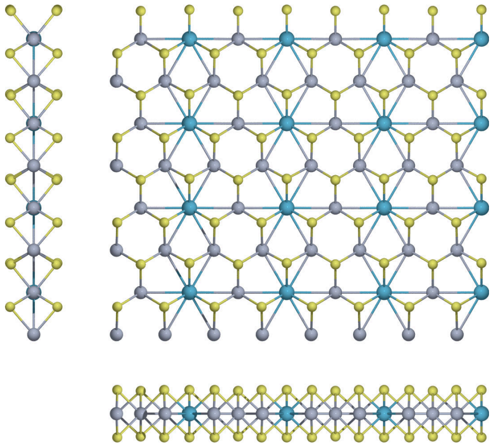

Figure 802: Structure representation

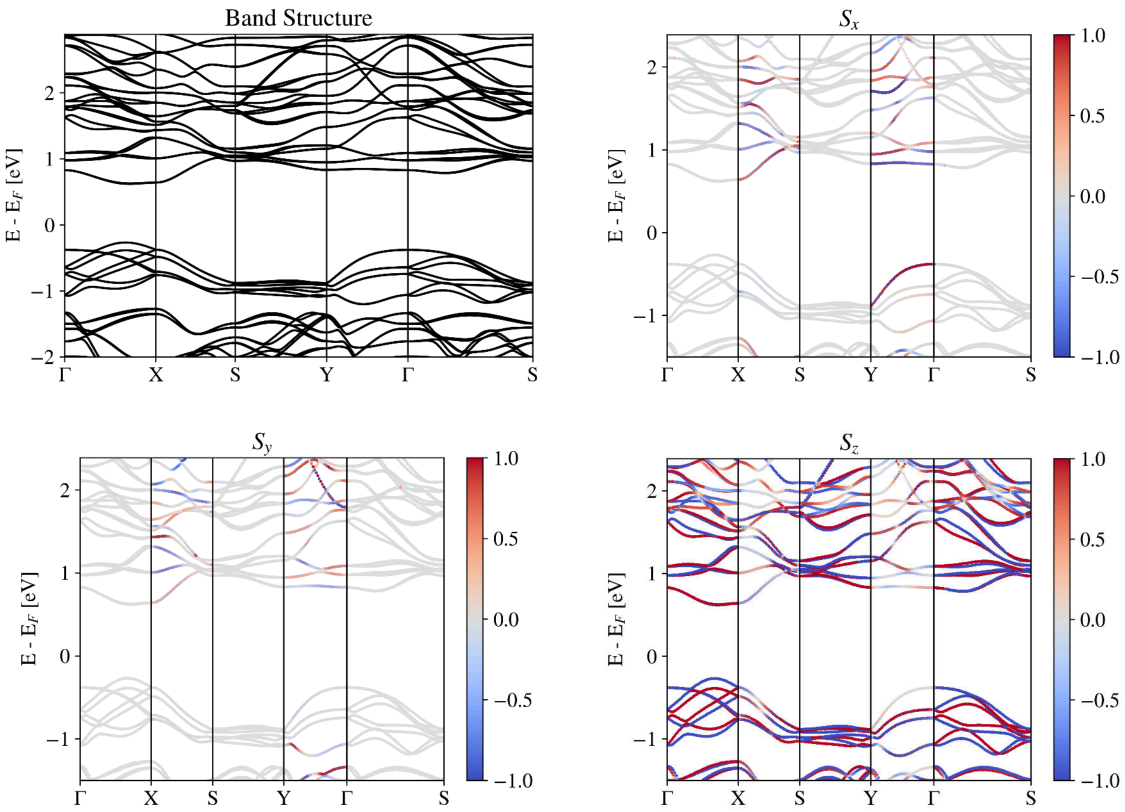


Figure 803: Band structure and spin polarization projections.

## 2.402 WCr3Se8-c798e725e2fb

- **Formula**: WCr3Se8
- **Structural Cluster**: AB3C8-22
- **Band gap (PBE)**: 0.698 eV
- **Energy above convex hull (C2DB)**: 0.009 eV
- **Space group symbol**: $P1$
- **Space group number**: 1
- **Polar structure**: True

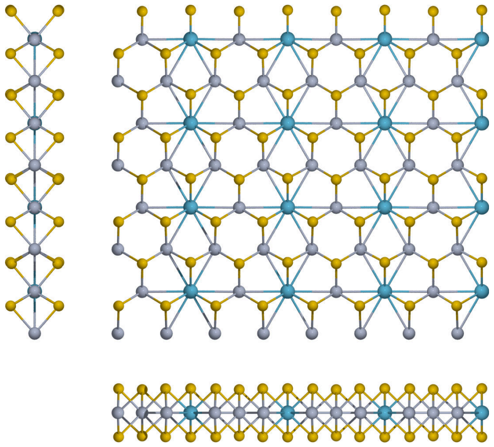

Figure 804: Structure representation

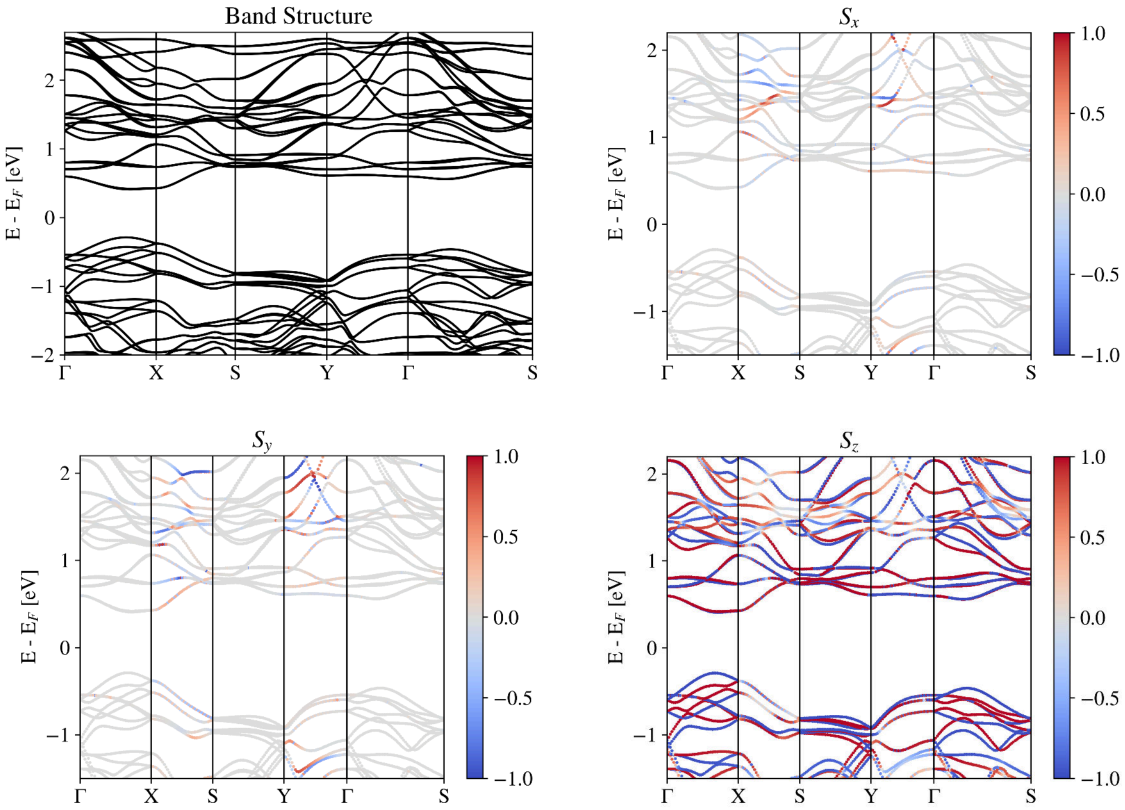


Figure 805: Band structure and spin polarization projections.

## 2.403 WCr3Te8-6523c349753c

- **Formula**: WCr3Te8
- **Structural Cluster**: AB3C8-22
- **Band gap (PBE)**: 0.459 eV
- **Energy above convex hull (C2DB)**: 0.097 eV
- **Space group symbol**: $P1$
- **Space group number**: 1
- **Polar structure**: True

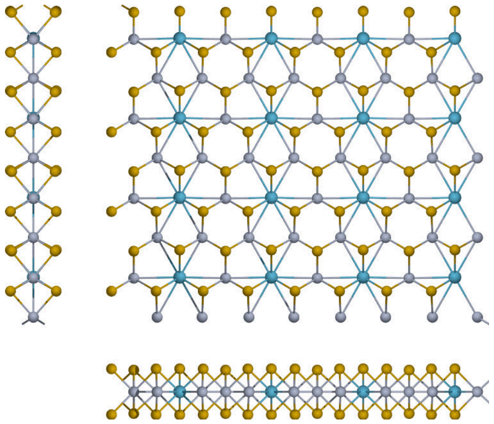

Figure 806: Structure representation

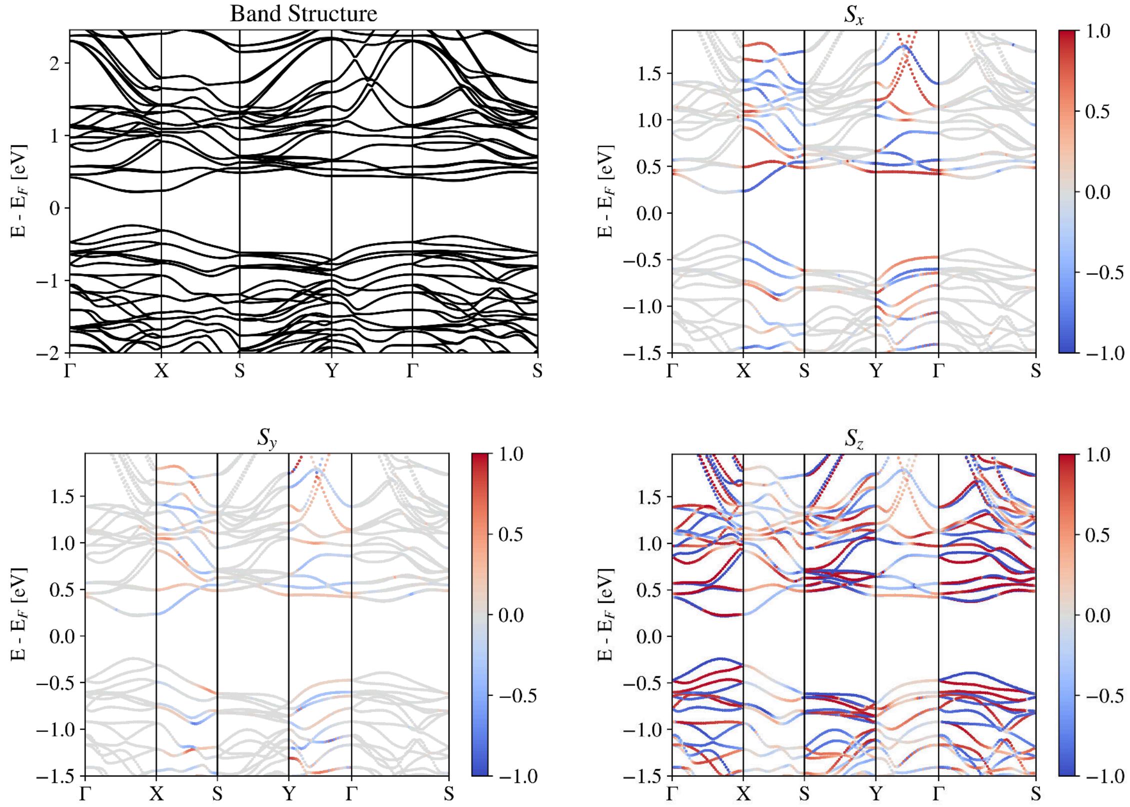


Figure 807: Band structure and spin polarization projections.

## 2.404 WMo3S8-9c2979187585

- **Formula**: WMo3S8
- **Structural Cluster**: AB3C8-22
- **Band gap (PBE)**: 1.582 eV
- **Energy above convex hull (C2DB)**: 0.0 eV
- **Space group symbol**: $P1$
- **Space group number**: 1
- **Polar structure**: True

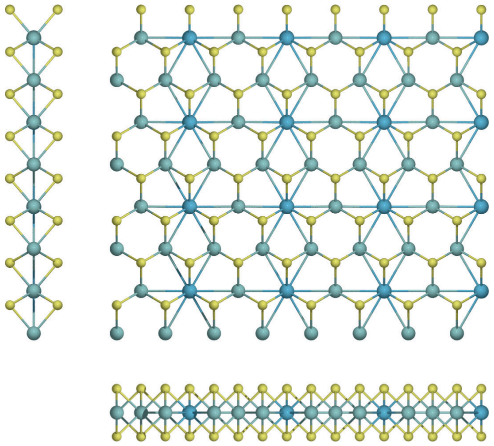

Figure 808: Structure representation

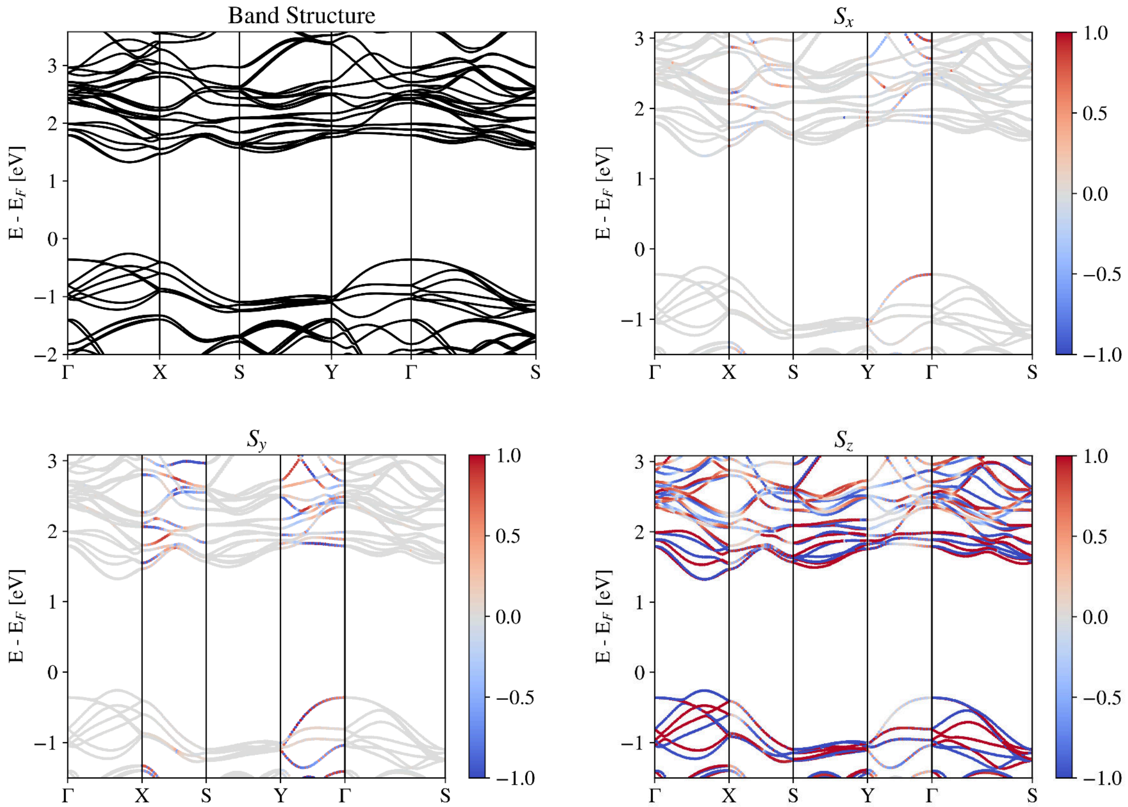


Figure 809: Band structure and spin polarization projections.

## 2.405 WMo3Se8-05a06afa3b20

- **Formula**: WMo3Se8
- **Structural Cluster**: AB3C8-22
- **Band gap (PBE)**: 1.32 eV
- **Energy above convex hull (C2DB)**: 0.0 eV
- **Space group symbol**: *Pm*
- **Space group number**: 6
- **Polar structure**: True

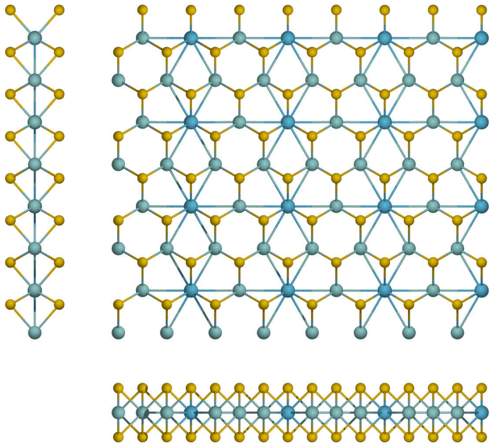

Figure 810: Structure representation

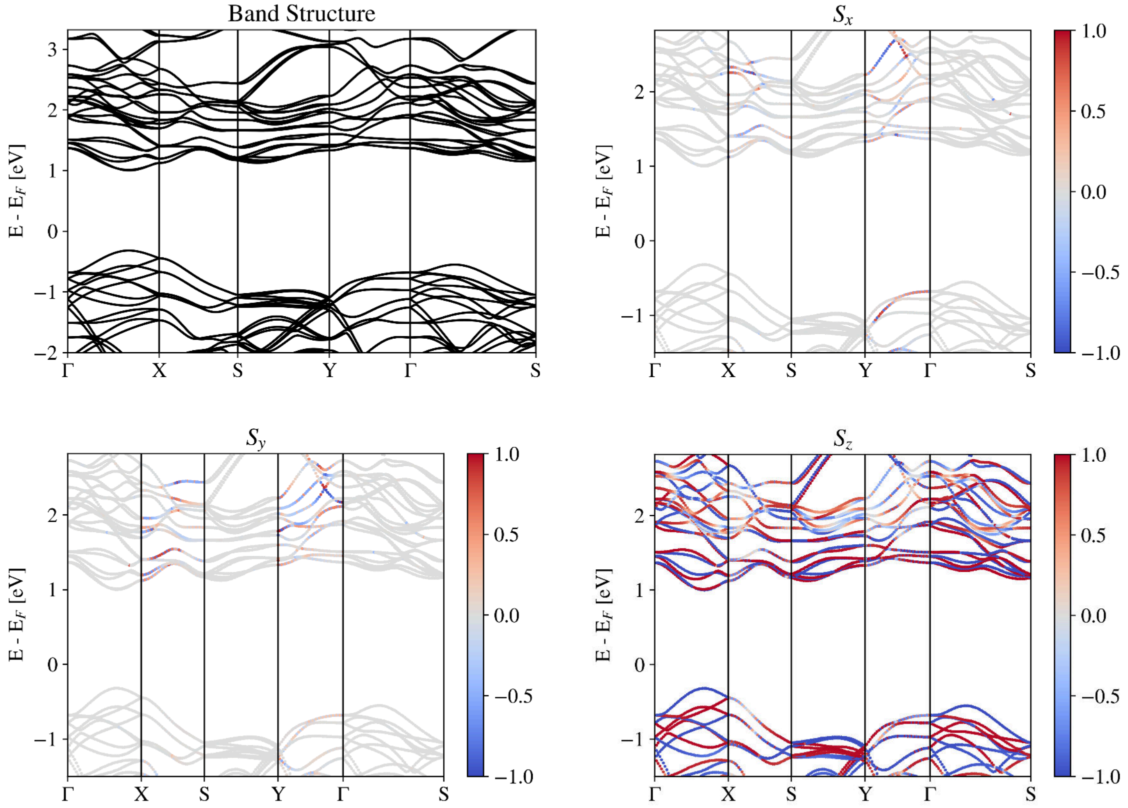


Figure 811: Band structure and spin polarization projections.

## 2.406 WMo3Te8-323fb700d903

- **Formula**: WMo3Te8
- **Structural Cluster**: AB3C8-22
- **Band gap (PBE)**: 0.923 eV
- **Energy above convex hull (C2DB)**: 0.005 eV
- **Space group symbol**: $P1$
- **Space group number**: 1
- **Polar structure**: True

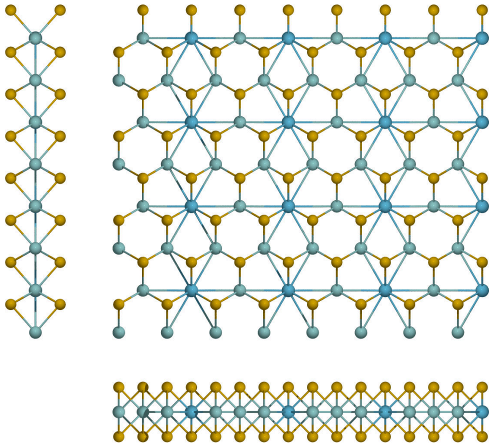

Figure 812: Structure representation

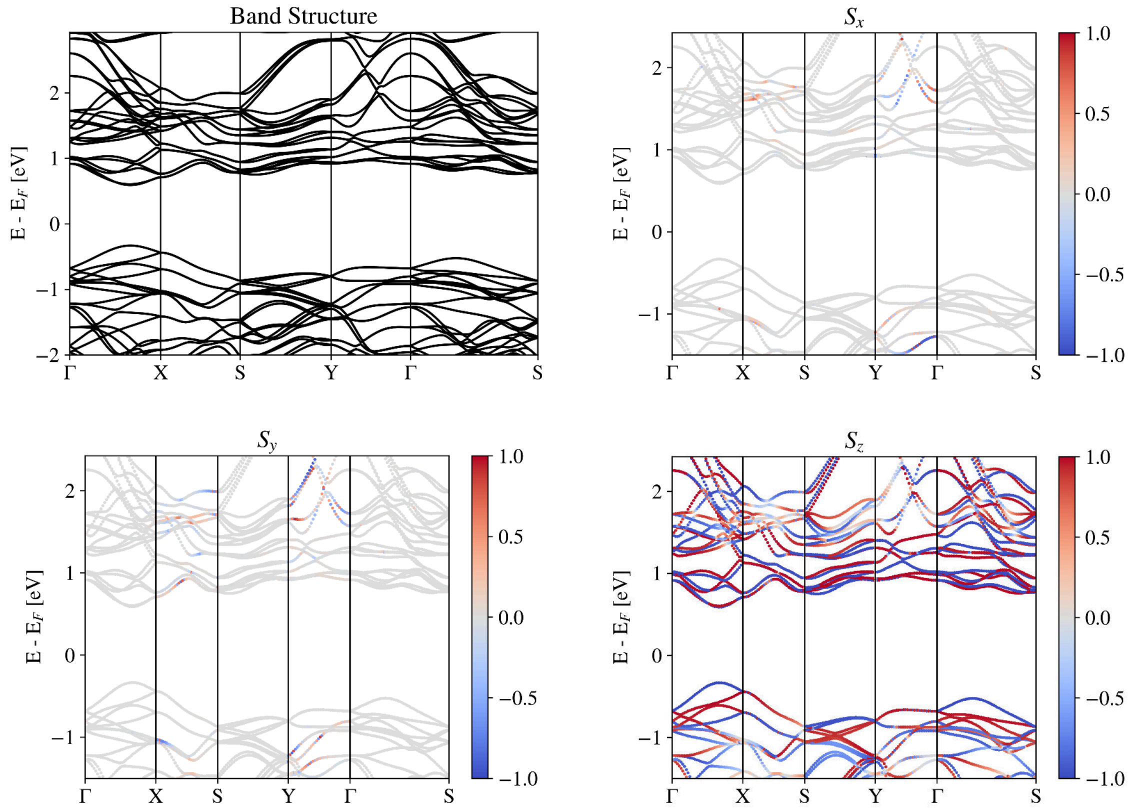


Figure 813: Band structure and spin polarization projections.

## 2.407 WO2-94cfbb3f9284

- **Formula**: WO2
- **Structural Cluster**: AB2-4
- **Band gap (PBE)**: 1.312 eV
- **Energy above convex hull (C2DB)**: 0.0 eV
- **Space group symbol**: $P\bar{6}m2$
- **Space group number**: 187
- **Polar structure**: False

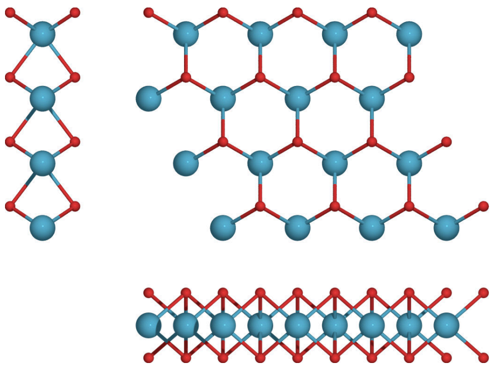

Figure 814: Structure representation

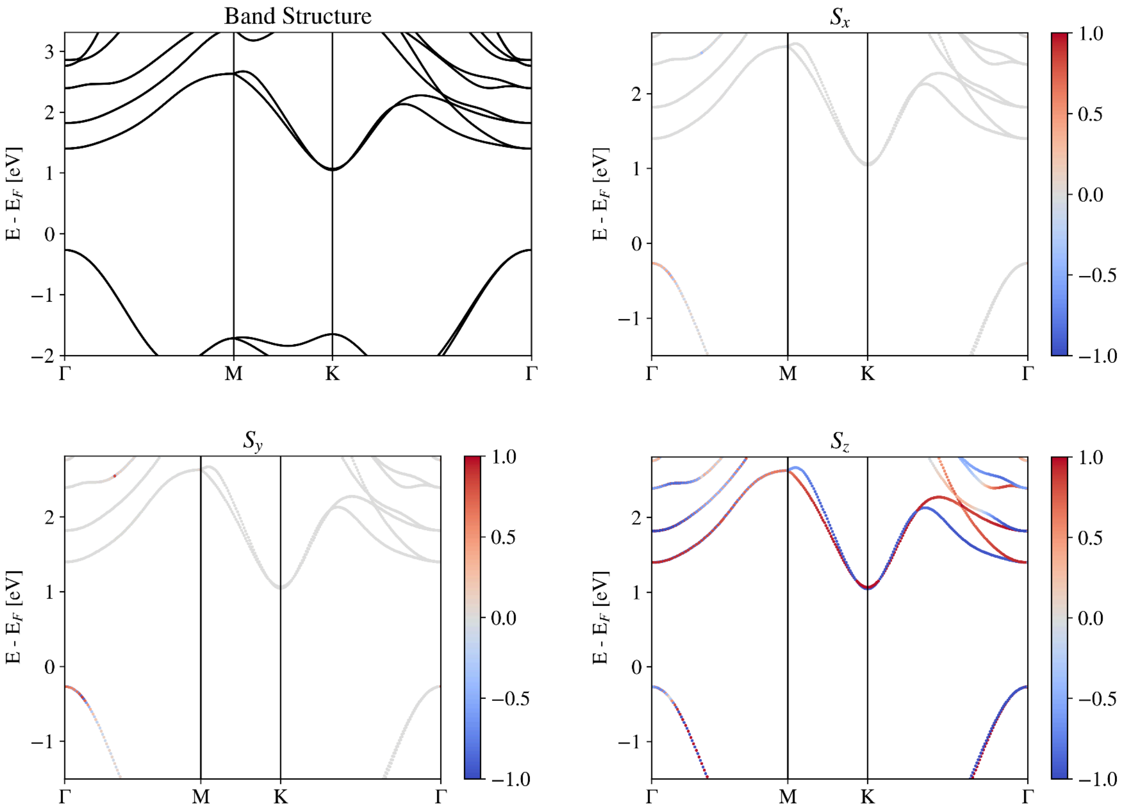


Figure 815: Band structure and spin polarization projections.

## 2.408 WS2-64090c9845f8

- **Formula**: WS2
- **Structural Cluster**: AB2-4
- **Band gap (PBE)**: 1.551 eV
- **Energy above convex hull (C2DB)**: 0.0 eV
- **Space group symbol**: $P\bar{6}m2$
- **Space group number**: 187
- **Polar structure**: False

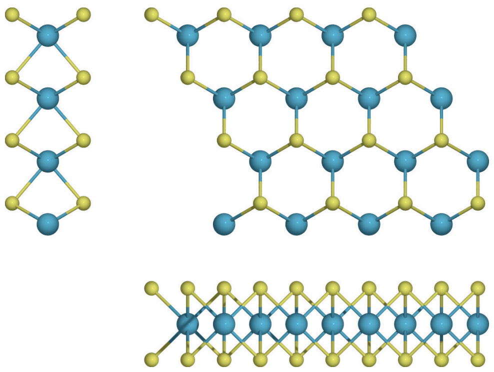

Figure 816: Structure representation

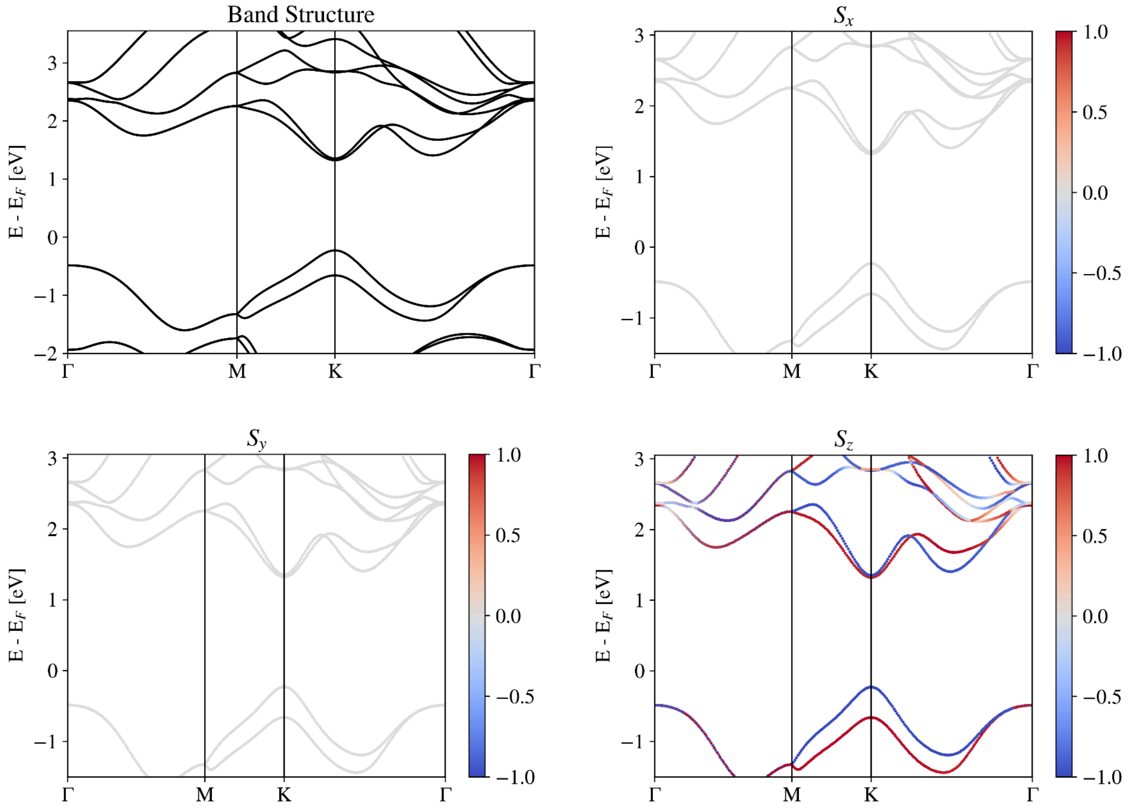


Figure 817: Band structure and spin polarization projections.

## 2.409 WSe2-1cfbe6183886

- **Formula**: WSe2
- **Structural Cluster**: AB2-4
- **Band gap (PBE)**: 1.255 eV
- **Energy above convex hull (C2DB)**: 0.0 eV
- **Space group symbol**: $P\bar{6}m2$
- **Space group number**: 187
- **Polar structure**: False

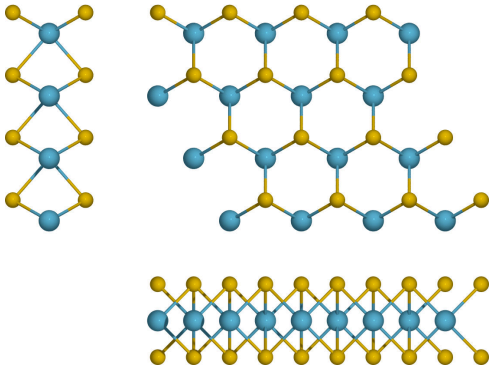

Figure 818: Structure representation

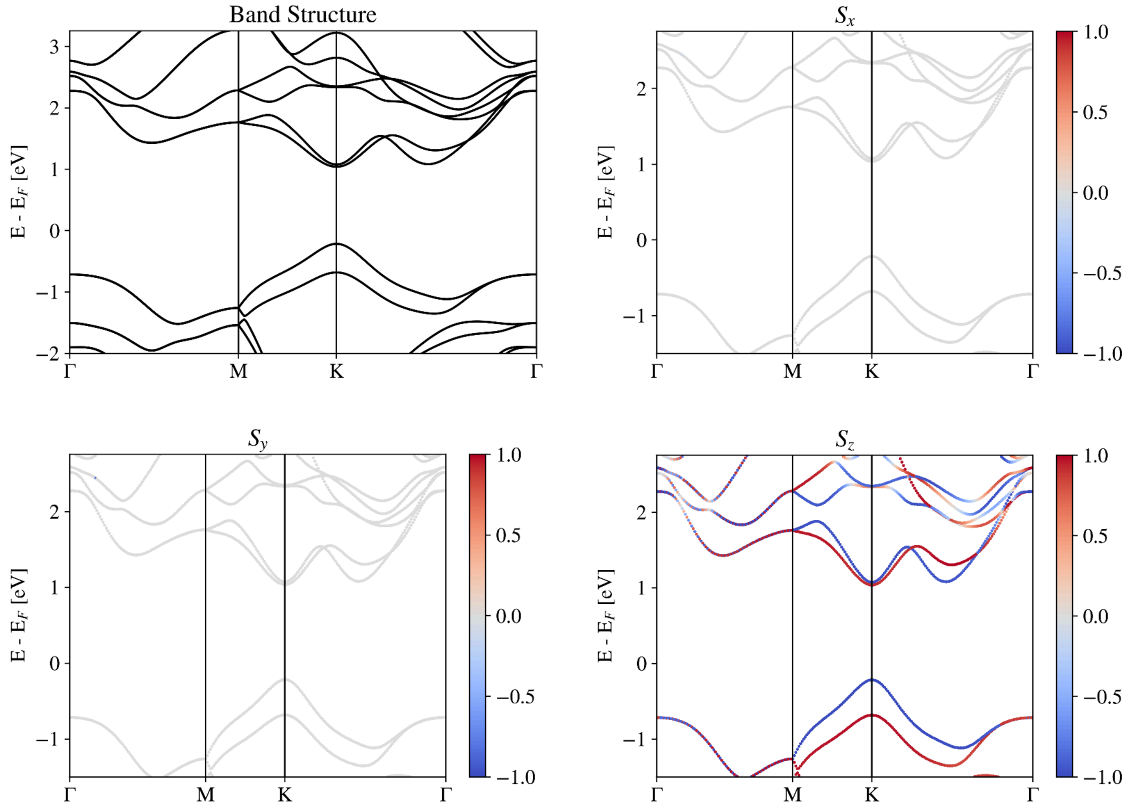


Figure 819: Band structure and spin polarization projections.

## 2.410 WTe2-3c87365bc48c

- **Formula**: WTe2
- **Structural Cluster**: AB2-4
- **Band gap (PBE)**: 0.754 eV
- **Energy above convex hull (C2DB)**: 0.026 eV
- **Space group symbol**: $P\bar{6}m2$
- **Space group number**: 187
- **Polar structure**: False

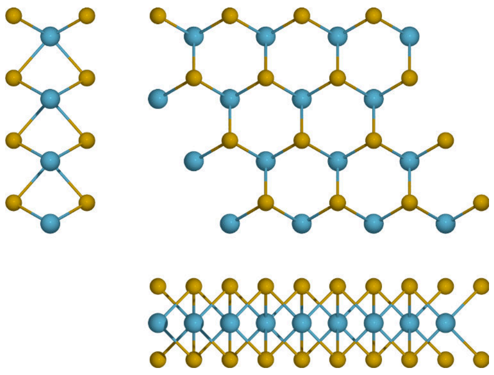

Figure 820: Structure representation

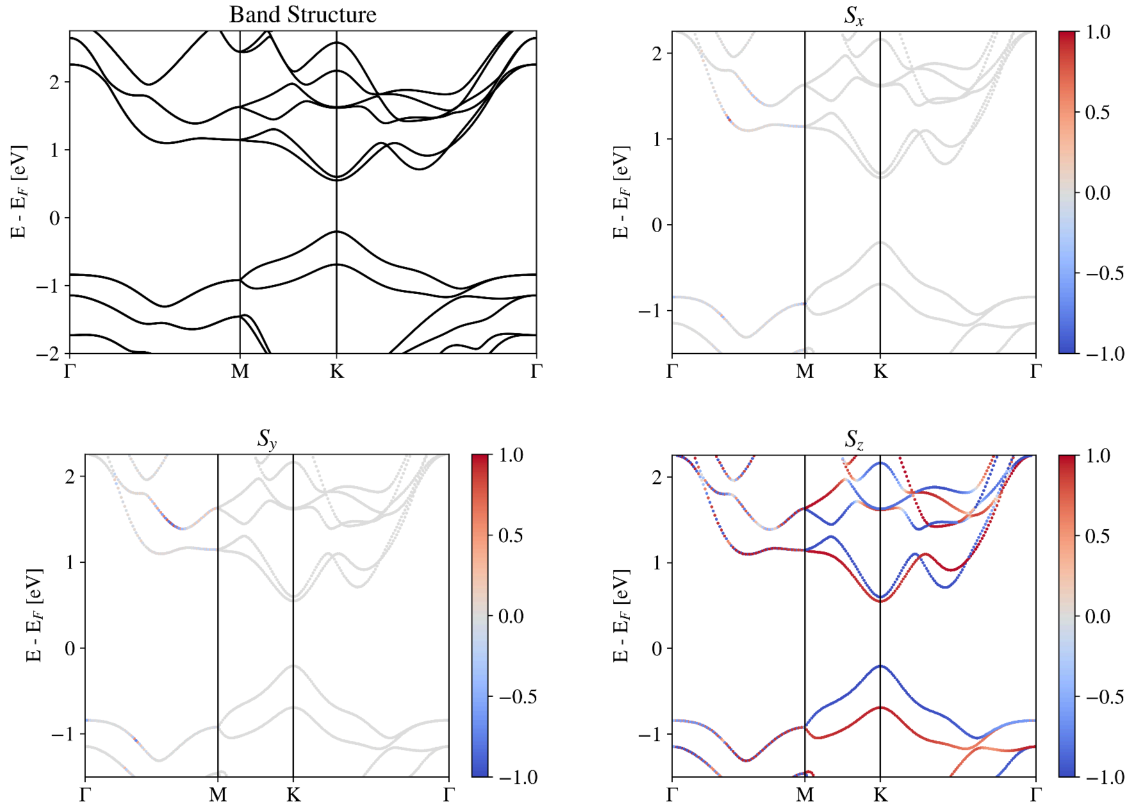


Figure 821: Band structure and spin polarization projections.

## 2.411 Y2Br6-6d976a1e88b3

- **Formula**: Y2Br6
- **Structural Cluster**: AB3-19
- **Band gap (PBE)**: 3.494 eV
- **Energy above convex hull (C2DB)**: 0.051 eV
- **Space group symbol**: $P\bar{6}2m$
- **Space group number**: 189
- **Polar structure**: False

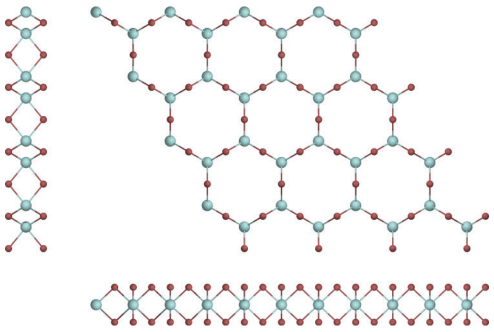

Figure 822: Structure representation

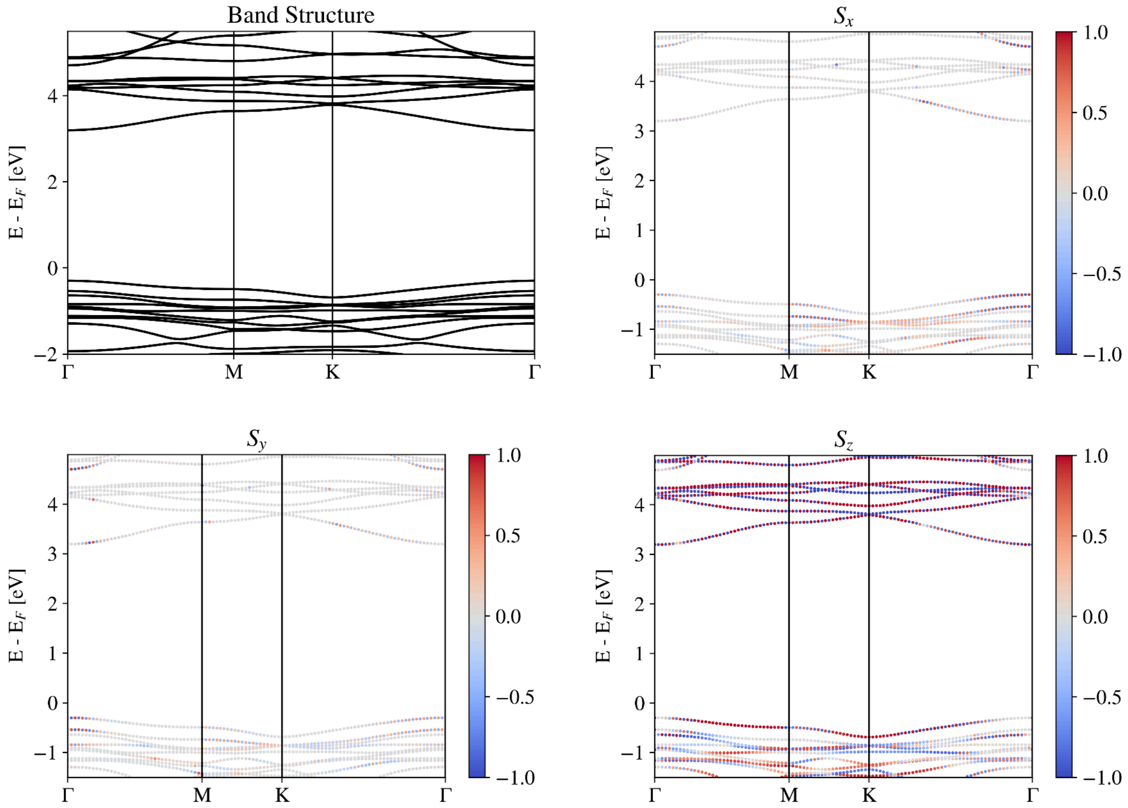


Figure 823: Band structure and spin polarization projections.

## 2.412 Y2Cl6-e9b63ba77c55

- **Formula**: Y2Cl6
- **Structural Cluster**: AB3-19
- **Band gap (PBE)**: 4.421 eV
- **Energy above convex hull (C2DB)**: 0.042 eV
- **Space group symbol**: $P\bar{6}2m$
- **Space group number**: 189
- **Polar structure**: False

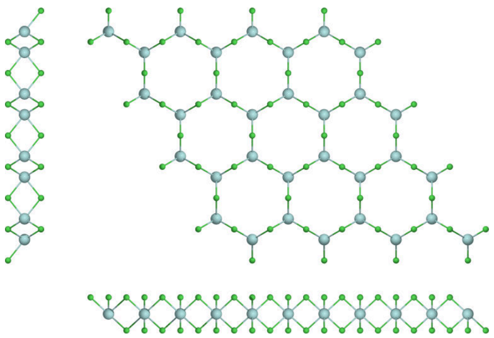

Figure 824: Structure representation

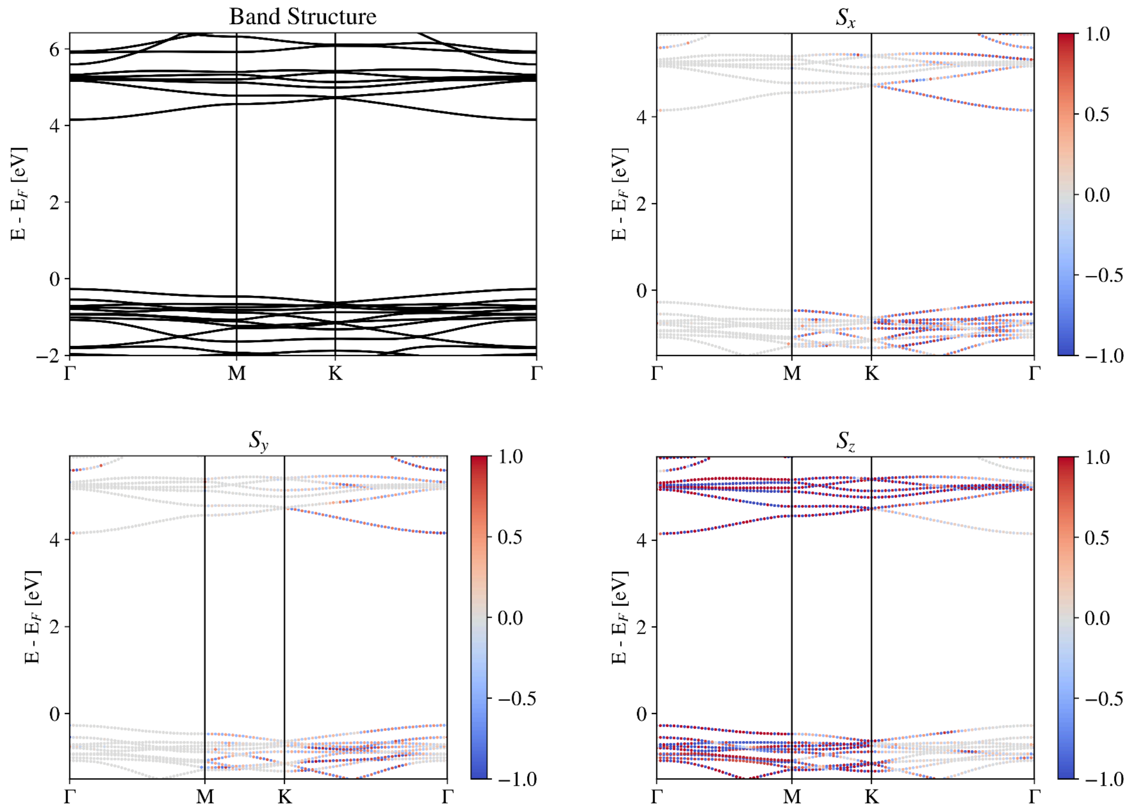


Figure 825: Band structure and spin polarization projections.

## 2.413 Y2I6-d2a7ba833570

- **Formula**: Y2I6
- **Structural Cluster**: AB3-19
- **Band gap (PBE)**: 2.61 eV
- **Energy above convex hull (C2DB)**: 0.065 eV
- **Space group symbol**: $P\bar{6}2m$
- **Space group number**: 189
- **Polar structure**: False

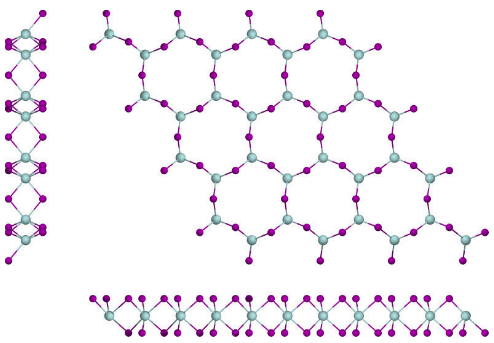

Figure 826: Structure representation

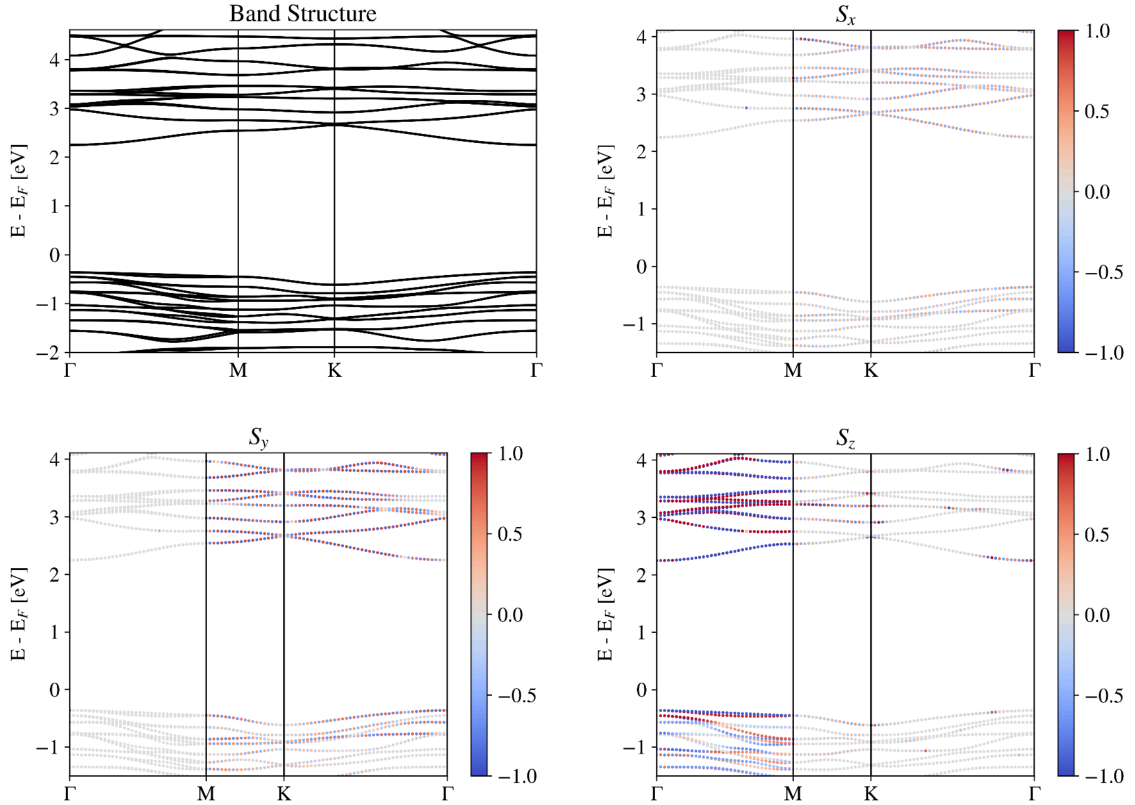


Figure 827: Band structure and spin polarization projections.

## 2.414 ZnBr2-4718298eb660

- **Formula**: ZnBr2
- **Structural Cluster**: AB2-11
- **Band gap (PBE)**: 3.278 eV
- **Energy above convex hull (C2DB)**: 0.0 eV
- **Space group symbol**: $P\bar{4}m2$
- **Space group number**: 115
- **Polar structure**: False

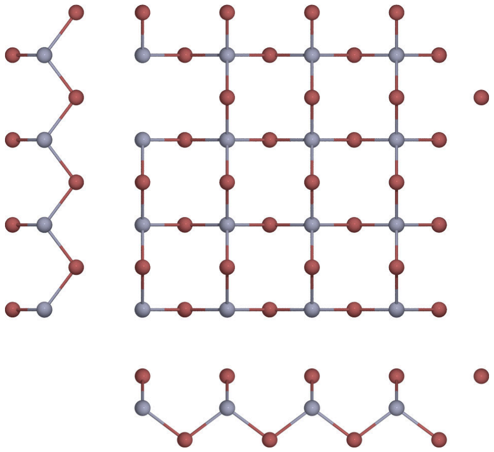

Figure 828: Structure representation

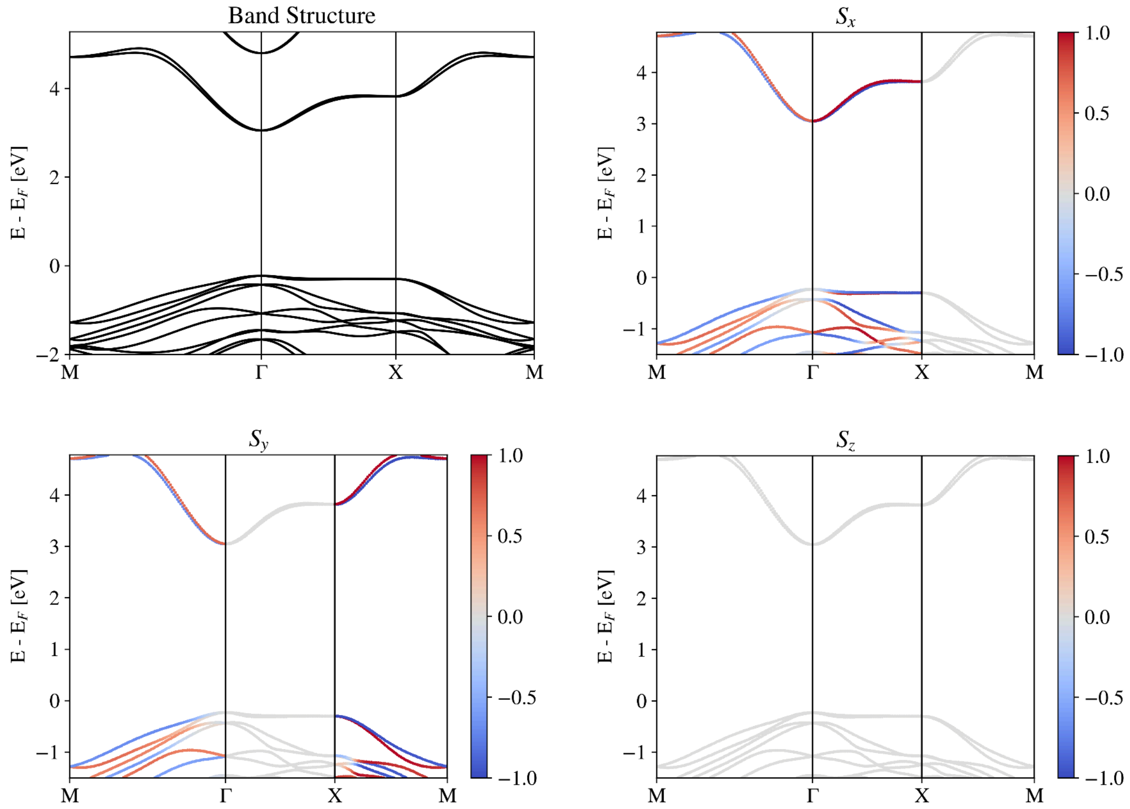


Figure 829: Band structure and spin polarization projections.

## 2.415 ZnBr2-553cb6a56984

- **Formula**: ZnBr2
- **Structural Cluster**: AB2-4
- **Band gap (PBE)**: 2.413 eV
- **Energy above convex hull (C2DB)**: 0.249 eV
- **Space group symbol**: $P\bar{6}m2$
- **Space group number**: 187
- **Polar structure**: False

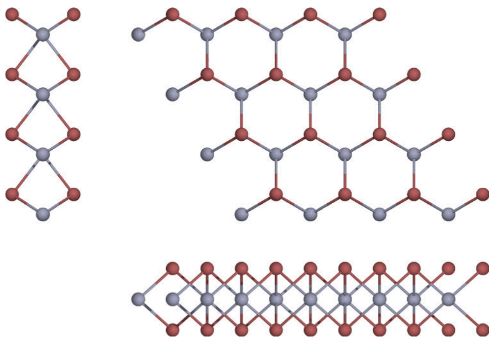

Figure 830: Structure representation

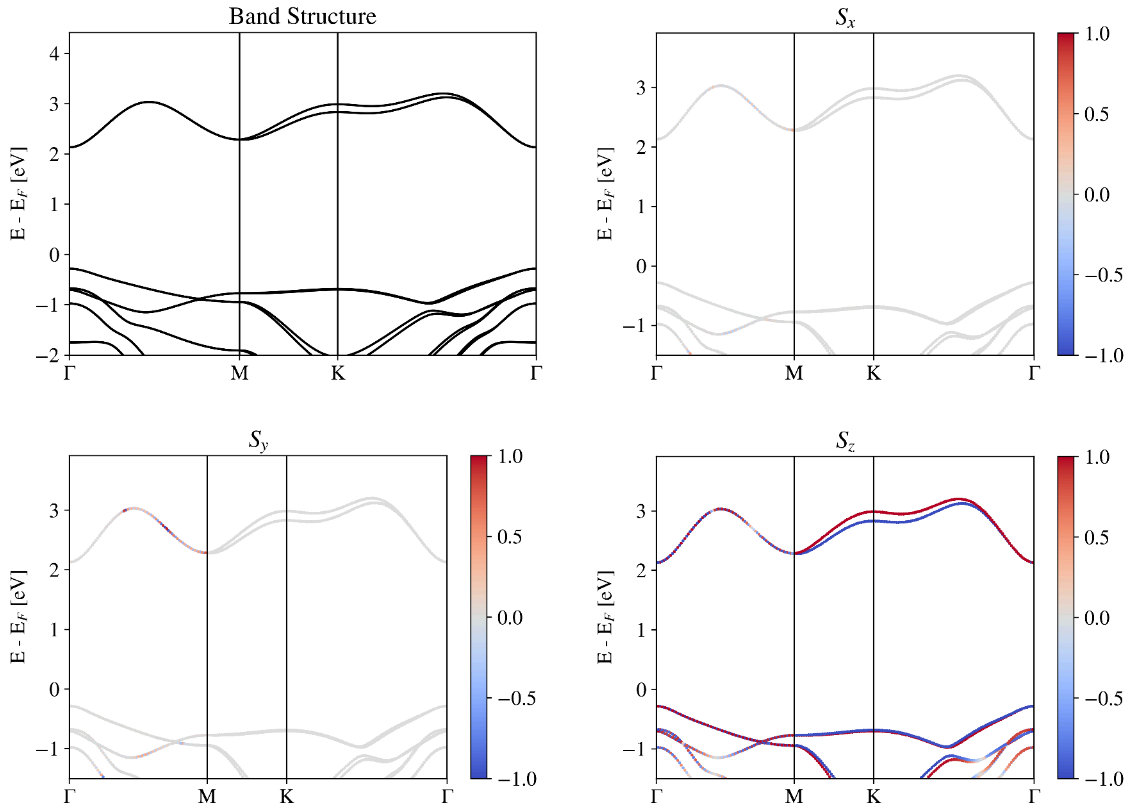


Figure 831: Band structure and spin polarization projections.

## 2.416 ZnCl2-1b7175e04416

- **Formula**: ZnCl2
- **Structural Cluster**: AB2-11
- **Band gap (PBE)**: 4.226 eV
- **Energy above convex hull (C2DB)**: 0.0 eV
- **Space group symbol**: $P\bar{4}m2$
- **Space group number**: 115
- **Polar structure**: False

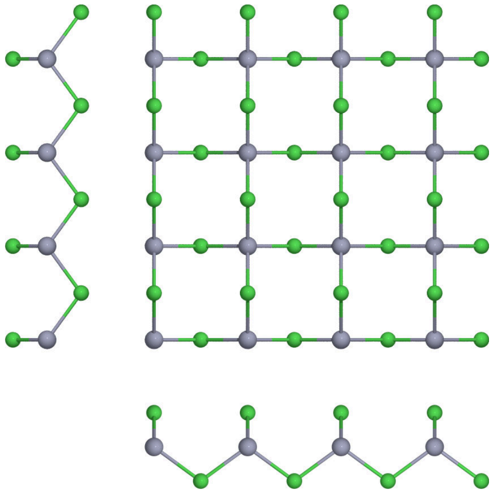

Figure 832: Structure representation

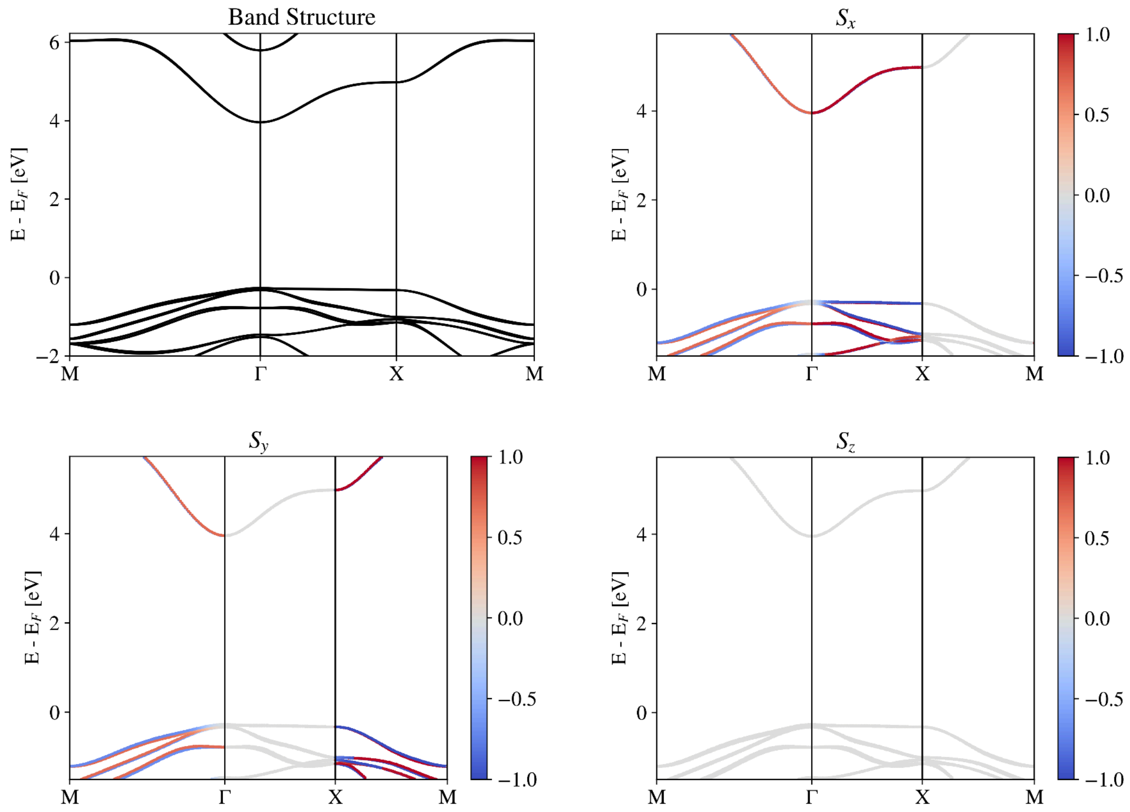


Figure 833: Band structure and spin polarization projections.

## 2.417 ZnCl2-62c6ee7a0a25

- **Formula**: ZnCl2
- **Structural Cluster**: AB2-4
- **Band gap (PBE)**: 3.437 eV
- **Energy above convex hull (C2DB)**: 0.236 eV
- **Space group symbol**: $P\bar{6}m2$
- **Space group number**: 187
- **Polar structure**: False

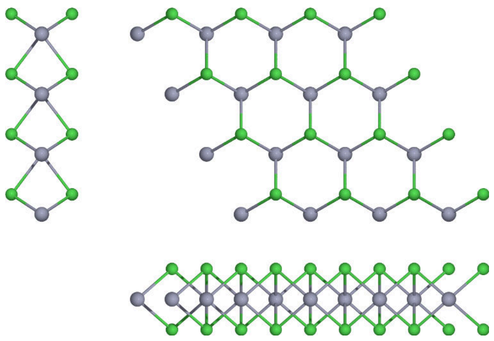

Figure 834: Structure representation

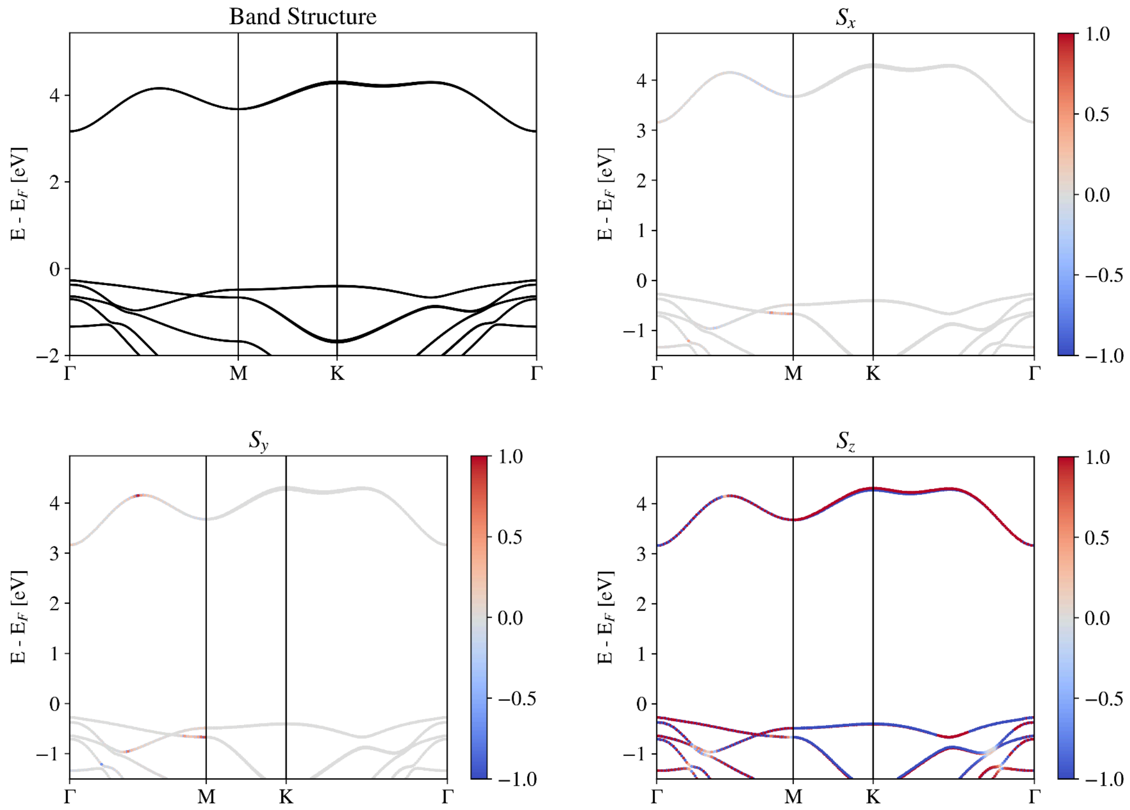


Figure 835: Band structure and spin polarization projections.

## 2.418 ZnF2-a57169b6ba57

- **Formula**: ZnF2
- **Structural Cluster**: AB2-11
- **Band gap (PBE)**: 4.412 eV
- **Energy above convex hull (C2DB)**: 0.112 eV
- **Space group symbol**: $P\bar{4}m2$
- **Space group number**: 115
- **Polar structure**: False

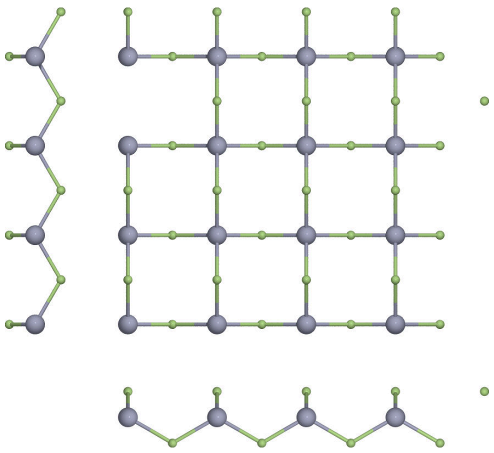

Figure 836: Structure representation

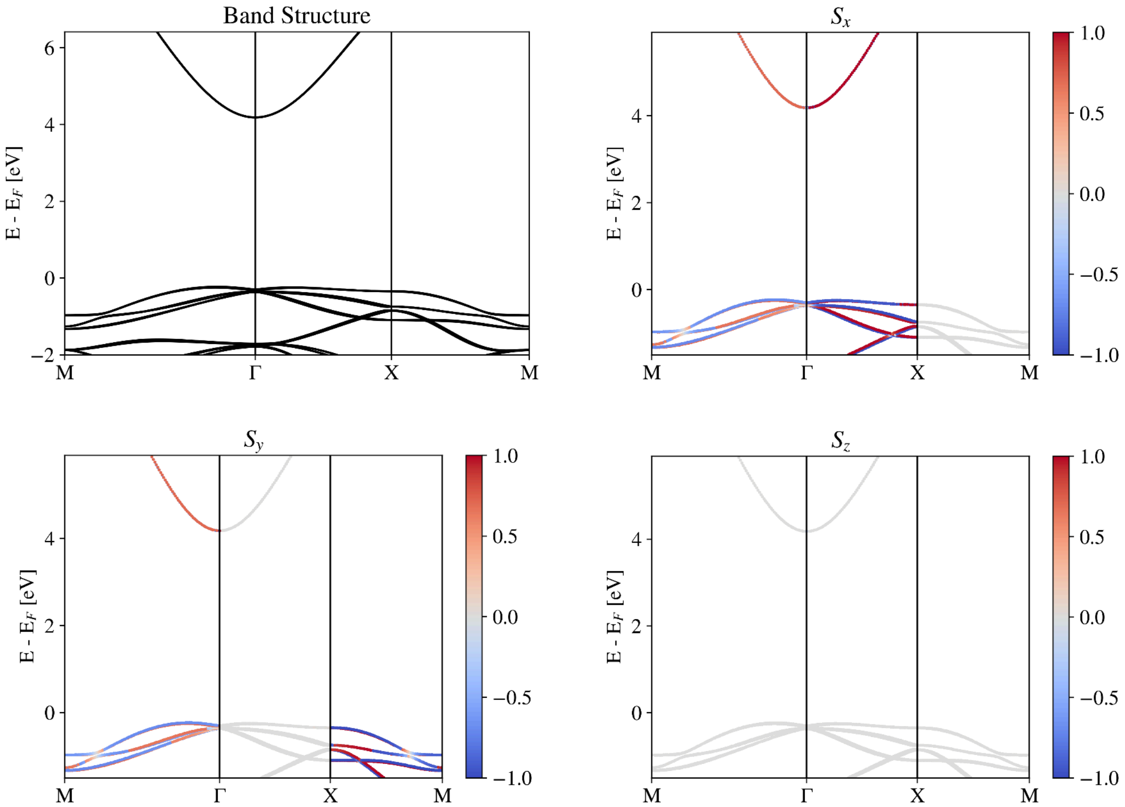


Figure 837: Band structure and spin polarization projections.

## 2.419 ZnI2-701e1fc14b22

- **Formula**: ZnI2
- **Structural Cluster**: AB2-4
- **Band gap (PBE)**: 0.931 eV
- **Energy above convex hull (C2DB)**: 0.302 eV
- **Space group symbol**: $P\bar{6}m2$
- **Space group number**: 187
- **Polar structure**: False

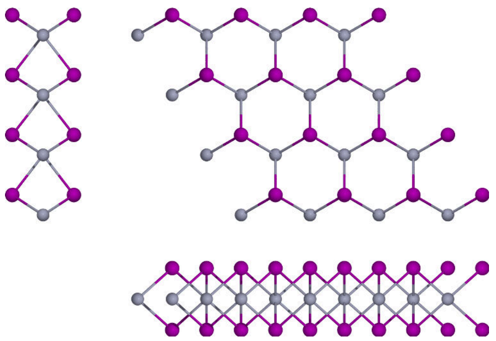

Figure 838: Structure representation

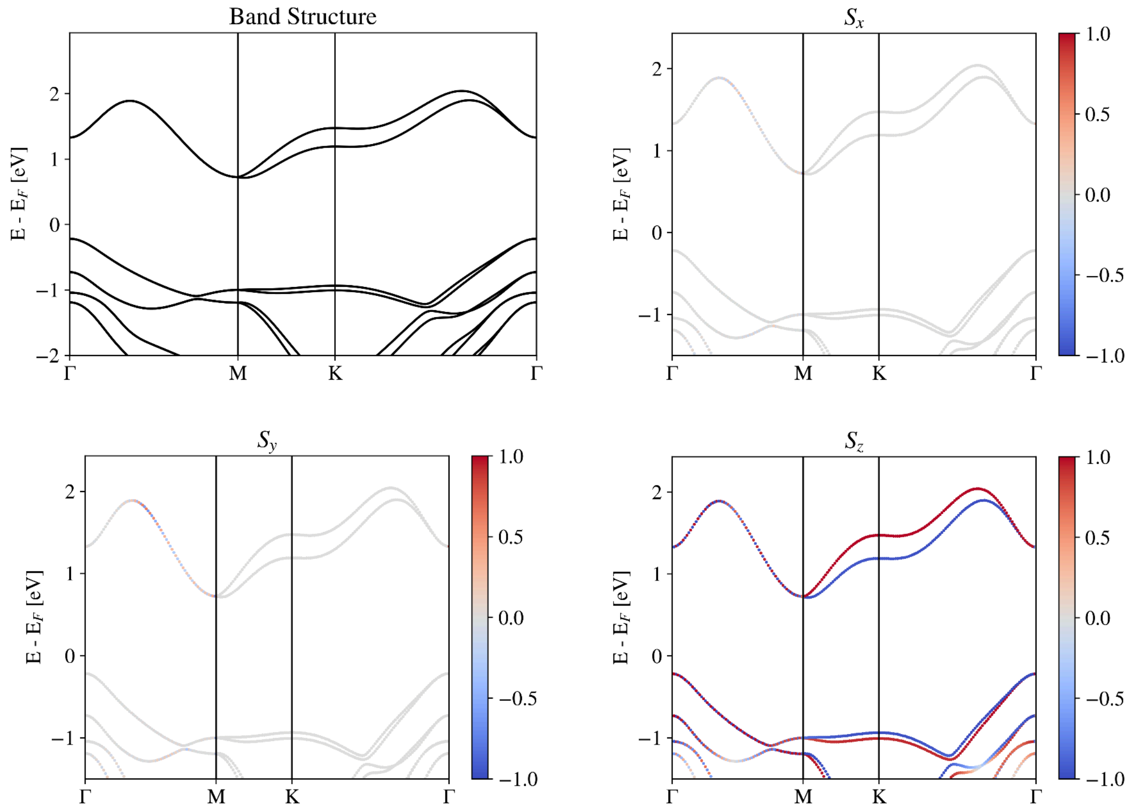


Figure 839: Band structure and spin polarization projections.

## 2.420 ZnI2-ce0e9cd74bb3

- **Formula**: ZnI2
- **Structural Cluster**: AB2-11
- **Band gap (PBE)**: 2.467 eV
- **Energy above convex hull (C2DB)**: 0.0 eV
- **Space group symbol**: $P\bar{4}m2$
- **Space group number**: 115
- **Polar structure**: False

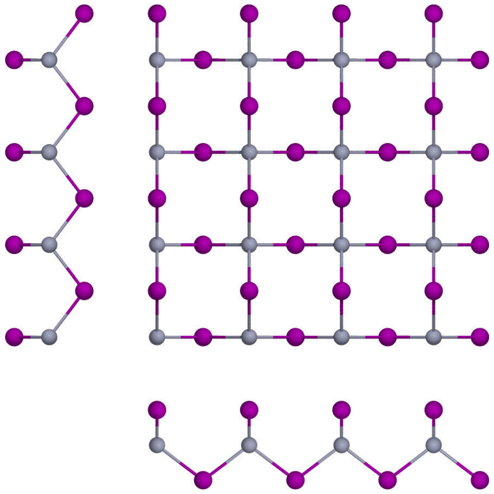

Figure 840: Structure representation

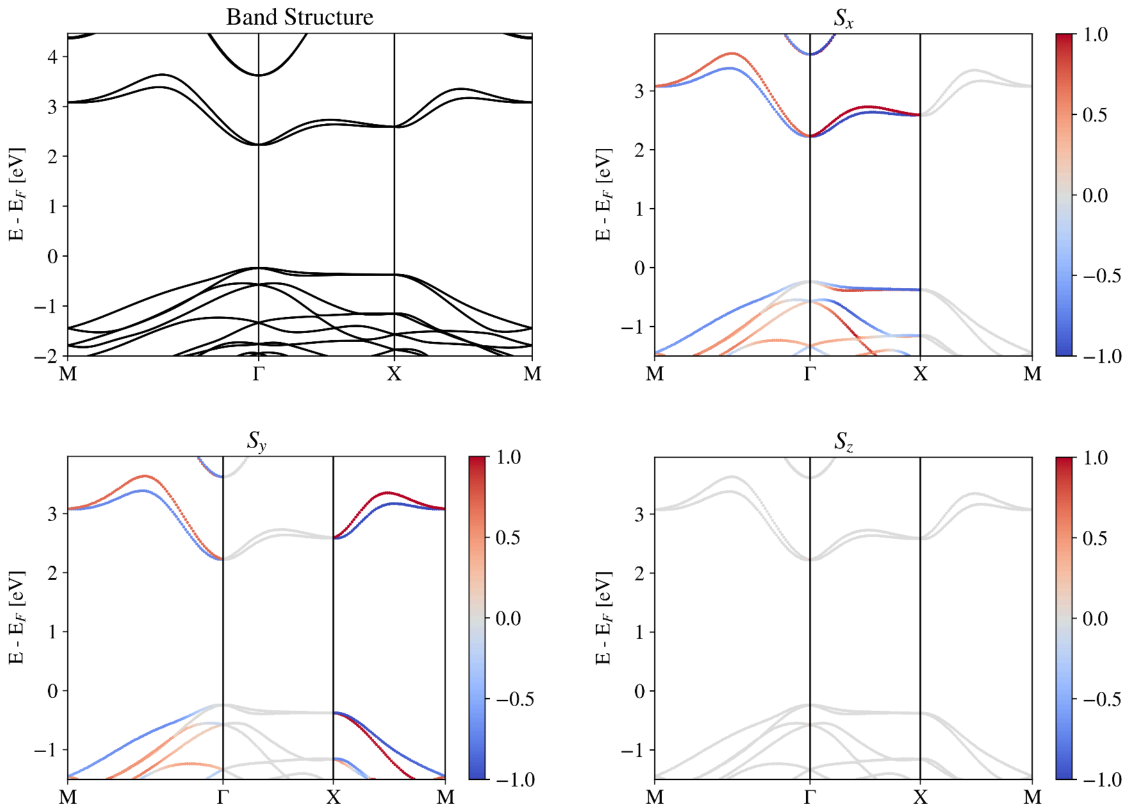


Figure 841: Band structure and spin polarization projections.

## 2.421 ZrBr2-7897c7cc2491

- **Formula**: ZrBr2
- **Structural Cluster**: AB2-4
- **Band gap (PBE)**: 0.827 eV
- **Energy above convex hull (C2DB)**: 0.0 eV
- **Space group symbol**: $P\bar{6}m2$
- **Space group number**: 187
- **Polar structure**: False

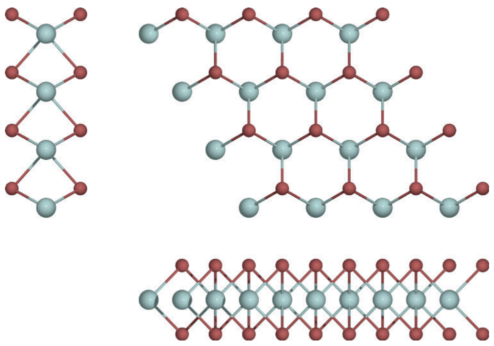

Figure 842: Structure representation

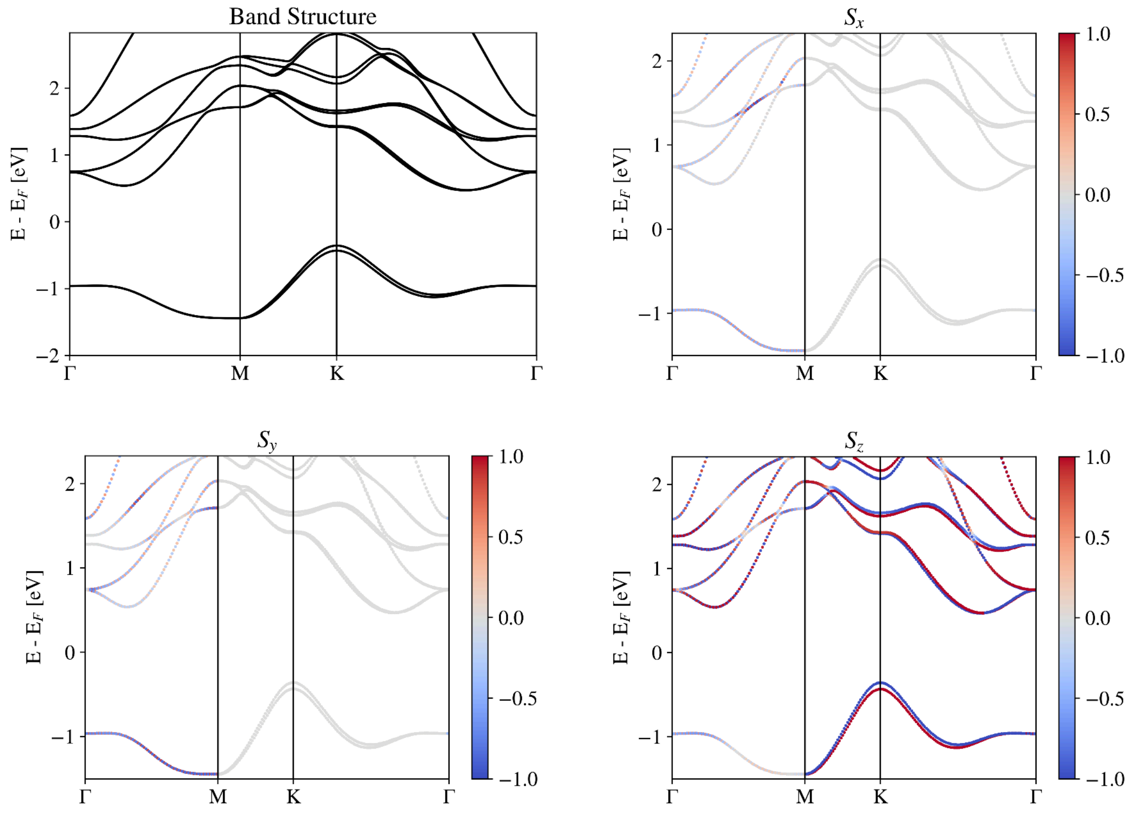


Figure 843: Band structure and spin polarization projections.

## 2.422 ZrCl2-dc09b7c396eb

- **Formula**: ZrCl2
- **Structural Cluster**: AB2-4
- **Band gap (PBE)**: 0.988 eV
- **Energy above convex hull (C2DB)**: 0.0 eV
- **Space group symbol**: $P\bar{6}m2$
- **Space group number**: 187
- **Polar structure**: False

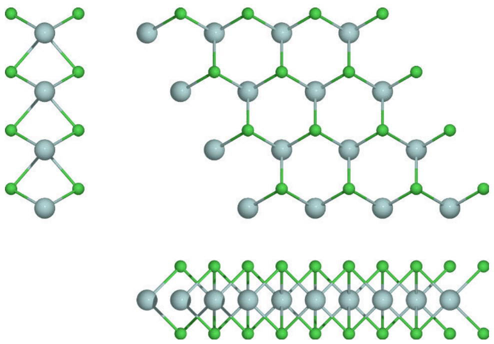

Figure 844: Structure representation

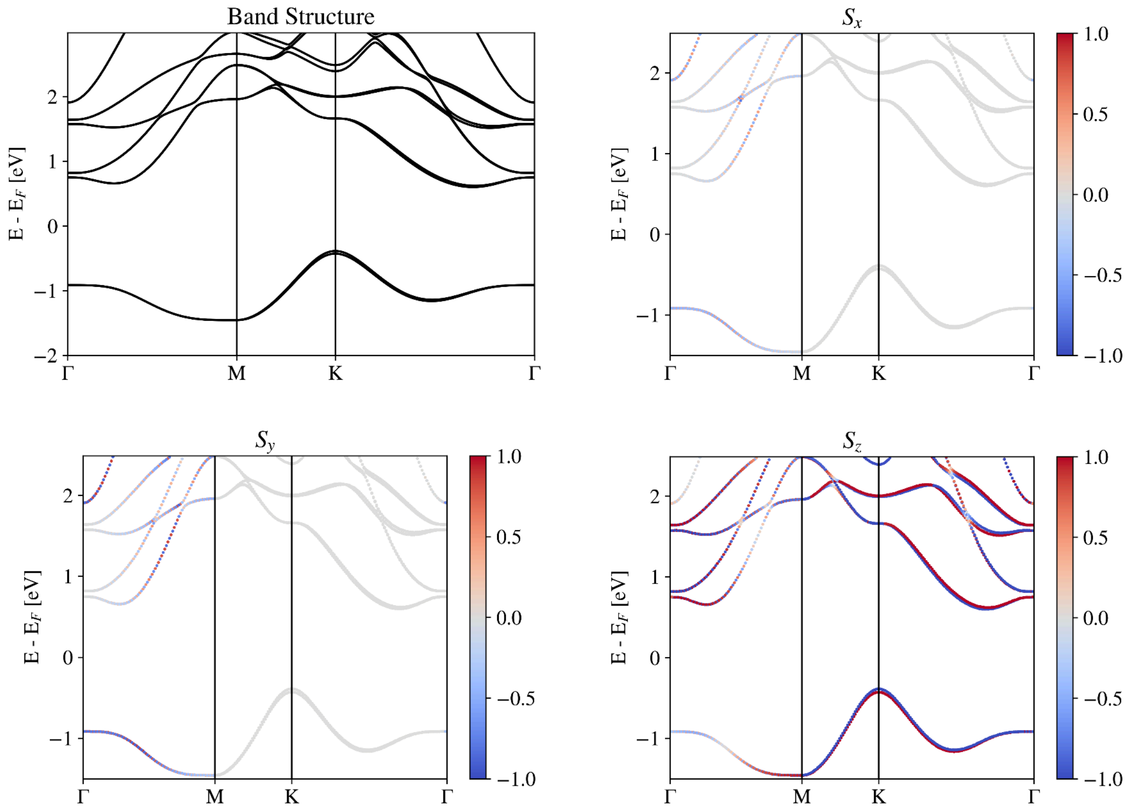


Figure 845: Band structure and spin polarization projections.

## 2.423 ZrHf3S8-9cbc09153aeb

- **Formula**: ZrHf3S8
- **Structural Cluster**: AB3C8-22
- **Band gap (PBE)**: 1.152 eV
- **Energy above convex hull (C2DB)**: 0.208 eV
- **Space group symbol**: $P1$
- **Space group number**: 1
- **Polar structure**: True

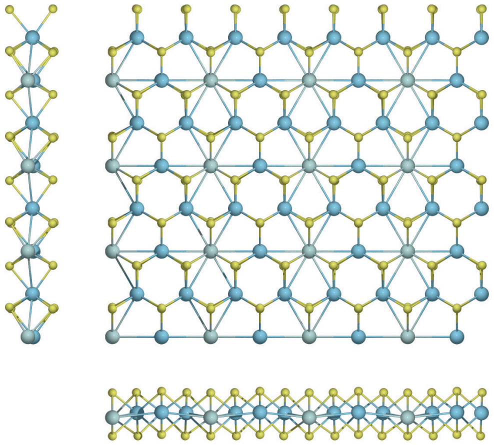

Figure 846: Structure representation

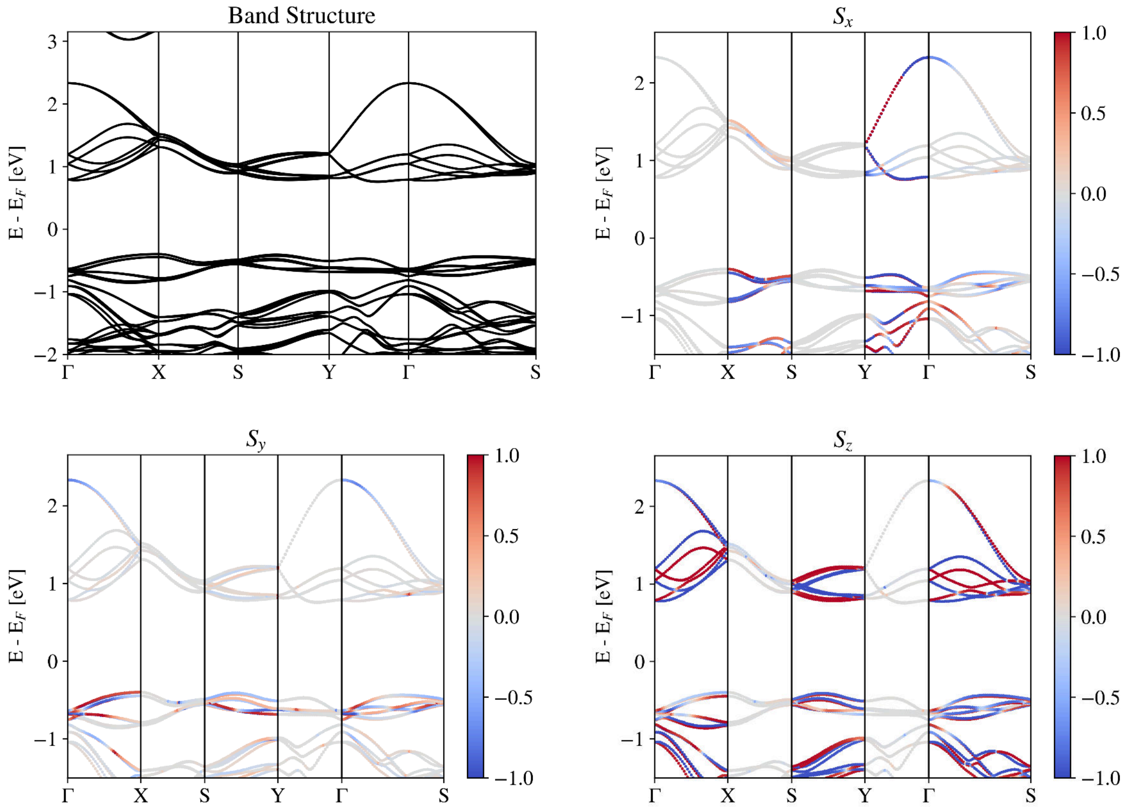


Figure 847: Band structure and spin polarization projections.

## 2.424 ZrHf3Se8-b8fb10416122

- **Formula**: ZrHf3Se8
- **Structural Cluster**: AB3C8-22
- **Band gap (PBE)**: 0.843 eV
- **Energy above convex hull (C2DB)**: 0.165 eV
- **Space group symbol**: $P1$
- **Space group number**: 1
- **Polar structure**: True

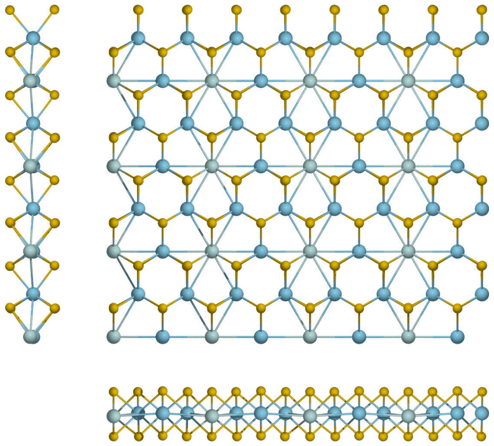

Figure 848: Structure representation

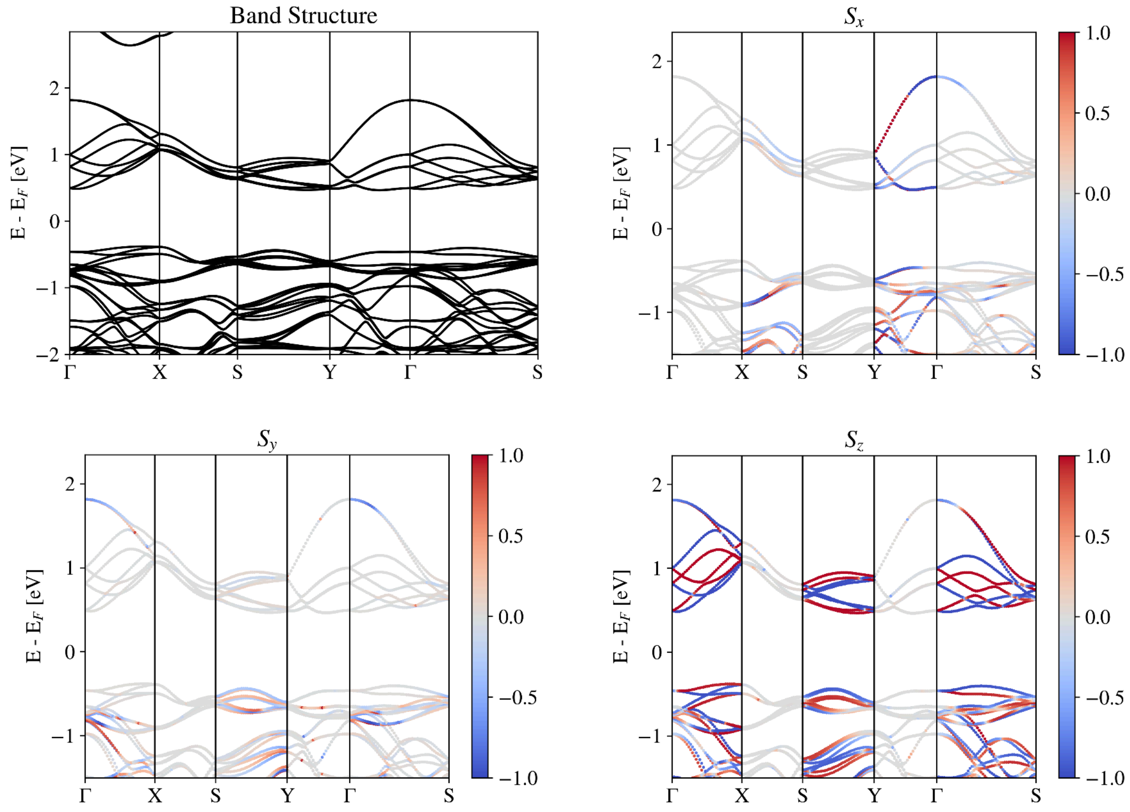


Figure 849: Band structure and spin polarization projections.

## 2.425 ZrHf3Te8-3663f526cdf1

- **Formula**: ZrHf3Te8
- **Structural Cluster**: AB3C8-22
- **Band gap (PBE)**: 0.165 eV
- **Energy above convex hull (C2DB)**: 0.127 eV
- **Space group symbol**: $P1$
- **Space group number**: 1
- **Polar structure**: True

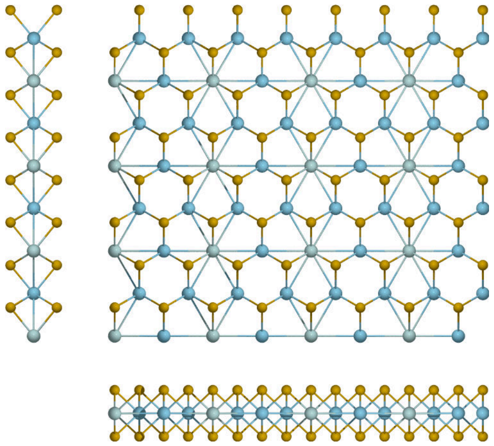

Figure 850: Structure representation

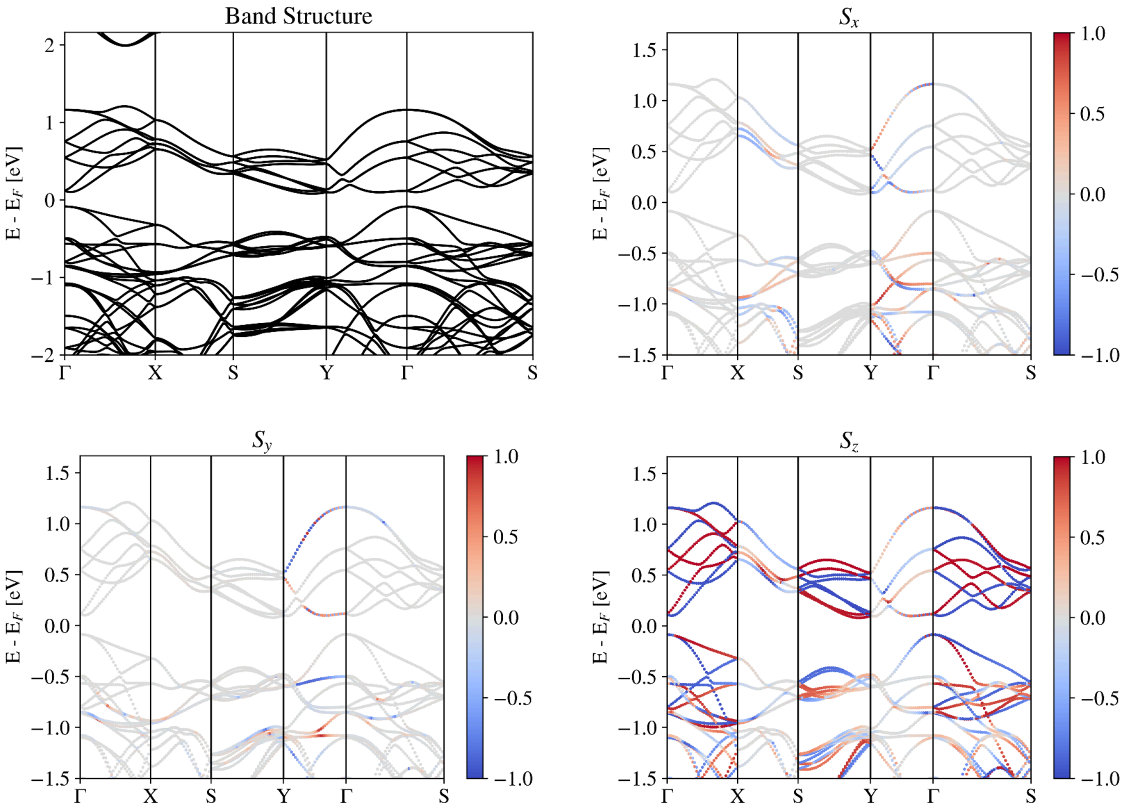


Figure 851: Band structure and spin polarization projections.

## 2.426 ZrI2-9c024b5a2e89

- **Formula**: ZrI2
- **Structural Cluster**: AB2-4
- **Band gap (PBE)**: 0.698 eV
- **Energy above convex hull (C2DB)**: 0.027 eV
- **Space group symbol**: $P\bar{6}m2$
- **Space group number**: 187
- **Polar structure**: False

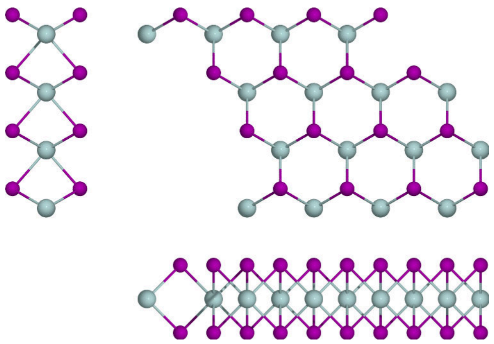

Figure 852: Structure representation

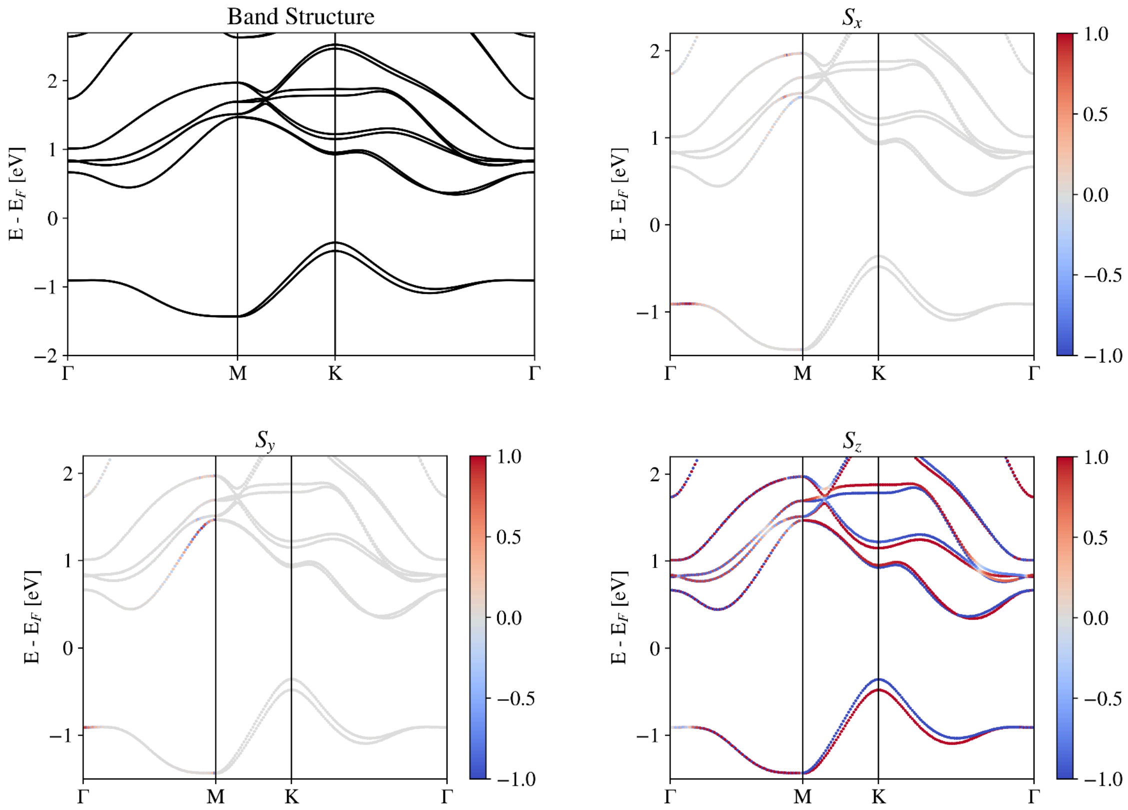


Figure 853: Band structure and spin polarization projections.

## 2.427 ZrO2-24a8929c68ce

- **Formula**: ZrO2
- **Structural Cluster**: AB2-4
- **Band gap (PBE)**: 1.683 eV
- **Energy above convex hull (C2DB)**: 0.766 eV
- **Space group symbol**: $P\bar{6}m2$
- **Space group number**: 187
- **Polar structure**: False

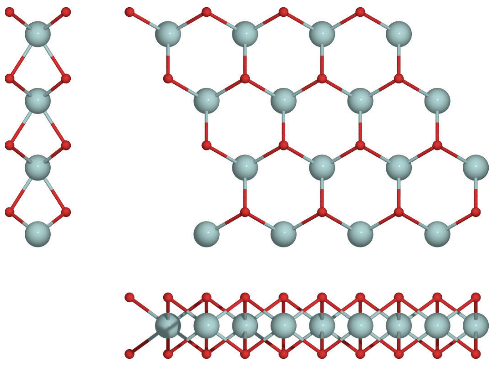

Figure 854: Structure representation

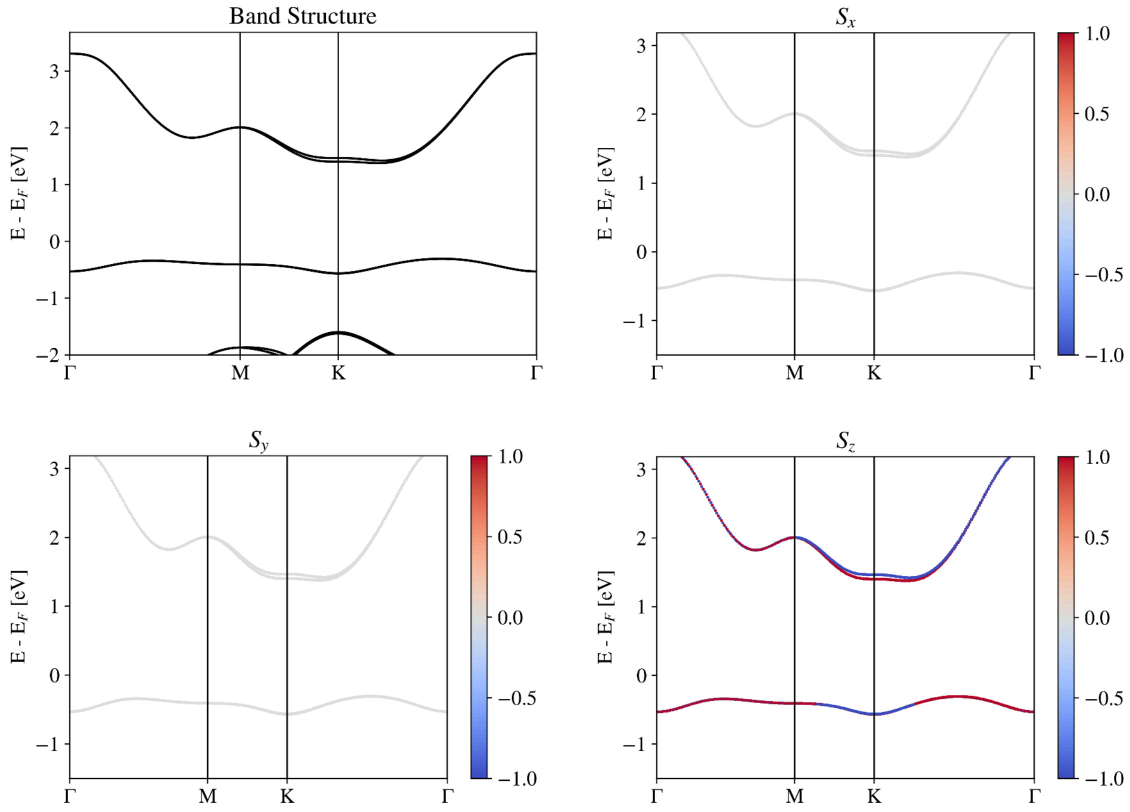


Figure 855: Band structure and spin polarization projections.

## 2.428 ZrO2-da3987f48688

- **Formula**: ZrO2
- **Structural Cluster**: AB2-11
- **Band gap (PBE)**: 4.378 eV
- **Energy above convex hull (C2DB)**: 0.464 eV
- **Space group symbol**: $P\bar{4}m2$
- **Space group number**: 115
- **Polar structure**: False

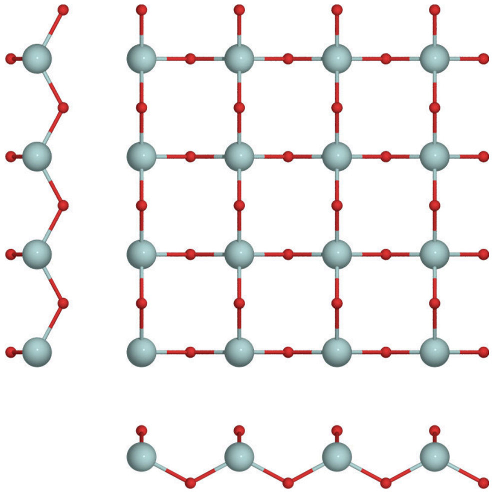

Figure 856: Structure representation

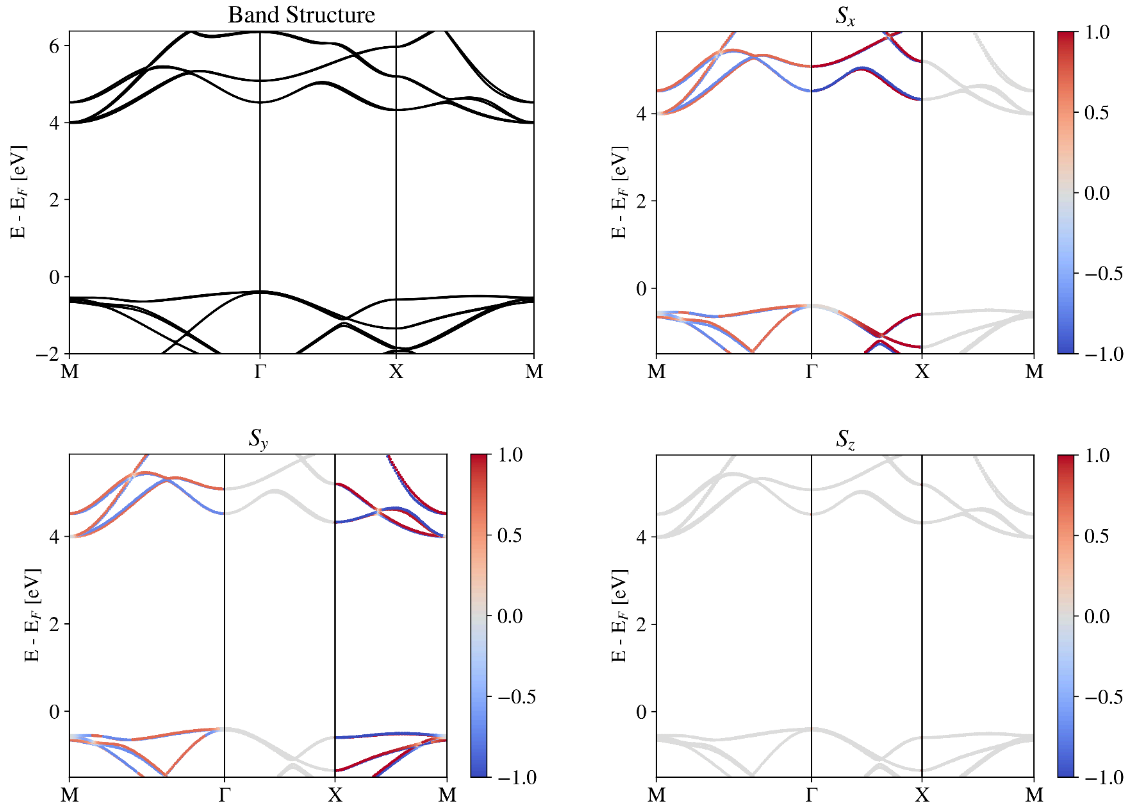


Figure 857: Band structure and spin polarization projections.

## 2.429 ZrS2-1a039e022308

- **Formula**: ZrS2
- **Structural Cluster**: AB2-4
- **Band gap (PBE)**: 0.97 eV
- **Energy above convex hull (C2DB)**: 0.19 eV
- **Space group symbol**: $P\bar{6}m2$
- **Space group number**: 187
- **Polar structure**: False

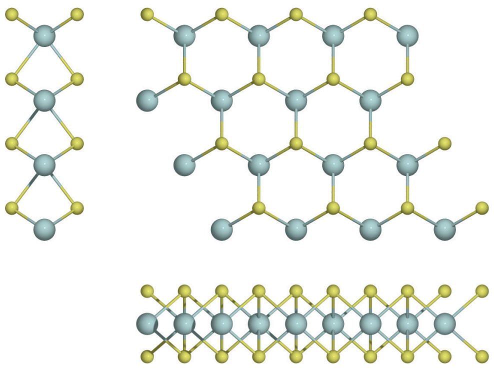

Figure 858: Structure representation

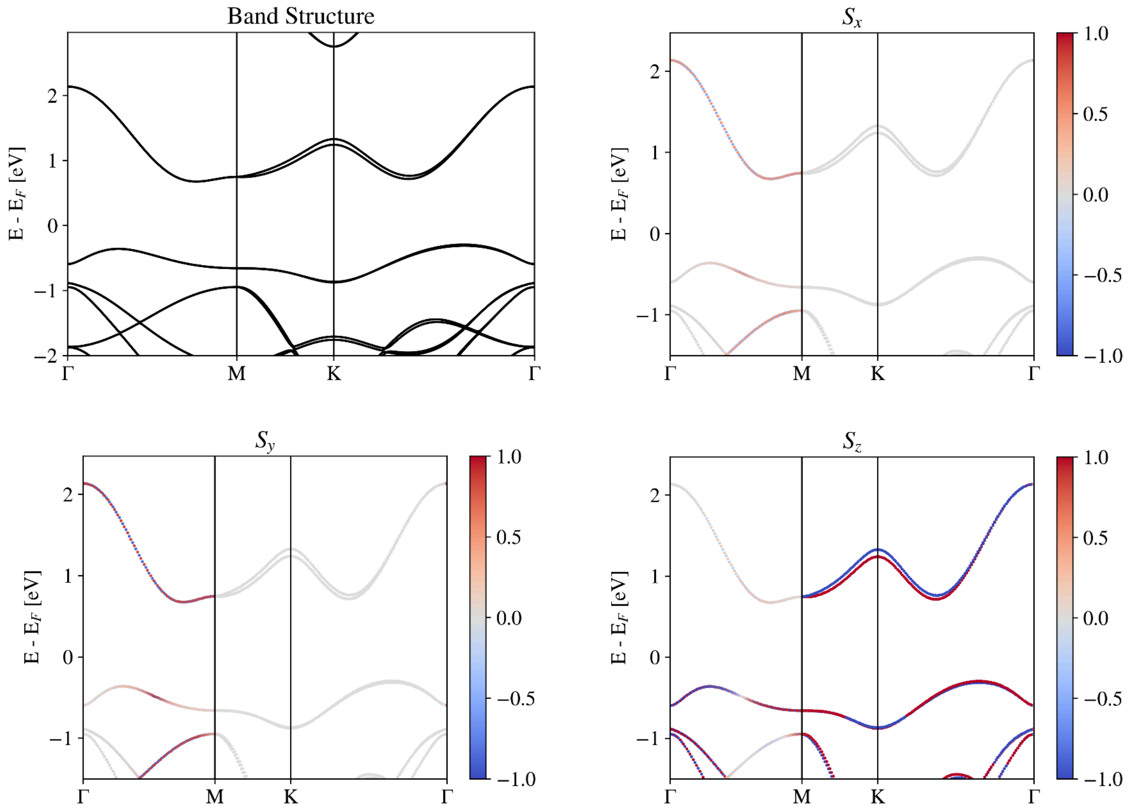


Figure 859: Band structure and spin polarization projections.

## 2.430 ZrS2-2e44a755e594

- **Formula**: ZrS2
- **Structural Cluster**: AB2-11
- **Band gap (PBE)**: 1.938 eV
- **Energy above convex hull (C2DB)**: 0.31 eV
- **Space group symbol**: $P\bar{4}m2$
- **Space group number**: 115
- **Polar structure**: False

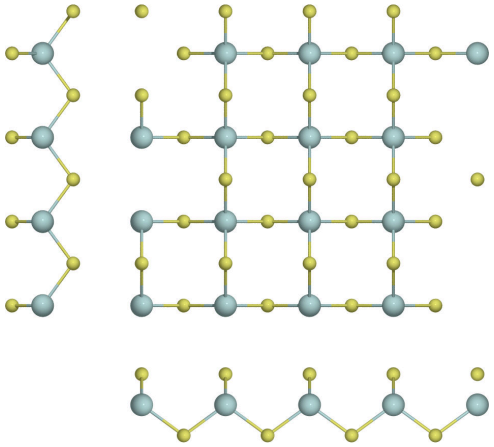

Figure 860: Structure representation

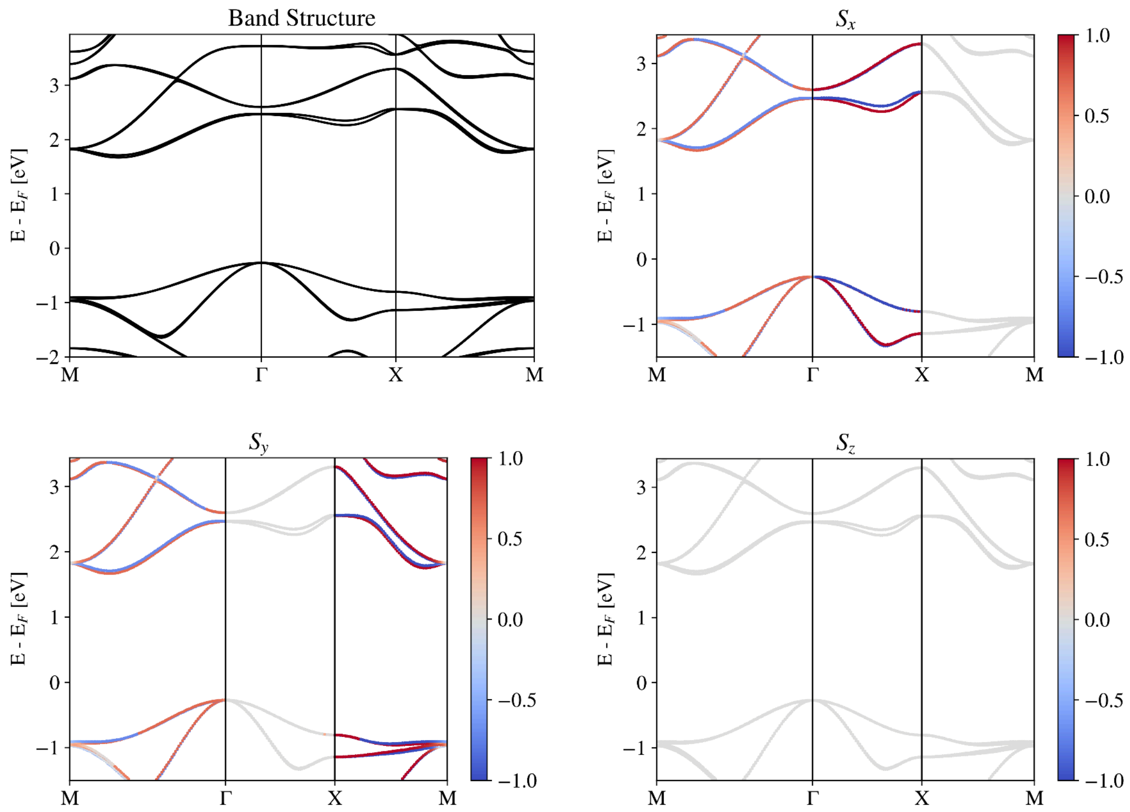


Figure 861: Band structure and spin polarization projections.

## 2.431 ZrSe2-001dfe9a7fa2

- **Formula**: ZrSe2
- **Structural Cluster**: AB2-11
- **Band gap (PBE)**: 1.452 eV
- **Energy above convex hull (C2DB)**: 0.319 eV
- **Space group symbol**: $P\bar{4}m2$
- **Space group number**: 115
- **Polar structure**: False

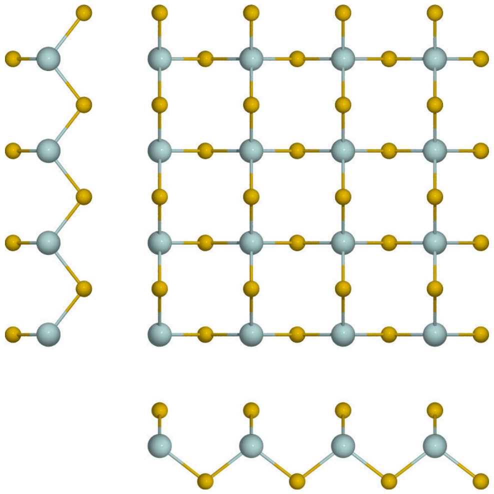

Figure 862: Structure representation

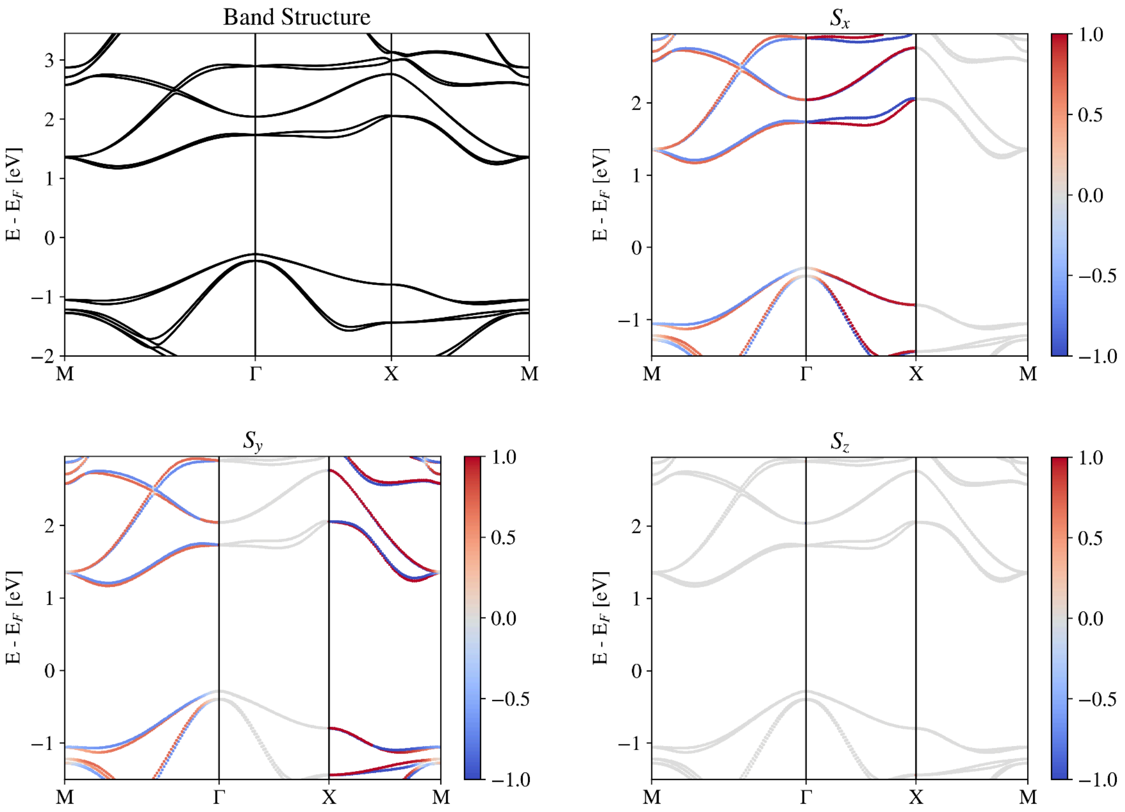


Figure 863: Band structure and spin polarization projections.

## 2.432 ZrSe2-f17029facf63

- **Formula**: ZrSe2
- **Structural Cluster**: AB2-4
- **Band gap (PBE)**: 0.734 eV
- **Energy above convex hull (C2DB)**: 0.144 eV
- **Space group symbol**: $P\bar{6}m2$
- **Space group number**: 187
- **Polar structure**: False

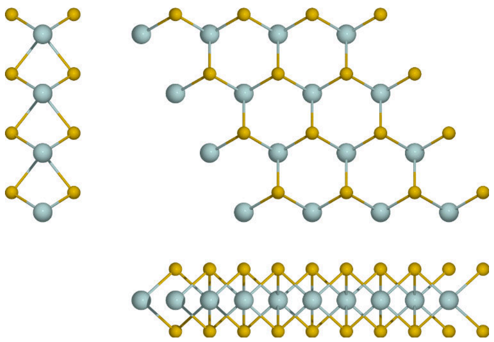

Figure 864: Structure representation

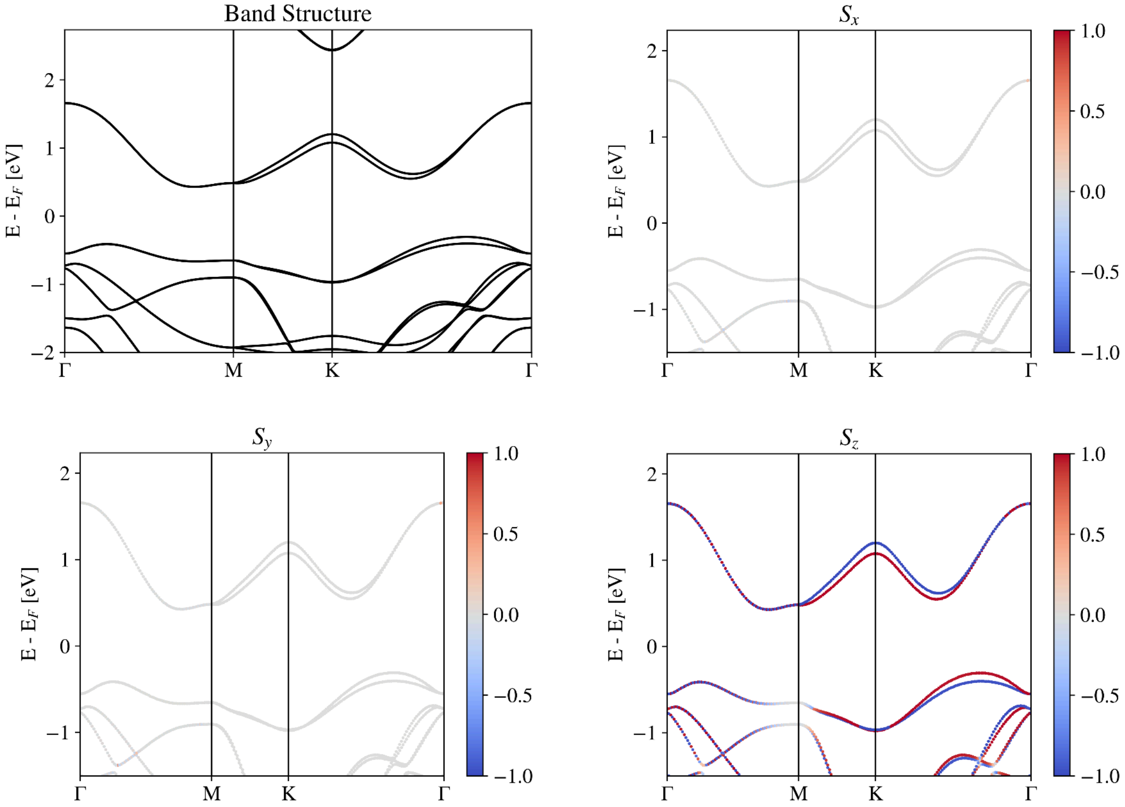


Figure 865: Band structure and spin polarization projections.

## 2.433 ZrTe2-599f0c912458

- **Formula**: ZrTe2
- **Structural Cluster**: AB2-11
- **Band gap (PBE)**: 0.816 eV
- **Energy above convex hull (C2DB)**: 0.373 eV
- **Space group symbol**: $P\bar{4}m2$
- **Space group number**: 115
- **Polar structure**: False

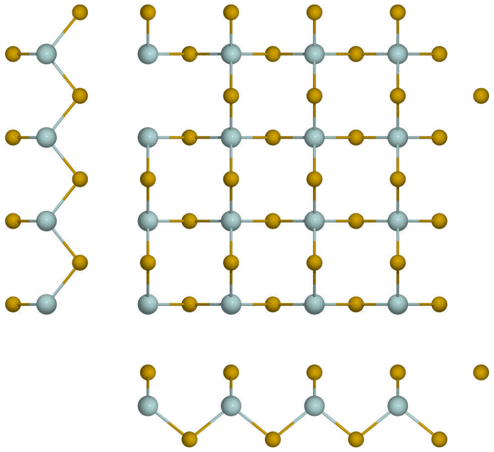

Figure 866: Structure representation

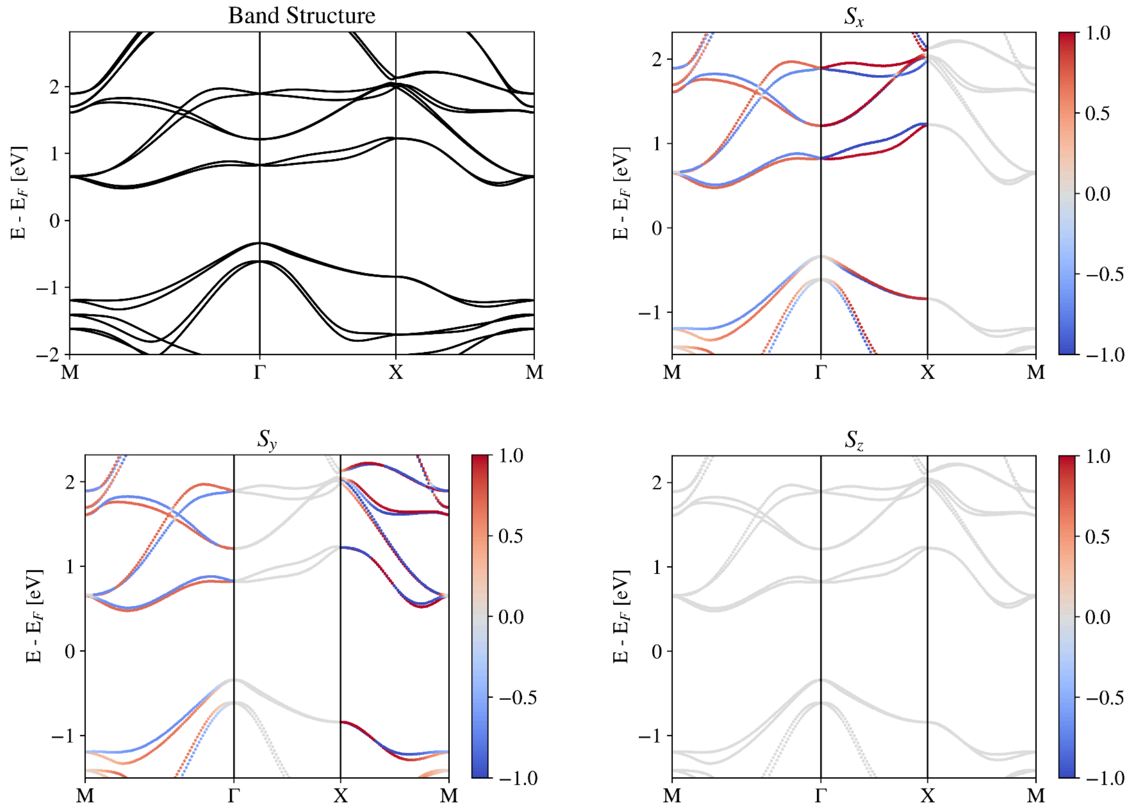


Figure 867: Band structure and spin polarization projections.

## 2.434 ZrTe2-f7ad606317e6

- **Formula**: ZrTe2
- **Structural Cluster**: AB2-4
- **Band gap (PBE)**: 0.275 eV
- **Energy above convex hull (C2DB)**: 0.11 eV
- **Space group symbol**: $P\bar{6}m2$
- **Space group number**: 187
- **Polar structure**: False

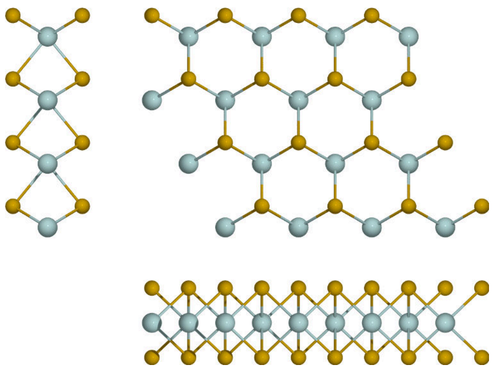

Figure 868: Structure representation

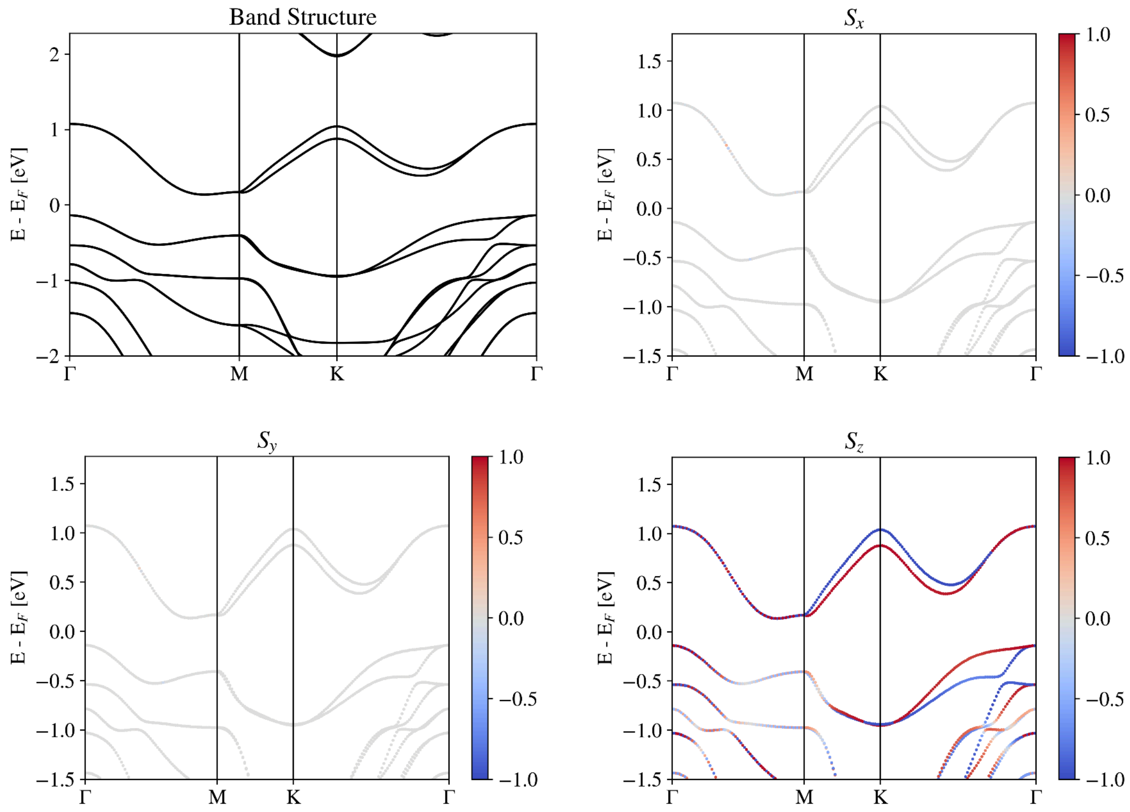


Figure 869: Band structure and spin polarization projections.

## 2.435 ZrTi3S8-e6e376c80c43

- **Formula**: ZrTi3S8
- **Structural Cluster**: AB3C8-22
- **Band gap (PBE)**: 0.833 eV
- **Energy above convex hull (C2DB)**: 0.163 eV
- **Space group symbol**: $P1$
- **Space group number**: 1
- **Polar structure**: True

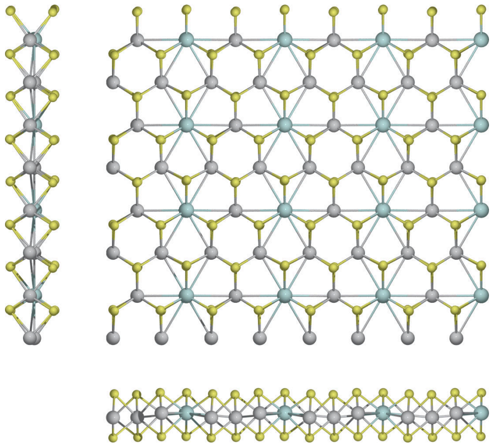

Figure 870: Structure representation

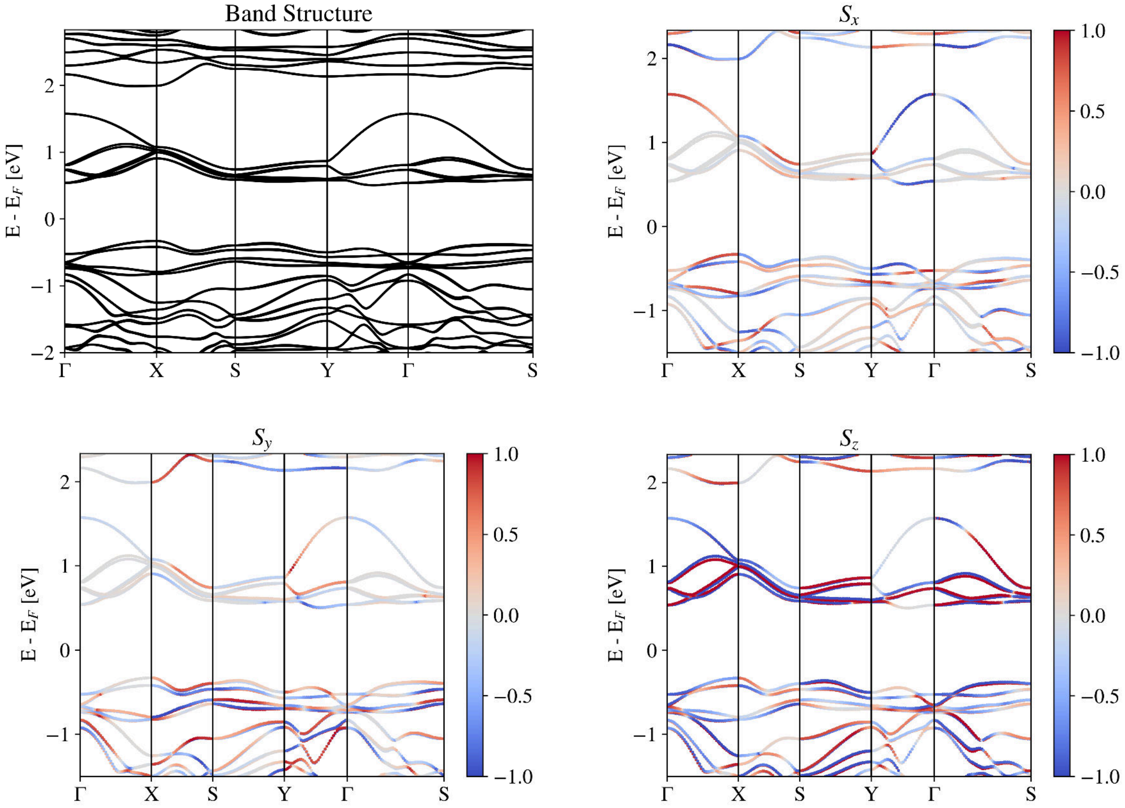


Figure 871: Band structure and spin polarization projections.

## 2.436 ZrTi3Se8-52a5e2b280d4

- **Formula**: ZrTi3Se8
- **Structural Cluster**: AB3C8-22
- **Band gap (PBE)**: 0.571 eV
- **Energy above convex hull (C2DB)**: 0.131 eV
- **Space group symbol**: $P1$
- **Space group number**: 1
- **Polar structure**: True

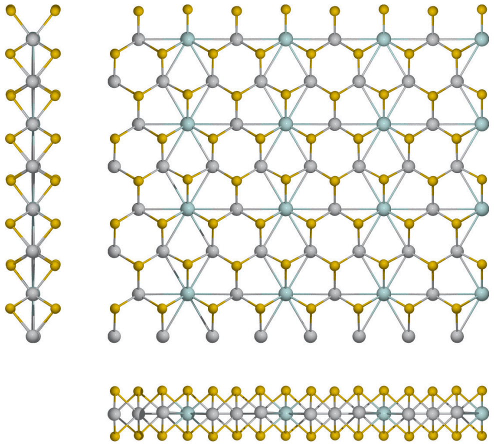

Figure 872: Structure representation

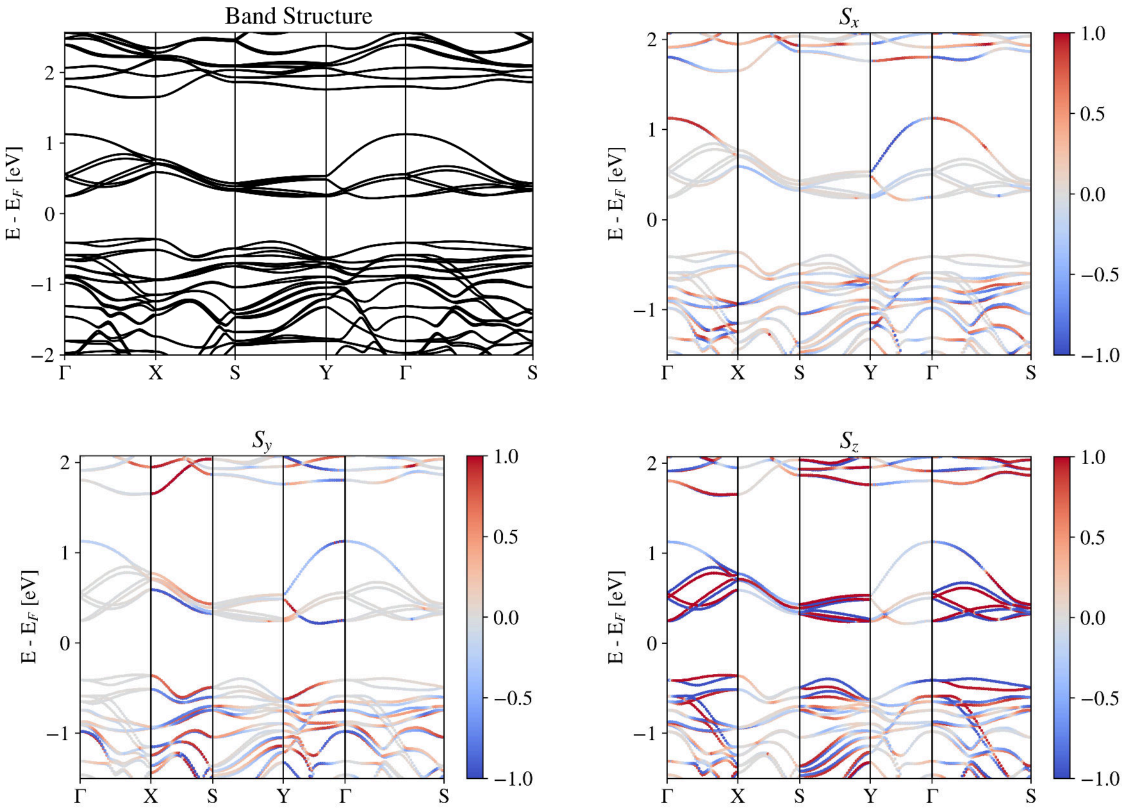


Figure 873: Band structure and spin polarization projections.

## Supplementary References

[1] Setyawan, Wahyu, and Stefano Curtarolo. "High-throughput electronic band structure calculations: Challenges and tools." *Computational materials science* 49.2 (2010): 299-312.

[2] Larsen, Ask Hjorth, et al. "The atomic simulation environment—a Python library for working with atoms." Journal of Physics: Condensed Matter 29.27 (2017): 273002.
