# Supplementary material for: High-throughput inverse design and Bayesian optimization of functionalities: spin splitting in two-dimensional compounds
Source: Sci Data. 2022 Apr 29;9:195. doi: 10.1038/s41597-022-01292-8 (PMC9054849; doi:10.1038/s41597-022-01292-8)
Supplement: Supplementary file 2 [file 41597_2022_1292_MOESM2_ESM.pdf]

Supplementary Information - Rashba SS Table

High-throughput inverse design and optimization of functionalities: spin splitting in two-dimensional compounds

Gabriel M. Nascimento[1,a], Elton Ogoshi[1,a], Adalberto Fazzio[1,2], Carlos Mera Acosta[1,*], and Gustavo M. Dalpian[1,*]

[a] *These authors contributed equally to this work.*
[1] *Center for Natural and Human Sciences, Federal University of ABC, Santo Andre, SP, Brazil*
[2] *Brazilian Nanotechnology National Laboratory (LNNano), CNPEM, 13083-970, Campinas, São Paulo, Brazil*
[*] *Corresponding authors: cmeraacosta@gmail.com; gustavo.dalpian@ufabc.edu.br*

Rashba SS Materials

Table S.1. List of Rashba SS prototypes identified in the valence (V) and/or conduction (C) bands for materials with polar structure. Each material is presented as a combination of chemical formula and ending with its respective ID from the C2DB Database [1]. *SG index* represents the space group symbol (number) of the material's structure according to the precision criteria employed in this work for symmetry identification. ΔE_{hull} is the energy above convex hull reported by the C2DB database. *Bandgap*, *k-path*, α_R, *SS*, ΔE_{SS} and *AC* stand for the energy band gap, k-path between high-symmetry k-points where the SS is identified, Rashba coefficient [eV/Å^{-1}], spin-splitting magnitude, difference in energy between the maximum value of the SS and its respective band edge (VBM or CBM) and the presence of anti-crossing bands, respectively. All energy-related values are in eV.

Entry Info					Spin Splitting Info					
Formula	C2DB ID	SG index	ΔE_{hull}	Bandgap	Band	k-path	α_R	SS	ΔE_{SS}	AC
ISbSe	df0019ec24b5	P3m1 (156)	0.0	1.061	V	M→K	2.431	0.146	0.402	False
					C	Γ→M	1.589	0.156	0.0	True
					C	Γ→K	1.616	0.154	0.0	True
BrSbTe	f1e78a09001d	P3m1 (156)	0.13	1.331	V	M→K	1.315	0.093	0.584	False
BrSbTe	18e62ba75259	P3m1 (156)	0.0	1.089	V	Γ→M	2.631	0.09	0.028	False
					V	Γ→K	2.454	0.108	0.0	False
ClSbSe	f705a30af945	P3m1 (156)	0.146	1.68	V	M→K	1.483	0.118	0.433	False
SSeW	001e03f2c095	P3m1 (156)	0.01	1.417	C	Γ→M	3.284	0.177	0.287	False
					C	Γ→K	3.288	0.214	0.041	False
BiBrTe	f4f45fcade85	P3m1 (156)	0.117	0.916	V	M→K	0.498	0.094	0.7	False
STeW	75ee10091f43	P3m1 (156)	0.086	1.168	C	M→Γ	2.27	0.268	0.188	False
					C	Γ→K	3.947	0.142	0.002	False
MoSTe	2ea941c8bc3c	P3m1 (156)	0.223	0.196	C	M→K	2.358	0.081	0.288	False
					C	Γ→K	2.525	0.224	0.102	False
ISbTe	0f02957b17cf	P3m1 (156)	0.0	0.886	V	Γ→M	3.658	0.076	0.014	True
					V	Γ→K	3.526	0.081	0.0	True
Sn2Te2	03bcf7dcdaf2	Pmn2$_1$(31)	0.063	0.595	C	Y→Γ	4.804	0.081	0.0	True
SeTeW	6e2a4c6f4f57	P3m1 (156)	0.042	1.058	C	M→Γ	1.394	0.182	0.331	False
S2Sn2	7a8373382b33	Pmn2$_1$(31)	0.043	1.434	C	Y→Γ	4.469	0.101	0.0	True
SeSn	d59c96fdfda1	P3m1 (156)	0.098	2.156	V	M→K	0.873	0.113	0.539	False
BiBrS	49b7be14f786	P3m1 (156)	0.0	1.227	C	Γ→M	1.271	0.164	0.0	False
					C	Γ→K	1.293	0.162	0.0	False
AsClTe	fba4cc0df459	P3m1 (156)	0.194	1.316	C	Γ→K	0.634	0.121	0.34	False
ClSbTe	04fdd7d1ec5c	P3m1 (156)	0.153	1.439	V	M→K	1.018	0.15	0.609	False
					C	M→Γ	0.711	0.114	0.081	False

	Entry Info				Spin Splitting Info					
Formula	C2DB ID	SG index	ΔE_{hull}	Bandgap	Band	k-path	α_R	SS	ΔE_{SS}	AC
BiBrSe	de5756e4fbfa	P3m1 (156)	0.0	1.03	V	Γ→M	2.784	0.08	0.022	True
					V	Γ→K	2.634	0.082	0.0	True
					C	Γ→M	1.366	0.14	0.0	True
					C	Γ→K	1.417	0.137	0.0	True
Bi2P2S6	287dcf4f1a19	P1 (1)	0.053	0.953	C	Γ→Y	0.795	0.176	0.0	False
					C	Y→Γ	1.406	0.183	0.0	False
					C	Y→H	1.248	0.245	0.133	False
					C	C→H	0.491	0.245	0.133	False
					C	Γ→X	0.882	0.112	0.022	False
BiClS	99fd027b1d0b	P3m1 (156)	0.12	1.841	C	M→Γ	0.461	0.224	0.231	False
AsBrS	1dcd471c2288	P3m1 (156)	0.034	1.38	V	M→K	3.109	0.125	0.497	False
STeW	916afba26723	P3m1 (156)	0.266	0.191	C	M→Γ	3.622	0.158	0.262	False
					C	M→K	3.414	0.266	0.281	False
					C	Γ→K	0.533	0.118	0.619	False
BiClTe	968a6902b7f5	P3m1 (156)	0.0	0.938	V	M→K	1.175	0.208	0.587	False
					C	M→K	0.992	0.113	1.155	False
AsBrTe	671e6de2497a	P3m1 (156)	0.163	1.098	C	Γ→M	0.694	0.079	0.297	False
					C	Γ→K	0.618	0.142	0.31	False
AsISe	5d829e480507	P3m1 (156)	0.0	1.164	V	M→K	1.626	0.153	0.516	False
					C	Γ→M	1.583	0.116	0.0	False
					C	Γ→K	1.615	0.113	0.0	True
ClSbSe	0c0fbdaf8f4a	P3m1 (156)	0.014	1.177	V	Γ→M	2.682	0.093	0.035	False
					V	Γ→K	2.448	0.094	0.0	False
ClSbTe	da5fd2bb47af	P3m1 (156)	0.008	1.291	V	M→Γ	2.891	0.127	0.034	False
					V	M→K	2.35	0.149	0.324	False
Cr2W2Te8	62bb754c4cb2	Pm (6)	0.082	0.512	V	Γ→X	1.535	0.211	0.0	False
					V	Y→S	1.105	0.207	0.369	False
					V	Γ→S	0.686	0.13	0.16	False
Mo2W2Se8	a1d716aad84d	P1 (1)	0.0	1.288	V	X→Γ	1.784	0.313	0.0	True
BiITe	2d41b3dd1772	P3m1 (156)	0.0	0.701	C	Γ→M	2.065	0.128	0.0	False
					C	M→K	1.391	0.137	1.113	True
					C	Γ→K	2.086	0.127	0.0	False
ZrTi3Se8	52a5e2b280d4	P1 (1)	0.131	0.571	V	S→Y	1.091	0.088	0.042	False
					V	S→Γ	0.48	0.102	0.04	False
BiBrSe	11db0908d9ef	P3m1 (156)	0.111	1.385	C	M→Γ	0.393	0.2	0.413	False
P2Sb2Te6	82b85dfd7723	P1 (1)	0.14	0.633	V	Y→Γ	2.466	0.226	0.007	False
					V	X→Γ	2.596	0.223	0.0	False
					C	Y→Γ	1.057	0.135	0.0	False
					C	X→Γ	0.92	0.134	0.002	False
WCr3S8	dc4259e69783	Pmm2 (25)	0.009	0.887	V	X→Γ	1.317	0.119	0.0	False
Cr2Mo2Te8	988b11badabb	P1 (1)	0.067	0.575	V	X→Γ	1.105	0.139	0.0	False
					V	Y→S	0.842	0.097	0.285	False
					V	Γ→S	0.735	0.095	0.123	False
ISbSe	343d2125478e	P3m1 (156)	0.13	1.078	C	Γ→M	1.17	0.101	0.195	False
					C	Γ→K	0.954	0.122	0.195	False
Bi2P2Te6	cf7927ab6730	P1 (1)	0.14	0.507	C	Γ→Y	1.814	0.139	0.001	False
					C	Y→Γ	1.725	0.091	0.066	False
					C	X→Γ	1.692	0.091	0.065	False
					C	Γ→X	1.809	0.133	0.0	False
HgTe	1a3bdd1b142a	P3m1 (156)	0.165	0.132	V	Γ→M	0.501	0.107	0.01	False
					V	Γ→K	0.618	0.104	0.009	False
BiBrTe	304bc6a92d82	P3m1 (156)	0.0	0.878	V	M→K	1.13	0.112	0.502	True
					C	M→K	1.08	0.117	1.162	True
MoW3S8	2f6f133abcc8	P1 (1)	0.0	1.552	V	X→Γ	2.241	0.354	0.0	False
					C	S→Y	1.202	0.083	0.093	True
BiClSe	7fe9c5cb910c	P3m1 (156)	0.119	1.601	V	M→K	0.679	0.11	0.399	False
					C	M→Γ	0.746	0.33	0.27	False

	Entry Info					Spin Splitting Info				
Formula	C2DB ID	SG index	ΔE_{hull}	Bandgap	Band	k-path	α_R	SS	ΔE_{SS}	AC
WMo3Te8	323fb700d903	P1 (1)	0.005	0.923	V	X→Γ	1.501	0.234	0.0	False
					V	Γ→S	1.292	0.21	0.192	False
AsIS	b13beafa16aa	P3m1 (156)	0.064	1.395	V	M→K	2.033	0.22	0.442	False
					C	Γ→M	1.305	0.151	0.0	False
					C	Γ→K	1.353	0.147	0.0	True
AsITe	114b3382699c	P3m1 (156)	0.162	0.416	C	Γ→M	0.502	0.145	0.259	False
					C	Γ→K	0.579	0.166	0.221	False
WCr3Se8	c798e725e2fb	P1 (1)	0.009	0.698	V	X→Γ	1.16	0.14	0.0	False
BiIS	acdcd16c0d76	P3m1 (156)	0.014	1.139	V	M→K	1.707	0.166	0.244	True
					C	Γ→M	1.858	0.266	0.0	False
					C	M→K	1.074	0.111	1.002	True
					C	Γ→K	1.645	0.297	0.0	False
MoW3Se8	24d6cc0a0fed	Pm (6)	0.0	1.276	V	X→Γ	2.097	0.373	0.0	False
					V	Γ→S	1.24	0.236	0.293	False
Cr2Mo2S8	72b286460831	Pma2 (28)	0.017	1.039	V	X→Γ	1.344	0.095	0.0	False
					V	Y→S	0.838	0.08	0.443	False
ISSb	4c49d27e66e5	P3m1 (156)	0.185	0.872	C	Γ→M	0.887	0.113	0.409	False
					C	Γ→K	0.907	0.11	0.409	False
SnTe	e688959ea45b	P3m1 (156)	0.119	1.592	V	M→K	0.806	0.231	1.011	False
Ti2Zr2Se8	846b50801a93	P1 (1)	0.142	0.616	V	S→Y	1.613	0.098	0.0	False
					V	Y→S	1.767	0.098	0.0	False
					V	Γ→S	0.552	0.094	0.061	False
GeTe	eadd37f03ca5	P3m1 (156)	0.087	1.488	V	M→K	0.952	0.219	1.322	True
MoCr3Te8	899032b4ad0c	P1 (1)	0.087	0.481	V	Γ→Y	1.409	0.104	0.0	False
					V	Γ→S	0.713	0.097	0.137	False
BiIS	40034665f9f1	P3m1 (156)	0.14	0.848	C	Γ→M	1.224	0.144	0.0	False
					C	Γ→K	1.254	0.141	0.0	False
BiISe	433f707c632c	P3m1 (156)	0.114	0.84	C	Γ→M	1.228	0.153	0.0	False
					C	Γ→K	1.255	0.152	0.0	False
BiITe	a84d988e38ac	P3m1 (156)	0.11	0.691	C	M→Γ	0.467	0.132	0.566	False
O2Pb2	20f098bd3f31	Pm (6)	0.287	0.215	V	Γ→X	1.132	0.178	0.0	False
					V	Γ→Y	0.95	0.091	0.138	True
					V	Γ→S	0.875	0.132	0.036	False
					C	S→X	1.405	0.132	0.007	False
					C	S→Y	1.726	0.158	0.0	False
					C	Y→Γ	1.914	0.083	2.264	False
					C	S→Γ	1.723	0.151	0.001	False
PbSe	a0dbdc6630fa	P3m1 (156)	0.217	1.68	V	M→K	0.855	0.166	0.727	False
					C	M→Γ	1.603	0.31	0.298	False
Bi2P2Se6	aa9a981d89aa	P1 (1)	0.054	0.875	V	Γ→Y	4.457	0.217	0.005	False
					V	X→Γ	2.074	0.211	0.0	False
					V	Γ→X	4.384	0.211	0.0	False
					C	Γ→Y	1.971	0.113	0.002	False
					C	Y→Γ	1.66	0.157	0.015	False
					C	X→Γ	1.729	0.166	0.011	False
					C	Γ→X	2.029	0.113	0.0	False
HfTi3Se8	c55716558616	P1 (1)	0.137	0.589	V	S→Y	1.191	0.095	0.054	False
Mo2W2Te8	c04fc052f2ca	Pm (6)	0.011	0.879	V	X→Γ	1.645	0.259	0.0	False
					V	Γ→S	1.315	0.217	0.224	False
					C	Γ→S	1.35	0.086	0.217	False
BiISe	70cbc0e44d36	P3m1 (156)	0.0	0.929	V	M→K	1.267	0.087	0.357	False
					C	Γ→M	2.045	0.232	0.0	False
					C	M→K	1.142	0.115	1.099	False
					C	Γ→K	2.089	0.228	0.0	True
Pb2Te6	3995fa1bee6e	$P2_1$(4)	0.129	0.322	V	X→Γ	3.064	0.184	0.214	False
					V	Γ→S	0.511	0.089	0.195	False
					C	X→Γ	1.451	0.107	0.052	False

	Entry Info				Spin Splitting Info					
Formula	C2DB ID	SG index	ΔE_{hull}	Bandgap	Band	k-path	α_R	SS	ΔE_{SS}	AC
					C	X→S	0.796	0.225	0.0	False
					C	S→Γ	1.059	0.1	0.407	False
Cr2W2S8	5974b6403c31	Pma2 (28)	0.014	0.967	V	X→Γ	1.592	0.179	0.0	True
					V	Y→S	1.151	0.079	0.562	False
HfZr3Se8	70e7ab872359	P1 (1)	0.15	0.819	V	Γ→S	0.95	0.083	0.025	False
GeSe	211bcb7f05d6	P3m1 (156)	0.04	2.215	V	M→K	0.94	0.107	0.86	False
Hf2Zr2Se8	81af2831dbb2	P1 (1)	0.158	0.845	V	Γ→S	0.698	0.088	0.022	False
MoW3Te8	5c3fe56a1a89	Pm (6)	0.018	0.825	V	X→Γ	2.045	0.311	0.0	False
					V	Γ→S	1.298	0.239	0.254	False
Mo2W2S8	449640ec4d30	Pc (7)	0.0	1.553	V	X→Γ	1.954	0.278	0.0	True
					C	S→Y	1.167	0.086	0.115	True
CrW3S8	a9f87eba4b96	Pm (6)	0.009	1.144	V	X→Γ	1.909	0.28	0.0	False
CrMo3S8	644f7c1c85c7	Pm (6)	0.011	1.206	V	X→Γ	1.46	0.117	0.0	False
AsClTe	4fd8ad708fb0	P3m1 (156)	0.018	1.496	V	M→Γ	2.895	0.136	0.038	False
					V	M→K	2.257	0.127	0.462	False
CrW3Te8	eef072f845ce	P1 (1)	0.056	0.563	V	X→Γ	1.592	0.251	0.0	False
					V	Y→S	1.042	0.108	0.472	False
					V	Γ→S	0.99	0.204	0.223	False
ISSb	5b94060698bc	P3m1 (156)	0.041	1.276	V	M→K	2.844	0.216	0.306	False
					C	Γ→M	1.507	0.195	0.0	False
					C	Γ→K	1.547	0.19	0.0	False
MoCr3S8	3fb52099b370	P1 (1)	0.011	0.922	V	X→Γ	1.221	0.08	0.0	False
CrW3Se8	0b7696e1f4c9	P1 (1)	0.01	0.902	V	X→Γ	1.756	0.303	0.0	False
TiHf3Te8	1667d1443160	P1 (1)	0.132	0.098	V	S→Y	1.512	0.089	0.334	False
CrMo3Te8	159f028a85d0	P1 (1)	0.035	0.665	V	Y→S	0.896	0.094	0.328	False
Ti2Zr2Te8	18e377cce57f	P1 (1)	0.118	0.143	V	S→Y	1.855	0.213	0.166	False
PbS	5e4ff1f56b4a	P3m1 (156)	0.231	1.979	C	M→Γ	1.241	0.292	0.266	True
PbTe	3bc08d486d65	P3m1 (156)	0.198	1.151	V	M→K	1.138	0.162	1.013	True
					C	M→Γ	2.18	0.319	0.208	False
P2Sb2Se6	5d1a32a28ffa	P1 (1)	0.058	1.004	V	Y→Γ	2.483	0.142	0.008	False
					V	X→Γ	2.546	0.142	0.0	False
					C	Y→Γ	1.261	0.107	0.002	False
					C	X→Γ	1.339	0.116	0.0	False
BiClTe	badda86cab42	P3m1 (156)	0.129	0.948	C	M→Γ	1.458	0.441	0.209	False
					C	M→K	0.863	0.123	0.52	True
TiZr3Te8	4f1ab08988cc	P1 (1)	0.115	0.21	V	S→Y	1.679	0.113	0.177	False
TiHf3Se8	3e1923c616ad	P1 (1)	0.166	0.722	V	S→Y	1.256	0.087	0.037	False
					V	S→Γ	0.626	0.106	0.029	False
Cr2Mo2Se8	60065d3bbcf2	P1 (1)	0.016	0.837	V	X→Γ	1.173	0.122	0.0	False
					V	Y→S	1.124	0.095	0.401	False
CrMo3Se8	a7233837cfe9	P1 (1)	0.01	0.971	V	X→Γ	1.288	0.148	0.0	False
					V	Y→S	1.311	0.097	0.498	False
					V	Γ→S	0.779	0.115	0.185	False
ZrHf3Se8	b8fb10416122	P1 (1)	0.165	0.843	V	S→Γ	0.725	0.097	0.023	False
WCr3Te8	6523c349753c	P1 (1)	0.097	0.459	V	X→Γ	0.99	0.135	0.0	False
Hf2Ti2Se8	cce78d90e899	P1 (1)	0.156	0.656	V	S→Y	1.409	0.109	0.0	False
					V	S→Γ	0.553	0.103	0.018	False
BiClSe	a80866a2c6b4	P3m1 (156)	0.0	1.139	V	Γ→M	2.393	0.118	0.018	True
					V	M→K	1.782	0.09	0.362	False
					V	Γ→K	2.216	0.124	0.0	True
					C	M→Γ	3.032	0.092	0.0	False
WMo3Se8	05a06afa3b20	Pm (6)	0.0	1.32	V	X→Γ	1.643	0.25	0.0	False
					V	Γ→S	1.001	0.195	0.232	False
BrSSb	4da5c6be60db	P3m1 (156)	0.028	1.233	V	M→K	2.39	0.117	0.28	False
Cr2W2Se8	548aa830244c	P1 (1)	0.015	0.778	V	X→Γ	1.39	0.204	0.0	False
					V	Y→S	1.322	0.128	0.497	False
WMo3S8	9c2979187585	P1 (1)	0.0	1.582	V	X→Γ	1.816	0.214	0.0	False

	Entry Info				Spin Splitting Info					
Formula	C2DB ID	SG index	ΔE_{hull}	Bandgap	Band	k-path	α_R	SS	ΔE_{SS}	AC
TiZr3Se8	a148361e5e9a	P1 (1)	0.144	0.701	V	S→Y	1.321	0.09	0.013	False
					V	Γ→S	0.88	0.086	0.041	False
BiClS	c96ef4fc869c	P3m1 (156)	0.0	1.334	C	Γ→M	1.146	0.115	0.0	True
					C	Γ→K	1.181	0.112	0.0	False
Ga2P2Te6	4cb4ea247ef4	P1 (1)	0.173	0.314	V	Y→Γ	3.695	0.163	0.001	False
					V	X→Γ	3.828	0.161	0.0	False
MoCr3Se8	961c37d6e527	Pm (6)	0.01	0.726	V	X→Γ	0.977	0.103	0.0	False
AsClSe	1a3be826b3e0	P3m1 (156)	0.013	1.364	V	Γ→M	3.717	0.097	0.039	False
					V	Γ→K	3.481	0.101	0.0	False

References

[1] Sten Haastrup et al. "The Computational 2D Materials Database: high-throughput modeling and discovery of atomically thin crystals". In: *2D Materials* 5 (4 Sept. 2018), p. 042002. ISSN: 2053-1583. DOI: 10.1088/2053-1583/AACFC1.
