# Supplementary material for: High-throughput inverse design and Bayesian optimization of functionalities: spin splitting in two-dimensional compounds
Source: Sci Data. 2022 Apr 29;9:195. doi: 10.1038/s41597-022-01292-8 (PMC9054849; doi:10.1038/s41597-022-01292-8)
Supplement: Supplementary file 3 [file 41597_2022_1292_MOESM3_ESM.pdf]

Supplementary Information - Dresselhaus SS Table

High-throughput inverse design and optimization of functionalities: spin splitting in two-dimensional compounds

Gabriel M. Nascimento[1,a], Elton Ogoshi[1,a], Adalberto Fazzio[1,2], Carlos Mera Acosta[1,*], and Gustavo M. Dalpian[1,*]

[a] *These authors contributed equally to this work.*
[1] *Center for Natural and Human Sciences, Federal University of ABC, Santo Andre, SP, Brazil*
[2] *Brazilian Nanotechnology National Laboratory (LNNano), CNPEM, 13083-970, Campinas, São Paulo, Brazil*
[*] *Corresponding authors: cmeraacosta@gmail.com; gustavo.dalpian@ufabc.edu.br*

Dresselhaus SS Materials

Table S.2. List of Dresselhaus SS prototypes identified in the valence (V) and/or conduction (C) bands for materials with non-polar structure. Each material is presented as a combination of chemical formula and its respective ID ending from the C2DB Database [1]. *SG index* represents the space group symbol (number) of the material's structure according to the precision criteria employed in this work for symmetry identification. ΔE_{hull} is the energy above the convex hull reported by the C2DB database. *Bandgap*, *k-path*, α_D, *SS*, ΔE_{SS} and *AC* stand for the energy band gap, k-path between high-symmetry k-points where the SS is identified, Dresselhaus coefficient (analogous to Rashba coefficient) [eV/Å^{-1}], spin-splitting magnitude, difference in energy between the maximum value of the SS and its respective band edge (VBM or CBM) and the presence of anti-crossing bands, respectively. All energy-related values are in eV.

Entry Info					Spin Splitting Info					
Formula	C2DB ID	SG index	ΔE_{hull}	Bandgap	Band	k-path	α_D	SS	ΔE_{SS}	AC
HfO2	512afaae525a	P-6m2 (187)	0.77	1.903	C	M→K	1.614	0.229	0.04	False
HgF2	f5965c8b3d89	P-4m2 (115)	0.162	1.995	V	Γ→M	0.85	0.161	0.0	False
					V	Γ→X	0.761	0.119	0.035	False
					V	X→Γ	0.324	0.115	0.035	False
					V	X→M	0.534	0.104	0.083	False
PbCl2	f9d58a299674	P-4m2 (115)	0.142	2.165	C	Γ→M	1.036	0.181	0.0	False
					C	Γ→X	1.031	0.178	0.001	False
					C	M→X	0.559	0.282	0.911	False
HfS2	3d4bfe131291	P-4m2 (115)	0.333	2.11	C	X→Γ	2.471	0.268	0.647	False
					C	M→X	1.102	0.12	0.028	True
ZrSe2	001dfe9a7fa2	P-4m2 (115)	0.319	1.452	C	X→Γ	1.998	0.101	0.521	False
O2Rh2	740bf2751050	P-6m2 (187)	0.246	0.057	V	M→K	4.914	0.11	0.0	True
PbTe2	dbf3271b4bb1	P-4m2 (115)	0.423	0.085	V	M→Γ	2.615	0.186	0.031	False
					V	Γ→M	2.556	0.171	0.031	True
					V	Γ→X	2.21	0.177	0.028	True
					C	X→M	0.846	0.104	0.101	False
PbI2	14411dde597c	P-4m2 (115)	0.145	1.531	V	M→Γ	0.951	0.244	0.0	False
					V	X→Γ	0.503	0.092	0.338	False
					V	M→X	0.931	0.229	0.037	False
					C	Γ→M	0.971	0.129	0.0	False
					C	Γ→X	0.943	0.126	0.001	False
					C	X→M	1.534	0.284	0.804	False
ZrS2	2e44a755e594	P-4m2 (115)	0.31	1.938	C	X→Γ	2.008	0.084	0.596	False
Ir2O2	06ebe3806790	P-6m2 (187)	0.51	0.099	V	M→K	4.665	0.265	0.012	True
					C	M→K	1.059	0.102	0.0	True

		Entry Info					Spin Splitting Info			
Formula	C2DB ID	SG index	ΔE_{hull}	Bandgap	Band	k-path	α_D	SS	ΔE_{SS}	AC
HgI2	7c2657e15a6f	P-4m2 (115)	0.0	1.512	C	M→Γ	0.311	0.145	0.823	False
OsBr2	bf30e1249164	P-4m2 (115)	0.677	0.092	V	X→Γ	2.633	0.121	0.0	True
					C	X→Γ	4.625	0.286	0.085	True
SnBr2	0155c4de2320	P-4m2 (115)	0.136	1.284	V	Γ→X	1.316	0.09	0.824	False
SrBr2	a4c9c803de7d	P-4m2 (115)	0.21	4.583	V	M→Γ	0.191	0.092	0.012	False
					V	M→X	0.244	0.092	0.0	False
GeI2	694ac91aec01	P-4m2 (115)	0.153	1.059	V	M→Γ	1.341	0.309	0.0	False
					V	X→Γ	0.637	0.139	0.532	False
					V	X→M	3.425	0.283	0.08	False
					C	X→M	2.307	0.16	1.134	False
HfO2	6e4ac7453419	P-4m2 (115)	0.51	4.494	C	X→Γ	0.703	0.113	0.546	False
Ir2S2	dd6289af8e01	P-6m2 (187)	0.305	0.135	C	M→K	2.202	0.164	0.0	True
PbF2	ccc95033446d	P-4m2 (115)	0.243	2.767	C	M→Γ	0.488	0.104	0.816	False
					C	X→Γ	2.465	0.082	0.0	False
OsCl2	d37ba63794ad	P-4m2 (115)	0.658	0.308	V	X→Γ	2.455	0.178	0.0	True
					C	Γ→M	1.181	0.462	0.0	False
					C	X→Γ	3.244	0.207	0.068	True
					C	X→M	0.836	0.312	0.036	False
PbBr2	cabd4ba0f21c	P-4m2 (115)	0.136	1.883	V	Γ→M	1.684	0.088	0.0	False
					V	Γ→X	0.927	0.082	0.482	False
					C	Γ→M	1.072	0.161	0.0	False
					C	Γ→X	1.03	0.161	0.001	False
					C	M→X	0.425	0.259	0.921	False
HfSe2	08401460f377	P-4m2 (115)	0.337	1.676	C	X→M	3.599	0.148	0.012	False
SnI2	7f0ca28e3229	P-4m2 (115)	0.157	1.143	V	M→Γ	0.844	0.251	0.0	False
					V	Γ→M	2.545	0.258	0.0	False
					V	Γ→X	1.421	0.138	0.498	False
					V	X→M	3.076	0.224	0.031	True
					V	M→X	0.834	0.214	0.031	False
					C	X→M	2.039	0.18	1.084	True
GeBr2	204ef2affa10	P-4m2 (115)	0.136	1.312	V	M→Γ	0.353	0.114	0.0	False
					V	X→Γ	0.499	0.096	0.705	False
					V	X→M	2.998	0.092	0.009	False
					C	X→M	2.139	0.085	1.471	False
CaBr2	49f279264c91	P-4m2 (115)	0.179	4.753	V	M→Γ	0.376	0.078	0.014	False
HfTe2	1e2c6946ca41	P-4m2 (115)	0.371	1.01	C	M→Γ	1.089	0.104	0.001	True
					C	M→X	1.458	0.159	0.0	True

References

[1] Sten Haastrup et al. “The Computational 2D Materials Database: high-throughput modeling and discovery of atomically thin crystals”. In: *2D Materials* 5 (4 Sept. 2018), p. 042002. ISSN: 2053-1583. DOI: 10.1088/2053-1583/AACFC1.
