# Supplementary material for: High-throughput inverse design and Bayesian optimization of functionalities: spin splitting in two-dimensional compounds
Source: Sci Data. 2022 Apr 29;9:195. doi: 10.1038/s41597-022-01292-8 (PMC9054849; doi:10.1038/s41597-022-01292-8)
Supplement: Supplementary file 4 [file 41597_2022_1292_MOESM4_ESM.pdf]

Supplementary Information - Zeeman SS Table

High-throughput inverse design and optimization of functionalities: spin splitting in two-dimensional compounds

Gabriel M. Nascimento[1,a], Elton Ogoshi[1,a], Adalberto Fazzio[1,2], Carlos Mera Acosta[1,*], and Gustavo M. Dalpian[1,*]

[a] *These authors contributed equally to this work.*
[1] *Center for Natural and Human Sciences, Federal University of ABC, Santo Andre, SP, Brazil*
[2] *Brazilian Nanotechnology National Laboratory (LNNano), CNPEM, 13083-970, Campinas, São Paulo, Brazil*
[*] *Corresponding authors: cmeraacosta@gmail.com; gustavo.dalpian@ufabc.edu.br*

Zeeman SS Materials

Table S.3. List of Zeeman SS prototypes identified in the valence (V) and/or conduction (C) bands for materials with all non-centrosymmetric structures. Each material is presented as a combination of chemical formula and its respective ID ending from the C2DB Database [1]. *SG index* represents the space group symbol (number) of the material's structure according to the precision criteria employed in this work for symmetry identification. ΔE_{hull} is the energy above the convex hull reported by the C2DB database. *Bandgap*, *k-point*, *SS* and ΔE_{SS} stand for the energy band gap, high-symmetry k-point where the SS is identified, spin-splitting magnitude and difference in energy between the maximum value of the SS and its respective band edge (VBM or CBM). All energy-related values are in eV.

	Entry Info				Spin Splitting Info			
Formula	C2DB ID	SG index	ΔE_{hull}	Bandgap	Band	k-path	SS	ΔE_{SS}
HgS	5256ed7d716e	P3m1 (156)	0.146	0.056	V	K	0.073	0.423
					C	K	0.047	3.733
HgO	a8678fa85c38	P-6m2 (187)	0.211	0.298	V	K	0.116	0.621
					C	K	0.013	3.96
CSiH2	8a1587098151	P3m1 (156)	0.0	4.008	C	K	0.002	1.268
ISbSe	df0019ec24b5	P3m1 (156)	0.0	1.061	V	K	0.149	0.414
					C	K	0.043	1.74
BrSbTe	f1e78a09001d	P3m1 (156)	0.13	1.331	V	K	0.078	0.586
					C	K	0.009	0.0
BrSbTe	18e62ba75259	P3m1 (156)	0.0	1.089	V	K	0.04	0.439
					C	K	0.177	1.38
ClSbSe	f705a30af945	P3m1 (156)	0.146	1.68	V	K	0.118	0.433
					C	K	0.115	0.037
BaCl2	54ec344f88a7	P-6m2 (187)	0.241	4.736	V	K	0.003	0.09
					C	K	0.018	0.08
PbS2	372c217dd52f	P-6m2 (187)	0.191	1.708	V	K	0.031	0.419
					C	K	0.73	0.636
SSeW	001e03f2c095	P3m1 (156)	0.01	1.417	V	K	0.445	0.0
					C	K	0.03	0.0
MgCl2	e73a5c5ae5ac	P-6m2 (187)	0.19	4.759	C	K	0.018	1.548
CaCl2	3ca106221b9b	P-6m2 (187)	0.164	4.784	C	K	0.012	0.402
ClITi	ae06e7424bb1	P3m1 (156)	0.115	0.746	V	K	0.063	0.0
					C	K	0.019	0.098
SnBr2	8d365ca62c55	P-6m2 (187)	0.086	2.514	V	K	0.212	0.042
					C	K	0.087	0.019
BiBrTe	f4f45fcade85	P3m1 (156)	0.117	0.916	V	K	0.065	0.718

Formula	C2DB ID	Entry Info<br>SG index	ΔE_{hull}	Bandgap	Band	Spin Splitting Info<br>k-path	SS	ΔE_{SS}
					C	K	0.496	0.314
CdBr2	a7bb757c6234	P-6m2 (187)	0.121	2.377	V	K	0.022	0.244
					C	K	0.155	1.122
Al2O2	bce0ccee4eca	P-6m2 (187)	0.237	1.324	V	K	0.003	0.0
					C	K	0.001	0.22
HfO2	512afaae525a	P-6m2 (187)	0.77	1.903	V	K	0.018	0.22
					C	K	0.234	0.04
MoSSe	de7ac5fc6945	P3m1 (156)	0.009	1.474	V	K	0.168	0.0
					C	K	0.013	0.0
ZrSe2	f17029facf63	P-6m2 (187)	0.144	0.734	V	K	0.011	0.659
					C	K	0.124	0.65
AsBrSe	206b9dcf2af6	P3m1 (156)	0.161	1.49	V	K	0.061	0.723
					C	K	0.037	0.0
Te2Zr2	8912432cb37b	P-6m2 (187)	0.624	0.458	V	K	0.019	0.765
					C	K	0.088	0.467
Al2Te2	e54041554385	P-6m2 (187)	0.0	1.763	V	K	0.087	1.134
					C	K	0.118	0.055
Ga2S2	ac002f4ce724	P-6m2 (187)	0.0	2.305	V	K	0.02	1.322
					C	K	0.02	0.453
STeW	75ee10091f43	P3m1 (156)	0.086	1.168	V	K	0.425	0.113
					C	K	0.03	0.0
MoSTe	2ea941c8bc3c	P3m1 (156)	0.223	0.196	V	K	0.092	0.0
					C	K	0.085	0.316
CdI2	66c5fba8ad87	P-6m2 (187)	0.156	1.527	V	K	0.07	0.559
					C	K	0.289	0.377
BrITi	233dbbf8f473	P3m1 (156)	0.057	0.68	V	K	0.07	0.0
					C	K	0.019	0.078
CrTe2	c31911a1b3f9	P-6m2 (187)	0.108	0.468	V	K	0.108	0.0
					C	K	0.02	0.0
Bi2O2	53ac438f321b	P-6m2 (187)	0.319	0.449	V	K	0.155	0.623
					C	K	1.314	0.0
TiI2	088e8488f895	P-6m2 (187)	0.052	0.602	V	K	0.08	0.0
					C	K	0.018	0.024
ISbTe	0f02957b17cf	P3m1 (156)	0.0	0.886	V	K	0.07	0.553
					C	K	0.155	1.49
AsISe	ca926a42865b	P3m1 (156)	0.174	0.519	V	K	0.132	1.19
					C	K	0.069	0.135
SeTeW	6e2a4c6f4f57	P3m1 (156)	0.042	1.058	V	K	0.46	0.0
					C	K	0.042	0.0
I2Tl2	c0f52097ab62	P1 (1)	0.076	2.657	V	H	0.003	0.002
					V	H1	0.003	0.002
					C	H	0.003	0.094
					C	H1	0.003	0.094
SeSn	d59c96fdfda1	P3m1 (156)	0.098	2.156	V	K	0.115	0.55
					C	K	0.079	0.143
GeO2	77905aa4e75f	P-6m2 (187)	0.639	1.392	V	K	0.002	0.0
					C	K	0.019	3.356
BrClTi	d3f135b9cf41	P3m1 (156)	0.015	0.826	V	K	0.045	0.0
					C	K	0.011	0.216
In2Te2	fcd97ff5abcd	P-6m2 (187)	0.0	1.249	V	K	0.005	1.203
					C	K	0.143	0.793
HfI2	05a69240794c	P-6m2 (187)	0.114	0.616	V	K	0.22	0.0
					C	K	0.105	0.671
CrS2	c5ee5e35d2b4	P-6m2 (187)	0.0	0.899	V	K	0.068	0.0
					C	K	0.004	0.0
BiBrS	49b7be14f786	P3m1 (156)	0.0	1.227	V	K	0.099	0.236
					C	K	0.078	1.825

		Entry Info				Spin Splitting Info		
Formula	C2DB ID	SG index	ΔE_{hull}	Bandgap	Band	k-path	SS	ΔE_{SS}
CrSeTe	5d9d3ded04de	P3m1 (156)	0.111	0.59	V	K	0.1	0.0
					C	K	0.019	0.0
AsClTe	fba4cc0df459	P3m1 (156)	0.194	1.316	V	K	0.065	0.71
					C	K	0.051	0.0
MoSeTe	42eb12e7b656	P3m1 (156)	0.025	1.159	V	K	0.2	0.0
					C	K	0.03	0.0
ClSbTe	04fdd7d1ec5c	P3m1 (156)	0.153	1.439	V	K	0.105	0.628
					C	K	0.053	0.0
SSn	f98da23471a1	P3m1 (156)	0.118	2.3	V	K	0.024	0.36
					C	K	0.141	0.115
C2O2Zr3	23672dbca7d0	P-6m2 (187)	0.211	0.381	V	K	0.009	1.323
					C	K	0.064	1.687
InN	8cf70870bc5b	P-6m2 (187)	0.482	0.606	V	K	0.016	0.0
					C	K	0.008	3.813
In2S2	172ef584c4a6	P-6m2 (187)	0.0	1.684	V	K	0.02	0.885
					C	K	0.075	1.023
BrSbSe	c2a344b393f0	P3m1 (156)	0.124	1.467	V	K	0.155	0.493
					C	K	0.071	0.078
Ag2F2	44f6ed525a5a	P1 (1)	0.048	0.52	V	H	0.047	0.236
					V	H1	0.047	0.236
					C	H	0.011	3.755
					C	H1	0.011	3.755
S2V2	605c732d5111	P-6m2 (187)	0.534	0.209	V	K	0.002	0.641
					C	K	0.047	0.052
BiBrSe	de5756e4fbfa	P3m1 (156)	0.0	1.03	V	K	0.012	0.39
					C	K	0.225	1.749
Cl2Cu2	c1a86f114149	P1 (1)	0.018	1.218	C	H	0.002	3.154
					C	H1	0.002	3.154
Bi2P2S6	287dcf4f1a19	P1 (1)	0.053	0.953	V	H	0.041	0.257
					V	H1	0.041	0.257
					C	H	0.123	0.188
					C	H1	0.123	0.188
BiClS	99fd027b1d0b	P3m1 (156)	0.12	1.841	V	K	0.062	0.298
					C	K	0.661	0.276
AsIn	c77a730c90f8	P3m1 (156)	0.4	0.681	V	K	0.031	0.108
					C	K	0.046	0.997
In2P2S6	793870f62166	P1 (1)	0.053	0.852	V	H	0.007	0.429
					V	H1	0.007	0.429
					C	H	0.003	0.372
					C	H1	0.003	0.372
ISbTe	052a3116531d	P3m1 (156)	0.123	1.031	V	K	0.051	0.682
					C	K	0.033	0.0
Se2V2	9cf30bd127fe	P-6m2 (187)	0.42	0.248	V	K	0.01	0.5
					C	K	0.045	0.07
AsGa	728f322893fe	P3m1 (156)	0.413	1.069	V	K	0.014	0.0
					C	K	0.035	0.691
AsBrS	1dcd471c2288	P3m1 (156)	0.034	1.38	V	K	0.127	0.51
					C	K	0.114	1.84
STeW	916afba26723	P3m1 (156)	0.266	0.191	V	K	0.029	0.0
					C	K	0.234	0.306
BiClTe	968a6902b7f5	P3m1 (156)	0.0	0.938	V	K	0.179	0.599
					C	K	0.722	1.354
AsBrTe	671e6de2497a	P3m1 (156)	0.163	1.098	V	K	0.133	0.673
					C	K	0.093	0.0
PbCl2	b0b142073783	P-6m2 (187)	0.105	3.136	V	K	0.067	0.0
					C	K	0.752	0.0
AsISe	5d829e480507	P3m1 (156)	0.0	1.164	V	K	0.138	0.614

	Entry Info				Spin Splitting Info			
Formula	C2DB ID	SG index	ΔE_{hull}	Bandgap	Band	k-path	SS	ΔE_{SS}
					C	K	0.07	1.81
WS2	64090c9845f8	P-6m2 (187)	0.0	1.551	V	K	0.43	0.0
					C	K	0.03	0.0
O2Sc2	b757b8efeeab	P-6m2 (187)	0.249	0.694	V	K	0.01	0.173
					C	K	0.009	0.269
PbI2	9e6494406d07	P-6m2 (187)	0.078	2.029	V	K	0.002	0.218
					C	K	0.479	0.118
SnO2	d5f47e5d4cf7	P-6m2 (187)	0.645	0.623	V	K	0.004	0.0
					C	K	0.018	4.057
TiS2	65d41aaec667	P-6m2 (187)	0.145	0.721	V	K	0.005	0.515
					C	K	0.04	0.576
S2Tl2	751e767bff79	P-6m2 (187)	0.119	0.666	V	K	0.012	1.013
					C	K	0.04	1.302
O2Rh2	740bf2751050	P-6m2 (187)	0.246	0.057	V	K	0.057	0.229
					C	K	0.014	1.062
BrIZr	28c61999c692	P3m1 (156)	0.038	0.782	V	K	0.1	0.0
					C	K	0.022	0.724
ClSbSe	0c0fbdaf8f4a	P3m1 (156)	0.014	1.177	V	K	0.065	0.311
					C	K	0.261	1.795
HgI2	0ff7ee261fec	P-6m2 (187)	0.183	0.666	V	K	0.159	0.473
					C	K	0.333	0.31
ClSbTe	da5fd2bb47af	P3m1 (156)	0.008	1.291	V	K	0.148	0.324
					C	K	0.208	1.333
AsB	b6e76caa350b	P-6m2 (187)	0.467	0.752	V	K	0.002	0.0
					C	K	0.003	0.0
ZrI2	9c024b5a2e89	P-6m2 (187)	0.027	0.698	V	K	0.122	0.0
					C	K	0.026	0.586
P2Sn2Se6	a056ab5346bf	P1 (1)	0.015	0.722	V	H	0.002	0.779
					V	H1	0.002	0.779
STeZr	3f3c7bc0ce7d	P3m1 (156)	0.122	0.218	V	K	0.001	1.067
					C	K	0.133	0.386
Se2Zr2	f89b20d72c95	P-6m2 (187)	0.436	0.06	V	K	0.046	1.201
					C	K	0.039	0.51
In2Se2	eb204c739879	P-6m2 (187)	0.0	1.399	V	K	0.092	1.06
					C	K	0.021	1.148
Se2Zn2	90835c470691	Cm (8)	0.245	1.61	V	H	0.003	0.089
					V	H1	0.003	0.089
BrClZr	8cb69386d06b	P3m1 (156)	0.01	0.912	V	K	0.059	0.0
					C	K	0.008	0.987
S2Sc2	e9d256b367c7	P-6m2 (187)	0.627	0.384	V	K	0.004	0.141
					C	K	0.008	0.326
HfSSe	9afb20358166	P3m1 (156)	0.193	0.91	V	K	0.035	0.541
					C	K	0.292	0.712
P2Ru2S6	9caed1a0620c	P1 (1)	0.179	0.322	V	H	0.047	0.129
					V	H1	0.047	0.129
BiITe	2d41b3dd1772	P3m1 (156)	0.0	0.701	V	K	0.01	0.567
					C	K	0.307	1.569
Rh2Cl6	06f695e97385	P321 (150)	0.464	0.208	C	K	0.002	0.0
BaBr2	1a59eff92917	P-6m2 (187)	0.167	4.149	V	K	0.02	0.11
					C	K	0.042	0.148
Ir2O2	06ebe3806790	P-6m2 (187)	0.51	0.099	V	K	0.302	0.36
					C	K	0.207	0.639
BiBrSe	11db0908d9ef	P3m1 (156)	0.111	1.385	V	K	0.057	0.403
					C	K	0.557	0.396
Te2V2	5da53e6996e3	P-6m2 (187)	0.49	0.22	V	K	0.013	0.434
					C	K	0.068	0.038
P2Sb2Te6	82b85dfd7723	P1 (1)	0.14	0.633	V	H	0.003	0.673

Entry Info					Spin Splitting Info			
Formula	C2DB ID	SG index	ΔE_{hull}	Bandgap	Band	k-path	SS	ΔE_{SS}
					V	H1	0.003	0.673
					C	H	0.001	0.073
					C	H1	0.001	0.073
PbBr2	cbdc15b42a05	P-6m2 (187)	0.082	2.662	V	K	0.136	0.022
					C	K	0.631	0.119
Al2P2S6	669d6f1af4d4	P1 (1)	0.083	1.301	C	H	0.002	0.021
					C	H1	0.002	0.021
ISbSe	343d2125478e	P3m1 (156)	0.13	1.078	V	K	0.034	0.711
					C	K	0.028	0.171
Bi2P2Te6	cf7927ab6730	P1 (1)	0.14	0.507	V	H	0.001	0.553
					V	H1	0.001	0.553
					C	H	0.034	0.212
					C	H1	0.034	0.212
MoTe2	38a53176109a	P-6m2 (187)	0.0	0.956	V	K	0.215	0.0
					C	K	0.034	0.0
HgTe	1a3bdd1b142a	P3m1 (156)	0.165	0.132	V	K	0.095	0.446
					C	K	0.236	2.168
CdCl2	46c028e03e8b	P-6m2 (187)	0.13	3.111	V	K	0.014	0.0
					C	K	0.039	1.631
BiBrTe	304bc6a92d82	P3m1 (156)	0.0	0.878	V	K	0.074	0.524
					C	K	0.597	1.437
SSeTi	358305cad463	P3m1 (156)	0.128	0.501	V	K	0.003	0.687
					C	K	0.055	0.515
ZrO2	24a8929c68ce	P-6m2 (187)	0.766	1.683	V	K	0.006	0.258
					C	K	0.065	0.026
AsIS	e23390b66883	P3m1 (156)	0.256	0.295	V	K	0.09	1.371
					C	K	0.009	0.393
MoO2	152bd69757aa	P-6m2 (187)	0.028	0.918	V	K	0.135	1.541
					C	K	0.004	0.0
BiClSe	7fe9c5cb910c	P3m1 (156)	0.119	1.601	V	K	0.114	0.411
					C	K	0.609	0.318
SrI2	6cfaae647808	P-6m2 (187)	0.096	3.448	V	K	0.022	0.182
					C	K	0.079	0.498
ZnBr2	553cb6a56984	P-6m2 (187)	0.249	2.413	V	K	0.016	0.402
					C	K	0.156	0.7
AsIS	b13beafa16aa	P3m1 (156)	0.064	1.395	V	K	0.2	0.529
					C	K	0.107	1.721
AsITe	114b3382699c	P3m1 (156)	0.162	0.416	V	K	0.189	1.022
					C	K	0.124	0.0
AsBiCr	b299416bff28	P3m1 (156)	0.461	0.037	V	K	0.041	1.339
					C	K	0.192	0.238
Ga2Se2	394e5709a3ac	P-6m2 (187)	0.0	1.736	V	K	0.011	1.375
					C	K	0.036	0.847
Sc2Te2	c3cac8e74dc1	P-6m2 (187)	0.614	0.287	V	K	0.002	0.051
					C	K	0.006	0.112
BaI2	c4707a226b8f	P-6m2 (187)	0.105	3.362	V	K	0.043	0.155
					C	K	0.075	0.267
BiIS	acdcd16c0d76	P3m1 (156)	0.014	1.139	V	K	0.164	0.26
					C	K	0.236	1.612
HgCl2	6cbe2e585099	P-6m2 (187)	0.131	2.023	V	K	0.073	0.0
					C	K	0.048	1.125
GaN	c973e283b023	P-6m2 (187)	0.416	1.818	V	K	0.006	0.0
					C	K	0.001	3.052
Te2Tl2	73117163f0e2	P-6m2 (187)	0.137	0.367	V	K	0.149	1.151
					C	K	0.368	0.566
N2O2Hf3	bb4e40ae9164	P-6m2 (187)	0.088	0.323	V	K	0.1	0.0
					C	K	0.115	1.31

	Entry Info				Spin Splitting Info			
Formula	C2DB ID	SG index	ΔE_{hull}	Bandgap	Band	k-path	SS	ΔE_{SS}
ISSb	4c49d27e66e5	P3m1 (156)	0.185	0.872	V	K	0.057	0.853
					C	K	0.085	0.327
Br2Tl2	948c61cd5626	P1 (1)	0.063	3.311	V	H	0.002	0.034
					V	H1	0.002	0.034
					C	H	0.002	0.006
					C	H1	0.002	0.006
Rh2Br6	c284d6de2b3e	P3 (143)	0.408	0.26	C	K	0.004	0.002
SnS2	8f2fa65321f0	P-6m2 (187)	0.286	0.754	V	K	0.005	0.186
					C	K	0.042	0.818
ClSSb	9188c300265c	P3m1 (156)	0.048	1.332	V	K	0.013	0.236
					C	K	0.313	1.878
SnTe	e688959ea45b	P3m1 (156)	0.119	1.592	V	K	0.228	1.08
					C	K	0.003	0.18
HfCl2	864f8b497185	P-6m2 (187)	0.007	0.891	V	K	0.099	0.0
					C	K	0.236	1.274
Cl2Pt2	93dfef2d1004	P1 (1)	0.0	1.329	V	H	0.002	0.393
					V	H1	0.002	0.393
MoS2	b3b4685fb6e1	P-6m2 (187)	0.0	1.603	V	K	0.148	0.0
					C	K	0.003	0.0
SrCl2	77398c835c11	P-6m2 (187)	0.194	4.958	V	K	0.001	0.051
					C	K	0.009	0.312
ClIZr	73202b4b7837	P3m1 (156)	0.078	0.883	V	K	0.087	0.0
					C	K	0.021	0.716
GeTe	eadd37f03ca5	P3m1 (156)	0.087	1.488	V	K	0.188	1.475
					C	K	0.079	0.283
CaBr2	fbb623b6f288	P-6m2 (187)	0.129	4.141	V	K	0.003	0.05
					C	K	0.04	0.485
BrHfI	836a1091409d	P3m1 (156)	0.087	0.695	V	K	0.182	0.0
					C	K	0.193	0.856
CrSSe	09e1e5ef94cb	P3m1 (156)	0.01	0.802	V	K	0.082	0.0
					C	K	0.01	0.0
ZrTe2	f7ad606317e6	P-6m2 (187)	0.11	0.275	V	K	0.01	0.802
					C	K	0.163	0.741
Ir2Cl6	be7870547213	P321 (150)	0.555	0.263	C	K	0.003	0.0
SrBr2	2876a0cb2478	P-6m2 (187)	0.14	4.324	V	K	0.008	0.067
					C	K	0.038	0.415
CrSe2	9a6ff6a3c41a	P-6m2 (187)	0.0	0.703	V	K	0.09	0.0
					C	K	0.015	0.0
SnCl2	514a8a12dca9	P-6m2 (187)	0.117	2.76	V	K	0.065	0.0
					C	K	0.187	0.039
OSn	026ebfd86b48	P3m1 (156)	0.329	1.682	V	K	0.009	0.0
					C	K	0.137	0.825
HfS2	2c5e65012601	P-6m2 (187)	0.217	1.082	V	K	0.033	0.471
					C	K	0.272	0.712
AsClS	0fd6ab210774	P3m1 (156)	0.224	1.732	V	K	0.055	0.648
					C	K	0.063	0.088
Ir2S2	dd6289af8e01	P-6m2 (187)	0.305	0.135	V	K	0.241	0.183
					C	K	0.221	0.859
ZrCl2	dc09b7c396eb	P-6m2 (187)	0.0	0.988	V	K	0.041	0.0
					C	K	0.001	1.06
BiIS	40034665f9f1	P3m1 (156)	0.14	0.848	V	K	0.097	0.799
					C	K	0.553	0.435
GaP	d467820f3f04	P3m1 (156)	0.447	1.555	V	K	0.007	0.0
					C	K	0.005	0.563
Al2S2	f9df9f4a5c34	P-6m2 (187)	0.005	2.099	V	K	0.012	0.654
					C	K	0.01	0.069
HfSSe	63618e5bf062	P3m1 (156)	0.0	0.705	V	K	0.024	2.114

Formula	C2DB ID	Entry Info SG index	ΔE_{hull}	Bandgap	Band	Spin Splitting Info k-path	SS	ΔE_{SS}
					C	K	0.002	1.466
BiISe	433f707c632c	P3m1 (156)	0.114	0.84	V	K	0.118	0.642
					C	K	0.495	0.459
AlAs	814ae25a188e	P-6m2 (187)	0.522	1.241	V	K	0.002	0.0
					C	K	0.002	1.092
ZrBr2	7897c7cc2491	P-6m2 (187)	0.0	0.827	V	K	0.076	0.0
					C	K	0.012	0.948
AsBrSe	989f469f06bd	P3m1 (156)	0.0	1.212	V	K	0.061	0.576
					C	K	0.069	1.752
ZrS2	1a039e022308	P-6m2 (187)	0.19	0.97	V	K	0.01	0.572
					C	K	0.087	0.567
HgSe	619ed885f677	P3m1 (156)	0.157	0.069	V	K	0.061	0.375
					C	K	0.136	2.954
BiITe	a84d988e38ac	P3m1 (156)	0.11	0.691	V	K	0.163	0.588
					C	K	0.43	0.387
AsBrTe	64921449e408	P3m1 (156)	0.0	1.253	V	K	0.005	0.611
					C	K	0.028	1.261
SSeZr	1a9901838600	P3m1 (156)	0.0	0.616	V	K	0.039	1.958
					C	K	0.007	1.241
P2Ta2Te6	601435d29c97	P1 (1)	0.222	0.206	C	H	0.001	0.107
					C	H1	0.001	0.107
GeCl2	a7216f084785	P-6m2 (187)	0.136	2.955	V	K	0.067	0.112
					C	K	0.057	0.0
TiBr2	57116f9a9a4e	P-6m2 (187)	0.0	0.756	V	K	0.056	0.0
					C	K	0.015	0.172
WO2	94cfbb3f9284	P-6m2 (187)	0.0	1.312	V	K	0.406	1.385
					C	K	0.02	0.0
Hg2P2S6	b81eb586acfd	P1 (1)	0.0	0.981	C	H	0.001	0.731
					C	H1	0.001	0.731
GeBr2	36a198743d35	P-6m2 (187)	0.112	2.543	V	K	0.175	0.251
					C	K	0.042	0.0
SSeZr	2be14f373da0	P3m1 (156)	0.163	0.831	V	K	0.008	0.623
					C	K	0.107	0.569
MoSe2	f61b14d398c7	P-6m2 (187)	0.0	1.342	V	K	0.185	0.0
					C	K	0.021	0.0
BrSbSe	89b15ddef41d	P3m1 (156)	0.0	1.072	V	K	0.044	0.383
					C	K	0.231	1.758
WSe2	1cfbe6183886	P-6m2 (187)	0.0	1.255	V	K	0.466	0.0
					C	K	0.037	0.0
PbSe	a0dbdc6630fa	P3m1 (156)	0.217	1.68	V	K	0.158	0.845
					C	K	0.431	0.647
CaI2	066f40f26c53	P-6m2 (187)	0.108	2.995	V	K	0.011	0.412
					C	K	0.079	0.582
Bi2P2Se6	aa9a981d89aa	P1 (1)	0.054	0.875	C	H	0.149	0.09
					C	H1	0.149	0.09
HfSeTe	305c779b8752	P3m1 (156)	0.149	0.16	V	K	0.035	1.04
					C	K	0.321	0.727
HfTe2	59c0e014651d	P-6m2 (187)	0.133	0.147	V	K	0.04	0.902
					C	K	0.343	0.848
AlSb	1734deee2ac1	P3m1 (156)	0.475	1.447	V	K	0.02	0.0
					C	K	0.046	0.017
BiISe	70cbc0e44d36	P3m1 (156)	0.0	0.929	V	K	0.087	0.383
					C	K	0.08	1.731
GeO	a42f736f1682	P3m1 (156)	0.311	2.093	V	K	0.008	0.0
					C	K	0.059	0.757
BrSSb	4ae37f15e1fe	P3m1 (156)	0.157	1.437	V	K	0.139	0.444
					C	K	0.129	0.21

	Entry Info				Spin Splitting Info			
Formula	C2DB ID	SG index	ΔE_{hull}	Bandgap	Band	k-path	SS	ΔE_{SS}
TiCl2	95688ba68ca1	P-6m2 (187)	0.001	0.901	V	K	0.032	0.0
					C	K	0.004	0.281
Rh2Se2	1a46a7cf8fab	P-6m2 (187)	0.164	0.063	V	K	0.136	0.638
					C	K	0.024	0.356
AsClSe	df329350eef2	P3m1 (156)	0.179	1.71	V	K	0.05	0.702
					C	K	0.004	0.0
GeSe	211bcb7f05d6	P3m1 (156)	0.04	2.215	V	K	0.109	0.914
					C	K	0.009	0.272
ClHfI	d3756ea15451	P3m1 (156)	0.14	0.806	V	K	0.158	0.0
					C	K	0.204	0.889
TiSe2	509ef368050d	P-6m2 (187)	0.117	0.515	V	K	0.004	0.602
					C	K	0.064	0.581
Sc2Se2	9fb15588e4d4	P-6m2 (187)	0.548	0.373	V	K	0.004	0.092
					C	K	0.007	0.185
In2O2	d14171d2ba1a	P-6m2 (187)	0.208	0.371	V	K	0.008	0.0
					C	K	0.073	2.911
Ag2Cl2	dd5f0964d63d	P1 (1)	0.003	1.597	C	H	0.002	3.303
					C	H1	0.002	3.303
C2O2Hf3	082ae1b027e9	P-6m2 (187)	0.216	0.419	V	K	0.007	1.227
					C	K	0.214	1.853
HfBr2	84e9162c0c53	P-6m2 (187)	0.004	0.722	V	K	0.149	0.0
					C	K	0.191	1.089
BrClHf	72257f9ad66d	P3m1 (156)	0.016	0.819	V	K	0.126	0.0
					C	K	0.222	1.164
AsClS	afd0d75a82a2	P3m1 (156)	0.056	1.532	V	K	0.018	0.466
					C	K	0.126	1.781
TiO2	1cfb690281c9	P-6m2 (187)	0.587	1.136	V	K	0.003	0.416
					C	K	0.035	0.141
OPb	2a393480e273	P3m1 (156)	0.315	1.806	V	K	0.018	0.106
					C	K	0.442	1.675
InSb	466fcf7fad66	P3m1 (156)	0.379	0.477	V	K	0.054	0.233
					C	K	0.098	0.598
AsClTe	4fd8ad708fb0	P3m1 (156)	0.018	1.496	V	K	0.112	0.475
					C	K	0.038	1.159
Se2Tl2	625697b299d1	P-6m2 (187)	0.076	0.488	V	K	0.051	1.134
					C	K	0.178	1.062
CrSTe	8a0864d30ce1	P3m1 (156)	0.161	0.288	V	K	0.095	0.4
					C	K	0.018	0.0
ISSb	5b94060698bc	P3m1 (156)	0.041	1.276	V	K	0.216	0.314
					C	K	0.193	1.644
Ir2Se2	53337987551a	P-6m2 (187)	0.426	0.287	V	K	0.367	0.202
					C	K	0.248	0.499
PbSe2	0bc5d11454a7	P-6m2 (187)	0.18	1.324	V	K	0.135	0.386
					C	K	0.192	0.721
BiBrS	3b305c3e2c18	P3m1 (156)	0.116	1.594	V	K	0.086	0.335
					C	K	0.61	0.357
PdSe2	0ae696751911	P-6m2 (187)	0.268	0.231	V	K	0.345	1.632
					C	K	0.026	0.106
CSiF2	ee1174d1d821	P3m1 (156)	0.6	1.91	V	K	0.01	2.415
					C	K	0.003	4.272
Te2Ti2	b43c14735d8e	P-6m2 (187)	0.61	0.232	V	K	0.012	0.704
					C	K	0.059	0.196
N2O2Zr3	c317fbd68215	P-6m2 (187)	0.094	0.404	V	K	0.031	0.013
					C	K	0.052	1.018
ZnI2	701e1fc14b22	P-6m2 (187)	0.302	0.931	V	K	0.072	0.718
					C	K	0.283	0.477
PbS	5e4ff1f56b4a	P3m1 (156)	0.231	1.979	V	K	0.019	0.699

		Entry Info				Spin Splitting Info		
Formula	C2DB ID	SG index	ΔE_{hull}	Bandgap	Band	k-path	SS	ΔE_{SS}
					C	K	0.489	0.588
InP	c5672c6c1c78	P3m1 (156)	0.432	1.072	V	K	0.02	0.0
					C	K	0.011	1.071
SeTeZr	dd69b684c867	P3m1 (156)	0.115	0.275	V	K	0.005	0.945
					C	K	0.146	0.595
PbTe	3bc08d486d65	P3m1 (156)	0.198	1.151	V	K	0.345	1.28
					C	K	0.357	0.573
SnI2	d9c422656482	P-6m2 (187)	0.085	1.965	V	K	0.182	0.251
					C	K	0.025	0.0
P2Sb2Se6	5d1a32a28ffa	P1 (1)	0.058	1.004	V	H	0.002	0.671
					V	H1	0.002	0.671
					C	H	0.057	0.121
					C	H1	0.057	0.121
AsBrS	d9f4d4011670	P3m1 (156)	0.201	1.425	V	K	0.011	0.769
					C	K	0.022	0.208
BiClTe	badda86cab42	P3m1 (156)	0.129	0.948	V	K	0.043	0.929
					C	K	0.553	0.239
MoSTe	e4bb8738150a	P3m1 (156)	0.065	1.027	V	K	0.186	0.27
					C	K	0.026	0.0
CrO2	2433700165bb	P-6m2 (187)	0.168	0.422	V	K	0.064	1.123
					C	K	0.002	0.0
MgI2	67bb6819958f	P-6m2 (187)	0.16	2.601	V	K	0.024	0.683
					C	K	0.208	0.497
HgBr2	9965e7e32aa2	P-6m2 (187)	0.12	1.521	V	K	0.076	0.18
					C	K	0.189	0.69
Hg2I2	f7e70d2b90ad	P1 (1)	0.0	1.265	V	H	0.001	0.65
					V	H1	0.001	0.65
HfSe2	d2d9fee03594	P-6m2 (187)	0.174	0.827	V	K	0.037	0.564
					C	K	0.311	0.778
Ga2O2	16c96094d1a0	P-6m2 (187)	0.06	1.48	V	K	0.007	0.063
					C	K	0.025	2.204
WCr3Te8	6523c349753c	P1 (1)	0.097	0.459	C	Y	0.001	0.222
ClSSb	0495f35048b5	P3m1 (156)	0.179	1.675	V	K	0.058	0.354
					C	K	0.174	0.147
BiClSe	a80866a2c6b4	P3m1 (156)	0.0	1.139	V	K	0.089	0.362
					C	K	0.355	1.732
O2W2	42fa50003592	P-6m2 (187)	0.503	0.04	V	K	0.237	0.428
					C	K	0.063	0.807
ZnCl2	62c6ee7a0a25	P-6m2 (187)	0.236	3.437	V	K	0.008	0.126
					C	K	0.039	1.109
BrSSb	4da5c6be60db	P3m1 (156)	0.028	1.233	V	K	0.117	0.28
					C	K	0.244	1.825
BSb	71730c0eaab1	P-6m2 (187)	0.806	0.301	V	K	0.013	0.0
					C	K	0.01	0.0
WTe2	3c87365bc48c	P-6m2 (187)	0.026	0.754	V	K	0.485	0.0
					C	K	0.052	0.0
Al2Se2	129a514b51ad	P-6m2 (187)	0.0	1.997	V	K	0.051	0.957
					C	K	0.056	0.063
GeS	227b12019ade	P3m1 (156)	0.053	2.467	V	K	0.024	0.692
					C	K	0.051	0.172
BiClS	c96ef4fc869c	P3m1 (156)	0.0	1.334	V	K	0.006	0.254
					C	K	0.206	1.839
Ga2P2Te6	4cb4ea247ef4	P1 (1)	0.173	0.314	V	H	0.143	0.629
					V	H1	0.143	0.629
					C	H	0.103	0.0
					C	H1	0.103	0.0
MgBr2	bee1987fb4e6	P-6m2 (187)	0.169	3.569	V	K	0.003	0.37

		Entry Info				Spin Splitting Info		
Formula	C2DB ID	SG index	ΔE_{hull}	Bandgap	Band	k-path	SS	ΔE_{SS}
AsITe	b6d803aafe3a	P3m1 (156)	0.0	1.009	C	K	0.08	1.554
					V	K	0.074	0.715
AsClSe	1a3be826b3e0	P3m1 (156)	0.013	1.364	C	K	0.022	1.44
					V	K	0.051	0.508
Ga2Te2	55c23ca88a05	P-6m2 (187)	0.002	1.289	C	K	0.08	1.706
					V	K	0.02	1.202

References

[1] Sten Haastrup et al. “The Computational 2D Materials Database: high-throughput modeling and discovery of atomically thin crystals”. In: *2D Materials* 5 (4 Sept. 2018), p. 042002. ISSN: 2053-1583. DOI: 10.1088/2053-1583/AACFC1.
