# Supplementary material for: High-throughput inverse design and Bayesian optimization of functionalities: spin splitting in two-dimensional compounds
Source: Sci Data. 2022 Apr 29;9:195. doi: 10.1038/s41597-022-01292-8 (PMC9054849; doi:10.1038/s41597-022-01292-8)
Supplement: Supplementary file 5 [file 41597_2022_1292_MOESM5_ESM.pdf]

Supplementary Information - High-order SS Table

High-throughput inverse design and optimization of functionalities: spin splitting in two-dimensional compounds

Gabriel M. Nascimento[1,a], Elton Ogoshi[1,a], Adalberto Fazzio[1,2], Carlos Mera Acosta[1,*], and Gustavo M. Dalpian[1,*]

[a] *These authors contributed equally to this work.*
[1] *Center for Natural and Human Sciences, Federal University of ABC, Santo Andre, SP, Brazil*
[2] *Brazilian Nanotechnology National Laboratory (LNNano), CNPEM, 13083-970, Campinas, São Paulo, Brazil*
[*] *Corresponding authors: cmeraacosta@gmail.com; gustavo.dalpian@ufabc.edu.br*

High-Order SS Materials

Table S.4. List of High-Order SS prototypes identified in the valence (V) and/or conduction (C) bands for materials with all non-centrosymmetric structures. Each material is presented as a combination of chemical formula and its respective ID ending from the C2DB Database [1]. *SG index* represents the space group symbol (number) of the material's structure according to the precision criteria employed in this work for symmetry identification. ΔE_{hull} is the energy above convex hull reported by the C2DB database. *Bandgap*, *k-path*, *SS*, ΔE_{SS} and *AC* stand for the energy band gap, k-path between high-symmetry k-points where the SS is identified, spin-splitting magnitude, difference in energy between the maximum value of the SS and its respective band edge (VBM or CBM) and the presence of anti-crossing bands, respectively. All energy-related values are in eV.

Entry Info					Spin Splitting Info				
Formula	C2DB ID	SG index	ΔE_{hull}	Bandgap	Band	k-point	SS	ΔE_{SS}	AC
HgS	5256ed7d716e	P3m1 (156)	0.146	0.056	V	Γ→M	0.017	0.006	False
					V	Γ→K	0.035	0.0	False
ISbSe	df0019ec24b5	P3m1 (156)	0.0	1.061	V	Γ→M	0.002	0.493	True
					V	Γ→K	0.003	0.492	True
BrSbTe	18e62ba75259	P3m1 (156)	0.0	1.089	C	Γ→K	0.167	1.381	False
ClSbSe	f705a30af945	P3m1 (156)	0.146	1.68	V	Γ→M	0.073	0.104	False
					V	Γ→K	0.107	0.0	False
SSeW	001e03f2c095	P3m1 (156)	0.01	1.417	V	M→Γ	0.019	1.265	False
TiSe2	0684166af1fd	P-4m2 (115)	0.261	0.864	V	Γ→X	0.01	0.909	False
					C	M→Γ	0.015	0.0	False
					C	M→X	0.02	0.057	False
SnBr2	8d365ca62c55	P-6m2 (187)	0.086	2.514	C	M→K	0.069	0.08	False
BiBrTe	f4f45fcade85	P3m1 (156)	0.117	0.916	V	Γ→M	0.013	0.782	False
TiO2	badf6957f0bb	P-4m2 (115)	0.213	3.422	V	M→Γ	0.014	0.098	False
					V	X→M	0.015	0.023	False
					V	M→X	0.015	0.023	False
					C	X→Γ	0.014	1.037	False
GeSe2	8af45b2cf14e	P-4m2 (115)	0.051	0.556	V	Γ→X	0.029	0.349	False
					V	X→M	0.006	0.883	False
					C	Γ→M	0.175	1.404	False
					C	Γ→X	0.039	0.328	False
ZrSe2	f17029facf63	P-6m2 (187)	0.144	0.734	V	Γ→K	0.097	0.0	False
SrF2	c99805c05244	P-4m2 (115)	0.376	6.025	C	X→M	0.001	6.025	False
AsBrSe	206b9dcf2af6	P3m1 (156)	0.161	1.49	C	M→K	0.058	0.433	False
Al2Te2	e54041554385	P-6m2 (187)	0.0	1.763	C	M→K	0.097	0.388	False

	Entry Info				Spin Splitting Info				
Formula	C2DB ID	SG index	ΔE_{hull}	Bandgap	Band	k-point	SS	ΔE_{SS}	AC
Ga2S2	ac002f4ce724	P-6m2 (187)	0.0	2.305	C	M→K	0.012	0.656	False
STeW	75ee10091f43	P3m1 (156)	0.086	1.168	V	M→Γ	0.046	0.951	False
MoSTe	2ea941c8bc3c	P3m1 (156)	0.223	0.196	C	M→Γ	0.011	0.252	False
HgF2	f5965c8b3d89	P-4m2 (115)	0.162	1.995	V	M→Γ	0.161	0.0	False
					C	X→Γ	0.002	0.0	False
CdBr2	bb3c9722fb14	P-4m2 (115)	0.02	2.939	V	X→M	0.01	0.0	False
					C	X→M	0.004	0.0	False
SnO2	96a036411ab6	P-4m2 (115)	0.434	2.065	V	M→Γ	0.01	0.101	False
					V	Γ→M	0.01	0.118	False
GeO2	21281ac194c2	P-4m2 (115)	0.152	2.944	V	M→Γ	0.014	0.166	False
					C	X→M	0.003	4.194	False
Sn2Te2	03bcf7dcdaf2	$Pmn2_1(31)$	0.063	0.595	C	S→Y	0.006	0.153	False
					C	Y→S	0.006	0.153	False
					C	S→Γ	0.012	0.412	False
AsISe	ca926a42865b	P3m1 (156)	0.174	0.519	C	M→K	0.07	0.547	False
SeTeW	6e2a4c6f4f57	P3m1 (156)	0.042	1.058	V	M→Γ	0.033	1.041	False
Pb2S2	d4ed2cd9ee0c	$Pmn2_1(31)$	0.054	1.341	V	Y→Γ	0.003	0.001	False
					C	S→Y	0.014	0.211	False
					C	Y→S	0.014	0.211	False
					C	Y→Γ	0.012	0.0	False
					C	S→Γ	0.005	0.649	False
ZrO2	da3987f48688	P-4m2 (115)	0.464	4.378	V	X→Γ	0.004	0.683	False
I2Tl2	c0f52097ab62	P1 (1)	0.076	2.657	C	C→H1	0.003	0.094	False
					C	X→H1	0.003	0.094	False
SeSn	d59c96fdfda1	P3m1 (156)	0.098	2.156	V	M→Γ	0.053	0.025	False
					C	Γ→M	0.143	0.781	False
					C	Γ→K	0.232	0.923	False
CdF2	14736784891b	P-4m2 (115)	0.208	3.802	C	X→M	0.001	0.0	False
BrClTi	d3f135b9cf41	P3m1 (156)	0.015	0.826	C	M→Γ	0.01	0.047	False
CrS2	c5ee5e35d2b4	P-6m2 (187)	0.0	0.899	C	M→K	0.017	0.273	False
BiBrS	49b7be14f786	P3m1 (156)	0.0	1.227	V	Γ→M	0.051	0.037	False
					V	M→K	0.099	0.236	False
					V	Γ→K	0.057	0.0	False
CrSeTe	5d9d3ded04de	P3m1 (156)	0.111	0.59	C	Γ→M	0.003	0.389	False
					C	Γ→K	0.042	0.292	False
PbCl2	f9d58a299674	P-4m2 (115)	0.142	2.165	V	Γ→M	0.004	0.0	False
					V	Γ→X	0.025	0.715	False
AsClTe	fba4cc0df459	P3m1 (156)	0.194	1.316	V	M→Γ	0.019	0.856	False
					C	M→Γ	0.016	0.242	False
MoSeTe	42eb12e7b656	P3m1 (156)	0.025	1.159	V	M→Γ	0.009	0.84	False
					C	Γ→M	0.018	0.332	False
					C	Γ→K	0.028	0.122	False
BaF2	4b7403281822	P-4m2 (115)	0.375	5.659	V	X→Γ	0.002	0.017	False
ClSbTe	04fdd7d1ec5c	P3m1 (156)	0.153	1.439	C	Γ→M	0.002	0.314	False
					C	Γ→K	0.076	0.315	False
SSn	f98da23471a1	P3m1 (156)	0.118	2.3	V	M→Γ	0.01	0.028	False
					C	M→K	0.084	0.287	False
InN	8cf70870bc5b	P-6m2 (187)	0.482	0.606	V	Γ→K	0.01	1.186	False
In2S2	172ef584c4a6	P-6m2 (187)	0.0	1.684	C	M→K	0.052	1.091	False
BrSbSe	c2a344b393f0	P3m1 (156)	0.124	1.467	V	Γ→M	0.01	0.138	False
					V	Γ→K	0.123	0.0	False
					C	M→K	0.084	0.297	False
Ag2F2	44f6ed525a5a	P1 (1)	0.048	0.52	C	Γ→Y	0.011	3.873	False
					C	Y→H	0.011	3.873	False
S2V2	605c732d5111	P-6m2 (187)	0.534	0.209	C	M→K	0.029	0.0	False
					C	Γ→K	0.022	0.007	False
HfS2	3d4bfe131291	P-4m2 (115)	0.333	2.11	V	X→M	0.012	0.671	False

	Entry Info				Spin Splitting Info				
Formula	C2DB ID	SG index	ΔE_{hull}	Bandgap	Band	k-point	SS	ΔE_{SS}	AC
					C	M→Γ	0.119	0.0	False
PbSe2	45aa9714a72b	P-4m2 (115)	0.372	0.286	V	X→M	0.006	0.0	False
					C	Γ→M	0.167	0.862	False
					C	Γ→X	0.036	0.281	False
SrI2	1059dc7f5fe4	P-4m2 (115)	0.188	3.964	C	Γ→M	0.025	0.811	False
Bi2P2S6	287dcf4f1a19	P1 (1)	0.053	0.953	V	X→H1	0.052	0.376	False
CdCl2	ff50eed37ec7	P-4m2 (115)	0.043	3.632	C	Γ→X	0.001	2.312	False
AsIn	c77a730c90f8	P3m1 (156)	0.4	0.681	V	Γ→M	0.005	0.985	False
					V	Γ→K	0.064	1.058	False
					C	Γ→M	0.082	1.406	False
					C	M→Γ	0.082	1.406	False
					C	Γ→K	0.121	1.669	False
In2P2S6	793870f62166	P1 (1)	0.053	0.852	V	Γ→Y	0.005	0.0	False
					V	Γ→X	0.005	0.0	False
ZrSe2	001dfe9a7fa2	P-4m2 (115)	0.319	1.452	V	Γ→M	0.058	0.784	False
					V	Γ→X	0.032	0.812	False
					V	X→M	0.032	0.812	False
TiTe2	bbb8e581bf27	P-4m2 (115)	0.338	0.397	V	Γ→X	0.014	0.958	False
					V	X→M	0.014	0.669	False
					C	M→Γ	0.014	0.0	False
					C	M→X	0.016	0.033	False
ISbTe	052a3116531d	P3m1 (156)	0.123	1.031	C	M→Γ	0.026	0.311	False
Se2V2	9cf30bd127fe	P-6m2 (187)	0.42	0.248	C	M→K	0.04	0.0	False
AsGa	728f322893fe	P3m1 (156)	0.413	1.069	V	Γ→M	0.016	1.087	False
					V	M→Γ	0.016	1.087	False
					V	Γ→K	0.043	1.243	False
					C	Γ→M	0.08	1.418	False
					C	M→Γ	0.08	1.418	False
					C	Γ→K	0.122	1.784	False
AsBrS	1dcd471c2288	P3m1 (156)	0.034	1.38	V	Γ→M	0.054	0.053	False
					V	Γ→K	0.068	0.0	False
AsBrTe	671e6de2497a	P3m1 (156)	0.163	1.098	V	M→Γ	0.012	0.938	False
Pb2Se2	f615d3b872f4	$Pmn2_1$(31)	0.072	0.949	V	Y→Γ	0.005	0.002	False
					C	S→Y	0.012	0.223	False
					C	Y→S	0.012	0.223	False
					C	Y→Γ	0.006	0.0	False
					C	S→Γ	0.004	0.571	False
AsISe	5d829e480507	P3m1 (156)	0.0	1.164	V	Γ→K	0.06	0.214	True
ZrHf3Te8	3663f526cdf1	P1 (1)	0.127	0.165	V	S→X	0.004	0.454	False
					V	Y→S	0.004	0.421	False
					V	Γ→S	0.018	0.165	False
					C	S→X	0.002	0.254	False
BrIZr	28c61999c692	P3m1 (156)	0.038	0.782	C	M→Γ	0.006	0.088	False
					C	M→K	0.022	1.038	False
ClSbSe	0c0fbdaf8f4a	P3m1 (156)	0.014	1.177	V	M→Γ	0.093	0.035	False
					C	M→K	0.222	1.824	False
					C	Γ→K	0.227	1.799	False
ClSbTe	da5fd2bb47af	P3m1 (156)	0.008	1.291	V	Γ→M	0.127	0.034	False
					V	Γ→K	0.168	0.0	False
					C	M→K	0.19	1.442	False
					C	Γ→K	0.125	1.373	False
PbI2	14411dde597c	P-4m2 (115)	0.145	1.531	V	Γ→M	0.247	0.0	False
					V	Γ→X	0.092	0.338	False
ZrS2	2e44a755e594	P-4m2 (115)	0.31	1.938	V	Γ→M	0.022	0.637	False
					C	M→Γ	0.042	0.0	False
					C	M→X	0.035	0.086	False
ZnI2	ce0e9cd74bb3	P-4m2 (115)	0.0	2.467	V	X→M	0.022	2.467	False

	Entry Info				Spin Splitting Info				
Formula	C2DB ID	SG index	ΔE_{hull}	Bandgap	Band	k-point	SS	ΔE_{SS}	AC
PbO2	8d2de90b58b6	P-4m2 (115)	0.339	1.084	V	Γ→X	0.004	0.053	False
					C	X→Γ	0.002	0.0	False
STeZr	3f3c7bc0ce7d	P3m1 (156)	0.122	0.218	C	M→Γ	0.01	0.0	False
Se2Zr2	f89b20d72c95	P-6m2 (187)	0.436	0.06	C	Γ→K	0.037	0.0	False
Hf2Zr2S8	540829ada792	P1 (1)	0.2	1.145	V	S→Y	0.018	0.0	False
					V	Y→S	0.018	0.0	False
					C	X→S	0.012	0.132	False
HfZr3S8	78bb1ac31c01	P1 (1)	0.193	1.145	V	Γ→X	0.002	0.0	False
					V	S→Y	0.017	0.02	False
					V	Y→S	0.017	0.02	False
					V	Y→Γ	0.005	0.07	False
					C	X→S	0.016	0.111	False
Cr2W2Te8	62bb754c4cb2	Pm (6)	0.082	0.512	V	S→Γ	0.136	0.16	False
					C	Γ→X	0.008	0.0	False
Mo2W2Se8	a1d716aad84d	P1 (1)	0.0	1.288	V	S→Γ	0.224	0.262	False
					C	X→Γ	0.011	0.0	True
BrClZr	8cb69386d06b	P3m1 (156)	0.01	0.912	C	M→K	0.018	1.368	False
HfSSe	9afb20358166	P3m1 (156)	0.193	0.91	C	M→Γ	0.008	0.0	False
P2Ru2S6	9caed1a0620c	P1 (1)	0.179	0.322	V	Γ→Y	0.072	0.0	False
					V	Γ→X	0.073	0.0	False
					C	Y→H	0.004	0.0	False
					C	C→H	0.004	0.0	False
					C	C→H1	0.004	0.0	False
					C	X→H1	0.004	0.0	False
BiITe	2d41b3dd1772	P3m1 (156)	0.0	0.701	V	Γ→M	0.11	0.012	False
					V	Γ→K	0.131	0.0	False
ZrHf3S8	9cbc09153aeb	P1 (1)	0.208	1.152	V	S→Y	0.02	0.008	False
					V	Y→S	0.02	0.008	False
					V	Y→Γ	0.007	0.068	False
					V	Γ→S	0.017	0.034	False
					C	X→S	0.015	0.135	False
					C	Γ→Y	0.007	0.0	False
ZrTi3Se8	52a5e2b280d4	P1 (1)	0.131	0.571	V	S→X	0.015	0.249	False
					C	X→Γ	0.033	0.161	False
Ir2O2	06ebe3806790	P-6m2 (187)	0.51	0.099	C	Γ→K	0.11	0.373	False
Te2V2	5da53e6996e3	P-6m2 (187)	0.49	0.22	C	M→K	0.054	0.0	False
HgI2	7c2657e15a6f	P-4m2 (115)	0.0	1.512	V	X→M	0.019	0.0	False
P2Sb2Te6	82b85dfd7723	P1 (1)	0.14	0.633	V	Γ→Y	0.226	0.007	False
					V	Γ→X	0.223	0.0	False
WCr3S8	dc4259e69783	Pmm2 (25)	0.009	0.887	C	X→Γ	0.007	0.0	False
Al2P2S6	669d6f1af4d4	P1 (1)	0.083	1.301	V	Y→Γ	0.01	0.0	False
					V	X→Γ	0.01	0.0	False
					C	Γ→Y	0.002	0.663	False
					C	Γ→X	0.002	0.667	False
Cr2Mo2Te8	988b11badabb	P1 (1)	0.067	0.575	V	S→Γ	0.095	0.123	False
ISbSe	343d2125478e	P3m1 (156)	0.13	1.078	C	M→Γ	0.069	0.34	False
					C	M→K	0.055	0.394	False
Bi2P2Te6	cf7927ab6730	P1 (1)	0.14	0.507	V	Γ→Y	0.241	0.004	False
					V	Y→Γ	0.241	0.004	False
					V	X→Γ	0.239	0.0	False
					V	Γ→X	0.239	0.0	False
HgTe	1a3bdd1b142a	P3m1 (156)	0.165	0.132	C	Γ→M	0.13	1.627	False
BiBrTe	304bc6a92d82	P3m1 (156)	0.0	0.878	V	Γ→K	0.001	0.065	False
ZrO2	24a8929c68ce	P-6m2 (187)	0.766	1.683	C	M→K	0.065	0.026	False
AsIS	e23390b66883	P3m1 (156)	0.256	0.295	C	M→K	0.016	0.65	False
Hf2Zr2Te8	93099006c996	P1 (1)	0.121	0.189	V	Y→S	0.012	0.375	False
					V	Γ→S	0.02	0.189	False

	Entry Info					Spin Splitting Info			
Formula	C2DB ID	SG index	ΔE_{hull}	Bandgap	Band	k-point	SS	ΔE_{SS}	AC
W2I6	37deba64dc68	P-62m (189)	0.19	0.205	V	Γ→M	0.018	0.0	False
					V	M→Γ	0.018	0.0	False
					C	M→Γ	0.085	0.258	False
GeF2	943ceb2df00b	P-4m2 (115)	0.215	1.623	V	Γ→X	0.001	1.956	False
WMo3Te8	323fb700d903	P1 (1)	0.005	0.923	V	Y→S	0.008	0.423	False
					V	S→Γ	0.21	0.192	False
					C	Γ→X	0.014	0.0	False
					C	X→Γ	0.014	0.0	False
					C	Γ→S	0.03	0.222	False
AsIS	b13beafa16aa	P3m1 (156)	0.064	1.395	V	Γ→K	0.135	0.079	True
WCr3Se8	c798e725e2fb	P1 (1)	0.009	0.698	V	Y→S	0.046	0.499	False
					V	S→Γ	0.117	0.173	False
AsBiCr	b299416bff28	P3m1 (156)	0.461	0.037	C	M→Γ	0.039	0.0	False
BaBr2	df54a81e64da	P-4m2 (115)	0.244	4.568	C	X→M	0.03	0.719	False
Ga2Se2	394e5709a3ac	P-6m2 (187)	0.0	1.736	C	M→K	0.063	1.04	False
MoW3Se8	24d6cc0a0fed	Pm (6)	0.0	1.276	V	S→Γ	0.245	0.293	False
					C	X→Γ	0.018	0.0	False
GaN	c973e283b023	P-6m2 (187)	0.416	1.818	V	Γ→K	0.019	1.549	False
Cr2Mo2S8	72b286460831	Pma2 (28)	0.017	1.039	C	S→Γ	0.024	0.305	False
HfZr3Te8	916e19eae465	P1 (1)	0.115	0.242	V	S→X	0.005	0.367	False
					V	S→Y	0.029	0.228	False
					V	Y→S	0.009	0.33	False
					V	Γ→S	0.019	0.242	False
					C	X→Γ	0.037	0.02	False
					C	S→X	0.002	0.2	False
ISSb	4c49d27e66e5	P3m1 (156)	0.185	0.872	C	M→K	0.057	0.469	False
GeI2	694ac91aec01	P-4m2 (115)	0.153	1.059	V	Γ→M	0.309	0.0	False
					V	Γ→X	0.139	0.532	False
					C	Γ→M	0.008	1.342	False
					C	Γ→X	0.036	1.45	False
					C	M→X	0.003	1.309	False
Rh2Br6	c284d6de2b3e	P3 (143)	0.408	0.26	V	K→Γ	0.003	0.001	False
ClSSb	9188c300265c	P3m1 (156)	0.048	1.332	V	Γ→M	0.006	0.045	False
					V	M→Γ	0.006	0.045	False
					V	Γ→K	0.01	0.0	False
					C	M→K	0.312	1.879	False
HfO2	6e4ac7453419	P-4m2 (115)	0.51	4.494	V	X→Γ	0.008	0.628	False
					C	Γ→M	0.184	1.209	False
					C	Γ→X	0.156	0.782	False
SnTe	e688959ea45b	P3m1 (156)	0.119	1.592	C	M→Γ	0.025	0.0	False
HfCl2	864f8b497185	P-6m2 (187)	0.007	0.891	C	M→K	0.15	1.596	False
GeCl2	3ea474649fa9	P-4m2 (115)	0.143	1.381	V	Γ→M	0.002	0.0	False
					V	Γ→X	0.027	1.107	False
					V	X→M	0.002	0.0	False
					C	Γ→M	0.056	1.708	False
ZnBr2	4718298eb660	P-4m2 (115)	0.0	3.278	V	X→M	0.012	4.046	False
					C	Γ→M	0.099	1.754	False
					C	Γ→X	0.017	0.768	False
					C	X→M	0.065	1.685	False
ClIZr	73202b4b7837	P3m1 (156)	0.078	0.883	C	M→K	0.013	0.92	False
P2Sc2Se6	093920d00119	P3 (143)	0.0	0.68	V	Γ→M	0.032	0.0	False
					V	M→Γ	0.032	0.0	False
					V	K→M	0.032	0.0	False
					V	K→Γ	0.031	0.005	False
					V	Γ→K	0.031	0.005	False
					C	Γ→M	0.013	0.29	False
					C	K→Γ	0.008	0.279	False

	Entry Info				Spin Splitting Info				
Formula	C2DB ID	SG index	ΔE_{hull}	Bandgap	Band	k-point	SS	ΔE_{SS}	AC
					C	Γ→K	0.008	0.279	False
Ti2Zr2Se8	846b50801a93	P1 (1)	0.142	0.616	C	X→Γ	0.025	0.149	False
TiHf3S8	eb71cb1c9077	P1 (1)	0.205	1.005	V	X→S	0.017	0.15	False
					V	Y→S	0.025	0.016	False
					V	Y→Γ	0.008	0.111	False
					C	X→Γ	0.028	0.033	False
					C	X→S	0.002	0.091	False
					C	Γ→Y	0.009	0.0	False
GeTe	eadd37f03ca5	P3m1 (156)	0.087	1.488	C	M→Γ	0.043	0.0	False
					C	M→K	0.233	0.813	True
Ir2Cl6	be7870547213	P321 (150)	0.555	0.263	V	M→Γ	0.002	0.0	False
					C	M→Γ	0.001	0.071	False
S2Si2	0726c763a59a	$Pmn2_1$(31)	0.446	1.433	C	S→Y	0.006	0.082	False
					C	Y→Γ	0.012	0.0	False
					C	S→Γ	0.005	0.879	False
BaCl2	ef1fab58e11f	P-4m2 (115)	0.3	5.195	C	X→M	0.031	0.724	False
OSn	026ebfd86b48	P3m1 (156)	0.329	1.682	C	Γ→M	0.102	1.698	False
					C	M→Γ	0.102	1.698	False
					C	Γ→K	0.071	2.315	False
SnTe2	c81de95356c1	P-4m2 (115)	0.139	0.39	V	X→Γ	0.088	0.171	False
					V	X→M	0.015	0.171	False
					C	Γ→M	0.344	0.876	False
					C	Γ→X	0.096	0.22	False
AsClS	0fd6ab210774	P3m1 (156)	0.224	1.732	C	M→K	0.05	0.414	False
W2Br6	14e114b103e1	P-62m (189)	0.271	0.222	C	M→Γ	0.077	0.375	False
Ir2S2	dd6289af8e01	P-6m2 (187)	0.305	0.135	C	Γ→K	0.029	1.386	False
CaI2	793f311ee701	P-4m2 (115)	0.174	4.022	C	Γ→M	0.012	0.388	False
MoCr3Te8	899032b4ad0c	P1 (1)	0.087	0.481	V	S→Γ	0.097	0.137	False
BiIS	40034665f9f1	P3m1 (156)	0.14	0.848	C	M→K	0.333	0.539	False
GaP	d467820f3f04	P3m1 (156)	0.447	1.555	C	M→Γ	0.01	1.217	False
					C	M→K	0.01	0.625	False
Al2S2	f9df9f4a5c34	P-6m2 (187)	0.005	2.099	V	M→K	0.012	0.649	False
HfSSe	63618e5bf062	P3m1 (156)	0.0	0.705	C	Γ→M	0.019	0.794	False
					C	Γ→K	0.065	0.959	False
ZrBr2	7897c7cc2491	P-6m2 (187)	0.0	0.827	C	M→K	0.01	1.413	False
AsBrSe	989f469f06bd	P3m1 (156)	0.0	1.212	C	M→Γ	0.005	0.982	False
HgSe	619ed885f677	P3m1 (156)	0.157	0.069	V	Γ→M	0.01	0.007	False
					V	M→Γ	0.048	0.899	False
					V	Γ→K	0.023	0.0	False
BiITe	a84d988e38ac	P3m1 (156)	0.11	0.691	V	Γ→K	0.029	0.165	False
AsBrTe	64921449e408	P3m1 (156)	0.0	1.253	V	Γ→M	0.092	0.023	False
					V	Γ→K	0.105	0.0	False
					C	Γ→M	0.02	0.838	False
SSeZr	1a9901838600	P3m1 (156)	0.0	0.616	C	Γ→M	0.005	0.609	False
					C	Γ→K	0.024	0.831	False
GeCl2	a7216f084785	P-6m2 (187)	0.136	2.955	C	M→K	0.045	0.089	False
O2Pb2	20f098bd3f31	Pm (6)	0.287	0.215	V	Y→S	0.03	0.155	False
					V	Y→Γ	0.091	0.138	False
SSeZr	2be14f373da0	P3m1 (156)	0.163	0.831	V	Γ→K	0.105	0.0	False
					C	Γ→M	0.003	0.0	False
					C	Γ→K	0.063	0.086	False
BrSbSe	89b15ddef41d	P3m1 (156)	0.0	1.072	V	M→Γ	0.036	0.035	False
					V	M→K	0.044	0.383	False
PbSe	a0dbdc6630fa	P3m1 (156)	0.217	1.68	V	Γ→M	0.047	0.002	False
					V	M→Γ	0.047	0.002	False
					V	Γ→K	0.053	0.0	False
					C	Γ→M	0.293	0.542	False

Entry Info					Spin Splitting Info				
Formula	C2DB ID	SG index	ΔE_{hull}	Bandgap	Band	k-point	SS	ΔE_{SS}	AC
					C	Γ→K	0.346	0.887	False
GeS2	69b36b84eb8c	P-4m2 (115)	0.049	1.36	V	Γ→X	0.003	0.382	False
					C	Γ→M	0.038	1.504	False
					C	X→M	0.028	0.97	False
HfSeTe	305c779b8752	P3m1 (156)	0.149	0.16	C	Γ→M	0.014	0.0	False
					C	Γ→K	0.212	0.298	False
SnCl2	95805103ce95	P-4m2 (115)	0.146	1.464	V	Γ→M	0.002	0.0	False
					V	Γ→X	0.02	1.116	False
					V	X→M	0.002	0.0	False
Pb2Te2	fdc4a7cc1d0d	$Pmn2_1$(31)	0.075	0.701	V	S→Y	0.003	0.213	False
					V	Y→S	0.003	0.213	False
HfTi3Se8	c55716558616	P1 (1)	0.137	0.589	V	S→X	0.012	0.268	False
					C	X→Γ	0.015	0.025	False
					C	Y→Γ	0.002	0.064	False
AlSb	1734deee2ac1	P3m1 (156)	0.475	1.447	V	Γ→M	0.018	0.969	False
					V	M→Γ	0.018	0.969	False
					V	Γ→K	0.011	1.117	False
					C	Γ→M	0.129	0.855	False
					C	Γ→K	0.223	1.151	False
Mo2W2Te8	c04fc052f2ca	Pm (6)	0.011	0.879	V	Y→S	0.022	0.477	False
					V	S→Γ	0.217	0.224	False
					C	X→Γ	0.01	0.0	False
BiISe	70cbc0e44d36	P3m1 (156)	0.0	0.929	V	Γ→K	0.037	0.206	True
GeO	a42f736f1682	P3m1 (156)	0.311	2.093	V	Γ→M	0.006	0.437	False
					C	Γ→M	0.002	2.191	False
					C	M→Γ	0.002	2.191	False
					C	M→K	0.057	0.759	False
					C	Γ→K	0.071	2.677	False
Pb2Te6	3995fa1bee6e	$P2_1$(4)	0.129	0.322	V	S→X	0.009	0.0	False
					V	S→Γ	0.063	0.024	False
					C	Y→Γ	0.026	0.759	False
					C	Γ→Y	0.04	0.781	False
Mo2Cl6	61d74efeaeeb	P-62m (189)	0.139	0.526	C	M→Γ	0.03	0.372	False
BrSSb	4ae37f15e1fe	P3m1 (156)	0.157	1.437	C	M→Γ	0.042	0.533	False
					C	M→K	0.1	0.357	False
ZrTe2	599f0c912458	P-4m2 (115)	0.373	0.816	V	Γ→M	0.112	0.858	False
					V	Γ→X	0.065	0.882	False
					V	X→M	0.065	0.882	False
					C	M→Γ	0.034	0.0	False
					C	X→M	0.038	0.044	False
Cr2W2S8	5974b6403c31	Pma2 (28)	0.014	0.967	C	X→Γ	0.009	0.0	True
					C	Y→S	0.025	0.199	False
HfZr3Se8	70e7ab872359	P1 (1)	0.15	0.819	V	X→S	0.01	0.214	False
					V	Y→Γ	0.003	0.09	False
					V	Γ→Y	0.003	0.09	False
					C	X→Γ	0.018	0.039	False
					C	X→S	0.004	0.137	False
					C	S→Y	0.03	0.041	False
HfSe2	08401460f377	P-4m2 (115)	0.337	1.676	V	Γ→M	0.081	0.718	False
					V	Γ→X	0.064	0.74	False
					V	X→M	0.064	0.74	False
					C	M→Γ	0.116	0.0	False
					C	M→X	0.148	0.012	False
Rh2Se2	1a46a7cf8fab	P-6m2 (187)	0.164	0.063	V	M→K	0.105	0.653	False
AsClSe	df329350eef2	P3m1 (156)	0.179	1.71	C	M→K	0.075	0.402	False
GeSe	211bcb7f05d6	P3m1 (156)	0.04	2.215	V	Γ→M	0.003	0.111	False
					V	M→Γ	0.072	0.054	False

	Entry Info				Spin Splitting Info				
Formula	C2DB ID	SG index	ΔE_{hull}	Bandgap	Band	k-point	SS	ΔE_{SS}	AC
					C	Γ→M	0.094	0.767	False
					C	M→K	0.104	0.611	False
					C	Γ→K	0.162	0.908	False
TiZr3S8	ec37c6657ea3	P1 (1)	0.184	0.997	V	Y→S	0.016	0.039	False
					V	Y→Γ	0.005	0.107	False
					C	X→Γ	0.006	0.052	False
					C	Y→Γ	0.003	0.0	False
Al2P2Se6	90d84f697622	P1 (1)	0.067	0.607	V	Γ→Y	0.056	0.0	False
					V	Γ→X	0.056	0.001	False
					C	Γ→Y	0.064	0.588	False
					C	Γ→X	0.063	0.589	False
Hf2Zr2Se8	81af2831dbb2	P1 (1)	0.158	0.845	C	X→Γ	0.036	0.025	False
MoW3Te8	5c3fe56a1a89	Pm (6)	0.018	0.825	V	Y→S	0.014	0.546	False
					V	S→Γ	0.239	0.254	False
Mo2W2S8	449640ec4d30	Pc (7)	0.0	1.553	C	X→Γ	0.013	0.0	True
HfTi3S8	fde2d81d10df	P1 (1)	0.169	0.848	V	S→X	0.009	0.154	False
					V	Y→S	0.02	0.014	False
					C	X→Γ	0.009	0.036	False
HgCl2	ce3ed4728e8f	P-4m2 (115)	0.033	2.404	C	X→M	0.001	0.0	False
GeBr2	204ef2affa10	P-4m2 (115)	0.136	1.312	C	Γ→M	0.007	1.599	False
Ga2Cl6	c306553fa81a	P-62m (189)	0.174	2.553	V	Γ→M	0.02	0.0	False
BrClHf	72257f9ad66d	P3m1 (156)	0.016	0.819	V	Γ→M	0.01	0.802	False
					V	Γ→K	0.018	0.793	False
					C	M→K	0.119	1.4	False
AsClS	afd0d75a82a2	P3m1 (156)	0.056	1.532	V	Γ→M	0.005	0.044	False
					V	M→K	0.018	0.465	False
					V	Γ→K	0.005	0.0	False
					C	Γ→M	0.016	0.935	False
					C	M→K	0.096	1.787	False
TiO2	1cfb690281c9	P-6m2 (187)	0.587	1.136	C	M→K	0.031	0.121	False
P2Sc2S6	bc8b8c21ad4f	P3 (143)	0.08	0.829	V	Γ→Y	0.007	0.0	False
					V	Γ→X	0.007	0.0	False
CrMo3S8	644f7c1c85c7	Pm (6)	0.011	1.206	V	S→Y	0.075	0.557	False
					C	Γ→X	0.011	0.0	False
					C	Γ→S	0.001	0.216	False
					C	S→Γ	0.011	0.278	False
OPb	2a393480e273	P3m1 (156)	0.315	1.806	V	Γ→M	0.017	0.032	False
					V	Γ→K	0.019	0.0	False
InSb	466fcf7fad66	P3m1 (156)	0.379	0.477	V	Γ→M	0.031	0.991	False
					V	M→Γ	0.031	0.991	False
					V	Γ→K	0.091	1.082	False
					C	Γ→M	0.187	1.174	False
					C	Γ→K	0.283	1.414	False
AsClTe	4fd8ad708fb0	P3m1 (156)	0.018	1.496	C	M→K	0.097	1.188	False
					C	Γ→K	0.038	1.159	False
CrW3Te8	eef072f845ce	P1 (1)	0.056	0.563	V	S→Γ	0.204	0.223	False
MoCr3S8	3fb52099b370	P1 (1)	0.011	0.922	C	X→Γ	0.006	0.0	False
					C	S→Γ	0.007	0.398	False
BiBrS	3b305c3e2c18	P3m1 (156)	0.116	1.594	C	M→K	0.321	0.478	False
CrW3Se8	0b7696e1f4c9	P1 (1)	0.01	0.902	V	S→Γ	0.191	0.256	False
PdSe2	0ae696751911	P-6m2 (187)	0.268	0.231	V	Γ→K	0.344	1.632	False
TiHf3Te8	1667d1443160	P1 (1)	0.132	0.098	V	Γ→X	0.003	0.513	False
					V	S→X	0.004	0.475	False
					V	Γ→S	0.017	0.098	False
Ti2Zr2Te8	18e377cce57f	P1 (1)	0.118	0.143	V	Γ→S	0.022	0.143	False
					C	X→Γ	0.05	0.25	False
N2O2Zr3	c317fbd68215	P-6m2 (187)	0.094	0.404	C	Γ→K	0.019	0.385	False

Entry Info					Spin Splitting Info				
Formula	C2DB ID	SG index	ΔE_{hull}	Bandgap	Band	k-point	SS	ΔE_{SS}	AC
PbS	5e4ff1f56b4a	P3m1 (156)	0.231	1.979	C	Γ→M	0.295	0.542	False
					C	M→K	0.268	0.681	False
					C	Γ→K	0.347	0.801	False
Ge2S2	ecbb7c185669	$Pmn2_1$(31)	0.031	1.714	C	Y→Γ	0.056	0.0	False
InP	c5672c6c1c78	P3m1 (156)	0.432	1.072	V	Γ→M	0.017	1.008	False
					V	M→Γ	0.017	1.008	False
					V	Γ→K	0.01	1.098	False
					C	Γ→M	0.01	1.378	False
					C	M→Γ	0.01	1.378	False
					C	M→K	0.017	1.1	False
					C	Γ→K	0.029	1.724	False
SeTeZr	dd69b684c867	P3m1 (156)	0.115	0.275	C	Γ→M	0.009	0.0	False
					C	M→Γ	0.009	0.0	False
					C	Γ→K	0.094	0.198	False
Hf2Ti2S8	c8cfffe63bfa	P1 (1)	0.192	0.917	V	X→S	0.006	0.19	False
					V	Y→S	0.027	0.0	False
					C	X→Γ	0.013	0.026	False
					C	X→S	0.001	0.09	False
PbTe	3bc08d486d65	P3m1 (156)	0.198	1.151	C	Γ→M	0.349	0.383	False
					C	Γ→K	0.398	0.869	False
ZrTi3S8	e6e376c80c43	P1 (1)	0.163	0.833	V	S→X	0.01	0.147	False
					V	Y→S	0.016	0.029	False
					C	X→Γ	0.002	0.036	False
CdI2	d63ad801fdb5	P-4m2 (115)	0.0	2.381	V	X→M	0.018	0.0	False
AsBrS	d9f4d4011670	P3m1 (156)	0.201	1.425	C	M→Γ	0.04	0.481	False
					C	M→K	0.018	0.52	False
TiZr3Te8	4f1ab08988cc	P1 (1)	0.115	0.21	C	X→Γ	0.026	0.247	False
MoSTe	e4bb8738150a	P3m1 (156)	0.065	1.027	V	M→Γ	0.006	0.917	False
TiHf3Se8	3e1923c616ad	P1 (1)	0.166	0.722	V	Γ→X	0.021	0.037	False
					V	X→S	0.018	0.228	False
					C	Y→Γ	0.006	0.062	False
CrO2	2433700165bb	P-6m2 (187)	0.168	0.422	V	M→K	0.049	1.259	False
Cr2Mo2Se8	60065d3bbcf2	P1 (1)	0.016	0.837	V	S→Γ	0.1	0.158	False
					C	X→Γ	0.012	0.0	False
					C	S→Γ	0.002	0.29	False
CrMo3Se8	a7233837cfe9	P1 (1)	0.01	0.971	V	S→Γ	0.115	0.185	False
					C	Γ→X	0.018	0.0	False
					C	S→Γ	0.004	0.253	False
Ti2Zr2S8	a99139546333	P1 (1)	0.18	0.842	V	S→X	0.005	0.205	False
					V	S→Y	0.021	0.0	False
					V	Y→S	0.021	0.0	False
ZrHf3Se8	b8fb10416122	P1 (1)	0.165	0.843	V	Y→Γ	0.002	0.083	False
					C	X→Γ	0.044	0.021	False
					C	X→S	0.002	0.167	False
					C	S→Y	0.036	0.004	False
ClSSb	0495f35048b5	P3m1 (156)	0.179	1.675	V	Γ→M	0.01	0.099	False
					V	M→Γ	0.01	0.099	False
					V	Γ→K	0.034	0.0	False
					C	M→K	0.135	0.292	False
Hf2Ti2Se8	cce78d90e899	P1 (1)	0.156	0.656	V	Γ→X	0.021	0.029	False
					V	X→S	0.002	0.236	False
					V	Γ→S	0.03	0.024	False
					C	X→Γ	0.021	0.053	False
					C	X→S	0.022	0.0	False
SnS2	08a9307b286e	P-4m2 (115)	0.082	1.451	V	Γ→X	0.004	0.258	False
					C	M→Γ	0.034	1.351	False
					C	Γ→M	0.034	1.351	False

		Entry Info					Spin Splitting Info		
Formula	C2DB ID	SG index	ΔE_{hull}	Bandgap	Band	k-point	SS	ΔE_{SS}	AC
					C	Γ→X	0.007	0.427	False
					C	X→Γ	0.007	0.427	False
					C	M→X	0.027	1.123	False
SnSe2	bfa429d647f9	P-4m2 (115)	0.056	0.854	V	X→M	0.006	1.212	False
					C	Γ→M	0.157	1.191	False
					C	Γ→X	0.024	0.358	False
PbS2	9842835dff03	P-4m2 (115)	0.418	0.671	V	M→Γ	0.002	0.169	False
					V	M→X	0.002	0.0	False
					C	X→M	0.033	0.896	False
WMo3Se8	05a06afa3b20	Pm (6)	0.0	1.32	V	S→Γ	0.195	0.232	False
					C	Γ→X	0.01	0.0	False
					C	X→Γ	0.01	0.0	False
					C	Y→S	0.025	0.124	False
O2W2	42fa50003592	P-6m2 (187)	0.503	0.04	V	Γ→K	0.163	0.0	False
BrSSb	4da5c6be60db	P3m1 (156)	0.028	1.233	V	M→Γ	0.061	0.056	False
Cr2W2Se8	548aa830244c	P1 (1)	0.015	0.778	V	S→Γ	0.153	0.195	False
					C	Y→S	0.009	0.187	False
ZnCl2	1b7175e04416	P-4m2 (115)	0.0	4.226	V	Γ→X	0.003	0.936	False
					C	Γ→M	0.016	2.096	False
					C	Γ→X	0.011	2.084	False
					C	X→M	0.011	2.084	False
TiZr3Se8	a148361e5e9a	P1 (1)	0.144	0.701	V	Y→Γ	0.002	0.112	False
					C	X→Γ	0.012	0.043	False
Al2Se2	129a514b51ad	P-6m2 (187)	0.0	1.997	C	M→K	0.034	0.328	False
GeS	227b12019ade	P3m1 (156)	0.053	2.467	C	Γ→M	0.057	1.039	False
					C	M→K	0.036	0.509	False
					C	Γ→K	0.078	1.141	False
BiClS	c96ef4fc869c	P3m1 (156)	0.0	1.334	V	Γ→M	0.01	0.031	True
					V	Γ→K	0.018	0.0	False
Ga2P2Te6	4cb4ea247ef4	P1 (1)	0.173	0.314	V	Γ→Y	0.022	0.406	False
					V	Γ→X	0.017	0.405	False
AsITe	b6d803aafe3a	P3m1 (156)	0.0	1.009	V	Γ→M	0.001	0.335	False
					V	M→K	0.059	0.779	False
					V	Γ→K	0.051	0.311	False
AsClSe	1a3be826b3e0	P3m1 (156)	0.013	1.364	C	M→K	0.073	1.703	False
HfTe2	1e2c6946ca41	P-4m2 (115)	0.371	1.01	V	Γ→M	0.147	0.78	False
					V	Γ→X	0.119	0.798	False
					V	X→M	0.119	0.798	False

References

[1] Sten Haastrup et al. "The Computational 2D Materials Database: high-throughput modeling and discovery of atomically thin crystals". In: *2D Materials* 5 (4 Sept. 2018), p. 042002. ISSN: 2053-1583. DOI: 10.1088/2053-1583/AACFC1.
