# Supplementary material for: High-throughput inverse design and Bayesian optimization of functionalities: spin splitting in two-dimensional compounds
Source: Sci Data. 2022 Apr 29;9:195. doi: 10.1038/s41597-022-01292-8 (PMC9054849; doi:10.1038/s41597-022-01292-8)
Supplement: Supplementary file 6 [file 41597_2022_1292_MOESM6_ESM.pdf]

Supplementary Information

High-throughput inverse design and optimization of functionalities: spin splitting in two-dimensional compounds

Gabriel M. Nascimento[1,a], Elton Ogoshi[1,a], Adalberto Fazzio[1,2], Carlos Mera Acosta[1,*], and Gustavo M. Dalpian[1,*]

[a] *These authors contributed equally to this work.*
[1] *Center for Natural and Human Sciences, Federal University of ABC, Santo Andre, SP, Brazil*
[2] *Brazilian Nanotechnology National Laboratory (LNNano), CNPEM, 13083-970, Campinas, São Paulo, Brazil*
[*] *Corresponding authors: cmeraacosta@gmail.com; gustavo.dalpian@ufabc.edu.br*

1 Structural clusters

To evaluate the crystal structure influence on target properties, an alternative is to group the materials by their structures in such a way that similar structures are grouped in a label and dissimilar are separated into different labels. Analyzing how a target property distributes along the different groups allows us to get to correlations between the property and certain characteristics of the structural clusters, such as the local environment of certain atoms, symmetry, etc.

A simple approach would be to group the structures using only the *space group* as a label. Figure 1a illustrates the case for the space group #156 ($P3m1$), which is comprised of the T-Phase and the H-Phase of MXY Janus materials, and also a binary hexagonal phase. The latter can be easily differentiated by simply adding its AB *stoichiometry* to the label. In contrast, the MXY Janus has the same stoichiometry and space group but different local environments for the blue in-plane atoms (octahedral for T-Phase and trigonal prismatic for H-Phase) resulting from the different occupied Wyckoff positions by the green out-of-plane atoms. This shows that using only stoichiometry + space group as a label is not enough to differentiate these two structures, as a set of different occupied Wyckoff positions potentially changes the crystal structure while preserving the space group symmetry.

The next natural step is then to use the *occupied Wyckoff positions* together with space group as a way to circumvent cases such as the one presented in Figure 1a. The C2DB's *crystal prototype* label concatenates the stoichiometry to space group together with the occupied Wyckoff positions. Then we can see that the T-Phase and H-Phase are correctly separated into two different labels, ABC-156-abc and ABC-156-ac, respectively.

The problem remains in the cases illustrated by Figure 1b and c. In Figure 1b we see two examples of how the Wyckoff positions should be the same but are different. In the space group #115, a and d are both maximal Wyckoff positions and also have the same site symmetry. In this case, separating into AB_2-115-ag and AB_2-115-dg labels is not desirable as both materials have the same crystal structure. We assume that this inconsistency might happen due to a different choice of origin in the unit cell and/or some different *spglib* [4] (the package used for crystal symmetry analysis) choice of parameters. The same happens for the pair of crystal prototypes AB_2-187-bi and AB_2-187-ai, and for many others.

The case illustrated by the Figure 1c points to another significant problem. The spglib package has a tolerance factor for analyzing and classifying crystal structures into different space groups. The C2DB was built using a considerably tight tolerance factor (TF) of 10^{-4}Å in comparison to other High-Throughput DFT databases, such as the Materials Project that uses 10^{-1}Å as TF. This results in a sparsely populated space of crystal prototypes due to many crystal structures being classified as low symmetry space groups. The three materials shown in Figure 1c have a X-M-M-X sequence of quasi-layers. All of them have the same stacking alignment and, with a looser TF, they would be classified as AB-12-i. Due to the tight TF used by C2DB, this results in many of them being separated into lower symmetry crystal prototypes, such as AB-2-i or AB-1-a.

In addition to not grouping what should be grouped, the label also groups what should not be grouped. This happens especially for low symmetry space groups due to the generality of their Wyckoff

positions. Figure 1d shows two different crystal structures grouped under the same label of AB-2-i.

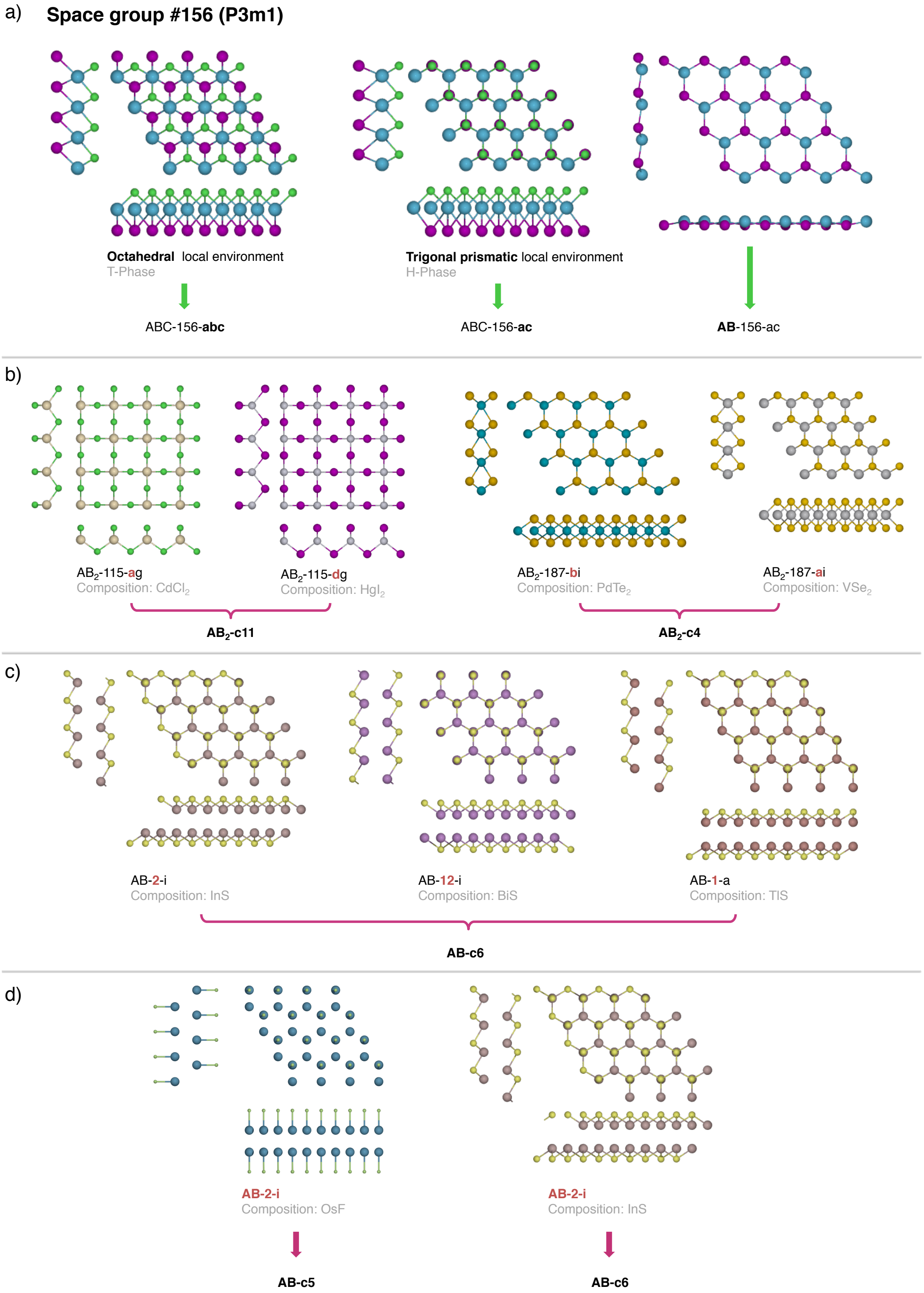

Figure 1: Four cases which the structural cluster labels defined in this work show to be important as a comparison to the C2DB's crystal prototypes: a) Three different structures with the same space group symmetry ($P3m1$ or space group #156). Using the stoichiometry and the occupied Wyckoff positions, such as the C2DB's crystal prototype label does, one can correctly separate these structures into different groups; b) Two cases in which there are two different C2DB's crystal prototypes (AB_2-115-ag and AB_2-115-dg; AB_2-187-bi and AB_2-187-ai) for the same structure. They are correctly grouped into AB_2-c11 and AB_2-c4; c) Three C2DB's crystal prototypes for the same crystal structure resulting from the tight tolerance factor employed. They are all grouped into AB-c6; d) Two different crystal structures under the same label (AB-2-i) when they should not be. They are correctly separated when using the structural cluster label.

To circumvent these problems, we decided to adopt an unsupervised approach to define structural labels/clusters. The proposed method has four stages:

1. Generating Crystal Fingerprints (CFs) for all materials in C2DB;
2. Embedding of CFs into a 2D space;
3. Clustering the 2D embedded space into clusters;
4. Using the material's stoichiometry to subdivide these clusters into *structural clusters*, i.e., the labels we use to categorize materials by their crystal structure.

To generate CFs we used the local coordination information of all occupied sites within the unit cell. For each occupied site it is generated a 61-dimensional vector ($\mathbf{v}^{\mathrm{site}}$) in which each dimension is a parameter characterizing the atom's coordination environment given by its coordination number CN and its coordination environment q (e.g. octahedral, tetrahedral, trigonal planar). CN is given by the CrystalNN [3] method and q by the method described by Zimmermann et al. [5]. Each dimension of $\mathbf{v}^{\mathrm{site}}$ gives the likelihood $w_{CN=n}$ of the site having a $CN = n$, where $n \in [0, 13]$, and additionally its likelihood $q_{\mathrm{CE=m}|_{\mathrm{CN}=i}}$ of having the m coordination environment (CE), where the set of possible m is given by n. As an example, the following $\mathbf{v}^{\mathrm{site}}$ vector shows that for $CN = 2$, some of the possible coordination environments are q_L for L-shaped geometries, q_{water} for a water-like angle geometry and q_{linear} for linear geometry:

$$\mathbf{v}^{\mathrm{site}} = [w_{\mathrm{CN}=1}, \quad w_{\mathrm{CN}=2}, \quad q_{\mathrm{L}|_{\mathrm{CN}=2}}, \quad q_{\mathrm{water}|_{\mathrm{CN}=2}}, \quad q_{\mathrm{linear}|_{\mathrm{CN}=2}}, \quad \ldots]^{\mathrm{T}}. \tag{1}$$

The set $\{\mathbf{v}^{\mathrm{i}}\}$ for each of the i sites in the unit cell is then processed to generate a $\mathbf{v}^{\mathrm{structure}}$, given by statistical measures of each of the 61 dimensions of (1) in all sites. We chose the minimum, maximum, mean, standard deviation, 25% quantile, 50% quantile, and 75% quantile values, resulting in a 427-dimensional (7 statistical values x 61 dimensions) $\mathbf{v}^{\mathrm{structure}}$ for each of the 3814 materials in the C2DB.

$$\begin{aligned}\mathbf{v}^{\mathrm{structure}} = {} & \mathit{minimum}(\{\mathbf{v}^{\mathrm{i}}\})^{\mathrm{T}} \oplus \mathit{maximum}(\{\mathbf{v}^{\mathrm{i}}\})^{\mathrm{T}} \oplus \mathit{mean}(\{\mathbf{v}^{\mathrm{i}}\})^{\mathrm{T}} \oplus \mathit{std}(\{\mathbf{v}^{\mathrm{i}}\})^{\mathrm{T}} \oplus \\ & \mathit{quantile}_{25\%}(\{\mathbf{v}^{\mathrm{i}}\})^{\mathrm{T}} \oplus \mathit{quantile}_{50\%}(\{\mathbf{v}^{\mathrm{i}}\})^{\mathrm{T}} \oplus \mathit{quantile}_{75\%}(\{\mathbf{v}^{\mathrm{i}}\})^{\mathrm{T}}.\end{aligned} \tag{2}$$

As the resulting CFs have a large dimensionality, we then used them as input for the UMAP [2] embedding technique. We used $n_{neighbors} = 50$ and $min\_dist = 0$ as parameters for the embedding, with a Euclidean distance metric. The result is a two-dimensional embedding that characterizes the structural differences and similarities of all materials in C2DB, as shown in Figure 2.

In order to delineate the clusters withing this 2d space, we used sklearn's implementation of DB-SCAN [1] with $eps = 0.7$ and $min\_samples = 5$. A total of 26 structural clusters were found in the 3814 materials of C2DB, each labeled by a number from 0 to 25. We then proceeded to further subdivide these clusters accordingly to the materials' stoichiometry. The labels of structural clusters were then defined as $\{$*stoichiometry*$\}$-c$\{$*cluster number*$\}$. The H-Phase of MoS_2 is then labeled as a AB_2-c22 because of its AB_2 stoichiometry and its cluster number being 22. We used this label as a categorical feature for the Bayesian Inference analysis in the main text. Figure 1 shows the structural cluster labels for each of the materials presented in the illustrative cases.

A total of 23 structural clusters were defined in the subset of the 436 screened materials. Figure 3 shows the distribution of these materials on the different structural clusters. We opted for using only the structural clusters with at least five entries for the Bayesian Inference analysis (a total of 13 clusters). Figure 4 shows their general structure.

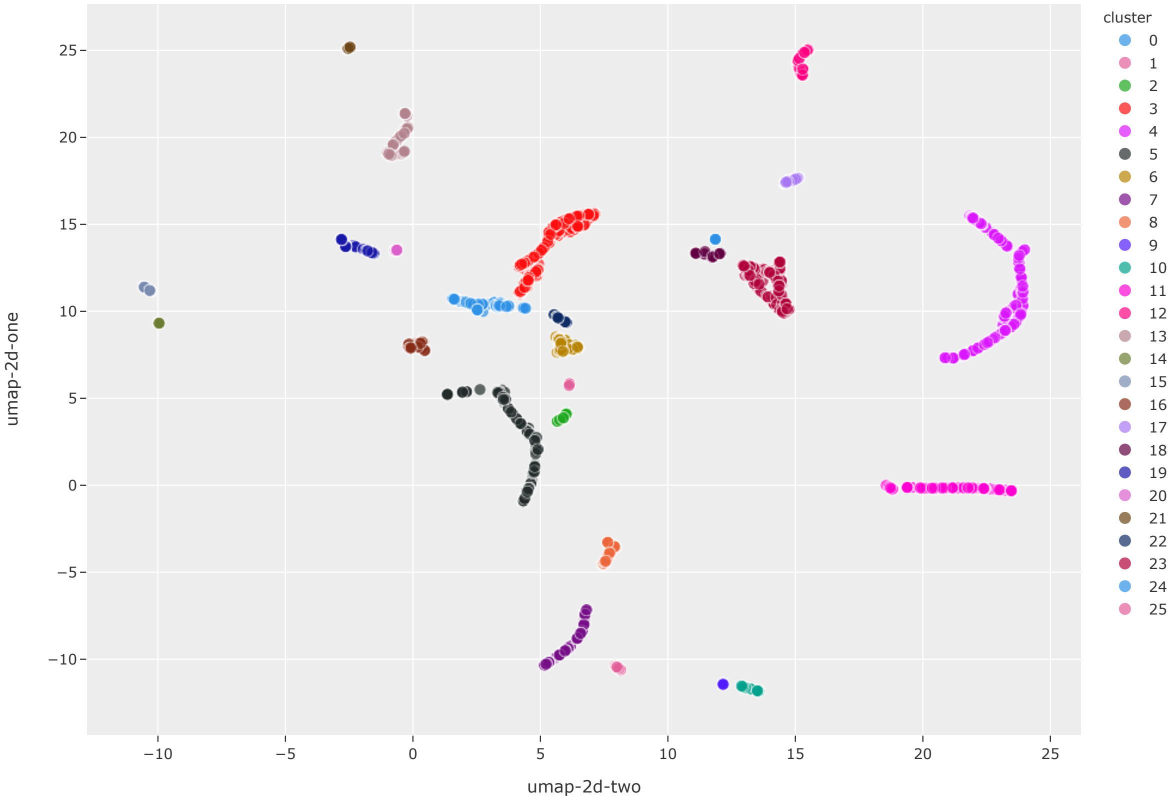

Figure 2: 2D Umap embedding used for structural clustering of all C2DB materials. Each color encodes a prior cluster, i.e. the structural clusters before the subdivision into different stoichiometries (e.g. the cluster 22 is subdivided into ABC_4-c22 and AB_3C_8-c22).

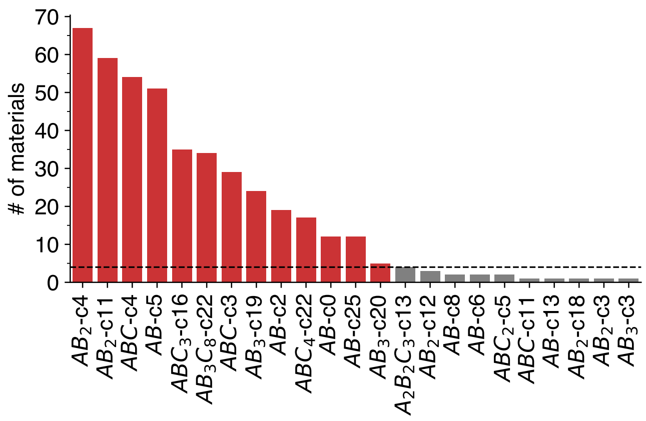

Figure 3: Distribution of structural clusters in the dataset. Only structural clusters with at least five materials (red) were used on Bayesian Inference analysis.

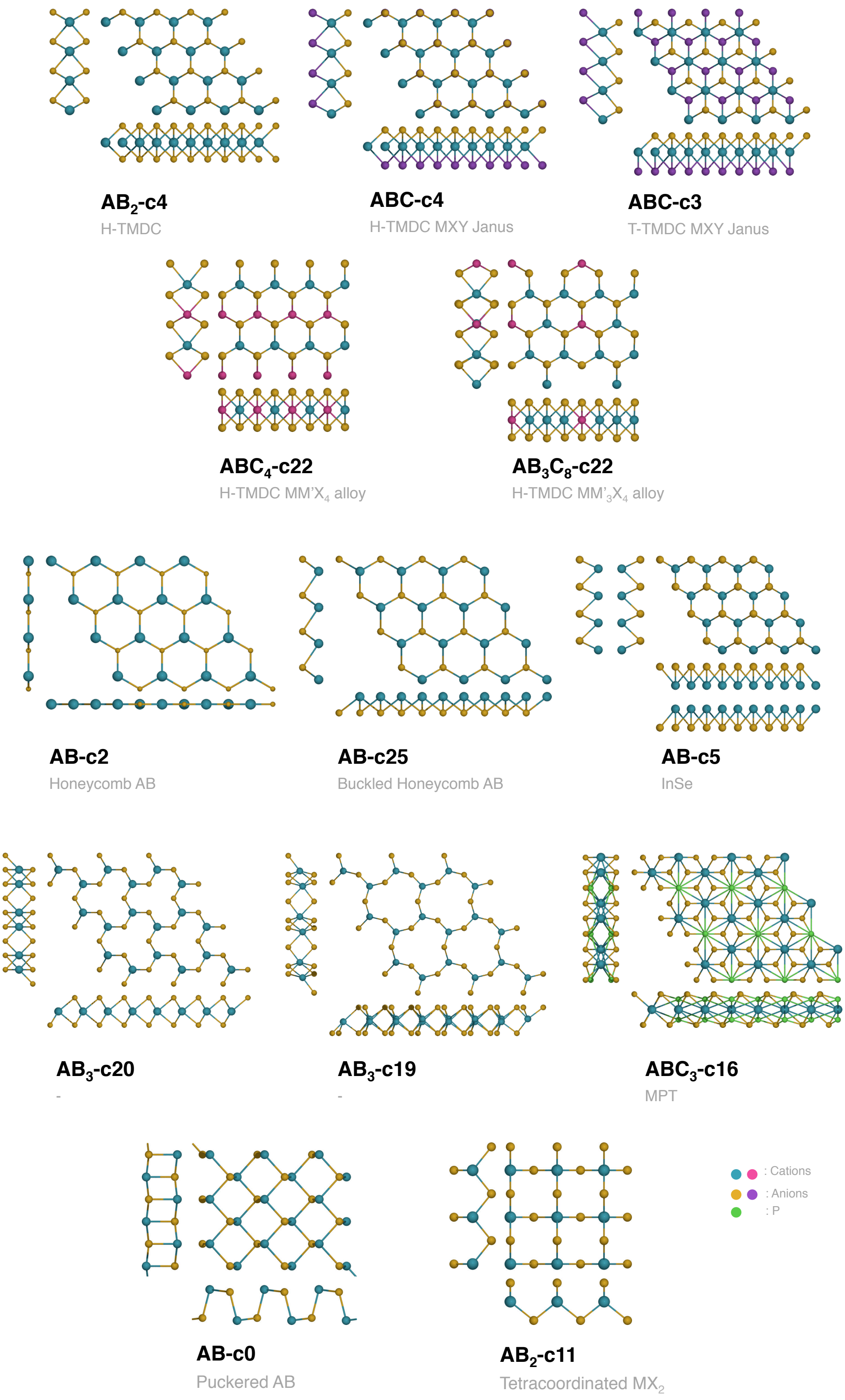

Figure 4: The 13 structural clusters and the top and side views of their general crystal structures.

2 Cations and anions distributions

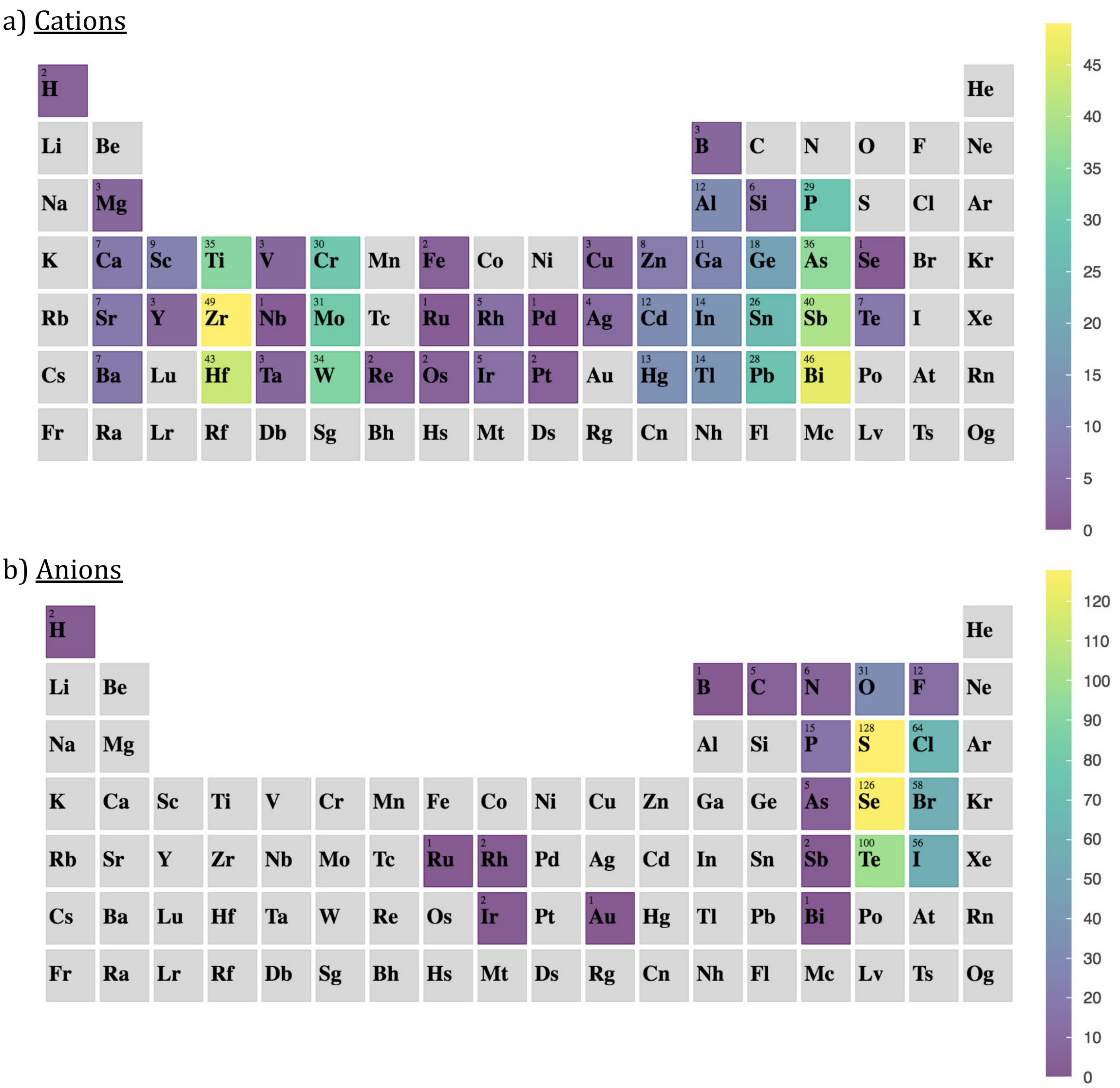

Figure 5: Cation and anion distributions of all 436 materials.

3 Target properties structural and compositional heatmaps

Rashba/Dresselhaus spin splitting

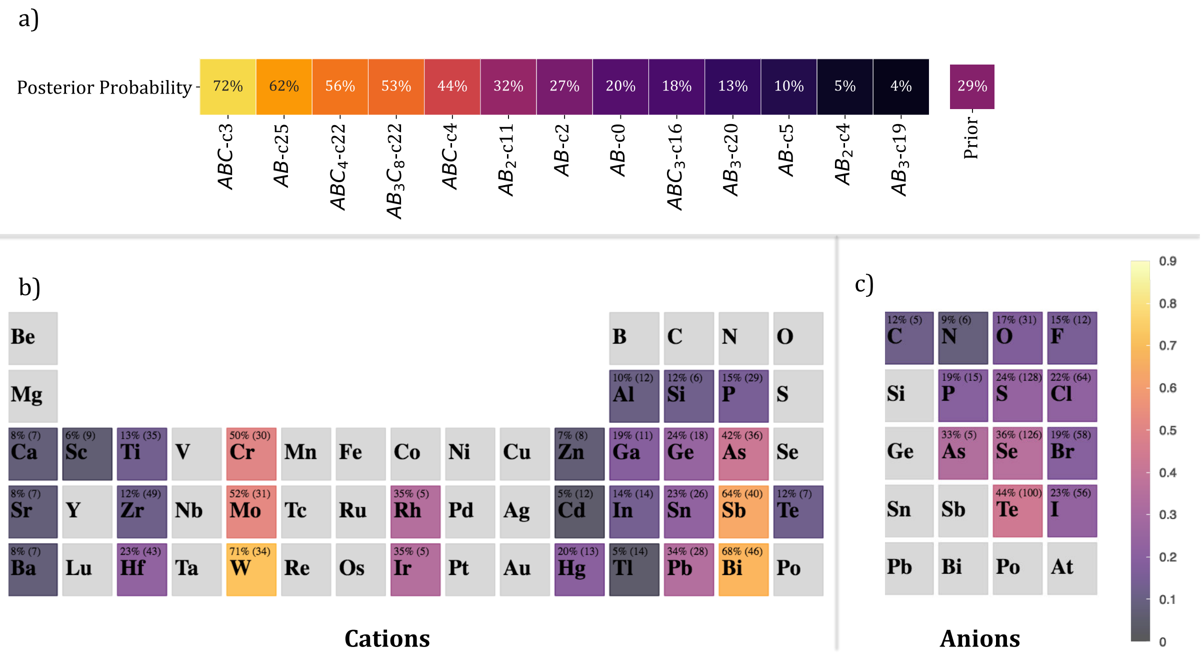

Figure 6: Bayesian inference heatmap for Rashba/Dresselhaus spin splitting greater than 100 meV. a) Given a structural cluster; b) Given cation; c) Given anion. The prior probability is 29%.

Rashba parameter

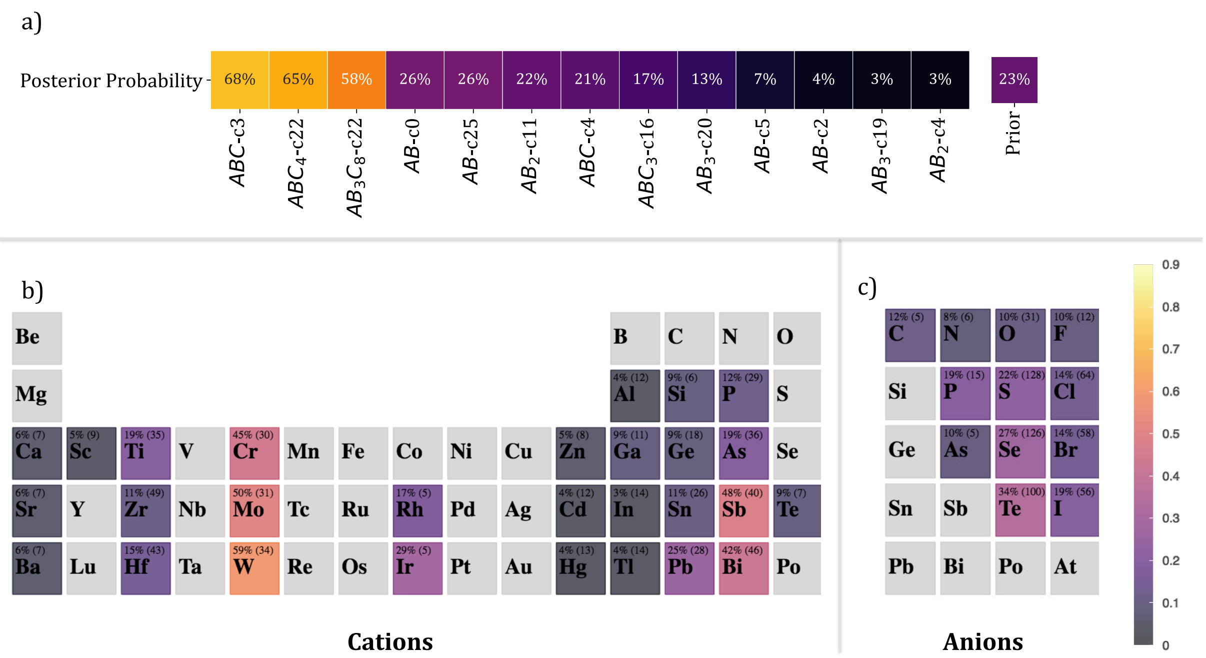

Figure 7: Bayesian inference heatmap for Rashba parameter greater than 1.0 eV.Å. a) Given a structural cluster; b) Given cation; c) Given anion. The prior probability is 23%.

Band gap

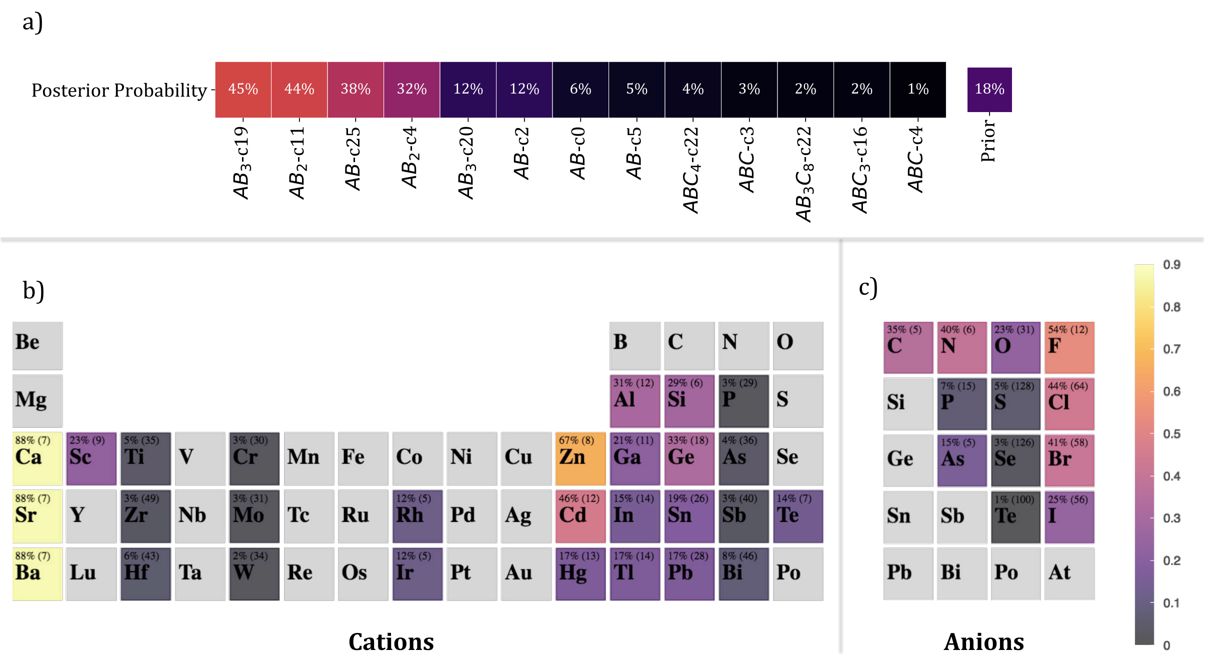

Figure 8: Bayesian inference heatmap for band gap greater than 2.0 eV. a) Given a structural cluster; b) Given cation; c) Given anion. The prior probability is 18%.

References

[1] Martin Ester et al. “A density-based algorithm for discovering clusters in large spatial databases with noise.” In: *Kdd*. Vol. 96. 1996, pp. 226–231.

[2] Leland McInnes, John Healy, and James Melville. “Umap: Uniform manifold approximation and projection for dimension reduction”. In: *arXiv preprint arXiv:1802.03426* (2018).

[3] Hillary Pan et al. “Benchmarking coordination number prediction algorithms on inorganic crystal structures”. In: *Inorganic chemistry* 60.3 (2021), pp. 1590–1603.

[4] Atsushi Togo and Isao Tanaka. “Spglib: a software library for crystal symmetry search”. In: *arXiv preprint arXiv:1808.01590* (2018).

[5] Nils ER Zimmermann and Anubhav Jain. “Local structure order parameters and site fingerprints for quantification of coordination environment and crystal structure similarity”. In: *RSC Advances* 10.10 (2020), pp. 6063–6081.
